# Hong Kong orchids on the EDGE: a phylogenetic framework for conservation planning, trade mitigation and population rescue

**DOI:** 10.3389/fpls.2026.1801915

**Published:** 2026-04-28

**Authors:** Jihong Li, Jinlong Zhang, Stephan W. Gale

**Affiliations:** Kadoorie Farm and Botanic Garden, Flora Conservation Department, Hong Kong, Hong Kong SAR, China

**Keywords:** Biodiversity Strategy and Action Plan (BSAP), conservation prioritization, EDGE framework, habitat fragmentation, *Orchidaceae*, Red List, wildlife trade

## Abstract

**Introduction:**

Hong Kong, a vital part of the Indo-Burma Biodiversity Hotspot, harbours a significant portion of China’s orchid diversity within a limited area. This unique flora faces severe treats from habitat degradation, illegal collection and trade, placing many species disproportionately at risk at the local level. Effective conservation prioritization is urgently needed. This study applies the latest EDGE framework to Hong Kong’s orchids, integrating phylogenetic distinctiveness with extinction risk to identify conservation priorities and establishing a scientific basis for targeted protection and rescue.

**Methods:**

We compiled a comprehensive checklist of Hong Kong orchids and constructed a time-calibrated phylogeny using nuclear (ITS) and plastid (*trn*L-F*, mat*K, *trn*H-*psb*A) sequence data. The EDGE2 metric, integrating Evolutionary Distinctiveness (ED) and extinction risk from regional and national Red Lists, was applied to identify conservation priorities. Correlations between threat status and four traits (habit, habitat specificity, mating system and occurrence in trade) were also examined.

**Results:**

Our assessment revealed that a significantly higher proportion of Hong Kong’s 138 native orchid taxa, comprising 134 species and four varieties, are threatened locally (76 taxa in the Hong Kong Red List) compared to nationally (43 taxa in the China Red List). Phylogenetic analysis of 134 taxa (130 species and four varieties) revealed a significant correlation between ED and Hong Kong Red List status, and EDGE2 analysis using local Red List data highlighted *Vanilla shenzhenica*, *Nervilia plicata*, *Apostasia nipponica*, *Bletilla striata* and *Acanthophippium gougahense* as the greatest priorities. Moreover, local threat status exhibited significant positive correlations with habit, habitat specificity, mating system and trade.

**Discussion:**

The EDGE2 framework effectively balances immediate extinction risk with the preservation of evolutionary history, revealing critical disparities between local and national threat assessments. Importantly, it identifies evolutionarily distinct species overlooked by threat-based methods alone. Positive correlations corroborate an association between threat status and certain ecological traits. Altogether, these findings advocate for the integration of phylogenetic and trait-based approaches into regional conservation strategies to effectively safeguard Hong Kong’s unique orchid diversity and its evolutionary heritage, offering a model for other regions globally.

## 1 Introduction

South China (encompassing Yunnan, Guangxi, Guangdong and Hainan Provinces, as well as the Special Administrative Regions of Hong Kong and Macau) contains a unique assemblage of species and ecosystems that together account for a significant portion of the world’s flora and fauna (Corlett, 2018; Wang et al., 2020). In part, it lies within the Indo-Burma Biodiversity Hotspot (Mittermeier et al., 2004), globally recognized as one of the ten most irreplaceable and five most threatened ecoregions (Myers et al., 2000). The area’s high taxonomic diversity, including numerous endemic species, underscores its ecological importance both within China and throughout seasonal tropical Asia (Mi et al., 2021; Zhu, 2017). In addition, its ecosystems confer essential supporting services for over 242 million inhabitants (Hong Kong Census and Statistics Department, 2023; National Bureau of Statistics of China, 2021; Statistics and Census Service, 2023). However, centuries of anthropogenic pressure, including habitat degradation, ongoing land conversion and other synergistic threats, place South China’s biodiversity and ecosystems at grave risk of further decline and elimination, demanding urgent conservation to protect and restore ecological integrity (Ren et al., 2007; Xu et al., 2024).

Chief threats include landscape-scale degradation linked to urbanization, agriculture and the expansion of plantation forestry (Han et al., 2019; Liu et al., 2019), as well as pollution, poaching and wildlife trade (Wang et al., 2020; Zhu, 2017), which in combination disproportionately affect certain vulnerable taxa (Corlett, 2018; Mi et al., 2021). The critically endangered Chinese pangolin (*Manis pentadactyla* L.) and Chinese giant salamander (*Andrias davidianus* Blanchard), for instance, are both flagship species emblematic of biodiversity declines linked to habitat loss and fragmentation, as well as collection for traditional Chinese medicine (TCM) and illegal wildlife trade (Lu et al., 2020; Xu et al., 2024; Zhang et al., 2022). Numerous plant species of the region’s tropical and subtropical forests, including conifers (Coniferae), oaks (Fagaceae), figs (Moraceae), Dipterocarps (Dipterocarpaceae) and orchids (Orchidaceae), have also been identified as being especially vulnerable to logging, poaching, manmade fire and fragmentation (Besi et al., 2019; Guo et al., 2024; Li et al., 2016; Mi et al., 2021; Zhu, 2017).

The Orchidaceae embody wide ecological and evolutionary diversity in South China (Zhang et al., 2015; Zhu, 2017), with in excess of 1,200 species (Chen et al., 2009; POWO, 2025). However, the specialized mycorrhizal dependencies, pollination strategies and narrow distribution ranges of many species renders them highly vulnerable to stochastic environmental impacts (Gravendeel et al., 2004; Liu et al., 2015; McCormick and Jacquemyn, 2014). This vulnerability is further exacerbated by direct and indirect anthropogenic threats, including habitat loss from urbanization and intensive overharvesting for both horticultural (e.g., in the case of *Cymbidium* Sw., *Dendrobium* Sw. and *Paphiopedilum* Pfitzer) and medicinal trade [e.g., in the case of *Bletilla striata* (Thunb.) Rchb.f. and multiple *Dendrobium* species], which have intensified in recent decades (Gale et al., 2019; Liu et al., 2014; Luo et al., 2003). This intersection of high orchid diversity and significant threat necessitates targeted prioritization to ensure that conservation resources are allocated efficiently (Brooks et al., 2006; Gale et al., 2018; Li et al., 2018; Zhou et al., 2021). To achieve this, a robust, evidence-based framework for prioritization is essential to safeguard orchid populations and their associated habitats across the region.

Conservation prioritization frequently relies on IUCN Red Listing (IUCN, 2024), which despite its utility in guiding funding allocation (Mooers et al., 2008; Possingham et al., 2002), suffers from taxonomic biases and knowledge gaps, particularly in terms of under-representation of certain taxonomic groups, including plants, potentially leading to skewed conservation focus (Forest et al., 2015; Guénard et al., 2025; Li et al., 2018). Emerging methods seek to address these gaps by integrating evolutionary metrics, such as Phylogenetic Diversity (PD), which quantifies evolutionary pathways (Faith, 1992), and Evolutionary Distinctiveness (ED), which measures unique evolutionary history (Isaac et al., 2007). These metrics underpin frameworks such as EDGE (evolutionarily distinct, globally endangered), which prioritises species that represent significant phylogenetic information at risk (Isaac et al., 2007). Initially validated for mammals with well-resolved phylogenies (Redding et al., 2010), the EDGE approach has since been extended to amphibians (Safi et al., 2013), birds (Jetz et al., 2014) and plants (Li et al., 2018), with updated models such as EDGE2 incorporating extinction risk adjustments (Gumbs et al., 2023). However, widespread adoption remains hindered by data gaps, as few jurisdictions or higher-level taxonomic groups (such as families) possess comprehensive phylogenies and Red List assessments, especially in the case of plants (Forest et al., 2015; Vitt et al., 2023; IUCN, 2024). Given these constraints, regional adaptations (e.g., EDRE, regionally endangered framework; Li et al., 2018) and trait-based strategies (e.g., correlations between habitat and conservation status; Brüniche-Olsen et al., 2021; Moser et al., 2016) offer opportunities to refine targeting. Ultimately, achieving global integration of evolutionary and conservation priorities demands coordinated efforts to address data deficiencies in understudied taxa and jurisdictions, as incomplete information continues to limit the scalability of these approaches (Gale et al., 2018; Li et al., 2018).

Hong Kong Special Administrative Region (henceforth ‘Hong Kong SAR’) exemplifies this requirement for effective integration of comprehensive biological data in a conservation context, with the territory offering key insights in terms of both thorough taxonomic understanding and complete conservation assessments that could be of significance at the scale of South China, as well as for seasonal tropical Asia as a whole (Baretto et al., 2011; Gale et al., 2014; Hong Kong Environment Bureau, 2016, 2025). Despite comprising only 0.01% of China’s land area, Hong Kong hosts nearly 10% of the country’s orchid diversity, representing all five subfamilies and exhibiting an array of ecological adaptations and growth habits, spanning terrestrial, epiphytic, lithophytic, myco-heterotrophic and vinous lifestyles (Baretto et al., 2011; Chen et al., 2009; Gale et al., 2014; Hong Kong Herbarium, 2021; Hu, 2022). Alarmingly, however, over half of these species face a heightened risk of extinction at the scale of Hong Kong (i.e. IUCN threat categories VU, EN, or CR; IUCN, 2024), primarily due to habitat degradation and illegal harvesting (Agriculture, Fisheries and Conservation Department, 2025). Given these challenges, implementation of the EDGE approach holds great promise for planning and actioning conservation for the territory’s threatened orchids. By elucidating the evolutionary distinctiveness of these taxa, EDGE analysis could enhance our understanding of their phylogenetic relationships and biogeographic history while identifying priority species that are critical for preserving phylogenetic diversity. Such an approach stands not only to optimize resources, but also underscores Hong Kong’s role as a model for aligning evolutionary insights with actionable conservation strategies, thereby strengthening regional biodiversity conservation efforts (Gale et al., 2018).

The application of the EDGE2 framework at a regional scale of Hong Kong advances beyond previous national or global EDGE assessments by enabling phylogenetically informed conservation prioritization for orchids, leveraging the territory’s comprehensively documented flora to bridge fine-scale data resolution with global evolutionary metrics. To achieve this, our hypotheses are that (1) phylogenetic prioritization will identify species diverging from Red List-based priorities, emphasizing evolutionarily distinct taxa under-represented in current conservation strategies; (2) Red List threat status will correlate with specific traits, such as growth habit, habitat specificity, mating system and vulnerability to trade, providing actionable insights for trait-driven conservation; and that (3) the combined use of phylogenetic and ecological data will enhance the spatial and functional scope of conservation efforts by strategically targeting taxa critical to evolutionary and ecosystem resilience. Furthermore, this study attempts to establish a Hong Kong orchid DNA barcoding framework, which can enhance species identification and monitoring, directly supporting conservation planning especially in the context of mitigating trade in orchids as ornamental plants and for TCM. This study evaluates the scalability of EDGE frameworks in data-rich regions with understudied biodiversity while addressing biases in conventional prioritization frameworks (e.g., Red Listing). It is anticipated that the insights gained from Hong Kong’s unique biodiversity and pressures could guide prioritization of under-studied and under-resourced groups in other global hotspots, offering a roadmap to bridge phylogenetic theory with actionable conservation.

## 2 Material and methods

### 2.1 Ethics statement

All orchids sampled in this study were acquired and/or are maintained by Kadoorie Farm and Botanic Garden (KFBG), Hong Kong, in line with all existing legislation. KFBG holds a possession licence from the Government of Hong Kong SAR to maintain a collection of native Hong Kong orchids, all of which are protected by law.

### 2.2 Study taxa and their conservation status

To facilitate our study, we constructed a checklist of all 138 accepted orchid names recorded in Hong Kong following Chen et al. (2009), Baretto et al. (2011), Gale et al. (2013, 2014, 2020, 2021), Kumar et al. (2014); Hong Kong Herbarium (2021); Hu (2022); Kumar (2023) and Kumar and Gale (2023). The checklist (Supplementary File S1 Table) was structured following currently accepted orchid classification (Chase et al., 2015). The regional conservation status of all native orchid species in Hong Kong was acquired from a recent comprehensive analysis compiled by the Agriculture, Fisheries and Conservation Department (2025) of the Government of Hong Kong SAR. In addition, the national conservation status of these species was sourced from the China Biodiversity Red List for Higher Plants, published by the Ministry of Ecology and Environment of the People’s Republic of China and the Chinese Academy of Sciences (2023).

### 2.3 Taxon sampling

Samples were collected from the wild or from plants of Hong Kong origin in cultivation at KFBG. Voucher specimens were formally identified to species level based on morphological characters and by making use of the available literature (cited above) and then deposited in the KFBG herbarium.

### 2.4 DNA extraction, PCR amplification and sequencing

Total DNA was extracted from leaf samples dried in silica gel using the QIAGEN DNeasy^®^ plant DNA Kit (Hiden, Germany) according to the manufacturer’s instructions. The ITS region of nuclear DNA (including ITS1, 5.8S rRNA gene and ITS2) was amplified and sequenced using primers ITS5/ITS4 (Li et al., 2018). For plastid markers, the *mat*K gene was amplified with primers Af2/2R (Li et al., 2018), while the *trn*L-F and *trn*H-*psb*A intergenic spacers were amplified using primers e/f (Taberlet et al., 1991) and *trn*H/*psb*A (Sang et al., 1997; Tate and Simpson, 2003), respectively. PCR was performed as described by Li et al. (2018). The thermal cycler programme consisted of an initial denaturation step at 98 °C for 30 s, followed by 35 cycles of 5 s at 98 °C, 5 s at 60 °C for the *trn*L-F intergenic spacer and 57 °C for the *trn*H-*psb*A intergenic spacer, 20 s at 72 °C, and a final extension at 72 °C for 2 min. Amplification products were purified and sequenced on an ABI3730 DNA Sequencer (Applied Biosystems, USA). All sequences have been deposited in GenBank (**Supplementary File S1 Table**).

### 2.5 Alignment, outgroups and verification of sequences

All sequences derived from the Hong Kong plants were manually edited and confirmed using the Verified Taxonomy Tool in Geneious v11.1.4 (Kearse et al., 2012). We also downloaded the sequence data of these four regions (ITS, *mat*K, *trn*L-F and *trn*H-*psb*A fragments) for as many of the orchid taxa (based on our checklist) as were available in GenBank (accessed February 2019). All four regions (**Supplementary Files S2A–S2D**) were aligned using the MAFFT multiple alignment plugin in Geneious, with subsequent adjustment by eye. Among the three widely used chloroplast barcodes (*mat*K gene, *trn*L-F and *trn*H-*psb*A intergenic spacers), both the *trn*L-F and *trn*H-*psb*A spacers exhibited substantial sequence divergence at the family level that impeded reliable multiple sequence alignment, a challenge that was especially acute in the *trn*H-*psb*A region owing to its frequent indels and complex structural polymorphism (Hollingsworth et al., 2011; Kress and Erickson, 2007). To mitigate these alignment challenges, we excluded a 98-base segment from the *trn*L-F spacer, containing one 71-base indel and one 28-base poly-A tract. For the *trn*H-*psb*A spacer, a total of 1039 bases were removed due to pronounced hypervariability, including four difficult-to-align regions spanning 981 bases and one 134-base indel (**Supplementary Files S2C, S2D**). Finally, two species of Hypoxidaceae (*Curculigo orchioides* Gaertn. and *Hypoxis rigidula* Baker) were used as outgroups based on previous subfamily-level molecular analyses of the orchids (Cameron et al., 1999; Kocyan et al., 2004).

For ITS, we used only the 5.8S rRNA gene for further analysis owing to its greater sequence conservation and higher evolutionary stability compared to the ITS1 and ITS2 regions (Li et al., 2018). To confirm the probable identity of all accessions based on phylogenetic position, the data sets for each of the four markers were subjected to Maximum Likelihood (ML) analysis in RAxML-HPC2 v8.0.9 using the Cyberinfrastructure for Phylogenetic Research (CIPRES) Science Gateway v3.3 (Miller et al., 2010), specifying 1,000 rapid bootstrap replicates, followed by a search for the best-scoring ML tree with other parameters set to default. Based on the best-scoring ML tree, we removed a total of 67 ITS (5.8S), 25 *mat*K, 16 *trn*L-F, and 13 *trn*H-*psb*A sequences due to their long branches, incorrect position or potential misidentification after confirming the probable identity of all accessions (Li et al., 2018) (**Supplementary File S1 Table**). The final dataset included 923 aligned 5.8S sequences representing 132 taxa, 799 *mat*K sequences representing 127 taxa, 517 *trn*L-F sequences representing 127 taxa, and 503 *trn*H-*psb*A sequences representing 121 taxa. The raw data alignment matrices for the four genetic markers are presented in **Supplementary Files S2A–D (Supporting Information)**.

### 2.6 Discriminatory power of barcodes for Hong Kong orchids

To evaluate the scale of interspecific and intraspecific genetic divergences, Kimura-2-parameter (K2P) distances were calculated for those species with multiple accessions within each matrix (**Supplementary Files S3A–S3D**) using Mega-X (Kumar et al., 2018). One-way ANOVA was used to quantify the magnitude of a ‘barcoding gap’, where one was found to exist, within and between species for each of the four regions.

### 2.7 Phylogenetic diversity of Hong Kong orchids

We reconstructed a phylogenetic tree for 134 of Hong Kong’s 138 native orchids after a single sequence was selected at random for each taxon with more than one accession. To resolve phylogenetic relationships, we combined the four data sets to improve phylogenetic accuracy, regardless of incongruence, following Cunningham (1997); Yoder et al. (2001); Baker et al. (2011) and Li et al. (2018), among others. The four combined data sets (**Supplementary File S4**) were subjected to partitioned Maximum Likelihood (ML) analysis by gene in RAxML-HPC2 v8.0.9 using the CIPRES Gateway v3.3 (Miller et al., 2010), specifying 1,000 rapid bootstrap replicates, followed by a search for the best-scoring ML tree with other parameters set to default.

We examined total evolutionary time based on the combined ML phylogram (**Supplementary File S5**). Divergence times were estimated by Non-Parametric Rate Smoothing (NPRS) in r8s (Sanderson, 2003) with the age of *Meliorchis* R.M.Bateman ex Ramírez, Gravend. & Chase (15–20 million years old; Ramírez et al., 2007) taken as the minimum age for the monophyletic orchid subtribe Goodyerinae, and the age of the oldest known fossil monocot (110–120 million years old) as the maximum age at the root of the tree (Friis et al., 2004). PD and ED were calculated in the time-calibrated tree (**Supplementary File S6**) using Picante (Kembel et al., 2010) in R software (R Core Team, 2024). A correlation between ED score and extinction risk (the weight of Red List extinction risk category: LC = 0, NT = 1, VU = 2, EN = 3, CR = 4; Isaac et al., 2007) was tested for using a linear regression.

### 2.8 Calculation of EDGE2

EDGE2 considers both the extinction risk of the focal taxon and its adjusted evolutionary distinctiveness, with the latter being based on the phylogenetic position of the focal taxon and its relationships with all other taxa in the tree (Gumbs et al., 2023), written as:

(1) $$EDGE2_{i}=ED2_{i}\times GE2_{i}$$

in which $EDGE2_{i}\;$the *EDGE2* score for species *i*, and $ED2_{i}$ is the relative evolutionary distinctiveness for species *i* (see Equation 2 below), whereas $GE2_{i}$ is the probability of extinction for species *i* at some given time in the future. The relative evolutionary distinctiveness of each species is calculated as:

(2) $$ED2_{i}=TBL_{i}+\sum_{j=2}^{n_{i}}(L_{i,j}\times \prod_{k\in C_{i,j}-i}p_{k})$$

in which $ED2_{i}$ (see Equation 1) the *EDGE2* score for species *i*, and *ED2i* is the relative evolutionary distinctiveness for taxon *i*’s expected unique phylogenetic diversity, or TBL, plus its adjusted branch length relative to the extinction risk of all other species in the phylogeny. Set $C_{i,j}$ represents all species descended from the corresponding branch with length $L_{i,j}$, and $p_{k}$ is the probability of extinction of species *k* (Gumbs et al., 2023).

The EDGE2 score for each species was computed based on the R functions available at https://github.com/rgumbs/EDGE2 (accessed 2024-09-10), in which GE.2.calc was used for calculating extinction risk for the Red List category based on resampling, and EDGE.2.calc was used for calculating the EDGE2 indices (**Supplementary File S1**). Detailed explanation of EDGE2 can be found in Gumbs et al. (2023).

To estimate GE2 for every taxon, we first generated a distribution based on polynomial regression for the IUCN Red List categories, in which the extinction risk for “CR”, “EN”, “VU”, “NT” and “LC” within the next 50 years were considered to be 0.97, 0.485, 0.2425, 0.12125 and 0.060625, respectively (Mooers et al., 2008). The extinction risk of each category was resampled until its median matched the expected value for that Red List category. As suggested by Gumbs et al. (2023), the probability of extinction risk sampled for each IUCN Red List category approximately falls within the following intervals: CR (0.69436, 0.99999), EN (0.33861, 0.69435), VU (0.17085, 0.33861), NT (0.09337, 0.17085) and LC (0.00174, 0.09337). Following the default settings of the R functions, we obtained 743,100 rows of extinction risks to represent the entire distribution of extinction probability for all Red List categories (CR: *n* = 197,163; EN: *n* = 182,966; VU: *n* = 139,860; NT: *n* = 66,784; LC: *n* = 156,327).

We computed EDGE2 using the official Red List both for Hong Kong (Agriculture, Fisheries and Conservation Department, 2025) and China (The Ministry of Ecology and Environment, the People’s Republic of China & The Chinese Academy of Sciences, 2023) by resampling from the above-mentioned distribution of extinction probabilities and the dated phylogeny. Species categorized as “EW” (extinct in the wild) or “RE” (regionally extinct) were regarded as falling within the same interval as CR, i.e. (0.69436, 0.99999). For species marked as “NA” (not available), “DD” (data deficient) or “NE” (not evaluated), extinction risk was sampled from the entire range of extinction probabilities, which ranged from 0.00174 to 0.99999. To quantify uncertainty, we randomly sampled the extinction risk 500 times for each taxon, allowing us to calculate the median and interquartile range (IQR) of its EDGE2 score. Results were then visualised using ggplot2 (Wickham, 2016) in R 4.4.1 (R Core Team, 2024).

### 2.9 Correlation between regional Red List and traits

To examine the potential impact of three biological attributes (habit, habitat specificity and mating system) and one targeted threat (occurrence in trade) on population viability at the regional level, we applied a Spearman’s rank correlation (Sedgwick, 2014) across ranked regional Red List categories and each of these four traits for all 138 Hong Kong orchid taxa (**Supplementary File S1 Table**). To do so, the Red List categories were assigned a numerical value (LC = 1, NT = 2, VU = 3, EN = 4, CR = 5 and RE = 6) and the states of the four traits were assigned based on information given in the references listed below supplemented with the authors’ own field observations (**Supplementary File S1 Table**):

- Habit (epiphyte/lithophyte = 1, terrestrial = 2), as documented by Chen et al. (2009), Baretto et al. (2011) and Gale et al. (2014).
- Habitat specificity (generalist = 1, specialist = 2), as indicated by Chen et al. (2009), Baretto et al. (2011), Gale et al. (2014); Hong Kong Herbarium (2021) and Hu (2022), supplemented by direct observation of plants in the wild. The orchids were firstly assigned to one or more of five distinct habitat types relevant for Hong Kong (lowland and hill forest, montane forest, upland grassland, shrubland and upland cliffs), and generalists were then defined as those taxa that occur in two or more habitat types, whilst specialists were defined as those that are restricted to a single habitat type (Gale et al., 2018).
- Mating system (selfing = 1, outcrossing = 2), as reported by Nilsson (1992); Sun (1997), Baretto et al. (2011), Tang et al. (2014); Hu et al. (2017); Liu et al. (2020); Zhou et al. (2021) and Hu (2022).
- Occurrence in trade (traded for ornamental use = 1, traded for medicinal use = 2, traded for both ornamental and medicinal uses = 3), as reported by Liu et al. (2014); Teoh (2016); Hinsley et al. (2018); Gale et al. (2019); Zhou et al. (2021) and Hu (2022).

The resulting Spearman’s rank correlation coefficients (*r_s_*) were interpreted according to widely adopted ecological guidelines (Hinkle et al., 2003), whereby |*r_s_*| < 0.2 indicated a very weak association, 0.2 ≤ |*r_s_*| < 0.4 weak, 0.4 ≤ |*r_s_*| < 0.6 moderate, and |*r_s_*| ≥ 0.6 strong. All underlying raw data supporting the trait and phylogenetic analyses are available in the Zenodo Repository, DOI: 10.5281/zenodo.18463567.

## 3 Results

We confirmed records for a total of 134 species and 4 varieties, representing 62 genera, 14 tribes and all five subfamilies of Orchidaceae in Hong Kong (**Supplementary File S1 Table**). Of these, the populations of *Cheirostylis jamesleungii* S.Y.Hu & Barretto, *Coelogyne fimbriata* var. *leungiana* (S.Y.Hu) P.J.Cribb & S.W.Gale. and *Crepidium allanii* (S.Y.Hu & Barretto) Kumar & S.W.Gale were regarded as endemic in Hong Kong. A further 17 taxa [i.e. 12% of the total; *Bulbophyllum kwangtungense* Schltr., *B. trigridum*, *Calanthe graciliflora* Hayata, *Cheirostylis clibborndyeri* S.Y.Hu & Barretto, *C. jamesleungii*, *C. monteiroi* S.Y.Hu & Barretto, *Coelogyne cantonensis* (Rolfe) R.Rice, *C. fimbriata* var. *leungiana*, *Crepidium allanii*, *C. cordilabium*, *Dendrobium linawianum* Rchb.f., *D.* cf. *mimicum*, *Gastrodia peichatieniana* S.S.Ying, *Habenaria coultousii* Barretto, *Habenaria leptoloba* Benth., *Peristylus tentaculatus*, *Tainia dunnii* Rolfe and *Vanilla shenzhenica* Z.J.Liu & S.C.Chen] are regarded as endemic to China. At the subfamily level, Epidendroideae (87 species, 65% of the total) has the greatest number of species, followed by Orchidoideae (43 species, 32% of the total), whilst at the tribal level, Malaxideae (31 species, 23% of the total) has the greatest number of species. Epidendroideae (11 genera, 18% of the total) has the largest number of genera. The five largest genera—namely, *Bulbophyllum* Thouars (11 species), *Dendrobium* (8 species), *Liparis* Rich. (8 species), *Habenaria* Willd. (7 species) and *Peristylus* Blume (7 species)—together account for about one-third of all Hong Kong orchids (41 species; 31% of the total).

### 3.1 Species richness, growth habit diversity and habitat extensiveness

Hong Kong’s orchids exhibit a variety of growth habits, with both terrestrial and epiphytic taxa occurring on different substrates. Of the 138 taxa, 84 (61% of the total) are primarily terrestrial in the wild (**Supplementary File S1 Table**), and six of these terrestrial taxa are myco-heterotrophs (**Supplementary File S1 Table**). In contrast, 54 taxa (39% of the total) are either epiphytic or lithophytic, of which 32 are lithophytic, 3 are epiphytic, and 19 are both (**Supplementary File S1 Table**). A single species, *V. shenzhenica*, is a climbing vine.

Hong Kong’s orchids also occur in a diverse array of habitats. Of the 138 taxa, 89 (64%) are found in lowland and hill forest, 57 (41%) are found in montane forest, 33 (24%) are found in upland grassland, 10 (7%) are found in shrubland and 16 (12%) are found on upland cliffs (**Supplementary File S1 Table**). In terms of habitat specificity, the number of specialists confined to a single habitat type (91 taxa; 66% of the total) was twice that of generalists occurring in two or more habitat types (47 taxa; 34% of the total). In terms of conservation status, 55 of the 91 specialists (60%) are regarded as threatened (i.e. VU, EN, CR), and a further 17 are considered regionally extinct in Hong Kong. In contrast, 21 of the 47 generalists (45%) are listed as threatened.

Data pertaining to mating system and pollination strategy were available for a total of 71 taxa. Among these, 61 taxa exhibit a predominantly outcrossing mating system while 10 taxa are primarily selfing (**Supplementary File S1 Table**). Of these, 41 of 61 outcrossing taxa (67%) are threatened and seven regionally extinct, and five of ten selfing taxa (50%) are threatened.

### 3.2 Hong Kong orchid conservation assessment coverage and trade status

**Figure 1:**
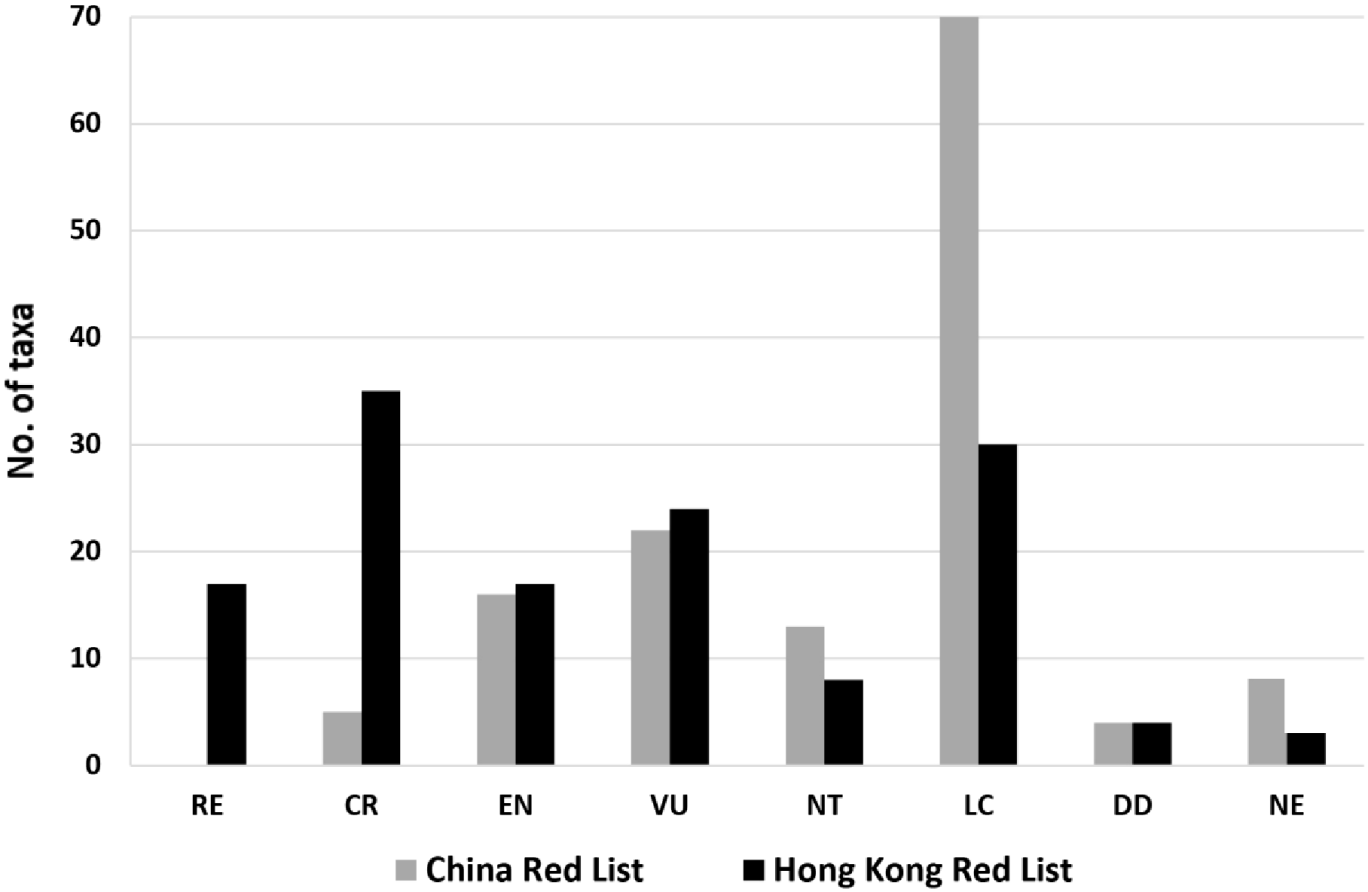
Hong Kong and China Red List status of Hong Kong orchids.

Only 7 Hong Kong orchids (i.e. 5% of the total) have been assessed in the IUCN global Red List. Among these, *Paphiopedilum purpuratum* (Lindl.) Stein is considered CR, *Coelogyne chinensis* (Lindl.) Rchb.f. is considered NT and the remaining five species, *Ania hongkongensis* (Rolfe) Tang & F.T.Wang, *Bulbophyllum delitescens* Hance, *Erythrodes blumei* (Lindl.) Schltr., *Porpax pusilla* (Griff.) Schuit., Y.P.Ng & H.A.Pedersen and *Spiranthes sinensis* (Pers.) Ames, are considered LC (**Supplementary File S1 Table**). By comparison, 130 Hong Kong orchids (i.e. 94% of the total) have been red-listed at the national level (i.e. for China as a whole), of which 5 are considered CR, 16 are considered EN, 22 are considered VU, 13 are considered NT, 70 are considered LC and 4 are considered DD (**Figure 1**, **Supplementary File S1 Table**). Thus, one-third (43 orchids, 33%) are considered threatened (i.e. CR, EN or VU), of which 13 (30% of the total) belong to one of the five largest genera.

Regional Red List assessments have been conducted for 135 taxa (131 species and 4 varieties) at the scale of Hong Kong, with 35 being considered CR, 17 being considered EN, 24 being considered VU, 8 being considered NT, 30 being considered LC, 4 being considered DD and 17 being considered regionally extinct (**Figure 1**, **Supplementary File S1 Table**). Evidently, more than half (76 taxa, 56%) are thus threatened (i.e. CR, EN or VU), of which CR taxa account for nearly half (35 taxa, 46%). In addition, 32 of the 41 taxa belonging to the five largest genera (i.e. 78%) are threatened (**Supplementary File S1 Table**). The number of threatened species, particularly those categorized as CR, is significantly higher in the Hong Kong Red List than in the China Red List (**Figure 1**).

Of the 138 Hong Kong orchids, 88 (64% of the total) are not known to be affected by trade. For the 50 taxa (36% of the total) that are documented in trade, horticultural trade is dominant (affecting 48 taxa; 96% of traded taxa), with trade for medicinal use affecting 9 taxa (18% of traded taxa); 7 taxa [14% of traded taxa; *Anoectochilus roxburghii* (Wall.) Lindl., *Bletilla striata*, *Dendrobium aduncum* Lindl., *D. linawianum*, *D. loddigesii* Rolfe, *Ludisia discolor* (Ker Gawl.) Blume and *Coelogyne chinensis*] are traded for both (**Supplementary File S1 Table**). Approximately one-third of traded taxa belong to the five largest genera (7 species in each of *Bulbophyllum* and *Dendrobium*, plus 2 *Liparis* species) (**Supplementary File S1 Table**).

### 3.3 Availability of Hong Kong orchid sequence data

Of the total 134 taxa (451 accessions) included in this study, sequence data pertaining to 122 were derived from both fresh collections and GenBank, while data for the remaining 12 species [*Cleisostoma williamsonii* (Rchb. f.) Garay, *Collabium chinense* (Rolfe) Tang & F. T. Wang, *Cymbidium kanran* Makino, *Dendrobium anosmum* Lindl., *Dendrobium crumenatum* Sw., *Disperis neilgherrensis* Wight, *Goodyera pusilla*, *Habenaria ciliolaris* Kraenzl., *Peristylus goodyeroides* (D. Don) Lindl., *Renanthera coccinea* Lour., *Tainia cordifolia* Hook. f. and *Thelasis pygmaea* (Griff.) Lindl.] were retrieved exclusively from GenBank, due to unavailability of fresh material (**Supplementary File S1 Table**). Data were unavailable for four species (*Eria gagnepainii* A.D.Hawkes & A.H.Heller, *Zeuxine membranacea* Lindl., *Habenaria coultousii* Barretto and *Peristylus gracilis* Blume) because either they are presumed locally extinct or they have only very recently been recorded in Hong Kong, and they are not accessioned in GenBank. ITS sequences were available for 446 accessions representing 133 taxa, *mat*K sequences for 415 accessions representing 127 taxa, *trn*L*-*F sequences for 435 accessions representing 126 taxa, and *trn*H-*psb*A sequences for 414 accessions representing 120 taxa. Sequence data were newly contributed to GenBank for 11, 19, 73 and 74 orchid taxa for ITS, *mat*K, *trn*L-F and *trn*H-*psb*A, respectively (**Supplementary File S1 Table**).

### 3.4 Discriminatory power of 5.8S, *mat*K, *trn*L-F and *trn*H-*psb*A

**Figure 2:**
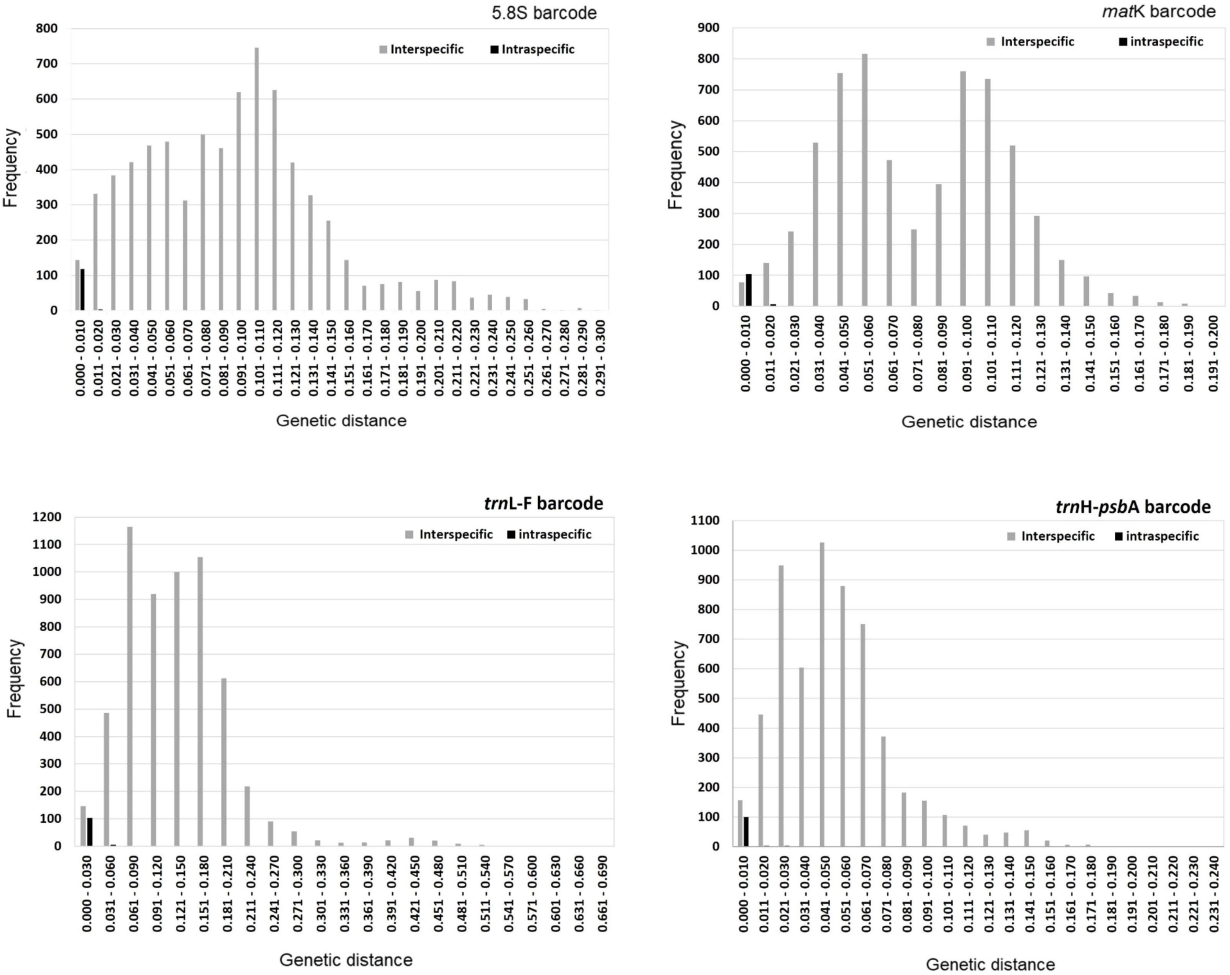
Interspecific and intraspecific genetic distance for 5.8S, *mat*K, *trn*L-F and *trn*H-*psb*A markers.

Among the native Hong Kong orchids, 121 and 113 species were represented by two or more accessions within the 5.8S and *mat*K data sets, respectively; 109 were represented by two or more accessions within each of the *trn*L-F and *trn*H-*psb*A data sets (**Table 1**, **Supplementary Files S3A–S3D**). Among these four barcodes, the ranges in both interspecific and intraspecific genetic distances were greatest for *trn*L-F (0–0.69 and 0–0.12, respectively) and moderate for *trn*H-*psb*A (0–0.24 and 0–0.05, respectively). The range in interspecific genetic distances was lowest for *mat*K (0–0.2) and the range in intraspecific genetic distances was lowest for 5.8S barcodes (0–0.02) (**Figure 2**). Overall, intraspecific genetic distances were skewed towards lower range classes (0–0.02 for 5.8S, 0–0.03 for *mat*K, 0–0.12 for *trn*L-F and 0–0.05 for *trn*H-*psb*A) and their total range was narrower, as compared to interspecific genetic distances (0–0.3 for 5.8S, 0–0.2 for *mat*K, 0–0.69 for *trn*L-F and 0–0.24 for *trn*H-*psb*A). A difference in frequency between interspecific and intraspecific genetic distances was more marked at lower range classes for *trn*L-F and *trn*H-*psb*A than for 5.8S and *mat*K (**Figure 2**). One-way ANOVA indicated a significant barcoding gap between interspecific and intraspecific distances in all four regions (*P* = 2.80E-07 for 5.8S, *P* = 2.10E-05 for *mat*K, *P* = 4.26E-03 for *trn*L-F and *P* = 1.17E-03 for *trn*H-*psb*A) (**Table 1**). This suggests that all four barcodes are suitable for the identification of the orchid species examined in this study.

**Table 1:** Interspecific and intraspecific genetic distances for 5.8S, *mat*K, *trn*L-F and *trn*H-*psb*A data sets.

Genetic distance and diversity	5.8S	*mat*K	*trn*L-F	*trn*H-*psb*A
No. of species	121	113	109	109
No. of accessions	912	785	498	487
Matrix length	182	1,234	690	1,039
Interspecific mean distance	0.0922 ± 0.0028	0.0771 ± 0.0093	0.1333 ± 0.0260	0.0515 ± 0.0090
Intraspecific mean distance	0.0019 ± 0.0003	0.0034 ± 0.0009	0.0070 ± 0.0026	0.0038 ± 0.0012
Overall mean distances	0.0877 ± 0.0168	0.0765 ± 0.0046	0.1297 ± 0.0106	0.0480 ± 0.0043
Interspecific mean diversity	0.0859 ± 0.0172	0.0731 ± 0.0045	0.1228 ± 0.0098	0.0442 ± 0.0038
Intraspecific mean diversity	0.0019 ± 0.0003	0.0034 ± 0.0002	0.0070 ± 0.0007	0.0038 ± 0.0003
Overall mean diversity	0.0877 ± 0.0164	0.0765 ± 0.0047	0.1297 ± 0.0100	0.0480 ± 0.0038
Coefficient of differentiation for diversity	0.9789 ± 0.0044	0.9558 ± 0.0025	0.9462 ± 0.0062	0.9205 ± 0.0072
*P* value for barcoding gap examination	2.80E-07	2.10E-05	4.26E-03	1.17E-03

Both interspecific and overall mean diversities were highest for the *trn*L-F region, with values of 0.1228 ± 0.0098 and 0.1297 ± 0.0100, respectively. In contrast, the *trn*H-*psb*A region exhibited the lowest diversities, yielding values of 0.0442 ± 0.0038 and 0.0480 ± 0.0038. Conversely, intraspecific mean diversities were greatest for the *trn*H-*psb*A barcodes, measuring 0.0038 ± 0.0003, while the *trn*L-F region had the lowest, with 0.0070 ± 0.0007 (**Table 1**). The interspecific, intraspecific and overall mean diversities were moderate for the 5.8S (0.0859 ± 0.0172 for interspecific, 0.0019 ± 0.0003 for intraspecific and 0.0877 ± 0.0164 overall) and *mat*K regions (0.0731 ± 0.0045 for interspecific, 0.0034 ± 0.0002 for intraspecific and 0.0765 ± 0.0047 overall) (**Table 1**).

### 3.5 Evolutionary distinctiveness of Hong Kong orchids

**Figure 3:**
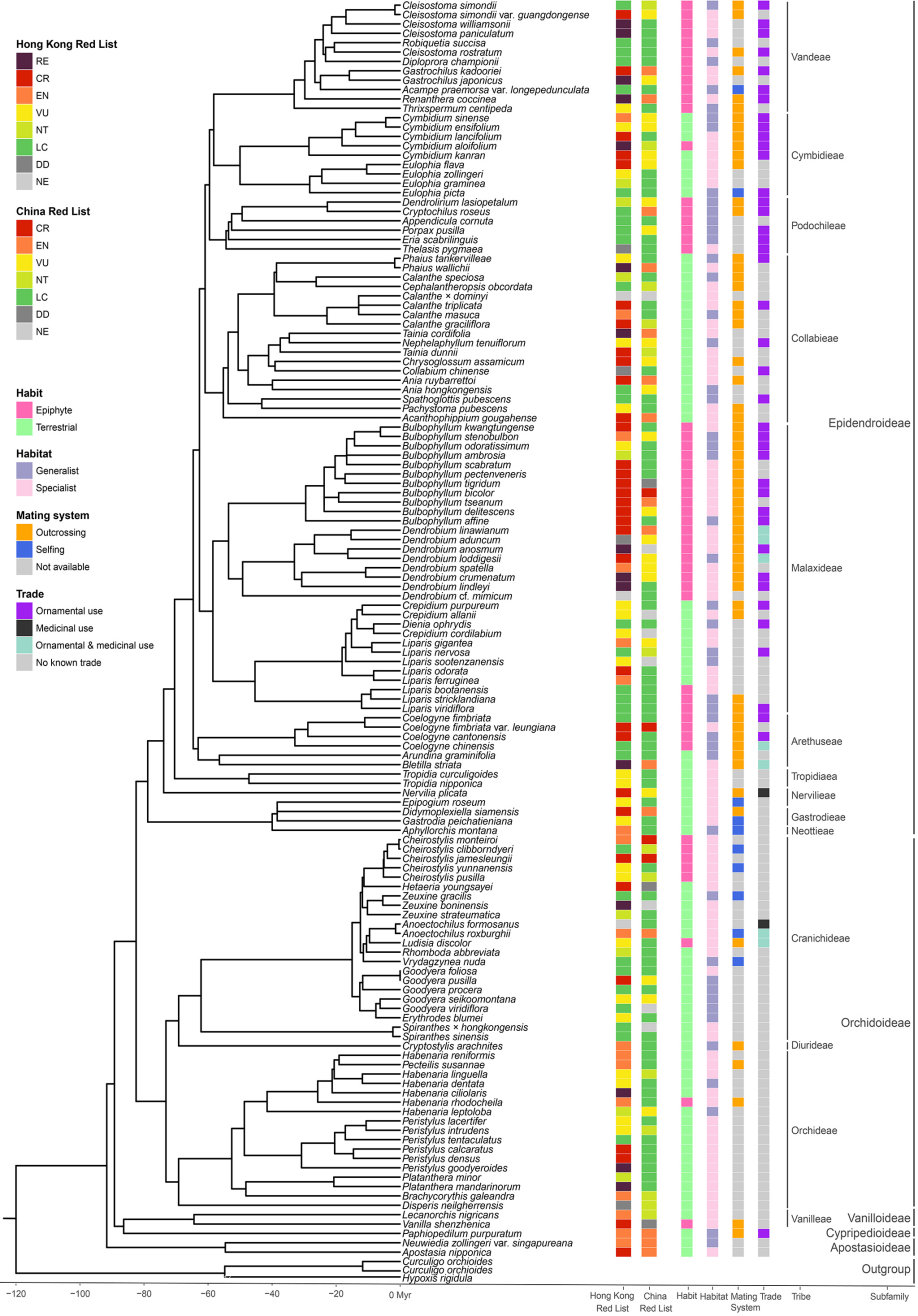
A Time-calibrated phylogenetic tree of Hong Kong orchid species, indicating Hong Kong and China Red List status and four traits (habit, habitat, mating system and trade). Red List: LC, least concern; NT, near threatened; VU, vulnerable; EN, endangered; CR, critically endangered; RE, regionally extinct in the wild; DD, data deficient and NE; not evaluated.

A time-calibrated tree containing 134 unique tip taxa (i.e. 130 species and 4 varieties, representing 62 genera, 14 tribes and all five orchid subfamilies, equivalent to 97% of all Hong Kong’s orchid taxa in this study) was reconstructed based on the ML phylogram (**Figure 3**, **Supplementary Files S5, S6**) generated from the combined data set comprising all four regions (**Supplementary File S4**). The topology of this phylogram closely reflects currently accepted phylogenetic relationships at the subfamily, tribal and generic levels. The total PD of all Hong Kong orchids included in the phylogram was 4,170.90 ± 134 million years (Myr).

**Figure 4:**
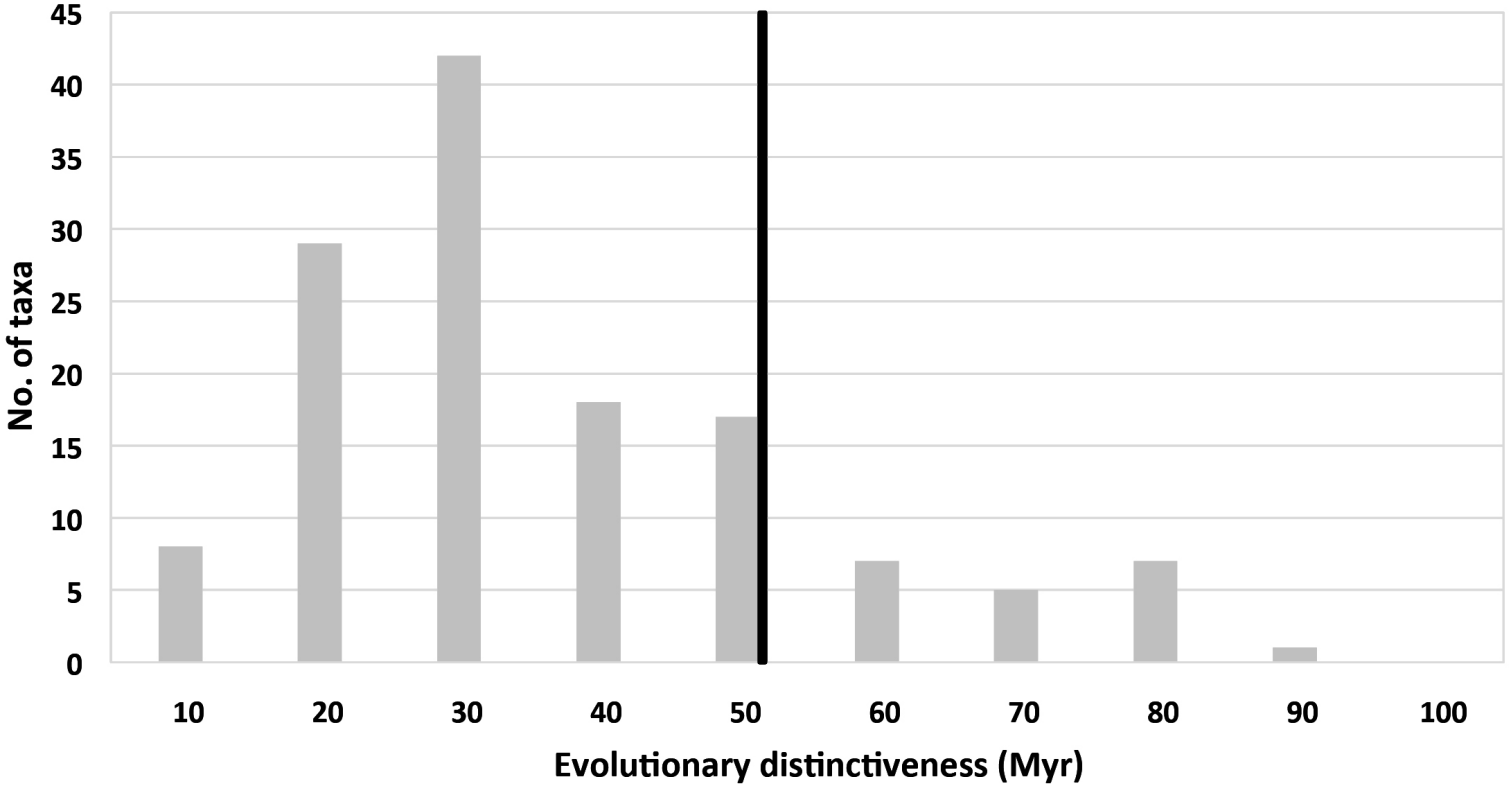
Distribution of ED scores of Hong Kong orchids. The black line indicates the threshold for 20 species with ED scores exceeding 50 Myr.

A histogram of ED scores for all Hong Kong orchids included in the phylogram reveals that most species have a relatively low ED, with the modal ED class of 20–30 Myr accounting for 31% of all taxa (42 taxa; **Figure 4**, **Supplementary File S1 Table**). Only a relatively small proportion of species (15%, 20 taxa) have relatively high ED scores exceeding 50 Myr, suggesting that the contribution of these early diverging lineages to overall phylogenetic diversity is disproportionately great (**Figure 4**). Of the highest-scoring 20 species with ED values exceeding 50 Myr, seven are threatened (i.e. 5 EN and 2 VU) in China (with *V. shenzhenica* being endemic to China) and 12 are threatened in Hong Kong (with *Bletilla striata* being regionally extinct in Hong Kong; **Table 2**). *Paphiopedilum purpuratum* is assigned the overall highest ED score of 88.4 Myr, followed by *Nervilia plicata* (Andrews) Schltr. with 78.26 Myr.

**Table 2:** Top-20 Hong Kong orchids with the highest-ranking ED scores exceeding 50 Myr.

Species	ED score (Myr)	China red list	Hong Kong red list
*Paphiopedilum purpuratum*	88.4	EN	EN
*Nervilia plicata*	78.26	VU	CR
*Vanilla shenzhenica*	76.4	DD	CR
*Lecanorchis nigricans*	76.4	NT	EN
*Disperis neilgherrensis*	74.5	NT	DD
*Cryptostylis arachnites*	74.49	LC	EN
*Apostasia nipponica*	73.12	EN	CR
*Neuwiedia zollingeri* var. *singapureana*	73.12	EN	EN
*Arundina graminifolia*	61.38	LC	LC
*Bletilla striata*	61.38	EN	RE
*Aphyllorchis montana*	61.31	LC	EN
*Tropidia curculigoides*	60.79	LC	VU
*Tropidia nipponica*	60.79	LC	VU
*Acanthophippium gougahense*	59.37	EN	CR
*Thelasis pygmaea*	57.95	LC	DD
*Brachycorythis galeandra*	55.86	NT	EN
*Eria scabrilinguis*	55.78	LC	LC
*Porpax pusilla*	54.2	VU	LC
*Dendrobium* cf. *mimicum*	53.57	LC	NA
*Appendicula cornuta*	51.78	LC	LC

In examining the relationship between ED scores and China Red List status, it was observed that the correlation coefficient was very low (*r*^2^=-0.100), indicating a weak connection. Statistical analysis revealed that this correlation was not significant (*P* = 0.954), suggesting that ED scores and China Red List status are unrelated. On the other hand, when evaluating the relationship between ED scores and Hong Kong Red List status, a low correlation coefficient was retrieved (*r*^2^ = 0.267), suggesting a slightly stronger association. Moreover, statistical analysis indicated that this correlation was significant (*P* = 0.049, 0.01< *P* < 0.05), implying that ED scores and Hong Kong Red List status are related, albeit to a limited extent.

### 3.6 Regional priority-setting based on EDGE2

**Figure 5:**
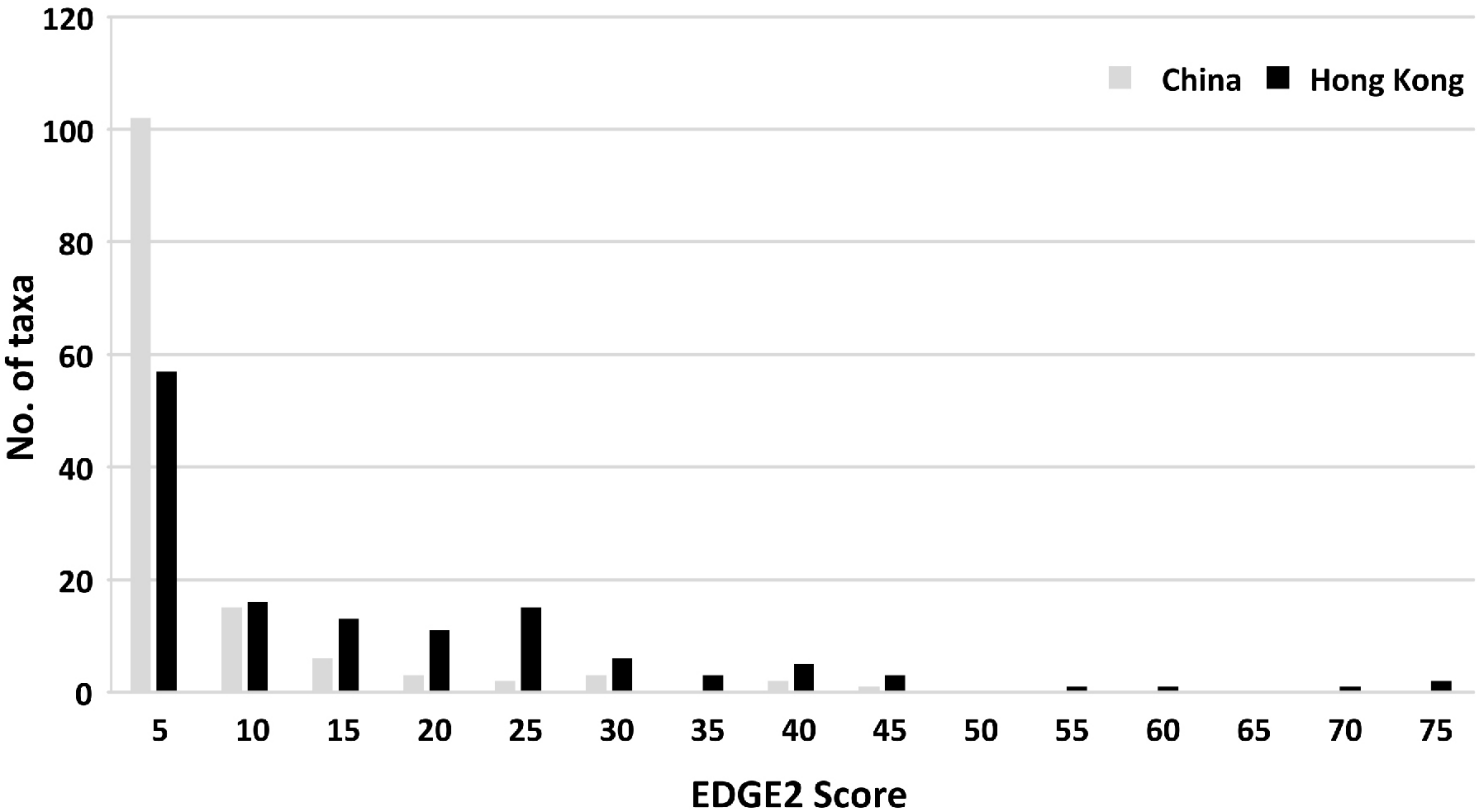
Distribution of EDGE2 scores of Hong Kong orchids.

Hong Kong Red List assessments are available for 131 (98%) of the 134 orchid taxa included in our phylogenetic tree (**Figure 3**, **Supplementary File S1 Table**). These species exhibit EDGE2 scores in the range of 0–75 Myr with the modal EDGE2 class of 0–5 Myr accounting for 43% of all taxa (57 taxa), followed by a further 12% (16 taxa) belonging to the 5–10 Myr range class (**Figure 5**, **Supplementary File S1 Table**). Notably, only five species [*Acanthophippium gougahense* (Guillaumin) Seidenf., *Bletilla striata*, *Apostasia nipponica* Masam., *Nervilia plicata* and *V. shenzhenica*] are ranked in the highest class, with an EDGE2 score of > 50 Myr; all five of these species rank among the top 20 species with the highest ED scores (**Supplementary File S1 Table**). Additionally, with the exception of *Bletilla striata*, which is extinct in the wild, the remaining four species are considered CR in Hong Kong (**Figure 3**, **Supplementary File S1 Table**).

In comparison, China Red List assessments are available for 126 (94%) of the 134 orchids included in our phylogenetic tree (**Figure 3**, **Supplementary File S1 Table**), revealing EDGE2 scores in the range of 0–45 Myr. Again, the lowest EDGE2 class (0–5 Myr) represents the modal group, comprising 76% of all taxa (102 taxa), with the next class (5–10 Myr) comprising a further 11% of all taxa (15 taxa; **Figure 5**, **Supplementary File S1 Table**). Six taxa [*V. shenzhenica*, *Acanthophippium gougahense*, *Bletilla striata*, *Neuwiedia zollingeri* var. *singapureana* (Wall. ex Baker) de Vogel, *Apostasia nipponica* and *Paphiopedilum purpuratum*] are ranked in the highest class with an EDNE score exceeding 25 Myr; all six species also rank among the top 20 species with the highest ED scores (**Supplementary File S1 Table**). Moreover, except for *V. shenzhenica*, which is considered DD, all remaining five species are considered EN in China (**Figure 3**, **Supplementary File S1 Table**). Overall, the number of taxa falling into the lowest EDGE2 class (0–5 Myr, **Figure 5**) is significantly lower based on Hong Kong Red List assessment data (57 taxa, 43%), as opposed to that utilizing China Red List data (102 taxa, 76%). Conversely, taxa with EDGE2 scores exceeding 15 Myr constitute a considerably larger proportion based on Hong Kong Red List data (61 taxa, 46%), as compared with China Red List data (17 taxa, 13%). These findings suggest that orchid populations in Hong Kong face a higher degree of threat than the same species do in China as a whole.

**Figure 6:**
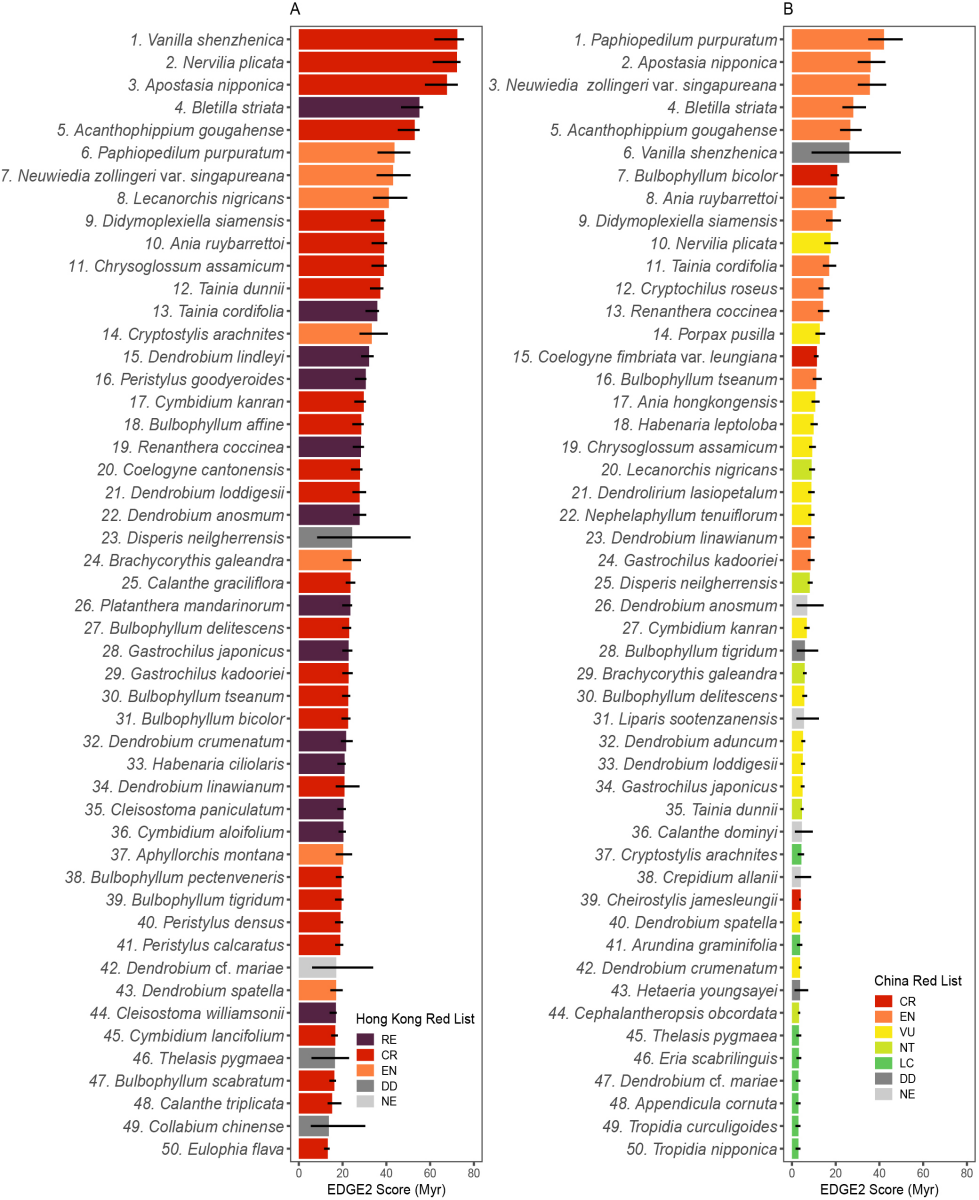
Top-50 taxa with highest EDGE2 scores based on Hong Kong regional **(A)** and China national **(B)** Red Lists. The coloured bars indicate median EDGE2 score (with colour representing Red List threat category) and the black lines indicate interquartile range.

Among the 50 species with the highest EDGE2 scores derived from the Hong Kong Red List, 33 are classified as threatened, comprising 26 CR and 7 EN taxa (**Figure 6A**). An additional 13 taxa are extinct in the wild, three are categorized as DD, and one has not been evaluated (**Figure 6A**). Of the CR taxa, *Bulbophyllum bicolor* Lindl. and *B. tseanum* (S.Y.Hu & Barretto) Z.H.Tsi are both Hong Kong near-endemics. In contrast, the 50 species with highest EDGE2 scores inferred from the national Red List of China features 31 taxa identified as threatened, including three CR, 13 EN and 15 VU taxa (**Figure 6B**). This list also contains 5 NT taxa, 6 LC taxa, 3 DD taxa and 5 taxa that have not been evaluated (**Figure 6B**). Notably, six threatened taxa, viz. *Cheirostylis jamesleungii*, *Coelogyne fimbriata* var. *leungiana*, *Dendrobium linawianum*, *Habenaria leptoloba, Tainia dunnii* and *V. shenzhenica*, are endemic to China.

Comparative assessment of these two top-50 lists reveals 31 taxa (62%) common to both (**Figures 6A, B**). Of these overlapping taxa, one CR taxon (*B. bicolor*) and three EN taxa [*Cryptostylis arachnites* (Blume) Hassk., *Neuwiedia zollingeri* var. *singapureana* and *Paphiopedilum purpuratum*] share the same threat category across both Red Lists, while 22 taxa have a higher risk designation in the Hong Kong Red List as compared with the China Red List (**Figures 6A, B**), suggesting higher threat levels at the regional scale. Notably, no taxa have a lower risk classification in the Hong Kong Red List as compared with the China national Red List (**Figures 6A, B**). The remaining five overlapping taxa have either not been evaluated or they are considered DD in one or both Red Lists (**Figures 6A, B**).

Despite considerable overlap, key differences remain between the two top 50 lists. Thus, there are 19 taxa unique to Hong Kong’s top 50 (38%; including 7 taxa that are extinct in the wild in Hong Kong, as well as 10 CR taxa, one EN and one DD taxon in the Hong Kong Red List) which do not feature in China’s ranking (**Figures 6A, B**). With the exception of *Collabium chinense*, which is considered DD in the Hong Kong Red List but LC in the China Red List, 18 of these taxa (95%) are assigned a higher risk category in Hong Kong’s regional assessment compared to the national classification (**Figures 6A, B**). Conversely, 19 taxa in China’s top 50 (38%; including two CR taxa, one EN taxon, 6 VU taxa, one NT taxon, 5 LC taxa, one DD taxon and three taxa that have not been evaluated) are absent from Hong Kong’s ranking (**Figures 6A, B**). Among these, aside from four taxa that have either not been evaluated or are categorized as DD in the China and/or Hong Kong Red Lists, six taxa share the same threat category across both Red Lists (**Figure 6A, B**). Additionally, six taxa are classified at a higher threat level in the China Red List compared to the Hong Kong Red List, while three taxa have a lower threat level in the China Red List (**Figures 6A, B**). For instance, *Cheirostylis jamesleungii* and *Coelogyne fimbriata* var. *leungiana*, both considered CR in the Hong Kong and China Red Lists, appear only in China’s top 50 list (**Figures 6A, B**). Similarly, *Dendrolirium lasiopetalum* (Willd.) S.C.Chen & J.J.Wood and *Habenaria leptoloba*, both classified as NT in the Hong Kong Red List but as VU in the China Red List, also feature solely in the China top 50 list (**Figures 6A, B**).

### 3.7 Correlation between Hong Kong Red List and four traits

At the national scale, Spearman’s rank correlation indicated a weak but statistically significant positive association between China Red List status and habitat (*r_s_* = 0.153, *P* = 0.0062; *P* < 0.05), and a negligible yet highly significant positive correlation with trade (*r_s_* = 0.009, P = 2.956E-13; *P* < 0.001). In contrast, a moderate positive correlation between China Red List status and mating system was not statistically significant (*r_s_* = 0.242, *P* = 0.2712; *P* > 0.05), while a very weak but significant negative correlation was found between China Red List status and habit (*r_s_* = –0.018, *P* = 0.0028; *P* < 0.05).

At the regional scale, highly significantly correlations were observed between Hong Kong Red List status and habit, habitat, mating system and trade. The strongest association was a moderate positive correlation with habitat (*r_s_* = 0.529, *P* = 4.0192E-23; *P* < 0.001), followed by a moderate positive correlation with mating system (*r_s_* = 0.369, *P* = 9.6715E-20; *P* < 0.001) and a weak positive correlation with trade (*r_s_* = 0.119, *P* = 3.5201E-33; *P* < 0.001). Habit again showed a very weak but highly significant negative correlation (*r_s_* = –0.041, *P* = 1.2063E-23; *P* < 0.001).

Overall, the correlations were consistently stronger and more significant for the Hong Kong Red List as compared with the China Red List, particularly for habitat and mating system.

## 4 Discussion

The orchid flora of Hong Kong faces significant threat, primarily due to habitat loss and pressures associated with collection for ornamental horticulture and TCM (Gale et al., 2014, 2019). To address these challenges effectively, accurate identification of species, whether in the wild or in trade, will be foundational for developing targeted conservation and management strategies. This study demonstrates the efficacy of certain genetic markers, specifically 5.8S, *mat*K, *trn*L-F and *trn*H-*psb*A, to clarify taxonomic ambiguities and place accessions in a robust phylogenetic framework, especially for congeneric taxa that are vegetatively highly similar in appearance and thus difficult to differentiate when not in flower [e.g. *Ania hongkongensis*. and *A. ruybarrettoi* S.Y.Hu & Barretto, *Calanthe graciliflora*, *C. masuca* (D.Don) Lindl. and *C. triplicata* (P.Willemet) Ames, *Cheirostylis clibborndyeri*, *C. monteiroi*, *Cleisostoma simondii* (Gagnep.) Seidenf. and *C. simondii* var. *guangdongense* Z.H.Tsi, *Coelogyne fimbriata* Lindl. and *C. fimbriata* var. *leungiana*, and *Phaius tankervilleae* (Banks) Blume and *P. wallichii* Lindl.]. In addition, our analysis reveals clear taxonomic relationships among Hong Kong’s 138 native orchid taxa, with Red List assessments being available for 135 of them, constituting 97% of the total. Application of the EDGE2 framework for conservation prioritization of 134 taxa (96% of the total) demonstrates the critical value robust phylogenetic data can play in translating biodiversity inventories into actionable conservation strategies. Such information is crucial for guiding conservation efforts and highlights the role of local institutions, such as Kadoorie Farm and Botanic Garden (KFBG), in compiling and managing locally representative living, herbarium and DNA collections to support regional conservation needs.

### 4.1 Phylogenetic prioritization and its role in conservation

Phylogenetic data has emerged as a critical tool for conservation prioritization, providing insights into evolutionary relationships among species and their unique genetic histories (Isaac et al., 2007; Mace et al., 2003; Purvis et al., 2000). Accurate species identification, utilizing genetic markers, is paramount in resolving taxonomic uncertainties and constructing reliable phylogenetic frameworks. This approach supports effective conservation while enabling institutions, such as KFBG, to enhance monitoring efforts for threatened species at the regional (in this case, Hong Kong) level, thereby aligning with both global initiatives (GSPC, 2002; Guerrant et al., 2004) and local frameworks such as the Hong Kong Biodiversity Strategy and Action Plan (Hong Kong Environment Bureau, 2016, 2025).

Incorporating phylogenetic information allows conservationists to identify species that represent unique evolutionary lineages, emphasizing the importance of genetic and evolutionary diversity alongside overall species richness (Faith, 1992; Forest et al., 2007). Prioritizing such species helps safeguard irreplaceable biodiversity and evolutionary history, which are often overlooked in conventional conservation frameworks that draw on Red List criteria alone (Isaac et al., 2007; Redding and Mooers, 2006). For instance, species such as the Indian Pangolin (*Manis crassicaudata* É. Geoffroy), considered VU, and the Himalayan Blue Pine (*Pinus wallichiana* A.B.Jacks), considered LC, have both experienced improved conservation outcomes due to integration of EDGE analysis into their action plans (IUCN SSC EDGE Internal Grant, 2024). However, despite its theoretical benefits, phylogenetic prioritization faces practical challenges, including incomplete or poorly resolved phylogenetic trees, particularly in regions with understudied taxa or limited genetic data (Bininda-Emonds et al., 2007; Faith, 1992). The technical demands associated with constructing and maintaining updated phylogenies further strain resource-limited initiatives, confounding their applicability in under-funded regions (Isaac et al., 2007; Sechrest et al., 2002). Addressing these barriers is essential for effectively scaling phylogenetic approaches in conservation decision-making.

### 4.2 Advancing phylogenetic prioritization through the EDGE framework

The current study applies the EDGE2 framework to evaluate the orchids of Hong Kong, demonstrating its advantages over previous approaches (such as EDGE1) through the integration of phylogenetic uncertainty and extinction probability distributions (Gumbs et al., 2023). Notably, 13 Regionally Endangered (RE) and three Data Deficient (DD) orchid species were ranked among Hong Kong’s top 50 EDGE2 species, underscoring their irreplaceable evolutionary value and precision in conservation prioritization that was unattainable under the EDGE1 framework (Gumbs et al., 2021; Veron et al., 2016). Additionally, by integrating phylogenetic complementarity (Faith, 2008) and uncertainties related to threat status (Jetz et al., 2014), the EDGE2 framework more effectively identifies species requiring immediate intervention.

Our findings reveal that 46% of Hong Kong’s orchids fall into high-ED categories (with an evolutionary distinctiveness score >15 million years), a significant deviation from the 13% observed nationally. This disparity underscores the importance of regional assessments in identifying localized evolutionary heritage at risk (Faith, 1992; Redding and Mooers, 2006; Gumbs et al., 2021). For example, among the top-20 Hong Kong orchids with ED scores exceeding 50 Myr (**Table 2**), two CR species, *Apostasia nipponica* and *Nervilia plicata*, are here designated as high priorities due to their exceptional evolutionary distinctiveness and severe threat status. In contrast, two species considered LC, *Arundina graminifolia* (D.Don) Hochr. and *Eria scabrilinguis* Lindl., are also identified as high priorities based on their considerable phylogenetic uniqueness. These findings highlight a gap in the alignment of conservation actions with evolutionary value (Isaac et al., 2007). Rapid radiations—as seen, for example, in the *Cheirostylis* clade, which includes Hong Kong endemics *C. jamesleungii* and *C. monteiroi*, as well as the *Bulbophyllum* clade, featuring near-endemics *B. tseanum* and *B. bicolor*—highlight southeast China as a region of recent diversification, underpinning the importance of local assessments of biodiversity towards the protection of regional ecosystem integrity (Erwin, 1991; Li et al., 2018). Thus, our findings emphasize the necessity of region-specific prioritization to avoid overlooking phylogenetically significant species that may be missed by broader-scale assessments (Gale et al., 2013a).

As demonstrated in its application to spatial conservation prioritization (Pipins et al., 2024), the EDGE2 framework optimizes conservation zonation strategies by identifying key geographic areas, termed “EDGE Zones”, delineated for their high concentrations of threatened and evolutionarily distinct species, thereby maximizing the preservation of unique evolutionary history in regions facing significant human pressure and insufficient protection. The EDGE2 framework also highlights discrepancies between conservation priorities identified in Hong Kong and China, reflecting scale-dependent threats and varying policy contexts (Veron et al., 2016). Although *Coelogyne fimbriata* var. *leungiana* and *Cheirostylis jamesleungii* are both regarded as Critically Endangered (CR) in Hong Kong and China, their exclusion from Hong Kong’s top-50 EDGE2 list despite being included in the top-50 list derived from China Red List assessments exemplifies divergent regional and national considerations. Such inconsistencies underscore the challenges of reconciling threat-based assessments with phylogenetic distinctness criteria, as evolutionary uniqueness and geographic endangerment may not always match.

To harmonize efforts, adaptive strategies must integrate phylogenetic metrics with localized threats (Purvis et al., 2000). In a previous threat-based prioritization exercise for the Hong Kong orchids (Gale et al., 2013b), species included in the resulting top-10 were selected primarily on the grounds of high extinction risk (e.g., CR, EN), very restricted ranges and the global significance of their local populations. The findings of the present EDGE2-based approach exhibit a 30% overlap with that top-10 list, with *Acanthophippium gougahense* (CR), *Ania ruybarrettoi* (CR) and *Paphiopedilum purpuratum* (EN) being included in both. The key commonality is that both frameworks prioritize species facing a high risk of extinction, with the major difference being that the EDGE2 approach also captures evolutionary distinctiveness. This highlights how phylogenetic metrics can shift focus towards the safeguarding of unique evolutionary histories.

By translating EDGE2 scores into actionable conservation priorities, stakeholders can direct resources toward the most evolutionarily distinct and threatened species, enabling targeted interventions such as habitat restoration and anti-poaching patrols, that directly improve the chances of species survival and persistence (Pipins et al., 2024; Secretariat of the Convention on Biological Diversity, 2021). Furthermore, these priorities support the development of evidence-based trade mitigation strategies, including policy reforms and alternative livelihood programs, which reduce exploitation pressure while engaging local communities in conservation efforts (Baselga et al., 2025). Integrated conservation planning, informed by EDGE2 metrics, thus facilitates a coordinated response to specific threats, from habitat loss to illegal trade, and enhances the efficacy of population rescue and recovery programs.

Although EDGE2 improves upon earlier metrics by incorporating phylogenetic uncertainty and extinction risk, it may undervalue recently radiated clades that play a vital role in maintaining ecosystem integrity by contributing phylogenetic and functional diversity and thus be of high ecological significance (Forest et al., 2015), when its scope is extended to encompass ecosystems or the entire ranges of phylogenetically defined plant and animal groups. Future iterations could also integrate functional traits (e.g., functional uniqueness; Pavoine and Ricotta, 2021) and ecological interactions (Margules and Pressey, 2000) to better capture broader biodiversity value (Purvis et al., 2000). We recommend that regional conservation plans adopt EDGE2 scores as a key prioritization metric whilst extending their application to functional uniqueness, with emphasis on protecting recently radiated yet ecologically significant clades that anchor regional ecosystem integrity. Given increasing population fragmentation and escalating threats from climate change and trade, it is critical to protect both high-EDGE2 species, which safeguard evolutionarily distinct units and irreplaceable genetic heritage (Gumbs et al., 2023; Isaac et al., 2007), and rapidly diversified lineages that underpin regional phylogenetic and functional diversity while maintaining ecosystem resilience (Forest et al., 2015; Li et al., 2018). Furthermore, in alignment with global frameworks (e.g., GSPC, 2002; Secretariat of the Convention on Biological Diversity, 2021), conservation actions should seek to protect species with specialized growth habits, for instance, lithophytic species such as *Bulbophyllum bicolor*, vinous species such as *V. shenzhenica*, and fully or partially myco-heterotrophic species such as *Didymoplexiella siamensis* and *Nervilia plicata*. By integrating regional initiatives with phylogenetic distinctiveness, conservation efforts can maximize the retention of evolutionary and functional diversity while addressing the localized drivers of decline (Brooks et al., 2006).

### 4.3 Regional Red List assessments and hyper-localized threats

The IUCN’s Red List of Threatened Species serves as the most comprehensive information source regarding the conservation status of animal, fungal and plant species globally (IUCN, 2024). It plays a crucial role in conservation priority-setting by evaluating species extinction risk, ultimately informing resource allocation and shaping policy decisions (Butchart et al., 2010; Mace et al., 2008; Rodrigues et al., 2006). Our findings indicate that the conservation status of 92 species is higher in the Hong Kong Red List than in the China Red List, and the absence of 17 regionally extinct species in Hong Kong from the national CR category further highlights the role of regional Red Lists in capturing hyper-localized threats. This pattern is echoed in Australia, where the regional Red List for New South Wales identifies *Diuris ochroma* D.L.Jones (Orchidaceae) as EN, whereas the national list assesses it as VU (New South Wales Government, 2021). Similarly, in Brazil, the state-level Red List for São Paulo evaluates *Melocactus violaceus* Pfeiff. (Cactaceae) as extinct in the wild, although it is categorized as VU in both national and global Red Lists. In the state of Rio de Janeiro, severe threats, including degradation and fragmentation of natural habitats, have caused significant local declines (Martinelli and Moraes, 2013). Such instances illustrate the importance of regional Red Lists in addressing ecological and biogeographical factors that may not be captured at national level, especially in biodiversity-rich ecoregions where conservation priorities may diverge (Gärdenfors et al., 2001; Miller et al., 2007). Strengthening regional Red Lists entails developing standardized protocols aligned with national frameworks while emphasizing local ecological nuances to ensure that all species at risk of genetic elimination are addressed.

The process of Red-Listing across global, national and regional levels is inherently resource- and labour-intensive, constituting an academically undervalued intellectual exercise (Keith et al., 2013; Li et al., 2018; Lindenmayer and Likens, 2011). Specifically, the development and maintenance of regional Red Lists necessitate detailed localized data, which is often incomplete or scarce, resulting in gaps in species assessments (Boitani et al., 2015; Rodrigues et al., 2006). Consequently, addressing data gaps and bolstering collaboration among stakeholders is critical for enhancing the role of regional Red Lists in biodiversity conservation. Ultimately, dedicated funding from governments and other stakeholders, as well as increased participation from citizen scientists in collecting and analysing sightings data (Cooper et al., 2007; Gardiner and Bachman, 2016), are crucial to expedite the Red Listing process for neglected taxa and ensure that regional ecological conditions are accurately reflected.

### 4.4 Ecological and anthropogenic drivers: correlating Red List status and specific traits in Hong Kong

The positive correlations observed (e.g., *rs*_habitat_ = 0.529, *rs*_mating system_ = 0.369, *rs*_trade_ = 0.119) indicate that ecological and anthropogenic drivers are critical in understanding the conservation status of Hong Kong’s orchids. This correlation pattern is notably reflected in habitat specialization. Our results reveal a 15% disparity in the proportion of threatened species between specialists and generalists (60% of specialists vs. 45% of generalists classified as threatened), highlighting the heightened vulnerability of species with narrow and specialized habitat requirements, such as *Ania ruybarrettoi* and *Dendrobium aduncum*. Such vulnerability is consistent with a well-established global pattern whereby habitat specialists are disproportionately threatened, showing heightened susceptibility to anthropogenic disturbance and climate change due primarily to narrow environmental tolerances (Early and Sax, 2014; Williams et al., 2008). A parallel trend emerges in reproductive ecology. Outcrossing species, which often rely on specific pollinators (Fenster et al., 2004; Gaskett, 2011; Johnson and Steiner, 2000) and face increased reproductive challenges in degraded habitats (Sechrest et al., 2002; Veron et al., 2016), exhibit a higher threat risk. In our study, this elevated risk is exemplified by outcrossing taxa such as *Bulbophyllum kwangtungense* and *Eulophia flava* (Lindl.) Hook.f. Our findings allow us to quantify this disparity as a 17% greater risk as measured by the proportion of threatened species between outcrossing and selfing groups (67% of outcrossing species vs. 50% of selfing species classified as threatened). Beyond these primary factors, specialized mycorrhizal associations, which constrain recruitment and distribution, can further exacerbate such vulnerabilities (McCormick and Jacquemyn, 2014; Rasmussen and Rasmussen, 2009). The severity of these threats is further underscored by patterns in regional extinction, which appear to be confined to specialized taxa: among specialists, 17 taxa (17 of 91 specialists; 9% of the total specialists) are regionally extinct, whereas no generalist is. Similarly, among outcrossing taxa, 7 taxa (7 of 61 outcrossing taxa; 11% of the total outcrossing taxa) are extinct, compared to none among selfing groups. Collectively, these data underscore the necessity for ecological trait-driven conservation strategies that prioritize orchids based on their unique ecological and evolutionary characteristics (Gale et al., 2019; Li et al., 2018; Thompson et al., 2023), ultimately improving conservation planning and interventions.

Building on these trait–threat correlations, the specific case of trade pressure exemplifies the critical, yet often under-represented, interplay between ecological traits and anthropogenic drivers in conservation (Gale et al., 2019; Purvis et al., 2000). In Hong Kong, ornamental taxa constitute 96% of traded orchids, with charismatic genera such as *Cymbidium* (e.g., *C. sinense* Willd., celebrated in Lunar New Year displays) and *Dendrobium* (e.g., *D. loddigesii*, prized for both horticultural and medicinal use) being particularly targeted due to their cultural and medicinal appeal, thereby intensifying exploitation pressures (Li et al., 2018; Phillips et al., 2020). However, while trade correlates with endangerment, disparities in Red List status cannot be attributed solely to commerce, with habitat degradation and climate change also amplifying vulnerabilities in a mutually reinforcing manner (Brook et al., 2008; Mantyka-Pringle et al., 2012; Isaac et al., 2007; Sechrest et al., 2002). Species with less commercially attractive characteristics, such as those with small, dull and non-showy flowers (e.g., *Thelasis pygmaea*) or challenging cultivation requirements (e.g., myco-heterotrophic species, *Lecanorchis nigricans* and *Didymoplexiella siamensis*), are often overlooked in trade, inadvertently reducing harvesting pressure. This paradox suggests that ecological specialization can, in some cases, buffer against anthropogenic pressures precisely because vulnerability is shaped by the interplay between species traits and market forces, underscoring the need for conservation strategies that integrate market regulations with habitat protections (Secretariat of the Convention on Biological Diversity, 2021).

The regional specificity of the combination of these ecological drivers and localized trade pressures in Hong Kong further emphasizes the importance of localized conservation strategies. The stronger trait–threat correlations observed at the regional scale compared to the national level in this study suggest that local assessments more sensitively capture ecological relationships, likely due to higher-resolution data and locally intensified human-induced threats (e.g. horticultural and medicinal trade) (Gärdenfors et al., 2001; Miller et al., 2007; Swarts and Dixon, 2009). This highlights the value of regionally tailored evaluations in identifying context-specific drivers of species vulnerability. Additionally, the stark discrepancies in Red List assessments at the Hong Kong and national levels highlight the intensified threats faced by more specialized orchids, particularly as urbanization and climate change escalate (Forest et al., 2007; Purvis et al., 2000). Therefore, by employing a trait-driven approach and focusing on ecological nuances, conservation efforts can be refined to prioritize not only the most threatened species but also the unique habitats that sustain them, while simultaneously implementing regulations to mitigate trade-driven pressures (Gale et al., 2018; Redding and Mooers, 2006).

### 4.5 Strategic recommendations for regional conservation

Regional conservation efforts are essential for addressing distinct ecological challenges within certain areas, complementing broader national and global strategies. Our analyses reveal that Hong Kong’s orchid populations are significantly more threatened than the same species in China, as demonstrated by comparative evaluation of Red Lists, ecological factors, anthropogenic threat and phylogenetic metrics such as Evolutionary Distinctiveness (ED) and EDGE2 scores. Prioritizing regional conservation will help address the complex interactions between species, habitat connectivity and landscape-level processes, including gene flow, population dynamics, and ecological interactions with pollinators and mycorrhizal fungi, that are essential for the long-term persistence of biodiversity (Aguilar et al., 2008; Haddad et al., 2015).

Moreover, the significant correlation between the regional Red List status of Hong Kong’s orchids and their habitat specificity and mating system is likely to also impact reproductive success, population viability and genetic diversity, factors that are known to be critically affected by habitat degradation and loss (Aguilar et al., 2008; Fahrig, 2003; Haddad et al., 2015). Consequently, safeguarding specific habitats, such as forest canopies for epiphytic orchids and rocky outcrops for lithophytic species, becomes imperative. Additional strategies should focus on maximizing species and ecosystem health (Ervin et al., 2010; Erwin, 1991; Li et al., 2018) across landscapes, facilitating gene flow and creating optimal conditions for ecological interactions, including those with specialized pollinators (Ackerman et al., 2023; Vitt et al., 2023) and mycorrhizal fungi (Batty et al., 2002; Wang et al., 2021). Implementing landscape-level interventions could significantly mitigate orchid population decline, ultimately benefiting overall biodiversity conservation.

Building on the findings derived from our analysis, we propose five integrated strategies aimed at enhancing resilience against anthropogenic threats and conserving plant biodiversity in a targeted, regional context:

- Phylogenetic reconstruction and taxonomic clarification
  We advocate for expanded DNA barcoding to resolve taxonomic uncertainties in under-represented groups, using universal and cost-effective markers (e.g., *mat*K and ITS) to improve species identification and regulate trade-driven pressures (e.g., *Cymbidium* and *Dendrobium*). This approach meets immediate conservation demands, while looking ahead, the adoption of genome sequencing approaches (e.g., genome skimming) would generate substantially larger datasets at increasingly lower cost than traditional sequencing, enabling more robust phylogenetic resolution at different evolutionary scales and providing multi-disciplinary data applicable to diverse conservation scenarios. Global collaboration is essential to address gaps in less-studied clades (e.g., *Cheirostylis* Blume and *Liparis*), which are critical for biodiversity conservation but lack genomic data. Regional institutions such as KFBG can strengthen these efforts by contributing to global databases, ensuring conservation strategies are phylogenetically informed.
- Enhanced Red List assessments
  Standardizing and routinely updating regional Red List classifications will ensure that vulnerable species receive adequate attention by incorporating localized threat information such as deforestation rates and fragmentation. This will refine species vulnerability assessments and align them with national conservation policy.
- Adoption of the EDGE2 framework
  Focusing on species with high EDGE2 scores, such as evolutionarily unique orchids, for habitat restoration and *ex situ* conservation will help protect irreplaceable genetic diversity, while simultaneously prioritizing rapidly diversified clades that play key roles in maintaining regional ecosystem integrity. Restoring ecological networks that support specialized taxa (e.g. habitat specialists, lithophytes, myco-heterotrophic species, and other orchids with narrow ecological niches) will be key (Bascompte et al., 2006), as will integrating EDGE2 metrics into regional policies to ensure alignment with national and global biodiversity targets.
- Trait-driven conservation
  To develop targeted habitat-specific interventions, it is essential to evaluate and utilize the correlations of threat status with both ecological factors and trade pressure, for example, protecting rocky outcrops for lithophytic orchids (Zhang et al., 2023). Additionally, we should address reproductive challenges by maintaining pollinator pathways (Ollerton et al., 2011) and ensuring habitat connectivity (Damschen et al., 2019; Haddad et al., 2015), particularly for species that are sensitive to environmental fragmentation.
- Global synergy and cross-regional collaboration
  Collaborative partnerships among institutions including botanic gardens, universities and herbaria will strengthen conservation effectiveness through the sharing of phylogenetic and threat data. Adopting standardized monitoring models strengthens alignment with global frameworks, including the Global Strategy for Plant Conservation (GSPC, 2002) and the State of the World’s Plants and Fungi (Antonelli et al., 2023). Exemplary models range from comprehensive taxonomic assessments such as the Global Tree Assessment (BGCI, 2023) and large-scale data infrastructures such as the Global Biodiversity Information Facility (GBIF; Edwards et al., 2000), to more regionally focused databases such as RAINBIO (a mega-database of tropical African vascular plants distributions; Dauby et al., 2016). These initiatives provide the essential data backbone, enabling regional Red Lists to effectively inform national policies, underpin strategies for regulating trade-related threats, and foster community engagement for habitat connectivity.

The implementation of regional conservation strategies is crucial for protecting local floras, such as Hong Kong’s threatened orchids. Our proposed initiatives, including phylogenetic reconstruction, enhanced assessments and global collaboration, could systematically address challenges related to regional habitat degradation and trade, while aligning with global biodiversity conservation objectives, contributing to a resilient ecological landscape (Folke et al., 2004; Oliver et al., 2015) for future generations.

## 5 Conclusions

Hong Kong’s orchid flora faces severe threat in the form of habitat loss and illegal harvesting, with over half of the species threatened at the regional level. The application of the EDGE2 framework effectively identifies conservation priorities by integrating evolutionary distinctiveness and extinction risk, highlighting species with unique evolutionary histories that are under-represented in traditional Red List assessments. Significant positive correlations were found between threat status and key ecological traits such as habitat specificity and mating system, as well as trade pressure. The findings underscore the urgent need for region-specific conservation strategies that combine phylogenetic metrics, trait-based approaches, and enhanced Red List assessments to preserve Hong Kong’s unique orchid diversity and evolutionary heritage.

## Data Availability

The datasets presented in this study are available in public online repositories, namely Zenodo and NCBI. The GenBank accession numbers are as follows: NCBI, PX054990–PX055338, PX052792–PX053203, PX052357–PX052782, PX050573–PX050951, and PV984418–PV984421.
