# Supplementary material for: Hong Kong orchids on the EDGE: a phylogenetic framework for conservation planning, trade mitigation and population rescue
Source: Front Plant Sci. 2026 Apr 28;17:1801915. doi: 10.3389/fpls.2026.1801915 (PMC13161184; doi:10.3389/fpls.2026.1801915)
Supplement: Supplementary file 2 [file Table2.docx]

S2A: 5.8S raw matrix.

#NEXUS

begin taxa;

dimensions ntax=990;

taxlabels

Acampe_praemorsa_var_longepedunculata_AB217526

Acampe_praemorsa_var_longepedunculata_KJ733385

Acampe_praemorsa_var_longepedunculata_KY966408

Acampe_praemorsa_var_longepedunculata_PK12086

Acampe_praemorsa_var_longepedunculata_PK12159

Acampe_praemorsa_var_longepedunculata_SG1199

Acanthophippium_gougahense_KFBG3161

Acanthophippium_gougahensis_KY966409

Ania_hongkongensis_KF560537

Ania_hongkongensis_KFBG4612

Ania_hongkongensis_KFBG4613

Ania_hongkongensis_KM025146

Ania_hongkongensis_KM025147

Ania_hongkongensis_KM025148

Ania_hongkongensis_KY966415

Ania_hongkongensis_PK12027

Ania_hongkongensis_SG1231

Ania_hongkongensis_SG1343

Ania_ruybarrettoi_KFBG43

Ania_ruybarrettoi_KM025152

Ania_ruybarrettoi_KY966416

Ania_ruybarrettoi_SG1395

Anoectochilus_formosanus_AY052780

Anoectochilus_formosanus_GQ328777

Anoectochilus_formosanus_GQ396668

Anoectochilus_formosanus_KR815833

Anoectochilus_formosanus_KR815839

Anoectochilus_formosanus_PK12215

Anoectochilus_roxburghii_EU817408

Anoectochilus_roxburghii_GQ328774

Anoectochilus_roxburghii_KF425501

Anoectochilus_roxburghii_KF425502

Anoectochilus_roxburghii_KF425503

Anoectochilus_roxburghii_KR815828

Anoectochilus_roxburghii_KR815829

Anoectochilus_roxburghii_KR815830

Anoectochilus_roxburghii_KR815831

Anoectochilus_roxburghii_KR815832

Anoectochilus_roxburghii_KR815836

Anoectochilus_roxburghii_KR815837

Anoectochilus_roxburghii_KR815838

Anoectochilus_roxburghii_KY966417

Anoectochilus_roxburghii_PK12043

Anoectochilus_roxburghii_PK12044

Anoectochilus_roxburghii_PK12068

Anoectochilus_roxburghii_PK12069

Anoectochilus_roxburghii_SG1219

Aphyllorchis_montana_FJ454867

Aphyllorchis_montana_PK12147

Aphyllorchis_montana_PK12148

Aphyllorchis_montana_SG1010

Apostasia_nipponica_PK12273

Apostasia_nipponica_PK12274

Appendicula_cornuta_AF521073

Appendicula_cornuta_KY239261

Appendicula_cornuta_KY966418

Appendicula_cornuta_KY966419

Appendicula_cornuta_PK12064

Appendicula_cornuta_PK12065

Arundina_graminifolia_AF273335

Arundina_graminifolia_AF302727

Arundina_graminifolia_AF461461

Arundina_graminifolia_JN114437

Arundina_graminifolia_JN114438

Arundina_graminifolia_JN114439

Arundina_graminifolia_JN114440

Arundina_graminifolia_KY966420

Arundina_graminifolia_SG1008

Arundina_graminifolia_SG1206

Arundina_graminifolia_SG1295

Bletilla_striata_AF273334

Bletilla_striata_AF461466

Bletilla_striata_EU100762

Bletilla_striata_KF560509

Bletilla_striata_KF560540

Bletilla_striata_KF680524

Bletilla_striata_KF698622

Bletilla_striata_KF698623

Bletilla_striata_KFBG2048

Bletilla_striata_KP866820

Bletilla_striata_KP866821

Bletilla_striata_KP866822

Bletilla_striata_KP866823

Bletilla_striata_KP866824

Bletilla_striata_KP866825

Bletilla_striata_KP866826

Bletilla_striata_KP866827

Bletilla_striata_KP866828

Bletilla_striata_KT119555

Bletilla_striata_KT338650

Bletilla_striata_KY966421

Brachycorythis_galeandra_PK12195

Brachycorythis_galeandra_SG1261

Bulbophyllum_affine_EF195916

Bulbophyllum_affine_KC568305

Bulbophyllum_affine_KF866246

Bulbophyllum_affine_KFBG412

Bulbophyllum_affine_KX455815

Bulbophyllum_affine_KY966422

Bulbophyllum_affine_MK164425

Bulbophyllum_affine_SG1606

Bulbophyllum_affine_SG1607

Bulbophyllum_ambrosia_JN619413

Bulbophyllum_ambrosia_KC568306

Bulbophyllum_ambrosia_KY966423

Bulbophyllum_ambrosia_KY966424

Bulbophyllum_ambrosia_KY966425

Bulbophyllum_ambrosia_KY966426

Bulbophyllum_ambrosia_MK164427

Bulbophyllum_ambrosia_PK12090

Bulbophyllum_ambrosia_PK12092

Bulbophyllum_ambrosia_SG1221

Bulbophyllum_bicolor_CL10

Bulbophyllum_bicolor_FT28

Bulbophyllum_bicolor_KFBG2210

Bulbophyllum_bicolor_KFBG3073

Bulbophyllum_bicolor_KFBG433A

Bulbophyllum_bicolor_KFBG445

Bulbophyllum_bicolor_KY022455

Bulbophyllum_bicolor_KY966430

Bulbophyllum_bicolor_KY966431

Bulbophyllum_bicolor_KY966432

Bulbophyllum_bicolor_LMP19

Bulbophyllum_bicolor_MK164432

Bulbophyllum_bicolor_MK164433

Bulbophyllum_bicolor_PSL45

Bulbophyllum_bicolor_TMS05

Bulbophyllum_bicolor_TT43

Bulbophyllum_delitescens_KY966439

Bulbophyllum_delitescens_KY966440

Bulbophyllum_delitescens_KY966441

Bulbophyllum_delitescens_KY966442

Bulbophyllum_delitescens_MK164448

Bulbophyllum_delitescens_SG1286

Bulbophyllum_delitescens_SG1287

Bulbophyllum_delitescens_SG1288

Bulbophyllum_kwangtungense_JN619414

Bulbophyllum_kwangtungense_KFBG27001

Bulbophyllum_kwangtungense_KFBG2798

Bulbophyllum_kwangtungense_KFBG2820

Bulbophyllum_kwangtungense_MK164466

Bulbophyllum_kwangtungense_PK12070

Bulbophyllum_odoratissimum_FJ428222

Bulbophyllum_odoratissimum_FJ428223

Bulbophyllum_odoratissimum_HQ114230

Bulbophyllum_odoratissimum_KF866242

Bulbophyllum_odoratissimum_KP265007

Bulbophyllum_odoratissimum_KY966466

Bulbophyllum_odoratissimum_KY966467

Bulbophyllum_odoratissimum_MK164483

Bulbophyllum_odoratissimum_PK12119

Bulbophyllum_odoratissimum_SG1275

Bulbophyllum_pectenveneris_JN619418

Bulbophyllum_pectenveneris_KY966470

Bulbophyllum_pectenveneris_KY966471

Bulbophyllum_pectenveneris_MK164486

Bulbophyllum_scabratum_JN619409

Bulbophyllum_scabratum_KY966453

Bulbophyllum_scabratum_MK164471

Bulbophyllum_scabratum_PK12041

Bulbophyllum_scabratum_PK12126

Bulbophyllum_scabratum_PK12127

Bulbophyllum_stenobulbon_KFBG2806

Bulbophyllum_stenobulbon_MK164513

Bulbophyllum_stenobulbon_PK12111

Bulbophyllum_stenobulbon_PK12112

Bulbophyllum_stenobulbon_PK12113

Bulbophyllum_stenobulbon_SG1226

Bulbophyllum_tigridum_KFBG468

Bulbophyllum_tigridum_KX455820

Bulbophyllum_tigridum_MK164520

Bulbophyllum_tigridum_SG1310

Bulbophyllum_tseanum_MK164524

Bulbophyllum_tseanum_SG1272

Bulbophyllum_tseanum_SG1616

Calanthe_dominyi_SG1359

Calanthe_graciliflora_AY882608

Calanthe_graciliflora_KF560484

Calanthe_graciliflora_KF560495

Calanthe_graciliflora_PK12206

Calanthe_graciliflora_PK12207

Calanthe_graciliflora_SG1225

Calanthe_masuca_KFBG11

Calanthe_masuca_PK12110

Calanthe_masuca_SG026

Calanthe_masuca_SG1360

Calanthe_masuca_SG1361

Calanthe_speciosa_KFBG136

Calanthe_speciosa_KY951546

Calanthe_speciosa_PK12168

Calanthe_speciosa_PK12169

Calanthe_speciosa_SG1313

Calanthe_speciosa_SG1368

Calanthe_triplicata_AY882614

Calanthe_triplicata_KF560480

Calanthe_triplicata_KF560491

Calanthe_triplicata_KFBG601

Calanthe_triplicata_KM025154

Calanthe_triplicata_KY966491

Calanthe_triplicata_SG1311

Cephalantheropsis_obcordata_KF560490

Cephalantheropsis_obcordata_KFBG2520

Cephalantheropsis_obcordata_KY966494

Cephalantheropsis_obcordata_KY966495

Cephalantheropsis_obcordata_PK12079

Cephalantheropsis_obcordata_PK12080

Cephalantheropsis_obcordata_PK12081

Cephalantheropsis_obcordata_SG1208

Cephalantheropsis_obcordata__KFBG140

Cephalantheropsis_obcordata__SG1209

Cheirostylis_clibborndyeri_KY966496

Cheirostylis_clibborndyeri_PK12096

Cheirostylis_clibborndyeri_SG1349

Cheirostylis_jamesleungii_PK12205

Cheirostylis_monteiroi_PK12093

Cheirostylis_monteiroi_PK12094

Cheirostylis_monteiroi_SG1344

Cheirostylis_pusilla_HK43263

Cheirostylis_yunnanensis_KT343978

Cheirostylis_yunnanensis_PK12097

Cheirostylis_yunnanensis_SG1227

Cheirostylis_yunnanensis_SG1228

Cheirostylis_yunnanensis_SG1229

Chrysoglossum_assamicum_SG1622

Chrysoglossum_assamicum_SG1623

Cleisostoma_paniculatum_KFBG516

Cleisostoma_paniculatum_KJ733401

Cleisostoma_paniculatum_KT223752

Cleisostoma_rostratum_KFBG789

Cleisostoma_rostratum_KJ733404

Cleisostoma_rostratum_KY966502

Cleisostoma_rostratum_PK12089

Cleisostoma_rostratum_PK12101

Cleisostoma_rostratum_PK12158

Cleisostoma_rostratum_SG1200

Cleisostoma_rostratum_SG1301

Cleisostoma_simondii_KJ733405

Cleisostoma_simondii_KY966503

Cleisostoma_simondii_KY966504

Cleisostoma_simondii_MG822849

Cleisostoma_simondii_PK12176

Cleisostoma_simondii_SG1314

Cleisostoma_simondii_SG1327

Cleisostoma_simondii_SG1328

Cleisostoma_simondii_SG1329

Cleisostoma_simondii_SG1330

Cleisostoma_simondii_var_guangdongense_KFBG2212

Cleisostoma_simondii_var_guangdongense_KJ733406

Cleisostoma_williamsonii_KJ733409

Coelogyne_cantonensis_KY966649

Coelogyne_cantonensis_SG1239

Coelogyne_cantonensis_SG1339

Coelogyne_cantonensis_SG1388

Coelogyne_chinensis_EU592035

Coelogyne_chinensis_KY966650

Coelogyne_chinensis_KY966651

Coelogyne_chinensis_PK12114

Coelogyne_chinensis_PK12115

Coelogyne_chinensis_SG1232

Coelogyne_chinensis_SG1251

Coelogyne_fimbriata_AF302745

Coelogyne_fimbriata_EU441205

Coelogyne_fimbriata_JF422074

Coelogyne_fimbriata_KFBG411

Coelogyne_fimbriata_KFBG523

Coelogyne_fimbriata_KR857330

Coelogyne_fimbriata_KY966506

Coelogyne_fimbriata_SG1059

Coelogyne_fimbriata_SG1061F

Coelogyne_fimbriata_SG1062D

Coelogyne_fimbriata_SG1079

Coelogyne_fimbriata_var_leungiana_KFBG640

Coelogyne_fimbriata_var_leungiana_KY966507

Coelogyne_fimbriata_var_leungiana_SG1058A

Collabium_chinense_KF560544

Collabium_chinense_KM025156

Crepidium_acuminatum_AB290884

Crepidium_acuminatum_JN114478

Crepidium_acuminatum_JN114479

Crepidium_acuminatum_JN114480

Crepidium_acuminatum_JN114481

Crepidium_acuminatum_JN114482

Crepidium_acuminatum_KJ459274

Crepidium_acuminatum_KX277725

Crepidium_acuminatum_MF287967

Crepidium_allanii_KFBG4610

Crepidium_allanii_KFBG4611

Crepidium_purpureum_PK12185

Crepidium_purpureum_SG1193

Crepidium_purpureum_SG1194

Crepidium_purpureum_SG1379

Cryptochilus_roseus_KFBG2118

Cryptochilus_roseus_KY239358

Cryptochilus_roseus_KY966513

Cryptochilus_roseus_KY966514

Cryptochilus_roseus_PK12087

Cryptochilus_roseus_PK12088

Cryptochilus_roseus_PK12187

Cryptostylis_arachnites_PK12188

Cryptostylis_arachnites_SG027

Cryptostylis_arachnites_SG1142

Cryptostylis_arachnites_SG1380

Curculigo_orchioides_PK12054

Cymbidium_aloifolium_AF284695

Cymbidium_aloifolium_AF470526

Cymbidium_aloifolium_AJ300269

Cymbidium_aloifolium_JF729014

Cymbidium_aloifolium_JN114485

Cymbidium_aloifolium_JN114486

Cymbidium_aloifolium_KFBG2049

Cymbidium_aloifolium_KFBG2205

Cymbidium_ensifolium_AF284716

Cymbidium_ensifolium_AF284717

Cymbidium_ensifolium_AF470496

Cymbidium_ensifolium_AF470512

Cymbidium_ensifolium_AJ300273

Cymbidium_ensifolium_FJ899748

Cymbidium_ensifolium_HQ263134

Cymbidium_ensifolium_JX399078

Cymbidium_ensifolium_KJ597843

Cymbidium_ensifolium_KJ597844

Cymbidium_ensifolium_KJ597845

Cymbidium_ensifolium_KJ597846

Cymbidium_ensifolium_KT338675

Cymbidium_ensifolium_PK12208

Cymbidium_ensifolium_SG1214

Cymbidium_ensifolium_SG1278

Cymbidium_ensifolium_SG1285

Cymbidium_kanran_AF284720

Cymbidium_kanran_AF470495

Cymbidium_kanran_AJ300279

Cymbidium_kanran_AJ300280

Cymbidium_kanran_AJ300281

Cymbidium_kanran_AJ300282

Cymbidium_kanran_HQ263140

Cymbidium_kanran_JX202654

Cymbidium_kanran_KF560534

Cymbidium_kanran_KJ597836

Cymbidium_kanran_KJ597837

Cymbidium_kanran_KJ597838

Cymbidium_kanran_KJ597839

Cymbidium_kanran_KT338677

Cymbidium_lancifolium_AF284724

Cymbidium_lancifolium_AF470509

Cymbidium_lancifolium_AF470520

Cymbidium_lancifolium_AJ300285

Cymbidium_lancifolium_FJ899751

Cymbidium_lancifolium_KFBG734

Cymbidium_lancifolium_KJ597851

Cymbidium_lancifolium_KJ597852

Cymbidium_lancifolium_KY966515

Cymbidium_lancifolium_PK12128

Cymbidium_lancifolium_SG1274

Cymbidium_sinense_AF284714

Cymbidium_sinense_AF284715

Cymbidium_sinense_AF470517

Cymbidium_sinense_AJ300272

Cymbidium_sinense_HQ263144

Cymbidium_sinense_KJ597847

Cymbidium_sinense_KJ597848

Cymbidium_sinense_KJ597849

Cymbidium_sinense_KJ597850

Cymbidium_sinense_SG1213

Cymbidium_sinense_SG1218

Cymbidium_sinense_SG1341

Cymbidium_sinense_SG1342

Dendrobium_aduncum_AB593484

Dendrobium_aduncum_GU339110

Dendrobium_aduncum_HM590372

Dendrobium_aduncum_HQ114249

Dendrobium_aduncum_HQ114250

Dendrobium_aduncum_JF713083

Dendrobium_aduncum_JN388580

Dendrobium_aduncum_KC346887

Dendrobium_aduncum_KC568295

Dendrobium_aduncum_KF143428

Dendrobium_aduncum_KFBG8766

Dendrobium_aduncum_KJ210409

Dendrobium_aduncum_KJ210410

Dendrobium_aduncum_KJ210411

Dendrobium_aduncum_KJ210412

Dendrobium_aduncum_KJ672619

Dendrobium_aduncum_KP159298

Dendrobium_aduncum_KR075042

Dendrobium_aduncum_KX600499

Dendrobium_anosmum_AB593499

Dendrobium_anosmum_AB972339

Dendrobium_anosmum_EU477499

Dendrobium_anosmum_JN388570

Dendrobium_anosmum_KJ672650

Dendrobium_anosmum_KJ944630

Dendrobium_anosmum_KP743542

Dendrobium_anosmum_KP743544

Dendrobium_anosmum_KY966516

Dendrobium_cf_mimicum_PK12237E

Dendrobium_cf_mimicum_PK12237J

Dendrobium_crumenatum_AB593537

Dendrobium_crumenatum_AB972336

Dendrobium_crumenatum_AF521608

Dendrobium_crumenatum_AY239963

Dendrobium_crumenatum_AY273708

Dendrobium_crumenatum_EU840700

Dendrobium_crumenatum_HM054625

Dendrobium_crumenatum_HM590370

Dendrobium_crumenatum_JF713095

Dendrobium_crumenatum_JF713096

Dendrobium_crumenatum_JN388587

Dendrobium_crumenatum_KC507780

Dendrobium_crumenatum_KJ672661

Dendrobium_linawianum_AB593599

Dendrobium_linawianum_AF401487

Dendrobium_linawianum_AF521613

Dendrobium_linawianum_EU003115

Dendrobium_linawianum_EU003117

Dendrobium_linawianum_HM590371

Dendrobium_linawianum_JN388573

Dendrobium_linawianum_KJ672629

Dendrobium_linawianum_KP159299

Dendrobium_linawianum_KT779776

Dendrobium_linawianum_KY966557

Dendrobium_linawianum_SG1347

Dendrobium_lindleyi_AB593600

Dendrobium_lindleyi_AB972341

Dendrobium_lindleyi_DQ058784

Dendrobium_lindleyi_GU339114

Dendrobium_lindleyi_HM054672

Dendrobium_lindleyi_HM054673

Dendrobium_lindleyi_JF713110

Dendrobium_lindleyi_JN388568

Dendrobium_lindleyi_KC568301

Dendrobium_lindleyi_KFBG203

Dendrobium_lindleyi_KJ210461

Dendrobium_lindleyi_KJ672682

Dendrobium_lindleyi_KX522640

Dendrobium_lindleyi_KY966558

Dendrobium_lindleyi_KY966559

Dendrobium_lindleyi_KY966560

Dendrobium_loddigesii_AB593604

Dendrobium_loddigesii_AB873183

Dendrobium_loddigesii_AF311778

Dendrobium_loddigesii_AY485703

Dendrobium_loddigesii_EU121418

Dendrobium_loddigesii_EU592016

Dendrobium_loddigesii_HM590374

Dendrobium_loddigesii_HQ114220

Dendrobium_loddigesii_JN388569

Dendrobium_loddigesii_KC205187

Dendrobium_loddigesii_KC346890

Dendrobium_loddigesii_KF143481

Dendrobium_loddigesii_KJ672683

Dendrobium_loddigesii_KP159301

Dendrobium_loddigesii_KP264997

Dendrobium_loddigesii_KT778746

Dendrobium_loddigesii_KY966562

Dendrobium_loddigesii_SG1255

Dendrobium_spatella_AB847671

Dendrobium_spatella_KF143512

Dendrobium_spatella_KF143513

Dendrobium_spatella_SG1357

Dendrobium_spatella_SG1358

Dendrolirium_lasiopetalum_KFBG701

Dendrolirium_lasiopetalum_PK12173

Dendrolirium_lasiopetalum_PK12174

Dendrolirium_lasiopetalum_SG1312

Didymoplexiella_siamensis_PK12024

Didymoplexiella_siamensis_SG1242

Didymoplexiella_siamensis_SG1243

Dienia_ophrydis_AY907111

Dienia_ophrydis_AY907114

Dienia_ophrydis_KFBG7819

Dienia_ophrydis_KJ459275

Dienia_ophrydis_MF287970

Dienia_ophrydis_SG1201

Dienia_ophrydis_SG1254

Dienia_ophrydis_SG1276

Diploprora_championii_KFBG628

Diploprora_championii_KJ733410

Diploprora_championii_KY966590

Diploprora_championii_KY966591

Diploprora_championii_PK12025

Diploprora_championii_PK12029

Diploprora_championii_PK12095

Diploprora_championii_SG1144

Diploprora_championii_SG1230

Disperis_neilgherrensis_KM583454

Epipogium_roseum_EU711232

Epipogium_roseum_SG1249

Epipogium_roseum_SG1250

Eria_scabrilinguis_KFBG916

Eria_scabrilinguis_KY239252

Eria_scabrilinguis_KY966596

Eria_scabrilinguis_PK12164

Eria_scabrilinguis_PK12165

Eria_scabrilinguis_SG1302

Erythrodes_blumei_JN166066

Erythrodes_blumei_KT343981

Erythrodes_blumei_KT343982

Erythrodes_blumei_PK12103

Erythrodes_blumei_PK12104

Eulophia_flava_JN114508

Eulophia_flava_JN114509

Eulophia_flava_SG1158

Eulophia_flava_SG1159

Eulophia_graminea_AF284727

Eulophia_graminea_FJ565666

Eulophia_graminea_KF318890

Eulophia_graminea_MH768268

Eulophia_graminea_MH768269

Eulophia_graminea_SG1270

Eulophia_graminea_SG1350

Eulophia_picta_AF284726

Eulophia_picta_JN114510

Eulophia_picta_JN114511

Eulophia_picta_JN114512

Eulophia_picta_JN114513

Eulophia_picta_PK12045

Eulophia_picta_PK12046

Eulophia_picta_PK12047

Eulophia_picta_PK12048

Eulophia_picta_PK12137

Eulophia_picta_PK12138

Eulophia_picta_PK12153

Eulophia_picta_SG1271

Eulophia_zollingeri_AB306313

Eulophia_zollingeri_SG1262

Eulophia_zollingeri_SG1263

Eulophia_zollingeri_SG1264

Eulophia_zollingeri_SG1265

Eulophia_zollingeri_SG1353

Gastrochilus_japonicus_AY228503

Gastrochilus_japonicus_KF545875

Gastrochilus_japonicus_KJ733418

Gastrochilus_japonicus_KY966598

Gastrochilus_japonicus_KY966599

Gastrochilus_kadooriei_KFBG3250

Gastrodia_peichatieniana_HK43268

Goodyera_foliosa_HM140995

Goodyera_foliosa_KT344001

Goodyera_foliosa_KT344002

Goodyera_foliosa_KT344003

Goodyera_foliosa_KT344004

Goodyera_foliosa_KT344005

Goodyera_foliosa_KT344006

Goodyera_foliosa_KT344007

Goodyera_foliosa_KT344008

Goodyera_foliosa_KT344009

Goodyera_foliosa_PK12067

Goodyera_foliosa_SG1309

Goodyera_foliosa_SG1315

Goodyera_foliosa_var_foliosa_KC205157

Goodyera_foliosa_var_laevis_HM140998

Goodyera_procera_HM151402

Goodyera_procera_HM222488

Goodyera_procera_JN114514

Goodyera_procera_JN114515

Goodyera_procera_JN114516

Goodyera_procera_JN114517

Goodyera_procera_JN114518

Goodyera_procera_KC237319

Goodyera_procera_KT344043

Goodyera_procera_KT344044

Goodyera_procera_KT344045

Goodyera_procera_KY966601

Goodyera_procera_SG1152

Goodyera_procera_SG1240

Goodyera_procera_SG1241

Goodyera_procera_SG1346

Goodyera_pusilla_KM593694

Goodyera_pusilla_KT344046

Goodyera_pusilla_KT344047

Goodyera_seikomontana_KT344068

Goodyera_seikomontana_KT344069

Goodyera_seikomontana_KY966602

Goodyera_seikoomontana_SG1252

Goodyera_seikoomontana_SG1253

Goodyera_seikoomontana_SG1354

Goodyera_viridiflora_JN166067

Goodyera_viridiflora_KC205154

Goodyera_viridiflora_KT344078

Goodyera_viridiflora_KT344079

Goodyera_viridiflora_KT344080

Goodyera_viridiflora_KT344081

Goodyera_viridiflora_KT344082

Goodyera_viridiflora_KT344083

Goodyera_viridiflora_KT344084

Goodyera_viridiflora_KT344085

Goodyera_viridiflora_PK12170

Goodyera_viridiflora_PK12172

Goodyera_viridiflora_SG1305

Goodyera_viridiflora_SG1306

Habenaria_ciliolaris_MF944286

Habenaria_ciliolaris_MF944287

Habenaria_dentata_KFBG1009

Habenaria_dentata_KFBG2126B

Habenaria_dentata_KJ460038

Habenaria_dentata_KY966605

Habenaria_dentata_PK12058

Habenaria_dentata_PK12059

Habenaria_dentata_SG1005

Habenaria_leptoloba_KY055535

Habenaria_leptoloba_PK12060

Habenaria_leptoloba_PK12061

Habenaria_leptoloba_PK12160

Habenaria_leptoloba_PK12161

Habenaria_leptoloba_PK12183

Habenaria_leptoloba_SG1304

Habenaria_leptoloba_SG1362

Habenaria_linguella_MF944303

Habenaria_linguella_PK12049

Habenaria_linguella_PK12134

Habenaria_linguella_PK12141

Habenaria_linguella_SG1195

Habenaria_linguella_SG1299

Habenaria_linguella_SG1300

Habenaria_reniformis_PK12142

Habenaria_reniformis_PK12143

Habenaria_reniformis_PK12144

Habenaria_reniformis_PK12145

Habenaria_reniformis_PK12146

Habenaria_reniformis_SG1296

Habenaria_reniformis_SG1297

Habenaria_rhodocheila_KJ460043

Habenaria_rhodocheila_KR350167

Habenaria_rhodocheila_KY966607

Habenaria_rhodocheila_MF944315

Habenaria_rhodocheila_PK12139

Habenaria_rhodocheila_SG1289

Habenaria_rhodocheila_SG1290

Habenaria_rhodocheila_SG1291

Habenaria_rodeiensis_HM777577

Hetaeria_youngsayei_KT344095

Hetaeria_youngsayei_KY966608

Hetaeria_youngsayei_SG1244

Hetaeria_youngsayei_SG1245

Hypoxis_rigidula_SG1207

Lecanorchis_nigricans_FJ425829

Lecanorchis_nigricans_SG1279

Lecanorchis_nigricans_SG1280

Liparis_bootanensis_KFBG238

Liparis_bootanensis_KJ459280

Liparis_bootanensis_KY966611

Liparis_bootanensis_PK12162

Liparis_bootanensis_SG1215

Liparis_bootanensis_SG1216

Liparis_bootanensis_SG1307

Liparis_bootanensis_SG1336

Liparis_ferruginea_SG1156

Liparis_ferruginea_SG1266

Liparis_ferruginea_SG1267

Liparis_gigantea_PK12116

Liparis_gigantea_PK12117

Liparis_gigantea_PK12118

Liparis_nervosa_AB289482

Liparis_nervosa_AY907092

Liparis_nervosa_JN114595

Liparis_nervosa_JN114596

Liparis_nervosa_JN114597

Liparis_nervosa_JN114598

Liparis_nervosa_JN114599

Liparis_nervosa_JN114600

Liparis_nervosa_JN114601

Liparis_nervosa_JN114602

Liparis_nervosa_JN114603

Liparis_nervosa_JN114604

Liparis_nervosa_KFBG330

Liparis_nervosa_KJ459294

Liparis_nervosa_KT338746

Liparis_nervosa_KT338747

Liparis_nervosa_SG1233

Liparis_nervosa_SG1234

Liparis_nervosa_SG1235

Liparis_odorata_KJ021033

Liparis_odorata_SG1256

Liparis_odorata_SG1257

Liparis_sootenzanensis_KJ021034

Liparis_sootenzanensis_SG1351

Liparis_sootenzanensis_SG1352

Liparis_stricklandiana_KF589873

Liparis_stricklandiana_KFBG124

Liparis_stricklandiana_KFBG818

Liparis_stricklandiana_KJ459298

Liparis_stricklandiana_KY966613

Liparis_stricklandiana_KY966614

Liparis_stricklandiana_PK12091

Liparis_stricklandiana_SG1332

Liparis_stricklandiana_SG1333

Liparis_stricklandiana_SG1337

Liparis_viridiflora_AY907107

Liparis_viridiflora_KJ459299

Liparis_viridiflora_KY966615

Liparis_viridiflora_KY966616

Liparis_viridiflora_PK12120

Liparis_viridiflora_SG1308

Liparis_viridiflora_SG1338

Ludisia_discolor_AJ539483

Ludisia_discolor_EF590781

Ludisia_discolor_EF590782

Ludisia_discolor_JN166073

Ludisia_discolor_KR815834

Ludisia_discolor_KT344102

Ludisia_discolor_KY966617

Ludisia_discolor_SG1236

Ludisia_discolor_SG1237

Ludisia_discolor_SG1348

Nephelaphyllum_tenuiflorum_KF560535

Nephelaphyllum_tenuiflorum_KM025159

Nephelaphyllum_tenuiflorum_KY966621

Nephelaphyllum_tenuiflorum_PK12121

Nephelaphyllum_tenuiflorum_PK12122

Nephelaphyllum_tenuiflorum_PK12123

Nephelaphyllum_tenuiflorum_SG1220

Nervilia_plicata_AF324179

Nervilia_plicata_JN114618

Nervilia_plicata_JN114619

Nervilia_plicata_JN114620

Nervilia_plicata_MG452049

Nervilia_plicata_SG1143

Nervilia_plicata_SG1277

Neuwiedia_zollingeri_var_singapureana_PK12124

Neuwiedia_zollingeri_var_singapureana_SG1268

Neuwiedia_zollingeri_var_singapureana_SG1269

Neuwiedia_zollingeri_var_singapureana_SG1340

Neuwiedia_zollingeri_var_singapureana_JF796932

Neuwiedia_zollingeri_var_singapureana_KY966622

Pachystoma_pubescens_PK12107

Pachystoma_pubescens_PK12108

Paphiopedilum_purpuratum_AJ564364

Paphiopedilum_purpuratum_EF156131

Paphiopedilum_purpuratum_FJ899756

Paphiopedilum_purpuratum_GU993850

Paphiopedilum_purpuratum_JX088564

Paphiopedilum_purpuratum_KX931030

Paphiopedilum_purpuratum_PK12075

Paphiopedilum_purpuratum_PK12082A

Paphiopedilum_purpuratum_PK12083C

Paphiopedilum_purpuratum_SG1149

Paphiopedilum_purpuratum_Z78440

Pecteilis_susannae_MF944351

Pecteilis_susannae_MF944352

Pecteilis_susannae_PK12051

Pecteilis_susannae_PK12136

Pecteilis_susannae_PK12154

Pecteilis_susannae_SG1292

Peristylus_calcaratus_PK12052

Peristylus_calcaratus_PK12053

Peristylus_calcaratus_SG1303

Peristylus_densus_SG1258

Peristylus_densus_SG1260

Peristylus_goodyeroides_MF944361

Peristylus_goodyeroides_MF944362

Peristylus_intrudens_PK12050

Peristylus_intrudens_PK12056

Peristylus_intrudens_SG1298

Peristylus_lacertifer_MF944365

Peristylus_lacertifer_MF944366

Peristylus_lacertifer_PK12149

Peristylus_lacertifer_PK12163

Peristylus_lacertifer_SG1006

Peristylus_tentaculatus_KJ460035

Persitylus_tentaculatus_PK12062

Persitylus_tentaculatus_PK12171

Persitylus_tentaculatus_SG1007

Phaius_tancarvilleae_AB222032

Phaius_tancarvilleae_AB239286

Phaius_tancarvilleae_AB239287

Phaius_tancarvilleae_AB239288

Phaius_tancarvilleae_AB239289

Phaius_tancarvilleae_JN114673

Phaius_tancarvilleae_KF560503

Phaius_tancarvilleae_KF560531

Phaius_tancarvilleae_KM025161

Phaius_tancarvilleae_KY966645

Phaius_tancarvilleae_MG869015

Phaius_tankervilleae_PK12084

Phaius_tankervilleae_PK12085

Phaius_tankervilleae_PK12099

Phaius_tankervilleae_PK12100

Phaius_wallichii_KF560532

Phaius_wallichii_KY966646

Platanthera_mandarinorum_JN696464

Platanthera_mandarinorum_subsp_mandarinorum_KT338772

Platanthera_minor_KJ460069

Platanthera_minor_KJ460079

Platanthera_minor_PK12030

Platanthera_minor_SG1154

Platanthera_minor_SG1223

Platanthera_minor_SG1224

Platanthera_minor_SG1238

Porpax_pusilla_KY239239

Porpax_pusilla_PK12071

Porpax_pusilla_PK12072

Porpax_pusilla_PK12125

Porpax_pusilla_SG1334

Renanthera_coccinea_KJ733441

Rhomboda_abbreviata_KT344110

Rhomboda_abbreviata_KY966662

Rhomboda_abbreviata_PK12166

Rhomboda_abbreviata_PK12175

Rhomboda_abbreviata_SG1203

Robiquetia_succisa_KJ733444

Robiquetia_succisa_KY966667

Robiquetia_succisa_PK12155

Robiquetia_succisa_PK12156

Robiquetia_succisa_PK12157

Robiquetia_succisa_SG1293

Robiquetia_succisa_SG1294

Robiquetia_succisa_SG1345

Spathoglottis_pubescens_KM025162

Spathoglottis_pubescens_KP751405

Spathoglottis_pubescens_KP751406

Spathoglottis_pubescens_MG869012

Spathoglottis_pubescens_PK12135

Spathoglottis_pubescens_PK12140

Spathoglottis_pubescens_SG1202

Spathoglottis_pubescens_SG1205

Spiranthes_hongkongensis_MF286484

Spiranthes_hongkongensis_MF286511

Spiranthes_hongkongensis_MH002629

Spiranthes_hongkongensis_MH002630

Spiranthes_hongkongensis_MH002631

Spiranthes_hongkongensis_MH002632

Spiranthes_hongkongensis_MH002633

Spiranthes_hongkongensis_MH002634

Spiranthes_hongkongensis_MH002635

Spiranthes_hongkongensis_MH002636

Spiranthes_hongkongensis_MH002637

Spiranthes_hongkongensis_MH002638

Spiranthes_hongkongensis_MH002639

Spiranthes_hongkongensis_MH002640

Spiranthes_hongkongensis_MH002641

Spiranthes_hongkongensis_MH002642

Spiranthes_hongkongensis_MH002643

Spiranthes_hongkongensis_MH002644

Spiranthes_hongkongensis_MH002645

Spiranthes_hongkongensis_MH002646

Spiranthes_hongkongensis_MH002647

Spiranthes_hongkongensis_MH002648

Spiranthes_hongkongensis_MH002649

Spiranthes_hongkongensis_MH002650

Spiranthes_hongkongensis_MH002651

Spiranthes_hongkongensis_MH002652

Spiranthes_hongkongensis_MH002653

Spiranthes_hongkongensis_MH002654

Spiranthes_hongkongensis_MH002655

Spiranthes_hongkongensis_MH002656

Spiranthes_hongkongensis_MH002657

Spiranthes_hongkongensis_MH002658

Spiranthes_hongkongensis_MH002659

Spiranthes_hongkongensis_MH002660

Spiranthes_hongkongensis_MH002661

Spiranthes_hongkongensis_MH002662

Spiranthes_hongkongensis_MH002663

Spiranthes_hongkongensis_MH002664

Spiranthes_hongkongensis_MH002665

Spiranthes_hongkongensis_MH002666

Spiranthes_hongkongensis_MH002667

Spiranthes_hongkongensis_MH002668

Spiranthes_hongkongensis_MH002669

Spiranthes_hongkongensis_MH002670

Spiranthes_hongkongensis_MH002671

Spiranthes_hongkongensis_MH002672

Spiranthes_hongkongensis_MH002673

Spiranthes_hongkongensis_MH002674

Spiranthes_hongkongensis_MH002675

Spiranthes_hongkongensis_MH002676

Spiranthes_hongkongensis_MH002677

Spiranthes_hongkongensis_MH002678

Spiranthes_hongkongensis_MH002679

Spiranthes_hongkongensis_MH002680

Spiranthes_hongkongensis_MH002681

Spiranthes_hongkongensis_MH002682

Spiranthes_hongkongensis_MH002683

Spiranthes_hongkongensis_MH002684

Spiranthes_hongkongensis_MH002685

Spiranthes_hongkongensis_MH002686

Spiranthes_hongkongensis_MH002687

Spiranthes_hongkongensis_MH002688

Spiranthes_hongkongensis_MH002689

Spiranthes_hongkongensis_MH002690

Spiranthes_hongkongensis_MH002691

Spiranthes_hongkongensis_MH002692

Spiranthes_hongkongensis_MH002693

Spiranthes_hongkongensis_MH002694

Spiranthes_hongkongensis_MH002695

Spiranthes_hongkongensis_MH002696

Spiranthes_hongkongensis_MH002697

Spiranthes_hongkongensis_MH002698

Spiranthes_hongkongensis_MH038786

Spiranthes_hongkongensis_MH038787

Spiranthes_hongkongensis_PK12028

Spiranthes_hongkongensis_PK12102

Spiranthes_hongkongensis_PK12179

Spiranthes_sinensis_HE575518

Spiranthes_sinensis_KM262399

Spiranthes_sinensis_KM262400

Spiranthes_sinensis_KT338780

Spiranthes_sinensis_KT338781

Spiranthes_sinensis_MF286485

Spiranthes_sinensis_MF286486

Spiranthes_sinensis_MF286487

Spiranthes_sinensis_MF286488

Spiranthes_sinensis_MF286489

Spiranthes_sinensis_MF286493

Spiranthes_sinensis_MF286494

Spiranthes_sinensis_MF286495

Spiranthes_sinensis_MF286496

Spiranthes_sinensis_MF286497

Spiranthes_sinensis_MF286498

Spiranthes_sinensis_MF286499

Spiranthes_sinensis_MF286504

Spiranthes_sinensis_MF286505

Spiranthes_sinensis_MF286506

Spiranthes_sinensis_MF286507

Spiranthes_sinensis_MF286509

Spiranthes_sinensis_MF286510

Spiranthes_sinensis_MH005035

Spiranthes_sinensis_MH005036

Spiranthes_sinensis_MH005037

Spiranthes_sinensis_MH005038

Spiranthes_sinensis_MH005039

Spiranthes_sinensis_MH005040

Spiranthes_sinensis_MH005041

Spiranthes_sinensis_MH005042

Spiranthes_sinensis_MH005043

Spiranthes_sinensis_MH005044

Spiranthes_sinensis_MH005045

Spiranthes_sinensis_MH005046

Spiranthes_sinensis_MH005047

Spiranthes_sinensis_MH005048

Spiranthes_sinensis_MH005049

Spiranthes_sinensis_MH005050

Spiranthes_sinensis_MH005051

Spiranthes_sinensis_MH005052

Spiranthes_sinensis_MH005053

Spiranthes_sinensis_MH005054

Spiranthes_sinensis_MH005055

Spiranthes_sinensis_MH005056

Spiranthes_sinensis_MH005057

Spiranthes_sinensis_MH005058

Spiranthes_sinensis_MH005059

Spiranthes_sinensis_MH038785

Spiranthes_sinensis_MH802049

Spiranthes_sinensis_MH802050

Spiranthes_sinensis_PK12106

Spiranthes_sinensis_PK12109

Spiranthes_sinensis_SG1153

Spiranthes_sinensis_SG1155

Tainia_cordifolia_KF560538

Tainia_cordifolia_KM025163

Tainia_cordifolia_KM025164

Tainia_dunnii_KF560536

Tainia_dunnii_KM025165

Tainia_dunnii_SG1273

Thelasis_pygmaea_KY239231

Thrixspermum_centipeda_AB217591

Thrixspermum_centipeda_KFBG3066D

Thrixspermum_centipeda_KJ733456

Thrixspermum_centipeda_KX679332

Thrixspermum_centipeda_KY966674

Thrixspermum_centipeda_KY966675

Thrixspermum_centipeda_PK12129

Thrixspermum_centipeda_PK12130

Thrixspermum_centipeda_PK12131

Thrixspermum_centipeda_PK12132

Thrixspermum_centipeda_PK12133

Tropidia_curculigoides_SG1281

Tropidia_curculigoides_SG1282

Tropidia_curculigoides_SG1283

Tropidia_curculigoides_SG1284

Tropidia_nipponica_PK12181

Tropidia_nipponica_SG1355

Tropidia_nipponica_SG1356

Tropidia_nipponica_var_hachijoensis_MH596706

Vanilla_shenzhenica_JF796930

Vanilla_shenzhenica_KFBG290

Vrydagzynea_nuda_SG1222

Vrydagzynea_nuda_SG1246A

Vrydagzynea_nuda_SG1246B

Vrydagzynea_nuda_SG1247

Zeuxine_boninensis_d16

Zeuxine_boninensis_Z3

Zeuxine_gracilis_JN166075

Zeuxine_gracilis_KC191732

Zeuxine_gracilis_PK12057

Zeuxine_gracilis_PK12066

Zeuxine_gracilis_SG1204

Zeuxine_strateumatica_KT344117

Zeuxine_strateumatica_KY966688

Zeuxine_strateumatica_SG1211

Zeuxine_strateumatica_SG1212

;

end;

begin characters;

dimensions nchar=183;

format datatype=dna missing=? gap=-;

matrix

Acampe_praemorsa_var_longepedunculata_AB217526 ACGAC-TCTCGACAATGGATATCTC-GGCTCTCGCATCGATGAAGAGCGCAGCGAAATGCGATACGTGGTGC-GAATTGCAGAATCCCGCGAACC-ATCGAGTCTTTGAACGCAAGTTGCGCCCGAGGCCAAT-CGGTCGAGGGCACGTCCGCCTG---GGCGTCAA-GCGTCGCGCCGCTCC

Acampe_praemorsa_var_longepedunculata_KJ733385 ACGAC-TCTCGACAATGGATATCTC-GGCTCTCGCATCGATGAAGAGCGCAGCGAAATGCGATACGTGGTGC-GAATTGCAGAATCCCGCGAACC-ATCGAGTCTTTGAACGCAAGTTGCGCCCGAGGCCAAT-CGGTCGAGGGCACGTCCGCCTG---GGCGTCAA-GCGTTGCGCCGCTCC

Acampe_praemorsa_var_longepedunculata_KY966408 ACGAC-TCTCGACAATGGATATCTC-GGCTCTCGCATCGATGAAGAGCGCAGCGAAATGCGATACGTGGTGC-GAATTGCAGAATCCCGCGAACC-ATCGAGTCTTTGAACGCAAGTTGCGCCCGAGGCCAAT-CGGTCGAGGGCACGTCCGCCTG---GGCGTCAA-GCGTTGCGCCGCTCC

Acampe_praemorsa_var_longepedunculata_PK12086 ACGAC-TCTCGACAATGGATATCTC-GGCTCTCGCATCGATGAAGAGCGCAGCGAAATGCGATACGTGGTGC-GAATTGCAGAATCCCGCGAACC-ATCGAGTCTTTGAACGCAAGTTGCGCCCGAGGCCAAT-CGGTCGAGGGCACGTCCGCCTG---GGCGTCAA-GCGTTGCGCCGCTCC

Acampe_praemorsa_var_longepedunculata_PK12159 ACGAC-TCTCGACAATGGATATCTC-GGCTCTCGCATCGATGAAGAGCGCAGCGAAATGCGATACGTGGTGC-GAATTGCAGAATCCCGCGAACC-ATCGAGTCTTTGAACGCAAGTTGCGCCCGAGGCCAAT-CGGTCGAGGGCACGTCCGCCTG---GGCGTCAA-GCGTTGCGCCGCTCC

Acampe_praemorsa_var_longepedunculata_SG1199 ACGAC-TCTCGACAATGGATATCTC-GGCTCTCGCATCGATGAAGAGCGCAGCGAAATGCGATACGTGGTGC-GAATTGCAGAATCCCGCGAACC-ATCGAGTCTTTGAACGCAAGTTGCGCCCGAGGCCAAT-CGGTCGAGGGCACGTCCGCCTG---GGCGTCAA-GCGTTGCGCCGCTCC

Acanthophippium_gougahense_KFBG3161 ATGAC-TCTCGGCAATGGATATCTC-GGCTCTCGCATCGATGAAGAGCGCAGCGAAATGCGATACGTGGTGC-GAATTGCAGAATCCCGCGAACC-ATCGAGTCTTTGAACGCAAGTTGCGCCTGAGGCCATC-CGGCCAAGGGCACGTCTGCCTG---GGCGTCAA-GCGTTGCGTCGCTTC

Acanthophippium_gougahensis_KY966409 ATGAC-TCTCGGCAATGGATATCTC-GGCTCTCGCATCGATGAAGAGCGCAGCGAAATGCGATACGTGGTGC-GAATTGCAGAATCCCGCGAACC-ATCGAGTCTTTGAACGCAAGTTGCGCCTGAGGCCATC-CGGCCAAGGGCACGTCTGCCTG---GGCGTCAA-GCGTTGCGTCGCTTC

Ania_hongkongensis_KF560537 ACGAC-TCTCGGCAATGGATATCTC-GGCTCTCGCATCGATGAAGAGCGCAGCGAAATGCGATACGTGGTGC-GAATTGCAGAATCCCGCGAACC-ATCGAGTCTTTGAACGCAAGTTGCGCCCGAGGCCAAC-CGGCCAAGGGCACGTCTGCCTG---GGCGTCAA-GCGTTGCGTCGCTCC

Ania_hongkongensis_KFBG4612 ACGAC-TCTCGGCAATGGATATCTC-GGCTCTCGCATCGATGAAGAGCGCAGCGAAATGCGATACGTGGTGC-GAATTGCAGAATCCCGCGAACC-ATCGAGTCTTTGAACGCAAGTTGCGCCCGAGGCCAAC-CGGCCAAGGGCACGTCTGCCTG---GGCGTCAA-GCGTTGCGTCGCTCC

Ania_hongkongensis_KFBG4613 ACGAC-TCTCGGCAATGGATATCTC-GGCTCTCGCATCGATGAAGAGCGCAGCGAAATGCGATACGTGGTGC-GAATTGCAGAATCCCGCGAACC-ATCGAGTCTTTGAACGCAAGTTGCGCCCGAGGCCAAC-CGGCCAAGGGCACGTCTGCCTG---GGCGTCAA-GCGTTGCGTCGCTCC

Ania_hongkongensis_KM025146 ACGAC-TCTCGGCAATGGATATCTC-GGCTCTCGCATCGATGAAGAGCGCAGCGAAATGCGATACGTGGTGC-GAATTGCAGAATCCCGCGAACC-ATCGAGTCTTTGAACGCAAGTTGCGCCCGAGGCCAAC-CGGCCAAGGGCACGTCTGCCTG---GGCGTCAA-GCGTTGCGTCGCTCC

Ania_hongkongensis_KM025147 ACGAC-TCTCGGCAATGGATATCTC-GGCTCTCGCATCGATGAAGAGCGCAGCGAAATGCGATACGTGGTGC-GAATTGCAGAATCCCGCGAACC-ATCGAGTCTTTGAACGCAAGTTGCGCCCGAGGCCAAC-CGGCCAAGGGCACGTCTGCCTG---GGCGTCAA-GCGTTGCGTCGCTCC

Ania_hongkongensis_KM025148 ACGAC-TCTCGGCAATGGATATCTC-GGCTCTCGCATCGATGAAGAGCGCAGCGAAATGCGATACGTGGTGC-GAATTGCAGAATCCCGCGAACC-ATCGAGTCTTTGAACGCAAGTTGCGCCCGAGACCAAC-CGGCCAAGGGCACGTCTGCCTG---GGCGTCGA-GCGTTGCGTCGCTCC

Ania_hongkongensis_KY966415 ACGAC-TCTCGGCAATGGATATCTC-GGCTCTCGCATCGATGAAGAGCGCAGCGAAATGCGATACGTGGTGC-GAATTGCAGAATCCCGCGAACC-ATCGAGTCTTTGAACGCAAGTTGCGCCCGAGGCCAAC-CGGCCAAGGGCACGTCTGCCTG---GGCGTCAA-GCGTTGCGTCGCTCC

Ania_hongkongensis_PK12027 ACGAC-TCTCGGCAATGGATATCTC-GGCTCTCGCATCGATGAAGAGCGCAGCGAAATGCGATACGTGGTGC-GAATTGCAGAATCCCGCGAACC-ATCGAGTCTTTGAACGCAAGTTGCGCCCGAGGCCAAC-CGGCCAAGGGCACGTCTGCCTG---GGCGTCAA-GCGTTGCGTCGCTCC

Ania_hongkongensis_SG1231 ACGAC-TCTCGGCAATGGATATCTC-GGCTCTCGCATCGATGAAGAGCGCAGCGAAATGCGATACGTGGTGC-GAATTGCAGAATCCCGCGAACC-ATCGAGTCTTTGAACGCAAGTTGCGCCCGAGGCCAAC-CGGCCAAGGGCACGTCTGCCTG---GGCGTCAA-GCGTTGCGTCGCTCC

Ania_hongkongensis_SG1343 ACGAC-TCTCGGCAATGGATATCTC-GGCTCTCGCATCGATGAAGAGCGCAGCGAAATGCGATACGTGGTGC-GAATTGCAGAATCCCGCGAACC-ATCGAGTCTTTGAACGCAAGTTGCGCCCGAGGCCAAC-CGGCCAAGGGCACGTCTGCCTG---GGCGTCAA-GCGTTGCGTCGCTCC

Ania_ruybarrettoi_KFBG43 ACGAC-TCTCGGCAATGGATATCTC-GGCTCTCGCATCGATGAAGAGCGCAGCGAAATGCGATACGTGGTGC-GAATTGCAGAATCCCGCGAACC-ATCGAGTCTTTGAACGCAAGTTGCGCCCGAGGCCAAC-CGGCCAAGGGCACGTCTGCCTG---GGCGTCAA-GCGTTGCGTCGCTCC

Ania_ruybarrettoi_KM025152 ACGAC-TCTCGGCAATGGATATCTC-GGCTCTCGCATCGATGAAGAGCGCAGCGAAATGCGATACGTGGTGC-GAATTGCAGAATCCCGCGAACC-ATCGAGTCTTTGAACGCAAGTTGCGCCCGAGGCCAAC-CGGCCAAGGGCACGTCTGCCTG---GGCGTCAA-GCGTTGCGTCGCTCC

Ania_ruybarrettoi_KY966416 ACGAC-TCTCGGCAATGGATATCTC-GGCTCTCGCATCGATGAAGAGCGCAGCGAAATGCGATACGTGGTGC-GAATTGCAGAATCCCGCGAACC-ATCGAGTCTTTGAACGCAAGTTGCGCCCGAGGCCAAC-CGGCCAAGGGCACGTCTGCCTG---GGCGTCAA-GCGTTGCGTCGCTCC

Ania_ruybarrettoi_SG1395 ACGAC-TCTCGGCAATGGATATCTC-GGCTCTCGCATCGATGAAGAGCGCAGCGAAATGCGATACGTGGTGC-GAATTGCAGAATCCCGCGAACC-ATCGAGTCTTTGAACGCAAGTTGCGCCCGAGGCCAAC-CGGCCAAGGGCACGTCTGCCTG---GGCGTCAA-GCGTTGCGTCGCTCC

Anoectochilus_formosanus_AY052780 ATGAC-TCTCGGCAATGGATATCTT-GGCTCTTGCATCGATGAAGAGCGCAGCGAAATGCGATACGTGGTGT-GAATTGCAGAATCCCGTGAACC-ATCAAATCTTTGAACGCAAGTTGCGCCTGAGGCCAAT-TGGCTAAGGGCACGTCCGCCTG---GGCGTCAA-GCATTACATCGCTTC

Anoectochilus_formosanus_GQ328777 ATGAC-TCTCGGCAATGGATATCTT-GGCTCTTGCATCGATGAAGAGCGCAGCGAAATGCGATACGTGGTGT-GAATTGCAGAATCCCGTGAACC-ATCAAATCTTTGAACGCAAGTTGCGCCTGAGGCCAAT-TGGCTAAGGGCACGTCCGCCTG---GGCGTCAA-GCATTACATCGCTTC

Anoectochilus_formosanus_GQ396668 ATGAC-TCTCGGCAATGGATATCTT-GGCTCTTGCATCGATGAAGAGCGCAGCGAAATGCGATACGTGGTGT-GAATTGCAGAATCCCGTGAACC-ATCAAATCTTTGAACGCAAGTTGCGCCTGAGGCCAAT-TGGCTAAGGGCACGTCCGCCTG---GGCGTCAA-GCATTACATCGCTTC

Anoectochilus_formosanus_KR815833 ATGAC-TCTCGGCAATGGATATCTT-GGCTCTTGCATCGATGAAGAGCGCAGCGAAATGCGATACGTGGTGT-GAATTGCAGAATCCCGTGAACC-ATCAAATCTTTGAACGCAAGTTGCGCCTGAGGCCAAT-TGGCTAAGGGCACGTCCGCCTG---GGCGTCAA-GCATTACATCGCTTC

Anoectochilus_formosanus_KR815839 ATGAC-TCTCGGCAATGGATATCTT-GGCTCTTGCATCGATGAAGAGCGCAGCGAAATGCGATACGTGGTGT-GAATTGCAGAATCCCGTGAACC-ATCAAATCTTTGAACGCAAGTTGCGCCTGAGGCCAAT-TGGCTAAGGGCACGTCCGCCTG---GGCGTCAA-GCATTACATCGCTTC

Anoectochilus_formosanus_PK12215 ATGAC-TCTCGGCAATGGATATCTT-GGCTCTTGCATCGATGAAGAGCGCAGCGAAATGCGATACGTGGTGT-GAATTGCAGAATCCCGTGAACC-ATCAAATCTTTGAACGCAAGTTGCGCCTGAGGCCAAT-TGGCTAAGGGCACGTCCGCCTG---GGCGTCAA-GCATTACATCGCTTC

Anoectochilus_roxburghii_EU817408 ATGAC-TCTCGGCAATGGATATCTT-GGCTCTTGCATCGATGAAGAGCGCAGCGAAATGCGATACGTGGTGT-GAATTGCAGAATCCCGTGAACC-ATCAAATCTTTGAACGCAAGTTGCGCCTGAGGCCAAT-TGGCTAAGGGCACGTCCGCCTG---GGCGTCAA-GCATTACATCGCTTC

Anoectochilus_roxburghii_GQ328774 ATGAC-TCTCGGCAATGGATATCTT-GGCTCTTGCATCGATGAAGAGCGCAGCGAAATGCGATACGTGGTGT-GAATTGCAGAATCCCGTGAACC-ATCAAATCTTTGAACGCAAGTTGCGCCTGAGGCCAAT-TGGCTAAGGGCACGTCCGCCTG---GGCGTCAA-GCATTACATCGCTTC

Anoectochilus_roxburghii_KF425501 ATGAC-TCTCGGCAATGGATATCTT-GGCTCTTGCATCGATGAAGAGCGCAGCGAAATGCGATACGTGGTGT-GAATTGCAGAATCCCGTGAACC-ATCAAATCTTTGAACGCAAGTTGCGCCTGAGGCCAAT-TGGCTAAGGGCACGTCCGCCTG---GGCGTCAA-GCATTACATCGCTTC

Anoectochilus_roxburghii_KF425502 ATGAC-TCTCGGCAATGGATATCTT-GGCTCTTGCATCGATGAAGAGCGCAGCGAAATGCGATACGTGGTGT-GAATTGCAGAATCCCGTGAACC-ATCAAATCTTTGAACGCAAGTTGCGCCTGAGGCCAAT-TGGCTAAGGGCACGTCCGCCTG---GGCGTCAA-GCATTACATCGCTTC

Anoectochilus_roxburghii_KF425503 ATGAC-TCTCGGCAATGGATATCTT-GGCTCTTGCATCGATGAAGAGCGCAGCGAAATGCGATACGTGGTGT-GAATTGCAGAATCCCGTGAACC-ATCAAATCTTTGAACGCAAGTTGCGCCTGAGGCCAAT-TGGCTAAGGGCACGTCCGCCTG---GGCGTCAA-GCATTACATCGCTTC

Anoectochilus_roxburghii_KR815828 ATGAC-TCTCGGCAATGGATATCTT-GGCTCTTGCATCGATGAAGAGCGCAGCGAAATGCGATACGTGGTGT-GAATTGCAGAATCCCGTGAACC-ATCAAATCTTTGAACGCAAGTTGCGCCTGAGGCCAAT-TGGCTAAGGGCACGTCCGCCTG---GGCGTCAA-GCATTACATCGCTTC

Anoectochilus_roxburghii_KR815829 ATGAC-TCTCGGCAATGGATATCTT-GGCTCTTGCATCGATGAAGAGCGCAGCGAAATGCGATACGTGGTGT-GAATTGCAGAATCCCGTGAACC-ATCAAATCTTTGAACGCAAGTTGCGCCTGAGGCCAAT-TGGCTAAGGGCACGTCCGCCTG---GGCGTCAA-GCATTACATCGCTTC

Anoectochilus_roxburghii_KR815830 ATGAC-TCTCGGCAATGGATATCTT-GGCTCTTGCATCGATGAAGAGCGCAGCGAAATGCGATACGTGGTGT-GAATTGCAGAATCCCGTGAACC-ATCAAATCTTTGAACGCAAGTTGCGCCTGAGGCCAAT-TGGCTAAGGGCACGTCCGCCTG---GGCGTCAA-GCATTACATCGCTTC

Anoectochilus_roxburghii_KR815831 ATGAC-TCTCGGCAATGGATATCTT-GGCTCTTGCATCGATGAAGAGCGCAGCGAAATGCGATACGTGGTGT-GAATTGCAGAATCCCGTGAACC-ATCAAATCTTTGAACGCAAGTTGCGCCTGAGGCCAAT-TGGCTAAGGGCACGTCCGCCTG---GGCGTCAA-GCATTACATCGCTTC

Anoectochilus_roxburghii_KR815832 ATGAC-TCTCGGCAATGGATATCTT-GGCTCTTGCATCGATGAAGAGCGCAGCGAAATGCGATACGTGGTGT-GAATTGCAGAATCCCGTGAACC-ATCAAATCTTTGAACGCAAGTTGCGCCTGAGGCCAAT-TGGCTAAGGGCACGTCCGCCTG---GGCGTCAA-GCATTACATCGCTTC

Anoectochilus_roxburghii_KR815836 ATGAC-TCTCGGCAATGGATATCTT-GGCTCTTGCATCGATGAAGAGCGCAGCGAAATGCGATACGTGGTGT-GAATTGCAGAATCCCGTGAACC-ATCAAATCTTTGAACGCAAGTTGCGCCTGAGGCCAAT-TGGCTAAGGGCACGTCCGCCTG---GGCGTCAA-GCATTACATCGCTTC

Anoectochilus_roxburghii_KR815837 ATGAC-TCTCGGCAATGGATATCTT-GGCTCTTGCATCGATGAAGAGCGCAGCGAAATGCGATACGTGGTGT-GAATTGCAGAATCCCGTGAACC-ATCAAATCTTTGAACGCAAGTTGCGCCTGAGGCCAAT-TGGCTAAGGGCACGTCCGCCTG---GGCGTCAA-GCATTACATCGCTTC

Anoectochilus_roxburghii_KR815838 ATGAC-TCTCGGCAATGGATATCTT-GGCTCTTGCATCGATGAAGAGCGCAGCGAAATGCGATACGTGGTGT-GAATTGCAGAATCCCGTGAACC-ATCAAATCTTTGAACGCAAGTTGCGCCTGAGGCCAAT-TGGCTAAGGGCACGTCCGCCTG---GGCGTCAA-GCATTACATCGCTTC

Anoectochilus_roxburghii_KY966417 ATGAC-TCTCGGCAATGGATATCTT-GGCTCTTGCATCGATGAAGAGCGCAGCGAAATGCGATACGTGGTGT-GAATTGCAGAATCCCGTGAACC-ATCAAATCTTTGAACGCAAGTTGCGCCTGAGGCCAAT-TGGCTAAGGGCACGTCCGCCTG---GGCGTCAA-GCATTACATCGCTTC

Anoectochilus_roxburghii_PK12043 ATGAC-TCTCGGCAATGGATATCTT-GGCTCTTGCATCGATGAAGAGCGCAGCGAAATGCGATACGTGGTGT-GAATTGCAGAATCCCGTGAACC-ATCAAATCTTTGAACGCAAGTTGCGCCTGAGGCCAAT-TGGCTAAGGGCACGTCCGCCTG---GGCGTCAA-GCATTACATCGCTTC

Anoectochilus_roxburghii_PK12044 ATGAC-TCTCGGCAATGGATATCTT-GGCTCTTGCATCGATGAAGAGCGCAGCGAAATGCGATACGTGGTGT-GAATTGCAGAATCCCGTGAACC-ATCAAATCTTTGAACGCAAGTTGCGCCTGAGGCCAAT-TGGCTAAGGGCACGTCCGCCTG---GGCGTCAA-GCATTACATCGCTTC

Anoectochilus_roxburghii_PK12068 ATGAC-TCTCGGCAATGGATATCTT-GGCTCTTGCATCGATGAAGAGCGCAGCGAAATGCGATACGTGGTGT-GAATTGCAGAATCCCGTGAACC-ATCAAATCTTTGAACGCAAGTTGCGCCTGAGGCCAAT-TGGCTAAGGGCACGTCCGCCTG---GGCGTCAA-GCATTACATCGCTTC

Anoectochilus_roxburghii_PK12069 ATGAC-TCTCGGCAATGGATATCTT-GGCTCTTGCATCGATGAAGAGCGCAGCGAAATGCGATACGTGGTGT-GAATTGCAGAATCCCGTGAACC-ATCAAATCTTTGAACGCAAGTTGCGCCTGAGGCCAAT-TGGCTAAGGGCACGTCCGCCTG---GGCGTCAA-GCATTACATCGCTTC

Anoectochilus_roxburghii_SG1219 ATGAC-TCTCGGCAATGGATATCTT-GGCTCTTGCATCGATGAAGAGCGCAGCGAAATGCGATACGTGGTGT-GAATTGCAGAATCCCGTGAACC-ATCAAATCTTTGAACGCAAGTTGCGCCTGAGGCCAAT-TGGCTAAGGGCACGTCCGCCTG---GGCGTCAA-GCATTACATCGCTTC

Aphyllorchis_montana_FJ454867 ATGAC-TCTCGGCAACGGATATCTC-GGCTCTCGCATCGATGAAGAGCGCAGCGAAATGCGATACGTGGTGC-GAATTGCAGAATCCCGTGAACC-ATCAAGTCTTTGAACGCAAGTTGCGCCCGAGGCCAAT-CGGCCAAGGGCACGTCTGCCTG---GGCGTCAA-GCGTTGCGTCGCTTC

Aphyllorchis_montana_PK12147 ATGAC-TCTCGGCAACGGATATCTC-GGCTCTCGCATCGATGAAGAGCGCAGCGAAATGCGATACGTGGTGC-GAATTGCAGAATCCCGTGAACC-ATCAAGTCTTTGAACGCAAGTTGCGCCCGAGGCCAAT-CGGCCAAGGGCACGTCTGCCTG---GGCGTCAA-GCGTTGCGTCGCTTC

Aphyllorchis_montana_PK12148 ATGAC-TCTCGGCAACGGATATCTC-GGCTCTCGCATCGATGAAGAGCGCAGCGAAATGCGATACGTGGTGC-GAATTGCAGAATCCCGTGAACC-ATCAAGTCTTTGAACGCAAGTTGCGCCCGAGGCCAAT-CGGCCAAGGGCACGTCTGCCTG---GGCGTCAA-GCGTTGCGTCGCTTC

Aphyllorchis_montana_SG1010 ATGAC-TCTCGGCAACGGATATCTC-GGCTCTCGCATCGATGAAGAGCGCAGCGAAATGCGATACGTGGTGC-GAATTGCAGAATCCCGTGAACC-ATCAAGTCTTTGAACGCAAGTTGCGCCCGAGGCCAAT-CGGCCAAGGGCACGTCTGCCTG---GGCGTCAA-GCGTTGCGTCGCTTC

Apostasia_nipponica_PK12273 AAGAC-TCTCGGCAACGGATATCTC-GGCTCTCGCATCGATGAAGAACGCAGCGAACCGCGATACGTGGTGT-GAATTGCAGAATCCCGCGAATC-ATCGAGTCTTTGAACGCAAGTTGCGCCCGAGGCCAAG-TGGACGAGGGCACGCCTGCCTG---GTCGTCAG-GCGCTGCGTCGCTCC

Apostasia_nipponica_PK12274 AAGAC-TCTCGGCAACGGATATCTC-GGCTCTCGCATCGATGAAGAACGCAGCGAACCGCGATACGTGGTGT-GAATTGCAGAATCCCGCGAATC-ATCGAGTCTTTGAACGCAAGTTGCGCCCGAGGCCAAG-TGGCCGAGGGCACGCCTGCCTG---GTCGTCAG-GCGCCGCGTCGCTCC

Appendicula_cornuta_AF521073 ATGAC-TCTCGGCAATGGATATCTC-GGCTCTCGCATCGATGAAGAGCGCAGCGAAATGCGATACGTGGTGT-GAATTGCAGAATCCCGCGAACC-ATCGAGTCTTTGAACGCAAGTTGCGCCCGAGGCCAAC-CGGCTGAGGGCACGTCTGCCTG---GGCGTCAA-ACGTTTCGTCGCTTC

Appendicula_cornuta_KY239261 ATGAC-TCTCGGCAATGGATATCTC-GGCTCTCGCATCGATGAAGAGCGCAGCGAAATGCGATACGTGGTGT-GAATTGCAGAATCCCGCGAACC-ATCGAGTCTTTGAACGCAAGTTGCGCCCGAGGCCAAC-CGGCTGAGGGCACGTCTGCCTG---GGCGTCAA-ACGTTTCGTCGCTTC

Appendicula_cornuta_KY966418 ACGAC-TCTCGGCAATGGATATCTC-GGCTCTCGCATCGATGAAGAGCGCAGCGAAATGCGATACGTGGTGT-GAATTGCAGAATCCCGCGAACC-ATCGAGTCTTTGAACGCAAGTTGCGCCCGAGGCCAAC-CGGCTGAGGGCACGTCCGCCTG---GGCGTCAA-GCATTGCGTCGCTCC

Appendicula_cornuta_KY966419 ATGAC-TCTCGGCAATGGATATCTC-GGCTCTCGCATCGATGAAGAGCGCAGCGAAATGCGATACGTGGTGT-GAATTGCAGAATCCCGCGAACC-ATCGAGTCTTTGAACGCAAGTTGCGCCCGAGGCCAAC-CGGCTGAGGGCACGTCTGCCTG---GGCGTCAA-ACGTTTCGTCGCTTC

Appendicula_cornuta_PK12064 ATGAC-TCTCGGCAATGGATATCTC-GGCTCTCGCATCGATGAAGAGCGCAGCGAAATGCGATACGTGGTGT-GAATTGCAGAATCCCGCGAACC-ATCGAGTCTTTGAACGCAAGTTGCGCCCGAGGCCAAC-CGGCTGAGGGCACGTCTGCCTG---GGCGTCAA-ACGTTTCGTCGCTTC

Appendicula_cornuta_PK12065 ATGAC-TCTCGGCAATGGATATCTC-GGCTCTCGCATCGATGAAGAGCGCAGCGAAATGCGATACGTGGTGT-GAATTGCAGAATCCCGCGAACC-ATCGAGTCTTTGAACGCAAGTTGCGCCCGAGGCCAAC-CGGCTGAGGGCACGTCTGCCTG---GGCGTCAA-ACGTTTCGTCGCTTC

Arundina_graminifolia_AF273335 ACGAC-TCTCGGCAATGGATATCTC-GGCTCTCGCATCGATGAAGAGCGCAGCGAAATGCGATACGTGGTGC-GAATTGCAGAATCCCGCGAACC-ATCGAGTCTTTGAACGCAAGTTGCGCCTGAGGCCAAC-CGGCCGAGGGCACGTCTGCCTG---GGCGTCAG-GCGTTACGTCGCTCC

Arundina_graminifolia_AF302727 ACGAC-TCTCGGCAATGGATATCTC-GGCTCTCGCATCGATGAAGAGCGCAGCGAAATGCGATACGTGGTGC-GAATTGCAGAATCCCGCGAACC-ATCGAGTCTTTGAACGCAAGTTGCGCCTGAGGCCAAC-CGGCCGAGGGCACGTCTGCCTG---GGCGTCAG-GCGTTACGTCGCTCC

Arundina_graminifolia_AF461461 ACGAC-TCTCGGCAATGGATATCTC-GGCTCTCGCATCGATGAAGAGCGCAGCGAAATGCGATACGTGGTGC-GAATTGCAGAATCCCGCGAACC-ATCGAGTCTTTGAACGCAAGTTGCGCCTGAGGCCAAC-CGGCCGAGGGCACGTCTGCCTG---GGCGTCAG-GCGTTACGTCGCTCC

Arundina_graminifolia_JN114437 ACGAC-TCTCGGCAATGGATATCTC-GGCTCTCGCATCGATGAAGAGCGCAGCGAAATGCGATACGTGGTGC-GAATTGCAGAATCCCGCGAACC-ATCGAGTCTTTGAACGCAAGTTGCGCCTGAGGCCAAC-CGGCCGAGGGCACGTCTGCCTG---GGCGTCAG-GCGTTACGTCGCTCC

Arundina_graminifolia_JN114438 ACGAC-TCTCGGCAATGGATATCTC-GGCTCTCGCATCGATGAAGAGCGCAGCGAAATGCGATACGTGGTGC-GAATTGCAGAATCCCGCGAACC-ATCGAGTCTTTGAACGCAAGTTGCGCCTGAGGCCAAC-CGGCCGAGGGCACGTCTGCCTG---GGCGTCAG-GCGTTACGTCGCTCC

Arundina_graminifolia_JN114439 ACGAC-TCTCGGCAATGGATATCTC-GGCTCTCGCATCGATGAAGAGCGCAGCGAAATGCGATACGTGGTGC-GAATTGCAGAATCCCGCGAACC-ATCGAGTCTTTGAACGCAAGTTGCGCCTGAGGCCAAC-CGGCCGAGGGCACGTCTGCCTG---GGCGTCAG-GCGTTACGTCGCTCC

Arundina_graminifolia_JN114440 ACGAC-TCTCGGCAATGGATATCTC-GGCTCTCGCATCGATGAAGAGCGCAGCGAAATGCGATACGTGGTGC-GAATTGCAGAATCCCGCGAACC-ATCGAGTCTTTGAACGCAAGTTGCGCCTGAGGCCAAC-CGGCCGAGGGCACGTCTGCCTG---GGCGTCAG-GCGTTACGTCGCTCC

Arundina_graminifolia_KY966420 ACGAC-TCTCGGCAATGGATATCTC-GGCTCTCGCATCGATGAAGAGCGCAGCGAAATGCGATACGTGGTGC-GAATTGCAGAATCCCGCGAACC-ATCGAGTCTTTGAACGCAAGTTGCGCCTGAGGCCAAC-CGGCCGAGGGCACGTCTGCCTG---GGCGTCAG-GCGTTACGTCGCTCC

Arundina_graminifolia_SG1008 ACGAC-TCTCGGCAATGGATATCTC-GGCTCTCGCATCGATGAAGAGCGCAGCGAAATGCGATACGTGGTGC-GAATTGCAGAATCCCGCGAACC-ATCGAGTCTTTGAACGCAAGTTGCGCCTGAGGCCAAC-CGGCCGAGGGCACGTCTGCCTG---GGCGTCAG-GCGTTACGTCGCTCC

Arundina_graminifolia_SG1206 ACGAC-TCTCGGCAATGGATATCTC-GGCTCTCGCATCGATGAAGAGCGCAGCGAAATGCGATACGTGGTGC-GAATTGCAGAATCCCGCGAACC-ATCGAGTCTTTGAACGCAAGTTGCGCCTGAGGCCAAC-CGGCCGAGGGCACGTCTGCCTG---GGCGTCAG-GCGTTACGTCGCTCC

Arundina_graminifolia_SG1295 ACGAC-TCTCGGCAATGGATATCTC-GGCTCTCGCATCGATGAAGAGCGCAGCGAAATGCGATACGTGGTGC-GAATTGCAGAATCCCGCGAACC-ATCGAGTCTTTGAACGCAAGTTGCGCCTGAGGCCAAC-CGGCCGAGGGCACGTCTGCCTG---GGCGTCAG-GCGTTACGTCGCTCC

Bletilla_striata_AF273334 ACGAC-TCTCGGCAATGGATATCTC-GGCTCTCGCATCGATGAAGAGCGCAGCGAAATGCGATACGTGGTGC-GAATTGCAGAATCCCGCGAACC-ATCGAATCTTTGAACGCAAGTTGCGCCCGAGGCCAAC-CGGCCGAGGGCACGTCCGCCTG---GGCGTCAA-GCGTCGCGTCGCTCC

Bletilla_striata_AF461466 ACGAC-TCTCGGCAATGGATATCTC-GGCTCTCGCATCGATGAAGAGCGCAGCGAAATGCGATACGTGGTGC-GAATTGCAGAATCCCGCGAACC-ATCGAATCTTTGAACGCAAGTTGCGCCCGAGGCCAAC-CGGCCGAGGGCACGTCCGCCTG---GGCGTCAA-GCGTCGCGTCGCTCC

Bletilla_striata_EU100762 ACGAC-TCTCGGCAATGGATATCTC-GGCTCTCGCATCGATGAAGAGCGCAGCGAAATGCGATACGTGGTGC-GAATTGCAGAATCCCGCGAACC-ATCGAGTCTTTGAACGCAAGTTGCGCCCGAGGCCAAC-CGGCCGAGGGCACGTCCGCCTG---GGCGTCAA-GCGTCGCGTCGCTCC

Bletilla_striata_KF560509 ACGAC-TCTCGGCAATGGATATCTC-GGCTCTCGCATCGATGAAGAGCGCAGCGAAATGCGATACGTGGTGC-GAATTGCAGAATCCCGCGAACC-ATCGAGTCTTTGAACGCAAGTTGCGCCCGAGGCCAAC-CGGCCAAGGGCACGTCTGCCTG---GGCGTCAA-GCGTTGCGTCGCTCC

Bletilla_striata_KF560540 ACGAC-TCTCGGCAATGGATATCTC-GGCTCTCGCATCGATGAAGAGCGCAGCGAAATGCGATACGTGGTGC-GAATTGCAGAATCCCGCGAACC-ATCGAGTCTTTGAACGCAAGTTGCGCCCGAGGCCAAC-CGGCCAAGGGCACGTCTGCCTG---GGCGTCAA-GCGTTGCGTCGCTCC

Bletilla_striata_KF680524 ACGAC-TCTCGGCAATGGATATCTC-GGCTCTCGCATCGATGAAGAGCGCAGCGAAATGCGATACGTGGTGC-GAATTGCAGAATCCCGCGAACC-ATCGAATCTTTGAACGCAAGTTGCGCCCGAGGCCAAC-CGGCCGAGGGCACGTCCGCCTG---GGCGTCAA-GCGTCGCGTCGCTCC

Bletilla_striata_KF698622 ACGAC-TCTCGGCAATGGATATCTC-GGCTCTCGCATCGATGAAGAGCGCAGCGAAATGCGATACGTGGTGC-GAATTGCAGAATCCCGCGAACC-ATCGAATCTTTGAACGCAAGTTGCGCCCGAGGCCAAC-CGGCCGAGGGCACGTCCGCCTG---GGCGTCAA-GCGTCGCGTCGCTCC

Bletilla_striata_KF698623 ACGAC-TCTCGGCAATGGATATCTC-GGCTCTCGCATCGATGAAGAGCGCAGCGAAATGCGATACGTGGTGC-GAATTGCAGAATCCCGCGAACC-ATCGAATCTTTGAACGCAAGTTGCGCCCGAGGCCAAC-CGGCCGAGGGCACGTCCGCCTG---GGCGTCAA-GCGTCGCGTCGCTCC

Bletilla_striata_KFBG2048 ACGAC-TCTCGGCAATGGATATCTC-GGCTCTCGCATCGATGAAGAGCGCAGCGAAATGCGATACGTGGTGC-GAATTGCAGAATCCCGCGAACC-ATCGAGTCTTTGAACGCAAGTTGCGCCCGAGGCCAAC-CGGCCGAGGGCACGTCCGCCTG---GGCGTCAA-GCGTCGCGTCGCTCC

Bletilla_striata_KP866820 ACGAC-TCTCGGCAATGGATATCTC-GGCTCTCGCATCGATGAAGAGCGCAGCGAAATGCGATACGTGGTGC-GAATTGCAGAATCCCGCGAACC-ATCGAATTGCTGAACGCAAGTTGCGCCCGAGGCCAAC-CGGCCGAGGGCACGTCCGCCTG---GGCGTCAA-GCGTCGCGTCGCTCC

Bletilla_striata_KP866821 ACGAC-TCTCGGCAATGGATATCTC-GGCTCTCGCATGGATGAAGAGCGCAGCGAAATGCGATACGTGGTGC-GAATTGCAGAATCCCGCGAACC-ATCGAATCTTTGAACGCAAGTTGCGCCCGAGGCCAAC-CGGCCGAGGGCACGTCCGCCTG---GGCGTCAA-GCGTCGCGTCGCTCC

Bletilla_striata_KP866822 ACGAC-TCTCGGCAATGGATATCTC-GGCTCTCGCATCGATGAAGAGCGCAGCGAAATGCGATACGTGGTGC-GAATTGCAGAATCCCGCGAACC-ATCGAATCTTTGAACGCAAGTTGCGCCCGAGGCCAAC-CGGCCGAGGGCACGTCCGCCTG---GGCGTCAA-GCGTCGCGTCGCTCC

Bletilla_striata_KP866823 ACGAC-TCTCGGCAATGGATATCTC-GGCTCTCGCATCGATGAAGAGCGCAGCGAAATGCGATACGTGGTGC-GAATTGCAGAATCCCGCGAACC-ATCGAATCTTTGAACGCAAGTTGCGCCCGAGGCCAAC-CGGCCGAGGGCACGTCCGCCTG---GGCGTCAA-GCGTCGCGTCGCTCC

Bletilla_striata_KP866824 ACGAC----CGGCAATGGATATCTC-GGCTCTCGCATCGATGAAGAGCGCAGCGAAATGCGATACGTGGTGC-GAATTGCAGAATCCCGCGAACC-ATCGAATTGCTGAACGCAAGTTGCGCCCGAGGCCAAC-CGGCCGAGGGCACGTCCGCCTG---GGCGTCAA-GCGTCGCGTCGCTCC

Bletilla_striata_KP866825 ACGAC-TCTCGGCAATGGATATCTC-GGCTCTCGCATCGATGAAGAGCGCAGCGAAATGCGATACGTGGTGC-GAATTGCAGAATCCCGCGAACC-ATCTAATTGCTGAACGCAAGTTGCGCCCGAGGCCAAC-CGGCCGAGGGCACGTCCGCCTG---GGCGTCAA-GCGTCGCGTCGCTCC

Bletilla_striata_KP866826 ACGAC-TCTCGGCAATGGATATCTC-GGCTCTCGCATCGATGAAGAGCGCAGCGAAATGCGATACGTGGTGC-GAATTGCAGAATCCCGCGAACC-ATCGAATTGCTGAACGCAAGTTGCGCCCGAGGCCAAC-CGGCCGAGGGCACGTCCGCCTG---GGCGTCAA-GCGTCGCGTCGCTCC

Bletilla_striata_KP866827 ACGAC----CGTCAATGGATATCTC-GGCTCTCGCATCGATGAAGAGCGCAGCGAAATGCGATACGTGGTGC-GAATTGCAGAATCCCGCGAACC-ATCGAATTGCTGAACGCAAGTTGCGCCCGAGGCCAAC-CGGCCGAGGGCACGTCCGCCTG---GGCGTCAA-GCGTCGCGTCGCTCC

Bletilla_striata_KP866828 ACGAC----CGGCAATGGATATCTC-GGCTCTCGCATCGATGAAGAGCGCAGCGAAATGCGATACGTGGTGC-AAATTGCAGAATCCCGCGAACC-ATCGAATTGCTGAACGCAAGTTGCGCCCGAGGCCAAC-CAGCCGAGGGCACGTCCGCCTG---GGCGTCAA-GCGTCGCGTCGCTCC

Bletilla_striata_KT119555 ACGAC-TCTCGGCAATGGATATCTC-GGCTCTCGCATCGATGAAGAGCGCAGCGAAATGCGATACGTGGTGC-GAATTGCAGAATCCCGCGAACC-ATCGAGTCTTTGAACGCAAGTTGCGCCCGAGGCCAAC-CGGCCGAGGGCACGTCCGCCTG---GGCGTCAA-GCGTCGCGTCGCTCC

Bletilla_striata_KT338650 ACGAC-TCTCGGCAATGGATATCTC-GGCTCTCGCATCGATGAAGAGCGCAGCGAAATGCGATACGTGGTGC-GAATTGCAGAATCCCGCGAACC-ATCGAGTCTTTGAACGCAAGTTGCGCCCGAGGCCAAC-CGGCCGAGGGCACGTCCGCCTG---GGCGTCAA-GCGTCGCGTCGCTCC

Bletilla_striata_KY966421 ACGAC-TCTCGGCAATGGATATCTC-GGCTCTCGCATCGATGAAGAGCGCAGCGAAATGCGATACGTGGTGC-GAATTGCAGAATCCCGCGAACC-ATCGAGTCTTTGAACGCAAGTTGCGCCCGAGGCCAAC-CGGCCGAGGGCACGTCCGCCTG---GGCGTCAA-GCGTCGCGTCGCTCC

Brachycorythis_galeandra_PK12195 AGGGC-TCTCGGCAATGGATATCTT-GGCTCTCGCATCGATGAAGAGCGCAACGAAATGCGATACGTGGTGC-GAATTGCAGAATCCCGTGAACC-ATCGAGTTTTTGAACGCAAGTTGCGCCTGAGGCCAGC-TGGCCAAAGGCACGTCCGCCTG---GGCGTCAA-GCATTGAATCGCTCC

Brachycorythis_galeandra_SG1261 AGGGC-TCTCGGCAATGGATATCTT-GGCTCTCGCATCGATGAAGAGCGCAACGAAATGCGATACGTGGTGC-GAATTGCAGAATCCCGTGAACC-ATCGAGTTTTTGAACGCAAGTTGCGCCTGAGGCCAGC-TGGCCAAAGGCACGTCCGCCTG---GGCGTCAA-GCATTGAATCGCTCC

Bulbophyllum_affine_EF195916 ACGAC-TCTCGGCAATGGATATCTC-GGCTCTCGCATCGATGAAGAGCGCAGCGAAATGCGATACGTGGTGC-GAATTGCAGAATCCCGCGAACC-ATCGAGTCTTTGAACGCAAGTTGCGCCCGAGGCCAAC-CGGCCAAGGGCACGTCCGCCTG---GGCGTCAA-GCGTTGCGTCACTCC

Bulbophyllum_affine_KC568305 ACGAC-TCTCGGCAATGGATATCTC-GGCTCTCGCATCGATGAAGAGCGCAGCGAAATGCGATACGTGGTGC-GAATTGCAGAATCCCGCGAACC-ATCGAGTCTTTGAACGCAAGTTGCGCCCGAGGCCAAC-CGGCCAAGGGCACGTCCGCCTG---GGCGTCAA-GCGTTGCGTCACTCC

Bulbophyllum_affine_KF866246 ACGAC-TCTCGGCAATGGATATCTC-GGCTCTCGCATCGATGAAGAGCGCAGCGAAATGCGATACGTGGTGC-GAATTGCAGAATCCCGCGAACC-ATCGAGTCTTTGAACGCAAGTTGCGCCCGAGGCCAAC-CGGCCAAGGGCACGTCCGCCTG---GGCGTCAA-GCGTTGCGTCACTCC

Bulbophyllum_affine_KFBG412 ACGAC-TCTCGGCAATGGATATCTC-GGCTCTCGCATCGATGAAGAGCGCAGCGAAATGCGATACGTGGTGC-GAATTGCAGAATCCCGCGAACC-ATCGAGTCTTTGAACGCAAGTTGCGCCCGAGGCCAAC-CGGCCAAGGGCACGTCCGCCTG---GGCGTCAA-GCGTTGCGTCACTCC

Bulbophyllum_affine_KX455815 ACGAC-TCTCGGCAATGGATATCTC-GGCTCTCGCATCGATGAAGAGCGCAGCGAAATGCGATACGTGGTGC-GAATTGCAGAATCCCGCGAACC-ATCGAGTCTTTGAACGCAAGTTGCGCCCGAGGCCAAC-CGGCCAAGGGCACGTCCGCCTG---GGCGTCAA-GCGTTGCGTCACTCC

Bulbophyllum_affine_KY966422 ACGAC-TCTCGGCAATGGATATCTC-GGCTCTCGCATCGATGAAGAGCGCAGCGAAATGCGATACGTGGTGC-GAATTGCAGAATCCCGCGAACC-ATCGAGTCTTTGAACGCAAGTTGCGCCCGAGGCCAAC-CGGCCAAGGGCACGTCCGCCTG---GGCGTCAA-GCGTTGCGTCACTCC

Bulbophyllum_affine_MK164425 ACGAC-TCTCGGCAATGGATATCTC-GGCTCTCGCATCGATGAAGAGCGCAGCGAAATGCGATACGTGGTGC-GAATTGCAGAATCCCGCGAACC-ATCGAGTCTTTGAACGCAAGTTGCGCCCGAGGCCAAC-CGGCCAAGGGCACGTCCGCCTG---GGCGTCAA-GCGTTGCGTCACTCC

Bulbophyllum_affine_SG1606 ACGAC-TCTCGGCAATGGATATCTC-GGCTCTCGCATCGATGAAGAGCGCAGCGAAATGCGATACGTGGTGC-GAATTGCAGAATCCCGCGAACC-ATCGAGTCTTTGAACGCAAGTTGCGCCCGAGGCCAAC-CGGCCAAGGGCACGTCCGCCTG---GGCGTCAA-GCGTTGCGTCACTCC

Bulbophyllum_affine_SG1607 ACGAC-TCTCGGCAATGGATATCTC-GGCTCTCGCATCGATGAAGAGCGCAGCGAAATGCGATACGTGGTGC-GAATTGCAGAATCCCGCGAACC-ATCGAGTCTTTGAACGCAAGTTGCGCCCGAGGCCAAC-CGGCCAAGGGCACGTCCGCCTG---GGCGTCAA-GCGTTGCGTCACTCC

Bulbophyllum_ambrosia_JN619413 ACGAC-TCTCGGCAATGGATATCTC-GGCTCTCGCATCGATGAAGAGCGCAGCGAAATGCGATACGTGGTGC-GAATTGCAGAATCCCGCGAACC-ATCGAGTCTTTGAACGCAAGTTGCGCCCGAGGCCGAC-CGGCCGAGGGCACGTCCGCCTG---GGCGTCAA-GCGTTGCGCCGCTCC

Bulbophyllum_ambrosia_KC568306 ACGAC-TCTCGGCAATGGATATCTC-GGCTCTCGCATCGATGAAGAGCGCAGCGAAATGCGATACGTGGTGC-GAATTGCAGAATCCCGCGAACC-ATCGAGTCTTTGAACGCAAGTTGCGCCCGAGGCCAAC-CGGCCGAGGGCACGTCCGCCTG---GGCGTCAA-GCGTTGCGTCGCTCC

Bulbophyllum_ambrosia_KY966423 ATCAC-TCTCAGCAATTGATTTCTC-GGCTTTCACATCGATGAAGAGAGCAGCGAAATGCGATACGTGGTGT-GAATTGCAGAATCCTGCAAACC-ATCGAGTCTTTGAACGCAAGTTACGCCCCAGGCCAAC-CGGCCGAGGGCACGTCCGCCTG---GGCATCAA-GCGTTGCGTCGCTCA

Bulbophyllum_ambrosia_KY966424 ACGAC-TCTCGGCAATGGATATCTC-GGCTCTCGCATCGATGAAGAGCGCAGCGAAATGCGATACGTGGTGC-GAATTGCAGAATCCCGCGAACC-ATCGAGTCTTTGAACGCAAGTTGCGCCCGAGGCCAAC-CGGCCGAGGGCACGTCCGCCTG---GGCGTCAA-GCGTTGCGTCGCTCC

Bulbophyllum_ambrosia_KY966425 ACGAC-TCTCGGCAATGGATATCTC-GGCTCTCGCATCGATGAAGAGCGCAGCGAAATGCGATACGTGGTGC-GAATTGCAGAATCCCGCGAACC-ATCGAGTCTTTGAACGCAAGTTGCGCCCGAGGCCAAC-CGGCCGAGGGCACGTCCGCCTG---GGCGTCAA-GCGTTGCGTCGCTCC

Bulbophyllum_ambrosia_KY966426 ACGAC-TCTCGGCAATGGATATCTC-GGCTCTCGCATCGATGAAGAGCGCAGCGAAATGCGATACGTGGTGC-GAATTGCAGAATCCCGCGAACC-ATCGAGTCTTTGAACGCAAGTTGCGCCCGAGGCCAAC-CGGCCGAGGGCACGTCCGCCTG---GGCGTCAA-GCGTTGCGTCGCTCC

Bulbophyllum_ambrosia_MK164427 ACGAC-TCTCGGCAATGGATATCTC-GGCTCTCGCATCGATGAAGAGCGCAGCGAAATGCGATACGTGGTGC-GAATTGCAGAATCCCGCGAACC-ATCGAGTCTTTGAACGCAAGTTGCGCCCGAGGCCAAC-CGGCCGAGGGCACGTCCGCCTG---GGCGTCAA-GCGTTGCGTCGCTCC

Bulbophyllum_ambrosia_PK12090 ACGAC-TCTCGGCAATGGATATCTC-GGCTCTCGCATCGATGAAGAGCGCAGCGAAATGCGATACGTGGTGC-GAATTGCAGAATCCCGCGAACC-ATCGAGTCTTTGAACGCAAGTTGCGCCCGAGGCCAGC-CGGCCGAGGGCACGTCCGCCTG---GGCGTCAA-GCGTTGCGTCGCTCC

Bulbophyllum_ambrosia_PK12092 ACGAC-TCTCGGCAATGGATATCTC-GGCTCTCGCATCGATGAAGAGCGCAGCGAAATGCGATACGTGGTGC-GAATTGCAGAATCCCGCGAACC-ATCGAGTCTTTGAACGCAAGTTGCGCCCGAGGCCAAC-CGGCCGAGGGCACGTCCGCCTG---GGCGTCAA-GCGTTGCGTCGCTCC

Bulbophyllum_ambrosia_SG1221 ACGAC-TCTCGGCAATGGATATCTC-GGCTCTCGCATCGATGAAGAGCGCAGCGAAATGCGATACGTGGTGC-GAATTGCAGAATCCCGCGAACC-ATCGAGTCTTTGAACGCAAGTTGCGCCCGAGGCCAAC-CGGCCGAGGGCACGTCCGCCTG---GGCGTCAA-GCGTTGCGTCGCTCC

Bulbophyllum_bicolor_CL10 ACGAC-TCTCGGCAATGGATATCTC-GGCTCTCGCATCGATGAAGAGCGCAGCGAAATGCGATACGTGGTGC-GAATTGCAGAATCCCGCGAACC-ATCGAGTCTTTGAACGCAAGTTGCGCCCGAGGCCAGC-CGGCCGAGGGCACGTCCGCCTG---GGCGTCAA-GCGTTGCGTCGCTCC

Bulbophyllum_bicolor_FT28 ACGAC-TCTCGGCAATGGATATCTC-GGCTCTCGCATCGATGAAGAGCGCAGCGAAATGCGATACGTGGTGC-GAATTGCAGAATCCCGCGAACC-ATCGAGTCTTTGAACGCAAGTTGCGCCCGAGGCCAGC-CGGCCGAGGGCACGTCCGCCTG---GGCGTCAA-GCGTTGCGTCGCTCC

Bulbophyllum_bicolor_KFBG2210 ACGAC-TCTCGGCAATGGATATCTC-GGCTCTCGCATCGATGAAGAGCGCAGCGAAATGCGATACGTGGTGC-GAATTGCAGAATCCCGCGAACC-ATCGAGTCTTTGAACGCAAGTTGCGCCCGAGGCCAGC-CGGCCGAGGGCACGTCCGCCTG---GGCGTCAA-GCGTTGCGTCGCTCC

Bulbophyllum_bicolor_KFBG3073 ACGAC-TCTCGGCAATGGATATCTC-GGCTCTCGCATCGATGAAGAGCGCAGCGAAATGCGATACGTGGTGC-GAATTGCAGAATCCCGCGAACC-ATCGAGTCTTTGAACGCAAGTTGCGCCCGAGGCCAGC-CGGCCGAGGGCACGTCCGCCTG---GGCGTCAA-GCGTTGCGTCGCTCC

Bulbophyllum_bicolor_KFBG433A ACGAC-TCTCGGCAATGGATATCTC-GGCTCTCGCATCGATGAAGAGCGCAGCGAAATGCGATACGTGGTGC-GAATTGCAGAATCCCGCGAACC-ATCGAGTCTTTGAACGCAAGTTGCGCCCGAGGCCAGC-CGGCCGAGGGCACGTCCGCCTG---GGCGTCAA-GCGTTGCGTCGCTCC

Bulbophyllum_bicolor_KFBG445 ACGAC-TCTCGGCAATGGATATCTC-GGCTCTCGCATCGATGAAGAGCGCAGCGAAATGCGATACGTGGTGC-GAATTGCAGAATCCCGCGAACC-ATCGAGTCTTTGAACGCAAGTTGCGCCCGAGGCCAAC-CGGCCGAGGGCACGTCCGCCTG---GGCGTCAA-GCGTTGCGTCGCTCC

Bulbophyllum_bicolor_KY022455 ACGAC-TCTCGGCAATGGATATCTC-GGCTCTCGCATCGATGAAGAGCGCAGCGAAATGCGATACGTGGTGC-GAATTGCAGAATCCCGCGAACC-ATCGAGTCTTTGAACGCAAGTTGCGCCCGAGGCCAGC-CGGCCGAGGGCACGTCCGCCTG---GGCGTCAA-GCGTTGCGTCGCTCC

Bulbophyllum_bicolor_KY966430 ACGAC-TCTCGGCAATGGATATCTC-GGCTCTCGCATCGATGAAGAGCGCAGCGAAATGCGATACGTGGTGC-GAATTGCAGAATCCCGCGAACC-ATCGAGTCTTTGAACGCAAGTTGCGCCCGAGGCCAGC-CGGCCGAGGGCACGTCCGCCTG---GGCGTCAA-GCGTTGCGTCGCTCC

Bulbophyllum_bicolor_KY966431 ACGAC-TCTCGGCAATGGATATCTC-GGCTCTCGCATCGATGAAGAGCGCAGCGAAATGCGATACGTGGTGC-GAATTGCAGAATCCCGCGAACC-ATCGAGTCTTTGAACGCAAGTTGCGCCCGAGGCCAGC-CGGCCGAGGGCACGTCCGCCTG---GGCGTCAA-GCGTTGCGTCGCTCC

Bulbophyllum_bicolor_KY966432 ACGAC-TCTCGGCAATGGATATCTC-GGCTCTCGCATCGATGAAGAGCGCAGCGAAATGCGATACGTGGTGC-GAATTGCAGAATCCCGCGAACC-ATCGAGTCTTTGAACGCAAGTTGCGCCCGAGGCCAGC-CGGCCGAGGGCACGTCCGCCTG---GGCGTCAA-GCGTTGCGTCGCTCC

Bulbophyllum_bicolor_LMP19 ACGAC-TCTCGGCAATGGATATCTC-GGCTCTCGCATCGATGAAGAGCGCAGCGAAATGCGATACGTGGTGC-GAATTGCAGAATCCCGCGAACC-ATCGAGTCTTTGAACGCAAGTTGCGCCCGAGGCCAGC-CGGCCGAGGGCACGTCCGCCTG---GGCGTCAA-GCGTTGCGTCGCTCC

Bulbophyllum_bicolor_MK164432 ACGAC-TCTCGGCAATGGATATCTC-GGCTCTCGCATCGATGAAGAGCGCAGCGAAATGCGATACGTGGTGC-GAATTGCAGAATCCCGCGAACC-ATCGAGTCTTTGAACGCAAGTTGCGCCCGAGGCCAGC-CGGCCGAGGGCACGTCCGCCTG---GGCGTCAA-GCGTTGCGTCGCTCC

Bulbophyllum_bicolor_MK164433 ACGAC-TCTCGGCAATGGATATCTC-GGCTCTCGCATCGATGAAGAGCGCAGCGAAATGCGATACGTGGTGC-GAATTGCAGAATCCCGCGAACC-ATCGAGTCTTTGAACGCAAGTTGCGCCCGAGGCCAGC-CGGCCGAGGGCACGTCCGCCTG---GGCGTCAA-GCGTTGCGTCGCTCC

Bulbophyllum_bicolor_PSL45 ACGAC-TCTCGGCAATGGATATCTC-GGCTCTCGCATCGATGAAGAGCGCAGCGAAATGCGATACGTGGTGC-GAATTGCAGAATCCCGCGAACC-ATCGAGTCTTTGAACGCAAGTTGCGCCCGAGGCCAGC-CGGCCGAGGGCACGTCCGCCTG---GGCGTCAA-GCGTTGCGTCGCTCC

Bulbophyllum_bicolor_TMS05 ACGAC-TCTCGGCAATGGATATCTC-GGCTCTCGCATCGATGAAGAGCGCAGCGAAATGCGATACGTGGTGC-GAATTGCAGAATCCCGCGAACC-ATCGAGTCTTTGAACGCAAGTTGCGCCCGAGGCCAGC-CGGCCGAGGGCACGTCCGCCTG---GGCGTCAA-GCGTTGCGTCGCTCC

Bulbophyllum_bicolor_TT43 ACGAC-TCTCGGCAATGGATATCTC-GGCTCTCGCATCGATGAAGAGCGCAGCGAAATGCGATACGTGGTGC-GAATTGCAGAATCCCGCGAACC-ATCGAGTCTTTGAACGCAAGTTGCGCCCGAGGCCAGC-CGGCCGAGGGCACGTCCGCCTG---GGCGTCAA-GCGTTGCGTCGCTCC

Bulbophyllum_delitescens_KY966439 ACGAC-TCTCGGCAATGGATATCTC-GGCTCTCGCATCGATGAAGAGCGCAGCGAAATGCGATACGTGGTGC-GAATTGCAGAATCCCGCGAACC-ATCGAGTCTTTGAACGCAAGTTGCGCCCGAGGCCAAC-CGGCCGAGGGCACGTCCGCCTG---GGCGTCAA-GCGTTGCGTCGCTCC

Bulbophyllum_delitescens_KY966440 ACGAC-TCTCGGCAATGGATATCTC-GGCTCTCGCATCGATGAAGAGCGCAGCGAAATGCGATACGTGGTGC-GAATTGCAGAATCCCGCGAACC-ATCGAGTCTTTGAACGCAAGTTGCGCCCGAGGCCAAC-CGGCCGAGGGCACGTCCGCCTG---GGCGTCAA-GCGTTGCGTCGCTCC

Bulbophyllum_delitescens_KY966441 ACGAC-TCTCGGCAATGGATATCTC-GGCTCTCGCATCGATGAAGAGCGCAGCGAAATGCGATACGTGGTGC-GAATTGCAGAATCCCGCGAACC-ATCGAGTCTTTGAACGCAAGTTGCGCCCGAGGCCAAC-CGGCCGAGGGCACGTCCGCCTG---GGCGTCAA-GCGTTGCGTCGCTCC

Bulbophyllum_delitescens_KY966442 ATGAC-TCTCGGCAATGAATATCTC-AGCTCTCGCATCGATGAAGAGCACAGCAAAATGCAATACATTGTGT-GAATTGCAGAATCCTATGAACT-ATCAAGTCTTTGAATGCAAGTTGTACCCAAGGCCAAC-CGGCCAAGGGCACGTCTGCCTG---GGCGTCAA-GCGTTGTGTTTCTCC

Bulbophyllum_delitescens_MK164448 ACGAC-TCTCGGCAATGGATATCTC-GGCTCTCGCATCGATGAAGAGCGCAGCGAAATGCGATACGTGGTGC-GAATTGCAGAATCCCGCGAACC-ATCGAGTCTTTGAACGCAAGTTGCGCCCGAGGCCAAC-CGGCCGAGGGCACGTCCGCCTG---GGCGTCAA-GCGTTGCGTCGCTCC

Bulbophyllum_delitescens_SG1286 ACGAC-TCTCGGCAATGGATATCTC-GGCTCTCGCATCGATGAAGAGCGCAGCGAAATGCGATACGTGGTGC-GAATTGCAGAATCCCGCGAACC-ATCGAGTCTTTGAACGCAAGTTGCGCCCGAGGCCAAC-CGGCCGAGGGCACGTCCGCCTG---GGCGTCAA-GCGTTGCGTCGCTCC

Bulbophyllum_delitescens_SG1287 ACGAC-TCTCGGCAATGGATATCTC-GGCTCTCGCATCGATGAAGAGCGCAGCGAAATGCGATACGTGGTGC-GAATTGCAGAATCCCGCGAACC-ATCGAGTCTTTGAACGCAAGTTGCGCCCGAGGCCGAC-CGGCCGAGGGCACGTCCGCCTG---GGCGTCAA-GCGTTGCGTCGCTCC

Bulbophyllum_delitescens_SG1288 ACGAC-TCTCGGCAATGGATATCTC-GGCTCTCGCATCGATGAAGAGCGCAGCGAAATGCGATACGTGGTGC-GAATTGCAGAATCCCGCGAACC-ATCGAGTCTTTGAACGCAAGTTGCGCCCGAGGCCGAC-CGGCCGAGGGCACGTCCGCCTG---GGCGTCAA-GCGTTGCGTCGCTCC

Bulbophyllum_kwangtungense_JN619414 ACGAC-TCTCGGCAATGGATATCTC-GGCTCTCGCATCGATGAAGAGCGCAGCGAAATGCGATACGTGGTGC-GAATTGCAGAATCACGCGAACC-ATCGAGTCTTTGAACGCAAGTTGCGCCCGAGGCCAAC-CGGCCGAGGGCACGTCCGCCTG---GGCGTCGA-GCGTTGCGTCGCTCC

Bulbophyllum_kwangtungense_KFBG27001 ACGAC-TCTCGGCAATGGATATCTC-GGCTCTCGCATCGATGAAGAGCGCAGCGAAATGCGATACGTGGTGC-GAATTGCAGAATCCCGCGAACC-ATCGAGTCTTTGAACGCAAGTTGCGCCCGAGGCCAAC-CGGCCGAGGGCACGTCCGCCTG---GGCGTCAA-GCGTTGCGCCGCTCC

Bulbophyllum_kwangtungense_KFBG2798 ACGAC-TCTCGGCAATGGATATCTC-GGCTCTCGCATCGATGAAGAGCGCAGCGAAATGCGATACGTGGTGC-GAATTGCAGAATCACGCGAACC-ATCGAGTCTTTGAACGCAAGTTGCGCCCGAGGCCAAC-CGGCCGAGGGCACGTCCGCCTG---GGCGTCAA-GCGTTGCGTCGCTCC

Bulbophyllum_kwangtungense_KFBG2820 ACGAC-TCTCGGCAATGGATATCTC-GGCTCTCGCATCGATGAAGAGCGCAGCGAAATGCGATACGTGGTGC-GAATTGCAGAATCACGCGAACC-ATCGAGTCTTTGAACGCAAGTTGCGCCCGAGGCCAAC-CGGCCGAGGGCACGTCCGCCTG---GGCGTCAA-GCGTTGCGTCGCTCC

Bulbophyllum_kwangtungense_MK164466 ACGAC-TCTCGGCAATGGATATCTC-GGCTCTCGCATCGATGAAGAGCGCAGCGAAATGCGATACGTGGTGC-GAATTGCAGAATCACGCGAACC-ATCGAGTCTTTGAACGCAAGTTGCGCCCGAGGCCAAC-CGGCCGAGGGCACGTCCGCCTG---GGCGTCAA-GCGTTGCGTCGCTCC

Bulbophyllum_kwangtungense_PK12070 ACGAC-TCTCGGCAATGGATATCTC-GGCTCTCGCATCGATGAAGAGCGCAGCGAAATGCGATACGTGGTGC-GAATTGCAGAATCACGCGAACC-ATCGAGTCTTTGAACGCAAGTTGCGCCCGAGGCCAAC-CGGCCGAGGGCACGTCCGCCTG---GGCGTCAA-GCGTTGCGTCGCTCC

Bulbophyllum_odoratissimum_FJ428222 ATGAC-TCTCGGCAATGGATATCTC-GGCTCTTGCATCGATGAAGAGCGCAGCGAAATGCGATACGTGGTGC-GAATTGCAGAATCCCGCGAACC-ATCGAGTCTTTGAACGCAAGTTGCGCCCGAGGCCAAC-CGGCCAAGGGCACGTTTGCCTG---GGCGTCAA-GCGTTGCGTCGCTTC

Bulbophyllum_odoratissimum_FJ428223 ATGAC-TCTCGGCAATGGATATCTC-GGCTCTTGCATCGATGAAGAGCGCAGCGAAATGCGATACGTGGTGC-GAATTGCAGAATCCCGCGAACC-ATCGAGTCTTTGAACGCAAGTTGCGCCCGAGGCCAAC-CGGCCAAGGGCACGTTTGCCTG---GGCGTCAA-GCGTTGCGTCGCTTC

Bulbophyllum_odoratissimum_HQ114230 ACGAC-TCTCGGCAATGGATATCTC-GGCTCTCGCATCGATGAAGAGCGCAGCGAAATGCGATACGTGGTGC-GAATTGCAGAATCCCGCGAACC-ATCGAGTCTTTGAACGCAAGTTGCGCCCGAGGCCAAC-CGGCCGAGGGCACGTCCGCCTG---GGCGTCAA-GCGTTGCGCCGCTCC

Bulbophyllum_odoratissimum_KF866242 ACGAC-TCTCGGCAATGGATATCTC-GGCTCTCGCATCGATGAAGAGCGCAGCGAAATGCGATACGTGGTGC-GAATTGCAGAATCCCGCGAACC-ATCGAGTCTTTGAACGCAAGTTGCGCCCGAGGCCAAC-CGGCCGAGGGCACGTCCGCCTG---GGCGTCAA-GCGTTGCGCCGCTCC

Bulbophyllum_odoratissimum_KP265007 ATGAC-TCTCGGCAATGGATATCTC-GGCTCTTGCATCGATGAAGAGCGCAGCGAAATGCGATACGTGGTGC-GAATTGCAGAATCCCGCGAACC-ATCGAGTCTTTGAACGCAAGTTGCGCCCGAGGCCAAC-CGGCCAAGGGCACGTTTGCCTG---GGCGTCAA-GCGTTGCGTCGCTTC

Bulbophyllum_odoratissimum_KY966466 ACGAC-TCTCGGCAATGGATATCTC-GGCTCTCGCATCGATGAAGAGCGCAGCGAAATGCGATACGTGGTGC-GAATTGCAGAATCCCGCGAACC-ATCGAGTCTTTGAACGCAAGTTGCGCCCGAGGCCAAC-CGGCCGAGGGCACGTCCGCCTG---GGCGTCAA-GCGTTGCGCCGCTCC

Bulbophyllum_odoratissimum_KY966467 ACGAC-TCTCGGCAATGGATATCTC-GGCTCTCGCATCGATGAAGAGCGCAGCGAAATGCGATACGTGGTGC-GAATTGCAGAATCACGCGAACC-ATCGAGTCTTTGAACGCAAGTTGCGCCCGAGGCCAAC-CGGCCGAGGGCACGTCCGCCTG---GGCGTCAA-GCGTTGCGTCGCTCC

Bulbophyllum_odoratissimum_MK164483 ACGAC-TCTCGGCAATGGATATCTC-GGCTCTCGCATCGATGAAGAGCGCAGCGAAATGCGATACGTGGTGC-GAATTGCAGAATCCCGCGAACC-ATCGAGTCTTTGAACGCAAGTTGCGCCCGAGGCCAAC-CGGCCGAGGGCACGTCCGCCTG---GGCGTCAA-GCGTTGCGCCGCTCC

Bulbophyllum_odoratissimum_PK12119 ACGAC-TCTCGGCAATGGATATCTC-GGCTCTCGCATCGATGAAGAGCGCAGCGAAATGCGATACGTGGTGC-GAATTGCAGAATCCCGCGAACC-ATCGAGTCTTTGAACGCAAGTTGCGCCCGAGGCCAAC-CGGCCGAGGGCACGTCCGCCTG---GGCGTCAA-GCGTTGCGCCGCTCC

Bulbophyllum_odoratissimum_SG1275 ACGAC-TCTCGGCAATGGATATCTC-GGCTCTCGCATCGATGAAGAGCGCAGCGAAATGCGATACGTGGTGC-GAATTGCAGAATCCCGCGAACC-ATCGAGTCTTTGAACGCAAGTTGCGCCCGAGGCCAAC-CGGCCGAGGGCACGTCCGCCTG---GGCGTCAA-GCGTTGCGCCGCTCC

Bulbophyllum_pectenveneris_JN619418 ACGAC-TCTCGGCAATGGATATCTC-GGCTCTCGCATCGATGAAGAGCGCAGCGAAATGCGATACGTGGTGC-GAATTGCAGAATCCCGCGAACC-ATCGAGTCTTTGAACGCAAGTTGCGCCCGAGGCCGAC-CGGCCGAGGGCACGTCCGCCTG---GGCGTCAA-GCGTTGCGTCGCTCC

Bulbophyllum_pectenveneris_KY966470 ACGAC-TCTCGGCAATGGATATCTC-GGCTCTCGCATCGATGAAGAGCGCAGCGAAATGCGATACGTGGTGC-GAATTGCAGAATCCCGCGAACC-ATCGAGTCTTTGAACGCAAGTTGCGCCCGAGGCCGAC-CGGCCGAGGGCACGTCCGCCTG---GGCGTCGA-GCGTTGCGTCGCTCC

Bulbophyllum_pectenveneris_KY966471 ACGAC-TCTCGGCAATGGATATCTC-GGCTCTCGCATCGATGAAGAGCGCAGCGAAATGCGATACGTGGTGC-GAATTGCAGAATCCCGCGAACC-ATCGAGTCTTTGAACGCAAGTTGCGCCCGAGGCCGAC-CGGCCGAGGGCACGTCCGCCTG---GGCGTCGA-GCGTTGCGTCGCTCC

Bulbophyllum_pectenveneris_MK164486 ACGAC-TCTCGGCAATGGATATCTC-GGCTCTCGCATCGATGAAGAGCGCAGCGAAATGCGATACGTGGTGC-GAATTGCAGAATCCCGCGAACC-ATCGAGTCTTTGAACGCAAGTTGCGCCCGAGGCCGAC-CGGCCGAGGGCACGTCCGCCTG---GGCGTCGA-GCGTTGCGTCGCTCC

Bulbophyllum_scabratum_JN619409 ACGAC-TCTCGGCAATGGATATCTC-GGCTCTCGCATCGATGAAGAGCGCAGCGAAATGCGATACGTGGTGC-GAATTGCAGAATCCCGCGAACC-ATCGAGTCTTTGAACGCAAGTTGCGCCCGAGGCCGAC-CGGCCGAGGGCACGTCCGCCTG---GGCGTCAA-GCGTTGCGTCGCTCC

Bulbophyllum_scabratum_KY966453 ACGAC-TCTCGGCAATGGATATCTC-GGCTCTCGCATCGATGAAGAGCGCAGCGAAATGCGATGCGTGGTGC-GAATTGCAGAATCCCGCGAACC-ATCGAGTCTTTGAACGCAAGTTGCGCCCGAGGCCGAC-CGGCCGAGGGCACGCCCGCCTG---GGCGTCAA-GCGTCGCGTCGCTCC

Bulbophyllum_scabratum_MK164471 ACGAC-TCTCGGCAATGGATATCTC-GGCTCTCGCATCGATGAAGAGCGCAGCGAAATGCGATACGTGGTGC-GAATTGCAGAATCCCGCGAACC-ATCGAGTCTTTGAACGCAAGTTGCGCCCGAGGCCGAC-CGGCCGAGGGCACGCCCGCCTG---GGCGTCAA-GCGTCGCGTCGCTCC

Bulbophyllum_scabratum_PK12041 ACGAC-TCTCGGCAATGGATATCTC-GGCTCTCGCATCGATGAAGAGCGCAGCGAAATGCGATACGTGGTGC-GAATTGCAGAATCCCGCGAACC-ATCGAGTCTTTGAACGCAAGTTGCGCCCGAGGCCGAC-CGGCCGAGGGCACGCCCGCCTG---GGCGTCAA-GCGTCGCGTCGCTCC

Bulbophyllum_scabratum_PK12126 ACGAC-TCTCGGCAATGGATATCTC-GGCTCTCGCATCGATGAAGAGCGCAGCGAAATGCGATACGTGGTGC-GAATTGCAGAATCCCGCGAACC-ATCGAGTCTTTGAACGCAAGTTGCGCCCGAGGCCGAC-CGGCCGAGGGCACGCCCGCCTG---GGCGTCAA-GCGTCGCGTCGCTCC

Bulbophyllum_scabratum_PK12127 ACGAC-TCTCGGCAATGGATATCTC-GGCTCTCGCATCGATGAAGAGCGCAGCGAAATGCGATACGTGGTGC-GAATTGCAGAATCCCGCGAACC-ATCGAGTCTTTGAACGCAAGTTGCGCCCGAGGCCGAC-CGGCCGAGGGCACGCCCGCCTG---GGCGTCAA-GCGTCGCGTCGCTCC

Bulbophyllum_stenobulbon_KFBG2806 ACGAC-TCTCGGCAATGGATATCTC-GGCTCTCGCATCGATGAAGAGCGCAGCGAAATGCGATACGTGGTGC-GAATTGCAGAATCACGCGAACC-ATCGAGTCTTTGAACGCAAGTTGCGCCCGAGGCCAAC-CGGCCGAGGGCACGTCCGCCTG---GGCGTCAA-GCGTTGCGTCGCTCC

Bulbophyllum_stenobulbon_MK164513 ACGAC-TCTCGGCAATGGATATCTC-GGCTCTCGCATCGATGAAGAGCGCAGCGAAATGCGATACGTGGTGC-GAATTGCAGAATCACGCGAACC-ATCGAGTCTTTGAACGCAAGTTGCGCCCGAGGCCAAC-CGGCCGAGGGCACGTCCGCCTG---GGCGTCAA-GCGTTGCGTCGCTCC

Bulbophyllum_stenobulbon_PK12111 ACGAC-TCTCGGCAATGGATATCTC-GGCTCTCGCATCGATGAAGAGCGCAGCGAAATGCGATACGTGGTGC-GAATTGCAGAATCACGCGAACC-ATCGAGTCTTTGAACGCAAGTTGCGCCCGAGGCCAAC-CGGCCGAGGGCACGTCCGCCTG---GGCGTCAA-GCGTTGCGTCGCTCC

Bulbophyllum_stenobulbon_PK12112 ACGAC-TCTCGGCAATGGATATCTC-GGCTCTCGCATCGATGAAGAGCGCAGCGAAATGCGATACGTGGTGC-GAATTGCAGAATCACGCGAACC-ATCGAGTCTTTGAACGCAAGTTGCGCCCGAGGCCAAC-CGGCCGAGGGCACGTCCGCCTG---GGCGTCAA-GCGTTGCGTCGCTCC

Bulbophyllum_stenobulbon_PK12113 ACGAC-TCTCGGCAATGGATATCTC-GGCTCTCGCATCGATGAAGAGCGCAGCGAAATGCGATACGTGGTGC-GAATTGCAGAATCACGCGAACC-ATCGAGTCTTTGAACGCAAGTTGCGCCCGAGGCCAAC-CGGCCGAGGGCACGTCCGCCTG---GGCGTCAA-GCGTTGCGTCGCTCC

Bulbophyllum_stenobulbon_SG1226 ACGAC-TCTCGGCAATGGATATCTC-GGCTCTCGCATCGATGAAGAGCGCAGCGAAATGCGATACGTGGTGC-GAATTGCAGAATCACGCGAACC-ATCGAGTCTTTGAACGCAAGTTGCGCCCGAGGCCAAC-CGGCCGAGGGCACGTCCGCCTG---GGCGTCAA-GCGTTGCGTCGCTCC

Bulbophyllum_tigridum_KFBG468 ACGAC-TCTCGGCAATGGATATCTC-GGCTCTCGCATCGATGAAGAGCGCAGCGAAATGCGATACGTGGTGC-GAATTGCAGAATCCCGCGAACC-ATCGAGTCTTTGAACGCAAGTTGCGCCCGAGGCCAGC-CGGCCGAGGGCACGTCYGCCTG---GGCGTCAA-GCGTTGCGTCGCTCC

Bulbophyllum_tigridum_KX455820 ACGAC-TCTCGGCAATGGATATCTC-GGCTCTCGCATCGATGAAGAGCGCAGCGAAATGCGATACGTGGTGC-GAATTGCAGAATCCCGCGAACC-ATCGAGTCTTTGAACGCAAGTTGCGCCCGAGGCCAAC-CGGCCGAGGGCACGTCCGCCTG---GGCGTCAA-GCGTTGCGTCGCTCC

Bulbophyllum_tigridum_MK164520 ACGAC-TCTCGGCAATGGATATCTC-GGCTCTCGCATCGATGAAGAGCGCAGCGAAATGCGATACGTGGTGC-GAATTGCAGAATCCCGCGAACC-ATCGAGTCTTTGAACGCAAGTTGCGCCCGAGGCCAGC-CGGCCGAGGGCACGTCCGCCTG---GGCGTCAA-GCGTTGCGTCGCTCC

Bulbophyllum_tigridum_SG1310 ACGAC-TCTCGGCAATGGATATCTC-GGCTCTCGCATCGATGAAGAGCGCAGCGAAATGCGATACGTGGTGC-GAATTGCAGAATCCCGCGAACC-ATCGAGTCTTTGAACGCAAGTTGCGCCCGAGGCCAGC-CGGCCGAGGGCACGTCCGCCTG---GGCGTCAA-GCGTTGCGTCGCTCC

Bulbophyllum_tseanum_MK164524 ACGAC-TCTCGGCAATGGATATCTC-GGCTCTCGCATCGATGAAGAGCGCAGCGAAATGCGATACGTGGTGC-GAATTGCAGAATCCCGCGAACC-ATCGAGTCTTTGAACGCAAGTTGCGCCCGAGGCCAAC-CGGCCGAGGGCACGTCCGCCTG---GGCGTCAA-GCGTCGCGTCGCTCC

Bulbophyllum_tseanum_SG1272 ACGAC-TCTCGGCAATGGATATCTC-GGCTCTCGCATCGATGAAGAGCGCAGCGAAATGCGATACGTGGTGC-GAATTGCAGAATCCCGCGAACC-ATCGAGTCTTTGAACGCAAGTTGCGCCCGAGGCCAAC-CGGCCGAGGGCACGTCCGCCTG---GGCGTCAA-GCGTCGCGTCGCTCC

Bulbophyllum_tseanum_SG1616 ACGAC-TCTCGGCAATGGATATCTC-GGCTCTCGCATCGATGAAGAGCGCAGCGAAATGCGATACGTGGTGC-GAATTGCAGAATCCCGCGAACC-ATCGAGTCTTTGAACGCAAGTTGCGCCCGAGGCCAAC-CGGCCGAGGGCACGTCCGCCTG---GGCGTCAA-GCGTCGCGTCGCTCC

Calanthe_dominyi_SG1359 ATGAC-TCTCGGCAATGGATATCTC-GGCTCTCGCATCGATGAAGAGCGCAGCGAAATGCGATACGTGGTGC-GAATTGCAGAATCCCGCGAACC-ATCGAGTCTTTGAACGCAAGTTGCGCCCGAGGCCAAT-CGGCCAAGGGCACGTCTGCCTG---GGCGTCAA-GCGTTGCATCGCTCT

Calanthe_graciliflora_AY882608 ATGAC-TCTCGGCAATGGATATCTC-GGCTCTCGCATCGATGAAGAGCGCAGCGAAATGCGATACGTGGTGC-GAATTGCAGAATCCCGCGAACC-ATCGAGTCTTTGAACGCAAGTTGCGCCCGAGGCCAAT-CGGCCAAGGGCACGTCTGCCTG---GGCGTCAA-GCGTTGCATCGCTCT

Calanthe_graciliflora_KF560484 ATGAC-TCTCGGCAATGGATATCTC-GGCTCTCGCATCGATGAAGAGCGCAGCGAAATGCGATACGTGGTGC-GAATTGCAGAATCCCGCGAACC-ATCGAGTCTTTGAACGCAAGTTGCGCCCGAGGCCAAT-CGGCCAAGGGCACGTCTGCCTG---GGCGTCAA-GCGTTGCATCGCTCT

Calanthe_graciliflora_KF560495 ATGAC-TCTCGGCAATGGATATCTC-GGCTCTCGCATCGATGAAGAGCGCAGCGAAATGCGATACGTGGTGC-GAATTGCAGAATCCCGCGAACC-ATCGAGTCTTTGAACGCAAGTTGCGCCCGAGGCCAAT-CGGCCAAGGGCACGTCTGCCTG---GGCGTCAA-GCGTTGCATCGCTCT

Calanthe_graciliflora_PK12206 ATGAC-TCTCGGCAATGGATATCTC-GGCTCTCGCATCGATGAAGAGCGCAGCGAAATGCGATACGTGGTGC-GAATTGCAGAATCCCGCGAACC-ATCGAGTCTTTGAACGCAAGTTGCGCCCGAGGCCAAT-CGGCCAAGGGCACGTCTGCCTG---GGCGTCAA-GCGTTGCATCGCTCT

Calanthe_graciliflora_PK12207 ATGAC-TCTCGGCAATGGATATCTC-GGCTCTCGCATCGATGAAGAGCGCAGCGAAATGCGATACGTGGTGC-GAATTGCAGAATCCCGCGAACC-ATCGAGTCTTTGAACGCAAGTTGCGCCCGAGGCCAAT-CGGCCAAGGGCACGTCTGCCTG---GGCGTCAA-GCGTTGCATCGCTCT

Calanthe_graciliflora_SG1225 ATGAC-TCTCGGCAATGGATATCTC-GGCTCTCGCATCGATGAAGAGCGCAGCGAAATGCGATACGTGGTGC-GAATTGCAGAATCCCGCGAACC-ATCGAGTCTTTGAACGCAAGTTGCGCCCGAGGCCAAT-CGGCCAAGGGCACGTCTGCCTG---GGCGTCAA-GCGTTGCATCGCTCT

Calanthe_masuca_KFBG11 ATGAC-TCTCGGCAATGGATATCTC-GGCTCTCGCATCGATGAAGAGCGCAGCGAAATGCGATACGTGGTGC-GAATTGCAGAATCCCGCGAACC-ATCGAGTCTTTGAACGCAAGTTGCGCCCGAGGCCAAT-CGGCCAAGGGCACGTCTGCCTG---GGCGTCAA-GCGTTGCATCGCTCT

Calanthe_masuca_PK12110 ATGAC-TCTCGGCAATGGATATCTC-GGCTCTCGCATCGATGAAGAGCGCAGCGAAATGCGATACGTGGTGC-GAATTGCAGAATCCCGCGAACC-ATCGAGTCTTTGAACGCAAGTTGCGCCCGAGGCCAAT-CGGCCAAGGGCACGTCTGCCTG---GGCGTCAA-GCGTTGCATCGCTCT

Calanthe_masuca_SG026 ATGAC-TCTCGGCAATGGATATCTC-GGCTCTCGCATCGATGAAGAGCGCAGCGAAATGCGATACGTGGTGC-GAATTGCAGAATCCCGCGAACC-ATCGAGTCTTTGAACGCAAGTTGCGCCCGAGGCCAAT-CGGCCAAGGGCACGTCTGCCTG---GGCGTCAA-GCGTTGCATCGCTCT

Calanthe_masuca_SG1360 ATGAC-TCTCGGCAATGGATATCTC-GGCTCTCGCATCGATGAAGAGCGCAGCGAAATGCGATACGTGGTGC-GAATTGCAGAATCCCGCGAACC-ATCGAGTCTTTGAACGCAAGTTGCGCCCGAGGCCAAT-CGGCCAAGGGCACGTCTGCCTG---GGCGTCAA-GCGTTGCATCGCTCT

Calanthe_masuca_SG1361 ATGAC-TCTCGGCAATGGATATCTC-GGCTCTCGCATCGATGAAGAGCGCAGCGAAATGCGATACGTGGTGC-GAATTGCAGAATCCCGCGAACC-ATCGAGTCTTTGAACGCAAGTTGCGCCCGAGGCCAAT-CGGCCAAGGGCACGTCTGCCTG---GGCGTCAA-GCGTTGCATCGCTCT

Calanthe_speciosa_KFBG136 ACGAC-TCTCGGCAATGGATATCTC-GGCTCTCGCATCGATGAAGAGCGCAGCGAAATGCGATACGTGGTGC-GAATTGCAGAATCCCGCGAACC-ATCGAGTCTTTGAACGCAAGTTGCGCCCGAGGCCAAC-CGGCCAAGGGCACGTCTGCCTG---GGCGTCAA-GCGTTGCATCGCTCT

Calanthe_speciosa_KY951546 ACGAC-TCTCGGCAATGGATATCTC-GGCTCTCGCATCGATGAAGAGCGCAGCGAAATGCGATACGTGGTGC-GAATTGCAGAATCCCGCGAACC-ATCGAGTCTTTGAACGCAAGTTGCGCCCGAGGCCAAC-CGGCCAAGGGCACGTCTGCCTG---GGCGTCAA-GCGTTGCATCGCTCT

Calanthe_speciosa_PK12168 ACGAC-TCTCGGCAATGGATATCTC-GGCTCTCGCATCGATGAAGAGCGCAGCGAAATGCGATACGTGGTGC-GAATTGCAGAATCCCGCGAACC-ATCGAGTCTTTGAACGCAAGTTGCGCCCGAGGCCAAC-CGGCCAAGGGCACGTCTGCCTG---GGCGTCAA-GCGTTGCATCGCTCT

Calanthe_speciosa_PK12169 ACGAC-TCTCGGCAATGGATATCTC-GGCTCTCGCATCGATGAAGAGCGCAGCGAAATGCGATACGTGGTGC-GAATTGCAGAATCCCGCGAACC-ATCGAGTCTTTGAACGCAAGTTGCGCCCGAGGCCAAC-CGGCCAAGGGCACGTCTGCCTG---GGCGTCAA-GCGTTGCATCGCTCT

Calanthe_speciosa_SG1313 ACGAC-TCTCGGCAATGGATATCTC-GGCTCTCGCATCGATGAAGAGCGCAGCGAAATGCGATACGTGGTGC-GAATTGCAGAATCCCGCGAACC-ATCGAGTCTTTGAACGCAAGTTGCGCCCGAGGCCAAC-CGGCCAAGGGCACGTCTGCCTG---GGCGTCAA-GCGTTGCATCGCTCT

Calanthe_speciosa_SG1368 ACGAC-TCTCGGCAATGGATATCTC-GGCTCTCGCATCGATGAAGAGCGCAGCGAAATGCGATACGTGGTGC-GAATTGCAGAATCCCGCGAACC-ATCGAGTCTTTGAACGCAAGTTGCGCCCGAGGCCAAC-CGGCCAAGGGCACGTCTGCCTG---GGCGTCAA-GCGTTGCATCGCTCT

Calanthe_triplicata_AY882614 ATGAC-TCTCGGCAATGGATATCTC-GGCTCTCGCATCGATGAAGAGCGCAGCGAAATGCGATACGTGGTGC-GAATTGCAGAATCCCGCGAACC-ATCGAGTCTTTGAACGCAAGTTGCGCCCGAGGCCAAT-CGGCCAAGGGCACGTCTGCCTG---GGCGTCAA-GCGTTGCATCGCTCT

Calanthe_triplicata_KF560480 ATGAC-TCTCGGCAATGGATATCTC-GGCTCTCGCATCGATGAAGAGCGCAGCGAAATGCGATACGTGGTGC-GAATTGCAGAATCCCGCGAACC-ATCGAGTCTTTGAACGCAAGTTGCGCCCGAGGCCAAT-CGGCCAAGGGCACGTCTGCCTG---GGCGTCAA-GCGTTGCATCGCTCT

Calanthe_triplicata_KF560491 ATGAC-TCTCGGCAATGGATATCTC-GGCTCTCGCATCGATGAAGAGCGCAGCGAAATGCGATACGTGGTGC-GAATTGCAGAATCCCGCGAACC-ATCGAGTCTTTGAACGCAAGTTGCGCCCGAGGCCAAT-CGGCCAAGGGCACGTCTGCCTG---GGCGTCAA-GCGTTGCATCGCTCT

Calanthe_triplicata_KFBG601 ATGAC-TCTCGGCAATGGATATCTC-GGCTCTCGCATCGATGAAGAGCGCAGCGAAATGCGATACGTGGTGC-GAATTGCAGAATCCCGCGAACC-ATCGAGTCTTTGAACGCAAGTTGCGCCCGAGGCCAAT-CGGCCAAGGGCACGTCTGCCTG---GGCGTCAA-GCGTTGCATCGCTCT

Calanthe_triplicata_KM025154 ATGAC-TCTCGGCAATGGATATCTC-GGCTCTCGCATCGATGAAGAGCGCAGCGAAATGCGATACGTGGTGC-GAATTGCAGAATCCCGCGAACC-ATCGAGTCTTTGAACGCAAGTTGCGCCCGAGGCCAAT-CGGCCAAGGGCACGTCTGCCTG---GGCGTCAA-GCGTTGCATCGCTCT

Calanthe_triplicata_KY966491 ATGAC-TCTCGGCAATGGATATCTC-GGCTCTCGCATCGATGAAGAGCGCAGCGAAATGCGATACGTGRTGC-GAATTGCAGAATCCCGCGAACC-ATCGAGTCTTTGAACGCAAGTTGCGCCCGAGGCCAAT-CGGCCAAGGGCACGTCTGCCTG---GGCGTCAA-GCGTTGCATCGCTCT

Calanthe_triplicata_SG1311 ATGAC-TCTCGGCAATGGATATCTC-GGCTCTCGCATCGATGAAGAGCGCAGCGAAATGCGATACGTGGTGC-GAATTGCAGAATCCCGCGAACC-ATCGAGTCTTTGAACGCAAGTTGCGCCCGAGGCCAAT-CGGCCAAGGGCACGTCTGCCTG---GGCGTCAA-GCGTTGCATCGCTCT

Cephalantheropsis_obcordata_KF560490 ACGAC-TCTCGGCAATGGATATCTC-GGCTCTCGCATCGATGAAGAGCGCAGCGAAATGCGATACGTGGTGC-GAATTGCAGAATCCCGCGAACC-ATCGAGTCTTTGAACGCAAGTTGCGCCCGAGGCCAAC-CGGCCAAGGGCACGTCTGCCTG---GGCGTCAA-GCGTTGCATCGCTCT

Cephalantheropsis_obcordata_KFBG2520 ACGAC-TCTCGGCAATGGATATCTC-GGCTCTCGCATCGATGAAGAGCGCAGCGAAATGCGATACGTGGTGC-GAATTGCAGAATCCCGCGAACC-ATCGAGTCTTTGAACGCAAGTTGCGCCCGAGGCCAAC-CGGCCAAGGGCACGTCTGCCTG---GGCGTCAA-GCGTTGCATCGCTCT

Cephalantheropsis_obcordata_KY966494 ACGAC-TCTCGGCAATGGATATCTC-GGCTCTCGCATCGATGAAGAGCGCAGCGAAATGCGATACGTGGTGC-GAATTGCAGAATCCCGCGAACC-ATCGAGTCTTTGAACGCAAGTTGCGCCCGAGGCCAAC-CGGCCAAGGGCACGTCTGCCTG---GGCGTCAA-GCGTTGCATCGCTCT

Cephalantheropsis_obcordata_KY966495 ACGAC-TCTCGGCAATGGATATCTC-GGCTCTCGCATCGATGAAGAGCGCAGCGAAATGCGATACGTGGTGC-GAATTGCAGAATCCCGCGAACC-ATCGAGTCTTTGAACGCAAGTTGCGCCCGAGGCCAAC-CGGCCAAGGGCACGTCTGCCTG---GGCGTCAA-GCGTTGCATCGCTCT

Cephalantheropsis_obcordata_PK12079 ACGAC-TCTCGGCAATGGATATCTC-GGCTCTCGCATCGATGAAGAGCGCAGCGAAATGCGATACGTGGTGC-GAATTGCAGAATCCCGCGAACC-ATCGAGTCTTTGAACGCAAGTTGCGCCCGAGGCCAAC-CGGCCAAGGGCACGTCTGCCTG---GGCGTCAA-GCGTTGCATCGCTCT

Cephalantheropsis_obcordata_PK12080 ACGAC-TCTCGGCAATGGATATCTC-GGCTCTCGCATCGATGAAGAGCGCAGCGAAATGCGATACGTGGTGC-GAATTGCAGAATCCCGCGAACC-ATCGAGTCTTTGAACGCAAGTTGCGCCCGAGGCCAAC-CGGCCAAGGGCACGTCTGCCTG---GGCGTCAA-GCGTTGCATCGCTCT

Cephalantheropsis_obcordata_PK12081 ACGAC-TCTCGGCAATGGATATCTC-GGCTCTCGCATCGATGAAGAGCGCAGCGAAATGCGATACGTGGTGC-GAATTGCAGAATCCCGCGAACC-ATCGAGTCTTTGAACGCAAGTTGCGCCCGAGGCCAAC-CGGCCAAGGGCACGTCTGCCTG---GGCGTCAA-GCGTTGCATCGCTCT

Cephalantheropsis_obcordata_SG1208 ACGAC-TCTCGGCAATGGATATCTC-GGCTCTCGCATCGATGAAGAGCGCAGCGAAATGCGATACGTGGTGC-GAATTGCAGAATCCCGCGAACC-ATCGAGTCTTTGAACGCAAGTTGCGCCCGAGGCCAAC-CGGCCAAGGGCACGTCTGCCTG---GGCGTCAA-GCGTTGCATCGCTCT

Cephalantheropsis_obcordata__KFBG140 ACGAC-TCTCGGCAATGGATATCTC-GGCTCTCGCATCGATGAAGAGCGCAGCGAAATGCGATACGTGGTGC-GAATTGCAGAATCCCGCGAACC-ATCGAGTCTTTGAACGCAAGTTGCGCCCGAGGCCAAC-CGGCCAAGGGCACGTCTGCCTG---GGCGTCAA-GCGTTGCATCGCTCT

Cephalantheropsis_obcordata__SG1209 ACGAC-TCTCGGCAATGGATATCTC-GGCTCTCGCATCGATGAAGAGCGCAGCGAAATGCGATACGTGGTGC-GAATTGCAGAATCCCGCGAACC-ATCGAGTCTTTGAACGCAAGTTGCGCCCGAGGCCAAC-CGGCCAAGGGCACGTCTGCCTG---GGCGTCAA-GCGTTGCATCGCTCT

Cheirostylis_clibborndyeri_KY966496 ATGAC-TCTCGGCAATGGATATCTT-GGCTCTTGCATCGATGAAGAGCGCAGCGAAATGCGATACGTGGTGT-GAATTGCAGAATCCCGTGAACC-ATCAAATATTTGAACGCAAGTTGCGCCTGAGGCCAAT-TGGCTAAGGGCACGTCCGCCTG---GGCGCCAA-GCATTATATCGCTTC

Cheirostylis_clibborndyeri_PK12096 ATGAC-TCTCGGCAATGGATATCTT-GGCTCTTGCATCGATGAAGAGCGCAGCGAAATGCGATACGTGGTGT-GAATTGCAGAATCCCGTGAACC-ATCAAATATTTGAACGCAAGTTGCGCCCGAGGCCAAT-TGGCTAAGGGCACGTCCGCCTG---GGCGTCAA-GCATTATATCGCTTC

Cheirostylis_clibborndyeri_SG1349 ATGAC-TCTCGGCAATGGATATCTT-GGCTCTTGCATCGATGAAGAGCGCAGCGAAATGCGATACGTGGTGT-GAATTGCAGAATCCCGTGAACC-ATCAAATATTTGAACGCAAGTTGCGCCCGAGGCCAAT-TGGCTAAGGGCACGTCCGCCTG---GGCGTCAA-GCATTATATCGCTTC

Cheirostylis_jamesleungii_PK12205 ATGAC-TCTCGGCAATGGATATCTT-GGCTCTTGCATCGATGAAGAGCGCAGCGAAATGCGATACGTGGTGT-GAATTGCAGAATCCCGTGAACC-ATCAAATATTTGAACGCAAGTTGCGCCTGAGGCCAAT-TGGCTAAGGGCACGTCCGCCTG---GGCGTCAA-GCATTTTATCGCTTC

Cheirostylis_monteiroi_PK12093 ATGAC-TCTCGGCAATGGATATCTT-GGCTCTTGCATCGATGAAGAGCGCAGCGAAATGCGATACGTGGTGT-GAATTGCAGAATCCCGTGAACC-ATCAAATATTTGAACGCAAGTTGCGCCTGAGGCCAAT-TGGCTAAGGGCACGTCCGCCTG---GGCGTCAA-GCATTATATCGCTTC

Cheirostylis_monteiroi_PK12094 ATGAC-TCTCGGCAATGGATATCTT-GGCTCTTGCATCGATGAAGAGCGCAGCGAAATGCGATACGTGGTGT-GAATTGCAGAATCCCGTGAACC-ATCAAATATTTGAACGCAAGTTGCGCCTGAGGCCAAT-TGGCTAAGGGCACGTCCGCCTG---GGCGTCAA-GCATTATATCGCTTC

Cheirostylis_monteiroi_SG1344 ATGAC-TCTCGGCAATGGATATCTT-GGCTCTTGCATCGATGAAGAGCGCAGCGAAATGCGATACGTGGTGT-GAATTGCAGAATCCCGTGAACC-ATCAAATATTTGAACGCAAGTTGCGCCCGAGGCCAAT-TGGCTAAGGGCACGTCCGCCTG---GGCGTCAA-GCATTATATCGCTTC

Cheirostylis_pusilla_HK43263 ATGAC-TCTCGGCAATGGATATCTT-GGCTCTTGCATCGATGAAGAGCGCAGCGAAATGCGATACGTGGTGT-GAATTGCAGAATCCCGTGAACC-ATCAAATATTTGAACGCAAGTTGCGCCCGAGGCCAAT-TGGCTAAGGGCATGTCCGCCTG---GGCGTCAA-GCTTTATATCGCTTC

Cheirostylis_yunnanensis_KT343978 ATGAC-TCTCGGCAATGGATATCTT-GGCTCTTGCATCGATGAAGAGCGCAGCGAAATGCGATACGTGGTGT-GAATTGCAGAATCCCGTGAACC-ATCAAATATTTGAACGCAAGTTGCGCCCGAGGCCAAT-TGGCTAAGGGCACGTCCGCCTG---GGCGTCAA-GC-TTATATCGCT--

Cheirostylis_yunnanensis_PK12097 ATGAC-TCTCGGCAATGGATATCTT-GGCTCTTGCATCGATGAAGAGCGCAGCGAAATGCGATACGTGGTGT-GAATTGCAGAATCCCGTGAACC-ATCAAATATTTGAACGCAAGTTGCGCCCGAGGCCAAT-TGGCTAAGGGCACGTCCGCCTG---GGCGTCAA-GCATTATATCGCTTC

Cheirostylis_yunnanensis_SG1227 ATGAC-TCTCGGCAATGGATATCTT-GGCTCTTGCATCGATGAAGAGCGCAGCGAAATGCGATACGTGGTGT-GAATTGCAGAATCCCGTGAACC-ATCAAATATTTGAACGCAAGTTGCGCCCGAGGCCAAT-TGGCTAAGGGCACGTCCGCCTG---GGCGTCAA-GCATTATATCGCTTC

Cheirostylis_yunnanensis_SG1228 ATGAC-TCTCGGCAATGGATATCTT-GGCTCTTGCATCGACGAAGAGCGCAGCGAAATGCGATACGTGGTGT-GAATTGCAGAATCCCGTGAACC-ATAAAATATTTGAACGCAAGTTGCGCCCGAGGCCAAT-TGGCTAAGGGCACGTCCGCCTG---GGCGTCAA-GCATTATATCGCTTC

Cheirostylis_yunnanensis_SG1229 ATGAC-TCTCGGCAATGGATATCTT-GGCTCTTGCATCGATGAAGAGCGCAGCGAAATGCGATACGTGGTGT-GAATTGCAGAATCCCGTGAACC-ATCAAATATTTGAACGCAAGTTGCGCCCGAGGCCAAT-TGGCTAAGGGCACGTCCGCCTG---GGCGTCAA-GCATTATATCGCTTC

Chrysoglossum_assamicum_SG1622 ACGAC-TCTCGGCAATGGATATCTC-GGCTCTCGCATCGATGAAGAGCGCAGCGAAATGCGATACGTGGTGC-GAATTGCAGAATCCCGCGAACC-ATCGAGTCTTTGAACGCAAGTTGCGCCCGAGGCCAAC-CGGCCAAGGGCACGTCTGCCTG---GGCGTCAA-GCGTTGCGTCGCTCC

Chrysoglossum_assamicum_SG1623 ACGAC-TCTCGGCAATGGATATCTC-GGCTCTCGCATCGATGAAGAGCGCAGCGAAATGCGATACGTGGTGC-GAATTGCAGAATCCCGCGAACC-ATCGAGTCTTTGAACGCAAGTTGCGCCCGAGGCCAAC-CGGCCAAGGGCACGTCTGCCTG---GGCGTCAA-GCGTTGCGTCGCTCC

Cleisostoma_paniculatum_KFBG516 ACGAC-TCTCGACAATGGATATCTC-GGCTCTCGCATCGATGAAGAGCGCAGCGAAATGCGATACGTGGTGC-GAATTGCAGAATCCCGCGAACC-ATCGAGTCTTTGAACGCAAGTTGCGCCCGAGGCCAAT-CGGTCGAGGGCACGTCCGCCTG---GGCGTCAA-GCGTTGCGCCGCTCC

Cleisostoma_paniculatum_KJ733401 ACGAC-TCTCGACAATGGATATCTC-GGCTCTCGCATCGATGAAGAGCGCAGCGAAATGCGATACGTGGTGC-GAATTGCAGAATCCCGCGAACC-ATCGAGTCTTTGAACGCAAGTTGCGCCCGAGGCCAAT-CGGTCGAGGGCACGTCCGCCTG---GGCGTCAA-GCGTTGCGCCGCTCC

Cleisostoma_paniculatum_KT223752 ACGAC-TCTCGACAATGGATATCTC-GGCTCTCGCATCGATGAAGAGCGCAGCGAAATGCGATACGTGGTGC-GAATTGCAGAATCCCGCGAACC-ATCGAGTCTTTGAACGCAAGTTGCGCCCGAGGCCAAT-CGGTCGAGGGCACGTCCGCCTG---GGCGTCAA-GCGTCGCGCCGCTCC

Cleisostoma_rostratum_KFBG789 ACGAC-TCTCGACAATGGATATCTC-GGCTCTCGCATCGATGAAGAGCGCAGCGAAATGCGATACGTGGTGC-GAATTGCAGAATCCCGCGAACC-ATCGAGTCTTTGAACGCAAGTTGCGCCCGAGGCCAAT-CGGTCGAGGGCACGTCCGCCTG---GGCGTCAA-GCGTCGCGCCGCTCC

Cleisostoma_rostratum_KJ733404 ACGAC-TCTCGACAATGGATATCTC-GGCTCTCGCATCGATGAAGAGCGCAGCGAAATGCGATACGTGGTGC-GAATTGCAGAATCCCGCGAACC-ATCGAGTCTTTGAACGCAAGTTGCGCCCGAGGCCAAT-CGGTCGAGGGCACGTCCGCCTG---GGCGTCAA-GCGTCGCGCCGCTCC

Cleisostoma_rostratum_KY966502 ACGAC-TCTCGACAATGGATATCTC-GGCTCTCGCATCGATGAAGAGCGCAGCGAAATGCGATACGTGGTGC-GAATTGCAGAATCCCGCGAACC-ATCGAGTCTTTGAACGCAAGTTGCGCCCGAGGCCAAT-CGGTCGAGGGCACGTCCGCCTG---GGCGTCAA-GCGTCGCGCCGCTCC

Cleisostoma_rostratum_PK12089 ACGAC-TCTCGACAATGGATATCTC-GGCTCTCGCATCGATGAAGAGCGCAGCGAAATGCGATACGTGGTGC-GAATTGCAGAATCCCGCGAACC-ATCGAGTCTTTGAACGCAAGTTGCGCCCGAGGCCAAT-CGGTCGAGGGCACGTCCGCCTG---GGCGTCAA-GCGTCGCGCCGCTCC

Cleisostoma_rostratum_PK12101 ACGAC-TCTCGACAATGGATATCTC-GGCTCTCGCATCGATGAAGAGCGCAGCGAAATGCGATACGTGGTGC-GAATTGCAGAATCCCGCGAACC-ATCGAGTCTTTGAACGCAAGTTGCGCCCGAGGCCAAT-CGGTCGAGGGCACGTCCGCCTG---GGCGTCAA-GCGTCGCGCCGCTCC

Cleisostoma_rostratum_PK12158 ACGAC-TCTCGACAATGGATATCTC-GGCTCTCGCATCGATGAAGAGCGCAGCGAAATGCGATACGTGGTGC-GAATTGCAGAATCCCGCGAACC-ATCGAGTCTTTGAACGCAAGTTGCGCCCGAGGCCAAT-CGGTCGAGGGCACGTCCGCCTG---GGCGTCAA-GCGTCGCGCCGCTCC

Cleisostoma_rostratum_SG1200 ACGAC-TCTCGACAATGGATATCTC-GGCTCTCGCATCGATGAAGAGCGCAGCGAAATGCGATACGTGGTGC-GAATTGCAGAATCCCGCGAACC-ATCGAGTCTTTGAACGCAAGTTGCGCCCGAGGCCAAT-CGGTCGAGGGCACGTCCGCCTG---GGCGTCAA-GCGTCGCGCCGCTCC

Cleisostoma_rostratum_SG1301 ACGAC-TCTCGACAATGGATATCTC-GGCTCTCGCATCGATGAAGAGCGCAGCGAAATGCGATACGTGGTGC-GAATTGCAGAATCCCGCGAACC-ATCGAGTCTTTGAACGCAAGTTGCGCCCGAGGCCAAT-CGGTCGAGGGCACGTCCGCCTG---GGCGTCAA-GCGTCGCGCCGCTCC

Cleisostoma_simondii_KJ733405 ACGAC-TCTCGACAATGGATATCTC-GGCTCTCGCATCGATGAAGAGCGCAGCGAAATGCGATACGTGGTGC-GAATTGCAGAATCCCGCGAACC-ATCGAGTCTTTGAACGCAAGTTGCGCCCGAGGCCAAT-CGGTCGAGGGCACGTCCGCCTG---GGCGTCAA-GCGTTGCGCCGCTCC

Cleisostoma_simondii_KY966503 ACGAC-TCTCGACAATGGATATCTC-GGCTCTCGCATCGATGAAGAGCGCAGCGAAATGCGATACGTGGTGC-GAATTGCAGAATCCCGCGAACC-ATCGAGTCTTTGAACGCAAGTTGCGCCCGAGGCCAAT-CGGTCGAGGGCACGTCCGCCTG---GGCGTCAA-GCGTTGCGCCGCTCC

Cleisostoma_simondii_KY966504 ACGAC-TCTCGACAATGGATATCTC-GGCTCTCGCATCGATGAAGAGCGCAGCGAAATGCGATACGTGGTGC-GAATTGCAGAATCCCGCGAACC-ATCGAGTCTTTGAACGCAAGTTGCGCCCGAGGCCAAT-CGGTCGAGGGCACGTCCGCCTG---GGCGTCAA-GCGTTGCGCCGCTCC

Cleisostoma_simondii_MG822849 ACGAC-TCTCGACAATGGATATCTC-GGCTCTCGCATCGATGAAGAGCGCAGCGAAATGCGATACGTGGTGC-GAATTGCAGAATCCCGCGAACC-ATCGAGTCTTTGAACGCAAGTTGCGCCCGAGGCCAAT-CGGTCGAGGGCACGTCCGCCTG---GGCGTCAA-GCGTTGCGCCGCTCC

Cleisostoma_simondii_PK12176 ACGAC-TCTCGACAATGGATATCTC-GGCTCTCGCATCGATGAAGAGCGCAGCGAAATGCGATACGTGGTGC-GAATTGCAGAATCCCGCGAACC-ATCGAGTCTTTGAACGCAAGTTGCGCCCGAGGCCAAT-CGGTCGAGGGCACGTCCGCCTG---GGCGTCAA-GCGTTGCGCCGCTCC

Cleisostoma_simondii_SG1314 ACGAC-TCTCGACAATGGATATCTC-GGCTCTCGCATCGATGAAGAGCGCAGCGAAATGCGATACGTGGTGC-GAATTGCAGAATCCCGCGAACC-ATCGAGTCTTTGAACGCAAGTTGCGCCCGAGGCCAAT-CGGTCGAGGGCACGTCCGCCTG---GGCGTCAA-GCGTTGCGCCGCTCC

Cleisostoma_simondii_SG1327 ACGAC-TCTCGACAATGGATATCTC-GGCTCTCGCATCGATGAAGAGCGCAGCGAAATGCGATACGTGGTGC-GAATTGCAGAATCCCGCGAACC-ATCGAGTCTTTGAACGCAAGTTGCGCCCGAGGCCAAT-CGGTCGAGGGCACGTCCGCCTG---GGCGTCAA-GCGTTGCGCCGCTCC

Cleisostoma_simondii_SG1328 ACGAC-TCTCGACAATGGATATCTC-GGCTCTCGCATCGATGAAGAGCGCAGCGAAATGCGATACGTGGTGC-GAATTGCAGAATCCCGCGAACC-ATCGAGTCTTTGAACGCAAGTTGCGCCCGAGGCCAAT-CGGTCGAGGGCACGTCCGCCTG---GGCGTCAA-GCGTTGCGCCGCTCC

Cleisostoma_simondii_SG1329 ACGAC-TCTCGACAATGGATATCTC-GGCTCTCGCATCGATGAAGAGCGCAGCGAAATGCGATACGTGGTGC-GAATTGCAGAATCCCGCGAACC-ATCGAGTCTTTGAACGCAAGTTGCGCCCGAGGCCAAT-CGGTCGAGGGCACGTCCGCCTG---GGCGTCAA-GCGTTGCGCCGCTCC

Cleisostoma_simondii_SG1330 ACGAC-TCTCGACAATGGATATCTC-GGCTCTCGCATCGATGAAGAGCGCAGCGAAATGCGATACGTGGTGC-GAATTGCAGAATCCCGCGAACC-ATCGAGTCTTTGAACGCAAGTTGCGCCCGAGGCCAAT-CGGTCGAGGGCACGTCCGCCTG---GGCGTCAA-GCGTTGCGCCGCTCC

Cleisostoma_simondii_var_guangdongense_KFBG2212 ACGAC-TCTCGACAATGGATATCTC-GGCTCTCGCATCGATGAAGAGCGCAGCGAAATGCGATACGTGGTGC-GAATTGCAGAATCCCGCGAACC-ATCGAGTCTTTGAACGCAAGTTGCGCCCGAGGCCAAT-CGGTCGAGGGCACGTCCGCCTG---GGCGTCAA-GCGTTGCGCCGCTCC

Cleisostoma_simondii_var_guangdongense_KJ733406 ACGAC-TCTCGACAATGGATATCTC-GGCTCTCGCATCGATGAAGAGCGCAGCGAAATGCGATACGTGGTGC-GAATTGCAGAATCCCGCGAACC-ATCGAGTCTTTGAACGCAAGTTGCGCCCGAGGCCAAT-CGGTCGAGGGCACGTCCGCCTG---GGCGTCAA-GCGTTGCGCCGCTCC

Cleisostoma_williamsonii_KJ733409 ACGAC-TCTCGACAATGGATATCTC-GGCTCTCGCATCGATGAAGAGCGCAGCGAAATGCGATACGTGGTGC-GAATTGCAGAATCCCGCGAACC-ATCGAGTCTTTGAACGCAAGTTGCGCCCGAGGCCAAT-CGGTCGAGGGCACGTCCGCCTG---GGCGTCAA-GCGTTGCGCCGCTCC

Coelogyne_cantonensis_KY966649 ACGAC-TCTCGGCAATGGATATCTC-GGCTCTCGCATCGATGAAGAGCGCAGCGAAATGCGATACGTGGTGC-GAATTGCAGAATCCCGCGAACC-ATCGAGTCTTTGAACGCAAGTTGCGCCCGAGGCCAAC-CGGCCAAGGGCACGTCTGCCTG---GGCGTCAA-GCGTTGCGTCGCTCC

Coelogyne_cantonensis_SG1239 ACGAC-TCTCGGCAATGGATATCTC-GGCTCTCGCATCGATGAAGAGCGCAGCGAAATGCGATACGTGGTGC-GAATTGCAGAATCCCGCGAACC-ATCGAGTCTTTGAACGCAAGTTGCGCCCGAGGCCAAC-CGGCCAAGGGCACGTCTGCCTG---GGCGTCAA-GCGTTGCGTCGCTCC

Coelogyne_cantonensis_SG1339 ACGAC-TCTCGGCAATGGATATCTC-GGCTCTCGCATCGATGAAGAGCGCAGCGAAATGCGATACGTGGTGC-GAATTGCAGAATCCCGCGAACC-ATCGAGTCTTTGAACGCAAGTTGCGCCCGAGGCCAAC-CGGCCAAGGGCACGTCTGCCTG---GGCGTCAA-GCGTTGCGTCGCTCC

Coelogyne_cantonensis_SG1388 ACGAC-TCTCGGCAATGGATATCTC-GGCTCTCGCATCGATGAAGAGCGCAGCGAAATGCGATACGTGGTGC-GAATTGCAGAATCCCGCGAACC-ATCGAGTCTTTGAACGCAAGTTGCGCCCGAGGCCAAC-CGGCCAAGGGCACGTCTGCCTG---GGCGTCAA-GCGTTGCGTCGCTCC

Coelogyne_chinensis_EU592035 ACGAC-TCTCGGCAATGGATATCTC-GGCTCTCGCATCGATGAAGAGCGCAGCGAAATGCGATACGTGGTGC-GAATTGCAGAATCCCGCGAACC-ATCGAGTCTTTGAACGCAAGTTGCGCCCGAGGCCAAC-CGGCCAAGGGCACGTCTGCCTG---GGCGTCAA-GCGTTGCGTCGCTCC

Coelogyne_chinensis_KY966650 ACGAC-TCTCGGCAATGGATATCTC-GGCTCTCGCATCGATGAAGAGCGCAGCGAAATGCGATACGTGGTGC-GAATTGCAGAATCCCGCGAACC-ATCGAGTCTTTGAACGCAAGTTGCGCCCGAGGCCAAC-CGGCCAAGGGCACGTCTGCCTG---GGCGTCAA-GCGTTGCGTCGCTCC

Coelogyne_chinensis_KY966651 ACGAC-TCTCGGCAATGGATATCTC-GGCTCTCGCATCGATGAAGAGCGCAGCGAAATGCGATACGTGGTGC-GAATTGCAGAATCCCGCGAACC-ATCGAGTCTTTGAACGCAAGTTGCGCCCGAGGCCAAC-CGGCCAAGGGCACGTCTGCCTG---GGCGTCAA-GCGTTGCGTCGCTCC

Coelogyne_chinensis_PK12114 ACGAC-TCTCGGCAATGGATATCTC-GGCTCTCGCATCGATGAAGAGCGCAGCGAAATGCGATACGTGGTGC-GAATTGCAGAATCCCGCGAACC-ATCGAGTCTTTGAACGCAAGTTGCGCCCGAGGCCAAC-CGGCCAAGGGCACGTCTGCCTG---GGCGTCAA-GCGTTGCGTCGCTCC

Coelogyne_chinensis_PK12115 ACGAC-TCTCGGCAATGGATATCTC-GGCTCTCGCATCGATGAAGAGCGCAGCGAAATGCGATACGTGGTGC-GAATTGCAGAATCCCGCGAACC-ATCGAGTCTTTGAACGCAAGTTGCGCCCGAGGCCAAC-CGGCCAAGGGCACGTCTGCCTG---GGCGTCAA-GCGTTGCGTCGCTCC

Coelogyne_chinensis_SG1232 ACGAC-TCTCGGCAATGGATATCTC-GGCTCTCGCATCGATGAAGAGCGCAGCGAAATGCGATACGTGGTGC-GAATTGCAGAATCCCGCGAACC-ATCGAGTCTTTGAACGCAAGTTGCGCCCGAGGCCAAC-CGGCCAAGGGCACGTCTGCCTG---GGCGTCAA-GCGTTGCGTCGCTCC

Coelogyne_chinensis_SG1251 ACGAC-TCTCGGCAATGGATATCTC-GGCTCTCGCATCGATGAAGAGCGCAGCGAAATGCGATACGTGGTGC-GAATTGCAGAATCCCGCGAACC-ATCGAGTCTTTGAACGCAAGTTGCGCCCGAGGCCAAC-CGGCCAAGGGCACGTCTGCCTG---GGCGTCAA-GCGTTGCGTCGCTCC

Coelogyne_fimbriata_AF302745 ACGAC-TCTCGGCAATGGATATCTC-GGCTCTCGCATCGATGAAGAGCGCAGCGAAATGCGATACGTGGTGC-GAATTGCAGAATCCCGCGAACC-ATCGAGTCTTTGAACGCAAGTTGCGCCCGAGGCCAAC-CGGCCAAGGGCACGTCTGCCTG---GGCGTCAA-GCGTTGCGTCGCTCC

Coelogyne_fimbriata_EU441205 ACGAC-TCTCGGCAATGGATATCTC-GGCTCTCGCATCGATGAAGAGCGCAGCGAAATGCGATACGTGGTGC-GAATTGCAGAATCCCGCGAACC-ATCGAGTCTTTGAACGCAAGTTGCGCCCGAGGCCAAC-CGGCCAAGGGCACGTCTGCCTG---GGCGTCAA-GCGTTGCGTCGCTCC

Coelogyne_fimbriata_JF422074 ACGAC-TCTCGGCAATGGATATCTC-GGCTCTCGCATCGATGAAGAGCGCAGCGAAATGCGATACGTGGTGC-GAATTGCAGAATCCCGCGAACC-ATCGAGTCTTTGAACGCAAGTTGCGCCCGAGGCCAAC-CGGCCAAGGGCACGTCTGCCTG---GGCGTCAA-GCGTTGCGTCGCTCC

Coelogyne_fimbriata_KFBG411 ACGAC-TCTCGGCAATGGATATCTC-GGCTCTCGCATCGATGAAGAGCGCAGCGAAATGCGATACGTGGTGC-GAATTGCAGAATCCCGCGAACC-ATCGAGTCTTTGAACGCAAGTTGCGCCCGAGGCCAAC-CGGCCAAGGGCACGTCTGCCTG---GGCGTCAA-GCGTTGCGTCGCTCC

Coelogyne_fimbriata_KFBG523 ACGAC-TCTCGGCAATGGATATCTC-GGCTCTCGCATCGATGAAGAGCGCAGCGAAATGCGATACGTGGTGC-GAATTGCAGAATCCCGCGAACC-ATCGAGTCTTTGAACGCAAGTTGCGCCCGAGGCCAAC-CGGCCAAGGGCACGTCTGCCTG---GGCGTCAA-GCGTTGCGTCGCTCC

Coelogyne_fimbriata_KR857330 ACGAC-TCTCGGCAATGGATATCTC-GGCTCTCGCATCGATGAAGAGCGCAGCGAAATGCGATACGTGGTGC-GAATTGCAGAATCCCGCGAACC-ATCGAGTCTTTGAACGCAAGTTGCGCCCGAGGCCAAC-CGGCCAAGGGCACGTCTGCCTG---GGCGTCAA-GCGTTGCGTCGCTCC

Coelogyne_fimbriata_KY966506 ACGAC-TCTCGGCAATGGATATCTC-GGCTCTCGCATCGATGAAGAGCGCAGCGAAATGCGATACGTGGTGC-GAATTGCAGAATCCCGCGAACC-ATCGAGTCTTTGAACGCAAGTTGCGCCCGAGGCCAAC-CGGCCAAGGGCACGTCTGCCTG---GGCGTCAA-GCGTTGCGTCGCTCC

Coelogyne_fimbriata_SG1059 ACGAC-TCTCGGCAATGGATATCTC-GGCTCTCGCATCGATGAAGAGCGCAGCGAAATGCGATACGTGGTGC-GAATTGCAGAATCCCGCGAACC-ATCGAGTCTTTGAACGCAAGTTGCGCCCGAGGCCAAC-CGGCCAAGGGCACGTCTGCCTG---GGCGTCAA-GCGTTGCGTCGCTCC

Coelogyne_fimbriata_SG1061F ACGAC-TCTCGGCAATGGATATCTC-GGCTCTCGCATCGATGAAGAGCGCAGCGAAATGCGATACGTGGTGC-GAATTGCAGAATCCCGCGAACC-ATCGAGTCTTTGAACGCAAGTTGCGCCCGAGGCCAAC-CGGCCAAGGGCACGTCTGCCTG---GGCGTCAA-GCGTTGCGTCGCTCC

Coelogyne_fimbriata_SG1062D ACGAC-TCTCGGCAATGGATATCTC-GGCTCTCGCATCGATGAAGAGCGCAGCGAAATGCGATACGTGGTGC-GAATTGCAGAATCCCGCGAACC-ATCGAGTCTTTGAACGCAAGTTGCGCCCGAGGCCAAC-CGGCCAAGGGCACGTCTGCCTG---GGCGTCAA-GCGTTGCGTCGCTCC

Coelogyne_fimbriata_SG1079 ACGAC-TCTCGGCAATGGATATCTC-GGCTCTCGCATCGATGAAGAGCGCAGCGAAATGCGATACGTGGTGC-GAATTGCAGAATCCCGCGAACC-ATCGAGTCTTTGAACGCAAGTTGCGCCCGAGGCCAAC-CGGCCAAGGGCACGTCTGCCTG---GGCGTCAA-GCGTTGCGTCGCTCC

Coelogyne_fimbriata_var_leungiana_KFBG640 ACGAC-TCTCGGCAATGGATATCTC-GGCTCTCGCATCGATGAAGAGCGCAGCGAAATGCGATACGTGGTGC-GAATTGCAGAATCCCGCGAACC-ATCGAGTCTTTGAACGCAAGTTGCGCCCGAGGCCAAC-CGGCCAAGGGCACGTCTGCCTG---GGCGTCAA-GCGTTGCGTCGCTCC

Coelogyne_fimbriata_var_leungiana_KY966507 ACGAC-TCTCGGCAATGGATATCTC-GGCTCTCGCATCGATGAAGAGCGCAGCGAAATGCGATACGTGGTGC-GAATTGCAGAATCCCGCGAACC-ATCGAGTCTTTGAACGCAAGTTGCGCCCGAGGCCAAC-CGGCCAAGGGCACGTCTGCCTG---GGCGTCAA-GCGTTGCGTCGCTCC

Coelogyne_fimbriata_var_leungiana_SG1058A ACGAC-TCTCGGCAATGGATATCTC-GGCTCTCGCATCGATGAAGAGCGCAGCGAAATGCGATACGTGGTGC-GAATTGCAGAATCCCGCGAACC-ATCGAGTCTTTGAACGCAAGTTGCGCCCGAGGCCAAC-CGGCCAAGGGCACGTCTGCCTG---GGCGTCAA-GCGTTGCGTCGCTCC

Collabium_chinense_KF560544 ACGAC-TCTCGGCAATGGATATCTC-GGCTCTCGCATCGATGAAGAGCGCAGCGAAATGCGATACGTGGTGC-GAATTGCAGAATCCCGCGAACC-ATCGAGTCTTTGAACGCAAGTTGCGCCCGAGGCCAAC-CGGCCAAGGGCACGTCTGCCTG---GGCGTCAA-GCGTTGCGTCGCTCC

Collabium_chinense_KM025156 ACGAC-TCTCGGCAATGGATATCTC-GGCTCTCGCATCGATGAAGAGCGCAGCGAAATGCGATACGTGGTGC-GAATTGCAGAATCCCGCGAACC-ATCGAGTCTTTGAACGCAAGTTGCGCCCGAGGCCAAC-CGGCCAAGGGCACGTCTGCCTG---GGCGTCAA-GCGTTGCGTCGCTCC

Crepidium_acuminatum_AB290884 ATGAC-TCTCGGCAATGGATATCTC-GGCTCTTGCATCGATGAAGAGCGCAGCAAAATGCGATACGTGATGC-GAATTGCAGAATCCCGCGAACC-ATCGAGTTTTTGAACGCAAGTTGCGCCCGAGGCCAAC-CGGTCAAGGGCACGTTTTCCTG---GGTGTCAA-GCGTTGCTTCGCTTT

Crepidium_acuminatum_JN114478 ATGAC-TCTCGGCAATGGATATCTC-GGCTCTTGCATCGATGAAGAGCGCAGCAAAATGCGATACGTGATGC-GAATTGCAGAATCCCGCGAACC-ATCGAGTTTTTGAACGCAAGTTGCGCCCGAGGCCAAC-CGGTCAAGGGCACGTTTTCCTG---GGTGTCAA-GCGTTGCTTCGCTTT

Crepidium_acuminatum_JN114479 ATGAC-TCTCGGCAATGGATATCTC-GGCTCTTGCATCGATGAAGAGCGCAGCAAAATGCGATACGTGATGC-GAATTGCAGAATCCCGCGAACC-ATCGAGTTTTTGAACGCAAGTTGCGCCCGAGGCCAAC-CGGTCAAGGGCACGTTTTCCTG---GGTGTCAA-GCGTTGCTTCGCTTT

Crepidium_acuminatum_JN114480 ATGAC-TCTCGGCAATGGATATCTC-GGCTCTTGCATCGATGAAGAGCGCAGCAAAATGCGATACGTGATGC-GAATTGCAGAATCCCGCGAACC-ATCGAGTTTTTGAACGCAAGTTGCGCCCGAGGCCAAC-CGGTCAAGGGCACGTTTTCCTG---GGTGTCAA-GCGTTGCTTCGCTTT

Crepidium_acuminatum_JN114481 ATGAC-TCTCGGCAATGGATATCTC-GGCTCTTGCATCGATGAAGAGCGCAGCAAAATGCGATACGTGATGC-GAATTGCAGAATCCCGCGAACC-ATCGAGTTTTTGAACGCAAGTTGCGCCCGAGGCCAAC-CGGTCAAGGGCACGTTTTCCTG---GGTGTCAA-GCGTTGCTTCGCTTT

Crepidium_acuminatum_JN114482 ATGAC-TCTCGGCAATGGATATCTC-GGCTCTTGCATCGATGAAGAGCGCAGCAAAATGCGATACGTGATGC-GAATTGCAGAATCCCGCGAACC-ATCGAGTTTTTGAACGCAAGTTGCGCCCGAGGCCAAC-CGGTCAAGGGCACGTTTTCCTG---GGTGTCAA-GCGTTGCTTCGCTTT

Crepidium_acuminatum_KJ459274 ATGAC-TCTCGGCAATGGATATCTC-GGCTCTTGCATCGATGAAGAGCGCAGCAAAATGCGATACGTGATGC-GAATTGCAGAATCCCGCGAACC-ATCGAGTTTTTGAACGCAAGTTGCGCCCGAGGCCAAC-CGGTCAAGGGCACGTTTTCCTG---GGTGTCAA-GCGTTGCTTCGCTTT

Crepidium_acuminatum_KX277725 ATGAC-TCTCGGCAATGGATATCTC-GGCTCTTGCATCGATGAAGAGCGCAGCAAAATGCGATACGTGATGC-GAATTGCAGAATCCCGCGAACC-ATCGAGTTTTTGAACGCAAGTTGCGCCCGAGGCCAAC-CGGTCAAGGGCACGTTTTCCTG---GGTGTCAA-GCGTTGCTTCGCTTT

Crepidium_acuminatum_MF287967 ATGAC-TCTCGGCAATGGATATCTC-GGCTCTTGCATCGATGAAGAGCGCAGCAAAATGCGATACGTGATGC-GAATTGCAGAATCCCGCGAACC-ATCGAGTTTTTGAACGCAAGTTGCGCCCGAGGCCAAC-CGGTCAAGGGCACGTTTTCCTG---GGTGTCAA-GCGTTGCTTCGCTTT

Crepidium_allanii_KFBG4610 ATGAC-TCTCGGCAATGGATATCTC-GGCTCTTGCATCGATGAAGAGCGCAGCAAAATGCGATACGTGATGC-GAATTGCAGAATCCCGCGAACC-ATCGAGTTTTTGAACGCAAGTTGCGCCCGAGGCCAAC-CGGTCAAGGGCACGTTTTCCTG---GGTGTCAA-GCTTTGCTTCGCTTT

Crepidium_allanii_KFBG4611 ATGAC-TCTCGGCAATGGATATCTC-GGCTCTTGCATCGATGAAGAGCGCAGCAAAATGCGATACGTGATGC-GAATTGCAGAATCCCGCGAACC-ATCGAGTTTTTGAACGCAAGTTGCGCCCGAGGCCAAC-CGGTCAAGGGCACGTTTTCCTG---GGTGTCAA-GCTTTGCTTCGCTTT

Crepidium_purpureum_PK12185 ATGAC-TCTCGGCAATGGATATCTC-GGCTCTTGCATCGATGAAGAGCGCAGCAAAATGCGATACGTGATGC-GAATTGCAGAATCCCGCGAACC-ATCGAGTATTTGAACGCAAGTTGCGCCCGAGGCCAAC-CGGTCAAGGGCACGTTTACCTG---GGTGTCAA-GCGTTGCTTCGCTTC

Crepidium_purpureum_SG1193 ATGAC-TCTCGGCAATGGATATCTC-GGCTCTTGCATCGATGAAGAGCGCAGCAAAATGCGATACGTGATGC-GAATTGCAGAATCCCGCGAACC-ATCGAGTTTTTGAACGCAAGTTGCGCCCGAGGCCAAC-CGGTCAAGGGCACGTCTTCCTG---GGTGTCAA-GCGTTGCTACGCTTT

Crepidium_purpureum_SG1194 ATGAC-TCTCGGCAATGGATATCTC-GGCTCTTGCATCGATGAAGAGCGCAGCAAAATGCGATACGTGATGC-GAATTGCAGAATCCCGCGAACC-ATCGAGTTTTTGAACGCAAGTTGCGCCCGAGGCCAAC-CGGTCAAGGGCACGTCTTCCTG---GGTGTCAA-GCGTTGCTACGCTTT

Crepidium_purpureum_SG1379 ATGAC-TCTCGGCAATGGATATCTC-GGCTCTTGCATCGATGAAGAGCGCAGCAAAATGCGATACGTGATGC-GAATTGCAGAATCCCGCGAACC-ATCGAGTTTTTGAACGCAAGTTGCGCCCGAGGCCAAC-CGGTCAAGGGCACGTCTTCCTG---GGTGTCAA-GCGTTGCTACGCTTT

Cryptochilus_roseus_KFBG2118 ACGAC-TCTCGGCAATGGATATCTC-GGCTCTCGCATCGATGAAGAGCGCAGCGAAATGCGATACGTGGTGT-GAATTGCAGAATCCCGCGAACC-ATCGAGTCTTTGAACGCAAGTTGCGCCCGAGGCCAAC-CGGCTGAGGGCACGTCTGCCTG---GGCGTCAA-GCATTATGTCACTCC

Cryptochilus_roseus_KY239358 ACGAC-TCTCGGCAATGGATATCTC-GGCTCTCGCATCGATGAAGAGCGCAGCGAAATGCGATACGTGGTGT-GAATTGCAGAATCCCGCGAACC-ATCGAGTCTTTGAACGCAAGTTGCGCCCGAGGCCAAC-CGGCTGAGGGCACGTCTGCCTG---GGCGTCAA-GCATTATGTCACTCC

Cryptochilus_roseus_KY966513 ACGAC-TCTCGGCAATGGATATCTC-GGCTCTCGCATCGATGAAGAGCGCAGCGAAATGCGATACGTGGTGT-GAATTGCAGAATCCCGCGAACC-ATCGAGTCTTTGAACGCAAGTTGCGCCCGAGGCCAAC-CGGCTGAGGGCACGTCTGCCTG---GGCGTCAA-GCATTATGTCACTCC

Cryptochilus_roseus_KY966514 ACGAC-TCTCGGCAATGGATATCTC-GGCTCTCGCATCGATGAAGAGCGCAGCGAAATGCGATACGTGGTGT-GAATTGCAGAATCCCGCGAACC-ATCGAGTCTTTGAACGCAAGTTGCGCCCGAGGCCAAC-CGGCTGAGGGCACGTCTGCCTG---GGCGTCAA-GCATTATGTCACTCC

Cryptochilus_roseus_PK12087 ACGAC-TCTCGGCAATGGATATCTC-GGCTCTCGCATCGATGAAGAGCGCAGCGAAATGCGATACGTGGTGT-GAATTGCAGAATCCCGCGAACC-ATCGAGTCTTTGAACGCAAGTTGCGCCCGAGGCCAAC-CGGCTGAGGGCACGTCTGCCTG---GGCGTCAA-GCATTATGTCACTCC

Cryptochilus_roseus_PK12088 ACGAC-TCTCGGCAATGGATATCTC-GGCTCTCGCATCGATGAAGAGCGCAGCGAAATGCGATACGTGGTGT-GAATTGCAGAATCCCGCGAACC-ATCGAGTCTTTGAACGCAAGTTGCGCCCGAGGCCAAC-CGGCTGAGGGCACGTCTGCCTG---GGCGTCAA-GCATTATGTCACTCC

Cryptochilus_roseus_PK12187 ACGAC-TCTCGGCAATGGATATCTC-GGCTCTCGCATCGATGAAGAGCGCAGCGAAATGCGATACGTGGTGT-GAATTGCAGAATCCCGCGAACC-ATCGAGTCTTTGAACGCAAGTTGCGCCCGAGGCCAAC-CGGCTGAGGGCACGTCTGCCTG---GGCGTCAA-GCATTATGTCACTCC

Cryptostylis_arachnites_PK12188 AGGAC-TCTCGGCAATGGATATCTT-GGCTCTTGCATCGATGAAGAGCGCAGCGAAATGCGATATGTGGTGT-GAATTGCAGGATCCCGCGAACC-ATCGAGTTTTTGAACGCAAGTTGCGCCTGAGGCCGAC-TAGCCGAGGGCACGTCTGCCTG---GGCGTCAT-GCATTATGTCGCTCC

Cryptostylis_arachnites_SG027 AGGAC-TCTCGGCAATGGATATCTT-GGCTCTTGCATCGATGAAGAGCGCAGCGAAATGCGATATGTGGTGT-GAATTGCAGGATCCCGCGAACC-ATCGAGTTTTTGAACGCAAGTTGCGCCTGAGGCCGAC-TAGCCGAGGGCACGTCTGCCTG---GGCGTCAT-GCATTATGTCGCTCC

Cryptostylis_arachnites_SG1142 AGGAC-TCTCGGCAATGGATATCTT-GGCTCTTGCATCGATGAAGAGCGCAGCGAAATGCGATATGTGGTGT-GAATTGCAGGATCCCGCGAACC-ATCGAGTTTTTGAACGCAAGTTGCGCCTGAGGCCGAC-TAGCCGAGGGCACGTCTGCCTG---GGCGTCAT-GCATTATGTCGCTCC

Cryptostylis_arachnites_SG1380 AGGAC-TCTCGGCAATGGATATCTT-GGCTCTTGCATCGATGAAGAGCGCAGCGAAATGCGATATGTGGTGT-GAATTGCAGGATCCCGCGAACC-ATCGAGTTTTTGAACGCAAGTTGCGCCTGAGGCCGAC-TAGCCGAGGGCACGTCTGCCTG---GGCGTCAT-GCATTATGTCGCTCC

Curculigo_orchioides_PK12054 ACGAC-TCTCGGCAACGGATATCTA-GGCTCTCGCATCGATGAAGAACGTAGCGAAATGCGATACTTGGTGT-GAATTGCAGAATCCCGTGAACC-ATCGAGTCTTTGAACGCAAGTTGCGCCCGAGGCCATC-CGGCCGAGGGCACGCCTGCCTG---GGCGTCACGCCATTGCGTCGCTCC

Cymbidium_aloifolium_AF284695 ACGAC-TCTCGGCAATGGATATCTC-GGCTCTCGCATCGATGAAGAGCGCAGCGAAATGCGATACGTGGTGC-GAATTGCAGAATCCCGCGAACC-ATCGAGTCTTTGAACGCAAGTTGCGCCCGAGGCCAGC-CGGCCGAGGGCACGTCCGCCTG---GGCGTCAA-GCATCGCGTCGCTCC

Cymbidium_aloifolium_AF470526 ACGAC-TCTCGGCAATGGATATCTC-GGCTCTCGCATCGATGAAGAGCGCAGCGAAATGCGATACGTGGTGA-GAATTGCAGAATCCCGCGAACC-ATTGAGTCTTTGAACGCAAGTTGCGCCCGAGGCCAGC-CGGCNGAGGGCACGTCCGCTTG---GGCGTCAA-GCATCGGGTCGCTCC

Cymbidium_aloifolium_AJ300269 ATGAC-TCTCGGTAATGGATATCTC-GACTCTCGCATCGATGAAGAGCGCAGTGAAATGCGATACGTGGTGC-GAATTACAGAATCCCGTGAACC-ATCGAGTCTTTGAACGCAAGTTATGCCCGAGGCTAGC-CGGCCGAGGGCACGTCCGCCTG---GGCGTCAA-GCATCGCATCGCTCC

Cymbidium_aloifolium_JF729014 ACGAC-TCTCGGCAATGGATATCTC-GGCTCTCGCATCGATGAAGAGCGCAGCGAAATGCGATACGTGGTGC-GAATTGCAGAATCCCGCGAACC-ATCGAGTCTTTGAACGCAAGTTGCGCCCGAGGCCAGC-CGGCCGAGGGCACGTCCGCCTG---GGCGTCAA-GCATCGCGTCGCTCC

Cymbidium_aloifolium_JN114485 ACGAC-TCTCGGCAATGGATATCTC-GGCTCTCGCATCGATGAAGAGCGCAGCGAAATGCGATACGTGGTGC-GAATTGCAGAATCCCGCGAACC-ATCGAGTCTTTGAACGCAAGTTGCGCCCGAGGCCAGC-CGGCCGAGGGCACGTCCGCCTG---GGCGTCAA-GCATCGCGTCGCTCC

Cymbidium_aloifolium_JN114486 ACGAC-TCTCGGCAATGGATATCTC-GGCTCTCGCATCGATGAAGAGCGCAGCGAAATGCGATACGTGGTGC-GAATTGCAGAATCCCGCGAACC-ATCGAGTCTTTGAACGCAAGTTGCGCCCGAGGCCAGC-CGGCCGAGGGCACGTCCGCCTG---GGCGTCAA-GCATCGCGTCGCTCC

Cymbidium_aloifolium_KFBG2049 ACGAC-TCTCGGCAATGGATATCTC-GGCTCTCGCATCGATGAAGAGCGCAGCGAAATGCGATACGTGGTGC-GAATTGCAGAATCCCGCGAACC-ATCGAGTCTTTGAACGCAAGTTGCGCCCGAGGCCAGC-CGGCCGAGGGCACGTCCGCCTG---GGCGTCAA-GCATCGCGTCGCTCC

Cymbidium_aloifolium_KFBG2205 ACGAC-TCTCGGCAATGGATATCTC-GGCTCTCGCATCGATGAAGAGCGCAGCGAAATGCGATACGTGGTGC-GAATTGCAGAATCCCGCGAACC-ATCGAGTCTTTGAACGCAAGTTGCGCCCGAGGCCAGC-CGGCCGAGGGCACGTCCGCCTG---GGCGTCAA-GCATCGCGTCGCTCC

Cymbidium_ensifolium_AF284716 ACGAC-TCTCGGCAATGGATATCTC-GGCTCTCGCATCGATGAAGAGCGCAGCGAAATGCGATACGTGGTGC-GAATTGCAGAATCCCGCGAACC-ATCGAGTCTTTGAACGCAAGTTGCGCCCGAGGCCAGC-CGGCCGAGGGCACGTCCGCCTG---GGCGTCAA-GCATCGCGTCGCTCC

Cymbidium_ensifolium_AF284717 ACGAC-TCTCGGCAATGGATATCTC-GGCTCTCGCATCGATGAAGAGCGCAGCGAAATGCGATACGTGGTGC-GAATTGCAGAATCCCGCGAACC-ATCGAGTCTTTGAACGCAAGTTGCGCCCGAGGCCAGC-CGGCCGAGGGCACGTCCGCCTG---GGCGTCAA-GCATCGCGTCGCTCC

Cymbidium_ensifolium_AF470496 ACGAC-TCTCGGCAATGGATATCTC-GGCTCTCGCATCGATGAAGAGCGCAGCGAAATGCGATACGTGGTGC-GAATTGCAGAATCCCGCGAACC-ATCGAGTCTTTGAACGCAAGTTGCGCCCGAGGCCAGC-CGGCCGAGGGCACGTCCGCCTG---GGCGTCAA-GCATCGCGTCGCTCC

Cymbidium_ensifolium_AF470512 ACGAC-TCTCGGCAATGGATATCTC-GGCTCTCGCATCGATGAAGAGCGCAGCGAAATGCGATACGTGGTGC-GAATTGCAGAATCCCGCGAACC-ATCGAGTCTTTGAACGCAAGTTGCGCCCGAGGCCAGC-CGGCCGAGGGCACGTCCGCCTG---GGCGTCAA-GCATCGCGTCGCTCC

Cymbidium_ensifolium_AJ300273 ATGAC-TCTTGATAATGGATATCTT-GGCTCTCGCATTGATGAAGAGTGCAGTGAAATGTGATACGTGGTGA-GAATTATAGAATCCCGTGAACC-ATCATGTCTTTGAACGCAAGATGCGCCCGAGGCTAGC-CGGCCAAGGGCGTGTCCGCCTA---GGCATCAA-GCATCTTGTCGCTCT

Cymbidium_ensifolium_FJ899748 ATGAC-TCTCAGCAATGGATATCTT-GGCTCTCACATTGATGAAGAGCATAACAAAATGCAATACGTGGTGC-GAATTATAGAATCCTGCGAACC-ATTGAGTCTTTGAACGCAAGTTACGCCCGAGGCCAGC-CGGCCGAGCGCACGTCCGCCTG---GGCGTCAA-GCATCGCATCACTCC

Cymbidium_ensifolium_HQ263134 ATGAC-TCTCGATAATGGATATCTC-GGCTCTCGCATTGATGAAGAGTGCAGC-AAATGTGATATGTTGTGC-GAATTACAGAGTCCCATGAACC-ATCAAGTCTTTGAATGCATATTGTGCCTGAGGCCAGC-CAGCCGAGGGCACGTCCGCCTG---GGTGTCAA-GCATCACGTCACTCT

Cymbidium_ensifolium_JX399078 ACGAC-TCTCAGCAATGGATATCTC-AGCTCTCGCATCGATGAAGAGCACAGCGAAATGTGATACGTGGTGT-GAATTGCAGAATCCCGTGAACC-ATCGAGTCTTTGACTGCAAGTTGCGCCCGAGGCCAGC-CGACTGAGGGCACGTCCGCCTG---GGCGTCAA-TTGTCGTGTCTCTCC

Cymbidium_ensifolium_KJ597843 ACGAC-TCTCGGCAATGGATATCTC-GGCTCTCGCATCGATGAAGAGCGCAGCGAAATGCGATACGTGGTGC-GAATTGCAGAATCCCGCGAACC-ATCGAGTCTTTGAACGCAAGTTGCGCCCGAGGCCAGC-CGGCCGAGGGCACGTCCGCCTG---GGCGTCAA-GCATCGCGTCGCTCC

Cymbidium_ensifolium_KJ597844 ACGAC-TCTCGGCAATGGATATCTC-GGCTCTCGCATCGATGAAGAGCGCAGCGAAATGCGATACGTGGTGC-GAATTGCAGAATCCCGCGAACC-ATCGAGTCTTTGAACGCAAGTTGCGCCCGAGGCCAGC-CGGCCGAGGGCACGTCCGCCTG---GGCGTCAA-GCATCGCGTCGCTCC

Cymbidium_ensifolium_KJ597845 ACGAC-TCTCGGCAATGGATATCTC-GGCTCTCGCATCGATGAAGAGCGCAGCGAAATGCGATACGTGGTGC-GAATTGCAGAATCCCGCGAACC-ATCGAGTCTTTGAACGCAAGTTGCGCCCGAGGCCAGC-CGGCCGAGGGCACGTCCGCCTG---GGCGTCAA-GCATCGCGTCGCTCC

Cymbidium_ensifolium_KJ597846 ACGAC-TCTCGGCAATGGATATCTC-GGCTCTCGCATCGATGAAGAGCGCAGCGAAATGCGATACGTGGTGC-GAATTGCAGAATCCCGCGAACC-ATCGAGTCTTTGAACGCAAGTTGCGCCCGAGGCCAGC-CGGCCGAGGGCACGTCCGCCTG---GGCGTCAA-GCATCGCGTCGCTCC

Cymbidium_ensifolium_KT338675 ACGAC-TCTCGGCAATGGATATCTC-GGCTCTCGCATCGATGAAGAGCGCAGCGAAATGCGATACGTGGTGC-GAATTGCAGAATCCCGCGAACC-ATCGAGTCTTTGAACGCAAGTTGCGCCCGAGGCCAGC-CGGCCGAGGGCACGTCCGCCTG---GGCGTCAA-GCATCGCGTCGCTCC

Cymbidium_ensifolium_PK12208 ACGAC-TCTCGGCAATGGATATCTC-GGCTCTCGCATCGATGAAGAGCGCAGCGAAATGCGATACGTGGTGC-GAATTGCAGAATCCCGCGAACC-ATCGAGTCTTTGAACGCAAGTTGCGCCCGAGGCCAGC-CGGCCGAGGGCACGTCCGCCTG---GGCGTCAA-GCATCGCGTCGCTCC

Cymbidium_ensifolium_SG1214 ACGAC-TCTCGGCAATGGATATCTC-GGCTCTCGCATCGATGAAGAGCGCAGCGAAATGCGATACGTGGTGC-GAATTGCAGAATCCCGCGAACC-ATCGAGTCTTTGAACGCAAGTTGCGCCCGAGGCCAGC-CGGCCGAGGGCACGTCCGCCTG---GGCGTCAA-GCATCGCGTCGCTCC

Cymbidium_ensifolium_SG1278 ACGAC-TCTCGGCAATGGATATCTC-GGCTCTCGCATCGATGAAGAGCGCAGCGAAATGCGATACGTGGTGC-GAATTGCAGAATCCCGCGAACC-ATCGAGTCTTTGAACGCAAGTTGCGCCCGAGGCCAGC-CGGCCGAGGGCACGTCCGCCTG---GGCGTCAA-GCATCGCGTCGCTCC

Cymbidium_ensifolium_SG1285 ACGAC-TCTCGGCAATGGATATCTC-GGCTCTCGCATCGATGAAGAGCGCAGCGAAATGCGATACGTGGTGC-GAATTGCAGAATCCCGCGAACC-ATCGAGTCTTTGAACGCAAGTTGCGCCCGAGGCCAGC-CGGCCGAGGGCACGTCCGCCTG---GGCGTCAA-GCATCGCGTCGCTCC

Cymbidium_kanran_AF284720 ACGAC-TCTCGGCAATGGATATCTC-GGCTCTCGCATCGATGAAGAGCGCAGCGAAATGCGATACGTGGTGC-GAATTGCAGAATCCCGCGAACC-ATCGAGTCTTTGAACGCAAGTTGCGCCCGAGGCCAGC-CGGCCGAGGGCACGTCCGCCTG---GGCGTCAA-GCATCGCGTCGCTCC

Cymbidium_kanran_AF470495 ACGAC-TCTCGGCAATGGATATCTC-GGCTCTCGCATCGATGAAGAGCGCAGCGAAATGCGATACGTGGTGC-GAATTGCAGAATCCCGCGAACC-ATCGAGTCTTTGAACGCAAGTTGCGCCCGAGGCCAGC-CGGCCGAGGGCACGTCCGCCTG---GGCGTCAA-GCATCGCGTCGCTCC

Cymbidium_kanran_AJ300279 ATGAC-TCTTGATAATGGATATCTT-GGCTCTCGCATTGATGAAGAGTGCAGTGAAATGTGATACGTGGTGA-GAATTATAGAATCCCGTGAACC-ATCATGTCTTTGAACGCAAGATGCGCCCGAGGCTAGC-CGGCCAAGGGCATGTCCGCCTA---GGCATCAA-GCATCTTGTCGCTCT

Cymbidium_kanran_AJ300280 ATGAC-TCTCGATAATGGATATCTC-AGCTCTCACATCAATGAAAAGCGTAGAAAAATGTGATATGTGGTGC-GAATTATAGAATCCCGTGAACC-ATTGAGTCTTTGAATGCAAGTTACGCTCAAGGTCAGC-TGACCGAGGGTACGTCCCCCTG---GGCATCAA-GCATCGTGTCGCTCT

Cymbidium_kanran_AJ300281 ATGAC-TCTCAACAATGGATATCTT-GGCTCTCACATTGATGAAGAGCACAACAAAATGCAATACATGGTGC-GAATTGTAGAATCCTGCGAACC-ATTGAGTCTTTGAATGCAAGTTACACTTGAGGCCAGC-CGGCCGAGCGCACGTCCGCCTG---GGCGTCAA-GCATCGCATCACTCC

Cymbidium_kanran_AJ300282 ATGAC-TCTCGACAATGGATATCTC-GGCTCTCACATCGATGAAGAGTGCAGCGAAATGTGATACGTGGTGT-GAATTGCAGAATCCCACGAACC-ATCGAGTCTTTGAATGCAAATTGTGCCCAAGGCCAGT-CGGCTGAGGGCACGTTCACCTG---GGCGTCAA-GCATCGCGTCGCTCC

Cymbidium_kanran_HQ263140 ATGAC-TCTTAGTAATGGATATCTT-GGTTCTCGCATCGATGAAGAGCGCAGCAAAA-GAGATATGTGGTAT-GAATTGTAGAATCTCGTGAACC-ATCGAGTCTTTGAACTAAAGTTGCGCCTGAGGGAAAC-CAGCTGAAGGCACATCCGCCTG---GGCATCAA-GAGTCACATCTCTTC

Cymbidium_kanran_JX202654 ACGAC-TCTCGGCAATGGATATCTC-GGCTCTCGCATCGATGAAGAGCGCAGCGAAATGCGATACGTGGTGC-GAATTGCAGAATCCCGCGAACC-ATCGAGTCTTTGAACGCAAGTTGCGCCCGAGGCCAGC-CGGCCGAGGGCACGTCCGCCTG---GGCGTCAA-GCATCGCGTCGCTCC

Cymbidium_kanran_KF560534 ACGAC-TCTCGGCAATGGATATCTC-GGCTCTCGCATCGATGAAGAGCGCAGCGAAATGCGATACGTGGTGC-GAATTGCAGAATCCCGCGAACC-ATCGAGTCTTTGAACGCAAGTTGCGCCCGAGGCCAGC-CGGCCGAGGGCACGTCCGCCTG---GGCGTCAA-GCATCGCGTCGCTCC

Cymbidium_kanran_KJ597836 ACGAC-TCTCGGCAATGGATATCTC-GGCTCTCGCATCGATGAAGAGCGCAGCGAAATGCGATACGTGGTGC-GAATTGCAGAATCCCGCGAACC-ATCGAGTCTTTGAACGCAAGTTGCGCCCGAGGCCAGC-CGGCCGAGGGCACGTCCGCCTG---GGCGTCAA-GCATCGCGTCGCTCC

Cymbidium_kanran_KJ597837 ACGAC-TCTCGGCAATGGATATCTC-GGCTCTCGCATCGATGAAGAGCGCAGCGAAATGCGATACGTGGTGC-GAATTGCAGAATCCCGCGAACC-ATCGAGTCTTTGAACGCAAGTTGCGCCCGAGGCCAGC-CGGCCGAGGGCACGTCCGCCTG---GGCGTCAA-GCATCGCGTCGCTCC

Cymbidium_kanran_KJ597838 ACGAC-TCTCGGCAATGGATATCTC-GGCTCTCGCATCGATGAAGAGCGCAGCGAAATGCGATACGTGGTGC-GAATTGCAGAATCCCGCGAACC-ATCGAGTCTTTGAACGCAAGTTGCGCCCGAGGCCAGC-CGGCCGAGGGCACGTCCGCCTG---GGCGTCAA-GCATCGCGTCGCTCC

Cymbidium_kanran_KJ597839 ACGAC-TCTCGGCAATGGATATCTC-GGCTCTCGCATCGATGAAGAGCGCAGCGAAATGCGATACGTGGTGC-GAATTGCAGAATCCCGCGAACC-ATCGAGTCTTTGAACGCAAGTTGCGCCCGAGGCCAGC-CGGCCGAGGGCACGTCCGCCTG---GGCGTCAA-GCATCGCGTCGCTCC

Cymbidium_kanran_KT338677 ACGAC-TCTCGGCAATGGATATCTC-GGCTCTCGCATCGATGAAGAGCGCAGCGAAATGCGATACGTGGTGC-GAATTGCAGAATCCCGCGAACC-ATCGAGTCTTTGAACGCAAGTTGCGCCCGAGGCCAGC-CGGCCGAGGGCACGTCCGCCTG---GGCGTCAA-GCATCGCGTCGCTCC

Cymbidium_lancifolium_AF284724 ACGAC-TCTCGGCAATGGATATCTC-GGCTCTCGCATCGATGAAGAGCGCAGCGAAATGCGATACGTGGTGC-GAATTGCAGAATCCCGCGAACC-ATCGAGTCTTTGAACGCAAGTTGCGCCCGAGGCCAGC-CGGCCGAGGGCACGTCCGCCTG---GGCGTCAA-GCGTCGCGTCGCTCC

Cymbidium_lancifolium_AF470509 ATGAC-TCTCGGCAATGGATATCTC-GGCTCTCGCATCGATGAAGAGCGCAGCGAAATGCGATACGTGGTGC-GAATTGCAGAATCCCGCGAACC-ATCGAGTCTTTGAACGCAAGTTGCGCCCGAGGCCAGC-CGGCCGAGGGCACGTCCGCCTG---GGCGTCAA-GCGTCGCGTCGCTCC

Cymbidium_lancifolium_AF470520 ACGAC-TCTCGGCAATGGATATCTC-GGCTCTCGCATCGATGAAGAGCGCAGCGAAATGCGATACGTGGTGC-GAATTGCAGAATCCCGCGAACC-ATCGAGTCTTTGAACGCAAGTTGCGCCCGAGGCCAGC-CGGCCGAGGGCACGTCCGCCTG---GGCGTCAA-GCGTCGCGTCGCTCC

Cymbidium_lancifolium_AJ300285 ATGTC-TCTCAGCAATGTATATCTC-AGCTCTCGCATCGATGAAAAGTGTAGTGAAATGCGATACGTGGTAA-GAATTGTAGAATCCTGTGAACC-ATCGAGTCTTTGAACGCAAGTTGCGCCTGAGGCCAGT-CGGCCGAGGGCACGTCCGCCTA---GGCGTCAA-GCATCACGTCGCTCC

Cymbidium_lancifolium_FJ899751 AAGAC-TCTCGGCAATGGATATCTC-AGCTCTCGCATTAATGAAGAGCACAGTGAAATGTGATACATGGTGT-GAATTGCAGAATCCTGCGAACC-ATCGAGTCTTTGAATGCAAGTTACGCCCGAGGCCAGC-CGGCCGAGGGCACATCCACCTG---GGCGTCAA-CTGTCGCATCGCTCC

Cymbidium_lancifolium_KFBG734 ACGAC-TCTCGGCAATGGATATCTC-GGCTCTCGCATCGATGAAGAGCGCAGCGAAATGCGATACGTGGTGC-GAATTGCAGAATCCCGCGAACC-ATCGAGTCTTTGAACGCAAGTTGCGCCCGAGGCCAGC-CGGCCGAGGGCACGTCCGCCTG---GGCGTCAA-GCGTCGCGTCGCTCC

Cymbidium_lancifolium_KJ597851 ACGAC-TCTCGGCAATGGATATCTC-GGCTCTCGCATCGATGAAGAGCGCAGCGAAATGCGATACGTGGTGC-GAATTGCAGAATCCCGCGAACC-ATCGAGTCTTTGAACGCAAGTTGCGCCCGAGGCCAGC-CGGCCGAGGGCACGTCCGCCTG---GGCGTCAA-GCGTCGCGTCGCTCC

Cymbidium_lancifolium_KJ597852 ACGAC-TCTCGGCAATGGATATCTC-GGCTCTCGCATCGATGAAGAGCGCAGCGAAATGCGATACGTGGTGC-GAATTGCAGAATCCCGCGAACC-ATCGAGTCTTTGAACGCAAGTTGCGCCCGAGGCCAGC-CGGCCGAGGGCACGTCCGCCTG---GGCGTCAA-GCGTCGCGTCGCTCC

Cymbidium_lancifolium_KY966515 ACGAC-TCTCGGCAATGGATATCTC-GGCTCTCGCATCGATGAAGAGCGCAGCGAAATGCGATACGTGGTGC-GAATTGCAGAATCCCGCGAACC-ATCGAGTCTTTGAACGCAAGTTGCGCCCGAGGCCAGC-CGGCCGAGGGCACGTCCGCCTG---GGCGTCMA-GCGTCGCGTCGCTCC

Cymbidium_lancifolium_PK12128 ACGAC-TCTCGGCAATGGATATCTC-GGCTCTCGCATCGATGAAGAGCGCAGCGAAATGCGATACGTGGTGC-GAATTGCAGAATCCCGCGAACC-ATCGAGTCTTTGAACGCAAGTTGCGCCCGAGGCCAGC-CGGCCGAGGGCACGTCCGCCTG---GGCGTCAA-GCGTCGCGTCGCTCC

Cymbidium_lancifolium_SG1274 ACGAC-TCTCGGCAATGGATATCTC-GGCTCTCGCATCGATGAAGAGCGCAGCGAAATGCGATACGTGGTGC-GAATTGCAGAATCCCGCGAACC-ATCGAGTCTTTGAACGCAAGTTGCGCCCGAGGCCAGC-CGGCCGAGGGCACGTCCGCCTG---GGCGTCAA-GCGTCGCGTCGCTCC

Cymbidium_sinense_AF284714 ACGAC-TCTCGGCAATGGATATCTC-GGCTCTCGCATCGATGAAGAGCGCAGCGAAATGCGATACGTGGTGC-GAATTGCAGAATCCCGCGAACC-ATCGAGTCTTTGAACGCAAGTTGCGCCCGAGGCCAGC-CGGCCGAGGGCACGTCCGCCTG---GGCGTCAA-GCATCGCGTCGCTCC

Cymbidium_sinense_AF284715 ACGAC-TCTCGGCAATGGATATCTC-GGCTCTCGCATCGATGAAGAGCGCAGCGAAATGCGATACGTGGTGC-GAATTGCAGAATCCCGCGAACC-ATCGAGTCTTTGAACGCAAGTTGCGCCCGAGGCCAGC-CGGCCGAGGGCACGTCCGCCTG---GGCGTCAA-GCATCGCGTCGCTCC

Cymbidium_sinense_AF470517 ACGAC-TCTCGGCAATGGATATCTC-GGCTCTCGCATCGATGAAGAGCGCAGCGAAATGCGATACGTGGTGC-GAATTGCAGAATCCCGCGAACC-ATCGAGTCTTTGAACGCAAGTTGCGCCCGAGGCCAGC-CGGCCGAGGGCACGTCCGCTTG---GGCGTCAA-GCATCGCGTCGCTCC

Cymbidium_sinense_AJ300272 ACGAC-TCTCGGCAATGGATATCTC-AGCTCTCACATCGATGAAGAGCGCAACAAAATGTGATACGTGGTGC-CAATTGCAAAATCCTGTGAACC-ATCAAGTCTTTGAACGCAAGTTACGCCCGAGGCCAGC-CAGCCGAGGGCACGTCCGCTTG---GGCGTCAA-GCATCACGTCGCTTC

Cymbidium_sinense_HQ263144 ACGAC-TCTCGAAAATGGATATCTT-GGCTCTCACATCAATGAAGAGCGTAGCGAAATGCGATATGTGATGC-GAATTGTAGAATCCCGCGAACC-ATCAAGTCTTTGAACGCAAGTTACGCTCGAGGTCAGC-CGGCCAAGGGCACGTCCGCTTG---GGCGTCAA-GCATCGCGTCTCTCT

Cymbidium_sinense_KJ597847 ACGAC-TCTCGGCAATGGATATCTC-GGCTCTCGCATCGATGAAGAGCGCAGCGAAATGCGATACGTGGTGC-GAATTGCAGAATCCCGCGAACC-ATCGAGTCTTTGAACGCAAGTTGCGCCCGAGGCCAGC-CGGCCGAGGGCACGTCCGCCTG---GGCGTCAA-GCATCGCGTCGCTCC

Cymbidium_sinense_KJ597848 ACGAC-TCTCGGCAATGGATATCTC-GGCTCTCGCATCGATGAAGAGCGCAGCGAAATGCGATACGTGGTGC-GAATTGCAGAATCCCGCGAACC-ATCGAGTCTTTGAACGCAAGTTGCGCCCGAGGCCAGC-CGGCCGAGGGCACGTCCGCCTG---GGCGTCAA-GCATCGCGTCGCTCC

Cymbidium_sinense_KJ597849 ACGAC-TCTCGGCAATGGATATCTC-GGCTCTCGCATCGATGAAGAGCGCAGCGAAATGCGATACGTGGTGC-GAATTGCAGAATCCCGCGAACC-ATCGAGTCTTTGAACGCAAGTTGCGCCCGAGGCCAGC-CGGCCGAGGGCACGTCCGCCTG---GGCGTCAA-GCATCGCGTCGCTCC

Cymbidium_sinense_KJ597850 ACGAC-TCTCGGCAATGGATATCTC-GGCTCTCGCATCGATGAAGAGCGCAGCGAAATGCGATACGTGGTGC-GAATTGCAGAATCCCGCGAACC-ATCGAGTCTTTGAACGCAAGTTGCGCCCGAGGCCAGC-CGGCCGAGGGCACGTCCGCCTG---GGCGTCAA-GCATCGCGTCGCTCC

Cymbidium_sinense_SG1213 ACGAC-TCTCGGCAATGGATATCTC-GGCTCTCGCATCGATGAAGAGCGCAGCGAAATGCGATACGTGGTGC-GAATTGCAGAATCCCGCGAACC-ATCGAGTCTTTGAACGCAAGTTGCGCCCGAGGCCAGC-CGGCCGAGGGCACGTCCGCCTG---GGCGTCAA-GCATCGCGTCGCTCC

Cymbidium_sinense_SG1218 ACGAC-TCTCGGCAATGGATATCTC-GGCTCTCGCATCGATGAAGAGCGCAGCGAAATGCGATACGTGGTGC-GAATTGCAGAATCCCGCGAACC-ATCGAGTCTTTGAACGCAAGTTGCGCCCGAGGCCAGC-CGGCCGAGGGCACGTCCGCCTG---GGCGTCAA-GCATCGCGTCGCTCC

Cymbidium_sinense_SG1341 ACGAC-TCTCGGCAATGGATATCTC-GGCTCTCGCATCGATGAAGAGCGCAGCGAAATGCGATACGTGGTGC-GAATTGCAGAATCCCGCGAACC-ATCGAGTCTTTGAACGCAAGTTGCGCCCGAGGCCAGC-CGGCCGAGGGCACGTCCGCCTG---GGCGTCAA-GCATCGCGTCGCTCC

Cymbidium_sinense_SG1342 ACGAC-TCTCGGCAATGGATATCTC-GGCTCTCGCATCGATGAAGAGCGCAGCGAAATGCGATACGTGGTGC-GAATTGCAGAATCCCGCGAACC-ATCGAGTCTTTGAACGCAAGTTGCGCCCGAGGCCAGC-CGGCCGAGGGCACGTCCGCCTG---GGCGTCAA-GCATCGCGTCGCTCC

Dendrobium_aduncum_AB593484 ACGAC-TCTCGGCAATGGATATCTC-GGCTCTCGCATCGATGAAGAGCGCAGCGAAATGCGATATGTGGTGC-GAATTGCAGAATCCCGCGAACC-ATCGAGTCTTTGAACGCAAGTTGCGCCCGAGGCCAAC-CGGCTAAGGGCACGTCCGCCTG---GGCGTCAA-GCATTTTATCGCTCC

Dendrobium_aduncum_GU339110 ACGAC-TCTCGGCAATGGATATCTC-GGCTCTCGCATCGATGAAGAGCGCAGTGAAATGCGATATGTGGTGC-GAATTGCAGAATCCCGCGAACC-ATCGAGTCTTTGAACGCAAGTTGCGCCCGAGGCCAAC-CGGCTAAGGGCACGTCCGCCTG---GGCGTCAA-GCATTTTATCGCTCC

Dendrobium_aduncum_HM590372 ACGAC-TCTCGGCAATGGATATCTC-GGCTCTCGCATCGATGAAGAGCGCAGCGAAATGCGATATGTGGTGC-GAATTGCAGAATCCCGCGAACC-ATCGAGTCTTTGAACGCAAGTTGCGCCTGAGGCCAAC-CGGCTGAGGGCACGTCCGCCTG---GGCGTCAA-GCATTTTATCGCTCC

Dendrobium_aduncum_HQ114249 ACGAC-TCTCGGCAATGGATATCTC-GGCTCTCGCATCGATGAAGAGCGCAGCGAAATGCGATACGTGGTGC-GAATTGCAGAATCCCGTGAACC-ATCGAGTCTTTGAACGCAAGTTGCGCCCGAGGCCAAC-CGGCCAAGGGCACGTCTGCCTG---GGCGTCAA-GCGTTATGTCGCTCT

Dendrobium_aduncum_HQ114250 ATGAC-TCTCGGCAATGGATATCTC-GGCTCTCGCATCGATGAAGAGCGCAGCGAAATGCGATATGTGGTGC-GAATTGCAGAATCCCGCGAACC-ATCGAGTCTTTGAACGCAAGTTGCGCCCGAGGCCAAC-CGGCCAAGGGCACGTCCGCCTG---GGCGTCAG-TCATTTTATCGCTCT

Dendrobium_aduncum_JF713083 ACGAC-TCTCGGCAATGGATATCTC-GGCTCTCGCATCGATGAAGAGCGCAGCGAAATGCGATATGTGGTGC-GAATTGCAGAATCCCGCGAACC-ATCGAGTCTTTGAACGCAAGTTGCGCCCGAGGCCAAC-CGGCTAAGGGCACGTCCGCCTG---GGCGTCAA-GCATTTTATCGCTCC

Dendrobium_aduncum_JN388580 ACGAC-TCTCGGCAATGGATATCTC-GGCTCTCGCATCGATGAAGAGCGCAGCGAAATGCGATATGTGGTGC-GAATTGCAGAATCCCGCGAACC-ATCGAGTCTTTGAACGCAAGTTGCGCCCGAGGCCAAC-CGGCTAAGGGCACGTCCGCCTG---GGCGTCAA-GCATTTTATCGCTCC

Dendrobium_aduncum_KC346887 ACGAC-TCTCGGCAATGGATATCTC-GGCTCTCGCATCGATGAAGAGCGCAGCGAAATGCGATATGTGGTGC-GAATTGCAGAATCCCGCGAACC-ATCGAGTCTTTGAACGCAAGTTGCGCCCGAGGCCAAC-CGGCTAAGGGCACGTCCGCCTG---GGCGTCAA-GCATTTTATCGCTCC

Dendrobium_aduncum_KC568295 ACGAC-TCTCGGCAATGGATATCTC-GGCTCTCGCATCGATGAAGAGCGCAGCGAAATGCGATATGTGGTGC-GAATTGCAGAATCCCGCGAACC-ATCGAGTCTTTGAACGCAAGTTGCGCCCGAGGCCAAC-CGGCTAAGGGCACGTCCGCCTG---GGCGTCAA-GCATTTTATCGCTCC

Dendrobium_aduncum_KF143428 ACGAC-TCTCGGCAATGGATATCTC-GGCTCTCGCATCGATGAAGAGCGCAGCGAAATGCGATATGTGGTGC-GAATTGCAGAATCCCGCGAACC-ATCGAGTCTTTGAACGCAAGTTGCGCCCGAGGCCAAC-CGGCTAAGGGCACGTCCGCCTG---GGCGTCAA-GCATTTTATCGCTCC

Dendrobium_aduncum_KFBG8766 ACGAC-TCTCGGCAATGGATATCTC-GGCTCTCGCATCGATGAAGAGCGCAGCGAAATGCGATATGTGGTGC-GAATTGCAGAATCCCGCGAACC-ATCGAGTCTTTGAACGCAAGTTGCGCCCGAGGCCAAC-CGGCTAAGGGCACGTCCGCCTG---GGCGTCAA-GCATTTTATCGCTCC

Dendrobium_aduncum_KJ210409 ACGAC-TCTCGGCAATGGATATCTC-GGCTCTCGCATCGATGAAGAGCGCAGCGAAATGCGATATGTGGTGC-GAATTGCAGAATCCCGCGAACC-ATCGAGTCTTTGAACGCAAGTTGCGCCCGAGGCCAAC-CGGCTAAGGGCACGTCCGCCTG---GGCGTCAA-GCATTTTATCGCTCC

Dendrobium_aduncum_KJ210410 ACGAC-TCTCGGCAATGGATATCTC-GGCTCTCGCATCGATGAAGAGCGCAGCGAAATGCGATATGTGGTGC-GAATTGCAGAATCCCGCGAACC-ATCGAGTCTTTGAACGCAAGTTGCGCCCGAGGCCAAC-CGGCTAAGGGCACGTCCGCCTG---GGCGTCAA-GCATTTTATCGCTCC

Dendrobium_aduncum_KJ210411 ACGAC-TCTCGGCAATGGATATCTC-GGCTCTCGCATCGATGAAGAGCGCAGCGAAATGCGATATGTGGTGC-GAATTGCAGAATCCCGCGAACC-ATCGAGTCTTTGAACGCAAGTTGCGCCCGAGGCCAAC-CGGCTAAGGGCACGTCCGCCTG---GGCGTCAA-GCATTTTATCGCTCC

Dendrobium_aduncum_KJ210412 ACGAC-TCTCGGCAATGGATATCTC-GGCTCTCGCATCGATGAAGAGCGCAGCGAAATGCGATATGTGGTGC-GAATTGCAGAATCCCGCGAACC-ATCGAGTCTTTGAACGCAAGTTGCGCCCGAGGCCAAC-CGGCTAAGGGCACGTCCGCCTG---GGCGTCAA-GCATTTTATCGCTCC

Dendrobium_aduncum_KJ672619 ACGAC-TCTCGGCAATGGATATCTC-GGCTCTCGCATCGATGAAGAGCGCAGCGAAATGCGATATGTGGTGC-GAATTGCAGAATCCCGCGAACC-ATCGAGTCTTTGAACGCAAGTTGCGCCCGAGGCCAAC-CGGCTAAGGGCACGTCCGCCTG---GGCGTCAA-GCATTTTATCGCTCC

Dendrobium_aduncum_KP159298 ACGAC-TCTCGGCAATGGATATCTC-GGCTCTCGCATCGATGAAGAGCGCAGCGAAATGCGATATGTGGTGC-GAATTGCAGAATCCCGCGAACC-ATCGAGTCTTTGAACGCAAGTTGCGCCTGAGGCCAAC-CGGCTGAGGGCACGTCCGCCTG---GGCGTCAA-GCATTTTATCGCTCC

Dendrobium_aduncum_KR075042 ACGAC-TCTCGGCAATGGATATCTC-GGCTCTCGCATCGATGAAGAGCGCAGTGAAATGCGATATGTGGTGC-GAATTGCAGAATCCCGCGAACC-ATCGAGTCTTTGAACGCAAGTTGCGCCCGAGGCCAAC-CGGCTAAGGGCACGTCCGCCTG---GGCGTCAA-GCATTTTATCGCTCC

Dendrobium_aduncum_KX600499 ACGAC-TCTCGGCAATGGATATCTC-GGCTCTCGCATCGATGAAGAGCGCAGCGAAATGCGATATGTGGTGC-GAATTGCAGAATCCCGCGAACC-ATCGAGTCTTTGAACGCAAGTTGCGCCCGAGGCCAAC-CGGCTAAGGGCACGTCCGCCTG---GGCGTCAA-GCATTTTATCGCTCC

Dendrobium_anosmum_AB593499 ACGAC-TCTCGGCAATGGATATCTC-GGCTCTCGCATCGATGAAGAGCGCAGCGAAATGCGATATGTGGTGC-GAATTGCAGAATCCCGCGAACC-ATCGAGTCTTTGAACGCAAGTTGCGCCCGAGGCCAAT-CGGCCAAGGGCACGTCCGCCTG---GGCGTCAG-GCATTTTGTCGCTTC

Dendrobium_anosmum_AB972339 ACGAC-TCTCGGCAATGGATATCTC-GGCTCTCGCATCGATGAAGAGCGCAGCGAAATGCGATATATGGTGC-GAATTGCAGAATCCCGCGAACC-ATCGAGTCTTTGAACGCAAGTTGCGCCCGAGGCCAAT-CGGCCAAGGGCACGTCCGCCTG---GGCGTCAG-GCATTTTGTCGCTTC

Dendrobium_anosmum_EU477499 ACGAC-TCTCGGCAATGGATATCTC-GGCTCTCGCATCGATGAAGAGCGCAGCGAAATGCGATATATGGTGC-GAATTGCAGAATCCCGCGAACC-ATCGAGTCTTTGAACGCAAGTTGCGCCCGAGGCCAAT-CGGCCAAGGGCACGTCCGCCTG---GGCGTCAG-GCATTTTGTCGCTTC

Dendrobium_anosmum_JN388570 ACGAC-TCTCGGCAATGGATATCTC-GGCTCTCGCATCGATGAAGAGCGCAGCGAAATGCGATATGTGGTGC-GAATTGCAGAATCCCGCGAACC-ATCGAGTCTTTGAACGCAAGTTGCGCCCGAGGCCAAT-CGGCCAAGGGCACGTCCGCCTG---GGCGTCAG-GCATTTTGTCGCTTC

Dendrobium_anosmum_KJ672650 ACGAC-TCTCGGCAATGGATATCTC-GGCTCTCGCATCGATGAAGAGCGCAGCGAAATGCGATATGTGGTGC-GAATTGCAGAATCCCGCGAACC-ATCGAGTCTTTGAACGCAAGTTGCGCCCGAGGCCAAT-CGGCCAAGGGCACGTCCGCCTG---GGCGTCAG-GCATTTTGTCGCTTC

Dendrobium_anosmum_KJ944630 ACGAC-TCTCGGCAATGGATATCTC-GGCTCTCGCATCGATGAAGAGCGCAGCGAAATGCGATATATGGTGC-GAATTGCAGAATCCCGCGAACC-ATCGAGTCTTTGAACGCAAGTTGCGCCCGAGGCCAAT-CGGCCAAGGGCACGTCCGCCTG---GGCGTCAG-GCATTTTGTCGCTTC

Dendrobium_anosmum_KP743542 ACGAC-TCTCGGCAATGGATATCTC-GGCTCTCGCATCGATGAAGAGCGCAGCGAAATGCGATATGTGGTGC-GAATTGCAGAATCCCGCGAACC-ATCGAGTCTTTGAACGCAAGTTGCGCCCGAGGCCAAT-CGGCCAAGGGCACGTCCGCCTG---GGCGTCAG-GCATTTTGTCGCTTC

Dendrobium_anosmum_KP743544 ACGAC-TCTCGGCAATGGATATCTC-GGCTCTCGCATCGATGAAGAGCGCAGCGAAATGCGATATATGGTGC-GAATTGCAGAATCCCGCGAACC-ATCGAGTCTTTGAACGCAAGTTGCGCCCGAGGCCAAT-CGGCCAAGGGCACGTCCGCCTG---GGCGTCAG-GCATTTTGTCGCTTC

Dendrobium_anosmum_KY966516 ACGAC-TCTCGGCAATGGATATCTC-GGCTCTCGCATCGATGAAGAGCGCAGCGAAATGCGATATGTGGTGC-GAATTGCAGAATCCCGCGAACC-ATCGAGTCTTTGAACGCAAGTTGCGCCCGAGGCCAAT-CGGCCAAGGGCACGTCCGCCTG---GGCGTCAG-GCATTTTGTCGCTTC

Dendrobium_cf_mimicum_PK12237E ACGAC-TCTCGGCAATGGATATCTC-GGCTCTTGCATCGATGAAGAGCGCAGCGAAATGCGATACGTGGTGC-GAATTGCAGAATCCCGCGAACC-ATCGAGTCTTTGAACGCAAGTTGCGCCCGAGGCCAAC-CGGCCAAGGGCACGTCCGCCTG---GGCGTCAA-GCGTTGCGTCGCTCC

Dendrobium_cf_mimicum_PK12237J ACGAC-TCTCGGCAATGGATATCTC-GGCTCTTGCATCGATGAAGAGCGCAGCGAAATGCGATACGTGGTGC-GAATTGCAGAATCCCGCGAACC-ATCGAGTCTTTGAACGCAAGTTGCGCCCGAGGCCAAC-CGGCCAAGGGCACGTCCGCCTG---GGCGTCAA-GCGTTGCGTCGCTCC

Dendrobium_crumenatum_AB593537 AGGAC-TCTCGACAATGGATATCTC-GGCTCTTGCATCGATGAAGAGCGCAGCGAAATGCGATACGTGGTGC-GAATTGCAGAATCCCGCGAACC-ATCGAGTCTTTGAACGCAAGTTGCGCCCGAGGCCAAT-CGGCCAAGGGCACGTCTGCCTG---GGCGTCAA-GCATTATGTCACTCC

Dendrobium_crumenatum_AB972336 AGGAC-TCTCGACAATGGATATCTC-GGCTCTTGCATCGATGAAGAGCGCAGCGAAATGCGATACGTGGTGC-GAATTGCAGAATCCCGCGAACC-ATCGAGTCTTTGAACGCAAGTTGCGCCCGAGGCCAAT-CGGCCAAGGGCACGTCTGCCTG---GGCGTCAA-GCATTATGTCACTCC

Dendrobium_crumenatum_AF521608 AGGAC-TCTCGACAATGGATATCTC-GGCTCTTGCATCGATGAAGAGCGCAGCGAAATGCGATACGTGGTGC-GAATTGCAGAATCCCGCGAACC-ATCGAGTCTTTGAACGCAAGTTGCGCCCGAGGCCAAT-CGGCCAAGGGCACGTCTGCCTG---GGCGTCAA-GCATTATGTCACTCC

Dendrobium_crumenatum_AY239963 AGGAC-TCTCGACAATGGATATCTC-GGCTCTTGCATCGATGAAGAGCGCAGCGAAATGCGATACGTGGTGC-GAATTGCAGAATCCCGCGAACC-ATCGAGTCTTTGAACGCAAGTTGCGCCCGAGGCCAAT-CGGCCAAGGGCACGTCTGCCTG---GGCGTCAA-GCATTATGTCACTCC

Dendrobium_crumenatum_AY273708 AGGAC-TCTCGACAATGAATATCTC-GGCTCTTGCATCGATGAAGAGCGCAGCGAAATGCGATACGTGGTGC-GAATTGCAGAATCCCGCGAACC-ATCGAGTCTTTGAACGCAAGTTGCGCCCGAGGCCAAT-CGGCCAAGGGCACGTTTGCCTG---GGCGTCAA-GCATTATGTCACTCC

Dendrobium_crumenatum_EU840700 AGGAC-TCTCGACAATGGATATCTC-GGCTCTTGCATCGATGAAGAGCGCAGCGAAATGCGATACATGGTGC-GAATTGCAGAATCCCGCGAACC-ATCGAGTCTTTGAACGCAAGTTGCGCCCCAGGCCAAT-CGGCCAAGGGCACGTTTGCCTG---GGCGTCAA-GCATTATGTCACTCC

Dendrobium_crumenatum_HM054625 AGGAC-TCTCGACAATGGATATCTC-GGCTCTTGCATCGATGAAGAGCGCAGCGAAATGCGATACATGGTGC-GAATTGCAGAATCCCGCGAACC-ATCGAGTCTTTGAACGCAAGTTGCGCCCGAGGCCAAT-CGGCCAAGGGCACGTCTGCCTG---GGCGTCAA-GCATTATGTCACTCC

Dendrobium_crumenatum_HM590370 AGGAC-TCTCGACAATGGATATCTC-GGCTCTTGCATCGATGAAGAGCGCAGCGAAATGCGATACATGGTGC-GAATTGCAGAATCCCGCGAACC-ATCGAGTCTTTGAACGCAAGTTGCGCCCGAGGCCAAT-CGGCCAAGGGCACGTCTGCCTG---GGCGTCAA-GCATTATGTCACTCC

Dendrobium_crumenatum_JF713095 AGGAC-TCTCGACAATGGATATCTC-GGCTCTTGCATCGATGAAGAGCGCAGCGAAATGCGATACATGGTGC-GAATTGCAGAATCCCGCGAACC-ATCGAGTCTTTGAACGCAAGTTGCGCCCGAGGCCAAT-CGGCCAAGGGCACGTCTGCCTG---GGCGTCAA-GCATTATGTCACTCC

Dendrobium_crumenatum_JF713096 AGGAC-TCTCGACAATGGATATCTC-GGCTCTTGCATCGATGAAGAGCGCAGCGAAATGCGATACATGGTGC-GAATTGCAGAATCCCGCGAACC-ATCGAGTCTTTGAACGCAAGTTGCGCCCGAGGCCAAT-CGGCCAAGGGCACGTCTGCCTG---GGCGTCAA-GCATTATGTCACTCC

Dendrobium_crumenatum_JN388587 AGGAC-TCTCGACAATGGATATCTC-GGCTCTTGCATCGATGAAGAGCGCAGCGAAATGCGATACATGGTGC-GAATTGCAGAATCCCGCGAACC-ATCGAGTCTTTGAACGCAAGTTGCGCCCGAGGCCAAT-CGGCCAAGGGCACGTCTGCCTG---GGCGTCAA-GCATTATGTCACTCC

Dendrobium_crumenatum_KC507780 AGGAC-TCTCGACAATGGATATCTC-GGCTCTTGCATCGATGAAGAGCGCAGCGAAATGCGATACGTGGTGC-GAATTGCAGAATCCCGCGAACC-ATCGAGTCTTTGAACGCAAGTTGCGCCCGAGGCCAAT-CGGCCAAGGGCACGTCTGCCTG---GGCGA----GC---ATGTCACTCC

Dendrobium_crumenatum_KJ672661 AGGAC-TCTCGACAATGGATATCTC-GGCTCTTGCATCGATGAAGAGCGCAGCGAAATGCGATACATGGTGC-GAATTGCAGAATCCCGCGAACC-ATCGAGTCTTTGAACGCAAGTTGCGCCCCAGGCCAAT-CGGCCAAGGGCACGTTTGCCTG---GGCGTCAA-GCATTATGTCACTCC

Dendrobium_linawianum_AB593599 ACGAC-TCTCGGCAATGGATATCTT-GGCTCTCGCATCGATGAAGAGCGCAGCGAAATGCGATATGTGGTGC-GAATTGCAGAATCCCGCGAACC-ATCGAGTCTTTGAACGCAAGTTGCGCCTGAGGCCAAC-CGGCTGAGGGCACGTCCGCCTG---GGCGTCAA-GCATTTTATCGCTCC

Dendrobium_linawianum_AF401487 -CCAA-TTTTGGGAATGGATTTTTTGGGTTTTCCCTTCAATAAAAACCCCACCAAATGGCAATTTGGGGGGC-AAATGGCAAATTCCCCCAACCC-TTCAATTTTTTAACCCCAAGTTGCCCTTAAGGCCACC-TGGTTAAGGCCCCTTCCCCTTG---GCCTTAAA-CCCTTTATTCATTCT

Dendrobium_linawianum_AF521613 ACGAC-TCTCGGCAATGGATATCTT-GGCTCTCGCATCGATGAAGAGCGCAGCGAAATGCGATATGTGGTGC-GAATTGCAGAATCCCGCGAACC-ATCGAGTCTTTGAACGCAAGTTGCGCCTGAGGCCAAC-CGGCTGAGGGCACGTCCGCCTG---GGCGTCAA-GCATTTTATCGCTCC

Dendrobium_linawianum_EU003115 ACGAC-TCTCGGCAATGGATATCTT-GGCTCTCGCATCGATGAAGAGCGCAGCGAAATGCGATATGTGGTGC-GAATTGCAGAATCCCGCGAACC-ATCGAGTCTTTGAACGCAAGTTGCGCCTGAGGCCAAC-CGGCTGAGGGCACGTCCGCCTG---GGCGTCAA-GCATTTTATCGCTCC

Dendrobium_linawianum_EU003117 ACGAC-TCTCGGCAATGGATATCTT-GGCTCTCGCATCGATGAAGAGCGCAGCGAAATGCGATATGTGGTGC-GAATTGCAGAATCCCGCGAACC-ATCGAGTCTTTGAACGCAAGTTGCGCCTGAGGCCAAC-CGGCTGAGGGCACGTCCGCCTG---GGCGTCAA-GCATTTTATCGCTCC

Dendrobium_linawianum_HM590371 ACGAC-TCTCGGCAATGGATATCTT-GGCTCTCGCATCGATGAAGAGCGCAGCGAAATGCGATATGTGGTGC-GAATTGCAGAATCCCGCGAACC-ATCGAGTCTTTGAACGCAAGTTGCGCCTGAGGCCAAC-CGGCTGAGGGCACGTCCGCCTG---GGCGTCAA-GCATTTTATCGCTCC

Dendrobium_linawianum_JN388573 ACGAC-TCTCGGCAATGGATATCTC-GGCTCTCGCATCGATGAAGAGCGCAGCGAAATGCGATATGTGGTGC-GAATTGCAGAATCCCGCGAACC-ATCGAGTCTTTGAACGCAAGTTGCGCCTGAGGCCAAC-CGGCTGAGGGCACGTCCGCCTG---GGCGTCAA-GCATTTTATCGCTCC

Dendrobium_linawianum_KJ672629 ACGAC-TCTCGGCAATGGATATCTT-GGCTCTCGCATCGATGAAGAGCGCAGCGAAATGCGATATGTGGTGC-GAATTGCAGAATCCCGCGAACC-ATCGAGTCTTTGAACGCAAGTTGCGCCTGAGGCCAAC-CGGCTGAGGGCACGTCCGCCTG---GGCGTCAA-GCATTTTATCGCTCC

Dendrobium_linawianum_KP159299 ACGAC-TCTCGGCAATGGATATCTT-GGCTCTCGCATCGATGAAGAGCGCAGCGAAATGCGATATGTGGTGC-GAATTGCAGAATCCCGCGAACC-ATCGAGTCTTTGAACGCAAGTTGCGCCTGAGGCCAAC-CGGCTGAGGGCACGTCCGCCTG---GGCGTCAA-GCATTTTATCGCTCC

Dendrobium_linawianum_KT779776 ACGAC-TCTCGGCAATGGATATCTC-GGCTCTCGCATCGATGAAGAGCGCAGCGAAATGCGATATGTGGTGC-GAATTGCAGAATCCCGCGAACC-ATCGAGTCTTTGAACGCAAGTTGCGCCTGAGGCCAAC-CGGCTGAGGGCACGTCCGCCTG---GGCGTCAA-GCATTTTATCGCTCC

Dendrobium_linawianum_KY966557 ACGAC-TCTCGGCAATGGATATCTT-GGCTCTCGCATCGATGAAGAGCGCAGCGAAATGCGATATGTGGTGC-GAATTGCAGAATCCCGCGAACC-ATCGAGTCTTTGAACGCAAGTTGCGCCTGAGGCCAAC-CGGCTGAGGGCACGTCCGCCTG---GGCGTCAA-GCATTTTATCGCTCC

Dendrobium_linawianum_SG1347 ACGAC-TCTCGGCAATGGATATCTT-GGCTCTCGCATCGATGAAGAGCGCAGCGAAATGCGATATGTGGTGC-GAATTGCAGAATCCCGCGAACC-ATCGAGTCTTTGAACGCAAGTTGCGCCTGAGGCCAAC-CGGCTGAGGGCACGTCCGCCTG---GGCGTCAA-GCATTTTATCGCTCC

Dendrobium_lindleyi_AB593600 ACGAC-TCTCGGCAATGGATATCTC-GGCTCTCGCATCGATGAAGAGCGCAGCGAAATGCGATACGTGGTGC-GAATTGCAGAATCCCGCGAACC-ATCGAGTCTTTGAACGCAAGTTGCGCCCGAGGCCAAT-CGGCCAAGGGCACGTTCGCCTG---GGCGTCAA-GCATTACGTTGCTCC

Dendrobium_lindleyi_AB972341 ACGAC-TCTCGGCAATGGATATCTC-GGCTCTCGCATCGATGAAGAGCGCAGCGAAATGCGATACGTGGTGC-GAATTGCAGAATCCCGCGAACC-ATCGAGTCTTTGAACGCAAGTTGCGCCCGAGGCCAAT-CGGCCAAGGGCACGTTCGCCTG---GGCGTCAA-GCATTACGTTGCTCC

Dendrobium_lindleyi_DQ058784 ACGAC-TCTCGGCAATGGATATCTC-GGCTCTCGCATCGATGAAGAGCGCAGCGAAATGCGATACGTGGTGC-GAATTGCAGAATCCCGCGAACC-ATCGAGTCTTTGAACGCAAGTTGCGCCCGAGGCCAAT-CGGCCAAGGGCACGTTCGCCTG---GGCGTCAA-GCATTATGACGCTCC

Dendrobium_lindleyi_GU339114 ACGAC-TCTCGGCAATGGATATCTC-GGCTCTCGCATCGATGAAGAGCGCAGCGAAATGTGATACGTGGTGC-GAATTGCAGAATCCCGCGAACCCATCGAGTCTTTGAACGCAAGTTGCGCCCGAGGCCAAT-CGGCCAAGGGCACGTTCGCCTG---GGCGTCAA-GCATTATGACGCTCC

Dendrobium_lindleyi_HM054672 ACGAC-TCTCGGCAATGGATATCTC-GGCTCTTGCATCGATGAAGAGCGCAGCGAAATGCGATACGTGGTGC-GAATTGCAGAATCCCGCGAACC-ATCGAGTCTTTGAACGCAAGTTGCGCCCGAGGCCAAT-CGGCCAAGGGCACGTTCGCCTG---GGCGTCAA-GCATTATGTTGCTCC

Dendrobium_lindleyi_HM054673 ACGAC-TCTCGGCAATGGATATCTC-GGCTCTTGCATCGATGAAGAGCGCAGCGAAATGCGATACGTGGTGC-GAATTGCAGAATCCCGCGAACC-ATCGAGTCTTTGAACGCAAGTTGCGCCCGAGGCCAAT-CGGCCAAGGGCACGTTCGCCTG---GGCGTCAA-GCATTATGTTGCTCC

Dendrobium_lindleyi_JF713110 ACGAC-TCTCGGCAATGGATATCTC-GGCTCTTGCATCGATGAAGAGCGCAGCGAAATGCGATACGTGGTGC-GAATTGCAGAATCCCGCGAACC-ATCGAGTCTTTGAACGCAAGTTGCGCCCGAGGCCAAT-CGGCCAAGGGCACGTTCGCCTG---GGCGTCAA-GCATTATGTTGCTCC

Dendrobium_lindleyi_JN388568 ACGAC-TCTCGGCAATGGATATCTC-GGCTCTCGCATCGATGAAGAGCGCAGCGAAATGCGATACGTGGTGC-GAATTGCAGAATCCCGCGAACC-ATCGAGTCTTTGAACGCAAGTTGCGCCCGAGGCCAAT-CGGCCAAGGGCACGTTCGCCTG---GGCGTCAA-GCATTATGACGCTCC

Dendrobium_lindleyi_KC568301 ACGAC-TCTCGGCAATGGATATCTC-GGCTCTCGCATCGATGAAGAGCGCAGCGAAATGCGATACGTGGTGC-GAATTGCAAAATCCCGCGAACC-ATCGAGTCTTTGAACGCAAGTTGCGCCCGAGGCCAAT-CGGCCAAGGGCACGTTCGCCTG---GGCGTCAA-GCATTATGACGCTCC

Dendrobium_lindleyi_KFBG203 ACGAC-TCTCGGCAATGGATATCTC-GGCTCTCGCATCGATGAAGAGCGCAGCGAAATGCGATACGTGGTGC-GAATTGCAGAATCCCGCGAACC-ATCGAGTCTTTGAACGCAAGTTGCGCCCGAGGCCAAT-CGGCCAAGGGCACGTTCGCCTG---GGCGTCAA-GCATTATGACGCTCC

Dendrobium_lindleyi_KJ210461 ACGAC-TCTCGGCAATGGATATCTC-GGCTCTCGCATCGATGAAGAGCGCAGCGAAATGCGATACGTGGTGC-GAATTGCAGAATCCCGCGAACC-ATCGAGTCTTTGAACGCAAGTTGCGCCCGAGGCCAAT-CGGCCAAGGGCACGTTCGCCTG---GGCGTCAA-GCATTATGACGCTCC

Dendrobium_lindleyi_KJ672682 ACGAC-TCTCGGCAATGGATATCTC-GGCTCTCGCATCGATGAAGAGCGCAGCGAAATGCGATACGTGGTGC-GAATTGCAGAATCCCGCGAACC-ATCGAGTCTTTGAACGCAAGTTGCGCCCGAGGCCAAT-CGGCCAAGGGCACGTTCGCCTG---GGCGTCAA-GCATTATGACGCTCC

Dendrobium_lindleyi_KX522640 ACGAC-TCTCGGCAATGGATATCTC-GGCTCTTGCATCGATGAAGAGCGCAGCGAAATGCGATACGTGGTGC-GAATTGCAGAATCCCGCGAACC-ATCGAGTCTTTGAACGCAAGTTGCGCCCGAGGCCAAT-CGGCCAAGGGCACGTTCGCCTG---GGCGTCAA-GCATTATGTTGCTCC

Dendrobium_lindleyi_KY966558 ACGAC-TCTCGGCAATGGATATCTC-GGCTCTCGCATCGATGAAGAGCGCAGCGAAATGCGATACGTGGTGC-GAATTGCAGAATCCCGCGAACC-ATCGAGTCTTTGAACGCAAGTTGCGCCCGAGGCCAAT-CGGCCAAGGGCACGTTCGCCTG---GGCGTCAA-GCATTATGACGCTCC

Dendrobium_lindleyi_KY966559 ACGAC-TCTCGGCAATGGATATCTC-GGCTCTCGCATCGATGAAGAGCGCAGCGAAATGCGATACGTGGTGC-GAATTGCAGAATCCCGCGAACC-ATCGAGTCTTTGAACGCAAGTTGCGCCCGAGGCCAAT-CGGCCAAGGGCACGTTCGCCTG---GGCGTCAA-GCATTATGACGCTCC

Dendrobium_lindleyi_KY966560 ACGAC-TCTCGGCAATGGATATCTC-GGCTCTCGCATCGATGAAGAGCGCAGCGAAATGCGATACGTGGTGC-GAATTGCAGAATCCCGCGAACC-ATCGAGTCTTTGAACGCAAGTTGCGCCCGAGGCCAAT-CGGCCAAGGGCACGTTCGCCTG---GGCGTCAA-GCATTACGTTGCTCC

Dendrobium_loddigesii_AB593604 ATGAC-TCTCGGCAATGGATATCTC-GGCTCTCGCATCGATGAAGAGCGCAGCGAAATGCGATATGTGGTGC-GAATTGCAGAATCCCGCGAACC-ATCGAGTCTTTGAACGCAAGTTGCGCCCGAGGCCAAC-CGGCCAAGGGCACGTCCGCCTG---GGCGTCAG-TCATTTTATCGCTCT

Dendrobium_loddigesii_AB873183 ATGAC-TCTCGGCAATGGATATCTC-GGCTCTCGCATCGATGAAGAGCGCAGCGAAATGCGATATGTGGTGC-GAATTGCAGAATCCCGCGAACC-ATCGAGTCTTTGAACGCAAGTTGCGCCCGAGGCCAAC-CGGCCAAGGGCACGTCCGCCTG---GGCGTCAG-TCATTTTATCGCTCT

Dendrobium_loddigesii_AF311778 ATGAC-TCTCGGCAATGGATATCTC-GGCTCTCGCATCGATGAAGAGCGCAGCGAAATGCGATATGTGGTGC-GAATTGCAGAATCCCGCGAACC-ATCGAGTCTTTGAACGCAAGTTGCGCCCGAGGCCAAC-CGGCCAAGGGCACGTCCGCCTG---GGCGTCAG-TCATTTTATCGCTCT

Dendrobium_loddigesii_AY485703 ATGACCTCTCGGCAATGGATATCTC-GGCTCTCGCATCGATGAAGAGCGCAGCGAAATGCGATATGTGGTGC-GAATTGCAGAATCCCGCGAACC-ATCGAGTCTTTGAACGCAAGTTGCGCCCGAGGCCAAC-CGGCCAAGGGCACGTCCGCCTG---GGCGTCAA-TCATTTTTTCGCTCT

Dendrobium_loddigesii_EU121418 ATGAC-TCTCGGCAATGGATATCTC-GGCTCTCGCATCGATGAAGAGCGCAGCGAAATGCGATATGTGGTGC-GAATTGCAGAATCCCGCGAACC-ATCGAGTCTTTGAACGCAAGTTGCGCCCGAGGCCAAC-CGGCCAAGGGCACGTCCGCCTG---GGCGTCAG-TCATTTTATCGCTCT

Dendrobium_loddigesii_EU592016 ACGAC-TCTCGGCAATGGATATCTC-GGCTCTCGCATCGATGAAGAGCGCAGCGAAATGCGATATGTGGTGC-GAATTGCAGAATCCCGCGAACC-ATCGAGTCTTTGAACGCAAGTTGCGCCTGAGGCCAAC-CGGCTGAGGGCACGTCCGCCTG---GGCGTCAA-GCATTTTATCGCTCT

Dendrobium_loddigesii_HM590374 ATGAC-TCTCGGCAATGGATATCTC-GGCTCTCGCATCGATGAAGAGCGCAGCGAAATGCGATATGTGGTGC-GAATTGCAGAATCCCGCGAACC-ATCGAGTCTTTGAACGCAAGTTGCGCCCGAGGCCAAC-CGGCCAAGGGCACGTCCGCCTG---GGCGTCAG-TCATTTTATCGCTCT

Dendrobium_loddigesii_HQ114220 ATGAC-TCTCGGCAATGGATATCTC-GGCTCTCGCATCGATGAAGAGCGCAGCGAAATGCGATATGTGGTGC-GAATTGCAGAATCCCGCGAACC-ATCGAGTCTTTGAACGCAAGTTGCGCCCGAGGCCAAC-CGGCCAAGGGCACGTCCGCCTG---GGCGTCAG-TCATTTTATCGCTCT

Dendrobium_loddigesii_JN388569 ATGAC-TCTCGGCAATGGATATCTC-GGCTCTCGCATCGATGAAGAGCGCAGCGAAATGCGATATGTGGTGC-GAATTGCAGAATCCCGCGAACC-ATCGAGTCTTTGAACGCAAGTTGCGCCCGAGGCCAAC-CGGCCAAGGGCACGTCCGCCTG---GGCGTCAG-TCATTTTATCGCTCT

Dendrobium_loddigesii_KC205187 ATGAC-TCTCGGCAATGGATATCTC-GGCTCTCGCATCGATGAAGAGCGCAGCGAAATGCGATATGTGGTGC-GAATTGCAGAATCCCGCGAACC-ATCGAGTCTTTGAACGCAAGTTGCGCCCGAGGCCAAC-CGGCCAAGGGCACGTCCGCCTG---GGCGTCAG-TCATTTTATCGCTCT

Dendrobium_loddigesii_KC346890 ACGAC-TCTCGGCAATGGATATCTC-GGCTCTCGCATCGATGAAGAGCGCAGCGAAATGCGATATGTGGTGC-GAATTGCAGAATCCCGCGAACC-ATCGAGTCTTTGAACGCAAGTTGCGCCCGAGGCCAAT-CGGCCAAGGGCACGCCTGCCTG---GGTGTCAA-GCATTTTATCGCTCC

Dendrobium_loddigesii_KF143481 ATGAC-TCTCGGCAATGGATATCTC-GGCTCTCGCATCGATGAAGAGCGCAGCGAAATGCGATATGTGGTGC-GAATTGCAGAATCCCGCGAACC-ATCGAGTCTTTGAACGCAAGTTGCGCCCGAGGCCAAC-CGGCCAAGGGCACGTCCGCCTG---GGCGTCAG-TCATTTTATCGCTCT

Dendrobium_loddigesii_KJ672683 ATGAC-TCTCGGCAATGGATATCTC-GGCTCTCGCATCGATGAAGAGCGCAGCGAAATGCGATATGTGGTGC-GAATTGCAGAATCCCGCGAACC-ATCGAGTCTTTGAACGCAAGTTGCGCCCGAGGCCAAC-CGGCCAAGGGCACGTCCGCCTG---GGCGTCAA-TCATTTTTTCGCTCT

Dendrobium_loddigesii_KP159301 ATGAC-TCTCGGCAATGGATATCTC-GGCTCTCGCATCGATGAAGAGCGCAGCGAAATGCGATATGTGGTGC-GAATTGCAGAATCCCGCGAACC-ATCGAGTCTTTGAACGCAAGTTGCGCCCGAGGCCAAC-CGGCCAAGGGCACGTCCGCCTG---GGCGTCAG-TCATTTTATCGCTCT

Dendrobium_loddigesii_KP264997 ATGAC-TCTCGGCAATGGATATCTC-GGCTCTCGCATCGATGAAGAGCGCAGCGAAATGCGATATGTGGTGC-GAATTGCAGAATCCCGCGAACC-ATCGAGTCTTTGAACGCAAGTTGCGCCCGAGGCCAAC-CGGCCAAGGGCACGTCCGCCTG---GGCGTCAG-TCATTTTATCGCTCT

Dendrobium_loddigesii_KT778746 ATGAC-TCTCGGCAATGGATATCTC-GGCTCTCGCATCGATGAAGAGCGCAGCGAAATGCGATATGTGGTGC-GAATTGCAGAATCCCGCGAACC-ATCGAGTCTTTGAACGCAAGTTGCGCCCGAGGCCAAC-CGGCCAAGGGCACGTCCGCCTG---GGCGTCAG-TCATTTTATCGCTCT

Dendrobium_loddigesii_KY966562 ATGAC-TCTCGGCAATGGATATCTC-GGCTCTCGCATCGATGAAGAGCGCAGCGAAATGCGATATGTGGTGC-GAATTGCAGAATCCCGCGAACC-ATCGAGTCTTTGAACGCAAGTTGCGCCCGAGGCCAAC-CGGCCAAGGGCACGTCCGCCTG---GGCGTCAG-TCATTTTATCGCTCT

Dendrobium_loddigesii_SG1255 ATGAC-TCTCGGCAATGGATATCTC-GGCTCTCGCATCGATGAAGAGCGCAGCGAAATGCGATATGTGGTGC-GAATTGCAGAATCCCGCGAACC-ATCGAGTCTTTGAACGCAAGTTGCGCCCGAGGCCAAC-CGGCCAAGGGCACGTCCGCCTG---GGCGTCAG-TCATTTTATCGCTCT

Dendrobium_spatella_AB847671 ATGAC-TCTCGACAATGGATATCTC-GGCTCTTGCATCGATGAAGAGCGCAGCGAAATGCGATACGTGGTGC-GAATTGCAGAATCCCGCGAACC-ATCGAGTCTTTGAACGCAAGTTGCGCCCGAGGCCAAC-CGGCCAAGGGCACGTCCGCCTG---GGCGTCAA-GCATTACGTCACTCC

Dendrobium_spatella_KF143512 ATGAC-TCTCGACAATGGATATCTC-GGCTCTTGCATCGATGAAGAGCGCAGCGAAATGCGATACGTGGTGC-GAATTGCAGAATCCCGCGAACC-ATCGAGTCTTTGAACGCAAGTTGCGCCCGAGGCCAAC-CGGCCAAGGGCACGTCCGCCTG---GGCGTCAA-GCATTACGTCACTCC

Dendrobium_spatella_KF143513 ATGAC-TCTCGACAATGGATATCTC-GGCTCTTGCATCGATGAAGAGCGCAGCGAAATGCGATACGTGGTGC-GAATTGCAGAATCCCGCGAACC-ATCGAGTCTTTGAACGCAAGTTGCGCCCGAGGCCAAC-CGGCCAAGGGCACGTCCGCCTG---GGCGTCAA-GCATTACGTCACTCC

Dendrobium_spatella_SG1357 ATGAC-TCTCGACAATGGATATCTA-GGCTCTTGCATCGATGAAGAGCGCAGCGAAATGCGATACGTGGTGC-GAATTGCAGAATCCCGCGAACC-ATCGAGTCTTTGAACGCAAGTTGCGCCCGAGGCCAAC-CGGCCAAGGGCACGTCCGCCTG---GGCGTCAA-GCATTACGTCACTCC

Dendrobium_spatella_SG1358 ATGAC-TCTCGACAATGGATATCTC-GGCTCTTGCATCGATGAAGAGCGCAGCGAAATGCGATACGTGGTGC-GAATTGCAGAATCCCGCGAACC-ATCGAGTCTTTGAACGCAAGTTGCGCCCGAGGCCAAC-CGGCCAAGGGCACGTCCGCCTG---GGCGTCAA-GCATTACGTCACTCC

Dendrolirium_lasiopetalum_KFBG701 ACGAC-TCTCGGCAATGGATATCTC-GGCTCTCGCATCGATGAAGAGCGCAGCGAAATGCGATACGTGGTGT-GAATTGCAGAATCCCGCGAACC-ATCGAGTCTTTGAACGCAAGTTGCGCCCGAGGCCAAC-CGGTTGAGGGCACGTCTGCCTG---GGCGTCAA-GCGTTACGTCGCTCC

Dendrolirium_lasiopetalum_PK12173 ACGAC-TCTCGGCAATGGATATCTC-GGCTCTCGCATCGATGAAGAGCGCAGCGAAATGCGATACGTGGTGT-GAATTGCAGAATCCCGCGAACC-ATCGAGTCTTTGAACGCAAGTTGCGCCCGAGGCCAAC-CGGTTGAGGGCACGTCTGCCTG---GGCGTCAA-GCGTTACGTCGCTCC

Dendrolirium_lasiopetalum_PK12174 ACGAC-TCTCGGCAATGGATATCTC-GGCTCTCGCATCGATGAAGAGCGCAGCGAAATGCGATACGTGGTGT-GAATTGCAGAATCCCGCGAACC-ATCGAGTCTTTGAACGCAAGTTGCGCCCGAGGCCAAC-CGGTTGAGGGCACGTCTGCCTG---GGCGTCAA-GCGTTACGTCGCTCC

Dendrolirium_lasiopetalum_SG1312 ACGAC-TCTCGGCAATGGATATCTC-GGCTCTCGCATCGATGAAGAGCGCAGCGAAATGCGATACGTGGTGT-GAATTGCAGAATCCCGCGAACC-ATCGAGTCTTTGAACGCAAGTTGCGCCCGAGGCCAAC-CGGTTGAGGGCACGTCTGCCTG---GGCGTCAA-GCGTTACGTCGCTCC

Didymoplexiella_siamensis_PK12024 ATGAC-TCTCGGCAATGGATATCTC-GGCTCTCGCATCGATGAAGAGCGCAGTGAAATGCGATACGTGGTGC-GAATTGCAGAATCCCGCGAACC-ATCGAGTCTTTGAACGCAAGTTGCGCCCGAGGCCAAT-CGGCCGAGGGCACGCCCGCCTG---GGCGACACAGCATTGCTTCGCTCC

Didymoplexiella_siamensis_SG1242 ATGAC-TCTCGGCAATGGATATCTC-GGCTCTCGCATCGATGAAGAGCGCAGTGAAATGCGATACGTGGTGC-GAATTGCAGAATCCCGCGAACC-ATCGAGTCTTTGAACGCAAGTTGCGCCCGAGGCCAAT-CGGCCGAGGGCACGCCCGCCTG---GGCGACACAGCATTGCTTCGCTCC

Didymoplexiella_siamensis_SG1243 ACGAC-TCTCGGCAATGGATATCTC-GGCTCTCGCATCGATGAAGAGCGCAGCGAAATGCGATACGTGGTGC-GAATTGCAGAATCCCGCGAACC-ATCGAGTCTTTGAACGCAAGTTGCGCCTGAGGTCAGC-TGGCCGAGGGCACGTCCGCCTG---GGCGTCAA-GCTTCGCGTCGCTCC

Dienia_ophrydis_AY907111 ATGAC-TCTCGGCAATGGATATCTC-GGCTCTTGCATCGATGAAGAGCGCAGCAAAATGCGATACGTGATGC-GAATTGCAGAATCCCGCGAACC-ATCGAGTATTTGAACGCAAGTTGCGCCCGAGGCCAAC-CGGTCAAGGGCACGTTTACCTG---GGTGTCAA-GCGTTGCTTCGCTTC

Dienia_ophrydis_AY907114 ATGAC-TCTCGGCAATGGATATCTC-GGCTCTTGCATCGATGAAGAGCGCAGCAAAATGCGATAC---ATGC-GAATTGCAGAATCCCGCGAACC-ATCGAGTATTTGAACGCAAGTTGCGCCCGAGGCCAAC-CGGTCAAGGGCACGTTTACCTG---GGTGTCAA-GCGTTGCTTCGCTTC

Dienia_ophrydis_KFBG7819 ATGAC-TCTCGGCAATGGATATCTC-GGCTCTTGCATCGATGAAGAGCGCAGCAAAATGCGATACGTGATGC-GAATTGCAGAATCCCGCGAACC-ATCGAGTATTTGAACGCAAGTTGCGCCCGAGGCCAAC-CGGTCAAGGGCACGTTTACCTG---GGTGTCAA-GCGTTGCTTCGCTTC

Dienia_ophrydis_KJ459275 ATGAC-TCTCGGCAATGGATATCTC-GGCTCTTGCATCGATGAAGAGCGCAGCAAAATGCGATACGTGATGC-GAATTGCAGAATCCCGCGAACC-ATCGAGTATTTGAACGCAAGTTGCGCCCGAGGCCAAC-CGGTCAAGGGCACGTTTACCTG---GGTGTCAA-GCGTTGCTTCGCTTC

Dienia_ophrydis_MF287970 ATGAC-TCTCGGCAATGGATATCTC-GGCTCTTGCATCGATGAAGAGCGCAGCAAAATGCGATACGTGATGC-GAATTGCAGAATCCCGCGAACC-ATCGAGTATTTGAACGCAAGTTGCGCCCGAGGCCAAC-CGGTCAAGGGCACGTTTACCTG---GGTGTCAA-GCGTTGCTTCGCTTC

Dienia_ophrydis_SG1201 ATGAC-TCTCGGCAATGGATATCTC-GGCTCTTGCATCGATGAAGAGCGCAGCAAAATGCGATACGTGATGC-GAATTGCAGAATCCCGCGAACC-ATCGAGTATTTGAACGCAAGTTGCGCCCGAGGCCAAC-CGGTCAAGGGCACGTTTACCTG---GGTGTCAA-GCGTTGCTTCGCTTC

Dienia_ophrydis_SG1254 ATGAC-TCTCGGCAATGGATATCTC-GGCTCTTGCATCGATGAAGAGCGCAGCAAAATGCGATACGTGATGC-GAATTGCAGAATCCCGCGAACC-ATCGAGTATTTGAACGCAAGTTGCGCCCGAGGCCAAC-CGGTCAAGGGCACGTTTACCTG---GGTGTCAA-GCGTTGCTTCGCTTC

Dienia_ophrydis_SG1276 ATGAC-TCTCGGCAATGGATATCTC-GGCTCTTGCATCGATGAAGAGCGCAGCAAAATGCGATACGTGATGC-GAATTGCAGAATCCCGCGAACC-ATCGAGTATTTGAACGCAAGTTGCGCCCGAGGCCAAC-CGGTCAAGGGCACGTTTACCTG---GGTGTCAA-GCGTTGCTTCGCTTC

Diploprora_championii_KFBG628 ACGAC-TCTCGACAATGGATATCTC-GGCTCTCGCATCGATGAAGAGCGCAGCGAAATGCGATACGTGGTGC-GAATTGCAGAATCCCGCGAACC-ATCGAGTCTTTGAACGCAAGTTGCGCCCGAGGCCAAT-CGGTCGAGGGCACGTCCGCCTG---GGCGTCAA-GCGTTGCGCCGCTCC

Diploprora_championii_KJ733410 ACGAC-TCTCGACAATGGATATCTC-GGCTCTCGCATCGATGAAGAGCGCAGCGAAATGCGATACGTGGTGC-GAATTGCAGAATCCCGCGAACC-ATCGAGTCTTTGAACGCAAGTTGCGCCCGAGGCCAAT-CGGTCGAGGGCACGTCCGCCTG---GGCGTCAA-GCGTTGCGCCGCTCC

Diploprora_championii_KY966590 ACGAC-TCTCGACAATGGATATCTC-GGCTCTCGCATCGATGAAGAGCGCAGCGAAATGCGATACGTGGTGC-GAATTGCAGAATCCCGCGAACC-ATCGAGTCTTTGAACGCAAGTTGCGCCCGAGGCCAAT-CGGTCGAGGGCACGTCCGCCTG---GGCGTCAA-GCGTTGCGCCGCTCC

Diploprora_championii_KY966591 ACGAC-TCTCGACAATGGATATCTC-GGCTCTCGCATCGATGAAGAGCGCAGCGAAATGCGATACGTGGTGC-GAATTGCAGAATCCCGCGAACC-ATCGAGTCTTTGAACGCAAGTTGCGCCCGAGGCCAAT-CGGTCGAGGGCACGTCCGCCTG---GGCGTCAA-GCGTTGCGCCGCTCC

Diploprora_championii_PK12025 ACGAC-TCTCGACAATGGATATCTC-GGCTCTCGCATCGATGAAGAGCGCAGCGAAATGCGATACGTGGTGC-GAATTGCAGAATCCCGCGAACC-ATCGAGTCTTTGAACGCAAGTTGCGCCCGAGGCCAAT-CGGTCGAGGGCACGTCCGCCTG---GGCGTCAA-GCGTTGCGCCGCTCC

Diploprora_championii_PK12029 ATGAC-TCTCGGCAATGGATATCTT-GGCTCTTGCATCGATGAAGAGCGCAGCGAAATGCGATACGTGGTGT-GAATTGCAGAATCCCGTGAACC-ATCAAATATTTGAACGCAAGTTGCGCCCGAGGCCAAT-TGGCTAAGGGCACGTCCGCCTG---GGCGTCAA-GCATTATATCGCTTC

Diploprora_championii_PK12095 ACGAC-TCTCGACAATGGATATCTC-GGCTCTCGCATCGATGAAGAGCGCAGCGAAATGCGATACGTGGTGC-GAATTGCAGAATCCCGCGAACC-ATCGAGTCTTTGAACGCAAGTTGCGCCCGAGGCCAAT-CGGTCGAGGGCACGTCCGCCTG---GGCGTCAA-GCGTTGCGCCGCTCC

Diploprora_championii_SG1144 ACGAC-TCTCGACAATGGATATCTC-GGCTCTCGCATCGATGAAGAGCGCAGCGAAATGCGATACGTGGTGC-GAATTGCAGAATCCCGCGAACC-ATCGAGTCTTTGAACGCAAGTTGCGCCCGAGGCCAAT-CGGTCGAGGGCACGTCCGCCTG---GGCGTCAA-GCGTTGCGCCGCTCC

Diploprora_championii_SG1230 ACGAC-TCTCGACAATGGATATCTC-GGCTCTCGCATCGATGAAGAGCGCAGCGAAATGCGATACGTGGTGC-GAATTGCAGAATCCCGCGAACC-ATCGAGTCTTTGAACGCAAGTTGCGCCCGAGGCCAAT-CGGTCGAGGGCACGTCCGCCTG---GGCGTCAA-GCGTTGCGCCGCTCC

Disperis_neilgherrensis_KM583454 ATGACTTCTTGGCAATGGATATCTT-GGCTCTTGCATCGATGAAGAACATAGCGAAATGTGATACATGGTGC-GAATTGCAGAATCCCGTGAACC-ATCGAGGTTTTGAATGCAAGTTGCGCCAAAGGCCAGC-AGGTCAAGGGCACGTCTGCTTG---GGAGTCAAAATAACTCATCGCTCT

Epipogium_roseum_EU711232 ACGAC-TCTCGACAATGGATATCTC-GGCTCTCGCATCGATGAAGAACGCAGCGAAATGCGATACGTGGTGC-GAATTGCAGAATCCCGCGAACC-ATCGAGTCTTTGAACGCAAGTTGCGCCCAAGGCCAGT-AGGCCAAGGGCACGCCCGCTTG---GGCGTCAA-GCATTGCATCTCTCC

Epipogium_roseum_SG1249 ACGAC-TCTCGACAATGGATATCTC-GGCTCTCGCATCGATGAAGAACGCAGCGAAATGCGATACGTGGTGC-GAATTGCAGAATCCCGCGAACC-ATCGAGTCTTTGAACGCAAGTTGCGCCCAAGGCCAGT-AGGCCAAGGGCACGCCCGCTTG---GGCGTCAT-GCATTGCATCTCTCC

Epipogium_roseum_SG1250 ACGAC-TCTCGACAATGGATATCTC-GGCTCTCGCATCGATGAAGAACGCAGCGAAATGCGATACGTGGTGC-GAATTGCAGAATCCCGCGAACC-ATCGAGTCTTTGAACGCAAGTTGCGCCCAAGGCCAGT-AGGCCAAGGGCACGCCCGCTTG---GGCGTCAT-GCATTGCATCTCTCC

Eria_scabrilinguis_KFBG916 ACGAC-TCTCGGCAATGGATATCTC-GGCTCTCGCATCGATGAAGAGCGCAGCGAAATGCGATACGTGGTGC-GAATTGCAGAATCCCGCGAACC-ATCGAGTCTTTGAACGCAAGTTGCGCCCGAGGCCAAC-CGGCCGAGGGCACGTCTGCCTG---GGCGTCAA-GCGTTGCATCGCTCC

Eria_scabrilinguis_KY239252 ACGAC-TCTCGGCAATGGATATCTC-GGCTCTCGCATCGATGAAGAGCGCAGCGAAATGCGATACGTGGTGC-GAATTGCAGAATCCCGCGAACC-ATCGAGTCTTTGAACGCAAGTTGCGCCCGAGGCCAAC-CGGCCGAGGGCACGTCTGCCTG---GGCGTCAA-GCGTTGCATCGCTCC

Eria_scabrilinguis_KY966596 ACGAC-TCTCGGCAATGGATATCTC-GGCTCTCGCATCGATGAAGAGCGCAGCGAAATGCGATACGTGGTGC-GAATTGCAGAATCCCGCGAACC-ATCGAGTCTTTGAACGCAAGTTGCGCCCGAGGCCAAC-CGGCCGAGGGCACGTCTGCCTG---GGCGTCAA-GCGTTGCATCGCTCC

Eria_scabrilinguis_PK12164 ACGAC-TCTCGGCAATGGATATCTC-GGCTCTCGCATCGATGAAGAGCGCAGCGAAATGCGATACGTGGTGC-GAATTGCAGAATCCCGCGAACC-ATCGAGTCTTTGAACGCAAGTTGCGCCCGAGGCCAAC-CGGCCGAGGGCACGTCTGCCTG---GGCGTCAA-GCGTTGCATCGCTCC

Eria_scabrilinguis_PK12165 ACGAC-TCTCGGCAATGGATATCTC-GGCTCTCGCATCGATGAAGAGCGCAGCGAAATGCGATACGTGGTGC-GAATTGCAGAATCCCGCGAACC-ATCGAGTCTTTGAACGCAAGTTGCGCCCGAGGCCAAC-CGGCCGAGGGCACGTCTGCCTG---GGCGTCAA-GCGTTGCATCGCTCC

Eria_scabrilinguis_SG1302 ACGAC-TCTCGGCAATGGATATCTC-GGCTCTCGCATCGATGAAGAGCGCAGCGAAATGCGATACGTGGTGC-GAATTGCAGAATCCCGCGAACC-ATCGAGTCTTTGAACGCAAGTTGCGCCCGAGGCCAAC-CGGCCGAGGGCACGTCTGCCTG---GGCGTCAA-GCGTTGCATCGCTCC

Erythrodes_blumei_JN166066 ATGAC-TCTCGGCAATGGATATCTT-GGCTCTTGCATCGATGAAGAGCGCAGCGAAATGCGATACGTGGTGT-GAATTGCAGAATTCCGTGAACC-ATCGAATTTTTGAACGCAAGTTGCGCCCGAGGCCAAT-TGGCTAAGGGCACGTCCGCCTG---GGCGTCAA-GCATTACATCGCTTC

Erythrodes_blumei_KT343981 ATGAC-TCTCGGCAATGGATATCTT-GGCTCTTGCATCGATGAAGAGCGCAGCGAAATGCGATACGTGGTGT-GAATTGCAGAATTCCGTGAACC-ATCGAATTTTTGAACGCAAGTTGCGCCCGAGGCCAAT-TGGCTAAGGGCACGTCCGCCTG---GGCGTCAA-GC-TTACATCGCTTA

Erythrodes_blumei_KT343982 ATGAC-TCTCGGCAATGGATATCTT-GGCTCTTGCATCGATGAAGAGCGCAGCGAAATGCGATACGTGGTGT-GAATTGCAGAATTCCGTGAACC-ATCGAATTTTTGAACGCAAGTTGCGCCCGAGGCCAAT-TGGCTAAGGGCACGTCCGCCTG---GGCGTCGA-GCATTACATCGCTTC

Erythrodes_blumei_PK12103 ATGAC-TCTCGGCAATGGATATCTT-GGCTCTTGCATCGATGAAGAGCGCAGCGAAATGCGATACGTGGTGT-GAATTGCAGAATTCCGTGAACC-ATCGAATTTTTGAACGCAAGTTGCGCCCGAGGCCAAT-TGGCTAAGGGCACGTCCGCCTG---GGCGTCAA-GCATTACATCGCTTC

Erythrodes_blumei_PK12104 ATGAC-TCTCGGCAATGGATATCTT-GGCTCTTGCATCGATGAAGAGCGCAGCGAAATGCGATACGTGGTGT-GAATTGCAGAATTCCGTGAACC-ATCGAATTTTTGAACGCAAGTTGCGCCCGAGGCCAAT-TGGCTAAGGGCACGTCCGCCTG---GGCGTCAA-GCATTACATCGCTTC

Eulophia_flava_JN114508 ACGAC-TCTCGGCAATGGATATCTC-GGCTCTCGCATCGATGAAGAGCGCAGCGAAATGCGATACGTGGTGC-GAATTGCAGAATCCCGCGAACC-ATCGAGTCTTTGAACGCAAGTTGCGCCTGAGGCCAGT-CGGCTGAGGGCACGTCCGCCTG---GGCGTCAA-GCTTCGCGTTGCTCC

Eulophia_flava_JN114509 ACGAC-TCTCGGCAATGGATATCTC-GGCTCTCGCATCGATGAAGAGCGCAGCGAAATGCGATACGTGGTGC-GAATTGCAGAATCCCGCGAACC-ATCGAGTCTTTGAACGCAAGTTGCGCCTGAGGCCAGT-CGGCTGAGGGCACGTCCGCCTG---GGCGTCAA-GCTTCGCGTTGCTCC

Eulophia_flava_SG1158 ACGAC-TCTCGGCAATGGATATCTC-GGCTCTCGCATCGATGAAGAGCGCAGCGAAATGCGATACGTGGTGC-GAATTGCAGAATCCCGCGAACC-ATCGAGTCTTTGAACGCAAGTTGCGCCTGAGGCCAGT-CGGCTGAGGGCACGTCCGCCTG---GGCGTCAA-GCTTCGCGTTGCTCC

Eulophia_flava_SG1159 ACGAC-TCTCGGCAATGGATATCTC-GGCTCTCGCATCGATGAAGAGCGCAGCGAAATGCGATACGTGGTGC-GAATTGCAGAATCCCGCGAACC-ATCGAGTCTTTGAACGCAAGTTGCGCCTGAGGCCAGT-CGGCTGAGGGCACGTCCGCCTG---GGCGTCAA-GCTTCGCGTTGCTCC

Eulophia_graminea_AF284727 ACGAC-TCTCGGCAATGGATATCTC-GGCTCTCGCATCGATGAAGAGCGCAGCGAAATGCGATACGTGGTGC-GAATTGCAGAATCCCGCGAACC-ATCGAGTCTTTGAACGCAAGTTGCGCCCGAGGCCAAC-CGGCCAAGGGCACGTCTGCCTG---GGCGTCAA-GCGTTGCGTCGCTCC

Eulophia_graminea_FJ565666 ACGAC-TCTCGGCAATGGATATCTC-GGCTCTCGCATCGATGAAGAGCGCAGCGAAATGCGATACGTGGTGC-GAATTGCAGAATCCCGCGAACC-GTCGAGTCTTTGAACGCAAGTTGCGCCTGAGGCCAGC-CGGCCGAGGGCACGTCCGCCTG---GGCGTCGA-GCTTCGCGTCGCTCC

Eulophia_graminea_KF318890 ACGAC-TCTCGGCAATGGATATCTC-GGCTCTCGCATCGATGAAGAGCGCAGCGAAATGCGATACGTGGTGC-GAATTGCAGAATCCCGCGAACC-GTCGAGTCTTTGAACGCAAGTTGCGCCTGAGGCCAGC-CGGCCGAGGGCACGTCCGCCTG---GGCGTCGA-GCTTCGCGTCGCTCC

Eulophia_graminea_MH768268 ACGAC-TCTCGGCAATGGATATCTC-GGCTCTCGCATCGATGAAGAGCGCAGCGAAATGCGATACGTGGTGC-GAATTGCAGAATCCCGCGAACC-GTCGAGTCTTTGAACGCAAGTTGCGCCTGAGGCCAGC-CGGCCGAGGGCACGTCCGCCTG---GGCGTCGA-GCTTCGCGTCGCTCC

Eulophia_graminea_MH768269 ACGAC-TCTCGGCAATGGATATCTC-GGCTCTCGCATCGATGAAGAGCGCAGCGAAATGCGATACGTGGTGC-GAATTGCAGAATCCCGCGAACC-GTCGAGTCTTTGAACGCAAGTTGCGCCTGAGGCCAGC-CGGCCGAGGGCACGTCCGCCTG---GGCGTCGA-GCTTCGCGTCGCTCC

Eulophia_graminea_SG1270 ACGAC-TCTCGGCAATGGATATCTC-GGCTCTCGCATCGATGAAGAGCGCAGCGAAATGCGATACGTGGTGC-GAATTGCAGAATCCCGCGAACC-GTCGAGTCTTTGAACGCAAGTTGCGCCTGAGGCCAGC-CGGCCGAGGGCACGTCCGCCTG---GGCGTCGA-GCTTCGCGTCGCTCC

Eulophia_graminea_SG1350 ACGAC-TCTCGGCAATGGATATCTC-GGCTCTCGCATCGATGAAGAGCGCAGCGAAATGCGATACGTGGTGC-GAATTGCAGAATCCCGCGAACC-GTCGAGTCTTTGAACGCAAGTTGCGCCTGAGGCCAGC-CGGCCGAGGGCACGTCCGCCTG---GGCGTCGA-GCTTCGCGTCGCTCC

Eulophia_picta_AF284726 ACGAC-TCTCGGCAATGGATATCTC-GGCTCTCGCATCGATGAAGAGCGCAGCGAAATGCGATACGTGGTGC-GAATTGCAGAATCCCGCGAACC-ATCGAGTCTTTGAACGCAAGTTGCGCCCGAGGCCAAC-CGGCCGAGGGCACGTCTGCCTG---GGCGTCAA-GCGTTGCGTCGCTCC

Eulophia_picta_JN114510 ACGAC-TCTCGGCAATGGATATCTC-GGCTCTCGCATCGATGAAGAGCGCAGCGAAATGCGATACGTGGTGC-GAATTGCAGAATCCCGCGAACC-ATCGAGTCTTTGAACGCAAGTTGCGCCTGAGGTCAGC-TGGCCGAGGGCACGTCCGCCTG---GGCGTCAA-GCTTCGCGTCGCTCC

Eulophia_picta_JN114511 ACGAC-TCTCGGCAATGGATATCTC-GGCTCTCGCATCGATGAAGAGCGCAGCGAAATGCGATACGTGGTGC-GAATTGCAGAATCCCGCGAACC-ATCGAGTCTTTGAACGCAAGTTGCGCCTGAGGTCAGC-TGGCCGAGGGCACGTCCGCCTG---GGCGTCAA-GCTTCGCGTCGCTCC

Eulophia_picta_JN114512 ACGAC-TCTCGGCAATGGATATCTC-GGCTCTCGCATCGATGAAGAGCGCAGCGAAATGCGATACGTGGTGC-GAATTGCAGAATCCCGCGAACC-ATCGAGTCTTTGAACGCAAGTTGCGCCTGAGGTCAGC-TGGCCGAGGGCACGTCCGCCTG---GGCGTCAA-GCTTCGCGTCGCTCC

Eulophia_picta_JN114513 ACGAC-TCTCGGCAATGGATATCTC-GGCTCTCGCATCGATGAAGAGCGCAGCGAAATGCGATACGTGGTGC-GAATTGCAGAATCCCGCGAACC-ATCGAGTCTTTGAACGCAAGTTGCGCCTGAGGTCAGC-TGGCCGAGGGCACGTCCGCCTG---GGCGTCAA-GCTTCGCGTCGCTCC

Eulophia_picta_PK12045 ACGAC-TCTCGGCAATGGATATCTC-GGCTCTCGCATCGATGAAGAGCGCAGCGAAATGCGATACGTGGTGC-GAATTGCAGAATCCCGCGAACC-ATCGAGTCTTTGAACGCAAGTTGCGCCTGAGGTCAGC-TGGCCGAGGGCACGTCCGCCTG---GGCGTCAA-GCTTCGCGTCGCTCC

Eulophia_picta_PK12046 ACGAC-TCTCGGCAATGGATATCTC-GGCTCTCGCATCGATGAAGAGCGCAGCGAAATGCGATACGTGGTGC-GAATTGCAGAATCCCGCGAACC-ATCGAGTCTTTGAACGCAAGTTGCGCCTGAGGTCAGC-TGGCCGAGGGCACGTCCGCCTG---GGCGTCAA-GCTTCGCGTCGCTCC

Eulophia_picta_PK12047 ACGAC-TCTCGGCAATGGATATCTC-GGCTCTCGCATCGATGAAGAGCGCAGCGAAATGCGATACGTGGTGC-GAATTGCAGAATCCCGCGAACC-ATCGAGTCTTTGAACGCAAGTTGCGCCTGAGGTCAGC-TGGCCGAGGGCACGTCCGCCTG---GGCGTCAA-GCTTCGCGTCGCTCC

Eulophia_picta_PK12048 ACGAC-TCTCGGCAATGGATATCTC-GGCTCTCGCATCGATGAAGAGCGCAGCGAAATGCGATACGTGGTGC-GAATTGCAGAATCCCGCGAACC-ATCGAGTCTTTGAACGCAAGTTGCGCCTGAGGTCAGC-TGGCCGAGGGCACGTCCGCCTG---GGCGTCAA-GCTTCGCGTCGCTCC

Eulophia_picta_PK12137 ACGAC-TCTCGGCAATGGATATCTC-GGCTCTCGCATCGATGAAGAGCGCAGCGAAATGCGATACGTGGTGC-GAATTGCAGAATCCCGCGAACC-ATCGAGTCTTTGAACGCAAGTTGCGCCTGAGGTCAGC-TGGCCGAGGGCACGTCCGCCTG---GGCGTCAA-GCTTCGCGTCGCTCC

Eulophia_picta_PK12138 ACGAC-TCTCGGCAATGGATATCTC-GGCTCTCGCATCGATGAAGAGCGCAGCGAAATGCGATACGTGGTGC-GAATTGCAGAATCCCGCGAACC-ATCGAGTCTTTGAACGCAAGTTGCGCCTGAGGTCAGC-TGGCCGAGGGCACGTCCGCCTG---GGCGTCAA-GCTTCGCGTCGCTCC

Eulophia_picta_PK12153 ACGAC-TCTCGGCAATGGATATCTC-GGCTCTCGCATCGATGAAGAGCGCAGCGAAATGCGATACGTGGTGC-GAATTGCAGAATCCCGCGAACC-ATCGAGTCTTTGAACGCAAGTTGCGCCTGAGGTCAGC-TGGCCGAGGGCACGTCCGCCTG---GGCGTCAA-GCTTCGCGTCGCTCC

Eulophia_picta_SG1271 ACGAC-TCTCGGCAATGGATATCTC-GGCTCTCGCATCGATGAAGAGCGCAGCGAAATGCGATACGTGGTGC-GAATTGCAGAATCCCGCGAACC-ATCGAGTCTTTGAACGCAAGTTGCGCCTGAGGTCAGC-TGGCCGAGGGCACGTCCGCCTG---GGCGTCAA-GCTTCGCGTCGCTCC

Eulophia_zollingeri_AB306313 ACGAC-TCTCGACAATGGATATCTC-GGCTCTCGCATCGATGAAGAGCGCAGCGAAATGCGATACGTGGTGC-GAATTGCAGAATCCCGCGAACC-ATCGAGTCTTTGAACGCAAGTTGCGCCTGAGGCCAGC-CGGCCGAGGGCACGTCCGCCTG---GGCGTCAA-GCTTCGCGTCGCTCC

Eulophia_zollingeri_SG1262 ACGAC-TCTCGACAATGGATATCTC-GGCTCTCGCATCGATGAAGAGCGCAGCAAAATGCGATACGTGGTGC-GAATTGCAGAATCCCGCGAACC-ATCGAGTCTTTGAACGCAAGTTGCGCCTGAGGCCAGC-CGGCCGAGGGCACGTCCGCCTG---GGCGCCAA-GCTTCGCGTCGCTCC

Eulophia_zollingeri_SG1263 ACGAC-TCTCGATAATGGATATCTC-AGCTCTCGCATCGATGAAGAGCGCAGCGAAATGCGATACGTGGTGC-GAATTGCAGAATCCCGCGAACC-ATCGAGTCTTTGAACGCAAGTTGCGCCTGAGGCCAGC-CGGCCGAGGGCACGTCCGCCTG---GGCGTCAA-GCTTCGCGTCGCTCC

Eulophia_zollingeri_SG1264 ACGAC-TCTCGACAATGGATATCTC-AGCTCTCGCATCGATGAAGAGCGCAGCGAAATGCGATACGTGGTGC-AAATTGCAGAATCCCGCGAACC-ATCGAGTCTTTGAACGCAAGTTGCGCGTGAGGCCAGC-CGGCCGAGGGCACGTCCGCCTG---GGCGTCAA-GCTTCGCGTCGCTCC

Eulophia_zollingeri_SG1265 ACGAC-TCTCGACAATGGATATCTC-GGCTCTCGCATCGATGAAGAGCGCAGCGAAATGCGATACGTGGTGC-TAATTGCAGAATCCCGCGAACC-ATCGAGTCTTTGAACGCAAGTTGCGCCTGAGGCCAGC-TGGCCGAGGGCACGTCCGCCTG---GGTGTCAA-GCTTCGCGTCGCTCC

Eulophia_zollingeri_SG1353 ACGAC-TCTCGACAATGGATATCTC-GGCTCTCGCATCGATGAAGAGCGCAGCGAAATGCGATACGTGGTGC-GAATTGCAGAATCCCGCGAACC-ATCGAGTCTTTGAACGCAAGTTGCGCCTGAGGCCAGC-CGGCCGAGGGCACGTCCGCCTG---GGCGTCAA-GCTTCGCGTCGCTCC

Gastrochilus_japonicus_AY228503 ACGAC-TCTCGGCAATGGATATCTC-GGCTCTCGCATCGATGAAGAGCGCAGCGAAATGCGATACGTGGTGC-GAATTGCAGAATCCCGCGAACC-ATCGAGTCTTTGTACGCAAGTTGCGCCCGAGGCCAAT-CGGTCGAGGGCACGTCCGCCTG---GGCGTCAA-GCGTCGCGCCGCTCC

Gastrochilus_japonicus_KF545875 ACGAC-TCTCGACAATGGATATCTC-GGCTCTCGCATCGATGAAGAGCGCAGCGAAATGCGATACGTGGTGC-GAATTGCAGAATCCCGCGAACC-ATCGAGTCTTTGAACGCAAGTTGCGCCCGAGGCCATT-CGGTCGAGGGCACGTCCGCCTG---GGCGTCAA-GCGTTGCGCCGCTCC

Gastrochilus_japonicus_KJ733418 ACGAC-TCTCGACAATGGATATCTC-GGCTCTCGCATCGATGAAGAGCGCAGCGAAATGCGATACGTGGTGC-GAATTGCAGAATCCCGCGAACC-ATCGAGTCTTTGAACGCAAGTTGCGCCCGAGGCCATT-CGGTCGAGGGCACGTCCGCCTG---GGCGTCAA-GCGTTGCGCCGCTCC

Gastrochilus_japonicus_KY966598 ACGAC-TCTCGACAATGGATATCTC-GGCTCTCGCATCGATGAAGAGCGCAGCGAAATGCGATACGTGGTGC-GAATTGCAGAATCCCGCGAACC-ATCGAGTCTTTGAACGCAAGTTGCGCCCGAGGCCAAT-CGGTCGAGGGCACGTCCGCCTG---GGCGTCAA-GCGTCGCGCCGCTCC

Gastrochilus_japonicus_KY966599 ACGAC-TCTCGACAATGGATATCTC-GGCTCTCGCATCGATGAAGAGCGCAGCGAAATGCGATACGTGGTGC-GAATTGCAGAATCCCGCGAACC-ATCGAGTCTTTGAACGCAAGTTGCGCCCGAGGCCAAT-CGGTCGAGGGCACGTCCGCCTG---GGCGTCAA-GCGTCGCGCCGCTCC

Gastrochilus_kadooriei_KFBG3250 ACGAC-TCTCGACAATGGATATCTC-GGCTCTCGCATCGATGAAGAGCGCAGCGAAATGCGATACGTGGTGC-GAATTGCAGAATCCCGCGAACC-ATCGAGTCTTTGAACGCAAGTTGCGCCCGAGGCCAAT-CGGTCGAGGGCACGTCCGCCTG---GGCGTCAA-GCGTTGCGCCGCTCC

Gastrodia_peichatieniana_HK43268 ACGAC-TCTCGGCAATGGATATCTC-GGCTCTCGCATCGATGAAGAGCGCAGCGAAATGCGATACGTGGTGC-GAATTGCAGAATCCCGCGAACC-ATCGAGTCTTTGAACGCAAGTTGCGCCCGAGGCCAAT-CGGCCAAGGGCACGCCCGCCTG---GGCGTCCA-GCATTACGCCGCTCC

Goodyera_foliosa_HM140995 ATGAC-TCTCGGCAATGGATATCTT-GGCTCTTGCATCGATGAAGAGCGCAGCGAAATGCGATACGTGGTGT-GAATTGCAGAATCCCGTGAACC-ATCGAGTTTTTGAACGCAAGTTGCGCCCGAGGCCAAT-TGGCTAAGGGCACGTCCGCCTG---GGCGTCAA-GCATTACGTCGCTTC

Goodyera_foliosa_KT344001 ATGAC-TCTCGGCAATGGATATCTT-GGCTCTTGCATCGATGAAGAGCGCAGCGAAATGCGATACGTGGTGT-GAATTGCAGAATCCCGTGAACC-ATCGAGTTTTTGAACGCAAGTTGCGCCCGAGGCCAAT-TGGCTAAGGGCACGTCCGCCTG---GGCGTCAA-GC-TTACGTCGCTTA

Goodyera_foliosa_KT344002 ATGAC-TCTCGGCAATGGATATCTT-GGCTCTTGCATCGATGAAGAGCGCAGCGAAATGCGATACGTGGTGT-GAATTGCAGAATCCCGTGAACC-ATCGAGTTTTTGAACGCAAGTTGCGCCCGAGGCCAAT-TGGCTAAGGGCACGTCCGCCTG---GGCGTCAA-GC-TTACGTCGCTTA

Goodyera_foliosa_KT344003 ATGAC-TCTCGGCAATGGATATCTT-GGCTCTTGCATCGATGAAGAGCGCAGCGAAATGCGATACGTGGTGT-GAATTGCAGAATCCCGTGAACC-ATCGAGTTTTTGAACGCAAGTTGCGCCCGAGGCCAAT-TGGCTAAGGGCACGTCCGCCTG---GGCGTCAA-GC-TTACGTCGCTTA

Goodyera_foliosa_KT344004 ATGAC-TCTCGGCAATGGATATCTT-GGCTCTTGCATCGATGAAGAGCGCAGCGAAATGCGATACGTGGTGT-GAATTGCAGAATCCCGTGAACC-ATCGAGTTTTTGAACGCAAGTTGCGCCCGAGGCCAAT-TGGCTAAGGGCACGTCCGCCTG---GGCGTCAA-GC-TTACGTCGCTTA

Goodyera_foliosa_KT344005 ATGAC-TCTCGGCAATGGATATCTT-GGCTCTTGCATCGATGAAGAGCGCAGCGAAATGCGATACGTGGTGT-GAATTGCAGAATCCCGTGAACC-ATCGAGTTTTTGAACGCAAGTTGCGCCCGAGGCCAAT-TGGCTAAGGGCACGTCCGCCTG---GGCGTCAA-GC-TTACGTCGCTTA

Goodyera_foliosa_KT344006 ATGAC-TCTCGGCAATGGATATCTT-GGCTCTTGCATCGATGAAGAGCGCAGCGAAATGCGATACGTGGTGT-GAATTGCAGAATCCCGTGAACC-ATCGAGTTTTTGAACGCAAGTTGCGCCCGAGGCCAAT-TGGCTAAGGGCACGTCCGCCTG---GGCGTCAA-GC-TTACGTCGCTTA

Goodyera_foliosa_KT344007 ATGAC-TCTCGGCAATGGATATCTT-GGCTCTTGCATCGATGAAGAGCGCAGCGAAATGCGATACGTGGTGT-GAATTGCAGAATCCCGTGAACC-ATCGAGTTTTTGAACGCAAGTTGCGCCCGAGGCCAAT-TGGCTAAGGGCACGTCCGCCTG---GGCGTCAA-GC-TTACGTCGCTTA

Goodyera_foliosa_KT344008 ATGAC-TCTCGGCAATGGATATCTT-GGCTCTTGCATCGATGAAGAGCGCAGCGAAATGCGATACGTGGTGT-GAATTGCAGAATCCCGTGAACC-ATCGAGTTTTTGAACGCAAGTTGCGCCCGAGGCCAAT-TGGCTAAGGGCACGTCCGCCTG---GGCGTCAA-GC-TTACGTCGCTTA

Goodyera_foliosa_KT344009 ATGAC-TCTCGGCAATGGATATCTT-GGCTCTTGCATCGATGAAGAGCGCAGCGAAATGCGATACGTGGTGT-GAATTGCAGAATCCCGTGAACC-ATCGAGTTTTTGAACGCAAGTTGCGCCCGAGGCCAAT-TGGCTAAGGGCACGTCCGCCTG---GGCGTCAA-GC-TTACGTCGCTTA

Goodyera_foliosa_PK12067 ATGAC-TCTCGGCAATGGATATCTT-GGCTCTTGCATCGATGAAGAGCGCAGCGAAATGCGATACGTGGTGT-GAATTGCAGAATCCCGTGAACC-ATCGAGTTTTTGAACGCAAGTTGCGCCCGAGGCCAAT-TGGCTAAGGGCACGTCCGCCTG---GGCGTCAA-GCATTACGTCGCTTC

Goodyera_foliosa_SG1309 ATGAC-TCTCGGCAATGGATATCTT-GGCTCTTGCATCGATGAAGAGCGCAGCGAAATGCGATACGTGGTGT-GAATTGCAGAATCCCGTGAACC-ATCGAGTTTTTGAACGCAAGTTGCGCCCGAGGCCAAT-TGGCTAAGGGCACGTCCGCCTG---GGCGTCAA-GCATTACGTCGCTTC

Goodyera_foliosa_SG1315 ATGAC-TCTCGGCAATGGATATCTT-GGCTCTTGCATCGATGAAGAGCGCAGCGAAATGCGATACGTGGTGT-GAATTGCAGAATCCCGTGAACC-ATCGAGTTTTTGAACGCAAGTTGCGCCCGAGGCCAAT-TGGCTAAGGGCACGTCCGCCTG---GGCGTCAA-GCATTACGTCGCTTC

Goodyera_foliosa_var_foliosa_KC205157 ATGAC-TCTCGGCAAAGGATTTCTT-GGCTCTTGCATCGATGAAGAGCGCAGCGAAATGCGATACGTGGTGG-GAATTGGAAAATCCCGTGAACC-ATCGAGTTTTTGAACGCAAGTTGCGCCCGAGGCCAAT-TGGCTAAGGGCACGTCCGCCTG---GGCGTCAA-GCATTACGTCGCTTC

Goodyera_foliosa_var_laevis_HM140998 ATGAC-TCTCGGCAATGGATATCTT-GGCTCTTGCATCGATGAAGAGCGCAGCGAAATGCGATACGTGGTGT-GAATTGCAGAATCCCGTGAACC-ATCGAGTTTTTGAACGCAAGTTGCGCCCGAGGCCAAT-TGGCTAAGGGCACGTCCGCCTG---GGCGTCAA-GCATTACGTCGCTTC

Goodyera_procera_HM151402 ATGAC-TCTCGGCAATGGATATCTT-GGCTCTTGCATCGATGAAGAGCGCAGCGAAATGCGATACGTGGTGT-GAATTGCAGAATCCCGTGAACC-ATCGAATTTTTGAACGCAAGTTGCGCCCGAGGCCAAT-TGGCTAAGGGCACGTCCGCCTG---GGCGTCAA-GCATTACGTCGCTTC

Goodyera_procera_HM222488 ATGAC-TCTCGGCAATGGATATCTT-GGCTCTTGCATCGATGAAGAGCGCAGCGAAATGCGATACGTGGTGT-GAATTGCAGAATCCCGTGAACC-ATCGAATTTTTGAACGCAAGTTGCGCCCGAGGCCAAT-TGGCTAAGGGCACGTCCGCCTG---GGCGTCAA-GCATTACGTCGCTTC

Goodyera_procera_JN114514 ATGAC-TCTCGGCAATGGATATCTT-GGCTCTTGCATCGATGAAGAGCGCAGCGAAATGCGATACGTGGTGT-GAATTGCAGAATCCCGTGAACC-ATCGAATTTTTGAACGCAAGTTGCGCCCGAGGCCAAT-TGGCTAAGGGCACGTCCGCCTG---GGCGTCAA-GCATTACGTCGCTTC

Goodyera_procera_JN114515 ATGAC-TCTCGGCAATGGATATCTT-GGCTCTTGCATCGATGAAGAGCGCAGCGAAATGCGATACGTGGTGT-GAATTGCAGAATCCCGTGAACC-ATCGAATTTTTGAACGCAAGTTGCGCCCGAGGCCAAT-TGGCTAAGGGCACGTCCGCCTG---GGCGTCAA-GCATTACGTCGCTTC

Goodyera_procera_JN114516 ATGAC-TCTCGGCAATGGATATCTT-GGCTCTTGCATCGATGAAGAGCGCAGCGAAATGCGATACGTGGTGT-GAATTGCAGAATCCCGTGAACC-ATCGAATTTTTGAACGCAAGTTGCGCCCGAGGCCAAT-TGGCTAAGGGCACGTCCGCCTG---GGCGTCAA-GCATTACGTCGCTTC

Goodyera_procera_JN114517 ATGAC-TCTCGGCAATGGATATCTT-GGCTCTTGCATCGATGAAGAGCGCAGCGAAATGCGATACGTGGTGT-GAATTGCAGAATCCCGTGAACC-ATCGAATTTTTGAACGCAAGTTGCGCCCGAGGCCAAT-TGGCTAAGGGCACGTCCGCCTG---GGCGTCAA-GCATTACGTCGCTTC

Goodyera_procera_JN114518 ATGAC-TCTCGGCAATGGATATCTT-GGCTCTTGCATCGATGAAGAGCGCAGCGAAATGCGATACGTGGTGT-GAATTGCAGAATCCCGTGAACC-ATCGAATTTTTGAACGCAAGTTGCGCCCGAGGCCAAT-TGGCTAAGGGCACGTCCGCCTG---GGCGTCAA-GCATTACGTCGCTTC

Goodyera_procera_KC237319 ATGAC-TCTCGGCAATGGATATCTT-GGCTCTTGCATCGATGAAGAGCGCAGCGAAATGCGATACGTGGTGT-GAATTGCAGAATCCCGTGAACC-ATCGAATTTTTGAACGCAAGTTGCGCCCGAGGCCAAT-TGGCTAAGGGCACGTCCGCCTG---GGCGTCAA-GCATTACGTCGCTTC

Goodyera_procera_KT344043 ATGAC-TCTCGGCAATGGATATCTT-GGCTCTTGCATCGATGAAGAGCGCAGCGAAATGCGATACGTGGTGT-GAATTGCAGAATCCCGTGAACC-ATCGAATTTTTGAACGCAAGTTGCGCCCGAGGCCAAT-TGGCTAAGGGCACGTCCGCCTG---GGCGTCAA-GC-TTACGTCGCTTA

Goodyera_procera_KT344044 ATGAC-TCTCGGCAATGGATATCTT-GGCTCTTGCATCGATGAAGAGCGCAGCGAAATGCGATACGTGGTGT-GAATTGCAGAATCCCGTGAACC-ATCGAATTTTTGAACGCAAGTTGCGCCCGAGGCCAAT-TGGCTAAGGGCACGTCCGCCTG---GGCGTCAA-GC-TTACGTCGCTT-

Goodyera_procera_KT344045 ATGAC-TCTCGGCAATGGATATCTT-GGCTCTTGCATCGATGAAGAGCGCAGCGAAATGCGATACGTGGTGT-GAATTGCAGAATCCCGTGAACC-ATCGAATTTTTGAACGCAAGTTGCGCCCGAGGCCAAT-TGGCTAAGGGCACGTCCGCCTG---GGCGTCAA-GC-TTACGTCGCTTA

Goodyera_procera_KY966601 ATGAC-TCTCGGCAATGGATATCTT-GGCTCTTGCATCGATGAAGAGCGCAGCGAAATGCGATACGTGGTGT-GAATTGCAGAATCCCGTGAACC-ATCGAATTTTTGAACGCAAGTTGCGCCCGAGGCCAAT-TGGCTAAGGGCACGTCCGCCTG---GGCGTCAA-GCATTACGTCGCTTC

Goodyera_procera_SG1152 ATGAC-TCTCGGCAATGGATATCTT-GGCTCTTGCATCGATGAAGAGCGCAGCGAAATGCGATACGTGGTGT-GAATTGCAGAATCCCGTGAACC-ATCGAATTTTTGAACGCAAGTTGCGCCCGAGGCCAAT-TGGCTAAGGGCACGTCCGCCTG---GGCGTCAA-GCATTACGTCGCTTC

Goodyera_procera_SG1240 ATGAC-TCTCGGCAATGGATATCTT-GGCTCTTGCATCGATGAAGAGCGCAGCGAAATGCGATACGTGGTGT-GAATTGCAGAATCCCGTGAACC-ATCGAATTTTTGAACGCAAGTTGCGCCCGAGGCCAAT-TGGCTAAGGGCACGTCCGCCTG---GGCGTCAA-GCATTACGTCGCTTC

Goodyera_procera_SG1241 ATGAC-TCTCGGCAATGGATATCTT-GGCTCTTGCATCGATGAAGAGCGCAGCGAAATGCGATACGTGGTGT-GAATTGCAGAATCCCGTGAACC-ATCGAATTTTTGAACGCAAGTTGCGCCCGAGGCCAAT-TGGCTAAGGGCACGTCCGCCTG---GGCGTCAA-GCATTACGTCGCTTC

Goodyera_procera_SG1346 ATGAC-TCTCGGCAATGGATATCTT-GGCTCTTGCATCGATGAAGAGCGCAGCGAAATGCGATACGTGGTGT-GAATTGCAGAATCCCGTGAACC-ATCGAATTTTTGAACGCAAGTTGCGCCCGAGGCCAAT-TGGCTAAGGGCACGTCCGCCTG---GGCGTCAA-GCATTACGTCGCTTC

Goodyera_pusilla_KM593694 ATGAC-TCTCGGCAATGGATATCTT-GGCTCTTGCATCGATGAAGAGCGCAGCGAAATGCGATACGTGGTGT-GAATTGCAGAATCCCGTGAACC-ATCGAGTTTTTGAACGCAAGTTGCGCCCGAGGCCAAT-TGGCTAAGGGCACGTCCGCCTG---GGCGTCAA-GCATTACGTCGCTTC

Goodyera_pusilla_KT344046 ATGAC-TCTCGGCAATGGATATCTT-GGCTCTTGCATCGATGAAGAGCGCAGCGAAATGCGATACGTGGTGT-GAATTGCAGAATCCCGTGAACC-ATCGAGTTTTTGAACGCAAGTTGCGCCCGAGGCCAAT-TGGCTAAGGGCACGTCCGCCTG---GGCGTCAA-GC-TTACGTCGCTTA

Goodyera_pusilla_KT344047 ATGAC-TCTCGGCAATGGATATCTT-GGCTCTTGCATCGATGAAGAGCGCAGCGAAATGCGATACGTGGTGT-GAATTGCAGAATCCCGTGAACC-ATCGAGTTTTTGAACGCAAGTTGCGCCCGAGGCCAAT-TGGCTAAGGGCACGTCCGCCTG---GGCGTCAA-GC-TTACGTCGCTTA

Goodyera_seikomontana_KT344068 ATGAC-TCTCGGCAATGGATATCTT-GGCTCTTGCATCGATGAAGAGCGCAGCGAAATGCGATACGTGGTGT-GAATTGCAGAATTCCGTGAACC-ATCGAATTTTTGAACGCAAGTTGCGCCCGAGGCCAAT-TGGCTGAGGGCACGTCCGCCTG---GGCGTCAA-GC-TTATGTCGCTTA

Goodyera_seikomontana_KT344069 ATGAC-TCTCGGCAATGGATATCTT-GGCTCTTGCATCGATGAAGAGCGCAGCGAAATGCGATACGTGGTGT-GAATTGCAGAATTCCGTGAACC-ATCGAATTTTTGAACGCAAGTTGCGCCCGAGGCCAAT-TGGCTGAGGGCACGTCCGCCTG---GGCGTCAA-GC-TTATGTCGCTTA

Goodyera_seikomontana_KY966602 ATGAC-TCTCGGCAATGGATATCTT-GGCTCTTGCATCGATGAAGAGCGCAGCGAAATGCGATACGTGGTGT-GAATTGCAGAATTCCGTGAACC-ATCGAATTTTTGAACGCAAGTTGCGCCCGAGGCCAAT-TGGCTGAGGGCACGTCCGCCTG---GGCGTCAA-GCATTATGTCGCTTC

Goodyera_seikoomontana_SG1252 ATGAC-TCTCGGCAATGGATATCTT-GGCTCTTGCATCGATGAAGAGCGCAGCGAAATGCGATACGTGGTGT-GAATTGCAGAATTCCGTGAACC-ATCGAATTTTTGAACGCAAGTTGCGCCCGAGGCCAAT-TGGCTGAGGGCACGTCCGCCTG---GGCGTCAA-GCATTATGTCGCTTC

Goodyera_seikoomontana_SG1253 ATGAC-TCTCGGCAATGGATATCTT-GGCTCTTGCATCGATGAAGAGCGCAGCGAAATGCGATACGTGGTGT-GAATTGCAGAATTCCGTGAACC-ATCGAATTTTTGAACGCAAGTTGCGCCCGAGGCCAAT-TGGCTGAGGGCACGTCCGCCTG---GGCGTCAA-GCATTATGTCGCTTC

Goodyera_seikoomontana_SG1354 ATGAC-TCTCGGCAATGGATATCTT-GGCTCTTGCATCGATGAAGAGCGCAGCGAAATGCGATACGTGGTGT-GAATTGCAGAATTCCGTGAACC-ATCGAATTTTTGAACGCAAGTTGCGCCCGAGGCCAAT-TGGCTGAGGGCACGTCCGCCTG---GGCGTCAA-GCATTATGTCGCTTC

Goodyera_viridiflora_JN166067 ATGAC-TCTCGGCAATGGATATCTT-GGCTCTTGCATCGATGAAGAGCGCAGCGAAATGCGATACGTGGTGT-GAATTGCAGAATTCCGTGAACC-ATCGAATTTTTGAACGCAAGTTGCGCCCGAGGCCAAT-TGGCTAAGGGCACGTCCGCCTG---GGCGTCAA-GCATTAYGTCGCTTC

Goodyera_viridiflora_KC205154 ATGAC-TCTCGGCAATGGATATCTT-GGCTCTTGCATCGATGAAGAGCGCAGCGAAATGCGATACGTGGTGT-GAATTGCAGAATTCCGTGAACC-ATCGAATTTTTGAACGCAAGTTGCGCCCGAGGCCAAT-TGGCTAAGGGCACGTCCGCCTG---GGCGTCAA-GCATTACATCGCTTC

Goodyera_viridiflora_KT344078 ATGAC-TCTCGGCAATGGATATCTT-GGCTCTTGCATCGATGAAGAGCGCAGCGAAATGCGATACGTGGTGT-GAATTGCAGAATTCCGTGAACC-ATCGAATTTTTGAACGCAAGTTGCGCCCGAGGCCAAT-TGGCTAAGGGCACGTCCGCCTG---GGCGTCAA-GC-TTACGTCGCTTA

Goodyera_viridiflora_KT344079 ATGAC-TCTCGGCAATGGATATCTT-GGCTCTTGCATCGATGAAGAGCGCAGCGAAATGCGATACGTGGTGT-GAATTGCAGAATTCCGTGAACC-ATCGAATTTTTGAACGCAAGTTGCGCCCGAAGCCAAT-TGGCTAAGGGCACGTCCGCCTG---GGCGTCAA-GC-TTACGTCGCTTA

Goodyera_viridiflora_KT344080 ATGAC-TCTCGGCAATGGATATCTT-GGCTCTTGCATCGATGAAGAGCGCAGCGAAATGCGATACGTGGTGT-GAATTGCAGAATTCCGTGAACC-ATCGAATTTTTGAACGCAAGTTGCGCCCGAGGCCAAT-TGGCTAAGGGCACGTCCGCCTG---GGCGTCAA-GC-TTATGTCGCTTA

Goodyera_viridiflora_KT344081 ATGAC-TCTCGGCAATGGATATCTT-GGCTCTTGCATCGATGAAGAGCGCAGCGAAATGCGATACGTGGTGT-GAATTGCAGAATTCCGTGAACC-ATCGAATTTTTGAACGCAAGTTGCGCCCGAGGCCAAT-TGGCTAAGGGCACGTCCGCCTG---GGCGTCAA-GC-TTACGTCGCTTA

Goodyera_viridiflora_KT344082 ATGAC-TCTCGGCAATGGATATCTT-GGCTCTTGCATCGATGAAGAGCGCAGCGAAATGCGATACGTGGTGT-GAATTGCAGAATTCCGTGAACC-ATCGAATTTTTGAACGCAAGTTGCGCCTGAGGCCAAT-TGGCTAAGGGCACGTCCGCCTG---GGCGTCAA-GC-TTACGTCGCTTA

Goodyera_viridiflora_KT344083 ATGAC-TCTCGGCAATGGATATCTT-GGCTCTTGCATCGATGAAGAGCGCAGCGAAATGCGATACGTGGTGT-GAATTGCAGAATTCCGTGAACC-ATCGAATTTTTGAACGCAAGTTGCGCCCGAGGCCAAT-TGGCTAAGGGCACGTCCGCCTG---GGCGTCAA-GC-TTATGTCGCTTA

Goodyera_viridiflora_KT344084 ATGAC-TCTCGGCAATGGATATCTT-GGCTCTTGCATCGATGAAGAGCGCAGCGAAATGCGATACGTGGTGT-GAATTGCAGAATTCCGTGAACC-ATCGAATTTTTGAACGCAAGTTGCGCCCGAGGCCAAT-TGGCTAAGGGCACGTCCGCCTG---GGCGTCAA-GC-TTACGTCGCTTA

Goodyera_viridiflora_KT344085 ATGAC-TCTCGGCAATGGATATCTT-GGCTCTTGCATCGATGAAGAGCGCAGCGAAATGCGATACGTGGTGT-GAATTGCAGAATTCCGTGAACC-ATCGAATTTTTGAACGCAAGTTGCGCCCGAGGCCAAT-TGGCTAAGGGCACGTCCGCCTG---GGCGTCAA-GC-TTATGTCGCTTA

Goodyera_viridiflora_PK12170 ATGAC-TCTCGGCAATGGATATCTT-GGCTCTTGCATYGATGAAGAGCGCAGCGAAATGCGATACGTGGTGT-GAATTGCAGAATTCCGTGAACC-ATCGAATTTTTGAACGCAAGTTGCGCCCGAGGCCAAT-TSGCTAAGGGCACGTCCGCCTG---GGCGTCAA-GCATTATGTCGCTTC

Goodyera_viridiflora_PK12172 ATGAC-TCTCGGCAATGGATATCTT-GGCTCTTGCATCGATGAAGAGCGCAGCGAAATGCGATACGTGGTGT-GAATTGCAGAATTCCGTGAACC-ATCGAATTTTTGAACGCAAGTTGCGCCYGAGGCCAAT-TGGCTAAGGGCACGTCCGCCTG---GGCGTCAA-GCATTACGTCGCTTC

Goodyera_viridiflora_SG1305 ATGAC-TCTCGGCAATGGATATCTT-GGCTCTTGCATCGATGAAGAGCGCAGCGAAATGCGATACGTGGTGT-GAATTGCAGAATTCCGTGAACC-ATCGAATTTTTGAACGCAAGTTGCGCCCGAGGCCAAT-TGGCTAAGGGCACGTCCGCCTG---GGCGTCAA-GCATTACGTCGCTTC

Goodyera_viridiflora_SG1306 ATGAC-TCTCGGCAATGGATATCTT-GGCTCTTGCATCGATGAAGAGCGCAGCGAAATGCGATACGTGGTGT-GAATTGCAGAATTCCGTGAACC-ATCGAATTTTTGAACGCAAGTTGCGCCCGAGGCCAAT-TGGCTAAGGGCACGTCCGCCTG---GGCGTCAA-GCATTACGTCGCTTC

Habenaria_ciliolaris_MF944286 AGGAC-TCTCGGCAATGGATATCTT-GGCTCTTGCATCGATGAAGAGCGCAGCGAAATGCGATACGTGGTGC-GAATTGCAGAATCCCGTGAACC-ATCGAGTTTTTGAACGCAAGTTGCGCCTGAGGCCACC-TGGCCAAGGGCACGTCCACCTG---GGCGTCAA-GCATTAAATCGCTCT

Habenaria_ciliolaris_MF944287 AGGAC-TCTCGGCAATGGATATCTT-GGCTCTTGCATCGATGAAGAGCGCAGCGAAATGCGATACGTGGTGC-GAATTGCAGAATCCCGTGAACC-ATCGAGTTTTTGAACGCAAGTTGCGCCTGAGGCCACC-TGGCCAAGGGCACGTCCACCTG---GGCGTCAA-GCATTAAATCGCTCT

Habenaria_dentata_KFBG1009 AGGAC-TCTCGGCAATGGATATCTT-GGCTCTTGCATCGATGAAGAGCGCAGCGAAATGCGATACGTGGTGC-GAATTGCAGAATCCCGTGAACC-ATCGAGTTTTTGAACGCAAGTTGCGCCTGAGGCCACC-TGGCCAAGGGCACGTCCACCTG---GGCGTCAA-GCATTAAATCGCTCT

Habenaria_dentata_KFBG2126B AGGAC-TCTCGGCAATGGATATCTT-GGCTCTTGCATCGATGAAGAGCGCAGCGAAATGCGATACGTGGTGC-GAATTGCAGAATCCCGTGAACC-ATCGAGTTTTTGAACGCAAGTTGCGCCTGAGGCCACC-TGGCCAAGGGCACGTCCACCTG---GGCGTCAA-GCATTAAATCGCTCT

Habenaria_dentata_KJ460038 AGGAC-TCTCGGCAATGGATATCTT-GGCTCTTGCATCGATGAAGAGCGCAGCGAAATGCGATACGTGGTGC-GAATTGCAGAATCCCGTGAACC-ATCGAGTTTTTGAACGCAAGTTGCGCCTGAGGCCACC-TGGCCAAGGGCACGTCCACCTG---GGCGTCAA-GCATTAAATCGCTCT

Habenaria_dentata_KY966605 AGGAC-TCTCGGCAATGGATATCTT-GGCTCTTGCATCGATGAAGAGCGCAGCGAAATGCGATACGTGGTGC-GAATTGCAGAATCCCGTGAACC-ATCGAGTTTTTGAACGCAAGTTGCGCCTGAGGCCACC-TGGCCAAGGGCACGTCCACCTG---GGCGTCAA-GCATTAAATCGCTCT

Habenaria_dentata_PK12058 AGGAC-TCTCGGCAATGGATATCTT-GGCTCTTGCATCGATGAAGAGCGCAGCGAAATGCGATACGTGGTGC-GAATTGCAGAATCCCGTGAACC-ATCGAGTTTTTGAACGCAAGTTGCGCCTGAGGCCACC-TGGCCAAGGGCACGTCCACCTG---GGCGTCAA-GCATTAAATCGCTCT

Habenaria_dentata_PK12059 AGGAC-TCTCGGCAATGGATATCTT-GGCTCTTGCATCGATGAAGAGCGCAGCGAAATGCGATACGTGGTGC-GAATTGCAGAATCCCGTGAACC-ATCGAGTTTTTGAACGCAAGTTGCGCCTGAGGCCACC-TGGCCAAGGGCACGTCCACCTG---GGCGTCAA-GCATTAAATCGCTCT

Habenaria_dentata_SG1005 AGGAC-TCTCGGCAATGGATATCTT-GGCTCTTGCATCGATGAAGAGCGCAGCGAAATGCGATACGTGGTGC-GAATTGCAGAATCCCGTGAACC-ATCGAGTTTTTGAACGCAAGTTGCGCCTGAGGCCACC-TGGCCAAGGGCACGTCCACCTG---GGCGTCAA-GCATTAAATCGCTCT

Habenaria_leptoloba_KY055535 AGGAC-TCTCGGCAATGGATA-CTT-GGCTCTTGCATCGATGAAGAGCGCAGCGAAATGCGATACGTGGTGC-GAATTGCAGAATCCCGTGAACC-ATCGAGTTTTTGAACGCAAGTTGCGCCTGAGGCCAGC-TGGCCAAGGGCACGTCCGCCTG---GGCGTCAA-GCAT-GAATCGCTAC

Habenaria_leptoloba_PK12060 AGGAC-TCTCGGCAATGGATATCTT-GGCTCTTGCATCGATGAAGAGCGCAGCGAAATGCGATACGTGGTGC-GAATTGCAGAATCCCGTGAACC-ATCGAGTTTTTGAACGCAAGTTGCGCCTGAGGCCAGC-TGGCCAAGGGCACGTCCGCCTG---GGCGTCAA-GCATTGAATCGCTAC

Habenaria_leptoloba_PK12061 AGGAC-TCTCGGCAATGGATATCTT-GGCTCTTGCATCGATGAAGAGCGCAGCGAAATGCGATACGTGGTGC-GAATTGCAGAATCCCGTGAACC-ATCGAGTTTTTGAACGCAAGTTGCGCCTGAGGCCAGC-TGGCCAAGGGCACGTCCGCCTG---GGCGTCAA-GCATTGAATCGCTAC

Habenaria_leptoloba_PK12160 AGGAC-TCTCGGCAATGGATATCTT-GGCTCTTGCATCGATGAAGAGCGCAGCGAAATGCGATACGTGGTGC-GAATTGCAGAATCCCGTGAACC-ATCGAGTTTTTGAACGCAAGTTGCGCCTGAGGCCAGC-TGGCCAAGGGCACGTCCGCCTG---GGCGTCAA-GCATTGAATCGCTAC

Habenaria_leptoloba_PK12161 AGGAC-TCTCGGCAATGGATATCTT-GGCTCTTGCATCGATGAAGAGCGCAGCGAAATGCGATACGTGGTGC-GAATTGCAGAATCCCGTGAACC-ATCGAGTTTTTGAACGCAAGTTGCGCCTGAGGCCAGC-TGGCCAAGGGCACGTCCGCCTG---GGCGTCAA-GCATTGAATCGCTAC

Habenaria_leptoloba_PK12183 ATGAC-TCCCGGCAACGGATATCTC-GGCTCTCGCATCGATGAAGAACGTAGCGAAATGCGATACTTGGTGT-GAATTGCAGAATCCCGTGAACC-ATCGAGTCTTTGAACGCAAGTTGCGCCCCAAGCCTTT-GGGCCGAGGGCACGTCTGCCTG---GGTGTCAC-GCATTGTAGCCCCCC

Habenaria_leptoloba_SG1304 AGGAC-TCTCGGCAATGGATATCTT-GGCTCTTGCATCGATGAAGAGCGCAGCGAAATGCGATACGTGGTGC-GAATTGCAGAATCCCGTGAACC-ATCGAGTTTTTGAACGCAAGTTGCGCCTGAGGCCAGC-TGGCCAAGGGCACGTCCGCCTG---GGCGTCAA-GCATTGAATCGCTAC

Habenaria_leptoloba_SG1362 AGGAC-TCTCGGCAATGGATATCTT-GGCTCTTGCATCGATGAAGAGCGCAGCGAAATGCGATACGTGGTGC-GAATTGCAGAATCCCGTGAACC-ATCGAGTTTTTGAACGCAAGTTGCGCCTGAGGCCAGC-TGGCCAAGGGCACGTCCGCCTG---GGCGTCAA-GCATTGAATCGCTAC

Habenaria_linguella_MF944303 AGGAC-TCTCGGCAATGGATATCTT-GGCTCTTGCATCGATGAAGAGCGCAGCGAAATGCGATACGTGGTGC-GAATTGCAGAATCCCGTGAACC-ATCGAGTTTTTGAACGCAAGTTGCGCCCGAGGCCACC-TGGCCAAGGGCACGTCCACCTG---GGCGTCAA-GCATTAAATCGCTCT

Habenaria_linguella_PK12049 AGGAC-TCTCGGCAATGGATATCTT-GGCTCTTGCATCGATGAAGAGCGCAGCGAAATGCGATACGTGGTGC-GAATTGCAGAATCCCGTGAACC-ATCGAGTTTTTGAACGCAAGTTGCGCCCGAGGCCACC-TGGCCAAGGGCACGTCCACCTG---GGCGTCAA-GCATTAAATCGCTCT

Habenaria_linguella_PK12134 AGGAC-TCTCGGCAATGGATATCTT-GGCTCTTGCATCGATGAAGAGCGCAGCGAAATGCGATACGTGGTGC-GAATTGCAGAATCCCGTGAACC-ATCGAGTTTTTGAACGCAAGTTGCGCCCGAGGCCACC-TGGCCAAGGGCACGTCCACCTG---GGCGTCAA-GCATTAAATCGCTCT

Habenaria_linguella_PK12141 AGGAC-TCTCGGCAATGGATATCTT-GGCTCTTGCATCGATGAAGAGCGCAGCGAAATGCGATACGTGGTGC-GAATTGCAGAATCCCGTGAACC-ATCGAGTTTTTGAACGCAAGTTGCGCCCGAGGCCACC-TGGCCAAGGGCACGTCCACCTG---GGCGTCAA-GCATTAAATCGCTCT

Habenaria_linguella_SG1195 AGGAC-TCTCGGCAATGGATATCTT-GGCTCTTGCATCGATGAAGAGCGCAGCGAAATGCGATACGTGGTGC-GAATTGCAGAATCCCGTGAACC-ATCGAGTTTTTGAACGCAAGTTGCGCCCGAGGCCACC-TGGCCAAGGGCACGTCCACCTG---GGCGTCAA-GCATTAAATCGCTCT

Habenaria_linguella_SG1299 AGGAC-TCTCGGCAATGGATATCTT-GGCTCTTGCATCGATGAAGAGCGCAGCGAAATGCGATACGTGGTGC-GAATTGCAGAATCCCGTGAACC-ATCGAGTTTTTGAACGCAAGTTGCGCCCGAGGCCACC-TGGCCAAGGGCACGTCCACCTG---GGCGTCAA-GCATTAAATCGCTCT

Habenaria_linguella_SG1300 AGGAC-TCTCGGCAATGGATATCTT-GGCTCTTGCATCGATGAAGAGCGCAGCGAAATGCGATACGTGGTGC-GAATTGCAGAATCCCGTGAACC-ATCGAGTTTTTGAACGCAAGTTGCGCCCGAGGCCACC-TGGCCAAGGGCACGTCCACCTG---GGCGTCAA-GCATTAAATCGCTCT

Habenaria_reniformis_PK12142 AGGAC-TCTCGGCAATGGATATCTT-GGCTCTTGCATCGATGAAGAGCGCAGCGAAATGCGATACGTGGTGC-GAATTGCAGAATCCCGTGAACC-ATCGAGTTTTTGAACGCAAGTTGCGCCTGAGGCCACC-TGGCCAAGGGCACGTCCACCTG---GGCGTCAA-GCATTAAATCGCTCA

Habenaria_reniformis_PK12143 AGGAC-TCTCGGCAATGGATATCTT-GGCTCTTGCATCGATGAAGAGCGCAGCGAAATGCGATACGTGGTGC-GAATTGCAGAATCCCGTGAACC-ATCGAGTTTTTGAACGCAAGTTGCGCCTGAGGCCACC-TGGCCAAGGGCACGTCCACCTG---GGCGTCAA-GCATTAAATCGCTCA

Habenaria_reniformis_PK12144 AGGAC-TCTCGGCAATGGATATCTT-GGCTCTTGCATCGATGAAGAGCGCAGCGAAATGCGATACGTGGTGC-GAATTGCAGAATCCCGTGAACC-ATCGAGTTTTTGAACGCAAGTTGCGCCTGAGGCCACC-TGGCCAAGGGCACGTCCACCTG---GGCGTCAA-GCATTAAATCGCTCA

Habenaria_reniformis_PK12145 AGGAC-TCTCGGCAATGGATATCTT-GGCTCTTGCATCGATGAAGAGCGCAGCGAAATGCGATACGTGGTGC-GAATTGCAGAATCCCGTGAACC-ATCGAGTTTTTGAACGCAAGTTGCGCCTGAGGCCACC-TGGCCAAGGGCACGTCCACCTG---GGCGTCAA-GCATTAAATCGCTCA

Habenaria_reniformis_PK12146 AGGAC-TCTCGGCAATGGATATCTT-GGCTCTTGCATCGATGAAGAGCGCAGCGAAATGCGATACGTGGTGC-GAATTGCAGAATCCCGTGAACC-ATCGAGTTTTTGAACGCAAGTTGCGCCTGAGGCCACC-TGGCCAAGGGCACGTCCACCTG---GGCGTCAA-GCATTAAATTGCTCA

Habenaria_reniformis_SG1296 AGGAC-TCTCGGCAATGGATATCTT-GGCTCTTGCATCGATGAAGAGCGCAGCGAAATGCGATACGTGGTGC-GAATTGCAGAATCCCGTGAACC-ATCGAGTTTTTGAACGCAAGTTGCGCCTGAGGCCACC-TGGCCAAGGGCACGTCCACCTG---GGCGTCAA-GCATTAAATCGCTCA

Habenaria_reniformis_SG1297 AGGAC-TCTCGGCAATGGATATCTT-GGCTCTTGCATCGATGAAGAGCGCAGCGAAATGCGATACGTGGTGC-GAATTGCAGAATCCCGTGAACC-ATCGAGTTTTTGAACGCAAGTTGCGCCTGAGGCCACC-TGGCCAAGGGCACGTCCACCTG---GGCGTCAA-GCATTAAATCGCTCA

Habenaria_rhodocheila_KJ460043 AGGAC-TCTCGGCAATGGATATCTT-GGCTCTTGCATCGATGAAGAGCGCAGCGAAATGCGATACGTGGTGC-GAATTGCAGAATCCCGTGAACC-ATCGAGTTTTTGAACGCAAGTTGCGCCTGAGGCCACC-TGGCCAAGGGCACGTCCACCTG---GGCGTCAA-GCATTAAATCGCTCT

Habenaria_rhodocheila_KR350167 AGGAC-TCTCGGCAATGGATATCTT-GGCTCTTGCATCGATGAAGAGCGCAGCGAAATGCGATACGTGGTGC-GAATTGCAGAATCCCGTGAACC-ATCGAGTTTTTGAACGCAAGTTGCGCCTGAGGCCACC-TGGCCAAGGGCACGTCCACCTG---GGCGTCAA-GCATTAAATCGCTCT

Habenaria_rhodocheila_KY966607 AGGAC-TCTCGGCAATGGATATCTT-GGCTCTTGCATCGATGAAGAGCGCAGCGAAATGCGATACGTGGTGC-GAATTGCAGAATCCCGTGAACC-ATCGAGTTTTTGAACGCAAGTTGCGCCTGAGGCCACC-TGGCCAAGGGCACGTCCACCTG---GGCGTCAA-GCATTAAATCGCTCT

Habenaria_rhodocheila_MF944315 AGGAC-TCTCGGCAATGGATATCTT-GGCTCTTGCATCGATGAAGAGCGCAGCGAAATGCGATACGTGGTGC-GAATTGCAGAATCCCGTGAACC-ATCGAGTTTTTGAACGCAAGTTGCGCCCGAGGCCACC-TGGCCAAGGGCACGTCCACCTG---GGCGTCAA-GCATTAAATCGCTCT

Habenaria_rhodocheila_PK12139 AGGAC-TCTCGGCAATGGATATCTT-GGCTCTTGCATCGATGAAGAGCGCAGCGAAATGCGATACGTGGTGC-GAATTGCAGAATCCCGTGAACC-ATCGAGTTTTTGAACGCAAGTTGCGCCTGAGGCCACC-TGGCCAAGGGCACGTCCACCTG---GGCGTCAA-GCATTAAATCGCTCT

Habenaria_rhodocheila_SG1289 AGGAC-TCTCGGCAATGGATATCTT-GGCTCTTGCATCGATGAAGAGCGCAGCGAAATGCGATACGTGGTGC-GAATTGCAGAATCCCGTGAACC-ATCGAGTTTTTGAACGCAAGTTGCGCCTGAGGCCACC-TGGCCAAGGGCACGTCCACCTG---GGCGTCAA-GCATTAAATCGCTCT

Habenaria_rhodocheila_SG1290 AGGAC-TCTCGGCAATGGATATCTT-GGCTCTTGCATCGATGAAGAGCGCAGCGAAATGCGATACGTGGTGC-GAATTGCAGAATCCCGTGAACC-ATCGAGTTTTTGAACGCAAGTTGCGCCTGAGGCCACC-TGGCCAAGGGCACGTCCACCTG---GGCGTCAA-GCATTAAATCGCTCT

Habenaria_rhodocheila_SG1291 AGGAC-TCTCGGCAATGGATATCTT-GGCTCTTGCATCGATGAAGAGCGCAGCGAAATGCGATACGTGGTGC-GAATTGCAGAATCCCGTGAACC-ATCGAGTTTTTGAACGCAAGTTGCGCCTGAGGCCACC-TGGCCAAGGGCACGTCCACCTG---GGCGTCAA-GCATTAAATCGCTCT

Habenaria_rodeiensis_HM777577 AGGAC-TCTCGGCAATGGATATCTT-GGCTCTCGCATCGATGAAGAGCGCAGCGAAATGCGATACGTGGTGC-GAATTGCAGAATCCCGTGAACC-ATCGAGTTTTTGAACGCAAGTTGCGCCCGAGGCCAGC-TGGCCAAGGGCACGTCCGCCTG---GGCGTCAA-ACATTGATTCGCTCC

Hetaeria_youngsayei_KT344095 ATGAC-TCTCGGCGATGGATATGTT-GCCTCTTGCATCGATGAACAGCGCAACGTACTGCAATACGTGGTGA-GAATTGCATAATCCCGTGAACC-ATCTCATCTATGAACGCTTGTTGCCCCCGACGCCTGT-TGACTGACGGCACGTCCGCCTG---GGCGTCAA-GC-TTACATCTCTTC

Hetaeria_youngsayei_KY966608 ATGAC-TCTCGGCAATGGATATCTT-GGCTCTTGCATCGATGAAGAGCGCAGCGAAATGCGATACGTGGTGT-GAATTGCAGAATCCCGTGAACC-ATCAAATCTTTGAACGCAAGTTGCGCCCGAGGCCAAT-TGGCTAAGGGCACGTCCGCCTG---GGCGTCAA-GCATTACATCGCTTC

Hetaeria_youngsayei_SG1244 ATGAC-TCTCGGCAATGGATATCTT-GGCTCTTGCATCGATGAAGAGCGCAGCGAAATGCGATACGTGGTGT-GAATTGCAGAATCCCGTGAACC-ATCAAATCTTTGAACGCAAGTTGCGCCCGAGGCCAAT-TGGCTAAGGGCACGTCCGCCTG---GGCGTCAA-GCATTACATCGCTTC

Hetaeria_youngsayei_SG1245 ATGAC-TCTCGGCAATGGATATCTT-GGCTCTTGCATCGATGAAGAGCGCAGCGAAATGCGATACGTGGTGT-GAATTGCAGAATCCCGTGAACC-ATCAAATCTTTGAACGCAAGTTGCGCCCGAGGCCAAT-TGGCTAAGGGCACGTCCGCCTG---GGCGTCAA-GCATTACATCGCTTC

Hypoxis_rigidula_SG1207 ACGAC-TCTCGGCAATGGATATCTC-GGCTCTCGCATCGATGAAGAGCGCAGCGAAATGCGATACGTGGTGC-GAATTGCAGAATCCCGCGAACC-ATCGAGTCTTTGAACGCAAGTTGCGCCTGAGGCCAAC-CGGCCGAGGGCACGTCTGCCTG---GGCGTCAG-GCGTTACGTCGCTCC

Lecanorchis_nigricans_FJ425829 AAGAC-TCTCGGCAACGGATATCTT-GGCTCTTGCATCGATGAAGAACGCAGCGAAATGCGATATGTGTTGT-GAATTGCAGAATCCCGTGAACC-ATCCAGTCTTTGAACGCAAGTCGCGCCCAAGGTTGCA--CGCCGAGGGCACGTCTGCATG---GGTGTGAT-GCGTTAAGTCGCTCT

Lecanorchis_nigricans_SG1279 AAGAC-TCTCGGCAACGGATATCTT-GGCTCTTGCATCGATGAAGAACGCAGCGAAATGCGATATGTGTTGT-GAATTGCAGAATCCCGTGAACC-ATCCAGTCTTTGAACGCAAGTCGCGCCCAAGGTTGCA--CGCCGAGGGCACGTCTGCATG---GGTGTGAT-GCGTTAAGTCGCTCT

Lecanorchis_nigricans_SG1280 AAGAC-TCTCGGCAACGGATATCTT-GGCTCTTGCATCGATGAAGAACGCAGCGAAATGCGATATGTGTTGT-GAATTGCAGAATCCCGTGAACC-ATCCAGTCTTTGAACGCAAGTCGCGCCCAAGGTTGCA--CGCCGAGGGCACGTCTGCATG---GGTGTGAT-GCGTTAAGTCGCTCT

Liparis_bootanensis_KFBG238 ACGAC-TCTCGGCAATGGATATCTC-GGCTCTTGCATCGATGAAGAGCGCAGCGAAATGCGATACGTGGTGC-GAATTGCAGAATCCCGCGAACC-ATCGAGTCTTTGAACGCAAGTTGCGCCCGAGGCCAAC-CGGCCAAGGGCACGTTTGCCTG---GGCGTCAA-GCGTTGCGTCGCTTC

Liparis_bootanensis_KJ459280 ACGAC-TCTCGGCAATGGATATCTC-GGCTCTTGCATCGATGAAGAGCGCAGCGAAATGCGATACGTGGTGC-GAATTGCAGAATCCCGCGAACC-ATCGAGTCTTTGAACGCAAGTTGCGCCCGAGGCCAAC-CGGCCAAGGGCACGTTTGCCTG---GGCGTCAA-GCGTTGCGTCGCTTC

Liparis_bootanensis_KY966611 ACGAC-TCTCGGCAATGGATATCTC-GGCTCTTGCATCGATGAAGAGCGCAGCGAAATGCGATACGTGGTGC-GAATTGCAGAATCCCGCGAACC-ATCGAGTCTTTGAACGCAAGTTGCGCCCGAGGCCAAC-CGGCCAAGGGCACGTTTGCCTG---GGCGTCAA-GCGTTGCGTCGCTTC

Liparis_bootanensis_PK12162 ACGAC-TCTCGGCAATGGATATCTC-GGCTCTTGCATCGATGAAGAGCGCAGCGAAATGCGATACGTGGTGC-GAATTGCAGAATCCCGCGAACC-ATCGAGTCTTTGAACGCAAGTTGCGCCCGAGGCCAAC-CGGCCAAGGGCACGTTTGCCTG---GGCGTCAA-GCGTTGCGTCGCTTC

Liparis_bootanensis_SG1215 ACGAC-TCTCGGCAATGGATATCTC-GGCTCTTGCATCGATGAAGAGCGCAGCGAAATGCGATACGTGGTGC-GAATTGCAGAATCCCGCGAACC-ATCGAGTCTTTGAACGCAAGTTGCGCCCGAGGCCAAC-CGGCCGAGGGCACGTTTGCCTG---GGCGTCAA-GCGTTGCGTCGCTTC

Liparis_bootanensis_SG1216 ACGAC-TCTCGGCAATGGATATCTC-GGCTCTTGCATCGATGAAGAGCGCAGCGAAATGCGATACGTGGTGC-GAATTGCAGAATCCCGCGAACC-ATCGAGTCTTTGAACGCAAGTTGCGCCCGAGGCCAAC-CGGCCGAGGGCACGTTTGCCTG---GGCGTCAA-GCGTTGCGTCGCTTC

Liparis_bootanensis_SG1307 ACGAC-TCTCGGCAATGGATATCTC-GGCTCTTGCATCGATGAAGAGCGCAGCGAAATGCGATACGTGGTGC-GAATTGCAGAATCCCGCGAACC-ATCGAGTCTTTGAACGCAAGTTGCGCCCGAGGCCAAC-CGGCCAAGGGCACGTTTGCCTG---GGCGTCAA-GCGTTGCGTCGCTTC

Liparis_bootanensis_SG1336 ACGAC-TCTCGGCAATGGATATCTC-GGCTCTTGCATCGATGAAGAGCGCAGCGAAATGCGATACGTGGTGC-GAATTGCAGAATCCCGCGAACC-ATCGAGTCTTTGAACGCAAGTTGCGCCCGAGGCCAAC-CGGCCAAGGGCACGTTTGCCTG---GGCGTCAA-GCGTTGCGTCGCTTC

Liparis_ferruginea_SG1156 ATGAC-TCTCGGCAATGGATATCTC-GGCTCTTGCATCGATGAAGAGCGCAGCAAAATGCGATACGTGATGC-GAATTGCAGAACCCCGCGAACC-ATCGAGTCTTTGAACGCAAGTTGCGCCCGAGGCCAAT-CGGTCAAGGGCACGCCTTCCTG---GGTGTCAA-GCGTTGCATCGCTTT

Liparis_ferruginea_SG1266 ATGAC-TCTCGGCAATGGATATCTC-GGCTCTTGCATCGATGAAGAGCGCAGCAAAATGCGATACGTGATGC-GAATTGCAGAACCCCGCGAACC-ATCGAGTCTTTGAACGCAAGTTGCGCCCGAGGCCAAT-CGGTCAAGGGCACGCCTTCCTG---GGTGTCAA-GCGTTGCATCGCTTT

Liparis_ferruginea_SG1267 ATGAC-TCTCGGCAATGGATATCTC-GGCTCTTGCATCGATGAAGAGCGCAGCAAAATGCGATACGTGATGC-GAATTGCAGAACCCCGCGAACC-ATCGAGTCTTTGAACGCAAGTTGCGCCCGAGGCCAAT-CGGTCAAGGGCACGCCTTCCTG---GGTGTCAA-GCGTTGCATCGCTTT

Liparis_gigantea_PK12116 ATGAC-TCTCGGCAATGGATATCTC-GGCTCTTGCATCGATGAAGAGCGCAGCAAAATGCGATACGTGATGC-GAATTGCAGAATCCCGCGAACC-ATCGAGTCTTTGAACGCAAGTTGCGCCCGAGGCCAAC-CGGTCAAGGGCACGTTTTCCTG---GGTGTCAA-GCGTTGCTTCGCTTT

Liparis_gigantea_PK12117 ATGAC-TCTCGGCAATGGATATCTC-GGCTCTTGCATCGATGAAGAGCGCAGCAAAATGCGATACGTGATGC-GAATTGCAGAATCCCGCGAACC-ATCGAGTCTTTGAACGCAAGTTGCGCCCGAGGCCAAC-CGGTCAAGGGCACGTTTTCCTG---GGTGTCAA-GCGTTGCTTCGCTTT

Liparis_gigantea_PK12118 ATGAC-TCTCGGCAATGGATATCTC-GGCTCTTGCATCGATGAAGAGCGCAGCAAAATGCGATACGTGATGC-GAATTGCAGAATCCCGCGAACC-ATCGAGTCTTTGAACGCAAGTTGCGCCCGAGGCCAAC-CGGTCAAGGGCACGTTTTCCTG---GGTGTCAA-GCGTTGCTTCGCTTT

Liparis_nervosa_AB289482 ATGAC-TCTCGGCAATGGATATNTC-GGCTCTTGCATCGATGAAGAGCGCAGCAAAATGCGATACGTGATGCGGAATTGCAGAATCCCGCGAACC-ATCGAGTCTTTGAACGCAAGTTGCGCCCGAGGCCAAC-CGGTCAAGGGCACGTTTTCCTG---GGTGTCAA-GCGTTGCTTCGCTTT

Liparis_nervosa_AY907092 ATGAC-TCTCGGCAATGGATATCTC-GGCTCTTGCATCGATGAAGAGCGCAGCAAAATGCGATACGTGATGC-GAATTGCAGAATCCCGCGAACC-ATCGAGTCTTTGAACGCAAGTTGCGCCCGAGGCCAAC-CGGTCAAGGGCACGTTTTCCTG---GGTGTCAA-GCGTTGCTTCGCTTT

Liparis_nervosa_JN114595 ATGAC-TCTCGGCAATGGATATCTC-GGCTCTTGCATCGATGAAGAGCGCAGCAAAATGCGATACGTGATGC-GAATTGCAGAATCCCGCGAACC-ATCGAGTCTTTGAACGCAAGTTGCGCCCGAGGCCAAC-CGGTCAAGGGCACGTTTTCCTG---GGTGTCAA-GCGTTGCTTCGCTTT

Liparis_nervosa_JN114596 ATGAC-TCTCGGCAATGGATATCTC-GGCTCTTGCATCGATGAAGAGCGCAGCAAAATGCGATACGTGATGC-GAATTGCAGAATCCCGCGAACC-ATCGAGTCTTTGAACGCAAGTTGCGCCCGAGGCCAAC-CGGTCAAGGGCACGTTTTCCTG---GGTGTCAA-GCGTTGCTTCGCTTT

Liparis_nervosa_JN114597 ATGAC-TCTCGGCAATGGATATCTC-GGCTCTTGCATCGATGAAGAGCGCAGCAAAATGCGATACGTGATGC-GAATTGCAGAATCCCGCGAACC-ATCGAGTCTTTGAACGCAAGTTGCGCCCGAGGCCAAC-CGGTCAAGGGCACGTTTTCCTG---GGTGTCAA-GCGTTGCTTCGCTTT

Liparis_nervosa_JN114598 ATGAC-TCTCGGCAATGGATATCTC-GGCTCTTGCATCGATGAAGAGCGCAGCAAAATGCGATACGTGATGC-GAATTGCAGAATCCCGCGAACC-ATCGAGTCTTTGAACGCAAGTTGCGCCCGAGGCCAAC-CGGTCAAGGGCACGTTTTCCTG---GGTGTCAA-GCGTTGCTTCGCTTT

Liparis_nervosa_JN114599 ATGAC-TCTCGGCAATGGATATCTC-GGCTCTTGCATCGATGAAGAGCGCAGCAAAATGCGATACGTGATGC-GAATTGCAGAATCCCGCGAACC-ATCGAGTCTTTGAACGCAAGTTGCGCCCGAGGCCAAC-CGGTCAAGGGCACGTTTTCCTG---GGTGTCAA-GCGTTGCTTCGCTTT

Liparis_nervosa_JN114600 ATGAC-TCTCGGCAATGGATATCTC-GGCTCTTGCATCGATGAAGAGCGCAGCAAAATGCGATACGTGATGC-GAATTGCAGAATCCCGCGAACC-ATCGAGTCTTTGAACGCAAGTTGCGCCCGAGGCCAAC-CGGTCAAGGGCACGTTTTCCTG---GGTGTCAA-GCGTTGCTTCGCTTT

Liparis_nervosa_JN114601 ATGAC-TCTCGGCAATGGATATCTC-GGCTCTTGCATCGATGAAGAGCGCAGCAAAATGCGATACGTGATGC-GAATTGCAGAATCCCGCGAACC-ATCGAGTCTTTGAACGCAAGTTGCGCCCGAGGCCAAC-CGGTCAAGGGCACGTTTTCCTG---GGTGTCAA-GCGTTGCTTCGCTTT

Liparis_nervosa_JN114602 ATGAC-TCTCGGCAATGGATATCTC-GGCTCTTGCATCGATGAAGAGCGCAGCAAAATGCGATACGTGATGC-GAATTGCAGAATCCCGCGAACC-ATCGAGTCTTTGAACGCAAGTTGCGCCCGAGGCCAAC-CGGTCAAGGGCACGTTTTCCTG---GGTGTCAA-GCGTTGCTTCGCTTT

Liparis_nervosa_JN114603 ATGAC-TCTCGGCAATGGATATCTC-GGCTCTTGCATCGATGAAGAGCGCAGCAAAATGCGATACGTGATGC-GAATTGCAGAATCCCGCGAACC-ATCGAGTCTTTGAACGCAAGTTGCGCCCGAGGCCAAC-CGGTCAAGGGCACGTTTTCCTG---GGTGTCAA-GCGTTGCTTCGCTTT

Liparis_nervosa_JN114604 ATGAC-TCTCGGCAATGGATATCTC-GGCTCTTGCATCGATGAAGAGCGCAGCAAAATGCGATACGTGATGC-GAATTGCAGAATCCCGCGAACC-ATCGAGTCTTTGAACGCAAGTTGCGCCCGAGGCCAAC-CGGTCAAGGGCACGTTTTCCTG---GGTGTCAA-GCGTTGCTTCGCTTT

Liparis_nervosa_KFBG330 ATGAC-TCTCGGCAATGGATATCTC-GGCTCTTGCATCGATGAAGAGCGCAGCAAAATGCGATACGTGATGC-GAATTGCAGAATCCCGCGAACC-ATCGAGTCTTTGAACGCAAGTTGCGCCCGAGGCCAAC-CGGTCAAGGGCACGTTTTCCTG---GGTGTCAA-GCGTTGCTTCGCTTC

Liparis_nervosa_KJ459294 ATGAC-TCTCGGCAATGGATATNTC-GGCTCTTGCATCGATGAAGAGCGCAGCAAAATGCGATACGTGATGCGGAATTGCAGAATCCCGCGAACC-ATCGAGTCTTTGAACGCAAGTTGCGCCCGAGGCCAAC-CGGTCAAGGGCACGTTTTCCTG---GGTGTCAA-GCGTTGCTTCGCTTT

Liparis_nervosa_KT338746 ATGAC-TCTCGGCAATGGATATCTC-GGCTCTTGCATCGATGAAGAGCGCAGCAAAATGCGATACGTGATGC-GAATTGCAGAATCCCGCGAACC-ATCGAGTCTTTGAACGCAAGTTGCGCCCGAGGCCAAC-CGGTCAAGGGCACGTTTTCCTG---GGTGTCAA-GCGTTGCTTCGCTTT

Liparis_nervosa_KT338747 ATGAC-TCTCGGCAATGGATATCTC-GGCTCTTGCATCGATGAAGAGCGCAGCAAAATGCGATACGTGATGC-GAATTGCAGAATCCCGCGAACC-ATCGAGTCTTTGAACGCAAGTTGCGCCCGAGGCCAAC-CGGTCAAGGGCACGTTTTCCTG---GGTGTCAA-GCGATGCTTCGCTTT

Liparis_nervosa_SG1233 ATGAC-TCTCGGCAATGGATATCTC-GGCTCTTGCATCGATGAAGAGCGCAGCAAAATGCGATACGTGATGC-GAATTGCAGAATCCCGCGAACC-ATCGAGTCTTTGAACGCAAGTTGCGCCCGAGGCCAAC-CGGTCAAGGGCACGTTTTCCTG---GGTGTCAA-GCGTTGCTTCGCTTC

Liparis_nervosa_SG1234 ATGAC-TCTCGGCAATGGATATCTC-GGCTCTTGCATCGATGAAGAGCGCAGCAAAATGCGATACGTGATGC-GAATTGCAGAATCCCGCGAACC-ATCGAGTCTTTGAACGCAAGTTGCGCCCGAGGCCAAC-CGGTCAAGGGCACGTTTTCCTG---GGTGTCAA-GCGTTGCTTCGCTTC

Liparis_nervosa_SG1235 ATGAC-TCTCGGCAATGGATATCTC-GGCTCTTGCATCGATGAAGAGCGCAGCAAAATGCGATACGTGATGC-GAATTGCAGAATCCCGCGAACC-ATCGAGTCTTTGAACGCAAGTTGCGCCCGAGGCCAAC-CGGTCAAGGGCACGTTTTCCTG---GGTGTCAA-GCGTTGCTTCGCTTC

Liparis_odorata_KJ021033 ATGAC-TCTCGGCAATGGATATCTC-GGCTCTTGCATCGATGAAGAGCGCAGCAAAATGCGATACGTGATGC-GAATTGCAGAATCCCGCGAACC-ATCGAGTCTTTGAACGCAAGTTGCGCCCGAGGCCAAC-CGGTCAAGGGCACGTTTTCCTG---GGTGTCAA-GCGTTGCTTCGCTTC

Liparis_odorata_SG1256 ACGAC-TCTCGGCAATGGATATCTC-GGCTCTTGCATCGATGAAGAGCGCAGCAAAATGCGATACGTGATGC-GAATTGCAGAACCCCGCGAACC-ATCGAGTCTTTGAACGCAAGTTGCGCCCGAGGCCAAC-CGGTCAAGGGCACGCTTTCCTG---GGCGTCAA-GCGTTGCATCGCTTT

Liparis_odorata_SG1257 ACGAC-TCTCGGCAATGGATATCTC-GGCTCTTGCATCGATGAAGAGCGCAGCAAAATGCGATACGTGATGC-GAATTGCAGAACCCCGCGAACC-ATCGAGTCTTTGAACGCAAGTTGCGCCCGAGGCCAAC-CGGTCAAGGGCACGCTTTCCTG---GGCGTCAA-GCGTTGCATCGCTTT

Liparis_sootenzanensis_KJ021034 ACGAC-TCTCGGCAATGGATATCTC-GGCTCTTGCATCGATGAAGAGCGCAGCAAAATGCGATACGTGATGC-GAATTGCAGAATCCCGCGAACC-ATCGAGTCTTTGAACGCAAGTTGCGCCCGAGGCCAAC-CGGTCAAGGGCACGCTTTCCTG---GGTGTCAA-GCGTTGCATCGCTTT

Liparis_sootenzanensis_SG1351 ACGAC-TCTCGGCAATGGATATCTC-GGCTCTTGCATCGATGAAGAGCGCAGCAAAATGCGATACGTGATGC-GAATTGCAGAATCCCGCGAACC-ATCGAGTCTTTGAACGCAAGTTGCGCCCGAGGCCAAC-CGGTCAAGGGCACGCTTTCCTG---GGTGTCAA-GCGTTGCATCGCTTT

Liparis_sootenzanensis_SG1352 ACGAC-TCTCGGCAATGGATATCTC-GGCTCTTGCATCGATGAAGAGCGCAGCAAAATGCGATACGTGATGC-GAATTGCAGAATCCCGCGAACC-ATCGAGTCTTTGAACGCAAGTTGCGCCCGAGGCCAAC-CGGTCAAGGGCACGCTTTCCTG---GGTGTCAA-GCGTTGCATCGCTTT

Liparis_stricklandiana_KF589873 ACGAC-TCTCGGCAATGGATATCTC-GGCTCTTGCATCGATGAAGAGCGCAGCGAAATGCGATACGTGGTGC-GAATTGCAGAATCCCGCGAACC-ATCGAGTCTTTGAACGCAAGTTGCGCCCGAGGCCAAC-CGGCCAAGGGCACGTTTGCCTG---GGCGTCAA-GCGTTGCGTCGCTYC

Liparis_stricklandiana_KFBG124 ACGAC-TCTCGGCAATGGATATCTC-GGCTCTTGCATCGATGAAGAGCGCAGCGAAATGCGATACGTGGTGC-GAATTGCAGAATCCCGCGAACC-ATCGAGTCTTTGAACGCAAGTTGCGCCCGAGGCCAAC-CGGCCAAGGGCACGTTTGCCTG---GGCGTCAA-GCGTTGCGTCGCTTC

Liparis_stricklandiana_KFBG818 ACGAC-TCTCGGCAATGGATATCTC-GGCTCTTGCATCGATGAAGAGCGCAGCGAAATGCGATACGTGGTGC-GAATTGCAGAATCCCGCGAACC-ATCGAGTCTTTGAACGCAAGTTGCGCCCGAGGCCAAC-CGGCCAAGGGCACGTTTGCCTG---GGCGTCAA-GCGTTGCGTCGCTCC

Liparis_stricklandiana_KJ459298 ACGAC-TCTCGGCAATGGATATCTC-GGCTCTTGCATCGATGAAGAGCGCAGCGAAATGCGATACGTGGTGC-GAATTGCAGAATCCCGCGAACC-ATCGAGTCTTTGAACGCAAGTTGCGCCCGAGGCCAAC-CGGCCAAGGGCACGTTTGCCTG---GGCGTCAA-GCGTTGCGTCGCTTC

Liparis_stricklandiana_KY966613 ACGAC-TCTCGGCAATGGATATCTC-GGCTCTTGCATCGATGAAGAGCGCAGCGAAATGCGATACGTGGTGC-GAATTGCAGAATCCCGCGAACC-ATCGAGTCTTTGAACGCAAGTTGCGCCCGAGGCCAAC-CGGCCAAGGGCACGTTTGCCTG---GGCGTCAA-GCGTTGCGTCGCTCC

Liparis_stricklandiana_KY966614 ACGAC-TCTCGGCAATGGATATCTC-GGCTCTTGCATCGATGAAGAGCGCAGCGAAATGCGATACGTGGTGC-GAATTGCAGAATCCCGCGAACC-ATCGAGTCTTTGAACGCAAGTTGCGCCCGAGGCCAAC-CGGCCAAGGGCACGTTTGCCTG---GGCGTCAA-GCGTTGCGTCGCTTC

Liparis_stricklandiana_PK12091 ACGAC-TCTCGGCAATGGATATCTC-GGCTCTTGCATCGATGAAGAGCGCAGCGAAATGCGATACGTGGTGC-GAATTGCAGAATCCCGCGAACC-ATCGAGTCTTTGAACGCAAGTTGCGCCCGAGGCCAAC-CGGCCAAGGGCACGTTTGCCTG---GGCGTCAA-GCGTTGCGTCGCTTC

Liparis_stricklandiana_SG1332 ACGAC-TCTCGGCAATGGATATCTC-GGCTCTTGCATCGATGAAGAGCGCAGCGAAATGCGATACGTGGTGC-GAATTGCAGAATCCCGCGAACC-ATCGAGTCTTTGAACGCAAGTTGCGCCCGAGGCCAAC-CGGCCAAGGGCACGTTTGCCTG---GGCGTCAA-GCGTTGCGTCGCTTC

Liparis_stricklandiana_SG1333 ACGAC-TCTCGGCAATGGATATCTC-GGCTCTTGCATCGATGAAGAGCGCAGCGAAATGCGATACGTGGTGC-GAATTGCAGAATCCCGCGAACC-ATCGAGTCTTTGAACGCAAGTTGCGCCCGAGGCCAAC-CGGCCAAGGGCACGTTTGCCTG---GGCGTCAA-GCGTTGCGTCGCTYC

Liparis_stricklandiana_SG1337 ACGAC-TCTCGGCAATGGATATCTC-GGCTCTTGCATCGATGAAGAGCGCAGCGAAATGCGATACGTGGTGC-GAATTGCAGAATCCCGCGAACC-ATCGAGTCTTTGAACGCAAGTTGCGCCCGAGGCCAAC-CGGCCAAGGGCACGTTTGCCTG---GGCGTCAA-GCGTTGCGTCGCTTC

Liparis_viridiflora_AY907107 ACGAC-TCTCGGCAATGGATATCTC-GGCTCTTGCATCGATGAAGAGCGCAGCGAAATGCGATACGTGGTGC-GAATTGCAGAATCCCGCGAACC-ATCGAGTATTTGAACGCAAGTTGCGCCCGAGGCCAAC-CGGCCGAGGGCACGTCCGCCTG---GGCGTCAA-GCGTCGCGTCGCTTC

Liparis_viridiflora_KJ459299 ACGAC-TCTCGGCAATGGATATCTC-GGCTCTTGCATCGATGAAGAGCGCAGCGAAATGCGATACGTGGTGC-GAATTGCAGAATCCCGCGAACC-ATCGAGTCTTTGAACGCAAGTTGCGCCCGAGGCCAAC-CGGCCAAGGGCACGTTTGCCTG---GGCGTCAA-GCGTTGCGTCGCTTC

Liparis_viridiflora_KY966615 ACGAC-TCTCGGCAATGGATATCTC-GGCTCTTGCATCGATGAAGAGCGCAGCGAAATGCGATACGTGGTGC-GAATTGCAGAATCCCGCGAACC-ATCGAGTCTTTGAACGCAAGTTGCGCCCGAGGCCAAC-CGGCCAAGGGCACGTTTGCCTG---GGCGTCAA-GCGTTGCGTCGCTTC

Liparis_viridiflora_KY966616 ACGAC-TCTCGGCAATGGATATCTC-GGCTCTTGCATCGATGAAGAGCGCAGCGAAATGCGATACGTGGTGC-GAATTGCAGAATCCCGCGAACC-ATCGAGTCTTTGAACGCAAGTTGCGCCCGAGGCCAAC-CGGCCAAGGGCACGTTTGCCTG---GGCGTCAA-GCGTTGCGTCGCTTC

Liparis_viridiflora_PK12120 ACGAC-TCTCGGCAATGGATATCTC-GGCTCTTGCATCGATGAAGAGCGCAGCGAAATGCGATACGTGGTGC-GAATTGCAGAATCCCGCGAACC-ATCGAGTCTTTGAACGCAAGTTGCGCCCGAGGCCAAC-CGGCCAAGGGCACGTTTGCCTG---GGCGTCAA-GCGTTGCGTCGCTTC

Liparis_viridiflora_SG1308 ACGAC-TCTCGGCAATGGATATCTC-GGCTCTTGCATCGATGAAGAGCGCAGCGAAATGCGATACGTGGTGC-GAATTGCAGAATCCCGCGAACC-ATCGAGTCTTTGAACGCAAGTTGCGCCCGAGGCCAAC-CGGCCAAGGGCACGTTTGCCTG---GGCGTCAA-GCGTTGCGTCGCTTC

Liparis_viridiflora_SG1338 ACGAC-TCTCGGCAATGGATATCTC-GGCTCTTGCATCGATGAAGAGCGCAGCGAAATGCGATACGTGGTGC-GAATTGCAGAATCCCGCGAACC-ATCGAGTCTTTGAACGCAAGTTGCGCCCGAGGCCAAC-CGGCCAAGGGCACGTTTGCCTG---GGCGTCAA-GCGTTGCGTCGCTTC

Ludisia_discolor_AJ539483 ATGAC-TCTCGGCAATGGATATCTT-GGCTCTTGCATCGATGAAGAGCGCAGCGAAATGCGATACGTGGTGT-GAATTGCAGAATCCCGTGAACC-ATCAAATCTTTGAACGCAAGTTGCGCCCGAGGCCATT-TGGCTAAGGGCACGTCCGCCTG---GGCGTCAA-GCATTACATCGCTTC

Ludisia_discolor_EF590781 -----------------------------TCTTGCATCGATGAAGAGCGCAGCGAAATGCGATACGTGGTGT-GAATTGCAGAATCCCGTGAACC-ATCAAATCTTTGAACGCAAGTTGCGCCCGAGGCCATT-TGGCTAAGGGCACGTCCGCCTG---GGCGTCAA-GCATTACATCGCTTC

Ludisia_discolor_EF590782 ----------------------------CTCTTGCATCGATGAAGAGCGCAGCGAAATGCGATACGTGGTGT-GAATTGCAGAATCCCGTGAACC-ATCAAATCTTTGAACGCAAGTTGCGCCCGAGGCCATT-TGGCTAAGGGCACGTCCGCCTG---GGCGTCAA-GCATTACATCGCTTC

Ludisia_discolor_JN166073 ATGAC-TCTCGGCAATGGATATCTT-GGCTCTTGCATCGATGAAGAGCGCAGCGAAATGCGATACGTGGTGT-GAATTGCAGAATCCCGTGAACC-ATCAAATCTTTGAACGCAAGTTGCGCCCGAGGCCATT-TGGCTAAGGGCACGTCCGCCTG---GGCGTCAA-GCATTACATCGCTTC

Ludisia_discolor_KR815834 ATGAC-TCTCGGCAATGGATATCTT-GGCTCTTGCATCGATGAAGAGCGCAGCGAAATGCGATACGTGGTGT-GAATTGCAGAATCCCGTGAACC-ATCAAATCTTTGAACGCAAGTTGCGCCCGAGGCCATT-TGGCTAAGGGCACGTCCGCCTG---GGCGTCAA-GCATTACATCGCTTC

Ludisia_discolor_KT344102 ATGAC-TCTCGGCAATGGATATCTT-GGCTCTTGCATCGATGAAGAGCGCAGCGAAATGCGATACGTGGTGT-GAATTGCAGAATCCCGTGAACC-ATCAAATCTTTGAACGCAAGTTGCGCCCGAGGCCATT-TGGCTAAGGGCACGTCCGCCTG---GGCGTCAA-GC-TTACATCGCTTA

Ludisia_discolor_KY966617 ATGAC-TCTCGGCAATGGATATCTT-GGCTCTTGCATCGATGAAGAGCGCAGCGAAATGCGATACGTGGTGT-GAATTGCAGAATCCCGTGAACC-ATCAAATCTTTGAACGCAAGTTGCGCCCGAGGCCATT-TGGCTAAGGGCACGTCCGCCTG---GGCGTCAA-GCATTACATCGCTTC

Ludisia_discolor_SG1236 ATGAC-TCTCGGCAATGGATATCTT-GGCTCTTGCATCGATGAAGAGCGCAGCGAAATGCGATACGTGGTGT-GAATTGCAGAATCCCGTGAACC-ATCAAATCTTTGAACGCAAGTTGCGCCCGAGGCCATT-TGGCTAAGGGCACGTCCGCCTG---GGCGTCAA-GCATTACATCGCTTC

Ludisia_discolor_SG1237 ATGAC-TCTCGGCAATGGATATCTT-GGCTCTTGCATCGATGAAGAGCGCAGCGAAATGCGATACGTGGTGT-GAATTGCAGAATCCCGTGAACC-ATCAAATCTTTGAACGCAAGTTGCGCCCGAGGCCATT-TGGCTAAGGGCACGTCCGCCTG---GGCGTCAA-GCATTACATCGCTTC

Ludisia_discolor_SG1348 ATGAC-TCTCGGCAATGGATATCTT-GGCTCTTGCATCGATGAAGAGCGCAGCGAAATGCGATACGTGGTGT-GAATTGCAGAATCCCGTGAACC-ATCAAATCTTTGAACGCAAGTTGCGCCCGAGGCCATT-TGGCTAAGGGCACGTCCGCCTG---GGCGTCAA-GCATTACATCGCTTC

Nephelaphyllum_tenuiflorum_KF560535 AAGAC-TCTCAACAATGGATATCTT-GGGTCTCGCATCGATGAAGAGCGCAGCGAAATGTGATATATGGTGT-GAATTGCAGAATCCCGCGAGCC-ATCGAGTCTTTGAACGCAAGTTGCGTCTGAGGCCAAT-AGGCCAAGGGCACGTCTGCCTG---GGCGTCAA-GCGTTTTGTCGCTCC

Nephelaphyllum_tenuiflorum_KM025159 AAGAC-TCTCAACAATGGATAACTT-GGGTCTCGCATCGATGAAGAGCGCAGCGAAATGTGATATATGGTGT-GAATTGCAGAATCCCGCGAGCC-ATCGAGTCTTTGAACGCAAGTTGCGTCTGAGGCCAAT-AGGCCAAGGGCACGTCTGCCTG---GGCGTCAA-GCGTTTTGTTGCTCT

Nephelaphyllum_tenuiflorum_KY966621 AAGAC-TCTCAACAATGGATATCTT-GGGTCTCGCATCGATGAAGAGCGCAGCGAAATGTGATATATGGTGT-GAATTGCAGAATCCCGCGAGCC-ATCGAGTCTTTGAACGCAAGTTGCGTCTGAGGCCAAT-AGGCCAAGGGCACGTCTGCCTG---GGCGTCAA-GCGTTTTGTTGCTCT

Nephelaphyllum_tenuiflorum_PK12121 AAGAC-TCTCAACAATGGATATCTT-GGGTCTCGCATCGATGAAGAGCGCAGCGAAATGTGATATATGGTGT-GAATTGCAGAATCCCGCGAGCC-ATCGAGTCTTTGAACGCAAGTTGCGTCTGAGGCCAAT-AGGCCAAGGGCACGTCTGCCTG---GGCGTCAA-GCGTTTTGTTGCTCT

Nephelaphyllum_tenuiflorum_PK12122 AAGAC-TCTCAACAATGGATATCTT-GGGTCTCGCATCGATGAAGAGCGCAGCGAAATGTGATATATGGTGT-GAATTGCAGAATCCCGCGAGCC-ATCGAGTCTTTGAACGCAAGTTGCGTCTGAGGCCAAT-AGGCCAAGGGCACGTCTGCCTG---GGCGTCAA-GCGTTTTGTTGCTCT

Nephelaphyllum_tenuiflorum_PK12123 AAGAC-TCTCAACAATGGATATCTT-GGGTCTCGCATCGATGAAGAGCGCAGCGAAATGTGATATATGGTGT-GAATTGCAGAATCCCGCGAGCC-ATCGAGTCTTTGAACGCAAGTTGCGTCTGAGGCCAAT-AGGCCAAGGGCACGTCTGCCTG---GGCGTCAA-GCGTTTTGTTGCTCT

Nephelaphyllum_tenuiflorum_SG1220 AAGAC-TCTCAACAATGGATATCTT-GGGTCTCGCATCGATGAAGAGCGCAGCGAAATGTGATATATGGTGT-GAATTGCAGAATCCCGCGAGCC-ATCGAGTCTTTGAACGCAAGTTGCGTCTGAGGCCAAT-AGGCCAAGGGCACGTCTGCCTG---GGCGTCAA-GCGTTTTGTTGCTCT

Nervilia_plicata_AF324179 ACGAC-TCTCGGCAATGGATATCTC-GGCTCTCGCATCGATGAAGAGCGCAGCGAAATGCGATACGTGGTGC-GAATTGCAGAATCCCGTGAACC-ATCGAGTCTTTGAACGCAAGTTGCGCCCGAGGCCCAC-CGGCCGAGGGCACGCCCGCCTG---GGCGTCAA-GCATCACGTCACTCC

Nervilia_plicata_JN114618 ACGAC-TCTCGGCAATGGATATCTC-GGCTCTCGCATCGATGAAGAGCGCAGCGAAATGCGATACGTGGTGC-GAATTGCAGAATCCCGTGAACC-ATCGAGTCTTTGAACGCAAGTTGCGCCCGAGGCCCAC-CGGCCGAGGGCACGCCCGCCTG---GGCGTCAA-GCACCGCGTCACTCC

Nervilia_plicata_JN114619 ACGAC-TCTCGGCAATGGATATCTC-GGCTCTCGCATCGATGAAGAGCGCAGCGAAATGCGATACGTGGTGC-GAATTGCAGAATCCCGTGAACC-ATCGAGTCTTTGAACGCAAGTTGCGCCCGAGGCCCAC-CGGCCGAGGGCACGCCCGCCTG---GGCGTCAA-GCACCGCGTCACTCC

Nervilia_plicata_JN114620 ACGAC-TCTCGGCAATGGATATCTC-GGCTCTCGCATCGATGAAGAGCGCAGCGAAATGCGATACGTGGTGC-GAATTGCAGAATCCCGTGAACC-ATCGAGTCTTTGAACGCAAGTTGCGCCCGAGGCCCAC-CGGCCGAGGGCACGCCCGCCTG---GGCGTCAA-GCACCGCGTCACTCC

Nervilia_plicata_MG452049 ACGAC-TCTCGGCAATGGATATCTC-GGCTCTCGCATCGATGAAGAGCGCAGCGAAATGCGATACGTGGTGC-GAATTGCAGAATCCCGTGAACC-ATCGAGTCTTTGAACGCAAGTTGCGCCCGAGGCCCAC-CGGCCGAGGGCACGCCCGCCTG---GGCGTCAA-GCACCGCGTCACTCC

Nervilia_plicata_SG1143 ACGAC-TCTCGGCAATGGATATCTC-GGCTCTCGCATCGATGAAGAGCGCAGCGAAATGCGATACGTGGTGC-GAATTGCAGAATCCCGTGAACC-ATCGAGTCTTTGAACGCAAGTTGCGCCCGAGGCCCAC-CGGCCGAGGGCACGCCCGCCTG---GGCGTCAA-GCACCGCATCACTCC

Nervilia_plicata_SG1277 ACGAC-TCTCGGCAATGGATATCTC-GGCTCTCGCATCGATGAAGAGCGCAGCGAAATGCGATACGTGGTGC-GAATTGCAGAATCCCGTGAACC-ATCGAGTCTTTGAACGCAAGTTGCGCCCGAGGCCCAC-CGGCCGAGGGCACGCCCGCCTG---GGCGTCAA-GCACCGCATCACTCC

Neuwiedia_zollingeri_var_singapureana_PK12124 ATGAC-TCTCGACAACGGATATCTT-GGCTCTTGCATCGATGAAGAACGCAGCGAAATGTGATATATGGTGT-GAATTGCAGAATCCCGTGAACC-ATCGAGTACTTGAACGCAAGTTGCGCCTGAGGCCAAG-TGGTTGATGGCACACCTGCCTGGTTGTCGTCGT-ATGTCGTCTCGCTCC

Neuwiedia_zollingeri_var_singapureana_SG1268 ATGAC-TCTCGACAACGGATATCTT-GGCTCTTGCATCGATGAAGAACGCAGCGAAATGTGATATATGGTGT-GAATTGCAGAATCCCGTGAACC-ATCGAGTACTTGAACGCAAGTTGCGCCTGAGGCCAAG-TGGTTGATGGCACACCTGCCTGGTTGTCGTCGT-ATGTCGTCTCGCTCC

Neuwiedia_zollingeri_var_singapureana_SG1269 ATGAC-TCTCGACAACGGATATCTT-GGCTCTTGCATCGATGAAGAACGCAGCGAAATGTGATATATGGTGT-GAATTGCAGAATCCCGTGAACC-ATCGAGTACTTGAACGCAAGTTGCGCCTGAGGCCAAG-TGGTTGATGGCACACCTGCCTGGTTGTCGTCGT-ATGTCGTCTCGCTCC

Neuwiedia_zollingeri_var_singapureana_SG1340 ATGAC-TCTCGACAACGGATATCTT-GGCTCTTGCATCGATGAAGAACGCAGCGAAATGTGATATATGGTGT-GAATTGCAGAATCCCGTGAACC-ATCGAGTACTTGAACGCAAGTTGCGCCTGAGGCCAAG-TGGTTGATGGCACACCTGCCTGGTTGTCGTCGT-ATGTCGTCTCGCTCC

Neuwiedia_zollingeri_var_singapureana_JF796932 ATGAC-TCTCGACAACGGATATCTT-GGCTCTTGCATCGATGAAGAACGCAGCGAAATGTGATATATGGTGT-GAATTGCAGAATCCCGTGAACC-ATCGAGTACTTGAACGCAAGTTGCGCCTGAGGCCAAG-TGGTTGATGGCACACCTGCCTGGTTGTCGTCG------TATGTCGTTTC

Neuwiedia_zollingeri_var_singapureana_KY966622 ATGAC-TCTCGACAACGGATATCTT-GGCTCTTGCATCGATGAAGAACGCAGCGAAATGTGATATATGGTGT-GAATTGCAGAATCCCGTGAACC-ATCGAGTACTTGAACGCAAGTTGCGCCTGAGGCCAAG-TGGTTGATGGCACACCTGCCTGGTTGTCGTCG------TATGTCGTCTC

Pachystoma_pubescens_PK12107 ACGAC-TCTCGGCAATGGATATCTC-GGCTCTCGCATCGATGAAGAGCGCAGCAAAATGCGATACGTGGTGC-GAATTGCAGAATCCCGCGAACC-ATCGAGTCTTTGAACGCAAGTTGCGCCCGAGGCCAAT-CGGCCAAGGGCACGTCCGCCTG---GGCGTCAA-GCGTCGCGTCGCTCC

Pachystoma_pubescens_PK12108 ACGAC-TCTCGGCAATGGATATCTC-GGCTCTCGCATCGATGAAGAGCGCAGCAAAATGCGATACGTGGTGC-GAATTGCAGAATCCCGCGAACC-ATCGAGTCTTTGAACGCAAGTTGCGCCCGAGGCCAAT-CGGCCAAGGGCACGTCCGCCTG---GGCGTCAA-GCGTCGCGTCGCTCC

Paphiopedilum_purpuratum_AJ564364 ACAAC-TCTCAGCAACGGATATCTC-GGCTCTTGCATCGATGAAGAACGCAGCGAAATGCGATAAATGGTGT-GAATTGAAGAATCCCGTGAACC-ATCGAGTCTTTGAACGCAAGTTGCGCCCGAGGCCATC-AGGCCAAGGGCACGCCTGCCTG---GGCATTGC-GAGTCATATCTCTCC

Paphiopedilum_purpuratum_EF156131 ACAAC-TCTCAGCAACGGATATCTC-GGCTCTTGCATCGATGAAGAACGCAGCGAAATGCGATAAATGGTGT-GAATTGCAGAATCCCGTGAACC-ATCGAGTCTTTGAACGCAAGTTACGCCCGAGGCCATC-AGGCCAAGGGCACGCCTGCCTG---GGCATTGC-GAGTCATATCTCTCC

Paphiopedilum_purpuratum_FJ899756 ACAAC-TCTCAGCAACGGATATCTC-GGCTCTTGCATCGATGAAGAACGCAGCGAAATGCGATAAATGGTGT-GAATTGCAGAATCCCGTGAACC-ATCGAGTCTTTGAACGCAAGTTGCGCCCGAGGCCATC-AGGCCAAGGGCACGCCTGCCTG---GGCATTGC-GAGTCATATCTCTCC

Paphiopedilum_purpuratum_GU993850 ACAAC-TCTCAGCAACGGATATCTC-GGCTCTAGCATCGATGAACAACGCAGCGAACTGCGATAAATGGTGT-GAATTGAATAATCCCGTGAACC-ATCGAGTCTTTGAACGCATGTTGCGCCCGAGGCCATC-ACGCCGAGGGCACGCCTGCCTG---GGCGTTGC-GAGTCATATCTCTCC

Paphiopedilum_purpuratum_JX088564 ACAAC-TCTCAGCAACGGATATCTC-GGCTCTTGCATCGATGAAGAACGCAGCGAAATGCGATAAATGGTGT-GAATTGCAGAATCCCGTGAACC-ATCGAGTCTTTGAACGCAAGTTGCGCCCGAGGCCATC-AGGCCAAGGGCACGCCTGCCTG---GGCATTGC-GAGTCATATCTCTCC

Paphiopedilum_purpuratum_KX931030 ACAAC-TCTCAGCAACGGATATCTC-GGCTCTTGCATCGATGAAGAACGCAGCGAAATGCGATAAATGGTGT-GAATTGCAGAATCCCGTGAACC-ATCGAGTCTTTGAACGCAAGTTGCGCCCGAGGCCATC-AGGCCAAGGGCACGCCTGCCTG---GGCATTGC-GAGTCATATCTCTCC

Paphiopedilum_purpuratum_PK12075 ACAAC-TCTCAGCAACGGATATCTC-GGCTCTTGCATCGATGAAGAACGCAGCGAAATGCGATAAATGGTGT-GAATTGCAGAATCCCGTGAACC-ATCGAGTCTTTGAACGCAAGTTGCGCCCGAGGCCATC-AGGCCAAGGGCACGCCTGCCTG---GGCATTGC-GAGTCATATCTCTCC

Paphiopedilum_purpuratum_PK12082A ACAAC-TCTCAGCAACGGATATCTC-GGCTCTTGCATCGATGAAGAACGCAGCGAAATGCGATAAATGGTGT-GAATTGCAGAATCCCGTGAACC-ATCGAGTCTTTGAACGCAAGTTGCGCCCGAGGCCATC-AGGCCAAGGGCACGCCTGCCTG---GGCATTGC-GAGTCATATCTCTCC

Paphiopedilum_purpuratum_PK12083C ACAAC-TCTCAGCAACGGATATCTC-GGCTCTTGCATCGATGAAGAACGCAGCGAAATGCGATAAATGGTGT-GAATTGCAGAATCCCGTGAACC-ATCGAGTCTTTGAACGCAAGTTGCGCCCGAGGCCATC-AGGCCAAGGGCACGCCTGCCTG---GGCATTGC-GAGTCATATCTCTCC

Paphiopedilum_purpuratum_SG1149 ACAAC-TCTCAGCAACGGATATCTC-GGCTCTTGCATCGATGAAGAACGCAGCGAAATGCGATAAATGGTGT-GAATTGCAGAATCCCGTGAACC-ATCGAGTCTTTGAACGCAAGTTGCGCCCGAGGCCATC-AGGCCAAGGGCACGCCTGCCTG---GGCATTGC-GAGTCATATCTCTCC

Paphiopedilum_purpuratum_Z78440 ACAAC-TCTCAGCAACGGATATCTC-AGCTCTTGCATCGTTGAAGAACCCACCGAAATGCGATAAATGGTGT-GAATTGCAGAATCCCGTGAACC-ATCGAGTCTTTGAACGCAAGTTGCGCCCGAGGCCATC-AGGCCAAGGGCACGCCTGCCTG---GGCATTGC-GAGTCATATCTCTCC

Pecteilis_susannae_MF944351 AGGAC-TCTCGGCAATGGATATCTT-GGCTCTTGCATCGATGAAGAGCGCAGCGAAATGCGATACGTGGTGC-GAATTGCAGAATCCCGTGAACC-ATCGAGTTTTTGAACGCAAGTTGCGCCTGAGGCCACC-TGGCCAAGGGCACGTCCACCTG---GGCGTCAA-GCATTAAATCGCTCT

Pecteilis_susannae_MF944352 AGGAC-TCTCGGCAATGGATATCTT-GGCTCTTGCATCGATGAAGAGCGCAGCGAAATGCGATACGTGGTGC-GAATTGCAGAATCCCGTGAACC-ATCGAGTTTTTGAACGCAAGTTGCGCCTGAGGCCACC-TGGCCAAGGGCACGTCCACCTG---GGCGTCAA-GCATTAAATCGCTCT

Pecteilis_susannae_PK12051 AGGAC-TCTCGGCAATGGATATCTT-GGCTCTTGCATCGATGAAGAGCGCAGCGAAATGCGATACGTGGTGC-GAATTGCAGAATCCCGTGAACC-ATCGAGTTTTTGAACGCAAGTTGCGCCTGAGGCCACC-TGGCCAAGGGCACGTCCACCTG---GGCGTCAA-GCATTAAATCGCTCT

Pecteilis_susannae_PK12136 AGGAC-TCTCGGCAATGGATATCTT-GGCTCTTGCATCGATGAAGAGCGCAGCGAAATGCGATACGTGGTGC-GAATTGCAGAATCCCGTGAACC-ATCGAGTTTTTGAACGCAAGTTGCGCCTGAGGCCACC-TGGCCAAGGGCACGTCCACCTG---GGCGTCAA-GCATTAAATCGCTCT

Pecteilis_susannae_PK12154 AGGAC-TCTCGGCAATGGATATCTT-GGCTCTTGCATCGATGAAGAGCGCAGCGAAATGCGATACGTGGTGC-GAATTGCAGAATCCCGTGAACC-ATCGAGTTTTTGAACGCAAGTTGCGCCTGAGGCCACC-TGGCCAAGGGCACGTCCACCTG---GGCGTCAA-GCATTAAATCGCTCT

Pecteilis_susannae_SG1292 AGGAC-TCTCGGCAATGGATATCTT-GGCTCTTGCATCGATGAAGAGCGCAGCGAAATGCGATACGTGGTGC-GAATTGCAGAATCCCGTGAACC-ATCGAGTTTTTGAACGCAAGTTGCGCCTGAGGCCACC-TGGCCAAGGGCACGTCCACCTG---GGCGTCAA-GCATTAAATCGCTCT

Peristylus_calcaratus_PK12052 AGGAC-TCTCGGCAATGGATATCTT-GGCTCTTGCATCGATGAAGAGCGCAGCGAAATGCGATACGTGGTGC-GAATTGCAGAATCCCGTGAACC-ATCGAGTATTTGAACGCAAGTTGCGCCCGAGGCCAGC-TGGTCGAGGGCACGTCCGCCTG---GGCGTCAA-GCATTGAATCGCCCC

Peristylus_calcaratus_PK12053 AGGAC-TCTCGGCAATGGATATCTT-GGCTCTTGCATCGATGAAGAGCGCAGCGAAATGCGATACGTGGTGC-GAATTGCAGAATCCCGTGAACC-ATCGAGTATTTGAACGCAAGTTGCGCCCGAGGCCAGC-TGGTCGAGGGCACGTCCGCCTG---GGCGTCAA-GCATTGAATCGCCCC

Peristylus_calcaratus_SG1303 AGGAC-TCTCGGCAATGGATATCTT-GGCTCTTGCATCGATGAAGAGCGCAGCGAAATGCGATACGTGGTGC-GAATTGCAGAATCCCGTGAACC-ATCGAGTATTTGAACGCAAGTTGCGCCCGAGGCCAGC-TGGTCGAGGGCACGTCCGCCTG---GGCGTCAA-GCATTGAATCGCCCC

Peristylus_densus_SG1258 AGGAC-TCTCGGCAATGGATATCTT-GGCTCTCGCATCGATGAAGAGCGCAGCGAAATGCGATACGTGGTGC-GAATTGCAGAATCCCGTGAACC-ATCGAGTATTTGAACGCAAGTTGCGCCCGAGGCCAGC-TGGCCGAGGGCACGTCCGACTG---GGCGTCAA-GCATTGAATCGCCCC

Peristylus_densus_SG1260 AGGAC-TCTCGGCAATGGATATCTT-GGCTCTCGCATCGATGAAGAGCGCAGCGAAATGCGATACGTGGTGC-GAATTGCAGAATCCCGTGAACC-ATCGAGTATTTGAACGCAAGTTGCGCCCGAGGCCAGC-TGGCCGAGGGCACGTCCGACTG---GGCGTCAA-GCATTGAATCGCCCC

Peristylus_goodyeroides_MF944361 AGGAC-TCTCGGCAATGGATATCTT-GGCTCTCGCATCGATGAAGAGCGCAGCGAAATGCGATACGTGGTGC-GAATTGCAGAATCCCGTGAACC-ATCGAGTTTTTGAACGCAAGTTGCGCCCGAGGCCAGCTTGGCCGAGGGCACGTCCGCCTG---GGCGTCAA-GCATTGAATCGCCCC

Peristylus_goodyeroides_MF944362 AGGAC-TCTCGGCAATGGATATCTT-GGCTCTCGCATCGATGAAGAGCGCAGCGAAATGCGATACGTGGTGC-GAATTGCAGAATCCCGTGAACC-ATCGAGTTTTTGAACGCAAGTTGCGCCCGAGGCCAGCTTGGCCGAGGGCACGTCCGCCTG---GGCGTCAA-GCATTGAATCGCCCC

Peristylus_intrudens_PK12050 AGGAC-TCTCGGCAATGGATATCTT-GGCTCTCGCATCGATGAAGAGCGCAGCGAAATGCGATACGTGGTGC-GAATTGCAGAATCCCGTGAACC-ATCGAGTTTTTGAACGCAAGTTGCGCCCGAGGCCAGC-TGGCCGAGGGCACGTCCGCCTG---GGCGTCAA-GCATTGAATCGCCCC

Peristylus_intrudens_PK12056 AGGAC-TCTCGGCAATGGATATCTT-GGCTCTCGCATCGATGAAGAGCGCAGCGAAATGCGATACGTGGTGC-GAATTGCAGAATCCCGTGAACC-ATCGAGTTTTTGAACGCAAGTTGCGCCCGAGGCCAGC-TGGCCGAGGGCACGTCCGCCTG---GGCGTCAA-GCGTTGAATCGCCCC

Peristylus_intrudens_SG1298 AGGAC-TCTCGGCAATGGATATCTT-GGCTCTCGCATCGATGAAGAGCGCAGCGAAATGCGATACGTGGTGC-GAATTGCAGAATCCCGTGAACC-ATCGAGTTTTTGAACGCAAGTTGCGCCCGAGGCCAGC-TGGCCGAGGGCACGTCCGCCTG---GGCGTCAA-GCATTGAATCGCCCC

Peristylus_lacertifer_MF944365 AGGAC-TCTCGGCAATGGATATCTT-GGCTCTCGCATCGATGAAGAGCGCAGCGAAATGCGATACGTGGTGC-GAATTGCAGAATCCCGTGAACC-ATCGAGTTTTTGAACGCAAGTTGCGCCCGAGGCCAGC-TGGCCGAGGGCACGTCCGCCTG---GGCGTCAA-GCATTGAATCGCCCC

Peristylus_lacertifer_MF944366 AGGAC-TCTCGGCAATGGATATCTT-GGCTCTCGCATCGATGAAGAGCGCAGCGAAATGCGATACGTGGTGC-GAATTGCAGAATCCCGTGAACC-ATCGAGTTTTTGAACGCAAGTTGCGCCCGAGGCCAGC-TGGCCGAGGGCACGTCCGCCTG---GGCGTCAA-GCATTGAATCGCCCC

Peristylus_lacertifer_PK12149 AGGAC-TCTCGGCAATGGATATCTT-GGCTCTCGCATCGATGAAGAGCGCAGCGAAATGCGATACGTGGTGC-GAATTGCAGAATCCCGTGAACC-ATCGAGTTTTTGAACGCAAGTTGCGCCCGAGGCCAGC-TGGCCGAGGGCACGTCCGCCTG---GGCGTCAA-GCATTGAATCGCCCC

Peristylus_lacertifer_PK12163 AGGAC-TCTCGGCAATGGATATCTT-GGCTCTCGCATCGATGAAGAGCGCAGCGAAATGCGATACGTGGTGC-GAATTGCAGAATCCCGTGAACC-ATCGAGTTTTTGAACGCAAGTTGCGCCCGAGGCCAGC-TGGCCGAGGGCACGTCCGCCTG---GGCGTCAA-GCATTGAATCGCCCC

Peristylus_lacertifer_SG1006 AGGAC-TCTCGGCAATGGATATCTT-GGCTCTCGCATCGATGAAGAGCGCAGCGAAATGCGATACGTGGTGC-GAATTGCAGAATCCCGTGAACC-ATCGAGTTTTTGAACGCAAGTTGCGCCCGAGGCCAGC-TGGCCGAGGGCACGTCCGCCTG---GGCGTCAA-GCATTGAATCGCCCC

Peristylus_tentaculatus_KJ460035 AGGAC-TCTCGGCAATGGATATCTT-GGCTCTCGCATCGATGAAGAGCGCAGCGAAATGCGATACGTGGTGC-GAATTGCAGAATCCCGTGAACC-ATCGAGTATTTGAACGCAAGTTGCGCCCGAGGCCAGC-TGGCCGAGGGCACGTCCGCCTG---GGCGTCAA-GCATTGAATCGCCCC

Persitylus_tentaculatus_PK12062 AGGAC-TCTCGGCAATGGATATCTT-GGCTCTCGCATCGATGAAGAGCGCAGCGAAATGCGATACGTGGTGC-GAATTGCAGAATCCCGTGAACC-ATCGAGTTTTTGAACGCAAGTTGCGCCCGAGGCCAGC-TGGCCGAGGGCACGTCCGCCTG---GGCGTCAA-GCATTGAATCGCCCC

Persitylus_tentaculatus_PK12171 AGGAC-TCTCGGCAATGGATATCTT-GGCTCTCGCATCGATGAAGAGCGCAGCGAAATGCGATACGTGGTGC-GAATTGCAGAATCCCGTGAACC-ATCGAGTTTTTGAACGCAAGTTGCGCCCGAGGCCAGC-TGGCCGAGGGCACGTCCGCCTG---GGCGTCAA-GCATTGAATCGCCCC

Persitylus_tentaculatus_SG1007 AGGAC-TCTCGGCAATGGATATCTT-GGCTCTCGCATCGATGAAGAGCGCAGCGAAATGCGATACGTGGTGC-GAATTGCAGAATCCCGTGAACC-ATCGAGTTTTTGAACGCAAGTTGCGCCCGAGGCCAGC-TGGCCGAGGGCACGTCCGCCTG---GGCGTCAA-GCATTGAATCGCCCC

Phaius_tancarvilleae_AB222032 ATGAC-TCTCGGCAATGGATATCTC-GGCTCTCGCATCGATGAAGAGCGCAGCGAAATGCGATACGTGGTGC-GAATTGCAGAATCCCGCGAACC-ATCGAGTCTTTGAACGCAAGTTGCGCCCGAGGTCAAC-CGGCCAAGGGCGCGTCTGCCTG---GGCGTCAA-GCGTTGCATCGCTCT

Phaius_tancarvilleae_AB239286 ATGAC-TCTCGGCAATGGATATCTC-GGCTCTCGCATCGATGAAGAGCGCAGCGAAATGCGATACGTGGTGC-GAATTGCAGAATCCCGCGAACC-ATCGAGTCTTTGAACGCAAGTTGCGCCCGAGGTCAAC-CGGCCAAGGGCACGTCTGCCTG---GGCGTCAA-GCGTTGCATCGCTCT

Phaius_tancarvilleae_AB239287 ATGAC-TCTCGGCAATGGATATCTC-GGCTCTCGCATCGATGAAGAGCGCAGCGAAATGCGATACGTGGTGC-GAATTGCAGAATCCCGCGAACC-ATCGAGTCTTTGAACGCAAGTTGCGCCCGAGGTCAAC-CGGCCAAGGGCACGTCTGCCTG---GGCGTCAA-GCGTTGCATCGCTCT

Phaius_tancarvilleae_AB239288 ATGAC-TCTCGGCAATGGATATCTC-GGCTCTCGCATCGATGAAGAGCGCAGCGAAATGCGATACGTGGTGC-GAATTGCAGAATCCCGCGAACC-ATCGAGTCTTTGAACGCAAGTTGCGCCCGAGGTCAAC-CGGCCAAGGGCACGTCTGCCTG---GGCGTCAA-GCGTTGCATCGCTCT

Phaius_tancarvilleae_AB239289 ATGAC-TCTCGGCAATGGATATCTC-GGCTCTCGCATCGATGAAGAGCGCAGCGAAATGCGATACGTGGTGC-GAATTGCAGAATCCCGCGAACC-ATCGAGTCTTTGAACGCAAGTTGCGCCCGAGGTCAAC-CGGCCAAGGGCACGTCTGCCTG---GGCGTCAA-GCGTTGCATCGCTCT

Phaius_tancarvilleae_JN114673 ATGAC-TCTCGGCAATGGATATCTC-GGCTCTCGCATCGATGAAGAGCGCAGCGAAATGCGATACGTGGTGC-GAATTGCAGAATCCCGCGAACC-ATCGAGTCTTTGAACGCAAGTTGCGCCTGAGGCCATC-CGGCCAAGGGCACGTCTGCCTG---GGCGTCAA-GCGTTGCGTCGCTTC

Phaius_tancarvilleae_KF560503 ATGAC-TCTCGGCAATGGATATCTC-GGCTCTCGCATCGATGAAGAGCGCAGCGAAATGCGATACGTGGTGC-GAATTGCAGAATCCCGCGAACC-ATCGAGTCTTTGAACGCAAGTTGCGCCCGAGGTCAAC-CGGCCAAGGGCACGTCTGCCTG---GGCGTCAA-GCGTTGCATCGCTCT

Phaius_tancarvilleae_KF560531 ATGAC-TCTCGGCAATGGATATCTC-GGCTCTCGCATCGATGAAGAGCGCAGCGAAATGCGATACGTGGTGC-GAATTGCAGAATCCCGCGAACC-ATCGAGTCTTTGAACGCAAGTTGCGCCCGAGGTCAAC-CGGCCAAGGGCACGTCTGCCTG---GGCGTCAA-GCGTTGCATCGCTCT

Phaius_tancarvilleae_KM025161 ATGAC-TCTCGGCAATGGATATCTC-GGCTCTCGCATCGATGAAGAGCGCAGCGAAATGCGATACGTGGTGC-GAATTGCAGAATCCCGCGAACC-ATCGAGTCTTTGAACGCAAGTTGCGCCCGAGGTCAAC-CGGCCAAGGGCACGTCTGCCTG---GGCGTCAA-GCGTTGCATCGCTCT

Phaius_tancarvilleae_KY966645 ATGAC-TCTCGGCAATGGATATCTC-GGCTCTCGCATCGATGAAGAGCGCAGCGAAATGCGATACGTGGTGC-GAATTGCAGAATCCCGCGAACC-ATCGAGTCTTTGAACGCAAGTTGCGCCCGAGGTCAAC-CGGCCAAGGGCACGTCTGCCTG---GGCGTCAA-GCGTTGCATCGCTCT

Phaius_tancarvilleae_MG869015 ATGAC-TCTCGGCAATGGATATCTC-GGCTCTCGCATCGATGAAGAGCGCAGCGAAATGCGATACGTGGTGC-GAATTGCAGAATCCCGCGAACC-ATCGAGTCTTTGAACGCAAGTTGCGCCCGAGGTCAAC-CGGCCAAGGGCACGTCTGCCTG---GGCGTCAA-GCGTTGCATCGCTCT

Phaius_tankervilleae_PK12084 ATGAC-TCTCGGCAATGGATATCTC-GGCTCTCGCATCGATGAAGAGCGCAGCGAAATGCGATACGTGGTGC-GAATTGCAGAATCCCGCGAACC-ATCGAGTCTTTGAACGCAAGTTGCGCCCGAGGTCAAC-CGGCCAAGGGCACGTCTGCCTG---GGCGTCAA-GCGTTGCATCGCTCT

Phaius_tankervilleae_PK12085 ATGAC-TCTCGGCAATGGATATCTC-GGCTCTCGCATCGATGAAGAGCGCAGCGAAATGCGATACGTGGTGC-GAATTGCAGAATCCCGCGAACC-ATCGAGTCTTTGAACGCAAGTTGCGCCCGAGGTCAAC-CGGCCAAGGGCACGTCTGCCTG---GGCGTCAA-GCGTTGCATCGCTCT

Phaius_tankervilleae_PK12099 ATGAC-TCTCGGCAATGGATATCTC-GGCTCTCGCATCGATGAAGAGCGCAGCGAAATGCGATACGTGGTGC-GAATTGCAGAATCCCGCGAACC-ATCGAGTCTTTGAACGCAAGTTGCGCCCGAGGTCAAC-CGGCCAAGGGCACGTCTGCCTG---GGCGTCAA-GCGTTGCATCGCTCT

Phaius_tankervilleae_PK12100 ATGAC-TCTCGGCAATGGATATCTC-GGCTCTCGCATCGATGAAGAGCGCAGCGAAATGCGATACGTGGTGC-GAATTGCAGAATCCCGCGAACC-ATCGAGTCTTTGAACGCAAGTTGCGCCCGAGGTCAAC-CGGCCAAGGGCACGTCTGCCTG---GGCGTCAA-GCGTTGCATCGCTCT

Phaius_wallichii_KF560532 ATGAC-TCTCGGCAATGGATATCTC-GGCTCTCGCATCGATGAAGAGCGCAGCGAAATGCGATACGTGGTGC-GAATTGCAGAATCCCGCGAACC-ATCGAGTCTTTGAACGCAAGTTGCGCCCGAGGTCAAC-CGGCCAAGGGCACGTCTGCCTG---GGCGTCAA-GCGTTGCATCGCTCT

Phaius_wallichii_KY966646 ATGAC-TCTCGGCAATGGATATCTC-GGCTCTCGCATCGATGAAAAGCGCAGCGAAATGCGATACGTGGTGC-GAATTGCAGAATCCCGCGAACC-ATCGAGTCTTTGAACGCAAGTTGCGCCCGAGGTCAAC-CGGCCAAGGGCACGTCTGCCTG---GGCGTCAA-GCGTTGCATCGCTCT

Platanthera_mandarinorum_JN696464 AGGAC-TCTCGACAATGGATATCTT-GGCTCTCGCATCGATGAAGAGCGCAGCGAAATGCGATACGTGGTGC-GAATTGCAGAATCCCGTGAACC-ATCGAGTTTTTGAACGCAAGTTGCGCCTGAGGCCAGC-TGGCCAAGGGCACGTCCGCCTG---GGCGTCAA-GCATTAAATCGCTCC

Platanthera_mandarinorum_subsp_mandarinorum_KT338772 AGGAC-TCTCGGCAATGGATATCTT-GGCTCTCGCATCGATGAAGAGCGCAGCGAAATGCGATACGTGGTGC-GAATTGCAGAATCCCGTGAACC-ATCGAGTTTTTGAACGCAAGTTGCGCCTGAGGCCAGC-TGGCCAAGGGCACGTCCGCCTG---GGCGTCAA-GCATTGAATCGCTCC

Platanthera_minor_KJ460069 AGGAC-TCTCGGCAATGGATATCTT-GGCTCTCGCATCGATGAAGAGCGCAGCGAAATGCGATACGTGGTGC-GAATTGCAGAATCCCGTGAACC-ATCGAGTTTTTGAACGCAAGTTGCGCCTGAGGCCAGC-TGGCCAAGGGCACGTCCGCCTG---GGCGTCAA-GCATTAAATCGCTCC

Platanthera_minor_KJ460079 AGGAC-TCTCGGCAATGGATATCTT-GGCTCTCGCATCGATGAAGAGCGCAGCGAAATGCGATACGTGGTGC-GAATTGCAGAATCCCGTGAACC-ATCGAGTTTTTGAACGCAAGTTGCGCCTGAGGCCAGC-TGGCCAAGGGCACGTCCGCCTG---GGCGTCAA-GCATTAAATCGCTCC

Platanthera_minor_PK12030 AGGGC-TCTCGGCAATGGATATCTT-GGCTCTCGCATCGATGAAGAGCGCAGCGAAATGCGATACGTGGTGC-GAATTGCAGAATCCCGTGAACC-ATCGAGTTTTTGAACGCAAGTTGCGCCTGAGGCCAGC-TGGCCAAGGGCACGTCCGCCTG---GGCGTCAT-GCATTAAATCGCTCC

Platanthera_minor_SG1154 AGGGC-TCTCGGCAATGGATATCTT-GGCTCTCGCATCGATGAAGAGCGCAGCGAAATGCGATACGTGGTGC-GAATTGCAGAATCCCGTGAACC-ATCGAGTTTTTGAACGCAAGTTGCGCCTGAGGCCAGC-TGGCCAAGGGCACGTCCGCCTG---GGCGTCAT-GCATTAAATCGCTCC

Platanthera_minor_SG1223 AGGGC-TCTCGGCAATGGATATCTT-GGCTCTCGCATCGATGAAGAGCGCAGCGAAATGCGATACGTGGTGC-GAATTGCAGAATCCCGTGAACC-ATCGAGTTTTTGAACGCAAGTTGCGCCTGAGGCCAGC-TGGCCAAGGGCACGTCCGCCTG---GGCGTCAT-GCATTAAATCGCTCC

Platanthera_minor_SG1224 AGGGC-TCTCGGCAATGGATATCTT-GGCTCTCGCATCGATGAAGAGCGCAGCGAAATGCGATACGTGGTGC-GAATTGCAGAATCCCGTGAACC-ATCGAGTTTTTGAACGCAAGTTGCGCCTGAGGCCAGC-TGGCCAAGGGCACGTCCGCCTG---GGCGTCAT-GCATTAAATCGCTCC

Platanthera_minor_SG1238 AGGGC-TCTCGGCAATGGATATCTT-GGCTCTCGCATCGATGAAGAGCGCAGCGAAATGCGATACGTGGTGC-GAATTGCAGAATCCCGTGAACC-ATCGAGTTTTTGAACGCAAGTTGCGCCTGAGGCCAGC-TGGCCAAGGGCACGTCCGCCTG---GGCGTCAT-GCATTAAATCGCTCC

Porpax_pusilla_KY239239 ACGAC-TCTCGGCAATGGATATCTC-GGCTCTTGCATCGATGAAGAGCGCAGCGAAATGCGATACGTGGTGC-GAATTGCAGAATCCCGTGAACC-ATCGAGTCTTTGAACGCAAGTTGCGCCCGAGGCCAAC-CGGCTGAGGGCACGTCTGCCTG---GGCGTCAA-ACGTTGCGTCGCTCT

Porpax_pusilla_PK12071 ACGAC-TCTCGGCAATGGATATCTC-GGCTCTTGCATCGATGAAGAGCGCAGCGAAATGCGATACGTGGTGC-GAATTGCAGAATCCCGTGAACC-ATCGAGTCTTTGAACGCAAGTTGCGCCCGAGGCCAAC-CGGCTGAGGGCACGTCTGCCTG---GGCGTCAA-ACGTCGCGTCGCTCT

Porpax_pusilla_PK12072 ACGAC-TCTCGGCAATGGATATCTC-GGCTCTTGCATCGATGAAGAGCGCAGCGAAATGCGATACGTGGTGC-GAATTGCAGAATCCCGTGAACC-ATCGAGTCTTTGAACGCAAGTTGCGCCCGAGGCCAAC-CGGCTGAGGGCACGTCTGCCTG---GGCGTCAA-ACGTCGCGTCGCTCT

Porpax_pusilla_PK12125 ACGAC-TCTCGGCAATGGATATCTC-GGCTCTTGCATCGATGAAGAGCGCAGCGAAATGCGATACGTGGTGC-GAATTGCAGAATCCCGTGAACC-ATCGAGTCTTTGAACGCAAGTTGCGCCCGAGGCCAAC-CGGCTGAGGGCACGTCTGCCTG---GGCGTCAA-ACGTTGCGTCGCTCT

Porpax_pusilla_SG1334 ACGAC-TCTCGGCAATGGATATCTC-GGCTCTTGCATCGATGAAGAGCGCAGCGAAATGCGATACGTGGTGC-GAATTGCAGAATCCCGTGAACC-ATCGAGTCTTTGAACGCAAGTTGCGCCCGAGGCCAAC-CGGCTGAGGGCACGTCTGCCTG---GGCGTCAA-ACGTTGCGTCGCTCT

Renanthera_coccinea_KJ733441 ACGAC-TCTCGACAATGGATATCTC-GGCTCTCGCATCGATGAAGAGCGCAGCGAAATGCGATACGTGGTGC-GAATTGCAGAATCCCGCGAACC-ATCGAGTCTTTGAACGCAAGTTGCGCCCGAGGCCAAT-CGGTCGAGGGCACGTCCGCCTG---GGCGTCAA-GCGTTGCGTCGCTCC

Rhomboda_abbreviata_KT344110 ATGAC-TCTCGACAATGGATATCTT-GGCTCTTGCATCGATGAAGAGCGCAGCGAAATGCGATACGTGGTGT-GAATTGCAGAATCCCGTGAACC-ATCAAATATTTGAACGCAAGTTGCGCCCGAGGCCAAT-TGGCTAAGGGCACGTCCGCCTG---GGCGTCAA-GC-TTACATCGCTTA

Rhomboda_abbreviata_KY966662 ATGAC-TCTCGACAATGGATATCTT-GGCTCTTGCATCGATGAAGAGCGCAGCGAAATGCGATACGTGGTGT-GAATTGCAGAATCCCGTGAACC-ATCAAATATTTGAACGCAAGTTGCGCCCGAGGCCAAT-TGGCTAAGGGCACGTCCGCCTG---GGCGTCAA-GCATTACATCGCTTC

Rhomboda_abbreviata_PK12166 ATGAC-TCTCGACAATGGATATCTT-GGCTCTTGCATCGATGAAGAGCGCAGCGAAATGCGATACGTGGTGT-GAATTGCAGAATCCCGTGAACC-ATCAAATATTTGAACGCAAGTTGCGCCCGAGGCCAAT-TGGCTAAGGGCACGTCCGCCTG---GGCGTCAA-GCATTACATCGCTTC

Rhomboda_abbreviata_PK12175 ATGAC-TCTCGACAATGGATATCTT-GGCTCTTGCATCGATGAAGAGCGCAGCGAAATGCGATACGTGGTGT-GAATTGCAGAATCCCGTGAACC-ATCAAATATTTGAACGCAAGTTGCGCCCGAGGCCAAT-TGGCTAAGGGCACGTCCGCCTG---GGCGTCAA-GCATTACATCGCTTC

Rhomboda_abbreviata_SG1203 ATGAC-TCTCGACAATGGATATCTT-GGCTCTTGCATCGATGAAGAGCGCAGCGAAATGCGATACGTGGTGT-GAATTGCAGAATCCCGTGAACC-ATCAAATATTTGAACGCAAGTTGCGCCCGAGGCCAAT-TGGCTAAGGGCACGTCCGCCTG---GGCGTCAA-GCATTACATCGCTTC

Robiquetia_succisa_KJ733444 ACGAC-TCTCGACAATGGATATCTC-GGCTCTCGCATCGATGAAGAGCGCAGCGAAATGCGATACGTGGTGC-GAATTGCAGAATCCCGCGAACC-ATCGAGTCTTTGAACGCAAGTTGCGCCCGAGGCCAAT-CGGTCGAGGGCACGTCCGCCTG---GGCGTCAA-GCGTTGCGCCGCTCC

Robiquetia_succisa_KY966667 ACGAC-TCTCGACAATGGATATCTC-GGCTCTCGCATCGATGAAGAGCGCAGCGAAATGCGATACGTGGTGC-GAATTGCAGAATCCCGCGAACC-ATCGAGTCTTTGAACGCAAGTTGCGCCCGAGGCCAAT-CGGTCGAGGGCACGTCCGCCTG---GGCGTCAA-GCGTTGCGCCGCTCC

Robiquetia_succisa_PK12155 ACGAC-TCTCGACAATGGATATCTC-GGCTCTCGCATCGATGAAGAGCGCAGCGAAATGCGATACGTGGTGC-GAATTGCAGAATCCCGCGAACC-ATCGAGTCTTTGAACGCAAGTTGCGCCCGAGGCCAAT-CGGTCGAGGGCACGTCCGCCTG---GGCGTCAA-GCGTTGCGCCGCTCC

Robiquetia_succisa_PK12156 ACGAC-TCTCGACAATGGATATCTC-GGCTCTCGCATCGATGAAGAGCGCAGCGAAATGCGATACGTGGTGC-GAATTGCAGAATCCCGCGAACC-ATCGAGTCTTTGAACGCAAGTTGCGCCCGAGGCCAAT-CGGTCGAGGGCACGTCCGCCTG---GGCGTCAA-GCGTTGCGCCGCTCC

Robiquetia_succisa_PK12157 ATGAC-TCTCGGCAACGGATATCTC-GGCTCTCGCATCGATGAAGAGCGCAGCGAAATGCGATACGTGGTGC-GAATTGCAGAATCCCGTGAACC-ATCAAGTCTTTGAACGCAAGTTGCGCCCGAGGCCAAT-CGGCCAAGGGCACGTCTGCCTG---GGCGTCAA-GCGTTGCGTCGCTTC

Robiquetia_succisa_SG1293 ACGAC-TCTCGACAATGGATATCTC-GGCTCTCGCATCGATGAAGAGCGCAGCGAAATGCGATACGTGGTGC-GAATTGCAGAATCCCGCGAACC-ATCGAGTCTTTGAACGCAAGTTGCGCCCGAGGCCAAT-CGGTCGAGGGCACGTCCGCCTG---GGCGTCAA-GCGTTGCGCCGCTCC

Robiquetia_succisa_SG1294 ACGAC-TCTCGACAATGGATATCTC-GGCTCTCGCATCGATGAAGAGCGCAGCGAAATGCGATACGTGGTGC-GAATTGCAGAATCCCGCGAACC-ATCGAGTCTTTGAACGCAAGTTGCGCCCGAGGCCAAT-CGGTCGAGGGCACGTCCGCCTG---GGCGTCAA-GCGTTGCGCCGCTCC

Robiquetia_succisa_SG1345 ACGAC-TCTCGACAATGGATATCTC-GGCTCTCGCATCGATGAAGAGCGCAGCGAAATGCGATACGTGGTGC-GAATTGCAGAATCCCGCGAACC-ATCGAGTCTTTGAACGCAAGTTGCGCCCGAGGCCAAT-CGGTCGAGGGCACGTCCGCCTG---GGCGTCAA-GCGTTGCGCCGCTCC

Spathoglottis_pubescens_KM025162 ACGAC-TCTCGGCAATGGATATCTC-GGCTCTCGCATCGATGAAGAGCGCAGCGAAATGCGATACGTGGTGC-GAATTGCAGAATCCCGCGAACC-ATCGAGTCTTTGAACGCAAGTTGCGCCCGAGGCCAAC-CGGCCAAGGGCACGTCTGCCTG---GGCGTCAA-GCGTTGCGTCGCTCC

Spathoglottis_pubescens_KP751405 ACGAC-TCTCGGCAATGGATATCTC-GGCTCTCGCATCGATGAAGAGCGCAGCGAAATGCGATACGTGGTGC-GAATTGCAGAATCCCGCGAACC-ATCGAGTCTTTGAACGCAAGTTGCGCCCGAGGCCAAC-CGGCCAAGGGCACGTCTGCCTG---GGCGTCAA-GCGTTGCGTCGCTCC

Spathoglottis_pubescens_KP751406 ACGAC-TCTCGGCAATGGATATCTC-GGCTCTCGCATCGATGAAGAGCGCAGCGAAATGCGATACGTGGTGC-GAATTGCAGAATCCCGCGAACC-ATCGAGTCTTTGAACGCAAGTTGCGCCCGAGGCCAAC-CGGCCAAGGGCACGTCTGCCTG---GGCGTCAA-GCGTTGCGTCGCTCC

Spathoglottis_pubescens_MG869012 ACGAC-TCTCGGCAATGGATATCTC-GGCTCTCGCATCGATGAAGAGCGCAGCGAAATGCGATACGTGGTGC-GAATTGCAGAATCCCGCGAACC-ATCGAGTCTTTGAACGCAAGTTGCGCCCGAGGCCAAC-CGGCCAAGGGCACGTCTGCCTG---GGCGTCAA-GCGTTGCGTCGCTCC

Spathoglottis_pubescens_PK12135 ACGAC-TCTCGGCAATGGATATCTC-GGCTCTCGCATCGATGAAGAGCGCAGCGAAATGCGATACGTGGTGC-GAATTGCAGAATCCCGCGAACC-ATCGAGTCTTTGAACGCAAGTTGCGCCCGAGGCCAAC-CGGCCAAGGGCACGTCTGCCTG---GGCGTCAA-GCGTTGCGTCGCTCC

Spathoglottis_pubescens_PK12140 ACGAC-TCTCGGCAATGGATATCTC-GGCTCTCGCATCGATGAAGAGCGCAGCGAAATGCGATACGTGGTGC-GAATTGCAGAATCCCGCGAACC-ATCGAGTCTTTGAACGCAAGTTGCGCCCGAGGCCAAC-CGGCCAAGGGCACGTCTGCCTG---GGCGTCAA-GCGTTGCGTCGCTCC

Spathoglottis_pubescens_SG1202 ACGAC-TCTCGGCAATGGATATCTC-GGCTCTCGCATCGATGAAGAGCGCAGCGAAATGCGATACGTGGTGC-GAATTGCAGAATCCCGCGAACC-ATCGAGTCTTTGAACGCAAGTTGCGCCCGAGGCCAAC-CGGCCAAGGGCACGTCTGCCTG---GGCGTCAA-GCGTTGCGTCGCTCC

Spathoglottis_pubescens_SG1205 ACGAC-TCTCGGCAATGGATATCTC-GGCTCTCGCATCGATGAAGAGCGCAGCGAAATGCGATACGTGGTGC-GAATTGCAGAATCCCGCGAACC-ATCGAGTCTTTGAACGCAAGTTGCGCCCGAGGCCAAC-CGGCCAAGGGCACGTCTGCCTG---GGCGTCAA-GCGTTGCGTCGCTCC

Spiranthes_hongkongensis_MF286484 ATGAC-TCCCGGCAATGGATATCTT-GGCTCTTGCATCGATGAAGAGCGCAGCGAAATGCGATACGTGGTGT-GAATTGCAGAATCCCGCGAACC-ATCGAGTTTTTGAACGCAAGTTGCGCCCGAGGCCAAT-TGGCTGAGGGCACGTCCGCCTG---GGCGTCAA-GCATTACATCGCTTC

Spiranthes_hongkongensis_MF286511 ATGAC-TCCCGGCAATGGATATCTT-GGCTCTTGCATCGATGAAGAGCGCAGCGAAATGCGATACGTGGTGT-GAATTGCAGAATCCCGTGAACC-ATCGAGTTTTTGAACGCAAGTTGCGCCCGAGGCCAAT-TGGCTGAGGGCACGTCCGCCTG---GGCGTCAA-GCATTACATCGCTTC

Spiranthes_hongkongensis_MH002629 ATGAC-TCCCGGCAATGGATATCTT-GGCTCTTGCATCGATGAAGAGCGCAGCGAAATGCGATACGTGGTGT-GAATTGCAGAATCCCGTGAACC-ATCGAGTTTTTGAACGCAAGTTGCGCCCGAGGCCAAT-TGGCTGAGGGCACGTCCGCCTG---GGCGTCAA-GCATTACATCGCTTC

Spiranthes_hongkongensis_MH002630 ATGAC-TCCCGGCAATGGATATCTT-GGCTCTTGCATCGATGAAGAGCGCAGCGAAATGCGATACGTGGTGT-GAATTGCAGAATCCCGTGAACC-ATCGAGTTTTTGAACGCAAGTTGCGCCCGAGGCCAAT-TGGCTGAGGGCACGTCCGCCTG---GGCGTCAA-GCATTACATCGCTTC

Spiranthes_hongkongensis_MH002631 ATGAC-TCCCGGCAATGGATATCTT-GGCTCTTGCATCGATGAAGAGCGCAGCGAAATGCGATACGTGGTGT-GGATTGCAGAATCCCGTGAACC-ATCGAGTTTTTGAACGCAAGTTGCGCCCGAGGCCAAT-TGGCTGAGGGCACGTCCGCCTG---GGCGTCAA-GCATTACATCGCTTC

Spiranthes_hongkongensis_MH002632 ATGAC-TCCCGGCAATGGATATCTT-GGCTCTTGCATCGATGAAGAGCGCAGCGAAATGCGATACGTGGTGT-GAATTGCAGAATCCCGTGAACC-ATCGAGTTTTTGAACGCAAGTTGCGCCCGAGGCCAAT-TGGCTGAGGGCACGTCCGCCTG---GGCGTCAA-GCATTACATCGCTTC

Spiranthes_hongkongensis_MH002633 ATGAC-TCCCGGCAATGGATATCTT-GGCTCTTGCATCGATGAAGAGCGCAGCGAAATGCGATACGTGGTGT-GAATTGCAGAATCCCGTGAACC-ATCGAGTTTTTGAACGCAAGTTGCGCCCGAGGCCAAT-TGGCTGAGGGCACGTCCGCCTG---GGCGTCAA-GCATTACATCGCTTC

Spiranthes_hongkongensis_MH002634 ATGAC-TCCCGGCAATGGATATCTT-GGCTCTTGCATCGATGAAGAGCGCAGCGAAATGCGATACGTGGTGT-GAATTGCAGAATCCCGTGAACC-ATCGAGTTTTTGAACGCAAGTTGCGCCCGAGGCCAAT-TGGCTGAGGGCACGTCCGCCTG---GGCGTCAA-GCATTACATCGCTTC

Spiranthes_hongkongensis_MH002635 ATGAC-TCCCGGCAATGGATATCTT-GGCTCTTGCATCGATGAAGAGCGCAGCGAAATGCGATACGTGGTGT-GAATTGCAGAATCCCGTGAACC-ATCGAGTTTTTGAACGCAAGTTGCGCCCGAGGCCAAT-TGGCTGAGGGCACGTCCGCCTG---GGCGTCAA-GCATTACATCGCTTC

Spiranthes_hongkongensis_MH002636 ATGAC-TCCCGGCAATGGATATCTT-GGCTCTTGCATCGATGAAGAGCGCAGCGAAATGCGATACGTGGTGT-GAATTGCAGAATCCCGTGAACC-ATCGAGTTTTTGAACGCAAGTTGCGCCCGAGGCCAAT-TGGCTGAGGGCACGTCCGCCTG---GGCGTCAA-GCATTACATCGCTTC

Spiranthes_hongkongensis_MH002637 ATGAC-TCCCGGCAATGGATATCTT-GGCTCTTGCATCGATGAAGAGCGCAGCGAAATGCGATACGTGGTGT-GAATTGCAGAATCCCGTGAACC-ATCGAGTTTTTGAACGCAAGTTGCGCCCGAGGCCAAT-TGGCTGAGGGCACGTCCGCCTG---GGCGTCAA-GCATTACATCGCTTC

Spiranthes_hongkongensis_MH002638 ATGAC-TCCCGGCAATGGATATCTT-GGCTCTTGCATCGATGAAGAGCGCAGCGAAATGCGATACGTGGTGT-GAATTGCAGAATCCCGTGAACC-ATCGAGTTTTTGAACGCAAGTTGCGCCCGAGGCCAAT-TGGCTGAGGGCACGTCCGCCTG---GGCGTCAA-GCATTACATCGCTTC

Spiranthes_hongkongensis_MH002639 ATGAC-TCCCGGCAATGGATATCTT-GGCTCTTGCATCGATGAAGAGCGCAGCGAAATGCGATACGTGGTGT-GAATTGCAGAATCCCGTGAACC-ATCGAGTTTTTGAACGCAAGTTGCGCCCGAGGCCAAT-TGGCTGAGGGCACGTCCGCCTG---GGCGTCAA-GCATTACATCGCTTC

Spiranthes_hongkongensis_MH002640 ATGAC-TCCCGGCAATGGATATCTT-GGCTCTTGCATCGATGAAGAGCGCAGCGAAATGCGATACGTGGTGT-GAATTGCAGAATCCCGTGAACC-ATCGAGTTTTTGAACGCAAGTTGCGCCCGAGGCCAAT-TGGCTGAGGGCACGTCCGCCTG---GGCGTCAA-GCATTACATCGCTTC

Spiranthes_hongkongensis_MH002641 ATGAC-TCCCGGCAATGGATATCTT-GGCTCTTGCATCGATGAAGAGCGCAGCGAAATGCGATACGTGGTGT-GAATTGCAGAATCCCGTGAACC-ATCGAGTTTTTGAACGCAAGTTGCGCCCGAGGCCAAT-TGGCTGAGGGCACGTCCGCCTG---GGCGTCAA-GCATTACATCGCTTC

Spiranthes_hongkongensis_MH002642 ATGAC-TCCCGGCAATGGATATCTT-GGCTCTTGCATCGATGAAGAGCGCAGCGAAATGCGATACGTGGTGT-GAATTGCAGAATCCCGTGAACC-ATCGAGTTTTTGAACGCAAGTTGCGCCCGAGGCCAAT-TGGCTGAGGGCACGTCCGCCTG---GGCGTCAA-GCATTACATCGCTTC

Spiranthes_hongkongensis_MH002643 ATGAC-TCCCGGCAATGGATATCTT-GGCTCTTGCATCGATGAAGAGCGCAGCGAAATGCGATACGTGGTGT-GAATTGCAGAATCCCGTGAACC-ATCGAGTTTTTGAACGCAAGTTGCGCCCGAGGCCAAT-TGGCTGAGGGCACGTCCGCCTG---GGCGTCAA-GCATTACATCGCTTC

Spiranthes_hongkongensis_MH002644 ATGAC-TCCCGGCAATGGATATCTT-GGCTCTTGCATCGATGAAGAGCGCAGCGAAATGCGATACGTGGTGT-GAATTGCAGAATCCCGTGAACC-ATCGAGTTTTTGAACGCAAGTTGCGCCCGAGGCCAAT-TGGCTGAGGGCACGTCCGCCTG---GGCGTCAA-GCATTGCATCGCTTC

Spiranthes_hongkongensis_MH002645 ATGAC-TCCCGGCAATGGATATCTT-GGCTCTTGCATCGATGAAGAGCGCAGCGAAATGCGATACGTGGTGT-GAATTGCAGAATCCCGTGAACC-ATCGAGTTTTTGAACGCAAGTTGCGCCCGAGGCCAAT-TGGCTGAGGGCACGTCCGCCTG---GGCGTCAA-GCATTACATCGCTTC

Spiranthes_hongkongensis_MH002646 ATGAC-TCCCGGCAATGGATATCTT-GGCTCTTGCATCGATGAAGAGCGCAGCGAAATGCGATACGTGGTGT-GAATTGCAGAATCCCGTGAACC-ATCGAGTTTTTGAACGCAAGTTGCGCCCGAGGCCAAT-TGGCTGAGGGCACGTCCGCCTG---GGCGTCAA-GCATTACATCGCTTC

Spiranthes_hongkongensis_MH002647 ATGAC-TCCCGGCAATGGATATCTT-GGCTCTTGCATCGATGAAGAGCGCAGCGAAATGCGATACGTGGTGT-GAATTGCAGAATCCCGTGAACC-ATCGAGTTTTTGAACGCAAGTTGCGCCCGAGGCCAAT-TGGCTGAGGGCACGTCCGCCTG---GGCGTCAA-GCATTACATCGCTTC

Spiranthes_hongkongensis_MH002648 ATGAC-TCCCGGCAATGGATATCTT-GGCTCTTGCATCGATGAAGAGCGCAGCGAAATGCGATACGTGGTGT-GAATTGCAGAATCCCGTGAACC-ATCGAGTTTTTGAACGCAAGTTGCGCCCGAGGCCAAT-TGGCTGAGGGCACGTCCGCCTG---GGCGTCAA-GCATTACATCGCTTC

Spiranthes_hongkongensis_MH002649 ATGAC-TCCCGGCAATGGATATCTT-GGCTCTTGCATCGATGAAGAGCGCAGCGAAATGCGATACGTGGTGT-GGATTGCAGAATCCCGTGAACC-ATCGAGTTTTTGAACGCAAGTTGCGCCCGAGGCCAAT-TGGCTGAGGGCACGTCCGCCTG---GGCGTCAA-GCATTACATCGCTTC

Spiranthes_hongkongensis_MH002650 ATGAC-TCCCGGCAATGGATATCTT-GGCTCTTGCATCGATGAAGAGCGCAGCGAAATGCGATACGTGGTGT-GAATTGCAGAATCCCGTGAACC-ATCGAGTTTTTGAACGCAAGTTGCGCCCGAGGCCAAT-TGGCTGAGGGCACGTCCGCCTG---GGCGTCAA-GCATTACATCGCTTC

Spiranthes_hongkongensis_MH002651 ATGAC-TCCCGGCAATGGATATCTT-GGCTCTTGCATCGATGAAGAGCGCAGCGAAATGCGATACGTGGTGT-GAATTGCAGAATCCCGTGAACC-ATCGAGTTTTTGAACGCAAGTTGCGCCCGAGGCCAAT-TGGCTGAGGGCACGTCCGCCTG---GGCGTCAA-GCATTACATCGCTTC

Spiranthes_hongkongensis_MH002652 ATGAC-TCCCGGCAATGGATATCTT-GGCTCTTGCATCGATGAAGAGCGCAGCGAAATGCGATACGTGGTGT-GAATTGCAGAATCCCGTGAACC-ATCGAGTTTTTGAACGCAAGTTGCGCCCGAGGCCAAT-TGGCTGAGGGCACGTCCGCCTG---GGCGTCAA-GCATTACATCGCTTC

Spiranthes_hongkongensis_MH002653 ATGAC-TCCCGGCAATGGATATCTT-GGCTCTTGCATCGATGAAGAGCGCAGCGAAATGCGATACGTGGTGT-GAATTGCAGAATCCCGTGAACC-ATCGAGTTTTTGAACGCAAGTTGCGCCCGAGGCCAAT-TGGCTGAGGGCACGTCCGCCTG---GGCGTCAA-GCATTACATCGCTTC

Spiranthes_hongkongensis_MH002654 ATGAC-TCCCGGCAATGGATATCTT-GGCTCTTGCATCGATGAAGAGCGCAGCGAAATGCGATACGTGGTGT-GAATTGCAGAATCCCGTGAACC-ATCGAGTTTTTGAACGCAAGTTGCGCCCGAGGCCAAT-TGGCTGAGGGCACGTCCGCCTG---GGCGTCAA-GCATTACATCGCTTC

Spiranthes_hongkongensis_MH002655 ATGAC-TCCCGGCAATGGATATCTT-GGCTCTTGCATCGATGAAGAGCGCAGCGAAATGCGATACGTGGTGT-GAATTGCAGAATCCCGTGAACC-ATCGAGTTTTTGAACGCAAGTTGCGCCCGAGGCCAAT-TGGCTGAGGGCACGTCCGCCTG---GGCGTCAA-GCATTACATCGCTTC

Spiranthes_hongkongensis_MH002656 ATGAC-TCCCGGCAATGGATATCTT-GGCTCTTGCATCGATGAAGAGCGCAGCGAAATGCGATACGTGGTGT-GAATTGCAGAATCCCGTGAACC-ATCGAGTTTTTGAACGCAAGTTGCGCCCGAGGCCAAT-TGGCTGAGGGCACGTCCGCCTG---GGCGTCAA-GCATTACATCGCTTC

Spiranthes_hongkongensis_MH002657 ATGAC-TCCCGGCAATGGATATCTT-GGCTCTTGCATCGATGAAGAGCGCAGCGAAATGCGATACGTGGTGT-GAATTGCAGAATCCCGTGAACC-ATCGAGTTTTTGAACGCAAGTTGCGCCCGAGGCCAAT-TGGCTGAGGGCACGTCCGCCTG---GGCGTCAA-GCATTACATCGCTTC

Spiranthes_hongkongensis_MH002658 ATGAC-TCCCGGCAATGGATATCTT-GGCTCTTGCATCGATGAAGAGCGCAGCGAAATGCGATACGTGGTGT-GAATTGCAGAATCCCGTGAACC-ATCGAGTTTTTGAACGCAAGTTGCGCCCGAGGCCAAT-TGGCTGAGGGCACGTCCGCCTG---GGCGTCAA-GCATTACATCGCTTC

Spiranthes_hongkongensis_MH002659 ATGAC-TCCCGGCAATGGATATCTT-GGCTCTTGCATCGATGAAGAGCGCAGCGAGATGCGATACGTGGAGC-GAATTGCAGAATCCCGTGAACC-ATCGAGTTTTTGAACGCAAGTTGCGCCCGAGGCCAAT-TGGCTGAGGGCACGTCCGCCTG---GGCGTCAA-GCATTACATCGCTTC

Spiranthes_hongkongensis_MH002660 ATGAC-TCCCGGCAATGGATATCTT-GGCTCTTGCATCGATGAAGAGCGCAGCGAAATGCGATACGTGGTGT-GAATTGCAGAATCCCGTGAACC-ATCGAGTTTTTGAACGCAAGTTGCGCCCGGGGCCAAT-TGGCTGAGGGCACGTCCGCCTG---GGCGTCAA-GCATTACATCGCTTC

Spiranthes_hongkongensis_MH002661 ATGAC-TCCCGGCAATGGATATCTT-GGCTCTTGCATCGATGAAGAGCGCAGCGAAATGCGATACGTGGTGT-GAATTGCAGAATCCCGTGAACC-ATCGAGTTTTTGAACGCAAGTTGCGCCCGAGGCCAAT-TGGCTGAGGGCACGTCCGCCTG---GGCGTCAA-GCATTACATCGCTTC

Spiranthes_hongkongensis_MH002662 ATGAC-TCCCGGCAATGGATATCTT-GGCTCTTGCATCGATGAAGAGCGCAGCGAAATGCGATACGTGGTGT-GAATTGCAGAATCCCGTGAACC-ATCGAGTTTTTGAACGCAAGTTGCGCCCGAGGCCAAT-TGGCTGAGGGCACGTCCGCCTG---GGCGTCAA-GCATTACATCGCTTC

Spiranthes_hongkongensis_MH002663 ATGAC-TCCCGGCAATGGATATCTT-GGCTCTTGCATCGATGAAGAGCGCAGCGAAATGCGATACGTGGTGT-GAATTGCAGAATCCCGTGAACC-ATCGAGTTTTTGAACGCAAGTTGCGCCCGAGGCCAAT-TGGCTGAGGGCACGTCCGCCTG---GGCGTCAA-GCATTACATCGCTTC

Spiranthes_hongkongensis_MH002664 ATGAC-TCCCGGCAATGGATATCTT-GGCTCTTGCATCGATGAAGAGCGCAGCGAAATGCGATACGTGGTGT-GAATTGCAGAATCCCGTGAACC-ATCGAGTTTTTGAACGCAAGTTGCGCCCGAGGCCAAT-TGGCTGAGGGCACGTCCGCCTG---GGCGTCAA-GCATTACATCGCTTC

Spiranthes_hongkongensis_MH002665 ATGAC-TCCCGGCAATGGATATCTT-GGCTCTTGCATCGATGAAGAGCGCAGCGAAATGCGATACGTGGTGT-GAATTGCAGAATCCCGTGAACC-ATCGAGTTTTTGAACGCAAGTTGCGCCCGAGGCCAAT-TGGCTGAGGGCACGTCCGCCTG---GGCGTCAA-GCATTACATCGCTTC

Spiranthes_hongkongensis_MH002666 ATGAC-TCCCGGCAATGGATATCTT-GGCTCTTGCATCGATGAAGAGTGCAGCGAAATGCGATACGTGGTGT-GAATTGCAGAATCCCGTGAACC-ATCGAGTTTTTGAATGCAAGTTGCGCCCGAGGCCAAT-TGGCTGAGGGCACGTCCGCCTG---GGCGTCAA-GCATTACATCGCTTC

Spiranthes_hongkongensis_MH002667 ATGAC-TCCCGGCAATGGATATCTT-GGCTCTTGCATCGATGAAGAGCGCAGCGAAATGCGATACGTGGTGT-GAATTGCAGAATCCCGTGAACC-ATCGAGTTTTTGAACGCAAGTTGCGCCCGAGGCCAAT-TGGCTGAGGGCACGTCCGCCTG---GGCGTCAA-GCATTACATCGCTTC

Spiranthes_hongkongensis_MH002668 ATGAC-TCCCGGCAATGGATATCTT-GGCTCTTGCATCGATGAAGAGCGCAGCGAAATGCGATACGTGGTGT-GAATTGCAGAATCCCGTGAACC-ATCGAGTTTTTGAACGCAAGTTGCGCCCGAGGCCAAT-TGGCTGAGGGCACGTCCGCCTG---GGCGTCAA-GCATTACATCGCTTC

Spiranthes_hongkongensis_MH002669 ATGAC-TCCCGGCAATGGATATCTT-GGCTCTTGCATCGATGAAGAGCGCAGCGAAATGCGATACGTGGTGT-GAATTGCAGAATCCCGTGAACC-ATCGAGTTTTTGAACGCAAGTCGCGCCCGAGGCCAAT-TGGCTGAGGGCACGTCCGCCTG---GGCGTCAA-GCATTACATCGCTTC

Spiranthes_hongkongensis_MH002670 ATGAC-TCCCGGCAATGGATATCTT-GGCTCTTGCATCGATGAAGAGCGCAGCGAAATGCGATACGTGGTGT-GAATTGCAGAATCCCGTGAACC-ATCGAGTTTTTGAACGCAAGTTGCGCCCGAGGCCAAT-TGGCTGAGGGCACGTCCGCCTG---GGCGTCAA-GCATTACATCGCTTC

Spiranthes_hongkongensis_MH002671 ATGAC-TCCCGGCAATGGATATCTT-GGCTCTTGCATCGATGAAGAGCGCAGCGAAATGCGATACGTGGTGT-GAATTGCAGAATCCCGTGAACC-ATCGAGTTTTTGAACGCAAGTTGCGCCCGAGGCCAAT-TGGCTGAGGGCACGTCCGCCTG---GGCGTCAA-GCATTACATCGCTTC

Spiranthes_hongkongensis_MH002672 ATGAC-TCCCGGCAATGGATATCTT-GGCTCTTGCATCGATGAAGAGCGCAGCGAAATGCGATACGTGGTGT-GAATTGCAGAATCCCGTGAACC-ATCGAGTTTTTGAACGCAAGTTGCGCCCGAGGCCAAT-TGGCTGAGGGCACGTCCGCCTG---GGCGTCAA-GCATTACATCGCTTC

Spiranthes_hongkongensis_MH002673 ATGAC-TCCCGGCAATGGATATCTT-GGCTCTTGCATCGATGAAGAGCGCAGCGAAATGCGATACGTGGTGT-GAATTGCAGAATCCCGCGAACC-ATCGAGTTTTTGAACGCAAGTTGCGCCCGAGGCCAAT-TGGCTGAGGGCACGTCCGCCTG---GGCGTCAA-GCATTACATCGCTTC

Spiranthes_hongkongensis_MH002674 ATGAC-TCCCGGCAATGGATATCTT-GGCTCTTGCATCGATGAAGAGCGCAGCGAAATGCGATACGTGGTGT-GAATTGCAGAATCCCGTGAACC-ATCGAGTTTTTGAACGCAAGTTGCGCCCGAGGCCAAT-TGGCTGAGGGCACGTCCGCCTG---GGCGTCAA-GCATTACATCGCTTC

Spiranthes_hongkongensis_MH002675 ATGAC-TCCCGGCAATGGATATCTT-GGCTCTTGCATCGATGAAGAGCGCAGCGAAATGCGATACGTGGTGT-GAATTGCAGAATCCCGTGAACC-ATCGAGTTTTTGAACGCAAGTTGCGCCCGAGGCCAAT-TGGCTGAGGGCACGTCCGCCTG---GGCGTCAA-GCATTACATCGCTTC

Spiranthes_hongkongensis_MH002676 ATGAC-TCCCGGCAATGGATATCTT-GGCTCTTGCATCGATGAAGAGCGCAGCGAAATGCGATACGTGGTGT-GAATTGCAGAATCCCGTGAACC-ATCGAGTTTTTGAACGCAAGTTGCGCCCGAGGCCAAT-TGGCTGAGGGCACGTCCGCCTG---GGCGTCAA-GCATTACATCGCTTC

Spiranthes_hongkongensis_MH002677 ATGAC-TCCCGGCAATGGATATCTT-GGCTCTTGCATCGATGAAGAGCGCAGCGAAATGCGATACGTGGTGT-GAATTGCAGAATCCCGTGAACC-ATCGAGTTTTTGAACGCAAGTTGCGCCCGAGGCCAAT-TGGCTGAGGGCACGTCCGCCTG---GGCGTCAA-GCATTACATCGCTTC

Spiranthes_hongkongensis_MH002678 ATGAC-TCCCGGCAATGGATATCTT-GGCTCTTGCATCGATGAAGAGCGCAGCGAAATGCGATACGTGGTGT-GAATTGCAGAATCCCGTGAACC-ATCGAGTTTTTGAACGCAAGTTGCGCCCGAGGCCAAT-TGGCTGAGGGCACGTCCGCCTG---GGCGTCAA-GCATTACATCGCTTC

Spiranthes_hongkongensis_MH002679 ATGAC-TCCCGGCAATGGATATCTT-GGCTCTTGCATCGATGAAGAGCGCAGCGAAATGCGATACGTGGTGT-GAATTGCAGAATCCCGTGAACC-ATCGAGTTTTTGAACGCAAGTTGCGCCCGAGGCCAAT-TGGCTGAGGGCACGTCCGCCTG---GGCGTCAA-GCATTACATCGCTTC

Spiranthes_hongkongensis_MH002680 ATGAC-TCCCGGCAATGGATATATT-GGCTCTTGCATCGATGAAGAGCGCAGCGAAATGCGATACGTGGTGT-GAATTGCAGAATCCCGTGAACC-ATCGAGTTTTTGAACGCAAGTTGCGCCCGAGGCCAAT-TGGCTGAGGGCACGTCCGCCTG---GGCGTCAA-GCATTACATCGCTTC

Spiranthes_hongkongensis_MH002681 ATGAC-TCCCGGCAATGGATATATT-GGCTCTTGCATCGATGAAGAGCGCAGCGAAATGCGATACGTGGTGT-GAATTGCAGAATCCCGTGAACC-ATCGAGTTTTTGAACGCAAGTTGCGCCCGAGGCCAAT-TGGCTGAGGGCACGTCCGCCTG---GGCGTCAA-GCATTACATCGCTTC

Spiranthes_hongkongensis_MH002682 ATGAC-TCCCGGCAATGGATATCTT-GGCTCTTGCATCGATGAAGAGCGCAGCGAAATGCGATACGTGGTGT-GAATTGCAGAATCCCGTGAACC-ATCGAGTTTTTGAACGCAAGTTGCGCCCGAGGCCAAT-TGGCTGAGGGCACGTCCGCCTG---GGCGTCAA-GCATTACATCGCTTC

Spiranthes_hongkongensis_MH002683 ATGAC-TCCCGGCAATGGATATCTT-GGCTCTTGCATCGATGAAGAGCGCAGCGAAATGCGATACGTGGTGT-GAATTGCAGAATCCCGTGAACC-ATCGAGTTTTTGAACGCAAGTTGCGCCCGAGGCCAAT-TGGCTGAGGGCACGTCCGCCTG---GGCGTCAA-GCATTACATCGCTTC

Spiranthes_hongkongensis_MH002684 ATGAC-TCCCGGCAATGGATATCTT-GGCTCTTGCATCGATGAAGAGCGCAGCGAAATGCGATACGTGGTGT-GAATTGCAGAATCCCGTGAACC-ATCGAGTTTTTGAACGCAAGTTGCGCCCGAGGCCAAT-TGGCTGAGGGCACGTCCGCCTG---GGCGTCAA-GCATTACATCGCTTC

Spiranthes_hongkongensis_MH002685 ATGAC-TCCCGGCAATGGATATCTT-GGCTCTTGCATCGATGAAGAGCGCAGCGAAATGCGATACGTGGTGT-GAATTGCAGAATCCCGTGAACC-ATCGAGTTTTTGAACGCAAGTTGCGCCCGAGGCCAAT-TGGCTGAGGGCACGTCCGCCTG---GGCGTCAA-GCATTACATCGCTTC

Spiranthes_hongkongensis_MH002686 ATGAC-TCCCGGCAATGGATATCTT-GGCTCTTGCATCGATGAAGAGCGCAGCGAAATGCGATACGTGGTGT-GAATTGCAGAATCCCGTGAACC-ATCGAGTTTTTGAACGCAAGTTGCGCCCGAGGCCAAT-TGGCTGAGGGCACGTCCGCCTG---GGCGTCAA-GCATTACATCGCTTC

Spiranthes_hongkongensis_MH002687 ATGAC-TCCCGGCAATGGATATCTT-GGCTCTTGCATCGATGAAGAGCGCAGCGAAATGCGATACGTGGTGT-GAATTGCAGAATCCCGTGAACC-ATCGAGTTTTTGAATGCAAGTTGCGCCCGAGGCCAAT-TGGCTGAGGGCACGTCCGCCTG---GGCGTCAA-GCATTACATCGCTTC

Spiranthes_hongkongensis_MH002688 ATGAC-TCCCGGCAATGGATATCTT-GGCTCTTGCATCGATGAAGAGCGCAGCGAAATGCGATACGTGGTGT-GAATTGCAGAATCCCGTGAACC-ATCGAGTTTTTGAACGCAAGTTGCGCCCGAGGCCAAT-TGGCTGAGGGCACGTCCGCCTG---GGCGTCAA-GCATTACATCGCTTC

Spiranthes_hongkongensis_MH002689 ATGAC-TCCCGGCAATGGATATCTT-GGCTCTTGCATCGATGAAGAGCGCAGCGAAATGCGATACGTGGTGT-GAATTGCAGAATCCCGTGAACC-ATCGAGTTTTTGAACGCAAGTTGCGCCCGAGGCCAAT-TGGCTGAGGGCACGTCCGCCTG---GGCGTCAA-GCATTACATCGCTTC

Spiranthes_hongkongensis_MH002690 ATGAC-TCCCGGCAATGGATATCTT-GGCTCTTGCATCGATGAAGAGCGCAGCGAAATGCGATACGTGGTGT-GAATTGCAGAATCCCGTGAACC-ATCGAGTTTTTGAACGCAAGTTGCGCCCGAGGCCAAT-TGGCTGAGGGCACGTCCGCCTG---GGCGTCAA-GCATTACATCGCTTC

Spiranthes_hongkongensis_MH002691 ATGAC-TCCCGGCAATGGATATCTT-GGCTCTTGCATCGATGAAGAGCGCAGCGAAATGCGATACGTGGTGT-GAATTGCAGAATCCCGTGAACC-ATCGAGTTTTTGAACGCAAGTTGCGCCCGAGGCCAAT-TGGCTGAGGGCACGTCCGCCTG---GGCGTCAA-GCATTACATCGCTTC

Spiranthes_hongkongensis_MH002692 ATGAC-TCCCGGCAATGGATATCTT-GGCTCTTGCATCGATGAAGAGCGCAGCGAAATGCGATACGTGGTGT-GAATTGCAGAATCCCGTGAACC-ATCGAGTTTTTGAACGCAAGTTGCGCCCGAGGCCAAT-TGGCTGAGGGCACGTCCGCCTG---GGCGTCAA-GCATTACATCGCTTC

Spiranthes_hongkongensis_MH002693 ATGAC-TCCCGGCAATGGATATCTT-GGCTCTTGCATCGATGAAGAGCGCAGCGAAATGCGATACGTGGTGT-GAATTGCAGAATCCCGTGAACC-ATCGAGTTTTTGAACGCAAGTTGCGCCCGAGGCCAAT-TGGCTGAGGGCACGTCCGCCTG---GGCGTCAA-GCATTACATCGCTTC

Spiranthes_hongkongensis_MH002694 ATGAC-TCCCGGCAATGGATATCTT-GGCTCTTGCATCGATGAAGAGCGCAGCGAAATGCGATACGTGGTGT-GAATTGCAGAATCCCGTGAACC-ATCGAGTTTTTGAACGCAAGTTGCGCCCGAGGCCAAT-TGGCTGAGGGCACGTCCGCCTG---GGCGTCAA-GCATTACATCGCTTC

Spiranthes_hongkongensis_MH002695 ATGAC-TCCCGGCAATGGATATCTT-GGCTCTTGCATCGATGAAGAGCGCAGCGAAATGCGATACGTGGTGT-GAATTGCAGAATCCCGTGAACC-ATCGAGTTTTTGAACGCAAGTCGCGCCCGAGGCCAAT-TGGCTGAGGGCACGTCCGCCTG---GGCGTCAA-GCATTACATCGCTTC

Spiranthes_hongkongensis_MH002696 ATGAC-TCCCGGCAATGGATATCTT-GGCTCTTGCATCGATGAAGAGCGCAGCGAAATGCGATACGTGGTGT-GAATTGCAGAATCCCGTGAACC-ATCGAGTTTTTGAACGCAAGTTGCGCCCGAGGCCAAT-TGGCTGAGGGCACGTCCGCCTG---GGCGTCAA-GCATTACATCGCTTC

Spiranthes_hongkongensis_MH002697 ATGAC-TCCCGGCAATGGATATCTT-GGCTCTTGCATCGATGAAGAGCGCAGCGAAATGCGATACGTGGTGT-GAATTGCAGAATCCCGTGAACC-ATCGAGTTTTTGAACGCAAGTTGCGCCCGAGGCCAAT-TGGCTGAGGGCACGTCCGCCTG---GGCGTCAA-GCATTACATCGCTTC

Spiranthes_hongkongensis_MH002698 ATGAC-TCCCGGCAATGGATATCTT-GGCTCTTGCATCGATGAAGAGCGCAGCGAAATGCGATACGTGGTGT-GAATTGCAGAATCCCGTGAACC-ATCGAGTTTTTGAACGCAAGTTGCGCCCGAGGCCAAT-TGGCTGAGGGCACGTCCGCCTG---GGCGTCAA-GCATTACATCGCTTC

Spiranthes_hongkongensis_MH038786 ATGAC-TCCCGGCAATGGATATCTT-GGCTCTTGCATCGATGAAGAGCGCAGCGAAATGCGATACGTGGTGT-GAATTGCAGAATCCCGTGAACC-ATCGAGTTTTTGAACGCAAGTTGCGCCCGAGGCCAAT-TGGCTGAGGGCACGTCCGCCTG---GGCGTCAA-GCATTACATCGCTTC

Spiranthes_hongkongensis_MH038787 ATGAC-TCCCGGCAATGGATATCTT-GGCTCTTGCATCGATGAAGAGCGCAGCGAAATGCGATACGTGGTGT-GAATTGCAGAATCCCGTGAACC-ATCGAGTTTTTGAACGCAAGTTGCGCCCGAGGCCAAT-TGGCTGAGGGCACGTCCGCCTG---GGCGTCAA-GCATTACATCGCTTC

Spiranthes_hongkongensis_PK12028 ATGAC-TCCCGGCAATGGATATCTT-GGCTCTTGCATCGATGAAGAGCGCAGCGAAATGCGATACGTGGTGT-GAATTGCAGAATCCCGTGAACC-ATCGAGTTTTTGAACGCAAGTTGCGCCCGAGGCCAAT-TGGCTGAGGGCACGTCCGCCTG---GGCGTCAA-GCATTACATCGCTTC

Spiranthes_hongkongensis_PK12102 ATGAC-TCCCGGCAATGGATATCTT-GGCTCTTGCATCGATGAAGAGCGCAGCGAAATGCGATACGTGGTGT-GAATTGCAGAATCCCGTGAACC-ATCGAGTTTTTGAACGCAAGTTGCGCCCGAGGCCAAT-TGGCTGAGGGCACGTCCGCCTG---GGCGTCAA-GCATTACATCGCTTC

Spiranthes_hongkongensis_PK12179 ATGAC-TCCCGGCAATGGATATCTT-GGCTCTTGCATCGATGAAGAGCGCAGCGAAATGCGATACGTGGTGT-GAATTGCAGAATCCCGTGAACC-ATCGAGTTTTTGAACGCAAGTTGCGCCCGAGGCCAAT-TGGCTGAGGGCACGTCCGCCTG---GGCGTCAA-GCATTACATCGCTTC

Spiranthes_sinensis_HE575518 ATGAC-TCCCGGCAATGGATATCTT-GGCTCTTGCATCGATGAAGAGCGCAGCGAAATGCGATACGTGGTGT-GAATTGCAGAATCCCGTGAACC-ATCGAGTTTTTGAACGCAAGTTGCGCCCGAGGCCAAT-TGGCTGAGGGCACGTCCGCCTG---GGCGTCAA-GCATTATATCGCTTC

Spiranthes_sinensis_KM262399 ATGAC-TCCCGGCAATGGATATCTT-GGCTCTTGCATCGATGAAGAGCGCAGCGAAATGCGATACGTGGTGT-GAATTGCAGAATCCCGTGAACC-ATCGAGTTTTTGAACGCAAGTTGCGCCCGAGGCCAAT-TGGCTGAGGGCACGTCCGCCTG---GGCGTCAA-GCATTACATCGCTTC

Spiranthes_sinensis_KM262400 ATGAC-TCCCGGCAATGGATATCTT-GGCTCTTGCATCGATGAAGAGCGCAGCGAAATGCGATACGTGGTGT-GAATTGCAGAATCCCGTGAACC-ATCGAGTTTTTGAACGCAAGTTGCGCCCGAGGCCAAT-TGGCTGAGGGCACGTCCGCCTG---GGCGTCAA-GCATTACATCGCTTC

Spiranthes_sinensis_KT338780 ATGAC-TCCCGGCAATGGATATCTT-GGCTCTTGCATCGATGAAGAGCGCAGCGAAATGCGATACGTGGTGT-GAATTGCAGAATCCCGTGAACC-ATCGAGTTTTTGAACGCAAGTTGCGCCCGAGGCCAAT-TGGCTGAGGGCACGTCCGCCTG---GGCGTCAA-GCATTACATCGCTTC

Spiranthes_sinensis_KT338781 ATGAC-TCCCGGCAATGGATATCTT-GGCTCTTGCATCGATGAAGAGCGCAGCGAAATGCGATACGTGGTGT-GAATTGCAGAATCCCGTGAACC-ATCGAGTTTTTGAACGCAAGTTGCGCCCGAGGCCAAT-TGGCTGAGGGCACGTCCGCCTG---GGCGTCAA-GCATTACATCGCTTC

Spiranthes_sinensis_MF286485 ATGAC-TCCCGGCAATGGATATCTT-GGCTCTTGCATCGATGAAGAGCGCAGCGAAATGCGATACGTGGTGT-GAATTGCAGAATCCCGTGAACC-ATCGAGTTTTTGAACGCAAGTTGCGCCCGAGGCCAAT-TGGCTGAGGGCACGTCCGCCTG---GGCGTCAA-GCATTACATCGCTTC

Spiranthes_sinensis_MF286486 ATGAC-TCCCGGCAATGGATATCTT-GGCTCTTGCATCGATGAAGAGCGCAGCGAAATGCGATACGTGGTGT-GAATTGCAGAATCCCGTGAACC-ATCGAGTTTTTGAACGCAAGTTGCGCCCGAGGCCAAT-TGGCTGAGGGCACGTCCGCCTG---GGCGTCAA-GCATTACATCGCTTC

Spiranthes_sinensis_MF286487 ATGAC-TCCCGGCAATGGATATCTT-GGCTCTTGCATCGATGAAGAGCGCAGCGAAATGCGATACGTGGTGT-GAATTGCAGAATCCCGTGAACC-ATCGAGTTTTTGAACGCAAGTTGCGCCCGAGGCCAAT-TGGCTGAGGGCACGTCCGCCTG---GGCGTCAA-GCATTACATCGCTTC

Spiranthes_sinensis_MF286488 ATGAC-TCCCGGCAATGGATATCTT-GGCTCTTGCATCGATGAAGAGCGCAGCGAAATGCGATACGTGGTGT-GAATTGCAGAATCCCGTGAACC-ATCGAGTTTTTGAACGCAAGTTGCGCCCGAGGCCAAT-TGGCTGAGGGCACGTCCGCCTG---GGCGTCAA-GCATTACATCGCTTC

Spiranthes_sinensis_MF286489 ATGAC-TCCCGGCAATGGATATCTT-GGCTCTTGCATCGATGAAGAGCGCAGCGAAATGCGATACGTGGTGT-GAATTGCAGAATCCCGTGAACC-ATCGAGTTTTTGAACGCAAGTTGCGCCCGAGGCCAAT-TGGCTGAGGGCACGTCCGCCTG---GGCGTCAA-GCATTACATCGCTTC

Spiranthes_sinensis_MF286493 ATGAC-TCCCGGCAATGGATATCTT-GGCTCTTGCATCGATGAAGAGCGCAGCGAAATGCGATACGTGGTGT-GAATTGCAGAATCCCGTGAACC-ATCGAGTTTTTGAACGCAAGTTGCGCCCGAGGCCAAT-TGGCTGAGGGCACGTCCGCCTG---GGCGTCAA-GCATTACATCGCTTC

Spiranthes_sinensis_MF286494 ATGAC-TCCCGGCAATGGATATCTT-GGCTCTTGCATCGATGAAGAGCGCAGCGAAATGCGATACGTGGTGT-GAATTGCAGAATCCCGTGAACC-ATCGAGTTTTTGAACGCAAGTTGCGCCCGAGGCCAAT-TGGCTGAGGGCACGTCCGCCTG---GGCGTCAA-GCATTACATCGCTTC

Spiranthes_sinensis_MF286495 ATGAC-TCCCGGCAATGGATATCTT-GGCTCTTGCATCGATGAAGAGCGCAGCGAAATGCGATACGTGGTGT-GAATTGCAGAATCCCGTGAACC-ATCGAGTTTTTGAACGCAAGTTGCGCCCGAGGCCAAT-TGGCTGAGGGCACGTCCGCCTG---GGCGTCAA-GCATTACATCGCCTC

Spiranthes_sinensis_MF286496 ATGAC-TCCCGGCAATGGATATCTT-GGCTCTTGCATCGATGAAGAGCGCAGCGAAATGCGATACGTGGTGT-GAATTGCAGAATCCCGTGAACC-ATCGAGTTTTTGAACGCAAGTTGCGCCCGAGGCCAAT-TGGCTGAGGGCACGTCCGCCTG---GGCGTCAA-GCATTACATCGCTTC

Spiranthes_sinensis_MF286497 ATGAC-TCCCGGCAATGGATATCTT-GGCTCTTGCATCGATGAAGAGCGCAGCGAAATGCGATACGTGGTGT-GAATTGCAGAATCCCGTGAACC-ATCGAGTTTTTGAACGCAAGTTGCGCCCGAGGCCAAT-TGGCTGAGGGCACGTCCGCCTG---GGCGTCAA-GCATTACATCGCTTC

Spiranthes_sinensis_MF286498 ATGAC-TCCCGGCAATGGATATCTT-GGCTCTTGCATCGATGAAGAGCGCAGCGAAATGCGATACGTGGTGT-GAATTGCAGAATCCCGTGAACC-ATCGAGTTTTTGAACGCAAGTTGCGCCCGAGGCCAAT-TGGCTGAGGGCACGTCCGCCTG---GGCGTCAA-GCATTACATCGCTTC

Spiranthes_sinensis_MF286499 ATGAC-TCCCGGCAATGGATATCTT-GGCTCTTGCATCGATGAAGAGCGCAGCGAAATGCGATACGTGGTGT-GAATTGCAGAATCCCGTGAACC-ATCGAGTTTTTGAACGCAAGTTGCGCCCGAGGCCAAT-TGGCTGAGGGCACGTCCGCCTG---GGCGTCAA-GCATTACATCGCTTT

Spiranthes_sinensis_MF286504 ATGAC-TCCCGGCAATGGATATCTT-GGCTCTTGCATCGATGAAGAGCGCAGCGAAATGCGATACGTGGTGT-GAATTGCAGAATCCCGTGAACC-ATCGAGTTTTTGAACGCAAGTTGCGCCCGAGGCCAAT-TGGCTGAGGGCACGTCCGCCTG---GGCGTCAA-GCATTACATCGCTTC

Spiranthes_sinensis_MF286505 ATGAC-TCCCGGCAATGGATATCTT-GGCTCTTGCATCGATGAAGAGCGCAGCGAAATGCGATACGTGGTGT-GAATTGCAGAATCCCGTGAACC-ATCGAGTTTTTGAACGCAAGTTGCGCCCGAGGCCAAT-TGGCTGAGGGCACGTCCGCCTG---GGCGTCAA-GCATTACATCGCTTC

Spiranthes_sinensis_MF286506 ATGAC-TCCCGGCAATGGATATCTT-GGCTCTTGCATCGATGAAGAGCGCAGCGAAATGCGATACGTGGTGT-GAATTGCAGAATCCCGTGAACC-ATCGAGTTTTTGAACGCAAGTTGCGCCCGAGGCCAAT-TGGCTGAGGGCACGTCCGCCTG---GGCGTCAA-GCATTACATCGCTTC

Spiranthes_sinensis_MF286507 ATGAC-TCCCGGCAATGGATATCTT-GGCTCTTGCATCGATGAAGAGCGCAGCGAAATGCGATACGTGGTGT-GAATTGCAGAATCCCGTGAACC-ATCGAGTTTTTGAACGCAAGTTGCGCCCGAGGCCAAT-TGGCTGAGGGCACGCCCGCCTG---GGCGTCAA-GCATTACATCGCTTC

Spiranthes_sinensis_MF286509 ATGAC-TCCCGGCAATGGATATCTT-GGCTCTTGCATCGATGAAGAGCGCAGCGAAATGCGATACGTGGTGT-GAATTGCAGAATCCCGTGAACC-ATCGAGTTTTTGAACGCAAGTTGCGCCCGAGGCCAAT-TGGCTGAGGGCACGTCCGCCTG---GGCGTCAA-GCATTACATCGCTTC

Spiranthes_sinensis_MF286510 ATGAC-TCCCGGCAATGGATATCTT-GGCTCTTGCATCGATGAAGAGCGCAGCGAAATGCGATACGTGGTGT-GAATTGCAGAATCCCGTGAACC-ATCGAGTTTTTGAACGCAAGTTGCGCCCGAGGCCAAT-TGGCTGAGGGCACGTCCGCCTG---GGCGTCAA-GCATTACATCGCTTC

Spiranthes_sinensis_MH005035 ATGAC-TCCCGGCAATGGATATCTT-GGCTCTTGCATCGATGAAGAGCGCAGCGAAATGCGATACGTGGTGT-GAATTGCAGAATCCCGTGAACC-ATCGAGTTTTTGAACGCAAGTTGCGCCCGAGGCCAAT-TGGCTGAGGGCACGTCCGCCTG---GGCGTCAA-GCATTACATCGCTTC

Spiranthes_sinensis_MH005036 ATGAC-TCCCGGCAATGGATATCTT-GGCTCTTGCATCGATGAAGAGCGCAGCGAAATGCGATACGTGGTGT-GAATTGCAGAATCCCGTGAACC-ATCGAGTTTTTGAACGCAAGTTGCGCCCGAGGCCAAT-TGGCTGAGGGCACGTCCGCCTG---GGCGTCAA-GCATTACATCGCTTC

Spiranthes_sinensis_MH005037 ATGAC-TCCCGGCAATGGATATCTT-GGCTCTTGCATCGATGAAGAGCGCAGCGAAATGCGATACGTGGTGT-GAATTGCAGAATCCCGTGAACC-ATCGAGTTTTTGAACGCAAGTTGCGCCCGAGGCCAAT-TGGCTGAGGGCACGTCCGCCTG---GGCGTCAA-GCATTACATCGCTTC

Spiranthes_sinensis_MH005038 ATGAC-TCCCGGCAATGGATATCTT-GGCTCTTGCATCGATGAAGAGCGCAGCGAAATGCGATACGTGGTGT-GAATTGCAGAATCCCGTGAACC-ATCGAGTTTTTGAACGCAAGTTGCGCCCGAGGCCAAT-TGGCTGAGGGCACGTCCGCCTG---GGCGTCAA-GCATTACATCGCTTC

Spiranthes_sinensis_MH005039 ATGAC-TCCCGGCAATGGATATCTT-GGCTCTTGCATCGATGAAGAGCGCAGCGAAATGCGATACGTGGTGT-GAATTGCAGAATCCCGTGAACC-ATCGAGTTTTTGAACGCAAGTTGCGCCCGAGGCCAAT-TGGCTGAGGGCACGTCCGCCTG---GGCGTCAA-GCATTACATCGCTTC

Spiranthes_sinensis_MH005040 ATGAC-TCCCGGCAATGGATATCTT-GGCTCTTGCATCGATGAAGAGCGCAGCGAAATGCGATACGTGGTGT-GAATTGCAGAATCCCGTGAACC-ATCGAGTTTTTGAACGCAAGTTGCGCCCGAGGCCAAT-TGGCTGAGGGCACGTCCGCCTG---GGCGTCAA-GCATTACATCGCTTC

Spiranthes_sinensis_MH005041 ATGAC-TCCCGGCAATGGATATCTT-GGCTCTTGCATCGATGAAGAGCGCAGCGAAATGCGATACGTGGTGT-GAATTGCAGAATCCCGTGAACC-ATCGAGTTTTTGAACGCAAGTTGCGCCCGAGGCCAAT-TGGCTGAGGGCACGTCCGCCTG---GGCGTCAA-GCATTACATCGCCTC

Spiranthes_sinensis_MH005042 ATGAC-TCCCGGCAATGGATATCTT-GGCTCTTGCATCGATGAAGAGCGCAGCGAAATGCGATACGTGGTGT-GAATTGCAGAATCCCGTGAACC-ATCGAGTTTTTGAACGCAAGTTGCGCCCGAGGCCAAT-TGGCTGAGGGCACGTCCGCCTG---GGCGTCAA-GCATTACATCGCTTC

Spiranthes_sinensis_MH005043 ATGAC-TCCCGGCAATGGATATCTT-GGCTCTTGCATCGATGAAGAGCGCAGCGAAATGCGATACGTGGTGT-GAATTGCAGAATCCCGTGAACC-ATCGAGTTTTTGAACGCAAGTTGCGCCCGAGGCCAAT-TGGCTGAGGGCACGTCCGCCTG---GGCGTCAA-GCATTACATCGCTTC

Spiranthes_sinensis_MH005044 ATGAC-TCCCGGCAATGGATATCTT-GGCTCTTGCATCGATGAAGAGCGCAGCGAAATGCGATACGTGGTGT-GAATTGCAGAATCCCGTGAACC-ATCGAGTTTTTGAACGCAAGTTGCGCCCGAGGCCAAT-TGGCTGAGGGCACGTCCGCCTG---GGCGTCAA-GCATTACATCGCTTC

Spiranthes_sinensis_MH005045 TTGAC-TCCCGGCAATGGATATCTT-GGCTCTTGCATCGATGAAGAGCGCAGCGAAATGCGATACGAGGTGT-GAATTGCAGAATCCCGTGAACC-ATCGAGTTTTTGAACGCAAGTTGCGCCCGAGGCCAAT-TGGCTGAGGGCACGTCCGCCTG---GGCGTCAA-GCATTACATCGCTTC

Spiranthes_sinensis_MH005046 ATGAC-TCCCGGCAATGGATATCTT-GGCTCTTGCATCGATGAAGAGCGCAGCGAAATGCGATACGTGGTGT-GAATTGCAGAATCCCGTGAACC-ATCGAGTTTTTGAACGCAAGTTGCGCCCGAGGCCAAT-TGGCTGAGGGCACGTCCGCTTG---GGCGTCAA-GCATTACATCGCTTC

Spiranthes_sinensis_MH005047 ATGAC-TCCCGGCAATGGATATCTT-GGCTCTTGCATCGATGAAGAGCGCAGCGAAATGCGATACGTGGTGT-GAATTGCAGAATCCCGTGAACC-ATCGAGTTTTTGAACGCAAGTTGCGCCCGAGGCCAAT-TGGCTGAGGGCACGTCCGCCTG---GGCGTCAA-GCATTACATCGCTTC

Spiranthes_sinensis_MH005048 ATGAC-TCCCGGCAATGGATATCTT-GGCTCTTGCATCGATGAAGAGCGCAGCGAAATGCGATACGTGGTGT-GAATTGCAGAATCCCGTGAACC-ATCGAGTTTTTGAACGCAAGTTGCGCCCGAGGCCAAT-TGGCTGAGGGCACGTCCGCCTG---GGCGTCAA-GCATTACATCGCTTC

Spiranthes_sinensis_MH005049 ATGAC-TCCCGGCAATGGATATCTT-GGCTCTTGCATCGATGAAGAGCGCAGCGAAATGCGATACGTGGTGT-GAATTGCAGAATCCCGTGAACC-ATCGAGTTTTTGAACGCAAGTTGCGCCCGAGGCCAAT-TGGCTGAGGGCACGTCCGCCTG---GGCGTCAA-GCATTACATCGCTTC

Spiranthes_sinensis_MH005050 ATGAC-TCCCGGCAATGGATATCTT-GGCTCTTGCATCGATGAAGAGCGCAGCGAAATGCGATACGTGGTGT-GAATTGCAGAATCCCGTGAACC-ATCGAGTTTTTGAACGCAAGTTGCGCCCGAGGCCAAT-TGGCTGAGGGCACGTCCGCCTG---GGCGTCAA-GCATTACATCGCTTC

Spiranthes_sinensis_MH005051 ATGAC-TCCCGGCAATGGATATCTT-GGCTCTTGCATCGATGAAGAGCGCAGCGAAATGCGATACGTGGTGT-GAATTGCAGAATCCCGTGAACC-ATCGAGTTTTTGAACGCAAGTTGCGCCCGAGGCCAAT-TGGCTGAGGGCACGTCCGCCTG---GGCGTCAA-GCATTACATCGCTTC

Spiranthes_sinensis_MH005052 ATGAC-TCCCGGCAATGGATATCTT-GGCTCTTGCATCGATGAAGAGCGCAGCGAAATGCGATACGTGGTGT-GAATTGCAGAATCCTGTGAACC-ATCAAGTTTTTGAATGCAAGTTGCGCCCAAGGCCAAT-TGGCTGAGGGCACGTCCTCTTG---GGCATCAA-GCATTACATCGCTTC

Spiranthes_sinensis_MH005053 ATGAC-TCCCGGCAATGGATATCTT-GGCTCTTGCATCGATGAAGAGCGCAGCGAAATGCGATACGTGGTGT-GAATTGCAGAATCCCGTGAACC-ATCGAGTTTTTGAACGCAAGTTGCGCCCGAGGCCAAT-TGGCTGAGGGCACGTCCGCCTG---GGCGTCAA-GCATTACATCGCTTC

Spiranthes_sinensis_MH005054 ATGAC-TCCCGGCAATGGATATCTT-GGCTCTTGCATCGATGTAGAGCGCAGCGAAATGCGATACGTGGTGT-GAATTGCAGAATCCCGTGAACC-ATCGAGTTTTTGAACGCAAGTTGCGCCCGAGGCCAAT-TGGCTGAGGGCACGTCCGCCTG---GGCGTCAA-GCATTACATCGCTTC

Spiranthes_sinensis_MH005055 ATGAC-TCCCGGCAATGGATATCTT-GGCTCTTGCATCGATGAAGAGCGCAGCGAAATGCGATACGTGGTGT-GAATTGCAGAATCCCGTGAACC-ATCGAGTTTTTGAACGCAAGTTGCGCCCGAGGCCAAT-TGGCTGAGGGCACGTCCGCCTG---GGCGTCAA-GCATTACATCGCTTC

Spiranthes_sinensis_MH005056 ATGAC-TCCCGGCAATGGATATCTT-GGCTCTTGCATCGATGAAGAGCGCAGCGAAATGCGATACGTGGTGT-GAATTGCAGAATCCCGTGAACC-ATCGAGTTTTTGAACGCAAGTTGCGCCCGAGGCCAAT-TGGCTGAGGGCACGTCCGCCTG---GGCGTCAA-GCATTACATCGCTTC

Spiranthes_sinensis_MH005057 ATGAC-CCCCGGCAATGGATATCTT-GGCTCTTGCATCGATGAAGAGCGCAGCGAAATGCGATACGTGGTGT-GAATTGCAGAATCCCGTGAACC-ATCGAGTTTTTGAACGCAAGTTGCGCCCGAGGCCAAT-TGGCTGAGGGCACGTCCGCCTG---GGCGTCAA-GCATTACATCGCTTC

Spiranthes_sinensis_MH005058 ATGAC-TCCCGGCAATGGATATCTT-GGCTCTTGCATCGATGAAGAGCGCAGCGAAATGCGATACGTGGTGT-GAATTGCAGAATCCCGTGAACC-ATCGAGTTTTTGAACGCAAGTTGCGCCCGAGGCCAAT-TGGCTGAGGGCACGTCCGCCTG---GGCGTCAA-GCATTACATCGCTTC

Spiranthes_sinensis_MH005059 ATGAC-TCCCGGCAATGGATATCTT-GGCTCTTGCATCGATGAAGAGCGCAGCGAAATGCGATACGTGGTGT-GAATTGCAGAATCCCGTGAACC-ATCGAGTTTTTGAACGCAAGTTGCGCCCGAGGCCAAT-TGGCTGAGGGCACGTCCGCCTG---GGCGTCAA-GCATTACATCGCTTC

Spiranthes_sinensis_MH038785 ATGAC-TCCCGGCAATGGATATCTT-GGCTCTTGCATCGATGAAGAGCGCAGCGAAATGCGATACGTGGTGT-GAATTGCAGAATCCCGTGAACC-ATCGAGTTTTTGAACGCAAGTTGCGCCCGAGGCCAAT-TGGCTGAGGGCACGTCCGCCTG---GGCGTCAA-GCATTACATCGCTTC

Spiranthes_sinensis_MH802049 ATGAC-TCCCGGCAATGGATATCTT-GGCTCTTGCATCGATGAAGAGCGCAGCGAAATGCGATACGTGGTGT-GAATTGCAGAATCCCGTGAACC-ATCGAGTTTTTGAACGCAAGTTGCGCCCGAGGCCAAT-TGGCTGAGGGCACGTCCGCCTG---GGCGTCAA-GCATTACATCGCTTC

Spiranthes_sinensis_MH802050 ATGAC-TCCCGGCAATGGATATCTT-GGCTCTTGCATCGATGAAGAGCGCAGCGAAATGCGATACGTGGTGT-GAATTGCAGAATCCCGTGAACC-ATCGAGTTTTTGAACGCAAGTTGCGCCCGAGGCCAAT-TGGCTGAGGGCACGTCCGCCTG---GGCGTCAA-GCATTACATCGCTTC

Spiranthes_sinensis_PK12106 ATGAC-TCCCGGCAATGGATATCTT-GGCTCTTGCATCGATGAAGAGCGCAGCGAAATGCGATACGTGGTGT-GAATTGCAGAATCCCGTGAACC-ATCGAGTTTTTGAACGCAAGTTGCGCCCGAGGCCAAT-TGGCTGAGGGCACGTCCGCCTG---GGCGTCAA-GCATTACATCGCTTC

Spiranthes_sinensis_PK12109 ATGAC-TCCCGGCAATGGATATCTT-GGCTCTTGCATCGATGAAGAGCGCAGCGAAATGCGATACGTGGTGT-GAATTGCAGAATCCCGTGAACC-ATCGAGTTTTTGAACGCAAGTTGCGCCCGAGGCCAAT-TGGCTGAGGGCACGTCCGCCTG---GGCGTCAA-GCATTACATCGCTTC

Spiranthes_sinensis_SG1153 ATGAC-TCCCGGCAATGGATATCTT-GGCTCTTGCATCGATGAAGAGCGCAGCGAAATGCGATACGTGGTGT-GAATTGCAGAATCCCGTGAACC-ATCGAGTTTTTGAACGCAAGTTGCGCCCGAGGCCAAT-TGGCTGAGGGCACGTCCGCCTG---GGCGTCAA-GCATTACATCGCTTC

Spiranthes_sinensis_SG1155 ATGAC-TCCCGGCAATGGATATCTT-GGCTCTTGCATCGATGAAGAGCGCAGCGAAATGCGATACGTGGTGT-GAATTGCAGAATCCCGTGAACC-ATCGAGTTTTTGAACGCAAGTTGCGCCCGAGGCCAAT-TGGCTGAGGGCACGTCCGCCTG---GGCGTCAA-GCATTACATCGCTTC

Tainia_cordifolia_KF560538 ACGAC-TCTCGGCAATGGATATCTC-GGCTCTCGCATCGATGAAGAGCGCAGCGAAATGTGATACGTGGTGC-GAATTGCAGAATCCCGCGAACC-ATCGAGTCTTTGAACGCAAGTTGCGCCCGAGGCCAAT-CGGCCAAGGGCACGTCTGCCTG---GGCGTCAA-GCTTTGTGTCGCTCC

Tainia_cordifolia_KM025163 ACGAC-TCTCGGCAATGGATATCTC-GGCTCTCGCATCGATGAAGAGCGCAGCGAAATGTGATACGTGGTGC-GAATTGCAGAATCCCGCGAACC-ATCGAGTCTTTGAACGCAAGTTGCGCCCGAGGCCAAT-CGGCCAAGGGCACGTCTGCCTG---GGCGTCAA-GCTTTGCGTCGCTCC

Tainia_cordifolia_KM025164 ACGAC-TCTCGGCAATGGATATCTC-GGCTCTCGCATCGATGAAGAGCGCAGCGAAATGTGATACGTGGTGC-GAATTGCAGAATCCCGCGAACC-ATCGAGTCTTTGAACGCAAGTTGCGCCCGAGGCCAAT-CGGCCAAGGGCACGTCTGCCTG---GGCGTCAA-GCTTTGCGTCGCTCC

Tainia_dunnii_KF560536 ACGAC-TCTCGGCAATGGATATCTC-GGCTCTCGCATCGATGAAGAGCGCAGCGAAATGCGATACGTGGTGC-GAATTGCAGAATCCCGCGAACC-ATCGAGTCTTTGAACGCAAGTTGCGCCCGAGGCCAAC-CGGCCAAGGGCACGTCTGCCTG---GGCGTCAA-GCGTTGCGTCGCTCC

Tainia_dunnii_KM025165 ACGAC-TCTCGGCAATGGATATCTC-GGCTCTCGCATCGATGAAGAGCGCAGCGAAATGCGATACGTGGTGC-GAATTGCAGAATCCCGCGAACC-ATCGAGTCTTTGAACGCAAGTTGCGCCCGAGGCCAAC-CGGCCAAGGGCACGTCTGCCTG---GGCGTCAA-GCGTTGCGTCGCTCC

Tainia_dunnii_SG1273 ACGAC-TCTCGGCAATGGATATCTC-GGCTCTCGCATCGATGAAGAGCGCAGCGAAATGCGATACGTGGTGC-GAATTGCAGAATCCCGCGAACC-ATCGAGTCTTTGAACGCAAGTTGCGCCCGAGGCCAAC-CGGCCAAGGGCACGTCTGCCTG---GGCGTCAA-GCGTTGCGTCGCTCC

Thelasis_pygmaea_KY239231 ATGAC-TCTCGACAATGGATATCTC-GGCTCTCGCATCGATGAAGAGCGCAGCGAAATGCGATATGTGGTGC-GAATTGCAGAATCCCGCGAACC-ATCGAGTCTTTGAACGCAAGTTGCGCCCGAGGCCAAT-CGGCCAAGGGCACGTCCGCCTG---GGCGTCAA-GCGTTTCGTCGCATC

Thrixspermum_centipeda_AB217591 ATGAC-TCTCGACAATGGATATCTC-GGCTCTCGCATCGATGAAGAGCGCAGCGAAATGCGATACGTGGTGC-GAATTGCAGAATCCCGCGAACC-ATCGAGTCTTTGAACGCAAGTTGCGCCCGAGGCCAAT-CGGTCGAGGGCACGTCCGCCTG---GGCGTCAA-GCGTTGCGCCGCTCC

Thrixspermum_centipeda_KFBG3066D ATGAC-TCTCGACAATGGATATCTC-GGCTCTCGCATCGATGAAGAGCGCAGCGAAATGCGATACGTGGTGC-GAATTGCAGAATCCCGCGAACC-ATCGAGTCTTTGAACGCAAGTTGCGCCCGAGGCCAAT-CGGTCGAGGGCACGTCCGCCTG---GGCGTCAA-GCGTTGCGCCGCTCC

Thrixspermum_centipeda_KJ733456 ATGAC-TCTCGACAATGGATATCTC-GGCTCTCGCATCGATGAAGAGCGCAGCGAAATGCGATACGTGGTGC-GAATTGCAGAATCCCGCGAACC-ATCGAGTCTTTGAACGCAAGTTGCGCCCGAGGCCAAT-CGGTCGAGGGCACGTCCGCCTG---GGCGTCAA-GCGTTGCGCCGCTCC

Thrixspermum_centipeda_KX679332 ATGAC-TCTCGACAATGGATATCTC-GGCTCTCGCATCGATGAAGAGCGCAGCGAAATGCGATACGTGGTGC-GAATTGCAGAATCCCGCGAACC-ATCGAGTCTTTGAACGCAAGTTGCGCCCGAGGCCAAT-CGGTCGAGGGCACGTCCGCCTG---GGCGTCAA-GCGTTGCGCCGCTCC

Thrixspermum_centipeda_KY966674 ATGAC-TCTCGACAATGGATATCTC-GGCTCTCGCATCGATGAAGAGCGCAGCGAAATGCGATACGTGGTGC-GAATTGCAGAATCCCGCGAACC-ATCGAGTCTTTGAACGCAAGTTGCGCCCGAGGCCAAT-CGGTCGAGGGCACGTCCGCCTG---GGCGTCAA-GCGTTGCGCCGCTCC

Thrixspermum_centipeda_KY966675 ATGAC-TCTCGACAATGGATATCTC-GGCTCTCGCATCGATGAAGAGCGCAGCGAAATGCGATACGTGGTGC-GAATTGCAGAATCCCGCGAACC-ATCGAGTCTTTGAACGCAAGTTGCGCCCGAGGCCAAT-CGGTCGAGGGCACGTCCGCCTG---GGCGTCAA-GCGTTGCGCCGCTCC

Thrixspermum_centipeda_PK12129 ATGAC-TCTCGACAATGGATATCTC-GGCTCTCGCATCGATGAAGAGCGCAGCGAAATGCGATACGTGGTGC-GAATTGCAGAATCCCGCGAACC-ATCGAGTCTTTGAACGCAAGTTGCGCCCGAGGCCAAT-CGGTCGAGGGCACGTCCGCCTG---GGCGTCAA-GCGTTGCGCCGCTCC

Thrixspermum_centipeda_PK12130 ATGAC-TCTCGACAATGGATATCTC-GGCTCTCGCATCGATGAAGAGCGCAGCGAAATGCGATACGTGGTGC-GAATTGCAGAATCCCGCGAACC-ATCGAGTCTTTGAACGCAAGTTGCGCCCGAGGCCAAT-CGGTCGAGGGCACGTCCGCCTG---GGCGTCAA-GCGTTGCGCCGCTCC

Thrixspermum_centipeda_PK12131 ATGAC-TCTCGACAATGGATATCTC-GGCTCTCGCATCGATGAAGAGCGCAGCGAAATGCGATACGTGGTGC-GAATTGCAGAATCCCGCGAACC-ATCGAGTCTTTGAACGCAAGTTGCGCCCGAGGCCAAT-CGGTCGAGGGCACGTCCGCCTG---GGCGTCAA-GCGTTGCGCCGCTCC

Thrixspermum_centipeda_PK12132 ATGAC-TCTCGACAATGGATATCTC-GGCTCTCGCATCGATGAAGAGCGCAGCGAAATGCGATACGTGGTGC-GAATTGCAGAATCCCGCGAACC-ATCGAGTCTTTGAACGCAAGTTGCGCCCGAGGCCAAT-CGGTCGAGGGCACGTCCGCCTG---GGCGTCAA-GCGTTGCGCCGCTCC

Thrixspermum_centipeda_PK12133 ATGAC-TCTCGACAATGGATATCTC-GGCTCTCGCATCGATGAAGAGCGCAGCGAAATGCGATACGTGGTGC-GAATTGCAGAATCCCGCGAACC-ATCGAGTCTTTGAACGCAAGTTGCGCCCGAGGCCAAT-CGGTCGAGGGCACGTCCGCCTG---GGCGTCAA-GCGTTGCGCCGCTCC

Tropidia_curculigoides_SG1281 ACGAC-TCTCGGCAATGGATATCTC-GGCTCTCGCATCGATGAAGAGCGCAGCGAAATGCGATACGTGGTGC-GAATTGCAGAATCCCGCGAACC-ATCGAGTCTTTGAACGCAAGTTGCGCCCGAGGCCAAC-CGGCCAAGGGCACGTCTGCCTG---GGCGTCAA-GCGCCGCGTCGCTCC

Tropidia_curculigoides_SG1282 ACGAC-TCTCGGCAATGGATATCTC-GGCTCTCGCATCGATGAAGAGCGCAGCGAAATGCGATACGTGGTGC-GAATTGCAGAATCCCGCGAACC-ATCGAGTCTTTGAACGCAAGTTGCGCCCGAGGCCAAC-CGGCCAAGGGCACGTCTGCCTG---GGCGTCAA-GCGCCGCGTCGCTCC

Tropidia_curculigoides_SG1283 ACGAC-TCTCGGCAATGGATATCTC-GGCTCTCGCATCGATGAAGAGCGCAGCGAAATGCGATACGTGGTGC-GAATTGCAGAATCCCGCGAACC-ATCGAGTCTTTGAACGCAAGTTGCGCCCGAGGCCAAC-CGGCCAAGGGCACGTCTGCCTG---GGCGTCAA-GCGCCGCGTCGCTCC

Tropidia_curculigoides_SG1284 ACGAC-TCTCGGCAATGGATATCTC-GGCTCTCGCATCGATGAAGAGCGCAGCGAAATGCGATACGTGGTGC-GAATTGCAGAATCCCGCGAACC-ATCGAGTCTTTGAACGCAAGTTGCGCCCGAGGCCAAC-CGGCCAAGGGCACGTCTGCCTG---GGCGTCAA-GCGCCGCGTCGCTCC

Tropidia_nipponica_PK12181 ACGAC-TCTCGGCAATGGATATCTC-GGCTCTCGCATCGATGAAGAGCGCAGCGAAATGCGATACGTGGTGC-GAATTGCAGAATCCCGCGAACC-ATCGAGTCTTTGAACGCAAGTTGCGCCCGAGGCCAAC-CGGCCAAGGGCACGCCTGCCTG---GGCGTCAA-GCGCCGCGTCGCTCC

Tropidia_nipponica_SG1355 ACGAC-TCTCGGCAATGGATATCTC-GGCTCTCGCATCGATGAAGAGCGCAGCGAAATGCGATACGTGGTGC-GAATTGCAGAATCCCGCGAACC-ATCGAGTCTTTGAACGCAAGTTGCGCCCGAGGCCAAC-CGGCCAAGGGCACGCCTGCCTG---GGCGTCAA-GCGCCGCGTCGCTCC

Tropidia_nipponica_SG1356 ACGAC-TCTCGGCAATGGATATCTC-GGCTCTCGCATCGATGAAGAGCGCAGCGAAATGCGATACGTGGTGC-GAATTGCAGAATCCCGCGAACC-ATCGAGTCTTTGAACGCAAGTTGCGCCCGAGGCCAAC-CGGCCAAGGGCACGCCTGCCTG---GGCGTCAA-GCGCCGCGTCGCTCC

Tropidia_nipponica_var_hachijoensis_MH596706 ACGAC-TCTCGGCAATGGATATCTC-GGCTCTCGCATCGATGAAGAGCGCAGCGAAATGCGATACGTGGTGC-GAATTGCAGAATCCCGCGAACC-ATCGAGTCTTTGAACGCAAGTTGCGCCCGAGGCCAAC-CGGCCAAGGGCACGTCTGCCTG---GGCGTCAA-GCGCCGCGTCGCTCC

Vanilla_shenzhenica_JF796930 ACGAC-TCTCGACAACGGATATCTT-GGCTCTCGCATCGATGAAGAACGCAGCGAAATGCGATACGTGTTGT-GAATTGTAGAATCCCGTGAACC-ATCCATTTTTTGAACGCAAGTTGCGCCCGAGGA-TGC-AAGCCAAGGGCACTCCTGCATG---GGTGTAAT-GCGTTCTGTCGCTCC

Vanilla_shenzhenica_KFBG290 ACGAC-TCTCGACAACGGATATCTT-GGCTCTCGCATCGATGAAGAACGCAGCGAAATGCGATACGTGTTGT-GAATTGTAGAATCCCGTGAACC-ATCCATTTTTTGAACGCAAGTTGCGCCCGAGGA-TGC-AAGCCAAGGGCACTCCTGCATG---GGTGTAAT-GCGTTCTGTCGCTCC

Vrydagzynea_nuda_SG1222 ATGAC-TCTCGGCAATGGATATCTT-GGCTCTTGCATCGATGAAGAGCGCAGCGAAATGCGATACGTGGTGT-GAATTGCAGAATCCCGTGAACC-ATCAAATCTTTGAACGCAAGTTGCGCCTGAGGCCAAT-TGGCTAAGGGCACGTCCGCCTG---GGCGTCAA-GCATTACATCGCTTC

Vrydagzynea_nuda_SG1246A ATGAC-TCTCGGCAATGGATATCTT-GGCTCTTGCATCGATGAAGAGCGCAGCGAAATGCGATACGTGGTGT-GAATTGCAGAATCCCGTGAACC-ATCAAATCTTTGAACGCAAGTTGCGCCTGAGGCCAAT-TGGCTAAGGGCACGTCCGCCTG---GGCGTCAA-GCATTACATCGCTTC

Vrydagzynea_nuda_SG1246B ATGAC-TCTCGGCAATGGATATCTT-GGCTCTTGCATCGATGAAGAGCGCAGCGAAATGCGATACGTGGTGT-GAATTGCAGAATCCCGTGAACC-ATCAAATCTTTGAACGCAAGTTGCGCCTGAGGCCAAT-TGGCTAAGGGCACGTCCGCCTG---GGCGTCAA-GCATTACATCGCTTC

Vrydagzynea_nuda_SG1247 ATGAC-TCTCGGCAATGGATATCTT-GGCTCTTGCATCGATGAAGAGCGCAGCGAAATGCGATACGTGGTGT-GAATTGCAGAATCCCGTGAACC-ATCAAATCTTTGAACGCAAGTTGCGCCTGAGGCCAAT-TGGCTAAGGGCACGTCCGCCTG---GGCGTCAA-GCATTACATCGCTTC

Zeuxine_boninensis_d16 ATGAC-TCTCGGCAATGGATATCTT-GGCTCTTGCATCGATGAAGAGCGCAGCGAAATGCGATACGTGGTGT-GAATTGCAGAATCCCGTGAACC-ATCAAATATTTGAACGCAAGTTGCGCCCGAGGCCAAT-TGGTTGAGGGCACGTCCGCCTG---GGCGTCAA-GCATTACATCGCTTC

Zeuxine_boninensis_Z3 ATGAC-TCTCGGCAATGGATATCTT-GGCTCTTGCATCGATGAAGAGCGCAGCGAAATGCGATACGTGGTGT-GAATTGCAGAATCCCGTGAACC-ATCAAATATTTGAACGCAAGTTGCGCCCGAGGCCAAT-TGGTTGAGGGCACGTCCGCCTG---GGCGTCAA-GCATTACATCGCTTC

Zeuxine_gracilis_JN166075 ATGAC-TCTCGGCAATGGATATCTT-GGCTCTTGCATCGATGAAGAGCGCAGCGAAATGCGATACGTGGTGT-GAATTGCAGAATCCCGTGAACC-ATCAAATATTTGAACGCAAGTTGCGCCCGAGGCCAAT-TGGTTGAGGGCACGTCCGCCTG---GGCGTCAA-GCATTACATCGCTTC

Zeuxine_gracilis_KC191732 ATGAC-TCTCGGCAATGGATATCTT-GGCTCTTGCATCGATGAAGAGCGCAACGAAATGCAATACGTGGTGT-GAATTGAATAATCCCGTGAACC-ATCAAATATTTGAACGCAAGTTGCGCACGAGGCCAAT-TGGATGAGGGCACGTCCGCCTG---GGCGTCAA-GCATTACATCGCTTC

Zeuxine_gracilis_PK12057 ATGAC-TCTCGACAATGGATATCTT-GGCTCTTGCATCGATGAAGAGCGCAGCGAAATGCGATACGTGGTGT-GAATTGCAGAATCCCGTGAACC-ATCAAATATTTGAACGCAAGTTGCGCCCGAGGCCAAT-TGGTTGAGGGCACGTCCGCCTG---GGCGTCAA-GCATTACATCGCTTC

Zeuxine_gracilis_PK12066 ATGAC-TCTCGGCAATGGATATCTT-GGCTCTTGCATCGATGAAGAGCGCAGCGAAATGCGATACGTGGTGT-GAATTGCAGAATCCCGTGAACC-ATCAAATATTTGAACGCAAGTTGCGCCCGAGGCCAAT-TGGTTGAGGGCACGTCCGCCTG---GGCGTCAA-GCATTACATCGCTTC

Zeuxine_gracilis_SG1204 ATGAC-TCTCGGCAATGGATATCTT-GGCTCTTGCATCGATGAAGAGCGCAGCGAAATGCGATACGTGGTGT-GAATTGCAGAATCCCGTGAACC-ATCAAATATTTGAACGCAAGTTGCGCCCGAGGCCAAT-TGGTTGAGGGCACGTCCGCCTG---GGCGTCAA-GCATTACATCGCTTC

Zeuxine_strateumatica_KT344117 ATGAC-TCTCGGCAATGGATATCTT-GGCTCTTGCATCGATGAAGAGCGCAGCGAAATGCGATACGTGGTGT-GAATTGCAGAATCCCGTGAACC-ATCAAATTTTTGAACGCAAGTTGCGCCCAAGGCCAAT-TGGTTGAGGGCACGTCCGCCTG---GGAGTCAA-GCATTACATCGCTTC

Zeuxine_strateumatica_KY966688 ATGAC-TCTCGGCAATGGATATCTT-GGCTCTTGCATCGATGAAGAGCGCAGCGAAATGCGATACGTGGTGT-GAATTGCAGAATCCCGTGAACC-ATCAAATTTTTGAACGCAAGTTGCGCCCAAGGCCAAT-TGGTTGAGGGCACGTCCGCCTG---GGAGTCAA-GCATTACATCGCTTC

Zeuxine_strateumatica_SG1211 ATGAC-TCTCGGCAATGGATATCTT-GGCTCTTGCATCGATGAAGAGCGCAGCGAAATGCGATACGTGGTGT-GAATTGCAGAATCCCGTGAACC-ATCAAATTTTTGAACGCAAGTTGCGCCCAAGGCCAAT-TGGTTGAGGGCACGTCCGCCTG---GGAGTCAA-GCATTACATCGCTTC

Zeuxine_strateumatica_SG1212 ATGAC-TCTCGGCAATGGATATCTT-GGCTCTTGCATCGATGAAGAGCGCAGCGAAATGCGATACGTGGTGT-GAATTGCAGAATCCCGTGAACC-ATCAAATTTTTGAACGCAAGTTGCGCCCAAGGCCAAT-TGGTTGAGGGCACGTCCGCCTG---GGAGTCAA-GCATTACATCGCTTC

;

end;

S2B: marK raw matrix.

#NEXUS

begin taxa;

dimensions ntax=824;

taxlabels

Acampe_praemorsa_var_longepedunculata_AB217702

Acampe_praemorsa_var_longepedunculata_KF421843

Acampe_praemorsa_var_longepedunculata_KJ733544

Acampe_praemorsa_var_longepedunculata_KY966701

Acampe_praemorsa_var_longepedunculata_PK12086

Acampe_praemorsa_var_longepedunculata_PK12159

Acampe_praemorsa_var_longepedunculata_SG1199

Acanthophippium_gougahense_KFBG3161

Ania_hongkongensis_KF673784

Ania_hongkongensis_KFBG4612

Ania_hongkongensis_KFBG4613

Ania_hongkongensis_KY966697

Ania_hongkongensis_PK12027

Ania_hongkongensis_SG1231

Ania_hongkongensis_SG1343

Ania_ruybarrettoi_KFBG43

Ania_ruybarrettoi_KY966707

Ania_ruybarrettoi_SG1395

Anoectochilus_formosanus_EU797513

Anoectochilus_formosanus_MG490281

Anoectochilus_formosanus_PK12215

Anoectochilus_roxburghii_EU817409

Anoectochilus_roxburghii_KF361656

Anoectochilus_roxburghii_KY966708

Anoectochilus_roxburghii_PK12043

Anoectochilus_roxburghii_PK12068

Anoectochilus_roxburghii_PK12069

Anoectochilus_roxburghii_SG1219

Aphyllorchis_montana_PK12147

Aphyllorchis_montana_PK12148

Aphyllorchis_montana_SG1010

Apostasia_nipponica_PK12273

Apostasia_nipponica_PK12274

Appendicula_cornuta_AY121739

Appendicula_cornuta_AY368393

Appendicula_cornuta_KF361651

Appendicula_cornuta_KY239521

Appendicula_cornuta_KY966709

Appendicula_cornuta_KY966710

Appendicula_cornuta_PK12065

Arundina_graminifolia_AF263626

Arundina_graminifolia_AF302692

Arundina_graminifolia_EF079333

Arundina_graminifolia_JN004354

Arundina_graminifolia_JN004355

Arundina_graminifolia_JN004356

Arundina_graminifolia_KF421845

Arundina_graminifolia_KX298566

Arundina_graminifolia_KY966712

Arundina_graminifolia_SG1008

Arundina_graminifolia_SG1206

Arundina_graminifolia_SG1295

Arundina_graminifolia_var_graminifolia_AB844195

Arundina_graminifolia_var_graminifolia_AB844196

Arundina_graminifolia_var_graminifolia_AB844197

Arundina_graminifolia_var_graminifolia_AB844198

Arundina_graminifolia_var_graminifolia_AB844199

Arundina_graminifolia_var_graminifolia_AB844200

Arundina_graminifolia_var_graminifolia_AB872252

Arundina_graminifolia_var_revoluta_AB844201

Arundina_graminifolia_var_revoluta_AB844202

Arundina_graminifolia_var_revoluta_AB844203

Arundina_graminifolia_var_revoluta_AB844204

Arundina_graminifolia_var_revoluta_AB844205

Arundina_graminifolia_var_revoluta_AB844206

Arundina_graminifolia_var_revoluta_AB844207

Arundina_graminifolia_var_revoluta_AB844208

Arundina_graminifolia_var_revoluta_AB844209

Arundina_graminifolia_var_revoluta_AB844210

Arundina_graminifolia_var_revoluta_AB844211

Arundina_graminifolia_var_revoluta_AB844212

Arundina_graminifolia_var_revoluta_AB844213

Arundina_graminifolia_var_revoluta_AB844214

Arundina_graminifolia_var_revoluta_AB844215

Arundina_graminifolia_var_revoluta_AB844216

Arundina_graminifolia_var_revoluta_AB844217

Bletilla_striata_AF263630

Bletilla_striata_EF079331

Bletilla_striata_EU490679

Bletilla_striata_GQ434129

Bletilla_striata_KC704596

Bletilla_striata_KC704597

Bletilla_striata_KF262041

Bletilla_striata_KF262042

Bletilla_striata_KF361655

Bletilla_striata_KF673785

Bletilla_striata_KF673786

Bletilla_striata_KFBG2048

Bletilla_striata_KY966713

Bletilla_striata_MG490283

Brachycorythis_galeandra_PK12195

Brachycorythis_galeandra_SG1261

Bulbophyllum_affine_KF361658

Bulbophyllum_affine_KF974494

Bulbophyllum_affine_KFBG412

Bulbophyllum_affine_KJ462086

Bulbophyllum_affine_KX455831

Bulbophyllum_affine_KY966714

Bulbophyllum_affine_SG1606

Bulbophyllum_affine_SG1607

Bulbophyllum_ambrosia_KF361657

Bulbophyllum_ambrosia_KY966715

Bulbophyllum_ambrosia_KY966716

Bulbophyllum_ambrosia_KY966717

Bulbophyllum_ambrosia_KY966718

Bulbophyllum_ambrosia_PK12090

Bulbophyllum_ambrosia_PK12092

Bulbophyllum_ambrosia_SG1221

Bulbophyllum_bicolor_CL10

Bulbophyllum_bicolor_FT28

Bulbophyllum_bicolor_KFBG2210

Bulbophyllum_bicolor_KFBG3073

Bulbophyllum_bicolor_KFBG433A

Bulbophyllum_bicolor_KFBG445

Bulbophyllum_bicolor_KY022445

Bulbophyllum_bicolor_KY966722

Bulbophyllum_bicolor_KY966723

Bulbophyllum_bicolor_KY966724

Bulbophyllum_bicolor_LMP19

Bulbophyllum_bicolor_PSL45

Bulbophyllum_bicolor_TMS05

Bulbophyllum_bicolor_TT43

Bulbophyllum_delitescens_KY966730

Bulbophyllum_delitescens_KY966731

Bulbophyllum_delitescens_KY966732

Bulbophyllum_delitescens_KY966733

Bulbophyllum_delitescens_SG1286

Bulbophyllum_delitescens_SG1287

Bulbophyllum_delitescens_SG1288

Bulbophyllum_kwangtungense_KFBG27001

Bulbophyllum_kwangtungense_KFBG2820

Bulbophyllum_kwangtungense_KY966740

Bulbophyllum_odoratissimum_FJ216638

Bulbophyllum_odoratissimum_FJ794042

Bulbophyllum_odoratissimum_KF974490

Bulbophyllum_odoratissimum_KP704477

Bulbophyllum_odoratissimum_KY966758

Bulbophyllum_odoratissimum_KY966759

Bulbophyllum_odoratissimum_PK12119

Bulbophyllum_odoratissimum_SG1275

Bulbophyllum_pectenveneris_KY966761

Bulbophyllum_pectenveneris_KY966762

Bulbophyllum_scabratum_KY966745

Bulbophyllum_scabratum_PK12041

Bulbophyllum_scabratum_PK12126

Bulbophyllum_scabratum_PK12127

Bulbophyllum_stenobulbon_KFBG2806

Bulbophyllum_stenobulbon_PK12111

Bulbophyllum_stenobulbon_PK12112

Bulbophyllum_stenobulbon_PK12113

Bulbophyllum_stenobulbon_SG1226

Bulbophyllum_tigridum_KFBG468

Bulbophyllum_tigridum_KX455836

Bulbophyllum_tigridum_PK12209

Bulbophyllum_tigridum_SG1310

Bulbophyllum_tseanum_SG1272

Bulbophyllum_tseanum_SG1616

Calanthe_dominyi_SG1359

Calanthe_graciliflora_KF673800

Calanthe_graciliflora_KF673801

Calanthe_graciliflora_KF852698

Calanthe_graciliflora_PK12206

Calanthe_graciliflora_PK12207

Calanthe_graciliflora_SG1225

Calanthe_masuca_KFBG11

Calanthe_masuca_PK12110

Calanthe_masuca_SG026

Calanthe_masuca_SG1360

Calanthe_masuca_SG1361

Calanthe_speciosa_KF673817

Calanthe_speciosa_KFBG136

Calanthe_speciosa_PK12168

Calanthe_speciosa_PK12169

Calanthe_speciosa_SG1313

Calanthe_speciosa_SG1368

Calanthe_triplicata_KF673822

Calanthe_triplicata_KF673849

Calanthe_triplicata_KF852713

Calanthe_triplicata_KFBG601

Calanthe_triplicata_KM894885

Calanthe_triplicata_KY966783

Calanthe_triplicata_SG1311

Cephalantheropsis_obcordata_KF673828

Cephalantheropsis_obcordata_KFBG140

Cephalantheropsis_obcordata_KFBG2520

Cephalantheropsis_obcordata_KY966786

Cephalantheropsis_obcordata_PK12079

Cephalantheropsis_obcordata_PK12080

Cheirostylis_monteiroi_SG1344

Cheirostylis_pusilla_HK43263

Cheirostylis_yunnanensis_PK12097

Cheirostylis_yunnanensis_SG1227

Cheirostylis_yunnanensis_SG1229

Chrysoglossum_assamicum_SG1622

Chrysoglossum_assamicum_SG1623

Cleisostoma_paniculatum_KFBG516

Cleisostoma_paniculatum_KJ733559

Cleisostoma_rostratum_KFBG789

Cleisostoma_rostratum_KJ733562

Cleisostoma_rostratum_KY966792

Cleisostoma_rostratum_PK12089

Cleisostoma_rostratum_PK12101

Cleisostoma_rostratum_PK12158

Cleisostoma_rostratum_SG1200

Cleisostoma_rostratum_SG1301

Cleisostoma_simondii_KJ733563

Cleisostoma_simondii_KY966793

Cleisostoma_simondii_KY966794

Cleisostoma_simondii_PK12176

Cleisostoma_simondii_SG1314

Cleisostoma_simondii_SG1327

Cleisostoma_simondii_SG1328

Cleisostoma_simondii_SG1329

Cleisostoma_simondii_SG1330

Cleisostoma_simondii_var_guangdongense_KJ733564

Cleisostoma_simondii_var_guangdongense_KY966696

Cleisostoma_williamsonii_KJ733567

Coelogyne_cantonensis_KY966934

Coelogyne_cantonensis_SG1239

Coelogyne_cantonensis_SG1339

Coelogyne_cantonensis_SG1388

Coelogyne_chinensis_AF448866

Coelogyne_chinensis_KF361635

Coelogyne_chinensis_KY966935

Coelogyne_chinensis_KY966936

Coelogyne_chinensis_PK12114

Coelogyne_chinensis_PK12115

Coelogyne_chinensis_SG1232

Coelogyne_chinensis_SG1251

Coelogyne_fimbriata_AF302710

Coelogyne_fimbriata_JF422078

Coelogyne_fimbriata_KF361619

Coelogyne_fimbriata_KFBG411

Coelogyne_fimbriata_KFBG523

Coelogyne_fimbriata_KR857337

Coelogyne_fimbriata_KR905392

Coelogyne_fimbriata_KY966796

Coelogyne_fimbriata_SG1059

Coelogyne_fimbriata_SG1061F

Coelogyne_fimbriata_SG1062

Coelogyne_fimbriata_SG1079

Coelogyne_fimbriata_var_leungiana_KFBG640

Coelogyne_fimbriata_var_leungiana_KY966797

Coelogyne_fimbriata_var_leungiana_SG1058

Collabium_chinense_KF673830

Collabium_chinense_KF852672

Crepidium_acuminatum_AB290892

Crepidium_acuminatum_JN004398

Crepidium_acuminatum_JN004399

Crepidium_acuminatum_JN004400

Crepidium_acuminatum_JN004401

Crepidium_acuminatum_JN004402

Crepidium_acuminatum_KJ459304

Crepidium_acuminatum_KX344572

Crepidium_allanii_KFBG4610

Crepidium_allanii_KFBG4611

Crepidium_cordilabium_PK12270

Crepidium_cordilabium_PK12271

Crepidium_cordilabium_PK12272

Crepidium_purpureum_PK12185

Crepidium_purpureum_SG1193

Crepidium_purpureum_SG1194

Crepidium_purpureum_SG1379

Cryptochilus_roseus_KFBG2118

Cryptochilus_roseus_KY239613

Cryptochilus_roseus_KY966803

Cryptochilus_roseus_KY966804

Cryptochilus_roseus_PK12087

Cryptochilus_roseus_PK12088

Cryptostylis_arachnites_PK12188

Cryptostylis_arachnites_SG1380

Curculigo_orchioides_SG1196

Cymbidium_aloifolium_AF470485

Cymbidium_aloifolium_HM137055

Cymbidium_aloifolium_JN004411

Cymbidium_aloifolium_JN004412

Cymbidium_aloifolium_KF421846

Cymbidium_aloifolium_KFBG2049

Cymbidium_aloifolium_KX298600

Cymbidium_aloifolium_KY966805

Cymbidium_aloifolium_MG019922

Cymbidium_ensifolium_AF263648

Cymbidium_ensifolium_AF470464

Cymbidium_ensifolium_HM137050

Cymbidium_ensifolium_KF262066

Cymbidium_ensifolium_KJ597877

Cymbidium_ensifolium_KJ597878

Cymbidium_ensifolium_KJ597879

Cymbidium_ensifolium_KJ597880

Cymbidium_ensifolium_KX298607

Cymbidium_ensifolium_PK12208

Cymbidium_ensifolium_SG1214

Cymbidium_ensifolium_SG1278

Cymbidium_ensifolium_SG1285

Cymbidium_kanran_JX202672

Cymbidium_kanran_KC704624

Cymbidium_kanran_KF262069

Cymbidium_kanran_KF673832

Cymbidium_kanran_KJ597870

Cymbidium_kanran_KJ597871

Cymbidium_kanran_KJ597872

Cymbidium_kanran_KJ597873

Cymbidium_lancifolium_AF470475

Cymbidium_lancifolium_HM137058

Cymbidium_lancifolium_KC704625

Cymbidium_lancifolium_KFBG734

Cymbidium_lancifolium_KJ597885

Cymbidium_lancifolium_KJ597886

Cymbidium_lancifolium_KT001524

Cymbidium_lancifolium_KX298612

Cymbidium_lancifolium_KY966806

Cymbidium_lancifolium_PK12128

Cymbidium_lancifolium_SG1274

Cymbidium_sinense_AF470480

Cymbidium_sinense_HM137056

Cymbidium_sinense_KC117305

Cymbidium_sinense_KJ597881

Cymbidium_sinense_KJ597882

Cymbidium_sinense_KJ597883

Cymbidium_sinense_KJ597884

Cymbidium_sinense_KT001526

Cymbidium_sinense_KX298618

Cymbidium_sinense_MF093731

Cymbidium_sinense_SG1213

Cymbidium_sinense_SG1218

Cymbidium_sinense_SG1341

Cymbidium_sinense_SG1342

Dendrobium_aduncum_FJ216659

Dendrobium_aduncum_HM055147

Dendrobium_aduncum_KF143427

Dendrobium_aduncum_KFBG8766

Dendrobium_aduncum_KJ187310

Dendrobium_aduncum_KP159290

Dendrobium_aduncum_KR075049

Dendrobium_aduncum_KR089057

Dendrobium_aduncum_MG490229

Dendrobium_aduncum_MG490230

Dendrobium_aduncum_MG490251

Dendrobium_anosmum_AB847694

Dendrobium_anosmum_AB972311

Dendrobium_anosmum_KF957849

Dendrobium_anosmum_KY966807

Dendrobium_anosmum_MG490279

Dendrobium_cf_mimicum_PK12237E

Dendrobium_cf_mimicum_PK12237J

Dendrobium_crumenatum_AB847734

Dendrobium_crumenatum_AB972308

Dendrobium_crumenatum_HM055235

Dendrobium_crumenatum_JF713399

Dendrobium_crumenatum_KC682479

Dendrobium_linawianum_AB847798

Dendrobium_linawianum_KP159291

Dendrobium_linawianum_KY966847

Dendrobium_linawianum_SG1347

Dendrobium_lindleyi_AB847799

Dendrobium_lindleyi_AB972313

Dendrobium_lindleyi_GQ248117

Dendrobium_lindleyi_GU906225

Dendrobium_lindleyi_HM055273

Dendrobium_lindleyi_HM055274

Dendrobium_lindleyi_JF713421

Dendrobium_lindleyi_KFBG203

Dendrobium_lindleyi_KY966848

Dendrobium_lindleyi_KY966849

Dendrobium_lindleyi_KY966850

Dendrobium_lindleyi_MF349993

Dendrobium_lindleyi_MF409032

Dendrobium_lindleyi_MG490247

Dendrobium_loddigesii_AB847803

Dendrobium_loddigesii_AF448864

Dendrobium_loddigesii_FJ216661

Dendrobium_loddigesii_GU565194

Dendrobium_loddigesii_KF143690

Dendrobium_loddigesii_KF361617

Dendrobium_loddigesii_KP159293

Dendrobium_loddigesii_KP704467

Dendrobium_loddigesii_KY966852

Dendrobium_loddigesii_LC086486

Dendrobium_loddigesii_MG490235

Dendrobium_loddigesii_SG1255

Dendrobium_spatella_AB847867

Dendrobium_spatella_KF143721

Dendrobium_spatella_KF143722

Dendrobium_spatella_SG1357

Dendrobium_spatella_SG1358

Dendrolirium_lasiopetalum_KFBG701

Dendrolirium_lasiopetalum_PK12173

Dendrolirium_lasiopetalum_PK12174

Dendrolirium_lasiopetalum_SG1312

Dienia_ophrydis_AY907177

Dienia_ophrydis_AY907181

Dienia_ophrydis_EF079340

Dienia_ophrydis_KF852679

Dienia_ophrydis_KFBG7819

Dienia_ophrydis_KJ459305

Dienia_ophrydis_SG1201

Dienia_ophrydis_SG1254

Dienia_ophrydis_SG1276

Diploprora_championii_KJ733568

Diploprora_championii_KX526719

Diploprora_championii_KY966879

Diploprora_championii_KY966880

Diploprora_championii_PK12025

Diploprora_championii_PK12029

Diploprora_championii_PK12095

Diploprora_championii_SG1144

Diploprora_championii_SG1230

Disperis_neilgherrensis_KM583464

Eria_scabrilinguis_KFBG916

Eria_scabrilinguis_KY239512

Eria_scabrilinguis_KY966883

Eria_scabrilinguis_PK12164

Eria_scabrilinguis_PK12165

Eria_scabrilinguis_SG1302

Erythrodes_blumei_JN166024

Erythrodes_blumei_KT385583

Erythrodes_blumei_PK12103

Erythrodes_blumei_PK12104

Eulophia_flava_JN004434

Eulophia_flava_JN004435

Eulophia_flava_SG1158

Eulophia_flava_SG1159

Eulophia_graminea_FJ565159

Eulophia_graminea_KF358078

Eulophia_graminea_MH767975

Eulophia_graminea_MH767976

Eulophia_graminea_SG1270

Eulophia_graminea_SG1350

Eulophia_picta_FR832768

Eulophia_picta_JN004440

Eulophia_picta_JN004441

Eulophia_picta_JN004442

Eulophia_picta_JN004443

Eulophia_picta_JN004444

Eulophia_picta_JN004445

Eulophia_picta_KF421850

Eulophia_picta_LN831613

Eulophia_picta_MG019924

Eulophia_picta_PK12046

Eulophia_picta_PK12047

Eulophia_picta_PK12048

Eulophia_picta_PK12137

Eulophia_picta_PK12138

Eulophia_picta_PK12153

Eulophia_picta_SG1271

Eulophia_zollingeri_SG1262

Eulophia_zollingeri_SG1263

Eulophia_zollingeri_SG1264

Eulophia_zollingeri_SG1265

Eulophia_zollingeri_SG1353

Gastrochilus_japonicus_KF262103

Gastrochilus_japonicus_KF545886

Gastrochilus_japonicus_KJ733575

Gastrochilus_japonicus_KY966885

Gastrochilus_japonicus_KY966886

Gastrochilus_kadooriei_PK12022

Gastrodia_peichatieniana_AFCDHK43268

Goodyera_foliosa_KT385599

Goodyera_foliosa_KT385600

Goodyera_foliosa_KT385601

Goodyera_foliosa_KT385602

Goodyera_foliosa_KT385603

Goodyera_foliosa_KT385604

Goodyera_foliosa_KT385605

Goodyera_foliosa_Pk12067

Goodyera_foliosa_SG1309

Goodyera_foliosa_SG1315

Goodyera_procera_JN004446

Goodyera_procera_JN004447

Goodyera_procera_JN004448

Goodyera_procera_JN004449

Goodyera_procera_JN004450

Goodyera_procera_KF421851

Goodyera_procera_KT385630

Goodyera_procera_KT385631

Goodyera_procera_KY966888

Goodyera_procera_SG1152

Goodyera_procera_SG1240

Goodyera_procera_SG1241

Goodyera_procera_SG1346

Goodyera_pusilla_JN166026

Goodyera_seikomontana_KT385644

Goodyera_seikomontana_KT385645

Goodyera_seikomontana_KY966695

Goodyera_seikoomontana_SG1252

Goodyera_seikoomontana_SG1253

Goodyera_seikoomontana_SG1354

Goodyera_viridiflora_AJ310035

Goodyera_viridiflora_JN166027

Goodyera_viridiflora_KT385652

Goodyera_viridiflora_KT385653

Goodyera_viridiflora_KT385654

Goodyera_viridiflora_KT385655

Goodyera_viridiflora_KT385656

Goodyera_viridiflora_PK12170

Goodyera_viridiflora_PK12172

Goodyera_viridiflora_SG1305

Goodyera_viridiflora_SG1306

Habenaria_ciliolaris_MF945424

Habenaria_ciliolaris_MF945533

Habenaria_dentata_JN696438

Habenaria_dentata_KF262035

Habenaria_dentata_KF262036

Habenaria_dentata_KFBG2126B

Habenaria_dentata_KY966891

Habenaria_dentata_PK12059

Habenaria_dentata_SG1005

Habenaria_dentata_SG1009

Habenaria_leptoceras_HM777855

Habenaria_leptoceras_KJ021370

Habenaria_leptoloba_PK12060

Habenaria_leptoloba_PK12160

Habenaria_leptoloba_PK12161

Habenaria_leptoloba_SG1304

Habenaria_leptoloba_SG1362

Habenaria_linguella_MF945416

Habenaria_linguella_PK12049

Habenaria_linguella_PK12134

Habenaria_linguella_PK12141

Habenaria_linguella_SG1195

Habenaria_linguella_SG1299

Habenaria_linguella_SG1300

Habenaria_reniformis_PK12142

Habenaria_reniformis_PK12143

Habenaria_reniformis_PK12144

Habenaria_reniformis_PK12145

Habenaria_reniformis_PK12146

Habenaria_reniformis_SG1296

Habenaria_reniformis_SG1297

Habenaria_rhodocheila_KJ452799

Habenaria_rhodocheila_KR350203

Habenaria_rhodocheila_KY966689

Habenaria_rhodocheila_MF945524

Habenaria_rhodocheila_PK12139

Habenaria_rhodocheila_SG1289

Habenaria_rhodocheila_SG1290

Habenaria_rhodocheila_SG1291

Hetaeria_youngsayei_KY966893

Hetaeria_youngsayei_SG1244

Hetaeria_youngsayei_SG1245

Lecanorchis_nigricans_SG1279

Liparis_bootanensis_KF361637

Liparis_bootanensis_KF852676

Liparis_bootanensis_KFBG238

Liparis_bootanensis_KJ459310

Liparis_bootanensis_KY966897

Liparis_bootanensis_PK12162

Liparis_bootanensis_SG1215

Liparis_bootanensis_SG1216

Liparis_bootanensis_SG1307

Liparis_bootanensis_SG1336

Liparis_ferruginea_SG1156

Liparis_ferruginea_SG1266

Liparis_ferruginea_SG1267

Liparis_gigantea_PK12116

Liparis_gigantea_PK12117

Liparis_gigantea_PK12118

Liparis_nervosa_AY907146

Liparis_nervosa_AY907158

Liparis_nervosa_EF065594

Liparis_nervosa_JN004484

Liparis_nervosa_JN004485

Liparis_nervosa_JN004486

Liparis_nervosa_JN004487

Liparis_nervosa_JN004488

Liparis_nervosa_JN004489

Liparis_nervosa_JN004490

Liparis_nervosa_JN004491

Liparis_nervosa_JN004492

Liparis_nervosa_KF262086

Liparis_nervosa_KFBG330

Liparis_nervosa_KJ459324

Liparis_nervosa_KU748298

Liparis_nervosa_KU748299

Liparis_nervosa_KU748300

Liparis_nervosa_SG1233

Liparis_nervosa_SG1234

Liparis_nervosa_SG1235

Liparis_odorata_KJ021029

Liparis_odorata_SG1256

Liparis_odorata_SG1257

Liparis_sootenzanensis_KJ021030

Liparis_sootenzanensis_SG1351

Liparis_sootenzanensis_SG1352

Liparis_stricklandiana_KF589879

Liparis_stricklandiana_KFBG124

Liparis_stricklandiana_KFBG818

Liparis_stricklandiana_KJ459328

Liparis_stricklandiana_KY966899

Liparis_stricklandiana_PK12091

Liparis_stricklandiana_SG1332

Liparis_stricklandiana_SG1333

Liparis_stricklandiana_SG1337

Liparis_viridiflora_AY907174

Liparis_viridiflora_KJ459329

Liparis_viridiflora_KY966900

Liparis_viridiflora_KY966901

Liparis_viridiflora_PK12120

Liparis_viridiflora_SG1308

Liparis_viridiflora_SG1338

Ludisia_discolor_AJ543911

Ludisia_discolor_KY966902

Ludisia_discolor_MG490282

Ludisia_discolor_SG1236

Ludisia_discolor_SG1237

Ludisia_discolor_SG1348

Nephelaphyllum_tenuiflorum_KC627280

Nephelaphyllum_tenuiflorum_KF673835

Nephelaphyllum_tenuiflorum_KF852689

Nephelaphyllum_tenuiflorum_KY966906

Nephelaphyllum_tenuiflorum_PK12121

Nephelaphyllum_tenuiflorum_PK12122

Nephelaphyllum_tenuiflorum_PK12123

Nephelaphyllum_tenuiflorum_SG1220

Nervilia_plicata_JN004511

Nervilia_plicata_JN004512

Nervilia_plicata_JN004513

Nervilia_plicata_JX865518

Nervilia_plicata_JX865519

Nervilia_plicata_KM986841

Nervilia_plicata_MG452083

Nervilia_plicata_SG1143

Nervilia_plicata_SG1277

Neuwiedia_zollingeri_var_singapureana_PK12124

Neuwiedia_zollingeri_var_singapureana_SG1268

Neuwiedia_zollingeri_var_singapureana_SG1269

Neuwiedia_zollingeri_var_singapureana_SG1340

Neuwiedia_zollingeri_var_singapureana_JN181463

Neuwiedia_zollingeri_var_singapureana_KC172551

Neuwiedia_zollingeri_var_singapureana_KY966907

Neuwiedia_zollingeri_var_singapureana_LC086542

Pachystoma_pubescens_FR832802

Pachystoma_pubescens_PK12107

Pachystoma_pubescens_PK12108

Paphiopedilum_purpuratum_KP312072

Paphiopedilum_purpuratum_KP312073

Paphiopedilum_purpuratum_PK12075

Paphiopedilum_purpuratum_PK12082A

Paphiopedilum_purpuratum_PK12083

Paphiopedilum_purpuratum_SG1149

Pecteilis_susannae_MF945423

Pecteilis_susannae_MF945437

Pecteilis_susannae_MF945502

Pecteilis_susannae_PK12051

Pecteilis_susannae_PK12136

Pecteilis_susannae_PK12154

Pecteilis_susannae_SG1292

Peristylus_calcaratus_MF945517

Peristylus_calcaratus_PK12053

Peristylus_calcaratus_SG1303

Peristylus_densus_JN004550

Peristylus_densus_JN004551

Peristylus_densus_JN004552

Peristylus_densus_JN004553

Peristylus_densus_JN004554

Peristylus_densus_JN004555

Peristylus_densus_JN004556

Peristylus_densus_JN004557

Peristylus_densus_JN004558

Peristylus_densus_JN004559

Peristylus_densus_KM651446

Peristylus_densus_MF945453

Peristylus_densus_SG1258

Peristylus_densus_SG1260

Peristylus_goodyeroides_MF945410

Peristylus_goodyeroides_MF945412

Peristylus_intrudens_PK12050

Peristylus_intrudens_PK12056

Peristylus_intrudens_SG1298

Peristylus_lacertifer_MF945477

Peristylus_lacertifer_MF945478

Peristylus_lacertifer_PK12149

Peristylus_lacertifer_PK12163

Peristylus_lacertifer_SG1006

Persitylus_tentaculatus_PK12062

Persitylus_tentaculatus_PK12171

Persitylus_tentaculatus_SG1007

Phaius_tancarvilleae_AB040205

Phaius_tancarvilleae_EF079306

Phaius_tancarvilleae_EU490700

Phaius_tancarvilleae_JN004564

Phaius_tancarvilleae_KF673843

Phaius_tancarvilleae_KF673844

Phaius_tancarvilleae_KF852704

Phaius_tancarvilleae_KF852707

Phaius_tancarvilleae_KP204599

Phaius_tancarvilleae_KP204600

Phaius_tancarvilleae_KP204601

Phaius_tancarvilleae_KY966927

Phaius_tankervilleae_PK12084

Phaius_tankervilleae_PK12085

Phaius_tankervilleae_PK12099

Phaius_wallichii_KF361633

Phaius_wallichii_KF673845

Phaius_wallichii_KY966928

Platanthera_mandarinorum_JN696443

Platanthera_mandarinorum_var_mandarinorum_KF262020

Platanthera_mandarinorum_var_mandarinorum_KF262021

Platanthera_minor_KJ452825

Platanthera_minor_KJ452835

Platanthera_minor_PK12030

Platanthera_minor_SG1154

Platanthera_minor_SG1223

Platanthera_minor_SG1224

Platanthera_minor_SG1238

Porpax_pusilla_PK12125

Porpax_pusilla_SG1334

Renanthera_coccinea_KJ733598

Rhomboda_abbreviata_KY966694

Rhomboda_abbreviata_PK12166

Rhomboda_abbreviata_PK12175

Robiquetia_succisa_KJ733601

Robiquetia_succisa_KY966951

Robiquetia_succisa_PK12155

Robiquetia_succisa_PK12156

Robiquetia_succisa_PK12157

Robiquetia_succisa_SG1293

Robiquetia_succisa_SG1294

Robiquetia_succisa_SG1345

Spathoglottis_pubescens_PK12135

Spathoglottis_pubescens_PK12140

Spathoglottis_pubescens_SG1205

Spiranthes_hongkongensis_MF286438

Spiranthes_hongkongensis_MF286439

Spiranthes_hongkongensis_MF286440

Spiranthes_hongkongensis_MF286442

Spiranthes_hongkongensis_MF286464

Spiranthes_hongkongensis_PK12028

Spiranthes_hongkongensis_PK12102

Spiranthes_hongkongensis_PK12179

Spiranthes_sinensis_AB040206

Spiranthes_sinensis_HE575508

Spiranthes_sinensis_JF972946

Spiranthes_sinensis_KC704665

Spiranthes_sinensis_KC704666

Spiranthes_sinensis_KC704667

Spiranthes_sinensis_KF262010

Spiranthes_sinensis_KF262011

Spiranthes_sinensis_KF262012

Spiranthes_sinensis_KF262013

Spiranthes_sinensis_KM262487

Spiranthes_sinensis_KM262488

Spiranthes_sinensis_KP769255

Spiranthes_sinensis_LT600870

Spiranthes_sinensis_MF286443

Spiranthes_sinensis_MF286444

Spiranthes_sinensis_MF286445

Spiranthes_sinensis_MF286446

Spiranthes_sinensis_MF286447

Spiranthes_sinensis_MF286450

Spiranthes_sinensis_MF286451

Spiranthes_sinensis_MF286452

Spiranthes_sinensis_MF286453

Spiranthes_sinensis_MF286454

Spiranthes_sinensis_MF286455

Spiranthes_sinensis_MF286456

Spiranthes_sinensis_MF286457

Spiranthes_sinensis_MF286458

Spiranthes_sinensis_MF286459

Spiranthes_sinensis_MF286460

Spiranthes_sinensis_MF286468

Spiranthes_sinensis_MF286469

Spiranthes_sinensis_MF286470

Spiranthes_sinensis_MF286471

Spiranthes_sinensis_MF286473

Spiranthes_sinensis_MF286474

Spiranthes_sinensis_MF286476

Spiranthes_sinensis_MF786821

Spiranthes_sinensis_MH036715

Spiranthes_sinensis_MH036716

Spiranthes_sinensis_MH036717

Spiranthes_sinensis_MH036718

Spiranthes_sinensis_MH036719

Spiranthes_sinensis_MH036720

Spiranthes_sinensis_MH036721

Spiranthes_sinensis_MH036722

Spiranthes_sinensis_MH036723

Spiranthes_sinensis_MH036724

Spiranthes_sinensis_MH036725

Spiranthes_sinensis_MH036726

Spiranthes_sinensis_MH036727

Spiranthes_sinensis_MH036728

Spiranthes_sinensis_MH785217

Spiranthes_sinensis_MH785218

Spiranthes_sinensis_PK12106

Spiranthes_sinensis_PK12109

Spiranthes_sinensis_SG1153

Spiranthes_sinensis_SG1155

Tainia_cordifolia_KF673834

Tainia_dunnii_KF673846

Tainia_dunnii_KF852708

Tainia_dunnii_SG1273

Thelasis_pygmaea_KY239491

Thrixspermum_centipeda_AB217767

Thrixspermum_centipeda_KFBG3066D

Thrixspermum_centipeda_KJ733621

Thrixspermum_centipeda_KY966960

Thrixspermum_centipeda_KY966961

Thrixspermum_centipeda_PK12129

Thrixspermum_centipeda_PK12130

Thrixspermum_centipeda_PK12131

Thrixspermum_centipeda_PK12132

Thrixspermum_centipeda_PK12133

Tropidia_curculigoides_SG1281

Tropidia_curculigoides_SG1282

Tropidia_curculigoides_SG1283

Tropidia_curculigoides_SG1284

Tropidia_nipponica_PK12181

Tropidia_nipponica_SG1355

Tropidia_nipponica_SG1356

Vanilla_shenzhenica_KFBG290

Vrydagzynea_nuda_SG1222

Vrydagzynea_nuda_SG1246A

Vrydagzynea_nuda_SG1246B

Vrydagzynea_nuda_SG1247

Zeuxine_boninensis_d16

Zeuxine_boninensis_Z3

Zeuxine_gracilis_JN166034

Zeuxine_gracilis_PK12066

Zeuxine_gracilis_SG1204

Zeuxine_strateumatica_AJ310080

Zeuxine_strateumatica_KY966975

Zeuxine_strateumatica_SG1211

Zeuxine_strateumatica_SG1212

;

end;

begin characters;

dimensions nchar=1446;

format datatype=dna missing=? gap=-;

matrix

Acampe_praemorsa_var_longepedunculata_AB217702 ACAAGAATTCTTTTTCTTATCATTTTTAT------------TCTCAAATGGTATCAGAAGGTTTTGGAGTCATTCTGGAAATTTCATTCTCGTCGCGATTAGTATCC---------TCCCTTGAAG---AAAAAAGAATACCAAAATCTCAGAATTTACGATCTATTCATTCAATATTTCCCTTTTTAGAGGATAAATTATCACATTTAAATTATGTGTCGG-ATC-TACT-AATACCCTATCCCATCCATC-TGGAAATATT-GGTTCAAATCCTTCAATGCTGG-ATCAAAGATGTTCCTTCT-TTGCATTTATTG-CAATTGAT--TTTCCACG------AATATCATAATT---------------TGAATAGTCTCAT---TACT-TC---------------AAAAAAATCCATTTACGTCT-TTTCAAAAAA---AAAGAAAAGAT-TCT-TTTGG-TTCCTACATA------ATTTTTAT-GTATAT-GAATGC----AAATATATAT-TCCTC---TTTCTTCGT--AAACAG-TCTTCT-TATT-TACG-AT-CAATAT-CTTCTGGA-GTCTAACT--GAGACAAACACATTT--TTATGGAAA--ATTAGGA------TATCTTAAAGT---TCTGTTCT-GTAATT------CTTTTCA---------GAGG-ATCCTATGGT-TATCCAA-AGATCTT-TTCA-TACATTAT------------GTCC-----GATATCCAGAAAAA---ACA-----ATAAA--GGCTTC-----AAAAG---GAACTCTT---------ATTC------------TGATGAATAAAT-GGAAATTT--CATTTT-GTGAA-TTTTTGGCA-ATC--TTATT-TTCACTTT-TGGTTTC--AACCTTATAGGA----TCCATATAAA-GCAATT--ACCCAATTATTCCTTCTCTTTTCTGGGA-TATTTTTCAAGTGTACTAAAAAA-TCCTTTGGT-AGTAA-GAAATC-AAATGCTAGAGAATTCATTA-CTAATAAAGACTCT-GACTAAGA-AATTAGATACCATA-GCTCCAGTT--ATTTATCTTATTGG-ATCATTGTCGAAAGCTCA-ATTTTGTACTGTATTAGGT-CATCCTGTTAGTAAACC-GATCTGGACCAATTTATCGGATTCTGA-------------TATCTTGGATCGATTTTGTCGGAT-----ATGTAGAAAT---CTTTGTCGT-TATCACAGCGGATCTTC-AAAGAA--------ACAGGTTTTGTATCGTATAAAGTA-TATACTTCGACTTTCGTGTGCTAG-AACTTTGGCTCGTAAACATAAAAGTACAGTACGCACTTTTATGCGAAGATTAGGTTCGGGATTCTTAG-AAGAATTTTTTTT------GGACGAAGAAC-AATCTCTTTCTT

Acampe_praemorsa_var_longepedunculata_KF421843 ------------------------------------------------------------------------------------------------------------------------------------------------------------------------------------------------------------------------------------------------------------------------------------------------------------------------------------------TTCCACG------AATATCATAATT---------------TGAATAGTCTCAT---TACT-TC---------------AAAAAAATCCATTTACGTCT-TTTCAAAAAA---AAAGAAAAGAT-TCT-TTTGG-TTCCTACATA------ATTTTTAT-GTATAT-GAATGC----GAATATATAT-TCCTC---TTTCTTCGT--AAACAG-TCTTCT-TATT-TACG-AT-CAATAT-CTTCTGGA-GTCTTTCTT-GAGCG-AACACATTT--TTATGGAAA--AATAGAA------TATCTTAGAGT---TGTTTCTT-GTAATT------CTTTTCA---------GAGG-ATCCTATGGT-TCCTCAA-AGATATT-TTCA-TACATTAT------------GTTC-----GATATCAAGGAAAA---GCA-----ATTTT--GGCTTC-----AAAAG---GAACTCTT---------ATTC------------TGATGAATAAAT-GGAAATTT--CATTTT-GTGAA-TTTTTGGCA-ATC--TTATT-TTCACTTT-TGGTTTC--AACCTTATAGGA----TCCATATAAA-GCAATT--ACCCAATTATTCCTTCTCTTTTCTGGGA-TATTTTTCAAGTGTACTAAAAAA-CCCTTTGGT-AGTAA-GAAATC-AAATGCTAGAGAATTCATTT-CTAATAAATACTCT-GACTAAGA-AATTAGATACCATA-GCTCCAGTT--CTTTTTCTTATTGG-ATCATTGTCGAAAGCTCA-ATTTTGTACTGTATTAGGT-CATCCTATTAGTAAACC-GATCTGGACCAATTTATCGGATTCTGA-------------TATTCTTGATCGATTTTGTCGGAT-----ATGTAGAAAT---CCTTGTCGT-TATCACAGCGGATCCTC-AAAGAA--------------------------------------------------------------------------------------------------------------------------------------------------------------------------

Acampe_praemorsa_var_longepedunculata_KJ733544 ACAAGAATTCTTTTTCTTATCATTTTTAT------------TCTCAAATGGTATCAGAAGGTTTTGGAGTCATTCTGGAAATTTCATTCTCGTCGCGATTAGTATCC---------TCCCTTGAAG---AAAAAAGAATACCAAAATCTCAGAATTTACGATCTATTCATTCAATATTTCCCTTTTTAGAGGATAAATTATCACATTTAAATTATGTGTCGG-ATC-TACT-AATACCCTATCCCATCCATC-TGGAAATCTT-GGTTCAAATCCTTCAATGCTGG-ATCAAAGATGTTCCTTCT-TTGCATTTCTTG-CGATTGAT--TTTCCACG------AATATCATAATT---------------TGAATAGTCTCAT---TACT-TC---------------AAAAAAATCCATTTACGTCT-TTTCAAAAAA---AAAGAAAAGAT-TCT-TTTGG-TTCCTACATA------ATTTTTAT-GTATAT-GAATGC----GAATATATAT-TCCTC---TTTCTTCGT--AAACAG-TCTTCT-TATT-TACG-AT-CAATAT-CTTCTGGA-GTCTTTCTT-GAGCG-AACACATTT--TTATGGAAA--AATAGAA------TATCTTAGAGT---CGTGTCTT-GTAATT------CTTTTCA---------GAGG-ATCCTATGGT-TCCTCAA-AGATATT-TTCA-TACATTAT------------GTTC-----GATATCAAGGAAAA---GCA-----ATTTT--GGCTTC-----AAAAG---GAACTCTT---------ATTC------------TGATGAATAAAT-GGAAATTT--CATTTT-GTGAA-TTTTTGGCA-ATC--TTATT-TTCACTTT-TGGTTTC--AACCTTATAGGA----TCCATATAAA-GCAATT--ACCCAATTATTCCTTCTCTTTTCTGGGA-TATTTTTCAAGTGTACTAAAAAA-CCCTTTGGT-AGTAA-GAAATC-AAATGCTAGAGAATTCATTT-CTAATAAATACTCT-GACTAAGA-AATTAGATACCATA-GCTCCAGTT--CTTTTTCTTATTGG-ATCATTGTCGAAAGCTCA-ATTTTGTACTGTATTAGGT-CATCCTATTAGTAAACC-GATCTGGACCAATTTATCGGATTCTGA-------------TATTCTTGATCGATTTTGTCGGAT-----ATGTAGAAAT---CTTTGTCGT-TATCACAGCGGATCCTC-AAAGAA--------ACAGGTTTTGTATCGTATAAAGTA-TATACTTCGACTTTCGTGTGCTAG-AACTTTGGCTCGTAAACATAAAAGTACAGTACGCACTTTTATGCGAAGATTAGGTTCGGGATTCTTAG-AAGAATTTTTTTT------GGAAGAAGAAC-AATTTCTTTCCT

Acampe_praemorsa_var_longepedunculata_KY966701 ACAAGAATTCTTTTTCTTATCATTTTTAT------------TCTCAAATGGTATCAGAAGGTTTTGGAGTCATTCTGGAAATTTCATTCTCGTCGCGATTAGTATCC---------TCCCTTGAAG---AAAAAAGAATACCAAAATCTCAAAATTTACGATCTATTCATTCAATATTTCCCTTTTTAGAGGATAAATTATCACATTTAAATTATGTGTCGG-ATC-TACT-AATACCCTATCCCATCCATC-TGGAAATCTT-GGTTCAAATCCTTCAATGCTGG-ATCAAAGATGTTCCTTCT-TTGCATTTCTTG-CGATTGAT--TTTCCACG------AATATCATAATT---------------TGAATAGTCTCAT---TACT-TC---------------AAAAAAATCCATTTACGTCT-TTTCAAAAAA---AAAGAAAAGAT-TCT-TTTGG-TTCCTACATA------ATTTTTAT-GTATAT-GAATGC----GAATATATAT-TCCTC---TTTCTTCGT--AAACAG-TCTTCT-TATT-TACG-AT-CAATAT-CTTCTGGA-GTCTTTCTT-GAGCG-AACACATTT--TTATGGAAA--AATAGAA------TATCTTAGAGT---CGTGTCTT-GTAATT------CTTTTCA---------GAGG-ATCCTATGGT-TCCTCAA-AGATATT-TTCA-TACATTAT------------GTTC-----GATATCAAGGAAAA---GCA-----ATTTT--GGCTTC-----AAAAG---GAACTCTT---------ATTC------------TGATGAATAAAT-GGAAATTT--CATTTT-GTGAA-TTTTTGGCA-ATC--TTATT-TTCACTTT-TGGTTTC--AACCTTATAGGA----TCCATATAAA-GCAATT--ACCCAATTATTCCTTCTCTTTTCTGGGA-TATTTTTCAAGTGTACTAAAAAA-CCCTTTGGT-AGTAA-GAAATC-AAATGCTAGAGAATTCATTT-CTAATAAATACTCT-GACTAAGA-AATTAGATACCATA-GCTCCAGTT--CTTTTTCTTATTGG-ATCATTGTCGAAAGCTCA-ATTTTGTACTGTATTAGGT-CATCCTATTAGTAAACC-GATCTGGACCAATTTATCGGATTCTGA-------------TATTCTTGATCGATTTTGTCGGAT-----ATGTAGAAAT---CTTTGTCGT-TATCACAGCGGATCCTC-AAAGAA--------ACAGGTTTTGTATCGTATAAAGTA-TATACTTCGACTTTCGTGTGCTAG-AACTTTGGCTCGTAAACATAAAAGTACAGTACGCACTTTTATGCGAAGATTAGGTTCGGGATTCTTAG-AAGAATTTTT---------------------------------

Acampe_praemorsa_var_longepedunculata_PK12086 ACAAGAATTCTTTTTCTTATCATTTTTAT------------TCTCAAATGGTATCAGAAGGTTTTGGAGTCATTCTGGAAATTTCATTCTCGTCGCGATTAGTATCC---------TCCCTTGAAG---AAAAAAGAATACCAAAATCTCAGAATTTACGATCTATTCATTCAATATTTCCCTTTTTAGAGGATAAATTATCACATTTAAATTATGTGTCGG-ATC-TACT-AATACCCTATCCCATCCATC-TGGAAATCTT-GGTTCAAATCCTTCAATGCTGG-ATCAAAGATGTTCCTTCT-TTGCATTTCTTG-CGATTGAT--TTTCCACG------AATATCATAATT---------------TGAATAGTCTCAT---TACT-TC---------------AAAAAAATCCATTTACGTCT-TTTCAAAAAA---AAAGAAAAGAT-TCT-TTTGG-TTCCTACATA------ATTTTTAT-GTATAT-GAATGC----GAATATATAT-TCCTC---TTTCTTCGT--AAACAG-TCTTCT-TATT-TACG-AT-CAATAT-CTTCTGGA-GTCTTTCTT-GAGCG-AACACATTT--TTATGGAAA--AATAGAA------TATCTTAGAGT---CGTGTCTT-GTAATT------CTTTTCA---------GAGG-ATCCTATGGT-TCCTCAA-AGATATT-TTCA-TACATTAT------------GTTC-----GATATCAAGGAAAA---GCA-----ATTTT--GGCTTC-----AAAAG---GAACTCTT---------ATTC------------TGATGAATAAAT-GGAAATTT--CATTTT-GTGAA-TTTTTGGCA-ATC--TTATT-TTCACTTT-TGGTTTC--AACCTTATAGGA----TCCATATAAA-GCAATT--ACCCAATTATTCCTTCTCTTTTCTGGGA-TATTTTTCAAGTGTACTAAAAAA-CCCTTTGGT-AGTAA-GAAATC-AAATGCTAGAGAATTCATTT-CTAATAAATACTCT-GACTAAGA-AATTAGATACCATA-GCTCCAGTT--CTTTTTCTTATTGG-ATCATTGTCGAAAGCTCA-ATTTTGTACTGTATTAGGT-CATCCTATTAGTAAACC-GATCTGGACCAATTTATCGGATTCTGA-------------TATTCTTGATCGATTTTGTCGGAT-----ATGTAGAAAT---CTTTGTCGT-TATCACAGCGGATCCTC-AAAGAA--------ACAGGTTTTGTATCGTATAAAGTA-TATACTTCGACTTTCGTGTGCTAG-AACTTTGGCTCGTAAACATAAAAGTACAGTACGCACTTTTATGCGAAGATTAGGTTCGGGATTCTTAG-AAGAATTTTTTTT------GGAAGAAGAAC-AATTTCTTTCCT

Acampe_praemorsa_var_longepedunculata_PK12159 ACAAGAATTCTTTTTCTTATCATTTTTAT------------TCTCAAATGGTATCAGAAGGTTTTGGAGTCATTCTGGAAATTTCATTCTCGTCGCGATTAGTATCC---------TCCCTTGAAG---AAAAAAGAATACCAAAATCTCAGAATTTACGATCTATTCATTCAATATTTCCCTTTTTAGAGGATAAATTATCACATTTAAATTATGTGTCGG-ATC-TACT-AATACCCTATCCCATCCATC-TGGAAATCTT-GGTTCAAATCCTTCAATGCTGG-ATCAAAGATGTTCCTTCT-TTGCATTTCTTG-CGATTGAT--TTTCCACG------AATATCATAATT---------------TGAATAGTCTCAT---TACT-TC---------------AAAAAAATCCATTTACGTCT-TTTCAAAAAA---AAAGAAAAGAT-TCT-TTTGG-TTCCTACATA------ATTTTTAT-GTATAT-GAATGC----GAATATATAT-TCCTC---TTTCTTCGT--AAACAG-TCTTCT-TATT-TACG-AT-CAATAT-CTTCTGGA-GTCTTTCTT-GAGCG-AACACATTT--TTATGGAAA--AATAGAA------TATCTTAGAGT---CGTGTCTT-GTAATT------CTTTTCA---------GAGG-ATCCTATGGT-TCCTCAA-AGATATT-TTCA-TACATTAT------------GTTC-----GATATCAAGGAAAA---GCA-----ATTTT--GGCTTC-----AAAAG---GAACTCTT---------ATTC------------TGATGAATAAAT-GGAAATTT--CATTTT-GTGAA-TTTTTGGCA-ATC--TTATT-TTCACTTT-TGGTTTC--AACCTTATAGGA----TCCATATAAA-GCAATT--ACCCAATTATTCCTTCTCTTTTCTGGGA-TATTTTTCAAGTGTACTAAAAAA-CCCTTTGGT-AGTAA-GAAATC-AAATGCTAGAGAATTCATTT-CTAATAAATACTCT-GACTAAGA-AATTAGATACCATA-GCTCCAGTT--CTTTTTCTTATTGG-ATCATTGTCGAAAGCTCA-ATTTTGTACTGTATTAGGT-CATCCTATTAGTAAACC-GATCTGGACCAATTTATCGGATTCTGA-------------TATTCTTGATCGATTTTGTCGGAT-----ATGTAGAAAT---CTTTGTCGT-TATCACAGCGGATCCTC-AAAGAA--------ACAGGTTTTGTATCGTATAAAGTA-TATACTTCGACTTTCGTGTGCTAG-AACTTTGGCTCGTAAACATAAAAGTACAGTACGCACTTTTATGCGAAGATTAGGTTCGGGATTCTTAG-AAGAATTTTTTTT------GGAAGAAGAAC-AATTTCTTTCCT

Acampe_praemorsa_var_longepedunculata_SG1199 ACAAGAATTCTTTTTCTTATCATTTTTAT------------TCTCAAATGGTATCAGAAGGTTTTGGAGTCATTCTGGAAATTTCATTCTCGTCGCGATTAGTATCC---------TCCCTTGAAG---AAAAAAGAATACCAAAATCTCAGAATTTACGATCTATTCATTCAATATTTCCCTTTTTAGAGGATAAATTATCACATTTAAATTATGTGTCGG-ATC-TACT-AATACCCTATCCCATCCATC-TGGAAATCTT-GGTTCAAATCCTTCAATGCTGG-ATCAAAGATGTTCCTTCT-TTGCATTTCTTG-CGATTGAT--TTTCCACG------AATATCATAATT---------------TGAATAGTCTCAT---TACT-TC---------------AAAAAAATCCATTTACGTCT-TTTCAAAAAA---AAAGAAAAGAT-TCT-TTTGG-TTCCTACATA------ATTTTTAT-GTATAT-GAATGC----GAATATATAT-TCCTC---TTTCTTCGT--AAACAG-TCTTCT-TATT-TACG-AT-CAATAT-CTTCTGGA-GTCTTTCTT-GAGCG-AACACATTT--TTATGGAAA--AATAGAA------TATCTTAGAGT---CGTGTCTT-GTAATT------CTTTTCA---------GAGG-ATCCTATGGT-TCCTCAA-AGATATT-TTCA-TACATTAT------------GTTC-----GATATCAAGGAAAA---GCA-----ATTTT--GGCTTC-----AAAAG---GAACTCTT---------ATTC------------TGATGAATAAAT-GGAAATTT--CATTTT-GTGAA-TTTTTGGCA-ATC--TTATT-TTCACTTT-TGGTTTC--AACCTTATAGGA----TCCATATAAA-GCAATT--ACCCAATTATTCCTTCTCTTTTCTGGGA-TATTTTTCAAGTGTACTAAAAAA-CCCTTTGGT-AGTAA-GAAATC-AAATGCTAGAGAATTCATTT-CTAATAAATACTCT-GACTAAGA-AATTAGATACCATA-GCTCCAGTT--CTTTTTCTTATTGG-ATCATTGTCGAAAGCTCA-ATTTTGTACTGTATTAGGT-CATCCTATTAGTAAACC-GATCTGGACCAATTTATCGGATTCTGA-------------TATTCTTGATCGATTTTGTCGGAT-----ATGTAGAAAT---CTTTGTCGT-TATCACAGCGGATCCTC-AAAGAA--------ACAGGTTTTGTATCGTATAAAGTA-TATACTTCGACTTTCGTGTGCTAG-AACTTTGGCTCGTAAACATAAAAGTACAGTACGCACTTTTATGCGAAGATTAGGTTCGGGATTCTTAG-AAGAATTTTTTTT------GGAAGAAGAAC-AATTTCTTTCCT

Acanthophippium_gougahense_KFBG3161 ACAAGAATTCTTTTTCTTCTCATTTTTCT------------TCTCAAATGGTATCAGAAGGTTTTGGAGTCATTCTGGAAATTCCATTCTCGTCGCGATTAGTATCT---------TCCCTTGAAG---AAAAAAGAATACCAAAATCTCAGAATTTACGATCTATTCATTCAATATTTCCCTTTTTAGAGGATAAATTCTCACATTTAAATTATGTGTCAG-ATC-TACT-AATACCCCATCCCATCCATC-TGGAAATCTT-GGTTCAAATCCTTCAATGCTGG-ATCAAAGATGTTCCTTCT-TTGCATTTATTG-CGATTGTT--TTTCCACG------AATATCATAATT---------------TGAATAGTCTCAT---TACT-TC---------------AATGAAATCCATTTACGTCT-TTTCAAAAAG---AAAGAAAAGAT-TCT-TTCGG-TTCCTACATA------ATTCTTAT-GTATAT-GAATGC----GAATATCTAT-TCCTG---TTTCTTCGT--AAACAG-TCTTCT-TATT-TACG-AT-CAAGAT-CTTCTGGA-GTCTTTCTT-GAGCG-AACACATTT--CTATGGAAA--AATAGAA------TATCTTATAGT---CGTGTGTT-GTAATT------ATTTTCA---------GAGG-ATCCTATGGT-TCCTCAA-AGATACT-TTCA-TACATTAT------------GTTC-----GATATCAAGGAAAA---GCG-----ATTCT--GGCTTC-----AAAAG---GAACTCTT---------ATTC------------TGATGAAGAAAT-GGAAATTT--CATCTT-GTAAA-TTTTTGGCA-ATC--TTATT-TTCACTTT-TGGTTTC--AACCTTATAGGA----TCCTTATAAA-GCAATT--ACCTAACTATTCCTTCTCTTTTCTGGGG-TATTTTTCAAGTGTACTAAAAAA-TCCTTTGGT-AGTAA-GAAATC-AAATGCTAGAGAATTCATTT-CTAATAAATACTCT-GACTAAGA-AATTAGATACCATA-GCCCCAGTT--ATTTCTCTTATTGG-ATCATTGTCGAAAGCTCA-ATTTTGTACTGTATTGGGT-CATCCTATTAGTAAACC-AATCTGGACCGATTTATCGGATTCTGA-------------TATTCTTTATCGATTTTGTCGGAT-----ATGTAGAAAT---CTTTGTCAT-TATTACAGTGGATCTTC-AAAGAA--------ACAGGTTTTGTATCGTATAAAGTA-TATACTTCGACTTTCGTGTGCTAG-AACTTTGGCTCGTAAACATAAAAGTACAGTACGCACTTTTATGCGAAGATTAGGTTCGGGATTCTTAG-AAGAATTTTTTTT------GGAAGAAGAAC-AATCTCTTTCTT

Ania_hongkongensis_KF673784 ACAAGAATTCTTTTTCTTCTCATTTTTCT------------TCTCAAATGGTATCAGAAGGTTTTGGAGTCATTCTGGAAATTCCATTCTCGTCGCGATTAGTATCT---------TCCCTTGAAG---AAAAAAGAATACCAAAATCTCAGAATTTACGATCTATTCATTCAATATTTCCCTTTTTAGAGGATAAATTATCACATTTAAATTATGTGTCAG-ATC-TACT-AATACCCCATCCCATCCATC-TGGAAATCTT-GGTTCAAATCCTTCAATGCTGG-ATCAAAGATGTTCCTTCT-TTGCATTTATTG-CGATTGTT--TTTCCACG------AATATCATAATT---------------TGAATAGTCTCAT---TACT-TC---------------AAAGAAATCCATTTACGTCT-TTTCAAAAAG---AAAGAAAAGAT-TCT-TTTGG-TTCCTACATA------ATTCTTAT-GTATAT-GAATGC----GAATATCTAT-TCCTG---TTTCTTCGT--AAACAG-TCTTCT-TATT-TACG-AT-CAATAT-CTTCTGGA-GTCTTTCTT-GAGCG-AACACATTT--CTATGGAAA--AATAGAA------TATCTTATAGT---CGTGTGTT-GTAATT------CTTTTCA---------GAGG-ATCCTATGGT-TCCTTAA-AGATACT-TTCA-TACATTAT------------GTTC-----GATATCAAGGAAAA---GCG-----ATTAT--GGCTTC-----AAAAG---GAACTCTT---------ATTC------------TGATGAAGAAAT-GGAAATTT--CATCTT-GTAAA-TTTTTGGCA-ATC--TTATT-TTCACTTT-TGGTTTC--AACCTTATAGGA----TCCATATAAA-GCAATT--ACCCAACTATTTCTTCTCTTTTCTGGGG-TATTTTTCAAGTGTACTAAAAAA-CCCTTTGGT-AGTAA-GAAATC-AAATGCTAGAGAATTCATTT-CTAATAAATACTCT-GACTAAGA-AATTAGATACCATA-GCCCCAGTT--ATTTCTCTTATTGG-ATCATTGTCGAAAGCTCA-ATTTTGTACTGTATTGGGT-CATCCTATTAGTAAACC-AATCTGGACCGATTTATCGGATTCTGA-------------TATTCTTGATCGATTTTGTCGGAT-----ATCTAGAAAT---CTTTGTCGT-TATCACAGCGGATCCTC-AAAGAA--------ACAGGTTTTATATCGTATAAAGTA-TATACTTCGACTTTCGTGTGCTAG-AACTTTGGCTCGTAAACATAAAAGTACAGTACGCACTCTTATGCGAAGATTAGGTTCGGGATTCTTAG-AAGAATTTTTTTT------GGAAGAAGAAC-AATCTCTTTCTT

Ania_hongkongensis_KFBG4612 ACAAGAATTCTTTTTCTTCTCATTTTTCT------------TCTCAAATGGTATCAGAAGGTTTTGGAGTCATTCTGGAAATTCCATTCTCGTCGCGATTAGTATCT---------TCCCTTGAAG---AAAAAAGAATACCAAAATCTCAGAATTTACGATCTATTCATTCAATATTTCCCTTTTTAGAGGATAAATTATCACATTTAAATTATGTGTCAG-ATC-TACT-AATACCCCATCCCATCCATC-TGGAAATCTT-GGTTCAAATCCTTCAATGCTGG-ATCAAAGATGTTCCTTCT-TTGCATTTATTG-CGATTGTT--TTTCCACG------AATATCATAATT---------------TGAATAGTCTCAT---TACT-TC---------------AAAGAAATCCATTTACGTCT-TTTCAAAAAG---AAAGAAAAGAT-TCT-TTTGG-TTCCTACATA------ATTCTTAT-GTATAT-GAATGC----GAATATCTAT-TCCTG---TTTCTTCGT--AAACAG-TCTTCT-TATT-TACG-AT-CAATAT-CTTCTGGA-GTCTTTCTT-GAGCG-AACACATTT--CTATGGAAA--AATAGAA------TATCTTATAGT---CGTGTGTT-GTAATT------CTTTTCA---------GAGG-ATCCTATGGT-TCCTTAA-AGATACT-TTCA-TACATTAT------------GTTC-----GATATCAAGGAAAA---GCG-----ATTAT--GGCTTC-----AAAAG---GAACTCTT---------ATTC------------TGATGAAGAAAT-GGAAATTT--CATCTT-GTAAA-TTTTTGGCA-ATC--TTATT-TTCACTTT-TGGTTTC--AACCTTATAGGA----TCCATATAAA-GCAATT--ACCCAACTATTTCTTCTCTTTTCTGGGG-TATTTTTCAAGTGTACTAAAAAA-CCCTTTGGT-AGTAA-GAAATC-AAATGCTAGAGAATTCATTT-CTAATAAATACTCT-GACTAAGA-AATTAGATACCATA-GCCCCAGTT--ATTTCTCTTATTGG-ATCATTGTCGAAAGCTCA-ATTTTGTACTGTATTGGGT-CATCCTATTAGTAAACC-AATCTGGACCGATTTATCGGATTCTGA-------------TATTCTTGATCGATTTTGTCGGAT-----ATCTAGAAAT---CTTTGTCGT-TATCACAGCGGATCCTC-AAAGAA--------ACAGGTTTTATATCGTATAAAGTA-TATACTTCGACTTTCGTGTGCTAG-AACTTTGGCTCGTAAACATAAAAGTACAGTACGCACTCTTATGCGAAGATTAGGTTCGGGATTCTTAG-AATAATTTTTTTT------GGAAGAAGAAC-AATCTCTTTCTT

Ania_hongkongensis_KFBG4613 ACAAGAATTCTTTTTCTTCTCATTTTTCT------------TCTCAAATGGTATCAGAAGGTTTTGGAGTCATTCTGGAAATTCCATTCTCGTCGCGATTAGTATCT---------TCCCTTGAAG---AAAAAAGAATACCAAAATCTCAGAATTTACGATCTATTCATTCAATATTTCCCTTTTTAGAGGATAAATTATCACATTTAAATTATGTGTCAG-ATC-TACT-AATACCCCATCCCATCCATC-TGGAAATCTT-GGTTCAAATCCTTCAATGCTGG-ATCAAAGATGTTCCTTCT-TTGCATTTATTG-CGATTGTT--TTTCCACG------AATATCATAATT---------------TGAATAGTCTCAT---TACT-TC---------------AAAGAAATCCATTTACGTCT-TTTCAAAAAG---AAAGAAAAGAT-TCT-TTTGG-TTCCTACATA------ATTCTTAT-GTATAT-GAATGC----GAATATCTAT-TCCTG---TTTCTTCGT--AAACAG-TCTTCT-TATT-TACG-AT-CAATAT-CTTCTGGA-GTCTTTCTT-GAGCG-AACACATTT--CTATGGAAA--AATAGAA------TATCTTATAGT---CGTGTGTT-GTAATT------CTTTTCA---------GAGG-ATCCTATGGT-TCCTTAA-AGATACT-TTCA-TACATTAT------------GTTC-----GATATCAAGGAAAA---GCG-----ATTAT--GGCTTC-----AAAAG---GAACTCTT---------ATTC------------TGATGAAGAAAT-GGAAATTT--CATCTT-GTAAA-TTTTTGGCA-ATC--TTATT-TTCACTTT-TGGTTTC--AACCTTATAGGA----TCCATATAAA-GCAATT--ACCCAACTATTTCTTCTCTTTTCTGGGG-TATTTTTCAAGTGTACTAAAAAA-CCCTTTGGT-AGTAA-GAAATC-AAATGCTAGAGAATTCATTT-CTAATAAATACTCT-GACTAAGA-AATTAGATACCATA-GCCCCAGTT--ATTTCTCTTATTGG-ATCATTGTCGAAAGCTCA-ATTTTGTACTGTATTGGGT-CATCCTATTAGTAAACC-AATCTGGACCGATTTATCGGATTCTGA-------------TATTCTTGATCGATTTTGTCGGAT-----ATCTAGAAAT---CTTTGTCGT-TATCACAGCGGATCCTC-AAAGAA--------ACAGGTTTTATATCGTATAAAGTA-TATACTTCGACTTTCGTGTGCTAG-AACTTTGGCTCGTAAACATAAAAGTACAGTACGCACTCTTATGCGAAGATTAGGTTCGGGATTCTTAG-AAGAATTTTTTTT------GGAAGAAGAAC-AATCTCTTTCTT

Ania_hongkongensis_KY966697 ACAAGAATTCTTTTTCTTCTCATTTTTCT------------TCTCAAATGGTATCAGAAGGTTTTGGAGTCATTCTGGAAATTCCATTCTCGTCGCGATTAGTATCT---------TCCCTTGAAG---AAAAAAGAATACCAAAATCTCAGAATTTACGATCTATTCATTCAATATTTCCCTTTTTAGAGGATAAATTATCACATTTAAATTATGTGTCAG-ATC-TACT-AATACCCCATCCCATCCATC-TGGAAATCTT-GGTTCAAATCCTTCAATGCTGG-ATCAAAGATGTTCCTTCT-TTGCATTTATTG-CGATTGTT--TTTCCACG------AATATCATAATT---------------TGAATAGTCCCAT---TACT-TC---------------AAAGAAATCCATTTACGTCT-TTTCAAAAAG---AAAGAAAAGAT-TCT-TTTGG-TTCCTACATA------AGAATTAT-GTATAT-GAATGC----GAATATCTAT-TCCTG---TTTCTTCGT--AAACAG-TCTTCT-TATT-TACG-AT-CAATAT-CTTCTGGA-GTCTTTCTT-GAGCG-AACACATTT--CTATGGAAA--AAGAGAA------TATCTTCTAGT---CGTGTGTT-GTAATT------CTTTTCA---------GAGG-ATCCTATGGT-TCCTCAA-AGATACT------TTCATTAT------------GTTC-----GATATCAAGGAAAA---GCG-----ATTCT--GGCTTC-----AAAAG---GAACTCTT---------ATTC------------TTATGAATAAAT-GGAAATTT--CATCTT-GTGAA-TTTTTGGCA-ATC--TTATT-TTCACTTT-TGGTTTC--AACC------GA----TCCATAGAAA-GCAATT--ACCCAACTATTCCTTC-------TGGGG-TCTTTTTCAAGTGTACTAAAAAA-TCCTTTGGT-AGTAA-GAAATC-AAATGCTAGAGAATTCATTT-CTCAGAAATACTCT-GACTAAGA-AATTAGATACCATA-G-CCCAGTT--ATTTCTCTTATTGG-ATCATTT------GCTCA-ATTTTGTACTGTATTGGGT-CATCCTATTAGTAAACC-GATCTGGACCGATTTCTCGGATTCTGA-------------TATTCTTGATCGATTTTGTCGGAT-----ATGTAGAAAT---CTTTGTCGT-TATCACAGCGGATCCTC-AAAGAA--------ACAGGTTTTGTATCGTAGAAAGTA-TATACTTCGACTTTCGTGTGCTAG-AACTTTGGCTCGTAAACATAAAAGTACAGTACGCACTCTTATGCGAAGATTAGGTTCGGGATTCTTAG-AAGAATTTTTTTT------GGAAGAAGAAC-AATCTCTTTCTT

Ania_hongkongensis_PK12027 ACAAGAATTCTTTTTCTTCTCATTTTTCT------------TCTCAAATGGTATCAGAAGGTTTTGGAGTCATTCTGGAAATTCCATTCTCGTCGCGATTAGTATCT---------TCCCTTGAAG---AAAAAAGAATACCAAAATCTCAGAATTTACGATCTATTCATTCAATATTTCCCTTTTTAGAGGATAAATTATCACATTTAAATTATGTGTCAG-ATC-TACT-AATACCCCATCCCATCCATC-TGGAAATCTT-GGTTCAAATCCTTCAATGCTGG-ATCAAAGATGTTCCTTCT-TTGCATTTATTG-CGATTGTT--TTTCCACG------AATATCATAATT---------------TGAATAGTCTCAT---TACT-TC---------------AAAGAAATCCATTTACGTCT-TTTCAAAAAG---AAAGAAAAGAT-TCT-TTTGG-TTCCTACATA------ATTCTTAT-GTATAT-GAATGC----GAATATCTAT-TCCTG---TTTCTTCGT--AAACAG-TCTTCT-TATT-TACG-AT-CAATAT-CTTCTGGA-GTCTTTCTT-GAGCG-AACACATTT--CTATGGAAA--AATAGAA------TATCTTATAGT---CGTGTGTT-GTAATT------CTTTTCA---------GAGG-ATCCTATGGT-TCCTTAA-AGATACT-TTCA-TACATTAT------------GTTC-----GATATCAAGGAAAA---GCG-----ATTAT--GGCTTC-----AAAAG---GAACTCTT---------ATTC------------TGATGAAGAAAT-GGAAATTT--CATCTT-GTAAA-TTTTTGGCA-ATC--TTATT-TTCACTTT-TGGTTTC--AACCTTATAGGA----TCCATATAAA-GCAATT--ACCCAACTATTTCTTCTCTTTTCTGGGG-TATTTTTCAAGTGTACTAAAAAA-CCCTTTGGT-AGTAA-GAAATC-AAATGCTAGAGAATTCATTT-CTAATAAATACTCT-GACTAAGA-AATTAGATACCATA-GCCCCAGTT--ATTTCTCTTATTGG-ATCATTGTCGAAAGCTCA-ATTTTGTACTGTATTGGGT-CATCCTATTAGTAAACC-AATCTGGACCGATTTATCGGATTCTGA-------------TATTCTTGATCGATTTTGTCGGAT-----ATCTAGAAAT---CTTTGTCGT-TATCACAGCGGATCCTC-AAAGAA--------ACAGGTTTTATATCGTATAAAGTA-TATACTTCGACTTTCGTGTGCTAG-AACTTTGGCTCGTAAACATAAAAGTACAGTACGCACTCTTATGCGAAGATTAGGTTCGGGATTCTTAG-AAGAATTTTTTTT------GGAAGAAGAAC-AATCTCTTTCTT

Ania_hongkongensis_SG1231 ACAAGAATTCTTTTTCTTCTCATTTTTCT------------TCTCAAATGGTATCAGAAGGTTTTGGAGTCATTCTGGAAATTCCATTCTCGTCGCGATTAGTATCT---------TCCCTTGAAG---AAAAAAGAATACCAAAATCTCAGAATTTACGATCTATTCATTCAATATTTCCCTTTTTAGAGGATAAATTATCACATTTAAATTATGTGTCAG-ATC-TACT-AATACCCCATCCCATCCATC-TGGAAATCTT-GGTTCAAATCCTTCAATGCTGG-ATCAAAGATGTTCCTTCT-TTGCATTTATTG-CGATTGTT--TTTCCACG------AATATCATAATT---------------TGAATAGTCTCAT---TACT-TC---------------AAAGAAATCCATTTACGTCT-TTTCAAAAAG---AAAGAAAAGAT-TCT-TTTGG-TTCCTACATA------ATTCTTAT-GTATAT-GAATGC----GAATATCTAT-TCCTG---TTTCTTCGT--AAACAG-TCTTCT-TATT-TACG-AT-CAATAT-CTTCTGGA-GTCTTTCTT-GAGCG-AACACATTT--CTATGGAAA--AATAGAA------TATCTTATAGT---CGTGTGTT-GTAATT------CTTTTCA---------GAGG-ATCCTATGGT-TCCTTAA-AGATACT-TTCA-TACATTAT------------GTTC-----GATATCAAGGAAAA---GCG-----ATTAT--GGCTTC-----AAAAG---GAACTCTT---------ATTC------------TGATGAAGAAAT-GGAAATTT--CATCTT-GTAAA-TTTTTGGCA-ATC--TTATT-TTCACTTT-TGGTTTC--AACCTTATAGGA----TCCATATAAA-GCAATT--ACCCAACTATTTCTTCTCTTTTCTGGGG-TATTTTTCAAGTGTACTAAAAAA-CCCTTTGGT-AGTAA-GAAATC-AAATGCTAGAGAATTCATTT-CTAATAAATACTCT-GACTAAGA-AATTAGATACCATA-GCCCCAGTT--ATTTCTCTTATTGG-ATCATTGTCGAAAGCTCA-ATTTTGTACTGTATTGGGT-CATCCTATTAGTAAACC-AATCTGGACCGATTTATCGGATTCTGA-------------TATTCTTGATCGATTTTGTCGGAT-----ATCTAGAAAT---CTTTGTCGT-TATCACAGCGGATCCTC-AAAGAA--------ACAGGTTTTATATCGTATAAAGTA-TATACTTCGACTTTCGTGTGCTAG-AACTTTGGCTCGTAAACATAAAAGTACAGTACGCACTCTTATGCGAAGATTAGGTTCGGGATTCTTAG-AAGAATTTTTTTT------GGAAGAAGAAC-AATCTCTTTCTT

Ania_hongkongensis_SG1343 ACAAGAATTCTTTTTCTTCTCATTTTTCT------------TCTCAAATGGTATCAGAAGGTTTTGGAGTCATTCTGGAAATTCCATTCTCGTCGCGATTAGTATCT---------TCCCTTGAAG---AAAAAAGAATACCAAAATCTCAGAATTTACGATCTATTCATTCAATATTTCCCTTTTTAGAGGATAAATTATCACATTTAAATTATGTGTCAG-ATC-TACT-AATACCCCATCCCATCCATC-TGGAAATCTT-GGTTCAAATCCTTCAATGCTGG-ATCAAAGATGTTCCTTCT-TTGCATTTATTG-CGATTGTT--TTTCCACG------AATATCATAATT---------------TGAATAGTCTCAT---TACT-TC---------------AAAGAAATCCATTTACGTCT-TTTCAAAAAG---AAAGAAAAGAT-TCT-TTTGG-TTCCTACATA------ATTCTTAT-GTATAT-GAATGC----GAATATCTAT-TCCTG---TTTCTTCGT--AAACAG-TCTTCT-TATT-TACG-AT-CAATAT-CTTCTGGA-GTCTTTCTT-GAGCG-AACACATTT--CTATGGAAA--AATAGAA------TATCTTATAGT---CGTGTGTT-GTAATT------CTTTTCA---------GAGG-ATCCTATGGT-TCCTTAA-AGATACT-TTCA-TACATTAT------------GTTC-----GATATCAAGGAAAA---GCG-----ATTAT--GGCTTC-----AAAAG---GAACTCTT---------ATTC------------TGATGAAGAAAT-GGAAATTT--CATCTT-GTAAA-TTTTTGGCA-ATC--TTATT-TTCACTTT-TGGTTTC--AACCTTATAGGA----TCCATATAAA-GCAATT--ACCCAACTATTTCTTCTCTTTTCTGGGG-TATTTTTCAAGTGTACTAAAAAA-CCCTTTGGT-AGTAA-GAAATC-AAATGCTAGAGAATTCATTT-CTAATAAATACTCT-GACTAAGA-AATTAGATACCATA-GCCCCAGTT--ATTTCTCTTATTGG-ATCATTGTCGAAAGCTCA-ATTTTGTACTGTATTGGGT-CATCCTATTAGTAAACC-AATCTGGACCGATTTATCGGATTCTGA-------------TATTCTTGATCGATTTTGTCGGAT-----ATCTAGAAAT---CTTTGTCGT-TATCACAGCGGATCCTC-AAAGAA--------ACAGGTTTTATATCGTATAAAGTA-TATACTTCGACTTTCGTGTGCTAG-AACTTTGGCTCGTAAACATAAAAGTACAGTACGCACTCTTATGCGAAGATTAGGTTCGGGATTCTTAG-AAGAATTTTTTTT------GGAAGAAGAAC-AATCTCTTTCTT

Ania_ruybarrettoi_KFBG43 ACAAGAATTCTTTTTCTTCTCATTTTTCT------------TCTCAAATGGTATCAGAAGGTTTTGGAGTCATTCTGGAAATTCCATTCTCGTCGCGATTAGTATCT---------TCCCTTGAAG---AAAAAAGAATACCAAAATCTCAGAATTTACGATCTATTCATTCAATATTTCCCTTTTTAGAGGATAAATTATCACATTTAAATTATGTGTCAG-ATC-TACT-AATACCCCATCCCATCCATC-TGGAAATCTT-GGTTCAAATCCTTCAATGCTGG-ATCAAAGATGTTCCTTCT-TTGCATTTATTG-CGATTGTT--TTTCCACG------AATATCATAATT---------------TGAATAGTCTCAT---TACT-TC---------------AAAGAAATCCATTTACGTCT-TTTCAAAAAG---AAAGAAAAGAT-TCT-TTTGG-TTCCTACATA------ATTCTTAT-GTATAT-GAATGC----GAATATCTAT-TCCTG---TTTCTTCGT--AAACAG-TCTTCT-TATT-TACG-AT-CAATAT-CTTCTGGA-GTCTTTCTT-GAGCG-AACACATTT--CTATGGAAA--AATAGAA------TATCTTATAGT---CGTGTGTT-GTAATT------CTTTTCA---------GAGG-ATCCTATGGT-TCCTCAA-AGATACT-TTCA-TACATTAT------------GTTC-----GATATCAAGGAAAA---GCG-----ATTAT--GGCTTC-----AAAAG---GAACTCTT---------ATTC------------TGATGAAGAAAT-GGAAATTT--CATCTT-GTAAA-TTTTTGGCA-ATC--TTATT-TTCACTTT-TGGTTTC--AACCTTATAGGA----TCCATATAAA-GCAATT--ACCCAACTATTTCTTCTCTTTTCTGGGG-TATTTTTCAAGTGTACTAAAAAA-CCCTTTGGT-AGTAA-GAAATC-AAATGCTAGAGAATTCATTT-CTAATAAATACTCT-GACTAAGA-AATTAGATACCATA-GCCCCAGTT--ATTTCTCTTATTGG-ATCATTGTCGAAAGCTCA-ATTTTGTACTGTATTGGGT-CATCCTATTAGTAAACC-AATCTGGACCGATTTATCGGATTCTGA-------------TATTCTTGATCGATTTTGTCGGAT-----ATCTAGAAAT---CTTTGTCGT-TATCACAGCGGATCCTC-AAAGAA--------ACAGGTTTTGTATCGTATAAAGTA-TATACTTCGACTTTCGTGTGCTAG-AACTTTGGCTCGTAAACATAAAAGTACAGTACGCACTTTTATGCGAAGATTAGGTTCGGGATTCTTAG-AAGAATTTTTTTT------GGAAGAAGAAC-AATCTCTTTCTT

Ania_ruybarrettoi_KY966707 ACAAGAATTCTTTTTCTTCTCATTTTTCT------------TCTCAAATGGTATCAGAAGGTTTTGGAGTCATTCTGGAAATTCCATTCTCGTCGCGATTAGTATCT---------TCCCTTGAAG---AAAAAAGAATACCAAAATCTCAGAATTTACGATCTATTCATTCAATATTTCCCTTTTTAGAGGATAAATTATCACATTTAAATTATGTGTCAG-ATC-TACT-AATACCCCATCCCATCCATC-TGGAAATCTT-GGTTCAAATCCTTCAATGCTGG-ATCAAAGATGTTCCTTCT-TTGCATTTATTG-CGATTGTT--TTTCCACG------AATATCATAATT---------------TGAATAGTCTCAT---TACT-TC---------------AAAGAAATCCATTTACGTCT-TTTCAAAAAG---AAAGAAAAGAT-TCT-TTTGG-TTCCTACATA------ATTCTTAT-GTATAT-GAATGC----GAATATCTAT-TCCTG---TTTCTTCGT--AAACAG-TCTTCT-TATT-TACG-AT-CAATAT-CTTCTGGA-GTCTTTCTT-GAGCG-AACACATTT--CTATGGAAA--AATAGAA------TATCTTATAGT---CGTGTGTT-GTAATT------CTTTTCA---------GAGG-ATCCTATGGT-TCCTCAA-AGATACT-TTCA-TACATTAT------------GTTC-----GATATCAAGGAAAA---GCG-----ATTAT--GGCTTC-----AAAAG---GAACTCTT---------ATTC------------TGATGAAGAAAT-GGAAATTT--CATCTT-GTAAA-TTTTTGGCA-ATC--TTATT-TTCACTTT-TGGTTTC--AACCTTATAGGA----TCCATATAAA-GCAATT--ACCCAACTATTTCTTCTCTTTTCTGGGG-TATTTTTCAAGTGTACTAAAAAA-CCCTTTGGT-AGTAA-GAAATC-AAATGCTAGAGAATTCATTT-CTAATAAATACTCT-GACTAAGA-AATTAGATACCATA-GCCCCAGTT--ATTTCTCTTATTGG-ATCATTGTCGAAAGCTCA-ATTTTGTACTGTATTGGGT-CATCCTATTAGTAAACC-AATCTGGACCGATTTATCGGATTCTGA-------------TATTCTTGATCGATTTTGTCGGAT-----ATCTAGAAAT---CTTTGTCGT-TATCACAGCGGATCCTC-AAAGAA--------ACAGGTTTTGTATCGTATAAAGTA-TATACTTCGACTTTCGTGTGCTAG-AACTTTGGCTCGTAAACATAAAAGTACAGTACGCACTTTTATGCGAAGATTAGGTTCGGGATTCTTAG-AAGAATTTTT---------------------------------

Ania_ruybarrettoi_SG1395 ACAAGAATTCTTTTTCTTCTCATTTTTCT------------TCTCAAATGGTATCAGAAGGTTTTGGAGTCATTCTGGAAATTCCATTCTCGTCGCGATTAGTATCT---------TCCCTTGAAG---AAAAAAGAATACCAAAATCTCAGAATTTACGATCTATTCATTCAATATTTCCCTTTTTAGAGGATAAATTATCACATTTAAATTATGTGTCAG-ATC-TACT-AATACCCCATCCCATCCATC-TGGAAATCTT-GGTTCAAATCCTTCAATGCTGG-ATCAAAGATGTTCCTTCT-TTGCATTTATTG-CGATTGTT--TTTCCACG------AATATCATAATT---------------TGAATAGTCTCAT---TACT-TC---------------AAAGAAATCCATTTACGTCT-TTTCAAAAAG---AAAGAAAAGAT-TCT-TTTGG-TTCCTACATA------ATTCTTAT-GTATAT-GAATGC----GAATATCTAT-TCCTG---TTTCTTCGT--AAACAG-TCTTCT-TATT-TACG-AT-CAATAT-CTTCTGGA-GTCTTTCTT-GAGCG-AACACATTT--CTATGGAAA--AATAGAA------TATCTTATAGT---CGTGTGTT-GTAATT------CTTTTCA---------GAGG-ATCCTATGGT-TCCTCAA-AGATACT-TTCA-TACATTAT------------GTTC-----GATATCAAGGAAAA---GCG-----ATTAT--GGCTTC-----AAAAG---GAACTCTT---------ATTC------------TGATGAAGAAAT-GGAAATTT--CATCTT-GTAAA-TTTTTGGCA-ATC--TTATT-TTCACTTT-TGGTTTC--AACCTTATAGGA----TCCATATAAA-GCAATT--ACCCAACTATTTCTTCTCTTTTCTGGGG-TATTTTTCAAGTGTACTAAAAAA-CCCTTTGGT-AGTAA-GAAATC-AAATGCTAGAGAATTCATTT-CTAATAAATACTCT-GACTAAGA-AATTAGATACCATA-GCCCCAGTT--ATTTCTCTTATTGG-ATCATTGTCGAAAGCTCA-ATTTTGTACTGTATTGGGT-CATCCTATTAGTAAACC-AATCTGGACCGATTTATCGGATTCTGA-------------TATTCTTGATCGATTTTGTCGGAT-----ATCTAGAAAT---CTTTGTCGT-TATCACAGCGGATCCTC-AAAGAA--------ACAGGTTTTGTATCGTATAAAGTA-TATACTTCGACTTTCGTGTGCTAG-AACTTTGGCTCGTAAACATAAAAGTACAGTACGCACTTTTATGCGAAGATTAGGTTCGGGATTCTTAG-AAGAATTTTTTTT------GGAAGAAGAAC-AATCTCTTTCTT

Anoectochilus_formosanus_EU797513 ATAAGAATTATTTTTCTTCTCATTTTTCT------------TTTCAAATACTATCAGAAGGTTTTGGAGTCGTTCTGGAAATTCCATTATCGTCGCGATTAGTATTC---------TCCCTTGAAG---AAAAAAAAATACCAAAATATCAGAATTTACGATCTATTCATTCAATATTTCCTTTTTTAGAGGATAAATTTTCACATTTAAATTCTGTGTCAG-ATC-TATT-AATACCCCATCCCATCCATC-TGGAAATCTT-GGTTCAAATCCTTCAATGCTGG-ATCAAAGATGTTCCTTCT-TTGCATTTGTTG-CGATTTAT--TTTCCACG------AATATCATAATT---------------TGAAGAGTATCAT---TACT-TC---------------AAATAAATCCATTCACGTTT-TTTCAAAAAA---AAAGAAAAGAA-TTT-TTTGG-TTCCTACATA------ATTTTTAT-GTATAT-GAATGC----GAATATCTCT-TTCTT---TTTCTTCGT--AAAAAT-TCTTCT-TATT-TACG-AT-CAACAT-CTTTTGGA-GTCTTTATT-GAGCG-AACACTTTT--TTATGTAAA--AATGGAA------TCTATTCTAGT---AGTATATT-TTAATT------CTTTTCA---------GAGG-ATTCTCTGGT-TCCTCAA-AGATCCT-TTCA-TACATTAT------------GTTC-----GATATCAAGGAAAA---GTA-----ATTCT--GACTTC-----AAAGG---TAACTCTT---------ATTC------------TGATGAAGAAAT-GGAATTTT--CATGTT-GTGAA-TTTTTGTCA-ATT--TTATT-TTCACTTT-TGGTCTC--AACTTTATAGGA----TCCATATAAA-GCAATT--ACCCAACTATTCCTTCTCTTTTCTGGGG-TATTTTTTAAGTGTACAAAAAAA-AACTTTGGT-AGTAA-GAAATC-AAATGCTAGAGAATTCCTTT-CTAATAAATACTAT-GACTAAGA-AATTAGATACCGTA-GCCCCAGTT--ATTTCTCTTATTGG-ATCATTGTCGAAAGCTCA-ATTTTGTACTATATCAGGT-CATCCTATTAGTAAACC-CATTTGGACTGATTTTTCGGATTCTGA-------------TATTATTGATCGATTTTGTCGGAA-----ATGTAGAAAT---CTTTGTCGT-TATCACAGCGGATCCTC-AAAAAA--------AAAAGTTTTGTATCGTATAAAATA-TATATTTCGACTTTCGTGTGCTAG-AACTTTGGCTCGTAAACATAAAAGTACAGTACGCACTTTTATGCGAAGATTGGGTTCGGTATTTTTAG-AAGAATTTTTTAT------GGAAGAAGAAC-AAGTTCTTTCTT

Anoectochilus_formosanus_MG490281 ATAAGAATTATTTTTCTTCTCATTTTTCT------------TTTCAAATACTATCAGAAGGTTTTGGAGTCGTTCTGGAAATTCCATTATCGTCGCGATTAGTATTC---------TCCCTTGAAG---AAAAAAAAATACCAAAATATCAGAATTTACGATCTATTCATTCAATATTTCCTTTTTTAGAGGATAAAATTTCACATTTAAATTCTGTGTCAG-ATC-TATT-AATACCCCATCCCATCCATC-TGGAAATCTT-GGTTCAAATCCTTCAATGCTGG-ATCAAAGATGTTCCTTCT-TTGCATTTGTTG-CGATTTAT--TTTCCACG------AATATCATAATT---------------TGAAGAGTATCAT---TACT-TC---------------AAATAAATCCATTCACGTTT-TTTCAAAAAA---AAAGAAAAGAA-TTT-TTTGG-TTCCTACATA------ATTTTTAT-GTATAT-GAATGC----GAATATCTCT-TTCTT---TTTCTTCGT--AAAAAT-TCTTCT-TATT-TACG-AT-CAACAT-CTTTTGGA-GTCTTTATT-GAGCG-AACACTTTT--TTATGTAAA--AATGGAA------TCTATTCTAGT---AGTATATT-TTAATT------CTTTTCA---------GAGG-ATTCTCTGGT-TCCTCAA-AGATCCT-TTCA-TACATTAT------------GTTC-----GATATCAAGGAAAA---GTA-----ATTCT--GACTTC-----AAAGG---TAACTCTT---------ATTC------------TGATGAAGAAAT-GGAATTTT--CATGTT-GTGAA-TTTTTGTCA-ATT--TTATT-TTCACTTT-TGGTCTC--AACCTTATAGGA----TCCATATAAA-GCAATT--ACCCAACTATTCCTTCTCTTTTCTGGGG-TATTTTTTAAGTGTACAAAAAAA-AACTTTGGT-AGTAA-GAAATC-AAATGCTAGAGAATTCCTTT-CTAATAAATACTAT-GACTAAGA-AATTAGATACCATA-GCCCCAGTT--ATTTCTCTTATTGG-ATCATTGTCGAAAGCTCA-ATTTTGTACTATATCAGGT-CATCCTATTAGTAAACC-CATTTGGACTGATTTTTCGGATTCTGA-------------TATTATTGATCAATTTTGTCGGAA-----ATGTAGAAAT---CTTTGTCGT-TATCACAGCGGATCCTC-AAAAAA--------AAAAG-------------------------------------------------------------------------------------------------------------------------------------------------------------

Anoectochilus_formosanus_PK12215 ATAAGAATTATTTTTCTTCTCATTTTTCT------------TTTCAAATACTATCAGAAGGTTTTGGAGTCGTTCTGGAAATTCCATTATCGTCGCGATTAGTATTC---------TCCCTTGAAG---AAAAAAAAATACCAAAATATCAGAATTTACGATCTATTCATTCAATATTTCCTTTTTTAGAGGATAAATTTTCACATTTAAATTCTGTGTCAG-ATC-TATT-AATACCCCATCCCATCCATC-TGGAAATCTT-GGTTCAAATCCTTCAATGCTGG-ATCAAAGATGTTCCTTCT-TTGCATTTGTTG-CGATTTAT--TTTCCACG------AATATCATAATT---------------TGAAGAGTATCAT---TACT-TC---------------AAATAAATCCATTCACGTTT-TTTCAAAAAA---AAAGAAAAGAA-TTT-TTTGG-TTCCTACATA------ATTTTTAT-GTATAT-GAATGC----GAATATCTCT-TTCTT---TTTCTTCGT--AAAAAT-TCTTCT-TATT-TACG-AT-CAACAT-CTTTTGGA-GTCTTTATT-GAGCG-AACACTTTT--TTATGTAAA--AATGGAA------TCTATTCTAGT---AGTATATT-TTAATT------CTTTTCA---------GAGG-ATTCTCTGGT-TCCTCAA-AGATCCT-TTCA-TACATTAT------------GTTC-----GATATCAAGGAAAA---GTA-----ATTCT--GACTTC-----AAAGG---TAACTCTT---------ATTC------------TGATGAAGAAAT-GGAATTTT--CATGTT-GTGAA-TTTTTGTCA-ATT--TTATT-TTCACTTT-TGGTCTC--AACCTTATAGGA----TCCATATAAA-GCAATT--ACCCAACTATTCCTTCTCTTTTCTGGGGGTATTTTTTAAGTGTACAAAAAAA-AACTTTGGT-AGTAA-GAAATC-AAATGCTAGAGAATTCCTTT-CTAATAAATACTAT-GACTAAGA-AATTAGATACCGTA-GCCCCAGTT--ATTTCTCTTATTGG-ATCATTGTCGAAAGCTCA-ATTTTGTACTATATCAGGT-CATCCTATTAGTAAACC-CATTTGGACTGATTTTTCGGATTTTGA-------------TATTATTGATCGATTTTGTCGGAA-----AAGTAGAAAT---CTTTGTCGT-TATCCCAGCGGATCCTC-AAAAAA--------AAAAGTTTTGTATCGTATAAAATA-TATATTTCGACTTTCGTGTGCTAG-AACTTTGGCTCGTAAACATAAAAGTACAGTACGCACTTTTATGCGAAGATTGGGTTCGGTATTTTTAG-AAGAATTTTTTAT------GGAAGAAGAAC-AAGTTCTTTCTT

Anoectochilus_roxburghii_EU817409 ATAAGAATTATTTTTCTTCTCATTTTTCT------------TTTCAAATACTATCAGAAGGTTTTGGAGTCGTTCTGGAAATTCCATTATCGTCGCGATTAGTATTC---------TCCCTTGAAG---AAAAAAAAATACCAAAATATCAGAATTTACGATCTATTCATTCAATATTTCCTTTTTTAGAGGATAAATTTTCACATTTAAATTCTGTGTCAG-ATC-TATT-AATACCCCATCCCATCCATC-TGGAAATCTT-GGTTCAAATCCTTCAATGCTGG-ATCAAAGATGTTCCTTCT-TTGCATTTGTTG-CGATTTAT--TTTCCACG------AATATCATAATT---------------TGAAGAGTATCAT---TACT-TC---------------AAAGAAATCCATTCACGTTT-TTTCAAAAAA---AAAGAAAAGAT-TTT-TTTGG-TTCCTACATA------ATTTTTAT-GTATAT-GAATGC----GAATATCTCT-TTCTT---TTTCTTCGT--AAAAAT-TCTTCT-TATT-TACG-AT-CAACAT-CTTTTGGA-GTCTTTATT-GAGCG-AACACTTTT--TTATGTAAA--AATGGAA------TCTATTCTAGT---AGTATATT-TTAATT------CTTTTCA---------GAGG-ATTCTCCGGT--CCTCAA-AGATCCT-TTCA-TACATTAT------------GTTC-----GATATCAAGGAAAG---GTA-----ATTCT--GACTTC-----AAAGG---GAACTCTT---------ATTC------------TGATGAAGAAAT-GGAATTTT--CATGTT-GTGAA-TTTTTGTCA-ATT--TTATT-TTCACTTT-TGGTCTC--AACCTTATAGGA----TCCATATAAA-GCAATT--ACCCAACTATTCCTTCTCTTTTCTGGGG-TATTTTTTAAGTGTACAAAAAAA-AACTTTGGT-AGTAA-GAAATC-AAATGCTAGAGAATTCCTTT-CTAATAAATACTAT-GACTAAGA-AATTAGATACCATA-GCCCCAGTT--ATTTCTCTTATTGG-ATCATTGTCGAAAGCTCA-ATTTTGTACTATATCGGGT-CATCCTATTAGTAAACC-CATTTGGACTGATTTTTCGGATTCTGA-------------TATTATTGATCTATTTTGTCGGAA-----ATGTAAAAAT---CTTTGTCGT-TATCACAGCGGATCCTC-AAAAAA--------AAAAGTTTTGTATCGTATAAAATA-TATATTTCGACTTTCGTGTGCTAG-AACTTTAGCTCGTAAACATAAAAGTACAGTACGCACTTTTATGCGAAGATTGGGTTCGGTATTTTTAG-AAGAATTTTTTAT------GGAAGAAGAAC-AAGTTCTTTCTT

Anoectochilus_roxburghii_KF361656 -----------------------------------------------------------------------------------------------------------------------------------------------------------------------------------------------------------------AATTCTGTGTCAG-ATC-TATT-AATACCCCATCCCATCCATC-TGGAAATCTT-GGTTCAAATCCTTCAATGCTGG-ATCAAAGATGTTCCTTCT-TTGCATTTGTTG-CGATTTAT--TTTCCACG------AATATCATAATT---------------TGAAGAGTATCAT---TACT-TC---------------AAATAAATCCATTCACGTTT-TTTCAAAAAA---AAAGAAAAGAA-TTT-TTTGG-TTCCTACATA------ATTTTTAT-GTATAT-GAATGC----GAATATCTCT-TTCTT---TTTCTTCGT--AAAAAT-TCTTCT-TATT-TACG-AT-CAACAT-CTTTTGGA-GTCTTTATT-GAGCG-AACACTTTT--TTATGTAAA--AATGGAA------TCTATTCTAGT---AGTATATT-TTAATT------CTTTTCA---------GAGG-ATTCTCTGGT-TCCTCAA-AGATCCT-TTCA-TACATTAT------------GTTC-----GATATCAAGGAAAA---GTA-----ATTCT--GACTTC-----AAAGG---TAACTCTT---------ATTC------------TGATGAAGAAAT-GGAATTTT--CATGTT-GTGAA-TTTTTGTCA-ATT--TTATT-TTCACTTT-TGGTCTC--AACCTTATAGGA----TCCATATAAA-GCAATT--ACCCAACTATTCCTTCTCTTTTCTGGGG-TATTTTTTAAGTGTACAAAAAAA-AACTTTGGT-AGTAA-GAAATC-AAATGCTAGAGAATTCCTTT-CTAATAAATACTAT-GACTAAGA-AATTAGATACCATA-GCCCCAGTT--ATTTCTCTTATTGG-ATCATTGTCGAAAGCTCA-ATTTTGTACTATATCAGGT-CATCCTATTAGTAAACC-CATTTGGACTGATTTTTCGGATTCTGA-------------TATTATTGATCAATTTTGTCGGAA-----ATGTAGAAAT---CTTTGTCGT-TATCACAGCGGATCCTC-AAAAAA--------AAAAGTTTTGTAT-----------------------------------------------------------------------------------------------------------------------------------------------------

Anoectochilus_roxburghii_KY966708 ATAAGAATTATTTTTCTTCTCATTTTTCT------------TTTCAGATACTATCAGAAGGTTTTGGAGTCGTTCTGGAAATTCCATTATCGTCGCGATTAGTATTC---------TCCCTTGAAG---AAAAAAAAATACCAAAATATCAGAATTTACGATCTATTCATTCAATATTTCCTTTTTTAGAGGATAAAATTTCACATTTAAATTCTGTGTCAG-ATC-TATT-AATACCCCATCCCATCCATC-TGGAAATCTT-GGTTCAAATCTTTCAATGCTGG-ATCAAAGATGTTCCTTCT-TTGCATTTGTTG-CGATTTAT--TTTCCACG------AATATCATAATT---------------TGAAGAGTATCAT---TACT-TC---------------AAATAAATCCATTCACGTTT-TTTCAAAAAA---AAAGAAAAGAA-TTT-TTTGG-TTCCTACATA------ATTTTTAT-GTATAT-GAATGC----GAATATCTCT-TTCTT---TTTCTTCGT--AAAAAT-TCTTCT-TATT-TACG-AT-CAACAT-CTTTTGGA-GTCTTTATT-GAGCG-AACACTTTT--TTATGTAAA--AATGGAA------TCTATTCTAGT---AGTATATT-TTAATT------CTTTTCA---------GAGG-ATTCTCTGGT-TCCTCAA-AGATCCT-TTCA-TACATTAT------------GTTC-----GATATCAAGGAAAA---GTA-----ATTCT--GACTTC-----AAAGG---TAACTCTT---------ATTC------------TGATGAAGAAAT-GGAATTTT--CATGTT-GTGAA-TTTTTGTCA-ATT--TTATT-TTCACTTT-TGGTCTC--AACCTTATAGGA----TCCATATAAA-GCAATT--ACCCAACTATTCCTTCTCTTTTCTGGGG-TATTTTTTAAGTGTACAAAAAAA-AACTTTGGT-AGTAA-GAAATC-AAATGCTAGAGAATTCCTTT-CTAATAAATACTAT-GACTAAGA-AATTAGATACCATA-GCCCCAGTT--ATTTCTCTTATTGG-ATCATTGTCGAAAGCTCA-ATTTTGTACTATATCAGGT-CATCCTATTAGTAAACC-CATTTGGACTGATTTTTCGGATTCTGA-------------TATTATTGATCAATTTTGTCGGAA-----ATGTAGAAAT---CTTTGTCGT-TATCACAGCGGATCCTC-AAAAAA--------AAAAGTTTTGTATCGTATAAAATA-TATATTTCGACTTTCGTGTGCTAG-AACTTTGGCTCGTAAACATAAAAGTACAGTACGCACTTTTATGCGAAGATTGGGTTCGGTATTTTTAG-AAGAATTTTT---------------------------------

Anoectochilus_roxburghii_PK12043 ATAAGAATTATTTTTCTTCTCATTTTTCT------------TTTCAAATACTATCAGAAGGTTTTGGAGTCGTTCTGGAAATTCCATTATCGTCGCGATTAGTATTC---------TCCCTTGAAG---AAAAAAAAATACCAAAATATCAGAATTTACGATCTATTCATTCAATATTTCCTTTTTTAGAGGATAAAATTTCACATTTAAATTCTGTGTCAG-ATC-TATT-AATACCCCATCCCATCCATC-TGGAAATCTT-GGTTCAAATCCTTCAATGCTGG-ATCAAAGATGTTCCTTCT-TTGCATTTGTTG-CGATTTAT--TTTCCACG------AATATCATAATT---------------TGAAGAGTATCAT---TACT-TC---------------AAATAAATCCATTCACGTTT-TTTCAAAAAA---AAAGAAAAGAA-TTT-TTTGG-TTCCTACATA------ATTTTTAT-GTATAT-GAATGC----GAATATCTCT-TTCTT---TTTCTTCGT--AAAAAT-TCTTCT-TATT-TACG-AT-CAACAT-CTTTTGGA-GTCTTTATT-GAGCG-AACACTTTT--TTATGTAAA--AATGGAA------TCTATTCTAGT---AGTATATT-TTAATT------CTTTTCA---------GAGG-ATTCTCTGGT-TCCTCAA-AGATCCT-TTCA-TACATTAT------------GTTC-----GATATCAAGGAAAA---GTA-----ATTCT--GACTTC-----AAAGG---TAACTCTT---------ATTC------------TGATGAAGAAAT-GGAATTTT--CATGTT-GTGAA-TTTTTGTCA-ATT--TTATT-TTCACTTT-TGGTCTC--AACCTTATAGGA----TCCATATAAA-GCAATT--ACCCAACTATTCCTTCTCTTTTCTGGGG-TATTTTTTAAGTGTACAAAAAAA-AACTTTGGT-AGTAA-GAAATC-AAATGCTAGAGAATTCCTTT-CTAATAAATACTAT-GACTAAGA-AATTAGATACCATA-GCCCCAGTT--ATTTCTCTTATTGG-ATCATTGTCGAAAGCTCA-ATTTTGTACTATATCAGGT-CATCCTATTAGTAAACC-CATTTGGACTGATTTTTCGGATTCTGA-------------TATTATTGATCAATTTTGTCGGAA-----ATGTAGAAAT---CTTTGTCGT-TATCACAGCGGATCCTC-AAAAAA--------AAAAGTTTTGTATCGTATAAAATA-TATATTTCGACTTTCGTGTGCTAG-AACTTTGGCTCGTAAACATAAAAGTACAGTACGCACTTTTATGCGAAGATTGGGTTCGGTATTTTTAG-AAGAATTTTTTAT------GGAAGAAGAAC-AAGTTCTTTCTT

Anoectochilus_roxburghii_PK12068 ATAAGAATTATTTTTCTTCTCATTTTTCT------------TTTCAAATACTATCAGAAGGTTTTGGAGTCGTTCTGGAAATTCCATTATCGTCGCGATTAGTATTC---------TCCCTTGAAG---AAAAAAAAATACCAAAATATCAGAATTTACGATCTATTCATTCAATATTTCCTTTTTTAGAGGATAAATTTTCACATTTAAATTCTGTGTCAG-ATC-TATT-AATACCCCATCCCATCCATC-TGGAAATCTT-GGTYCAAATCCTTCAATGCTGG-ATCAAAGATGTTCCTTCT-TTGCATTTGTTG-CGATTTAT--TTTCCACG------AATATCATAATT---------------TGAAGAGTATCAT---TACT-TC---------------AAATAAATCCATTCACGTTT-TTTCAAAAAA---AAAGAAAAGAA-TTT-TTTGG-TTCCTACATA------ATTTTTAT-GTATAT-GAATGC----GAATATCTCT-TTCTT---TTTCTTCGT--AAAAAT-TCTTCT-TATT-TACG-AT-CAACAT-CTTTTGGA-GTCTTTATT-GAGCG-AACACTTTT--TTATGTAAA--AATGGAA------TCTATTCTAGT---AGTATATT-TTAATT------CTTTTCA---------GAGG-ATTCTCTGGT-TCCTCAA-AGATCCT-TTCA-TACATTAT------------GTTC-----GATATCAAGGAAAA---GTA-----ATTCT--GACTTC-----AAAGG---TAACTCTT---------ATTC------------TGATGAAGAAAT-GGAATTTT--CATGTT-GTGAA-TTTTTGTCA-ATT--TTATT-TTCACTTT-TGGTCTC--AACCTTATAGGA----TCCATATAAA-GCAATT--ACCCAACTATTCCTTCTCTTTTCTGGGG-TATTTTTTAAGTGTACAAAAAAA-AACTTTGGT-AGTAA-GAAATC-AAATGCTAGAGAATTCCTTT-CTAATAAATACTAT-GACTAAGA-AATTAGATACCATA-GCCCCAGTT--ATTTCTCTTATTGG-ATCATTGTCGAAAGCTCA-ATTTTGTACTATATCAGGT-CATCCTATTAGTAAACC-CATTTGGACTGATTTTTCGGATTCTGA-------------TATTATTGATCAATTTTGTCGGAA-----ATGTAGAAAT---CTTTGTCGT-TATCACAGCGGATCCTC--AAAAA--------AAAAGTTTTGTATCGTATAAAATA-TATATTTCGACTTTCGTGTGCTAG-AACTTTGGCTCGTAAACATAAAAGTACAGTACGCACTTTTATGCGAAGATTGGGTTCGGTATTTTTAG-AAGAATTTTTTAT------GGAAGAAGAAC-AAGTTCTTTCTT

Anoectochilus_roxburghii_PK12069 ATAAGAATTATTTTTCTTCTCATTTTTCT------------TTTCAAATACTATCAGAAGGTTTTGGAGTCGTTCTGGAAATTCCATTATCGTCGCGATTAGTATTC---------TCCCTTGAAG---AAAAAAAAATACCAAAATATCAGAATTTACGATCTATTCATTCAATATTTCCTTTTTTAGAGGATAAAATTTCACATTTAAATTCTGTGTCAG-ATC-TATT-AATACCCCATCCCATCCATC-TGGAAATCTT-GGTTCAAATCCTTCAATGCTGG-ATCAAAGATGTTCCTTCT-TTGCATTTGTTG-CGATTTAT--TTTCCACG------AATATCATAATT---------------TGAAGAGTATCAT---TACT-TC---------------AAATAAATCCATTCACGTTT-TTTCAAAAAA---AAAGAAAAGAA-TTT-TTTGG-TTCCTACATA------ATTTTTAT-GTATAT-GAATGC----GAATATCTCT-TTCTT---TTTCTTCGT--AAAAAT-TCTTCT-TATT-TACG-AT-CAACAT-CTTTTGGA-GTCTTTATT-GAGCG-AACACTTTT--TTATGTAAA--AATGGAA------TCTATTCTAGT---AGTATATT-TTAATT------CTTTTCA---------GAGG-ATTCTCTGGT-TCCTCAA-AGATCCT-TTCA-TACATTAT------------GTTC-----GATATCAAGGAAAA---GTA-----ATTCT--GACTTC-----AAAGG---TAACTCTT---------ATTC------------TGATGAAGAAAT-GGAATTTT--CATGTT-GTGAA-TTTTTGTCA-ATT--TTATT-TTCACTTT-TGGTCTC--AACCTTATAGGA----TCCATATAAA-GCAATT--ACCCAACTATTCCTTCTCTTTTCTGGGG-TATTTTTTAAGTGTACAAAAAAA-AACTTTGGT-AGTAA-GAAATC-AAATGCTAGAGAATTCCTTT-CTAATAAATACTAT-GACTAAGA-AATTAGATACCATA-GCCCCAGTT--ATTTCTCTTATTGG-ATCATTGTCGAAAGCTCA-ATTTTGTACTATATCAGGT-CATCCTATTAGTAAACC-CATTTGGACTGATTTTTCGGATTCTGA-------------TATTATTGATCAATTTTGTCGGAA-----ATGTAGAAAT---CTTTGTCGT-TATCACAGCGGATCCTC-AAAAAA--------AAAAGTTTTGTATCGTATAAAATA-TATATTTCGACTTTCGTGTGCTAG-AACTTTGGCTCGTAAACATAAAAGTACAGTACGCACTTTTATGCGAAGATTGGGTTCGGTATTTTTAG-AAGAATTTTTTAT------GGAAGAAGAAC-AAGTTCTTTCTT

Anoectochilus_roxburghii_SG1219 ATAAGAATTATTTTTCTTCTCATTTTTCT------------TTTCAAATACTATCAGAAGGTTTTGGAGTCGTTCTGGAAATTCCATTATCGTCGCGATTAGTATTC---------TCCCTTGAAG---AAAAAAAAATACCAAAATATCAGAATTTACGATCTATTCATTCAATATTTCCTTTTTTAGAGGATAAAATTTCACATTTAAATTCTGTGTCAG-ATC-TATT-AATACCCCATCCCATCCATC-TGGAAATCTT-GGTTCAAATCCTTCAATGCTGG-ATCAAAGATGTTCCTTCT-TTGCATTTGTTG-CGATTTAT--TTTCCACG------AATATCATAATT---------------TGAAGAGTATCAT---TACT-TC---------------AAATAAATCCATTCACGTTT-TTTCAAAAAA---AAAGAAAAGAA-TTT-TTTGG-TTCCTACATA------ATTTTTAT-GTATAT-GAATGC----GAATATCTCT-TTCTT---TTTCTTCGT--AAAAAT-TCTTCT-TATT-TACG-AT-CAACAT-CTTTTGGA-GTCTTTATT-GAGCG-AACACTTTT--TTATGTAAA--AATGGAA------TCTATTCTAGT---AGTATATT-TTAATT------CTTTTCA---------GAGG-ATTCTCTGGT-TCCTCAA-AGATCCT-TTCA-TACATTAT------------GTTC-----GATATCAAGGAAAA---GTA-----ATTCT--GACTTC-----AAAGG---TAACTCTT---------ATTC------------TGATGAAGAAAT-GGAATTTT--CATGTT-GTGAA-TTTTTGTCA-ATT--TTATT-TTCACTTT-TGGTCTC--AACCTTATAGGA----TCCATATAAA-GCAATT--ACCCAACTATTCCTTCTCTTTTCTGGGG-TATTTTTTAAGTGTACAAAAAAA-AACTTTGGT-AGTAA-GAAATC-AAATGCTAGAGAATTCCTTT-CTAATAAATACTAT-GACTAAGA-AATTAGATACCATA-GCCCCAGTT--ATTTCTCTTATTGG-ATCATTGTCGAAAGCTCA-ATTTTGTACTATATCAGGT-CATCCTATTAGTAAACC-CATTTGGACTGATTTTTCGGATTCTGA-------------TATTATTGATCAATTTTGTCGGAA-----ATGTAGAAAT---CTTTGTCGT-TATCACAGCGGATCCTC-AAAAAA--------AAAAGTTTTGTATCGTATAAAATA-TATATTTCGACTTTCGTGTGCTAG-AACTTTGGCTCGTAAACATAAAAGTACAGTACGCACTTTTATGCGAAGATTGGGTTCGGTATTTTTAG-AAGAATTTTTTAT------GGAAGAAGAAC-AAGTTCTTTCTT

Aphyllorchis_montana_PK12147 ACAAGAATTCCTTTTCTTCTCATTTTTCT------------TCTCAAATGGTATCAGAAGGTTTTGGAATCATTCTGGAAATTCCATTCTCGTCGCGATTAGTATCT---------TCCCTTGAAG---AAAAAAAAAGACCAAAATCTCAGAATTTACGATCTATTCATTCAATATTTCCCTTTTTAGAGGATAAATTCTCGCATTTAAATTATGTGTCAG-ATC-TAAT-AATACCCCATCCCCTCCATC-TGGAAATCTT-GGTTCAAATCCTTCAATGCTGG-ATCAAAGATGTTCCTTCT-TTGCATTTATTG-CGATCTTT--TTTCCACG------AATATCATAATT---------------TGAATAGTCTCAT---TACT-TC---------------AAAGAAATCCATTCATGTCT-TTTCGAAAAG---AAAGAAAAGAT-TCT-TTTGG-TTCCTACATA------ATTCTTAT-GTATAT-GAATGC----GAATATATAT-TCCTT---TTTCTTCGT--AAAGAG-TCTTCT-TATT-TACG-AT-CAACAT-CTTCTGGA-GTCTTTCTT-GAGCG-AACACATTT--CTATGGAAA--AATAGAA------TATCTTCTAGT---AGTGTGTT-TTAATT------CTTTTCG---------GAGG-ATTCTATGGT-TCCTCAA-AGATCCT-TTCA-TACATTAT------------GCTC-----GATATCAAGGAAAA---GCA-----ATTAT--GGCTTC-----AAAGG---GAACTCTT---------ATTC------------TGATGAAGAAAT-GGAAATTT--CATCTT-GTGAA-TCTTTGGCA-ATC--TTATT-TTCACTTT-TGGTCTC--AACCTTATAGGA----TCCATATAAA-GCAATT--ACCCAACTCTTCCTTCTCTTTTCTGGGG-TATTTTTCAAGTGTACTAAAAAA-TACTTTGGT-AGTAA-GAAATC-AAATGCTAGAGAATTCATTT-CTAATAAATACTCT-GACTAAAA-AATTCGATACCATA-GCCCCAGTT--ATTTCTCTTATTGG-ATCATTGTCGAAAGCTCA-ATTTTGTACTGTATTGGGT-CATCCTATTAGTAAGCC-GATCTGGACCGATTTATCGGATTCTGA-------------TATTCTTGATCGATTTTGTCGGAT-----ATGTAGAAAT---CTTTGTCGT-TATCACAGCGGATCCTC-AAAGAA--------ACAGGTTTTGTATCGTATAAAGTA-TATACTTCGACTTTCGTGTGCTAG-AACTTTGGCTCGTAAACATAAAAGTACAGTACGCACTTTTATGCGAAGATTAGGTTCGGGATTCTTAG-AAGAATTTTTTTT------GGAAGAAGAAA-AAGTTCTTTCTT

Aphyllorchis_montana_PK12148 ACAAGAATTCCTTTTCTTCTCATTTTTCT------------TCTCAAATGGTATCAGAAGGTTTTGGAATCATTCTGGAAATTCCATTCTCGTCGCGATTAGTATCT---------TCCCTTGAAG---AAAAAAAAAGACCAAAATCTCAGAATTTACGATCTATTCATTCAATATTTCCCTTTTTAGAGGATAAATTCTCGCATTTAAATTATGTGTCAG-ATC-TAAT-AATACCCCATCCCCTCCATC-TGGAAATCTT-GGTTCAAATCCTTCAATGCTGG-ATCAAAGATGTTCCTTCT-TTGCATTTATTG-CGATCTTT--TTTCCACG------AATATCATAATT---------------TGAATAGTCTCAT---TACT-TC---------------AAAGAAATCCATTCATGTCT-TTTCGAAAAG---AAAGAAAAGAT-TCT-TTTGG-TTCCTACATA------ATTCTTAT-GTATAT-GAATGC----GAATATATAT-TCCTT---TTTCTTCGT--AAAGAG-TCTTCT-TATT-TACG-AT-CAACAT-CTTCTGGA-GTCTTTCTT-GAGCG-AACACATTT--CTATGGAAA--AATAGAA------TATCTTCTAGT---AGTGTGTT-TTAATT------CTTTTCG---------GAGG-ATTCTATGGT-TCCTCAA-AGATCCT-TTCA-TACATTAT------------GCTC-----GATATCAAGGAAAA---GCA-----ATTAT--GGCTTC-----AAAGG---GAACTCTT---------ATTC------------TGATGAAGAAAT-GGAAATTT--CATCTT-GTGAA-TCTTTGGCA-ATC--TTATT-TTCACTTT-TGGTCTC--AACCTTATAGGA----TCCATATAAA-GCAATT--ACCCAACTCTTCCTTCTCTTTTCTGGGG-TATTTTTCAAGTGTACTAAAAAA-TACTTTGGT-AGTAA-GAAATC-AAATGCTAGAGAATTCATTT-CTAATAAATACTCT-GACTAAAA-AATTCGATACCATA-GCCCCAGTT--ATTTCTCTTATTGG-ATCATTGTCGAAAGCTCA-ATTTTGTACTGTATTGGGT-CATCCTATTAGTAAGCC-GATCTGGACCGATTTATCGGATTCTGA-------------TATTCTTGATCGATTTTGTCGGAT-----ATGTAGAAAT---CTTTGTCGT-TATCACAGCGGATCCTC-AAAGAA--------ACAGGTTTTGTATCGTATAAAGTA-TATACTTCGACTTTCGTGTGCTAG-AACTTTGGCTCGTAAACATAAAAGTACAGTACGCACTTTTATGCGAAGATTAGGTTCGGGATTCTTAG-AAGAATTTTTTTT------GGAAGAAGAAA-AAGTTCTTTCTT

Aphyllorchis_montana_SG1010 ACAAGAATTCCTTTTCTTCTCATTTTTCT------------TCTCAAATGGTATCAGAAGGTTTTGGAATCATTCTGGAAATTCCATTCTCGTCGCGATTAGTATCT---------TCCCTTGAAG---AAAAAAAAAGACCAAAATCTCAGAATTTACGATCTATTCATTCAATATTTCCCTTTTTAGAGGATAAATTCTCGCATTTAAATTATGTGTCAG-ATC-TAAT-AATACCCCATCCCCTCCATC-TGGAAATCTT-GGTTCAAATCCTTCAATGCTGG-ATCAAAGATGTTCCTTCT-TTGCATTTATTG-CGATCTTT--TTTCCACG------AATATCATAATT---------------TGAATAGTCTCAT---TACT-TC---------------AAAGAAATCCATTCATGTCT-TTTCGAAAAG---AAAGAAAAGAT-TCT-TTTGG-TTCCTACATA------ATTCTTAT-GTATAT-GAATGC----GAATATATAT-TCCTT---TTTCTTCGT--AAAGAG-TCTTCT-TATT-TACG-AT-CAACAT-CTTCTGGA-GTCTTTCTT-GAGCG-AACACATTT--CTATGGAAA--AATAGAA------TATCTTCTAGT---AGTGTGTT-TTAATT------CTTTTCG---------GAGG-ATTCTATGGT-TCCTCAA-AGATCCT-TTCA-TACATTAT------------GCTC-----GATATCAAGGAAAA---GCA-----ATTAT--GGCTTC-----AAAGG---GAACTCTT---------ATTC------------TGATGAAGAAAT-GGAAATTT--CATCTT-GTGAA-TCTTTGGCA-ATC--TTATT-TTCACTTT-TGGTCTC--AACCTTATAGGA----TCCATATAAA-GCAATT--ACCCAACTCTTCCTTCTCTTTTCTGGGG-TATTTTTCAAGTGTACTAAAAAA-TACTTTGGT-AGTAA-GAAATC-AAATGCTAGAGAATTCATTT-CTAATAAATACTCT-GACTAAAA-AATTCGATACCATA-GCCCCAGTT--ATTTCTCTTATTGG-ATCATTGTCGAAAGCTCA-ATTTTGTACTGTATTGGGT-CATCCTATTAGTAAGCC-GATCTGGACCGATTTATCGGATTCTGA-------------TATTCTTGATCGATTTTGTCGGAT-----ATGTAGAAAT---CTTTGTCGT-TATCACAGCGGATCCTC-AAAGAA--------ACAGGTTTTGTATCGTATAAAGTA-TATACTTCGACTTTCGTGTGCTAG-AACTTTGGCTCGTAAACATAAAAGTACAGTACGCACTTTTATGCGAAGATTAGGTTCGGGATTCTTAG-AAGAATTTTTTTT------GGAAGAAGAAA-AAGTTCTTTCTT

Apostasia_nipponica_PK12273 ACAAGAATTATTTTTCTTCTAATTTTGCT------------TTTCAAATGGCATCAGAAGGTTTTGGAGTCATTCTGGAAATTCCATTCTCGTCGCGATTAGTTTCT---------TCCCCTGAAG---AAATAAAAATACCAAAATCTCTGAATTTACGATCTATTCATTCAATATTTCCTTTTTTAGAGGACAAATTCTTACATTTAAATTATGTATTAG-GTA-TACT-AATACCCCACCCCATCCATC-TGGGAATCTT-GGTTCAAATCCTTCAATGCTGG-ATCAAAGATGCTCCTTCT-TTGCATTTATTG-TTAAAAAT--TTTTCACG------AATATTATAATT---------------CGAATAGTCTTTT---TACT-TC----------------------------------------------AAAAAAAAGAAGATT----TTTGGCTTCTTATATA------ATTCTTAT-ATATGT-GAATGC----GAATTTCTTT-TACTA---TTTTTTCGT--AAACAG-TCTTCC-TACT-TACG-AT-CAACAT-CTTCTGGA-GTTTTTCTT-GAACG-AATGCATTT--CTATGGAAA--AATAGAG------TATCTTGTAGT------ATGTT-GTAATT------CTTTTCA---------AAGG-ATCCCATGCT-TCTTCAA-AAATCTT-TTCA-TGCATTAT------------GTTC-----GATATCAAGGAAAA---GGA-----ATTCT--GGCTTC-----AAAGG---GTACTCTT---------ATTC------------TGATGAAAAAAT-GGAAATAT--CATCTT-GTAAA-TTTTTGGCA-ATC--TTATT-TTCGCTTT-TGGTCTC--AACCATATAGGA----TACATATAAA-ACAATT--ATTCAACTATTCCTTTTCGTTTATGGGG-TATTTTTCAAGTGTACTAAGATA-TTCTTTGGT-AGTAA-GAAATC-AAATGCTAGAGAATTCATTT-CTCATGGATATTCT-GATTAAGA-AATTAGATACCATA-GTTCCAGTT--GTTTCTCTTATTGG-ATCAATGTCGAAAGCCCA-ATTTTGTACTGTATTGGGT-CATCCTATTAGTAAACC-GATTTGGACTGATTTTTCAGATTCTGA-------------TATTCTTGATCGATTTTGTAGAAT-----ATGTAAAAAT---CTTTGTCGT-TTTTACAGTGGATCCTC-AAAAAA--------ACATGTTTTGTATCATATAAAATA-TATACTTCGACTTTCGTGTGCTAG-AACTTTAGCTCGTAAACATAAAAGTACAGTACGTACTTTGATGCGAAGATTAGGTTCGGGATTCTTAG-AAAAATTCTTTAT------GGAAAAAGAAC-AAGTTCTTTCTT

Apostasia_nipponica_PK12274 ACAAGAATTATTTTTCTTCTAATTTTGCT------------TTTCAAATGGCATCAGAAGGTTTTGGAGTCATTCTGGAAATTCCATTCTCGTCGCGATTAGTTTCT---------TCCCCTGAAG---AAATAAAAATACCAAAATCTCTGAATTTACGATCTATTCATTCAATATTTCCTTTTTTAGAGGACAAATTCTTACATTTAAATTATGTATTAG-GTA-TACT-AATACCCCACCCCATCCATC-TGGGAATCTT-GGTTCAAATCCTTCAATGCTGG-ATCAAAGATGCTCCTTCT-TTGCATTTATTG-TTAAAAAT--TTTTCACG------AATATTATAATT---------------CGAATAGTCTTTT---TACT-TC----------------------------------------------AAAAAAAAGAAGATT----TTTGGCTTCTTATATA------ATTCTTAT-ATATGT-GAATGC----GAATTTCTTT-TACTA---TTTTTTCGT--AAACAG-TCTTCC-TACT-TACG-AT-CAACAT-CTTCTGGA-GTTTTTCTT-GAACG-AATGCATTT--CTATGGAAA--AATAGAG------TATCTTGTAGT------ATGTT-GTAATT------CTTTTCA---------AAGG-ATCCCATGCT-TCTTCAA-AAATCTT-TTCA-TGCATTAT------------GTTC-----GATATCAAGGAAAA---GGA-----ATTCT--GGCTTC-----AAAGG---GTACTCTT---------ATTC------------TGATGAAAAAAT-GGAAATAT--CATCTT-GTAAA-TTTTTGGCA-ATC--TTATT-TTCGCTTT-TGGTCTC--AACCATATAGGA----TACATATAAA-ACAATT--ATTCAACTATTCCTTTTCGTTTATGGGG-TATTTTTCAAGTGTACTAAGATA-TTCTTTGGT-AGTAA-GAAATC-AAATGCTAGAGAATTCATTT-CTCATGGATATTCT-GATTAAGA-AATTAGATACCATA-GTTCCAGTT--GTTTCTCTTATTGG-ATCAATGTCGAAAGCCCA-ATTTTGTACTGTATTGGGT-CATCCTATTAGTAAACC-GATTTGGACTGATTTTTCAGATTCTGA-------------TATTCTTGATCGATTTTGTAGAAT-----ATGTAAAAAT---CTTTGTCGT-TTTTACAGTGGATCCTC-AAAAAA--------ACATGTTTTGTATCATATAAAATA-TATACTTCGACTTTCGTGTGCTAG-AACTTTAGCTCGTAAACATAAAAGTACAGTACGTACTTTGATGCGAAGATTAGGTTCGGGATTCTTAG-AAAAATTCTTTAT------GGAAAAAGAAC-AAGTTCTTTCTT

Appendicula_cornuta_AY121739 ACAAGAATTCTTTTTCT------------------------TCTCAAATGGTATCAGAAGGTTTTGGAGTCATTCTGGAAATTCCATTCTCGTCGCGATTAGTATCT---------TTCCTTGAAG---AAAAAAGAATACCAAAATATCAGAATTTACGATCTATTCATTCAATATTTCCCTTTTTAGAGGATAAATTATTACATTTAAATTATGTGTCAG-ATC-TACT-AATACCCCATCCCATCCATC-TGGAAATATT-GGTTCAAATTCTTCAATGCTGG-ATCAAAGATGTTCCTTCT-TTGCATTTATTA-CGATTGTT--TTTTCACG------AATATCATAATT---------------TGAATAGTCTCAT---TATT-TC---------------AAAGAAATCCATTTACGTCT-TTTCAAAAAG---AAAGAAAAGAT-TAT-TTTTG-TTCCTACATA------ATTCCTAT-GTATAT-GAATGC----GAATATCTAT-TCCTG---TTTCTTCGT--AAACAG-TCTTCT-TATT-TACG-AT-CAATAT-CTTCTGGA-GTCTTTCTT-GAGCG-AACACATTT--CTATGTAAA--AATAGAA------TATCTTATAGC---CGTATATT-GTAATT------CTTTTCA---------TAGG-ATCCTATGGT-TCCTCAA-AGATACT-TTCA-TACATTAT------------GTTC-----GATATCAAGGAAAA---GCG-----ATTCT--GGCTTC-----AAAAG---GAACTCTT---------ATTC------------TGATGAATAAAT-GGAAATTT--CATCTT-GTGAA-TCTTTGGCA-ATC--TTATT-TTCACTTT-TGGTTTC--AACCTGATAGGA----TCCATATAAA-GCAATT--ACCCAACTATTCTTTCTCTTTTCTGGGG-TATTTTTCAAGTGTACTAAAAAA-TCCTTTGGT-AGTAA-GAAATC-AAATGTTAGATAATTCATTT-CTAATAAATACTCT-ATCTAAGA-AATTAGATACCATA-GTCCCAGTT--ATTTCTCTTATAGG-ATCATTGTCGAAAGCTCA-ATTTTGTACTGTATTGGGT-CATCCTATTAGTAAACC-GATCTGGACCGATTTATCGGATTCTGA-------------TATTCTTGATCGATTTTGTCGAAT-----ATGTAGAGAT---CTTTGTCGT-TATCACAGCGGATCCTC-AAAGAA--------ACAGGTTTTGTATCGTATAAAGTA-TATACTTCGACTTTCGTGTGCTAG-AACTTTGGCTCGTAAACATAAAAGTACAGTACGCACTTTTATGCGAAGATTAGGTTCGGGATTCTTAG-AAGAATTCTTTTT------GGAAGAAGAAC-AATCTCTTTCTT

Appendicula_cornuta_AY368393 ACAAGAATTCTTTTTCT------------------------TCTCAAATGGTATCAGAAGGTTTTGGAGTCATTCTGGAAATTCCATTCTCGTCGCGATTAGTATCT---------TTCCTTGAAG---AAAAAAGAATACCAAAATATCAGAATTTACGATCTATTCATTCAATATTTCCCTTTTTAGAGGATAAATTATTACATTTAAATTATGTGTCAG-ATC-TACT-AATACCCCATCCCATCCATC-TGGAAATATT-GGTTCAAATTCTTCAATGCTGG-ATCAAAGATGTTCCTTCT-TTGCATTTATTA-CGATTGTT--TTTTCACG------AATATCATAATT---------------TGAATAGTCTCAT---TATT-TC---------------AAAGAAATCCATTTACGTCT-TTTCAAAAAG---AAAGAAAAGAT-TAT-TTTTG-TTCCTACATA------ATTCCTAT-GTATAT-GAATGC----GAATATCTAT-TCCTG---TTTCTTCGT--AAACAG-TCTTCT-TATT-TACG-AT-CAATAT-CTTCTGGA-GTCTTTCTT-GAGCG-AACACATTT--CTATGTAAA--AATAGAA------TATCTTATAGC---CGTATATT-GTAATT------CTTTTCA---------TAGG-ATCCTATGGT-TCCTCAA-AGATACT-TTCA-TACATTAT------------GTTC-----GATATCAAGGAAAA---GCG-----ATTCT--GGCTTC-----AAAAG---GAACTCTT---------ATTC------------TGATGAATAAAT-GGAAATTT--CATCTT-GTGAA-TCTTTGGCA-ATC--TTATT-TTCACTTT-TGGTTTC--AACCTGATAGGA----TCCATATAAA-GCAATT--ACCCAACTATTCTTTCTCTTTTCTGGGG-TATTTTTCAAGTGTACTAAAAAA-TCCTTTGGT-AGTAA-GAAATC-AAATGTTAGATAATTCATTT-CTAATAAATACTCT-ATCTAAGA-AATTAGATACCATA-GTCCCAGTT--ATTTCTCTTATAGG-ATCATTGTCGAAAGCTCA-ATTTTGTACTGTATTGGGT-CATCCTATTAGTAAACC-GATCTGGACCGATTTATCGGATTCTGA-------------TATTCTTGATCGATTTTGTCGAAT-----ATGTAGAGAT---CTTTGTCGT-TATCACAGCGGATCCTC-AAAGAA--------ACAGGTTTTGTATCGTATAAAGTA-TATACTTCGACTTTCGTGTGCTAG-AACTTTGGCTCGTAAACATAAAAGTACAGTACGCACTTTTATGCGAAGATTAGGTTCGGGATTCTTAG-AAGAATTCTTTTT------GGAAGAAGAAC-AATCTCTTTCTT

Appendicula_cornuta_KF361651 -----------------------------------------------------------------------------------------------------------------------------------------------------------------------------------------------------------------AATTATGTGTCAG-ATC-TACT-AATACCCCATCCCATCCATC-TGGAAATATT-GGTTCAAATTCTTCAATGCTGG-ATCAAAGATGTTCCTTCT-TTGCATTTATTG-CGATTGTT--TTTTCACG------AATATCATAATT---------------TGAATAGTCTCAT---TATT-TC---------------AAAGAAATCCATTTACGTCT-TTTCAAAAAG---AAAGAAAAGAT-TAT-TTTTG-TTCCTACATA------ATTCCTAT-GTATAT-GAATGC----GAATATCTAT-TCCTG---TTTCTTCGT--AAACAG-TCTTCT-TATT-TACG-AT-CAATAT-CTTCTGGA-GTCTTTCTT-GAGCG-AACACATTT--CTATGTAAA--AATAGAA------TATCTTATAGC---CGTATATT-GTAATT------CTTTTCA---------TAGG-ATCCTATGGT-TCCTCAA-AGATACT-TTCA-TAAATTAT------------GTTC-----GATATCAAGGAAAA---GCG-----ATTCT--GGCTTC-----AAAAG---GAACTCTT---------ATTC------------TGATGAATAAAT-GGAAATTT--CATCTT-GTGAA-TCTTTGGCA-ATC--TTATT-TTCACTTT-TGGTTTC--AACCTTATAGGA----TCCATATAAA-GCAATT--ACCCAACTATTCTTTCTCTTTTCTGGGG-TATTTTTCAAGTGTACTAAAAAA-TCCTTTGGT-AGTAA-GAAATC-AAATGTTAGATAATTCATTT-CTAATAAATACTCT-ATCTAAGA-AATTAGATACCATA-GTCCCAGTT--ATTTCTCTTATAGG-ATCATTGTCGAAAGCTCA-ATTTTGTACTGTATTGGGT-CATCCTATTAGTAAACC-GATCTGGACCGATTTATCGGATTCTGA-------------TATTCTTGATCGATTTTGTCGAAT-----ATGTAGAGAT---CTTTGTCGT-TATCACAGCGGATCCTC-AAAGAA--------ACAGGTTTTGTAT-----------------------------------------------------------------------------------------------------------------------------------------------------

Appendicula_cornuta_KY239521 ACAAGAATTCTTTTTCT------------------------TCTCAAATGGTATCAGAAGGTTTTGGAGTCATTCTGGAAATTCCATTCTCGTCGCGATTAGTATCT---------TTCCTTGAAG---AAAAAAGAATACCAAAATATCAGAATTTACGATCTATTCATTCAATATTTCCCTTTTTAGAGGATAAATTATTACATTTAAATTATGTGTCAG-ATC-TACT-AATACCCCATCCCATCCATC-TGGAAATATT-GGTTCAAATTCTTCAATGCTGG-ATCAAAGATGTTCCTTCT-TTGCATTTATTA-CGATTGTT--TTTTCACG------AATATCATAATT---------------TGAATAGTCTCAT---TATT-TC---------------AAAGAAATCCATTTACGTCT-TTTCAAAAAG---AAAGAAAAGAT-TAT-TTTTG-TTCCTACATA------ATTCCTAT-GTATAT-GAATGC----GAATATCTAT-TCCTG---TTTCTTCGT--AAACAG-TCTTCT-TATT-TACG-AT-CAATAT-CTTCTGGA-GTCTTTCTT-GAGCG-AACACATTT--CTATGTAAA--AATAGAA------TATCTTATAGC---CGTATATT-GTAATT------CTTTTCA---------TAGG-ATCCTATGGT-TCCTCAA-AGATACT-TTCA-TACATTAT------------GTTC-----GATATCAAGGAAAA---GCG-----ATTCT--GGCTTC-----AAAAG---GAACTCTT---------ATTC------------TGATGAATAAAT-GGAAATTT--CATCTT-GTGAA-TCTTTGGCA-ATC--TTATT-TTCACTTT-TGGTTTC--AACCTGATAGGA----TCCATATAAA-GCAATT--ACCCAACTATTCTTTCTCTTTTCTGGGG-TATTTTTCAAGTGTACTAAAAAA-TCCTTTGGT-AGTAA-GAAATC-AAATGTTAGATAATTCATTT-CTAATAAATACTCT-ATCTAAGA-AATTAGATACCATA-GTCCCAGTT--ATTTCTCTTATAGG-ATCATTGTCGAAAGCTCA-ATTTTGTACTGTATTGGGT-CATCCTATTAGTAAACC-GATCTGGACCGATTTATCGGATTCTGA-------------TATTCTTGATCGATTTTGTCGAAT-----ATGTAGAGAT---CTTTGTCGT-TATCACAGCGGATCCTC-AAAGAA--------ACAGGTTTTGTATCGTATAAAGTA-TATACTTCGACTTTCGTGTGCTAG-AACTTTGGCTCGTAAACATAAAAGTACAGTACGCACTTTTATGCGAAGATTAGGTTCGGGATTCTTAG-AAGAATTCTTTTT------GGAAGAAGAAC-AATCTCTTTCTT

Appendicula_cornuta_KY966709 ACAAGAATTCTTTTTCT------------------------TCTCAAATGGTATCAGAAAGTTTTGGAGTCATTCTGGAAATTCCATTCTCGTCGCGATTAGTATCT---------TTCCTTGAAG---AAAGAAGAATACCAAAATCTCAGAATTTACGATCTATTCATTCAATATTTCCCTTTTTAGAGGATAAATTATTACATTTAAATTATGTGTCAG-ATC-TACT-AATACCCCATCCCATCCATC-TGGAAATATT-GGTTCAAATTCTTCAATGCTGG-ATCAAAGATGTTCCTTCT-TTGCATTTCTTG-CGATTGTT--TTTTCACG------AATATCATAATT---------------TGAATAGTCTCAT---TATT-TC---------------AAAGAAATCCATTTACGTCT-TTTCAAAAAG---AAAGAAAAGAT-TAT-TTTTG-TTCCTACATA------ATTCTTAT-GTATAT-GAATGC----GAATATCTAT-TCCTG---TTTCTTCGT--AAACAC-TCTTCT-TATT-TACG-AT-CAATAT-CTTCTGGA-ATCTTTCTT-GAGCG-AACACATTT--CTATGTAAA--AATAGAA------TATCTTATAGC---CGTGTATT-GTAATT------TTTTTCA---------GAGG-ATCCTATGGT-TCCTCAA-AGATACT-TTCA-TACATTAT------------GTTC-----GATATCAAGGAAAA---GCG-----ATCCT--GGCTTC-----AAAAG---GAACTCTT---------ATTC------------TGATGAAGAAAT-GGCAATTT--CATCTT-GTGAA-TTTTTGGCA-ATC--TTATT-TTCACTTT-TGGTTTC--AACCTTATAGGA----TCCATATAAA-GCAATT--ACCCAACTATTCCTTCTCTTTTCTGGGG-TATTTTTCAAGTGTACTAAAAAA-TAATTTGGT-AGTAA-GAAATC-AAATGTTAGAGAATTCATTT-CTAATAAATACTCT-ATCTAATA-AATTAGATACCATA-GTACCAGTT--ATTTCTCTTATAGG-ATCATTGTCGAAAGCTCA-ATTTTGTACTGTATTGGGC-CATCCTATTAGTAAACC-GATCTGGACCGATTTATCGGATTCTGA-------------TATTCTTGATCGATTTTGTCGTAT-----ATGTAGAGAT---CTTTGTCGT-TATCACAGGGGATCCTC-AAAGAA--------ACAGGTTTTGTATCGTATAAAGTA-TATACTTCGAATTTCGTGTGCTAG-AACTTTGGCTCGTAAACATAAAAGTACAGTACGCACTTTTATGCGAAGATTAGGTTCGGGATTCTTAG-AAGAATTCTT---------------------------------

Appendicula_cornuta_KY966710 ACAAGAATTCTTTTTCT------------------------TCTCAAATGGTATCAGAAGGTTTTGGAGTCATTCTGGAAATTCCATTCTCGTCGCGATTAGTATCT---------TTCCTTGAAG---AAAAAAGAATACCAAAATATCAGAATTTACGATCTATTCATTCAATATTTCCCTTTTTAGAGGATAAATTATTACATTTAAATTATGTGTCAG-ATC-TACT-AATACCCCATCCCATCCATC-TGGAAATATT-GGTTCAAATTCTTCAATGCTGG-ATCAAAGATGTTCCTTCT-TTGCATTTATTG-CGATTGTT--TTTTCACG------AATATCATAATT---------------TGAATAGTCTCAT---TATT-TC---------------AAAGAAATCCATTTACGTCT-TTTCAAAAAG---AAAGAAAAGAT-TAT-TTTTG-TTCCTACATA------ATTCCTAT-GTATAT-GAATGC----GAATATCTAT-TCCTG---TTTCTTCGT--AAACAG-TCTTCT-TATT-TACG-AT-CAATAT-CTTCTGGA-GTCTTTCTT-GAGCG-AACACATTT--CTATGTAAA--AATAGAA------TATCTTATAGC---CGTATATT-GTAATT------CTTTTCA---------TAGG-ATCCTATGGT-TCCTCAA-AGATACT-TTCA-TAAATTAT------------GTTC-----GATATCAAGGAAAA---GCG-----ATTCT--GGCTTC-----AAAAG---GAACTCTT---------ATTC------------TGATGAATAAAT-GGAAATTT--CATCTT-GTGAA-TCTTTGGCA-ATC--TTATT-TTCACTTT-TGGTTTC--AACCTTATAGGA----TCCATATAAA-GCAATT--ACCCAACTATTCTTTCTCTTTTCTGGGG-TATTTTTCAAGTGTACTAAAAAA-TCCTTTGGT-AGTAA-GAAATC-AAATGTTAGATAATTCATTT-CTAATAAATACTCT-ATCTAAGA-AATTAGATACCATA-GTCCCAGTT--ATTTCTCTTATAGG-ATCATTGTCGAAAGCTCA-ATTTTGTACTGTATTGGGT-CATCCTATTAGTAAACC-GATCTGGACCGATTTATCGGATTCTGA-------------TATTCTTGATCGATTTTGTCGAAT-----ATGTAGAGAT---CTTTGTCGT-TATCACAGCGGATCCTC-AAAGAA--------ACAGGTTTTGTATCGTATAAAGTA-TATACTTCGACTTTCGTGTGCTAG-AACTTTGGCTCGTAAACATAAAAGTACAGTACGCACTTTTATGCGAAGATTAGGTTCGGGATTCTTAG-AAGAATTCTT---------------------------------

Appendicula_cornuta_PK12065 ACAAGAATTCTTTTTCT------------------------TCTCAAATGGTATCAGAAGGTTTTGGAGTCATTCTGGAAATTCCATTCTCGTCGCGATTAGTATCT---------TTCCTTGAAG---AAAAAAGAATACCAAAATATCAGAATTTACGATCTATTCATTCAATATTTCCCTTTTTAGAGGATAAATTATTACATTTAAATTATGTGTCAG-ATC-TACT-AATACCCCATCCCATCCATC-TGGAAATATT-GGTTCAAATTCTTCAATGCTGG-ATCAAAGATGTTCCTTCT-TTGCATTTATTG-CGATTGTT--TTTTCACG------AATATCATAATT---------------TGAATAGTCTCAT---TATT-TC---------------AAAGAAATCCATTTACGTCT-TTTCAAAAAG---AAAGAAAAGAT-TAT-TTTTG-TTCCTACATA------ATTCCTAT-GTATAT-GAATGC----GAATATCTAT-TCCTG---TTTCTTCGT--AAACAG-TCTTCT-TATT-TACG-AT-CAATAT-CTTCTGGA-GTCTTTCTT-GAGCG-AACACATTT--CTATGTAAA--AATAGAA------TATCTTATAGC---CGTATATT-GTAATT------CTTTTCA---------TAGG-ATCCTATGGT-TCCTCAA-AGATACT-TTCA-TAAATTAT------------GTTC-----GATATCAAGGAAAA---GCG-----ATTCT--GGCTTC-----AAAAG---GAACTCTT---------ATTC------------TGATGAATAAAT-GGAAATTT--CATCTT-GTGAA-TCTTTGGCA-ATC--TTATT-TTCACTTT-TGGTTTC--AACCTTATAGGA----TCCATATAAA-GCAATT--ACCCAACTATTCTTTCTCTTTTCTGGGG-TATTTTTCAAGTGTACTAAAAAA-TCCTTTGGT-AGTAA-GAAATC-AAATGTTAGATAATTCATTT-CTAATAAATACTCT-ATCTAAGA-AATTAGATACCATA-GTCCCAGTT--ATTTCTCTTATAGG-ATCATTGTCGAAAGCTCA-ATTTTGTACTGTATTGGGT-CATCCTATTAGTAAACC-GATCTGGACCGATTTATCGGATTCTGA-------------TATTCTTGATCGATTTTGTCGAAT-----ATGTAGAGAT---CTTTGTCGT-TATCACAGCGGATCCTC-AAAGAA--------ACAGGTTTTGTATCGTATAAAGTA-TATACTTCGACTTTCGTGTGCTAG-AACTTTGGCTCGTAAACATAAAAGTACAGTACGCACTTTTATGCGAAGATTAGGTTCGGGATTCTTAG-AAGAATTCTTTTT------GGAAGAAGAAC-AATCTCTTTCTT

Arundina_graminifolia_AF263626 ACAAGAATTCTTTTTCTTCTCATTTTTCT------------TCTCAAATGGTATCAGAAGGTTTTGGAGTCATTCTGGAAATTCCATTCTCGTCGCAATTAGTATCT---------TCCCTTGAAG---ATAACAGAATACCAAAATTTCAGAATTTACTATCTATTCATTCAATATTTCCCTTTTTAGAGGATAAATTATCACATTTCAATTATGTGTCAG-ATC-TACT-AATACCCCATCCCATCCATC-TGGAAATCTT-GGTTCAAATCCTTCAATGCTGG-ATTAAAGATGTTTCTTCT-TTGCATTTCTTG-CGATTGTT--TTTCCACG------AATATCATAATT---------------TGAATAGTCTCAT---TACT-TC---------------AAATAAATCCATTTACGTCT-TTTCAAAAAG---AACCAAAAGAT-TCT-TTTGG-TTCCTACATA------ACTCTTAT-GTATAT-GAATGC----GAATATATAT-TCCTG---TTTCTTCGT--AAACAG-TCTTCT-TATT-TACG-AT-CAATAT-CTTCTGGA-GTCTTTCTT-GAGCG-AACACATTT--CTATGGAAA--AATAGAA------TATCTTATAGT---CGTGTGTT-GTAATT------CTTTTCA---------GAGG-ATCCTATGGT-TCCTCAA-AGATACT-TTCA-TACATTAT------------GTTC-----GATATCAAGGAAAA---ACA-----ATTCT--GGTTTC-----AAAAG---GAACTCTT---------ATTC------------TGATTAAGAAAT-GGAAATTT--CATCGT-GTGTA-GTTTTGGCA-ATC--TAATT-TTCACTTT-TGGTTTC--AACCTTATAGGA----TTCATATAAA-GCAATT--ACCCAACTATTCCTTCTCTTTTCTGGGG-TATTTTTCAAGTGTACTAAAAAA-TAATTTGGT-AATAA-GAAATC-AAATGCTAGAGAATTCATTT-CTAATAAATACTCT-GACTAAGA-AATTAGATACCATA-GCCCCAGTT--ACTTCTATTATTGG-ATCATTGTCGAAAGCTCA-ATTTTGTACTGTATTGGGT-CATCCTATTAGTAAACC-GATCTGGACCGATTTATCGGATTCTGA-------------TATTCTTGATCGATTTTGTCGGAT-----ATGTAGAAAT---CTTTGTCGT-TATCACAGCGGATCTTC-AAAGAA--------ACAGGTTTTGTATCGTATAAAGTA-TATACTTCGACTTTCGTGTGCTAG-AACTTTGGCTCGTAAACATAAAAGTACAGTACGCACTTTTATGCGAAGATTAGGTTCGGGATTCTTAG-AAGAATTTTTTTTT------GAAGAAGAAC-AATCTCTTTCTT

Arundina_graminifolia_AF302692 ACAAGAATTCTTTTTCTTCTCATTTTTCT------------TCTCAAATGGTATCAGAAGGTTTTGGAGTCATTCTGGAAATTCCATTCTCGTCGCAATTAGTATCT---------TCCCTTGAAG---ATAACAGAATACCAAAATTTCAGAATTTACTATCTATTCATTCAATATTTCCCTTTTTAGAGGATAAATTATCACATTTCAATTATGTGTCAG-ATC-TACT-AATACCCCATCCCATCCATC-TGGAAATCTT-GGTTCAAATCCTTCAATGCTGG-ATTAAAGATGTTTCTTCT-TTGCATTTCTTG-CGATTGTT--TTTCCACG------AATATCATAATT---------------TGAATAGTCTCAT---TACT-TC---------------AAATAAATCCATTTACGTCT-TTTCAAAAAG---AACCAAAAGAT-TCT-TTTGG-TTCCTACATA------ACTCTTAT-GTATAT-GAATGC----GAATATATAT-TCCTG---TTTCTTCGT--AAACAG-TCTTCT-TATT-TACG-AT-CAATAT-CTTCTGGA-GTCTTTCTT-GAGCG-AACACATTT--CTATGGAAA--AATAGAA------TATCTTATAGT---CGTGTGTT-GTAATT------CTTTTCA---------GAGG-ATCCTATGGT-TCCTCAA-AGATACT-TTCA-TACATTAT------------GTTC-----GATATCAAGGAAAA---ACA-----ATTCT--GGTTTC-----AAAAG---GAACTCTT---------ATTC------------TGATTAAGAAAT-GGAAATTT--CATCGT-GTGTA-GTTTTGGCA-ATC--TAATT-TTCACTTT-TGGTTTC--AACCTTATAGGA----TTCATATAAA-GCAATT--ACCCAACTATTCCTTCTCTTTTCTGGGG-TATTTTTCAAGTGTACTAAAAAA-TAATTTGGT-AATAA-GAAATC-AAATGCTAGAGAATTCATTT-CTAATAAATACTCT-GACTAAGA-AATTAGATACCATA-GCCCCAGTT--ACTTCTATTATTGG-ATCATTGTCGAAAGCTCA-ATTTTGTACTGTATTGGGT-CATCCTATTAGTAAACC-GATCTGGACCGATTTATCGGATTCTGA-------------TATTCTTGATCGATTTTGTCGGAT-----ATGTAGAAAT---CTTTGTCGT-TATCACAGCGGATCTTC-AAAGAA--------ACAGGTTTTGTATCGTATAAAGTA-TATACTTCGACTTTCGTGTGCTAG-AACTTTGGCTCGTAAACATAAAAGTACAGTACGCACTTTTATGCGAAGATTAGGTTCGGGATTCTTAG-AAGAATTTTTTTTT------GAAGAAGAAC-AATCTCTTTCTT

Arundina_graminifolia_EF079333 ACAAGAATTCTTTTTCTTCTCATTTTTCT------------TCTCAAATGGTATCAGAAGGTTTTGGAGTCATTCTGGAAATTCCATTCTCGTCGCAATTAGTATCT---------TCCCTTGAAG---ATAACAGAATACCAAAATTTCAGAATTTACTATCTATTCATTCAATATTTCCCTTTTTAGAGGATAAATTATCACATTTCAATTATGTGTCAG-ATC-TACT-AATACCCCATCCCATCCATC-TGGAAATCTT-GGTTCAAATCCTTCAATGCTGG-ATTAAAGATGTTTCTTCT-TTGCATTTCTTG-CGATTGTT--TTTCCACG------AATATCATAATT---------------TGAATAGTCTCAT---TACT-TC---------------AAATAAATCCATTTACGTCT-TTTCAAAAAG---AACCAAAAGAT-TCT-TTTGG-TTCCTACATA------ATTCTTAT-GTATAT-GAATGC----GAATATATAT-TCCTG---TTTCTTCGT--AAACAG-TCTTCT-TATT-TACG-AT-CAATAT-CTTCTGGA-GTCTTTCTT-GAGCG-AACACATTT--CTATGGAAA--AATAGAA------TATCTTATAGT---CGTGTGTT-GTAATT------CTTTTCA---------GAGG-ATCCTATGGT-TCCTCAA-AGATACT-TTCA-TACATTAT------------GTTC-----GATATCAAGGAAAA---ACA-----ATTCT--GGTTTC-----AAAAG---GAACTCTT---------ATTC------------TGATTAAGAAAT-GGAAATTT--CATCTT-GTGAA-TTTTTGGCA-ATC--TTATT-TTCACTTT-TGGTTTC--AACCTTATAGGA----TTCATATAAA-GCAATT--ACCCAACTATTCCTTCTCTTTTCTGGGG-TATTTTTCAAGTGTACTAAAAAA-TAATTTGGT-AATAA-GAAATC-AAATGCTAGAGAATTCATTT-CTAATAAATACTCT-GACTAAGA-AATTAGATACCATA-GCCCCAGTT--ACTTCTATTATTGG-ATCATTGTCGAAAGCTCA-ATTTTGTACTGTATTGGGT-CATCCTATTAGTAAACC-GATCTGGACCGATTTATCGGATTCTGA-------------TATTTTTGATCGATTTTGTCGGAT-----ATGTAGAAAT---CTTTGTCGT-TATCACAGCGGATCTTC-AAAGAA--------ACAGGTTTTGTATCGTATAAAGTA-TATACTTCGACTTTCGTGTGCTAG-AACTTTGGCTCGTAAACATAAAAGTACAGTACGCACTTTTATGCGAAGATTAGGTTCGGGATTCTTAG-AAGAATTTTTTTT------TGAAGAAGAAC-AATCTCTTTCTT

Arundina_graminifolia_JN004354 ----------------------------------------------------------------------------------------------------------------------------------------------------------------------------------------------------------------------------------------------------------------------------------------------------------------------ATTTCTTG-CGATTGTT--TTTCCACG------AATATCATAATT---------------TGAATAGTCTCAT---TACT-TC---------------AAATAAATCCATTTACGTCT-TTTCAAAAAG---AACCAAAAGAT-TCT-TTTGG-TTCCTACATA------ATTCTTAT-GTATAT-GAATGC----GAATATATAT-TCCTG---TTTCTTCGT--AAACAG-TCTTCT-TATT-TACG-AT-CAATAT-CTTCTGGA-GTCTTTCTT-GAGCG-AACACATTT--CTATGGAAA--AATAGAA------TATCTTATAGT---CGTGTGTT-GTAATT------CTTTTCA---------GAGG-ATCCTATGGT-TCCTCAA-AGATACT-TTCA-TACATTAT------------GTTC-----GATATCAAGGAAAA---ACA-----ATTCT--GGTTTC-----AAAAG---GAACTCTT---------ATTC------------TGATTAAGAAAT-GGAAATTT--CATCTT-GTGAA-TTTTTGGCA-ATC--TTATT-TTCACTTT-TGGTTTC--AACCTTATAGGA----TTCATATAAA-GCAATT--ACCCAACTATTCCTTCTCTTTTCTGGGG-TATTTTTCAAGTGTACTAAAAAA-TAATTTGGT-AATAA-GAAATC-AAATGCTAGAGAATTCATTT-CTAATAAATACTCT-GACTAAGA-AATTAGATACCATA-GCCCCAGTT--ACTTCTATTATTGG-ATCATTGTCGAAAGCTCA-ATTTTGTACTGTATTGGGT-CATCCTATTAGTAAACC-GATCTGGACCGATTTATCGGATTCTGA-------------TATTTTTGATCGATTTTGTCGGAT-----ATGTAGAAAT---CTTTGTCGT-TATCACAGCGGATCTTC-AAAGAA--------ACAGGTTT----------------------------------------------------------------------------------------------------------------------------------------------------------

Arundina_graminifolia_JN004355 ----------------------------------------------------------------------------------------------------------------------------------------------------------------------------------------------------------------------------------------------------------------------------------------------------------------------ATTTCTTG-CGATTGTT--TTTCCACG------AATATCATAATT---------------TGAATAGTCTCAT---TACT-TC---------------AAATAAATCCATTTACGTCT-TTTCAAAAAG---AACCAAAAGAT-TCT-TTTGG-TTCCTACATA------ATTCTTAT-GTATAT-GAATGC----GAATATATAT-TCCTG---TTTCTTCGT--AAACAG-TCTTCT-TATT-TACG-AT-CAATAT-CTTCTGGA-GTCTTTCTT-GAGCG-AACACATTT--CTATGGAAA--AATAGAA------TATCTTATAGT---CGTGTGTT-GTAATT------CTTTTCA---------GAGG-ATCCTATGGT-TCCTCAA-AGATACT-TTCA-TACATTAT------------GTTC-----GATATCAAGGAAAA---ACA-----ATTCT--GGTTTC-----AAAAG---GAACTCTT---------ATTC------------TGATTAAGAAAT-GGAAATTT--CATCTT-GTGAA-TTTTTGGCA-ATC--TTATT-TTCACTTT-TGGTTTC--AACCTTATAGGA----TTCATATAAA-GCAATT--ACCCAACTATTCCTTCTCTTTTCTGGGG-TATTTTTCAAGTGTACTAAAAAA-TAATTTGGT-AATAA-GAAATC-AAATGCTAGAGAATTCATTT-CTAATAAATACTCT-GACTAAGA-AATTAGATACCATA-GCCCCAGTT--ACTTCTATTATTGG-ATCATTGTCGAAAGCTCA-ATTTTGTACTGTATTGGGT-CATCCTATTAGTAAACC-GATCTGGACCGATTTATCGGATTCTGA-------------TATTTTTGATCGATTTTGTCGGAT-----ATGTAGAAAT---CTTTGTCGT-TATCACAGCGGATCTT----------------------------------------------------------------------------------------------------------------------------------------------------------------------------------

Arundina_graminifolia_JN004356 --------------------------------------------------------------------------------------------------------------------------------------------------------------------------------------------------------------------------------------------------------------------------------------------------------------TCT-TTGCATTTCTTG-CGATTGTT--TTTCCACG------AATATCATAATT---------------TGAATAGTCTCAT---TACT-TC---------------AAATAAATCCATTTACGTCT-TTTCAAAAAG---AACCAAAAGAT-TCT-TTTGG-TTCCTACATA------ATTCTTAT-GTATAT-GAATGC----GAATATATAT-TCCTG---TTTCTTCGT--AAACAG-TCTTCT-TATT-TACG-AT-CAATAT-CTTCTGGA-GTCTTTCTT-GAGCG-AACACATTT--CTATGGAAA--AATAGAA------TATCTTATAGT---CGTGTGTT-GTAATT------CTTTTCA---------GAGG-ATCCTATGGT-TCCTCAA-AGATACT-TTCA-TACATTAT------------GTTC-----GATATCAAGGAAAA---ACA-----ATTCT--GGTTTC-----AAAAG---GAACTCTT---------ATTC------------TGATTAAGAAAT-GGAAATTT--CATCTT-GTGAA-TTTTTGGCA-ATC--TTATT-TTCACTTT-TGGTTTC--AACCTTATAGGA----TTCATATAAA-GCAATT--ACCCAACTATTCCTTCTCTTTTCTGGGG-TATTTTTCAAGTGTACTAAAAAA-TAATTTGGT-AATAA-GAAATC-AAATGCTAGAGAATTCATTT-CTAATAAATACTCT-GACTAAGA-AATTAGATACCATA-GCCCCAGTT--ACTTCTATTATTGG-ATCATTGTCGAAAGCTCA-ATTTTGTACTGTATTGGGT-CATCCTATTAGTAAACC-GATCTGGACCGATTTATCGGATTCTGA-------------TATTTTTGATCGATTTTGTCGGAT-----ATGTAGAAAT---CTTTGTCGT-TA------------------------------------------------------------------------------------------------------------------------------------------------------------------------------------------------

Arundina_graminifolia_KF421845 ------------------------------------------------------------------------------------------------------------------------------------------------------------------------------------------------------------------------------------------------------------------------------------------------------------------------------------------TTCCACG------AATATCATAATT---------------TGAATAGTCTCAT---TACT-TC---------------AAATAAATCCATTTACGTCT-TTTCAAAAAG---AACCAAAAGAT-TCT-TTTGG-TTCCTACATA------ATTCTTAT-GTATAT-GAATGC----GAATATCTAT-TCCTG---TTTCTTCGT--AAACAG-TCTTCT-TATT-TACG-AT-CAATAT-CTTCTGGA-GTCTTTCTT-GAGCG-AACACATTT--CTATGGAAA--AATAGAA------TATCTTATAGT---CGTGTGTT-GTAATT------CTTTTCA---------GAGG-ATCCTATGGT-TCCTCAA-AGATACT-TTCA-TACATTAT------------GTTC-----GATATCAAGGAAAA---ACA-----ATTCT--GGTTTC-----AAAAG---GAACTCTT---------ATTC------------TGATTAAGAAAT-GGAAATTT--CATCTT-GTGAA-TTTTTGGCA-ATC--TTATT-TTCACTTT-TGGTTTC--AACCTTATAGGA----TTCATATAAA-GCAATT--ACCCAACTATTCCTTCTCTTTTCTGGGG-TATTTTTCAAGTGTACTAAAAAA-TAATTTGGT-AATAA-GAAATC-AAATGCTAGAGAATTCATTT-CTAATAAATACTCT-GACTAAGA-AATTAGATACCATA-GCCCCAGTT--ACTTCTATTATTGG-ATCATTGTCGAAAGCTCA-ATTTTGTACTGTATTGGGT-CATCCTATTAGTAAACC-GATCTGGACCGATTTATCGGATTCTGA-------------TATTTTTGATCGATTTTGTCGGAT-----ATGTAGAAAT---CTTTGTCGT-TATCACAGCGGATCTTC-AAAGAA--------------------------------------------------------------------------------------------------------------------------------------------------------------------------

Arundina_graminifolia_KX298566 -----------------------------------------------------------------------------------------------------------------------------------------------------------------------------------------------------------------------------------------------------------------------------------------------------------------------------G-------------------------AATATCATAATT---------------TGAATAGTCTCAT---TACT-TC---------------AAATAAATCCATTTACGTCT-TTTCAAAAAG---AACCAAAAGAT-TCT-TTTGG-TTCCTACATA------ATTCTTAT-GTATAT-GAATGC----GAATATATAT-TCCTG---TTTCTTCGT--AAACAG-TCTTCT-TATT-TACG-AT-CAATAT-CTTCTGGA-GTCTTTCTT-GAGCG-AACACATTT--CTATGGAAA--AATAGAA------TATCTTATAGT---CGTGTGTT-GTAATT------CTTTTCA---------GAGG-ATCCTATGGT-TCCTCAA-AGATACT-TTCA-TACATTAT------------GTTC-----GATATCAAGGAAAA---ACA-----ATTCT--GGTTTC-----AAAAG---GAACTCTT---------ATTC------------TGATTAAGAAAT-GGAAATTT--CATCTT-GTGAA-TTTTTGGCA-ATC--TTATT-TTCACTTT-TGGTTTC--AACCTTATAGGA----TTCATATAAA-GCAATT--ACCCAACTATTCCTTCTCTTTTCTGGGG-TATTTTTCAAGTGTACTAAAAAA-TAATTTGGT-AATAA-GAAATC-AAATGCTAGAGAATTCATTT-CTAATAAATACTCT-GACTAAGA-AATTAGATACCATA-GCCCCAGTT--ACTTCTATTATTGG-ATCATTGTCGAAAGCTCA-ATTTTGTACTGTATTGGGT-CATCCTATTAGTAAACC-GATCTGGACCGATTTATCGGATTCTGA-------------TATTTTTGATCGATTTTGTCGGAT-----ATGTAGAAAT---CTTTGTCGT-TATCACAGCGGA--------------------------------------------------------------------------------------------------------------------------------------------------------------------------------------

Arundina_graminifolia_KY966712 ACAAGAATTCTTTTTCTTCTCATTTTTCT------------TCTCAAATGGTATCAGAAGGTTTTGGAGTCATTCTGGAAATTCCATTCTCGTCGCAATTAGTATCT---------TCCCTTGAAG---ATAACAGAATACCAAAATTTCAGAATTTACTATCTATTCATTCAATATTTCCCTTTTTAGAGGATAAATTATCACATTTCAATTATGTGTCAG-ATC-TACT-AATACCCCATCCCATCCATC-TGGAAATCTT-GGTTCAAATCCTTCAATGCTGG-ATTAAAGATGTTTCTTCT-TTGCATTTCTTG-CGATTGTT--TTTCCACG------AATATCATAATT---------------TGAATAGTCTCAT---TACT-TC---------------AAATAAATCCATTTACGTCT-TTTCAAAAAG---AACCAAAAGAT-TCT-TTTGG-TTCCTACATA------ATTCTTAT-GTATAT-GAATGC----GAATATATAT-TCCTG---TTTCTTCGT--AAACAG-TCTTCT-TATT-TACG-AT-CAATAT-CTTCTGGA-GTCTTTCTT-GAGCG-AACACATTT--CTATGGAAA--AATAGAA------TATCTTATAGT---CGTGTGTT-GTAATT------CTTTTCA---------GAGG-ATCCTATGGT-TCCTCAA-AGATACT-TTCA-TACATTAT------------GTTC-----GATATCAAGGAAAA---ACA-----ATTCT--GGTTTC-----AAAAG---GAACTCTT---------ATTC------------TGATTAAGAAAT-GGAAATTT--CATCTT-GTGAA-TTTTTGGCA-ATC--TTATT-TTCACTTT-TGGTTTC--AACCTTATAGGA----TTCATATAAA-GCAATT--ACCCAACTATTCCTTCTCTTTTCTGGGG-TATTTTTCAAGTGTACTAAAAAA-TAATTTGGT-AATAA-GAAATC-AAATGCTAGAGAATTCATTT-CTAATAAATACTCT-GACTAAGA-AATTAGATACCATA-GCCCCAGTT--ACTTCTATTATTGG-ATCATTGTCGAAAGCTCA-ATTTTGTACTGTATTGGGT-CATCCTATTAGTAAACC-GATCTGGACCGATTTATCGGATTCTGA-------------TATTTTTGATCGATTTTGTCGGAT-----ATGTAGAAAT---CTTTGTCGT-TATCACAGCGGATCTTC-AAAGAA--------ACAGGTTTTGTATCGTATAAAGTA-TATACTTCGACTTTCGTGTGCTAG-AACTTTGGCTCGTAAACATAAAAGTACAGTACGCACTTTTATGCGAAGATTAGGTTCGGGATTCTTAG-AAGAATTTTT---------------------------------

Arundina_graminifolia_SG1008 ACAAGAATTCTTTTTCTTCTCATTTTTCT------------TCTCAAATGGTATCAGAAGGTTTTGGAGTCATTCTGGAAATTCCATTCTCGTCGCAATTAGTATCT---------TCCCTTGAAG---ATAACAGAATACCAAAATTTCAGAATTTACTATCTATTCATTCAATATTTCCCTTTTTAGAGGATAAATTATCACATTTCAATTATGTGTCAG-ATC-TACT-AATACCCCATCCCATCCATC-TGGAAATCTT-GGTTCAAATCCTTCAATGCTGG-ATTAAAGATGTTTCTTCT-TTGCATTTCTTG-CGATTGTT--TTTCCACG------AATATCATAATT---------------TGAATAGTCTCAT---TACT-TC---------------AAATAAATCCATTTACGTCT-TTTCAAAAAG---AACCAAAAGAT-TCT-TTTGG-TTCCTACATA------ATTCTTAT-GTATAT-GAATGC----GAATATATAT-TCCTG---TTTCTTCGT--AAACAG-TCTTCT-TATT-TACG-AT-CAATAT-CTTCTGGA-GTCTTTCTT-GAGCG-AACACATTT--CTATGGAAA--AATAGAA------TATCTTATAGT---CGTGTGTT-GTAATT------CTTTTCA---------GAGG-ATCCTATGGT-TCCTCAA-AGATACT-TTCA-TACATTAT------------GTTC-----GATATCAAGGAAAA---ACA-----ATTCT--GGTTTC-----AAAAG---GAACTCTT---------ATTC------------TGATTAAGAAAT-GGAAATTT--CATCTT-GTGAA-TTTTTGGCA-ATC--TTATT-TTCACTTT-TGGTTTC--AACCTTATAGGA----TTCATATAAA-GCAATT--ACCCAACTATTCCTTCTCTTTTCTGGGG-TATTTTTCAAGTGTACTAAAAAA-TAATTTGGT-AATAA-GAAATC-AAATGCTAGAGAATTCATTT-CTAATAAATACTCT-GACTAAGA-AATTAGATACCATA-GCCCCAGTT--ACTTCTATTATTGG-ATCATTGTCGAAAGCTCA-ATTTTGTACTGTATTGGGT-CATCCTATTAGTAAACC-GATCTGGACCGATTTATCGGATTCTGA-------------TATTTTTGATCGATTTTGTCGGAT-----ATGTAGAAAT---CTTTGTCGT-TATCACAGCGGATCTTC-AAAGAA--------ACAGGTTTTGTATCGTATAAAGTA-TATACTTCGACTTTCGTGTGCTAG-AACTTTGGCTCGTAAACATAAAAGTACAGTACGCACTTTTATGCGAAGATTAGGTTCGGGATTCTTAG-AAGAATTTTTTTT------TGAAGAAGAAC-AATCTCTTTCTT

Arundina_graminifolia_SG1206 ACAAGAATTCTTTTTCTTCTCATTTTTCT------------TCTCAAATGGTATCAGAAGGTTTTGGAGTCATTCTGGAAATTCCATTCTCGTCGCAATTAGTATCT---------TCCCTTGAAG---ATAACAGAATACCAAAATTTCAGAATTTACTATCTATTCATTCAATATTTCCCTTTTTAGAGGATAAATTATCACATTTCAATTATGTGTCAG-ATC-TACT-AATACCCCATCCCATCCATC-TGGAAATCTT-GGTTCAAATCCTTCAATGCTGG-ATTAAAGATGTTTCTTCT-TTGCATTTCTTG-CGATTGTT--TTTCCACG------AATATCATAATT---------------TGAATAGTCTCAT---TACT-TC---------------AAATAAATCCATTTACGTCT-TTTCAAAAAG---AACCAAAAGAT-TCT-TTTGG-TTCCTACATA------ACTCTTAT-GTATAT-GAATGC----GAATATATAT-TCCTG---TTTCTTCGT--AAACAG-TCTTCT-TATT-TACG-AT-CAATAT-CTTCTGGA-GTCTTTCTT-GAGCG-AACACATTT--CTATGGAAA--AATAGAA------TATCTTATAGT---CGTGTGTT-GTAATT------CTTTTCA---------GAGG-ATCCTATGGT-TCCTCAA-AGATACT-TTCA-TACATTAT------------GTTC-----GATATCAAGGAAAA---ACA-----ATTCT--GGTTTC-----AAAAG---GAACTCTT---------ATTC------------TGATTAAGAAAT-GGAAATTT--CATCTT-GTGAA-TTTTTGGCA-ATC--TTATT-TTCACTTT-TGGTTTC--AACCTTATAGGA----TTCATATAAA-GCAATT--ACCCAACTATTCCTTCTCTTTTCTGGGG-TATTTTTCAAGTGTACTAAAAAA-TAATTTGGT-AATAA-GAAATC-AAATGCTAGAGAATTCATTT-CTAATAAATACTCT-GACTAAGA-AATTAGATACCATA-GCCCCAGTT--ACTTCTATTATTGG-ATCATTGTCGAAAGCTCA-ATTTTGTACTGTATTGGGT-CATCCTATTAGTAAACC-GATCTGGACCGATTTATCGGATTCTGA-------------TATTTTTGATCGATTTTGTCGGAT-----ATGTAGAAAT---CTTTGTCGT-TATCACAGCGGATCTTC-AAAGAA--------ACAGGTTTTGTATCGTATAAAGTA-TATACTTCGACTTTCGTGTGCTAG-AACTTTGGCTCGTAAACATAAAAGTACAGTACGCACTTTTATGCGAAGATTAGGTTCGGGATTCTTAG-AAGAATTTTTTTT------TGAAGAAGAAC-AATCTCTTTCTT

Arundina_graminifolia_SG1295 ---AGAATTCTTTTTCTTCTCATTTTTCT------------TCTCAAATGGTATCAGAAGGTTTTGGAGTCATTCTGGAAATTCCATTCTCGTCGCAATTAGTATCT---------TCCCTTGAAG---ATAACAGAATACCAAAATTTCAGAATTTACTATCTATTCATTCAATATTTCCCTTTTTAGAGGATAAATTATCACATTTCAATTATGTGTCAG-ATC-TACT-AATACCCCATCCCATCCATC-TGGAAATCTT-GGTTCAAATCCTTCAATGCTGG-ATTAAAGATGTTTCTTCT-TTGCATTTCTTG-CGATTGTT--TTTCCACG------AATATCATAATT---------------TGAATAGTCTCAT---TACT-TC---------------AAATAAATCCATTTACGTCT-TTTCAAAAAG---AACCAAAAGAT-TCT-TTTGG-TTCCTACATA------ACTCTTAT-GTATAT-GAATGC----GAATATATAT-TCCTG---TTTCTTCGT--AAACAG-TCTTCT-TATT-TACG-AT-CAATAT-CTTCTGGA-GTCTTTCTT-GAGCG-AACACATTT--CTATGGAAA--AATAGAA------TATCTTATAGT---CGTGTGTT-GTAATT------CTTTTCA---------GAGG-ATCCTATGGT-TCCTCAA-AGATACT-TTCA-TACATTAT------------GTTC-----GATATCAAGGAAAA---ACA-----ATTCT--GGTTTC-----AAAAG---GAACTCTT---------ATTC------------TGATTAAGAAAT-GGAAATTT--CATCTT-GTGAA-TTTTTGGCA-ATC--TTATT-TTCACTTT-TGGTTTC--AACCTTATAGGA----TTCATATAAA-GCAATT--ACCCAACTATTCCTTCTCTTTTCTGGGG-TATTTTTCAAGTGTACTAAAAAA-TAATTTGGT-AATAA-GAAATC-AAATGCTAGAGAATTCATTT-CTAATAAATACTCT-GACTAAGA-AATTAGATACCATA-GCCCCAGTT--ACTTCTATTATTGG-ATCATTGTCGAAAGCTCA-ATTTTGTACTGTATTGGGT-CATCCTATTAGTAAACC-GATCTGGACCGATTTATCGGATTCTGA-------------TATTTTTGATCGATTTTGTCGGAT-----ATGTAGAAAT---CTTTGTCGT-TATCACAGCGGATCTTC-AAAGAA--------ACAGGTTTTGTATCGTATAAAGTA-TATACTTCGACTTTCGTGTGCTAG-AACTTTGGCTCGTAAACATAAAAGTACAGTACGCACTTTTATGCGAAGATTAGGTTCGGGATTCTTAG-AAGAATTTTTTTT------TGAAGAAGAAC-AATCTCTTTCTT

Arundina_graminifolia_var_graminifolia_AB844195 ACAAGAATTCTTTTTCTTCTCATTTTTCT------------TCTCAAATGGTATCAGAAGGTTTTGGAGTCATTCTGGAAATTCCATTCTCGTCGCAATTAGTATCT---------TCCCTTGAAG---ATAACAGAATACCAAAATTTCAGAATTTACTATCTATTCATTCAATATTTCCCTTTTTAGAGGATAAATTATCACATTTCAATTATGTGTCAG-ATC-TACT-AATACCCCATCCCATCCATC-TGGAAATCTT-GGTTCAAATCCTTCAATGCTGG-ATTAAAGATGTTTCTTCT-TTGCATTTCTTG-CGATTGTT--TTTCCACG------AATATCATAATT---------------TGAATAGTCTCAT---TACT-TC---------------AAATAAATCCATTTACGTCT-TTTCAAAAAG---AACCAAAAGAA-TCT-TTTGG-TTCCTACATA------ATTCTTAT-GTATAT-GAATGC----GAATATATAT-TCCTG---TTTCTTCGT--AAACAG-TCTTCT-TATT-TACG-AT-CAATAT-CTTCTGGA-GTCTTTCTT-GAGCG-AACACATTT--CTATGGAAA--AATAGAA------TATCTTATAGT---CGTGTGTT-GTAATT------CTTTTCA---------GAGG-ATCCTATGGT-TCCTCAA-AGATACT-TTCA-TACATTAT------------GTTC-----GATATCAAGGAAAA---ACA-----ATTCT--GGTTTC-----AAAAG---GAACTCTT---------ATTC------------TGATTAAGAAAT-GGAAATTT--CATCTT-GTGAA-TTTTTGGCA-ATC--TTATT-TTCACTTT-TGGTTTC--AACCTTATAGGA----TTCATATAAA-GCAATT--ACCCAACTATTCCTTCTCTTTTCTGGGG-TATTTTTCAAGTGTACTAAAAAA-TAATTTGGT-AATAA-GAAATC-AAATGCTAGAGAATTCATTT-CTAATAAATACTCT-GACTAAGA-AATTAGATACCATA-GCCCCAGTT--ACTTCTATTATTGG-ATCATTGTCGAAAGCTCA-ATTTTGTACTGTATTGGGT-CATCCTATTAGTAAACC-GATCTGGACCGATTTATCGGATTCTGA-------------TATTTTTGATCGATTTTGTCGGAT-----ATGTAGAAAT---CTTTGTCGT-TATCACAGCGGATCTTC-AAAGAA--------ACAGGTTTTGTATCGTATAAAGTA-TATACTTCGACTTTCGTGTGCTAG-AACTTTGGCTCGTAAACATAAAAGTACAGTACGCACTTTTATGCGAAGATTAGGTTCGGGATTCTTAG-AAGAATTTTTTTT------TGAAGAAGAAC-AATCTCTTTCTT

Arundina_graminifolia_var_graminifolia_AB844196 ACAAGAATTCTTTTTCTTCTCATTTTTCT------------TCTCAAATGGTATCAGAAGGTTTTGGAGTCATTCTGGAAATTCCATTCTCGTCGCAATTAGTATCT---------TCCCTTGAAG---ATAACAGAATACCAAAATTTCAGAATTTACTATCTATTCATTCAATATTTCCCTTTTTAGAGGATAAATTATCACATTTCAATTATGTGTCAG-ATC-TACT-AATACCCCATCCCATCCATC-TGGAAATCTT-GGTTCAAATCCTTCAATGCTGG-ATTAAAGATGTTTCTTCT-TTGCATTTCTTG-CGATTGTT--TTTCCACG------AATATCATAATT---------------TGAATAGTCTCAT---TACT-TC---------------AAATAAATCCATTTACGTCT-TTTCAAAAAG---AACCAAAAGAA-TCT-TTTGG-TTCCTACATA------ATTCTTAT-GTATAT-GAATGC----GAATATATAT-TCCTG---TTTCTTCGT--AAACAG-TCTTCT-TATT-TACG-AT-CAATAT-CTTCTGGA-GTCTTTCTT-GAGCG-AACACATTT--CTATGGAAA--AATAGAA------TATCTTATAGT---CGTGTGTT-GTAATT------CTTTTCA---------GAGG-ATCCTATGGT-TCCTCAA-AGATACT-TTCA-TACATTAT------------GTTC-----GATATCAAGGAAAA---ACA-----ATTCT--GGTTTC-----AAAAG---GAACTCTT---------ATTC------------TGATTAAGAAAT-GGAAATTT--CATCTT-GTGAA-TTTTTGGCA-ATC--TTATT-TTCACTTT-TGGTTTC--AACCTTATAGGA----TTCATATAAA-GCAATT--ACCCAACTATTCCTTCTCTTTTCTGGGG-TATTTTTCAAGTGTACTAAAAAA-TAATTTGGT-AATAA-GAAATC-AAATGCTAGAGAATTCATTT-CTAATAAATACTCT-GACTAAGA-AATTAGATACCATA-GCCCCAGTT--ACTTCTATTATTGG-ATCATTGTCGAAAGCTCA-ATTTTGTACTGTATTGGGT-CATCCTATTAGTAAACC-GATCTGGACCGATTTATCGGATTCTGA-------------TATTTTTGATCGATTTTGTCGGAT-----ATGTAGAAAT---CTTTGTCGT-TATCACAGCGGATCTTC-AAAGAA--------ACAGGTTTTGTATCGTATAAAGTA-TATACTTCGACTTTCGTGTGCTAG-AACTTTGGCTCGTAAACATAAAAGTACAGTACGCACTTTTATGCGAAGATTAGGTTCGGGATTCTTAG-AAGAATTTTTTTT------TGAAGAAGAAC-AATCTCTTTCTT

Arundina_graminifolia_var_graminifolia_AB844197 ACAAGAATTCTTTTTCTTCTCATTTTTCT------------TCTCAAATGGTATCAGAAGGTTTTGGAGTCATTCTGGAAATTCCATTCTCGTCGCAATTAGTATCT---------TCCCTTGAAG---ATAACAGAATACCAAAATTTCAGAATTTACTATCTATTCATTCAATATTTCCCTTTTTAGAGGATAAATTATCACATTTCAATTATGTGTCAG-ATC-TACT-AATACCCCATCCCATCCATC-TGGAAATCTT-GGTTCAAATCCTTCAATGCTGG-ATTAAAGATGTTTCTTCT-TTGCATTTCTTG-CGATTGTT--TTTCCACG------AATATCATAATT---------------TGAATAGTCTCAT---TACT-TC---------------AAATAAATCCATTTACGTCT-TTTCAAAAAG---AACCAAAAGAA-TCT-TTTGG-TTCCTACATA------ATTCTTAT-GTATAT-GAATGC----GAATATATAT-TCCTG---TTTCTTCGT--AAACAG-TCTTCT-TATT-TACG-AT-CAATAT-CTTCTGGA-GTCTTTCTT-GAGCG-AACACATTT--CTATGGAAA--AATAGAA------TATCTTATAGT---CGTGTGTT-GTAATT------CTTTTCA---------GAGG-ATCCTATGGT-TCCTCAA-AGATACT-TTCA-TACATTAT------------GTTC-----GATATCAAGGAAAA---ACA-----ATTCT--GGTTTC-----AAAAG---GAACTCTT---------ATTC------------TGATTAAGAAAT-GGAAATTT--CATCTT-GTGAA-TTTTTGGCA-ATC--TTATT-TTCACTTT-TGGTTTC--AACCTTATAGGA----TTCATATAAA-GCAATT--ACCCAACTATTCCTTCTCTTTTCTGGGG-TATTTTTCAAGTGTACTAAAAAA-TAATTTGGT-AATAA-GAAATC-AAATGCTAGAGAATTCATTT-CTAATAAATACTCT-GACTAAGA-AATTAGATACCATA-GCCCCAGTT--ACTTCTATTATTGG-ATCATTGTCGAAAGCTCA-ATTTTGTACTGTATTGGGT-CATCCTATTAGTAAACC-GATCTGGACCGATTTATCGGATTCTGA-------------TATTTTTGATCGATTTTGTCGGAT-----ATGTAGAAAT---CTTTGTCGT-TATCACAGCGGATCTTC-AAAGAA--------ACAGGTTTTGTATCGTATAAAGTA-TATACTTCGACTTTCGTGTGCTAG-AACTTTGGCTCGTAAACATAAAAGTACAGTACGCACTTTTATGCGAAGATTAGGTTCGGGATTCTTAG-AAGAATTTTTTTT------TGAAGAAGAAC-AATCTCTTTCTT

Arundina_graminifolia_var_graminifolia_AB844198 ACAAGAATTCTTTTTCTTCTCATTTTTCT------------TCTCAAATGGTATCAGAAGGTTTTGGAGTCATTCTGGAAATTCCATTCTCGTCGCAATTAGTATCT---------TCCCTTGAAG---ATAACAGAATACCAAAATTTCAGAATTTACTATCTATTCATTCAATATTTCCCTTTTTAGAGGATAAATTATCACATTTCAATTATGTGTCAG-ATC-TACT-AATACCCCATCCCATCCATC-TGGAAATCTT-GGTTCAAATCCTTCAATGCTGG-ATTAAAGATGTTTCTTCT-TTGCATTTCTTG-CGATTGTT--TTTCCACG------AATATCATAATT---------------TGAATAGTCTCAT---TACT-TC---------------AAATAAATCCATTTACGTCT-TTTCAAAAAG---AACCAAAAGAT-TCT-TTTGG-TTCCTACATA------ATTCTTAT-GTATAT-GAATGC----GAATATATAT-TCCTG---TTTCTTCGT--AAACAG-TCTTCT-TATT-TACG-AT-CAATAT-CTTCTGGA-GTCTTTCTT-GAGCG-AACACATTT--CTATGGAAA--AATAGAA------TATCTTATAGT---CGTGTGTT-GTAATT------CTTTTCA---------GAGG-ATCCTATGGT-TCCTCAA-AGATACT-TTCA-TACATTAT------------GTTC-----GATATCAAGGAAAA---ACA-----ATTCT--GGTTTC-----AAAAG---GAACTCTT---------ATTC------------TGATTAAGAAAT-GGAAATTT--CATCTT-GTGAA-TTTTTGGCA-ATC--TTATT-TTCACTTT-TGGTTTC--AACCTTATAGGA----TTCATATAAA-GCAATT--ACCCAACTATTCCTTCTCTTTTCTGGGG-TATTTTTCAAGTGTACTAAAAAA-TAATTTGGT-AATAA-GAAATC-AAATGCTAGAGAATTCATTT-CTAATAAATACTCT-GACTAAGA-AATTAGATACCATA-GCCCCAGTT--ACTTCTATTATTGG-ATCATTGTCGAAAGCTCA-ATTTTGTACTGTATTGGGT-CATCCTATTAGTAAACC-GATCTGGACCGATTTATCGGATTCTGA-------------TATTTTTGATCGATTTTGTCGGAT-----ATGTAGAAAT---CTTTGTCGT-TATCACAGCGGATCTTC-AAAGAA--------ACAGGTTTTGTATCGTATAAAGTA-TATACTTCGACTTTCGTGTGCTAG-AACTTTGGCTCGTAAACATAAAAGTACAGTACGCACTTTTATGCGAAGATTAGGTTCGGGATTCTTAG-AAGAATTTTTTTT------TGAAGAAGAAC-AATCTCTTTCTT

Arundina_graminifolia_var_graminifolia_AB844199 ACAAGAATTCTTTTTCTTCTCATTTTTCT------------TCTCAAATGGTATCAGAAGGTTTTGGAGTCATTCTGGAAATTCCATTCTCGTCGCAATTAGTATCT---------TCCCTTGAAG---ATAACAGAATACCAAAATTTCAGAATTTACTATCTATTCATTCAATATTTCCCTTTTTAGAGGATAAATTATCACATTTCAATTATGTGTCAG-ATC-TACT-AATACCCCATCCCATCCATC-TGGAAATCTT-GGTTCAAATCCTTCAATGCTGG-ATTAAAGATGTTTCTTCT-TTGCATTTCTTG-CGATTGTT--TTTCCACG------AATATCATAATT---------------TGAATAGTCTCAT---TACT-TC---------------AAATAAATCCATTTACGTCT-TTTCAAAAAG---AACCAAAAGAT-TCT-TTTGG-TTCCTACATA------ACTCTTAT-GTATAT-GAATGC----GAATATATAT-TCCTG---TTTCTTCGT--AAACAG-TCTTCT-TATT-TACG-AT-CAATAT-CTTCTGGA-GTCTTTCTT-GAGCG-AACACATTT--CTATGGAAA--AATAGAA------TATCTTATAGT---CGTGTGTT-GTAATT------CTTTTCA---------GAGG-ATCCTATGGT-TCCTCAA-AGATACT-TTCA-TACATTAT------------GTTC-----GATATCAAGGAAAA---ACA-----ATTCT--GGTTTC-----AAAAG---GAACTCTT---------ATTC------------TGATTAAGAAAT-GGAAATTT--CATCTT-GTGAA-TTTTTGGCA-ATC--TTATT-TTCACTTT-TGGTTTC--AACCTTATAGGA----TTCATATAAA-GCAATT--ACCCAACTATTCCTTCTCTTTTCTGGGG-TATTTTTCAAGTGTACTAAAAAA-TAATTTGGT-AATAA-GAAATC-AAATGCTAGAGAATTCATTT-CTAATAAATACTCT-GACTAAGA-AATTAGATACCATA-GCCCCAGTT--ACTTCTATTATTGG-ATCATTGTCGAAAGCTCA-ATTTTGTACTGTATTGGGT-CATCCTATTAGTAAACC-GATCTGGACCGATTTATCGGATTCTGA-------------TATTTTTGATCGATTTTGTCGGAT-----ATGTAGAAAT---CTTTGTCGT-TATCACAGCGGATCTTC-AAAGAA--------ACAGGTTTTGTATCGTATAAAGTA-TATACTTCGACTTTCGTGTGCTAG-AACTTTGGCTCGTAAACATAAAAGTACAGTACGCACTTTTATGCGAAGATTAGGTTCGGGATTCTTAG-AAGAATTTTTTTT------TGAAGAAGAAC-AATCTCTTTCTT

Arundina_graminifolia_var_graminifolia_AB844200 ACAAGAATTCTTTTTCTTCTCATTTTTCT------------TCTCAAATGGTATCAGAAGGTTTTGGAGTCATTCTGGAAATTCCATTCTCGTCGCAATTAGTATCT---------TCCCTTGAAG---ATAACAGAATACCAAAATTTCAGAATTTACTATCTATTCATTCAATATTTCCCTTTTTAGAGGATAAATTATCACATTTCAATTATGTGTCAG-ATC-TACT-AATACCCCATCCCATCCATC-TGGAAATCTT-GGTTCAAATCCTTCAATGCTGG-ATTAAAGATGTTTCTTCT-TTGCATTTCTTG-CGATTGTT--TTTCCACG------AATATCATAATT---------------TGAATAGTCTCAT---TACT-TC---------------AAATAAATCCATTTACGTCT-TTTCAAAAAG---AACCAAAAGAT-TCT-TTTGG-TTCCTACATA------ACTCTTAT-GTATAT-GAATGC----GAATATATAT-TCCTG---TTTCTTCGT--AAACAG-TCTTCT-TATT-TACG-AT-CAATAT-CTTCTGGA-GTCTTTCTT-GAGCG-AACACATTT--CTATGGAAA--AATAGAA------TATCTTATAGT---CGTGTGTT-GTAATT------CTTTTCA---------GAGG-ATCCTATGGT-TCCTCAA-AGATACT-TTCA-TACATTAT------------GTTC-----GATATCAAGGAAAA---ACA-----ATTCT--GGTTTC-----AAAAG---GAACTCTT---------ATTC------------TGATTAAGAAAT-GGAAATTT--CATCTT-GTGAA-TTTTTGGCA-ATC--TTATT-TTCACTTT-TGGTTTC--AACCTTATAGGA----TTCATATAAA-GCAATT--ACCCAACTATTCCTTCTCTTTTCTGGGG-TATTTTTCAAGTGTACTAAAAAA-TAATTTGGT-AATAA-GAAATC-AAATGCTAGAGAATTCATTT-CTAATAAATACTCT-GACTAAGA-AATTAGATACCATA-GCCCCAGTT--ACTTCTATTATTGG-ATCATTGTCGAAAGCTCA-ATTTTGTACTGTATTGGGT-CATCCTATTAGTAAACC-GATCTGGACCGATTTATCGGATTCTGA-------------TATTTTTGATCGATTTTGTCGGAT-----ATGTAGAAAT---CTTTGTCGT-TATCACAGCGGATCTTC-AAAGAA--------ACAGGTTTTGTATCGTATAAAGTA-TATACTTCGACTTTCGTGTGCTAG-AACTTTGGCTCGTAAACATAAAAGTACAGTACGCACTTTTATGCGAAGATTAGGTTCGGGATTCTTAG-AAGAATTTTTTTT------TGAAGAAGAAC-AATCTCTTTCTT

Arundina_graminifolia_var_graminifolia_AB872252 ACAAGAATTCTTTTTCTTCTCATTTTTCT------------TCTCAAATGGTATCAGAAGGTTTTGGAGTCATTCTGGAAATTCCATTCTCGTCGCAATTAGTATCT---------TCCCTTGAAG---ATAACAGAATACCAAAATTTCAGAATTTACTATCTATTCATTCAATATTTCCCTTTTTAGAGGATAAATTATCACATTTCAATTATGTGTCAG-ATC-TACT-AATACCCCATCCCATCCATC-TGGAAATCTT-GGTTCAAATCCTTCAATGCTGG-ATTAAAGATGTTTCTTCT-TTGCATTTCTTG-CGATTGTT--TTTCCACG------AATATCATAATT---------------TGAATAGTCTCAT---TACT-TC---------------AAATAAATCCATTTACGTCT-TTTCAAAAAG---AACCAAAAGAA-TCT-TTTGG-TTCCTACATA------ATTCTTAT-GTATAT-GAATGC----GAATATATAT-TCCTG---TTTCTTCGT--AAACAG-TCTTCT-TATT-TACG-AT-CAATAT-CTTCTGGA-GTCTTTCTT-GAGCG-AACACATTT--CTATGGAAA--AATAGAA------TATCTTATAGT---CGTGTGTT-GTAATT------CTTTTCA---------GAGG-ATCCTATGGT-TCCTCAA-AGATACT-TTCA-TACATTAT------------GTTC-----GATATCAAGGAAAA---ACA-----ATTCT--GGTTTC-----AAAAG---GAACTCTT---------ATTC------------TGATTAAGAAAT-GGAAATTT--CATCTT-GTGAA-TTTTTGGCA-ATC--TTATT-TTCACTTT-TGGTTTC--AACCTTATAGGA----TTCATATAAA-GCAATT--ACCCAACTATTCCTTCTCTTTTCTGGGG-TATTTTTCAAGTGTACTAAAAAA-TAATTTGGT-AATAA-GAAATC-AAATGCTAGAGAATTCATTT-CTAATAAATACTCT-GACTAAGA-AATTAGATACCATA-GCCCCAGTT--ACTTCTATTATTGG-ATCATTGTCGAAAGCTCA-ATTTTGTACTGTATTGGGT-CATCCTATTAGTAAACC-GATCTGGACCGATTTATCGGATTCTGA-------------TATTTTTGATCGATTTTGTCGGAT-----ATGTAGAAAT---CTTTGTCGT-TATCACAGCGGATCTTC-AAAGAA--------ACAGGTTTTGTATCGTATAAAGTA-TATACTTCGACTTTCGTGTGCTAG-AACTTTGGCTCGTAAACATAAAAGTACAGTACGCACTTTTATGCGAAGATTAGGTTCGGGATTCTTAG-AAGAATTTTTTTT------TGAAGAAGAAC-AATCTCTTTCTT

Arundina_graminifolia_var_revoluta_AB844201 ACAAGAATTCTTTTTCTTCTCATTTTTCT------------TCTCAAATGGTATCAGAAGGTTTTGGAGTCATTCTGGAAATTCCATTCTCGTCGCAATTAGTATCT---------TCCCTTGAAG---ATAACAGAATACCAAAATTTCAGAATTTACTATCTATTCATTCAATATTTCCCTTTTTAGAGGATAAATTATCACATTTCAATTATGTGTCAG-ATC-TACT-AATACCCCATCCCATCCATC-TGGAAATCTT-GGTTCAAATCCTTCAATGCTGG-ATTAAAGATGTTTCTTCT-TTGCATTTCTTG-CGATTGTT--TTTCCACG------AATATCATAATT---------------TGAATAGTCTCAT---TACT-TC---------------AAATAAATCCATTTACGTCT-TTTCAAAAAG---AACCAAAAGAA-TCT-TTTGG-TTCCTACATA------ATTCTTAT-GTATAT-GAATGC----GAATATATAT-TCCTG---TTTCTTCGT--AAACAG-TCTTCT-TATT-TACG-AT-CAATAT-CTTCTGGA-GTCTTTCTT-GAGCG-AACACATTT--CTATGGAAA--AATAGAA------TATCTTATAGT---CGTGTGTT-GTAATT------CTTTTCA---------GAGG-ATCCTATGGT-TCCTCAA-AGATACT-TTCA-TACATTAT------------GTTC-----GATATCAAGGAAAA---ACA-----ATTCT--GGTTTC-----AAAAG---GAACTCTT---------ATTC------------TGATTAAGAAAT-GGAAATTT--CATCTT-GTGAA-TTTTTGGCA-ATC--TTATT-TTCACTTT-TGGTTTC--AACCTTATAGGA----TTCATATAAA-GCAATT--ACCCAACTATTCCTTCTCTTTTCTGGGG-TATTTTTCAAGTGTACTAAAAAA-TAATTTGGT-AATAA-GAAATC-AAATGCTAGAGAATTCATTT-CTAATAAATACTCT-GACTAAGA-AATTAGATACCATA-GCCCCAGTT--ACTTCTATTATTGG-ATCATTGTCGAAAGCTCA-ATTTTGTACTGTATTGGGT-CATCCTATTAGTAAACC-GATCTGGACCGATTTATCGGATTCTGA-------------TATTTTTGATCGATTTTGTCGGAT-----ATGTAGAAAT---CTTTGTCGT-TATCACAGCGGATCTTC-AAAGAA--------ACAGGTTTTGTATCGTATAAAGTA-TATACTTCGACTTTCGTGTGCTAG-AACTTTGGCTCGTAAACATAAAAGTACAGTACGCACTTTTATGCGAAGATTAGGTTCGGGATTCTTAG-AAGAATTTTTTTT------TGAAGAAGAAC-AATCTCTTTCTT

Arundina_graminifolia_var_revoluta_AB844202 ACAAGAATTCTTTTTCTTCTCATTTTTCT------------TCTCAAATGGTATCAGAAGGTTTTGGAGTCATTCTGGAAATTCCATTCTCGTCGCAATTAGTATCT---------TCCCTTGAAG---ATAACAGAATACCAAAATTTCAGAATTTACTATCTATTCATTCAATATTTCCCTTTTTAGAGGATAAATTATCACATTTCAATTATGTGTCAG-ATC-TACT-AATACCCCATCCCATCCATC-TGGAAATCTT-GGTTCAAATCCTTCAATGCTGG-ATTAAAGATGTTTCTTCT-TTGCATTTCTTG-CGATTGTT--TTTCCACG------AATATCATAATT---------------TGAATAGTCTCAT---TACT-TC---------------AAATAAATCCATTTACGTCT-TTTCAAAAAG---AACCAAAAGAA-TCT-TTTGG-TTCCTACATA------ATTCTTAT-GTATAT-GAATGC----GAATATATAT-TCCTG---TTTCTTCGT--AAACAG-TCTTCT-TATT-TACG-AT-CAATAT-CTTCTGGA-GTCTTTCTT-GAGCG-AACACATTT--CTATGGAAA--AATAGAA------TATCTTATAGT---CGTGTGTT-GTAATT------CTTTTCA---------GAGG-ATCCTATGGT-TCCTCAA-AGATACT-TTCA-TACATTAT------------GTTC-----GATATCAAGGAAAA---ACA-----ATTCT--GGTTTC-----AAAAG---GAACTCTT---------ATTC------------TGATTAAGAAAT-GGAAATTT--CATCTT-GTGAA-TTTTTGGCA-ATC--TTATT-TTCACTTT-TGGTTTC--AACCTTATAGGA----TTCATATAAA-GCAATT--ACCCAACTATTCCTTCTCTTTTCTGGGG-TATTTTTCAAGTGTACTAAAAAA-TAATTTGGT-AATAA-GAAATC-AAATGCTAGAGAATTCATTT-CTAATAAATACTCT-GACTAAGA-AATTAGATACCATA-GCCCCAGTT--ACTTCTATTATTGG-ATCATTGTCGAAAGCTCA-ATTTTGTACTGTATTGGGT-CATCCTATTAGTAAACC-GATCTGGACCGATTTATCGGATTCTGA-------------TATTTTTGATCGATTTTGTCGGAT-----ATGTAGAAAT---CTTTGTCGT-TATCACAGCGGATCTTC-AAAGAA--------ACAGGTTTTGTATCGTATAAAGTA-TATACTTCGACTTTCGTGTGCTAG-AACTTTGGCTCGTAAACATAAAAGTACAGTACGCACTTTTATGCGAAGATTAGGTTCGGGATTCTTAG-AAGAATTTTTTTT------TGAAGAAGAAC-AATCTCTTTCTT

Arundina_graminifolia_var_revoluta_AB844203 ACAAGAATTCTTTTTCTTCTCATTTTTCT------------TCTCAAATGGTATCAGAAGGTTTTGGAGTCATTCTGGAAATTCCATTCTCGTCGCAATTAGTATCT---------TCCCTTGAAG---ATAACAGAATACCAAAATTTCAGAATTTACTATCTATTCATTCAATATTTCCCTTTTTAGAGGATAAATTATCACATTTCAATTATGTGTCAG-ATC-TACT-AATACCCCATCCCATCCATC-TGGAAATCTT-GGTTCAAATCCTTCAATGCTGG-ATTAAAGATGTTTCTTCT-TTGCATTTCTTG-CGATTGTT--TTTCCACG------AATATCATAATT---------------TGAATAGTCTCAT---TACT-TC---------------AAATAAATCCATTTACGTCT-TTTCAAAAAG---AACCAAAAGAA-TCT-TTTGG-TTCCTACATA------ATTCTTAT-GTATAT-GAATGC----GAATATATAT-TCCTG---TTTCTTCGT--AAACAG-TCTTCT-TATT-TACG-AT-CAATAT-CTTCTGGA-GTCTTTCTT-GAGCG-AACACATTT--CTATGGAAA--AATAGAA------TATCTTATAGT---CGTGTGTT-GTAATT------CTTTTCA---------GAGG-ATCCTATGGT-TCCTCAA-AGATACT-TTCA-TACATTAT------------GTTC-----GATATCAAGGAAAA---ACA-----ATTCT--GGTTTC-----AAAAG---GAACTCTT---------ATTC------------TGATTAAGAAAT-GGAAATTT--CATCTT-GTGAA-TTTTTGGCA-ATC--TTATT-TTCACTTT-TGGTTTC--AACCTTATAGGA----TTCATATAAA-GCAATT--ACCCAACTATTCCTTCTCTTTTCTGGGG-TATTTTTCAAGTGTACTAAAAAA-TAATTTGGT-AATAA-GAAATC-AAATGCTAGAGAATTCATTT-CTAATAAATACTCT-GACTAAGA-AATTAGATACCATA-GCCCCAGTT--ACTTCTATTATTGG-ATCATTGTCGAAAGCTCA-ATTTTGTACTGTATTGGGT-CATCCTATTAGTAAACC-GATCTGGACCGATTTATCGGATTCTGA-------------TATTTTTGATCGATTTTGTCGGAT-----ATGTAGAAAT---CTTTGTCGT-TATCACAGCGGATCTTC-AAAGAA--------ACAGGTTTTGTATCGTATAAAGTA-TATACTTCGACTTTCGTGTGCTAG-AACTTTGGCTCGTAAACATAAAAGTACAGTACGCACTTTTATGCGAAGATTAGGTTCGGGATTCTTAG-AAGAATTTTTTTT------TGAAGAAGAAC-AATCTCTTTCTT

Arundina_graminifolia_var_revoluta_AB844204 ACAAGAATTCTTTTTCTTCTCATTTTTCT------------TCTCAAATGGTATCAGAAGGTTTTGGAGTCATTCTGGAAATTCCATTCTCGTCGCAATTAGTATCT---------TCCCTTGAAG---ATAACAGAATACCAAAATTTCAGAATTTACTATCTATTCATTCAATATTTCCCTTTTTAGAGGATAAATTATCACATTTCAATTATGTGTCAG-ATC-TACT-AATACCCCATCCCATCCATC-TGGAAATCTT-GGTTCAAATCCTTCAATGCTGG-ATTAAAGATGTTTCTTCT-TTGCATTTCTTG-CGATTGTT--TTTCCACG------AATATCATAATT---------------TGAATAGTCTCAT---TACT-TC---------------AAATAAATCCATTTACGTCT-TTTCAAAAAG---AACCAAAAGAA-TCT-TTTGG-TTCCTACATA------ATTCTTAT-GTATAT-GAATGC----GAATATATAT-TCCTG---TTTCTTCGT--AAACAG-TCTTCT-TATT-TACG-AT-CAATAT-CTTCTGGA-GTCTTTCTT-GAGCG-AACACATTT--CTATGGAAA--AATAGAA------TATCTTATAGT---CGTGTGTT-GTAATT------CTTTTCA---------GAGG-ATCCTATGGT-TCCTCAA-AGATACT-TTCA-TACATTAT------------GTTC-----GATATCAAGGAAAA---ACA-----ATTCT--GGTTTC-----AAAAG---GAACTCTT---------ATTC------------TGATTAAGAAAT-GGAAATTT--CATCTT-GTGAA-TTTTTGGCA-ATC--TTATT-TTCACTTT-TGGTTTC--AACCTTATAGGA----TTCATATAAA-GCAATT--ACCCAACTATTCCTTCTCTTTTCTGGGG-TATTTTTCAAGTGTACTAAAAAA-TAATTTGGT-AATAA-GAAATC-AAATGCTAGAGAATTCATTT-CTAATAAATACTCT-GACTAAGA-AATTAGATACCATA-GCCCCAGTT--ACTTCTATTATTGG-ATCATTGTCGAAAGCTCA-ATTTTGTACTGTATTGGGT-CATCCTATTAGTAAACC-GATCTGGACCGATTTATCGGATTCTGA-------------TATTTTTGATCGATTTTGTCGGAT-----ATGTAGAAAT---CTTTGTCGT-TATCACAGCGGATCTTC-AAAGAA--------ACAGGTTTTGTATCGTATAAAGTA-TATACTTCGACTTTCGTGTGCTAG-AACTTTGGCTCGTAAACATAAAAGTACAGTACGCACTTTTATGCGAAGATTAGGTTCGGGATTCTTAG-AAGAATTTTTTTT------TGAAGAAGAAC-AATCTCTTTCTT

Arundina_graminifolia_var_revoluta_AB844205 ACAAGAATTCTTTTTCTTCTCATTTTTCT------------TCTCAAATGGTATCAGAAGGTTTTGGAGTCATTCTGGAAATTCCATTCTCGTCGCAATTAGTATCT---------TCCCTTGAAG---ATAACAGAATACCAAAATTTCAGAATTTACTATCTATTCATTCAATATTTCCCTTTTTAGAGGATAAATTATCACATTTCAATTATGTGTCAG-ATC-TACT-AATACCCCATCCCATCCATC-TGGAAATCTT-GGTTCAAATCCTTCAATGCTGG-ATTAAAGATGTTTCTTCT-TTGCATTTCTTG-CGATTGTT--TTTCCACG------AATATCATAATT---------------TGAATAGTCTTAT---TACT-TC---------------AAATAAATCCATTTACGTCT-TTTCAAAAAG---AACCAAAAGAT-TCT-TTTGG-TTCCTACATA------ATTCTTAT-GTATAT-GAATGC----GAATATATAT-TCCTG---TTTCTTCGT--AAACAG-TCTTCT-TATT-TACG-AT-CAATAT-CTTCTGGA-GTCTTTCTT-GAGCG-AACACATTT--CTATGGAAA--AATAGAA------TATCTTATAGT---CGTGTGTT-GTAATT------CTTTTCA---------GAGG-ATCCTATGGT-TCCTCAA-AGATACT-TTCA-TACATTAT------------GTTC-----GATATCAAGGAAAA---ACA-----ATTCT--GGTTTC-----AAAAG---GAACTCTT---------ATTC------------TGATTAAGAAAT-GGAAATTT--CATCTT-GTGAA-TTTTTGGCA-ATC--TTATT-TTCACTTT-TGGTTTC--AACCTTATAGGA----TTCATATAAA-GCAATT--ACCCAACTATTCCTTCTCTTTTCTGGGG-TATTTTTCAAGTGTACTAAAAAA-TAATTTGGT-AATAA-GAAATC-AAATGCTAGAGAATTCATTT-CTAATAAATACTCT-GACTAAGA-AATTAGATACCATA-GCCCCAGTT--ACTTCTATTATTGG-ATCATTGTCGAAAGCTCA-ATTTTGTACTGTATTGGGT-CATCCTATTAGTAAACC-GATCTGGACCGATTTATCGGATTCTGA-------------TATTTTTGATCGATTTTGTCGGAT-----ATGTAGAAAT---CTTTGTCGT-TATCACAGCGGATCTTC-AAAGAA--------ACAGGTTTTGTATCGTATAAAGTA-TATACTTCGACTTTCGTGTGCTAG-AACTTTGGCTCGTAAACATAAAAGTACAGTACGCACTTTTATGCGAAGATTAGGTTCGGGATTCTTAG-AAGAATTTTTTTTTGAATTTGAAGAAGAAC-AATCTCTTTCTT

Arundina_graminifolia_var_revoluta_AB844206 ACAAGAATTCTTTTTCTTCTCATTTTTCT------------TCTCAAATGGTATCAGAAGGTTTTGGAGTCATTCTGGAAATTCCATTCTCGTCGCAATTAGTATCT---------TCCCTTGAAG---ATAACAGAATACCAAAATTTCAGAATTTACTATCTATTCATTCAATATTTCCCTTTTTAGAGGATAAATTATCACATTTCAATTATGTGTCAG-ATC-TACT-AATACCCCATCCCATCCATC-TGGAAATCTT-GGTTCAAATCCTTCAATGCTGG-ATTAAAGATGTTTCTTCT-TTGCATTTCTTG-CGATTGTT--TTTCCACG------AATATCATAATT---------------TGAATAGTCTCAT---TACT-TC---------------AAATAAATCCATTTACGTCT-TTTCAAAAAG---AACCAAAAGAA-TCT-TTTGG-TTCCTACATA------ATTCTTAT-GTATAT-GAATGC----GAATATATAT-TCCTG---TTTCTTCGT--AAACAG-TCTTCT-TATT-TACG-AT-CAATAT-CTTCTGGA-GTCTTTCTT-GAGCG-AACACATTT--CTATGGAAA--AATAGAA------TATCTTATAGT---CGTGTGTT-GTAATT------CTTTTCA---------GAGG-ATCCTATGGT-TCCTCAA-AGATACT-TTCA-TACATTAT------------GTTC-----GATATCAAGGAAAA---ACA-----ATTCT--GGTTTC-----AAAAG---GAACTCTT---------ATTC------------TGATTAAGAAAT-GGAAATTT--CATCTT-GTGAA-TTTTTGGCA-ATC--TTATT-TTCACTTT-TGGTTTC--AACCTTATAGGA----TTCATATAAA-GCAATT--ACCCAACTATTCCTTCTCTTTTCTGGGG-TATTTTTCAAGTGTACTAAAAAA-TAATTTGGT-AATAA-GAAATC-AAATGCTAGAGAATTCATTT-CTAATAAATACTCT-GACTAAGA-AATTAGATACCATA-GCCCCAGTT--ACTTCTATTATTGG-ATCATTGTCGAAAGCTCA-ATTTTGTACTGTATTGGGT-CATCCTATTAGTAAACC-GATCTGGACCGATTTATCGGATTCTGA-------------TATTTTTGATCGATTTTGTCGGAT-----ATGTAGAAAT---CTTTGTCGT-TATCACAGCGGATCTTC-AAAGAA--------ACAGGTTTTGTATCGTATAAAGTA-TATACTTCGACTTTCGTGTGCTAG-AACTTTGGCTCGTAAACATAAAAGTACAGTACGCACTTTTATGCGAAGATTAGGTTCGGGATTCTTAG-AAGAATTTTTTTT------TGAAGAAGAAC-AATCTCTTTCTT

Arundina_graminifolia_var_revoluta_AB844207 ACAAGAATTCTTTTTCTTCTCATTTTTCT------------TCTCAAATGGTATCAGAAGGTTTTGGAGTCATTCTGGAAATTCCATTCTCGTCGCAATTAGTATCT---------TCCCTTGAAG---ATAACAGAATACCAAAATTTCAGAATTTACTATCTATTCATTCAATATTTCCCTTTTTAGAGGATAAATTATCACATTTCAATTATGTGTCAG-ATC-TACT-AATACCCCATCCCATCCATC-TGGAAATCTT-GGTTCAAATCCTTCAATGCTGG-ATTAAAGATGTTTCTTCT-TTGCATTTCTTG-CGATTGTT--TTTCCACG------AATATCATAATT---------------TGAATAGTCTTAT---TACT-TC---------------AAATAAATCCATTTACGTCT-TTTCAAAAAG---AACCAAAAGAT-TCT-TTTGG-TTCCTACATA------ATTCTTAT-GTATAT-GAATGC----GAATATATAT-TCCTG---TTTCTTCGT--AAACAG-TCTTCT-TATT-TACG-AT-CAATAT-CTTCTGGA-GTCTTTCTT-GAGCG-AACACATTT--CTATGGAAA--AATAGAA------TATCTTATAGT---CGTGTGTT-GTAATT------CTTTTCA---------GAGG-ATCCTATGGT-TCCTCAA-AGATACT-TTCA-TACATTAT------------GTTC-----GATATCAAGGAAAA---ACA-----ATTCT--GGTTTC-----AAAAG---GAACTCTT---------ATTC------------TGATTAAGAAAT-GGAAATTT--CATCTT-GTGAA-TTTTTGGCA-ATC--TTATT-TTCACTTT-TGGTTTC--AACCTTATAGGA----TTCATATAAA-GCAATT--ACCCAACTATTCCTTCTCTTTTCTGGGG-TATTTTTCAAGTGTACTAAAAAA-TAATTTGGT-AATAA-GAAATC-AAATGCTAGAGAATTCATTT-CTAATAAATACTCT-GACTAAGA-AATTAGATACCATA-GCCCCAGTT--ACTTCTATTATTGG-ATCATTGTCGAAAGCTCA-ATTTTGTACTGTATTGGGT-CATCCTATTAGTAAACC-GATCTGGACCGATTTATCGGATTCTGA-------------TATTTTTGATCGATTTTGTCGGAT-----ATGTAGAAAT---CTTTGTCGT-TATCACAGCGGATCTTC-AAAGAA--------ACAGGTTTTGTATCGTATAAAGTA-TATACTTCGACTTTCGTGTGCTAG-AACTTTGGCTCGTAAACATAAAAGTACAGTACGCACTTTTATGCGAAGATTAGGTTCGGGATTCTTAG-AAGAATTTTTTTTTGAATTTGAAGAAGAAC-AATCTCTTTCTT

Arundina_graminifolia_var_revoluta_AB844208 ACAAGAATTCTTTTTCTTCTCATTTTTCT------------TCTCAAATGGTATCAGAAGGTTTTGGAGTCATTCTGGAAATTCCATTCTCGTCGCAATTAGTATCT---------TCCCTTGAAG---ATAACAGAATACCAAAATTTCAGAATTTACTATCTATTCATTCAATATTTCCCTTTTTAGAGGATAAATTATCACATTTCAATTATGTGTCAG-ATC-TACT-AATACCCCATCCCATCCATC-TGGAAATCTT-GGTTCAAATCCTTCAATGCTGG-ATTAAAGATGTTTCTTCT-TTGCATTTCTTG-CGATTGTT--TTTCCACG------AATATCATAATT---------------TGAATAGTCTCAT---TACT-TC---------------AAATAAATCCATTTACGTCT-TTTCAAAAAG---AACCAAAAGAA-TCT-TTTGG-TTCCTACATA------ATTCTTAT-GTATAT-GAATGC----GAATATATAT-TCCTG---TTTCTTCGT--AAACAG-TCTTCT-TATT-TACG-AT-CAATAT-CTTCTGGA-GTCTTTCTT-GAGCG-AACACATTT--CTATGGAAA--AATAGAA------TATCTTATAGT---CGTGTGTT-GTAATT------CTTTTCA---------GAGG-ATCCTATGGT-TCCTCAA-AGATACT-TTCA-TACATTAT------------GTTC-----GATATCAAGGAAAA---ACA-----ATTCT--GGTTTC-----AAAAG---GAACTCTT---------ATTC------------TGATTAAGAAAT-GGAAATTT--CATCTT-GTGAA-TTTTTGGCA-ATC--TTATT-TTCACTTT-TGGTTTC--AACCTTATAGGA----TTCATATAAA-GCAATT--ACCCAACTATTCCTTCTCTTTTCTGGGG-TATTTTTCAAGTGTACTAAAAAA-TAATTTGGT-AATAA-GAAATC-AAATGCTAGAGAATTCATTT-CTAATAAATACTCT-GACTAAGA-AATTAGATACCATA-GCCCCAGTT--ACTTCTATTATTGG-ATCATTGTCGAAAGCTCA-ATTTTGTACTGTATTGGGT-CATCCTATTAGTAAACC-GATCTGGACCGATTTATCGGATTCTGA-------------TATTTTTGATCGATTTTGTCGGAT-----ATGTAGAAAT---CTTTGTCGT-TATCACAGCGGATCTTC-AAAGAA--------ACAGGTTTTGTATCGTATAAAGTA-TATACTTCGACTTTCGTGTGCTAG-AACTTTGGCTCGTAAACATAAAAGTACAGTACGCACTTTTATGCGAAGATTAGGTTCGGGATTCTTAG-AAGAATTTTTTTT------TGAAGAAGAAC-AATCTCTTTCTT

Arundina_graminifolia_var_revoluta_AB844209 ACAAGAATTCTTTTTCTTCTCATTTTTCT------------TCTCAAATGGTATCAGAAGGTTTTGGAGTCATTCTGGAAATTCCATTCTCGTCGCAATTAGTATCT---------TCCCTTGAAG---ATAACAGAATACCAAAATTTCAGAATTTACTATCTATTCATTCAATATTTCCCTTTTTAGAGGATAAATTATCACATTTCAATTATGTGTCAG-ATC-TACT-AATACCCCATCCCATCCATC-TGGAAATCTT-GGTTCAAATCCTTCAATGCTGG-ATTAAAGATGTTTCTTCT-TTGCATTTCTTG-CGATTGTT--TTTCCACG------AATATCATAATT---------------TGAATAGTCTTAT---TACT-TC---------------AAATAAATCCATTTACGTCT-TTTCAAAAAG---AACCAAAAGAT-TCT-TTTGG-TTCCTACATA------ATTCTTAT-GTATAT-GAATGC----GAATATATAT-TCCTG---TTTCTTCGT--AAACAG-TCTTCT-TATT-TACG-AT-CAATAT-CTTCTGGA-GTCTTTCTT-GAGCG-AACACATTT--CTATGGAAA--AATAGAA------TATCTTATAGT---CGTGTGTT-GTAATT------CTTTTCA---------GAGG-ATCCTATGGT-TCCTCAA-AGATACT-TTCA-TACATTAT------------GTTC-----GATATCAAGGAAAA---ACA-----ATTCT--GGTTTC-----AAAAG---GAACTCTT---------ATTC------------TGATTAAGAAAT-GGAAATTT--CATCTT-GTGAA-TTTTTGGCA-ATC--TTATT-TTCACTTT-TGGTTTC--AACCTTATAGGA----TTCATATAAA-GCAATT--ACCCAACTATTCCTTCTCTTTTCTGGGG-TATTTTTCAAGTGTACTAAAAAA-TAATTTGGT-AATAA-GAAATC-AAATGCTAGAGAATTCATTT-CTAATAAATACTCT-GACTAAGA-AATTAGATACCATA-GCCCCAGTT--ACTTCTATTATTGG-ATCATTGTCGAAAGCTCA-ATTTTGTACTGTATTGGGT-CATCCTATTAGTAAACC-GATCTGGACCGATTTATCGGATTCTGA-------------TATTTTTGATCGATTTTGTCGGAT-----ATGTAGAAAT---CTTTGTCGT-TATCACAGCGGATCTTC-AAAGAA--------ACAGGTTTTGTATCGTATAAAGTA-TATACTTCGACTTTCGTGTGCTAG-AACTTTGGCTCGTAAACATAAAAGTACAGTACGCACTTTTATGCGAAGATTAGGTTCGGGATTCTTAG-AAGAATTTTTTTTTGAATTTGAAGAAGAAC-AATCTCTTTCTT

Arundina_graminifolia_var_revoluta_AB844210 ACAAGAATTCTTTTTCTTCTCATTTTTCT------------TCTCAAATGGTATCAGAAGGTTTTGGAGTCATTCTGGAAATTCCATTCTCGTCGCAATTAGTATCT---------TCCCTTGAAG---ATAACAGAATACCAAAATTTCAGAATTTACTATCTATTCATTCAATATTTCCCTTTTTAGAGGATAAATTATCACATTTCAATTATGTGTCAG-ATC-TACT-AATACCCCATCCCATCCATC-TGGAAATCTT-GGTTCAAATCCTTCAATGCTGG-ATTAAAGATGTTTCTTCT-TTGCATTTCTTG-CGATTGTT--TTTCCACG------AATATCATAATT---------------TGAATAGTCTTAT---TACT-TC---------------AAATAAATCCATTTACGTCT-TTTCAAAAAG---AACCAAAAGAT-TCT-TTTGG-TTCCTACATA------ATTCTTAT-GTATAT-GAATGC----GAATATATAT-TCCTG---TTTCTTCGT--AAACAG-TCTTCT-TATT-TACG-AT-CAATAT-CTTCTGGA-GTCTTTCTT-GAGCG-AACACATTT--CTATGGAAA--AATAGAA------TATCTTATAGT---CGTGTGTT-GTAATT------CTTTTCA---------GAGG-ATCCTATGGT-TCCTCAA-AGATACT-TTCA-TACATTAT------------GTTC-----GATATCAAGGAAAA---ACA-----ATTCT--GGTTTC-----AAAAG---GAACTCTT---------ATTC------------TGATTAAGAAAT-GGAAATTT--CATCTT-GTGAA-TTTTTGGCA-ATC--TTATT-TTCACTTT-TGGTTTC--AACCTTATAGGA----TTCATATAAA-GCAATT--ACCCAACTATTCCTTCTCTTTTCTGGGG-TATTTTTCAAGTGTACTAAAAAA-TAATTTGGT-AATAA-GAAATC-AAATGCTAGAGAATTCATTT-CTAATAAATACTCT-GACTAAGA-AATTAGATACCATA-GCCCCAGTT--ACTTCTATTATTGG-ATCATTGTCGAAAGCTCA-ATTTTGTACTGTATTGGGT-CATCCTATTAGTAAACC-GATCTGGACCGATTTATCGGATTCTGA-------------TATTTTTGATCGATTTTGTCGGAT-----ATGTAGAAAT---CTTTGTCGT-TATCACAGCGGATCTTC-AAAGAA--------ACAGGTTTTGTATCGTATAAAGTA-TATACTTCGACTTTCGTGTGCTAG-AACTTTGGCTCGTAAACATAAAAGTACAGTACGCACTTTTATGCGAAGATTAGGTTCGGGATTCTTAG-AAGAATTTTTTTTTGAATTTGAAGAAGAAC-AATCTCTTTCTT

Arundina_graminifolia_var_revoluta_AB844211 ACAAGAATTCTTTTTCTTCTCATTTTTCT------------TCTCAAATGGTATCAGAAGGTTTTGGAGTCATTCTGGAAATTCCATTCTCGTCGCAATTAGTATCT---------TCCCTTGAAG---ATAACAGAATACCAAAATTTCAGAATTTACTATCTATTCATTCAATATTTCCCTTTTTAGAGGATAAATTATCACATTTCAATTATGTGTCAG-ATC-TACT-AATACCCCATCCCATCCATC-TGGAAATCTT-GGTTCAAATCCTTCAATGCTGG-ATTAAAGATGTTTCTTCT-TTGCATTTCTTG-CGATTGTT--TTTCCACG------AATATCATAATT---------------TGAATAGTCTCAT---TACT-TC---------------AAATAAATCCATTTACGTCT-TTTCAAAAAG---AACCAAAAGAA-TCT-TTTGG-TTCCTACATA------ATTCTTAT-GTATAT-GAATGC----GAATATATAT-TCCTG---TTTCTTCGT--AAACAG-TCTTCT-TATT-TACG-AT-CAATAT-CTTCTGGA-GTCTTTCTT-GAGCG-AACACATTT--CTATGGAAA--AATAGAA------TATCTTATAGT---CGTGTGTT-GTAATT------CTTTTCA---------GAGG-ATCCTATGGT-TCCTCAA-AGATACT-TTCA-TACATTAT------------GTTC-----GATATCAAGGAAAA---ACA-----ATTCT--GGTTTC-----AAAAG---GAACTCTT---------ATTC------------TGATTAAGAAAT-GGAAATTT--CATCTT-GTGAA-TTTTTGGCA-ATC--TTATT-TTCACTTT-TGGTTTC--AACCTTATAGGA----TTCATATAAA-GCAATT--ACCCAACTATTCCTTCTCTTTTCTGGGG-TATTTTTCAAGTGTACTAAAAAA-TAATTTGGT-AATAA-GAAATC-AAATGCTAGAGAATTCATTT-CTAATAAATACTCT-GACTAAGA-AATTAGATACCATA-GCCCCAGTT--ACTTCTATTATTGG-ATCATTGTCGAAAGCTCA-ATTTTGTACTGTATTGGGT-CATCCTATTAGTAAACC-GATCTGGACCGATTTATCGGATTCTGA-------------TATTTTTGATCGATTTTGTCGGAT-----ATGTAGAAAT---CTTTGTCGT-TATCACAGCGGATCTTC-AAAGAA--------ACAGGTTTTGTATCGTATAAAGTA-TATACTTCGACTTTCGTGTGCTAG-AACTTTGGCTCGTAAACATAAAAGTACAGTACGCACTTTTATGCGAAGATTAGGTTCGGGATTCTTAG-AAGAATTTTTTTT------TGAAGAAGAAC-AATCTCTTTCTT

Arundina_graminifolia_var_revoluta_AB844212 ACAAGAATTCTTTTTCTTCTCATTTTTCT------------TCTCAAATGGTATCAGAAGGTTTTGGAGTCATTCTGGAAATTCCATTCTCGTCGCAATTAGTATCT---------TCCCTTGAAG---ATAACAGAATACCAAAATTTCAGAATTTACTATCTATTCATTCAATATTTCCCTTTTTAGAGGATAAATTATCACATTTCAATTATGTGTCAG-ATC-TACT-AATACCCCATCCCATCCATC-TGGAAATCTT-GGTTCAAATCCTTCAATGCTGG-ATTAAAGATGTTTCTTCT-TTGCATTTCTTG-CGATTGTT--TTTCCACG------AATATCATAATT---------------TGAATAGTCTTAT---TACT-TC---------------AAATAAATCCATTTACGTCT-TTTCAAAAAG---AACCAAAAGAT-TCT-TTTGG-TTCCTACATA------ATTCTTAT-GTATAT-GAATGC----GAATATATAT-TCCTG---TTTCTTCGT--AAACAG-TCTTCT-TATT-TACG-AT-CAATAT-CTTCTGGA-GTCTTTCTT-GAGCG-AACACATTT--CTATGGAAA--AATAGAA------TATCTTATAGT---CGTGTGTT-GTAATT------CTTTTCA---------GAGG-ATCCTATGGT-TCCTCAA-AGATACT-TTCA-TACATTAT------------GTTC-----GATATCAAGGAAAA---ACA-----ATTCT--GGTTTC-----AAAAG---GAACTCTT---------ATTC------------TGATTAAGAAAT-GGAAATTT--CATCTT-GTGAA-TTTTTGGCA-ATC--TTATT-TTCACTTT-TGGTTTC--AACCTTATAGGA----TTCATATAAA-GCAATT--ACCCAACTATTCCTTCTCTTTTCTGGGG-TATTTTTCAAGTGTACTAAAAAA-TAATTTGGT-AATAA-GAAATC-AAATGCTAGAGAATTCATTT-CTAATAAATACTCT-GACTAAGA-AATTAGATACCATA-GCCCCAGTT--ACTTCTATTATTGG-ATCATTGTCGAAAGCTCA-ATTTTGTACTGTATTGGGT-CATCCTATTAGTAAACC-GATCTGGACCGATTTATCGGATTCTGA-------------TATTTTTGATCGATTTTGTCGGAT-----ATGTAGAAAT---CTTTGTCGT-TATCACAGCGGATCTTC-AAAGAA--------ACAGGTTTTGTATCGTATAAAGTA-TATACTTCGACTTTCGTGTGCTAG-AACTTTGGCTCGTAAACATAAAAGTACAGTACGCACTTTTATGCGAAGATTAGGTTCGGGATTCTTAG-AAGAATTTTTTTTTGAATTTGAAGAAGAAC-AATCTCTTTCTT

Arundina_graminifolia_var_revoluta_AB844213 ACAAGAATTCTTTTTCTTCTCATTTTTCT------------TCTCAAATGGTATCAGAAGGTTTTGGAGTCATTCTGGAAATTCCATTCTCGTCGCAATTAGTATCT---------TCCCTTGAAG---ATAACAGAATACCAAAATTTCAGAATTTACTATCTATTCATTCAATATTTCCCTTTTTAGAGGATAAATTATCACATTTCAATTATGTGTCAG-ATC-TACT-AATACCCCATCCCATCCATC-TGGAAATCTT-GGTTCAAATCCTTCAATGCTGG-ATTAAAGATGTTTCTTCT-TTGCATTTCTTG-CGATTGTT--TTTCCACG------AATATCATAATT---------------TGAATAGTCTCAT---TACT-TC---------------AAATAAATCCATTTACGTCT-TTTCAAAAAG---AACCAAAAGAA-TCT-TTTGG-TTCCTACATA------ATTCTTAT-GTATAT-GAATGC----GAATATATAT-TCCTG---TTTCTTCGT--AAACAG-TCTTCT-TATT-TACG-AT-CAATAT-CTTCTGGA-GTCTTTCTT-GAGCG-AACACATTT--CTATGGAAA--AATAGAA------TATCTTATAGT---CGTGTGTT-GTAATT------CTTTTCA---------GAGG-ATCCTATGGT-TCCTCAA-AGATACT-TTCA-TACATTAT------------GTTC-----GATATCAAGGAAAA---ACA-----ATTCT--GGTTTC-----AAAAG---GAACTCTT---------ATTC------------TGATTAAGAAAT-GGAAATTT--CATCTT-GTGAA-TTTTTGGCA-ATC--TTATT-TTCACTTT-TGGTTTC--AACCTTATAGGA----TTCATATAAA-GCAATT--ACCCAACTATTCCTTCTCTTTTCTGGGG-TATTTTTCAAGTGTACTAAAAAA-TAATTTGGT-AATAA-GAAATC-AAATGCTAGAGAATTCATTT-CTAATAAATACTCT-GACTAAGA-AATTAGATACCATA-GCCCCAGTT--ACTTCTATTATTGG-ATCATTGTCGAAAGCTCA-ATTTTGTACTGTATTGGGT-CATCCTATTAGTAAACC-GATCTGGACCGATTTATCGGATTCTGA-------------TATTTTTGATCGATTTTGTCGGAT-----ATGTAGAAAT---CTTTGTCGT-TATCACAGCGGATCTTC-AAAGAA--------ACAGGTTTTGTATCGTATAAAGTA-TATACTTCGACTTTCGTGTGCTAG-AACTTTGGCTCGTAAACATAAAAGTACAGTACGCACTTTTATGCGAAGATTAGGTTCGGGATTCTTAG-AAGAATTTTTTTT------TGAAGAAGAAC-AATCTCTTTCTT

Arundina_graminifolia_var_revoluta_AB844214 ACAAGAATTCTTTTTCTTCTCATTTTTCT------------TCTCAAATGGTATCAGAAGGTTTTGGAGTCATTCTGGAAATTCCATTCTCGTCGCAATTAGTATCT---------TCCCTTGAAG---ATAACAGAATACCAAAATTTCAGAATTTACTATCTATTCATTCAATATTTCCCTTTTTAGAGGATAAATTATCACATTTCAATTATGTGTCAG-ATC-TACT-AATACCCCATCCCATCCATC-TGGAAATCTT-GGTTCAAATCCTTCAATGCTGG-ATTAAAGATGTTTCTTCT-TTGCATTTCTTG-CGATTGTT--TTTCCACG------AATATCATAATT---------------TGAATAGTCTCAT---TACT-TC---------------AAATAAATCCATTTACGTCT-TTTCAAAAAG---AACCAAAAGAA-TCT-TTTGG-TTCCTACATA------ATTCTTAT-GTATAT-GAATGC----GAATATATAT-TCCTG---TTTCTTCGT--AAACAG-TCTTCT-TATT-TACG-AT-CAATAT-CTTCTGGA-GTCTTTCTT-GAGCG-AACACATTT--CTATGGAAA--AATAGAA------TATCTTATAGT---CGTGTGTT-GTAATT------CTTTTCA---------GAGG-ATCCTATGGT-TCCTCAA-AGATACT-TTCA-TACATTAT------------GTTC-----GATATCAAGGAAAA---ACA-----ATTCT--GGTTTC-----AAAAG---GAACTCTT---------ATTC------------TGATTAAGAAAT-GGAAATTT--CATCTT-GTGAA-TTTTTGGCA-ATC--TTATT-TTCACTTT-TGGTTTC--AACCTTATAGGA----TTCATATAAA-GCAATT--ACCCAACTATTCCTTCTCTTTTCTGGGG-TATTTTTCAAGTGTACTAAAAAA-TAATTTGGT-AATAA-GAAATC-AAATGCTAGAGAATTCATTT-CTAATAAATACTCT-GACTAAGA-AATTAGATACCATA-GCCCCAGTT--ACTTCTATTATTGG-ATCATTGTCGAAAGCTCA-ATTTTGTACTGTATTGGGT-CATCCTATTAGTAAACC-GATCTGGACCGATTTATCGGATTCTGA-------------TATTTTTGATCGATTTTGTCGGAT-----ATGTAGAAAT---CTTTGTCGT-TATCACAGCGGATCTTC-AAAGAA--------ACAGGTTTTGTATCGTATAAAGTA-TATACTTCGACTTTCGTGTGCTAG-AACTTTGGCTCGTAAACATAAAAGTACAGTACGCACTTTTATGCGAAGATTAGGTTCGGGATTCTTAG-AAGAATTTTTTTT------TGAAGAAGAAC-AATCTCTTTCTT

Arundina_graminifolia_var_revoluta_AB844215 ACAAGAATTCTTTTTCTTCTCATTTTTCT------------TCTCAAATGGTATCAGAAGGTTTTGGAGTCATTCTGGAAATTCCATTCTCGTCGCAATTAGTATCT---------TCCCTTGAAG---ATAACAGAATACCAAAATTTCAGAATTTACTATCTATTCATTCAATATTTCCCTTTTTAGAGGATAAATTATCACATTTCAATTATGTGTCAG-ATC-TACT-AATACCCCATCCCATCCATC-TGGAAATCTT-GGTTCAAATCCTTCAATGCTGG-ATTAAAGATGTTTCTTCT-TTGCATTTCTTG-CGATTGTT--TTTCCACG------AATATCATAATT---------------TGAATAGTCTCAT---TACT-TC---------------AAATAAATCCATTTACGTCT-TTTCAAAAAG---AACCAAAAGAA-TCT-TTTGG-TTCCTACATA------ATTCTTAT-GTATAT-GAATGC----GAATATATAT-TCCTG---TTTCTTCGT--AAACAG-TCTTCT-TATT-TACG-AT-CAATAT-CTTCTGGA-GTCTTTCTT-GAGCG-AACACATTT--CTATGGAAA--AATAGAA------TATCTTATAGT---CGTGTGTT-GTAATT------CTTTTCA---------GAGG-ATCCTATGGT-TCCTCAA-AGATACT-TTCA-TACATTAT------------GTTC-----GATATCAAGGAAAA---ACA-----ATTCT--GGTTTC-----AAAAG---GAACTCTT---------ATTC------------TGATTAAGAAAT-GGAAATTT--CATCTT-GTGAA-TTTTTGGCA-ATC--TTATT-TTCACTTT-TGGTTTC--AACCTTATAGGA----TTCATATAAA-GCAATT--ACCCAACTATTCCTTCTCTTTTCTGGGG-TATTTTTCAAGTGTACTAAAAAA-TAATTTGGT-AATAA-GAAATC-AAATGCTAGAGAATTCATTT-CTAATAAATACTCT-GACTAAGA-AATTAGATACCATA-GCCCCAGTT--ACTTCTATTATTGG-ATCATTGTCGAAAGCTCA-ATTTTGTACTGTATTGGGT-CATCCTATTAGTAAACC-GATCTGGACCGATTTATCGGATTCTGA-------------TATTTTTGATCGATTTTGTCGGAT-----ATGTAGAAAT---CTTTGTCGT-TATCACAGCGGATCTTC-AAAGAA--------ACAGGTTTTGTATCGTATAAAGTA-TATACTTCGACTTTCGTGTGCTAG-AACTTTGGCTCGTAAACATAAAAGTACAGTACGCACTTTTATGCGAAGATTAGGTTCGGGATTCTTAG-AAGAATTTTTTTT------TGAAGAAGAAC-AATCTCTTTCTT

Arundina_graminifolia_var_revoluta_AB844216 ACAAGAATTCTTTTTCTTCTCATTTTTCT------------TCTCAAATGGTATCAGAAGGTTTTGGAGTCATTCTGGAAATTCCATTCTCGTCGCAATTAGTATCT---------TCCCTTGAAG---ATAACAGAATACCAAAATTTCAGAATTTACTATCTATTCATTCAATATTTCCCTTTTTAGAGGATAAATTATCACATTTCAATTATGTGTCAG-ATC-TACT-AATACCCCATCCCATCCATC-TGGAAATCTT-GGTTCAAATCCTTCAATGCTGG-ATTAAAGATGTTTCTTCT-TTGCATTTCTTG-CGATTGTT--TTTCCACG------AATATCATAATT---------------TGAATAGTCTCAT---TACT-TC---------------AAATAAATCCATTTACGTCT-TTTCAAAAAG---AACCAAAAGAA-TCT-TTTGG-TTCCTACATA------ATTCTTAT-GTATAT-GAATGC----GAATATATAT-TCCTG---TTTCTTCGT--AAACAG-TCTTCT-TATT-TACG-AT-CAATAT-CTTCTGGA-GTCTTTCTT-GAGCG-AACACATTT--CTATGGAAA--AATAGAA------TATCTTATAGT---CGTGTGTT-GTAATT------CTTTTCA---------GAGG-ATCCTATGGT-TCCTCAA-AGATACT-TTCA-TACATTAT------------GTTC-----GATATCAAGGAAAA---ACA-----ATTCT--GGTTTC-----AAAAG---GAACTCTT---------ATTC------------TGATTAAGAAAT-GGAAATTT--CATCTT-GTGAA-TTTTTGGCA-ATC--TTATT-TTCACTTT-TGGTTTC--AACCTTATAGGA----TTCATATAAA-GCAATT--ACCCAACTATTCCTTCTCTTTTCTGGGG-TATTTTTCAAGTGTACTAAAAAA-TAATTTGGT-AATAA-GAAATC-AAATGCTAGAGAATTCATTT-CTAATAAATACTCT-GACTAAGA-AATTAGATACCATA-GCCCCAGTT--ACTTCTATTATTGG-ATCATTGTCGAAAGCTCA-ATTTTGTACTGTATTGGGT-CATCCTATTAGTAAACC-GATCTGGACCGATTTATCGGATTCTGA-------------TATTTTTGATCGATTTTGTCGGAT-----ATGTAGAAAT---CTTTGTCGT-TATCACAGCGGATCTTC-AAAGAA--------ACAGGTTTTGTATCGTATAAAGTA-TATACTTCGACTTTCGTGTGCTAG-AACTTTGGCTCGTAAACATAAAAGTACAGTACGCACTTTTATGCGAAGATTAGGTTCGGGATTCTTAG-AAGAATTTTTTTT------TGAAGAAGAAC-AATCTCTTTCTT

Arundina_graminifolia_var_revoluta_AB844217 ACAAGAATTCTTTTTCTTCTCATTTTTCT------------TCTCAAATGGTATCAGAAGGTTTTGGAGTCATTCTGGAAATTCCATTCTCGTCGCAATTAGTATCT---------TCCCTTGAAG---ATAACAGAATACCAAAATTTCAGAATTTACTATCTATTCATTCAATATTTCCCTTTTTAGAGGATAAATTATCACATTTCAATTATGTGTCAG-ATC-TACT-AATACCCCATCCCATCCATC-TGGAAATCTT-GGTTCAAATCCTTCAATGCTGG-ATTAAAGATGTTTCTTCT-TTGCATTTCTTG-CGATTGTT--TTTCCACG------AATATCATAATT---------------TGAATAGTCTCAT---TACT-TC---------------AAATAAATCCATTTACGTCT-TTTCAAAAAG---AACCAAAAGAA-TCT-TTTGG-TTCCTACATA------ATTCTTAT-GTATAT-GAATGC----GAATATATAT-TCCTG---TTTCTTCGT--AAACAG-TCTTCT-TATT-TACG-AT-CAATAT-CTTCTGGA-GTCTTTCTT-GAGCG-AACACATTT--CTATGGAAA--AATAGAA------TATCTTATAGT---CGTGTGTT-GTAATT------CTTTTCA---------GAGG-ATCCTATGGT-TCCTCAA-AGATACT-TTCA-TACATTAT------------GTTC-----GATATCAAGGAAAA---ACA-----ATTCT--GGTTTC-----AAAAG---GAACTCTT---------ATTC------------TGATTAAGAAAT-GGAAATTT--CATCTT-GTGAA-TTTTTGGCA-ATC--TTATT-TTCACTTT-TGGTTTC--AACCTTATAGGA----TTCATATAAA-GCAATT--ACCCAACTATTCCTTCTCTTTTCTGGGG-TATTTTTCAAGTGTACTAAAAAA-TAATTTGGT-AATAA-GAAATC-AAATGCTAGAGAATTCATTT-CTAATAAATACTCT-GACTAAGA-AATTAGATACCATA-GCCCCAGTT--ACTTCTATTATTGG-ATCATTGTCGAAAGCTCA-ATTTTGTACTGTATTGGGT-CATCCTATTAGTAAACC-GATCTGGACCGATTTATCGGATTCTGA-------------TATTTTTGATCGATTTTGTCGGAT-----ATGTAGAAAT---CTTTGTCGT-TATCACAGCGGATCTTC-AAAGAA--------ACAGGTTTTGTATCGTATAAAGTA-TATACTTCGACTTTCGTGTGCTAG-AACTTTGGCTCGTAAACATAAAAGTACAGTACGCACTTTTATGCGAAGATTAGGTTCGGGATTCTTAG-AAGAATTTTTTTT------TGAAGAAGAAC-AATCTCTTTCTT

Bletilla_striata_AF263630 ACAAGAATTCTTTTTCTTCTCATTTTTCT------------TCTCAAATGGTATCAGAAGGTTTTGGAGTCATTCTGGAAATTCCATTCTCGTCGCGATTAGTATCT---------TCCCTTGAAG---AAAAAAGAATACCAAAATCTCAGAATTTACGATCTATTCATTCAATATTTCCCTTTTTAGAGGATAAATTATCACATTTAAATTATGTGTCAG-ATC-TACT-AATACCCCATCCCATACATC-TGGAAATCTT-GGTTCAAATCCTTCAATGCTGG-ATCAAAGATGTTTCTTCT-TTGCATTTCTTG-CGATTGTT--TTTCCATG------AATATCATAATT---------------TGAATAGTCTCAT---TACT-TC---------------AAATAAATCCACTTACGTCT-TTTCAAAAAG---AAAGAAAAGAT-TCT-TTTGG-TTCCTACATA------ATTCTTAT-GTATAT-GAATGC----GAATATCTAT-TCCTG---TTTCTTCGT--AAAAAG-TCTTCT-TATT-TACG-AT-CAATAT-CTTCTGGA-GTTTTTCTT-GAGCG-AACACATTT--CTATGGAAA--AATAGAA------TATCTTATAGT---CGTGTGTT-GTAATT------CTTTTCA---------GAGT-ATCCTATGGT-TCCTCAA-AGATACT-TTCA-TACATTAT------------GTTC-----GATATCAAGGAAAA---GCA-----ATTCT--GGCTTC-----AAAAG---GAACTCTT---------ATTC------------TGATGAAGAAAT-GGAAGTTT--TATCTT-GTGAA-TTTTTGGCA-ATC--TTATT-TTCACTTT-TGGTTTC--AACCTTATAGGA----TCTATATAAA-GCAATT--ACCCAACTATTCCTTCTCTTTTCTGGGA-TATTTCTCAAGTGTACTAAAAAA-TCCTTTGGT-AGTAA-GAAATC-AAATGCTAGATAATTCATTT-CTAATAAATACTCT-GACTAAGA-AATTAGATACCGGA-GCCCCAGTT--ATTTCTCTTATTGG-ATCATTGTCGAAAGCTCA-ATTTTGTACTGTATTGGGT-CATCCTATTAGTAAACC-GATCTGGACCGATTTATCGGATTCTGA-------------TATTCTTGATCGATTTTGTCGGAT-----ATGTAGAAAT---CTTTGTCGT-TATCACAGCGGATCCTC-AAAGAA--------GCAGGTTTTGTATCGTATAAAGTA-TATACTTCGACTTTCGTGTGCTAG-AACTTTGGCTCGCAAACATAAAAGTACAGTACGCACTTTTATGCGAAGATTAGGTTCGGGATTCTTAG-AAGAATTTTTTTT------GGAAGAAGAAC-AATCTCTTTCTT

Bletilla_striata_EF079331 ACAAGAATTCTTTTTCTTCTCATTTTTCT------------TCTCAAATGGTATCAGAAGGTTTTGGAGTCATTCTGGAAATTCCATTCTCGTCGCGATTAGTATCT---------TCCCTTGAAG---AAAAAAGAATACCAAAATCTCAGAATTTACGATCTATTCATTCAATATTTCCCTTTTTAGAGGATAAATTATCACATTTAAATTATGTGTCAG-ATC-TACT-AATACCCCATCCCATACATC-TGGAAATCTT-GGTTCAAATCCTTCAATGCTGG-ATCAAAGATGTTTCTTCT-TTGCATTTCTTG-CGATTGTT--TTTCCATG------AATATCATAATT---------------TGAATAGTCTCAT---TACT-TC---------------AAATAAATCCACTTACGTCT-TTTCAAAAAG---AAAGAAAAGAT-TCT-TTTGG-TTCCTACATA------ATTCTTAT-GTATAT-GAATGC----GAATATCTAT-TCCTG---TTTCTTCGT--AAAAAG-TCTTCT-TATT-TACG-AT-CAATAT-CTTCTGGA-GTTTTTCTT-GAGCG-AACACATTT--CTATGGAAA--AATAGAA------TATCTTATAGT---CGTGTGTT-GTAATT------CTTTTCA---------GAGT-ATCCTATGGT-TCCTCAA-AGATACT-TTCA-TACATTAT------------GTTC-----GATATCAAGGAAAA---GCA-----ATTCT--GGCTTC-----AAAAG---GAACTCTT---------ATTC------------TGATGAAGAAAT-GGAAATTT--TATCTT-GTGAA-TTTTTGGCA-ATC--TTATT-TTCACTTT-TGGTTTC--AACCTTATAGGA----TCTATATAAA-GCAATT--ACCCAACTATTCCTTCTCTTTTCTGGGA-TATTTTTCAAGTGTACTAAAAAA-TCCTTTGGT-AGTAA-GAAATC-AAATGCTAGATAATTCATTT-CTAATAAATACTCT-GACTAAGA-AATTAGATACCGTA-GCCCCAGTT--ATTTCTCTTATTGG-ATCATTGTCGAAAGCTCA-ATTTTGTACTGTATTGGGT-CATCCTATTAGTAAACC-GATCTGGACCGATTTATCGGATTCTGA-------------TATTCTTGATCGATTTTGTCGGAT-----ATGTAGAAAT---CTTTGTCGT-TATCACAGCGGATCCTC-AAAGAA--------ACAGGTTTTGTATCGTATAAAGTA-TATACTTCGACTTTCGTGTGCTAG-AACTTTGGCTCGTAAACGTAAAAGTACCGTACGCACTTTTATGCGAAGATTAGGTTCGGGATTCTTAG-AAGAATTTTTTTT------GGAAGAAGAAC-AATCTCTTTCTT

Bletilla_striata_EU490679 ACAAGAATTCTTTTTCTTCTCATTTTTCT------------TCTCAAATGGTATCAGAAGGTTTTGGAGTCATTCTGGAAATTCCATTCTCGTCGCGATTAGTATCT---------TCCCTTGAAG---AAAAAAGAATACCAAAATCTCAGAATTTACGATCTATTCATTCAATATTTCCCTTTTTAGAGGATAAATTATCACATTTAAATTATGTGTCAG-ATC-TACT-AATACCCCATCCCATACATC-TGGAAATCTT-GGTTCAAATCCTTCAATGCTGG-ATCAAAGATGTTTCTTCT-TTGCATTTCTTG-CGATTGTT--TTTCCATG------AATATCATAATT---------------TGAATAGTCTCAT---TACT-TC---------------AAATAAATCCACTTACGTCT-TTTCAAAAAG---AAAGAAAAGAT-TCT-TTTGG-TTCCTACATA------ATTCTTAT-GTATAT-GAATGC----GAATATCTAT-TCCTG---TTTCTTCGT--AAAAAG-TCTTCT-TATT-TACG-AT-CAATAT-CTTCTGGA-GTTTTTCTT-GAGCG-AACACATTT--CTATGGAAA--AATAGAA------TATCTTATAGT---CGTGTGTT-GTAATT------CTTTTCA---------GAGT-ATCCTATGGT-TCCTCAA-AGATACT-TTCA-TACATTAT------------GTTC-----GATATCAAGGAAAA---GCA-----ATTCT--GGCTTC-----AAAAG---GAACTCTT---------ATTC------------TGATGAAGAAAT-GGAAATTT--TATCTT-GTGAA-TTTTTGGCA-ATC--TTATT-TTCACTTT-TGGTTTC--AACCTTATAGGA----TCTATATAAA-GCAATT--ACCCAACTATTCCTTCTCTTTTCTGGGA-TATTTTTCAAGTGTACTAAAAAA-TCCTTTGGT-AGTAA-GAAATC-AAATGCTAGATAATTCATTT-CTAATAAATACTCT-GACTAAGA-AATTAGATACCGTA-GCCCCAGTT--ATTTCTCTTATTGG-ATCATTGTCGAAAGCTCA-ATTTTGTACTGTATTGGGT-CATCCTATTAGTAAACC-GATCTGGACCGATTTATCGGATTCTGA-------------TATTCTTGATCGATTTTGTCGGAT-----ATGTAGAAAT---CTTTGTCGT-TATCACAGCGGATCCTC-AAAGAA--------ACAGGTTTTGTATCGTATAAAGTA-TATACTTCGACTTTCGTGTGCTAG-AACTTTGGCTCGTAAACATAAAAGTACAGTACGCACTTTTATGCGAAGATTAGGTTCGGGATTCTTAG-AAGAATTTTTTTT------GGAAGAAGAA--------------

Bletilla_striata_GQ434129 -------------------------------------------------------------------------------------------------------------------------------------------------------------------------------------------------------------------------------ATC-TACT-AATACCCCATCCCATACATC-TGGAAATCTT-GGTTCAAATCCTTCAATGCTGG-ATCAAAGATGTTTCTTCT-TTGCATTTCTTG-CGATTGTT--TTTCCATG------AATATCATAATT---------------TGAATAGTCTCAT---TACT-TC---------------AAATAAATCCACTTACGTCT-TTTCAAAAAG---AAAGAAAAGAT-TCT-TTTGG-TTCCTACATA------ATTCTTAT-GTATAT-GAATGC----GAATATCTAT-TCCTG---TTTCTTCGT--AAAAAG-TCTTCT-TATT-TACG-AT-CAATAT-CTTCTGGA-GTTTTTCTT-GAGCG-AACACATTT--CTATGGAAA--AATAGAA------TATCTTATAGT---CGTGTGTT-GTAATT------CTTTTCA---------GAGT-ATCCTATGGT-TCCTCAA-AGATACT-TTCA-TACATTAT------------GTTC-----GATATCAAGGAAAA---GCA-----ATTCT--GGCTTC-----AAAAG---GAACTCTT---------ATTC------------TGATGAAGAAAT-GGAAATTT--TATCTT-GTGAA-TTTTTGGCA-ATC--TTATT-TTCACTTT-TGGTTTC--AACCTTATAGGA----TCTATATAAA-GCAATT--ACCCAACTATTCCTTCTCTTTTCTGGGA-TATTTTTCAAGTGTACTAAAAAA-TCCTTTGGT-AGTAA-GAAATC-AAATGCTAGATAATTCATTT-CTAATAAATACTCT-GACTAAGA-AATTAGATACCGTA-GCCCCAGTT--ATTTCTCTTATTGG-ATCATTGTCGAAAGCTCA-ATTTTGTACTGTATTGGGT-CATCCTATTAGTAAACC-GATCTGGACCGATTTATCGGATTCTGA-------------TATTCTTGATCGATTTTGTCGGAT-----ATGTAGAAAT---CTTTGTC-----------------------------------------------------------------------------------------------------------------------------------------------------------------------------------------------------

Bletilla_striata_KC704596 ------------------------------------------------------------------------------------------------------------------------------------------------------------------------------------------------------------------------------------------------------------------------------AATCCTTCAATGCTGG-ATCAAAGATGTTTCTTCT-TTGCATTTCTTG-CGATTGTT--TTTCCATG------AATATCATAATT---------------TGAATAGTCTCAT---TACT-TC---------------AAATAAATCCACTTACGTCT-TTTCAAAAAG---AAAGAAAAGAT-TCT-TTTGG-TTCCTACATA------ATTCTTAT-GTATAT-GAATGC----GAATATCTAT-TCCTG---TTTCTTCGT--AAAAAG-TCTTCT-TATT-TACG-AT-CAATAT-CTTCTGGA-GTTTTTCTT-GAGCG-AACACATTT--CTATGGAAA--AATAGAA------TATCTTATAGT---CGTGTGTT-GTAATT------CTTTTCA---------GAGT-ATCCTATGGT-TCCTCAA-AGATACT-TTCA-TACATTAT------------GTTC-----GATATCAAGGAAAA---GCA-----ATTCT--GGCTTC-----AAAAG---GAACTCTT---------ATTC------------TGATGAAGAAAT-GGAAATTT--TATCTT-GTGAA-TTTTTGGCA-ATC--TTATT-TTCACTTT-TGGTTTC--AACCTTATAGGA----TCTATATAAA-GCAATT--ACCCAACTATTCCTTCTCTTTTCTGGGA-TATTTTTCAAGTGTACTAAAAAA-TCCTTTGGT-AGTAA-GAAATC-AAATGCTAGATAATTCATTT-CTAATAAATACTCT-GACTAAGA-AATTAGATACCGTA-GCCCCAGTT--ATTTCTCTTATTGG-ATCATTGTCGAAAGCTCA-ATTTTGTACTGTATTGGGT-CATCCTATTAGTAAACC-GATCTGGACCGATTTATCGGATTCTGA-------------TATTCTTGATCGATTTTGTCGGAT-----ATGTAGAAAT---CTTTGTCGT-TATCACAGCGGATCCTC-AAAGAA--------ACAGGTTTTGTATCGTATAAAGTA-TATACTTCGACTTTCGTGTGCTAG-AACTTTGG--------------------------------------------------------------------------------------------------------

Bletilla_striata_KC704597 ------------------------------------------------------------------------------------------------------------------------------------------------------------------------------------------------------------------------------------------------------------------------------AATCCTTCAATGCTGG-ATCAAAGATGTTTCTTCT-TTGCATTTCTTG-CGATTGTT--TTTCCATG------AATATCATAATT---------------TGAATAGTCTCAT---TACT-TC---------------AAATAAATCCACTTACGTCT-TTTCAAAAAG---AAAGAAAAGAT-TCT-TTTGG-TTCCTACATA------ATTCTTAT-GTATAT-GAATGC----GAATATCTAT-TCCTG---TTTCTTCGT--AAAAAG-TCTTCT-TATT-TACG-AT-CAATAT-CTTCTGGA-GTTTTTCTT-GAGCG-AACACATTT--CTATGGAAA--AATAGAA------TATCTTATAGT---CGTGTGTT-GTAATT------CTTTTCA---------GAGT-ATCCTATGGT-TCCTCAA-AGATACT-TTCA-TACATTAT------------GTTC-----GATATCAAGGAAAA---GCA-----ATTCT--GGCTTC-----AAAAG---GAACTCTT---------ATTC------------TGATGAAGAAAT-GGAAATTT--TATCTT-GTGAA-TTTTTGGCA-ATC--TTATT-TTCACTTT-TGGTTTC--AACCTTATAGGA----TCTATATAAA-GCAATT--ACCCAACTATTCCTTCTCTTTTCTGGGA-TATTTTTCAAGTGTACTAAAAAA-TCCTTTGGT-AGTAA-GAAATC-AAATGCTAGATAATTCATTT-CTAATAAATACTCT-GACTAAGA-AATTAGATACCGTA-GCCCCAGTT--ATTTCTCTTATTGG-ATCATTGTCGAAAGCTCA-ATTTTGTACTGTATTGGGT-CATCCTATTAGTAAACC-GATCTGGACCGATTTATCGGATTCTGA-------------TATTCTTGATCGATTTTGTCGGAT-----ATGTAGAAAT---CTTTGTCGT-TATCACAGCGGATCCTC-AAAGAA--------ACAGGTTTTGTATCGTATAAAGTA-TATACTTCGACTTTCGTGTGCTAG-AACTTTGG--------------------------------------------------------------------------------------------------------

Bletilla_striata_KF262041 --------------------------------------------------------------------------------------------------------------------------------------------------------------------------------------------------------------------------------------------------------------------------------------------------------GTTCCTTCT-TTGCATTTCTTG-CGATTGTT--TTTCCATG------AATATCATAATT---------------TGAATAGTCTCAT---TACT-TC---------------AAATAAATCCACTTACGTCT-TTTCAAAAAG---AAAGAAAAGAT-TCT-TTTGG-TTCCTACATA------ATTCTTAT-GTATAT-GAATGC----GAATATCTAT-TCCTG---TTTCTTCGT--AAAAAG-TCTTCT-TATT-TACG-AT-CAATAT-CTTCTGGA-GTTTTTCTT-GAGCG-AACACATTT--CTATGGAAA--AATAGAA------TATCTTATAGT---CGTGTGTT-GTAATT------CTTTTCA---------GAGT-ATCCTATGGT-TCCTCAA-AGATACT-TTCA-TACATTAT------------GTTC-----GATATCAAGGAAAA---GCA-----ATTCT--GGCTTC-----AAAAG---GAACTCTT---------ATTC------------TGATGAAGAAAT-GGAAATTT--TATCTT-GTGAA-TTTTTGGCA-ATC--TTATT-TTCACTTT-TGGTTTC--AACCTTATAGGA----TCTATATAAA-GCAATT--ACCCAACTATTCCTTCTCTTTTCTGGGA-TATTTTTCAAGTGTACTAAAAAA-TCCTTTGGT-AGTAA-GAAATC-AAATGCTAGATAATTCATTT-CTAATAAATACTCT-GACTAAGA-AATTAGATACCGTA-GCCCCAGTT--ATTTCTCTTATTGG-ATCATTGTCGAAAGCTCA-ATTTTGTACTGTATTGGGT-CATCCTATTAGTAAACC-GATCTGGACCGATTTATCGGATTCTGA-------------TATTCTTGATCGATTTTGTCGGAT-----ATGTAGAAAT---CTTTGTCGT-TATC----------------------------------------------------------------------------------------------------------------------------------------------------------------------------------------------

Bletilla_striata_KF262042 --------------------------------------------------------------------------------------------------------------------------------------------------------------------------------------------------------------------------------------------------------------------------------------------------------GTTCCTTCT-TTGCATTTCTTG-CGATTGTT--TTTCCATG------AATATCATAATT---------------TGAATAGTCTCAT---TACT-TC---------------AAATAAATCCACTTACGTCT-TTTCAAAAAG---AAAGAAAAGAT-TCT-TTTGG-TTCCTACATA------ATTCTTAT-GTATAT-GAATGC----GAATATCTAT-TCCTG---TTTCTTCGT--AAAAAG-TCTTCT-TATT-TACG-AT-CAATAT-CTTCTGGA-GTTTTTCTT-GAGCG-AACACATTT--CTATGGAAA--AATAGAA------TATCTTATAGT---CGTGTGTT-GTAATT------CTTTTCA---------GAGT-ATCCTATGGT-TCCTCAA-AGATACT-TTCA-TACATTAT------------GTTC-----GATATCAAGGAAAA---GCA-----ATTCT--GGCTTC-----AAAAG---GAACTCTT---------ATTC------------TGATGAAGAAAT-GGAAATTT--TATCTT-GTGAA-TTTTTGGCA-ATC--TTATT-TTCACTTT-TGGTTTC--AACCTTATAGGA----TCTATATAAA-GCAATT--ACCCAACTATTCCTTCTCTTTTCTGGGA-TATTTTTCAAGTGTACTAAAAAA-TCCTTTGGT-AGTAA-GAAATC-AAATGCTAGATAATTCATTT-CTAATAAATACTCT-GACTAAGA-AATTAGATACCGTA-GCCCCAGTT--ATTTCTCTTATTGG-ATCATTGTCGAAAGCTCA-ATTTTGTACTGTATTGGGT-CATCCTATTAGTAAACC-GATCTGGACCGATTTATCGGATTCTGA-------------TATTCTTGATCGATTTTGTCGGAT-----ATGTAGAAAT---CTTTGTCGT-TATC----------------------------------------------------------------------------------------------------------------------------------------------------------------------------------------------

Bletilla_striata_KF361655 -----------------------------------------------------------------------------------------------------------------------------------------------------------------------------------------------------------------AATTATGTGTCAG-ATC-TACT-AATACCCCATCCCATACATC-TGGAAATCTT-GGTTCAAATCCTTCAATGCTGG-ATCAAAGATGTTTCTTCT-TTGCATTTCTTG-CGATTGTT--TTTCCATG------AATATCATAATT---------------TGAATAGTCTCAT---TACT-TC---------------AAATAAATCCACTTACGTCT-TTTCAAAAAG---AAAGAAAAGAT-TCT-TTTGG-TTCCTACATA------ATTCTTAT-GTATAT-GAATGC----GAATATCTAT-TCCTG---TTTCTTCGT--AAAAAG-TCTTCT-TATT-TACG-AT-CAATAT-CTTCTGGA-GTTTTTCTT-GAGCG-AACACATTT--CTATGGAAA--AATAGAA------TATCTTATAGT---CGTGTGTT-GTAATT------CTTTTCA---------GAGT-ATCCTATGGT-TCCTCAA-AGATACT-TTCA-TACATTAT------------GTTC-----GATATCAAGGAAAA---GCA-----ATTCT--GGCTTC-----AAAAG---GAACTCTT---------ATTC------------TGATGAAGAAAT-GGAAATTT--CATCTT-GTGAA-TTTTTGGCA-ATC--TTATT-TTCACTTT-TGGTTTC--AACCTTATAGGA----TCTATATAAA-GCAATT--ACCCAACTATTCCTTCTCTTTTCTGGGA-TATTTTTCAAGTGTACTAAAAAA-TCCTTTGGT-AGTAA-GAAATC-AAATGCTAGAGAATTCATTT-CTAATAAATACTCT-GACTAAGA-AATTAGATACCGTA-GCCCCAGTT--ATTTCTCTTATTGG-ATCATTGTCGAAAGCTCA-ATTTTGTACTGTATTGGGT-CATCCTATTAGTAAACC-GACCTGGACCAATTTATCGGATTCTGA-------------TATTCTTGATCGATTTTGTCGGAT-----ATGTAGAAAT---CTTTGTCGT-TATCACAGCGGATCCTC-AAAGAA--------ACAGGTTTTGTAT-----------------------------------------------------------------------------------------------------------------------------------------------------

Bletilla_striata_KF673785 ACAAGAATTCTTTTTCTTCTCATTTTTCT------------TCTCAAATGGTATCAGAAGGTTTTGGAGTCATTCTGGAAATTCCATTCTCGTCGCGATTAGTATCT---------TCCCTTGAAG---AAAAAAGAATACCAAAATCTCAGAATTTACGATCTATTCATTCAATATTTCCCTTTTTAGAGGATAAATTATCACATTTAAATTATGTGTCAG-ATC-TACT-AATACCCCATCCCATACATC-TGGAAATCTT-GGTTCAAATCCTTCAATGCTGG-ATCAAAGATGTTTCTTCT-TTGCATTTCTTG-CGATTGTT--TTTCCATG------AATATCATAATT---------------TGAATAGTCTCAT---TACT-TC---------------AAATAAATCCACTTACGTCT-TTTCAAAAAG---AAAGAAAAGAT-TCT-TTTGG-TTCCTACATA------ATTCTTAT-GTATAT-GAATGC----GAATATCTAT-TCCTG---TTTCTTCGT--AAAAAG-TCTTCT-TATT-TACG-AT-CAATAT-CTTCTGGA-GTTTTTCTT-GAGCG-AACACATTT--CTATGGAAA--AATAGAA------TATCTTATAGT---CGTGTGTT-GTAATT------CTTTTCA---------GAGT-ATCCTATGGT-TCCTCAA-AGATACT-TTCA-TACATTAT------------GTTC-----GATATCAAGGAAAA---GCA-----ATTCT--GGCTTC-----AAAAG---GAACTCTT---------ATTC------------TGATGAAGAAAT-GGAAATTT--CATCTT-GTGAA-TTTTTGGCA-ATC--TTATT-TTCACTTT-TGGTTTC--AACCTTATAGGA----TCTATATAAA-GCAATT--ACCCAACTATTCCTTCTCTTTTCTGGGA-TATTTTTCAAGTGTACTAAAAAA-TCCTTTGGT-AGTAA-GAAATC-AAATGCTAGAGAATTCATTT-CTAATAAAGACTCT-GACTAAGA-AATTAGATACCGTA-GCCCCAGTT--ATTTCTCTTATTGG-ATCATTGTCGAAAGCTCA-ATTTTGTACTGTATTGGGT-CATCCTATTAGTAAACC-GACCTGGACCAATTTATCGGATTCTGA-------------TATTCTTGATCGATTTTGTCGGAT-----ATGTAGAAAT---CTTTGTCGT-TATCACAGCGGATCCTC-AAAGAA--------ACAGGTTTTGTATCGTATAAAGTA-TATACTTCGACTTTCGTGTGCTAG-AACTTTGGCTCGTAAACATAAAAGTACAGTACGCACTTTTATGCGAAGATTAGGTTCGGGATTCTTAG-AAGAATTTTTTTT------GGAAGAAGAAC-AATCTCTTTCTT

Bletilla_striata_KF673786 ACAAGAATTCTTTTTCTTCTCATTTTTCT------------TCTCAAATGGTATCAGAAGGTTTTGGAGTCATTCTGGAAATTCCATTCTCGTCGCGATTAGTATCT---------TCCCTTGAAG---AAAAAAGAATACCAAAATCTCAGAATTTACGATCTATTCATTCAATATTTCCCTTTTTAGAGGATAAATTATCACATTTAAATTATGTGTCAG-ATC-TACT-AATACCCCATCCCATACATC-TGGAAATCTT-GGTTCAAATCCTTCAATGCTGG-ATCAAAGATGTTTCTTCT-TTGCATTTCTTG-CGATTGTT--TTTCCATG------AATATCATAATT---------------TGAATAGTCTCAT---TACT-TC---------------AAATAAATCCACTTACGTCT-TTTCAAAAAG---AAAGAAAAGAT-TCT-TTTGG-TTCCTACATA------ATTCTTAT-GTATAT-GAATGC----GAATATCTAT-TCCTG---TTTCTTCGT--AAAAAG-TCTTCT-TATT-TACG-AT-CAATAT-CTTCTGGA-GTTTTTCTT-GAGCG-AACACATTT--CTATGGAAA--AATAGAA------TATCTTATAGT---CGTGTGTT-GTAATT------CTTTTCA---------GAGT-ATCCTATGGT-TCCTCAA-AGATACT-TTCA-TACATTAT------------GTTC-----GATATCAAGGAAAA---GCA-----ATTCT--GGCTTC-----AAAAG---GAACTCTT---------ATTC------------TGATGAAGAAAT-GGAAATTT--TATCTT-GTGAA-TTTTTGGCA-ATC--TTATT-TTCACTTT-TGGTTTC--AACCTTATAGGA----TCTATATAAA-GCAATT--ACCCAACTATTCCTTCTCTTTTCTGGGA-TATTTTTCAAGTGTACTAAAAAA-TCCTTTGGT-AGTAA-GAAATC-AAATGCTAGATAATTCATTT-CTAATAAATACTCT-GACTAAGA-AATTAGATACCGTA-GCCCCAGTT--ATTTCTCTTATTGG-ATCATTGTCGAAAGCTCA-ATTTTGTACTGTATTGGGT-CATCCTATTAGTAAACC-GATCTGGACCGATTTATCGGATTCTGA-------------TATTCTTGATCGATTTTGTCGGAT-----ATGTAGAAAT---CTTTGTCGT-TATCACAGCGGATCCTC-AAAGAA--------ACAGGTTTTGTATCGTATAAAGTA-TATACTTCGACTTTCGTGTGCTAG-AACTTTGGCTCGTAAACATAAAAGTACAGTACGCACTTTTATGCGAAGATTAGGTTCGGGATTCTTAG-AAGAATTTTTTTT------GGAAGAAGAAC-AATCTCTTTCTT

Bletilla_striata_KFBG2048 ACAAGAATTCTTTTTCTTCTCATTTTTCT------------TCTCAAATGGTATCAGAAGGTTTTGGAGTCATTCTGGAAATTCCATTCTCGTCGCGATTAGTATCT---------TCCCTTGAAG---AAAAAAGAATACCAAAATCTCAGAATTTACGATCTATTCATTCAATATTTCCCTTTTTAGAGGATAAATTATCACATTTAAATTATGTGTCAG-ATC-TACT-AATACCCCATCCCATACATC-TGGAAATCTT-GGTTCAAATCCTTCAATGCTGG-ATCAAAGATGTTTCTTCT-TTGCATTTCTTG-CGATTGTT--TTTCCATG------AATATCATAATT---------------TGAATAGTCTCAT---TACT-TC---------------AAATAAATCCACTTACGTCT-TTTCAAAAAG---AAAGAAAAGAT-TCT-TTTGG-TTCCTACATA------ATTCTTAT-GTATAT-GAATGC----GAATATCTAT-TCCTG---TTTCTTCGT--AAAAAG-TCTTCT-TATT-TACG-AT-CAATAT-CTTCTGGA-GTTTTTCTT-GAGCG-AACACATTT--CTATGGAAA--AATAGAA------TATCTTATAGT---CGTGTGTT-GTAATT------CTTTTCA---------GAGT-ATCCTATGGT-TCCTCAA-AGATACT-TTCA-TACATTAT------------GTTC-----GATATCAAGGAAAA---GCA-----ATTCT--GGCTTC-----AAAAG---GAACTCTT---------ATTC------------TGATGAAGAAAT-GGAAATTT--TATCTT-GTGAA-TTTTTGGCA-ATC--TTATT-TTCACTTT-TGGTTTC--AACCTTATAGGA----TCTATATAAA-GCAATT--ACCCAACTATTCCTTCTCTTTTCTGGGA-TATTTTTCAAGTGTACTAAAAAA-TCCTTTGGT-AGTAA-GAAATC-AAATGCTAGATAATTCATTT-CTAATAAATACTCT-GACTAAGA-AATTAGATACCGTA-GCCCCAGTT--ATTTCTCTTATTGG-ATCATTGTCGAAAGCTCA-ATTTTGTACTGTATTGGGT-CATCCTATTAGTAAACC-GATCTGGACCGATTTATCGGATTCTGA-------------TATTCTTGATCGATTTTGTCGGAT-----ATGTAGAAAT---CTTTGTCGT-TATCACAGCGGATCCTC-AAAGAA--------ACAGGTTTTGTATCGTATAAAGTA-TATTCTTCGACTTTCGTGTGCTAG-AACTTTGGCTCGTAAACATAAAAGTACAGTACGCACTTTTATGCGAAGATTAGGTTCGGGATTCTTAG-AAGAATATTTTC-------GGAAGA------------------
[truncated: 2,695,909 more chars]
