# Supplementary material for: Hong Kong orchids on the EDGE: a phylogenetic framework for conservation planning, trade mitigation and population rescue
Source: Front Plant Sci. 2026 Apr 28;17:1801915. doi: 10.3389/fpls.2026.1801915 (PMC13161184; doi:10.3389/fpls.2026.1801915)
Supplement: Supplementary file 3 [file Table3.docx]

S3A: 5.8S matrix

#NEXUS

begin taxa;

dimensions ntax=912;

taxlabels

Acampe_praemorsa_var_longepedunculata_AB217526

Acampe_praemorsa_var_longepedunculata_KJ733385

Acampe_praemorsa_var_longepedunculata_KY966408

Acampe_praemorsa_var_longepedunculata_PK12086

Acampe_praemorsa_var_longepedunculata_PK12159

Acampe_praemorsa_var_longepedunculata_SG1199

Acanthophippium_gougahense_KFBG3161

Acanthophippium_gougahensis_KY966409

Ania_hongkongensis_KF560537

Ania_hongkongensis_KFBG4612

Ania_hongkongensis_KFBG4613

Ania_hongkongensis_KM025146

Ania_hongkongensis_KM025147

Ania_hongkongensis_KM025148

Ania_hongkongensis_KY966415

Ania_hongkongensis_PK12027

Ania_hongkongensis_SG1231

Ania_hongkongensis_SG1343

Ania_ruybarrettoi_KFBG43

Ania_ruybarrettoi_KM025152

Ania_ruybarrettoi_KY966416

Ania_ruybarrettoi_SG1395

Anoectochilus_formosanus_AY052780

Anoectochilus_formosanus_GQ328777

Anoectochilus_formosanus_GQ396668

Anoectochilus_formosanus_KR815833

Anoectochilus_formosanus_KR815839

Anoectochilus_formosanus_PK12215

Anoectochilus_roxburghii_EU817408

Anoectochilus_roxburghii_GQ328774

Anoectochilus_roxburghii_KF425501

Anoectochilus_roxburghii_KF425502

Anoectochilus_roxburghii_KF425503

Anoectochilus_roxburghii_KR815828

Anoectochilus_roxburghii_KR815829

Anoectochilus_roxburghii_KR815830

Anoectochilus_roxburghii_KR815831

Anoectochilus_roxburghii_KR815832

Anoectochilus_roxburghii_KR815836

Anoectochilus_roxburghii_KR815837

Anoectochilus_roxburghii_KR815838

Anoectochilus_roxburghii_KY966417

Anoectochilus_roxburghii_PK12043

Anoectochilus_roxburghii_PK12044

Anoectochilus_roxburghii_PK12068

Anoectochilus_roxburghii_PK12069

Anoectochilus_roxburghii_SG1219

Aphyllorchis_montana_FJ454867

Aphyllorchis_montana_PK12147

Aphyllorchis_montana_PK12148

Aphyllorchis_montana_SG1010

Apostasia_nipponica_PK12273

Apostasia_nipponica_PK12274

Appendicula_cornuta_AF521073

Appendicula_cornuta_KY239261

Appendicula_cornuta_KY966419

Appendicula_cornuta_PK12064

Appendicula_cornuta_PK12065

Arundina_graminifolia_AF273335

Arundina_graminifolia_AF302727

Arundina_graminifolia_AF461461

Arundina_graminifolia_JN114437

Arundina_graminifolia_JN114438

Arundina_graminifolia_JN114439

Arundina_graminifolia_JN114440

Arundina_graminifolia_KY966420

Arundina_graminifolia_SG1008

Arundina_graminifolia_SG1206

Arundina_graminifolia_SG1295

Bletilla_striata_KF560509

Bletilla_striata_KF560540

Brachycorythis_galeandra_PK12195

Brachycorythis_galeandra_SG1261

Bulbophyllum_affine_EF195916

Bulbophyllum_affine_KC568305

Bulbophyllum_affine_KF866246

Bulbophyllum_affine_KFBG412

Bulbophyllum_affine_KX455815

Bulbophyllum_affine_KY966422

Bulbophyllum_affine_MK164425

Bulbophyllum_affine_SG1606

Bulbophyllum_affine_SG1607

Bulbophyllum_ambrosia_JN619413

Bulbophyllum_ambrosia_KC568306

Bulbophyllum_ambrosia_KY966424

Bulbophyllum_ambrosia_KY966425

Bulbophyllum_ambrosia_KY966426

Bulbophyllum_ambrosia_MK164427

Bulbophyllum_ambrosia_PK12090

Bulbophyllum_ambrosia_PK12092

Bulbophyllum_ambrosia_SG1221

Bulbophyllum_bicolor_CL10

Bulbophyllum_bicolor_FT28

Bulbophyllum_bicolor_KFBG2210

Bulbophyllum_bicolor_KFBG3073

Bulbophyllum_bicolor_KFBG433A

Bulbophyllum_bicolor_KFBG445

Bulbophyllum_bicolor_KY022455

Bulbophyllum_bicolor_KY966430

Bulbophyllum_bicolor_KY966431

Bulbophyllum_bicolor_KY966432

Bulbophyllum_bicolor_LMP19

Bulbophyllum_bicolor_MK164432

Bulbophyllum_bicolor_MK164433

Bulbophyllum_bicolor_PSL45

Bulbophyllum_bicolor_TMS05

Bulbophyllum_bicolor_TT43

Bulbophyllum_delitescens_KY966439

Bulbophyllum_delitescens_KY966440

Bulbophyllum_delitescens_KY966441

Bulbophyllum_delitescens_MK164448

Bulbophyllum_delitescens_SG1286

Bulbophyllum_delitescens_SG1287

Bulbophyllum_delitescens_SG1288

Bulbophyllum_kwangtungense_JN619414

Bulbophyllum_kwangtungense_KFBG27001

Bulbophyllum_kwangtungense_KFBG2798

Bulbophyllum_kwangtungense_KFBG2820

Bulbophyllum_kwangtungense_MK164466

Bulbophyllum_kwangtungense_PK12070

Bulbophyllum_odoratissimum_HQ114230

Bulbophyllum_odoratissimum_KF866242

Bulbophyllum_odoratissimum_KY966466

Bulbophyllum_odoratissimum_KY966467

Bulbophyllum_odoratissimum_MK164483

Bulbophyllum_odoratissimum_PK12119

Bulbophyllum_odoratissimum_SG1275

Bulbophyllum_pectenveneris_JN619418

Bulbophyllum_pectenveneris_KY966470

Bulbophyllum_pectenveneris_KY966471

Bulbophyllum_pectenveneris_MK164486

Bulbophyllum_scabratum_KY966453

Bulbophyllum_scabratum_MK164471

Bulbophyllum_scabratum_PK12041

Bulbophyllum_scabratum_PK12126

Bulbophyllum_scabratum_PK12127

Bulbophyllum_stenobulbon_KFBG2806

Bulbophyllum_stenobulbon_MK164513

Bulbophyllum_stenobulbon_PK12111

Bulbophyllum_stenobulbon_PK12112

Bulbophyllum_stenobulbon_PK12113

Bulbophyllum_stenobulbon_SG1226

Bulbophyllum_tigridum_KFBG468

Bulbophyllum_tigridum_KX455820

Bulbophyllum_tigridum_MK164520

Bulbophyllum_tigridum_SG1310

Bulbophyllum_tseanum_MK164524

Bulbophyllum_tseanum_SG1272

Bulbophyllum_tseanum_SG1616

Calanthe_graciliflora_AY882608

Calanthe_graciliflora_KF560484

Calanthe_graciliflora_KF560495

Calanthe_graciliflora_PK12206

Calanthe_graciliflora_PK12207

Calanthe_graciliflora_SG1225

Calanthe_masuca_KFBG11

Calanthe_masuca_PK12110

Calanthe_masuca_SG026

Calanthe_masuca_SG1360

Calanthe_masuca_SG1361

Calanthe_speciosa_KFBG136

Calanthe_speciosa_KY951546

Calanthe_speciosa_PK12168

Calanthe_speciosa_PK12169

Calanthe_speciosa_SG1313

Calanthe_speciosa_SG1368

Calanthe_triplicata_AY882614

Calanthe_triplicata_KF560480

Calanthe_triplicata_KF560491

Calanthe_triplicata_KFBG601

Calanthe_triplicata_KM025154

Calanthe_triplicata_KY966491

Calanthe_triplicata_SG1311

Cephalantheropsis_obcordata_KF560490

Cephalantheropsis_obcordata_KFBG2520

Cephalantheropsis_obcordata_KY966494

Cephalantheropsis_obcordata_KY966495

Cephalantheropsis_obcordata_PK12079

Cephalantheropsis_obcordata_PK12080

Cephalantheropsis_obcordata_PK12081

Cephalantheropsis_obcordata_SG1208

Cephalantheropsis_obcordata__KFBG140

Cephalantheropsis_obcordata__SG1209

Cheirostylis_clibborndyeri_KY966496

Cheirostylis_clibborndyeri_PK12096

Cheirostylis_clibborndyeri_SG1349

Cheirostylis_monteiroi_PK12093

Cheirostylis_monteiroi_PK12094

Cheirostylis_monteiroi_SG1344

Cheirostylis_yunnanensis_KT343978

Cheirostylis_yunnanensis_PK12097

Cheirostylis_yunnanensis_SG1227

Cheirostylis_yunnanensis_SG1228

Cheirostylis_yunnanensis_SG1229

Chrysoglossum_assamicum_SG1622

Chrysoglossum_assamicum_SG1623

Cleisostoma_paniculatum_KFBG516

Cleisostoma_paniculatum_KJ733401

Cleisostoma_paniculatum_KT223752

Cleisostoma_rostratum_KFBG789

Cleisostoma_rostratum_KJ733404

Cleisostoma_rostratum_KY966502

Cleisostoma_rostratum_PK12089

Cleisostoma_rostratum_PK12101

Cleisostoma_rostratum_PK12158

Cleisostoma_rostratum_SG1200

Cleisostoma_rostratum_SG1301

Cleisostoma_simondii_KJ733405

Cleisostoma_simondii_KY966503

Cleisostoma_simondii_KY966504

Cleisostoma_simondii_MG822849

Cleisostoma_simondii_PK12176

Cleisostoma_simondii_SG1314

Cleisostoma_simondii_SG1327

Cleisostoma_simondii_SG1328

Cleisostoma_simondii_SG1329

Cleisostoma_simondii_SG1330

Cleisostoma_simondii_var_guangdongense_KFBG2212

Cleisostoma_simondii_var_guangdongense_KJ733406

Coelogyne_cantonensis_KY966649

Coelogyne_cantonensis_SG1239

Coelogyne_cantonensis_SG1339

Coelogyne_cantonensis_SG1388

Coelogyne_chinensis_EU592035

Coelogyne_chinensis_KY966650

Coelogyne_chinensis_KY966651

Coelogyne_chinensis_PK12114

Coelogyne_chinensis_PK12115

Coelogyne_chinensis_SG1232

Coelogyne_chinensis_SG1251

Coelogyne_fimbriata_AF302745

Coelogyne_fimbriata_EU441205

Coelogyne_fimbriata_JF422074

Coelogyne_fimbriata_KFBG411

Coelogyne_fimbriata_KFBG523

Coelogyne_fimbriata_KR857330

Coelogyne_fimbriata_KY966506

Coelogyne_fimbriata_SG1059

Coelogyne_fimbriata_SG1061F

Coelogyne_fimbriata_SG1062D

Coelogyne_fimbriata_SG1079

Coelogyne_fimbriata_var_leungiana_KFBG640

Coelogyne_fimbriata_var_leungiana_KY966507

Coelogyne_fimbriata_var_leungiana_SG1058A

Collabium_chinense_KF560544

Collabium_chinense_KM025156

Crepidium_acuminatum_AB290884

Crepidium_acuminatum_JN114478

Crepidium_acuminatum_JN114479

Crepidium_acuminatum_JN114480

Crepidium_acuminatum_JN114481

Crepidium_acuminatum_JN114482

Crepidium_acuminatum_KJ459274

Crepidium_acuminatum_KX277725

Crepidium_acuminatum_MF287967

Crepidium_allanii_KFBG4610

Crepidium_allanii_KFBG4611

Crepidium_purpureum_SG1193

Crepidium_purpureum_SG1194

Crepidium_purpureum_SG1379

Cryptochilus_roseus_KFBG2118

Cryptochilus_roseus_KY239358

Cryptochilus_roseus_KY966513

Cryptochilus_roseus_KY966514

Cryptochilus_roseus_PK12087

Cryptochilus_roseus_PK12088

Cryptochilus_roseus_PK12187

Cryptostylis_arachnites_PK12188

Cryptostylis_arachnites_SG027

Cryptostylis_arachnites_SG1142

Cryptostylis_arachnites_SG1380

Cymbidium_aloifolium_AF284695

Cymbidium_aloifolium_JF729014

Cymbidium_aloifolium_JN114485

Cymbidium_aloifolium_JN114486

Cymbidium_aloifolium_KFBG2049

Cymbidium_aloifolium_KFBG2205

Cymbidium_ensifolium_AF284716

Cymbidium_ensifolium_AF284717

Cymbidium_ensifolium_AF470496

Cymbidium_ensifolium_AF470512

Cymbidium_ensifolium_KJ597843

Cymbidium_ensifolium_KJ597844

Cymbidium_ensifolium_KJ597845

Cymbidium_ensifolium_KJ597846

Cymbidium_ensifolium_KT338675

Cymbidium_ensifolium_PK12208

Cymbidium_ensifolium_SG1214

Cymbidium_ensifolium_SG1278

Cymbidium_ensifolium_SG1285

Cymbidium_kanran_AF284720

Cymbidium_kanran_AF470495

Cymbidium_kanran_JX202654

Cymbidium_kanran_KF560534

Cymbidium_kanran_KJ597836

Cymbidium_kanran_KJ597837

Cymbidium_kanran_KJ597838

Cymbidium_kanran_KJ597839

Cymbidium_kanran_KT338677

Cymbidium_lancifolium_AF284724

Cymbidium_lancifolium_AF470520

Cymbidium_lancifolium_KFBG734

Cymbidium_lancifolium_KJ597851

Cymbidium_lancifolium_KJ597852

Cymbidium_lancifolium_KY966515

Cymbidium_lancifolium_PK12128

Cymbidium_lancifolium_SG1274

Cymbidium_sinense_AF284714

Cymbidium_sinense_AF284715

Cymbidium_sinense_KJ597847

Cymbidium_sinense_KJ597848

Cymbidium_sinense_KJ597849

Cymbidium_sinense_KJ597850

Cymbidium_sinense_SG1213

Cymbidium_sinense_SG1218

Cymbidium_sinense_SG1341

Cymbidium_sinense_SG1342

Dendrobium_aduncum_AB593484

Dendrobium_aduncum_GU339110

Dendrobium_aduncum_HM590372

Dendrobium_aduncum_HQ114250

Dendrobium_aduncum_JF713083

Dendrobium_aduncum_JN388580

Dendrobium_aduncum_KC346887

Dendrobium_aduncum_KC568295

Dendrobium_aduncum_KF143428

Dendrobium_aduncum_KFBG8766

Dendrobium_aduncum_KJ210409

Dendrobium_aduncum_KJ210410

Dendrobium_aduncum_KJ210411

Dendrobium_aduncum_KJ210412

Dendrobium_aduncum_KJ672619

Dendrobium_aduncum_KP159298

Dendrobium_aduncum_KR075042

Dendrobium_aduncum_KX600499

Dendrobium_anosmum_AB593499

Dendrobium_anosmum_AB972339

Dendrobium_anosmum_EU477499

Dendrobium_anosmum_JN388570

Dendrobium_anosmum_KJ672650

Dendrobium_anosmum_KJ944630

Dendrobium_anosmum_KP743542

Dendrobium_anosmum_KP743544

Dendrobium_anosmum_KY966516

Dendrobium_cf_mimicum_PK12237E

Dendrobium_cf_mimicum_PK12237J

Dendrobium_crumenatum_AB593537

Dendrobium_crumenatum_AB972336

Dendrobium_crumenatum_AF521608

Dendrobium_crumenatum_AY239963

Dendrobium_crumenatum_AY273708

Dendrobium_crumenatum_EU840700

Dendrobium_crumenatum_HM054625

Dendrobium_crumenatum_HM590370

Dendrobium_crumenatum_JF713095

Dendrobium_crumenatum_JF713096

Dendrobium_crumenatum_JN388587

Dendrobium_crumenatum_KC507780

Dendrobium_crumenatum_KJ672661

Dendrobium_linawianum_AB593599

Dendrobium_linawianum_AF521613

Dendrobium_linawianum_EU003115

Dendrobium_linawianum_EU003117

Dendrobium_linawianum_HM590371

Dendrobium_linawianum_JN388573

Dendrobium_linawianum_KJ672629

Dendrobium_linawianum_KP159299

Dendrobium_linawianum_KT779776

Dendrobium_linawianum_KY966557

Dendrobium_linawianum_SG1347

Dendrobium_lindleyi_AB593600

Dendrobium_lindleyi_AB972341

Dendrobium_lindleyi_DQ058784

Dendrobium_lindleyi_GU339114

Dendrobium_lindleyi_HM054672

Dendrobium_lindleyi_HM054673

Dendrobium_lindleyi_JF713110

Dendrobium_lindleyi_JN388568

Dendrobium_lindleyi_KC568301

Dendrobium_lindleyi_KFBG203

Dendrobium_lindleyi_KJ210461

Dendrobium_lindleyi_KJ672682

Dendrobium_lindleyi_KX522640

Dendrobium_lindleyi_KY966558

Dendrobium_lindleyi_KY966559

Dendrobium_lindleyi_KY966560

Dendrobium_loddigesii_AB593604

Dendrobium_loddigesii_AB873183

Dendrobium_loddigesii_AF311778

Dendrobium_loddigesii_AY485703

Dendrobium_loddigesii_EU121418

Dendrobium_loddigesii_EU592016

Dendrobium_loddigesii_HM590374

Dendrobium_loddigesii_HQ114220

Dendrobium_loddigesii_JN388569

Dendrobium_loddigesii_KC205187

Dendrobium_loddigesii_KF143481

Dendrobium_loddigesii_KJ672683

Dendrobium_loddigesii_KP159301

Dendrobium_loddigesii_KP264997

Dendrobium_loddigesii_KT778746

Dendrobium_loddigesii_KY966562

Dendrobium_loddigesii_SG1255

Dendrobium_spatella_AB847671

Dendrobium_spatella_KF143512

Dendrobium_spatella_KF143513

Dendrobium_spatella_SG1357

Dendrobium_spatella_SG1358

Dendrolirium_lasiopetalum_KFBG701

Dendrolirium_lasiopetalum_PK12173

Dendrolirium_lasiopetalum_PK12174

Dendrolirium_lasiopetalum_SG1312

Didymoplexiella_siamensis_PK12024

Didymoplexiella_siamensis_SG1242

Dienia_ophrydis_AY907111

Dienia_ophrydis_AY907114

Dienia_ophrydis_KFBG7819

Dienia_ophrydis_KJ459275

Dienia_ophrydis_MF287970

Dienia_ophrydis_SG1201

Dienia_ophrydis_SG1254

Dienia_ophrydis_SG1276

Diploprora_championii_KFBG628

Diploprora_championii_KJ733410

Diploprora_championii_KY966590

Diploprora_championii_KY966591

Diploprora_championii_PK12025

Diploprora_championii_PK12095

Diploprora_championii_SG1144

Diploprora_championii_SG1230

Epipogium_roseum_EU711232

Epipogium_roseum_SG1249

Epipogium_roseum_SG1250

Eria_scabrilinguis_KFBG916

Eria_scabrilinguis_KY239252

Eria_scabrilinguis_KY966596

Eria_scabrilinguis_PK12164

Eria_scabrilinguis_PK12165

Eria_scabrilinguis_SG1302

Erythrodes_blumei_JN166066

Erythrodes_blumei_KT343981

Erythrodes_blumei_KT343982

Erythrodes_blumei_PK12103

Erythrodes_blumei_PK12104

Eulophia_flava_JN114508

Eulophia_flava_JN114509

Eulophia_flava_SG1158

Eulophia_flava_SG1159

Eulophia_graminea_FJ565666

Eulophia_graminea_KF318890

Eulophia_graminea_MH768268

Eulophia_graminea_MH768269

Eulophia_graminea_SG1270

Eulophia_graminea_SG1350

Eulophia_picta_JN114510

Eulophia_picta_JN114511

Eulophia_picta_JN114512

Eulophia_picta_JN114513

Eulophia_picta_PK12045

Eulophia_picta_PK12046

Eulophia_picta_PK12047

Eulophia_picta_PK12048

Eulophia_picta_PK12137

Eulophia_picta_PK12138

Eulophia_picta_PK12153

Eulophia_picta_SG1271

Eulophia_zollingeri_AB306313

Eulophia_zollingeri_SG1262

Eulophia_zollingeri_SG1263

Eulophia_zollingeri_SG1264

Eulophia_zollingeri_SG1265

Eulophia_zollingeri_SG1353

Gastrochilus_japonicus_AY228503

Gastrochilus_japonicus_KY966598

Gastrochilus_japonicus_KY966599

Goodyera_foliosa_HM140995

Goodyera_foliosa_KT344001

Goodyera_foliosa_KT344002

Goodyera_foliosa_KT344003

Goodyera_foliosa_KT344004

Goodyera_foliosa_KT344005

Goodyera_foliosa_KT344006

Goodyera_foliosa_KT344007

Goodyera_foliosa_KT344008

Goodyera_foliosa_KT344009

Goodyera_foliosa_PK12067

Goodyera_foliosa_SG1309

Goodyera_foliosa_SG1315

Goodyera_foliosa_var_foliosa_KC205157

Goodyera_foliosa_var_laevis_HM140998

Goodyera_procera_HM151402

Goodyera_procera_HM222488

Goodyera_procera_JN114514

Goodyera_procera_JN114515

Goodyera_procera_JN114516

Goodyera_procera_JN114517

Goodyera_procera_JN114518

Goodyera_procera_KC237319

Goodyera_procera_KT344043

Goodyera_procera_KT344044

Goodyera_procera_KT344045

Goodyera_procera_KY966601

Goodyera_procera_SG1152

Goodyera_procera_SG1240

Goodyera_procera_SG1241

Goodyera_procera_SG1346

Goodyera_pusilla_KM593694

Goodyera_pusilla_KT344046

Goodyera_pusilla_KT344047

Goodyera_seikomontana_KT344068

Goodyera_seikomontana_KT344069

Goodyera_seikomontana_KY966602

Goodyera_seikoomontana_SG1252

Goodyera_seikoomontana_SG1253

Goodyera_seikoomontana_SG1354

Goodyera_viridiflora_JN166067

Goodyera_viridiflora_KC205154

Goodyera_viridiflora_KT344078

Goodyera_viridiflora_KT344079

Goodyera_viridiflora_KT344080

Goodyera_viridiflora_KT344081

Goodyera_viridiflora_KT344082

Goodyera_viridiflora_KT344083

Goodyera_viridiflora_KT344084

Goodyera_viridiflora_KT344085

Goodyera_viridiflora_PK12170

Goodyera_viridiflora_PK12172

Goodyera_viridiflora_SG1305

Goodyera_viridiflora_SG1306

Habenaria_ciliolaris_MF944286

Habenaria_ciliolaris_MF944287

Habenaria_dentata_KFBG1009

Habenaria_dentata_KFBG2126B

Habenaria_dentata_KJ460038

Habenaria_dentata_KY966605

Habenaria_dentata_PK12058

Habenaria_dentata_PK12059

Habenaria_dentata_SG1005

Habenaria_leptoloba_KY055535

Habenaria_leptoloba_PK12060

Habenaria_leptoloba_PK12061

Habenaria_leptoloba_PK12160

Habenaria_leptoloba_PK12161

Habenaria_leptoloba_SG1304

Habenaria_leptoloba_SG1362

Habenaria_linguella_MF944303

Habenaria_linguella_PK12049

Habenaria_linguella_PK12134

Habenaria_linguella_PK12141

Habenaria_linguella_SG1195

Habenaria_linguella_SG1299

Habenaria_linguella_SG1300

Habenaria_reniformis_PK12142

Habenaria_reniformis_PK12143

Habenaria_reniformis_PK12144

Habenaria_reniformis_PK12145

Habenaria_reniformis_PK12146

Habenaria_reniformis_SG1296

Habenaria_reniformis_SG1297

Habenaria_rhodocheila_KJ460043

Habenaria_rhodocheila_KR350167

Habenaria_rhodocheila_KY966607

Habenaria_rhodocheila_PK12139

Habenaria_rhodocheila_SG1289

Habenaria_rhodocheila_SG1290

Habenaria_rhodocheila_SG1291

Hetaeria_youngsayei_KY966608

Hetaeria_youngsayei_SG1244

Hetaeria_youngsayei_SG1245

Lecanorchis_nigricans_SG1279

Lecanorchis_nigricans_SG1280

Liparis_bootanensis_KFBG238

Liparis_bootanensis_KJ459280

Liparis_bootanensis_KY966611

Liparis_bootanensis_PK12162

Liparis_bootanensis_SG1215

Liparis_bootanensis_SG1216

Liparis_bootanensis_SG1307

Liparis_bootanensis_SG1336

Liparis_ferruginea_SG1156

Liparis_ferruginea_SG1266

Liparis_ferruginea_SG1267

Liparis_gigantea_PK12116

Liparis_gigantea_PK12117

Liparis_gigantea_PK12118

Liparis_nervosa_AB289482

Liparis_nervosa_AY907092

Liparis_nervosa_JN114595

Liparis_nervosa_JN114596

Liparis_nervosa_JN114597

Liparis_nervosa_JN114598

Liparis_nervosa_JN114599

Liparis_nervosa_JN114600

Liparis_nervosa_JN114601

Liparis_nervosa_JN114602

Liparis_nervosa_JN114603

Liparis_nervosa_JN114604

Liparis_nervosa_KFBG330

Liparis_nervosa_KJ459294

Liparis_nervosa_KT338746

Liparis_nervosa_KT338747

Liparis_nervosa_SG1233

Liparis_nervosa_SG1234

Liparis_nervosa_SG1235

Liparis_odorata_SG1256

Liparis_odorata_SG1257

Liparis_sootenzanensis_KJ021034

Liparis_sootenzanensis_SG1351

Liparis_sootenzanensis_SG1352

Liparis_stricklandiana_KF589873

Liparis_stricklandiana_KFBG124

Liparis_stricklandiana_KFBG818

Liparis_stricklandiana_KJ459298

Liparis_stricklandiana_KY966613

Liparis_stricklandiana_KY966614

Liparis_stricklandiana_PK12091

Liparis_stricklandiana_SG1332

Liparis_stricklandiana_SG1333

Liparis_stricklandiana_SG1337

Liparis_viridiflora_KJ459299

Liparis_viridiflora_KY966615

Liparis_viridiflora_KY966616

Liparis_viridiflora_PK12120

Liparis_viridiflora_SG1308

Liparis_viridiflora_SG1338

Ludisia_discolor_AJ539483

Ludisia_discolor_EF590781

Ludisia_discolor_EF590782

Ludisia_discolor_JN166073

Ludisia_discolor_KR815834

Ludisia_discolor_KT344102

Ludisia_discolor_KY966617

Ludisia_discolor_SG1236

Ludisia_discolor_SG1237

Ludisia_discolor_SG1348

Nephelaphyllum_tenuiflorum_KF560535

Nephelaphyllum_tenuiflorum_KM025159

Nephelaphyllum_tenuiflorum_KY966621

Nephelaphyllum_tenuiflorum_PK12121

Nephelaphyllum_tenuiflorum_PK12122

Nephelaphyllum_tenuiflorum_PK12123

Nephelaphyllum_tenuiflorum_SG1220

Nervilia_plicata_AF324179

Nervilia_plicata_JN114618

Nervilia_plicata_JN114619

Nervilia_plicata_JN114620

Nervilia_plicata_MG452049

Nervilia_plicata_SG1143

Nervilia_plicata_SG1277

Neuwiedia_zollingeri_var_singapureana_PK12124

Neuwiedia_zollingeri_var_singapureana_SG1268

Neuwiedia_zollingeri_var_singapureana_SG1269

Neuwiedia_zollingeri_var_singapureana_SG1340

Neuwiedia_zollingeri_var_singapureana_JF796932

Neuwiedia_zollingeri_var_singapureana_KY966622

Pachystoma_pubescens_PK12107

Pachystoma_pubescens_PK12108

Paphiopedilum_purpuratum_AJ564364

Paphiopedilum_purpuratum_EF156131

Paphiopedilum_purpuratum_FJ899756

Paphiopedilum_purpuratum_JX088564

Paphiopedilum_purpuratum_KX931030

Paphiopedilum_purpuratum_PK12075

Paphiopedilum_purpuratum_PK12082A

Paphiopedilum_purpuratum_PK12083C

Paphiopedilum_purpuratum_SG1149

Paphiopedilum_purpuratum_Z78440

Pecteilis_susannae_MF944351

Pecteilis_susannae_MF944352

Pecteilis_susannae_PK12051

Pecteilis_susannae_PK12136

Pecteilis_susannae_PK12154

Pecteilis_susannae_SG1292

Peristylus_calcaratus_PK12052

Peristylus_calcaratus_PK12053

Peristylus_calcaratus_SG1303

Peristylus_densus_SG1258

Peristylus_densus_SG1260

Peristylus_goodyeroides_MF944361

Peristylus_goodyeroides_MF944362

Peristylus_intrudens_PK12050

Peristylus_intrudens_PK12056

Peristylus_intrudens_SG1298

Peristylus_lacertifer_MF944365

Peristylus_lacertifer_MF944366

Peristylus_lacertifer_PK12149

Peristylus_lacertifer_PK12163

Peristylus_lacertifer_SG1006

Peristylus_tentaculatus_KJ460035

Persitylus_tentaculatus_PK12062

Persitylus_tentaculatus_PK12171

Persitylus_tentaculatus_SG1007

Phaius_tancarvilleae_AB222032

Phaius_tancarvilleae_AB239286

Phaius_tancarvilleae_AB239287

Phaius_tancarvilleae_AB239288

Phaius_tancarvilleae_AB239289

Phaius_tancarvilleae_KF560503

Phaius_tancarvilleae_KF560531

Phaius_tancarvilleae_KM025161

Phaius_tancarvilleae_KY966645

Phaius_tancarvilleae_MG869015

Phaius_tankervilleae_PK12084

Phaius_tankervilleae_PK12085

Phaius_tankervilleae_PK12099

Phaius_tankervilleae_PK12100

Phaius_wallichii_KF560532

Phaius_wallichii_KY966646

Platanthera_minor_KJ460069

Platanthera_minor_KJ460079

Platanthera_minor_PK12030

Platanthera_minor_SG1154

Platanthera_minor_SG1223

Platanthera_minor_SG1224

Platanthera_minor_SG1238

Porpax_pusilla_KY239239

Porpax_pusilla_PK12071

Porpax_pusilla_PK12072

Porpax_pusilla_PK12125

Porpax_pusilla_SG1334

Rhomboda_abbreviata_KT344110

Rhomboda_abbreviata_KY966662

Rhomboda_abbreviata_PK12166

Rhomboda_abbreviata_PK12175

Rhomboda_abbreviata_SG1203

Robiquetia_succisa_KJ733444

Robiquetia_succisa_KY966667

Robiquetia_succisa_PK12155

Robiquetia_succisa_PK12156

Robiquetia_succisa_SG1293

Robiquetia_succisa_SG1294

Robiquetia_succisa_SG1345

Spathoglottis_pubescens_KM025162

Spathoglottis_pubescens_KP751405

Spathoglottis_pubescens_KP751406

Spathoglottis_pubescens_MG869012

Spathoglottis_pubescens_PK12135

Spathoglottis_pubescens_PK12140

Spathoglottis_pubescens_SG1202

Spathoglottis_pubescens_SG1205

Spiranthes_hongkongensis_MF286484

Spiranthes_hongkongensis_MF286511

Spiranthes_hongkongensis_MH002629

Spiranthes_hongkongensis_MH002630

Spiranthes_hongkongensis_MH002631

Spiranthes_hongkongensis_MH002632

Spiranthes_hongkongensis_MH002633

Spiranthes_hongkongensis_MH002634

Spiranthes_hongkongensis_MH002635

Spiranthes_hongkongensis_MH002636

Spiranthes_hongkongensis_MH002637

Spiranthes_hongkongensis_MH002638

Spiranthes_hongkongensis_MH002639

Spiranthes_hongkongensis_MH002640

Spiranthes_hongkongensis_MH002641

Spiranthes_hongkongensis_MH002642

Spiranthes_hongkongensis_MH002643

Spiranthes_hongkongensis_MH002644

Spiranthes_hongkongensis_MH002645

Spiranthes_hongkongensis_MH002646

Spiranthes_hongkongensis_MH002647

Spiranthes_hongkongensis_MH002648

Spiranthes_hongkongensis_MH002649

Spiranthes_hongkongensis_MH002650

Spiranthes_hongkongensis_MH002651

Spiranthes_hongkongensis_MH002652

Spiranthes_hongkongensis_MH002653

Spiranthes_hongkongensis_MH002654

Spiranthes_hongkongensis_MH002655

Spiranthes_hongkongensis_MH002656

Spiranthes_hongkongensis_MH002657

Spiranthes_hongkongensis_MH002658

Spiranthes_hongkongensis_MH002659

Spiranthes_hongkongensis_MH002660

Spiranthes_hongkongensis_MH002661

Spiranthes_hongkongensis_MH002662

Spiranthes_hongkongensis_MH002663

Spiranthes_hongkongensis_MH002664

Spiranthes_hongkongensis_MH002665

Spiranthes_hongkongensis_MH002666

Spiranthes_hongkongensis_MH002667

Spiranthes_hongkongensis_MH002668

Spiranthes_hongkongensis_MH002669

Spiranthes_hongkongensis_MH002670

Spiranthes_hongkongensis_MH002671

Spiranthes_hongkongensis_MH002672

Spiranthes_hongkongensis_MH002673

Spiranthes_hongkongensis_MH002674

Spiranthes_hongkongensis_MH002675

Spiranthes_hongkongensis_MH002676

Spiranthes_hongkongensis_MH002677

Spiranthes_hongkongensis_MH002678

Spiranthes_hongkongensis_MH002679

Spiranthes_hongkongensis_MH002680

Spiranthes_hongkongensis_MH002681

Spiranthes_hongkongensis_MH002682

Spiranthes_hongkongensis_MH002683

Spiranthes_hongkongensis_MH002684

Spiranthes_hongkongensis_MH002685

Spiranthes_hongkongensis_MH002686

Spiranthes_hongkongensis_MH002687

Spiranthes_hongkongensis_MH002688

Spiranthes_hongkongensis_MH002689

Spiranthes_hongkongensis_MH002690

Spiranthes_hongkongensis_MH002691

Spiranthes_hongkongensis_MH002692

Spiranthes_hongkongensis_MH002693

Spiranthes_hongkongensis_MH002694

Spiranthes_hongkongensis_MH002695

Spiranthes_hongkongensis_MH002696

Spiranthes_hongkongensis_MH002697

Spiranthes_hongkongensis_MH002698

Spiranthes_hongkongensis_MH038786

Spiranthes_hongkongensis_MH038787

Spiranthes_hongkongensis_PK12028

Spiranthes_hongkongensis_PK12102

Spiranthes_hongkongensis_PK12179

Spiranthes_sinensis_HE575518

Spiranthes_sinensis_KM262399

Spiranthes_sinensis_KM262400

Spiranthes_sinensis_KT338780

Spiranthes_sinensis_KT338781

Spiranthes_sinensis_MF286485

Spiranthes_sinensis_MF286486

Spiranthes_sinensis_MF286487

Spiranthes_sinensis_MF286488

Spiranthes_sinensis_MF286489

Spiranthes_sinensis_MF286493

Spiranthes_sinensis_MF286494

Spiranthes_sinensis_MF286495

Spiranthes_sinensis_MF286496

Spiranthes_sinensis_MF286497

Spiranthes_sinensis_MF286498

Spiranthes_sinensis_MF286499

Spiranthes_sinensis_MF286504

Spiranthes_sinensis_MF286505

Spiranthes_sinensis_MF286506

Spiranthes_sinensis_MF286507

Spiranthes_sinensis_MF286509

Spiranthes_sinensis_MF286510

Spiranthes_sinensis_MH005035

Spiranthes_sinensis_MH005036

Spiranthes_sinensis_MH005037

Spiranthes_sinensis_MH005038

Spiranthes_sinensis_MH005039

Spiranthes_sinensis_MH005040

Spiranthes_sinensis_MH005041

Spiranthes_sinensis_MH005042

Spiranthes_sinensis_MH005043

Spiranthes_sinensis_MH005044

Spiranthes_sinensis_MH005045

Spiranthes_sinensis_MH005046

Spiranthes_sinensis_MH005047

Spiranthes_sinensis_MH005048

Spiranthes_sinensis_MH005049

Spiranthes_sinensis_MH005050

Spiranthes_sinensis_MH005051

Spiranthes_sinensis_MH005052

Spiranthes_sinensis_MH005053

Spiranthes_sinensis_MH005054

Spiranthes_sinensis_MH005055

Spiranthes_sinensis_MH005056

Spiranthes_sinensis_MH005057

Spiranthes_sinensis_MH005058

Spiranthes_sinensis_MH005059

Spiranthes_sinensis_MH038785

Spiranthes_sinensis_MH802049

Spiranthes_sinensis_MH802050

Spiranthes_sinensis_PK12106

Spiranthes_sinensis_PK12109

Spiranthes_sinensis_SG1153

Spiranthes_sinensis_SG1155

Tainia_cordifolia_KF560538

Tainia_cordifolia_KM025163

Tainia_cordifolia_KM025164

Tainia_dunnii_KF560536

Tainia_dunnii_KM025165

Tainia_dunnii_SG1273

Thrixspermum_centipeda_AB217591

Thrixspermum_centipeda_KFBG3066D

Thrixspermum_centipeda_KJ733456

Thrixspermum_centipeda_KX679332

Thrixspermum_centipeda_KY966674

Thrixspermum_centipeda_KY966675

Thrixspermum_centipeda_PK12129

Thrixspermum_centipeda_PK12130

Thrixspermum_centipeda_PK12131

Thrixspermum_centipeda_PK12132

Thrixspermum_centipeda_PK12133

Tropidia_curculigoides_SG1281

Tropidia_curculigoides_SG1282

Tropidia_curculigoides_SG1283

Tropidia_curculigoides_SG1284

Tropidia_nipponica_PK12181

Tropidia_nipponica_SG1355

Tropidia_nipponica_SG1356

Vanilla_shenzhenica_JF796930

Vanilla_shenzhenica_KFBG290

Vrydagzynea_nuda_SG1222

Vrydagzynea_nuda_SG1246A

Vrydagzynea_nuda_SG1246B

Vrydagzynea_nuda_SG1247

Zeuxine_boninensisi_Z3

Zeuxine_boninensis_d16

Zeuxine_gracilis_JN166075

Zeuxine_gracilis_PK12057

Zeuxine_gracilis_PK12066

Zeuxine_gracilis_SG1204

Zeuxine_strateumatica_KT344117

Zeuxine_strateumatica_KY966688

Zeuxine_strateumatica_SG1211

Zeuxine_strateumatica_SG1212

;

end;

begin characters;

dimensions nchar=182;

format datatype=dna missing=? gap=-;

matrix

Acampe_praemorsa_var_longepedunculata_AB217526 ACGAC-TCTCGACAATGGATATCTCGGCTCTCGCATCGATGAAGAGCGCAGCGAAATGCGATACGTGGTGC-GAATTGCAGAATCCCGCGAACC-ATCGAGTCTTTGAACGCAAGTTGCGCCCGAGGCCAAT-CGGTCGAGGGCACGTCCGCCTG---GGCGTCA-AGCGTCGCGCCGCTCC

Acampe_praemorsa_var_longepedunculata_KJ733385 ACGAC-TCTCGACAATGGATATCTCGGCTCTCGCATCGATGAAGAGCGCAGCGAAATGCGATACGTGGTGC-GAATTGCAGAATCCCGCGAACC-ATCGAGTCTTTGAACGCAAGTTGCGCCCGAGGCCAAT-CGGTCGAGGGCACGTCCGCCTG---GGCGTCA-AGCGTTGCGCCGCTCC

Acampe_praemorsa_var_longepedunculata_KY966408 ACGAC-TCTCGACAATGGATATCTCGGCTCTCGCATCGATGAAGAGCGCAGCGAAATGCGATACGTGGTGC-GAATTGCAGAATCCCGCGAACC-ATCGAGTCTTTGAACGCAAGTTGCGCCCGAGGCCAAT-CGGTCGAGGGCACGTCCGCCTG---GGCGTCA-AGCGTTGCGCCGCTCC

Acampe_praemorsa_var_longepedunculata_PK12086 ACGAC-TCTCGACAATGGATATCTCGGCTCTCGCATCGATGAAGAGCGCAGCGAAATGCGATACGTGGTGC-GAATTGCAGAATCCCGCGAACC-ATCGAGTCTTTGAACGCAAGTTGCGCCCGAGGCCAAT-CGGTCGAGGGCACGTCCGCCTG---GGCGTCA-AGCGTTGCGCCGCTCC

Acampe_praemorsa_var_longepedunculata_PK12159 ACGAC-TCTCGACAATGGATATCTCGGCTCTCGCATCGATGAAGAGCGCAGCGAAATGCGATACGTGGTGC-GAATTGCAGAATCCCGCGAACC-ATCGAGTCTTTGAACGCAAGTTGCGCCCGAGGCCAAT-CGGTCGAGGGCACGTCCGCCTG---GGCGTCA-AGCGTTGCGCCGCTCC

Acampe_praemorsa_var_longepedunculata_SG1199 ACGAC-TCTCGACAATGGATATCTCGGCTCTCGCATCGATGAAGAGCGCAGCGAAATGCGATACGTGGTGC-GAATTGCAGAATCCCGCGAACC-ATCGAGTCTTTGAACGCAAGTTGCGCCCGAGGCCAAT-CGGTCGAGGGCACGTCCGCCTG---GGCGTCA-AGCGTTGCGCCGCTCC

Acanthophippium_gougahense_KFBG3161 ATGAC-TCTCGGCAATGGATATCTCGGCTCTCGCATCGATGAAGAGCGCAGCGAAATGCGATACGTGGTGC-GAATTGCAGAATCCCGCGAACC-ATCGAGTCTTTGAACGCAAGTTGCGCCTGAGGCCATC-CGGCCAAGGGCACGTCTGCCTG---GGCGTCA-AGCGTTGCGTCGCTTC

Acanthophippium_gougahensis_KY966409 ATGAC-TCTCGGCAATGGATATCTCGGCTCTCGCATCGATGAAGAGCGCAGCGAAATGCGATACGTGGTGC-GAATTGCAGAATCCCGCGAACC-ATCGAGTCTTTGAACGCAAGTTGCGCCTGAGGCCATC-CGGCCAAGGGCACGTCTGCCTG---GGCGTCA-AGCGTTGCGTCGCTTC

Ania_hongkongensis_KF560537 ACGAC-TCTCGGCAATGGATATCTCGGCTCTCGCATCGATGAAGAGCGCAGCGAAATGCGATACGTGGTGC-GAATTGCAGAATCCCGCGAACC-ATCGAGTCTTTGAACGCAAGTTGCGCCCGAGGCCAAC-CGGCCAAGGGCACGTCTGCCTG---GGCGTCA-AGCGTTGCGTCGCTCC

Ania_hongkongensis_KFBG4612 ACGAC-TCTCGGCAATGGATATCTCGGCTCTCGCATCGATGAAGAGCGCAGCGAAATGCGATACGTGGTGC-GAATTGCAGAATCCCGCGAACC-ATCGAGTCTTTGAACGCAAGTTGCGCCCGAGGCCAAC-CGGCCAAGGGCACGTCTGCCTG---GGCGTCA-AGCGTTGCGTCGCTCC

Ania_hongkongensis_KFBG4613 ACGAC-TCTCGGCAATGGATATCTCGGCTCTCGCATCGATGAAGAGCGCAGCGAAATGCGATACGTGGTGC-GAATTGCAGAATCCCGCGAACC-ATCGAGTCTTTGAACGCAAGTTGCGCCCGAGGCCAAC-CGGCCAAGGGCACGTCTGCCTG---GGCGTCA-AGCGTTGCGTCGCTCC

Ania_hongkongensis_KM025146 ACGAC-TCTCGGCAATGGATATCTCGGCTCTCGCATCGATGAAGAGCGCAGCGAAATGCGATACGTGGTGC-GAATTGCAGAATCCCGCGAACC-ATCGAGTCTTTGAACGCAAGTTGCGCCCGAGGCCAAC-CGGCCAAGGGCACGTCTGCCTG---GGCGTCA-AGCGTTGCGTCGCTCC

Ania_hongkongensis_KM025147 ACGAC-TCTCGGCAATGGATATCTCGGCTCTCGCATCGATGAAGAGCGCAGCGAAATGCGATACGTGGTGC-GAATTGCAGAATCCCGCGAACC-ATCGAGTCTTTGAACGCAAGTTGCGCCCGAGGCCAAC-CGGCCAAGGGCACGTCTGCCTG---GGCGTCA-AGCGTTGCGTCGCTCC

Ania_hongkongensis_KM025148 ACGAC-TCTCGGCAATGGATATCTCGGCTCTCGCATCGATGAAGAGCGCAGCGAAATGCGATACGTGGTGC-GAATTGCAGAATCCCGCGAACC-ATCGAGTCTTTGAACGCAAGTTGCGCCCGAGACCAAC-CGGCCAAGGGCACGTCTGCCTG---GGCGTCG-AGCGTTGCGTCGCTCC

Ania_hongkongensis_KY966415 ACGAC-TCTCGGCAATGGATATCTCGGCTCTCGCATCGATGAAGAGCGCAGCGAAATGCGATACGTGGTGC-GAATTGCAGAATCCCGCGAACC-ATCGAGTCTTTGAACGCAAGTTGCGCCCGAGGCCAAC-CGGCCAAGGGCACGTCTGCCTG---GGCGTCA-AGCGTTGCGTCGCTCC

Ania_hongkongensis_PK12027 ACGAC-TCTCGGCAATGGATATCTCGGCTCTCGCATCGATGAAGAGCGCAGCGAAATGCGATACGTGGTGC-GAATTGCAGAATCCCGCGAACC-ATCGAGTCTTTGAACGCAAGTTGCGCCCGAGGCCAAC-CGGCCAAGGGCACGTCTGCCTG---GGCGTCA-AGCGTTGCGTCGCTCC

Ania_hongkongensis_SG1231 ACGAC-TCTCGGCAATGGATATCTCGGCTCTCGCATCGATGAAGAGCGCAGCGAAATGCGATACGTGGTGC-GAATTGCAGAATCCCGCGAACC-ATCGAGTCTTTGAACGCAAGTTGCGCCCGAGGCCAAC-CGGCCAAGGGCACGTCTGCCTG---GGCGTCA-AGCGTTGCGTCGCTCC

Ania_hongkongensis_SG1343 ACGAC-TCTCGGCAATGGATATCTCGGCTCTCGCATCGATGAAGAGCGCAGCGAAATGCGATACGTGGTGC-GAATTGCAGAATCCCGCGAACC-ATCGAGTCTTTGAACGCAAGTTGCGCCCGAGGCCAAC-CGGCCAAGGGCACGTCTGCCTG---GGCGTCA-AGCGTTGCGTCGCTCC

Ania_ruybarrettoi_KFBG43 ACGAC-TCTCGGCAATGGATATCTCGGCTCTCGCATCGATGAAGAGCGCAGCGAAATGCGATACGTGGTGC-GAATTGCAGAATCCCGCGAACC-ATCGAGTCTTTGAACGCAAGTTGCGCCCGAGGCCAAC-CGGCCAAGGGCACGTCTGCCTG---GGCGTCA-AGCGTTGCGTCGCTCC

Ania_ruybarrettoi_KM025152 ACGAC-TCTCGGCAATGGATATCTCGGCTCTCGCATCGATGAAGAGCGCAGCGAAATGCGATACGTGGTGC-GAATTGCAGAATCCCGCGAACC-ATCGAGTCTTTGAACGCAAGTTGCGCCCGAGGCCAAC-CGGCCAAGGGCACGTCTGCCTG---GGCGTCA-AGCGTTGCGTCGCTCC

Ania_ruybarrettoi_KY966416 ACGAC-TCTCGGCAATGGATATCTCGGCTCTCGCATCGATGAAGAGCGCAGCGAAATGCGATACGTGGTGC-GAATTGCAGAATCCCGCGAACC-ATCGAGTCTTTGAACGCAAGTTGCGCCCGAGGCCAAC-CGGCCAAGGGCACGTCTGCCTG---GGCGTCA-AGCGTTGCGTCGCTCC

Ania_ruybarrettoi_SG1395 ACGAC-TCTCGGCAATGGATATCTCGGCTCTCGCATCGATGAAGAGCGCAGCGAAATGCGATACGTGGTGC-GAATTGCAGAATCCCGCGAACC-ATCGAGTCTTTGAACGCAAGTTGCGCCCGAGGCCAAC-CGGCCAAGGGCACGTCTGCCTG---GGCGTCA-AGCGTTGCGTCGCTCC

Anoectochilus_formosanus_AY052780 ATGAC-TCTCGGCAATGGATATCTTGGCTCTTGCATCGATGAAGAGCGCAGCGAAATGCGATACGTGGTGT-GAATTGCAGAATCCCGTGAACC-ATCAAATCTTTGAACGCAAGTTGCGCCTGAGGCCAAT-TGGCTAAGGGCACGTCCGCCTG---GGCGTCA-AGCATTACATCGCTTC

Anoectochilus_formosanus_GQ328777 ATGAC-TCTCGGCAATGGATATCTTGGCTCTTGCATCGATGAAGAGCGCAGCGAAATGCGATACGTGGTGT-GAATTGCAGAATCCCGTGAACC-ATCAAATCTTTGAACGCAAGTTGCGCCTGAGGCCAAT-TGGCTAAGGGCACGTCCGCCTG---GGCGTCA-AGCATTACATCGCTTC

Anoectochilus_formosanus_GQ396668 ATGAC-TCTCGGCAATGGATATCTTGGCTCTTGCATCGATGAAGAGCGCAGCGAAATGCGATACGTGGTGT-GAATTGCAGAATCCCGTGAACC-ATCAAATCTTTGAACGCAAGTTGCGCCTGAGGCCAAT-TGGCTAAGGGCACGTCCGCCTG---GGCGTCA-AGCATTACATCGCTTC

Anoectochilus_formosanus_KR815833 ATGAC-TCTCGGCAATGGATATCTTGGCTCTTGCATCGATGAAGAGCGCAGCGAAATGCGATACGTGGTGT-GAATTGCAGAATCCCGTGAACC-ATCAAATCTTTGAACGCAAGTTGCGCCTGAGGCCAAT-TGGCTAAGGGCACGTCCGCCTG---GGCGTCA-AGCATTACATCGCTTC

Anoectochilus_formosanus_KR815839 ATGAC-TCTCGGCAATGGATATCTTGGCTCTTGCATCGATGAAGAGCGCAGCGAAATGCGATACGTGGTGT-GAATTGCAGAATCCCGTGAACC-ATCAAATCTTTGAACGCAAGTTGCGCCTGAGGCCAAT-TGGCTAAGGGCACGTCCGCCTG---GGCGTCA-AGCATTACATCGCTTC

Anoectochilus_formosanus_PK12215 ATGAC-TCTCGGCAATGGATATCTTGGCTCTTGCATCGATGAAGAGCGCAGCGAAATGCGATACGTGGTGT-GAATTGCAGAATCCCGTGAACC-ATCAAATCTTTGAACGCAAGTTGCGCCTGAGGCCAAT-TGGCTAAGGGCACGTCCGCCTG---GGCGTCA-AGCATTACATCGCTTC

Anoectochilus_roxburghii_EU817408 ATGAC-TCTCGGCAATGGATATCTTGGCTCTTGCATCGATGAAGAGCGCAGCGAAATGCGATACGTGGTGT-GAATTGCAGAATCCCGTGAACC-ATCAAATCTTTGAACGCAAGTTGCGCCTGAGGCCAAT-TGGCTAAGGGCACGTCCGCCTG---GGCGTCA-AGCATTACATCGCTTC

Anoectochilus_roxburghii_GQ328774 ATGAC-TCTCGGCAATGGATATCTTGGCTCTTGCATCGATGAAGAGCGCAGCGAAATGCGATACGTGGTGT-GAATTGCAGAATCCCGTGAACC-ATCAAATCTTTGAACGCAAGTTGCGCCTGAGGCCAAT-TGGCTAAGGGCACGTCCGCCTG---GGCGTCA-AGCATTACATCGCTTC

Anoectochilus_roxburghii_KF425501 ATGAC-TCTCGGCAATGGATATCTTGGCTCTTGCATCGATGAAGAGCGCAGCGAAATGCGATACGTGGTGT-GAATTGCAGAATCCCGTGAACC-ATCAAATCTTTGAACGCAAGTTGCGCCTGAGGCCAAT-TGGCTAAGGGCACGTCCGCCTG---GGCGTCA-AGCATTACATCGCTTC

Anoectochilus_roxburghii_KF425502 ATGAC-TCTCGGCAATGGATATCTTGGCTCTTGCATCGATGAAGAGCGCAGCGAAATGCGATACGTGGTGT-GAATTGCAGAATCCCGTGAACC-ATCAAATCTTTGAACGCAAGTTGCGCCTGAGGCCAAT-TGGCTAAGGGCACGTCCGCCTG---GGCGTCA-AGCATTACATCGCTTC

Anoectochilus_roxburghii_KF425503 ATGAC-TCTCGGCAATGGATATCTTGGCTCTTGCATCGATGAAGAGCGCAGCGAAATGCGATACGTGGTGT-GAATTGCAGAATCCCGTGAACC-ATCAAATCTTTGAACGCAAGTTGCGCCTGAGGCCAAT-TGGCTAAGGGCACGTCCGCCTG---GGCGTCA-AGCATTACATCGCTTC

Anoectochilus_roxburghii_KR815828 ATGAC-TCTCGGCAATGGATATCTTGGCTCTTGCATCGATGAAGAGCGCAGCGAAATGCGATACGTGGTGT-GAATTGCAGAATCCCGTGAACC-ATCAAATCTTTGAACGCAAGTTGCGCCTGAGGCCAAT-TGGCTAAGGGCACGTCCGCCTG---GGCGTCA-AGCATTACATCGCTTC

Anoectochilus_roxburghii_KR815829 ATGAC-TCTCGGCAATGGATATCTTGGCTCTTGCATCGATGAAGAGCGCAGCGAAATGCGATACGTGGTGT-GAATTGCAGAATCCCGTGAACC-ATCAAATCTTTGAACGCAAGTTGCGCCTGAGGCCAAT-TGGCTAAGGGCACGTCCGCCTG---GGCGTCA-AGCATTACATCGCTTC

Anoectochilus_roxburghii_KR815830 ATGAC-TCTCGGCAATGGATATCTTGGCTCTTGCATCGATGAAGAGCGCAGCGAAATGCGATACGTGGTGT-GAATTGCAGAATCCCGTGAACC-ATCAAATCTTTGAACGCAAGTTGCGCCTGAGGCCAAT-TGGCTAAGGGCACGTCCGCCTG---GGCGTCA-AGCATTACATCGCTTC

Anoectochilus_roxburghii_KR815831 ATGAC-TCTCGGCAATGGATATCTTGGCTCTTGCATCGATGAAGAGCGCAGCGAAATGCGATACGTGGTGT-GAATTGCAGAATCCCGTGAACC-ATCAAATCTTTGAACGCAAGTTGCGCCTGAGGCCAAT-TGGCTAAGGGCACGTCCGCCTG---GGCGTCA-AGCATTACATCGCTTC

Anoectochilus_roxburghii_KR815832 ATGAC-TCTCGGCAATGGATATCTTGGCTCTTGCATCGATGAAGAGCGCAGCGAAATGCGATACGTGGTGT-GAATTGCAGAATCCCGTGAACC-ATCAAATCTTTGAACGCAAGTTGCGCCTGAGGCCAAT-TGGCTAAGGGCACGTCCGCCTG---GGCGTCA-AGCATTACATCGCTTC

Anoectochilus_roxburghii_KR815836 ATGAC-TCTCGGCAATGGATATCTTGGCTCTTGCATCGATGAAGAGCGCAGCGAAATGCGATACGTGGTGT-GAATTGCAGAATCCCGTGAACC-ATCAAATCTTTGAACGCAAGTTGCGCCTGAGGCCAAT-TGGCTAAGGGCACGTCCGCCTG---GGCGTCA-AGCATTACATCGCTTC

Anoectochilus_roxburghii_KR815837 ATGAC-TCTCGGCAATGGATATCTTGGCTCTTGCATCGATGAAGAGCGCAGCGAAATGCGATACGTGGTGT-GAATTGCAGAATCCCGTGAACC-ATCAAATCTTTGAACGCAAGTTGCGCCTGAGGCCAAT-TGGCTAAGGGCACGTCCGCCTG---GGCGTCA-AGCATTACATCGCTTC

Anoectochilus_roxburghii_KR815838 ATGAC-TCTCGGCAATGGATATCTTGGCTCTTGCATCGATGAAGAGCGCAGCGAAATGCGATACGTGGTGT-GAATTGCAGAATCCCGTGAACC-ATCAAATCTTTGAACGCAAGTTGCGCCTGAGGCCAAT-TGGCTAAGGGCACGTCCGCCTG---GGCGTCA-AGCATTACATCGCTTC

Anoectochilus_roxburghii_KY966417 ATGAC-TCTCGGCAATGGATATCTTGGCTCTTGCATCGATGAAGAGCGCAGCGAAATGCGATACGTGGTGT-GAATTGCAGAATCCCGTGAACC-ATCAAATCTTTGAACGCAAGTTGCGCCTGAGGCCAAT-TGGCTAAGGGCACGTCCGCCTG---GGCGTCA-AGCATTACATCGCTTC

Anoectochilus_roxburghii_PK12043 ATGAC-TCTCGGCAATGGATATCTTGGCTCTTGCATCGATGAAGAGCGCAGCGAAATGCGATACGTGGTGT-GAATTGCAGAATCCCGTGAACC-ATCAAATCTTTGAACGCAAGTTGCGCCTGAGGCCAAT-TGGCTAAGGGCACGTCCGCCTG---GGCGTCA-AGCATTACATCGCTTC

Anoectochilus_roxburghii_PK12044 ATGAC-TCTCGGCAATGGATATCTTGGCTCTTGCATCGATGAAGAGCGCAGCGAAATGCGATACGTGGTGT-GAATTGCAGAATCCCGTGAACC-ATCAAATCTTTGAACGCAAGTTGCGCCTGAGGCCAAT-TGGCTAAGGGCACGTCCGCCTG---GGCGTCA-AGCATTACATCGCTTC

Anoectochilus_roxburghii_PK12068 ATGAC-TCTCGGCAATGGATATCTTGGCTCTTGCATCGATGAAGAGCGCAGCGAAATGCGATACGTGGTGT-GAATTGCAGAATCCCGTGAACC-ATCAAATCTTTGAACGCAAGTTGCGCCTGAGGCCAAT-TGGCTAAGGGCACGTCCGCCTG---GGCGTCA-AGCATTACATCGCTTC

Anoectochilus_roxburghii_PK12069 ATGAC-TCTCGGCAATGGATATCTTGGCTCTTGCATCGATGAAGAGCGCAGCGAAATGCGATACGTGGTGT-GAATTGCAGAATCCCGTGAACC-ATCAAATCTTTGAACGCAAGTTGCGCCTGAGGCCAAT-TGGCTAAGGGCACGTCCGCCTG---GGCGTCA-AGCATTACATCGCTTC

Anoectochilus_roxburghii_SG1219 ATGAC-TCTCGGCAATGGATATCTTGGCTCTTGCATCGATGAAGAGCGCAGCGAAATGCGATACGTGGTGT-GAATTGCAGAATCCCGTGAACC-ATCAAATCTTTGAACGCAAGTTGCGCCTGAGGCCAAT-TGGCTAAGGGCACGTCCGCCTG---GGCGTCA-AGCATTACATCGCTTC

Aphyllorchis_montana_FJ454867 ATGAC-TCTCGGCAACGGATATCTCGGCTCTCGCATCGATGAAGAGCGCAGCGAAATGCGATACGTGGTGC-GAATTGCAGAATCCCGTGAACC-ATCAAGTCTTTGAACGCAAGTTGCGCCCGAGGCCAAT-CGGCCAAGGGCACGTCTGCCTG---GGCGTCA-AGCGTTGCGTCGCTTC

Aphyllorchis_montana_PK12147 ATGAC-TCTCGGCAACGGATATCTCGGCTCTCGCATCGATGAAGAGCGCAGCGAAATGCGATACGTGGTGC-GAATTGCAGAATCCCGTGAACC-ATCAAGTCTTTGAACGCAAGTTGCGCCCGAGGCCAAT-CGGCCAAGGGCACGTCTGCCTG---GGCGTCA-AGCGTTGCGTCGCTTC

Aphyllorchis_montana_PK12148 ATGAC-TCTCGGCAACGGATATCTCGGCTCTCGCATCGATGAAGAGCGCAGCGAAATGCGATACGTGGTGC-GAATTGCAGAATCCCGTGAACC-ATCAAGTCTTTGAACGCAAGTTGCGCCCGAGGCCAAT-CGGCCAAGGGCACGTCTGCCTG---GGCGTCA-AGCGTTGCGTCGCTTC

Aphyllorchis_montana_SG1010 ATGAC-TCTCGGCAACGGATATCTCGGCTCTCGCATCGATGAAGAGCGCAGCGAAATGCGATACGTGGTGC-GAATTGCAGAATCCCGTGAACC-ATCAAGTCTTTGAACGCAAGTTGCGCCCGAGGCCAAT-CGGCCAAGGGCACGTCTGCCTG---GGCGTCA-AGCGTTGCGTCGCTTC

Apostasia_nipponica_PK12273 AAGAC-TCTCGGCAACGGATATCTCGGCTCTCGCATCGATGAAGAACGCAGCGAACCGCGATACGTGGTGT-GAATTGCAGAATCCCGCGAATC-ATCGAGTCTTTGAACGCAAGTTGCGCCCGAGGCCAAG-TGGACGAGGGCACGCCTGCCTG---GTCGTCAG-GCGCTGCGTCGCTCC

Apostasia_nipponica_PK12274 AAGAC-TCTCGGCAACGGATATCTCGGCTCTCGCATCGATGAAGAACGCAGCGAACCGCGATACGTGGTGT-GAATTGCAGAATCCCGCGAATC-ATCGAGTCTTTGAACGCAAGTTGCGCCCGAGGCCAAG-TGGCCGAGGGCACGCCTGCCTG---GTCGTCAG-GCGCCGCGTCGCTCC

Appendicula_cornuta_AF521073 ATGAC-TCTCGGCAATGGATATCTCGGCTCTCGCATCGATGAAGAGCGCAGCGAAATGCGATACGTGGTGT-GAATTGCAGAATCCCGCGAACC-ATCGAGTCTTTGAACGCAAGTTGCGCCCGAGGCCAAC-CGGCTGAGGGCACGTCTGCCTG---GGCGTCA-AACGTTTCGTCGCTTC

Appendicula_cornuta_KY239261 ATGAC-TCTCGGCAATGGATATCTCGGCTCTCGCATCGATGAAGAGCGCAGCGAAATGCGATACGTGGTGT-GAATTGCAGAATCCCGCGAACC-ATCGAGTCTTTGAACGCAAGTTGCGCCCGAGGCCAAC-CGGCTGAGGGCACGTCTGCCTG---GGCGTCA-AACGTTTCGTCGCTTC

Appendicula_cornuta_KY966419 ATGAC-TCTCGGCAATGGATATCTCGGCTCTCGCATCGATGAAGAGCGCAGCGAAATGCGATACGTGGTGT-GAATTGCAGAATCCCGCGAACC-ATCGAGTCTTTGAACGCAAGTTGCGCCCGAGGCCAAC-CGGCTGAGGGCACGTCTGCCTG---GGCGTCA-AACGTTTCGTCGCTTC

Appendicula_cornuta_PK12064 ATGAC-TCTCGGCAATGGATATCTCGGCTCTCGCATCGATGAAGAGCGCAGCGAAATGCGATACGTGGTGT-GAATTGCAGAATCCCGCGAACC-ATCGAGTCTTTGAACGCAAGTTGCGCCCGAGGCCAAC-CGGCTGAGGGCACGTCTGCCTG---GGCGTCA-AACGTTTCGTCGCTTC

Appendicula_cornuta_PK12065 ATGAC-TCTCGGCAATGGATATCTCGGCTCTCGCATCGATGAAGAGCGCAGCGAAATGCGATACGTGGTGT-GAATTGCAGAATCCCGCGAACC-ATCGAGTCTTTGAACGCAAGTTGCGCCCGAGGCCAAC-CGGCTGAGGGCACGTCTGCCTG---GGCGTCA-AACGTTTCGTCGCTTC

Arundina_graminifolia_AF273335 ACGAC-TCTCGGCAATGGATATCTCGGCTCTCGCATCGATGAAGAGCGCAGCGAAATGCGATACGTGGTGC-GAATTGCAGAATCCCGCGAACC-ATCGAGTCTTTGAACGCAAGTTGCGCCTGAGGCCAAC-CGGCCGAGGGCACGTCTGCCTG---GGCGTCA-GGCGTTACGTCGCTCC

Arundina_graminifolia_AF302727 ACGAC-TCTCGGCAATGGATATCTCGGCTCTCGCATCGATGAAGAGCGCAGCGAAATGCGATACGTGGTGC-GAATTGCAGAATCCCGCGAACC-ATCGAGTCTTTGAACGCAAGTTGCGCCTGAGGCCAAC-CGGCCGAGGGCACGTCTGCCTG---GGCGTCA-GGCGTTACGTCGCTCC

Arundina_graminifolia_AF461461 ACGAC-TCTCGGCAATGGATATCTCGGCTCTCGCATCGATGAAGAGCGCAGCGAAATGCGATACGTGGTGC-GAATTGCAGAATCCCGCGAACC-ATCGAGTCTTTGAACGCAAGTTGCGCCTGAGGCCAAC-CGGCCGAGGGCACGTCTGCCTG---GGCGTCA-GGCGTTACGTCGCTCC

Arundina_graminifolia_JN114437 ACGAC-TCTCGGCAATGGATATCTCGGCTCTCGCATCGATGAAGAGCGCAGCGAAATGCGATACGTGGTGC-GAATTGCAGAATCCCGCGAACC-ATCGAGTCTTTGAACGCAAGTTGCGCCTGAGGCCAAC-CGGCCGAGGGCACGTCTGCCTG---GGCGTCA-GGCGTTACGTCGCTCC

Arundina_graminifolia_JN114438 ACGAC-TCTCGGCAATGGATATCTCGGCTCTCGCATCGATGAAGAGCGCAGCGAAATGCGATACGTGGTGC-GAATTGCAGAATCCCGCGAACC-ATCGAGTCTTTGAACGCAAGTTGCGCCTGAGGCCAAC-CGGCCGAGGGCACGTCTGCCTG---GGCGTCA-GGCGTTACGTCGCTCC

Arundina_graminifolia_JN114439 ACGAC-TCTCGGCAATGGATATCTCGGCTCTCGCATCGATGAAGAGCGCAGCGAAATGCGATACGTGGTGC-GAATTGCAGAATCCCGCGAACC-ATCGAGTCTTTGAACGCAAGTTGCGCCTGAGGCCAAC-CGGCCGAGGGCACGTCTGCCTG---GGCGTCA-GGCGTTACGTCGCTCC

Arundina_graminifolia_JN114440 ACGAC-TCTCGGCAATGGATATCTCGGCTCTCGCATCGATGAAGAGCGCAGCGAAATGCGATACGTGGTGC-GAATTGCAGAATCCCGCGAACC-ATCGAGTCTTTGAACGCAAGTTGCGCCTGAGGCCAAC-CGGCCGAGGGCACGTCTGCCTG---GGCGTCA-GGCGTTACGTCGCTCC

Arundina_graminifolia_KY966420 ACGAC-TCTCGGCAATGGATATCTCGGCTCTCGCATCGATGAAGAGCGCAGCGAAATGCGATACGTGGTGC-GAATTGCAGAATCCCGCGAACC-ATCGAGTCTTTGAACGCAAGTTGCGCCTGAGGCCAAC-CGGCCGAGGGCACGTCTGCCTG---GGCGTCA-GGCGTTACGTCGCTCC

Arundina_graminifolia_SG1008 ACGAC-TCTCGGCAATGGATATCTCGGCTCTCGCATCGATGAAGAGCGCAGCGAAATGCGATACGTGGTGC-GAATTGCAGAATCCCGCGAACC-ATCGAGTCTTTGAACGCAAGTTGCGCCTGAGGCCAAC-CGGCCGAGGGCACGTCTGCCTG---GGCGTCA-GGCGTTACGTCGCTCC

Arundina_graminifolia_SG1206 ACGAC-TCTCGGCAATGGATATCTCGGCTCTCGCATCGATGAAGAGCGCAGCGAAATGCGATACGTGGTGC-GAATTGCAGAATCCCGCGAACC-ATCGAGTCTTTGAACGCAAGTTGCGCCTGAGGCCAAC-CGGCCGAGGGCACGTCTGCCTG---GGCGTCA-GGCGTTACGTCGCTCC

Arundina_graminifolia_SG1295 ACGAC-TCTCGGCAATGGATATCTCGGCTCTCGCATCGATGAAGAGCGCAGCGAAATGCGATACGTGGTGC-GAATTGCAGAATCCCGCGAACC-ATCGAGTCTTTGAACGCAAGTTGCGCCTGAGGCCAAC-CGGCCGAGGGCACGTCTGCCTG---GGCGTCA-GGCGTTACGTCGCTCC

Bletilla_striata_KF560509 ACGAC-TCTCGGCAATGGATATCTCGGCTCTCGCATCGATGAAGAGCGCAGCGAAATGCGATACGTGGTGC-GAATTGCAGAATCCCGCGAACC-ATCGAGTCTTTGAACGCAAGTTGCGCCCGAGGCCAAC-CGGCCAAGGGCACGTCTGCCTG---GGCGTCA-AGCGTTGCGTCGCTCC

Bletilla_striata_KF560540 ACGAC-TCTCGGCAATGGATATCTCGGCTCTCGCATCGATGAAGAGCGCAGCGAAATGCGATACGTGGTGC-GAATTGCAGAATCCCGCGAACC-ATCGAGTCTTTGAACGCAAGTTGCGCCCGAGGCCAAC-CGGCCAAGGGCACGTCTGCCTG---GGCGTCA-AGCGTTGCGTCGCTCC

Brachycorythis_galeandra_PK12195 AGGGC-TCTCGGCAATGGATATCTTGGCTCTCGCATCGATGAAGAGCGCAACGAAATGCGATACGTGGTGC-GAATTGCAGAATCCCGTGAACC-ATCGAGTTTTTGAACGCAAGTTGCGCCTGAGGCCAGC-TGGCCAAAGGCACGTCCGCCTG---GGCGTCA-AGCATTGAATCGCTCC

Brachycorythis_galeandra_SG1261 AGGGC-TCTCGGCAATGGATATCTTGGCTCTCGCATCGATGAAGAGCGCAACGAAATGCGATACGTGGTGC-GAATTGCAGAATCCCGTGAACC-ATCGAGTTTTTGAACGCAAGTTGCGCCTGAGGCCAGC-TGGCCAAAGGCACGTCCGCCTG---GGCGTCA-AGCATTGAATCGCTCC

Bulbophyllum_affine_EF195916 ACGAC-TCTCGGCAATGGATATCTCGGCTCTCGCATCGATGAAGAGCGCAGCGAAATGCGATACGTGGTGC-GAATTGCAGAATCCCGCGAACC-ATCGAGTCTTTGAACGCAAGTTGCGCCCGAGGCCAAC-CGGCCAAGGGCACGTCCGCCTG---GGCGTCA-AGCGTTGCGTCACTCC

Bulbophyllum_affine_KC568305 ACGAC-TCTCGGCAATGGATATCTCGGCTCTCGCATCGATGAAGAGCGCAGCGAAATGCGATACGTGGTGC-GAATTGCAGAATCCCGCGAACC-ATCGAGTCTTTGAACGCAAGTTGCGCCCGAGGCCAAC-CGGCCAAGGGCACGTCCGCCTG---GGCGTCA-AGCGTTGCGTCACTCC

Bulbophyllum_affine_KF866246 ACGAC-TCTCGGCAATGGATATCTCGGCTCTCGCATCGATGAAGAGCGCAGCGAAATGCGATACGTGGTGC-GAATTGCAGAATCCCGCGAACC-ATCGAGTCTTTGAACGCAAGTTGCGCCCGAGGCCAAC-CGGCCAAGGGCACGTCCGCCTG---GGCGTCA-AGCGTTGCGTCACTCC

Bulbophyllum_affine_KFBG412 ACGAC-TCTCGGCAATGGATATCTCGGCTCTCGCATCGATGAAGAGCGCAGCGAAATGCGATACGTGGTGC-GAATTGCAGAATCCCGCGAACC-ATCGAGTCTTTGAACGCAAGTTGCGCCCGAGGCCAAC-CGGCCAAGGGCACGTCCGCCTG---GGCGTCA-AGCGTTGCGTCACTCC

Bulbophyllum_affine_KX455815 ACGAC-TCTCGGCAATGGATATCTCGGCTCTCGCATCGATGAAGAGCGCAGCGAAATGCGATACGTGGTGC-GAATTGCAGAATCCCGCGAACC-ATCGAGTCTTTGAACGCAAGTTGCGCCCGAGGCCAAC-CGGCCAAGGGCACGTCCGCCTG---GGCGTCA-AGCGTTGCGTCACTCC

Bulbophyllum_affine_KY966422 ACGAC-TCTCGGCAATGGATATCTCGGCTCTCGCATCGATGAAGAGCGCAGCGAAATGCGATACGTGGTGC-GAATTGCAGAATCCCGCGAACC-ATCGAGTCTTTGAACGCAAGTTGCGCCCGAGGCCAAC-CGGCCAAGGGCACGTCCGCCTG---GGCGTCA-AGCGTTGCGTCACTCC

Bulbophyllum_affine_MK164425 ACGAC-TCTCGGCAATGGATATCTCGGCTCTCGCATCGATGAAGAGCGCAGCGAAATGCGATACGTGGTGC-GAATTGCAGAATCCCGCGAACC-ATCGAGTCTTTGAACGCAAGTTGCGCCCGAGGCCAAC-CGGCCAAGGGCACGTCCGCCTG---GGCGTCA-AGCGTTGCGTCACTCC

Bulbophyllum_affine_SG1606 ACGAC-TCTCGGCAATGGATATCTCGGCTCTCGCATCGATGAAGAGCGCAGCGAAATGCGATACGTGGTGC-GAATTGCAGAATCCCGCGAACC-ATCGAGTCTTTGAACGCAAGTTGCGCCCGAGGCCAAC-CGGCCAAGGGCACGTCCGCCTG---GGCGTCA-AGCGTTGCGTCACTCC

Bulbophyllum_affine_SG1607 ACGAC-TCTCGGCAATGGATATCTCGGCTCTCGCATCGATGAAGAGCGCAGCGAAATGCGATACGTGGTGC-GAATTGCAGAATCCCGCGAACC-ATCGAGTCTTTGAACGCAAGTTGCGCCCGAGGCCAAC-CGGCCAAGGGCACGTCCGCCTG---GGCGTCA-AGCGTTGCGTCACTCC

Bulbophyllum_ambrosia_JN619413 ACGAC-TCTCGGCAATGGATATCTCGGCTCTCGCATCGATGAAGAGCGCAGCGAAATGCGATACGTGGTGC-GAATTGCAGAATCCCGCGAACC-ATCGAGTCTTTGAACGCAAGTTGCGCCCGAGGCCGAC-CGGCCGAGGGCACGTCCGCCTG---GGCGTCA-AGCGTTGCGCCGCTCC

Bulbophyllum_ambrosia_KC568306 ACGAC-TCTCGGCAATGGATATCTCGGCTCTCGCATCGATGAAGAGCGCAGCGAAATGCGATACGTGGTGC-GAATTGCAGAATCCCGCGAACC-ATCGAGTCTTTGAACGCAAGTTGCGCCCGAGGCCAAC-CGGCCGAGGGCACGTCCGCCTG---GGCGTCA-AGCGTTGCGTCGCTCC

Bulbophyllum_ambrosia_KY966424 ACGAC-TCTCGGCAATGGATATCTCGGCTCTCGCATCGATGAAGAGCGCAGCGAAATGCGATACGTGGTGC-GAATTGCAGAATCCCGCGAACC-ATCGAGTCTTTGAACGCAAGTTGCGCCCGAGGCCAAC-CGGCCGAGGGCACGTCCGCCTG---GGCGTCA-AGCGTTGCGTCGCTCC

Bulbophyllum_ambrosia_KY966425 ACGAC-TCTCGGCAATGGATATCTCGGCTCTCGCATCGATGAAGAGCGCAGCGAAATGCGATACGTGGTGC-GAATTGCAGAATCCCGCGAACC-ATCGAGTCTTTGAACGCAAGTTGCGCCCGAGGCCAAC-CGGCCGAGGGCACGTCCGCCTG---GGCGTCA-AGCGTTGCGTCGCTCC

Bulbophyllum_ambrosia_KY966426 ACGAC-TCTCGGCAATGGATATCTCGGCTCTCGCATCGATGAAGAGCGCAGCGAAATGCGATACGTGGTGC-GAATTGCAGAATCCCGCGAACC-ATCGAGTCTTTGAACGCAAGTTGCGCCCGAGGCCAAC-CGGCCGAGGGCACGTCCGCCTG---GGCGTCA-AGCGTTGCGTCGCTCC

Bulbophyllum_ambrosia_MK164427 ACGAC-TCTCGGCAATGGATATCTCGGCTCTCGCATCGATGAAGAGCGCAGCGAAATGCGATACGTGGTGC-GAATTGCAGAATCCCGCGAACC-ATCGAGTCTTTGAACGCAAGTTGCGCCCGAGGCCAAC-CGGCCGAGGGCACGTCCGCCTG---GGCGTCA-AGCGTTGCGTCGCTCC

Bulbophyllum_ambrosia_PK12090 ACGAC-TCTCGGCAATGGATATCTCGGCTCTCGCATCGATGAAGAGCGCAGCGAAATGCGATACGTGGTGC-GAATTGCAGAATCCCGCGAACC-ATCGAGTCTTTGAACGCAAGTTGCGCCCGAGGCCAGC-CGGCCGAGGGCACGTCCGCCTG---GGCGTCA-AGCGTTGCGTCGCTCC

Bulbophyllum_ambrosia_PK12092 ACGAC-TCTCGGCAATGGATATCTCGGCTCTCGCATCGATGAAGAGCGCAGCGAAATGCGATACGTGGTGC-GAATTGCAGAATCCCGCGAACC-ATCGAGTCTTTGAACGCAAGTTGCGCCCGAGGCCAAC-CGGCCGAGGGCACGTCCGCCTG---GGCGTCA-AGCGTTGCGTCGCTCC

Bulbophyllum_ambrosia_SG1221 ACGAC-TCTCGGCAATGGATATCTCGGCTCTCGCATCGATGAAGAGCGCAGCGAAATGCGATACGTGGTGC-GAATTGCAGAATCCCGCGAACC-ATCGAGTCTTTGAACGCAAGTTGCGCCCGAGGCCAAC-CGGCCGAGGGCACGTCCGCCTG---GGCGTCA-AGCGTTGCGTCGCTCC

Bulbophyllum_bicolor_CL10 ACGAC-TCTCGGCAATGGATATCTCGGCTCTCGCATCGATGAAGAGCGCAGCGAAATGCGATACGTGGTGC-GAATTGCAGAATCCCGCGAACC-ATCGAGTCTTTGAACGCAAGTTGCGCCCGAGGCCAGC-CGGCCGAGGGCACGTCCGCCTG---GGCGTCA-AGCGTTGCGTCGCTCC

Bulbophyllum_bicolor_FT28 ACGAC-TCTCGGCAATGGATATCTCGGCTCTCGCATCGATGAAGAGCGCAGCGAAATGCGATACGTGGTGC-GAATTGCAGAATCCCGCGAACC-ATCGAGTCTTTGAACGCAAGTTGCGCCCGAGGCCAGC-CGGCCGAGGGCACGTCCGCCTG---GGCGTCA-AGCGTTGCGTCGCTCC

Bulbophyllum_bicolor_KFBG2210 ACGAC-TCTCGGCAATGGATATCTCGGCTCTCGCATCGATGAAGAGCGCAGCGAAATGCGATACGTGGTGC-GAATTGCAGAATCCCGCGAACC-ATCGAGTCTTTGAACGCAAGTTGCGCCCGAGGCCAGC-CGGCCGAGGGCACGTCCGCCTG---GGCGTCA-AGCGTTGCGTCGCTCC

Bulbophyllum_bicolor_KFBG3073 ACGAC-TCTCGGCAATGGATATCTCGGCTCTCGCATCGATGAAGAGCGCAGCGAAATGCGATACGTGGTGC-GAATTGCAGAATCCCGCGAACC-ATCGAGTCTTTGAACGCAAGTTGCGCCCGAGGCCAGC-CGGCCGAGGGCACGTCCGCCTG---GGCGTCA-AGCGTTGCGTCGCTCC

Bulbophyllum_bicolor_KFBG433A ACGAC-TCTCGGCAATGGATATCTCGGCTCTCGCATCGATGAAGAGCGCAGCGAAATGCGATACGTGGTGC-GAATTGCAGAATCCCGCGAACC-ATCGAGTCTTTGAACGCAAGTTGCGCCCGAGGCCAGC-CGGCCGAGGGCACGTCCGCCTG---GGCGTCA-AGCGTTGCGTCGCTCC

Bulbophyllum_bicolor_KFBG445 ACGAC-TCTCGGCAATGGATATCTCGGCTCTCGCATCGATGAAGAGCGCAGCGAAATGCGATACGTGGTGC-GAATTGCAGAATCCCGCGAACC-ATCGAGTCTTTGAACGCAAGTTGCGCCCGAGGCCAAC-CGGCCGAGGGCACGTCCGCCTG---GGCGTCA-AGCGTTGCGTCGCTCC

Bulbophyllum_bicolor_KY022455 ACGAC-TCTCGGCAATGGATATCTCGGCTCTCGCATCGATGAAGAGCGCAGCGAAATGCGATACGTGGTGC-GAATTGCAGAATCCCGCGAACC-ATCGAGTCTTTGAACGCAAGTTGCGCCCGAGGCCAGC-CGGCCGAGGGCACGTCCGCCTG---GGCGTCA-AGCGTTGCGTCGCTCC

Bulbophyllum_bicolor_KY966430 ACGAC-TCTCGGCAATGGATATCTCGGCTCTCGCATCGATGAAGAGCGCAGCGAAATGCGATACGTGGTGC-GAATTGCAGAATCCCGCGAACC-ATCGAGTCTTTGAACGCAAGTTGCGCCCGAGGCCAGC-CGGCCGAGGGCACGTCCGCCTG---GGCGTCA-AGCGTTGCGTCGCTCC

Bulbophyllum_bicolor_KY966431 ACGAC-TCTCGGCAATGGATATCTCGGCTCTCGCATCGATGAAGAGCGCAGCGAAATGCGATACGTGGTGC-GAATTGCAGAATCCCGCGAACC-ATCGAGTCTTTGAACGCAAGTTGCGCCCGAGGCCAGC-CGGCCGAGGGCACGTCCGCCTG---GGCGTCA-AGCGTTGCGTCGCTCC

Bulbophyllum_bicolor_KY966432 ACGAC-TCTCGGCAATGGATATCTCGGCTCTCGCATCGATGAAGAGCGCAGCGAAATGCGATACGTGGTGC-GAATTGCAGAATCCCGCGAACC-ATCGAGTCTTTGAACGCAAGTTGCGCCCGAGGCCAGC-CGGCCGAGGGCACGTCCGCCTG---GGCGTCA-AGCGTTGCGTCGCTCC

Bulbophyllum_bicolor_LMP19 ACGAC-TCTCGGCAATGGATATCTCGGCTCTCGCATCGATGAAGAGCGCAGCGAAATGCGATACGTGGTGC-GAATTGCAGAATCCCGCGAACC-ATCGAGTCTTTGAACGCAAGTTGCGCCCGAGGCCAGC-CGGCCGAGGGCACGTCCGCCTG---GGCGTCA-AGCGTTGCGTCGCTCC

Bulbophyllum_bicolor_MK164432 ACGAC-TCTCGGCAATGGATATCTCGGCTCTCGCATCGATGAAGAGCGCAGCGAAATGCGATACGTGGTGC-GAATTGCAGAATCCCGCGAACC-ATCGAGTCTTTGAACGCAAGTTGCGCCCGAGGCCAGC-CGGCCGAGGGCACGTCCGCCTG---GGCGTCA-AGCGTTGCGTCGCTCC

Bulbophyllum_bicolor_MK164433 ACGAC-TCTCGGCAATGGATATCTCGGCTCTCGCATCGATGAAGAGCGCAGCGAAATGCGATACGTGGTGC-GAATTGCAGAATCCCGCGAACC-ATCGAGTCTTTGAACGCAAGTTGCGCCCGAGGCCAGC-CGGCCGAGGGCACGTCCGCCTG---GGCGTCA-AGCGTTGCGTCGCTCC

Bulbophyllum_bicolor_PSL45 ACGAC-TCTCGGCAATGGATATCTCGGCTCTCGCATCGATGAAGAGCGCAGCGAAATGCGATACGTGGTGC-GAATTGCAGAATCCCGCGAACC-ATCGAGTCTTTGAACGCAAGTTGCGCCCGAGGCCAGC-CGGCCGAGGGCACGTCCGCCTG---GGCGTCA-AGCGTTGCGTCGCTCC

Bulbophyllum_bicolor_TMS05 ACGAC-TCTCGGCAATGGATATCTCGGCTCTCGCATCGATGAAGAGCGCAGCGAAATGCGATACGTGGTGC-GAATTGCAGAATCCCGCGAACC-ATCGAGTCTTTGAACGCAAGTTGCGCCCGAGGCCAGC-CGGCCGAGGGCACGTCCGCCTG---GGCGTCA-AGCGTTGCGTCGCTCC

Bulbophyllum_bicolor_TT43 ACGAC-TCTCGGCAATGGATATCTCGGCTCTCGCATCGATGAAGAGCGCAGCGAAATGCGATACGTGGTGC-GAATTGCAGAATCCCGCGAACC-ATCGAGTCTTTGAACGCAAGTTGCGCCCGAGGCCAGC-CGGCCGAGGGCACGTCCGCCTG---GGCGTCA-AGCGTTGCGTCGCTCC

Bulbophyllum_delitescens_KY966439 ACGAC-TCTCGGCAATGGATATCTCGGCTCTCGCATCGATGAAGAGCGCAGCGAAATGCGATACGTGGTGC-GAATTGCAGAATCCCGCGAACC-ATCGAGTCTTTGAACGCAAGTTGCGCCCGAGGCCAAC-CGGCCGAGGGCACGTCCGCCTG---GGCGTCA-AGCGTTGCGTCGCTCC

Bulbophyllum_delitescens_KY966440 ACGAC-TCTCGGCAATGGATATCTCGGCTCTCGCATCGATGAAGAGCGCAGCGAAATGCGATACGTGGTGC-GAATTGCAGAATCCCGCGAACC-ATCGAGTCTTTGAACGCAAGTTGCGCCCGAGGCCAAC-CGGCCGAGGGCACGTCCGCCTG---GGCGTCA-AGCGTTGCGTCGCTCC

Bulbophyllum_delitescens_KY966441 ACGAC-TCTCGGCAATGGATATCTCGGCTCTCGCATCGATGAAGAGCGCAGCGAAATGCGATACGTGGTGC-GAATTGCAGAATCCCGCGAACC-ATCGAGTCTTTGAACGCAAGTTGCGCCCGAGGCCAAC-CGGCCGAGGGCACGTCCGCCTG---GGCGTCA-AGCGTTGCGTCGCTCC

Bulbophyllum_delitescens_MK164448 ACGAC-TCTCGGCAATGGATATCTCGGCTCTCGCATCGATGAAGAGCGCAGCGAAATGCGATACGTGGTGC-GAATTGCAGAATCCCGCGAACC-ATCGAGTCTTTGAACGCAAGTTGCGCCCGAGGCCAAC-CGGCCGAGGGCACGTCCGCCTG---GGCGTCA-AGCGTTGCGTCGCTCC

Bulbophyllum_delitescens_SG1286 ACGAC-TCTCGGCAATGGATATCTCGGCTCTCGCATCGATGAAGAGCGCAGCGAAATGCGATACGTGGTGC-GAATTGCAGAATCCCGCGAACC-ATCGAGTCTTTGAACGCAAGTTGCGCCCGAGGCCAAC-CGGCCGAGGGCACGTCCGCCTG---GGCGTCA-AGCGTTGCGTCGCTCC

Bulbophyllum_delitescens_SG1287 ACGAC-TCTCGGCAATGGATATCTCGGCTCTCGCATCGATGAAGAGCGCAGCGAAATGCGATACGTGGTGC-GAATTGCAGAATCCCGCGAACC-ATCGAGTCTTTGAACGCAAGTTGCGCCCGAGGCCGAC-CGGCCGAGGGCACGTCCGCCTG---GGCGTCA-AGCGTTGCGTCGCTCC

Bulbophyllum_delitescens_SG1288 ACGAC-TCTCGGCAATGGATATCTCGGCTCTCGCATCGATGAAGAGCGCAGCGAAATGCGATACGTGGTGC-GAATTGCAGAATCCCGCGAACC-ATCGAGTCTTTGAACGCAAGTTGCGCCCGAGGCCGAC-CGGCCGAGGGCACGTCCGCCTG---GGCGTCA-AGCGTTGCGTCGCTCC

Bulbophyllum_kwangtungense_JN619414 ACGAC-TCTCGGCAATGGATATCTCGGCTCTCGCATCGATGAAGAGCGCAGCGAAATGCGATACGTGGTGC-GAATTGCAGAATCACGCGAACC-ATCGAGTCTTTGAACGCAAGTTGCGCCCGAGGCCAAC-CGGCCGAGGGCACGTCCGCCTG---GGCGTCG-AGCGTTGCGTCGCTCC

Bulbophyllum_kwangtungense_KFBG27001 ACGAC-TCTCGGCAATGGATATCTCGGCTCTCGCATCGATGAAGAGCGCAGCGAAATGCGATACGTGGTGC-GAATTGCAGAATCCCGCGAACC-ATCGAGTCTTTGAACGCAAGTTGCGCCCGAGGCCAAC-CGGCCGAGGGCACGTCCGCCTG---GGCGTCA-AGCGTTGCGCCGCTCC

Bulbophyllum_kwangtungense_KFBG2798 ACGAC-TCTCGGCAATGGATATCTCGGCTCTCGCATCGATGAAGAGCGCAGCGAAATGCGATACGTGGTGC-GAATTGCAGAATCACGCGAACC-ATCGAGTCTTTGAACGCAAGTTGCGCCCGAGGCCAAC-CGGCCGAGGGCACGTCCGCCTG---GGCGTCA-AGCGTTGCGTCGCTCC

Bulbophyllum_kwangtungense_KFBG2820 ACGAC-TCTCGGCAATGGATATCTCGGCTCTCGCATCGATGAAGAGCGCAGCGAAATGCGATACGTGGTGC-GAATTGCAGAATCACGCGAACC-ATCGAGTCTTTGAACGCAAGTTGCGCCCGAGGCCAAC-CGGCCGAGGGCACGTCCGCCTG---GGCGTCA-AGCGTTGCGTCGCTCC

Bulbophyllum_kwangtungense_MK164466 ACGAC-TCTCGGCAATGGATATCTCGGCTCTCGCATCGATGAAGAGCGCAGCGAAATGCGATACGTGGTGC-GAATTGCAGAATCACGCGAACC-ATCGAGTCTTTGAACGCAAGTTGCGCCCGAGGCCAAC-CGGCCGAGGGCACGTCCGCCTG---GGCGTCA-AGCGTTGCGTCGCTCC

Bulbophyllum_kwangtungense_PK12070 ACGAC-TCTCGGCAATGGATATCTCGGCTCTCGCATCGATGAAGAGCGCAGCGAAATGCGATACGTGGTGC-GAATTGCAGAATCACGCGAACC-ATCGAGTCTTTGAACGCAAGTTGCGCCCGAGGCCAAC-CGGCCGAGGGCACGTCCGCCTG---GGCGTCA-AGCGTTGCGTCGCTCC

Bulbophyllum_odoratissimum_HQ114230 ACGAC-TCTCGGCAATGGATATCTCGGCTCTCGCATCGATGAAGAGCGCAGCGAAATGCGATACGTGGTGC-GAATTGCAGAATCCCGCGAACC-ATCGAGTCTTTGAACGCAAGTTGCGCCCGAGGCCAAC-CGGCCGAGGGCACGTCCGCCTG---GGCGTCA-AGCGTTGCGCCGCTCC

Bulbophyllum_odoratissimum_KF866242 ACGAC-TCTCGGCAATGGATATCTCGGCTCTCGCATCGATGAAGAGCGCAGCGAAATGCGATACGTGGTGC-GAATTGCAGAATCCCGCGAACC-ATCGAGTCTTTGAACGCAAGTTGCGCCCGAGGCCAAC-CGGCCGAGGGCACGTCCGCCTG---GGCGTCA-AGCGTTGCGCCGCTCC

Bulbophyllum_odoratissimum_KY966466 ACGAC-TCTCGGCAATGGATATCTCGGCTCTCGCATCGATGAAGAGCGCAGCGAAATGCGATACGTGGTGC-GAATTGCAGAATCCCGCGAACC-ATCGAGTCTTTGAACGCAAGTTGCGCCCGAGGCCAAC-CGGCCGAGGGCACGTCCGCCTG---GGCGTCA-AGCGTTGCGCCGCTCC

Bulbophyllum_odoratissimum_KY966467 ACGAC-TCTCGGCAATGGATATCTCGGCTCTCGCATCGATGAAGAGCGCAGCGAAATGCGATACGTGGTGC-GAATTGCAGAATCACGCGAACC-ATCGAGTCTTTGAACGCAAGTTGCGCCCGAGGCCAAC-CGGCCGAGGGCACGTCCGCCTG---GGCGTCA-AGCGTTGCGTCGCTCC

Bulbophyllum_odoratissimum_MK164483 ACGAC-TCTCGGCAATGGATATCTCGGCTCTCGCATCGATGAAGAGCGCAGCGAAATGCGATACGTGGTGC-GAATTGCAGAATCCCGCGAACC-ATCGAGTCTTTGAACGCAAGTTGCGCCCGAGGCCAAC-CGGCCGAGGGCACGTCCGCCTG---GGCGTCA-AGCGTTGCGCCGCTCC

Bulbophyllum_odoratissimum_PK12119 ACGAC-TCTCGGCAATGGATATCTCGGCTCTCGCATCGATGAAGAGCGCAGCGAAATGCGATACGTGGTGC-GAATTGCAGAATCCCGCGAACC-ATCGAGTCTTTGAACGCAAGTTGCGCCCGAGGCCAAC-CGGCCGAGGGCACGTCCGCCTG---GGCGTCA-AGCGTTGCGCCGCTCC

Bulbophyllum_odoratissimum_SG1275 ACGAC-TCTCGGCAATGGATATCTCGGCTCTCGCATCGATGAAGAGCGCAGCGAAATGCGATACGTGGTGC-GAATTGCAGAATCCCGCGAACC-ATCGAGTCTTTGAACGCAAGTTGCGCCCGAGGCCAAC-CGGCCGAGGGCACGTCCGCCTG---GGCGTCA-AGCGTTGCGCCGCTCC

Bulbophyllum_pectenveneris_JN619418 ACGAC-TCTCGGCAATGGATATCTCGGCTCTCGCATCGATGAAGAGCGCAGCGAAATGCGATACGTGGTGC-GAATTGCAGAATCCCGCGAACC-ATCGAGTCTTTGAACGCAAGTTGCGCCCGAGGCCGAC-CGGCCGAGGGCACGTCCGCCTG---GGCGTCA-AGCGTTGCGTCGCTCC

Bulbophyllum_pectenveneris_KY966470 ACGAC-TCTCGGCAATGGATATCTCGGCTCTCGCATCGATGAAGAGCGCAGCGAAATGCGATACGTGGTGC-GAATTGCAGAATCCCGCGAACC-ATCGAGTCTTTGAACGCAAGTTGCGCCCGAGGCCGAC-CGGCCGAGGGCACGTCCGCCTG---GGCGTCG-AGCGTTGCGTCGCTCC

Bulbophyllum_pectenveneris_KY966471 ACGAC-TCTCGGCAATGGATATCTCGGCTCTCGCATCGATGAAGAGCGCAGCGAAATGCGATACGTGGTGC-GAATTGCAGAATCCCGCGAACC-ATCGAGTCTTTGAACGCAAGTTGCGCCCGAGGCCGAC-CGGCCGAGGGCACGTCCGCCTG---GGCGTCG-AGCGTTGCGTCGCTCC

Bulbophyllum_pectenveneris_MK164486 ACGAC-TCTCGGCAATGGATATCTCGGCTCTCGCATCGATGAAGAGCGCAGCGAAATGCGATACGTGGTGC-GAATTGCAGAATCCCGCGAACC-ATCGAGTCTTTGAACGCAAGTTGCGCCCGAGGCCGAC-CGGCCGAGGGCACGTCCGCCTG---GGCGTCG-AGCGTTGCGTCGCTCC

Bulbophyllum_scabratum_KY966453 ACGAC-TCTCGGCAATGGATATCTCGGCTCTCGCATCGATGAAGAGCGCAGCGAAATGCGATGCGTGGTGC-GAATTGCAGAATCCCGCGAACC-ATCGAGTCTTTGAACGCAAGTTGCGCCCGAGGCCGAC-CGGCCGAGGGCACGCCCGCCTG---GGCGTCA-AGCGTCGCGTCGCTCC

Bulbophyllum_scabratum_MK164471 ACGAC-TCTCGGCAATGGATATCTCGGCTCTCGCATCGATGAAGAGCGCAGCGAAATGCGATACGTGGTGC-GAATTGCAGAATCCCGCGAACC-ATCGAGTCTTTGAACGCAAGTTGCGCCCGAGGCCGAC-CGGCCGAGGGCACGCCCGCCTG---GGCGTCA-AGCGTCGCGTCGCTCC

Bulbophyllum_scabratum_PK12041 ACGAC-TCTCGGCAATGGATATCTCGGCTCTCGCATCGATGAAGAGCGCAGCGAAATGCGATACGTGGTGC-GAATTGCAGAATCCCGCGAACC-ATCGAGTCTTTGAACGCAAGTTGCGCCCGAGGCCGAC-CGGCCGAGGGCACGCCCGCCTG---GGCGTCA-AGCGTCGCGTCGCTCC

Bulbophyllum_scabratum_PK12126 ACGAC-TCTCGGCAATGGATATCTCGGCTCTCGCATCGATGAAGAGCGCAGCGAAATGCGATACGTGGTGC-GAATTGCAGAATCCCGCGAACC-ATCGAGTCTTTGAACGCAAGTTGCGCCCGAGGCCGAC-CGGCCGAGGGCACGCCCGCCTG---GGCGTCA-AGCGTCGCGTCGCTCC

Bulbophyllum_scabratum_PK12127 ACGAC-TCTCGGCAATGGATATCTCGGCTCTCGCATCGATGAAGAGCGCAGCGAAATGCGATACGTGGTGC-GAATTGCAGAATCCCGCGAACC-ATCGAGTCTTTGAACGCAAGTTGCGCCCGAGGCCGAC-CGGCCGAGGGCACGCCCGCCTG---GGCGTCA-AGCGTCGCGTCGCTCC

Bulbophyllum_stenobulbon_KFBG2806 ACGAC-TCTCGGCAATGGATATCTCGGCTCTCGCATCGATGAAGAGCGCAGCGAAATGCGATACGTGGTGC-GAATTGCAGAATCACGCGAACC-ATCGAGTCTTTGAACGCAAGTTGCGCCCGAGGCCAAC-CGGCCGAGGGCACGTCCGCCTG---GGCGTCA-AGCGTTGCGTCGCTCC

Bulbophyllum_stenobulbon_MK164513 ACGAC-TCTCGGCAATGGATATCTCGGCTCTCGCATCGATGAAGAGCGCAGCGAAATGCGATACGTGGTGC-GAATTGCAGAATCACGCGAACC-ATCGAGTCTTTGAACGCAAGTTGCGCCCGAGGCCAAC-CGGCCGAGGGCACGTCCGCCTG---GGCGTCA-AGCGTTGCGTCGCTCC

Bulbophyllum_stenobulbon_PK12111 ACGAC-TCTCGGCAATGGATATCTCGGCTCTCGCATCGATGAAGAGCGCAGCGAAATGCGATACGTGGTGC-GAATTGCAGAATCACGCGAACC-ATCGAGTCTTTGAACGCAAGTTGCGCCCGAGGCCAAC-CGGCCGAGGGCACGTCCGCCTG---GGCGTCA-AGCGTTGCGTCGCTCC

Bulbophyllum_stenobulbon_PK12112 ACGAC-TCTCGGCAATGGATATCTCGGCTCTCGCATCGATGAAGAGCGCAGCGAAATGCGATACGTGGTGC-GAATTGCAGAATCACGCGAACC-ATCGAGTCTTTGAACGCAAGTTGCGCCCGAGGCCAAC-CGGCCGAGGGCACGTCCGCCTG---GGCGTCA-AGCGTTGCGTCGCTCC

Bulbophyllum_stenobulbon_PK12113 ACGAC-TCTCGGCAATGGATATCTCGGCTCTCGCATCGATGAAGAGCGCAGCGAAATGCGATACGTGGTGC-GAATTGCAGAATCACGCGAACC-ATCGAGTCTTTGAACGCAAGTTGCGCCCGAGGCCAAC-CGGCCGAGGGCACGTCCGCCTG---GGCGTCA-AGCGTTGCGTCGCTCC

Bulbophyllum_stenobulbon_SG1226 ACGAC-TCTCGGCAATGGATATCTCGGCTCTCGCATCGATGAAGAGCGCAGCGAAATGCGATACGTGGTGC-GAATTGCAGAATCACGCGAACC-ATCGAGTCTTTGAACGCAAGTTGCGCCCGAGGCCAAC-CGGCCGAGGGCACGTCCGCCTG---GGCGTCA-AGCGTTGCGTCGCTCC

Bulbophyllum_tigridum_KFBG468 ACGAC-TCTCGGCAATGGATATCTCGGCTCTCGCATCGATGAAGAGCGCAGCGAAATGCGATACGTGGTGC-GAATTGCAGAATCCCGCGAACC-ATCGAGTCTTTGAACGCAAGTTGCGCCCGAGGCCAGC-CGGCCGAGGGCACGTCYGCCTG---GGCGTCA-AGCGTTGCGTCGCTCC

Bulbophyllum_tigridum_KX455820 ACGAC-TCTCGGCAATGGATATCTCGGCTCTCGCATCGATGAAGAGCGCAGCGAAATGCGATACGTGGTGC-GAATTGCAGAATCCCGCGAACC-ATCGAGTCTTTGAACGCAAGTTGCGCCCGAGGCCAAC-CGGCCGAGGGCACGTCCGCCTG---GGCGTCA-AGCGTTGCGTCGCTCC

Bulbophyllum_tigridum_MK164520 ACGAC-TCTCGGCAATGGATATCTCGGCTCTCGCATCGATGAAGAGCGCAGCGAAATGCGATACGTGGTGC-GAATTGCAGAATCCCGCGAACC-ATCGAGTCTTTGAACGCAAGTTGCGCCCGAGGCCAGC-CGGCCGAGGGCACGTCCGCCTG---GGCGTCA-AGCGTTGCGTCGCTCC

Bulbophyllum_tigridum_SG1310 ACGAC-TCTCGGCAATGGATATCTCGGCTCTCGCATCGATGAAGAGCGCAGCGAAATGCGATACGTGGTGC-GAATTGCAGAATCCCGCGAACC-ATCGAGTCTTTGAACGCAAGTTGCGCCCGAGGCCAGC-CGGCCGAGGGCACGTCCGCCTG---GGCGTCA-AGCGTTGCGTCGCTCC

Bulbophyllum_tseanum_MK164524 ACGAC-TCTCGGCAATGGATATCTCGGCTCTCGCATCGATGAAGAGCGCAGCGAAATGCGATACGTGGTGC-GAATTGCAGAATCCCGCGAACC-ATCGAGTCTTTGAACGCAAGTTGCGCCCGAGGCCAAC-CGGCCGAGGGCACGTCCGCCTG---GGCGTCA-AGCGTCGCGTCGCTCC

Bulbophyllum_tseanum_SG1272 ACGAC-TCTCGGCAATGGATATCTCGGCTCTCGCATCGATGAAGAGCGCAGCGAAATGCGATACGTGGTGC-GAATTGCAGAATCCCGCGAACC-ATCGAGTCTTTGAACGCAAGTTGCGCCCGAGGCCAAC-CGGCCGAGGGCACGTCCGCCTG---GGCGTCA-AGCGTCGCGTCGCTCC

Bulbophyllum_tseanum_SG1616 ACGAC-TCTCGGCAATGGATATCTCGGCTCTCGCATCGATGAAGAGCGCAGCGAAATGCGATACGTGGTGC-GAATTGCAGAATCCCGCGAACC-ATCGAGTCTTTGAACGCAAGTTGCGCCCGAGGCCAAC-CGGCCGAGGGCACGTCCGCCTG---GGCGTCA-AGCGTCGCGTCGCTCC

Calanthe_graciliflora_AY882608 ATGAC-TCTCGGCAATGGATATCTCGGCTCTCGCATCGATGAAGAGCGCAGCGAAATGCGATACGTGGTGC-GAATTGCAGAATCCCGCGAACC-ATCGAGTCTTTGAACGCAAGTTGCGCCCGAGGCCAAT-CGGCCAAGGGCACGTCTGCCTG---GGCGTCA-AGCGTTGCATCGCTCT

Calanthe_graciliflora_KF560484 ATGAC-TCTCGGCAATGGATATCTCGGCTCTCGCATCGATGAAGAGCGCAGCGAAATGCGATACGTGGTGC-GAATTGCAGAATCCCGCGAACC-ATCGAGTCTTTGAACGCAAGTTGCGCCCGAGGCCAAT-CGGCCAAGGGCACGTCTGCCTG---GGCGTCA-AGCGTTGCATCGCTCT

Calanthe_graciliflora_KF560495 ATGAC-TCTCGGCAATGGATATCTCGGCTCTCGCATCGATGAAGAGCGCAGCGAAATGCGATACGTGGTGC-GAATTGCAGAATCCCGCGAACC-ATCGAGTCTTTGAACGCAAGTTGCGCCCGAGGCCAAT-CGGCCAAGGGCACGTCTGCCTG---GGCGTCA-AGCGTTGCATCGCTCT

Calanthe_graciliflora_PK12206 ATGAC-TCTCGGCAATGGATATCTCGGCTCTCGCATCGATGAAGAGCGCAGCGAAATGCGATACGTGGTGC-GAATTGCAGAATCCCGCGAACC-ATCGAGTCTTTGAACGCAAGTTGCGCCCGAGGCCAAT-CGGCCAAGGGCACGTCTGCCTG---GGCGTCA-AGCGTTGCATCGCTCT

Calanthe_graciliflora_PK12207 ATGAC-TCTCGGCAATGGATATCTCGGCTCTCGCATCGATGAAGAGCGCAGCGAAATGCGATACGTGGTGC-GAATTGCAGAATCCCGCGAACC-ATCGAGTCTTTGAACGCAAGTTGCGCCCGAGGCCAAT-CGGCCAAGGGCACGTCTGCCTG---GGCGTCA-AGCGTTGCATCGCTCT

Calanthe_graciliflora_SG1225 ATGAC-TCTCGGCAATGGATATCTCGGCTCTCGCATCGATGAAGAGCGCAGCGAAATGCGATACGTGGTGC-GAATTGCAGAATCCCGCGAACC-ATCGAGTCTTTGAACGCAAGTTGCGCCCGAGGCCAAT-CGGCCAAGGGCACGTCTGCCTG---GGCGTCA-AGCGTTGCATCGCTCT

Calanthe_masuca_KFBG11 ATGAC-TCTCGGCAATGGATATCTCGGCTCTCGCATCGATGAAGAGCGCAGCGAAATGCGATACGTGGTGC-GAATTGCAGAATCCCGCGAACC-ATCGAGTCTTTGAACGCAAGTTGCGCCCGAGGCCAAT-CGGCCAAGGGCACGTCTGCCTG---GGCGTCA-AGCGTTGCATCGCTCT

Calanthe_masuca_PK12110 ATGAC-TCTCGGCAATGGATATCTCGGCTCTCGCATCGATGAAGAGCGCAGCGAAATGCGATACGTGGTGC-GAATTGCAGAATCCCGCGAACC-ATCGAGTCTTTGAACGCAAGTTGCGCCCGAGGCCAAT-CGGCCAAGGGCACGTCTGCCTG---GGCGTCA-AGCGTTGCATCGCTCT

Calanthe_masuca_SG026 ATGAC-TCTCGGCAATGGATATCTCGGCTCTCGCATCGATGAAGAGCGCAGCGAAATGCGATACGTGGTGC-GAATTGCAGAATCCCGCGAACC-ATCGAGTCTTTGAACGCAAGTTGCGCCCGAGGCCAAT-CGGCCAAGGGCACGTCTGCCTG---GGCGTCA-AGCGTTGCATCGCTCT

Calanthe_masuca_SG1360 ATGAC-TCTCGGCAATGGATATCTCGGCTCTCGCATCGATGAAGAGCGCAGCGAAATGCGATACGTGGTGC-GAATTGCAGAATCCCGCGAACC-ATCGAGTCTTTGAACGCAAGTTGCGCCCGAGGCCAAT-CGGCCAAGGGCACGTCTGCCTG---GGCGTCA-AGCGTTGCATCGCTCT

Calanthe_masuca_SG1361 ATGAC-TCTCGGCAATGGATATCTCGGCTCTCGCATCGATGAAGAGCGCAGCGAAATGCGATACGTGGTGC-GAATTGCAGAATCCCGCGAACC-ATCGAGTCTTTGAACGCAAGTTGCGCCCGAGGCCAAT-CGGCCAAGGGCACGTCTGCCTG---GGCGTCA-AGCGTTGCATCGCTCT

Calanthe_speciosa_KFBG136 ACGAC-TCTCGGCAATGGATATCTCGGCTCTCGCATCGATGAAGAGCGCAGCGAAATGCGATACGTGGTGC-GAATTGCAGAATCCCGCGAACC-ATCGAGTCTTTGAACGCAAGTTGCGCCCGAGGCCAAC-CGGCCAAGGGCACGTCTGCCTG---GGCGTCA-AGCGTTGCATCGCTCT

Calanthe_speciosa_KY951546 ACGAC-TCTCGGCAATGGATATCTCGGCTCTCGCATCGATGAAGAGCGCAGCGAAATGCGATACGTGGTGC-GAATTGCAGAATCCCGCGAACC-ATCGAGTCTTTGAACGCAAGTTGCGCCCGAGGCCAAC-CGGCCAAGGGCACGTCTGCCTG---GGCGTCA-AGCGTTGCATCGCTCT

Calanthe_speciosa_PK12168 ACGAC-TCTCGGCAATGGATATCTCGGCTCTCGCATCGATGAAGAGCGCAGCGAAATGCGATACGTGGTGC-GAATTGCAGAATCCCGCGAACC-ATCGAGTCTTTGAACGCAAGTTGCGCCCGAGGCCAAC-CGGCCAAGGGCACGTCTGCCTG---GGCGTCA-AGCGTTGCATCGCTCT

Calanthe_speciosa_PK12169 ACGAC-TCTCGGCAATGGATATCTCGGCTCTCGCATCGATGAAGAGCGCAGCGAAATGCGATACGTGGTGC-GAATTGCAGAATCCCGCGAACC-ATCGAGTCTTTGAACGCAAGTTGCGCCCGAGGCCAAC-CGGCCAAGGGCACGTCTGCCTG---GGCGTCA-AGCGTTGCATCGCTCT

Calanthe_speciosa_SG1313 ACGAC-TCTCGGCAATGGATATCTCGGCTCTCGCATCGATGAAGAGCGCAGCGAAATGCGATACGTGGTGC-GAATTGCAGAATCCCGCGAACC-ATCGAGTCTTTGAACGCAAGTTGCGCCCGAGGCCAAC-CGGCCAAGGGCACGTCTGCCTG---GGCGTCA-AGCGTTGCATCGCTCT

Calanthe_speciosa_SG1368 ACGAC-TCTCGGCAATGGATATCTCGGCTCTCGCATCGATGAAGAGCGCAGCGAAATGCGATACGTGGTGC-GAATTGCAGAATCCCGCGAACC-ATCGAGTCTTTGAACGCAAGTTGCGCCCGAGGCCAAC-CGGCCAAGGGCACGTCTGCCTG---GGCGTCA-AGCGTTGCATCGCTCT

Calanthe_triplicata_AY882614 ATGAC-TCTCGGCAATGGATATCTCGGCTCTCGCATCGATGAAGAGCGCAGCGAAATGCGATACGTGGTGC-GAATTGCAGAATCCCGCGAACC-ATCGAGTCTTTGAACGCAAGTTGCGCCCGAGGCCAAT-CGGCCAAGGGCACGTCTGCCTG---GGCGTCA-AGCGTTGCATCGCTCT

Calanthe_triplicata_KF560480 ATGAC-TCTCGGCAATGGATATCTCGGCTCTCGCATCGATGAAGAGCGCAGCGAAATGCGATACGTGGTGC-GAATTGCAGAATCCCGCGAACC-ATCGAGTCTTTGAACGCAAGTTGCGCCCGAGGCCAAT-CGGCCAAGGGCACGTCTGCCTG---GGCGTCA-AGCGTTGCATCGCTCT

Calanthe_triplicata_KF560491 ATGAC-TCTCGGCAATGGATATCTCGGCTCTCGCATCGATGAAGAGCGCAGCGAAATGCGATACGTGGTGC-GAATTGCAGAATCCCGCGAACC-ATCGAGTCTTTGAACGCAAGTTGCGCCCGAGGCCAAT-CGGCCAAGGGCACGTCTGCCTG---GGCGTCA-AGCGTTGCATCGCTCT

Calanthe_triplicata_KFBG601 ATGAC-TCTCGGCAATGGATATCTCGGCTCTCGCATCGATGAAGAGCGCAGCGAAATGCGATACGTGGTGC-GAATTGCAGAATCCCGCGAACC-ATCGAGTCTTTGAACGCAAGTTGCGCCCGAGGCCAAT-CGGCCAAGGGCACGTCTGCCTG---GGCGTCA-AGCGTTGCATCGCTCT

Calanthe_triplicata_KM025154 ATGAC-TCTCGGCAATGGATATCTCGGCTCTCGCATCGATGAAGAGCGCAGCGAAATGCGATACGTGGTGC-GAATTGCAGAATCCCGCGAACC-ATCGAGTCTTTGAACGCAAGTTGCGCCCGAGGCCAAT-CGGCCAAGGGCACGTCTGCCTG---GGCGTCA-AGCGTTGCATCGCTCT

Calanthe_triplicata_KY966491 ATGAC-TCTCGGCAATGGATATCTCGGCTCTCGCATCGATGAAGAGCGCAGCGAAATGCGATACGTGRTGC-GAATTGCAGAATCCCGCGAACC-ATCGAGTCTTTGAACGCAAGTTGCGCCCGAGGCCAAT-CGGCCAAGGGCACGTCTGCCTG---GGCGTCA-AGCGTTGCATCGCTCT

Calanthe_triplicata_SG1311 ATGAC-TCTCGGCAATGGATATCTCGGCTCTCGCATCGATGAAGAGCGCAGCGAAATGCGATACGTGGTGC-GAATTGCAGAATCCCGCGAACC-ATCGAGTCTTTGAACGCAAGTTGCGCCCGAGGCCAAT-CGGCCAAGGGCACGTCTGCCTG---GGCGTCA-AGCGTTGCATCGCTCT

Cephalantheropsis_obcordata_KF560490 ACGAC-TCTCGGCAATGGATATCTCGGCTCTCGCATCGATGAAGAGCGCAGCGAAATGCGATACGTGGTGC-GAATTGCAGAATCCCGCGAACC-ATCGAGTCTTTGAACGCAAGTTGCGCCCGAGGCCAAC-CGGCCAAGGGCACGTCTGCCTG---GGCGTCA-AGCGTTGCATCGCTCT

Cephalantheropsis_obcordata_KFBG2520 ACGAC-TCTCGGCAATGGATATCTCGGCTCTCGCATCGATGAAGAGCGCAGCGAAATGCGATACGTGGTGC-GAATTGCAGAATCCCGCGAACC-ATCGAGTCTTTGAACGCAAGTTGCGCCCGAGGCCAAC-CGGCCAAGGGCACGTCTGCCTG---GGCGTCA-AGCGTTGCATCGCTCT

Cephalantheropsis_obcordata_KY966494 ACGAC-TCTCGGCAATGGATATCTCGGCTCTCGCATCGATGAAGAGCGCAGCGAAATGCGATACGTGGTGC-GAATTGCAGAATCCCGCGAACC-ATCGAGTCTTTGAACGCAAGTTGCGCCCGAGGCCAAC-CGGCCAAGGGCACGTCTGCCTG---GGCGTCA-AGCGTTGCATCGCTCT

Cephalantheropsis_obcordata_KY966495 ACGAC-TCTCGGCAATGGATATCTCGGCTCTCGCATCGATGAAGAGCGCAGCGAAATGCGATACGTGGTGC-GAATTGCAGAATCCCGCGAACC-ATCGAGTCTTTGAACGCAAGTTGCGCCCGAGGCCAAC-CGGCCAAGGGCACGTCTGCCTG---GGCGTCA-AGCGTTGCATCGCTCT

Cephalantheropsis_obcordata_PK12079 ACGAC-TCTCGGCAATGGATATCTCGGCTCTCGCATCGATGAAGAGCGCAGCGAAATGCGATACGTGGTGC-GAATTGCAGAATCCCGCGAACC-ATCGAGTCTTTGAACGCAAGTTGCGCCCGAGGCCAAC-CGGCCAAGGGCACGTCTGCCTG---GGCGTCA-AGCGTTGCATCGCTCT

Cephalantheropsis_obcordata_PK12080 ACGAC-TCTCGGCAATGGATATCTCGGCTCTCGCATCGATGAAGAGCGCAGCGAAATGCGATACGTGGTGC-GAATTGCAGAATCCCGCGAACC-ATCGAGTCTTTGAACGCAAGTTGCGCCCGAGGCCAAC-CGGCCAAGGGCACGTCTGCCTG---GGCGTCA-AGCGTTGCATCGCTCT

Cephalantheropsis_obcordata_PK12081 ACGAC-TCTCGGCAATGGATATCTCGGCTCTCGCATCGATGAAGAGCGCAGCGAAATGCGATACGTGGTGC-GAATTGCAGAATCCCGCGAACC-ATCGAGTCTTTGAACGCAAGTTGCGCCCGAGGCCAAC-CGGCCAAGGGCACGTCTGCCTG---GGCGTCA-AGCGTTGCATCGCTCT

Cephalantheropsis_obcordata_SG1208 ACGAC-TCTCGGCAATGGATATCTCGGCTCTCGCATCGATGAAGAGCGCAGCGAAATGCGATACGTGGTGC-GAATTGCAGAATCCCGCGAACC-ATCGAGTCTTTGAACGCAAGTTGCGCCCGAGGCCAAC-CGGCCAAGGGCACGTCTGCCTG---GGCGTCA-AGCGTTGCATCGCTCT

Cephalantheropsis_obcordata__KFBG140 ACGAC-TCTCGGCAATGGATATCTCGGCTCTCGCATCGATGAAGAGCGCAGCGAAATGCGATACGTGGTGC-GAATTGCAGAATCCCGCGAACC-ATCGAGTCTTTGAACGCAAGTTGCGCCCGAGGCCAAC-CGGCCAAGGGCACGTCTGCCTG---GGCGTCA-AGCGTTGCATCGCTCT

Cephalantheropsis_obcordata__SG1209 ACGAC-TCTCGGCAATGGATATCTCGGCTCTCGCATCGATGAAGAGCGCAGCGAAATGCGATACGTGGTGC-GAATTGCAGAATCCCGCGAACC-ATCGAGTCTTTGAACGCAAGTTGCGCCCGAGGCCAAC-CGGCCAAGGGCACGTCTGCCTG---GGCGTCA-AGCGTTGCATCGCTCT

Cheirostylis_clibborndyeri_KY966496 ATGAC-TCTCGGCAATGGATATCTTGGCTCTTGCATCGATGAAGAGCGCAGCGAAATGCGATACGTGGTGT-GAATTGCAGAATCCCGTGAACC-ATCAAATATTTGAACGCAAGTTGCGCCTGAGGCCAAT-TGGCTAAGGGCACGTCCGCCTG---GGCGCCA-AGCATTATATCGCTTC

Cheirostylis_clibborndyeri_PK12096 ATGAC-TCTCGGCAATGGATATCTTGGCTCTTGCATCGATGAAGAGCGCAGCGAAATGCGATACGTGGTGT-GAATTGCAGAATCCCGTGAACC-ATCAAATATTTGAACGCAAGTTGCGCCCGAGGCCAAT-TGGCTAAGGGCACGTCCGCCTG---GGCGTCA-AGCATTATATCGCTTC

Cheirostylis_clibborndyeri_SG1349 ATGAC-TCTCGGCAATGGATATCTTGGCTCTTGCATCGATGAAGAGCGCAGCGAAATGCGATACGTGGTGT-GAATTGCAGAATCCCGTGAACC-ATCAAATATTTGAACGCAAGTTGCGCCCGAGGCCAAT-TGGCTAAGGGCACGTCCGCCTG---GGCGTCA-AGCATTATATCGCTTC

Cheirostylis_monteiroi_PK12093 ATGAC-TCTCGGCAATGGATATCTTGGCTCTTGCATCGATGAAGAGCGCAGCGAAATGCGATACGTGGTGT-GAATTGCAGAATCCCGTGAACC-ATCAAATATTTGAACGCAAGTTGCGCCTGAGGCCAAT-TGGCTAAGGGCACGTCCGCCTG---GGCGTCA-AGCATTATATCGCTTC

Cheirostylis_monteiroi_PK12094 ATGAC-TCTCGGCAATGGATATCTTGGCTCTTGCATCGATGAAGAGCGCAGCGAAATGCGATACGTGGTGT-GAATTGCAGAATCCCGTGAACC-ATCAAATATTTGAACGCAAGTTGCGCCTGAGGCCAAT-TGGCTAAGGGCACGTCCGCCTG---GGCGTCA-AGCATTATATCGCTTC

Cheirostylis_monteiroi_SG1344 ATGAC-TCTCGGCAATGGATATCTTGGCTCTTGCATCGATGAAGAGCGCAGCGAAATGCGATACGTGGTGT-GAATTGCAGAATCCCGTGAACC-ATCAAATATTTGAACGCAAGTTGCGCCCGAGGCCAAT-TGGCTAAGGGCACGTCCGCCTG---GGCGTCA-AGCATTATATCGCTTC

Cheirostylis_yunnanensis_KT343978 ATGAC-TCTCGGCAATGGATATCTTGGCTCTTGCATCGATGAAGAGCGCAGCGAAATGCGATACGTGGTGT-GAATTGCAGAATCCCGTGAACC-ATCAAATATTTGAACGCAAGTTGCGCCCGAGGCCAAT-TGGCTAAGGGCACGTCCGCCTG---GGCGTCA-AGC-TTATATCGCT--

Cheirostylis_yunnanensis_PK12097 ATGAC-TCTCGGCAATGGATATCTTGGCTCTTGCATCGATGAAGAGCGCAGCGAAATGCGATACGTGGTGT-GAATTGCAGAATCCCGTGAACC-ATCAAATATTTGAACGCAAGTTGCGCCCGAGGCCAAT-TGGCTAAGGGCACGTCCGCCTG---GGCGTCA-AGCATTATATCGCTTC

Cheirostylis_yunnanensis_SG1227 ATGAC-TCTCGGCAATGGATATCTTGGCTCTTGCATCGATGAAGAGCGCAGCGAAATGCGATACGTGGTGT-GAATTGCAGAATCCCGTGAACC-ATCAAATATTTGAACGCAAGTTGCGCCCGAGGCCAAT-TGGCTAAGGGCACGTCCGCCTG---GGCGTCA-AGCATTATATCGCTTC

Cheirostylis_yunnanensis_SG1228 ATGAC-TCTCGGCAATGGATATCTTGGCTCTTGCATCGACGAAGAGCGCAGCGAAATGCGATACGTGGTGT-GAATTGCAGAATCCCGTGAACC-ATAAAATATTTGAACGCAAGTTGCGCCCGAGGCCAAT-TGGCTAAGGGCACGTCCGCCTG---GGCGTCA-AGCATTATATCGCTTC

Cheirostylis_yunnanensis_SG1229 ATGAC-TCTCGGCAATGGATATCTTGGCTCTTGCATCGATGAAGAGCGCAGCGAAATGCGATACGTGGTGT-GAATTGCAGAATCCCGTGAACC-ATCAAATATTTGAACGCAAGTTGCGCCCGAGGCCAAT-TGGCTAAGGGCACGTCCGCCTG---GGCGTCA-AGCATTATATCGCTTC

Chrysoglossum_assamicum_SG1622 ACGAC-TCTCGGCAATGGATATCTCGGCTCTCGCATCGATGAAGAGCGCAGCGAAATGCGATACGTGGTGC-GAATTGCAGAATCCCGCGAACC-ATCGAGTCTTTGAACGCAAGTTGCGCCCGAGGCCAAC-CGGCCAAGGGCACGTCTGCCTG---GGCGTCAA-GCGTTGCGTCGCTCC

Chrysoglossum_assamicum_SG1623 ACGAC-TCTCGGCAATGGATATCTCGGCTCTCGCATCGATGAAGAGCGCAGCGAAATGCGATACGTGGTGC-GAATTGCAGAATCCCGCGAACC-ATCGAGTCTTTGAACGCAAGTTGCGCCCGAGGCCAAC-CGGCCAAGGGCACGTCTGCCTG---GGCGTCAA-GCGTTGCGTCGCTCC

Cleisostoma_paniculatum_KFBG516 ACGAC-TCTCGACAATGGATATCTCGGCTCTCGCATCGATGAAGAGCGCAGCGAAATGCGATACGTGGTGC-GAATTGCAGAATCCCGCGAACC-ATCGAGTCTTTGAACGCAAGTTGCGCCCGAGGCCAAT-CGGTCGAGGGCACGTCCGCCTG---GGCGTCA-AGCGTTGCGCCGCTCC

Cleisostoma_paniculatum_KJ733401 ACGAC-TCTCGACAATGGATATCTCGGCTCTCGCATCGATGAAGAGCGCAGCGAAATGCGATACGTGGTGC-GAATTGCAGAATCCCGCGAACC-ATCGAGTCTTTGAACGCAAGTTGCGCCCGAGGCCAAT-CGGTCGAGGGCACGTCCGCCTG---GGCGTCA-AGCGTTGCGCCGCTCC

Cleisostoma_paniculatum_KT223752 ACGAC-TCTCGACAATGGATATCTCGGCTCTCGCATCGATGAAGAGCGCAGCGAAATGCGATACGTGGTGC-GAATTGCAGAATCCCGCGAACC-ATCGAGTCTTTGAACGCAAGTTGCGCCCGAGGCCAAT-CGGTCGAGGGCACGTCCGCCTG---GGCGTCA-AGCGTCGCGCCGCTCC

Cleisostoma_rostratum_KFBG789 ACGAC-TCTCGACAATGGATATCTCGGCTCTCGCATCGATGAAGAGCGCAGCGAAATGCGATACGTGGTGC-GAATTGCAGAATCCCGCGAACC-ATCGAGTCTTTGAACGCAAGTTGCGCCCGAGGCCAAT-CGGTCGAGGGCACGTCCGCCTG---GGCGTCA-AGCGTCGCGCCGCTCC

Cleisostoma_rostratum_KJ733404 ACGAC-TCTCGACAATGGATATCTCGGCTCTCGCATCGATGAAGAGCGCAGCGAAATGCGATACGTGGTGC-GAATTGCAGAATCCCGCGAACC-ATCGAGTCTTTGAACGCAAGTTGCGCCCGAGGCCAAT-CGGTCGAGGGCACGTCCGCCTG---GGCGTCA-AGCGTCGCGCCGCTCC

Cleisostoma_rostratum_KY966502 ACGAC-TCTCGACAATGGATATCTCGGCTCTCGCATCGATGAAGAGCGCAGCGAAATGCGATACGTGGTGC-GAATTGCAGAATCCCGCGAACC-ATCGAGTCTTTGAACGCAAGTTGCGCCCGAGGCCAAT-CGGTCGAGGGCACGTCCGCCTG---GGCGTCA-AGCGTCGCGCCGCTCC

Cleisostoma_rostratum_PK12089 ACGAC-TCTCGACAATGGATATCTCGGCTCTCGCATCGATGAAGAGCGCAGCGAAATGCGATACGTGGTGC-GAATTGCAGAATCCCGCGAACC-ATCGAGTCTTTGAACGCAAGTTGCGCCCGAGGCCAAT-CGGTCGAGGGCACGTCCGCCTG---GGCGTCA-AGCGTCGCGCCGCTCC

Cleisostoma_rostratum_PK12101 ACGAC-TCTCGACAATGGATATCTCGGCTCTCGCATCGATGAAGAGCGCAGCGAAATGCGATACGTGGTGC-GAATTGCAGAATCCCGCGAACC-ATCGAGTCTTTGAACGCAAGTTGCGCCCGAGGCCAAT-CGGTCGAGGGCACGTCCGCCTG---GGCGTCA-AGCGTCGCGCCGCTCC

Cleisostoma_rostratum_PK12158 ACGAC-TCTCGACAATGGATATCTCGGCTCTCGCATCGATGAAGAGCGCAGCGAAATGCGATACGTGGTGC-GAATTGCAGAATCCCGCGAACC-ATCGAGTCTTTGAACGCAAGTTGCGCCCGAGGCCAAT-CGGTCGAGGGCACGTCCGCCTG---GGCGTCA-AGCGTCGCGCCGCTCC

Cleisostoma_rostratum_SG1200 ACGAC-TCTCGACAATGGATATCTCGGCTCTCGCATCGATGAAGAGCGCAGCGAAATGCGATACGTGGTGC-GAATTGCAGAATCCCGCGAACC-ATCGAGTCTTTGAACGCAAGTTGCGCCCGAGGCCAAT-CGGTCGAGGGCACGTCCGCCTG---GGCGTCA-AGCGTCGCGCCGCTCC

Cleisostoma_rostratum_SG1301 ACGAC-TCTCGACAATGGATATCTCGGCTCTCGCATCGATGAAGAGCGCAGCGAAATGCGATACGTGGTGC-GAATTGCAGAATCCCGCGAACC-ATCGAGTCTTTGAACGCAAGTTGCGCCCGAGGCCAAT-CGGTCGAGGGCACGTCCGCCTG---GGCGTCA-AGCGTCGCGCCGCTCC

Cleisostoma_simondii_KJ733405 ACGAC-TCTCGACAATGGATATCTCGGCTCTCGCATCGATGAAGAGCGCAGCGAAATGCGATACGTGGTGC-GAATTGCAGAATCCCGCGAACC-ATCGAGTCTTTGAACGCAAGTTGCGCCCGAGGCCAAT-CGGTCGAGGGCACGTCCGCCTG---GGCGTCA-AGCGTTGCGCCGCTCC

Cleisostoma_simondii_KY966503 ACGAC-TCTCGACAATGGATATCTCGGCTCTCGCATCGATGAAGAGCGCAGCGAAATGCGATACGTGGTGC-GAATTGCAGAATCCCGCGAACC-ATCGAGTCTTTGAACGCAAGTTGCGCCCGAGGCCAAT-CGGTCGAGGGCACGTCCGCCTG---GGCGTCA-AGCGTTGCGCCGCTCC

Cleisostoma_simondii_KY966504 ACGAC-TCTCGACAATGGATATCTCGGCTCTCGCATCGATGAAGAGCGCAGCGAAATGCGATACGTGGTGC-GAATTGCAGAATCCCGCGAACC-ATCGAGTCTTTGAACGCAAGTTGCGCCCGAGGCCAAT-CGGTCGAGGGCACGTCCGCCTG---GGCGTCA-AGCGTTGCGCCGCTCC

Cleisostoma_simondii_MG822849 ACGAC-TCTCGACAATGGATATCTCGGCTCTCGCATCGATGAAGAGCGCAGCGAAATGCGATACGTGGTGC-GAATTGCAGAATCCCGCGAACC-ATCGAGTCTTTGAACGCAAGTTGCGCCCGAGGCCAAT-CGGTCGAGGGCACGTCCGCCTG---GGCGTCA-AGCGTTGCGCCGCTCC

Cleisostoma_simondii_PK12176 ACGAC-TCTCGACAATGGATATCTCGGCTCTCGCATCGATGAAGAGCGCAGCGAAATGCGATACGTGGTGC-GAATTGCAGAATCCCGCGAACC-ATCGAGTCTTTGAACGCAAGTTGCGCCCGAGGCCAAT-CGGTCGAGGGCACGTCCGCCTG---GGCGTCA-AGCGTTGCGCCGCTCC

Cleisostoma_simondii_SG1314 ACGAC-TCTCGACAATGGATATCTCGGCTCTCGCATCGATGAAGAGCGCAGCGAAATGCGATACGTGGTGC-GAATTGCAGAATCCCGCGAACC-ATCGAGTCTTTGAACGCAAGTTGCGCCCGAGGCCAAT-CGGTCGAGGGCACGTCCGCCTG---GGCGTCA-AGCGTTGCGCCGCTCC

Cleisostoma_simondii_SG1327 ACGAC-TCTCGACAATGGATATCTCGGCTCTCGCATCGATGAAGAGCGCAGCGAAATGCGATACGTGGTGC-GAATTGCAGAATCCCGCGAACC-ATCGAGTCTTTGAACGCAAGTTGCGCCCGAGGCCAAT-CGGTCGAGGGCACGTCCGCCTG---GGCGTCA-AGCGTTGCGCCGCTCC

Cleisostoma_simondii_SG1328 ACGAC-TCTCGACAATGGATATCTCGGCTCTCGCATCGATGAAGAGCGCAGCGAAATGCGATACGTGGTGC-GAATTGCAGAATCCCGCGAACC-ATCGAGTCTTTGAACGCAAGTTGCGCCCGAGGCCAAT-CGGTCGAGGGCACGTCCGCCTG---GGCGTCA-AGCGTTGCGCCGCTCC

Cleisostoma_simondii_SG1329 ACGAC-TCTCGACAATGGATATCTCGGCTCTCGCATCGATGAAGAGCGCAGCGAAATGCGATACGTGGTGC-GAATTGCAGAATCCCGCGAACC-ATCGAGTCTTTGAACGCAAGTTGCGCCCGAGGCCAAT-CGGTCGAGGGCACGTCCGCCTG---GGCGTCA-AGCGTTGCGCCGCTCC

Cleisostoma_simondii_SG1330 ACGAC-TCTCGACAATGGATATCTCGGCTCTCGCATCGATGAAGAGCGCAGCGAAATGCGATACGTGGTGC-GAATTGCAGAATCCCGCGAACC-ATCGAGTCTTTGAACGCAAGTTGCGCCCGAGGCCAAT-CGGTCGAGGGCACGTCCGCCTG---GGCGTCA-AGCGTTGCGCCGCTCC

Cleisostoma_simondii_var_guangdongense_KFBG2212 ACGAC-TCTCGACAATGGATATCTCGGCTCTCGCATCGATGAAGAGCGCAGCGAAATGCGATACGTGGTGC-GAATTGCAGAATCCCGCGAACC-ATCGAGTCTTTGAACGCAAGTTGCGCCCGAGGCCAAT-CGGTCGAGGGCACGTCCGCCTG---GGCGTCA-AGCGTTGCGCCGCTCC

Cleisostoma_simondii_var_guangdongense_KJ733406 ACGAC-TCTCGACAATGGATATCTCGGCTCTCGCATCGATGAAGAGCGCAGCGAAATGCGATACGTGGTGC-GAATTGCAGAATCCCGCGAACC-ATCGAGTCTTTGAACGCAAGTTGCGCCCGAGGCCAAT-CGGTCGAGGGCACGTCCGCCTG---GGCGTCA-AGCGTTGCGCCGCTCC

Coelogyne_cantonensis_KY966649 ACGAC-TCTCGGCAATGGATATCTCGGCTCTCGCATCGATGAAGAGCGCAGCGAAATGCGATACGTGGTGC-GAATTGCAGAATCCCGCGAACC-ATCGAGTCTTTGAACGCAAGTTGCGCCCGAGGCCAAC-CGGCCAAGGGCACGTCTGCCTG---GGCGTCA-AGCGTTGCGTCGCTCC

Coelogyne_cantonensis_SG1239 ACGAC-TCTCGGCAATGGATATCTCGGCTCTCGCATCGATGAAGAGCGCAGCGAAATGCGATACGTGGTGC-GAATTGCAGAATCCCGCGAACC-ATCGAGTCTTTGAACGCAAGTTGCGCCCGAGGCCAAC-CGGCCAAGGGCACGTCTGCCTG---GGCGTCA-AGCGTTGCGTCGCTCC

Coelogyne_cantonensis_SG1339 ACGAC-TCTCGGCAATGGATATCTCGGCTCTCGCATCGATGAAGAGCGCAGCGAAATGCGATACGTGGTGC-GAATTGCAGAATCCCGCGAACC-ATCGAGTCTTTGAACGCAAGTTGCGCCCGAGGCCAAC-CGGCCAAGGGCACGTCTGCCTG---GGCGTCA-AGCGTTGCGTCGCTCC

Coelogyne_cantonensis_SG1388 ACGAC-TCTCGGCAATGGATATCTCGGCTCTCGCATCGATGAAGAGCGCAGCGAAATGCGATACGTGGTGC-GAATTGCAGAATCCCGCGAACC-ATCGAGTCTTTGAACGCAAGTTGCGCCCGAGGCCAAC-CGGCCAAGGGCACGTCTGCCTG---GGCGTCA-AGCGTTGCGTCGCTCC

Coelogyne_chinensis_EU592035 ACGAC-TCTCGGCAATGGATATCTCGGCTCTCGCATCGATGAAGAGCGCAGCGAAATGCGATACGTGGTGC-GAATTGCAGAATCCCGCGAACC-ATCGAGTCTTTGAACGCAAGTTGCGCCCGAGGCCAAC-CGGCCAAGGGCACGTCTGCCTG---GGCGTCA-AGCGTTGCGTCGCTCC

Coelogyne_chinensis_KY966650 ACGAC-TCTCGGCAATGGATATCTCGGCTCTCGCATCGATGAAGAGCGCAGCGAAATGCGATACGTGGTGC-GAATTGCAGAATCCCGCGAACC-ATCGAGTCTTTGAACGCAAGTTGCGCCCGAGGCCAAC-CGGCCAAGGGCACGTCTGCCTG---GGCGTCA-AGCGTTGCGTCGCTCC

Coelogyne_chinensis_KY966651 ACGAC-TCTCGGCAATGGATATCTCGGCTCTCGCATCGATGAAGAGCGCAGCGAAATGCGATACGTGGTGC-GAATTGCAGAATCCCGCGAACC-ATCGAGTCTTTGAACGCAAGTTGCGCCCGAGGCCAAC-CGGCCAAGGGCACGTCTGCCTG---GGCGTCA-AGCGTTGCGTCGCTCC

Coelogyne_chinensis_PK12114 ACGAC-TCTCGGCAATGGATATCTCGGCTCTCGCATCGATGAAGAGCGCAGCGAAATGCGATACGTGGTGC-GAATTGCAGAATCCCGCGAACC-ATCGAGTCTTTGAACGCAAGTTGCGCCCGAGGCCAAC-CGGCCAAGGGCACGTCTGCCTG---GGCGTCA-AGCGTTGCGTCGCTCC

Coelogyne_chinensis_PK12115 ACGAC-TCTCGGCAATGGATATCTCGGCTCTCGCATCGATGAAGAGCGCAGCGAAATGCGATACGTGGTGC-GAATTGCAGAATCCCGCGAACC-ATCGAGTCTTTGAACGCAAGTTGCGCCCGAGGCCAAC-CGGCCAAGGGCACGTCTGCCTG---GGCGTCA-AGCGTTGCGTCGCTCC

Coelogyne_chinensis_SG1232 ACGAC-TCTCGGCAATGGATATCTCGGCTCTCGCATCGATGAAGAGCGCAGCGAAATGCGATACGTGGTGC-GAATTGCAGAATCCCGCGAACC-ATCGAGTCTTTGAACGCAAGTTGCGCCCGAGGCCAAC-CGGCCAAGGGCACGTCTGCCTG---GGCGTCA-AGCGTTGCGTCGCTCC

Coelogyne_chinensis_SG1251 ACGAC-TCTCGGCAATGGATATCTCGGCTCTCGCATCGATGAAGAGCGCAGCGAAATGCGATACGTGGTGC-GAATTGCAGAATCCCGCGAACC-ATCGAGTCTTTGAACGCAAGTTGCGCCCGAGGCCAAC-CGGCCAAGGGCACGTCTGCCTG---GGCGTCA-AGCGTTGCGTCGCTCC

Coelogyne_fimbriata_AF302745 ACGAC-TCTCGGCAATGGATATCTCGGCTCTCGCATCGATGAAGAGCGCAGCGAAATGCGATACGTGGTGC-GAATTGCAGAATCCCGCGAACC-ATCGAGTCTTTGAACGCAAGTTGCGCCCGAGGCCAAC-CGGCCAAGGGCACGTCTGCCTG---GGCGTCA-AGCGTTGCGTCGCTCC

Coelogyne_fimbriata_EU441205 ACGAC-TCTCGGCAATGGATATCTCGGCTCTCGCATCGATGAAGAGCGCAGCGAAATGCGATACGTGGTGC-GAATTGCAGAATCCCGCGAACC-ATCGAGTCTTTGAACGCAAGTTGCGCCCGAGGCCAAC-CGGCCAAGGGCACGTCTGCCTG---GGCGTCA-AGCGTTGCGTCGCTCC

Coelogyne_fimbriata_JF422074 ACGAC-TCTCGGCAATGGATATCTCGGCTCTCGCATCGATGAAGAGCGCAGCGAAATGCGATACGTGGTGC-GAATTGCAGAATCCCGCGAACC-ATCGAGTCTTTGAACGCAAGTTGCGCCCGAGGCCAAC-CGGCCAAGGGCACGTCTGCCTG---GGCGTCA-AGCGTTGCGTCGCTCC

Coelogyne_fimbriata_KFBG411 ACGAC-TCTCGGCAATGGATATCTCGGCTCTCGCATCGATGAAGAGCGCAGCGAAATGCGATACGTGGTGC-GAATTGCAGAATCCCGCGAACC-ATCGAGTCTTTGAACGCAAGTTGCGCCCGAGGCCAAC-CGGCCAAGGGCACGTCTGCCTG---GGCGTCA-AGCGTTGCGTCGCTCC

Coelogyne_fimbriata_KFBG523 ACGAC-TCTCGGCAATGGATATCTCGGCTCTCGCATCGATGAAGAGCGCAGCGAAATGCGATACGTGGTGC-GAATTGCAGAATCCCGCGAACC-ATCGAGTCTTTGAACGCAAGTTGCGCCCGAGGCCAAC-CGGCCAAGGGCACGTCTGCCTG---GGCGTCA-AGCGTTGCGTCGCTCC

Coelogyne_fimbriata_KR857330 ACGAC-TCTCGGCAATGGATATCTCGGCTCTCGCATCGATGAAGAGCGCAGCGAAATGCGATACGTGGTGC-GAATTGCAGAATCCCGCGAACC-ATCGAGTCTTTGAACGCAAGTTGCGCCCGAGGCCAAC-CGGCCAAGGGCACGTCTGCCTG---GGCGTCA-AGCGTTGCGTCGCTCC

Coelogyne_fimbriata_KY966506 ACGAC-TCTCGGCAATGGATATCTCGGCTCTCGCATCGATGAAGAGCGCAGCGAAATGCGATACGTGGTGC-GAATTGCAGAATCCCGCGAACC-ATCGAGTCTTTGAACGCAAGTTGCGCCCGAGGCCAAC-CGGCCAAGGGCACGTCTGCCTG---GGCGTCA-AGCGTTGCGTCGCTCC

Coelogyne_fimbriata_SG1059 ACGAC-TCTCGGCAATGGATATCTCGGCTCTCGCATCGATGAAGAGCGCAGCGAAATGCGATACGTGGTGC-GAATTGCAGAATCCCGCGAACC-ATCGAGTCTTTGAACGCAAGTTGCGCCCGAGGCCAAC-CGGCCAAGGGCACGTCTGCCTG---GGCGTCA-AGCGTTGCGTCGCTCC

Coelogyne_fimbriata_SG1061F ACGAC-TCTCGGCAATGGATATCTCGGCTCTCGCATCGATGAAGAGCGCAGCGAAATGCGATACGTGGTGC-GAATTGCAGAATCCCGCGAACC-ATCGAGTCTTTGAACGCAAGTTGCGCCCGAGGCCAAC-CGGCCAAGGGCACGTCTGCCTG---GGCGTCA-AGCGTTGCGTCGCTCC

Coelogyne_fimbriata_SG1062D ACGAC-TCTCGGCAATGGATATCTCGGCTCTCGCATCGATGAAGAGCGCAGCGAAATGCGATACGTGGTGC-GAATTGCAGAATCCCGCGAACC-ATCGAGTCTTTGAACGCAAGTTGCGCCCGAGGCCAAC-CGGCCAAGGGCACGTCTGCCTG---GGCGTCA-AGCGTTGCGTCGCTCC

Coelogyne_fimbriata_SG1079 ACGAC-TCTCGGCAATGGATATCTCGGCTCTCGCATCGATGAAGAGCGCAGCGAAATGCGATACGTGGTGC-GAATTGCAGAATCCCGCGAACC-ATCGAGTCTTTGAACGCAAGTTGCGCCCGAGGCCAAC-CGGCCAAGGGCACGTCTGCCTG---GGCGTCA-AGCGTTGCGTCGCTCC

Coelogyne_fimbriata_var_leungiana_KFBG640 ACGAC-TCTCGGCAATGGATATCTCGGCTCTCGCATCGATGAAGAGCGCAGCGAAATGCGATACGTGGTGC-GAATTGCAGAATCCCGCGAACC-ATCGAGTCTTTGAACGCAAGTTGCGCCCGAGGCCAAC-CGGCCAAGGGCACGTCTGCCTG---GGCGTCA-AGCGTTGCGTCGCTCC

Coelogyne_fimbriata_var_leungiana_KY966507 ACGAC-TCTCGGCAATGGATATCTCGGCTCTCGCATCGATGAAGAGCGCAGCGAAATGCGATACGTGGTGC-GAATTGCAGAATCCCGCGAACC-ATCGAGTCTTTGAACGCAAGTTGCGCCCGAGGCCAAC-CGGCCAAGGGCACGTCTGCCTG---GGCGTCA-AGCGTTGCGTCGCTCC

Coelogyne_fimbriata_var_leungiana_SG1058A ACGAC-TCTCGGCAATGGATATCTCGGCTCTCGCATCGATGAAGAGCGCAGCGAAATGCGATACGTGGTGC-GAATTGCAGAATCCCGCGAACC-ATCGAGTCTTTGAACGCAAGTTGCGCCCGAGGCCAAC-CGGCCAAGGGCACGTCTGCCTG---GGCGTCA-AGCGTTGCGTCGCTCC

Collabium_chinense_KF560544 ACGAC-TCTCGGCAATGGATATCTCGGCTCTCGCATCGATGAAGAGCGCAGCGAAATGCGATACGTGGTGC-GAATTGCAGAATCCCGCGAACC-ATCGAGTCTTTGAACGCAAGTTGCGCCCGAGGCCAAC-CGGCCAAGGGCACGTCTGCCTG---GGCGTCA-AGCGTTGCGTCGCTCC

Collabium_chinense_KM025156 ACGAC-TCTCGGCAATGGATATCTCGGCTCTCGCATCGATGAAGAGCGCAGCGAAATGCGATACGTGGTGC-GAATTGCAGAATCCCGCGAACC-ATCGAGTCTTTGAACGCAAGTTGCGCCCGAGGCCAAC-CGGCCAAGGGCACGTCTGCCTG---GGCGTCA-AGCGTTGCGTCGCTCC

Crepidium_acuminatum_AB290884 ATGAC-TCTCGGCAATGGATATCTCGGCTCTTGCATCGATGAAGAGCGCAGCAAAATGCGATACGTGATGC-GAATTGCAGAATCCCGCGAACC-ATCGAGTTTTTGAACGCAAGTTGCGCCCGAGGCCAAC-CGGTCAAGGGCACGTTTTCCTG---GGTGTCA-AGCGTTGCTTCGCTTT

Crepidium_acuminatum_JN114478 ATGAC-TCTCGGCAATGGATATCTCGGCTCTTGCATCGATGAAGAGCGCAGCAAAATGCGATACGTGATGC-GAATTGCAGAATCCCGCGAACC-ATCGAGTTTTTGAACGCAAGTTGCGCCCGAGGCCAAC-CGGTCAAGGGCACGTTTTCCTG---GGTGTCA-AGCGTTGCTTCGCTTT

Crepidium_acuminatum_JN114479 ATGAC-TCTCGGCAATGGATATCTCGGCTCTTGCATCGATGAAGAGCGCAGCAAAATGCGATACGTGATGC-GAATTGCAGAATCCCGCGAACC-ATCGAGTTTTTGAACGCAAGTTGCGCCCGAGGCCAAC-CGGTCAAGGGCACGTTTTCCTG---GGTGTCA-AGCGTTGCTTCGCTTT

Crepidium_acuminatum_JN114480 ATGAC-TCTCGGCAATGGATATCTCGGCTCTTGCATCGATGAAGAGCGCAGCAAAATGCGATACGTGATGC-GAATTGCAGAATCCCGCGAACC-ATCGAGTTTTTGAACGCAAGTTGCGCCCGAGGCCAAC-CGGTCAAGGGCACGTTTTCCTG---GGTGTCA-AGCGTTGCTTCGCTTT

Crepidium_acuminatum_JN114481 ATGAC-TCTCGGCAATGGATATCTCGGCTCTTGCATCGATGAAGAGCGCAGCAAAATGCGATACGTGATGC-GAATTGCAGAATCCCGCGAACC-ATCGAGTTTTTGAACGCAAGTTGCGCCCGAGGCCAAC-CGGTCAAGGGCACGTTTTCCTG---GGTGTCA-AGCGTTGCTTCGCTTT

Crepidium_acuminatum_JN114482 ATGAC-TCTCGGCAATGGATATCTCGGCTCTTGCATCGATGAAGAGCGCAGCAAAATGCGATACGTGATGC-GAATTGCAGAATCCCGCGAACC-ATCGAGTTTTTGAACGCAAGTTGCGCCCGAGGCCAAC-CGGTCAAGGGCACGTTTTCCTG---GGTGTCA-AGCGTTGCTTCGCTTT

Crepidium_acuminatum_KJ459274 ATGAC-TCTCGGCAATGGATATCTCGGCTCTTGCATCGATGAAGAGCGCAGCAAAATGCGATACGTGATGC-GAATTGCAGAATCCCGCGAACC-ATCGAGTTTTTGAACGCAAGTTGCGCCCGAGGCCAAC-CGGTCAAGGGCACGTTTTCCTG---GGTGTCA-AGCGTTGCTTCGCTTT

Crepidium_acuminatum_KX277725 ATGAC-TCTCGGCAATGGATATCTCGGCTCTTGCATCGATGAAGAGCGCAGCAAAATGCGATACGTGATGC-GAATTGCAGAATCCCGCGAACC-ATCGAGTTTTTGAACGCAAGTTGCGCCCGAGGCCAAC-CGGTCAAGGGCACGTTTTCCTG---GGTGTCA-AGCGTTGCTTCGCTTT

Crepidium_acuminatum_MF287967 ATGAC-TCTCGGCAATGGATATCTCGGCTCTTGCATCGATGAAGAGCGCAGCAAAATGCGATACGTGATGC-GAATTGCAGAATCCCGCGAACC-ATCGAGTTTTTGAACGCAAGTTGCGCCCGAGGCCAAC-CGGTCAAGGGCACGTTTTCCTG---GGTGTCA-AGCGTTGCTTCGCTTT

Crepidium_allanii_KFBG4610 ATGAC-TCTCGGCAATGGATATCTCGGCTCTTGCATCGATGAAGAGCGCAGCAAAATGCGATACGTGATGC-GAATTGCAGAATCCCGCGAACC-ATCGAGTTTTTGAACGCAAGTTGCGCCCGAGGCCAAC-CGGTCAAGGGCACGTTTTCCTG---GGTGTCA-AGCTTTGCTTCGCTTT

Crepidium_allanii_KFBG4611 ATGAC-TCTCGGCAATGGATATCTCGGCTCTTGCATCGATGAAGAGCGCAGCAAAATGCGATACGTGATGC-GAATTGCAGAATCCCGCGAACC-ATCGAGTTTTTGAACGCAAGTTGCGCCCGAGGCCAAC-CGGTCAAGGGCACGTTTTCCTG---GGTGTCA-AGCTTTGCTTCGCTTT

Crepidium_purpureum_SG1193 ATGAC-TCTCGGCAATGGATATCTCGGCTCTTGCATCGATGAAGAGCGCAGCAAAATGCGATACGTGATGC-GAATTGCAGAATCCCGCGAACC-ATCGAGTTTTTGAACGCAAGTTGCGCCCGAGGCCAAC-CGGTCAAGGGCACGTCTTCCTG---GGTGTCA-AGCGTTGCTACGCTTT

Crepidium_purpureum_SG1194 ATGAC-TCTCGGCAATGGATATCTCGGCTCTTGCATCGATGAAGAGCGCAGCAAAATGCGATACGTGATGC-GAATTGCAGAATCCCGCGAACC-ATCGAGTTTTTGAACGCAAGTTGCGCCCGAGGCCAAC-CGGTCAAGGGCACGTCTTCCTG---GGTGTCA-AGCGTTGCTACGCTTT

Crepidium_purpureum_SG1379 ATGAC-TCTCGGCAATGGATATCTCGGCTCTTGCATCGATGAAGAGCGCAGCAAAATGCGATACGTGATGC-GAATTGCAGAATCCCGCGAACC-ATCGAGTTTTTGAACGCAAGTTGCGCCCGAGGCCAAC-CGGTCAAGGGCACGTCTTCCTG---GGTGTCA-AGCGTTGCTACGCTTT

Cryptochilus_roseus_KFBG2118 ACGAC-TCTCGGCAATGGATATCTCGGCTCTCGCATCGATGAAGAGCGCAGCGAAATGCGATACGTGGTGT-GAATTGCAGAATCCCGCGAACC-ATCGAGTCTTTGAACGCAAGTTGCGCCCGAGGCCAAC-CGGCTGAGGGCACGTCTGCCTG---GGCGTCA-AGCATTATGTCACTCC

Cryptochilus_roseus_KY239358 ACGAC-TCTCGGCAATGGATATCTCGGCTCTCGCATCGATGAAGAGCGCAGCGAAATGCGATACGTGGTGT-GAATTGCAGAATCCCGCGAACC-ATCGAGTCTTTGAACGCAAGTTGCGCCCGAGGCCAAC-CGGCTGAGGGCACGTCTGCCTG---GGCGTCA-AGCATTATGTCACTCC

Cryptochilus_roseus_KY966513 ACGAC-TCTCGGCAATGGATATCTCGGCTCTCGCATCGATGAAGAGCGCAGCGAAATGCGATACGTGGTGT-GAATTGCAGAATCCCGCGAACC-ATCGAGTCTTTGAACGCAAGTTGCGCCCGAGGCCAAC-CGGCTGAGGGCACGTCTGCCTG---GGCGTCA-AGCATTATGTCACTCC

Cryptochilus_roseus_KY966514 ACGAC-TCTCGGCAATGGATATCTCGGCTCTCGCATCGATGAAGAGCGCAGCGAAATGCGATACGTGGTGT-GAATTGCAGAATCCCGCGAACC-ATCGAGTCTTTGAACGCAAGTTGCGCCCGAGGCCAAC-CGGCTGAGGGCACGTCTGCCTG---GGCGTCA-AGCATTATGTCACTCC

Cryptochilus_roseus_PK12087 ACGAC-TCTCGGCAATGGATATCTCGGCTCTCGCATCGATGAAGAGCGCAGCGAAATGCGATACGTGGTGT-GAATTGCAGAATCCCGCGAACC-ATCGAGTCTTTGAACGCAAGTTGCGCCCGAGGCCAAC-CGGCTGAGGGCACGTCTGCCTG---GGCGTCA-AGCATTATGTCACTCC

Cryptochilus_roseus_PK12088 ACGAC-TCTCGGCAATGGATATCTCGGCTCTCGCATCGATGAAGAGCGCAGCGAAATGCGATACGTGGTGT-GAATTGCAGAATCCCGCGAACC-ATCGAGTCTTTGAACGCAAGTTGCGCCCGAGGCCAAC-CGGCTGAGGGCACGTCTGCCTG---GGCGTCA-AGCATTATGTCACTCC

Cryptochilus_roseus_PK12187 ACGAC-TCTCGGCAATGGATATCTCGGCTCTCGCATCGATGAAGAGCGCAGCGAAATGCGATACGTGGTGT-GAATTGCAGAATCCCGCGAACC-ATCGAGTCTTTGAACGCAAGTTGCGCCCGAGGCCAAC-CGGCTGAGGGCACGTCTGCCTG---GGCGTCA-AGCATTATGTCACTCC

Cryptostylis_arachnites_PK12188 AGGAC-TCTCGGCAATGGATATCTTGGCTCTTGCATCGATGAAGAGCGCAGCGAAATGCGATATGTGGTGT-GAATTGCAGGATCCCGCGAACC-ATCGAGTTTTTGAACGCAAGTTGCGCCTGAGGCCGAC-TAGCCGAGGGCACGTCTGCCTG---GGCGTCA-TGCATTATGTCGCTCC

Cryptostylis_arachnites_SG027 AGGAC-TCTCGGCAATGGATATCTTGGCTCTTGCATCGATGAAGAGCGCAGCGAAATGCGATATGTGGTGT-GAATTGCAGGATCCCGCGAACC-ATCGAGTTTTTGAACGCAAGTTGCGCCTGAGGCCGAC-TAGCCGAGGGCACGTCTGCCTG---GGCGTCA-TGCATTATGTCGCTCC

Cryptostylis_arachnites_SG1142 AGGAC-TCTCGGCAATGGATATCTTGGCTCTTGCATCGATGAAGAGCGCAGCGAAATGCGATATGTGGTGT-GAATTGCAGGATCCCGCGAACC-ATCGAGTTTTTGAACGCAAGTTGCGCCTGAGGCCGAC-TAGCCGAGGGCACGTCTGCCTG---GGCGTCA-TGCATTATGTCGCTCC

Cryptostylis_arachnites_SG1380 AGGAC-TCTCGGCAATGGATATCTTGGCTCTTGCATCGATGAAGAGCGCAGCGAAATGCGATATGTGGTGT-GAATTGCAGGATCCCGCGAACC-ATCGAGTTTTTGAACGCAAGTTGCGCCTGAGGCCGAC-TAGCCGAGGGCACGTCTGCCTG---GGCGTCA-TGCATTATGTCGCTCC

Cymbidium_aloifolium_AF284695 ACGAC-TCTCGGCAATGGATATCTCGGCTCTCGCATCGATGAAGAGCGCAGCGAAATGCGATACGTGGTGC-GAATTGCAGAATCCCGCGAACC-ATCGAGTCTTTGAACGCAAGTTGCGCCCGAGGCCAGC-CGGCCGAGGGCACGTCCGCCTG---GGCGTCA-AGCATCGCGTCGCTCC

Cymbidium_aloifolium_JF729014 ACGAC-TCTCGGCAATGGATATCTCGGCTCTCGCATCGATGAAGAGCGCAGCGAAATGCGATACGTGGTGC-GAATTGCAGAATCCCGCGAACC-ATCGAGTCTTTGAACGCAAGTTGCGCCCGAGGCCAGC-CGGCCGAGGGCACGTCCGCCTG---GGCGTCA-AGCATCGCGTCGCTCC

Cymbidium_aloifolium_JN114485 ACGAC-TCTCGGCAATGGATATCTCGGCTCTCGCATCGATGAAGAGCGCAGCGAAATGCGATACGTGGTGC-GAATTGCAGAATCCCGCGAACC-ATCGAGTCTTTGAACGCAAGTTGCGCCCGAGGCCAGC-CGGCCGAGGGCACGTCCGCCTG---GGCGTCA-AGCATCGCGTCGCTCC

Cymbidium_aloifolium_JN114486 ACGAC-TCTCGGCAATGGATATCTCGGCTCTCGCATCGATGAAGAGCGCAGCGAAATGCGATACGTGGTGC-GAATTGCAGAATCCCGCGAACC-ATCGAGTCTTTGAACGCAAGTTGCGCCCGAGGCCAGC-CGGCCGAGGGCACGTCCGCCTG---GGCGTCA-AGCATCGCGTCGCTCC

Cymbidium_aloifolium_KFBG2049 ACGAC-TCTCGGCAATGGATATCTCGGCTCTCGCATCGATGAAGAGCGCAGCGAAATGCGATACGTGGTGC-GAATTGCAGAATCCCGCGAACC-ATCGAGTCTTTGAACGCAAGTTGCGCCCGAGGCCAGC-CGGCCGAGGGCACGTCCGCCTG---GGCGTCA-AGCATCGCGTCGCTCC

Cymbidium_aloifolium_KFBG2205 ACGAC-TCTCGGCAATGGATATCTCGGCTCTCGCATCGATGAAGAGCGCAGCGAAATGCGATACGTGGTGC-GAATTGCAGAATCCCGCGAACC-ATCGAGTCTTTGAACGCAAGTTGCGCCCGAGGCCAGC-CGGCCGAGGGCACGTCCGCCTG---GGCGTCA-AGCATCGCGTCGCTCC

Cymbidium_ensifolium_AF284716 ACGAC-TCTCGGCAATGGATATCTCGGCTCTCGCATCGATGAAGAGCGCAGCGAAATGCGATACGTGGTGC-GAATTGCAGAATCCCGCGAACC-ATCGAGTCTTTGAACGCAAGTTGCGCCCGAGGCCAGC-CGGCCGAGGGCACGTCCGCCTG---GGCGTCA-AGCATCGCGTCGCTCC

Cymbidium_ensifolium_AF284717 ACGAC-TCTCGGCAATGGATATCTCGGCTCTCGCATCGATGAAGAGCGCAGCGAAATGCGATACGTGGTGC-GAATTGCAGAATCCCGCGAACC-ATCGAGTCTTTGAACGCAAGTTGCGCCCGAGGCCAGC-CGGCCGAGGGCACGTCCGCCTG---GGCGTCA-AGCATCGCGTCGCTCC

Cymbidium_ensifolium_AF470496 ACGAC-TCTCGGCAATGGATATCTCGGCTCTCGCATCGATGAAGAGCGCAGCGAAATGCGATACGTGGTGC-GAATTGCAGAATCCCGCGAACC-ATCGAGTCTTTGAACGCAAGTTGCGCCCGAGGCCAGC-CGGCCGAGGGCACGTCCGCCTG---GGCGTCA-AGCATCGCGTCGCTCC

Cymbidium_ensifolium_AF470512 ACGAC-TCTCGGCAATGGATATCTCGGCTCTCGCATCGATGAAGAGCGCAGCGAAATGCGATACGTGGTGC-GAATTGCAGAATCCCGCGAACC-ATCGAGTCTTTGAACGCAAGTTGCGCCCGAGGCCAGC-CGGCCGAGGGCACGTCCGCCTG---GGCGTCA-AGCATCGCGTCGCTCC

Cymbidium_ensifolium_KJ597843 ACGAC-TCTCGGCAATGGATATCTCGGCTCTCGCATCGATGAAGAGCGCAGCGAAATGCGATACGTGGTGC-GAATTGCAGAATCCCGCGAACC-ATCGAGTCTTTGAACGCAAGTTGCGCCCGAGGCCAGC-CGGCCGAGGGCACGTCCGCCTG---GGCGTCA-AGCATCGCGTCGCTCC

Cymbidium_ensifolium_KJ597844 ACGAC-TCTCGGCAATGGATATCTCGGCTCTCGCATCGATGAAGAGCGCAGCGAAATGCGATACGTGGTGC-GAATTGCAGAATCCCGCGAACC-ATCGAGTCTTTGAACGCAAGTTGCGCCCGAGGCCAGC-CGGCCGAGGGCACGTCCGCCTG---GGCGTCA-AGCATCGCGTCGCTCC

Cymbidium_ensifolium_KJ597845 ACGAC-TCTCGGCAATGGATATCTCGGCTCTCGCATCGATGAAGAGCGCAGCGAAATGCGATACGTGGTGC-GAATTGCAGAATCCCGCGAACC-ATCGAGTCTTTGAACGCAAGTTGCGCCCGAGGCCAGC-CGGCCGAGGGCACGTCCGCCTG---GGCGTCA-AGCATCGCGTCGCTCC

Cymbidium_ensifolium_KJ597846 ACGAC-TCTCGGCAATGGATATCTCGGCTCTCGCATCGATGAAGAGCGCAGCGAAATGCGATACGTGGTGC-GAATTGCAGAATCCCGCGAACC-ATCGAGTCTTTGAACGCAAGTTGCGCCCGAGGCCAGC-CGGCCGAGGGCACGTCCGCCTG---GGCGTCA-AGCATCGCGTCGCTCC

Cymbidium_ensifolium_KT338675 ACGAC-TCTCGGCAATGGATATCTCGGCTCTCGCATCGATGAAGAGCGCAGCGAAATGCGATACGTGGTGC-GAATTGCAGAATCCCGCGAACC-ATCGAGTCTTTGAACGCAAGTTGCGCCCGAGGCCAGC-CGGCCGAGGGCACGTCCGCCTG---GGCGTCA-AGCATCGCGTCGCTCC

Cymbidium_ensifolium_PK12208 ACGAC-TCTCGGCAATGGATATCTCGGCTCTCGCATCGATGAAGAGCGCAGCGAAATGCGATACGTGGTGC-GAATTGCAGAATCCCGCGAACC-ATCGAGTCTTTGAACGCAAGTTGCGCCCGAGGCCAGC-CGGCCGAGGGCACGTCCGCCTG---GGCGTCA-AGCATCGCGTCGCTCC

Cymbidium_ensifolium_SG1214 ACGAC-TCTCGGCAATGGATATCTCGGCTCTCGCATCGATGAAGAGCGCAGCGAAATGCGATACGTGGTGC-GAATTGCAGAATCCCGCGAACC-ATCGAGTCTTTGAACGCAAGTTGCGCCCGAGGCCAGC-CGGCCGAGGGCACGTCCGCCTG---GGCGTCA-AGCATCGCGTCGCTCC

Cymbidium_ensifolium_SG1278 ACGAC-TCTCGGCAATGGATATCTCGGCTCTCGCATCGATGAAGAGCGCAGCGAAATGCGATACGTGGTGC-GAATTGCAGAATCCCGCGAACC-ATCGAGTCTTTGAACGCAAGTTGCGCCCGAGGCCAGC-CGGCCGAGGGCACGTCCGCCTG---GGCGTCA-AGCATCGCGTCGCTCC

Cymbidium_ensifolium_SG1285 ACGAC-TCTCGGCAATGGATATCTCGGCTCTCGCATCGATGAAGAGCGCAGCGAAATGCGATACGTGGTGC-GAATTGCAGAATCCCGCGAACC-ATCGAGTCTTTGAACGCAAGTTGCGCCCGAGGCCAGC-CGGCCGAGGGCACGTCCGCCTG---GGCGTCA-AGCATCGCGTCGCTCC

Cymbidium_kanran_AF284720 ACGAC-TCTCGGCAATGGATATCTCGGCTCTCGCATCGATGAAGAGCGCAGCGAAATGCGATACGTGGTGC-GAATTGCAGAATCCCGCGAACC-ATCGAGTCTTTGAACGCAAGTTGCGCCCGAGGCCAGC-CGGCCGAGGGCACGTCCGCCTG---GGCGTCA-AGCATCGCGTCGCTCC

Cymbidium_kanran_AF470495 ACGAC-TCTCGGCAATGGATATCTCGGCTCTCGCATCGATGAAGAGCGCAGCGAAATGCGATACGTGGTGC-GAATTGCAGAATCCCGCGAACC-ATCGAGTCTTTGAACGCAAGTTGCGCCCGAGGCCAGC-CGGCCGAGGGCACGTCCGCCTG---GGCGTCA-AGCATCGCGTCGCTCC

Cymbidium_kanran_JX202654 ACGAC-TCTCGGCAATGGATATCTCGGCTCTCGCATCGATGAAGAGCGCAGCGAAATGCGATACGTGGTGC-GAATTGCAGAATCCCGCGAACC-ATCGAGTCTTTGAACGCAAGTTGCGCCCGAGGCCAGC-CGGCCGAGGGCACGTCCGCCTG---GGCGTCA-AGCATCGCGTCGCTCC

Cymbidium_kanran_KF560534 ACGAC-TCTCGGCAATGGATATCTCGGCTCTCGCATCGATGAAGAGCGCAGCGAAATGCGATACGTGGTGC-GAATTGCAGAATCCCGCGAACC-ATCGAGTCTTTGAACGCAAGTTGCGCCCGAGGCCAGC-CGGCCGAGGGCACGTCCGCCTG---GGCGTCA-AGCATCGCGTCGCTCC

Cymbidium_kanran_KJ597836 ACGAC-TCTCGGCAATGGATATCTCGGCTCTCGCATCGATGAAGAGCGCAGCGAAATGCGATACGTGGTGC-GAATTGCAGAATCCCGCGAACC-ATCGAGTCTTTGAACGCAAGTTGCGCCCGAGGCCAGC-CGGCCGAGGGCACGTCCGCCTG---GGCGTCA-AGCATCGCGTCGCTCC

Cymbidium_kanran_KJ597837 ACGAC-TCTCGGCAATGGATATCTCGGCTCTCGCATCGATGAAGAGCGCAGCGAAATGCGATACGTGGTGC-GAATTGCAGAATCCCGCGAACC-ATCGAGTCTTTGAACGCAAGTTGCGCCCGAGGCCAGC-CGGCCGAGGGCACGTCCGCCTG---GGCGTCA-AGCATCGCGTCGCTCC

Cymbidium_kanran_KJ597838 ACGAC-TCTCGGCAATGGATATCTCGGCTCTCGCATCGATGAAGAGCGCAGCGAAATGCGATACGTGGTGC-GAATTGCAGAATCCCGCGAACC-ATCGAGTCTTTGAACGCAAGTTGCGCCCGAGGCCAGC-CGGCCGAGGGCACGTCCGCCTG---GGCGTCA-AGCATCGCGTCGCTCC

Cymbidium_kanran_KJ597839 ACGAC-TCTCGGCAATGGATATCTCGGCTCTCGCATCGATGAAGAGCGCAGCGAAATGCGATACGTGGTGC-GAATTGCAGAATCCCGCGAACC-ATCGAGTCTTTGAACGCAAGTTGCGCCCGAGGCCAGC-CGGCCGAGGGCACGTCCGCCTG---GGCGTCA-AGCATCGCGTCGCTCC

Cymbidium_kanran_KT338677 ACGAC-TCTCGGCAATGGATATCTCGGCTCTCGCATCGATGAAGAGCGCAGCGAAATGCGATACGTGGTGC-GAATTGCAGAATCCCGCGAACC-ATCGAGTCTTTGAACGCAAGTTGCGCCCGAGGCCAGC-CGGCCGAGGGCACGTCCGCCTG---GGCGTCA-AGCATCGCGTCGCTCC

Cymbidium_lancifolium_AF284724 ACGAC-TCTCGGCAATGGATATCTCGGCTCTCGCATCGATGAAGAGCGCAGCGAAATGCGATACGTGGTGC-GAATTGCAGAATCCCGCGAACC-ATCGAGTCTTTGAACGCAAGTTGCGCCCGAGGCCAGC-CGGCCGAGGGCACGTCCGCCTG---GGCGTCA-AGCGTCGCGTCGCTCC

Cymbidium_lancifolium_AF470520 ACGAC-TCTCGGCAATGGATATCTCGGCTCTCGCATCGATGAAGAGCGCAGCGAAATGCGATACGTGGTGC-GAATTGCAGAATCCCGCGAACC-ATCGAGTCTTTGAACGCAAGTTGCGCCCGAGGCCAGC-CGGCCGAGGGCACGTCCGCCTG---GGCGTCA-AGCGTCGCGTCGCTCC

Cymbidium_lancifolium_KFBG734 ACGAC-TCTCGGCAATGGATATCTCGGCTCTCGCATCGATGAAGAGCGCAGCGAAATGCGATACGTGGTGC-GAATTGCAGAATCCCGCGAACC-ATCGAGTCTTTGAACGCAAGTTGCGCCCGAGGCCAGC-CGGCCGAGGGCACGTCCGCCTG---GGCGTCA-AGCGTCGCGTCGCTCC

Cymbidium_lancifolium_KJ597851 ACGAC-TCTCGGCAATGGATATCTCGGCTCTCGCATCGATGAAGAGCGCAGCGAAATGCGATACGTGGTGC-GAATTGCAGAATCCCGCGAACC-ATCGAGTCTTTGAACGCAAGTTGCGCCCGAGGCCAGC-CGGCCGAGGGCACGTCCGCCTG---GGCGTCA-AGCGTCGCGTCGCTCC

Cymbidium_lancifolium_KJ597852 ACGAC-TCTCGGCAATGGATATCTCGGCTCTCGCATCGATGAAGAGCGCAGCGAAATGCGATACGTGGTGC-GAATTGCAGAATCCCGCGAACC-ATCGAGTCTTTGAACGCAAGTTGCGCCCGAGGCCAGC-CGGCCGAGGGCACGTCCGCCTG---GGCGTCA-AGCGTCGCGTCGCTCC

Cymbidium_lancifolium_KY966515 ACGAC-TCTCGGCAATGGATATCTCGGCTCTCGCATCGATGAAGAGCGCAGCGAAATGCGATACGTGGTGC-GAATTGCAGAATCCCGCGAACC-ATCGAGTCTTTGAACGCAAGTTGCGCCCGAGGCCAGC-CGGCCGAGGGCACGTCCGCCTG---GGCGTCM-AGCGTCGCGTCGCTCC

Cymbidium_lancifolium_PK12128 ACGAC-TCTCGGCAATGGATATCTCGGCTCTCGCATCGATGAAGAGCGCAGCGAAATGCGATACGTGGTGC-GAATTGCAGAATCCCGCGAACC-ATCGAGTCTTTGAACGCAAGTTGCGCCCGAGGCCAGC-CGGCCGAGGGCACGTCCGCCTG---GGCGTCA-AGCGTCGCGTCGCTCC

Cymbidium_lancifolium_SG1274 ACGAC-TCTCGGCAATGGATATCTCGGCTCTCGCATCGATGAAGAGCGCAGCGAAATGCGATACGTGGTGC-GAATTGCAGAATCCCGCGAACC-ATCGAGTCTTTGAACGCAAGTTGCGCCCGAGGCCAGC-CGGCCGAGGGCACGTCCGCCTG---GGCGTCA-AGCGTCGCGTCGCTCC

Cymbidium_sinense_AF284714 ACGAC-TCTCGGCAATGGATATCTCGGCTCTCGCATCGATGAAGAGCGCAGCGAAATGCGATACGTGGTGC-GAATTGCAGAATCCCGCGAACC-ATCGAGTCTTTGAACGCAAGTTGCGCCCGAGGCCAGC-CGGCCGAGGGCACGTCCGCCTG---GGCGTCA-AGCATCGCGTCGCTCC

Cymbidium_sinense_AF284715 ACGAC-TCTCGGCAATGGATATCTCGGCTCTCGCATCGATGAAGAGCGCAGCGAAATGCGATACGTGGTGC-GAATTGCAGAATCCCGCGAACC-ATCGAGTCTTTGAACGCAAGTTGCGCCCGAGGCCAGC-CGGCCGAGGGCACGTCCGCCTG---GGCGTCA-AGCATCGCGTCGCTCC

Cymbidium_sinense_KJ597847 ACGAC-TCTCGGCAATGGATATCTCGGCTCTCGCATCGATGAAGAGCGCAGCGAAATGCGATACGTGGTGC-GAATTGCAGAATCCCGCGAACC-ATCGAGTCTTTGAACGCAAGTTGCGCCCGAGGCCAGC-CGGCCGAGGGCACGTCCGCCTG---GGCGTCA-AGCATCGCGTCGCTCC

Cymbidium_sinense_KJ597848 ACGAC-TCTCGGCAATGGATATCTCGGCTCTCGCATCGATGAAGAGCGCAGCGAAATGCGATACGTGGTGC-GAATTGCAGAATCCCGCGAACC-ATCGAGTCTTTGAACGCAAGTTGCGCCCGAGGCCAGC-CGGCCGAGGGCACGTCCGCCTG---GGCGTCA-AGCATCGCGTCGCTCC

Cymbidium_sinense_KJ597849 ACGAC-TCTCGGCAATGGATATCTCGGCTCTCGCATCGATGAAGAGCGCAGCGAAATGCGATACGTGGTGC-GAATTGCAGAATCCCGCGAACC-ATCGAGTCTTTGAACGCAAGTTGCGCCCGAGGCCAGC-CGGCCGAGGGCACGTCCGCCTG---GGCGTCA-AGCATCGCGTCGCTCC

Cymbidium_sinense_KJ597850 ACGAC-TCTCGGCAATGGATATCTCGGCTCTCGCATCGATGAAGAGCGCAGCGAAATGCGATACGTGGTGC-GAATTGCAGAATCCCGCGAACC-ATCGAGTCTTTGAACGCAAGTTGCGCCCGAGGCCAGC-CGGCCGAGGGCACGTCCGCCTG---GGCGTCA-AGCATCGCGTCGCTCC

Cymbidium_sinense_SG1213 ACGAC-TCTCGGCAATGGATATCTCGGCTCTCGCATCGATGAAGAGCGCAGCGAAATGCGATACGTGGTGC-GAATTGCAGAATCCCGCGAACC-ATCGAGTCTTTGAACGCAAGTTGCGCCCGAGGCCAGC-CGGCCGAGGGCACGTCCGCCTG---GGCGTCA-AGCATCGCGTCGCTCC

Cymbidium_sinense_SG1218 ACGAC-TCTCGGCAATGGATATCTCGGCTCTCGCATCGATGAAGAGCGCAGCGAAATGCGATACGTGGTGC-GAATTGCAGAATCCCGCGAACC-ATCGAGTCTTTGAACGCAAGTTGCGCCCGAGGCCAGC-CGGCCGAGGGCACGTCCGCCTG---GGCGTCA-AGCATCGCGTCGCTCC

Cymbidium_sinense_SG1341 ACGAC-TCTCGGCAATGGATATCTCGGCTCTCGCATCGATGAAGAGCGCAGCGAAATGCGATACGTGGTGC-GAATTGCAGAATCCCGCGAACC-ATCGAGTCTTTGAACGCAAGTTGCGCCCGAGGCCAGC-CGGCCGAGGGCACGTCCGCCTG---GGCGTCA-AGCATCGCGTCGCTCC

Cymbidium_sinense_SG1342 ACGAC-TCTCGGCAATGGATATCTCGGCTCTCGCATCGATGAAGAGCGCAGCGAAATGCGATACGTGGTGC-GAATTGCAGAATCCCGCGAACC-ATCGAGTCTTTGAACGCAAGTTGCGCCCGAGGCCAGC-CGGCCGAGGGCACGTCCGCCTG---GGCGTCA-AGCATCGCGTCGCTCC

Dendrobium_aduncum_AB593484 ACGAC-TCTCGGCAATGGATATCTCGGCTCTCGCATCGATGAAGAGCGCAGCGAAATGCGATATGTGGTGC-GAATTGCAGAATCCCGCGAACC-ATCGAGTCTTTGAACGCAAGTTGCGCCCGAGGCCAAC-CGGCTAAGGGCACGTCCGCCTG---GGCGTCA-AGCATTTTATCGCTCC

Dendrobium_aduncum_GU339110 ACGAC-TCTCGGCAATGGATATCTCGGCTCTCGCATCGATGAAGAGCGCAGTGAAATGCGATATGTGGTGC-GAATTGCAGAATCCCGCGAACC-ATCGAGTCTTTGAACGCAAGTTGCGCCCGAGGCCAAC-CGGCTAAGGGCACGTCCGCCTG---GGCGTCA-AGCATTTTATCGCTCC

Dendrobium_aduncum_HM590372 ACGAC-TCTCGGCAATGGATATCTCGGCTCTCGCATCGATGAAGAGCGCAGCGAAATGCGATATGTGGTGC-GAATTGCAGAATCCCGCGAACC-ATCGAGTCTTTGAACGCAAGTTGCGCCTGAGGCCAAC-CGGCTGAGGGCACGTCCGCCTG---GGCGTCA-AGCATTTTATCGCTCC

Dendrobium_aduncum_HQ114250 ATGAC-TCTCGGCAATGGATATCTCGGCTCTCGCATCGATGAAGAGCGCAGCGAAATGCGATATGTGGTGC-GAATTGCAGAATCCCGCGAACC-ATCGAGTCTTTGAACGCAAGTTGCGCCCGAGGCCAAC-CGGCCAAGGGCACGTCCGCCTG---GGCGTCA-GTCATTTTATCGCTCT

Dendrobium_aduncum_JF713083 ACGAC-TCTCGGCAATGGATATCTCGGCTCTCGCATCGATGAAGAGCGCAGCGAAATGCGATATGTGGTGC-GAATTGCAGAATCCCGCGAACC-ATCGAGTCTTTGAACGCAAGTTGCGCCCGAGGCCAAC-CGGCTAAGGGCACGTCCGCCTG---GGCGTCA-AGCATTTTATCGCTCC

Dendrobium_aduncum_JN388580 ACGAC-TCTCGGCAATGGATATCTCGGCTCTCGCATCGATGAAGAGCGCAGCGAAATGCGATATGTGGTGC-GAATTGCAGAATCCCGCGAACC-ATCGAGTCTTTGAACGCAAGTTGCGCCCGAGGCCAAC-CGGCTAAGGGCACGTCCGCCTG---GGCGTCA-AGCATTTTATCGCTCC

Dendrobium_aduncum_KC346887 ACGAC-TCTCGGCAATGGATATCTCGGCTCTCGCATCGATGAAGAGCGCAGCGAAATGCGATATGTGGTGC-GAATTGCAGAATCCCGCGAACC-ATCGAGTCTTTGAACGCAAGTTGCGCCCGAGGCCAAC-CGGCTAAGGGCACGTCCGCCTG---GGCGTCA-AGCATTTTATCGCTCC

Dendrobium_aduncum_KC568295 ACGAC-TCTCGGCAATGGATATCTCGGCTCTCGCATCGATGAAGAGCGCAGCGAAATGCGATATGTGGTGC-GAATTGCAGAATCCCGCGAACC-ATCGAGTCTTTGAACGCAAGTTGCGCCCGAGGCCAAC-CGGCTAAGGGCACGTCCGCCTG---GGCGTCA-AGCATTTTATCGCTCC

Dendrobium_aduncum_KF143428 ACGAC-TCTCGGCAATGGATATCTCGGCTCTCGCATCGATGAAGAGCGCAGCGAAATGCGATATGTGGTGC-GAATTGCAGAATCCCGCGAACC-ATCGAGTCTTTGAACGCAAGTTGCGCCCGAGGCCAAC-CGGCTAAGGGCACGTCCGCCTG---GGCGTCA-AGCATTTTATCGCTCC

Dendrobium_aduncum_KFBG8766 ACGAC-TCTCGGCAATGGATATCTCGGCTCTCGCATCGATGAAGAGCGCAGCGAAATGCGATATGTGGTGC-GAATTGCAGAATCCCGCGAACC-ATCGAGTCTTTGAACGCAAGTTGCGCCCGAGGCCAAC-CGGCTAAGGGCACGTCCGCCTG---GGCGTCA-AGCATTTTATCGCTCC

Dendrobium_aduncum_KJ210409 ACGAC-TCTCGGCAATGGATATCTCGGCTCTCGCATCGATGAAGAGCGCAGCGAAATGCGATATGTGGTGC-GAATTGCAGAATCCCGCGAACC-ATCGAGTCTTTGAACGCAAGTTGCGCCCGAGGCCAAC-CGGCTAAGGGCACGTCCGCCTG---GGCGTCA-AGCATTTTATCGCTCC

Dendrobium_aduncum_KJ210410 ACGAC-TCTCGGCAATGGATATCTCGGCTCTCGCATCGATGAAGAGCGCAGCGAAATGCGATATGTGGTGC-GAATTGCAGAATCCCGCGAACC-ATCGAGTCTTTGAACGCAAGTTGCGCCCGAGGCCAAC-CGGCTAAGGGCACGTCCGCCTG---GGCGTCA-AGCATTTTATCGCTCC

Dendrobium_aduncum_KJ210411 ACGAC-TCTCGGCAATGGATATCTCGGCTCTCGCATCGATGAAGAGCGCAGCGAAATGCGATATGTGGTGC-GAATTGCAGAATCCCGCGAACC-ATCGAGTCTTTGAACGCAAGTTGCGCCCGAGGCCAAC-CGGCTAAGGGCACGTCCGCCTG---GGCGTCA-AGCATTTTATCGCTCC

Dendrobium_aduncum_KJ210412 ACGAC-TCTCGGCAATGGATATCTCGGCTCTCGCATCGATGAAGAGCGCAGCGAAATGCGATATGTGGTGC-GAATTGCAGAATCCCGCGAACC-ATCGAGTCTTTGAACGCAAGTTGCGCCCGAGGCCAAC-CGGCTAAGGGCACGTCCGCCTG---GGCGTCA-AGCATTTTATCGCTCC

Dendrobium_aduncum_KJ672619 ACGAC-TCTCGGCAATGGATATCTCGGCTCTCGCATCGATGAAGAGCGCAGCGAAATGCGATATGTGGTGC-GAATTGCAGAATCCCGCGAACC-ATCGAGTCTTTGAACGCAAGTTGCGCCCGAGGCCAAC-CGGCTAAGGGCACGTCCGCCTG---GGCGTCA-AGCATTTTATCGCTCC

Dendrobium_aduncum_KP159298 ACGAC-TCTCGGCAATGGATATCTCGGCTCTCGCATCGATGAAGAGCGCAGCGAAATGCGATATGTGGTGC-GAATTGCAGAATCCCGCGAACC-ATCGAGTCTTTGAACGCAAGTTGCGCCTGAGGCCAAC-CGGCTGAGGGCACGTCCGCCTG---GGCGTCA-AGCATTTTATCGCTCC

Dendrobium_aduncum_KR075042 ACGAC-TCTCGGCAATGGATATCTCGGCTCTCGCATCGATGAAGAGCGCAGTGAAATGCGATATGTGGTGC-GAATTGCAGAATCCCGCGAACC-ATCGAGTCTTTGAACGCAAGTTGCGCCCGAGGCCAAC-CGGCTAAGGGCACGTCCGCCTG---GGCGTCA-AGCATTTTATCGCTCC

Dendrobium_aduncum_KX600499 ACGAC-TCTCGGCAATGGATATCTCGGCTCTCGCATCGATGAAGAGCGCAGCGAAATGCGATATGTGGTGC-GAATTGCAGAATCCCGCGAACC-ATCGAGTCTTTGAACGCAAGTTGCGCCCGAGGCCAAC-CGGCTAAGGGCACGTCCGCCTG---GGCGTCA-AGCATTTTATCGCTCC

Dendrobium_anosmum_AB593499 ACGAC-TCTCGGCAATGGATATCTCGGCTCTCGCATCGATGAAGAGCGCAGCGAAATGCGATATGTGGTGC-GAATTGCAGAATCCCGCGAACC-ATCGAGTCTTTGAACGCAAGTTGCGCCCGAGGCCAAT-CGGCCAAGGGCACGTCCGCCTG---GGCGTCA-GGCATTTTGTCGCTTC

Dendrobium_anosmum_AB972339 ACGAC-TCTCGGCAATGGATATCTCGGCTCTCGCATCGATGAAGAGCGCAGCGAAATGCGATATATGGTGC-GAATTGCAGAATCCCGCGAACC-ATCGAGTCTTTGAACGCAAGTTGCGCCCGAGGCCAAT-CGGCCAAGGGCACGTCCGCCTG---GGCGTCA-GGCATTTTGTCGCTTC

Dendrobium_anosmum_EU477499 ACGAC-TCTCGGCAATGGATATCTCGGCTCTCGCATCGATGAAGAGCGCAGCGAAATGCGATATATGGTGC-GAATTGCAGAATCCCGCGAACC-ATCGAGTCTTTGAACGCAAGTTGCGCCCGAGGCCAAT-CGGCCAAGGGCACGTCCGCCTG---GGCGTCA-GGCATTTTGTCGCTTC

Dendrobium_anosmum_JN388570 ACGAC-TCTCGGCAATGGATATCTCGGCTCTCGCATCGATGAAGAGCGCAGCGAAATGCGATATGTGGTGC-GAATTGCAGAATCCCGCGAACC-ATCGAGTCTTTGAACGCAAGTTGCGCCCGAGGCCAAT-CGGCCAAGGGCACGTCCGCCTG---GGCGTCA-GGCATTTTGTCGCTTC

Dendrobium_anosmum_KJ672650 ACGAC-TCTCGGCAATGGATATCTCGGCTCTCGCATCGATGAAGAGCGCAGCGAAATGCGATATGTGGTGC-GAATTGCAGAATCCCGCGAACC-ATCGAGTCTTTGAACGCAAGTTGCGCCCGAGGCCAAT-CGGCCAAGGGCACGTCCGCCTG---GGCGTCA-GGCATTTTGTCGCTTC

Dendrobium_anosmum_KJ944630 ACGAC-TCTCGGCAATGGATATCTCGGCTCTCGCATCGATGAAGAGCGCAGCGAAATGCGATATATGGTGC-GAATTGCAGAATCCCGCGAACC-ATCGAGTCTTTGAACGCAAGTTGCGCCCGAGGCCAAT-CGGCCAAGGGCACGTCCGCCTG---GGCGTCA-GGCATTTTGTCGCTTC

Dendrobium_anosmum_KP743542 ACGAC-TCTCGGCAATGGATATCTCGGCTCTCGCATCGATGAAGAGCGCAGCGAAATGCGATATGTGGTGC-GAATTGCAGAATCCCGCGAACC-ATCGAGTCTTTGAACGCAAGTTGCGCCCGAGGCCAAT-CGGCCAAGGGCACGTCCGCCTG---GGCGTCA-GGCATTTTGTCGCTTC

Dendrobium_anosmum_KP743544 ACGAC-TCTCGGCAATGGATATCTCGGCTCTCGCATCGATGAAGAGCGCAGCGAAATGCGATATATGGTGC-GAATTGCAGAATCCCGCGAACC-ATCGAGTCTTTGAACGCAAGTTGCGCCCGAGGCCAAT-CGGCCAAGGGCACGTCCGCCTG---GGCGTCA-GGCATTTTGTCGCTTC

Dendrobium_anosmum_KY966516 ACGAC-TCTCGGCAATGGATATCTCGGCTCTCGCATCGATGAAGAGCGCAGCGAAATGCGATATGTGGTGC-GAATTGCAGAATCCCGCGAACC-ATCGAGTCTTTGAACGCAAGTTGCGCCCGAGGCCAAT-CGGCCAAGGGCACGTCCGCCTG---GGCGTCA-GGCATTTTGTCGCTTC

Dendrobium_cf_mimicum_PK12237E ACGAC-TCTCGGCAATGGATATCTCGGCTCTTGCATCGATGAAGAGCGCAGCGAAATGCGATACGTGGTGC-GAATTGCAGAATCCCGCGAACC-ATCGAGTCTTTGAACGCAAGTTGCGCCCGAGGCCAAC-CGGCCAAGGGCACGTCCGCCTG---GGCGTCAA-GCGTTGCGTCGCTCC

Dendrobium_cf_mimicum_PK12237J ACGAC-TCTCGGCAATGGATATCTCGGCTCTTGCATCGATGAAGAGCGCAGCGAAATGCGATACGTGGTGC-GAATTGCAGAATCCCGCGAACC-ATCGAGTCTTTGAACGCAAGTTGCGCCCGAGGCCAAC-CGGCCAAGGGCACGTCCGCCTG---GGCGTCAA-GCGTTGCGTCGCTCC

Dendrobium_crumenatum_AB593537 AGGAC-TCTCGACAATGGATATCTCGGCTCTTGCATCGATGAAGAGCGCAGCGAAATGCGATACGTGGTGC-GAATTGCAGAATCCCGCGAACC-ATCGAGTCTTTGAACGCAAGTTGCGCCCGAGGCCAAT-CGGCCAAGGGCACGTCTGCCTG---GGCGTCA-AGCATTATGTCACTCC

Dendrobium_crumenatum_AB972336 AGGAC-TCTCGACAATGGATATCTCGGCTCTTGCATCGATGAAGAGCGCAGCGAAATGCGATACGTGGTGC-GAATTGCAGAATCCCGCGAACC-ATCGAGTCTTTGAACGCAAGTTGCGCCCGAGGCCAAT-CGGCCAAGGGCACGTCTGCCTG---GGCGTCA-AGCATTATGTCACTCC

Dendrobium_crumenatum_AF521608 AGGAC-TCTCGACAATGGATATCTCGGCTCTTGCATCGATGAAGAGCGCAGCGAAATGCGATACGTGGTGC-GAATTGCAGAATCCCGCGAACC-ATCGAGTCTTTGAACGCAAGTTGCGCCCGAGGCCAAT-CGGCCAAGGGCACGTCTGCCTG---GGCGTCA-AGCATTATGTCACTCC

Dendrobium_crumenatum_AY239963 AGGAC-TCTCGACAATGGATATCTCGGCTCTTGCATCGATGAAGAGCGCAGCGAAATGCGATACGTGGTGC-GAATTGCAGAATCCCGCGAACC-ATCGAGTCTTTGAACGCAAGTTGCGCCCGAGGCCAAT-CGGCCAAGGGCACGTCTGCCTG---GGCGTCA-AGCATTATGTCACTCC

Dendrobium_crumenatum_AY273708 AGGAC-TCTCGACAATGAATATCTCGGCTCTTGCATCGATGAAGAGCGCAGCGAAATGCGATACGTGGTGC-GAATTGCAGAATCCCGCGAACC-ATCGAGTCTTTGAACGCAAGTTGCGCCCGAGGCCAAT-CGGCCAAGGGCACGTTTGCCTG---GGCGTCA-AGCATTATGTCACTCC

Dendrobium_crumenatum_EU840700 AGGAC-TCTCGACAATGGATATCTCGGCTCTTGCATCGATGAAGAGCGCAGCGAAATGCGATACATGGTGC-GAATTGCAGAATCCCGCGAACC-ATCGAGTCTTTGAACGCAAGTTGCGCCCCAGGCCAAT-CGGCCAAGGGCACGTTTGCCTG---GGCGTCA-AGCATTATGTCACTCC

Dendrobium_crumenatum_HM054625 AGGAC-TCTCGACAATGGATATCTCGGCTCTTGCATCGATGAAGAGCGCAGCGAAATGCGATACATGGTGC-GAATTGCAGAATCCCGCGAACC-ATCGAGTCTTTGAACGCAAGTTGCGCCCGAGGCCAAT-CGGCCAAGGGCACGTCTGCCTG---GGCGTCA-AGCATTATGTCACTCC

Dendrobium_crumenatum_HM590370 AGGAC-TCTCGACAATGGATATCTCGGCTCTTGCATCGATGAAGAGCGCAGCGAAATGCGATACATGGTGC-GAATTGCAGAATCCCGCGAACC-ATCGAGTCTTTGAACGCAAGTTGCGCCCGAGGCCAAT-CGGCCAAGGGCACGTCTGCCTG---GGCGTCA-AGCATTATGTCACTCC

Dendrobium_crumenatum_JF713095 AGGAC-TCTCGACAATGGATATCTCGGCTCTTGCATCGATGAAGAGCGCAGCGAAATGCGATACATGGTGC-GAATTGCAGAATCCCGCGAACC-ATCGAGTCTTTGAACGCAAGTTGCGCCCGAGGCCAAT-CGGCCAAGGGCACGTCTGCCTG---GGCGTCA-AGCATTATGTCACTCC

Dendrobium_crumenatum_JF713096 AGGAC-TCTCGACAATGGATATCTCGGCTCTTGCATCGATGAAGAGCGCAGCGAAATGCGATACATGGTGC-GAATTGCAGAATCCCGCGAACC-ATCGAGTCTTTGAACGCAAGTTGCGCCCGAGGCCAAT-CGGCCAAGGGCACGTCTGCCTG---GGCGTCA-AGCATTATGTCACTCC

Dendrobium_crumenatum_JN388587 AGGAC-TCTCGACAATGGATATCTCGGCTCTTGCATCGATGAAGAGCGCAGCGAAATGCGATACATGGTGC-GAATTGCAGAATCCCGCGAACC-ATCGAGTCTTTGAACGCAAGTTGCGCCCGAGGCCAAT-CGGCCAAGGGCACGTCTGCCTG---GGCGTCA-AGCATTATGTCACTCC

Dendrobium_crumenatum_KC507780 AGGAC-TCTCGACAATGGATATCTCGGCTCTTGCATCGATGAAGAGCGCAGCGAAATGCGATACGTGGTGC-GAATTGCAGAATCCCGCGAACC-ATCGAGTCTTTGAACGCAAGTTGCGCCCGAGGCCAAT-CGGCCAAGGGCACGTCTGCCTG---GGCG----AGC---ATGTCACTCC

Dendrobium_crumenatum_KJ672661 AGGAC-TCTCGACAATGGATATCTCGGCTCTTGCATCGATGAAGAGCGCAGCGAAATGCGATACATGGTGC-GAATTGCAGAATCCCGCGAACC-ATCGAGTCTTTGAACGCAAGTTGCGCCCCAGGCCAAT-CGGCCAAGGGCACGTTTGCCTG---GGCGTCA-AGCATTATGTCACTCC

Dendrobium_linawianum_AB593599 ACGAC-TCTCGGCAATGGATATCTTGGCTCTCGCATCGATGAAGAGCGCAGCGAAATGCGATATGTGGTGC-GAATTGCAGAATCCCGCGAACC-ATCGAGTCTTTGAACGCAAGTTGCGCCTGAGGCCAAC-CGGCTGAGGGCACGTCCGCCTG---GGCGTCA-AGCATTTTATCGCTCC

Dendrobium_linawianum_AF521613 ACGAC-TCTCGGCAATGGATATCTTGGCTCTCGCATCGATGAAGAGCGCAGCGAAATGCGATATGTGGTGC-GAATTGCAGAATCCCGCGAACC-ATCGAGTCTTTGAACGCAAGTTGCGCCTGAGGCCAAC-CGGCTGAGGGCACGTCCGCCTG---GGCGTCA-AGCATTTTATCGCTCC

Dendrobium_linawianum_EU003115 ACGAC-TCTCGGCAATGGATATCTTGGCTCTCGCATCGATGAAGAGCGCAGCGAAATGCGATATGTGGTGC-GAATTGCAGAATCCCGCGAACC-ATCGAGTCTTTGAACGCAAGTTGCGCCTGAGGCCAAC-CGGCTGAGGGCACGTCCGCCTG---GGCGTCA-AGCATTTTATCGCTCC

Dendrobium_linawianum_EU003117 ACGAC-TCTCGGCAATGGATATCTTGGCTCTCGCATCGATGAAGAGCGCAGCGAAATGCGATATGTGGTGC-GAATTGCAGAATCCCGCGAACC-ATCGAGTCTTTGAACGCAAGTTGCGCCTGAGGCCAAC-CGGCTGAGGGCACGTCCGCCTG---GGCGTCA-AGCATTTTATCGCTCC

Dendrobium_linawianum_HM590371 ACGAC-TCTCGGCAATGGATATCTTGGCTCTCGCATCGATGAAGAGCGCAGCGAAATGCGATATGTGGTGC-GAATTGCAGAATCCCGCGAACC-ATCGAGTCTTTGAACGCAAGTTGCGCCTGAGGCCAAC-CGGCTGAGGGCACGTCCGCCTG---GGCGTCA-AGCATTTTATCGCTCC

Dendrobium_linawianum_JN388573 ACGAC-TCTCGGCAATGGATATCTCGGCTCTCGCATCGATGAAGAGCGCAGCGAAATGCGATATGTGGTGC-GAATTGCAGAATCCCGCGAACC-ATCGAGTCTTTGAACGCAAGTTGCGCCTGAGGCCAAC-CGGCTGAGGGCACGTCCGCCTG---GGCGTCA-AGCATTTTATCGCTCC

Dendrobium_linawianum_KJ672629 ACGAC-TCTCGGCAATGGATATCTTGGCTCTCGCATCGATGAAGAGCGCAGCGAAATGCGATATGTGGTGC-GAATTGCAGAATCCCGCGAACC-ATCGAGTCTTTGAACGCAAGTTGCGCCTGAGGCCAAC-CGGCTGAGGGCACGTCCGCCTG---GGCGTCA-AGCATTTTATCGCTCC

Dendrobium_linawianum_KP159299 ACGAC-TCTCGGCAATGGATATCTTGGCTCTCGCATCGATGAAGAGCGCAGCGAAATGCGATATGTGGTGC-GAATTGCAGAATCCCGCGAACC-ATCGAGTCTTTGAACGCAAGTTGCGCCTGAGGCCAAC-CGGCTGAGGGCACGTCCGCCTG---GGCGTCA-AGCATTTTATCGCTCC

Dendrobium_linawianum_KT779776 ACGAC-TCTCGGCAATGGATATCTCGGCTCTCGCATCGATGAAGAGCGCAGCGAAATGCGATATGTGGTGC-GAATTGCAGAATCCCGCGAACC-ATCGAGTCTTTGAACGCAAGTTGCGCCTGAGGCCAAC-CGGCTGAGGGCACGTCCGCCTG---GGCGTCA-AGCATTTTATCGCTCC

Dendrobium_linawianum_KY966557 ACGAC-TCTCGGCAATGGATATCTTGGCTCTCGCATCGATGAAGAGCGCAGCGAAATGCGATATGTGGTGC-GAATTGCAGAATCCCGCGAACC-ATCGAGTCTTTGAACGCAAGTTGCGCCTGAGGCCAAC-CGGCTGAGGGCACGTCCGCCTG---GGCGTCA-AGCATTTTATCGCTCC

Dendrobium_linawianum_SG1347 ACGAC-TCTCGGCAATGGATATCTTGGCTCTCGCATCGATGAAGAGCGCAGCGAAATGCGATATGTGGTGC-GAATTGCAGAATCCCGCGAACC-ATCGAGTCTTTGAACGCAAGTTGCGCCTGAGGCCAAC-CGGCTGAGGGCACGTCCGCCTG---GGCGTCA-AGCATTTTATCGCTCC

Dendrobium_lindleyi_AB593600 ACGAC-TCTCGGCAATGGATATCTCGGCTCTCGCATCGATGAAGAGCGCAGCGAAATGCGATACGTGGTGC-GAATTGCAGAATCCCGCGAACC-ATCGAGTCTTTGAACGCAAGTTGCGCCCGAGGCCAAT-CGGCCAAGGGCACGTTCGCCTG---GGCGTCA-AGCATTACGTTGCTCC

Dendrobium_lindleyi_AB972341 ACGAC-TCTCGGCAATGGATATCTCGGCTCTCGCATCGATGAAGAGCGCAGCGAAATGCGATACGTGGTGC-GAATTGCAGAATCCCGCGAACC-ATCGAGTCTTTGAACGCAAGTTGCGCCCGAGGCCAAT-CGGCCAAGGGCACGTTCGCCTG---GGCGTCA-AGCATTACGTTGCTCC

Dendrobium_lindleyi_DQ058784 ACGAC-TCTCGGCAATGGATATCTCGGCTCTCGCATCGATGAAGAGCGCAGCGAAATGCGATACGTGGTGC-GAATTGCAGAATCCCGCGAACC-ATCGAGTCTTTGAACGCAAGTTGCGCCCGAGGCCAAT-CGGCCAAGGGCACGTTCGCCTG---GGCGTCA-AGCATTATGACGCTCC

Dendrobium_lindleyi_GU339114 ACGAC-TCTCGGCAATGGATATCTCGGCTCTCGCATCGATGAAGAGCGCAGCGAAATGTGATACGTGGTGC-GAATTGCAGAATCCCGCGAACCCATCGAGTCTTTGAACGCAAGTTGCGCCCGAGGCCAAT-CGGCCAAGGGCACGTTCGCCTG---GGCGTCA-AGCATTATGACGCTCC

Dendrobium_lindleyi_HM054672 ACGAC-TCTCGGCAATGGATATCTCGGCTCTTGCATCGATGAAGAGCGCAGCGAAATGCGATACGTGGTGC-GAATTGCAGAATCCCGCGAACC-ATCGAGTCTTTGAACGCAAGTTGCGCCCGAGGCCAAT-CGGCCAAGGGCACGTTCGCCTG---GGCGTCA-AGCATTATGTTGCTCC

Dendrobium_lindleyi_HM054673 ACGAC-TCTCGGCAATGGATATCTCGGCTCTTGCATCGATGAAGAGCGCAGCGAAATGCGATACGTGGTGC-GAATTGCAGAATCCCGCGAACC-ATCGAGTCTTTGAACGCAAGTTGCGCCCGAGGCCAAT-CGGCCAAGGGCACGTTCGCCTG---GGCGTCA-AGCATTATGTTGCTCC

Dendrobium_lindleyi_JF713110 ACGAC-TCTCGGCAATGGATATCTCGGCTCTTGCATCGATGAAGAGCGCAGCGAAATGCGATACGTGGTGC-GAATTGCAGAATCCCGCGAACC-ATCGAGTCTTTGAACGCAAGTTGCGCCCGAGGCCAAT-CGGCCAAGGGCACGTTCGCCTG---GGCGTCA-AGCATTATGTTGCTCC

Dendrobium_lindleyi_JN388568 ACGAC-TCTCGGCAATGGATATCTCGGCTCTCGCATCGATGAAGAGCGCAGCGAAATGCGATACGTGGTGC-GAATTGCAGAATCCCGCGAACC-ATCGAGTCTTTGAACGCAAGTTGCGCCCGAGGCCAAT-CGGCCAAGGGCACGTTCGCCTG---GGCGTCA-AGCATTATGACGCTCC

Dendrobium_lindleyi_KC568301 ACGAC-TCTCGGCAATGGATATCTCGGCTCTCGCATCGATGAAGAGCGCAGCGAAATGCGATACGTGGTGC-GAATTGCAAAATCCCGCGAACC-ATCGAGTCTTTGAACGCAAGTTGCGCCCGAGGCCAAT-CGGCCAAGGGCACGTTCGCCTG---GGCGTCA-AGCATTATGACGCTCC

Dendrobium_lindleyi_KFBG203 ACGAC-TCTCGGCAATGGATATCTCGGCTCTCGCATCGATGAAGAGCGCAGCGAAATGCGATACGTGGTGC-GAATTGCAGAATCCCGCGAACC-ATCGAGTCTTTGAACGCAAGTTGCGCCCGAGGCCAAT-CGGCCAAGGGCACGTTCGCCTG---GGCGTCA-AGCATTATGACGCTCC

Dendrobium_lindleyi_KJ210461 ACGAC-TCTCGGCAATGGATATCTCGGCTCTCGCATCGATGAAGAGCGCAGCGAAATGCGATACGTGGTGC-GAATTGCAGAATCCCGCGAACC-ATCGAGTCTTTGAACGCAAGTTGCGCCCGAGGCCAAT-CGGCCAAGGGCACGTTCGCCTG---GGCGTCA-AGCATTATGACGCTCC

Dendrobium_lindleyi_KJ672682 ACGAC-TCTCGGCAATGGATATCTCGGCTCTCGCATCGATGAAGAGCGCAGCGAAATGCGATACGTGGTGC-GAATTGCAGAATCCCGCGAACC-ATCGAGTCTTTGAACGCAAGTTGCGCCCGAGGCCAAT-CGGCCAAGGGCACGTTCGCCTG---GGCGTCA-AGCATTATGACGCTCC

Dendrobium_lindleyi_KX522640 ACGAC-TCTCGGCAATGGATATCTCGGCTCTTGCATCGATGAAGAGCGCAGCGAAATGCGATACGTGGTGC-GAATTGCAGAATCCCGCGAACC-ATCGAGTCTTTGAACGCAAGTTGCGCCCGAGGCCAAT-CGGCCAAGGGCACGTTCGCCTG---GGCGTCA-AGCATTATGTTGCTCC

Dendrobium_lindleyi_KY966558 ACGAC-TCTCGGCAATGGATATCTCGGCTCTCGCATCGATGAAGAGCGCAGCGAAATGCGATACGTGGTGC-GAATTGCAGAATCCCGCGAACC-ATCGAGTCTTTGAACGCAAGTTGCGCCCGAGGCCAAT-CGGCCAAGGGCACGTTCGCCTG---GGCGTCA-AGCATTATGACGCTCC

Dendrobium_lindleyi_KY966559 ACGAC-TCTCGGCAATGGATATCTCGGCTCTCGCATCGATGAAGAGCGCAGCGAAATGCGATACGTGGTGC-GAATTGCAGAATCCCGCGAACC-ATCGAGTCTTTGAACGCAAGTTGCGCCCGAGGCCAAT-CGGCCAAGGGCACGTTCGCCTG---GGCGTCA-AGCATTATGACGCTCC

Dendrobium_lindleyi_KY966560 ACGAC-TCTCGGCAATGGATATCTCGGCTCTCGCATCGATGAAGAGCGCAGCGAAATGCGATACGTGGTGC-GAATTGCAGAATCCCGCGAACC-ATCGAGTCTTTGAACGCAAGTTGCGCCCGAGGCCAAT-CGGCCAAGGGCACGTTCGCCTG---GGCGTCA-AGCATTACGTTGCTCC

Dendrobium_loddigesii_AB593604 ATGAC-TCTCGGCAATGGATATCTCGGCTCTCGCATCGATGAAGAGCGCAGCGAAATGCGATATGTGGTGC-GAATTGCAGAATCCCGCGAACC-ATCGAGTCTTTGAACGCAAGTTGCGCCCGAGGCCAAC-CGGCCAAGGGCACGTCCGCCTG---GGCGTCA-GTCATTTTATCGCTCT

Dendrobium_loddigesii_AB873183 ATGAC-TCTCGGCAATGGATATCTCGGCTCTCGCATCGATGAAGAGCGCAGCGAAATGCGATATGTGGTGC-GAATTGCAGAATCCCGCGAACC-ATCGAGTCTTTGAACGCAAGTTGCGCCCGAGGCCAAC-CGGCCAAGGGCACGTCCGCCTG---GGCGTCA-GTCATTTTATCGCTCT

Dendrobium_loddigesii_AF311778 ATGAC-TCTCGGCAATGGATATCTCGGCTCTCGCATCGATGAAGAGCGCAGCGAAATGCGATATGTGGTGC-GAATTGCAGAATCCCGCGAACC-ATCGAGTCTTTGAACGCAAGTTGCGCCCGAGGCCAAC-CGGCCAAGGGCACGTCCGCCTG---GGCGTCA-GTCATTTTATCGCTCT

Dendrobium_loddigesii_AY485703 ATGACCTCTCGGCAATGGATATCTCGGCTCTCGCATCGATGAAGAGCGCAGCGAAATGCGATATGTGGTGC-GAATTGCAGAATCCCGCGAACC-ATCGAGTCTTTGAACGCAAGTTGCGCCCGAGGCCAAC-CGGCCAAGGGCACGTCCGCCTG---GGCGTCA-ATCATTTTTTCGCTCT

Dendrobium_loddigesii_EU121418 ATGAC-TCTCGGCAATGGATATCTCGGCTCTCGCATCGATGAAGAGCGCAGCGAAATGCGATATGTGGTGC-GAATTGCAGAATCCCGCGAACC-ATCGAGTCTTTGAACGCAAGTTGCGCCCGAGGCCAAC-CGGCCAAGGGCACGTCCGCCTG---GGCGTCA-GTCATTTTATCGCTCT

Dendrobium_loddigesii_EU592016 ACGAC-TCTCGGCAATGGATATCTCGGCTCTCGCATCGATGAAGAGCGCAGCGAAATGCGATATGTGGTGC-GAATTGCAGAATCCCGCGAACC-ATCGAGTCTTTGAACGCAAGTTGCGCCTGAGGCCAAC-CGGCTGAGGGCACGTCCGCCTG---GGCGTCA-AGCATTTTATCGCTCT

Dendrobium_loddigesii_HM590374 ATGAC-TCTCGGCAATGGATATCTCGGCTCTCGCATCGATGAAGAGCGCAGCGAAATGCGATATGTGGTGC-GAATTGCAGAATCCCGCGAACC-ATCGAGTCTTTGAACGCAAGTTGCGCCCGAGGCCAAC-CGGCCAAGGGCACGTCCGCCTG---GGCGTCA-GTCATTTTATCGCTCT

Dendrobium_loddigesii_HQ114220 ATGAC-TCTCGGCAATGGATATCTCGGCTCTCGCATCGATGAAGAGCGCAGCGAAATGCGATATGTGGTGC-GAATTGCAGAATCCCGCGAACC-ATCGAGTCTTTGAACGCAAGTTGCGCCCGAGGCCAAC-CGGCCAAGGGCACGTCCGCCTG---GGCGTCA-GTCATTTTATCGCTCT

Dendrobium_loddigesii_JN388569 ATGAC-TCTCGGCAATGGATATCTCGGCTCTCGCATCGATGAAGAGCGCAGCGAAATGCGATATGTGGTGC-GAATTGCAGAATCCCGCGAACC-ATCGAGTCTTTGAACGCAAGTTGCGCCCGAGGCCAAC-CGGCCAAGGGCACGTCCGCCTG---GGCGTCA-GTCATTTTATCGCTCT

Dendrobium_loddigesii_KC205187 ATGAC-TCTCGGCAATGGATATCTCGGCTCTCGCATCGATGAAGAGCGCAGCGAAATGCGATATGTGGTGC-GAATTGCAGAATCCCGCGAACC-ATCGAGTCTTTGAACGCAAGTTGCGCCCGAGGCCAAC-CGGCCAAGGGCACGTCCGCCTG---GGCGTCA-GTCATTTTATCGCTCT

Dendrobium_loddigesii_KF143481 ATGAC-TCTCGGCAATGGATATCTCGGCTCTCGCATCGATGAAGAGCGCAGCGAAATGCGATATGTGGTGC-GAATTGCAGAATCCCGCGAACC-ATCGAGTCTTTGAACGCAAGTTGCGCCCGAGGCCAAC-CGGCCAAGGGCACGTCCGCCTG---GGCGTCA-GTCATTTTATCGCTCT

Dendrobium_loddigesii_KJ672683 ATGAC-TCTCGGCAATGGATATCTCGGCTCTCGCATCGATGAAGAGCGCAGCGAAATGCGATATGTGGTGC-GAATTGCAGAATCCCGCGAACC-ATCGAGTCTTTGAACGCAAGTTGCGCCCGAGGCCAAC-CGGCCAAGGGCACGTCCGCCTG---GGCGTCA-ATCATTTTTTCGCTCT

Dendrobium_loddigesii_KP159301 ATGAC-TCTCGGCAATGGATATCTCGGCTCTCGCATCGATGAAGAGCGCAGCGAAATGCGATATGTGGTGC-GAATTGCAGAATCCCGCGAACC-ATCGAGTCTTTGAACGCAAGTTGCGCCCGAGGCCAAC-CGGCCAAGGGCACGTCCGCCTG---GGCGTCA-GTCATTTTATCGCTCT

Dendrobium_loddigesii_KP264997 ATGAC-TCTCGGCAATGGATATCTCGGCTCTCGCATCGATGAAGAGCGCAGCGAAATGCGATATGTGGTGC-GAATTGCAGAATCCCGCGAACC-ATCGAGTCTTTGAACGCAAGTTGCGCCCGAGGCCAAC-CGGCCAAGGGCACGTCCGCCTG---GGCGTCA-GTCATTTTATCGCTCT

Dendrobium_loddigesii_KT778746 ATGAC-TCTCGGCAATGGATATCTCGGCTCTCGCATCGATGAAGAGCGCAGCGAAATGCGATATGTGGTGC-GAATTGCAGAATCCCGCGAACC-ATCGAGTCTTTGAACGCAAGTTGCGCCCGAGGCCAAC-CGGCCAAGGGCACGTCCGCCTG---GGCGTCA-GTCATTTTATCGCTCT

Dendrobium_loddigesii_KY966562 ATGAC-TCTCGGCAATGGATATCTCGGCTCTCGCATCGATGAAGAGCGCAGCGAAATGCGATATGTGGTGC-GAATTGCAGAATCCCGCGAACC-ATCGAGTCTTTGAACGCAAGTTGCGCCCGAGGCCAAC-CGGCCAAGGGCACGTCCGCCTG---GGCGTCA-GTCATTTTATCGCTCT

Dendrobium_loddigesii_SG1255 ATGAC-TCTCGGCAATGGATATCTCGGCTCTCGCATCGATGAAGAGCGCAGCGAAATGCGATATGTGGTGC-GAATTGCAGAATCCCGCGAACC-ATCGAGTCTTTGAACGCAAGTTGCGCCCGAGGCCAAC-CGGCCAAGGGCACGTCCGCCTG---GGCGTCA-GTCATTTTATCGCTCT

Dendrobium_spatella_AB847671 ATGAC-TCTCGACAATGGATATCTCGGCTCTTGCATCGATGAAGAGCGCAGCGAAATGCGATACGTGGTGC-GAATTGCAGAATCCCGCGAACC-ATCGAGTCTTTGAACGCAAGTTGCGCCCGAGGCCAAC-CGGCCAAGGGCACGTCCGCCTG---GGCGTCA-AGCATTACGTCACTCC

Dendrobium_spatella_KF143512 ATGAC-TCTCGACAATGGATATCTCGGCTCTTGCATCGATGAAGAGCGCAGCGAAATGCGATACGTGGTGC-GAATTGCAGAATCCCGCGAACC-ATCGAGTCTTTGAACGCAAGTTGCGCCCGAGGCCAAC-CGGCCAAGGGCACGTCCGCCTG---GGCGTCA-AGCATTACGTCACTCC

Dendrobium_spatella_KF143513 ATGAC-TCTCGACAATGGATATCTCGGCTCTTGCATCGATGAAGAGCGCAGCGAAATGCGATACGTGGTGC-GAATTGCAGAATCCCGCGAACC-ATCGAGTCTTTGAACGCAAGTTGCGCCCGAGGCCAAC-CGGCCAAGGGCACGTCCGCCTG---GGCGTCA-AGCATTACGTCACTCC

Dendrobium_spatella_SG1357 ATGAC-TCTCGACAATGGATATCTAGGCTCTTGCATCGATGAAGAGCGCAGCGAAATGCGATACGTGGTGC-GAATTGCAGAATCCCGCGAACC-ATCGAGTCTTTGAACGCAAGTTGCGCCCGAGGCCAAC-CGGCCAAGGGCACGTCCGCCTG---GGCGTCA-AGCATTACGTCACTCC

Dendrobium_spatella_SG1358 ATGAC-TCTCGACAATGGATATCTCGGCTCTTGCATCGATGAAGAGCGCAGCGAAATGCGATACGTGGTGC-GAATTGCAGAATCCCGCGAACC-ATCGAGTCTTTGAACGCAAGTTGCGCCCGAGGCCAAC-CGGCCAAGGGCACGTCCGCCTG---GGCGTCA-AGCATTACGTCACTCC

Dendrolirium_lasiopetalum_KFBG701 ACGAC-TCTCGGCAATGGATATCTCGGCTCTCGCATCGATGAAGAGCGCAGCGAAATGCGATACGTGGTGT-GAATTGCAGAATCCCGCGAACC-ATCGAGTCTTTGAACGCAAGTTGCGCCCGAGGCCAAC-CGGTTGAGGGCACGTCTGCCTG---GGCGTCA-AGCGTTACGTCGCTCC

Dendrolirium_lasiopetalum_PK12173 ACGAC-TCTCGGCAATGGATATCTCGGCTCTCGCATCGATGAAGAGCGCAGCGAAATGCGATACGTGGTGT-GAATTGCAGAATCCCGCGAACC-ATCGAGTCTTTGAACGCAAGTTGCGCCCGAGGCCAAC-CGGTTGAGGGCACGTCTGCCTG---GGCGTCA-AGCGTTACGTCGCTCC

Dendrolirium_lasiopetalum_PK12174 ACGAC-TCTCGGCAATGGATATCTCGGCTCTCGCATCGATGAAGAGCGCAGCGAAATGCGATACGTGGTGT-GAATTGCAGAATCCCGCGAACC-ATCGAGTCTTTGAACGCAAGTTGCGCCCGAGGCCAAC-CGGTTGAGGGCACGTCTGCCTG---GGCGTCA-AGCGTTACGTCGCTCC

Dendrolirium_lasiopetalum_SG1312 ACGAC-TCTCGGCAATGGATATCTCGGCTCTCGCATCGATGAAGAGCGCAGCGAAATGCGATACGTGGTGT-GAATTGCAGAATCCCGCGAACC-ATCGAGTCTTTGAACGCAAGTTGCGCCCGAGGCCAAC-CGGTTGAGGGCACGTCTGCCTG---GGCGTCA-AGCGTTACGTCGCTCC

Didymoplexiella_siamensis_PK12024 ATGAC-TCTCGGCAATGGATATCTCGGCTCTCGCATCGATGAAGAGCGCAGTGAAATGCGATACGTGGTGC-GAATTGCAGAATCCCGCGAACC-ATCGAGTCTTTGAACGCAAGTTGCGCCCGAGGCCAAT-CGGCCGAGGGCACGCCCGCCTG---GGCGACACAGCATTGCTTCGCTCC

Didymoplexiella_siamensis_SG1242 ATGAC-TCTCGGCAATGGATATCTCGGCTCTCGCATCGATGAAGAGCGCAGTGAAATGCGATACGTGGTGC-GAATTGCAGAATCCCGCGAACC-ATCGAGTCTTTGAACGCAAGTTGCGCCCGAGGCCAAT-CGGCCGAGGGCACGCCCGCCTG---GGCGACACAGCATTGCTTCGCTCC

Dienia_ophrydis_AY907111 ATGAC-TCTCGGCAATGGATATCTCGGCTCTTGCATCGATGAAGAGCGCAGCAAAATGCGATACGTGATGC-GAATTGCAGAATCCCGCGAACC-ATCGAGTATTTGAACGCAAGTTGCGCCCGAGGCCAAC-CGGTCAAGGGCACGTTTACCTG---GGTGTCA-AGCGTTGCTTCGCTTC

Dienia_ophrydis_AY907114 ATGAC-TCTCGGCAATGGATATCTCGGCTCTTGCATCGATGAAGAGCGCAGCAAAATGCGATAC---ATGC-GAATTGCAGAATCCCGCGAACC-ATCGAGTATTTGAACGCAAGTTGCGCCCGAGGCCAAC-CGGTCAAGGGCACGTTTACCTG---GGTGTCA-AGCGTTGCTTCGCTTC

Dienia_ophrydis_KFBG7819 ATGAC-TCTCGGCAATGGATATCTCGGCTCTTGCATCGATGAAGAGCGCAGCAAAATGCGATACGTGATGC-GAATTGCAGAATCCCGCGAACC-ATCGAGTATTTGAACGCAAGTTGCGCCCGAGGCCAAC-CGGTCAAGGGCACGTTTACCTG---GGTGTCA-AGCGTTGCTTCGCTTC

Dienia_ophrydis_KJ459275 ATGAC-TCTCGGCAATGGATATCTCGGCTCTTGCATCGATGAAGAGCGCAGCAAAATGCGATACGTGATGC-GAATTGCAGAATCCCGCGAACC-ATCGAGTATTTGAACGCAAGTTGCGCCCGAGGCCAAC-CGGTCAAGGGCACGTTTACCTG---GGTGTCA-AGCGTTGCTTCGCTTC

Dienia_ophrydis_MF287970 ATGAC-TCTCGGCAATGGATATCTCGGCTCTTGCATCGATGAAGAGCGCAGCAAAATGCGATACGTGATGC-GAATTGCAGAATCCCGCGAACC-ATCGAGTATTTGAACGCAAGTTGCGCCCGAGGCCAAC-CGGTCAAGGGCACGTTTACCTG---GGTGTCA-AGCGTTGCTTCGCTTC

Dienia_ophrydis_SG1201 ATGAC-TCTCGGCAATGGATATCTCGGCTCTTGCATCGATGAAGAGCGCAGCAAAATGCGATACGTGATGC-GAATTGCAGAATCCCGCGAACC-ATCGAGTATTTGAACGCAAGTTGCGCCCGAGGCCAAC-CGGTCAAGGGCACGTTTACCTG---GGTGTCA-AGCGTTGCTTCGCTTC

Dienia_ophrydis_SG1254 ATGAC-TCTCGGCAATGGATATCTCGGCTCTTGCATCGATGAAGAGCGCAGCAAAATGCGATACGTGATGC-GAATTGCAGAATCCCGCGAACC-ATCGAGTATTTGAACGCAAGTTGCGCCCGAGGCCAAC-CGGTCAAGGGCACGTTTACCTG---GGTGTCA-AGCGTTGCTTCGCTTC

Dienia_ophrydis_SG1276 ATGAC-TCTCGGCAATGGATATCTCGGCTCTTGCATCGATGAAGAGCGCAGCAAAATGCGATACGTGATGC-GAATTGCAGAATCCCGCGAACC-ATCGAGTATTTGAACGCAAGTTGCGCCCGAGGCCAAC-CGGTCAAGGGCACGTTTACCTG---GGTGTCA-AGCGTTGCTTCGCTTC

Diploprora_championii_KFBG628 ACGAC-TCTCGACAATGGATATCTCGGCTCTCGCATCGATGAAGAGCGCAGCGAAATGCGATACGTGGTGC-GAATTGCAGAATCCCGCGAACC-ATCGAGTCTTTGAACGCAAGTTGCGCCCGAGGCCAAT-CGGTCGAGGGCACGTCCGCCTG---GGCGTCA-AGCGTTGCGCCGCTCC

Diploprora_championii_KJ733410 ACGAC-TCTCGACAATGGATATCTCGGCTCTCGCATCGATGAAGAGCGCAGCGAAATGCGATACGTGGTGC-GAATTGCAGAATCCCGCGAACC-ATCGAGTCTTTGAACGCAAGTTGCGCCCGAGGCCAAT-CGGTCGAGGGCACGTCCGCCTG---GGCGTCA-AGCGTTGCGCCGCTCC

Diploprora_championii_KY966590 ACGAC-TCTCGACAATGGATATCTCGGCTCTCGCATCGATGAAGAGCGCAGCGAAATGCGATACGTGGTGC-GAATTGCAGAATCCCGCGAACC-ATCGAGTCTTTGAACGCAAGTTGCGCCCGAGGCCAAT-CGGTCGAGGGCACGTCCGCCTG---GGCGTCA-AGCGTTGCGCCGCTCC

Diploprora_championii_KY966591 ACGAC-TCTCGACAATGGATATCTCGGCTCTCGCATCGATGAAGAGCGCAGCGAAATGCGATACGTGGTGC-GAATTGCAGAATCCCGCGAACC-ATCGAGTCTTTGAACGCAAGTTGCGCCCGAGGCCAAT-CGGTCGAGGGCACGTCCGCCTG---GGCGTCA-AGCGTTGCGCCGCTCC

Diploprora_championii_PK12025 ACGAC-TCTCGACAATGGATATCTCGGCTCTCGCATCGATGAAGAGCGCAGCGAAATGCGATACGTGGTGC-GAATTGCAGAATCCCGCGAACC-ATCGAGTCTTTGAACGCAAGTTGCGCCCGAGGCCAAT-CGGTCGAGGGCACGTCCGCCTG---GGCGTCA-AGCGTTGCGCCGCTCC

Diploprora_championii_PK12095 ACGAC-TCTCGACAATGGATATCTCGGCTCTCGCATCGATGAAGAGCGCAGCGAAATGCGATACGTGGTGC-GAATTGCAGAATCCCGCGAACC-ATCGAGTCTTTGAACGCAAGTTGCGCCCGAGGCCAAT-CGGTCGAGGGCACGTCCGCCTG---GGCGTCA-AGCGTTGCGCCGCTCC

Diploprora_championii_SG1144 ACGAC-TCTCGACAATGGATATCTCGGCTCTCGCATCGATGAAGAGCGCAGCGAAATGCGATACGTGGTGC-GAATTGCAGAATCCCGCGAACC-ATCGAGTCTTTGAACGCAAGTTGCGCCCGAGGCCAAT-CGGTCGAGGGCACGTCCGCCTG---GGCGTCA-AGCGTTGCGCCGCTCC

Diploprora_championii_SG1230 ACGAC-TCTCGACAATGGATATCTCGGCTCTCGCATCGATGAAGAGCGCAGCGAAATGCGATACGTGGTGC-GAATTGCAGAATCCCGCGAACC-ATCGAGTCTTTGAACGCAAGTTGCGCCCGAGGCCAAT-CGGTCGAGGGCACGTCCGCCTG---GGCGTCA-AGCGTTGCGCCGCTCC

Epipogium_roseum_EU711232 ACGAC-TCTCGACAATGGATATCTCGGCTCTCGCATCGATGAAGAACGCAGCGAAATGCGATACGTGGTGC-GAATTGCAGAATCCCGCGAACC-ATCGAGTCTTTGAACGCAAGTTGCGCCCAAGGCCAGT-AGGCCAAGGGCACGCCCGCTTG---GGCGTCA-AGCATTGCATCTCTCC

Epipogium_roseum_SG1249 ACGAC-TCTCGACAATGGATATCTCGGCTCTCGCATCGATGAAGAACGCAGCGAAATGCGATACGTGGTGC-GAATTGCAGAATCCCGCGAACC-ATCGAGTCTTTGAACGCAAGTTGCGCCCAAGGCCAGT-AGGCCAAGGGCACGCCCGCTTG---GGCGTCA-TGCATTGCATCTCTCC

Epipogium_roseum_SG1250 ACGAC-TCTCGACAATGGATATCTCGGCTCTCGCATCGATGAAGAACGCAGCGAAATGCGATACGTGGTGC-GAATTGCAGAATCCCGCGAACC-ATCGAGTCTTTGAACGCAAGTTGCGCCCAAGGCCAGT-AGGCCAAGGGCACGCCCGCTTG---GGCGTCA-TGCATTGCATCTCTCC

Eria_scabrilinguis_KFBG916 ACGAC-TCTCGGCAATGGATATCTCGGCTCTCGCATCGATGAAGAGCGCAGCGAAATGCGATACGTGGTGC-GAATTGCAGAATCCCGCGAACC-ATCGAGTCTTTGAACGCAAGTTGCGCCCGAGGCCAAC-CGGCCGAGGGCACGTCTGCCTG---GGCGTCA-AGCGTTGCATCGCTCC

Eria_scabrilinguis_KY239252 ACGAC-TCTCGGCAATGGATATCTCGGCTCTCGCATCGATGAAGAGCGCAGCGAAATGCGATACGTGGTGC-GAATTGCAGAATCCCGCGAACC-ATCGAGTCTTTGAACGCAAGTTGCGCCCGAGGCCAAC-CGGCCGAGGGCACGTCTGCCTG---GGCGTCA-AGCGTTGCATCGCTCC

Eria_scabrilinguis_KY966596 ACGAC-TCTCGGCAATGGATATCTCGGCTCTCGCATCGATGAAGAGCGCAGCGAAATGCGATACGTGGTGC-GAATTGCAGAATCCCGCGAACC-ATCGAGTCTTTGAACGCAAGTTGCGCCCGAGGCCAAC-CGGCCGAGGGCACGTCTGCCTG---GGCGTCA-AGCGTTGCATCGCTCC

Eria_scabrilinguis_PK12164 ACGAC-TCTCGGCAATGGATATCTCGGCTCTCGCATCGATGAAGAGCGCAGCGAAATGCGATACGTGGTGC-GAATTGCAGAATCCCGCGAACC-ATCGAGTCTTTGAACGCAAGTTGCGCCCGAGGCCAAC-CGGCCGAGGGCACGTCTGCCTG---GGCGTCA-AGCGTTGCATCGCTCC

Eria_scabrilinguis_PK12165 ACGAC-TCTCGGCAATGGATATCTCGGCTCTCGCATCGATGAAGAGCGCAGCGAAATGCGATACGTGGTGC-GAATTGCAGAATCCCGCGAACC-ATCGAGTCTTTGAACGCAAGTTGCGCCCGAGGCCAAC-CGGCCGAGGGCACGTCTGCCTG---GGCGTCA-AGCGTTGCATCGCTCC

Eria_scabrilinguis_SG1302 ACGAC-TCTCGGCAATGGATATCTCGGCTCTCGCATCGATGAAGAGCGCAGCGAAATGCGATACGTGGTGC-GAATTGCAGAATCCCGCGAACC-ATCGAGTCTTTGAACGCAAGTTGCGCCCGAGGCCAAC-CGGCCGAGGGCACGTCTGCCTG---GGCGTCA-AGCGTTGCATCGCTCC

Erythrodes_blumei_JN166066 ATGAC-TCTCGGCAATGGATATCTTGGCTCTTGCATCGATGAAGAGCGCAGCGAAATGCGATACGTGGTGT-GAATTGCAGAATTCCGTGAACC-ATCGAATTTTTGAACGCAAGTTGCGCCCGAGGCCAAT-TGGCTAAGGGCACGTCCGCCTG---GGCGTCA-AGCATTACATCGCTTC

Erythrodes_blumei_KT343981 ATGAC-TCTCGGCAATGGATATCTTGGCTCTTGCATCGATGAAGAGCGCAGCGAAATGCGATACGTGGTGT-GAATTGCAGAATTCCGTGAACC-ATCGAATTTTTGAACGCAAGTTGCGCCCGAGGCCAAT-TGGCTAAGGGCACGTCCGCCTG---GGCGTCA-AGC-TTACATCGCTTA

Erythrodes_blumei_KT343982 ATGAC-TCTCGGCAATGGATATCTTGGCTCTTGCATCGATGAAGAGCGCAGCGAAATGCGATACGTGGTGT-GAATTGCAGAATTCCGTGAACC-ATCGAATTTTTGAACGCAAGTTGCGCCCGAGGCCAAT-TGGCTAAGGGCACGTCCGCCTG---GGCGTCG-AGCATTACATCGCTTC

Erythrodes_blumei_PK12103 ATGAC-TCTCGGCAATGGATATCTTGGCTCTTGCATCGATGAAGAGCGCAGCGAAATGCGATACGTGGTGT-GAATTGCAGAATTCCGTGAACC-ATCGAATTTTTGAACGCAAGTTGCGCCCGAGGCCAAT-TGGCTAAGGGCACGTCCGCCTG---GGCGTCA-AGCATTACATCGCTTC

Erythrodes_blumei_PK12104 ATGAC-TCTCGGCAATGGATATCTTGGCTCTTGCATCGATGAAGAGCGCAGCGAAATGCGATACGTGGTGT-GAATTGCAGAATTCCGTGAACC-ATCGAATTTTTGAACGCAAGTTGCGCCCGAGGCCAAT-TGGCTAAGGGCACGTCCGCCTG---GGCGTCA-AGCATTACATCGCTTC

Eulophia_flava_JN114508 ACGAC-TCTCGGCAATGGATATCTCGGCTCTCGCATCGATGAAGAGCGCAGCGAAATGCGATACGTGGTGC-GAATTGCAGAATCCCGCGAACC-ATCGAGTCTTTGAACGCAAGTTGCGCCTGAGGCCAGT-CGGCTGAGGGCACGTCCGCCTG---GGCGTCA-AGCTTCGCGTTGCTCC

Eulophia_flava_JN114509 ACGAC-TCTCGGCAATGGATATCTCGGCTCTCGCATCGATGAAGAGCGCAGCGAAATGCGATACGTGGTGC-GAATTGCAGAATCCCGCGAACC-ATCGAGTCTTTGAACGCAAGTTGCGCCTGAGGCCAGT-CGGCTGAGGGCACGTCCGCCTG---GGCGTCA-AGCTTCGCGTTGCTCC

Eulophia_flava_SG1158 ACGAC-TCTCGGCAATGGATATCTCGGCTCTCGCATCGATGAAGAGCGCAGCGAAATGCGATACGTGGTGC-GAATTGCAGAATCCCGCGAACC-ATCGAGTCTTTGAACGCAAGTTGCGCCTGAGGCCAGT-CGGCTGAGGGCACGTCCGCCTG---GGCGTCA-AGCTTCGCGTTGCTCC

Eulophia_flava_SG1159 ACGAC-TCTCGGCAATGGATATCTCGGCTCTCGCATCGATGAAGAGCGCAGCGAAATGCGATACGTGGTGC-GAATTGCAGAATCCCGCGAACC-ATCGAGTCTTTGAACGCAAGTTGCGCCTGAGGCCAGT-CGGCTGAGGGCACGTCCGCCTG---GGCGTCA-AGCTTCGCGTTGCTCC

Eulophia_graminea_FJ565666 ACGAC-TCTCGGCAATGGATATCTCGGCTCTCGCATCGATGAAGAGCGCAGCGAAATGCGATACGTGGTGC-GAATTGCAGAATCCCGCGAACC-GTCGAGTCTTTGAACGCAAGTTGCGCCTGAGGCCAGC-CGGCCGAGGGCACGTCCGCCTG---GGCGTCG-AGCTTCGCGTCGCTCC

Eulophia_graminea_KF318890 ACGAC-TCTCGGCAATGGATATCTCGGCTCTCGCATCGATGAAGAGCGCAGCGAAATGCGATACGTGGTGC-GAATTGCAGAATCCCGCGAACC-GTCGAGTCTTTGAACGCAAGTTGCGCCTGAGGCCAGC-CGGCCGAGGGCACGTCCGCCTG---GGCGTCG-AGCTTCGCGTCGCTCC

Eulophia_graminea_MH768268 ACGAC-TCTCGGCAATGGATATCTCGGCTCTCGCATCGATGAAGAGCGCAGCGAAATGCGATACGTGGTGC-GAATTGCAGAATCCCGCGAACC-GTCGAGTCTTTGAACGCAAGTTGCGCCTGAGGCCAGC-CGGCCGAGGGCACGTCCGCCTG---GGCGTCG-AGCTTCGCGTCGCTCC

Eulophia_graminea_MH768269 ACGAC-TCTCGGCAATGGATATCTCGGCTCTCGCATCGATGAAGAGCGCAGCGAAATGCGATACGTGGTGC-GAATTGCAGAATCCCGCGAACC-GTCGAGTCTTTGAACGCAAGTTGCGCCTGAGGCCAGC-CGGCCGAGGGCACGTCCGCCTG---GGCGTCG-AGCTTCGCGTCGCTCC

Eulophia_graminea_SG1270 ACGAC-TCTCGGCAATGGATATCTCGGCTCTCGCATCGATGAAGAGCGCAGCGAAATGCGATACGTGGTGC-GAATTGCAGAATCCCGCGAACC-GTCGAGTCTTTGAACGCAAGTTGCGCCTGAGGCCAGC-CGGCCGAGGGCACGTCCGCCTG---GGCGTCG-AGCTTCGCGTCGCTCC

Eulophia_graminea_SG1350 ACGAC-TCTCGGCAATGGATATCTCGGCTCTCGCATCGATGAAGAGCGCAGCGAAATGCGATACGTGGTGC-GAATTGCAGAATCCCGCGAACC-GTCGAGTCTTTGAACGCAAGTTGCGCCTGAGGCCAGC-CGGCCGAGGGCACGTCCGCCTG---GGCGTCG-AGCTTCGCGTCGCTCC

Eulophia_picta_JN114510 ACGAC-TCTCGGCAATGGATATCTCGGCTCTCGCATCGATGAAGAGCGCAGCGAAATGCGATACGTGGTGC-GAATTGCAGAATCCCGCGAACC-ATCGAGTCTTTGAACGCAAGTTGCGCCTGAGGTCAGC-TGGCCGAGGGCACGTCCGCCTG---GGCGTCA-AGCTTCGCGTCGCTCC

Eulophia_picta_JN114511 ACGAC-TCTCGGCAATGGATATCTCGGCTCTCGCATCGATGAAGAGCGCAGCGAAATGCGATACGTGGTGC-GAATTGCAGAATCCCGCGAACC-ATCGAGTCTTTGAACGCAAGTTGCGCCTGAGGTCAGC-TGGCCGAGGGCACGTCCGCCTG---GGCGTCA-AGCTTCGCGTCGCTCC

Eulophia_picta_JN114512 ACGAC-TCTCGGCAATGGATATCTCGGCTCTCGCATCGATGAAGAGCGCAGCGAAATGCGATACGTGGTGC-GAATTGCAGAATCCCGCGAACC-ATCGAGTCTTTGAACGCAAGTTGCGCCTGAGGTCAGC-TGGCCGAGGGCACGTCCGCCTG---GGCGTCA-AGCTTCGCGTCGCTCC

Eulophia_picta_JN114513 ACGAC-TCTCGGCAATGGATATCTCGGCTCTCGCATCGATGAAGAGCGCAGCGAAATGCGATACGTGGTGC-GAATTGCAGAATCCCGCGAACC-ATCGAGTCTTTGAACGCAAGTTGCGCCTGAGGTCAGC-TGGCCGAGGGCACGTCCGCCTG---GGCGTCA-AGCTTCGCGTCGCTCC

Eulophia_picta_PK12045 ACGAC-TCTCGGCAATGGATATCTCGGCTCTCGCATCGATGAAGAGCGCAGCGAAATGCGATACGTGGTGC-GAATTGCAGAATCCCGCGAACC-ATCGAGTCTTTGAACGCAAGTTGCGCCTGAGGTCAGC-TGGCCGAGGGCACGTCCGCCTG---GGCGTCA-AGCTTCGCGTCGCTCC

Eulophia_picta_PK12046 ACGAC-TCTCGGCAATGGATATCTCGGCTCTCGCATCGATGAAGAGCGCAGCGAAATGCGATACGTGGTGC-GAATTGCAGAATCCCGCGAACC-ATCGAGTCTTTGAACGCAAGTTGCGCCTGAGGTCAGC-TGGCCGAGGGCACGTCCGCCTG---GGCGTCA-AGCTTCGCGTCGCTCC

Eulophia_picta_PK12047 ACGAC-TCTCGGCAATGGATATCTCGGCTCTCGCATCGATGAAGAGCGCAGCGAAATGCGATACGTGGTGC-GAATTGCAGAATCCCGCGAACC-ATCGAGTCTTTGAACGCAAGTTGCGCCTGAGGTCAGC-TGGCCGAGGGCACGTCCGCCTG---GGCGTCA-AGCTTCGCGTCGCTCC

Eulophia_picta_PK12048 ACGAC-TCTCGGCAATGGATATCTCGGCTCTCGCATCGATGAAGAGCGCAGCGAAATGCGATACGTGGTGC-GAATTGCAGAATCCCGCGAACC-ATCGAGTCTTTGAACGCAAGTTGCGCCTGAGGTCAGC-TGGCCGAGGGCACGTCCGCCTG---GGCGTCA-AGCTTCGCGTCGCTCC

Eulophia_picta_PK12137 ACGAC-TCTCGGCAATGGATATCTCGGCTCTCGCATCGATGAAGAGCGCAGCGAAATGCGATACGTGGTGC-GAATTGCAGAATCCCGCGAACC-ATCGAGTCTTTGAACGCAAGTTGCGCCTGAGGTCAGC-TGGCCGAGGGCACGTCCGCCTG---GGCGTCA-AGCTTCGCGTCGCTCC

Eulophia_picta_PK12138 ACGAC-TCTCGGCAATGGATATCTCGGCTCTCGCATCGATGAAGAGCGCAGCGAAATGCGATACGTGGTGC-GAATTGCAGAATCCCGCGAACC-ATCGAGTCTTTGAACGCAAGTTGCGCCTGAGGTCAGC-TGGCCGAGGGCACGTCCGCCTG---GGCGTCA-AGCTTCGCGTCGCTCC

Eulophia_picta_PK12153 ACGAC-TCTCGGCAATGGATATCTCGGCTCTCGCATCGATGAAGAGCGCAGCGAAATGCGATACGTGGTGC-GAATTGCAGAATCCCGCGAACC-ATCGAGTCTTTGAACGCAAGTTGCGCCTGAGGTCAGC-TGGCCGAGGGCACGTCCGCCTG---GGCGTCA-AGCTTCGCGTCGCTCC

Eulophia_picta_SG1271 ACGAC-TCTCGGCAATGGATATCTCGGCTCTCGCATCGATGAAGAGCGCAGCGAAATGCGATACGTGGTGC-GAATTGCAGAATCCCGCGAACC-ATCGAGTCTTTGAACGCAAGTTGCGCCTGAGGTCAGC-TGGCCGAGGGCACGTCCGCCTG---GGCGTCA-AGCTTCGCGTCGCTCC

Eulophia_zollingeri_AB306313 ACGAC-TCTCGACAATGGATATCTCGGCTCTCGCATCGATGAAGAGCGCAGCGAAATGCGATACGTGGTGC-GAATTGCAGAATCCCGCGAACC-ATCGAGTCTTTGAACGCAAGTTGCGCCTGAGGCCAGC-CGGCCGAGGGCACGTCCGCCTG---GGCGTCA-AGCTTCGCGTCGCTCC

Eulophia_zollingeri_SG1262 ACGAC-TCTCGACAATGGATATCTCGGCTCTCGCATCGATGAAGAGCGCAGCAAAATGCGATACGTGGTGC-GAATTGCAGAATCCCGCGAACC-ATCGAGTCTTTGAACGCAAGTTGCGCCTGAGGCCAGC-CGGCCGAGGGCACGTCCGCCTG---GGCGCCA-AGCTTCGCGTCGCTCC

Eulophia_zollingeri_SG1263 ACGAC-TCTCGATAATGGATATCTCAGCTCTCGCATCGATGAAGAGCGCAGCGAAATGCGATACGTGGTGC-GAATTGCAGAATCCCGCGAACC-ATCGAGTCTTTGAACGCAAGTTGCGCCTGAGGCCAGC-CGGCCGAGGGCACGTCCGCCTG---GGCGTCA-AGCTTCGCGTCGCTCC

Eulophia_zollingeri_SG1264 ACGAC-TCTCGACAATGGATATCTCAGCTCTCGCATCGATGAAGAGCGCAGCGAAATGCGATACGTGGTGC-AAATTGCAGAATCCCGCGAACC-ATCGAGTCTTTGAACGCAAGTTGCGCGTGAGGCCAGC-CGGCCGAGGGCACGTCCGCCTG---GGCGTCA-AGCTTCGCGTCGCTCC

Eulophia_zollingeri_SG1265 ACGAC-TCTCGACAATGGATATCTCGGCTCTCGCATCGATGAAGAGCGCAGCGAAATGCGATACGTGGTGC-TAATTGCAGAATCCCGCGAACC-ATCGAGTCTTTGAACGCAAGTTGCGCCTGAGGCCAGC-TGGCCGAGGGCACGTCCGCCTG---GGTGTCA-AGCTTCGCGTCGCTCC

Eulophia_zollingeri_SG1353 ACGAC-TCTCGACAATGGATATCTCGGCTCTCGCATCGATGAAGAGCGCAGCGAAATGCGATACGTGGTGC-GAATTGCAGAATCCCGCGAACC-ATCGAGTCTTTGAACGCAAGTTGCGCCTGAGGCCAGC-CGGCCGAGGGCACGTCCGCCTG---GGCGTCA-AGCTTCGCGTCGCTCC

Gastrochilus_japonicus_AY228503 ACGAC-TCTCGGCAATGGATATCTCGGCTCTCGCATCGATGAAGAGCGCAGCGAAATGCGATACGTGGTGC-GAATTGCAGAATCCCGCGAACC-ATCGAGTCTTTGTACGCAAGTTGCGCCCGAGGCCAAT-CGGTCGAGGGCACGTCCGCCTG---GGCGTCA-AGCGTCGCGCCGCTCC

Gastrochilus_japonicus_KY966598 ACGAC-TCTCGACAATGGATATCTCGGCTCTCGCATCGATGAAGAGCGCAGCGAAATGCGATACGTGGTGC-GAATTGCAGAATCCCGCGAACC-ATCGAGTCTTTGAACGCAAGTTGCGCCCGAGGCCAAT-CGGTCGAGGGCACGTCCGCCTG---GGCGTCA-AGCGTCGCGCCGCTCC

Gastrochilus_japonicus_KY966599 ACGAC-TCTCGACAATGGATATCTCGGCTCTCGCATCGATGAAGAGCGCAGCGAAATGCGATACGTGGTGC-GAATTGCAGAATCCCGCGAACC-ATCGAGTCTTTGAACGCAAGTTGCGCCCGAGGCCAAT-CGGTCGAGGGCACGTCCGCCTG---GGCGTCA-AGCGTCGCGCCGCTCC

Goodyera_foliosa_HM140995 ATGAC-TCTCGGCAATGGATATCTTGGCTCTTGCATCGATGAAGAGCGCAGCGAAATGCGATACGTGGTGT-GAATTGCAGAATCCCGTGAACC-ATCGAGTTTTTGAACGCAAGTTGCGCCCGAGGCCAAT-TGGCTAAGGGCACGTCCGCCTG---GGCGTCA-AGCATTACGTCGCTTC

Goodyera_foliosa_KT344001 ATGAC-TCTCGGCAATGGATATCTTGGCTCTTGCATCGATGAAGAGCGCAGCGAAATGCGATACGTGGTGT-GAATTGCAGAATCCCGTGAACC-ATCGAGTTTTTGAACGCAAGTTGCGCCCGAGGCCAAT-TGGCTAAGGGCACGTCCGCCTG---GGCGTCA-AGC-TTACGTCGCTTA

Goodyera_foliosa_KT344002 ATGAC-TCTCGGCAATGGATATCTTGGCTCTTGCATCGATGAAGAGCGCAGCGAAATGCGATACGTGGTGT-GAATTGCAGAATCCCGTGAACC-ATCGAGTTTTTGAACGCAAGTTGCGCCCGAGGCCAAT-TGGCTAAGGGCACGTCCGCCTG---GGCGTCA-AGC-TTACGTCGCTTA

Goodyera_foliosa_KT344003 ATGAC-TCTCGGCAATGGATATCTTGGCTCTTGCATCGATGAAGAGCGCAGCGAAATGCGATACGTGGTGT-GAATTGCAGAATCCCGTGAACC-ATCGAGTTTTTGAACGCAAGTTGCGCCCGAGGCCAAT-TGGCTAAGGGCACGTCCGCCTG---GGCGTCA-AGC-TTACGTCGCTTA

Goodyera_foliosa_KT344004 ATGAC-TCTCGGCAATGGATATCTTGGCTCTTGCATCGATGAAGAGCGCAGCGAAATGCGATACGTGGTGT-GAATTGCAGAATCCCGTGAACC-ATCGAGTTTTTGAACGCAAGTTGCGCCCGAGGCCAAT-TGGCTAAGGGCACGTCCGCCTG---GGCGTCA-AGC-TTACGTCGCTTA

Goodyera_foliosa_KT344005 ATGAC-TCTCGGCAATGGATATCTTGGCTCTTGCATCGATGAAGAGCGCAGCGAAATGCGATACGTGGTGT-GAATTGCAGAATCCCGTGAACC-ATCGAGTTTTTGAACGCAAGTTGCGCCCGAGGCCAAT-TGGCTAAGGGCACGTCCGCCTG---GGCGTCA-AGC-TTACGTCGCTTA

Goodyera_foliosa_KT344006 ATGAC-TCTCGGCAATGGATATCTTGGCTCTTGCATCGATGAAGAGCGCAGCGAAATGCGATACGTGGTGT-GAATTGCAGAATCCCGTGAACC-ATCGAGTTTTTGAACGCAAGTTGCGCCCGAGGCCAAT-TGGCTAAGGGCACGTCCGCCTG---GGCGTCA-AGC-TTACGTCGCTTA

Goodyera_foliosa_KT344007 ATGAC-TCTCGGCAATGGATATCTTGGCTCTTGCATCGATGAAGAGCGCAGCGAAATGCGATACGTGGTGT-GAATTGCAGAATCCCGTGAACC-ATCGAGTTTTTGAACGCAAGTTGCGCCCGAGGCCAAT-TGGCTAAGGGCACGTCCGCCTG---GGCGTCA-AGC-TTACGTCGCTTA

Goodyera_foliosa_KT344008 ATGAC-TCTCGGCAATGGATATCTTGGCTCTTGCATCGATGAAGAGCGCAGCGAAATGCGATACGTGGTGT-GAATTGCAGAATCCCGTGAACC-ATCGAGTTTTTGAACGCAAGTTGCGCCCGAGGCCAAT-TGGCTAAGGGCACGTCCGCCTG---GGCGTCA-AGC-TTACGTCGCTTA

Goodyera_foliosa_KT344009 ATGAC-TCTCGGCAATGGATATCTTGGCTCTTGCATCGATGAAGAGCGCAGCGAAATGCGATACGTGGTGT-GAATTGCAGAATCCCGTGAACC-ATCGAGTTTTTGAACGCAAGTTGCGCCCGAGGCCAAT-TGGCTAAGGGCACGTCCGCCTG---GGCGTCA-AGC-TTACGTCGCTTA

Goodyera_foliosa_PK12067 ATGAC-TCTCGGCAATGGATATCTTGGCTCTTGCATCGATGAAGAGCGCAGCGAAATGCGATACGTGGTGT-GAATTGCAGAATCCCGTGAACC-ATCGAGTTTTTGAACGCAAGTTGCGCCCGAGGCCAAT-TGGCTAAGGGCACGTCCGCCTG---GGCGTCA-AGCATTACGTCGCTTC

Goodyera_foliosa_SG1309 ATGAC-TCTCGGCAATGGATATCTTGGCTCTTGCATCGATGAAGAGCGCAGCGAAATGCGATACGTGGTGT-GAATTGCAGAATCCCGTGAACC-ATCGAGTTTTTGAACGCAAGTTGCGCCCGAGGCCAAT-TGGCTAAGGGCACGTCCGCCTG---GGCGTCA-AGCATTACGTCGCTTC

Goodyera_foliosa_SG1315 ATGAC-TCTCGGCAATGGATATCTTGGCTCTTGCATCGATGAAGAGCGCAGCGAAATGCGATACGTGGTGT-GAATTGCAGAATCCCGTGAACC-ATCGAGTTTTTGAACGCAAGTTGCGCCCGAGGCCAAT-TGGCTAAGGGCACGTCCGCCTG---GGCGTCA-AGCATTACGTCGCTTC

Goodyera_foliosa_var_foliosa_KC205157 ATGAC-TCTCGGCAAAGGATTTCTTGGCTCTTGCATCGATGAAGAGCGCAGCGAAATGCGATACGTGGTGG-GAATTGGAAAATCCCGTGAACC-ATCGAGTTTTTGAACGCAAGTTGCGCCCGAGGCCAAT-TGGCTAAGGGCACGTCCGCCTG---GGCGTCA-AGCATTACGTCGCTTC

Goodyera_foliosa_var_laevis_HM140998 ATGAC-TCTCGGCAATGGATATCTTGGCTCTTGCATCGATGAAGAGCGCAGCGAAATGCGATACGTGGTGT-GAATTGCAGAATCCCGTGAACC-ATCGAGTTTTTGAACGCAAGTTGCGCCCGAGGCCAAT-TGGCTAAGGGCACGTCCGCCTG---GGCGTCA-AGCATTACGTCGCTTC

Goodyera_procera_HM151402 ATGAC-TCTCGGCAATGGATATCTTGGCTCTTGCATCGATGAAGAGCGCAGCGAAATGCGATACGTGGTGT-GAATTGCAGAATCCCGTGAACC-ATCGAATTTTTGAACGCAAGTTGCGCCCGAGGCCAAT-TGGCTAAGGGCACGTCCGCCTG---GGCGTCA-AGCATTACGTCGCTTC

Goodyera_procera_HM222488 ATGAC-TCTCGGCAATGGATATCTTGGCTCTTGCATCGATGAAGAGCGCAGCGAAATGCGATACGTGGTGT-GAATTGCAGAATCCCGTGAACC-ATCGAATTTTTGAACGCAAGTTGCGCCCGAGGCCAAT-TGGCTAAGGGCACGTCCGCCTG---GGCGTCA-AGCATTACGTCGCTTC

Goodyera_procera_JN114514 ATGAC-TCTCGGCAATGGATATCTTGGCTCTTGCATCGATGAAGAGCGCAGCGAAATGCGATACGTGGTGT-GAATTGCAGAATCCCGTGAACC-ATCGAATTTTTGAACGCAAGTTGCGCCCGAGGCCAAT-TGGCTAAGGGCACGTCCGCCTG---GGCGTCA-AGCATTACGTCGCTTC

Goodyera_procera_JN114515 ATGAC-TCTCGGCAATGGATATCTTGGCTCTTGCATCGATGAAGAGCGCAGCGAAATGCGATACGTGGTGT-GAATTGCAGAATCCCGTGAACC-ATCGAATTTTTGAACGCAAGTTGCGCCCGAGGCCAAT-TGGCTAAGGGCACGTCCGCCTG---GGCGTCA-AGCATTACGTCGCTTC

Goodyera_procera_JN114516 ATGAC-TCTCGGCAATGGATATCTTGGCTCTTGCATCGATGAAGAGCGCAGCGAAATGCGATACGTGGTGT-GAATTGCAGAATCCCGTGAACC-ATCGAATTTTTGAACGCAAGTTGCGCCCGAGGCCAAT-TGGCTAAGGGCACGTCCGCCTG---GGCGTCA-AGCATTACGTCGCTTC

Goodyera_procera_JN114517 ATGAC-TCTCGGCAATGGATATCTTGGCTCTTGCATCGATGAAGAGCGCAGCGAAATGCGATACGTGGTGT-GAATTGCAGAATCCCGTGAACC-ATCGAATTTTTGAACGCAAGTTGCGCCCGAGGCCAAT-TGGCTAAGGGCACGTCCGCCTG---GGCGTCA-AGCATTACGTCGCTTC

Goodyera_procera_JN114518 ATGAC-TCTCGGCAATGGATATCTTGGCTCTTGCATCGATGAAGAGCGCAGCGAAATGCGATACGTGGTGT-GAATTGCAGAATCCCGTGAACC-ATCGAATTTTTGAACGCAAGTTGCGCCCGAGGCCAAT-TGGCTAAGGGCACGTCCGCCTG---GGCGTCA-AGCATTACGTCGCTTC

Goodyera_procera_KC237319 ATGAC-TCTCGGCAATGGATATCTTGGCTCTTGCATCGATGAAGAGCGCAGCGAAATGCGATACGTGGTGT-GAATTGCAGAATCCCGTGAACC-ATCGAATTTTTGAACGCAAGTTGCGCCCGAGGCCAAT-TGGCTAAGGGCACGTCCGCCTG---GGCGTCA-AGCATTACGTCGCTTC

Goodyera_procera_KT344043 ATGAC-TCTCGGCAATGGATATCTTGGCTCTTGCATCGATGAAGAGCGCAGCGAAATGCGATACGTGGTGT-GAATTGCAGAATCCCGTGAACC-ATCGAATTTTTGAACGCAAGTTGCGCCCGAGGCCAAT-TGGCTAAGGGCACGTCCGCCTG---GGCGTCA-AGC-TTACGTCGCTTA

Goodyera_procera_KT344044 ATGAC-TCTCGGCAATGGATATCTTGGCTCTTGCATCGATGAAGAGCGCAGCGAAATGCGATACGTGGTGT-GAATTGCAGAATCCCGTGAACC-ATCGAATTTTTGAACGCAAGTTGCGCCCGAGGCCAAT-TGGCTAAGGGCACGTCCGCCTG---GGCGTCA-AGC-TTACGTCGCTT-

Goodyera_procera_KT344045 ATGAC-TCTCGGCAATGGATATCTTGGCTCTTGCATCGATGAAGAGCGCAGCGAAATGCGATACGTGGTGT-GAATTGCAGAATCCCGTGAACC-ATCGAATTTTTGAACGCAAGTTGCGCCCGAGGCCAAT-TGGCTAAGGGCACGTCCGCCTG---GGCGTCA-AGC-TTACGTCGCTTA

Goodyera_procera_KY966601 ATGAC-TCTCGGCAATGGATATCTTGGCTCTTGCATCGATGAAGAGCGCAGCGAAATGCGATACGTGGTGT-GAATTGCAGAATCCCGTGAACC-ATCGAATTTTTGAACGCAAGTTGCGCCCGAGGCCAAT-TGGCTAAGGGCACGTCCGCCTG---GGCGTCA-AGCATTACGTCGCTTC

Goodyera_procera_SG1152 ATGAC-TCTCGGCAATGGATATCTTGGCTCTTGCATCGATGAAGAGCGCAGCGAAATGCGATACGTGGTGT-GAATTGCAGAATCCCGTGAACC-ATCGAATTTTTGAACGCAAGTTGCGCCCGAGGCCAAT-TGGCTAAGGGCACGTCCGCCTG---GGCGTCA-AGCATTACGTCGCTTC

Goodyera_procera_SG1240 ATGAC-TCTCGGCAATGGATATCTTGGCTCTTGCATCGATGAAGAGCGCAGCGAAATGCGATACGTGGTGT-GAATTGCAGAATCCCGTGAACC-ATCGAATTTTTGAACGCAAGTTGCGCCCGAGGCCAAT-TGGCTAAGGGCACGTCCGCCTG---GGCGTCA-AGCATTACGTCGCTTC

Goodyera_procera_SG1241 ATGAC-TCTCGGCAATGGATATCTTGGCTCTTGCATCGATGAAGAGCGCAGCGAAATGCGATACGTGGTGT-GAATTGCAGAATCCCGTGAACC-ATCGAATTTTTGAACGCAAGTTGCGCCCGAGGCCAAT-TGGCTAAGGGCACGTCCGCCTG---GGCGTCA-AGCATTACGTCGCTTC

Goodyera_procera_SG1346 ATGAC-TCTCGGCAATGGATATCTTGGCTCTTGCATCGATGAAGAGCGCAGCGAAATGCGATACGTGGTGT-GAATTGCAGAATCCCGTGAACC-ATCGAATTTTTGAACGCAAGTTGCGCCCGAGGCCAAT-TGGCTAAGGGCACGTCCGCCTG---GGCGTCA-AGCATTACGTCGCTTC

Goodyera_pusilla_KM593694 ATGAC-TCTCGGCAATGGATATCTTGGCTCTTGCATCGATGAAGAGCGCAGCGAAATGCGATACGTGGTGT-GAATTGCAGAATCCCGTGAACC-ATCGAGTTTTTGAACGCAAGTTGCGCCCGAGGCCAAT-TGGCTAAGGGCACGTCCGCCTG---GGCGTCA-AGCATTACGTCGCTTC

Goodyera_pusilla_KT344046 ATGAC-TCTCGGCAATGGATATCTTGGCTCTTGCATCGATGAAGAGCGCAGCGAAATGCGATACGTGGTGT-GAATTGCAGAATCCCGTGAACC-ATCGAGTTTTTGAACGCAAGTTGCGCCCGAGGCCAAT-TGGCTAAGGGCACGTCCGCCTG---GGCGTCA-AGC-TTACGTCGCTTA

Goodyera_pusilla_KT344047 ATGAC-TCTCGGCAATGGATATCTTGGCTCTTGCATCGATGAAGAGCGCAGCGAAATGCGATACGTGGTGT-GAATTGCAGAATCCCGTGAACC-ATCGAGTTTTTGAACGCAAGTTGCGCCCGAGGCCAAT-TGGCTAAGGGCACGTCCGCCTG---GGCGTCA-AGC-TTACGTCGCTTA

Goodyera_seikomontana_KT344068 ATGAC-TCTCGGCAATGGATATCTTGGCTCTTGCATCGATGAAGAGCGCAGCGAAATGCGATACGTGGTGT-GAATTGCAGAATTCCGTGAACC-ATCGAATTTTTGAACGCAAGTTGCGCCCGAGGCCAAT-TGGCTGAGGGCACGTCCGCCTG---GGCGTCA-AGC-TTATGTCGCTTA

Goodyera_seikomontana_KT344069 ATGAC-TCTCGGCAATGGATATCTTGGCTCTTGCATCGATGAAGAGCGCAGCGAAATGCGATACGTGGTGT-GAATTGCAGAATTCCGTGAACC-ATCGAATTTTTGAACGCAAGTTGCGCCCGAGGCCAAT-TGGCTGAGGGCACGTCCGCCTG---GGCGTCA-AGC-TTATGTCGCTTA

Goodyera_seikomontana_KY966602 ATGAC-TCTCGGCAATGGATATCTTGGCTCTTGCATCGATGAAGAGCGCAGCGAAATGCGATACGTGGTGT-GAATTGCAGAATTCCGTGAACC-ATCGAATTTTTGAACGCAAGTTGCGCCCGAGGCCAAT-TGGCTGAGGGCACGTCCGCCTG---GGCGTCA-AGCATTATGTCGCTTC

Goodyera_seikoomontana_SG1252 ATGAC-TCTCGGCAATGGATATCTTGGCTCTTGCATCGATGAAGAGCGCAGCGAAATGCGATACGTGGTGT-GAATTGCAGAATTCCGTGAACC-ATCGAATTTTTGAACGCAAGTTGCGCCCGAGGCCAAT-TGGCTGAGGGCACGTCCGCCTG---GGCGTCA-AGCATTATGTCGCTTC

Goodyera_seikoomontana_SG1253 ATGAC-TCTCGGCAATGGATATCTTGGCTCTTGCATCGATGAAGAGCGCAGCGAAATGCGATACGTGGTGT-GAATTGCAGAATTCCGTGAACC-ATCGAATTTTTGAACGCAAGTTGCGCCCGAGGCCAAT-TGGCTGAGGGCACGTCCGCCTG---GGCGTCA-AGCATTATGTCGCTTC

Goodyera_seikoomontana_SG1354 ATGAC-TCTCGGCAATGGATATCTTGGCTCTTGCATCGATGAAGAGCGCAGCGAAATGCGATACGTGGTGT-GAATTGCAGAATTCCGTGAACC-ATCGAATTTTTGAACGCAAGTTGCGCCCGAGGCCAAT-TGGCTGAGGGCACGTCCGCCTG---GGCGTCA-AGCATTATGTCGCTTC

Goodyera_viridiflora_JN166067 ATGAC-TCTCGGCAATGGATATCTTGGCTCTTGCATCGATGAAGAGCGCAGCGAAATGCGATACGTGGTGT-GAATTGCAGAATTCCGTGAACC-ATCGAATTTTTGAACGCAAGTTGCGCCCGAGGCCAAT-TGGCTAAGGGCACGTCCGCCTG---GGCGTCA-AGCATTAYGTCGCTTC

Goodyera_viridiflora_KC205154 ATGAC-TCTCGGCAATGGATATCTTGGCTCTTGCATCGATGAAGAGCGCAGCGAAATGCGATACGTGGTGT-GAATTGCAGAATTCCGTGAACC-ATCGAATTTTTGAACGCAAGTTGCGCCCGAGGCCAAT-TGGCTAAGGGCACGTCCGCCTG---GGCGTCA-AGCATTACATCGCTTC

Goodyera_viridiflora_KT344078 ATGAC-TCTCGGCAATGGATATCTTGGCTCTTGCATCGATGAAGAGCGCAGCGAAATGCGATACGTGGTGT-GAATTGCAGAATTCCGTGAACC-ATCGAATTTTTGAACGCAAGTTGCGCCCGAGGCCAAT-TGGCTAAGGGCACGTCCGCCTG---GGCGTCA-AGC-TTACGTCGCTTA

Goodyera_viridiflora_KT344079 ATGAC-TCTCGGCAATGGATATCTTGGCTCTTGCATCGATGAAGAGCGCAGCGAAATGCGATACGTGGTGT-GAATTGCAGAATTCCGTGAACC-ATCGAATTTTTGAACGCAAGTTGCGCCCGAAGCCAAT-TGGCTAAGGGCACGTCCGCCTG---GGCGTCA-AGC-TTACGTCGCTTA

Goodyera_viridiflora_KT344080 ATGAC-TCTCGGCAATGGATATCTTGGCTCTTGCATCGATGAAGAGCGCAGCGAAATGCGATACGTGGTGT-GAATTGCAGAATTCCGTGAACC-ATCGAATTTTTGAACGCAAGTTGCGCCCGAGGCCAAT-TGGCTAAGGGCACGTCCGCCTG---GGCGTCA-AGC-TTATGTCGCTTA

Goodyera_viridiflora_KT344081 ATGAC-TCTCGGCAATGGATATCTTGGCTCTTGCATCGATGAAGAGCGCAGCGAAATGCGATACGTGGTGT-GAATTGCAGAATTCCGTGAACC-ATCGAATTTTTGAACGCAAGTTGCGCCCGAGGCCAAT-TGGCTAAGGGCACGTCCGCCTG---GGCGTCA-AGC-TTACGTCGCTTA

Goodyera_viridiflora_KT344082 ATGAC-TCTCGGCAATGGATATCTTGGCTCTTGCATCGATGAAGAGCGCAGCGAAATGCGATACGTGGTGT-GAATTGCAGAATTCCGTGAACC-ATCGAATTTTTGAACGCAAGTTGCGCCTGAGGCCAAT-TGGCTAAGGGCACGTCCGCCTG---GGCGTCA-AGC-TTACGTCGCTTA

Goodyera_viridiflora_KT344083 ATGAC-TCTCGGCAATGGATATCTTGGCTCTTGCATCGATGAAGAGCGCAGCGAAATGCGATACGTGGTGT-GAATTGCAGAATTCCGTGAACC-ATCGAATTTTTGAACGCAAGTTGCGCCCGAGGCCAAT-TGGCTAAGGGCACGTCCGCCTG---GGCGTCA-AGC-TTATGTCGCTTA

Goodyera_viridiflora_KT344084 ATGAC-TCTCGGCAATGGATATCTTGGCTCTTGCATCGATGAAGAGCGCAGCGAAATGCGATACGTGGTGT-GAATTGCAGAATTCCGTGAACC-ATCGAATTTTTGAACGCAAGTTGCGCCCGAGGCCAAT-TGGCTAAGGGCACGTCCGCCTG---GGCGTCA-AGC-TTACGTCGCTTA

Goodyera_viridiflora_KT344085 ATGAC-TCTCGGCAATGGATATCTTGGCTCTTGCATCGATGAAGAGCGCAGCGAAATGCGATACGTGGTGT-GAATTGCAGAATTCCGTGAACC-ATCGAATTTTTGAACGCAAGTTGCGCCCGAGGCCAAT-TGGCTAAGGGCACGTCCGCCTG---GGCGTCA-AGC-TTATGTCGCTTA

Goodyera_viridiflora_PK12170 ATGAC-TCTCGGCAATGGATATCTTGGCTCTTGCATYGATGAAGAGCGCAGCGAAATGCGATACGTGGTGT-GAATTGCAGAATTCCGTGAACC-ATCGAATTTTTGAACGCAAGTTGCGCCCGAGGCCAAT-TSGCTAAGGGCACGTCCGCCTG---GGCGTCA-AGCATTATGTCGCTTC

Goodyera_viridiflora_PK12172 ATGAC-TCTCGGCAATGGATATCTTGGCTCTTGCATCGATGAAGAGCGCAGCGAAATGCGATACGTGGTGT-GAATTGCAGAATTCCGTGAACC-ATCGAATTTTTGAACGCAAGTTGCGCCYGAGGCCAAT-TGGCTAAGGGCACGTCCGCCTG---GGCGTCA-AGCATTACGTCGCTTC

Goodyera_viridiflora_SG1305 ATGAC-TCTCGGCAATGGATATCTTGGCTCTTGCATCGATGAAGAGCGCAGCGAAATGCGATACGTGGTGT-GAATTGCAGAATTCCGTGAACC-ATCGAATTTTTGAACGCAAGTTGCGCCCGAGGCCAAT-TGGCTAAGGGCACGTCCGCCTG---GGCGTCA-AGCATTACGTCGCTTC

Goodyera_viridiflora_SG1306 ATGAC-TCTCGGCAATGGATATCTTGGCTCTTGCATCGATGAAGAGCGCAGCGAAATGCGATACGTGGTGT-GAATTGCAGAATTCCGTGAACC-ATCGAATTTTTGAACGCAAGTTGCGCCCGAGGCCAAT-TGGCTAAGGGCACGTCCGCCTG---GGCGTCA-AGCATTACGTCGCTTC

Habenaria_ciliolaris_MF944286 AGGAC-TCTCGGCAATGGATATCTTGGCTCTTGCATCGATGAAGAGCGCAGCGAAATGCGATACGTGGTGC-GAATTGCAGAATCCCGTGAACC-ATCGAGTTTTTGAACGCAAGTTGCGCCTGAGGCCACC-TGGCCAAGGGCACGTCCACCTG---GGCGTCA-AGCATTAAATCGCTCT

Habenaria_ciliolaris_MF944287 AGGAC-TCTCGGCAATGGATATCTTGGCTCTTGCATCGATGAAGAGCGCAGCGAAATGCGATACGTGGTGC-GAATTGCAGAATCCCGTGAACC-ATCGAGTTTTTGAACGCAAGTTGCGCCTGAGGCCACC-TGGCCAAGGGCACGTCCACCTG---GGCGTCA-AGCATTAAATCGCTCT

Habenaria_dentata_KFBG1009 AGGAC-TCTCGGCAATGGATATCTTGGCTCTTGCATCGATGAAGAGCGCAGCGAAATGCGATACGTGGTGC-GAATTGCAGAATCCCGTGAACC-ATCGAGTTTTTGAACGCAAGTTGCGCCTGAGGCCACC-TGGCCAAGGGCACGTCCACCTG---GGCGTCA-AGCATTAAATCGCTCT

Habenaria_dentata_KFBG2126B AGGAC-TCTCGGCAATGGATATCTTGGCTCTTGCATCGATGAAGAGCGCAGCGAAATGCGATACGTGGTGC-GAATTGCAGAATCCCGTGAACC-ATCGAGTTTTTGAACGCAAGTTGCGCCTGAGGCCACC-TGGCCAAGGGCACGTCCACCTG---GGCGTCA-AGCATTAAATCGCTCT

Habenaria_dentata_KJ460038 AGGAC-TCTCGGCAATGGATATCTTGGCTCTTGCATCGATGAAGAGCGCAGCGAAATGCGATACGTGGTGC-GAATTGCAGAATCCCGTGAACC-ATCGAGTTTTTGAACGCAAGTTGCGCCTGAGGCCACC-TGGCCAAGGGCACGTCCACCTG---GGCGTCA-AGCATTAAATCGCTCT

Habenaria_dentata_KY966605 AGGAC-TCTCGGCAATGGATATCTTGGCTCTTGCATCGATGAAGAGCGCAGCGAAATGCGATACGTGGTGC-GAATTGCAGAATCCCGTGAACC-ATCGAGTTTTTGAACGCAAGTTGCGCCTGAGGCCACC-TGGCCAAGGGCACGTCCACCTG---GGCGTCA-AGCATTAAATCGCTCT

Habenaria_dentata_PK12058 AGGAC-TCTCGGCAATGGATATCTTGGCTCTTGCATCGATGAAGAGCGCAGCGAAATGCGATACGTGGTGC-GAATTGCAGAATCCCGTGAACC-ATCGAGTTTTTGAACGCAAGTTGCGCCTGAGGCCACC-TGGCCAAGGGCACGTCCACCTG---GGCGTCA-AGCATTAAATCGCTCT

Habenaria_dentata_PK12059 AGGAC-TCTCGGCAATGGATATCTTGGCTCTTGCATCGATGAAGAGCGCAGCGAAATGCGATACGTGGTGC-GAATTGCAGAATCCCGTGAACC-ATCGAGTTTTTGAACGCAAGTTGCGCCTGAGGCCACC-TGGCCAAGGGCACGTCCACCTG---GGCGTCA-AGCATTAAATCGCTCT

Habenaria_dentata_SG1005 AGGAC-TCTCGGCAATGGATATCTTGGCTCTTGCATCGATGAAGAGCGCAGCGAAATGCGATACGTGGTGC-GAATTGCAGAATCCCGTGAACC-ATCGAGTTTTTGAACGCAAGTTGCGCCTGAGGCCACC-TGGCCAAGGGCACGTCCACCTG---GGCGTCA-AGCATTAAATCGCTCT

Habenaria_leptoloba_KY055535 AGGAC-TCTCGGCAATGGATA-CTTGGCTCTTGCATCGATGAAGAGCGCAGCGAAATGCGATACGTGGTGC-GAATTGCAGAATCCCGTGAACC-ATCGAGTTTTTGAACGCAAGTTGCGCCTGAGGCCAGC-TGGCCAAGGGCACGTCCGCCTG---GGCGTCA-AGCAT-GAATCGCTAC

Habenaria_leptoloba_PK12060 AGGAC-TCTCGGCAATGGATATCTTGGCTCTTGCATCGATGAAGAGCGCAGCGAAATGCGATACGTGGTGC-GAATTGCAGAATCCCGTGAACC-ATCGAGTTTTTGAACGCAAGTTGCGCCTGAGGCCAGC-TGGCCAAGGGCACGTCCGCCTG---GGCGTCA-AGCATTGAATCGCTAC

Habenaria_leptoloba_PK12061 AGGAC-TCTCGGCAATGGATATCTTGGCTCTTGCATCGATGAAGAGCGCAGCGAAATGCGATACGTGGTGC-GAATTGCAGAATCCCGTGAACC-ATCGAGTTTTTGAACGCAAGTTGCGCCTGAGGCCAGC-TGGCCAAGGGCACGTCCGCCTG---GGCGTCA-AGCATTGAATCGCTAC

Habenaria_leptoloba_PK12160 AGGAC-TCTCGGCAATGGATATCTTGGCTCTTGCATCGATGAAGAGCGCAGCGAAATGCGATACGTGGTGC-GAATTGCAGAATCCCGTGAACC-ATCGAGTTTTTGAACGCAAGTTGCGCCTGAGGCCAGC-TGGCCAAGGGCACGTCCGCCTG---GGCGTCA-AGCATTGAATCGCTAC

Habenaria_leptoloba_PK12161 AGGAC-TCTCGGCAATGGATATCTTGGCTCTTGCATCGATGAAGAGCGCAGCGAAATGCGATACGTGGTGC-GAATTGCAGAATCCCGTGAACC-ATCGAGTTTTTGAACGCAAGTTGCGCCTGAGGCCAGC-TGGCCAAGGGCACGTCCGCCTG---GGCGTCA-AGCATTGAATCGCTAC

Habenaria_leptoloba_SG1304 AGGAC-TCTCGGCAATGGATATCTTGGCTCTTGCATCGATGAAGAGCGCAGCGAAATGCGATACGTGGTGC-GAATTGCAGAATCCCGTGAACC-ATCGAGTTTTTGAACGCAAGTTGCGCCTGAGGCCAGC-TGGCCAAGGGCACGTCCGCCTG---GGCGTCA-AGCATTGAATCGCTAC

Habenaria_leptoloba_SG1362 AGGAC-TCTCGGCAATGGATATCTTGGCTCTTGCATCGATGAAGAGCGCAGCGAAATGCGATACGTGGTGC-GAATTGCAGAATCCCGTGAACC-ATCGAGTTTTTGAACGCAAGTTGCGCCTGAGGCCAGC-TGGCCAAGGGCACGTCCGCCTG---GGCGTCA-AGCATTGAATCGCTAC

Habenaria_linguella_MF944303 AGGAC-TCTCGGCAATGGATATCTTGGCTCTTGCATCGATGAAGAGCGCAGCGAAATGCGATACGTGGTGC-GAATTGCAGAATCCCGTGAACC-ATCGAGTTTTTGAACGCAAGTTGCGCCCGAGGCCACC-TGGCCAAGGGCACGTCCACCTG---GGCGTCA-AGCATTAAATCGCTCT

Habenaria_linguella_PK12049 AGGAC-TCTCGGCAATGGATATCTTGGCTCTTGCATCGATGAAGAGCGCAGCGAAATGCGATACGTGGTGC-GAATTGCAGAATCCCGTGAACC-ATCGAGTTTTTGAACGCAAGTTGCGCCCGAGGCCACC-TGGCCAAGGGCACGTCCACCTG---GGCGTCA-AGCATTAAATCGCTCT

Habenaria_linguella_PK12134 AGGAC-TCTCGGCAATGGATATCTTGGCTCTTGCATCGATGAAGAGCGCAGCGAAATGCGATACGTGGTGC-GAATTGCAGAATCCCGTGAACC-ATCGAGTTTTTGAACGCAAGTTGCGCCCGAGGCCACC-TGGCCAAGGGCACGTCCACCTG---GGCGTCA-AGCATTAAATCGCTCT

Habenaria_linguella_PK12141 AGGAC-TCTCGGCAATGGATATCTTGGCTCTTGCATCGATGAAGAGCGCAGCGAAATGCGATACGTGGTGC-GAATTGCAGAATCCCGTGAACC-ATCGAGTTTTTGAACGCAAGTTGCGCCCGAGGCCACC-TGGCCAAGGGCACGTCCACCTG---GGCGTCA-AGCATTAAATCGCTCT

Habenaria_linguella_SG1195 AGGAC-TCTCGGCAATGGATATCTTGGCTCTTGCATCGATGAAGAGCGCAGCGAAATGCGATACGTGGTGC-GAATTGCAGAATCCCGTGAACC-ATCGAGTTTTTGAACGCAAGTTGCGCCCGAGGCCACC-TGGCCAAGGGCACGTCCACCTG---GGCGTCA-AGCATTAAATCGCTCT

Habenaria_linguella_SG1299 AGGAC-TCTCGGCAATGGATATCTTGGCTCTTGCATCGATGAAGAGCGCAGCGAAATGCGATACGTGGTGC-GAATTGCAGAATCCCGTGAACC-ATCGAGTTTTTGAACGCAAGTTGCGCCCGAGGCCACC-TGGCCAAGGGCACGTCCACCTG---GGCGTCA-AGCATTAAATCGCTCT

Habenaria_linguella_SG1300 AGGAC-TCTCGGCAATGGATATCTTGGCTCTTGCATCGATGAAGAGCGCAGCGAAATGCGATACGTGGTGC-GAATTGCAGAATCCCGTGAACC-ATCGAGTTTTTGAACGCAAGTTGCGCCCGAGGCCACC-TGGCCAAGGGCACGTCCACCTG---GGCGTCA-AGCATTAAATCGCTCT

Habenaria_reniformis_PK12142 AGGAC-TCTCGGCAATGGATATCTTGGCTCTTGCATCGATGAAGAGCGCAGCGAAATGCGATACGTGGTGC-GAATTGCAGAATCCCGTGAACC-ATCGAGTTTTTGAACGCAAGTTGCGCCTGAGGCCACC-TGGCCAAGGGCACGTCCACCTG---GGCGTCA-AGCATTAAATCGCTCA

Habenaria_reniformis_PK12143 AGGAC-TCTCGGCAATGGATATCTTGGCTCTTGCATCGATGAAGAGCGCAGCGAAATGCGATACGTGGTGC-GAATTGCAGAATCCCGTGAACC-ATCGAGTTTTTGAACGCAAGTTGCGCCTGAGGCCACC-TGGCCAAGGGCACGTCCACCTG---GGCGTCA-AGCATTAAATCGCTCA

Habenaria_reniformis_PK12144 AGGAC-TCTCGGCAATGGATATCTTGGCTCTTGCATCGATGAAGAGCGCAGCGAAATGCGATACGTGGTGC-GAATTGCAGAATCCCGTGAACC-ATCGAGTTTTTGAACGCAAGTTGCGCCTGAGGCCACC-TGGCCAAGGGCACGTCCACCTG---GGCGTCA-AGCATTAAATCGCTCA

Habenaria_reniformis_PK12145 AGGAC-TCTCGGCAATGGATATCTTGGCTCTTGCATCGATGAAGAGCGCAGCGAAATGCGATACGTGGTGC-GAATTGCAGAATCCCGTGAACC-ATCGAGTTTTTGAACGCAAGTTGCGCCTGAGGCCACC-TGGCCAAGGGCACGTCCACCTG---GGCGTCA-AGCATTAAATCGCTCA

Habenaria_reniformis_PK12146 AGGAC-TCTCGGCAATGGATATCTTGGCTCTTGCATCGATGAAGAGCGCAGCGAAATGCGATACGTGGTGC-GAATTGCAGAATCCCGTGAACC-ATCGAGTTTTTGAACGCAAGTTGCGCCTGAGGCCACC-TGGCCAAGGGCACGTCCACCTG---GGCGTCA-AGCATTAAATTGCTCA

Habenaria_reniformis_SG1296 AGGAC-TCTCGGCAATGGATATCTTGGCTCTTGCATCGATGAAGAGCGCAGCGAAATGCGATACGTGGTGC-GAATTGCAGAATCCCGTGAACC-ATCGAGTTTTTGAACGCAAGTTGCGCCTGAGGCCACC-TGGCCAAGGGCACGTCCACCTG---GGCGTCA-AGCATTAAATCGCTCA

Habenaria_reniformis_SG1297 AGGAC-TCTCGGCAATGGATATCTTGGCTCTTGCATCGATGAAGAGCGCAGCGAAATGCGATACGTGGTGC-GAATTGCAGAATCCCGTGAACC-ATCGAGTTTTTGAACGCAAGTTGCGCCTGAGGCCACC-TGGCCAAGGGCACGTCCACCTG---GGCGTCA-AGCATTAAATCGCTCA

Habenaria_rhodocheila_KJ460043 AGGAC-TCTCGGCAATGGATATCTTGGCTCTTGCATCGATGAAGAGCGCAGCGAAATGCGATACGTGGTGC-GAATTGCAGAATCCCGTGAACC-ATCGAGTTTTTGAACGCAAGTTGCGCCTGAGGCCACC-TGGCCAAGGGCACGTCCACCTG---GGCGTCA-AGCATTAAATCGCTCT

Habenaria_rhodocheila_KR350167 AGGAC-TCTCGGCAATGGATATCTTGGCTCTTGCATCGATGAAGAGCGCAGCGAAATGCGATACGTGGTGC-GAATTGCAGAATCCCGTGAACC-ATCGAGTTTTTGAACGCAAGTTGCGCCTGAGGCCACC-TGGCCAAGGGCACGTCCACCTG---GGCGTCA-AGCATTAAATCGCTCT

Habenaria_rhodocheila_KY966607 AGGAC-TCTCGGCAATGGATATCTTGGCTCTTGCATCGATGAAGAGCGCAGCGAAATGCGATACGTGGTGC-GAATTGCAGAATCCCGTGAACC-ATCGAGTTTTTGAACGCAAGTTGCGCCTGAGGCCACC-TGGCCAAGGGCACGTCCACCTG---GGCGTCA-AGCATTAAATCGCTCT

Habenaria_rhodocheila_PK12139 AGGAC-TCTCGGCAATGGATATCTTGGCTCTTGCATCGATGAAGAGCGCAGCGAAATGCGATACGTGGTGC-GAATTGCAGAATCCCGTGAACC-ATCGAGTTTTTGAACGCAAGTTGCGCCTGAGGCCACC-TGGCCAAGGGCACGTCCACCTG---GGCGTCA-AGCATTAAATCGCTCT

Habenaria_rhodocheila_SG1289 AGGAC-TCTCGGCAATGGATATCTTGGCTCTTGCATCGATGAAGAGCGCAGCGAAATGCGATACGTGGTGC-GAATTGCAGAATCCCGTGAACC-ATCGAGTTTTTGAACGCAAGTTGCGCCTGAGGCCACC-TGGCCAAGGGCACGTCCACCTG---GGCGTCA-AGCATTAAATCGCTCT

Habenaria_rhodocheila_SG1290 AGGAC-TCTCGGCAATGGATATCTTGGCTCTTGCATCGATGAAGAGCGCAGCGAAATGCGATACGTGGTGC-GAATTGCAGAATCCCGTGAACC-ATCGAGTTTTTGAACGCAAGTTGCGCCTGAGGCCACC-TGGCCAAGGGCACGTCCACCTG---GGCGTCA-AGCATTAAATCGCTCT

Habenaria_rhodocheila_SG1291 AGGAC-TCTCGGCAATGGATATCTTGGCTCTTGCATCGATGAAGAGCGCAGCGAAATGCGATACGTGGTGC-GAATTGCAGAATCCCGTGAACC-ATCGAGTTTTTGAACGCAAGTTGCGCCTGAGGCCACC-TGGCCAAGGGCACGTCCACCTG---GGCGTCA-AGCATTAAATCGCTCT

Hetaeria_youngsayei_KY966608 ATGAC-TCTCGGCAATGGATATCTTGGCTCTTGCATCGATGAAGAGCGCAGCGAAATGCGATACGTGGTGT-GAATTGCAGAATCCCGTGAACC-ATCAAATCTTTGAACGCAAGTTGCGCCCGAGGCCAAT-TGGCTAAGGGCACGTCCGCCTG---GGCGTCA-AGCATTACATCGCTTC

Hetaeria_youngsayei_SG1244 ATGAC-TCTCGGCAATGGATATCTTGGCTCTTGCATCGATGAAGAGCGCAGCGAAATGCGATACGTGGTGT-GAATTGCAGAATCCCGTGAACC-ATCAAATCTTTGAACGCAAGTTGCGCCCGAGGCCAAT-TGGCTAAGGGCACGTCCGCCTG---GGCGTCA-AGCATTACATCGCTTC

Hetaeria_youngsayei_SG1245 ATGAC-TCTCGGCAATGGATATCTTGGCTCTTGCATCGATGAAGAGCGCAGCGAAATGCGATACGTGGTGT-GAATTGCAGAATCCCGTGAACC-ATCAAATCTTTGAACGCAAGTTGCGCCCGAGGCCAAT-TGGCTAAGGGCACGTCCGCCTG---GGCGTCA-AGCATTACATCGCTTC

Lecanorchis_nigricans_SG1279 AAGAC-TCTCGGCAACGGATATCTTGGCTCTTGCATCGATGAAGAACGCAGCGAAATGCGATATGTGTTGT-GAATTGCAGAATCCCGTGAACC-ATCCAGTCTTTGAACGCAAGTCGCGCCCAAGGTTGCA-CG-CCGAGGGCACGTCTGCATG---GGTGTGA-TGCGTTAAGTCGCTCT

Lecanorchis_nigricans_SG1280 AAGAC-TCTCGGCAACGGATATCTTGGCTCTTGCATCGATGAAGAACGCAGCGAAATGCGATATGTGTTGT-GAATTGCAGAATCCCGTGAACC-ATCCAGTCTTTGAACGCAAGTCGCGCCCAAGGTTGCA-CG-CCGAGGGCACGTCTGCATG---GGTGTGA-TGCGTTAAGTCGCTCT

Liparis_bootanensis_KFBG238 ACGAC-TCTCGGCAATGGATATCTCGGCTCTTGCATCGATGAAGAGCGCAGCGAAATGCGATACGTGGTGC-GAATTGCAGAATCCCGCGAACC-ATCGAGTCTTTGAACGCAAGTTGCGCCCGAGGCCAAC-CGGCCAAGGGCACGTTTGCCTG---GGCGTCA-AGCGTTGCGTCGCTTC

Liparis_bootanensis_KJ459280 ACGAC-TCTCGGCAATGGATATCTCGGCTCTTGCATCGATGAAGAGCGCAGCGAAATGCGATACGTGGTGC-GAATTGCAGAATCCCGCGAACC-ATCGAGTCTTTGAACGCAAGTTGCGCCCGAGGCCAAC-CGGCCAAGGGCACGTTTGCCTG---GGCGTCA-AGCGTTGCGTCGCTTC

Liparis_bootanensis_KY966611 ACGAC-TCTCGGCAATGGATATCTCGGCTCTTGCATCGATGAAGAGCGCAGCGAAATGCGATACGTGGTGC-GAATTGCAGAATCCCGCGAACC-ATCGAGTCTTTGAACGCAAGTTGCGCCCGAGGCCAAC-CGGCCAAGGGCACGTTTGCCTG---GGCGTCA-AGCGTTGCGTCGCTTC

Liparis_bootanensis_PK12162 ACGAC-TCTCGGCAATGGATATCTCGGCTCTTGCATCGATGAAGAGCGCAGCGAAATGCGATACGTGGTGC-GAATTGCAGAATCCCGCGAACC-ATCGAGTCTTTGAACGCAAGTTGCGCCCGAGGCCAAC-CGGCCAAGGGCACGTTTGCCTG---GGCGTCA-AGCGTTGCGTCGCTTC

Liparis_bootanensis_SG1215 ACGAC-TCTCGGCAATGGATATCTCGGCTCTTGCATCGATGAAGAGCGCAGCGAAATGCGATACGTGGTGC-GAATTGCAGAATCCCGCGAACC-ATCGAGTCTTTGAACGCAAGTTGCGCCCGAGGCCAAC-CGGCCGAGGGCACGTTTGCCTG---GGCGTCA-AGCGTTGCGTCGCTTC

Liparis_bootanensis_SG1216 ACGAC-TCTCGGCAATGGATATCTCGGCTCTTGCATCGATGAAGAGCGCAGCGAAATGCGATACGTGGTGC-GAATTGCAGAATCCCGCGAACC-ATCGAGTCTTTGAACGCAAGTTGCGCCCGAGGCCAAC-CGGCCGAGGGCACGTTTGCCTG---GGCGTCA-AGCGTTGCGTCGCTTC

Liparis_bootanensis_SG1307 ACGAC-TCTCGGCAATGGATATCTCGGCTCTTGCATCGATGAAGAGCGCAGCGAAATGCGATACGTGGTGC-GAATTGCAGAATCCCGCGAACC-ATCGAGTCTTTGAACGCAAGTTGCGCCCGAGGCCAAC-CGGCCAAGGGCACGTTTGCCTG---GGCGTCA-AGCGTTGCGTCGCTTC

Liparis_bootanensis_SG1336 ACGAC-TCTCGGCAATGGATATCTCGGCTCTTGCATCGATGAAGAGCGCAGCGAAATGCGATACGTGGTGC-GAATTGCAGAATCCCGCGAACC-ATCGAGTCTTTGAACGCAAGTTGCGCCCGAGGCCAAC-CGGCCAAGGGCACGTTTGCCTG---GGCGTCA-AGCGTTGCGTCGCTTC

Liparis_ferruginea_SG1156 ATGAC-TCTCGGCAATGGATATCTCGGCTCTTGCATCGATGAAGAGCGCAGCAAAATGCGATACGTGATGC-GAATTGCAGAACCCCGCGAACC-ATCGAGTCTTTGAACGCAAGTTGCGCCCGAGGCCAAT-CGGTCAAGGGCACGCCTTCCTG---GGTGTCA-AGCGTTGCATCGCTTT

Liparis_ferruginea_SG1266 ATGAC-TCTCGGCAATGGATATCTCGGCTCTTGCATCGATGAAGAGCGCAGCAAAATGCGATACGTGATGC-GAATTGCAGAACCCCGCGAACC-ATCGAGTCTTTGAACGCAAGTTGCGCCCGAGGCCAAT-CGGTCAAGGGCACGCCTTCCTG---GGTGTCA-AGCGTTGCATCGCTTT

Liparis_ferruginea_SG1267 ATGAC-TCTCGGCAATGGATATCTCGGCTCTTGCATCGATGAAGAGCGCAGCAAAATGCGATACGTGATGC-GAATTGCAGAACCCCGCGAACC-ATCGAGTCTTTGAACGCAAGTTGCGCCCGAGGCCAAT-CGGTCAAGGGCACGCCTTCCTG---GGTGTCA-AGCGTTGCATCGCTTT

Liparis_gigantea_PK12116 ATGAC-TCTCGGCAATGGATATCTCGGCTCTTGCATCGATGAAGAGCGCAGCAAAATGCGATACGTGATGC-GAATTGCAGAATCCCGCGAACC-ATCGAGTCTTTGAACGCAAGTTGCGCCCGAGGCCAAC-CGGTCAAGGGCACGTTTTCCTG---GGTGTCA-AGCGTTGCTTCGCTTT

Liparis_gigantea_PK12117 ATGAC-TCTCGGCAATGGATATCTCGGCTCTTGCATCGATGAAGAGCGCAGCAAAATGCGATACGTGATGC-GAATTGCAGAATCCCGCGAACC-ATCGAGTCTTTGAACGCAAGTTGCGCCCGAGGCCAAC-CGGTCAAGGGCACGTTTTCCTG---GGTGTCA-AGCGTTGCTTCGCTTT

Liparis_gigantea_PK12118 ATGAC-TCTCGGCAATGGATATCTCGGCTCTTGCATCGATGAAGAGCGCAGCAAAATGCGATACGTGATGC-GAATTGCAGAATCCCGCGAACC-ATCGAGTCTTTGAACGCAAGTTGCGCCCGAGGCCAAC-CGGTCAAGGGCACGTTTTCCTG---GGTGTCA-AGCGTTGCTTCGCTTT

Liparis_nervosa_AB289482 ATGAC-TCTCGGCAATGGATATNTCGGCTCTTGCATCGATGAAGAGCGCAGCAAAATGCGATACGTGATGCGGAATTGCAGAATCCCGCGAACC-ATCGAGTCTTTGAACGCAAGTTGCGCCCGAGGCCAAC-CGGTCAAGGGCACGTTTTCCTG---GGTGTCA-AGCGTTGCTTCGCTTT

Liparis_nervosa_AY907092 ATGAC-TCTCGGCAATGGATATCTCGGCTCTTGCATCGATGAAGAGCGCAGCAAAATGCGATACGTGATGC-GAATTGCAGAATCCCGCGAACC-ATCGAGTCTTTGAACGCAAGTTGCGCCCGAGGCCAAC-CGGTCAAGGGCACGTTTTCCTG---GGTGTCA-AGCGTTGCTTCGCTTT

Liparis_nervosa_JN114595 ATGAC-TCTCGGCAATGGATATCTCGGCTCTTGCATCGATGAAGAGCGCAGCAAAATGCGATACGTGATGC-GAATTGCAGAATCCCGCGAACC-ATCGAGTCTTTGAACGCAAGTTGCGCCCGAGGCCAAC-CGGTCAAGGGCACGTTTTCCTG---GGTGTCA-AGCGTTGCTTCGCTTT

Liparis_nervosa_JN114596 ATGAC-TCTCGGCAATGGATATCTCGGCTCTTGCATCGATGAAGAGCGCAGCAAAATGCGATACGTGATGC-GAATTGCAGAATCCCGCGAACC-ATCGAGTCTTTGAACGCAAGTTGCGCCCGAGGCCAAC-CGGTCAAGGGCACGTTTTCCTG---GGTGTCA-AGCGTTGCTTCGCTTT

Liparis_nervosa_JN114597 ATGAC-TCTCGGCAATGGATATCTCGGCTCTTGCATCGATGAAGAGCGCAGCAAAATGCGATACGTGATGC-GAATTGCAGAATCCCGCGAACC-ATCGAGTCTTTGAACGCAAGTTGCGCCCGAGGCCAAC-CGGTCAAGGGCACGTTTTCCTG---GGTGTCA-AGCGTTGCTTCGCTTT

Liparis_nervosa_JN114598 ATGAC-TCTCGGCAATGGATATCTCGGCTCTTGCATCGATGAAGAGCGCAGCAAAATGCGATACGTGATGC-GAATTGCAGAATCCCGCGAACC-ATCGAGTCTTTGAACGCAAGTTGCGCCCGAGGCCAAC-CGGTCAAGGGCACGTTTTCCTG---GGTGTCA-AGCGTTGCTTCGCTTT

Liparis_nervosa_JN114599 ATGAC-TCTCGGCAATGGATATCTCGGCTCTTGCATCGATGAAGAGCGCAGCAAAATGCGATACGTGATGC-GAATTGCAGAATCCCGCGAACC-ATCGAGTCTTTGAACGCAAGTTGCGCCCGAGGCCAAC-CGGTCAAGGGCACGTTTTCCTG---GGTGTCA-AGCGTTGCTTCGCTTT

Liparis_nervosa_JN114600 ATGAC-TCTCGGCAATGGATATCTCGGCTCTTGCATCGATGAAGAGCGCAGCAAAATGCGATACGTGATGC-GAATTGCAGAATCCCGCGAACC-ATCGAGTCTTTGAACGCAAGTTGCGCCCGAGGCCAAC-CGGTCAAGGGCACGTTTTCCTG---GGTGTCA-AGCGTTGCTTCGCTTT

Liparis_nervosa_JN114601 ATGAC-TCTCGGCAATGGATATCTCGGCTCTTGCATCGATGAAGAGCGCAGCAAAATGCGATACGTGATGC-GAATTGCAGAATCCCGCGAACC-ATCGAGTCTTTGAACGCAAGTTGCGCCCGAGGCCAAC-CGGTCAAGGGCACGTTTTCCTG---GGTGTCA-AGCGTTGCTTCGCTTT

Liparis_nervosa_JN114602 ATGAC-TCTCGGCAATGGATATCTCGGCTCTTGCATCGATGAAGAGCGCAGCAAAATGCGATACGTGATGC-GAATTGCAGAATCCCGCGAACC-ATCGAGTCTTTGAACGCAAGTTGCGCCCGAGGCCAAC-CGGTCAAGGGCACGTTTTCCTG---GGTGTCA-AGCGTTGCTTCGCTTT

Liparis_nervosa_JN114603 ATGAC-TCTCGGCAATGGATATCTCGGCTCTTGCATCGATGAAGAGCGCAGCAAAATGCGATACGTGATGC-GAATTGCAGAATCCCGCGAACC-ATCGAGTCTTTGAACGCAAGTTGCGCCCGAGGCCAAC-CGGTCAAGGGCACGTTTTCCTG---GGTGTCA-AGCGTTGCTTCGCTTT

Liparis_nervosa_JN114604 ATGAC-TCTCGGCAATGGATATCTCGGCTCTTGCATCGATGAAGAGCGCAGCAAAATGCGATACGTGATGC-GAATTGCAGAATCCCGCGAACC-ATCGAGTCTTTGAACGCAAGTTGCGCCCGAGGCCAAC-CGGTCAAGGGCACGTTTTCCTG---GGTGTCA-AGCGTTGCTTCGCTTT

Liparis_nervosa_KFBG330 ATGAC-TCTCGGCAATGGATATCTCGGCTCTTGCATCGATGAAGAGCGCAGCAAAATGCGATACGTGATGC-GAATTGCAGAATCCCGCGAACC-ATCGAGTCTTTGAACGCAAGTTGCGCCCGAGGCCAAC-CGGTCAAGGGCACGTTTTCCTG---GGTGTCA-AGCGTTGCTTCGCTTC

Liparis_nervosa_KJ459294 ATGAC-TCTCGGCAATGGATATNTCGGCTCTTGCATCGATGAAGAGCGCAGCAAAATGCGATACGTGATGCGGAATTGCAGAATCCCGCGAACC-ATCGAGTCTTTGAACGCAAGTTGCGCCCGAGGCCAAC-CGGTCAAGGGCACGTTTTCCTG---GGTGTCA-AGCGTTGCTTCGCTTT

Liparis_nervosa_KT338746 ATGAC-TCTCGGCAATGGATATCTCGGCTCTTGCATCGATGAAGAGCGCAGCAAAATGCGATACGTGATGC-GAATTGCAGAATCCCGCGAACC-ATCGAGTCTTTGAACGCAAGTTGCGCCCGAGGCCAAC-CGGTCAAGGGCACGTTTTCCTG---GGTGTCA-AGCGTTGCTTCGCTTT

Liparis_nervosa_KT338747 ATGAC-TCTCGGCAATGGATATCTCGGCTCTTGCATCGATGAAGAGCGCAGCAAAATGCGATACGTGATGC-GAATTGCAGAATCCCGCGAACC-ATCGAGTCTTTGAACGCAAGTTGCGCCCGAGGCCAAC-CGGTCAAGGGCACGTTTTCCTG---GGTGTCA-AGCGATGCTTCGCTTT

Liparis_nervosa_SG1233 ATGAC-TCTCGGCAATGGATATCTCGGCTCTTGCATCGATGAAGAGCGCAGCAAAATGCGATACGTGATGC-GAATTGCAGAATCCCGCGAACC-ATCGAGTCTTTGAACGCAAGTTGCGCCCGAGGCCAAC-CGGTCAAGGGCACGTTTTCCTG---GGTGTCA-AGCGTTGCTTCGCTTC

Liparis_nervosa_SG1234 ATGAC-TCTCGGCAATGGATATCTCGGCTCTTGCATCGATGAAGAGCGCAGCAAAATGCGATACGTGATGC-GAATTGCAGAATCCCGCGAACC-ATCGAGTCTTTGAACGCAAGTTGCGCCCGAGGCCAAC-CGGTCAAGGGCACGTTTTCCTG---GGTGTCA-AGCGTTGCTTCGCTTC

Liparis_nervosa_SG1235 ATGAC-TCTCGGCAATGGATATCTCGGCTCTTGCATCGATGAAGAGCGCAGCAAAATGCGATACGTGATGC-GAATTGCAGAATCCCGCGAACC-ATCGAGTCTTTGAACGCAAGTTGCGCCCGAGGCCAAC-CGGTCAAGGGCACGTTTTCCTG---GGTGTCA-AGCGTTGCTTCGCTTC

Liparis_odorata_SG1256 ACGAC-TCTCGGCAATGGATATCTCGGCTCTTGCATCGATGAAGAGCGCAGCAAAATGCGATACGTGATGC-GAATTGCAGAACCCCGCGAACC-ATCGAGTCTTTGAACGCAAGTTGCGCCCGAGGCCAAC-CGGTCAAGGGCACGCTTTCCTG---GGCGTCA-AGCGTTGCATCGCTTT

Liparis_odorata_SG1257 ACGAC-TCTCGGCAATGGATATCTCGGCTCTTGCATCGATGAAGAGCGCAGCAAAATGCGATACGTGATGC-GAATTGCAGAACCCCGCGAACC-ATCGAGTCTTTGAACGCAAGTTGCGCCCGAGGCCAAC-CGGTCAAGGGCACGCTTTCCTG---GGCGTCA-AGCGTTGCATCGCTTT

Liparis_sootenzanensis_KJ021034 ACGAC-TCTCGGCAATGGATATCTCGGCTCTTGCATCGATGAAGAGCGCAGCAAAATGCGATACGTGATGC-GAATTGCAGAATCCCGCGAACC-ATCGAGTCTTTGAACGCAAGTTGCGCCCGAGGCCAAC-CGGTCAAGGGCACGCTTTCCTG---GGTGTCA-AGCGTTGCATCGCTTT

Liparis_sootenzanensis_SG1351 ACGAC-TCTCGGCAATGGATATCTCGGCTCTTGCATCGATGAAGAGCGCAGCAAAATGCGATACGTGATGC-GAATTGCAGAATCCCGCGAACC-ATCGAGTCTTTGAACGCAAGTTGCGCCCGAGGCCAAC-CGGTCAAGGGCACGCTTTCCTG---GGTGTCA-AGCGTTGCATCGCTTT

Liparis_sootenzanensis_SG1352 ACGAC-TCTCGGCAATGGATATCTCGGCTCTTGCATCGATGAAGAGCGCAGCAAAATGCGATACGTGATGC-GAATTGCAGAATCCCGCGAACC-ATCGAGTCTTTGAACGCAAGTTGCGCCCGAGGCCAAC-CGGTCAAGGGCACGCTTTCCTG---GGTGTCA-AGCGTTGCATCGCTTT

Liparis_stricklandiana_KF589873 ACGAC-TCTCGGCAATGGATATCTCGGCTCTTGCATCGATGAAGAGCGCAGCGAAATGCGATACGTGGTGC-GAATTGCAGAATCCCGCGAACC-ATCGAGTCTTTGAACGCAAGTTGCGCCCGAGGCCAAC-CGGCCAAGGGCACGTTTGCCTG---GGCGTCA-AGCGTTGCGTCGCTYC

Liparis_stricklandiana_KFBG124 ACGAC-TCTCGGCAATGGATATCTCGGCTCTTGCATCGATGAAGAGCGCAGCGAAATGCGATACGTGGTGC-GAATTGCAGAATCCCGCGAACC-ATCGAGTCTTTGAACGCAAGTTGCGCCCGAGGCCAAC-CGGCCAAGGGCACGTTTGCCTG---GGCGTCA-AGCGTTGCGTCGCTTC

Liparis_stricklandiana_KFBG818 ACGAC-TCTCGGCAATGGATATCTCGGCTCTTGCATCGATGAAGAGCGCAGCGAAATGCGATACGTGGTGC-GAATTGCAGAATCCCGCGAACC-ATCGAGTCTTTGAACGCAAGTTGCGCCCGAGGCCAAC-CGGCCAAGGGCACGTTTGCCTG---GGCGTCA-AGCGTTGCGTCGCTCC

Liparis_stricklandiana_KJ459298 ACGAC-TCTCGGCAATGGATATCTCGGCTCTTGCATCGATGAAGAGCGCAGCGAAATGCGATACGTGGTGC-GAATTGCAGAATCCCGCGAACC-ATCGAGTCTTTGAACGCAAGTTGCGCCCGAGGCCAAC-CGGCCAAGGGCACGTTTGCCTG---GGCGTCA-AGCGTTGCGTCGCTTC

Liparis_stricklandiana_KY966613 ACGAC-TCTCGGCAATGGATATCTCGGCTCTTGCATCGATGAAGAGCGCAGCGAAATGCGATACGTGGTGC-GAATTGCAGAATCCCGCGAACC-ATCGAGTCTTTGAACGCAAGTTGCGCCCGAGGCCAAC-CGGCCAAGGGCACGTTTGCCTG---GGCGTCA-AGCGTTGCGTCGCTCC

Liparis_stricklandiana_KY966614 ACGAC-TCTCGGCAATGGATATCTCGGCTCTTGCATCGATGAAGAGCGCAGCGAAATGCGATACGTGGTGC-GAATTGCAGAATCCCGCGAACC-ATCGAGTCTTTGAACGCAAGTTGCGCCCGAGGCCAAC-CGGCCAAGGGCACGTTTGCCTG---GGCGTCA-AGCGTTGCGTCGCTTC

Liparis_stricklandiana_PK12091 ACGAC-TCTCGGCAATGGATATCTCGGCTCTTGCATCGATGAAGAGCGCAGCGAAATGCGATACGTGGTGC-GAATTGCAGAATCCCGCGAACC-ATCGAGTCTTTGAACGCAAGTTGCGCCCGAGGCCAAC-CGGCCAAGGGCACGTTTGCCTG---GGCGTCA-AGCGTTGCGTCGCTTC

Liparis_stricklandiana_SG1332 ACGAC-TCTCGGCAATGGATATCTCGGCTCTTGCATCGATGAAGAGCGCAGCGAAATGCGATACGTGGTGC-GAATTGCAGAATCCCGCGAACC-ATCGAGTCTTTGAACGCAAGTTGCGCCCGAGGCCAAC-CGGCCAAGGGCACGTTTGCCTG---GGCGTCA-AGCGTTGCGTCGCTTC

Liparis_stricklandiana_SG1333 ACGAC-TCTCGGCAATGGATATCTCGGCTCTTGCATCGATGAAGAGCGCAGCGAAATGCGATACGTGGTGC-GAATTGCAGAATCCCGCGAACC-ATCGAGTCTTTGAACGCAAGTTGCGCCCGAGGCCAAC-CGGCCAAGGGCACGTTTGCCTG---GGCGTCA-AGCGTTGCGTCGCTYC

Liparis_stricklandiana_SG1337 ACGAC-TCTCGGCAATGGATATCTCGGCTCTTGCATCGATGAAGAGCGCAGCGAAATGCGATACGTGGTGC-GAATTGCAGAATCCCGCGAACC-ATCGAGTCTTTGAACGCAAGTTGCGCCCGAGGCCAAC-CGGCCAAGGGCACGTTTGCCTG---GGCGTCA-AGCGTTGCGTCGCTTC

Liparis_viridiflora_KJ459299 ACGAC-TCTCGGCAATGGATATCTCGGCTCTTGCATCGATGAAGAGCGCAGCGAAATGCGATACGTGGTGC-GAATTGCAGAATCCCGCGAACC-ATCGAGTCTTTGAACGCAAGTTGCGCCCGAGGCCAAC-CGGCCAAGGGCACGTTTGCCTG---GGCGTCA-AGCGTTGCGTCGCTTC

Liparis_viridiflora_KY966615 ACGAC-TCTCGGCAATGGATATCTCGGCTCTTGCATCGATGAAGAGCGCAGCGAAATGCGATACGTGGTGC-GAATTGCAGAATCCCGCGAACC-ATCGAGTCTTTGAACGCAAGTTGCGCCCGAGGCCAAC-CGGCCAAGGGCACGTTTGCCTG---GGCGTCA-AGCGTTGCGTCGCTTC

Liparis_viridiflora_KY966616 ACGAC-TCTCGGCAATGGATATCTCGGCTCTTGCATCGATGAAGAGCGCAGCGAAATGCGATACGTGGTGC-GAATTGCAGAATCCCGCGAACC-ATCGAGTCTTTGAACGCAAGTTGCGCCCGAGGCCAAC-CGGCCAAGGGCACGTTTGCCTG---GGCGTCA-AGCGTTGCGTCGCTTC

Liparis_viridiflora_PK12120 ACGAC-TCTCGGCAATGGATATCTCGGCTCTTGCATCGATGAAGAGCGCAGCGAAATGCGATACGTGGTGC-GAATTGCAGAATCCCGCGAACC-ATCGAGTCTTTGAACGCAAGTTGCGCCCGAGGCCAAC-CGGCCAAGGGCACGTTTGCCTG---GGCGTCA-AGCGTTGCGTCGCTTC

Liparis_viridiflora_SG1308 ACGAC-TCTCGGCAATGGATATCTCGGCTCTTGCATCGATGAAGAGCGCAGCGAAATGCGATACGTGGTGC-GAATTGCAGAATCCCGCGAACC-ATCGAGTCTTTGAACGCAAGTTGCGCCCGAGGCCAAC-CGGCCAAGGGCACGTTTGCCTG---GGCGTCA-AGCGTTGCGTCGCTTC

Liparis_viridiflora_SG1338 ACGAC-TCTCGGCAATGGATATCTCGGCTCTTGCATCGATGAAGAGCGCAGCGAAATGCGATACGTGGTGC-GAATTGCAGAATCCCGCGAACC-ATCGAGTCTTTGAACGCAAGTTGCGCCCGAGGCCAAC-CGGCCAAGGGCACGTTTGCCTG---GGCGTCA-AGCGTTGCGTCGCTTC

Ludisia_discolor_AJ539483 ATGAC-TCTCGGCAATGGATATCTTGGCTCTTGCATCGATGAAGAGCGCAGCGAAATGCGATACGTGGTGT-GAATTGCAGAATCCCGTGAACC-ATCAAATCTTTGAACGCAAGTTGCGCCCGAGGCCATT-TGGCTAAGGGCACGTCCGCCTG---GGCGTCA-AGCATTACATCGCTTC

Ludisia_discolor_EF590781 ----------------------------TCTTGCATCGATGAAGAGCGCAGCGAAATGCGATACGTGGTGT-GAATTGCAGAATCCCGTGAACC-ATCAAATCTTTGAACGCAAGTTGCGCCCGAGGCCATT-TGGCTAAGGGCACGTCCGCCTG---GGCGTCA-AGCATTACATCGCTTC

Ludisia_discolor_EF590782 ---------------------------CTCTTGCATCGATGAAGAGCGCAGCGAAATGCGATACGTGGTGT-GAATTGCAGAATCCCGTGAACC-ATCAAATCTTTGAACGCAAGTTGCGCCCGAGGCCATT-TGGCTAAGGGCACGTCCGCCTG---GGCGTCA-AGCATTACATCGCTTC

Ludisia_discolor_JN166073 ATGAC-TCTCGGCAATGGATATCTTGGCTCTTGCATCGATGAAGAGCGCAGCGAAATGCGATACGTGGTGT-GAATTGCAGAATCCCGTGAACC-ATCAAATCTTTGAACGCAAGTTGCGCCCGAGGCCATT-TGGCTAAGGGCACGTCCGCCTG---GGCGTCA-AGCATTACATCGCTTC

Ludisia_discolor_KR815834 ATGAC-TCTCGGCAATGGATATCTTGGCTCTTGCATCGATGAAGAGCGCAGCGAAATGCGATACGTGGTGT-GAATTGCAGAATCCCGTGAACC-ATCAAATCTTTGAACGCAAGTTGCGCCCGAGGCCATT-TGGCTAAGGGCACGTCCGCCTG---GGCGTCA-AGCATTACATCGCTTC

Ludisia_discolor_KT344102 ATGAC-TCTCGGCAATGGATATCTTGGCTCTTGCATCGATGAAGAGCGCAGCGAAATGCGATACGTGGTGT-GAATTGCAGAATCCCGTGAACC-ATCAAATCTTTGAACGCAAGTTGCGCCCGAGGCCATT-TGGCTAAGGGCACGTCCGCCTG---GGCGTCA-AGC-TTACATCGCTTA

Ludisia_discolor_KY966617 ATGAC-TCTCGGCAATGGATATCTTGGCTCTTGCATCGATGAAGAGCGCAGCGAAATGCGATACGTGGTGT-GAATTGCAGAATCCCGTGAACC-ATCAAATCTTTGAACGCAAGTTGCGCCCGAGGCCATT-TGGCTAAGGGCACGTCCGCCTG---GGCGTCA-AGCATTACATCGCTTC

Ludisia_discolor_SG1236 ATGAC-TCTCGGCAATGGATATCTTGGCTCTTGCATCGATGAAGAGCGCAGCGAAATGCGATACGTGGTGT-GAATTGCAGAATCCCGTGAACC-ATCAAATCTTTGAACGCAAGTTGCGCCCGAGGCCATT-TGGCTAAGGGCACGTCCGCCTG---GGCGTCA-AGCATTACATCGCTTC

Ludisia_discolor_SG1237 ATGAC-TCTCGGCAATGGATATCTTGGCTCTTGCATCGATGAAGAGCGCAGCGAAATGCGATACGTGGTGT-GAATTGCAGAATCCCGTGAACC-ATCAAATCTTTGAACGCAAGTTGCGCCCGAGGCCATT-TGGCTAAGGGCACGTCCGCCTG---GGCGTCA-AGCATTACATCGCTTC

Ludisia_discolor_SG1348 ATGAC-TCTCGGCAATGGATATCTTGGCTCTTGCATCGATGAAGAGCGCAGCGAAATGCGATACGTGGTGT-GAATTGCAGAATCCCGTGAACC-ATCAAATCTTTGAACGCAAGTTGCGCCCGAGGCCATT-TGGCTAAGGGCACGTCCGCCTG---GGCGTCA-AGCATTACATCGCTTC

Nephelaphyllum_tenuiflorum_KF560535 AAGAC-TCTCAACAATGGATATCTTGGGTCTCGCATCGATGAAGAGCGCAGCGAAATGTGATATATGGTGT-GAATTGCAGAATCCCGCGAGCC-ATCGAGTCTTTGAACGCAAGTTGCGTCTGAGGCCAAT-AGGCCAAGGGCACGTCTGCCTG---GGCGTCA-AGCGTTTTGTCGCTCC

Nephelaphyllum_tenuiflorum_KM025159 AAGAC-TCTCAACAATGGATAACTTGGGTCTCGCATCGATGAAGAGCGCAGCGAAATGTGATATATGGTGT-GAATTGCAGAATCCCGCGAGCC-ATCGAGTCTTTGAACGCAAGTTGCGTCTGAGGCCAAT-AGGCCAAGGGCACGTCTGCCTG---GGCGTCA-AGCGTTTTGTTGCTCT

Nephelaphyllum_tenuiflorum_KY966621 AAGAC-TCTCAACAATGGATATCTTGGGTCTCGCATCGATGAAGAGCGCAGCGAAATGTGATATATGGTGT-GAATTGCAGAATCCCGCGAGCC-ATCGAGTCTTTGAACGCAAGTTGCGTCTGAGGCCAAT-AGGCCAAGGGCACGTCTGCCTG---GGCGTCA-AGCGTTTTGTTGCTCT

Nephelaphyllum_tenuiflorum_PK12121 AAGAC-TCTCAACAATGGATATCTTGGGTCTCGCATCGATGAAGAGCGCAGCGAAATGTGATATATGGTGT-GAATTGCAGAATCCCGCGAGCC-ATCGAGTCTTTGAACGCAAGTTGCGTCTGAGGCCAAT-AGGCCAAGGGCACGTCTGCCTG---GGCGTCA-AGCGTTTTGTTGCTCT

Nephelaphyllum_tenuiflorum_PK12122 AAGAC-TCTCAACAATGGATATCTTGGGTCTCGCATCGATGAAGAGCGCAGCGAAATGTGATATATGGTGT-GAATTGCAGAATCCCGCGAGCC-ATCGAGTCTTTGAACGCAAGTTGCGTCTGAGGCCAAT-AGGCCAAGGGCACGTCTGCCTG---GGCGTCA-AGCGTTTTGTTGCTCT

Nephelaphyllum_tenuiflorum_PK12123 AAGAC-TCTCAACAATGGATATCTTGGGTCTCGCATCGATGAAGAGCGCAGCGAAATGTGATATATGGTGT-GAATTGCAGAATCCCGCGAGCC-ATCGAGTCTTTGAACGCAAGTTGCGTCTGAGGCCAAT-AGGCCAAGGGCACGTCTGCCTG---GGCGTCA-AGCGTTTTGTTGCTCT

Nephelaphyllum_tenuiflorum_SG1220 AAGAC-TCTCAACAATGGATATCTTGGGTCTCGCATCGATGAAGAGCGCAGCGAAATGTGATATATGGTGT-GAATTGCAGAATCCCGCGAGCC-ATCGAGTCTTTGAACGCAAGTTGCGTCTGAGGCCAAT-AGGCCAAGGGCACGTCTGCCTG---GGCGTCA-AGCGTTTTGTTGCTCT

Nervilia_plicata_AF324179 ACGAC-TCTCGGCAATGGATATCTCGGCTCTCGCATCGATGAAGAGCGCAGCGAAATGCGATACGTGGTGC-GAATTGCAGAATCCCGTGAACC-ATCGAGTCTTTGAACGCAAGTTGCGCCCGAGGCCCAC-CGGCCGAGGGCACGCCCGCCTG---GGCGTCA-AGCATCACGTCACTCC

Nervilia_plicata_JN114618 ACGAC-TCTCGGCAATGGATATCTCGGCTCTCGCATCGATGAAGAGCGCAGCGAAATGCGATACGTGGTGC-GAATTGCAGAATCCCGTGAACC-ATCGAGTCTTTGAACGCAAGTTGCGCCCGAGGCCCAC-CGGCCGAGGGCACGCCCGCCTG---GGCGTCA-AGCACCGCGTCACTCC

Nervilia_plicata_JN114619 ACGAC-TCTCGGCAATGGATATCTCGGCTCTCGCATCGATGAAGAGCGCAGCGAAATGCGATACGTGGTGC-GAATTGCAGAATCCCGTGAACC-ATCGAGTCTTTGAACGCAAGTTGCGCCCGAGGCCCAC-CGGCCGAGGGCACGCCCGCCTG---GGCGTCA-AGCACCGCGTCACTCC

Nervilia_plicata_JN114620 ACGAC-TCTCGGCAATGGATATCTCGGCTCTCGCATCGATGAAGAGCGCAGCGAAATGCGATACGTGGTGC-GAATTGCAGAATCCCGTGAACC-ATCGAGTCTTTGAACGCAAGTTGCGCCCGAGGCCCAC-CGGCCGAGGGCACGCCCGCCTG---GGCGTCA-AGCACCGCGTCACTCC

Nervilia_plicata_MG452049 ACGAC-TCTCGGCAATGGATATCTCGGCTCTCGCATCGATGAAGAGCGCAGCGAAATGCGATACGTGGTGC-GAATTGCAGAATCCCGTGAACC-ATCGAGTCTTTGAACGCAAGTTGCGCCCGAGGCCCAC-CGGCCGAGGGCACGCCCGCCTG---GGCGTCA-AGCACCGCGTCACTCC

Nervilia_plicata_SG1143 ACGAC-TCTCGGCAATGGATATCTCGGCTCTCGCATCGATGAAGAGCGCAGCGAAATGCGATACGTGGTGC-GAATTGCAGAATCCCGTGAACC-ATCGAGTCTTTGAACGCAAGTTGCGCCCGAGGCCCAC-CGGCCGAGGGCACGCCCGCCTG---GGCGTCA-AGCACCGCATCACTCC

Nervilia_plicata_SG1277 ACGAC-TCTCGGCAATGGATATCTCGGCTCTCGCATCGATGAAGAGCGCAGCGAAATGCGATACGTGGTGC-GAATTGCAGAATCCCGTGAACC-ATCGAGTCTTTGAACGCAAGTTGCGCCCGAGGCCCAC-CGGCCGAGGGCACGCCCGCCTG---GGCGTCA-AGCACCGCATCACTCC

Neuwiedia_zollingeri_var_singapureana_PK12124 ATGAC-TCTCGACAACGGATATCTTGGCTCTTGCATCGATGAAGAACGCAGCGAAATGTGATATATGGTGT-GAATTGCAGAATCCCGTGAACC-ATCGAGTACTTGAACGCAAGTTGCGCCTGAGGCCAAG-TGGTTGATGGCACACCTGCCTGGTTGTCGTCG-TATGTCGTCTCGCTCC

Neuwiedia_zollingeri_var_singapureana_SG1268 ATGAC-TCTCGACAACGGATATCTTGGCTCTTGCATCGATGAAGAACGCAGCGAAATGTGATATATGGTGT-GAATTGCAGAATCCCGTGAACC-ATCGAGTACTTGAACGCAAGTTGCGCCTGAGGCCAAG-TGGTTGATGGCACACCTGCCTGGTTGTCGTCG-TATGTCGTCTCGCTCC

Neuwiedia_zollingeri_var_singapureana_SG1269 ATGAC-TCTCGACAACGGATATCTTGGCTCTTGCATCGATGAAGAACGCAGCGAAATGTGATATATGGTGT-GAATTGCAGAATCCCGTGAACC-ATCGAGTACTTGAACGCAAGTTGCGCCTGAGGCCAAG-TGGTTGATGGCACACCTGCCTGGTTGTCGTCG-TATGTCGTCTCGCTCC

Neuwiedia_zollingeri_var_singapureana_SG1340 ATGAC-TCTCGACAACGGATATCTTGGCTCTTGCATCGATGAAGAACGCAGCGAAATGTGATATATGGTGT-GAATTGCAGAATCCCGTGAACC-ATCGAGTACTTGAACGCAAGTTGCGCCTGAGGCCAAG-TGGTTGATGGCACACCTGCCTGGTTGTCGTCG-TATGTCGTCTCGCTCC

Neuwiedia_zollingeri_var_singapureana_JF796932 ATGAC-TCTCGACAACGGATATCTTGGCTCTTGCATCGATGAAGAACGCAGCGAAATGTGATATATGGTGT-GAATTGCAGAATCCCGTGAACC-ATCGAGTACTTGAACGCAAGTTGCGCCTGAGGCCAAG-TGGTTGATGGCACACCTGCCTGGTTGTCGTCG-TATGTCGTTTC-----

Neuwiedia_zollingeri_var_singapureana_KY966622 ATGAC-TCTCGACAACGGATATCTTGGCTCTTGCATCGATGAAGAACGCAGCGAAATGTGATATATGGTGT-GAATTGCAGAATCCCGTGAACC-ATCGAGTACTTGAACGCAAGTTGCGCCTGAGGCCAAG-TGGTTGATGGCACACCTGCCTGGTTGTCGTCG-TATGTCGTCTC-----

Pachystoma_pubescens_PK12107 ACGAC-TCTCGGCAATGGATATCTCGGCTCTCGCATCGATGAAGAGCGCAGCAAAATGCGATACGTGGTGC-GAATTGCAGAATCCCGCGAACC-ATCGAGTCTTTGAACGCAAGTTGCGCCCGAGGCCAAT-CGGCCAAGGGCACGTCCGCCTG---GGCGTCA-AGCGTCGCGTCGCTCC

Pachystoma_pubescens_PK12108 ACGAC-TCTCGGCAATGGATATCTCGGCTCTCGCATCGATGAAGAGCGCAGCAAAATGCGATACGTGGTGC-GAATTGCAGAATCCCGCGAACC-ATCGAGTCTTTGAACGCAAGTTGCGCCCGAGGCCAAT-CGGCCAAGGGCACGTCCGCCTG---GGCGTCA-AGCGTCGCGTCGCTCC

Paphiopedilum_purpuratum_AJ564364 ACAAC-TCTCAGCAACGGATATCTCGGCTCTTGCATCGATGAAGAACGCAGCGAAATGCGATAAATGGTGT-GAATTGAAGAATCCCGTGAACC-ATCGAGTCTTTGAACGCAAGTTGCGCCCGAGGCCATC-AGGCCAAGGGCACGCCTGCCTG---GGCATTG-CGAGTCATATCTCTCC

Paphiopedilum_purpuratum_EF156131 ACAAC-TCTCAGCAACGGATATCTCGGCTCTTGCATCGATGAAGAACGCAGCGAAATGCGATAAATGGTGT-GAATTGCAGAATCCCGTGAACC-ATCGAGTCTTTGAACGCAAGTTACGCCCGAGGCCATC-AGGCCAAGGGCACGCCTGCCTG---GGCATTG-CGAGTCATATCTCTCC

Paphiopedilum_purpuratum_FJ899756 ACAAC-TCTCAGCAACGGATATCTCGGCTCTTGCATCGATGAAGAACGCAGCGAAATGCGATAAATGGTGT-GAATTGCAGAATCCCGTGAACC-ATCGAGTCTTTGAACGCAAGTTGCGCCCGAGGCCATC-AGGCCAAGGGCACGCCTGCCTG---GGCATTG-CGAGTCATATCTCTCC

Paphiopedilum_purpuratum_JX088564 ACAAC-TCTCAGCAACGGATATCTCGGCTCTTGCATCGATGAAGAACGCAGCGAAATGCGATAAATGGTGT-GAATTGCAGAATCCCGTGAACC-ATCGAGTCTTTGAACGCAAGTTGCGCCCGAGGCCATC-AGGCCAAGGGCACGCCTGCCTG---GGCATTG-CGAGTCATATCTCTCC

Paphiopedilum_purpuratum_KX931030 ACAAC-TCTCAGCAACGGATATCTCGGCTCTTGCATCGATGAAGAACGCAGCGAAATGCGATAAATGGTGT-GAATTGCAGAATCCCGTGAACC-ATCGAGTCTTTGAACGCAAGTTGCGCCCGAGGCCATC-AGGCCAAGGGCACGCCTGCCTG---GGCATTG-CGAGTCATATCTCTCC

Paphiopedilum_purpuratum_PK12075 ACAAC-TCTCAGCAACGGATATCTCGGCTCTTGCATCGATGAAGAACGCAGCGAAATGCGATAAATGGTGT-GAATTGCAGAATCCCGTGAACC-ATCGAGTCTTTGAACGCAAGTTGCGCCCGAGGCCATC-AGGCCAAGGGCACGCCTGCCTG---GGCATTG-CGAGTCATATCTCTCC

Paphiopedilum_purpuratum_PK12082A ACAAC-TCTCAGCAACGGATATCTCGGCTCTTGCATCGATGAAGAACGCAGCGAAATGCGATAAATGGTGT-GAATTGCAGAATCCCGTGAACC-ATCGAGTCTTTGAACGCAAGTTGCGCCCGAGGCCATC-AGGCCAAGGGCACGCCTGCCTG---GGCATTG-CGAGTCATATCTCTCC

Paphiopedilum_purpuratum_PK12083C ACAAC-TCTCAGCAACGGATATCTCGGCTCTTGCATCGATGAAGAACGCAGCGAAATGCGATAAATGGTGT-GAATTGCAGAATCCCGTGAACC-ATCGAGTCTTTGAACGCAAGTTGCGCCCGAGGCCATC-AGGCCAAGGGCACGCCTGCCTG---GGCATTG-CGAGTCATATCTCTCC

Paphiopedilum_purpuratum_SG1149 ACAAC-TCTCAGCAACGGATATCTCGGCTCTTGCATCGATGAAGAACGCAGCGAAATGCGATAAATGGTGT-GAATTGCAGAATCCCGTGAACC-ATCGAGTCTTTGAACGCAAGTTGCGCCCGAGGCCATC-AGGCCAAGGGCACGCCTGCCTG---GGCATTG-CGAGTCATATCTCTCC

Paphiopedilum_purpuratum_Z78440 ACAAC-TCTCAGCAACGGATATCTCAGCTCTTGCATCGTTGAAGAACCCACCGAAATGCGATAAATGGTGT-GAATTGCAGAATCCCGTGAACC-ATCGAGTCTTTGAACGCAAGTTGCGCCCGAGGCCATC-AGGCCAAGGGCACGCCTGCCTG---GGCATTG-CGAGTCATATCTCTCC

Pecteilis_susannae_MF944351 AGGAC-TCTCGGCAATGGATATCTTGGCTCTTGCATCGATGAAGAGCGCAGCGAAATGCGATACGTGGTGC-GAATTGCAGAATCCCGTGAACC-ATCGAGTTTTTGAACGCAAGTTGCGCCTGAGGCCACC-TGGCCAAGGGCACGTCCACCTG---GGCGTCA-AGCATTAAATCGCTCT

Pecteilis_susannae_MF944352 AGGAC-TCTCGGCAATGGATATCTTGGCTCTTGCATCGATGAAGAGCGCAGCGAAATGCGATACGTGGTGC-GAATTGCAGAATCCCGTGAACC-ATCGAGTTTTTGAACGCAAGTTGCGCCTGAGGCCACC-TGGCCAAGGGCACGTCCACCTG---GGCGTCA-AGCATTAAATCGCTCT

Pecteilis_susannae_PK12051 AGGAC-TCTCGGCAATGGATATCTTGGCTCTTGCATCGATGAAGAGCGCAGCGAAATGCGATACGTGGTGC-GAATTGCAGAATCCCGTGAACC-ATCGAGTTTTTGAACGCAAGTTGCGCCTGAGGCCACC-TGGCCAAGGGCACGTCCACCTG---GGCGTCA-AGCATTAAATCGCTCT

Pecteilis_susannae_PK12136 AGGAC-TCTCGGCAATGGATATCTTGGCTCTTGCATCGATGAAGAGCGCAGCGAAATGCGATACGTGGTGC-GAATTGCAGAATCCCGTGAACC-ATCGAGTTTTTGAACGCAAGTTGCGCCTGAGGCCACC-TGGCCAAGGGCACGTCCACCTG---GGCGTCA-AGCATTAAATCGCTCT

Pecteilis_susannae_PK12154 AGGAC-TCTCGGCAATGGATATCTTGGCTCTTGCATCGATGAAGAGCGCAGCGAAATGCGATACGTGGTGC-GAATTGCAGAATCCCGTGAACC-ATCGAGTTTTTGAACGCAAGTTGCGCCTGAGGCCACC-TGGCCAAGGGCACGTCCACCTG---GGCGTCA-AGCATTAAATCGCTCT

Pecteilis_susannae_SG1292 AGGAC-TCTCGGCAATGGATATCTTGGCTCTTGCATCGATGAAGAGCGCAGCGAAATGCGATACGTGGTGC-GAATTGCAGAATCCCGTGAACC-ATCGAGTTTTTGAACGCAAGTTGCGCCTGAGGCCACC-TGGCCAAGGGCACGTCCACCTG---GGCGTCA-AGCATTAAATCGCTCT

Peristylus_calcaratus_PK12052 AGGAC-TCTCGGCAATGGATATCTTGGCTCTTGCATCGATGAAGAGCGCAGCGAAATGCGATACGTGGTGC-GAATTGCAGAATCCCGTGAACC-ATCGAGTATTTGAACGCAAGTTGCGCCCGAGGCCAGC-TGGTCGAGGGCACGTCCGCCTG---GGCGTCA-AGCATTGAATCGCCCC

Peristylus_calcaratus_PK12053 AGGAC-TCTCGGCAATGGATATCTTGGCTCTTGCATCGATGAAGAGCGCAGCGAAATGCGATACGTGGTGC-GAATTGCAGAATCCCGTGAACC-ATCGAGTATTTGAACGCAAGTTGCGCCCGAGGCCAGC-TGGTCGAGGGCACGTCCGCCTG---GGCGTCA-AGCATTGAATCGCCCC

Peristylus_calcaratus_SG1303 AGGAC-TCTCGGCAATGGATATCTTGGCTCTTGCATCGATGAAGAGCGCAGCGAAATGCGATACGTGGTGC-GAATTGCAGAATCCCGTGAACC-ATCGAGTATTTGAACGCAAGTTGCGCCCGAGGCCAGC-TGGTCGAGGGCACGTCCGCCTG---GGCGTCA-AGCATTGAATCGCCCC

Peristylus_densus_SG1258 AGGAC-TCTCGGCAATGGATATCTTGGCTCTCGCATCGATGAAGAGCGCAGCGAAATGCGATACGTGGTGC-GAATTGCAGAATCCCGTGAACC-ATCGAGTATTTGAACGCAAGTTGCGCCCGAGGCCAGC-TGGCCGAGGGCACGTCCGACTG---GGCGTCA-AGCATTGAATCGCCCC

Peristylus_densus_SG1260 AGGAC-TCTCGGCAATGGATATCTTGGCTCTCGCATCGATGAAGAGCGCAGCGAAATGCGATACGTGGTGC-GAATTGCAGAATCCCGTGAACC-ATCGAGTATTTGAACGCAAGTTGCGCCCGAGGCCAGC-TGGCCGAGGGCACGTCCGACTG---GGCGTCA-AGCATTGAATCGCCCC

Peristylus_goodyeroides_MF944361 AGGAC-TCTCGGCAATGGATATCTTGGCTCTCGCATCGATGAAGAGCGCAGCGAAATGCGATACGTGGTGC-GAATTGCAGAATCCCGTGAACC-ATCGAGTTTTTGAACGCAAGTTGCGCCCGAGGCCAGCTTGGCCGAGGGCACGTCCGCCTG---GGCGTCA-AGCATTGAATCGCCCC

Peristylus_goodyeroides_MF944362 AGGAC-TCTCGGCAATGGATATCTTGGCTCTCGCATCGATGAAGAGCGCAGCGAAATGCGATACGTGGTGC-GAATTGCAGAATCCCGTGAACC-ATCGAGTTTTTGAACGCAAGTTGCGCCCGAGGCCAGCTTGGCCGAGGGCACGTCCGCCTG---GGCGTCA-AGCATTGAATCGCCCC

Peristylus_intrudens_PK12050 AGGAC-TCTCGGCAATGGATATCTTGGCTCTCGCATCGATGAAGAGCGCAGCGAAATGCGATACGTGGTGC-GAATTGCAGAATCCCGTGAACC-ATCGAGTTTTTGAACGCAAGTTGCGCCCGAGGCCAGC-TGGCCGAGGGCACGTCCGCCTG---GGCGTCA-AGCATTGAATCGCCCC

Peristylus_intrudens_PK12056 AGGAC-TCTCGGCAATGGATATCTTGGCTCTCGCATCGATGAAGAGCGCAGCGAAATGCGATACGTGGTGC-GAATTGCAGAATCCCGTGAACC-ATCGAGTTTTTGAACGCAAGTTGCGCCCGAGGCCAGC-TGGCCGAGGGCACGTCCGCCTG---GGCGTCA-AGCGTTGAATCGCCCC

Peristylus_intrudens_SG1298 AGGAC-TCTCGGCAATGGATATCTTGGCTCTCGCATCGATGAAGAGCGCAGCGAAATGCGATACGTGGTGC-GAATTGCAGAATCCCGTGAACC-ATCGAGTTTTTGAACGCAAGTTGCGCCCGAGGCCAGC-TGGCCGAGGGCACGTCCGCCTG---GGCGTCA-AGCATTGAATCGCCCC

Peristylus_lacertifer_MF944365 AGGAC-TCTCGGCAATGGATATCTTGGCTCTCGCATCGATGAAGAGCGCAGCGAAATGCGATACGTGGTGC-GAATTGCAGAATCCCGTGAACC-ATCGAGTTTTTGAACGCAAGTTGCGCCCGAGGCCAGC-TGGCCGAGGGCACGTCCGCCTG---GGCGTCA-AGCATTGAATCGCCCC

Peristylus_lacertifer_MF944366 AGGAC-TCTCGGCAATGGATATCTTGGCTCTCGCATCGATGAAGAGCGCAGCGAAATGCGATACGTGGTGC-GAATTGCAGAATCCCGTGAACC-ATCGAGTTTTTGAACGCAAGTTGCGCCCGAGGCCAGC-TGGCCGAGGGCACGTCCGCCTG---GGCGTCA-AGCATTGAATCGCCCC

Peristylus_lacertifer_PK12149 AGGAC-TCTCGGCAATGGATATCTTGGCTCTCGCATCGATGAAGAGCGCAGCGAAATGCGATACGTGGTGC-GAATTGCAGAATCCCGTGAACC-ATCGAGTTTTTGAACGCAAGTTGCGCCCGAGGCCAGC-TGGCCGAGGGCACGTCCGCCTG---GGCGTCA-AGCATTGAATCGCCCC

Peristylus_lacertifer_PK12163 AGGAC-TCTCGGCAATGGATATCTTGGCTCTCGCATCGATGAAGAGCGCAGCGAAATGCGATACGTGGTGC-GAATTGCAGAATCCCGTGAACC-ATCGAGTTTTTGAACGCAAGTTGCGCCCGAGGCCAGC-TGGCCGAGGGCACGTCCGCCTG---GGCGTCA-AGCATTGAATCGCCCC

Peristylus_lacertifer_SG1006 AGGAC-TCTCGGCAATGGATATCTTGGCTCTCGCATCGATGAAGAGCGCAGCGAAATGCGATACGTGGTGC-GAATTGCAGAATCCCGTGAACC-ATCGAGTTTTTGAACGCAAGTTGCGCCCGAGGCCAGC-TGGCCGAGGGCACGTCCGCCTG---GGCGTCA-AGCATTGAATCGCCCC

Peristylus_tentaculatus_KJ460035 AGGAC-TCTCGGCAATGGATATCTTGGCTCTCGCATCGATGAAGAGCGCAGCGAAATGCGATACGTGGTGC-GAATTGCAGAATCCCGTGAACC-ATCGAGTATTTGAACGCAAGTTGCGCCCGAGGCCAGC-TGGCCGAGGGCACGTCCGCCTG---GGCGTCA-AGCATTGAATCGCCCC

Persitylus_tentaculatus_PK12062 AGGAC-TCTCGGCAATGGATATCTTGGCTCTCGCATCGATGAAGAGCGCAGCGAAATGCGATACGTGGTGC-GAATTGCAGAATCCCGTGAACC-ATCGAGTTTTTGAACGCAAGTTGCGCCCGAGGCCAGC-TGGCCGAGGGCACGTCCGCCTG---GGCGTCA-AGCATTGAATCGCCCC

Persitylus_tentaculatus_PK12171 AGGAC-TCTCGGCAATGGATATCTTGGCTCTCGCATCGATGAAGAGCGCAGCGAAATGCGATACGTGGTGC-GAATTGCAGAATCCCGTGAACC-ATCGAGTTTTTGAACGCAAGTTGCGCCCGAGGCCAGC-TGGCCGAGGGCACGTCCGCCTG---GGCGTCA-AGCATTGAATCGCCCC

Persitylus_tentaculatus_SG1007 AGGAC-TCTCGGCAATGGATATCTTGGCTCTCGCATCGATGAAGAGCGCAGCGAAATGCGATACGTGGTGC-GAATTGCAGAATCCCGTGAACC-ATCGAGTTTTTGAACGCAAGTTGCGCCCGAGGCCAGC-TGGCCGAGGGCACGTCCGCCTG---GGCGTCA-AGCATTGAATCGCCCC

Phaius_tancarvilleae_AB222032 ATGAC-TCTCGGCAATGGATATCTCGGCTCTCGCATCGATGAAGAGCGCAGCGAAATGCGATACGTGGTGC-GAATTGCAGAATCCCGCGAACC-ATCGAGTCTTTGAACGCAAGTTGCGCCCGAGGTCAAC-CGGCCAAGGGCGCGTCTGCCTG---GGCGTCA-AGCGTTGCATCGCTCT

Phaius_tancarvilleae_AB239286 ATGAC-TCTCGGCAATGGATATCTCGGCTCTCGCATCGATGAAGAGCGCAGCGAAATGCGATACGTGGTGC-GAATTGCAGAATCCCGCGAACC-ATCGAGTCTTTGAACGCAAGTTGCGCCCGAGGTCAAC-CGGCCAAGGGCACGTCTGCCTG---GGCGTCA-AGCGTTGCATCGCTCT

Phaius_tancarvilleae_AB239287 ATGAC-TCTCGGCAATGGATATCTCGGCTCTCGCATCGATGAAGAGCGCAGCGAAATGCGATACGTGGTGC-GAATTGCAGAATCCCGCGAACC-ATCGAGTCTTTGAACGCAAGTTGCGCCCGAGGTCAAC-CGGCCAAGGGCACGTCTGCCTG---GGCGTCA-AGCGTTGCATCGCTCT

Phaius_tancarvilleae_AB239288 ATGAC-TCTCGGCAATGGATATCTCGGCTCTCGCATCGATGAAGAGCGCAGCGAAATGCGATACGTGGTGC-GAATTGCAGAATCCCGCGAACC-ATCGAGTCTTTGAACGCAAGTTGCGCCCGAGGTCAAC-CGGCCAAGGGCACGTCTGCCTG---GGCGTCA-AGCGTTGCATCGCTCT

Phaius_tancarvilleae_AB239289 ATGAC-TCTCGGCAATGGATATCTCGGCTCTCGCATCGATGAAGAGCGCAGCGAAATGCGATACGTGGTGC-GAATTGCAGAATCCCGCGAACC-ATCGAGTCTTTGAACGCAAGTTGCGCCCGAGGTCAAC-CGGCCAAGGGCACGTCTGCCTG---GGCGTCA-AGCGTTGCATCGCTCT

Phaius_tancarvilleae_KF560503 ATGAC-TCTCGGCAATGGATATCTCGGCTCTCGCATCGATGAAGAGCGCAGCGAAATGCGATACGTGGTGC-GAATTGCAGAATCCCGCGAACC-ATCGAGTCTTTGAACGCAAGTTGCGCCCGAGGTCAAC-CGGCCAAGGGCACGTCTGCCTG---GGCGTCA-AGCGTTGCATCGCTCT

Phaius_tancarvilleae_KF560531 ATGAC-TCTCGGCAATGGATATCTCGGCTCTCGCATCGATGAAGAGCGCAGCGAAATGCGATACGTGGTGC-GAATTGCAGAATCCCGCGAACC-ATCGAGTCTTTGAACGCAAGTTGCGCCCGAGGTCAAC-CGGCCAAGGGCACGTCTGCCTG---GGCGTCA-AGCGTTGCATCGCTCT

Phaius_tancarvilleae_KM025161 ATGAC-TCTCGGCAATGGATATCTCGGCTCTCGCATCGATGAAGAGCGCAGCGAAATGCGATACGTGGTGC-GAATTGCAGAATCCCGCGAACC-ATCGAGTCTTTGAACGCAAGTTGCGCCCGAGGTCAAC-CGGCCAAGGGCACGTCTGCCTG---GGCGTCA-AGCGTTGCATCGCTCT

Phaius_tancarvilleae_KY966645 ATGAC-TCTCGGCAATGGATATCTCGGCTCTCGCATCGATGAAGAGCGCAGCGAAATGCGATACGTGGTGC-GAATTGCAGAATCCCGCGAACC-ATCGAGTCTTTGAACGCAAGTTGCGCCCGAGGTCAAC-CGGCCAAGGGCACGTCTGCCTG---GGCGTCA-AGCGTTGCATCGCTCT

Phaius_tancarvilleae_MG869015 ATGAC-TCTCGGCAATGGATATCTCGGCTCTCGCATCGATGAAGAGCGCAGCGAAATGCGATACGTGGTGC-GAATTGCAGAATCCCGCGAACC-ATCGAGTCTTTGAACGCAAGTTGCGCCCGAGGTCAAC-CGGCCAAGGGCACGTCTGCCTG---GGCGTCA-AGCGTTGCATCGCTCT

Phaius_tankervilleae_PK12084 ATGAC-TCTCGGCAATGGATATCTCGGCTCTCGCATCGATGAAGAGCGCAGCGAAATGCGATACGTGGTGC-GAATTGCAGAATCCCGCGAACC-ATCGAGTCTTTGAACGCAAGTTGCGCCCGAGGTCAAC-CGGCCAAGGGCACGTCTGCCTG---GGCGTCA-AGCGTTGCATCGCTCT

Phaius_tankervilleae_PK12085 ATGAC-TCTCGGCAATGGATATCTCGGCTCTCGCATCGATGAAGAGCGCAGCGAAATGCGATACGTGGTGC-GAATTGCAGAATCCCGCGAACC-ATCGAGTCTTTGAACGCAAGTTGCGCCCGAGGTCAAC-CGGCCAAGGGCACGTCTGCCTG---GGCGTCA-AGCGTTGCATCGCTCT

Phaius_tankervilleae_PK12099 ATGAC-TCTCGGCAATGGATATCTCGGCTCTCGCATCGATGAAGAGCGCAGCGAAATGCGATACGTGGTGC-GAATTGCAGAATCCCGCGAACC-ATCGAGTCTTTGAACGCAAGTTGCGCCCGAGGTCAAC-CGGCCAAGGGCACGTCTGCCTG---GGCGTCA-AGCGTTGCATCGCTCT

Phaius_tankervilleae_PK12100 ATGAC-TCTCGGCAATGGATATCTCGGCTCTCGCATCGATGAAGAGCGCAGCGAAATGCGATACGTGGTGC-GAATTGCAGAATCCCGCGAACC-ATCGAGTCTTTGAACGCAAGTTGCGCCCGAGGTCAAC-CGGCCAAGGGCACGTCTGCCTG---GGCGTCA-AGCGTTGCATCGCTCT

Phaius_wallichii_KF560532 ATGAC-TCTCGGCAATGGATATCTCGGCTCTCGCATCGATGAAGAGCGCAGCGAAATGCGATACGTGGTGC-GAATTGCAGAATCCCGCGAACC-ATCGAGTCTTTGAACGCAAGTTGCGCCCGAGGTCAAC-CGGCCAAGGGCACGTCTGCCTG---GGCGTCA-AGCGTTGCATCGCTCT

Phaius_wallichii_KY966646 ATGAC-TCTCGGCAATGGATATCTCGGCTCTCGCATCGATGAAAAGCGCAGCGAAATGCGATACGTGGTGC-GAATTGCAGAATCCCGCGAACC-ATCGAGTCTTTGAACGCAAGTTGCGCCCGAGGTCAAC-CGGCCAAGGGCACGTCTGCCTG---GGCGTCA-AGCGTTGCATCGCTCT

Platanthera_minor_KJ460069 AGGAC-TCTCGGCAATGGATATCTTGGCTCTCGCATCGATGAAGAGCGCAGCGAAATGCGATACGTGGTGC-GAATTGCAGAATCCCGTGAACC-ATCGAGTTTTTGAACGCAAGTTGCGCCTGAGGCCAGC-TGGCCAAGGGCACGTCCGCCTG---GGCGTCA-AGCATTAAATCGCTCC

Platanthera_minor_KJ460079 AGGAC-TCTCGGCAATGGATATCTTGGCTCTCGCATCGATGAAGAGCGCAGCGAAATGCGATACGTGGTGC-GAATTGCAGAATCCCGTGAACC-ATCGAGTTTTTGAACGCAAGTTGCGCCTGAGGCCAGC-TGGCCAAGGGCACGTCCGCCTG---GGCGTCA-AGCATTAAATCGCTCC

Platanthera_minor_PK12030 AGGGC-TCTCGGCAATGGATATCTTGGCTCTCGCATCGATGAAGAGCGCAGCGAAATGCGATACGTGGTGC-GAATTGCAGAATCCCGTGAACC-ATCGAGTTTTTGAACGCAAGTTGCGCCTGAGGCCAGC-TGGCCAAGGGCACGTCCGCCTG---GGCGTCA-TGCATTAAATCGCTCC

Platanthera_minor_SG1154 AGGGC-TCTCGGCAATGGATATCTTGGCTCTCGCATCGATGAAGAGCGCAGCGAAATGCGATACGTGGTGC-GAATTGCAGAATCCCGTGAACC-ATCGAGTTTTTGAACGCAAGTTGCGCCTGAGGCCAGC-TGGCCAAGGGCACGTCCGCCTG---GGCGTCA-TGCATTAAATCGCTCC

Platanthera_minor_SG1223 AGGGC-TCTCGGCAATGGATATCTTGGCTCTCGCATCGATGAAGAGCGCAGCGAAATGCGATACGTGGTGC-GAATTGCAGAATCCCGTGAACC-ATCGAGTTTTTGAACGCAAGTTGCGCCTGAGGCCAGC-TGGCCAAGGGCACGTCCGCCTG---GGCGTCA-TGCATTAAATCGCTCC

Platanthera_minor_SG1224 AGGGC-TCTCGGCAATGGATATCTTGGCTCTCGCATCGATGAAGAGCGCAGCGAAATGCGATACGTGGTGC-GAATTGCAGAATCCCGTGAACC-ATCGAGTTTTTGAACGCAAGTTGCGCCTGAGGCCAGC-TGGCCAAGGGCACGTCCGCCTG---GGCGTCA-TGCATTAAATCGCTCC

Platanthera_minor_SG1238 AGGGC-TCTCGGCAATGGATATCTTGGCTCTCGCATCGATGAAGAGCGCAGCGAAATGCGATACGTGGTGC-GAATTGCAGAATCCCGTGAACC-ATCGAGTTTTTGAACGCAAGTTGCGCCTGAGGCCAGC-TGGCCAAGGGCACGTCCGCCTG---GGCGTCA-TGCATTAAATCGCTCC

Porpax_pusilla_KY239239 ACGAC-TCTCGGCAATGGATATCTCGGCTCTTGCATCGATGAAGAGCGCAGCGAAATGCGATACGTGGTGC-GAATTGCAGAATCCCGTGAACC-ATCGAGTCTTTGAACGCAAGTTGCGCCCGAGGCCAAC-CGGCTGAGGGCACGTCTGCCTG---GGCGTCA-AACGTTGCGTCGCTCT

Porpax_pusilla_PK12071 ACGAC-TCTCGGCAATGGATATCTCGGCTCTTGCATCGATGAAGAGCGCAGCGAAATGCGATACGTGGTGC-GAATTGCAGAATCCCGTGAACC-ATCGAGTCTTTGAACGCAAGTTGCGCCCGAGGCCAAC-CGGCTGAGGGCACGTCTGCCTG---GGCGTCA-AACGTCGCGTCGCTCT

Porpax_pusilla_PK12072 ACGAC-TCTCGGCAATGGATATCTCGGCTCTTGCATCGATGAAGAGCGCAGCGAAATGCGATACGTGGTGC-GAATTGCAGAATCCCGTGAACC-ATCGAGTCTTTGAACGCAAGTTGCGCCCGAGGCCAAC-CGGCTGAGGGCACGTCTGCCTG---GGCGTCA-AACGTCGCGTCGCTCT

Porpax_pusilla_PK12125 ACGAC-TCTCGGCAATGGATATCTCGGCTCTTGCATCGATGAAGAGCGCAGCGAAATGCGATACGTGGTGC-GAATTGCAGAATCCCGTGAACC-ATCGAGTCTTTGAACGCAAGTTGCGCCCGAGGCCAAC-CGGCTGAGGGCACGTCTGCCTG---GGCGTCA-AACGTTGCGTCGCTCT

Porpax_pusilla_SG1334 ACGAC-TCTCGGCAATGGATATCTCGGCTCTTGCATCGATGAAGAGCGCAGCGAAATGCGATACGTGGTGC-GAATTGCAGAATCCCGTGAACC-ATCGAGTCTTTGAACGCAAGTTGCGCCCGAGGCCAAC-CGGCTGAGGGCACGTCTGCCTG---GGCGTCA-AACGTTGCGTCGCTCT

Rhomboda_abbreviata_KT344110 ATGAC-TCTCGACAATGGATATCTTGGCTCTTGCATCGATGAAGAGCGCAGCGAAATGCGATACGTGGTGT-GAATTGCAGAATCCCGTGAACC-ATCAAATATTTGAACGCAAGTTGCGCCCGAGGCCAAT-TGGCTAAGGGCACGTCCGCCTG---GGCGTCA-AGC-TTACATCGCTTA

Rhomboda_abbreviata_KY966662 ATGAC-TCTCGACAATGGATATCTTGGCTCTTGCATCGATGAAGAGCGCAGCGAAATGCGATACGTGGTGT-GAATTGCAGAATCCCGTGAACC-ATCAAATATTTGAACGCAAGTTGCGCCCGAGGCCAAT-TGGCTAAGGGCACGTCCGCCTG---GGCGTCA-AGCATTACATCGCTTC

Rhomboda_abbreviata_PK12166 ATGAC-TCTCGACAATGGATATCTTGGCTCTTGCATCGATGAAGAGCGCAGCGAAATGCGATACGTGGTGT-GAATTGCAGAATCCCGTGAACC-ATCAAATATTTGAACGCAAGTTGCGCCCGAGGCCAAT-TGGCTAAGGGCACGTCCGCCTG---GGCGTCA-AGCATTACATCGCTTC

Rhomboda_abbreviata_PK12175 ATGAC-TCTCGACAATGGATATCTTGGCTCTTGCATCGATGAAGAGCGCAGCGAAATGCGATACGTGGTGT-GAATTGCAGAATCCCGTGAACC-ATCAAATATTTGAACGCAAGTTGCGCCCGAGGCCAAT-TGGCTAAGGGCACGTCCGCCTG---GGCGTCA-AGCATTACATCGCTTC

Rhomboda_abbreviata_SG1203 ATGAC-TCTCGACAATGGATATCTTGGCTCTTGCATCGATGAAGAGCGCAGCGAAATGCGATACGTGGTGT-GAATTGCAGAATCCCGTGAACC-ATCAAATATTTGAACGCAAGTTGCGCCCGAGGCCAAT-TGGCTAAGGGCACGTCCGCCTG---GGCGTCA-AGCATTACATCGCTTC

Robiquetia_succisa_KJ733444 ACGAC-TCTCGACAATGGATATCTCGGCTCTCGCATCGATGAAGAGCGCAGCGAAATGCGATACGTGGTGC-GAATTGCAGAATCCCGCGAACC-ATCGAGTCTTTGAACGCAAGTTGCGCCCGAGGCCAAT-CGGTCGAGGGCACGTCCGCCTG---GGCGTCA-AGCGTTGCGCCGCTCC

Robiquetia_succisa_KY966667 ACGAC-TCTCGACAATGGATATCTCGGCTCTCGCATCGATGAAGAGCGCAGCGAAATGCGATACGTGGTGC-GAATTGCAGAATCCCGCGAACC-ATCGAGTCTTTGAACGCAAGTTGCGCCCGAGGCCAAT-CGGTCGAGGGCACGTCCGCCTG---GGCGTCA-AGCGTTGCGCCGCTCC

Robiquetia_succisa_PK12155 ACGAC-TCTCGACAATGGATATCTCGGCTCTCGCATCGATGAAGAGCGCAGCGAAATGCGATACGTGGTGC-GAATTGCAGAATCCCGCGAACC-ATCGAGTCTTTGAACGCAAGTTGCGCCCGAGGCCAAT-CGGTCGAGGGCACGTCCGCCTG---GGCGTCA-AGCGTTGCGCCGCTCC

Robiquetia_succisa_PK12156 ACGAC-TCTCGACAATGGATATCTCGGCTCTCGCATCGATGAAGAGCGCAGCGAAATGCGATACGTGGTGC-GAATTGCAGAATCCCGCGAACC-ATCGAGTCTTTGAACGCAAGTTGCGCCCGAGGCCAAT-CGGTCGAGGGCACGTCCGCCTG---GGCGTCA-AGCGTTGCGCCGCTCC

Robiquetia_succisa_SG1293 ACGAC-TCTCGACAATGGATATCTCGGCTCTCGCATCGATGAAGAGCGCAGCGAAATGCGATACGTGGTGC-GAATTGCAGAATCCCGCGAACC-ATCGAGTCTTTGAACGCAAGTTGCGCCCGAGGCCAAT-CGGTCGAGGGCACGTCCGCCTG---GGCGTCA-AGCGTTGCGCCGCTCC

Robiquetia_succisa_SG1294 ACGAC-TCTCGACAATGGATATCTCGGCTCTCGCATCGATGAAGAGCGCAGCGAAATGCGATACGTGGTGC-GAATTGCAGAATCCCGCGAACC-ATCGAGTCTTTGAACGCAAGTTGCGCCCGAGGCCAAT-CGGTCGAGGGCACGTCCGCCTG---GGCGTCA-AGCGTTGCGCCGCTCC

Robiquetia_succisa_SG1345 ACGAC-TCTCGACAATGGATATCTCGGCTCTCGCATCGATGAAGAGCGCAGCGAAATGCGATACGTGGTGC-GAATTGCAGAATCCCGCGAACC-ATCGAGTCTTTGAACGCAAGTTGCGCCCGAGGCCAAT-CGGTCGAGGGCACGTCCGCCTG---GGCGTCA-AGCGTTGCGCCGCTCC

Spathoglottis_pubescens_KM025162 ACGAC-TCTCGGCAATGGATATCTCGGCTCTCGCATCGATGAAGAGCGCAGCGAAATGCGATACGTGGTGC-GAATTGCAGAATCCCGCGAACC-ATCGAGTCTTTGAACGCAAGTTGCGCCCGAGGCCAAC-CGGCCAAGGGCACGTCTGCCTG---GGCGTCA-AGCGTTGCGTCGCTCC

Spathoglottis_pubescens_KP751405 ACGAC-TCTCGGCAATGGATATCTCGGCTCTCGCATCGATGAAGAGCGCAGCGAAATGCGATACGTGGTGC-GAATTGCAGAATCCCGCGAACC-ATCGAGTCTTTGAACGCAAGTTGCGCCCGAGGCCAAC-CGGCCAAGGGCACGTCTGCCTG---GGCGTCA-AGCGTTGCGTCGCTCC

Spathoglottis_pubescens_KP751406 ACGAC-TCTCGGCAATGGATATCTCGGCTCTCGCATCGATGAAGAGCGCAGCGAAATGCGATACGTGGTGC-GAATTGCAGAATCCCGCGAACC-ATCGAGTCTTTGAACGCAAGTTGCGCCCGAGGCCAAC-CGGCCAAGGGCACGTCTGCCTG---GGCGTCA-AGCGTTGCGTCGCTCC

Spathoglottis_pubescens_MG869012 ACGAC-TCTCGGCAATGGATATCTCGGCTCTCGCATCGATGAAGAGCGCAGCGAAATGCGATACGTGGTGC-GAATTGCAGAATCCCGCGAACC-ATCGAGTCTTTGAACGCAAGTTGCGCCCGAGGCCAAC-CGGCCAAGGGCACGTCTGCCTG---GGCGTCA-AGCGTTGCGTCGCTCC

Spathoglottis_pubescens_PK12135 ACGAC-TCTCGGCAATGGATATCTCGGCTCTCGCATCGATGAAGAGCGCAGCGAAATGCGATACGTGGTGC-GAATTGCAGAATCCCGCGAACC-ATCGAGTCTTTGAACGCAAGTTGCGCCCGAGGCCAAC-CGGCCAAGGGCACGTCTGCCTG---GGCGTCA-AGCGTTGCGTCGCTCC

Spathoglottis_pubescens_PK12140 ACGAC-TCTCGGCAATGGATATCTCGGCTCTCGCATCGATGAAGAGCGCAGCGAAATGCGATACGTGGTGC-GAATTGCAGAATCCCGCGAACC-ATCGAGTCTTTGAACGCAAGTTGCGCCCGAGGCCAAC-CGGCCAAGGGCACGTCTGCCTG---GGCGTCA-AGCGTTGCGTCGCTCC

Spathoglottis_pubescens_SG1202 ACGAC-TCTCGGCAATGGATATCTCGGCTCTCGCATCGATGAAGAGCGCAGCGAAATGCGATACGTGGTGC-GAATTGCAGAATCCCGCGAACC-ATCGAGTCTTTGAACGCAAGTTGCGCCCGAGGCCAAC-CGGCCAAGGGCACGTCTGCCTG---GGCGTCA-AGCGTTGCGTCGCTCC

Spathoglottis_pubescens_SG1205 ACGAC-TCTCGGCAATGGATATCTCGGCTCTCGCATCGATGAAGAGCGCAGCGAAATGCGATACGTGGTGC-GAATTGCAGAATCCCGCGAACC-ATCGAGTCTTTGAACGCAAGTTGCGCCCGAGGCCAAC-CGGCCAAGGGCACGTCTGCCTG---GGCGTCA-AGCGTTGCGTCGCTCC

Spiranthes_hongkongensis_MF286484 ATGAC-TCCCGGCAATGGATATCTTGGCTCTTGCATCGATGAAGAGCGCAGCGAAATGCGATACGTGGTGT-GAATTGCAGAATCCCGCGAACC-ATCGAGTTTTTGAACGCAAGTTGCGCCCGAGGCCAAT-TGGCTGAGGGCACGTCCGCCTG---GGCGTCA-AGCATTACATCGCTTC

Spiranthes_hongkongensis_MF286511 ATGAC-TCCCGGCAATGGATATCTTGGCTCTTGCATCGATGAAGAGCGCAGCGAAATGCGATACGTGGTGT-GAATTGCAGAATCCCGTGAACC-ATCGAGTTTTTGAACGCAAGTTGCGCCCGAGGCCAAT-TGGCTGAGGGCACGTCCGCCTG---GGCGTCA-AGCATTACATCGCTTC

Spiranthes_hongkongensis_MH002629 ATGAC-TCCCGGCAATGGATATCTTGGCTCTTGCATCGATGAAGAGCGCAGCGAAATGCGATACGTGGTGT-GAATTGCAGAATCCCGTGAACC-ATCGAGTTTTTGAACGCAAGTTGCGCCCGAGGCCAAT-TGGCTGAGGGCACGTCCGCCTG---GGCGTCA-AGCATTACATCGCTTC

Spiranthes_hongkongensis_MH002630 ATGAC-TCCCGGCAATGGATATCTTGGCTCTTGCATCGATGAAGAGCGCAGCGAAATGCGATACGTGGTGT-GAATTGCAGAATCCCGTGAACC-ATCGAGTTTTTGAACGCAAGTTGCGCCCGAGGCCAAT-TGGCTGAGGGCACGTCCGCCTG---GGCGTCA-AGCATTACATCGCTTC

Spiranthes_hongkongensis_MH002631 ATGAC-TCCCGGCAATGGATATCTTGGCTCTTGCATCGATGAAGAGCGCAGCGAAATGCGATACGTGGTGT-GGATTGCAGAATCCCGTGAACC-ATCGAGTTTTTGAACGCAAGTTGCGCCCGAGGCCAAT-TGGCTGAGGGCACGTCCGCCTG---GGCGTCA-AGCATTACATCGCTTC

Spiranthes_hongkongensis_MH002632 ATGAC-TCCCGGCAATGGATATCTTGGCTCTTGCATCGATGAAGAGCGCAGCGAAATGCGATACGTGGTGT-GAATTGCAGAATCCCGTGAACC-ATCGAGTTTTTGAACGCAAGTTGCGCCCGAGGCCAAT-TGGCTGAGGGCACGTCCGCCTG---GGCGTCA-AGCATTACATCGCTTC

Spiranthes_hongkongensis_MH002633 ATGAC-TCCCGGCAATGGATATCTTGGCTCTTGCATCGATGAAGAGCGCAGCGAAATGCGATACGTGGTGT-GAATTGCAGAATCCCGTGAACC-ATCGAGTTTTTGAACGCAAGTTGCGCCCGAGGCCAAT-TGGCTGAGGGCACGTCCGCCTG---GGCGTCA-AGCATTACATCGCTTC

Spiranthes_hongkongensis_MH002634 ATGAC-TCCCGGCAATGGATATCTTGGCTCTTGCATCGATGAAGAGCGCAGCGAAATGCGATACGTGGTGT-GAATTGCAGAATCCCGTGAACC-ATCGAGTTTTTGAACGCAAGTTGCGCCCGAGGCCAAT-TGGCTGAGGGCACGTCCGCCTG---GGCGTCA-AGCATTACATCGCTTC

Spiranthes_hongkongensis_MH002635 ATGAC-TCCCGGCAATGGATATCTTGGCTCTTGCATCGATGAAGAGCGCAGCGAAATGCGATACGTGGTGT-GAATTGCAGAATCCCGTGAACC-ATCGAGTTTTTGAACGCAAGTTGCGCCCGAGGCCAAT-TGGCTGAGGGCACGTCCGCCTG---GGCGTCA-AGCATTACATCGCTTC

Spiranthes_hongkongensis_MH002636 ATGAC-TCCCGGCAATGGATATCTTGGCTCTTGCATCGATGAAGAGCGCAGCGAAATGCGATACGTGGTGT-GAATTGCAGAATCCCGTGAACC-ATCGAGTTTTTGAACGCAAGTTGCGCCCGAGGCCAAT-TGGCTGAGGGCACGTCCGCCTG---GGCGTCA-AGCATTACATCGCTTC

Spiranthes_hongkongensis_MH002637 ATGAC-TCCCGGCAATGGATATCTTGGCTCTTGCATCGATGAAGAGCGCAGCGAAATGCGATACGTGGTGT-GAATTGCAGAATCCCGTGAACC-ATCGAGTTTTTGAACGCAAGTTGCGCCCGAGGCCAAT-TGGCTGAGGGCACGTCCGCCTG---GGCGTCA-AGCATTACATCGCTTC

Spiranthes_hongkongensis_MH002638 ATGAC-TCCCGGCAATGGATATCTTGGCTCTTGCATCGATGAAGAGCGCAGCGAAATGCGATACGTGGTGT-GAATTGCAGAATCCCGTGAACC-ATCGAGTTTTTGAACGCAAGTTGCGCCCGAGGCCAAT-TGGCTGAGGGCACGTCCGCCTG---GGCGTCA-AGCATTACATCGCTTC

Spiranthes_hongkongensis_MH002639 ATGAC-TCCCGGCAATGGATATCTTGGCTCTTGCATCGATGAAGAGCGCAGCGAAATGCGATACGTGGTGT-GAATTGCAGAATCCCGTGAACC-ATCGAGTTTTTGAACGCAAGTTGCGCCCGAGGCCAAT-TGGCTGAGGGCACGTCCGCCTG---GGCGTCA-AGCATTACATCGCTTC

Spiranthes_hongkongensis_MH002640 ATGAC-TCCCGGCAATGGATATCTTGGCTCTTGCATCGATGAAGAGCGCAGCGAAATGCGATACGTGGTGT-GAATTGCAGAATCCCGTGAACC-ATCGAGTTTTTGAACGCAAGTTGCGCCCGAGGCCAAT-TGGCTGAGGGCACGTCCGCCTG---GGCGTCA-AGCATTACATCGCTTC

Spiranthes_hongkongensis_MH002641 ATGAC-TCCCGGCAATGGATATCTTGGCTCTTGCATCGATGAAGAGCGCAGCGAAATGCGATACGTGGTGT-GAATTGCAGAATCCCGTGAACC-ATCGAGTTTTTGAACGCAAGTTGCGCCCGAGGCCAAT-TGGCTGAGGGCACGTCCGCCTG---GGCGTCA-AGCATTACATCGCTTC

Spiranthes_hongkongensis_MH002642 ATGAC-TCCCGGCAATGGATATCTTGGCTCTTGCATCGATGAAGAGCGCAGCGAAATGCGATACGTGGTGT-GAATTGCAGAATCCCGTGAACC-ATCGAGTTTTTGAACGCAAGTTGCGCCCGAGGCCAAT-TGGCTGAGGGCACGTCCGCCTG---GGCGTCA-AGCATTACATCGCTTC

Spiranthes_hongkongensis_MH002643 ATGAC-TCCCGGCAATGGATATCTTGGCTCTTGCATCGATGAAGAGCGCAGCGAAATGCGATACGTGGTGT-GAATTGCAGAATCCCGTGAACC-ATCGAGTTTTTGAACGCAAGTTGCGCCCGAGGCCAAT-TGGCTGAGGGCACGTCCGCCTG---GGCGTCA-AGCATTACATCGCTTC

Spiranthes_hongkongensis_MH002644 ATGAC-TCCCGGCAATGGATATCTTGGCTCTTGCATCGATGAAGAGCGCAGCGAAATGCGATACGTGGTGT-GAATTGCAGAATCCCGTGAACC-ATCGAGTTTTTGAACGCAAGTTGCGCCCGAGGCCAAT-TGGCTGAGGGCACGTCCGCCTG---GGCGTCA-AGCATTGCATCGCTTC

Spiranthes_hongkongensis_MH002645 ATGAC-TCCCGGCAATGGATATCTTGGCTCTTGCATCGATGAAGAGCGCAGCGAAATGCGATACGTGGTGT-GAATTGCAGAATCCCGTGAACC-ATCGAGTTTTTGAACGCAAGTTGCGCCCGAGGCCAAT-TGGCTGAGGGCACGTCCGCCTG---GGCGTCA-AGCATTACATCGCTTC

Spiranthes_hongkongensis_MH002646 ATGAC-TCCCGGCAATGGATATCTTGGCTCTTGCATCGATGAAGAGCGCAGCGAAATGCGATACGTGGTGT-GAATTGCAGAATCCCGTGAACC-ATCGAGTTTTTGAACGCAAGTTGCGCCCGAGGCCAAT-TGGCTGAGGGCACGTCCGCCTG---GGCGTCA-AGCATTACATCGCTTC

Spiranthes_hongkongensis_MH002647 ATGAC-TCCCGGCAATGGATATCTTGGCTCTTGCATCGATGAAGAGCGCAGCGAAATGCGATACGTGGTGT-GAATTGCAGAATCCCGTGAACC-ATCGAGTTTTTGAACGCAAGTTGCGCCCGAGGCCAAT-TGGCTGAGGGCACGTCCGCCTG---GGCGTCA-AGCATTACATCGCTTC

Spiranthes_hongkongensis_MH002648 ATGAC-TCCCGGCAATGGATATCTTGGCTCTTGCATCGATGAAGAGCGCAGCGAAATGCGATACGTGGTGT-GAATTGCAGAATCCCGTGAACC-ATCGAGTTTTTGAACGCAAGTTGCGCCCGAGGCCAAT-TGGCTGAGGGCACGTCCGCCTG---GGCGTCA-AGCATTACATCGCTTC

Spiranthes_hongkongensis_MH002649 ATGAC-TCCCGGCAATGGATATCTTGGCTCTTGCATCGATGAAGAGCGCAGCGAAATGCGATACGTGGTGT-GGATTGCAGAATCCCGTGAACC-ATCGAGTTTTTGAACGCAAGTTGCGCCCGAGGCCAAT-TGGCTGAGGGCACGTCCGCCTG---GGCGTCA-AGCATTACATCGCTTC

Spiranthes_hongkongensis_MH002650 ATGAC-TCCCGGCAATGGATATCTTGGCTCTTGCATCGATGAAGAGCGCAGCGAAATGCGATACGTGGTGT-GAATTGCAGAATCCCGTGAACC-ATCGAGTTTTTGAACGCAAGTTGCGCCCGAGGCCAAT-TGGCTGAGGGCACGTCCGCCTG---GGCGTCA-AGCATTACATCGCTTC

Spiranthes_hongkongensis_MH002651 ATGAC-TCCCGGCAATGGATATCTTGGCTCTTGCATCGATGAAGAGCGCAGCGAAATGCGATACGTGGTGT-GAATTGCAGAATCCCGTGAACC-ATCGAGTTTTTGAACGCAAGTTGCGCCCGAGGCCAAT-TGGCTGAGGGCACGTCCGCCTG---GGCGTCA-AGCATTACATCGCTTC

Spiranthes_hongkongensis_MH002652 ATGAC-TCCCGGCAATGGATATCTTGGCTCTTGCATCGATGAAGAGCGCAGCGAAATGCGATACGTGGTGT-GAATTGCAGAATCCCGTGAACC-ATCGAGTTTTTGAACGCAAGTTGCGCCCGAGGCCAAT-TGGCTGAGGGCACGTCCGCCTG---GGCGTCA-AGCATTACATCGCTTC

Spiranthes_hongkongensis_MH002653 ATGAC-TCCCGGCAATGGATATCTTGGCTCTTGCATCGATGAAGAGCGCAGCGAAATGCGATACGTGGTGT-GAATTGCAGAATCCCGTGAACC-ATCGAGTTTTTGAACGCAAGTTGCGCCCGAGGCCAAT-TGGCTGAGGGCACGTCCGCCTG---GGCGTCA-AGCATTACATCGCTTC

Spiranthes_hongkongensis_MH002654 ATGAC-TCCCGGCAATGGATATCTTGGCTCTTGCATCGATGAAGAGCGCAGCGAAATGCGATACGTGGTGT-GAATTGCAGAATCCCGTGAACC-ATCGAGTTTTTGAACGCAAGTTGCGCCCGAGGCCAAT-TGGCTGAGGGCACGTCCGCCTG---GGCGTCA-AGCATTACATCGCTTC

Spiranthes_hongkongensis_MH002655 ATGAC-TCCCGGCAATGGATATCTTGGCTCTTGCATCGATGAAGAGCGCAGCGAAATGCGATACGTGGTGT-GAATTGCAGAATCCCGTGAACC-ATCGAGTTTTTGAACGCAAGTTGCGCCCGAGGCCAAT-TGGCTGAGGGCACGTCCGCCTG---GGCGTCA-AGCATTACATCGCTTC

Spiranthes_hongkongensis_MH002656 ATGAC-TCCCGGCAATGGATATCTTGGCTCTTGCATCGATGAAGAGCGCAGCGAAATGCGATACGTGGTGT-GAATTGCAGAATCCCGTGAACC-ATCGAGTTTTTGAACGCAAGTTGCGCCCGAGGCCAAT-TGGCTGAGGGCACGTCCGCCTG---GGCGTCA-AGCATTACATCGCTTC

Spiranthes_hongkongensis_MH002657 ATGAC-TCCCGGCAATGGATATCTTGGCTCTTGCATCGATGAAGAGCGCAGCGAAATGCGATACGTGGTGT-GAATTGCAGAATCCCGTGAACC-ATCGAGTTTTTGAACGCAAGTTGCGCCCGAGGCCAAT-TGGCTGAGGGCACGTCCGCCTG---GGCGTCA-AGCATTACATCGCTTC

Spiranthes_hongkongensis_MH002658 ATGAC-TCCCGGCAATGGATATCTTGGCTCTTGCATCGATGAAGAGCGCAGCGAAATGCGATACGTGGTGT-GAATTGCAGAATCCCGTGAACC-ATCGAGTTTTTGAACGCAAGTTGCGCCCGAGGCCAAT-TGGCTGAGGGCACGTCCGCCTG---GGCGTCA-AGCATTACATCGCTTC

Spiranthes_hongkongensis_MH002659 ATGAC-TCCCGGCAATGGATATCTTGGCTCTTGCATCGATGAAGAGCGCAGCGAGATGCGATACGTGGAGC-GAATTGCAGAATCCCGTGAACC-ATCGAGTTTTTGAACGCAAGTTGCGCCCGAGGCCAAT-TGGCTGAGGGCACGTCCGCCTG---GGCGTCA-AGCATTACATCGCTTC

Spiranthes_hongkongensis_MH002660 ATGAC-TCCCGGCAATGGATATCTTGGCTCTTGCATCGATGAAGAGCGCAGCGAAATGCGATACGTGGTGT-GAATTGCAGAATCCCGTGAACC-ATCGAGTTTTTGAACGCAAGTTGCGCCCGGGGCCAAT-TGGCTGAGGGCACGTCCGCCTG---GGCGTCA-AGCATTACATCGCTTC

Spiranthes_hongkongensis_MH002661 ATGAC-TCCCGGCAATGGATATCTTGGCTCTTGCATCGATGAAGAGCGCAGCGAAATGCGATACGTGGTGT-GAATTGCAGAATCCCGTGAACC-ATCGAGTTTTTGAACGCAAGTTGCGCCCGAGGCCAAT-TGGCTGAGGGCACGTCCGCCTG---GGCGTCA-AGCATTACATCGCTTC

Spiranthes_hongkongensis_MH002662 ATGAC-TCCCGGCAATGGATATCTTGGCTCTTGCATCGATGAAGAGCGCAGCGAAATGCGATACGTGGTGT-GAATTGCAGAATCCCGTGAACC-ATCGAGTTTTTGAACGCAAGTTGCGCCCGAGGCCAAT-TGGCTGAGGGCACGTCCGCCTG---GGCGTCA-AGCATTACATCGCTTC

Spiranthes_hongkongensis_MH002663 ATGAC-TCCCGGCAATGGATATCTTGGCTCTTGCATCGATGAAGAGCGCAGCGAAATGCGATACGTGGTGT-GAATTGCAGAATCCCGTGAACC-ATCGAGTTTTTGAACGCAAGTTGCGCCCGAGGCCAAT-TGGCTGAGGGCACGTCCGCCTG---GGCGTCA-AGCATTACATCGCTTC

Spiranthes_hongkongensis_MH002664 ATGAC-TCCCGGCAATGGATATCTTGGCTCTTGCATCGATGAAGAGCGCAGCGAAATGCGATACGTGGTGT-GAATTGCAGAATCCCGTGAACC-ATCGAGTTTTTGAACGCAAGTTGCGCCCGAGGCCAAT-TGGCTGAGGGCACGTCCGCCTG---GGCGTCA-AGCATTACATCGCTTC

Spiranthes_hongkongensis_MH002665 ATGAC-TCCCGGCAATGGATATCTTGGCTCTTGCATCGATGAAGAGCGCAGCGAAATGCGATACGTGGTGT-GAATTGCAGAATCCCGTGAACC-ATCGAGTTTTTGAACGCAAGTTGCGCCCGAGGCCAAT-TGGCTGAGGGCACGTCCGCCTG---GGCGTCA-AGCATTACATCGCTTC

Spiranthes_hongkongensis_MH002666 ATGAC-TCCCGGCAATGGATATCTTGGCTCTTGCATCGATGAAGAGTGCAGCGAAATGCGATACGTGGTGT-GAATTGCAGAATCCCGTGAACC-ATCGAGTTTTTGAATGCAAGTTGCGCCCGAGGCCAAT-TGGCTGAGGGCACGTCCGCCTG---GGCGTCA-AGCATTACATCGCTTC

Spiranthes_hongkongensis_MH002667 ATGAC-TCCCGGCAATGGATATCTTGGCTCTTGCATCGATGAAGAGCGCAGCGAAATGCGATACGTGGTGT-GAATTGCAGAATCCCGTGAACC-ATCGAGTTTTTGAACGCAAGTTGCGCCCGAGGCCAAT-TGGCTGAGGGCACGTCCGCCTG---GGCGTCA-AGCATTACATCGCTTC

Spiranthes_hongkongensis_MH002668 ATGAC-TCCCGGCAATGGATATCTTGGCTCTTGCATCGATGAAGAGCGCAGCGAAATGCGATACGTGGTGT-GAATTGCAGAATCCCGTGAACC-ATCGAGTTTTTGAACGCAAGTTGCGCCCGAGGCCAAT-TGGCTGAGGGCACGTCCGCCTG---GGCGTCA-AGCATTACATCGCTTC

Spiranthes_hongkongensis_MH002669 ATGAC-TCCCGGCAATGGATATCTTGGCTCTTGCATCGATGAAGAGCGCAGCGAAATGCGATACGTGGTGT-GAATTGCAGAATCCCGTGAACC-ATCGAGTTTTTGAACGCAAGTCGCGCCCGAGGCCAAT-TGGCTGAGGGCACGTCCGCCTG---GGCGTCA-AGCATTACATCGCTTC

Spiranthes_hongkongensis_MH002670 ATGAC-TCCCGGCAATGGATATCTTGGCTCTTGCATCGATGAAGAGCGCAGCGAAATGCGATACGTGGTGT-GAATTGCAGAATCCCGTGAACC-ATCGAGTTTTTGAACGCAAGTTGCGCCCGAGGCCAAT-TGGCTGAGGGCACGTCCGCCTG---GGCGTCA-AGCATTACATCGCTTC

Spiranthes_hongkongensis_MH002671 ATGAC-TCCCGGCAATGGATATCTTGGCTCTTGCATCGATGAAGAGCGCAGCGAAATGCGATACGTGGTGT-GAATTGCAGAATCCCGTGAACC-ATCGAGTTTTTGAACGCAAGTTGCGCCCGAGGCCAAT-TGGCTGAGGGCACGTCCGCCTG---GGCGTCA-AGCATTACATCGCTTC

Spiranthes_hongkongensis_MH002672 ATGAC-TCCCGGCAATGGATATCTTGGCTCTTGCATCGATGAAGAGCGCAGCGAAATGCGATACGTGGTGT-GAATTGCAGAATCCCGTGAACC-ATCGAGTTTTTGAACGCAAGTTGCGCCCGAGGCCAAT-TGGCTGAGGGCACGTCCGCCTG---GGCGTCA-AGCATTACATCGCTTC

Spiranthes_hongkongensis_MH002673 ATGAC-TCCCGGCAATGGATATCTTGGCTCTTGCATCGATGAAGAGCGCAGCGAAATGCGATACGTGGTGT-GAATTGCAGAATCCCGCGAACC-ATCGAGTTTTTGAACGCAAGTTGCGCCCGAGGCCAAT-TGGCTGAGGGCACGTCCGCCTG---GGCGTCA-AGCATTACATCGCTTC

Spiranthes_hongkongensis_MH002674 ATGAC-TCCCGGCAATGGATATCTTGGCTCTTGCATCGATGAAGAGCGCAGCGAAATGCGATACGTGGTGT-GAATTGCAGAATCCCGTGAACC-ATCGAGTTTTTGAACGCAAGTTGCGCCCGAGGCCAAT-TGGCTGAGGGCACGTCCGCCTG---GGCGTCA-AGCATTACATCGCTTC

Spiranthes_hongkongensis_MH002675 ATGAC-TCCCGGCAATGGATATCTTGGCTCTTGCATCGATGAAGAGCGCAGCGAAATGCGATACGTGGTGT-GAATTGCAGAATCCCGTGAACC-ATCGAGTTTTTGAACGCAAGTTGCGCCCGAGGCCAAT-TGGCTGAGGGCACGTCCGCCTG---GGCGTCA-AGCATTACATCGCTTC

Spiranthes_hongkongensis_MH002676 ATGAC-TCCCGGCAATGGATATCTTGGCTCTTGCATCGATGAAGAGCGCAGCGAAATGCGATACGTGGTGT-GAATTGCAGAATCCCGTGAACC-ATCGAGTTTTTGAACGCAAGTTGCGCCCGAGGCCAAT-TGGCTGAGGGCACGTCCGCCTG---GGCGTCA-AGCATTACATCGCTTC

Spiranthes_hongkongensis_MH002677 ATGAC-TCCCGGCAATGGATATCTTGGCTCTTGCATCGATGAAGAGCGCAGCGAAATGCGATACGTGGTGT-GAATTGCAGAATCCCGTGAACC-ATCGAGTTTTTGAACGCAAGTTGCGCCCGAGGCCAAT-TGGCTGAGGGCACGTCCGCCTG---GGCGTCA-AGCATTACATCGCTTC

Spiranthes_hongkongensis_MH002678 ATGAC-TCCCGGCAATGGATATCTTGGCTCTTGCATCGATGAAGAGCGCAGCGAAATGCGATACGTGGTGT-GAATTGCAGAATCCCGTGAACC-ATCGAGTTTTTGAACGCAAGTTGCGCCCGAGGCCAAT-TGGCTGAGGGCACGTCCGCCTG---GGCGTCA-AGCATTACATCGCTTC

Spiranthes_hongkongensis_MH002679 ATGAC-TCCCGGCAATGGATATCTTGGCTCTTGCATCGATGAAGAGCGCAGCGAAATGCGATACGTGGTGT-GAATTGCAGAATCCCGTGAACC-ATCGAGTTTTTGAACGCAAGTTGCGCCCGAGGCCAAT-TGGCTGAGGGCACGTCCGCCTG---GGCGTCA-AGCATTACATCGCTTC

Spiranthes_hongkongensis_MH002680 ATGAC-TCCCGGCAATGGATATATTGGCTCTTGCATCGATGAAGAGCGCAGCGAAATGCGATACGTGGTGT-GAATTGCAGAATCCCGTGAACC-ATCGAGTTTTTGAACGCAAGTTGCGCCCGAGGCCAAT-TGGCTGAGGGCACGTCCGCCTG---GGCGTCA-AGCATTACATCGCTTC

Spiranthes_hongkongensis_MH002681 ATGAC-TCCCGGCAATGGATATATTGGCTCTTGCATCGATGAAGAGCGCAGCGAAATGCGATACGTGGTGT-GAATTGCAGAATCCCGTGAACC-ATCGAGTTTTTGAACGCAAGTTGCGCCCGAGGCCAAT-TGGCTGAGGGCACGTCCGCCTG---GGCGTCA-AGCATTACATCGCTTC

Spiranthes_hongkongensis_MH002682 ATGAC-TCCCGGCAATGGATATCTTGGCTCTTGCATCGATGAAGAGCGCAGCGAAATGCGATACGTGGTGT-GAATTGCAGAATCCCGTGAACC-ATCGAGTTTTTGAACGCAAGTTGCGCCCGAGGCCAAT-TGGCTGAGGGCACGTCCGCCTG---GGCGTCA-AGCATTACATCGCTTC

Spiranthes_hongkongensis_MH002683 ATGAC-TCCCGGCAATGGATATCTTGGCTCTTGCATCGATGAAGAGCGCAGCGAAATGCGATACGTGGTGT-GAATTGCAGAATCCCGTGAACC-ATCGAGTTTTTGAACGCAAGTTGCGCCCGAGGCCAAT-TGGCTGAGGGCACGTCCGCCTG---GGCGTCA-AGCATTACATCGCTTC

Spiranthes_hongkongensis_MH002684 ATGAC-TCCCGGCAATGGATATCTTGGCTCTTGCATCGATGAAGAGCGCAGCGAAATGCGATACGTGGTGT-GAATTGCAGAATCCCGTGAACC-ATCGAGTTTTTGAACGCAAGTTGCGCCCGAGGCCAAT-TGGCTGAGGGCACGTCCGCCTG---GGCGTCA-AGCATTACATCGCTTC

Spiranthes_hongkongensis_MH002685 ATGAC-TCCCGGCAATGGATATCTTGGCTCTTGCATCGATGAAGAGCGCAGCGAAATGCGATACGTGGTGT-GAATTGCAGAATCCCGTGAACC-ATCGAGTTTTTGAACGCAAGTTGCGCCCGAGGCCAAT-TGGCTGAGGGCACGTCCGCCTG---GGCGTCA-AGCATTACATCGCTTC

Spiranthes_hongkongensis_MH002686 ATGAC-TCCCGGCAATGGATATCTTGGCTCTTGCATCGATGAAGAGCGCAGCGAAATGCGATACGTGGTGT-GAATTGCAGAATCCCGTGAACC-ATCGAGTTTTTGAACGCAAGTTGCGCCCGAGGCCAAT-TGGCTGAGGGCACGTCCGCCTG---GGCGTCA-AGCATTACATCGCTTC

Spiranthes_hongkongensis_MH002687 ATGAC-TCCCGGCAATGGATATCTTGGCTCTTGCATCGATGAAGAGCGCAGCGAAATGCGATACGTGGTGT-GAATTGCAGAATCCCGTGAACC-ATCGAGTTTTTGAATGCAAGTTGCGCCCGAGGCCAAT-TGGCTGAGGGCACGTCCGCCTG---GGCGTCA-AGCATTACATCGCTTC

Spiranthes_hongkongensis_MH002688 ATGAC-TCCCGGCAATGGATATCTTGGCTCTTGCATCGATGAAGAGCGCAGCGAAATGCGATACGTGGTGT-GAATTGCAGAATCCCGTGAACC-ATCGAGTTTTTGAACGCAAGTTGCGCCCGAGGCCAAT-TGGCTGAGGGCACGTCCGCCTG---GGCGTCA-AGCATTACATCGCTTC

Spiranthes_hongkongensis_MH002689 ATGAC-TCCCGGCAATGGATATCTTGGCTCTTGCATCGATGAAGAGCGCAGCGAAATGCGATACGTGGTGT-GAATTGCAGAATCCCGTGAACC-ATCGAGTTTTTGAACGCAAGTTGCGCCCGAGGCCAAT-TGGCTGAGGGCACGTCCGCCTG---GGCGTCA-AGCATTACATCGCTTC

Spiranthes_hongkongensis_MH002690 ATGAC-TCCCGGCAATGGATATCTTGGCTCTTGCATCGATGAAGAGCGCAGCGAAATGCGATACGTGGTGT-GAATTGCAGAATCCCGTGAACC-ATCGAGTTTTTGAACGCAAGTTGCGCCCGAGGCCAAT-TGGCTGAGGGCACGTCCGCCTG---GGCGTCA-AGCATTACATCGCTTC

Spiranthes_hongkongensis_MH002691 ATGAC-TCCCGGCAATGGATATCTTGGCTCTTGCATCGATGAAGAGCGCAGCGAAATGCGATACGTGGTGT-GAATTGCAGAATCCCGTGAACC-ATCGAGTTTTTGAACGCAAGTTGCGCCCGAGGCCAAT-TGGCTGAGGGCACGTCCGCCTG---GGCGTCA-AGCATTACATCGCTTC

Spiranthes_hongkongensis_MH002692 ATGAC-TCCCGGCAATGGATATCTTGGCTCTTGCATCGATGAAGAGCGCAGCGAAATGCGATACGTGGTGT-GAATTGCAGAATCCCGTGAACC-ATCGAGTTTTTGAACGCAAGTTGCGCCCGAGGCCAAT-TGGCTGAGGGCACGTCCGCCTG---GGCGTCA-AGCATTACATCGCTTC

Spiranthes_hongkongensis_MH002693 ATGAC-TCCCGGCAATGGATATCTTGGCTCTTGCATCGATGAAGAGCGCAGCGAAATGCGATACGTGGTGT-GAATTGCAGAATCCCGTGAACC-ATCGAGTTTTTGAACGCAAGTTGCGCCCGAGGCCAAT-TGGCTGAGGGCACGTCCGCCTG---GGCGTCA-AGCATTACATCGCTTC

Spiranthes_hongkongensis_MH002694 ATGAC-TCCCGGCAATGGATATCTTGGCTCTTGCATCGATGAAGAGCGCAGCGAAATGCGATACGTGGTGT-GAATTGCAGAATCCCGTGAACC-ATCGAGTTTTTGAACGCAAGTTGCGCCCGAGGCCAAT-TGGCTGAGGGCACGTCCGCCTG---GGCGTCA-AGCATTACATCGCTTC

Spiranthes_hongkongensis_MH002695 ATGAC-TCCCGGCAATGGATATCTTGGCTCTTGCATCGATGAAGAGCGCAGCGAAATGCGATACGTGGTGT-GAATTGCAGAATCCCGTGAACC-ATCGAGTTTTTGAACGCAAGTCGCGCCCGAGGCCAAT-TGGCTGAGGGCACGTCCGCCTG---GGCGTCA-AGCATTACATCGCTTC

Spiranthes_hongkongensis_MH002696 ATGAC-TCCCGGCAATGGATATCTTGGCTCTTGCATCGATGAAGAGCGCAGCGAAATGCGATACGTGGTGT-GAATTGCAGAATCCCGTGAACC-ATCGAGTTTTTGAACGCAAGTTGCGCCCGAGGCCAAT-TGGCTGAGGGCACGTCCGCCTG---GGCGTCA-AGCATTACATCGCTTC

Spiranthes_hongkongensis_MH002697 ATGAC-TCCCGGCAATGGATATCTTGGCTCTTGCATCGATGAAGAGCGCAGCGAAATGCGATACGTGGTGT-GAATTGCAGAATCCCGTGAACC-ATCGAGTTTTTGAACGCAAGTTGCGCCCGAGGCCAAT-TGGCTGAGGGCACGTCCGCCTG---GGCGTCA-AGCATTACATCGCTTC

Spiranthes_hongkongensis_MH002698 ATGAC-TCCCGGCAATGGATATCTTGGCTCTTGCATCGATGAAGAGCGCAGCGAAATGCGATACGTGGTGT-GAATTGCAGAATCCCGTGAACC-ATCGAGTTTTTGAACGCAAGTTGCGCCCGAGGCCAAT-TGGCTGAGGGCACGTCCGCCTG---GGCGTCA-AGCATTACATCGCTTC

Spiranthes_hongkongensis_MH038786 ATGAC-TCCCGGCAATGGATATCTTGGCTCTTGCATCGATGAAGAGCGCAGCGAAATGCGATACGTGGTGT-GAATTGCAGAATCCCGTGAACC-ATCGAGTTTTTGAACGCAAGTTGCGCCCGAGGCCAAT-TGGCTGAGGGCACGTCCGCCTG---GGCGTCA-AGCATTACATCGCTTC

Spiranthes_hongkongensis_MH038787 ATGAC-TCCCGGCAATGGATATCTTGGCTCTTGCATCGATGAAGAGCGCAGCGAAATGCGATACGTGGTGT-GAATTGCAGAATCCCGTGAACC-ATCGAGTTTTTGAACGCAAGTTGCGCCCGAGGCCAAT-TGGCTGAGGGCACGTCCGCCTG---GGCGTCA-AGCATTACATCGCTTC

Spiranthes_hongkongensis_PK12028 ATGAC-TCCCGGCAATGGATATCTTGGCTCTTGCATCGATGAAGAGCGCAGCGAAATGCGATACGTGGTGT-GAATTGCAGAATCCCGTGAACC-ATCGAGTTTTTGAACGCAAGTTGCGCCCGAGGCCAAT-TGGCTGAGGGCACGTCCGCCTG---GGCGTCA-AGCATTACATCGCTTC

Spiranthes_hongkongensis_PK12102 ATGAC-TCCCGGCAATGGATATCTTGGCTCTTGCATCGATGAAGAGCGCAGCGAAATGCGATACGTGGTGT-GAATTGCAGAATCCCGTGAACC-ATCGAGTTTTTGAACGCAAGTTGCGCCCGAGGCCAAT-TGGCTGAGGGCACGTCCGCCTG---GGCGTCA-AGCATTACATCGCTTC

Spiranthes_hongkongensis_PK12179 ATGAC-TCCCGGCAATGGATATCTTGGCTCTTGCATCGATGAAGAGCGCAGCGAAATGCGATACGTGGTGT-GAATTGCAGAATCCCGTGAACC-ATCGAGTTTTTGAACGCAAGTTGCGCCCGAGGCCAAT-TGGCTGAGGGCACGTCCGCCTG---GGCGTCA-AGCATTACATCGCTTC

Spiranthes_sinensis_HE575518 ATGAC-TCCCGGCAATGGATATCTTGGCTCTTGCATCGATGAAGAGCGCAGCGAAATGCGATACGTGGTGT-GAATTGCAGAATCCCGTGAACC-ATCGAGTTTTTGAACGCAAGTTGCGCCCGAGGCCAAT-TGGCTGAGGGCACGTCCGCCTG---GGCGTCA-AGCATTATATCGCTTC

Spiranthes_sinensis_KM262399 ATGAC-TCCCGGCAATGGATATCTTGGCTCTTGCATCGATGAAGAGCGCAGCGAAATGCGATACGTGGTGT-GAATTGCAGAATCCCGTGAACC-ATCGAGTTTTTGAACGCAAGTTGCGCCCGAGGCCAAT-TGGCTGAGGGCACGTCCGCCTG---GGCGTCA-AGCATTACATCGCTTC

Spiranthes_sinensis_KM262400 ATGAC-TCCCGGCAATGGATATCTTGGCTCTTGCATCGATGAAGAGCGCAGCGAAATGCGATACGTGGTGT-GAATTGCAGAATCCCGTGAACC-ATCGAGTTTTTGAACGCAAGTTGCGCCCGAGGCCAAT-TGGCTGAGGGCACGTCCGCCTG---GGCGTCA-AGCATTACATCGCTTC

Spiranthes_sinensis_KT338780 ATGAC-TCCCGGCAATGGATATCTTGGCTCTTGCATCGATGAAGAGCGCAGCGAAATGCGATACGTGGTGT-GAATTGCAGAATCCCGTGAACC-ATCGAGTTTTTGAACGCAAGTTGCGCCCGAGGCCAAT-TGGCTGAGGGCACGTCCGCCTG---GGCGTCA-AGCATTACATCGCTTC

Spiranthes_sinensis_KT338781 ATGAC-TCCCGGCAATGGATATCTTGGCTCTTGCATCGATGAAGAGCGCAGCGAAATGCGATACGTGGTGT-GAATTGCAGAATCCCGTGAACC-ATCGAGTTTTTGAACGCAAGTTGCGCCCGAGGCCAAT-TGGCTGAGGGCACGTCCGCCTG---GGCGTCA-AGCATTACATCGCTTC

Spiranthes_sinensis_MF286485 ATGAC-TCCCGGCAATGGATATCTTGGCTCTTGCATCGATGAAGAGCGCAGCGAAATGCGATACGTGGTGT-GAATTGCAGAATCCCGTGAACC-ATCGAGTTTTTGAACGCAAGTTGCGCCCGAGGCCAAT-TGGCTGAGGGCACGTCCGCCTG---GGCGTCA-AGCATTACATCGCTTC

Spiranthes_sinensis_MF286486 ATGAC-TCCCGGCAATGGATATCTTGGCTCTTGCATCGATGAAGAGCGCAGCGAAATGCGATACGTGGTGT-GAATTGCAGAATCCCGTGAACC-ATCGAGTTTTTGAACGCAAGTTGCGCCCGAGGCCAAT-TGGCTGAGGGCACGTCCGCCTG---GGCGTCA-AGCATTACATCGCTTC

Spiranthes_sinensis_MF286487 ATGAC-TCCCGGCAATGGATATCTTGGCTCTTGCATCGATGAAGAGCGCAGCGAAATGCGATACGTGGTGT-GAATTGCAGAATCCCGTGAACC-ATCGAGTTTTTGAACGCAAGTTGCGCCCGAGGCCAAT-TGGCTGAGGGCACGTCCGCCTG---GGCGTCA-AGCATTACATCGCTTC

Spiranthes_sinensis_MF286488 ATGAC-TCCCGGCAATGGATATCTTGGCTCTTGCATCGATGAAGAGCGCAGCGAAATGCGATACGTGGTGT-GAATTGCAGAATCCCGTGAACC-ATCGAGTTTTTGAACGCAAGTTGCGCCCGAGGCCAAT-TGGCTGAGGGCACGTCCGCCTG---GGCGTCA-AGCATTACATCGCTTC

Spiranthes_sinensis_MF286489 ATGAC-TCCCGGCAATGGATATCTTGGCTCTTGCATCGATGAAGAGCGCAGCGAAATGCGATACGTGGTGT-GAATTGCAGAATCCCGTGAACC-ATCGAGTTTTTGAACGCAAGTTGCGCCCGAGGCCAAT-TGGCTGAGGGCACGTCCGCCTG---GGCGTCA-AGCATTACATCGCTTC

Spiranthes_sinensis_MF286493 ATGAC-TCCCGGCAATGGATATCTTGGCTCTTGCATCGATGAAGAGCGCAGCGAAATGCGATACGTGGTGT-GAATTGCAGAATCCCGTGAACC-ATCGAGTTTTTGAACGCAAGTTGCGCCCGAGGCCAAT-TGGCTGAGGGCACGTCCGCCTG---GGCGTCA-AGCATTACATCGCTTC

Spiranthes_sinensis_MF286494 ATGAC-TCCCGGCAATGGATATCTTGGCTCTTGCATCGATGAAGAGCGCAGCGAAATGCGATACGTGGTGT-GAATTGCAGAATCCCGTGAACC-ATCGAGTTTTTGAACGCAAGTTGCGCCCGAGGCCAAT-TGGCTGAGGGCACGTCCGCCTG---GGCGTCA-AGCATTACATCGCTTC

Spiranthes_sinensis_MF286495 ATGAC-TCCCGGCAATGGATATCTTGGCTCTTGCATCGATGAAGAGCGCAGCGAAATGCGATACGTGGTGT-GAATTGCAGAATCCCGTGAACC-ATCGAGTTTTTGAACGCAAGTTGCGCCCGAGGCCAAT-TGGCTGAGGGCACGTCCGCCTG---GGCGTCA-AGCATTACATCGCCTC

Spiranthes_sinensis_MF286496 ATGAC-TCCCGGCAATGGATATCTTGGCTCTTGCATCGATGAAGAGCGCAGCGAAATGCGATACGTGGTGT-GAATTGCAGAATCCCGTGAACC-ATCGAGTTTTTGAACGCAAGTTGCGCCCGAGGCCAAT-TGGCTGAGGGCACGTCCGCCTG---GGCGTCA-AGCATTACATCGCTTC

Spiranthes_sinensis_MF286497 ATGAC-TCCCGGCAATGGATATCTTGGCTCTTGCATCGATGAAGAGCGCAGCGAAATGCGATACGTGGTGT-GAATTGCAGAATCCCGTGAACC-ATCGAGTTTTTGAACGCAAGTTGCGCCCGAGGCCAAT-TGGCTGAGGGCACGTCCGCCTG---GGCGTCA-AGCATTACATCGCTTC

Spiranthes_sinensis_MF286498 ATGAC-TCCCGGCAATGGATATCTTGGCTCTTGCATCGATGAAGAGCGCAGCGAAATGCGATACGTGGTGT-GAATTGCAGAATCCCGTGAACC-ATCGAGTTTTTGAACGCAAGTTGCGCCCGAGGCCAAT-TGGCTGAGGGCACGTCCGCCTG---GGCGTCA-AGCATTACATCGCTTC

Spiranthes_sinensis_MF286499 ATGAC-TCCCGGCAATGGATATCTTGGCTCTTGCATCGATGAAGAGCGCAGCGAAATGCGATACGTGGTGT-GAATTGCAGAATCCCGTGAACC-ATCGAGTTTTTGAACGCAAGTTGCGCCCGAGGCCAAT-TGGCTGAGGGCACGTCCGCCTG---GGCGTCA-AGCATTACATCGCTTT

Spiranthes_sinensis_MF286504 ATGAC-TCCCGGCAATGGATATCTTGGCTCTTGCATCGATGAAGAGCGCAGCGAAATGCGATACGTGGTGT-GAATTGCAGAATCCCGTGAACC-ATCGAGTTTTTGAACGCAAGTTGCGCCCGAGGCCAAT-TGGCTGAGGGCACGTCCGCCTG---GGCGTCA-AGCATTACATCGCTTC

Spiranthes_sinensis_MF286505 ATGAC-TCCCGGCAATGGATATCTTGGCTCTTGCATCGATGAAGAGCGCAGCGAAATGCGATACGTGGTGT-GAATTGCAGAATCCCGTGAACC-ATCGAGTTTTTGAACGCAAGTTGCGCCCGAGGCCAAT-TGGCTGAGGGCACGTCCGCCTG---GGCGTCA-AGCATTACATCGCTTC

Spiranthes_sinensis_MF286506 ATGAC-TCCCGGCAATGGATATCTTGGCTCTTGCATCGATGAAGAGCGCAGCGAAATGCGATACGTGGTGT-GAATTGCAGAATCCCGTGAACC-ATCGAGTTTTTGAACGCAAGTTGCGCCCGAGGCCAAT-TGGCTGAGGGCACGTCCGCCTG---GGCGTCA-AGCATTACATCGCTTC

Spiranthes_sinensis_MF286507 ATGAC-TCCCGGCAATGGATATCTTGGCTCTTGCATCGATGAAGAGCGCAGCGAAATGCGATACGTGGTGT-GAATTGCAGAATCCCGTGAACC-ATCGAGTTTTTGAACGCAAGTTGCGCCCGAGGCCAAT-TGGCTGAGGGCACGCCCGCCTG---GGCGTCA-AGCATTACATCGCTTC

Spiranthes_sinensis_MF286509 ATGAC-TCCCGGCAATGGATATCTTGGCTCTTGCATCGATGAAGAGCGCAGCGAAATGCGATACGTGGTGT-GAATTGCAGAATCCCGTGAACC-ATCGAGTTTTTGAACGCAAGTTGCGCCCGAGGCCAAT-TGGCTGAGGGCACGTCCGCCTG---GGCGTCA-AGCATTACATCGCTTC

Spiranthes_sinensis_MF286510 ATGAC-TCCCGGCAATGGATATCTTGGCTCTTGCATCGATGAAGAGCGCAGCGAAATGCGATACGTGGTGT-GAATTGCAGAATCCCGTGAACC-ATCGAGTTTTTGAACGCAAGTTGCGCCCGAGGCCAAT-TGGCTGAGGGCACGTCCGCCTG---GGCGTCA-AGCATTACATCGCTTC

Spiranthes_sinensis_MH005035 ATGAC-TCCCGGCAATGGATATCTTGGCTCTTGCATCGATGAAGAGCGCAGCGAAATGCGATACGTGGTGT-GAATTGCAGAATCCCGTGAACC-ATCGAGTTTTTGAACGCAAGTTGCGCCCGAGGCCAAT-TGGCTGAGGGCACGTCCGCCTG---GGCGTCA-AGCATTACATCGCTTC

Spiranthes_sinensis_MH005036 ATGAC-TCCCGGCAATGGATATCTTGGCTCTTGCATCGATGAAGAGCGCAGCGAAATGCGATACGTGGTGT-GAATTGCAGAATCCCGTGAACC-ATCGAGTTTTTGAACGCAAGTTGCGCCCGAGGCCAAT-TGGCTGAGGGCACGTCCGCCTG---GGCGTCA-AGCATTACATCGCTTC

Spiranthes_sinensis_MH005037 ATGAC-TCCCGGCAATGGATATCTTGGCTCTTGCATCGATGAAGAGCGCAGCGAAATGCGATACGTGGTGT-GAATTGCAGAATCCCGTGAACC-ATCGAGTTTTTGAACGCAAGTTGCGCCCGAGGCCAAT-TGGCTGAGGGCACGTCCGCCTG---GGCGTCA-AGCATTACATCGCTTC

Spiranthes_sinensis_MH005038 ATGAC-TCCCGGCAATGGATATCTTGGCTCTTGCATCGATGAAGAGCGCAGCGAAATGCGATACGTGGTGT-GAATTGCAGAATCCCGTGAACC-ATCGAGTTTTTGAACGCAAGTTGCGCCCGAGGCCAAT-TGGCTGAGGGCACGTCCGCCTG---GGCGTCA-AGCATTACATCGCTTC

Spiranthes_sinensis_MH005039 ATGAC-TCCCGGCAATGGATATCTTGGCTCTTGCATCGATGAAGAGCGCAGCGAAATGCGATACGTGGTGT-GAATTGCAGAATCCCGTGAACC-ATCGAGTTTTTGAACGCAAGTTGCGCCCGAGGCCAAT-TGGCTGAGGGCACGTCCGCCTG---GGCGTCA-AGCATTACATCGCTTC

Spiranthes_sinensis_MH005040 ATGAC-TCCCGGCAATGGATATCTTGGCTCTTGCATCGATGAAGAGCGCAGCGAAATGCGATACGTGGTGT-GAATTGCAGAATCCCGTGAACC-ATCGAGTTTTTGAACGCAAGTTGCGCCCGAGGCCAAT-TGGCTGAGGGCACGTCCGCCTG---GGCGTCA-AGCATTACATCGCTTC

Spiranthes_sinensis_MH005041 ATGAC-TCCCGGCAATGGATATCTTGGCTCTTGCATCGATGAAGAGCGCAGCGAAATGCGATACGTGGTGT-GAATTGCAGAATCCCGTGAACC-ATCGAGTTTTTGAACGCAAGTTGCGCCCGAGGCCAAT-TGGCTGAGGGCACGTCCGCCTG---GGCGTCA-AGCATTACATCGCCTC

Spiranthes_sinensis_MH005042 ATGAC-TCCCGGCAATGGATATCTTGGCTCTTGCATCGATGAAGAGCGCAGCGAAATGCGATACGTGGTGT-GAATTGCAGAATCCCGTGAACC-ATCGAGTTTTTGAACGCAAGTTGCGCCCGAGGCCAAT-TGGCTGAGGGCACGTCCGCCTG---GGCGTCA-AGCATTACATCGCTTC

Spiranthes_sinensis_MH005043 ATGAC-TCCCGGCAATGGATATCTTGGCTCTTGCATCGATGAAGAGCGCAGCGAAATGCGATACGTGGTGT-GAATTGCAGAATCCCGTGAACC-ATCGAGTTTTTGAACGCAAGTTGCGCCCGAGGCCAAT-TGGCTGAGGGCACGTCCGCCTG---GGCGTCA-AGCATTACATCGCTTC

Spiranthes_sinensis_MH005044 ATGAC-TCCCGGCAATGGATATCTTGGCTCTTGCATCGATGAAGAGCGCAGCGAAATGCGATACGTGGTGT-GAATTGCAGAATCCCGTGAACC-ATCGAGTTTTTGAACGCAAGTTGCGCCCGAGGCCAAT-TGGCTGAGGGCACGTCCGCCTG---GGCGTCA-AGCATTACATCGCTTC

Spiranthes_sinensis_MH005045 TTGAC-TCCCGGCAATGGATATCTTGGCTCTTGCATCGATGAAGAGCGCAGCGAAATGCGATACGAGGTGT-GAATTGCAGAATCCCGTGAACC-ATCGAGTTTTTGAACGCAAGTTGCGCCCGAGGCCAAT-TGGCTGAGGGCACGTCCGCCTG---GGCGTCA-AGCATTACATCGCTTC

Spiranthes_sinensis_MH005046 ATGAC-TCCCGGCAATGGATATCTTGGCTCTTGCATCGATGAAGAGCGCAGCGAAATGCGATACGTGGTGT-GAATTGCAGAATCCCGTGAACC-ATCGAGTTTTTGAACGCAAGTTGCGCCCGAGGCCAAT-TGGCTGAGGGCACGTCCGCTTG---GGCGTCA-AGCATTACATCGCTTC

Spiranthes_sinensis_MH005047 ATGAC-TCCCGGCAATGGATATCTTGGCTCTTGCATCGATGAAGAGCGCAGCGAAATGCGATACGTGGTGT-GAATTGCAGAATCCCGTGAACC-ATCGAGTTTTTGAACGCAAGTTGCGCCCGAGGCCAAT-TGGCTGAGGGCACGTCCGCCTG---GGCGTCA-AGCATTACATCGCTTC

Spiranthes_sinensis_MH005048 ATGAC-TCCCGGCAATGGATATCTTGGCTCTTGCATCGATGAAGAGCGCAGCGAAATGCGATACGTGGTGT-GAATTGCAGAATCCCGTGAACC-ATCGAGTTTTTGAACGCAAGTTGCGCCCGAGGCCAAT-TGGCTGAGGGCACGTCCGCCTG---GGCGTCA-AGCATTACATCGCTTC

Spiranthes_sinensis_MH005049 ATGAC-TCCCGGCAATGGATATCTTGGCTCTTGCATCGATGAAGAGCGCAGCGAAATGCGATACGTGGTGT-GAATTGCAGAATCCCGTGAACC-ATCGAGTTTTTGAACGCAAGTTGCGCCCGAGGCCAAT-TGGCTGAGGGCACGTCCGCCTG---GGCGTCA-AGCATTACATCGCTTC

Spiranthes_sinensis_MH005050 ATGAC-TCCCGGCAATGGATATCTTGGCTCTTGCATCGATGAAGAGCGCAGCGAAATGCGATACGTGGTGT-GAATTGCAGAATCCCGTGAACC-ATCGAGTTTTTGAACGCAAGTTGCGCCCGAGGCCAAT-TGGCTGAGGGCACGTCCGCCTG---GGCGTCA-AGCATTACATCGCTTC

Spiranthes_sinensis_MH005051 ATGAC-TCCCGGCAATGGATATCTTGGCTCTTGCATCGATGAAGAGCGCAGCGAAATGCGATACGTGGTGT-GAATTGCAGAATCCCGTGAACC-ATCGAGTTTTTGAACGCAAGTTGCGCCCGAGGCCAAT-TGGCTGAGGGCACGTCCGCCTG---GGCGTCA-AGCATTACATCGCTTC

Spiranthes_sinensis_MH005052 ATGAC-TCCCGGCAATGGATATCTTGGCTCTTGCATCGATGAAGAGCGCAGCGAAATGCGATACGTGGTGT-GAATTGCAGAATCCTGTGAACC-ATCAAGTTTTTGAATGCAAGTTGCGCCCAAGGCCAAT-TGGCTGAGGGCACGTCCTCTTG---GGCATCA-AGCATTACATCGCTTC

Spiranthes_sinensis_MH005053 ATGAC-TCCCGGCAATGGATATCTTGGCTCTTGCATCGATGAAGAGCGCAGCGAAATGCGATACGTGGTGT-GAATTGCAGAATCCCGTGAACC-ATCGAGTTTTTGAACGCAAGTTGCGCCCGAGGCCAAT-TGGCTGAGGGCACGTCCGCCTG---GGCGTCA-AGCATTACATCGCTTC

Spiranthes_sinensis_MH005054 ATGAC-TCCCGGCAATGGATATCTTGGCTCTTGCATCGATGTAGAGCGCAGCGAAATGCGATACGTGGTGT-GAATTGCAGAATCCCGTGAACC-ATCGAGTTTTTGAACGCAAGTTGCGCCCGAGGCCAAT-TGGCTGAGGGCACGTCCGCCTG---GGCGTCA-AGCATTACATCGCTTC

Spiranthes_sinensis_MH005055 ATGAC-TCCCGGCAATGGATATCTTGGCTCTTGCATCGATGAAGAGCGCAGCGAAATGCGATACGTGGTGT-GAATTGCAGAATCCCGTGAACC-ATCGAGTTTTTGAACGCAAGTTGCGCCCGAGGCCAAT-TGGCTGAGGGCACGTCCGCCTG---GGCGTCA-AGCATTACATCGCTTC

Spiranthes_sinensis_MH005056 ATGAC-TCCCGGCAATGGATATCTTGGCTCTTGCATCGATGAAGAGCGCAGCGAAATGCGATACGTGGTGT-GAATTGCAGAATCCCGTGAACC-ATCGAGTTTTTGAACGCAAGTTGCGCCCGAGGCCAAT-TGGCTGAGGGCACGTCCGCCTG---GGCGTCA-AGCATTACATCGCTTC

Spiranthes_sinensis_MH005057 ATGAC-CCCCGGCAATGGATATCTTGGCTCTTGCATCGATGAAGAGCGCAGCGAAATGCGATACGTGGTGT-GAATTGCAGAATCCCGTGAACC-ATCGAGTTTTTGAACGCAAGTTGCGCCCGAGGCCAAT-TGGCTGAGGGCACGTCCGCCTG---GGCGTCA-AGCATTACATCGCTTC

Spiranthes_sinensis_MH005058 ATGAC-TCCCGGCAATGGATATCTTGGCTCTTGCATCGATGAAGAGCGCAGCGAAATGCGATACGTGGTGT-GAATTGCAGAATCCCGTGAACC-ATCGAGTTTTTGAACGCAAGTTGCGCCCGAGGCCAAT-TGGCTGAGGGCACGTCCGCCTG---GGCGTCA-AGCATTACATCGCTTC

Spiranthes_sinensis_MH005059 ATGAC-TCCCGGCAATGGATATCTTGGCTCTTGCATCGATGAAGAGCGCAGCGAAATGCGATACGTGGTGT-GAATTGCAGAATCCCGTGAACC-ATCGAGTTTTTGAACGCAAGTTGCGCCCGAGGCCAAT-TGGCTGAGGGCACGTCCGCCTG---GGCGTCA-AGCATTACATCGCTTC

Spiranthes_sinensis_MH038785 ATGAC-TCCCGGCAATGGATATCTTGGCTCTTGCATCGATGAAGAGCGCAGCGAAATGCGATACGTGGTGT-GAATTGCAGAATCCCGTGAACC-ATCGAGTTTTTGAACGCAAGTTGCGCCCGAGGCCAAT-TGGCTGAGGGCACGTCCGCCTG---GGCGTCA-AGCATTACATCGCTTC

Spiranthes_sinensis_MH802049 ATGAC-TCCCGGCAATGGATATCTTGGCTCTTGCATCGATGAAGAGCGCAGCGAAATGCGATACGTGGTGT-GAATTGCAGAATCCCGTGAACC-ATCGAGTTTTTGAACGCAAGTTGCGCCCGAGGCCAAT-TGGCTGAGGGCACGTCCGCCTG---GGCGTCA-AGCATTACATCGCTTC

Spiranthes_sinensis_MH802050 ATGAC-TCCCGGCAATGGATATCTTGGCTCTTGCATCGATGAAGAGCGCAGCGAAATGCGATACGTGGTGT-GAATTGCAGAATCCCGTGAACC-ATCGAGTTTTTGAACGCAAGTTGCGCCCGAGGCCAAT-TGGCTGAGGGCACGTCCGCCTG---GGCGTCA-AGCATTACATCGCTTC

Spiranthes_sinensis_PK12106 ATGAC-TCCCGGCAATGGATATCTTGGCTCTTGCATCGATGAAGAGCGCAGCGAAATGCGATACGTGGTGT-GAATTGCAGAATCCCGTGAACC-ATCGAGTTTTTGAACGCAAGTTGCGCCCGAGGCCAAT-TGGCTGAGGGCACGTCCGCCTG---GGCGTCA-AGCATTACATCGCTTC

Spiranthes_sinensis_PK12109 ATGAC-TCCCGGCAATGGATATCTTGGCTCTTGCATCGATGAAGAGCGCAGCGAAATGCGATACGTGGTGT-GAATTGCAGAATCCCGTGAACC-ATCGAGTTTTTGAACGCAAGTTGCGCCCGAGGCCAAT-TGGCTGAGGGCACGTCCGCCTG---GGCGTCA-AGCATTACATCGCTTC

Spiranthes_sinensis_SG1153 ATGAC-TCCCGGCAATGGATATCTTGGCTCTTGCATCGATGAAGAGCGCAGCGAAATGCGATACGTGGTGT-GAATTGCAGAATCCCGTGAACC-ATCGAGTTTTTGAACGCAAGTTGCGCCCGAGGCCAAT-TGGCTGAGGGCACGTCCGCCTG---GGCGTCA-AGCATTACATCGCTTC

Spiranthes_sinensis_SG1155 ATGAC-TCCCGGCAATGGATATCTTGGCTCTTGCATCGATGAAGAGCGCAGCGAAATGCGATACGTGGTGT-GAATTGCAGAATCCCGTGAACC-ATCGAGTTTTTGAACGCAAGTTGCGCCCGAGGCCAAT-TGGCTGAGGGCACGTCCGCCTG---GGCGTCA-AGCATTACATCGCTTC

Tainia_cordifolia_KF560538 ACGAC-TCTCGGCAATGGATATCTCGGCTCTCGCATCGATGAAGAGCGCAGCGAAATGTGATACGTGGTGC-GAATTGCAGAATCCCGCGAACC-ATCGAGTCTTTGAACGCAAGTTGCGCCCGAGGCCAAT-CGGCCAAGGGCACGTCTGCCTG---GGCGTCA-AGCTTTGTGTCGCTCC

Tainia_cordifolia_KM025163 ACGAC-TCTCGGCAATGGATATCTCGGCTCTCGCATCGATGAAGAGCGCAGCGAAATGTGATACGTGGTGC-GAATTGCAGAATCCCGCGAACC-ATCGAGTCTTTGAACGCAAGTTGCGCCCGAGGCCAAT-CGGCCAAGGGCACGTCTGCCTG---GGCGTCA-AGCTTTGCGTCGCTCC

Tainia_cordifolia_KM025164 ACGAC-TCTCGGCAATGGATATCTCGGCTCTCGCATCGATGAAGAGCGCAGCGAAATGTGATACGTGGTGC-GAATTGCAGAATCCCGCGAACC-ATCGAGTCTTTGAACGCAAGTTGCGCCCGAGGCCAAT-CGGCCAAGGGCACGTCTGCCTG---GGCGTCA-AGCTTTGCGTCGCTCC

Tainia_dunnii_KF560536 ACGAC-TCTCGGCAATGGATATCTCGGCTCTCGCATCGATGAAGAGCGCAGCGAAATGCGATACGTGGTGC-GAATTGCAGAATCCCGCGAACC-ATCGAGTCTTTGAACGCAAGTTGCGCCCGAGGCCAAC-CGGCCAAGGGCACGTCTGCCTG---GGCGTCA-AGCGTTGCGTCGCTCC

Tainia_dunnii_KM025165 ACGAC-TCTCGGCAATGGATATCTCGGCTCTCGCATCGATGAAGAGCGCAGCGAAATGCGATACGTGGTGC-GAATTGCAGAATCCCGCGAACC-ATCGAGTCTTTGAACGCAAGTTGCGCCCGAGGCCAAC-CGGCCAAGGGCACGTCTGCCTG---GGCGTCA-AGCGTTGCGTCGCTCC

Tainia_dunnii_SG1273 ACGAC-TCTCGGCAATGGATATCTCGGCTCTCGCATCGATGAAGAGCGCAGCGAAATGCGATACGTGGTGC-GAATTGCAGAATCCCGCGAACC-ATCGAGTCTTTGAACGCAAGTTGCGCCCGAGGCCAAC-CGGCCAAGGGCACGTCTGCCTG---GGCGTCA-AGCGTTGCGTCGCTCC

Thrixspermum_centipeda_AB217591 ATGAC-TCTCGACAATGGATATCTCGGCTCTCGCATCGATGAAGAGCGCAGCGAAATGCGATACGTGGTGC-GAATTGCAGAATCCCGCGAACC-ATCGAGTCTTTGAACGCAAGTTGCGCCCGAGGCCAAT-CGGTCGAGGGCACGTCCGCCTG---GGCGTCA-AGCGTTGCGCCGCTCC

Thrixspermum_centipeda_KFBG3066D ATGAC-TCTCGACAATGGATATCTCGGCTCTCGCATCGATGAAGAGCGCAGCGAAATGCGATACGTGGTGC-GAATTGCAGAATCCCGCGAACC-ATCGAGTCTTTGAACGCAAGTTGCGCCCGAGGCCAAT-CGGTCGAGGGCACGTCCGCCTG---GGCGTCA-AGCGTTGCGCCGCTCC

Thrixspermum_centipeda_KJ733456 ATGAC-TCTCGACAATGGATATCTCGGCTCTCGCATCGATGAAGAGCGCAGCGAAATGCGATACGTGGTGC-GAATTGCAGAATCCCGCGAACC-ATCGAGTCTTTGAACGCAAGTTGCGCCCGAGGCCAAT-CGGTCGAGGGCACGTCCGCCTG---GGCGTCA-AGCGTTGCGCCGCTCC

Thrixspermum_centipeda_KX679332 ATGAC-TCTCGACAATGGATATCTCGGCTCTCGCATCGATGAAGAGCGCAGCGAAATGCGATACGTGGTGC-GAATTGCAGAATCCCGCGAACC-ATCGAGTCTTTGAACGCAAGTTGCGCCCGAGGCCAAT-CGGTCGAGGGCACGTCCGCCTG---GGCGTCA-AGCGTTGCGCCGCTCC

Thrixspermum_centipeda_KY966674 ATGAC-TCTCGACAATGGATATCTCGGCTCTCGCATCGATGAAGAGCGCAGCGAAATGCGATACGTGGTGC-GAATTGCAGAATCCCGCGAACC-ATCGAGTCTTTGAACGCAAGTTGCGCCCGAGGCCAAT-CGGTCGAGGGCACGTCCGCCTG---GGCGTCA-AGCGTTGCGCCGCTCC

Thrixspermum_centipeda_KY966675 ATGAC-TCTCGACAATGGATATCTCGGCTCTCGCATCGATGAAGAGCGCAGCGAAATGCGATACGTGGTGC-GAATTGCAGAATCCCGCGAACC-ATCGAGTCTTTGAACGCAAGTTGCGCCCGAGGCCAAT-CGGTCGAGGGCACGTCCGCCTG---GGCGTCA-AGCGTTGCGCCGCTCC

Thrixspermum_centipeda_PK12129 ATGAC-TCTCGACAATGGATATCTCGGCTCTCGCATCGATGAAGAGCGCAGCGAAATGCGATACGTGGTGC-GAATTGCAGAATCCCGCGAACC-ATCGAGTCTTTGAACGCAAGTTGCGCCCGAGGCCAAT-CGGTCGAGGGCACGTCCGCCTG---GGCGTCA-AGCGTTGCGCCGCTCC

Thrixspermum_centipeda_PK12130 ATGAC-TCTCGACAATGGATATCTCGGCTCTCGCATCGATGAAGAGCGCAGCGAAATGCGATACGTGGTGC-GAATTGCAGAATCCCGCGAACC-ATCGAGTCTTTGAACGCAAGTTGCGCCCGAGGCCAAT-CGGTCGAGGGCACGTCCGCCTG---GGCGTCA-AGCGTTGCGCCGCTCC

Thrixspermum_centipeda_PK12131 ATGAC-TCTCGACAATGGATATCTCGGCTCTCGCATCGATGAAGAGCGCAGCGAAATGCGATACGTGGTGC-GAATTGCAGAATCCCGCGAACC-ATCGAGTCTTTGAACGCAAGTTGCGCCCGAGGCCAAT-CGGTCGAGGGCACGTCCGCCTG---GGCGTCA-AGCGTTGCGCCGCTCC

Thrixspermum_centipeda_PK12132 ATGAC-TCTCGACAATGGATATCTCGGCTCTCGCATCGATGAAGAGCGCAGCGAAATGCGATACGTGGTGC-GAATTGCAGAATCCCGCGAACC-ATCGAGTCTTTGAACGCAAGTTGCGCCCGAGGCCAAT-CGGTCGAGGGCACGTCCGCCTG---GGCGTCA-AGCGTTGCGCCGCTCC

Thrixspermum_centipeda_PK12133 ATGAC-TCTCGACAATGGATATCTCGGCTCTCGCATCGATGAAGAGCGCAGCGAAATGCGATACGTGGTGC-GAATTGCAGAATCCCGCGAACC-ATCGAGTCTTTGAACGCAAGTTGCGCCCGAGGCCAAT-CGGTCGAGGGCACGTCCGCCTG---GGCGTCA-AGCGTTGCGCCGCTCC

Tropidia_curculigoides_SG1281 ACGAC-TCTCGGCAATGGATATCTCGGCTCTCGCATCGATGAAGAGCGCAGCGAAATGCGATACGTGGTGC-GAATTGCAGAATCCCGCGAACC-ATCGAGTCTTTGAACGCAAGTTGCGCCCGAGGCCAAC-CGGCCAAGGGCACGTCTGCCTG---GGCGTCA-AGCGCCGCGTCGCTCC

Tropidia_curculigoides_SG1282 ACGAC-TCTCGGCAATGGATATCTCGGCTCTCGCATCGATGAAGAGCGCAGCGAAATGCGATACGTGGTGC-GAATTGCAGAATCCCGCGAACC-ATCGAGTCTTTGAACGCAAGTTGCGCCCGAGGCCAAC-CGGCCAAGGGCACGTCTGCCTG---GGCGTCA-AGCGCCGCGTCGCTCC

Tropidia_curculigoides_SG1283 ACGAC-TCTCGGCAATGGATATCTCGGCTCTCGCATCGATGAAGAGCGCAGCGAAATGCGATACGTGGTGC-GAATTGCAGAATCCCGCGAACC-ATCGAGTCTTTGAACGCAAGTTGCGCCCGAGGCCAAC-CGGCCAAGGGCACGTCTGCCTG---GGCGTCA-AGCGCCGCGTCGCTCC

Tropidia_curculigoides_SG1284 ACGAC-TCTCGGCAATGGATATCTCGGCTCTCGCATCGATGAAGAGCGCAGCGAAATGCGATACGTGGTGC-GAATTGCAGAATCCCGCGAACC-ATCGAGTCTTTGAACGCAAGTTGCGCCCGAGGCCAAC-CGGCCAAGGGCACGTCTGCCTG---GGCGTCA-AGCGCCGCGTCGCTCC

Tropidia_nipponica_PK12181 ACGAC-TCTCGGCAATGGATATCTCGGCTCTCGCATCGATGAAGAGCGCAGCGAAATGCGATACGTGGTGC-GAATTGCAGAATCCCGCGAACC-ATCGAGTCTTTGAACGCAAGTTGCGCCCGAGGCCAAC-CGGCCAAGGGCACGCCTGCCTG---GGCGTCA-AGCGCCGCGTCGCTCC

Tropidia_nipponica_SG1355 ACGAC-TCTCGGCAATGGATATCTCGGCTCTCGCATCGATGAAGAGCGCAGCGAAATGCGATACGTGGTGC-GAATTGCAGAATCCCGCGAACC-ATCGAGTCTTTGAACGCAAGTTGCGCCCGAGGCCAAC-CGGCCAAGGGCACGCCTGCCTG---GGCGTCA-AGCGCCGCGTCGCTCC

Tropidia_nipponica_SG1356 ACGAC-TCTCGGCAATGGATATCTCGGCTCTCGCATCGATGAAGAGCGCAGCGAAATGCGATACGTGGTGC-GAATTGCAGAATCCCGCGAACC-ATCGAGTCTTTGAACGCAAGTTGCGCCCGAGGCCAAC-CGGCCAAGGGCACGCCTGCCTG---GGCGTCA-AGCGCCGCGTCGCTCC

Vanilla_shenzhenica_JF796930 ACGAC-TCTCGACAACGGATATCTTGGCTCTCGCATCGATGAAGAACGCAGCGAAATGCGATACGTGTTGT-GAATTGTAGAATCCCGTGAACC-ATCCATTTTTTGAACGCAAGTTGCGCCCGAGGA-TGC-AAGCCAAGGGCACTCCTGCATG---GGTGTAA-TGCGTTCTGTCGCTCC

Vanilla_shenzhenica_KFBG290 ACGAC-TCTCGACAACGGATATCTTGGCTCTCGCATCGATGAAGAACGCAGCGAAATGCGATACGTGTTGT-GAATTGTAGAATCCCGTGAACC-ATCCATTTTTTGAACGCAAGTTGCGCCCGAGGA-TGC-AAGCCAAGGGCACTCCTGCATG---GGTGTAA-TGCGTTCTGTCGCTCC

Vrydagzynea_nuda_SG1222 ATGAC-TCTCGGCAATGGATATCTTGGCTCTTGCATCGATGAAGAGCGCAGCGAAATGCGATACGTGGTGT-GAATTGCAGAATCCCGTGAACC-ATCAAATCTTTGAACGCAAGTTGCGCCTGAGGCCAAT-TGGCTAAGGGCACGTCCGCCTG---GGCGTCA-AGCATTACATCGCTTC

Vrydagzynea_nuda_SG1246A ATGAC-TCTCGGCAATGGATATCTTGGCTCTTGCATCGATGAAGAGCGCAGCGAAATGCGATACGTGGTGT-GAATTGCAGAATCCCGTGAACC-ATCAAATCTTTGAACGCAAGTTGCGCCTGAGGCCAAT-TGGCTAAGGGCACGTCCGCCTG---GGCGTCA-AGCATTACATCGCTTC

Vrydagzynea_nuda_SG1246B ATGAC-TCTCGGCAATGGATATCTTGGCTCTTGCATCGATGAAGAGCGCAGCGAAATGCGATACGTGGTGT-GAATTGCAGAATCCCGTGAACC-ATCAAATCTTTGAACGCAAGTTGCGCCTGAGGCCAAT-TGGCTAAGGGCACGTCCGCCTG---GGCGTCA-AGCATTACATCGCTTC

Vrydagzynea_nuda_SG1247 ATGAC-TCTCGGCAATGGATATCTTGGCTCTTGCATCGATGAAGAGCGCAGCGAAATGCGATACGTGGTGT-GAATTGCAGAATCCCGTGAACC-ATCAAATCTTTGAACGCAAGTTGCGCCTGAGGCCAAT-TGGCTAAGGGCACGTCCGCCTG---GGCGTCA-AGCATTACATCGCTTC

Zeuxine_boninensisi_Z3 ATGAC-TCTCGGCAATGGATATCTTGGCTCTTGCATCGATGAAGAGCGCAGCGAAATGCGATACGTGGTGT-GAATTGCAGAATCCCGTGAACC-ATCAAATATTTGAACGCAAGTTGCGCCCGAGGCCAAT-TGGTTGAGGGCACGTCCGCCTG---GGCGTCA-AGCATTACATCGCTTC

Zeuxine_boninensis_d16 ATGAC-TCTCGGCAATGGATATCTTGGCTCTTGCATCGATGAAGAGCGCAGCGAAATGCGATACGTGGTGT-GAATTGCAGAATCCCGTGAACC-ATCAAATATTTGAACGCAAGTTGCGCCCGAGGCCAAT-TGGTTGAGGGCACGTCCGCCTG---GGCGTCA-AGCATTACATCGCTTC

Zeuxine_gracilis_JN166075 ATGAC-TCTCGGCAATGGATATCTTGGCTCTTGCATCGATGAAGAGCGCAGCGAAATGCGATACGTGGTGT-GAATTGCAGAATCCCGTGAACC-ATCAAATATTTGAACGCAAGTTGCGCCCGAGGCCAAT-TGGTTGAGGGCACGTCCGCCTG---GGCGTCA-AGCATTACATCGCTTC

Zeuxine_gracilis_PK12057 ATGAC-TCTCGACAATGGATATCTTGGCTCTTGCATCGATGAAGAGCGCAGCGAAATGCGATACGTGGTGT-GAATTGCAGAATCCCGTGAACC-ATCAAATATTTGAACGCAAGTTGCGCCCGAGGCCAAT-TGGTTGAGGGCACGTCCGCCTG---GGCGTCA-AGCATTACATCGCTTC

Zeuxine_gracilis_PK12066 ATGAC-TCTCGGCAATGGATATCTTGGCTCTTGCATCGATGAAGAGCGCAGCGAAATGCGATACGTGGTGT-GAATTGCAGAATCCCGTGAACC-ATCAAATATTTGAACGCAAGTTGCGCCCGAGGCCAAT-TGGTTGAGGGCACGTCCGCCTG---GGCGTCA-AGCATTACATCGCTTC

Zeuxine_gracilis_SG1204 ATGAC-TCTCGGCAATGGATATCTTGGCTCTTGCATCGATGAAGAGCGCAGCGAAATGCGATACGTGGTGT-GAATTGCAGAATCCCGTGAACC-ATCAAATATTTGAACGCAAGTTGCGCCCGAGGCCAAT-TGGTTGAGGGCACGTCCGCCTG---GGCGTCA-AGCATTACATCGCTTC

Zeuxine_strateumatica_KT344117 ATGAC-TCTCGGCAATGGATATCTTGGCTCTTGCATCGATGAAGAGCGCAGCGAAATGCGATACGTGGTGT-GAATTGCAGAATCCCGTGAACC-ATCAAATTTTTGAACGCAAGTTGCGCCCAAGGCCAAT-TGGTTGAGGGCACGTCCGCCTG---GGAGTCA-AGCATTACATCGCTTC

Zeuxine_strateumatica_KY966688 ATGAC-TCTCGGCAATGGATATCTTGGCTCTTGCATCGATGAAGAGCGCAGCGAAATGCGATACGTGGTGT-GAATTGCAGAATCCCGTGAACC-ATCAAATTTTTGAACGCAAGTTGCGCCCAAGGCCAAT-TGGTTGAGGGCACGTCCGCCTG---GGAGTCA-AGCATTACATCGCTTC

Zeuxine_strateumatica_SG1211 ATGAC-TCTCGGCAATGGATATCTTGGCTCTTGCATCGATGAAGAGCGCAGCGAAATGCGATACGTGGTGT-GAATTGCAGAATCCCGTGAACC-ATCAAATTTTTGAACGCAAGTTGCGCCCAAGGCCAAT-TGGTTGAGGGCACGTCCGCCTG---GGAGTCA-AGCATTACATCGCTTC

Zeuxine_strateumatica_SG1212 ATGAC-TCTCGGCAATGGATATCTTGGCTCTTGCATCGATGAAGAGCGCAGCGAAATGCGATACGTGGTGT-GAATTGCAGAATCCCGTGAACC-ATCAAATTTTTGAACGCAAGTTGCGCCCAAGGCCAAT-TGGTTGAGGGCACGTCCGCCTG---GGAGTCA-AGCATTACATCGCTTC

;

end;

S3B: matK matix

#NEXUS

begin taxa;

dimensions ntax=785;

taxlabels

Acampe_praemorsa_var_longepedunculata_KF421843

Acampe_praemorsa_var_longepedunculata_KJ733544

Acampe_praemorsa_var_longepedunculata_KY966701

Acampe_praemorsa_var_longepedunculata_PK12086

Acampe_praemorsa_var_longepedunculata_PK12159

Acampe_rigida_SG1199

Ania_hongkongensis_KF673784

Ania_hongkongensis_KFBG4612

Ania_hongkongensis_KFBG4613

Ania_hongkongensis_KY966697

Ania_hongkongensis_PK12027

Ania_hongkongensis_SG1231

Ania_hongkongensis_SG1343

Ania_ruybarrettoi_KFBG43

Ania_ruybarrettoi_KY966707

Ania_ruybarrettoi_SG1395

Anoectochilus_formosanus_EU797513

Anoectochilus_formosanus_MG490281

Anoectochilus_formosanus_PK12215

Anoectochilus_roxburghii_EU817409

Anoectochilus_roxburghii_KF361656

Anoectochilus_roxburghii_KY966708

Anoectochilus_roxburghii_PK12043

Anoectochilus_roxburghii_PK12068

Anoectochilus_roxburghii_PK12069

Anoectochilus_roxburghii_SG1219

Aphyllorchis_montana_PK12147

Aphyllorchis_montana_PK12148

Aphyllorchis_montana_SG1010

Apostasia_nipponica_PK12273

Apostasia_nipponica_PK12274

Appendicula_cornuta_AY121739

Appendicula_cornuta_AY368393

Appendicula_cornuta_KF361651

Appendicula_cornuta_KY239521

Appendicula_cornuta_KY966709

Appendicula_cornuta_KY966710

Appendicula_cornuta_PK12065

Arundina_graminifolia_AF263626

Arundina_graminifolia_AF302692

Arundina_graminifolia_EF079333

Arundina_graminifolia_JN004354

Arundina_graminifolia_JN004355

Arundina_graminifolia_JN004356

Arundina_graminifolia_KF421845

Arundina_graminifolia_KX298566

Arundina_graminifolia_KY966712

Arundina_graminifolia_SG1008

Arundina_graminifolia_SG1206

Arundina_graminifolia_SG1295

Arundina_graminifolia_var_graminifolia_AB844195

Arundina_graminifolia_var_graminifolia_AB844196

Arundina_graminifolia_var_graminifolia_AB844197

Arundina_graminifolia_var_graminifolia_AB844198

Arundina_graminifolia_var_graminifolia_AB844199

Arundina_graminifolia_var_graminifolia_AB844200

Arundina_graminifolia_var_graminifolia_AB872252

Arundina_graminifolia_var_revoluta_AB844201

Arundina_graminifolia_var_revoluta_AB844202

Arundina_graminifolia_var_revoluta_AB844203

Arundina_graminifolia_var_revoluta_AB844204

Arundina_graminifolia_var_revoluta_AB844205

Arundina_graminifolia_var_revoluta_AB844206

Arundina_graminifolia_var_revoluta_AB844207

Arundina_graminifolia_var_revoluta_AB844208

Arundina_graminifolia_var_revoluta_AB844209

Arundina_graminifolia_var_revoluta_AB844210

Arundina_graminifolia_var_revoluta_AB844211

Arundina_graminifolia_var_revoluta_AB844212

Arundina_graminifolia_var_revoluta_AB844213

Arundina_graminifolia_var_revoluta_AB844214

Arundina_graminifolia_var_revoluta_AB844215

Arundina_graminifolia_var_revoluta_AB844216

Arundina_graminifolia_var_revoluta_AB844217

Bletilla_striata_AF263630

Bletilla_striata_EF079331

Bletilla_striata_EU490679

Bletilla_striata_GQ434129

Bletilla_striata_KC704596

Bletilla_striata_KC704597

Bletilla_striata_KF262041

Bletilla_striata_KF262042

Bletilla_striata_KF361655

Bletilla_striata_KF673785

Bletilla_striata_KF673786

Bletilla_striata_KFBG2048

Bletilla_striata_KY966713

Bletilla_striata_MG490283

Brachycorythis_galeandra_PK12195

Brachycorythis_galeandra_SG1261

Bulbophyllum_affine_KF361658

Bulbophyllum_affine_KF974494

Bulbophyllum_affine_KFBG412

Bulbophyllum_affine_KJ462086

Bulbophyllum_affine_KX455831

Bulbophyllum_affine_KY966714

Bulbophyllum_affine_SG1606

Bulbophyllum_affine_SG1607

Bulbophyllum_ambrosia_KF361657

Bulbophyllum_ambrosia_KY966715

Bulbophyllum_ambrosia_KY966716

Bulbophyllum_ambrosia_KY966717

Bulbophyllum_ambrosia_KY966718

Bulbophyllum_ambrosia_PK12090

Bulbophyllum_ambrosia_PK12092

Bulbophyllum_ambrosia_SG1221

Bulbophyllum_bicolor_CL10

Bulbophyllum_bicolor_FT28

Bulbophyllum_bicolor_KFBG2210

Bulbophyllum_bicolor_KFBG3073

Bulbophyllum_bicolor_KFBG433A

Bulbophyllum_bicolor_KFBG445

Bulbophyllum_bicolor_KY022445

Bulbophyllum_bicolor_KY966722

Bulbophyllum_bicolor_KY966723

Bulbophyllum_bicolor_KY966724

Bulbophyllum_bicolor_LMP19

Bulbophyllum_bicolor_PSL45

Bulbophyllum_bicolor_TMS05

Bulbophyllum_bicolor_TT43

Bulbophyllum_delitescens_KY966730

Bulbophyllum_delitescens_KY966731

Bulbophyllum_delitescens_KY966732

Bulbophyllum_delitescens_KY966733

Bulbophyllum_delitescens_SG1286

Bulbophyllum_delitescens_SG1287

Bulbophyllum_delitescens_SG1288

Bulbophyllum_kwangtungense_KFBG2820

Bulbophyllum_kwangtungense_KY966740

Bulbophyllum_odoratissimum_FJ216638

Bulbophyllum_odoratissimum_KF974490

Bulbophyllum_odoratissimum_KY966758

Bulbophyllum_odoratissimum_KY966759

Bulbophyllum_odoratissimum_PK12119

Bulbophyllum_odoratissimum_SG1275

Bulbophyllum_pectenveneris_KY966761

Bulbophyllum_pectenveneris_KY966762

Bulbophyllum_scabratum_KY966745

Bulbophyllum_scabratum_PK12041

Bulbophyllum_scabratum_PK12126

Bulbophyllum_scabratum_PK12127

Bulbophyllum_stenobulbon_KFBG2806

Bulbophyllum_stenobulbon_PK12111

Bulbophyllum_stenobulbon_PK12112

Bulbophyllum_stenobulbon_PK12113

Bulbophyllum_stenobulbon_SG1226

Bulbophyllum_tigridum_KFBG468

Bulbophyllum_tigridum_KX455836

Bulbophyllum_tigridum_PK12209

Bulbophyllum_tigridum_SG1310

Bulbophyllum_tseanum_SG1272

Bulbophyllum_tseanum_SG1616

Calanthe_graciliflora_KF673800

Calanthe_graciliflora_KF673801

Calanthe_graciliflora_KF852698

Calanthe_graciliflora_PK12206

Calanthe_graciliflora_PK12207

Calanthe_graciliflora_SG1225

Calanthe_masuca_KFBG11

Calanthe_masuca_PK12110

Calanthe_masuca_SG026

Calanthe_masuca_SG1360

Calanthe_masuca_SG1361

Calanthe_speciosa_KF673817

Calanthe_speciosa_KFBG136

Calanthe_speciosa_PK12168

Calanthe_speciosa_PK12169

Calanthe_speciosa_SG1313

Calanthe_speciosa_SG1368

Calanthe_triplicata_KF673822

Calanthe_triplicata_KF852713

Calanthe_triplicata_KFBG601

Calanthe_triplicata_KM894885

Calanthe_triplicata_KY966783

Calanthe_triplicata_SG1311

Cephalantheropsis_obcordata_KF673828

Cephalantheropsis_obcordata_KFBG140

Cephalantheropsis_obcordata_KFBG2520

Cephalantheropsis_obcordata_KY966786

Cephalantheropsis_obcordata_PK12079

Cheirostylis_yunnanensis_PK12097

Cheirostylis_yunnanensis_SG1227

Cheirostylis_yunnanensis_SG1229

Chrysoglossum_assamicum_SG1622

Chrysoglossum_assamicum_SG1623

Cleisostoma_paniculatum_KFBG516

Cleisostoma_paniculatum_KJ733559

Cleisostoma_rostratum_KFBG789

Cleisostoma_rostratum_KJ733562

Cleisostoma_rostratum_KY966792

Cleisostoma_rostratum_PK12089

Cleisostoma_rostratum_PK12101

Cleisostoma_rostratum_PK12158

Cleisostoma_rostratum_SG1200

Cleisostoma_rostratum_SG1301

Cleisostoma_simondii_KJ733563

Cleisostoma_simondii_KY966793

Cleisostoma_simondii_KY966794

Cleisostoma_simondii_PK12176

Cleisostoma_simondii_SG1314

Cleisostoma_simondii_SG1327

Cleisostoma_simondii_SG1328

Cleisostoma_simondii_SG1329

Cleisostoma_simondii_SG1330

Cleisostoma_simondii_var_guangdongense_KY966696

Coelogyne_cantonensis_KY966934

Coelogyne_cantonensis_SG1239

Coelogyne_cantonensis_SG1339

Coelogyne_cantonensis_SG1388

Coelogyne_chinensis_AF448866

Coelogyne_chinensis_KF361635

Coelogyne_chinensis_KY966935

Coelogyne_chinensis_KY966936

Coelogyne_chinensis_PK12114

Coelogyne_chinensis_PK12115

Coelogyne_chinensis_SG1232

Coelogyne_chinensis_SG1251

Coelogyne_fimbriata_AF302710

Coelogyne_fimbriata_JF422078

Coelogyne_fimbriata_KF361619

Coelogyne_fimbriata_KFBG411

Coelogyne_fimbriata_KFBG523

Coelogyne_fimbriata_KR857337

Coelogyne_fimbriata_KR905392

Coelogyne_fimbriata_KY966796

Coelogyne_fimbriata_SG1059

Coelogyne_fimbriata_SG1061F

Coelogyne_fimbriata_SG1062

Coelogyne_fimbriata_SG1079

Coelogyne_fimbriata_var_leungiana_KFBG640

Coelogyne_fimbriata_var_leungiana_KY966797

Coelogyne_fimbriata_var_leungiana_SG1058

Collabium_chinense_KF673830

Collabium_chinense_KF852672

Crepidium_acuminatum_AB290892

Crepidium_acuminatum_JN004398

Crepidium_acuminatum_JN004399

Crepidium_acuminatum_JN004400

Crepidium_acuminatum_JN004401

Crepidium_acuminatum_JN004402

Crepidium_acuminatum_KJ459304

Crepidium_acuminatum_KX344572

Crepidium_allanii_KFBG4610

Crepidium_allanii_KFBG4611

Crepidium_cordilabium_PK12270

Crepidium_cordilabium_PK12271

Crepidium_cordilabium_PK12272

Crepidium_purpureum_PK12185

Crepidium_purpureum_SG1193

Crepidium_purpureum_SG1194

Crepidium_purpureum_SG1379

Cryptochilus_roseus_KFBG2118

Cryptochilus_roseus_KY239613

Cryptochilus_roseus_KY966803

Cryptochilus_roseus_KY966804

Cryptochilus_roseus_PK12087

Cryptochilus_roseus_PK12088

Cryptostylis_arachnites_PK12188

Cryptostylis_arachnites_SG1380

Cymbidium_aloifolium_AF470485

Cymbidium_aloifolium_JN004411

Cymbidium_aloifolium_JN004412

Cymbidium_aloifolium_KF421846

Cymbidium_aloifolium_KFBG2049

Cymbidium_aloifolium_KX298600

Cymbidium_aloifolium_KY966805

Cymbidium_aloifolium_MG019922

Cymbidium_ensifolium_AF263648

Cymbidium_ensifolium_AF470464

Cymbidium_ensifolium_KF262066

Cymbidium_ensifolium_KJ597877

Cymbidium_ensifolium_KJ597878

Cymbidium_ensifolium_KJ597879

Cymbidium_ensifolium_KJ597880

Cymbidium_ensifolium_KX298607

Cymbidium_ensifolium_PK12208

Cymbidium_ensifolium_SG1214

Cymbidium_ensifolium_SG1278

Cymbidium_ensifolium_SG1285

Cymbidium_kanran_JX202672

Cymbidium_kanran_KC704624

Cymbidium_kanran_KF262069

Cymbidium_kanran_KF673832

Cymbidium_kanran_KJ597870

Cymbidium_kanran_KJ597871

Cymbidium_kanran_KJ597872

Cymbidium_kanran_KJ597873

Cymbidium_lancifolium_AF470475

Cymbidium_lancifolium_KC704625

Cymbidium_lancifolium_KFBG734

Cymbidium_lancifolium_KJ597885

Cymbidium_lancifolium_KJ597886

Cymbidium_lancifolium_KT001524

Cymbidium_lancifolium_KX298612

Cymbidium_lancifolium_KY966806

Cymbidium_lancifolium_PK12128

Cymbidium_lancifolium_SG1274

Cymbidium_sinense_AF470480

Cymbidium_sinense_KC117305

Cymbidium_sinense_KJ597881

Cymbidium_sinense_KJ597882

Cymbidium_sinense_KJ597883

Cymbidium_sinense_KJ597884

Cymbidium_sinense_KT001526

Cymbidium_sinense_KX298618

Cymbidium_sinense_MF093731

Cymbidium_sinense_SG1213

Cymbidium_sinense_SG1218

Cymbidium_sinense_SG1341

Cymbidium_sinense_SG1342

Dendrobium_aduncum_FJ216659

Dendrobium_aduncum_HM055147

Dendrobium_aduncum_KF143427

Dendrobium_aduncum_KFBG8766

Dendrobium_aduncum_KJ187310

Dendrobium_aduncum_KP159290

Dendrobium_aduncum_KR075049

Dendrobium_aduncum_KR089057

Dendrobium_aduncum_MG490229

Dendrobium_aduncum_MG490230

Dendrobium_aduncum_MG490251

Dendrobium_anosmum_AB847694

Dendrobium_anosmum_AB972311

Dendrobium_anosmum_KF957849

Dendrobium_anosmum_KY966807

Dendrobium_anosmum_MG490279

Dendrobium_cf_mimicum_PK12237E

Dendrobium_cf_mimicum_PK12237J

Dendrobium_crumenatum_AB847734

Dendrobium_crumenatum_AB972308

Dendrobium_crumenatum_HM055235

Dendrobium_crumenatum_JF713399

Dendrobium_crumenatum_KC682479

Dendrobium_linawianum_AB847798

Dendrobium_linawianum_KP159291

Dendrobium_linawianum_KY966847

Dendrobium_linawianum_SG1347

Dendrobium_lindleyi_AB847799

Dendrobium_lindleyi_AB972313

Dendrobium_lindleyi_GQ248117

Dendrobium_lindleyi_GU906225

Dendrobium_lindleyi_HM055273

Dendrobium_lindleyi_HM055274

Dendrobium_lindleyi_JF713421

Dendrobium_lindleyi_KFBG203

Dendrobium_lindleyi_KY966848

Dendrobium_lindleyi_KY966849

Dendrobium_lindleyi_KY966850

Dendrobium_lindleyi_MF349993

Dendrobium_lindleyi_MF409032

Dendrobium_lindleyi_MG490247

Dendrobium_loddigesii_AB847803

Dendrobium_loddigesii_AF448864

Dendrobium_loddigesii_FJ216661

Dendrobium_loddigesii_GU565194

Dendrobium_loddigesii_KF143690

Dendrobium_loddigesii_KF361617

Dendrobium_loddigesii_KP159293

Dendrobium_loddigesii_KP704467

Dendrobium_loddigesii_KY966852

Dendrobium_loddigesii_LC086486

Dendrobium_loddigesii_MG490235

Dendrobium_loddigesii_SG1255

Dendrobium_spatella_AB847867

Dendrobium_spatella_KF143721

Dendrobium_spatella_KF143722

Dendrobium_spatella_SG1357

Dendrobium_spatella_SG1358

Dendrolirium_lasiopetalum_KFBG701

Dendrolirium_lasiopetalum_PK12173

Dendrolirium_lasiopetalum_PK12174

Dendrolirium_lasiopetalum_SG1312

Dienia_ophrydis_AY907177

Dienia_ophrydis_AY907181

Dienia_ophrydis_EF079340

Dienia_ophrydis_KF852679

Dienia_ophrydis_KFBG7819

Dienia_ophrydis_KJ459305

Dienia_ophrydis_SG1201

Dienia_ophrydis_SG1254

Dienia_ophrydis_SG1276

Diploprora_championii_KJ733568

Diploprora_championii_KX526719

Diploprora_championii_KY966879

Diploprora_championii_KY966880

Diploprora_championii_PK12025

Diploprora_championii_PK12029

Diploprora_championii_PK12095

Diploprora_championii_SG1144

Diploprora_championii_SG1230

Eria_scabrilinguis_KFBG916

Eria_scabrilinguis_KY239512

Eria_scabrilinguis_KY966883

Eria_scabrilinguis_PK12164

Eria_scabrilinguis_PK12165

Eria_scabrilinguis_SG1302

Erythrodes_blumei_JN166024

Erythrodes_blumei_KT385583

Erythrodes_blumei_PK12103

Erythrodes_blumei_PK12104

Eulophia_flava_JN004434

Eulophia_flava_JN004435

Eulophia_flava_SG1158

Eulophia_flava_SG1159

Eulophia_graminea_FJ565159

Eulophia_graminea_KF358078

Eulophia_graminea_MH767975

Eulophia_graminea_MH767976

Eulophia_graminea_SG1270

Eulophia_graminea_SG1350

Eulophia_picta_FR832768

Eulophia_picta_JN004440

Eulophia_picta_JN004441

Eulophia_picta_JN004442

Eulophia_picta_JN004443

Eulophia_picta_JN004444

Eulophia_picta_JN004445

Eulophia_picta_KF421850

Eulophia_picta_LN831613

Eulophia_picta_MG019924

Eulophia_picta_PK12046

Eulophia_picta_PK12047

Eulophia_picta_PK12048

Eulophia_picta_PK12137

Eulophia_picta_PK12138

Eulophia_picta_PK12153

Eulophia_picta_SG1271

Eulophia_zollingeri_SG1262

Eulophia_zollingeri_SG1263

Eulophia_zollingeri_SG1264

Eulophia_zollingeri_SG1265

Eulophia_zollingeri_SG1353

Gastrochilus_japonicus_KF262103

Gastrochilus_japonicus_KF545886

Gastrochilus_japonicus_KJ733575

Gastrochilus_japonicus_KY966885

Gastrochilus_japonicus_KY966886

Goodyera_foliosa_KT385599

Goodyera_foliosa_KT385600

Goodyera_foliosa_KT385601

Goodyera_foliosa_KT385602

Goodyera_foliosa_KT385603

Goodyera_foliosa_KT385604

Goodyera_foliosa_KT385605

Goodyera_foliosa_Pk12067

Goodyera_foliosa_SG1309

Goodyera_foliosa_SG1315

Goodyera_procera_JN004446

Goodyera_procera_JN004447

Goodyera_procera_JN004448

Goodyera_procera_JN004449

Goodyera_procera_JN004450

Goodyera_procera_KF421851

Goodyera_procera_KT385630

Goodyera_procera_KT385631

Goodyera_procera_KY966888

Goodyera_procera_SG1152

Goodyera_procera_SG1240

Goodyera_procera_SG1241

Goodyera_procera_SG1346

Goodyera_seikomontana_KY966695

Goodyera_seikoomontana_SG1252

Goodyera_seikoomontana_SG1253

Goodyera_viridiflora_AJ310035

Goodyera_viridiflora_JN166027

Goodyera_viridiflora_KT385652

Goodyera_viridiflora_KT385653

Goodyera_viridiflora_KT385654

Goodyera_viridiflora_KT385655

Goodyera_viridiflora_KT385656

Goodyera_viridiflora_PK12170

Goodyera_viridiflora_PK12172

Goodyera_viridiflora_SG1305

Goodyera_viridiflora_SG1306

Habenaria_ciliolaris_MF945424

Habenaria_ciliolaris_MF945533

Habenaria_dentata_JN696438

Habenaria_dentata_KF262035

Habenaria_dentata_KF262036

Habenaria_dentata_KFBG2126B

Habenaria_dentata_KY966891

Habenaria_dentata_PK12059

Habenaria_dentata_SG1005

Habenaria_dentata_SG1009

Habenaria_leptoceras_HM777855

Habenaria_leptoceras_KJ021370

Habenaria_leptoloba_PK12060

Habenaria_leptoloba_PK12160

Habenaria_leptoloba_PK12161

Habenaria_leptoloba_SG1304

Habenaria_leptoloba_SG1362

Habenaria_linguella_MF945416

Habenaria_linguella_PK12049

Habenaria_linguella_PK12134

Habenaria_linguella_PK12141

Habenaria_linguella_SG1195

Habenaria_linguella_SG1299

Habenaria_linguella_SG1300

Habenaria_reniformis_PK12142

Habenaria_reniformis_PK12143

Habenaria_reniformis_PK12144

Habenaria_reniformis_PK12145

Habenaria_reniformis_PK12146

Habenaria_reniformis_SG1296

Habenaria_reniformis_SG1297

Habenaria_rhodocheila_KJ452799

Habenaria_rhodocheila_KR350203

Habenaria_rhodocheila_KY966689

Habenaria_rhodocheila_MF945524

Habenaria_rhodocheila_PK12139

Habenaria_rhodocheila_SG1289

Habenaria_rhodocheila_SG1290

Habenaria_rhodocheila_SG1291

Hetaeria_youngsayei_KY966893

Hetaeria_youngsayei_SG1244

Hetaeria_youngsayei_SG1245

Liparis_bootanensis_KF361637

Liparis_bootanensis_KF852676

Liparis_bootanensis_KFBG238

Liparis_bootanensis_KJ459310

Liparis_bootanensis_KY966897

Liparis_bootanensis_PK12162

Liparis_bootanensis_SG1215

Liparis_bootanensis_SG1216

Liparis_bootanensis_SG1307

Liparis_bootanensis_SG1336

Liparis_ferruginea_SG1156

Liparis_ferruginea_SG1266

Liparis_ferruginea_SG1267

Liparis_gigantea_PK12116

Liparis_gigantea_PK12117

Liparis_gigantea_PK12118

Liparis_nervosa_AY907146

Liparis_nervosa_AY907158

Liparis_nervosa_EF065594

Liparis_nervosa_JN004484

Liparis_nervosa_JN004485

Liparis_nervosa_JN004486

Liparis_nervosa_JN004487

Liparis_nervosa_JN004488

Liparis_nervosa_JN004489

Liparis_nervosa_JN004490

Liparis_nervosa_JN004491

Liparis_nervosa_JN004492

Liparis_nervosa_KF262086

Liparis_nervosa_KFBG330

Liparis_nervosa_KJ459324

Liparis_nervosa_KU748298

Liparis_nervosa_KU748299

Liparis_nervosa_KU748300

Liparis_nervosa_SG1233

Liparis_nervosa_SG1234

Liparis_nervosa_SG1235

Liparis_odorata_KJ021029

Liparis_odorata_SG1256

Liparis_odorata_SG1257

Liparis_sootenzanensis_KJ021030

Liparis_sootenzanensis_SG1351

Liparis_sootenzanensis_SG1352

Liparis_stricklandiana_KF589879

Liparis_stricklandiana_KFBG124

Liparis_stricklandiana_KFBG818

Liparis_stricklandiana_KJ459328

Liparis_stricklandiana_KY966899

Liparis_stricklandiana_PK12091

Liparis_stricklandiana_SG1332

Liparis_stricklandiana_SG1333

Liparis_stricklandiana_SG1337

Liparis_viridiflora_AY907174

Liparis_viridiflora_KJ459329

Liparis_viridiflora_KY966900

Liparis_viridiflora_KY966901

Liparis_viridiflora_PK12120

Liparis_viridiflora_SG1308

Liparis_viridiflora_SG1338

Ludisia_discolor_AJ543911

Ludisia_discolor_KY966902

Ludisia_discolor_MG490282

Ludisia_discolor_SG1236

Ludisia_discolor_SG1237

Ludisia_discolor_SG1348

Nephelaphyllum_tenuiflorum_KC627280

Nephelaphyllum_tenuiflorum_KF673835

Nephelaphyllum_tenuiflorum_KF852689

Nephelaphyllum_tenuiflorum_KY966906

Nephelaphyllum_tenuiflorum_PK12121

Nephelaphyllum_tenuiflorum_PK12122

Nephelaphyllum_tenuiflorum_PK12123

Nephelaphyllum_tenuiflorum_SG1220

Nervilia_plicata_JN004511

Nervilia_plicata_JN004512

Nervilia_plicata_JN004513

Nervilia_plicata_JX865518

Nervilia_plicata_JX865519

Nervilia_plicata_KM986841

Nervilia_plicata_MG452083

Nervilia_plicata_SG1143

Nervilia_plicata_SG1277

Neuwiedia_zollingeri_var_singapureana_SG1268

Neuwiedia_zollingeri_var_singapureana_SG1269

Neuwiedia_zollingeri_var_singapureana_SG1340

Neuwiedia_zollingeri_var_singapureana_JN181463

Neuwiedia_zollingeri_var_singapureana_KC172551

Neuwiedia_zollingeri_var_singapureana_KY966907

Neuwiedia_zollingeri_var_singapureana_LC086542

Pachystoma_pubescens_PK12107

Pachystoma_pubescens_PK12108

Paphiopedilum_purpuratum_KP312072

Paphiopedilum_purpuratum_KP312073

Paphiopedilum_purpuratum_PK12075

Paphiopedilum_purpuratum_SG1149

Pecteilis_susannae_MF945423

Pecteilis_susannae_MF945437

Pecteilis_susannae_MF945502

Pecteilis_susannae_PK12051

Pecteilis_susannae_PK12136

Pecteilis_susannae_PK12154

Pecteilis_susannae_SG1292

Peristylus_calcaratus_MF945517

Peristylus_calcaratus_PK12053

Peristylus_calcaratus_SG1303

Peristylus_densus_JN004550

Peristylus_densus_JN004551

Peristylus_densus_JN004552

Peristylus_densus_JN004553

Peristylus_densus_JN004554

Peristylus_densus_JN004555

Peristylus_densus_JN004556

Peristylus_densus_JN004557

Peristylus_densus_JN004558

Peristylus_densus_JN004559

Peristylus_densus_KM651446

Peristylus_densus_MF945453

Peristylus_densus_SG1258

Peristylus_densus_SG1260

Peristylus_goodyeroides_MF945410

Peristylus_goodyeroides_MF945412

Peristylus_intrudens_PK12050

Peristylus_intrudens_PK12056

Peristylus_intrudens_SG1298

Peristylus_lacertifer_MF945477

Peristylus_lacertifer_MF945478

Peristylus_lacertifer_PK12149

Peristylus_lacertifer_PK12163

Peristylus_lacertifer_SG1006

Persitylus_tentaculatus_PK12062

Persitylus_tentaculatus_PK12171

Persitylus_tentaculatus_SG1007

Phaius_tancarvilleae_AB040205

Phaius_tancarvilleae_EF079306

Phaius_tancarvilleae_EU490700

Phaius_tancarvilleae_KF673843

Phaius_tancarvilleae_KF673844

Phaius_tancarvilleae_KF852704

Phaius_tancarvilleae_KF852707

Phaius_tancarvilleae_KP204599

Phaius_tancarvilleae_KP204600

Phaius_tancarvilleae_KP204601

Phaius_tancarvilleae_KY966927

Phaius_tankervilleae_PK12084

Phaius_tankervilleae_PK12085

Phaius_tankervilleae_PK12099

Phaius_wallichii_KF361633

Phaius_wallichii_KF673845

Phaius_wallichii_KY966928

Platanthera_mandarinorum_JN696443

Platanthera_mandarinorum_var_mandarinorum_KF262020

Platanthera_mandarinorum_var_mandarinorum_KF262021

Platanthera_minor_KJ452825

Platanthera_minor_KJ452835

Platanthera_minor_PK12030

Platanthera_minor_SG1154

Platanthera_minor_SG1223

Platanthera_minor_SG1224

Platanthera_minor_SG1238

Rhomboda_abbreviata_KY966694

Rhomboda_abbreviata_PK12166

Rhomboda_abbreviata_PK12175

Robiquetia_succisa_KJ733601

Robiquetia_succisa_KY966951

Robiquetia_succisa_PK12155

Robiquetia_succisa_PK12156

Robiquetia_succisa_PK12157

Robiquetia_succisa_SG1293

Robiquetia_succisa_SG1294

Robiquetia_succisa_SG1345

Spathoglottis_pubescens_PK12135

Spathoglottis_pubescens_PK12140

Spathoglottis_pubescens_SG1205

Spiranthes_hongkongensis_MF286438

Spiranthes_hongkongensis_MF286439

Spiranthes_hongkongensis_MF286440

Spiranthes_hongkongensis_MF286442

Spiranthes_hongkongensis_MF286464

Spiranthes_hongkongensis_PK12028

Spiranthes_hongkongensis_PK12102

Spiranthes_hongkongensis_PK12179

Spiranthes_sinensis_AB040206

Spiranthes_sinensis_HE575508

Spiranthes_sinensis_JF972946

Spiranthes_sinensis_KC704665

Spiranthes_sinensis_KC704666

Spiranthes_sinensis_KC704667

Spiranthes_sinensis_KF262010

Spiranthes_sinensis_KF262011

Spiranthes_sinensis_KF262012

Spiranthes_sinensis_KF262013

Spiranthes_sinensis_KM262487

Spiranthes_sinensis_KM262488

Spiranthes_sinensis_KP769255

Spiranthes_sinensis_LT600870

Spiranthes_sinensis_MF286443

Spiranthes_sinensis_MF286444

Spiranthes_sinensis_MF286445

Spiranthes_sinensis_MF286446

Spiranthes_sinensis_MF286447

Spiranthes_sinensis_MF286450

Spiranthes_sinensis_MF286451

Spiranthes_sinensis_MF286452

Spiranthes_sinensis_MF286453

Spiranthes_sinensis_MF286454

Spiranthes_sinensis_MF286455

Spiranthes_sinensis_MF286456

Spiranthes_sinensis_MF286457

Spiranthes_sinensis_MF286458

Spiranthes_sinensis_MF286459

Spiranthes_sinensis_MF286460

Spiranthes_sinensis_MF286468

Spiranthes_sinensis_MF286469

Spiranthes_sinensis_MF286470

Spiranthes_sinensis_MF286471

Spiranthes_sinensis_MF286473

Spiranthes_sinensis_MF286474

Spiranthes_sinensis_MF286476

Spiranthes_sinensis_MH036715

Spiranthes_sinensis_MH036716

Spiranthes_sinensis_MH036717

Spiranthes_sinensis_MH036718

Spiranthes_sinensis_MH036719

Spiranthes_sinensis_MH036720

Spiranthes_sinensis_MH036721

Spiranthes_sinensis_MH036722

Spiranthes_sinensis_MH036723

Spiranthes_sinensis_MH036724

Spiranthes_sinensis_MH036725

Spiranthes_sinensis_MH036726

Spiranthes_sinensis_MH036727

Spiranthes_sinensis_MH036728

Spiranthes_sinensis_MH785217

Spiranthes_sinensis_MH785218

Spiranthes_sinensis_PK12106

Spiranthes_sinensis_PK12109

Spiranthes_sinensis_SG1153

Spiranthes_sinensis_SG1155

Tainia_dunnii_KF673846

Tainia_dunnii_KF852708

Tainia_dunnii_SG1273

Thrixspermum_centipeda_AB217767

Thrixspermum_centipeda_KFBG3066D

Thrixspermum_centipeda_KJ733621

Thrixspermum_centipeda_KY966960

Thrixspermum_centipeda_KY966961

Thrixspermum_centipeda_PK12129

Thrixspermum_centipeda_PK12130

Thrixspermum_centipeda_PK12131

Thrixspermum_centipeda_PK12132

Thrixspermum_centipeda_PK12133

Tropidia_curculigoides_SG1281

Tropidia_curculigoides_SG1282

Tropidia_curculigoides_SG1283

Tropidia_curculigoides_SG1284

Tropidia_nipponica_PK12181

Tropidia_nipponica_SG1355

Tropidia_nipponica_SG1356

Vrydagzynea_nuda_SG1222

Vrydagzynea_nuda_SG1246A

Vrydagzynea_nuda_SG1246B

Vrydagzynea_nuda_SG1247

Zeuxine_gracilis_JN166034

Zeuxine_gracilis_PK12066

Zeuxine_gracilis_SG1204

Zeuxine_strateumatica_AJ310080

Zeuxine_strateumatica_KY966975

Zeuxine_strateumatica_SG1211

Zeuxine_strateumatica_SG1212

;

end;

begin characters;

dimensions nchar=1234;

format datatype=dna missing=? gap=-;

matrix

Acampe_praemorsa_var_longepedunculata_KF421843 -----------------------------------------------------------------------------------------------------------------------------------------------------------------------------------------------------------------------------------------------------------------------------------------------------------------------------------TTCCACGAATATCATAATTTGAATAGTCTCATTACTTC----------AAAAAAATCCATTTACGTCTTTTCAAAAAAAAA---GAAAAGA-TTCTTTTGG-TTCCTACATAATTTTTATGTATATGAATGCGAATATATATTCCTCTTTCTTCGTAAACAGTCTTCTTATTTACGATCAATATCTTCTGGAGTCTTTCTTGAGCGAACACATTTTTATGGAAAAATAGAATATCTTAGAGT---TGTTTCTTGTAATTCTTTTCA---------GAGGATCCTATGGTTCCTCAA-AGATATTTTCATACATTATGTTCGATATCAAGGAAAAGCAATTTTGGCTTCAAAAGGAACTCTTATTCTGATGAATAAAT-GGAAATTTCATTTTGTGAATTTTTGGCAATCTTATTTTCACTTTTGGTTTCAACCTTATAGGATCCATATAAAGCAATTACCCAATTATTCCTTCTCTTTTCTGGGA-TATTTTTCAAGTGTACTAAAAAACCCTTTGGTAGTAA-GAAATCAAATGCTAGAGAATTCATTT-CTAATAAATACTCT-GACTAAGAAATTAGATACCATAGCTCCAGTTCTTTTTCTTATTGGATCATTGTCGAAAGCTCAATTTTGTACTGTATTAGGTCATCCTATTAGTAAACCGATCTGGACCAATTTATCGGATTCTGATATTCTTGATCGATTTTGTCGGATATGTAGAAATCCTTGTCGTTATCACAGCGGATCCTCAAAGAA---------------------------------------------------------------------------------------------------------------------------------------------------------

Acampe_praemorsa_var_longepedunculata_KJ733544 ACAAGAATTCTTTTTCTTATCATTTTTAT------------TCTCAAATGGTATCAGAAGGTTTTGGAGTCATTCTGGAAATTTCATTCTCGTCGCGATTAGTATCC---------TCCCTTGAAG---AAAAAAGAATACCAAAATCTCAGAATTTACGATCTATTCATTCAATATTTCCCTTTTTAGAGGATAAATTATCACATTTAAATTATGTGTCGG-ATC-TACT-AATACCCTATCCCATCCATCTGGAAATCTTGGTTCAAATCCTTCAATGCTGGATCAAAGATGTTCCTTCTTTGCATTTCTTGCGATTGATTTTCCACGAATATCATAATTTGAATAGTCTCATTACTTC----------AAAAAAATCCATTTACGTCTTTTCAAAAAAAAA---GAAAAGA-TTCTTTTGG-TTCCTACATAATTTTTATGTATATGAATGCGAATATATATTCCTCTTTCTTCGTAAACAGTCTTCTTATTTACGATCAATATCTTCTGGAGTCTTTCTTGAGCGAACACATTTTTATGGAAAAATAGAATATCTTAGAGT---CGTGTCTTGTAATTCTTTTCA---------GAGGATCCTATGGTTCCTCAA-AGATATTTTCATACATTATGTTCGATATCAAGGAAAAGCAATTTTGGCTTCAAAAGGAACTCTTATTCTGATGAATAAAT-GGAAATTTCATTTTGTGAATTTTTGGCAATCTTATTTTCACTTTTGGTTTCAACCTTATAGGATCCATATAAAGCAATTACCCAATTATTCCTTCTCTTTTCTGGGA-TATTTTTCAAGTGTACTAAAAAACCCTTTGGTAGTAA-GAAATCAAATGCTAGAGAATTCATTT-CTAATAAATACTCT-GACTAAGAAATTAGATACCATAGCTCCAGTTCTTTTTCTTATTGGATCATTGTCGAAAGCTCAATTTTGTACTGTATTAGGTCATCCTATTAGTAAACCGATCTGGACCAATTTATCGGATTCTGATATTCTTGATCGATTTTGTCGGATATGTAGAAATCTTTGTCGTTATCACAGCGGATCCTCAAAGAAACAGGTTTTGTATCGTATAAAGTATATACTTCGACTTTCGTGTGCTAGAACTTTGGCTCGTAAACATAAAAGTACAGTACGCACTTTTATGCGAAGATTAGGTTCGGGATTCTTAG-AAGAATTTTTTTTGGAAGAAGAACAATTTCTTTCCT

Acampe_praemorsa_var_longepedunculata_KY966701 ACAAGAATTCTTTTTCTTATCATTTTTAT------------TCTCAAATGGTATCAGAAGGTTTTGGAGTCATTCTGGAAATTTCATTCTCGTCGCGATTAGTATCC---------TCCCTTGAAG---AAAAAAGAATACCAAAATCTCAAAATTTACGATCTATTCATTCAATATTTCCCTTTTTAGAGGATAAATTATCACATTTAAATTATGTGTCGG-ATC-TACT-AATACCCTATCCCATCCATCTGGAAATCTTGGTTCAAATCCTTCAATGCTGGATCAAAGATGTTCCTTCTTTGCATTTCTTGCGATTGATTTTCCACGAATATCATAATTTGAATAGTCTCATTACTTC----------AAAAAAATCCATTTACGTCTTTTCAAAAAAAAA---GAAAAGA-TTCTTTTGG-TTCCTACATAATTTTTATGTATATGAATGCGAATATATATTCCTCTTTCTTCGTAAACAGTCTTCTTATTTACGATCAATATCTTCTGGAGTCTTTCTTGAGCGAACACATTTTTATGGAAAAATAGAATATCTTAGAGT---CGTGTCTTGTAATTCTTTTCA---------GAGGATCCTATGGTTCCTCAA-AGATATTTTCATACATTATGTTCGATATCAAGGAAAAGCAATTTTGGCTTCAAAAGGAACTCTTATTCTGATGAATAAAT-GGAAATTTCATTTTGTGAATTTTTGGCAATCTTATTTTCACTTTTGGTTTCAACCTTATAGGATCCATATAAAGCAATTACCCAATTATTCCTTCTCTTTTCTGGGA-TATTTTTCAAGTGTACTAAAAAACCCTTTGGTAGTAA-GAAATCAAATGCTAGAGAATTCATTT-CTAATAAATACTCT-GACTAAGAAATTAGATACCATAGCTCCAGTTCTTTTTCTTATTGGATCATTGTCGAAAGCTCAATTTTGTACTGTATTAGGTCATCCTATTAGTAAACCGATCTGGACCAATTTATCGGATTCTGATATTCTTGATCGATTTTGTCGGATATGTAGAAATCTTTGTCGTTATCACAGCGGATCCTCAAAGAAACAGGTTTTGTATCGTATAAAGTATATACTTCGACTTTCGTGTGCTAGAACTTTGGCTCGTAAACATAAAAGTACAGTACGCACTTTTATGCGAAGATTAGGTTCGGGATTCTTAG-AAGAATTTTT--------------------------

Acampe_praemorsa_var_longepedunculata_PK12086 ACAAGAATTCTTTTTCTTATCATTTTTAT------------TCTCAAATGGTATCAGAAGGTTTTGGAGTCATTCTGGAAATTTCATTCTCGTCGCGATTAGTATCC---------TCCCTTGAAG---AAAAAAGAATACCAAAATCTCAGAATTTACGATCTATTCATTCAATATTTCCCTTTTTAGAGGATAAATTATCACATTTAAATTATGTGTCGG-ATC-TACT-AATACCCTATCCCATCCATCTGGAAATCTTGGTTCAAATCCTTCAATGCTGGATCAAAGATGTTCCTTCTTTGCATTTCTTGCGATTGATTTTCCACGAATATCATAATTTGAATAGTCTCATTACTTC----------AAAAAAATCCATTTACGTCTTTTCAAAAAAAAA---GAAAAGA-TTCTTTTGG-TTCCTACATAATTTTTATGTATATGAATGCGAATATATATTCCTCTTTCTTCGTAAACAGTCTTCTTATTTACGATCAATATCTTCTGGAGTCTTTCTTGAGCGAACACATTTTTATGGAAAAATAGAATATCTTAGAGT---CGTGTCTTGTAATTCTTTTCA---------GAGGATCCTATGGTTCCTCAA-AGATATTTTCATACATTATGTTCGATATCAAGGAAAAGCAATTTTGGCTTCAAAAGGAACTCTTATTCTGATGAATAAAT-GGAAATTTCATTTTGTGAATTTTTGGCAATCTTATTTTCACTTTTGGTTTCAACCTTATAGGATCCATATAAAGCAATTACCCAATTATTCCTTCTCTTTTCTGGGA-TATTTTTCAAGTGTACTAAAAAACCCTTTGGTAGTAA-GAAATCAAATGCTAGAGAATTCATTT-CTAATAAATACTCT-GACTAAGAAATTAGATACCATAGCTCCAGTTCTTTTTCTTATTGGATCATTGTCGAAAGCTCAATTTTGTACTGTATTAGGTCATCCTATTAGTAAACCGATCTGGACCAATTTATCGGATTCTGATATTCTTGATCGATTTTGTCGGATATGTAGAAATCTTTGTCGTTATCACAGCGGATCCTCAAAGAAACAGGTTTTGTATCGTATAAAGTATATACTTCGACTTTCGTGTGCTAGAACTTTGGCTCGTAAACATAAAAGTACAGTACGCACTTTTATGCGAAGATTAGGTTCGGGATTCTTAG-AAGAATTTTTTTTGGAAGAAGAACAATTTCTTTCCT

Acampe_praemorsa_var_longepedunculata_PK12159 ACAAGAATTCTTTTTCTTATCATTTTTAT------------TCTCAAATGGTATCAGAAGGTTTTGGAGTCATTCTGGAAATTTCATTCTCGTCGCGATTAGTATCC---------TCCCTTGAAG---AAAAAAGAATACCAAAATCTCAGAATTTACGATCTATTCATTCAATATTTCCCTTTTTAGAGGATAAATTATCACATTTAAATTATGTGTCGG-ATC-TACT-AATACCCTATCCCATCCATCTGGAAATCTTGGTTCAAATCCTTCAATGCTGGATCAAAGATGTTCCTTCTTTGCATTTCTTGCGATTGATTTTCCACGAATATCATAATTTGAATAGTCTCATTACTTC----------AAAAAAATCCATTTACGTCTTTTCAAAAAAAAA---GAAAAGA-TTCTTTTGG-TTCCTACATAATTTTTATGTATATGAATGCGAATATATATTCCTCTTTCTTCGTAAACAGTCTTCTTATTTACGATCAATATCTTCTGGAGTCTTTCTTGAGCGAACACATTTTTATGGAAAAATAGAATATCTTAGAGT---CGTGTCTTGTAATTCTTTTCA---------GAGGATCCTATGGTTCCTCAA-AGATATTTTCATACATTATGTTCGATATCAAGGAAAAGCAATTTTGGCTTCAAAAGGAACTCTTATTCTGATGAATAAAT-GGAAATTTCATTTTGTGAATTTTTGGCAATCTTATTTTCACTTTTGGTTTCAACCTTATAGGATCCATATAAAGCAATTACCCAATTATTCCTTCTCTTTTCTGGGA-TATTTTTCAAGTGTACTAAAAAACCCTTTGGTAGTAA-GAAATCAAATGCTAGAGAATTCATTT-CTAATAAATACTCT-GACTAAGAAATTAGATACCATAGCTCCAGTTCTTTTTCTTATTGGATCATTGTCGAAAGCTCAATTTTGTACTGTATTAGGTCATCCTATTAGTAAACCGATCTGGACCAATTTATCGGATTCTGATATTCTTGATCGATTTTGTCGGATATGTAGAAATCTTTGTCGTTATCACAGCGGATCCTCAAAGAAACAGGTTTTGTATCGTATAAAGTATATACTTCGACTTTCGTGTGCTAGAACTTTGGCTCGTAAACATAAAAGTACAGTACGCACTTTTATGCGAAGATTAGGTTCGGGATTCTTAG-AAGAATTTTTTTTGGAAGAAGAACAATTTCTTTCCT

Acampe_rigida_SG1199 ACAAGAATTCTTTTTCTTATCATTTTTAT------------TCTCAAATGGTATCAGAAGGTTTTGGAGTCATTCTGGAAATTTCATTCTCGTCGCGATTAGTATCC---------TCCCTTGAAG---AAAAAAGAATACCAAAATCTCAGAATTTACGATCTATTCATTCAATATTTCCCTTTTTAGAGGATAAATTATCACATTTAAATTATGTGTCGG-ATC-TACT-AATACCCTATCCCATCCATCTGGAAATCTTGGTTCAAATCCTTCAATGCTGGATCAAAGATGTTCCTTCTTTGCATTTCTTGCGATTGATTTTCCACGAATATCATAATTTGAATAGTCTCATTACTTC----------AAAAAAATCCATTTACGTCTTTTCAAAAAAAAA---GAAAAGA-TTCTTTTGG-TTCCTACATAATTTTTATGTATATGAATGCGAATATATATTCCTCTTTCTTCGTAAACAGTCTTCTTATTTACGATCAATATCTTCTGGAGTCTTTCTTGAGCGAACACATTTTTATGGAAAAATAGAATATCTTAGAGT---CGTGTCTTGTAATTCTTTTCA---------GAGGATCCTATGGTTCCTCAA-AGATATTTTCATACATTATGTTCGATATCAAGGAAAAGCAATTTTGGCTTCAAAAGGAACTCTTATTCTGATGAATAAAT-GGAAATTTCATTTTGTGAATTTTTGGCAATCTTATTTTCACTTTTGGTTTCAACCTTATAGGATCCATATAAAGCAATTACCCAATTATTCCTTCTCTTTTCTGGGA-TATTTTTCAAGTGTACTAAAAAACCCTTTGGTAGTAA-GAAATCAAATGCTAGAGAATTCATTT-CTAATAAATACTCT-GACTAAGAAATTAGATACCATAGCTCCAGTTCTTTTTCTTATTGGATCATTGTCGAAAGCTCAATTTTGTACTGTATTAGGTCATCCTATTAGTAAACCGATCTGGACCAATTTATCGGATTCTGATATTCTTGATCGATTTTGTCGGATATGTAGAAATCTTTGTCGTTATCACAGCGGATCCTCAAAGAAACAGGTTTTGTATCGTATAAAGTATATACTTCGACTTTCGTGTGCTAGAACTTTGGCTCGTAAACATAAAAGTACAGTACGCACTTTTATGCGAAGATTAGGTTCGGGATTCTTAG-AAGAATTTTTTTTGGAAGAAGAACAATTTCTTTCCT

Ania_hongkongensis_KF673784 ACAAGAATTCTTTTTCTTCTCATTTTTCT------------TCTCAAATGGTATCAGAAGGTTTTGGAGTCATTCTGGAAATTCCATTCTCGTCGCGATTAGTATCT---------TCCCTTGAAG---AAAAAAGAATACCAAAATCTCAGAATTTACGATCTATTCATTCAATATTTCCCTTTTTAGAGGATAAATTATCACATTTAAATTATGTGTCAG-ATC-TACT-AATACCCCATCCCATCCATCTGGAAATCTTGGTTCAAATCCTTCAATGCTGGATCAAAGATGTTCCTTCTTTGCATTTATTGCGATTGTTTTTCCACGAATATCATAATTTGAATAGTCTCATTACTTC----------AAAGAAATCCATTTACGTCTTTTCAAAAAGAAA---GAAAAGA-TTCTTTTGG-TTCCTACATAATTCTTATGTATATGAATGCGAATATCTATTCCTGTTTCTTCGTAAACAGTCTTCTTATTTACGATCAATATCTTCTGGAGTCTTTCTTGAGCGAACACATTTCTATGGAAAAATAGAATATCTTATAGT---CGTGTGTTGTAATTCTTTTCA---------GAGGATCCTATGGTTCCTTAA-AGATACTTTCATACATTATGTTCGATATCAAGGAAAAGCGATTATGGCTTCAAAAGGAACTCTTATTCTGATGAAGAAAT-GGAAATTTCATCTTGTAAATTTTTGGCAATCTTATTTTCACTTTTGGTTTCAACCTTATAGGATCCATATAAAGCAATTACCCAACTATTTCTTCTCTTTTCTGGGG-TATTTTTCAAGTGTACTAAAAAACCCTTTGGTAGTAA-GAAATCAAATGCTAGAGAATTCATTT-CTAATAAATACTCT-GACTAAGAAATTAGATACCATAGCCCCAGTTATTTCTCTTATTGGATCATTGTCGAAAGCTCAATTTTGTACTGTATTGGGTCATCCTATTAGTAAACCAATCTGGACCGATTTATCGGATTCTGATATTCTTGATCGATTTTGTCGGATATCTAGAAATCTTTGTCGTTATCACAGCGGATCCTCAAAGAAACAGGTTTTATATCGTATAAAGTATATACTTCGACTTTCGTGTGCTAGAACTTTGGCTCGTAAACATAAAAGTACAGTACGCACTCTTATGCGAAGATTAGGTTCGGGATTCTTAG-AAGAATTTTTTTTGGAAGAAGAACAATCTCTTTCTT

Ania_hongkongensis_KFBG4612 ACAAGAATTCTTTTTCTTCTCATTTTTCT------------TCTCAAATGGTATCAGAAGGTTTTGGAGTCATTCTGGAAATTCCATTCTCGTCGCGATTAGTATCT---------TCCCTTGAAG---AAAAAAGAATACCAAAATCTCAGAATTTACGATCTATTCATTCAATATTTCCCTTTTTAGAGGATAAATTATCACATTTAAATTATGTGTCAG-ATC-TACT-AATACCCCATCCCATCCATCTGGAAATCTTGGTTCAAATCCTTCAATGCTGGATCAAAGATGTTCCTTCTTTGCATTTATTGCGATTGTTTTTCCACGAATATCATAATTTGAATAGTCTCATTACTTC----------AAAGAAATCCATTTACGTCTTTTCAAAAAGAAA---GAAAAGA-TTCTTTTGG-TTCCTACATAATTCTTATGTATATGAATGCGAATATCTATTCCTGTTTCTTCGTAAACAGTCTTCTTATTTACGATCAATATCTTCTGGAGTCTTTCTTGAGCGAACACATTTCTATGGAAAAATAGAATATCTTATAGT---CGTGTGTTGTAATTCTTTTCA---------GAGGATCCTATGGTTCCTTAA-AGATACTTTCATACATTATGTTCGATATCAAGGAAAAGCGATTATGGCTTCAAAAGGAACTCTTATTCTGATGAAGAAAT-GGAAATTTCATCTTGTAAATTTTTGGCAATCTTATTTTCACTTTTGGTTTCAACCTTATAGGATCCATATAAAGCAATTACCCAACTATTTCTTCTCTTTTCTGGGG-TATTTTTCAAGTGTACTAAAAAACCCTTTGGTAGTAA-GAAATCAAATGCTAGAGAATTCATTT-CTAATAAATACTCT-GACTAAGAAATTAGATACCATAGCCCCAGTTATTTCTCTTATTGGATCATTGTCGAAAGCTCAATTTTGTACTGTATTGGGTCATCCTATTAGTAAACCAATCTGGACCGATTTATCGGATTCTGATATTCTTGATCGATTTTGTCGGATATCTAGAAATCTTTGTCGTTATCACAGCGGATCCTCAAAGAAACAGGTTTTATATCGTATAAAGTATATACTTCGACTTTCGTGTGCTAGAACTTTGGCTCGTAAACATAAAAGTACAGTACGCACTCTTATGCGAAGATTAGGTTCGGGATTCTTAG-AATAATTTTTTTTGGAAGAAGAACAATCTCTTTCTT

Ania_hongkongensis_KFBG4613 ACAAGAATTCTTTTTCTTCTCATTTTTCT------------TCTCAAATGGTATCAGAAGGTTTTGGAGTCATTCTGGAAATTCCATTCTCGTCGCGATTAGTATCT---------TCCCTTGAAG---AAAAAAGAATACCAAAATCTCAGAATTTACGATCTATTCATTCAATATTTCCCTTTTTAGAGGATAAATTATCACATTTAAATTATGTGTCAG-ATC-TACT-AATACCCCATCCCATCCATCTGGAAATCTTGGTTCAAATCCTTCAATGCTGGATCAAAGATGTTCCTTCTTTGCATTTATTGCGATTGTTTTTCCACGAATATCATAATTTGAATAGTCTCATTACTTC----------AAAGAAATCCATTTACGTCTTTTCAAAAAGAAA---GAAAAGA-TTCTTTTGG-TTCCTACATAATTCTTATGTATATGAATGCGAATATCTATTCCTGTTTCTTCGTAAACAGTCTTCTTATTTACGATCAATATCTTCTGGAGTCTTTCTTGAGCGAACACATTTCTATGGAAAAATAGAATATCTTATAGT---CGTGTGTTGTAATTCTTTTCA---------GAGGATCCTATGGTTCCTTAA-AGATACTTTCATACATTATGTTCGATATCAAGGAAAAGCGATTATGGCTTCAAAAGGAACTCTTATTCTGATGAAGAAAT-GGAAATTTCATCTTGTAAATTTTTGGCAATCTTATTTTCACTTTTGGTTTCAACCTTATAGGATCCATATAAAGCAATTACCCAACTATTTCTTCTCTTTTCTGGGG-TATTTTTCAAGTGTACTAAAAAACCCTTTGGTAGTAA-GAAATCAAATGCTAGAGAATTCATTT-CTAATAAATACTCT-GACTAAGAAATTAGATACCATAGCCCCAGTTATTTCTCTTATTGGATCATTGTCGAAAGCTCAATTTTGTACTGTATTGGGTCATCCTATTAGTAAACCAATCTGGACCGATTTATCGGATTCTGATATTCTTGATCGATTTTGTCGGATATCTAGAAATCTTTGTCGTTATCACAGCGGATCCTCAAAGAAACAGGTTTTATATCGTATAAAGTATATACTTCGACTTTCGTGTGCTAGAACTTTGGCTCGTAAACATAAAAGTACAGTACGCACTCTTATGCGAAGATTAGGTTCGGGATTCTTAG-AAGAATTTTTTTTGGAAGAAGAACAATCTCTTTCTT

Ania_hongkongensis_KY966697 ACAAGAATTCTTTTTCTTCTCATTTTTCT------------TCTCAAATGGTATCAGAAGGTTTTGGAGTCATTCTGGAAATTCCATTCTCGTCGCGATTAGTATCT---------TCCCTTGAAG---AAAAAAGAATACCAAAATCTCAGAATTTACGATCTATTCATTCAATATTTCCCTTTTTAGAGGATAAATTATCACATTTAAATTATGTGTCAG-ATC-TACT-AATACCCCATCCCATCCATCTGGAAATCTTGGTTCAAATCCTTCAATGCTGGATCAAAGATGTTCCTTCTTTGCATTTATTGCGATTGTTTTTCCACGAATATCATAATTTGAATAGTCCCATTACTTC----------AAAGAAATCCATTTACGTCTTTTCAAAAAGAAA---GAAAAGA-TTCTTTTGG-TTCCTACATAAGAATTATGTATATGAATGCGAATATCTATTCCTGTTTCTTCGTAAACAGTCTTCTTATTTACGATCAATATCTTCTGGAGTCTTTCTTGAGCGAACACATTTCTATGGAAAAAGAGAATATCTTCTAGT---CGTGTGTTGTAATTCTTTTCA---------GAGGATCCTATGGTTCCTCAA-AGATACTTT----CATTATGTTCGATATCAAGGAAAAGCGATTCTGGCTTCAAAAGGAACTCTTATTCTTATGAATAAAT-GGAAATTTCATCTTGTGAATTTTTGGCAATCTTATTTTCACTTTTGGTTTCAACC------GATCCATAGAAAGCAATTACCCAACTATTCCTTC-------TGGGG-TCTTTTTCAAGTGTACTAAAAAATCCTTTGGTAGTAA-GAAATCAAATGCTAGAGAATTCATTT-CTCAGAAATACTCT-GACTAAGAAATTAGATACCATAG-CCCAGTTATTTCTCTTATTGGATCATTT------GCTCAATTTTGTACTGTATTGGGTCATCCTATTAGTAAACCGATCTGGACCGATTTCTCGGATTCTGATATTCTTGATCGATTTTGTCGGATATGTAGAAATCTTTGTCGTTATCACAGCGGATCCTCAAAGAAACAGGTTTTGTATCGTAGAAAGTATATACTTCGACTTTCGTGTGCTAGAACTTTGGCTCGTAAACATAAAAGTACAGTACGCACTCTTATGCGAAGATTAGGTTCGGGATTCTTAG-AAGAATTTTTTTTGGAAGAAGAACAATCTCTTTCTT

Ania_hongkongensis_PK12027 ACAAGAATTCTTTTTCTTCTCATTTTTCT------------TCTCAAATGGTATCAGAAGGTTTTGGAGTCATTCTGGAAATTCCATTCTCGTCGCGATTAGTATCT---------TCCCTTGAAG---AAAAAAGAATACCAAAATCTCAGAATTTACGATCTATTCATTCAATATTTCCCTTTTTAGAGGATAAATTATCACATTTAAATTATGTGTCAG-ATC-TACT-AATACCCCATCCCATCCATCTGGAAATCTTGGTTCAAATCCTTCAATGCTGGATCAAAGATGTTCCTTCTTTGCATTTATTGCGATTGTTTTTCCACGAATATCATAATTTGAATAGTCTCATTACTTC----------AAAGAAATCCATTTACGTCTTTTCAAAAAGAAA---GAAAAGA-TTCTTTTGG-TTCCTACATAATTCTTATGTATATGAATGCGAATATCTATTCCTGTTTCTTCGTAAACAGTCTTCTTATTTACGATCAATATCTTCTGGAGTCTTTCTTGAGCGAACACATTTCTATGGAAAAATAGAATATCTTATAGT---CGTGTGTTGTAATTCTTTTCA---------GAGGATCCTATGGTTCCTTAA-AGATACTTTCATACATTATGTTCGATATCAAGGAAAAGCGATTATGGCTTCAAAAGGAACTCTTATTCTGATGAAGAAAT-GGAAATTTCATCTTGTAAATTTTTGGCAATCTTATTTTCACTTTTGGTTTCAACCTTATAGGATCCATATAAAGCAATTACCCAACTATTTCTTCTCTTTTCTGGGG-TATTTTTCAAGTGTACTAAAAAACCCTTTGGTAGTAA-GAAATCAAATGCTAGAGAATTCATTT-CTAATAAATACTCT-GACTAAGAAATTAGATACCATAGCCCCAGTTATTTCTCTTATTGGATCATTGTCGAAAGCTCAATTTTGTACTGTATTGGGTCATCCTATTAGTAAACCAATCTGGACCGATTTATCGGATTCTGATATTCTTGATCGATTTTGTCGGATATCTAGAAATCTTTGTCGTTATCACAGCGGATCCTCAAAGAAACAGGTTTTATATCGTATAAAGTATATACTTCGACTTTCGTGTGCTAGAACTTTGGCTCGTAAACATAAAAGTACAGTACGCACTCTTATGCGAAGATTAGGTTCGGGATTCTTAG-AAGAATTTTTTTTGGAAGAAGAACAATCTCTTTCTT

Ania_hongkongensis_SG1231 ACAAGAATTCTTTTTCTTCTCATTTTTCT------------TCTCAAATGGTATCAGAAGGTTTTGGAGTCATTCTGGAAATTCCATTCTCGTCGCGATTAGTATCT---------TCCCTTGAAG---AAAAAAGAATACCAAAATCTCAGAATTTACGATCTATTCATTCAATATTTCCCTTTTTAGAGGATAAATTATCACATTTAAATTATGTGTCAG-ATC-TACT-AATACCCCATCCCATCCATCTGGAAATCTTGGTTCAAATCCTTCAATGCTGGATCAAAGATGTTCCTTCTTTGCATTTATTGCGATTGTTTTTCCACGAATATCATAATTTGAATAGTCTCATTACTTC----------AAAGAAATCCATTTACGTCTTTTCAAAAAGAAA---GAAAAGA-TTCTTTTGG-TTCCTACATAATTCTTATGTATATGAATGCGAATATCTATTCCTGTTTCTTCGTAAACAGTCTTCTTATTTACGATCAATATCTTCTGGAGTCTTTCTTGAGCGAACACATTTCTATGGAAAAATAGAATATCTTATAGT---CGTGTGTTGTAATTCTTTTCA---------GAGGATCCTATGGTTCCTTAA-AGATACTTTCATACATTATGTTCGATATCAAGGAAAAGCGATTATGGCTTCAAAAGGAACTCTTATTCTGATGAAGAAAT-GGAAATTTCATCTTGTAAATTTTTGGCAATCTTATTTTCACTTTTGGTTTCAACCTTATAGGATCCATATAAAGCAATTACCCAACTATTTCTTCTCTTTTCTGGGG-TATTTTTCAAGTGTACTAAAAAACCCTTTGGTAGTAA-GAAATCAAATGCTAGAGAATTCATTT-CTAATAAATACTCT-GACTAAGAAATTAGATACCATAGCCCCAGTTATTTCTCTTATTGGATCATTGTCGAAAGCTCAATTTTGTACTGTATTGGGTCATCCTATTAGTAAACCAATCTGGACCGATTTATCGGATTCTGATATTCTTGATCGATTTTGTCGGATATCTAGAAATCTTTGTCGTTATCACAGCGGATCCTCAAAGAAACAGGTTTTATATCGTATAAAGTATATACTTCGACTTTCGTGTGCTAGAACTTTGGCTCGTAAACATAAAAGTACAGTACGCACTCTTATGCGAAGATTAGGTTCGGGATTCTTAG-AAGAATTTTTTTTGGAAGAAGAACAATCTCTTTCTT

Ania_hongkongensis_SG1343 ACAAGAATTCTTTTTCTTCTCATTTTTCT------------TCTCAAATGGTATCAGAAGGTTTTGGAGTCATTCTGGAAATTCCATTCTCGTCGCGATTAGTATCT---------TCCCTTGAAG---AAAAAAGAATACCAAAATCTCAGAATTTACGATCTATTCATTCAATATTTCCCTTTTTAGAGGATAAATTATCACATTTAAATTATGTGTCAG-ATC-TACT-AATACCCCATCCCATCCATCTGGAAATCTTGGTTCAAATCCTTCAATGCTGGATCAAAGATGTTCCTTCTTTGCATTTATTGCGATTGTTTTTCCACGAATATCATAATTTGAATAGTCTCATTACTTC----------AAAGAAATCCATTTACGTCTTTTCAAAAAGAAA---GAAAAGA-TTCTTTTGG-TTCCTACATAATTCTTATGTATATGAATGCGAATATCTATTCCTGTTTCTTCGTAAACAGTCTTCTTATTTACGATCAATATCTTCTGGAGTCTTTCTTGAGCGAACACATTTCTATGGAAAAATAGAATATCTTATAGT---CGTGTGTTGTAATTCTTTTCA---------GAGGATCCTATGGTTCCTTAA-AGATACTTTCATACATTATGTTCGATATCAAGGAAAAGCGATTATGGCTTCAAAAGGAACTCTTATTCTGATGAAGAAAT-GGAAATTTCATCTTGTAAATTTTTGGCAATCTTATTTTCACTTTTGGTTTCAACCTTATAGGATCCATATAAAGCAATTACCCAACTATTTCTTCTCTTTTCTGGGG-TATTTTTCAAGTGTACTAAAAAACCCTTTGGTAGTAA-GAAATCAAATGCTAGAGAATTCATTT-CTAATAAATACTCT-GACTAAGAAATTAGATACCATAGCCCCAGTTATTTCTCTTATTGGATCATTGTCGAAAGCTCAATTTTGTACTGTATTGGGTCATCCTATTAGTAAACCAATCTGGACCGATTTATCGGATTCTGATATTCTTGATCGATTTTGTCGGATATCTAGAAATCTTTGTCGTTATCACAGCGGATCCTCAAAGAAACAGGTTTTATATCGTATAAAGTATATACTTCGACTTTCGTGTGCTAGAACTTTGGCTCGTAAACATAAAAGTACAGTACGCACTCTTATGCGAAGATTAGGTTCGGGATTCTTAG-AAGAATTTTTTTTGGAAGAAGAACAATCTCTTTCTT

Ania_ruybarrettoi_KFBG43 ACAAGAATTCTTTTTCTTCTCATTTTTCT------------TCTCAAATGGTATCAGAAGGTTTTGGAGTCATTCTGGAAATTCCATTCTCGTCGCGATTAGTATCT---------TCCCTTGAAG---AAAAAAGAATACCAAAATCTCAGAATTTACGATCTATTCATTCAATATTTCCCTTTTTAGAGGATAAATTATCACATTTAAATTATGTGTCAG-ATC-TACT-AATACCCCATCCCATCCATCTGGAAATCTTGGTTCAAATCCTTCAATGCTGGATCAAAGATGTTCCTTCTTTGCATTTATTGCGATTGTTTTTCCACGAATATCATAATTTGAATAGTCTCATTACTTC----------AAAGAAATCCATTTACGTCTTTTCAAAAAGAAA---GAAAAGA-TTCTTTTGG-TTCCTACATAATTCTTATGTATATGAATGCGAATATCTATTCCTGTTTCTTCGTAAACAGTCTTCTTATTTACGATCAATATCTTCTGGAGTCTTTCTTGAGCGAACACATTTCTATGGAAAAATAGAATATCTTATAGT---CGTGTGTTGTAATTCTTTTCA---------GAGGATCCTATGGTTCCTCAA-AGATACTTTCATACATTATGTTCGATATCAAGGAAAAGCGATTATGGCTTCAAAAGGAACTCTTATTCTGATGAAGAAAT-GGAAATTTCATCTTGTAAATTTTTGGCAATCTTATTTTCACTTTTGGTTTCAACCTTATAGGATCCATATAAAGCAATTACCCAACTATTTCTTCTCTTTTCTGGGG-TATTTTTCAAGTGTACTAAAAAACCCTTTGGTAGTAA-GAAATCAAATGCTAGAGAATTCATTT-CTAATAAATACTCT-GACTAAGAAATTAGATACCATAGCCCCAGTTATTTCTCTTATTGGATCATTGTCGAAAGCTCAATTTTGTACTGTATTGGGTCATCCTATTAGTAAACCAATCTGGACCGATTTATCGGATTCTGATATTCTTGATCGATTTTGTCGGATATCTAGAAATCTTTGTCGTTATCACAGCGGATCCTCAAAGAAACAGGTTTTGTATCGTATAAAGTATATACTTCGACTTTCGTGTGCTAGAACTTTGGCTCGTAAACATAAAAGTACAGTACGCACTTTTATGCGAAGATTAGGTTCGGGATTCTTAG-AAGAATTTTTTTTGGAAGAAGAACAATCTCTTTCTT

Ania_ruybarrettoi_KY966707 ACAAGAATTCTTTTTCTTCTCATTTTTCT------------TCTCAAATGGTATCAGAAGGTTTTGGAGTCATTCTGGAAATTCCATTCTCGTCGCGATTAGTATCT---------TCCCTTGAAG---AAAAAAGAATACCAAAATCTCAGAATTTACGATCTATTCATTCAATATTTCCCTTTTTAGAGGATAAATTATCACATTTAAATTATGTGTCAG-ATC-TACT-AATACCCCATCCCATCCATCTGGAAATCTTGGTTCAAATCCTTCAATGCTGGATCAAAGATGTTCCTTCTTTGCATTTATTGCGATTGTTTTTCCACGAATATCATAATTTGAATAGTCTCATTACTTC----------AAAGAAATCCATTTACGTCTTTTCAAAAAGAAA---GAAAAGA-TTCTTTTGG-TTCCTACATAATTCTTATGTATATGAATGCGAATATCTATTCCTGTTTCTTCGTAAACAGTCTTCTTATTTACGATCAATATCTTCTGGAGTCTTTCTTGAGCGAACACATTTCTATGGAAAAATAGAATATCTTATAGT---CGTGTGTTGTAATTCTTTTCA---------GAGGATCCTATGGTTCCTCAA-AGATACTTTCATACATTATGTTCGATATCAAGGAAAAGCGATTATGGCTTCAAAAGGAACTCTTATTCTGATGAAGAAAT-GGAAATTTCATCTTGTAAATTTTTGGCAATCTTATTTTCACTTTTGGTTTCAACCTTATAGGATCCATATAAAGCAATTACCCAACTATTTCTTCTCTTTTCTGGGG-TATTTTTCAAGTGTACTAAAAAACCCTTTGGTAGTAA-GAAATCAAATGCTAGAGAATTCATTT-CTAATAAATACTCT-GACTAAGAAATTAGATACCATAGCCCCAGTTATTTCTCTTATTGGATCATTGTCGAAAGCTCAATTTTGTACTGTATTGGGTCATCCTATTAGTAAACCAATCTGGACCGATTTATCGGATTCTGATATTCTTGATCGATTTTGTCGGATATCTAGAAATCTTTGTCGTTATCACAGCGGATCCTCAAAGAAACAGGTTTTGTATCGTATAAAGTATATACTTCGACTTTCGTGTGCTAGAACTTTGGCTCGTAAACATAAAAGTACAGTACGCACTTTTATGCGAAGATTAGGTTCGGGATTCTTAG-AAGAATTTTT--------------------------

Ania_ruybarrettoi_SG1395 ACAAGAATTCTTTTTCTTCTCATTTTTCT------------TCTCAAATGGTATCAGAAGGTTTTGGAGTCATTCTGGAAATTCCATTCTCGTCGCGATTAGTATCT---------TCCCTTGAAG---AAAAAAGAATACCAAAATCTCAGAATTTACGATCTATTCATTCAATATTTCCCTTTTTAGAGGATAAATTATCACATTTAAATTATGTGTCAG-ATC-TACT-AATACCCCATCCCATCCATCTGGAAATCTTGGTTCAAATCCTTCAATGCTGGATCAAAGATGTTCCTTCTTTGCATTTATTGCGATTGTTTTTCCACGAATATCATAATTTGAATAGTCTCATTACTTC----------AAAGAAATCCATTTACGTCTTTTCAAAAAGAAA---GAAAAGA-TTCTTTTGG-TTCCTACATAATTCTTATGTATATGAATGCGAATATCTATTCCTGTTTCTTCGTAAACAGTCTTCTTATTTACGATCAATATCTTCTGGAGTCTTTCTTGAGCGAACACATTTCTATGGAAAAATAGAATATCTTATAGT---CGTGTGTTGTAATTCTTTTCA---------GAGGATCCTATGGTTCCTCAA-AGATACTTTCATACATTATGTTCGATATCAAGGAAAAGCGATTATGGCTTCAAAAGGAACTCTTATTCTGATGAAGAAAT-GGAAATTTCATCTTGTAAATTTTTGGCAATCTTATTTTCACTTTTGGTTTCAACCTTATAGGATCCATATAAAGCAATTACCCAACTATTTCTTCTCTTTTCTGGGG-TATTTTTCAAGTGTACTAAAAAACCCTTTGGTAGTAA-GAAATCAAATGCTAGAGAATTCATTT-CTAATAAATACTCT-GACTAAGAAATTAGATACCATAGCCCCAGTTATTTCTCTTATTGGATCATTGTCGAAAGCTCAATTTTGTACTGTATTGGGTCATCCTATTAGTAAACCAATCTGGACCGATTTATCGGATTCTGATATTCTTGATCGATTTTGTCGGATATCTAGAAATCTTTGTCGTTATCACAGCGGATCCTCAAAGAAACAGGTTTTGTATCGTATAAAGTATATACTTCGACTTTCGTGTGCTAGAACTTTGGCTCGTAAACATAAAAGTACAGTACGCACTTTTATGCGAAGATTAGGTTCGGGATTCTTAG-AAGAATTTTTTTTGGAAGAAGAACAATCTCTTTCTT

Anoectochilus_formosanus_EU797513 ATAAGAATTATTTTTCTTCTCATTTTTCT------------TTTCAAATACTATCAGAAGGTTTTGGAGTCGTTCTGGAAATTCCATTATCGTCGCGATTAGTATTC---------TCCCTTGAAG---AAAAAAAAATACCAAAATATCAGAATTTACGATCTATTCATTCAATATTTCCTTTTTTAGAGGATAAATTTTCACATTTAAATTCTGTGTCAG-ATC-TATT-AATACCCCATCCCATCCATCTGGAAATCTTGGTTCAAATCCTTCAATGCTGGATCAAAGATGTTCCTTCTTTGCATTTGTTGCGATTTATTTTCCACGAATATCATAATTTGAAGAGTATCATTACTTC----------AAATAAATCCATTCACGTTTTTTCAAAAAAAAA---GAAAAGA-ATTTTTTGG-TTCCTACATAATTTTTATGTATATGAATGCGAATATCTCTTTCTTTTTCTTCGTAAAAATTCTTCTTATTTACGATCAACATCTTTTGGAGTCTTTATTGAGCGAACACTTTTTTATGTAAAAATGGAATCTATTCTAGT---AGTATATTTTAATTCTTTTCA---------GAGGATTCTCTGGTTCCTCAA-AGATCCTTTCATACATTATGTTCGATATCAAGGAAAAGTAATTCTGACTTCAAAGGTAACTCTTATTCTGATGAAGAAAT-GGAATTTTCATGTTGTGAATTTTTGTCAATTTTATTTTCACTTTTGGTCTCAACTTTATAGGATCCATATAAAGCAATTACCCAACTATTCCTTCTCTTTTCTGGGG-TATTTTTTAAGTGTACAAAAAAAAACTTTGGTAGTAA-GAAATCAAATGCTAGAGAATTCCTTT-CTAATAAATACTAT-GACTAAGAAATTAGATACCGTAGCCCCAGTTATTTCTCTTATTGGATCATTGTCGAAAGCTCAATTTTGTACTATATCAGGTCATCCTATTAGTAAACCCATTTGGACTGATTTTTCGGATTCTGATATTATTGATCGATTTTGTCGGAAATGTAGAAATCTTTGTCGTTATCACAGCGGATCCTCAAAAAAAAAAGTTTTGTATCGTATAAAATATATATTTCGACTTTCGTGTGCTAGAACTTTGGCTCGTAAACATAAAAGTACAGTACGCACTTTTATGCGAAGATTGGGTTCGGTATTTTTAG-AAGAATTTTTTATGGAAGAAGAACAAGTTCTTTCTT

Anoectochilus_formosanus_MG490281 ATAAGAATTATTTTTCTTCTCATTTTTCT------------TTTCAAATACTATCAGAAGGTTTTGGAGTCGTTCTGGAAATTCCATTATCGTCGCGATTAGTATTC---------TCCCTTGAAG---AAAAAAAAATACCAAAATATCAGAATTTACGATCTATTCATTCAATATTTCCTTTTTTAGAGGATAAAATTTCACATTTAAATTCTGTGTCAG-ATC-TATT-AATACCCCATCCCATCCATCTGGAAATCTTGGTTCAAATCCTTCAATGCTGGATCAAAGATGTTCCTTCTTTGCATTTGTTGCGATTTATTTTCCACGAATATCATAATTTGAAGAGTATCATTACTTC----------AAATAAATCCATTCACGTTTTTTCAAAAAAAAA---GAAAAGA-ATTTTTTGG-TTCCTACATAATTTTTATGTATATGAATGCGAATATCTCTTTCTTTTTCTTCGTAAAAATTCTTCTTATTTACGATCAACATCTTTTGGAGTCTTTATTGAGCGAACACTTTTTTATGTAAAAATGGAATCTATTCTAGT---AGTATATTTTAATTCTTTTCA---------GAGGATTCTCTGGTTCCTCAA-AGATCCTTTCATACATTATGTTCGATATCAAGGAAAAGTAATTCTGACTTCAAAGGTAACTCTTATTCTGATGAAGAAAT-GGAATTTTCATGTTGTGAATTTTTGTCAATTTTATTTTCACTTTTGGTCTCAACCTTATAGGATCCATATAAAGCAATTACCCAACTATTCCTTCTCTTTTCTGGGG-TATTTTTTAAGTGTACAAAAAAAAACTTTGGTAGTAA-GAAATCAAATGCTAGAGAATTCCTTT-CTAATAAATACTAT-GACTAAGAAATTAGATACCATAGCCCCAGTTATTTCTCTTATTGGATCATTGTCGAAAGCTCAATTTTGTACTATATCAGGTCATCCTATTAGTAAACCCATTTGGACTGATTTTTCGGATTCTGATATTATTGATCAATTTTGTCGGAAATGTAGAAATCTTTGTCGTTATCACAGCGGATCCTCAAAAAAAAAAG----------------------------------------------------------------------------------------------------------------------------------------------------

Anoectochilus_formosanus_PK12215 ATAAGAATTATTTTTCTTCTCATTTTTCT------------TTTCAAATACTATCAGAAGGTTTTGGAGTCGTTCTGGAAATTCCATTATCGTCGCGATTAGTATTC---------TCCCTTGAAG---AAAAAAAAATACCAAAATATCAGAATTTACGATCTATTCATTCAATATTTCCTTTTTTAGAGGATAAATTTTCACATTTAAATTCTGTGTCAG-ATC-TATT-AATACCCCATCCCATCCATCTGGAAATCTTGGTTCAAATCCTTCAATGCTGGATCAAAGATGTTCCTTCTTTGCATTTGTTGCGATTTATTTTCCACGAATATCATAATTTGAAGAGTATCATTACTTC----------AAATAAATCCATTCACGTTTTTTCAAAAAAAAA---GAAAAGA-ATTTTTTGG-TTCCTACATAATTTTTATGTATATGAATGCGAATATCTCTTTCTTTTTCTTCGTAAAAATTCTTCTTATTTACGATCAACATCTTTTGGAGTCTTTATTGAGCGAACACTTTTTTATGTAAAAATGGAATCTATTCTAGT---AGTATATTTTAATTCTTTTCA---------GAGGATTCTCTGGTTCCTCAA-AGATCCTTTCATACATTATGTTCGATATCAAGGAAAAGTAATTCTGACTTCAAAGGTAACTCTTATTCTGATGAAGAAAT-GGAATTTTCATGTTGTGAATTTTTGTCAATTTTATTTTCACTTTTGGTCTCAACCTTATAGGATCCATATAAAGCAATTACCCAACTATTCCTTCTCTTTTCTGGGGGTATTTTTTAAGTGTACAAAAAAAAACTTTGGTAGTAA-GAAATCAAATGCTAGAGAATTCCTTT-CTAATAAATACTAT-GACTAAGAAATTAGATACCGTAGCCCCAGTTATTTCTCTTATTGGATCATTGTCGAAAGCTCAATTTTGTACTATATCAGGTCATCCTATTAGTAAACCCATTTGGACTGATTTTTCGGATTTTGATATTATTGATCGATTTTGTCGGAAAAGTAGAAATCTTTGTCGTTATCCCAGCGGATCCTCAAAAAAAAAAGTTTTGTATCGTATAAAATATATATTTCGACTTTCGTGTGCTAGAACTTTGGCTCGTAAACATAAAAGTACAGTACGCACTTTTATGCGAAGATTGGGTTCGGTATTTTTAG-AAGAATTTTTTATGGAAGAAGAACAAGTTCTTTCTT

Anoectochilus_roxburghii_EU817409 ATAAGAATTATTTTTCTTCTCATTTTTCT------------TTTCAAATACTATCAGAAGGTTTTGGAGTCGTTCTGGAAATTCCATTATCGTCGCGATTAGTATTC---------TCCCTTGAAG---AAAAAAAAATACCAAAATATCAGAATTTACGATCTATTCATTCAATATTTCCTTTTTTAGAGGATAAATTTTCACATTTAAATTCTGTGTCAG-ATC-TATT-AATACCCCATCCCATCCATCTGGAAATCTTGGTTCAAATCCTTCAATGCTGGATCAAAGATGTTCCTTCTTTGCATTTGTTGCGATTTATTTTCCACGAATATCATAATTTGAAGAGTATCATTACTTC----------AAAGAAATCCATTCACGTTTTTTCAAAAAAAAA---GAAAAGA-TTTTTTTGG-TTCCTACATAATTTTTATGTATATGAATGCGAATATCTCTTTCTTTTTCTTCGTAAAAATTCTTCTTATTTACGATCAACATCTTTTGGAGTCTTTATTGAGCGAACACTTTTTTATGTAAAAATGGAATCTATTCTAGT---AGTATATTTTAATTCTTTTCA---------GAGGATTCTCCGGT-CCTCAA-AGATCCTTTCATACATTATGTTCGATATCAAGGAAAGGTAATTCTGACTTCAAAGGGAACTCTTATTCTGATGAAGAAAT-GGAATTTTCATGTTGTGAATTTTTGTCAATTTTATTTTCACTTTTGGTCTCAACCTTATAGGATCCATATAAAGCAATTACCCAACTATTCCTTCTCTTTTCTGGGG-TATTTTTTAAGTGTACAAAAAAAAACTTTGGTAGTAA-GAAATCAAATGCTAGAGAATTCCTTT-CTAATAAATACTAT-GACTAAGAAATTAGATACCATAGCCCCAGTTATTTCTCTTATTGGATCATTGTCGAAAGCTCAATTTTGTACTATATCGGGTCATCCTATTAGTAAACCCATTTGGACTGATTTTTCGGATTCTGATATTATTGATCTATTTTGTCGGAAATGTAAAAATCTTTGTCGTTATCACAGCGGATCCTCAAAAAAAAAAGTTTTGTATCGTATAAAATATATATTTCGACTTTCGTGTGCTAGAACTTTAGCTCGTAAACATAAAAGTACAGTACGCACTTTTATGCGAAGATTGGGTTCGGTATTTTTAG-AAGAATTTTTTATGGAAGAAGAACAAGTTCTTTCTT

Anoectochilus_roxburghii_KF361656 -----------------------------------------------------------------------------------------------------------------------------------------------------------------------------------------------------------------AATTCTGTGTCAG-ATC-TATT-AATACCCCATCCCATCCATCTGGAAATCTTGGTTCAAATCCTTCAATGCTGGATCAAAGATGTTCCTTCTTTGCATTTGTTGCGATTTATTTTCCACGAATATCATAATTTGAAGAGTATCATTACTTC----------AAATAAATCCATTCACGTTTTTTCAAAAAAAAA---GAAAAGA-ATTTTTTGG-TTCCTACATAATTTTTATGTATATGAATGCGAATATCTCTTTCTTTTTCTTCGTAAAAATTCTTCTTATTTACGATCAACATCTTTTGGAGTCTTTATTGAGCGAACACTTTTTTATGTAAAAATGGAATCTATTCTAGT---AGTATATTTTAATTCTTTTCA---------GAGGATTCTCTGGTTCCTCAA-AGATCCTTTCATACATTATGTTCGATATCAAGGAAAAGTAATTCTGACTTCAAAGGTAACTCTTATTCTGATGAAGAAAT-GGAATTTTCATGTTGTGAATTTTTGTCAATTTTATTTTCACTTTTGGTCTCAACCTTATAGGATCCATATAAAGCAATTACCCAACTATTCCTTCTCTTTTCTGGGG-TATTTTTTAAGTGTACAAAAAAAAACTTTGGTAGTAA-GAAATCAAATGCTAGAGAATTCCTTT-CTAATAAATACTAT-GACTAAGAAATTAGATACCATAGCCCCAGTTATTTCTCTTATTGGATCATTGTCGAAAGCTCAATTTTGTACTATATCAGGTCATCCTATTAGTAAACCCATTTGGACTGATTTTTCGGATTCTGATATTATTGATCAATTTTGTCGGAAATGTAGAAATCTTTGTCGTTATCACAGCGGATCCTCAAAAAAAAAAGTTTTGTAT--------------------------------------------------------------------------------------------------------------------------------------------

Anoectochilus_roxburghii_KY966708 ATAAGAATTATTTTTCTTCTCATTTTTCT------------TTTCAGATACTATCAGAAGGTTTTGGAGTCGTTCTGGAAATTCCATTATCGTCGCGATTAGTATTC---------TCCCTTGAAG---AAAAAAAAATACCAAAATATCAGAATTTACGATCTATTCATTCAATATTTCCTTTTTTAGAGGATAAAATTTCACATTTAAATTCTGTGTCAG-ATC-TATT-AATACCCCATCCCATCCATCTGGAAATCTTGGTTCAAATCTTTCAATGCTGGATCAAAGATGTTCCTTCTTTGCATTTGTTGCGATTTATTTTCCACGAATATCATAATTTGAAGAGTATCATTACTTC----------AAATAAATCCATTCACGTTTTTTCAAAAAAAAA---GAAAAGA-ATTTTTTGG-TTCCTACATAATTTTTATGTATATGAATGCGAATATCTCTTTCTTTTTCTTCGTAAAAATTCTTCTTATTTACGATCAACATCTTTTGGAGTCTTTATTGAGCGAACACTTTTTTATGTAAAAATGGAATCTATTCTAGT---AGTATATTTTAATTCTTTTCA---------GAGGATTCTCTGGTTCCTCAA-AGATCCTTTCATACATTATGTTCGATATCAAGGAAAAGTAATTCTGACTTCAAAGGTAACTCTTATTCTGATGAAGAAAT-GGAATTTTCATGTTGTGAATTTTTGTCAATTTTATTTTCACTTTTGGTCTCAACCTTATAGGATCCATATAAAGCAATTACCCAACTATTCCTTCTCTTTTCTGGGG-TATTTTTTAAGTGTACAAAAAAAAACTTTGGTAGTAA-GAAATCAAATGCTAGAGAATTCCTTT-CTAATAAATACTAT-GACTAAGAAATTAGATACCATAGCCCCAGTTATTTCTCTTATTGGATCATTGTCGAAAGCTCAATTTTGTACTATATCAGGTCATCCTATTAGTAAACCCATTTGGACTGATTTTTCGGATTCTGATATTATTGATCAATTTTGTCGGAAATGTAGAAATCTTTGTCGTTATCACAGCGGATCCTCAAAAAAAAAAGTTTTGTATCGTATAAAATATATATTTCGACTTTCGTGTGCTAGAACTTTGGCTCGTAAACATAAAAGTACAGTACGCACTTTTATGCGAAGATTGGGTTCGGTATTTTTAG-AAGAATTTTT--------------------------

Anoectochilus_roxburghii_PK12043 ATAAGAATTATTTTTCTTCTCATTTTTCT------------TTTCAAATACTATCAGAAGGTTTTGGAGTCGTTCTGGAAATTCCATTATCGTCGCGATTAGTATTC---------TCCCTTGAAG---AAAAAAAAATACCAAAATATCAGAATTTACGATCTATTCATTCAATATTTCCTTTTTTAGAGGATAAAATTTCACATTTAAATTCTGTGTCAG-ATC-TATT-AATACCCCATCCCATCCATCTGGAAATCTTGGTTCAAATCCTTCAATGCTGGATCAAAGATGTTCCTTCTTTGCATTTGTTGCGATTTATTTTCCACGAATATCATAATTTGAAGAGTATCATTACTTC----------AAATAAATCCATTCACGTTTTTTCAAAAAAAAA---GAAAAGA-ATTTTTTGG-TTCCTACATAATTTTTATGTATATGAATGCGAATATCTCTTTCTTTTTCTTCGTAAAAATTCTTCTTATTTACGATCAACATCTTTTGGAGTCTTTATTGAGCGAACACTTTTTTATGTAAAAATGGAATCTATTCTAGT---AGTATATTTTAATTCTTTTCA---------GAGGATTCTCTGGTTCCTCAA-AGATCCTTTCATACATTATGTTCGATATCAAGGAAAAGTAATTCTGACTTCAAAGGTAACTCTTATTCTGATGAAGAAAT-GGAATTTTCATGTTGTGAATTTTTGTCAATTTTATTTTCACTTTTGGTCTCAACCTTATAGGATCCATATAAAGCAATTACCCAACTATTCCTTCTCTTTTCTGGGG-TATTTTTTAAGTGTACAAAAAAAAACTTTGGTAGTAA-GAAATCAAATGCTAGAGAATTCCTTT-CTAATAAATACTAT-GACTAAGAAATTAGATACCATAGCCCCAGTTATTTCTCTTATTGGATCATTGTCGAAAGCTCAATTTTGTACTATATCAGGTCATCCTATTAGTAAACCCATTTGGACTGATTTTTCGGATTCTGATATTATTGATCAATTTTGTCGGAAATGTAGAAATCTTTGTCGTTATCACAGCGGATCCTCAAAAAAAAAAGTTTTGTATCGTATAAAATATATATTTCGACTTTCGTGTGCTAGAACTTTGGCTCGTAAACATAAAAGTACAGTACGCACTTTTATGCGAAGATTGGGTTCGGTATTTTTAG-AAGAATTTTTTATGGAAGAAGAACAAGTTCTTTCTT

Anoectochilus_roxburghii_PK12068 ATAAGAATTATTTTTCTTCTCATTTTTCT------------TTTCAAATACTATCAGAAGGTTTTGGAGTCGTTCTGGAAATTCCATTATCGTCGCGATTAGTATTC---------TCCCTTGAAG---AAAAAAAAATACCAAAATATCAGAATTTACGATCTATTCATTCAATATTTCCTTTTTTAGAGGATAAATTTTCACATTTAAATTCTGTGTCAG-ATC-TATT-AATACCCCATCCCATCCATCTGGAAATCTTGGTYCAAATCCTTCAATGCTGGATCAAAGATGTTCCTTCTTTGCATTTGTTGCGATTTATTTTCCACGAATATCATAATTTGAAGAGTATCATTACTTC----------AAATAAATCCATTCACGTTTTTTCAAAAAAAAA---GAAAAGA-ATTTTTTGG-TTCCTACATAATTTTTATGTATATGAATGCGAATATCTCTTTCTTTTTCTTCGTAAAAATTCTTCTTATTTACGATCAACATCTTTTGGAGTCTTTATTGAGCGAACACTTTTTTATGTAAAAATGGAATCTATTCTAGT---AGTATATTTTAATTCTTTTCA---------GAGGATTCTCTGGTTCCTCAA-AGATCCTTTCATACATTATGTTCGATATCAAGGAAAAGTAATTCTGACTTCAAAGGTAACTCTTATTCTGATGAAGAAAT-GGAATTTTCATGTTGTGAATTTTTGTCAATTTTATTTTCACTTTTGGTCTCAACCTTATAGGATCCATATAAAGCAATTACCCAACTATTCCTTCTCTTTTCTGGGG-TATTTTTTAAGTGTACAAAAAAAAACTTTGGTAGTAA-GAAATCAAATGCTAGAGAATTCCTTT-CTAATAAATACTAT-GACTAAGAAATTAGATACCATAGCCCCAGTTATTTCTCTTATTGGATCATTGTCGAAAGCTCAATTTTGTACTATATCAGGTCATCCTATTAGTAAACCCATTTGGACTGATTTTTCGGATTCTGATATTATTGATCAATTTTGTCGGAAATGTAGAAATCTTTGTCGTTATCACAGCGGATCCTC-AAAAAAAAAGTTTTGTATCGTATAAAATATATATTTCGACTTTCGTGTGCTAGAACTTTGGCTCGTAAACATAAAAGTACAGTACGCACTTTTATGCGAAGATTGGGTTCGGTATTTTTAG-AAGAATTTTTTATGGAAGAAGAACAAGTTCTTTCTT

Anoectochilus_roxburghii_PK12069 ATAAGAATTATTTTTCTTCTCATTTTTCT------------TTTCAAATACTATCAGAAGGTTTTGGAGTCGTTCTGGAAATTCCATTATCGTCGCGATTAGTATTC---------TCCCTTGAAG---AAAAAAAAATACCAAAATATCAGAATTTACGATCTATTCATTCAATATTTCCTTTTTTAGAGGATAAAATTTCACATTTAAATTCTGTGTCAG-ATC-TATT-AATACCCCATCCCATCCATCTGGAAATCTTGGTTCAAATCCTTCAATGCTGGATCAAAGATGTTCCTTCTTTGCATTTGTTGCGATTTATTTTCCACGAATATCATAATTTGAAGAGTATCATTACTTC----------AAATAAATCCATTCACGTTTTTTCAAAAAAAAA---GAAAAGA-ATTTTTTGG-TTCCTACATAATTTTTATGTATATGAATGCGAATATCTCTTTCTTTTTCTTCGTAAAAATTCTTCTTATTTACGATCAACATCTTTTGGAGTCTTTATTGAGCGAACACTTTTTTATGTAAAAATGGAATCTATTCTAGT---AGTATATTTTAATTCTTTTCA---------GAGGATTCTCTGGTTCCTCAA-AGATCCTTTCATACATTATGTTCGATATCAAGGAAAAGTAATTCTGACTTCAAAGGTAACTCTTATTCTGATGAAGAAAT-GGAATTTTCATGTTGTGAATTTTTGTCAATTTTATTTTCACTTTTGGTCTCAACCTTATAGGATCCATATAAAGCAATTACCCAACTATTCCTTCTCTTTTCTGGGG-TATTTTTTAAGTGTACAAAAAAAAACTTTGGTAGTAA-GAAATCAAATGCTAGAGAATTCCTTT-CTAATAAATACTAT-GACTAAGAAATTAGATACCATAGCCCCAGTTATTTCTCTTATTGGATCATTGTCGAAAGCTCAATTTTGTACTATATCAGGTCATCCTATTAGTAAACCCATTTGGACTGATTTTTCGGATTCTGATATTATTGATCAATTTTGTCGGAAATGTAGAAATCTTTGTCGTTATCACAGCGGATCCTCAAAAAAAAAAGTTTTGTATCGTATAAAATATATATTTCGACTTTCGTGTGCTAGAACTTTGGCTCGTAAACATAAAAGTACAGTACGCACTTTTATGCGAAGATTGGGTTCGGTATTTTTAG-AAGAATTTTTTATGGAAGAAGAACAAGTTCTTTCTT

Anoectochilus_roxburghii_SG1219 ATAAGAATTATTTTTCTTCTCATTTTTCT------------TTTCAAATACTATCAGAAGGTTTTGGAGTCGTTCTGGAAATTCCATTATCGTCGCGATTAGTATTC---------TCCCTTGAAG---AAAAAAAAATACCAAAATATCAGAATTTACGATCTATTCATTCAATATTTCCTTTTTTAGAGGATAAAATTTCACATTTAAATTCTGTGTCAG-ATC-TATT-AATACCCCATCCCATCCATCTGGAAATCTTGGTTCAAATCCTTCAATGCTGGATCAAAGATGTTCCTTCTTTGCATTTGTTGCGATTTATTTTCCACGAATATCATAATTTGAAGAGTATCATTACTTC----------AAATAAATCCATTCACGTTTTTTCAAAAAAAAA---GAAAAGA-ATTTTTTGG-TTCCTACATAATTTTTATGTATATGAATGCGAATATCTCTTTCTTTTTCTTCGTAAAAATTCTTCTTATTTACGATCAACATCTTTTGGAGTCTTTATTGAGCGAACACTTTTTTATGTAAAAATGGAATCTATTCTAGT---AGTATATTTTAATTCTTTTCA---------GAGGATTCTCTGGTTCCTCAA-AGATCCTTTCATACATTATGTTCGATATCAAGGAAAAGTAATTCTGACTTCAAAGGTAACTCTTATTCTGATGAAGAAAT-GGAATTTTCATGTTGTGAATTTTTGTCAATTTTATTTTCACTTTTGGTCTCAACCTTATAGGATCCATATAAAGCAATTACCCAACTATTCCTTCTCTTTTCTGGGG-TATTTTTTAAGTGTACAAAAAAAAACTTTGGTAGTAA-GAAATCAAATGCTAGAGAATTCCTTT-CTAATAAATACTAT-GACTAAGAAATTAGATACCATAGCCCCAGTTATTTCTCTTATTGGATCATTGTCGAAAGCTCAATTTTGTACTATATCAGGTCATCCTATTAGTAAACCCATTTGGACTGATTTTTCGGATTCTGATATTATTGATCAATTTTGTCGGAAATGTAGAAATCTTTGTCGTTATCACAGCGGATCCTCAAAAAAAAAAGTTTTGTATCGTATAAAATATATATTTCGACTTTCGTGTGCTAGAACTTTGGCTCGTAAACATAAAAGTACAGTACGCACTTTTATGCGAAGATTGGGTTCGGTATTTTTAG-AAGAATTTTTTATGGAAGAAGAACAAGTTCTTTCTT

Aphyllorchis_montana_PK12147 ACAAGAATTCCTTTTCTTCTCATTTTTCT------------TCTCAAATGGTATCAGAAGGTTTTGGAATCATTCTGGAAATTCCATTCTCGTCGCGATTAGTATCT---------TCCCTTGAAG---AAAAAAAAAGACCAAAATCTCAGAATTTACGATCTATTCATTCAATATTTCCCTTTTTAGAGGATAAATTCTCGCATTTAAATTATGTGTCAG-ATC-TAAT-AATACCCCATCCCCTCCATCTGGAAATCTTGGTTCAAATCCTTCAATGCTGGATCAAAGATGTTCCTTCTTTGCATTTATTGCGATCTTTTTTCCACGAATATCATAATTTGAATAGTCTCATTACTTC----------AAAGAAATCCATTCATGTCTTTTCGAAAAGAAA---GAAAAGA-TTCTTTTGG-TTCCTACATAATTCTTATGTATATGAATGCGAATATATATTCCTTTTTCTTCGTAAAGAGTCTTCTTATTTACGATCAACATCTTCTGGAGTCTTTCTTGAGCGAACACATTTCTATGGAAAAATAGAATATCTTCTAGT---AGTGTGTTTTAATTCTTTTCG---------GAGGATTCTATGGTTCCTCAA-AGATCCTTTCATACATTATGCTCGATATCAAGGAAAAGCAATTATGGCTTCAAAGGGAACTCTTATTCTGATGAAGAAAT-GGAAATTTCATCTTGTGAATCTTTGGCAATCTTATTTTCACTTTTGGTCTCAACCTTATAGGATCCATATAAAGCAATTACCCAACTCTTCCTTCTCTTTTCTGGGG-TATTTTTCAAGTGTACTAAAAAATACTTTGGTAGTAA-GAAATCAAATGCTAGAGAATTCATTT-CTAATAAATACTCT-GACTAAAAAATTCGATACCATAGCCCCAGTTATTTCTCTTATTGGATCATTGTCGAAAGCTCAATTTTGTACTGTATTGGGTCATCCTATTAGTAAGCCGATCTGGACCGATTTATCGGATTCTGATATTCTTGATCGATTTTGTCGGATATGTAGAAATCTTTGTCGTTATCACAGCGGATCCTCAAAGAAACAGGTTTTGTATCGTATAAAGTATATACTTCGACTTTCGTGTGCTAGAACTTTGGCTCGTAAACATAAAAGTACAGTACGCACTTTTATGCGAAGATTAGGTTCGGGATTCTTAG-AAGAATTTTTTTTGGAAGAAGAAAAAGTTCTTTCTT

Aphyllorchis_montana_PK12148 ACAAGAATTCCTTTTCTTCTCATTTTTCT------------TCTCAAATGGTATCAGAAGGTTTTGGAATCATTCTGGAAATTCCATTCTCGTCGCGATTAGTATCT---------TCCCTTGAAG---AAAAAAAAAGACCAAAATCTCAGAATTTACGATCTATTCATTCAATATTTCCCTTTTTAGAGGATAAATTCTCGCATTTAAATTATGTGTCAG-ATC-TAAT-AATACCCCATCCCCTCCATCTGGAAATCTTGGTTCAAATCCTTCAATGCTGGATCAAAGATGTTCCTTCTTTGCATTTATTGCGATCTTTTTTCCACGAATATCATAATTTGAATAGTCTCATTACTTC----------AAAGAAATCCATTCATGTCTTTTCGAAAAGAAA---GAAAAGA-TTCTTTTGG-TTCCTACATAATTCTTATGTATATGAATGCGAATATATATTCCTTTTTCTTCGTAAAGAGTCTTCTTATTTACGATCAACATCTTCTGGAGTCTTTCTTGAGCGAACACATTTCTATGGAAAAATAGAATATCTTCTAGT---AGTGTGTTTTAATTCTTTTCG---------GAGGATTCTATGGTTCCTCAA-AGATCCTTTCATACATTATGCTCGATATCAAGGAAAAGCAATTATGGCTTCAAAGGGAACTCTTATTCTGATGAAGAAAT-GGAAATTTCATCTTGTGAATCTTTGGCAATCTTATTTTCACTTTTGGTCTCAACCTTATAGGATCCATATAAAGCAATTACCCAACTCTTCCTTCTCTTTTCTGGGG-TATTTTTCAAGTGTACTAAAAAATACTTTGGTAGTAA-GAAATCAAATGCTAGAGAATTCATTT-CTAATAAATACTCT-GACTAAAAAATTCGATACCATAGCCCCAGTTATTTCTCTTATTGGATCATTGTCGAAAGCTCAATTTTGTACTGTATTGGGTCATCCTATTAGTAAGCCGATCTGGACCGATTTATCGGATTCTGATATTCTTGATCGATTTTGTCGGATATGTAGAAATCTTTGTCGTTATCACAGCGGATCCTCAAAGAAACAGGTTTTGTATCGTATAAAGTATATACTTCGACTTTCGTGTGCTAGAACTTTGGCTCGTAAACATAAAAGTACAGTACGCACTTTTATGCGAAGATTAGGTTCGGGATTCTTAG-AAGAATTTTTTTTGGAAGAAGAAAAAGTTCTTTCTT

Aphyllorchis_montana_SG1010 ACAAGAATTCCTTTTCTTCTCATTTTTCT------------TCTCAAATGGTATCAGAAGGTTTTGGAATCATTCTGGAAATTCCATTCTCGTCGCGATTAGTATCT---------TCCCTTGAAG---AAAAAAAAAGACCAAAATCTCAGAATTTACGATCTATTCATTCAATATTTCCCTTTTTAGAGGATAAATTCTCGCATTTAAATTATGTGTCAG-ATC-TAAT-AATACCCCATCCCCTCCATCTGGAAATCTTGGTTCAAATCCTTCAATGCTGGATCAAAGATGTTCCTTCTTTGCATTTATTGCGATCTTTTTTCCACGAATATCATAATTTGAATAGTCTCATTACTTC----------AAAGAAATCCATTCATGTCTTTTCGAAAAGAAA---GAAAAGA-TTCTTTTGG-TTCCTACATAATTCTTATGTATATGAATGCGAATATATATTCCTTTTTCTTCGTAAAGAGTCTTCTTATTTACGATCAACATCTTCTGGAGTCTTTCTTGAGCGAACACATTTCTATGGAAAAATAGAATATCTTCTAGT---AGTGTGTTTTAATTCTTTTCG---------GAGGATTCTATGGTTCCTCAA-AGATCCTTTCATACATTATGCTCGATATCAAGGAAAAGCAATTATGGCTTCAAAGGGAACTCTTATTCTGATGAAGAAAT-GGAAATTTCATCTTGTGAATCTTTGGCAATCTTATTTTCACTTTTGGTCTCAACCTTATAGGATCCATATAAAGCAATTACCCAACTCTTCCTTCTCTTTTCTGGGG-TATTTTTCAAGTGTACTAAAAAATACTTTGGTAGTAA-GAAATCAAATGCTAGAGAATTCATTT-CTAATAAATACTCT-GACTAAAAAATTCGATACCATAGCCCCAGTTATTTCTCTTATTGGATCATTGTCGAAAGCTCAATTTTGTACTGTATTGGGTCATCCTATTAGTAAGCCGATCTGGACCGATTTATCGGATTCTGATATTCTTGATCGATTTTGTCGGATATGTAGAAATCTTTGTCGTTATCACAGCGGATCCTCAAAGAAACAGGTTTTGTATCGTATAAAGTATATACTTCGACTTTCGTGTGCTAGAACTTTGGCTCGTAAACATAAAAGTACAGTACGCACTTTTATGCGAAGATTAGGTTCGGGATTCTTAG-AAGAATTTTTTTTGGAAGAAGAAAAAGTTCTTTCTT

Apostasia_nipponica_PK12273 ACAAGAATTATTTTTCTTCTAATTTTGCT------------TTTCAAATGGCATCAGAAGGTTTTGGAGTCATTCTGGAAATTCCATTCTCGTCGCGATTAGTTTCT---------TCCCCTGAAG---AAATAAAAATACCAAAATCTCTGAATTTACGATCTATTCATTCAATATTTCCTTTTTTAGAGGACAAATTCTTACATTTAAATTATGTATTAG-GTA-TACT-AATACCCCACCCCATCCATCTGGGAATCTTGGTTCAAATCCTTCAATGCTGGATCAAAGATGCTCCTTCTTTGCATTTATTGTTAAAAATTTTTCACGAATATTATAATTCGAATAGTCTTTTTACTTC----------------------------------------AAAAAAAAGAAGA---TTTTTGGCTTCTTATATAATTCTTATATATGTGAATGCGAATTTCTTTTACTATTTTTTCGTAAACAGTCTTCCTACTTACGATCAACATCTTCTGGAGTTTTTCTTGAACGAATGCATTTCTATGGAAAAATAGAGTATCTTGTAGT------ATGTTT-AATTCTTTTCA---------AAGGATCCCATGCTTCTTCAA-AAATCTTTTCATGCATTATGTTCGATATCAAGGAAAAGGAATTCTGGCTTCAAAGGGTACTCTTATTCTGATGAAAAAAT-GGAAATATCATCTTGTAAATTTTTGGCAATCTTATTTTCGCTTTTGGTCTCAACCATATAGGATACATATAAAACAATTATTCAACTATTCCTTTTCGTTTATGGGG-TATTTTTCAAGTGTACTAAGATATTCTTTGGTAGTAA-GAAATCAAATGCTAGAGAATTCATTT-CTCATGGATATTCT-GATTAAGAAATTAGATACCATAGTTCCAGTTGTTTCTCTTATTGGATCAATGTCGAAAGCCCAATTTTGTACTGTATTGGGTCATCCTATTAGTAAACCGATTTGGACTGATTTTTCAGATTCTGATATTCTTGATCGATTTTGTAGAATATGTAAAAATCTTTGTCGTTTTTACAGTGGATCCTCAAAAAAACATGTTTTGTATCATATAAAATATATACTTCGACTTTCGTGTGCTAGAACTTTAGCTCGTAAACATAAAAGTACAGTACGTACTTTGATGCGAAGATTAGGTTCGGGATTCTTAG-AAAAATTCTTTAT-----------------------

Apostasia_nipponica_PK12274 ACAAGAATTATTTTTCTTCTAATTTTGCT------------TTTCAAATGGCATCAGAAGGTTTTGGAGTCATTCTGGAAATTCCATTCTCGTCGCGATTAGTTTCT---------TCCCCTGAAG---AAATAAAAATACCAAAATCTCTGAATTTACGATCTATTCATTCAATATTTCCTTTTTTAGAGGACAAATTCTTACATTTAAATTATGTATTAG-GTA-TACT-AATACCCCACCCCATCCATCTGGGAATCTTGGTTCAAATCCTTCAATGCTGGATCAAAGATGCTCCTTCTTTGCATTTATTGTTAAAAATTTTTCACGAATATTATAATTCGAATAGTCTTTTTACTTC----------------------------------------AAAAAAAAGAAGA---TTTTTGGCTTCTTATATAATTCTTATATATGTGAATGCGAATTTCTTTTACTATTTTTTCGTAAACAGTCTTCCTACTTACGATCAACATCTTCTGGAGTTTTTCTTGAACGAATGCATTTCTATGGAAAAATAGAGTATCTTGTAGT------ATGTTT-AATTCTTTTCA---------AAGGATCCCATGCTTCTTCAA-AAATCTTTTCATGCATTATGTTCGATATCAAGGAAAAGGAATTCTGGCTTCAAAGGGTACTCTTATTCTGATGAAAAAAT-GGAAATATCATCTTGTAAATTTTTGGCAATCTTATTTTCGCTTTTGGTCTCAACCATATAGGATACATATAAAACAATTATTCAACTATTCCTTTTCGTTTATGGGG-TATTTTTCAAGTGTACTAAGATATTCTTTGGTAGTAA-GAAATCAAATGCTAGAGAATTCATTT-CTCATGGATATTCT-GATTAAGAAATTAGATACCATAGTTCCAGTTGTTTCTCTTATTGGATCAATGTCGAAAGCCCAATTTTGTACTGTATTGGGTCATCCTATTAGTAAACCGATTTGGACTGATTTTTCAGATTCTGATATTCTTGATCGATTTTGTAGAATATGTAAAAATCTTTGTCGTTTTTACAGTGGATCCTCAAAAAAACATGTTTTGTATCATATAAAATATATACTTCGACTTTCGTGTGCTAGAACTTTAGCTCGTAAACATAAAAGTACAGTACGTACTTTGATGCGAAGATTAGGTTCGGGATTCTTAG-AAAAATTCTTTAT-----------------------

Appendicula_cornuta_AY121739 ACAAGAATTCTTTTTCT------------------------TCTCAAATGGTATCAGAAGGTTTTGGAGTCATTCTGGAAATTCCATTCTCGTCGCGATTAGTATCT---------TTCCTTGAAG---AAAAAAGAATACCAAAATATCAGAATTTACGATCTATTCATTCAATATTTCCCTTTTTAGAGGATAAATTATTACATTTAAATTATGTGTCAG-ATC-TACT-AATACCCCATCCCATCCATCTGGAAATATTGGTTCAAATTCTTCAATGCTGGATCAAAGATGTTCCTTCTTTGCATTTATTACGATTGTTTTTTCACGAATATCATAATTTGAATAGTCTCATTATTTC----------AAAGAAATCCATTTACGTCTTTTCAAAAAGAAA---GAAAAGA-TTATTTTTG-TTCCTACATAATTCCTATGTATATGAATGCGAATATCTATTCCTGTTTCTTCGTAAACAGTCTTCTTATTTACGATCAATATCTTCTGGAGTCTTTCTTGAGCGAACACATTTCTATGTAAAAATAGAATATCTTATAGC---CGTATATTGTAATTCTTTTCA---------TAGGATCCTATGGTTCCTCAA-AGATACTTTCATACATTATGTTCGATATCAAGGAAAAGCGATTCTGGCTTCAAAAGGAACTCTTATTCTGATGAATAAAT-GGAAATTTCATCTTGTGAATCTTTGGCAATCTTATTTTCACTTTTGGTTTCAACCTGATAGGATCCATATAAAGCAATTACCCAACTATTCTTTCTCTTTTCTGGGG-TATTTTTCAAGTGTACTAAAAAATCCTTTGGTAGTAA-GAAATCAAATGTTAGATAATTCATTT-CTAATAAATACTCT-ATCTAAGAAATTAGATACCATAGTCCCAGTTATTTCTCTTATAGGATCATTGTCGAAAGCTCAATTTTGTACTGTATTGGGTCATCCTATTAGTAAACCGATCTGGACCGATTTATCGGATTCTGATATTCTTGATCGATTTTGTCGAATATGTAGAGATCTTTGTCGTTATCACAGCGGATCCTCAAAGAAACAGGTTTTGTATCGTATAAAGTATATACTTCGACTTTCGTGTGCTAGAACTTTGGCTCGTAAACATAAAAGTACAGTACGCACTTTTATGCGAAGATTAGGTTCGGGATTCTTAG-AAGAATTCTTTTTGGAAGAAGAACAATCTCTTTCTT

Appendicula_cornuta_AY368393 ACAAGAATTCTTTTTCT------------------------TCTCAAATGGTATCAGAAGGTTTTGGAGTCATTCTGGAAATTCCATTCTCGTCGCGATTAGTATCT---------TTCCTTGAAG---AAAAAAGAATACCAAAATATCAGAATTTACGATCTATTCATTCAATATTTCCCTTTTTAGAGGATAAATTATTACATTTAAATTATGTGTCAG-ATC-TACT-AATACCCCATCCCATCCATCTGGAAATATTGGTTCAAATTCTTCAATGCTGGATCAAAGATGTTCCTTCTTTGCATTTATTACGATTGTTTTTTCACGAATATCATAATTTGAATAGTCTCATTATTTC----------AAAGAAATCCATTTACGTCTTTTCAAAAAGAAA---GAAAAGA-TTATTTTTG-TTCCTACATAATTCCTATGTATATGAATGCGAATATCTATTCCTGTTTCTTCGTAAACAGTCTTCTTATTTACGATCAATATCTTCTGGAGTCTTTCTTGAGCGAACACATTTCTATGTAAAAATAGAATATCTTATAGC---CGTATATTGTAATTCTTTTCA---------TAGGATCCTATGGTTCCTCAA-AGATACTTTCATACATTATGTTCGATATCAAGGAAAAGCGATTCTGGCTTCAAAAGGAACTCTTATTCTGATGAATAAAT-GGAAATTTCATCTTGTGAATCTTTGGCAATCTTATTTTCACTTTTGGTTTCAACCTGATAGGATCCATATAAAGCAATTACCCAACTATTCTTTCTCTTTTCTGGGG-TATTTTTCAAGTGTACTAAAAAATCCTTTGGTAGTAA-GAAATCAAATGTTAGATAATTCATTT-CTAATAAATACTCT-ATCTAAGAAATTAGATACCATAGTCCCAGTTATTTCTCTTATAGGATCATTGTCGAAAGCTCAATTTTGTACTGTATTGGGTCATCCTATTAGTAAACCGATCTGGACCGATTTATCGGATTCTGATATTCTTGATCGATTTTGTCGAATATGTAGAGATCTTTGTCGTTATCACAGCGGATCCTCAAAGAAACAGGTTTTGTATCGTATAAAGTATATACTTCGACTTTCGTGTGCTAGAACTTTGGCTCGTAAACATAAAAGTACAGTACGCACTTTTATGCGAAGATTAGGTTCGGGATTCTTAG-AAGAATTCTTTTTGGAAGAAGAACAATCTCTTTCTT

Appendicula_cornuta_KF361651 -----------------------------------------------------------------------------------------------------------------------------------------------------------------------------------------------------------------AATTATGTGTCAG-ATC-TACT-AATACCCCATCCCATCCATCTGGAAATATTGGTTCAAATTCTTCAATGCTGGATCAAAGATGTTCCTTCTTTGCATTTATTGCGATTGTTTTTTCACGAATATCATAATTTGAATAGTCTCATTATTTC----------AAAGAAATCCATTTACGTCTTTTCAAAAAGAAA---GAAAAGA-TTATTTTTG-TTCCTACATAATTCCTATGTATATGAATGCGAATATCTATTCCTGTTTCTTCGTAAACAGTCTTCTTATTTACGATCAATATCTTCTGGAGTCTTTCTTGAGCGAACACATTTCTATGTAAAAATAGAATATCTTATAGC---CGTATATTGTAATTCTTTTCA---------TAGGATCCTATGGTTCCTCAA-AGATACTTTCATAAATTATGTTCGATATCAAGGAAAAGCGATTCTGGCTTCAAAAGGAACTCTTATTCTGATGAATAAAT-GGAAATTTCATCTTGTGAATCTTTGGCAATCTTATTTTCACTTTTGGTTTCAACCTTATAGGATCCATATAAAGCAATTACCCAACTATTCTTTCTCTTTTCTGGGG-TATTTTTCAAGTGTACTAAAAAATCCTTTGGTAGTAA-GAAATCAAATGTTAGATAATTCATTT-CTAATAAATACTCT-ATCTAAGAAATTAGATACCATAGTCCCAGTTATTTCTCTTATAGGATCATTGTCGAAAGCTCAATTTTGTACTGTATTGGGTCATCCTATTAGTAAACCGATCTGGACCGATTTATCGGATTCTGATATTCTTGATCGATTTTGTCGAATATGTAGAGATCTTTGTCGTTATCACAGCGGATCCTCAAAGAAACAGGTTTTGTAT--------------------------------------------------------------------------------------------------------------------------------------------

Appendicula_cornuta_KY239521 ACAAGAATTCTTTTTCT------------------------TCTCAAATGGTATCAGAAGGTTTTGGAGTCATTCTGGAAATTCCATTCTCGTCGCGATTAGTATCT---------TTCCTTGAAG---AAAAAAGAATACCAAAATATCAGAATTTACGATCTATTCATTCAATATTTCCCTTTTTAGAGGATAAATTATTACATTTAAATTATGTGTCAG-ATC-TACT-AATACCCCATCCCATCCATCTGGAAATATTGGTTCAAATTCTTCAATGCTGGATCAAAGATGTTCCTTCTTTGCATTTATTACGATTGTTTTTTCACGAATATCATAATTTGAATAGTCTCATTATTTC----------AAAGAAATCCATTTACGTCTTTTCAAAAAGAAA---GAAAAGA-TTATTTTTG-TTCCTACATAATTCCTATGTATATGAATGCGAATATCTATTCCTGTTTCTTCGTAAACAGTCTTCTTATTTACGATCAATATCTTCTGGAGTCTTTCTTGAGCGAACACATTTCTATGTAAAAATAGAATATCTTATAGC---CGTATATTGTAATTCTTTTCA---------TAGGATCCTATGGTTCCTCAA-AGATACTTTCATACATTATGTTCGATATCAAGGAAAAGCGATTCTGGCTTCAAAAGGAACTCTTATTCTGATGAATAAAT-GGAAATTTCATCTTGTGAATCTTTGGCAATCTTATTTTCACTTTTGGTTTCAACCTGATAGGATCCATATAAAGCAATTACCCAACTATTCTTTCTCTTTTCTGGGG-TATTTTTCAAGTGTACTAAAAAATCCTTTGGTAGTAA-GAAATCAAATGTTAGATAATTCATTT-CTAATAAATACTCT-ATCTAAGAAATTAGATACCATAGTCCCAGTTATTTCTCTTATAGGATCATTGTCGAAAGCTCAATTTTGTACTGTATTGGGTCATCCTATTAGTAAACCGATCTGGACCGATTTATCGGATTCTGATATTCTTGATCGATTTTGTCGAATATGTAGAGATCTTTGTCGTTATCACAGCGGATCCTCAAAGAAACAGGTTTTGTATCGTATAAAGTATATACTTCGACTTTCGTGTGCTAGAACTTTGGCTCGTAAACATAAAAGTACAGTACGCACTTTTATGCGAAGATTAGGTTCGGGATTCTTAG-AAGAATTCTTTTTGGAAGAAGAACAATCTCTTTCTT

Appendicula_cornuta_KY966709 ACAAGAATTCTTTTTCT------------------------TCTCAAATGGTATCAGAAAGTTTTGGAGTCATTCTGGAAATTCCATTCTCGTCGCGATTAGTATCT---------TTCCTTGAAG---AAAGAAGAATACCAAAATCTCAGAATTTACGATCTATTCATTCAATATTTCCCTTTTTAGAGGATAAATTATTACATTTAAATTATGTGTCAG-ATC-TACT-AATACCCCATCCCATCCATCTGGAAATATTGGTTCAAATTCTTCAATGCTGGATCAAAGATGTTCCTTCTTTGCATTTCTTGCGATTGTTTTTTCACGAATATCATAATTTGAATAGTCTCATTATTTC----------AAAGAAATCCATTTACGTCTTTTCAAAAAGAAA---GAAAAGA-TTATTTTTG-TTCCTACATAATTCTTATGTATATGAATGCGAATATCTATTCCTGTTTCTTCGTAAACACTCTTCTTATTTACGATCAATATCTTCTGGAATCTTTCTTGAGCGAACACATTTCTATGTAAAAATAGAATATCTTATAGC---CGTGTATTGTAATTTTTTTCA---------GAGGATCCTATGGTTCCTCAA-AGATACTTTCATACATTATGTTCGATATCAAGGAAAAGCGATCCTGGCTTCAAAAGGAACTCTTATTCTGATGAAGAAAT-GGCAATTTCATCTTGTGAATTTTTGGCAATCTTATTTTCACTTTTGGTTTCAACCTTATAGGATCCATATAAAGCAATTACCCAACTATTCCTTCTCTTTTCTGGGG-TATTTTTCAAGTGTACTAAAAAATAATTTGGTAGTAA-GAAATCAAATGTTAGAGAATTCATTT-CTAATAAATACTCT-ATCTAATAAATTAGATACCATAGTACCAGTTATTTCTCTTATAGGATCATTGTCGAAAGCTCAATTTTGTACTGTATTGGGCCATCCTATTAGTAAACCGATCTGGACCGATTTATCGGATTCTGATATTCTTGATCGATTTTGTCGTATATGTAGAGATCTTTGTCGTTATCACAGGGGATCCTCAAAGAAACAGGTTTTGTATCGTATAAAGTATATACTTCGAATTTCGTGTGCTAGAACTTTGGCTCGTAAACATAAAAGTACAGTACGCACTTTTATGCGAAGATTAGGTTCGGGATTCTTAG-AAGAATTCTT--------------------------

Appendicula_cornuta_KY966710 ACAAGAATTCTTTTTCT------------------------TCTCAAATGGTATCAGAAGGTTTTGGAGTCATTCTGGAAATTCCATTCTCGTCGCGATTAGTATCT---------TTCCTTGAAG---AAAAAAGAATACCAAAATATCAGAATTTACGATCTATTCATTCAATATTTCCCTTTTTAGAGGATAAATTATTACATTTAAATTATGTGTCAG-ATC-TACT-AATACCCCATCCCATCCATCTGGAAATATTGGTTCAAATTCTTCAATGCTGGATCAAAGATGTTCCTTCTTTGCATTTATTGCGATTGTTTTTTCACGAATATCATAATTTGAATAGTCTCATTATTTC----------AAAGAAATCCATTTACGTCTTTTCAAAAAGAAA---GAAAAGA-TTATTTTTG-TTCCTACATAATTCCTATGTATATGAATGCGAATATCTATTCCTGTTTCTTCGTAAACAGTCTTCTTATTTACGATCAATATCTTCTGGAGTCTTTCTTGAGCGAACACATTTCTATGTAAAAATAGAATATCTTATAGC---CGTATATTGTAATTCTTTTCA---------TAGGATCCTATGGTTCCTCAA-AGATACTTTCATAAATTATGTTCGATATCAAGGAAAAGCGATTCTGGCTTCAAAAGGAACTCTTATTCTGATGAATAAAT-GGAAATTTCATCTTGTGAATCTTTGGCAATCTTATTTTCACTTTTGGTTTCAACCTTATAGGATCCATATAAAGCAATTACCCAACTATTCTTTCTCTTTTCTGGGG-TATTTTTCAAGTGTACTAAAAAATCCTTTGGTAGTAA-GAAATCAAATGTTAGATAATTCATTT-CTAATAAATACTCT-ATCTAAGAAATTAGATACCATAGTCCCAGTTATTTCTCTTATAGGATCATTGTCGAAAGCTCAATTTTGTACTGTATTGGGTCATCCTATTAGTAAACCGATCTGGACCGATTTATCGGATTCTGATATTCTTGATCGATTTTGTCGAATATGTAGAGATCTTTGTCGTTATCACAGCGGATCCTCAAAGAAACAGGTTTTGTATCGTATAAAGTATATACTTCGACTTTCGTGTGCTAGAACTTTGGCTCGTAAACATAAAAGTACAGTACGCACTTTTATGCGAAGATTAGGTTCGGGATTCTTAG-AAGAATTCTT--------------------------

Appendicula_cornuta_PK12065 ACAAGAATTCTTTTTCT------------------------TCTCAAATGGTATCAGAAGGTTTTGGAGTCATTCTGGAAATTCCATTCTCGTCGCGATTAGTATCT---------TTCCTTGAAG---AAAAAAGAATACCAAAATATCAGAATTTACGATCTATTCATTCAATATTTCCCTTTTTAGAGGATAAATTATTACATTTAAATTATGTGTCAG-ATC-TACT-AATACCCCATCCCATCCATCTGGAAATATTGGTTCAAATTCTTCAATGCTGGATCAAAGATGTTCCTTCTTTGCATTTATTGCGATTGTTTTTTCACGAATATCATAATTTGAATAGTCTCATTATTTC----------AAAGAAATCCATTTACGTCTTTTCAAAAAGAAA---GAAAAGA-TTATTTTTG-TTCCTACATAATTCCTATGTATATGAATGCGAATATCTATTCCTGTTTCTTCGTAAACAGTCTTCTTATTTACGATCAATATCTTCTGGAGTCTTTCTTGAGCGAACACATTTCTATGTAAAAATAGAATATCTTATAGC---CGTATATTGTAATTCTTTTCA---------TAGGATCCTATGGTTCCTCAA-AGATACTTTCATAAATTATGTTCGATATCAAGGAAAAGCGATTCTGGCTTCAAAAGGAACTCTTATTCTGATGAATAAAT-GGAAATTTCATCTTGTGAATCTTTGGCAATCTTATTTTCACTTTTGGTTTCAACCTTATAGGATCCATATAAAGCAATTACCCAACTATTCTTTCTCTTTTCTGGGG-TATTTTTCAAGTGTACTAAAAAATCCTTTGGTAGTAA-GAAATCAAATGTTAGATAATTCATTT-CTAATAAATACTCT-ATCTAAGAAATTAGATACCATAGTCCCAGTTATTTCTCTTATAGGATCATTGTCGAAAGCTCAATTTTGTACTGTATTGGGTCATCCTATTAGTAAACCGATCTGGACCGATTTATCGGATTCTGATATTCTTGATCGATTTTGTCGAATATGTAGAGATCTTTGTCGTTATCACAGCGGATCCTCAAAGAAACAGGTTTTGTATCGTATAAAGTATATACTTCGACTTTCGTGTGCTAGAACTTTGGCTCGTAAACATAAAAGTACAGTACGCACTTTTATGCGAAGATTAGGTTCGGGATTCTTAG-AAGAATTCTTTTTGGAAGAAGAACAATCTCTTTCTT

Arundina_graminifolia_AF263626 ACAAGAATTCTTTTTCTTCTCATTTTTCT------------TCTCAAATGGTATCAGAAGGTTTTGGAGTCATTCTGGAAATTCCATTCTCGTCGCAATTAGTATCT---------TCCCTTGAAG---ATAACAGAATACCAAAATTTCAGAATTTACTATCTATTCATTCAATATTTCCCTTTTTAGAGGATAAATTATCACATTTCAATTATGTGTCAG-ATC-TACT-AATACCCCATCCCATCCATCTGGAAATCTTGGTTCAAATCCTTCAATGCTGGATTAAAGATGTTTCTTCTTTGCATTTCTTGCGATTGTTTTTCCACGAATATCATAATTTGAATAGTCTCATTACTTC----------AAATAAATCCATTTACGTCTTTTCAAAAAGAAC---CAAAAGA-TTCTTTTGG-TTCCTACATAACTCTTATGTATATGAATGCGAATATATATTCCTGTTTCTTCGTAAACAGTCTTCTTATTTACGATCAATATCTTCTGGAGTCTTTCTTGAGCGAACACATTTCTATGGAAAAATAGAATATCTTATAGT---CGTGTGTTGTAATTCTTTTCA---------GAGGATCCTATGGTTCCTCAA-AGATACTTTCATACATTATGTTCGATATCAAGGAAAAACAATTCTGGTTTCAAAAGGAACTCTTATTCTGATTAAGAAAT-GGAAATTTCATCGTGTGTAGTTTTGGCAATCTAATTTTCACTTTTGGTTTCAACCTTATAGGATTCATATAAAGCAATTACCCAACTATTCCTTCTCTTTTCTGGGG-TATTTTTCAAGTGTACTAAAAAATAATTTGGTAATAA-GAAATCAAATGCTAGAGAATTCATTT-CTAATAAATACTCT-GACTAAGAAATTAGATACCATAGCCCCAGTTACTTCTATTATTGGATCATTGTCGAAAGCTCAATTTTGTACTGTATTGGGTCATCCTATTAGTAAACCGATCTGGACCGATTTATCGGATTCTGATATTCTTGATCGATTTTGTCGGATATGTAGAAATCTTTGTCGTTATCACAGCGGATCTTCAAAGAAACAGGTTTTGTATCGTATAAAGTATATACTTCGACTTTCGTGTGCTAGAACTTTGGCTCGTAAACATAAAAGTACAGTACGCACTTTTATGCGAAGATTAGGTTCGGGATTCTTAG-AAGAATTTTTTTT-GAAGAAGAACAATCTCTTTCTT

Arundina_graminifolia_AF302692 ACAAGAATTCTTTTTCTTCTCATTTTTCT------------TCTCAAATGGTATCAGAAGGTTTTGGAGTCATTCTGGAAATTCCATTCTCGTCGCAATTAGTATCT---------TCCCTTGAAG---ATAACAGAATACCAAAATTTCAGAATTTACTATCTATTCATTCAATATTTCCCTTTTTAGAGGATAAATTATCACATTTCAATTATGTGTCAG-ATC-TACT-AATACCCCATCCCATCCATCTGGAAATCTTGGTTCAAATCCTTCAATGCTGGATTAAAGATGTTTCTTCTTTGCATTTCTTGCGATTGTTTTTCCACGAATATCATAATTTGAATAGTCTCATTACTTC----------AAATAAATCCATTTACGTCTTTTCAAAAAGAAC---CAAAAGA-TTCTTTTGG-TTCCTACATAACTCTTATGTATATGAATGCGAATATATATTCCTGTTTCTTCGTAAACAGTCTTCTTATTTACGATCAATATCTTCTGGAGTCTTTCTTGAGCGAACACATTTCTATGGAAAAATAGAATATCTTATAGT---CGTGTGTTGTAATTCTTTTCA---------GAGGATCCTATGGTTCCTCAA-AGATACTTTCATACATTATGTTCGATATCAAGGAAAAACAATTCTGGTTTCAAAAGGAACTCTTATTCTGATTAAGAAAT-GGAAATTTCATCGTGTGTAGTTTTGGCAATCTAATTTTCACTTTTGGTTTCAACCTTATAGGATTCATATAAAGCAATTACCCAACTATTCCTTCTCTTTTCTGGGG-TATTTTTCAAGTGTACTAAAAAATAATTTGGTAATAA-GAAATCAAATGCTAGAGAATTCATTT-CTAATAAATACTCT-GACTAAGAAATTAGATACCATAGCCCCAGTTACTTCTATTATTGGATCATTGTCGAAAGCTCAATTTTGTACTGTATTGGGTCATCCTATTAGTAAACCGATCTGGACCGATTTATCGGATTCTGATATTCTTGATCGATTTTGTCGGATATGTAGAAATCTTTGTCGTTATCACAGCGGATCTTCAAAGAAACAGGTTTTGTATCGTATAAAGTATATACTTCGACTTTCGTGTGCTAGAACTTTGGCTCGTAAACATAAAAGTACAGTACGCACTTTTATGCGAAGATTAGGTTCGGGATTCTTAG-AAGAATTTTTTTT-GAAGAAGAACAATCTCTTTCTT

Arundina_graminifolia_EF079333 ACAAGAATTCTTTTTCTTCTCATTTTTCT------------TCTCAAATGGTATCAGAAGGTTTTGGAGTCATTCTGGAAATTCCATTCTCGTCGCAATTAGTATCT---------TCCCTTGAAG---ATAACAGAATACCAAAATTTCAGAATTTACTATCTATTCATTCAATATTTCCCTTTTTAGAGGATAAATTATCACATTTCAATTATGTGTCAG-ATC-TACT-AATACCCCATCCCATCCATCTGGAAATCTTGGTTCAAATCCTTCAATGCTGGATTAAAGATGTTTCTTCTTTGCATTTCTTGCGATTGTTTTTCCACGAATATCATAATTTGAATAGTCTCATTACTTC----------AAATAAATCCATTTACGTCTTTTCAAAAAGAAC---CAAAAGA-TTCTTTTGG-TTCCTACATAATTCTTATGTATATGAATGCGAATATATATTCCTGTTTCTTCGTAAACAGTCTTCTTATTTACGATCAATATCTTCTGGAGTCTTTCTTGAGCGAACACATTTCTATGGAAAAATAGAATATCTTATAGT---CGTGTGTTGTAATTCTTTTCA---------GAGGATCCTATGGTTCCTCAA-AGATACTTTCATACATTATGTTCGATATCAAGGAAAAACAATTCTGGTTTCAAAAGGAACTCTTATTCTGATTAAGAAAT-GGAAATTTCATCTTGTGAATTTTTGGCAATCTTATTTTCACTTTTGGTTTCAACCTTATAGGATTCATATAAAGCAATTACCCAACTATTCCTTCTCTTTTCTGGGG-TATTTTTCAAGTGTACTAAAAAATAATTTGGTAATAA-GAAATCAAATGCTAGAGAATTCATTT-CTAATAAATACTCT-GACTAAGAAATTAGATACCATAGCCCCAGTTACTTCTATTATTGGATCATTGTCGAAAGCTCAATTTTGTACTGTATTGGGTCATCCTATTAGTAAACCGATCTGGACCGATTTATCGGATTCTGATATTTTTGATCGATTTTGTCGGATATGTAGAAATCTTTGTCGTTATCACAGCGGATCTTCAAAGAAACAGGTTTTGTATCGTATAAAGTATATACTTCGACTTTCGTGTGCTAGAACTTTGGCTCGTAAACATAAAAGTACAGTACGCACTTTTATGCGAAGATTAGGTTCGGGATTCTTAG-AAGAATTTTTTTTTGAAGAAGAACAATCTCTTTCTT

Arundina_graminifolia_JN004354 ------------------------------------------------------------------------------------------------------------------------------------------------------------------------------------------------------------------------------------------------------------------------------------------------------------------ATTTCTTGCGATTGTTTTTCCACGAATATCATAATTTGAATAGTCTCATTACTTC----------AAATAAATCCATTTACGTCTTTTCAAAAAGAAC---CAAAAGA-TTCTTTTGG-TTCCTACATAATTCTTATGTATATGAATGCGAATATATATTCCTGTTTCTTCGTAAACAGTCTTCTTATTTACGATCAATATCTTCTGGAGTCTTTCTTGAGCGAACACATTTCTATGGAAAAATAGAATATCTTATAGT---CGTGTGTTGTAATTCTTTTCA---------GAGGATCCTATGGTTCCTCAA-AGATACTTTCATACATTATGTTCGATATCAAGGAAAAACAATTCTGGTTTCAAAAGGAACTCTTATTCTGATTAAGAAAT-GGAAATTTCATCTTGTGAATTTTTGGCAATCTTATTTTCACTTTTGGTTTCAACCTTATAGGATTCATATAAAGCAATTACCCAACTATTCCTTCTCTTTTCTGGGG-TATTTTTCAAGTGTACTAAAAAATAATTTGGTAATAA-GAAATCAAATGCTAGAGAATTCATTT-CTAATAAATACTCT-GACTAAGAAATTAGATACCATAGCCCCAGTTACTTCTATTATTGGATCATTGTCGAAAGCTCAATTTTGTACTGTATTGGGTCATCCTATTAGTAAACCGATCTGGACCGATTTATCGGATTCTGATATTTTTGATCGATTTTGTCGGATATGTAGAAATCTTTGTCGTTATCACAGCGGATCTTCAAAGAAACAGGTTT-------------------------------------------------------------------------------------------------------------------------------------------------

Arundina_graminifolia_JN004355 ------------------------------------------------------------------------------------------------------------------------------------------------------------------------------------------------------------------------------------------------------------------------------------------------------------------ATTTCTTGCGATTGTTTTTCCACGAATATCATAATTTGAATAGTCTCATTACTTC----------AAATAAATCCATTTACGTCTTTTCAAAAAGAAC---CAAAAGA-TTCTTTTGG-TTCCTACATAATTCTTATGTATATGAATGCGAATATATATTCCTGTTTCTTCGTAAACAGTCTTCTTATTTACGATCAATATCTTCTGGAGTCTTTCTTGAGCGAACACATTTCTATGGAAAAATAGAATATCTTATAGT---CGTGTGTTGTAATTCTTTTCA---------GAGGATCCTATGGTTCCTCAA-AGATACTTTCATACATTATGTTCGATATCAAGGAAAAACAATTCTGGTTTCAAAAGGAACTCTTATTCTGATTAAGAAAT-GGAAATTTCATCTTGTGAATTTTTGGCAATCTTATTTTCACTTTTGGTTTCAACCTTATAGGATTCATATAAAGCAATTACCCAACTATTCCTTCTCTTTTCTGGGG-TATTTTTCAAGTGTACTAAAAAATAATTTGGTAATAA-GAAATCAAATGCTAGAGAATTCATTT-CTAATAAATACTCT-GACTAAGAAATTAGATACCATAGCCCCAGTTACTTCTATTATTGGATCATTGTCGAAAGCTCAATTTTGTACTGTATTGGGTCATCCTATTAGTAAACCGATCTGGACCGATTTATCGGATTCTGATATTTTTGATCGATTTTGTCGGATATGTAGAAATCTTTGTCGTTATCACAGCGGATCTT----------------------------------------------------------------------------------------------------------------------------------------------------------------

Arundina_graminifolia_JN004356 -----------------------------------------------------------------------------------------------------------------------------------------------------------------------------------------------------------------------------------------------------------------------------------------------------------TCTTTGCATTTCTTGCGATTGTTTTTCCACGAATATCATAATTTGAATAGTCTCATTACTTC----------AAATAAATCCATTTACGTCTTTTCAAAAAGAAC---CAAAAGA-TTCTTTTGG-TTCCTACATAATTCTTATGTATATGAATGCGAATATATATTCCTGTTTCTTCGTAAACAGTCTTCTTATTTACGATCAATATCTTCTGGAGTCTTTCTTGAGCGAACACATTTCTATGGAAAAATAGAATATCTTATAGT---CGTGTGTTGTAATTCTTTTCA---------GAGGATCCTATGGTTCCTCAA-AGATACTTTCATACATTATGTTCGATATCAAGGAAAAACAATTCTGGTTTCAAAAGGAACTCTTATTCTGATTAAGAAAT-GGAAATTTCATCTTGTGAATTTTTGGCAATCTTATTTTCACTTTTGGTTTCAACCTTATAGGATTCATATAAAGCAATTACCCAACTATTCCTTCTCTTTTCTGGGG-TATTTTTCAAGTGTACTAAAAAATAATTTGGTAATAA-GAAATCAAATGCTAGAGAATTCATTT-CTAATAAATACTCT-GACTAAGAAATTAGATACCATAGCCCCAGTTACTTCTATTATTGGATCATTGTCGAAAGCTCAATTTTGTACTGTATTGGGTCATCCTATTAGTAAACCGATCTGGACCGATTTATCGGATTCTGATATTTTTGATCGATTTTGTCGGATATGTAGAAATCTTTGTCGTTA------------------------------------------------------------------------------------------------------------------------------------------------------------------------------

Arundina_graminifolia_KF421845 -----------------------------------------------------------------------------------------------------------------------------------------------------------------------------------------------------------------------------------------------------------------------------------------------------------------------------------TTCCACGAATATCATAATTTGAATAGTCTCATTACTTC----------AAATAAATCCATTTACGTCTTTTCAAAAAGAAC---CAAAAGA-TTCTTTTGG-TTCCTACATAATTCTTATGTATATGAATGCGAATATCTATTCCTGTTTCTTCGTAAACAGTCTTCTTATTTACGATCAATATCTTCTGGAGTCTTTCTTGAGCGAACACATTTCTATGGAAAAATAGAATATCTTATAGT---CGTGTGTTGTAATTCTTTTCA---------GAGGATCCTATGGTTCCTCAA-AGATACTTTCATACATTATGTTCGATATCAAGGAAAAACAATTCTGGTTTCAAAAGGAACTCTTATTCTGATTAAGAAAT-GGAAATTTCATCTTGTGAATTTTTGGCAATCTTATTTTCACTTTTGGTTTCAACCTTATAGGATTCATATAAAGCAATTACCCAACTATTCCTTCTCTTTTCTGGGG-TATTTTTCAAGTGTACTAAAAAATAATTTGGTAATAA-GAAATCAAATGCTAGAGAATTCATTT-CTAATAAATACTCT-GACTAAGAAATTAGATACCATAGCCCCAGTTACTTCTATTATTGGATCATTGTCGAAAGCTCAATTTTGTACTGTATTGGGTCATCCTATTAGTAAACCGATCTGGACCGATTTATCGGATTCTGATATTTTTGATCGATTTTGTCGGATATGTAGAAATCTTTGTCGTTATCACAGCGGATCTTCAAAGAA---------------------------------------------------------------------------------------------------------------------------------------------------------

Arundina_graminifolia_KX298566 -----------------------------------------------------------------------------------------------------------------------------------------------------------------------------------------------------------------------------------------------------------------------------------------------------------------------------------------GAATATCATAATTTGAATAGTCTCATTACTTC----------AAATAAATCCATTTACGTCTTTTCAAAAAGAAC---CAAAAGA-TTCTTTTGG-TTCCTACATAATTCTTATGTATATGAATGCGAATATATATTCCTGTTTCTTCGTAAACAGTCTTCTTATTTACGATCAATATCTTCTGGAGTCTTTCTTGAGCGAACACATTTCTATGGAAAAATAGAATATCTTATAGT---CGTGTGTTGTAATTCTTTTCA---------GAGGATCCTATGGTTCCTCAA-AGATACTTTCATACATTATGTTCGATATCAAGGAAAAACAATTCTGGTTTCAAAAGGAACTCTTATTCTGATTAAGAAAT-GGAAATTTCATCTTGTGAATTTTTGGCAATCTTATTTTCACTTTTGGTTTCAACCTTATAGGATTCATATAAAGCAATTACCCAACTATTCCTTCTCTTTTCTGGGG-TATTTTTCAAGTGTACTAAAAAATAATTTGGTAATAA-GAAATCAAATGCTAGAGAATTCATTT-CTAATAAATACTCT-GACTAAGAAATTAGATACCATAGCCCCAGTTACTTCTATTATTGGATCATTGTCGAAAGCTCAATTTTGTACTGTATTGGGTCATCCTATTAGTAAACCGATCTGGACCGATTTATCGGATTCTGATATTTTTGATCGATTTTGTCGGATATGTAGAAATCTTTGTCGTTATCACAGCGGA--------------------------------------------------------------------------------------------------------------------------------------------------------------------

Arundina_graminifolia_KY966712 ACAAGAATTCTTTTTCTTCTCATTTTTCT------------TCTCAAATGGTATCAGAAGGTTTTGGAGTCATTCTGGAAATTCCATTCTCGTCGCAATTAGTATCT---------TCCCTTGAAG---ATAACAGAATACCAAAATTTCAGAATTTACTATCTATTCATTCAATATTTCCCTTTTTAGAGGATAAATTATCACATTTCAATTATGTGTCAG-ATC-TACT-AATACCCCATCCCATCCATCTGGAAATCTTGGTTCAAATCCTTCAATGCTGGATTAAAGATGTTTCTTCTTTGCATTTCTTGCGATTGTTTTTCCACGAATATCATAATTTGAATAGTCTCATTACTTC----------AAATAAATCCATTTACGTCTTTTCAAAAAGAAC---CAAAAGA-TTCTTTTGG-TTCCTACATAATTCTTATGTATATGAATGCGAATATATATTCCTGTTTCTTCGTAAACAGTCTTCTTATTTACGATCAATATCTTCTGGAGTCTTTCTTGAGCGAACACATTTCTATGGAAAAATAGAATATCTTATAGT---CGTGTGTTGTAATTCTTTTCA---------GAGGATCCTATGGTTCCTCAA-AGATACTTTCATACATTATGTTCGATATCAAGGAAAAACAATTCTGGTTTCAAAAGGAACTCTTATTCTGATTAAGAAAT-GGAAATTTCATCTTGTGAATTTTTGGCAATCTTATTTTCACTTTTGGTTTCAACCTTATAGGATTCATATAAAGCAATTACCCAACTATTCCTTCTCTTTTCTGGGG-TATTTTTCAAGTGTACTAAAAAATAATTTGGTAATAA-GAAATCAAATGCTAGAGAATTCATTT-CTAATAAATACTCT-GACTAAGAAATTAGATACCATAGCCCCAGTTACTTCTATTATTGGATCATTGTCGAAAGCTCAATTTTGTACTGTATTGGGTCATCCTATTAGTAAACCGATCTGGACCGATTTATCGGATTCTGATATTTTTGATCGATTTTGTCGGATATGTAGAAATCTTTGTCGTTATCACAGCGGATCTTCAAAGAAACAGGTTTTGTATCGTATAAAGTATATACTTCGACTTTCGTGTGCTAGAACTTTGGCTCGTAAACATAAAAGTACAGTACGCACTTTTATGCGAAGATTAGGTTCGGGATTCTTAG-AAGAATTTTT--------------------------

Arundina_graminifolia_SG1008 ACAAGAATTCTTTTTCTTCTCATTTTTCT------------TCTCAAATGGTATCAGAAGGTTTTGGAGTCATTCTGGAAATTCCATTCTCGTCGCAATTAGTATCT---------TCCCTTGAAG---ATAACAGAATACCAAAATTTCAGAATTTACTATCTATTCATTCAATATTTCCCTTTTTAGAGGATAAATTATCACATTTCAATTATGTGTCAG-ATC-TACT-AATACCCCATCCCATCCATCTGGAAATCTTGGTTCAAATCCTTCAATGCTGGATTAAAGATGTTTCTTCTTTGCATTTCTTGCGATTGTTTTTCCACGAATATCATAATTTGAATAGTCTCATTACTTC----------AAATAAATCCATTTACGTCTTTTCAAAAAGAAC---CAAAAGA-TTCTTTTGG-TTCCTACATAATTCTTATGTATATGAATGCGAATATATATTCCTGTTTCTTCGTAAACAGTCTTCTTATTTACGATCAATATCTTCTGGAGTCTTTCTTGAGCGAACACATTTCTATGGAAAAATAGAATATCTTATAGT---CGTGTGTTGTAATTCTTTTCA---------GAGGATCCTATGGTTCCTCAA-AGATACTTTCATACATTATGTTCGATATCAAGGAAAAACAATTCTGGTTTCAAAAGGAACTCTTATTCTGATTAAGAAAT-GGAAATTTCATCTTGTGAATTTTTGGCAATCTTATTTTCACTTTTGGTTTCAACCTTATAGGATTCATATAAAGCAATTACCCAACTATTCCTTCTCTTTTCTGGGG-TATTTTTCAAGTGTACTAAAAAATAATTTGGTAATAA-GAAATCAAATGCTAGAGAATTCATTT-CTAATAAATACTCT-GACTAAGAAATTAGATACCATAGCCCCAGTTACTTCTATTATTGGATCATTGTCGAAAGCTCAATTTTGTACTGTATTGGGTCATCCTATTAGTAAACCGATCTGGACCGATTTATCGGATTCTGATATTTTTGATCGATTTTGTCGGATATGTAGAAATCTTTGTCGTTATCACAGCGGATCTTCAAAGAAACAGGTTTTGTATCGTATAAAGTATATACTTCGACTTTCGTGTGCTAGAACTTTGGCTCGTAAACATAAAAGTACAGTACGCACTTTTATGCGAAGATTAGGTTCGGGATTCTTAG-AAGAATTTTTTTTTGAAGAAGAACAATCTCTTTCTT

Arundina_graminifolia_SG1206 ACAAGAATTCTTTTTCTTCTCATTTTTCT------------TCTCAAATGGTATCAGAAGGTTTTGGAGTCATTCTGGAAATTCCATTCTCGTCGCAATTAGTATCT---------TCCCTTGAAG---ATAACAGAATACCAAAATTTCAGAATTTACTATCTATTCATTCAATATTTCCCTTTTTAGAGGATAAATTATCACATTTCAATTATGTGTCAG-ATC-TACT-AATACCCCATCCCATCCATCTGGAAATCTTGGTTCAAATCCTTCAATGCTGGATTAAAGATGTTTCTTCTTTGCATTTCTTGCGATTGTTTTTCCACGAATATCATAATTTGAATAGTCTCATTACTTC----------AAATAAATCCATTTACGTCTTTTCAAAAAGAAC---CAAAAGA-TTCTTTTGG-TTCCTACATAACTCTTATGTATATGAATGCGAATATATATTCCTGTTTCTTCGTAAACAGTCTTCTTATTTACGATCAATATCTTCTGGAGTCTTTCTTGAGCGAACACATTTCTATGGAAAAATAGAATATCTTATAGT---CGTGTGTTGTAATTCTTTTCA---------GAGGATCCTATGGTTCCTCAA-AGATACTTTCATACATTATGTTCGATATCAAGGAAAAACAATTCTGGTTTCAAAAGGAACTCTTATTCTGATTAAGAAAT-GGAAATTTCATCTTGTGAATTTTTGGCAATCTTATTTTCACTTTTGGTTTCAACCTTATAGGATTCATATAAAGCAATTACCCAACTATTCCTTCTCTTTTCTGGGG-TATTTTTCAAGTGTACTAAAAAATAATTTGGTAATAA-GAAATCAAATGCTAGAGAATTCATTT-CTAATAAATACTCT-GACTAAGAAATTAGATACCATAGCCCCAGTTACTTCTATTATTGGATCATTGTCGAAAGCTCAATTTTGTACTGTATTGGGTCATCCTATTAGTAAACCGATCTGGACCGATTTATCGGATTCTGATATTTTTGATCGATTTTGTCGGATATGTAGAAATCTTTGTCGTTATCACAGCGGATCTTCAAAGAAACAGGTTTTGTATCGTATAAAGTATATACTTCGACTTTCGTGTGCTAGAACTTTGGCTCGTAAACATAAAAGTACAGTACGCACTTTTATGCGAAGATTAGGTTCGGGATTCTTAG-AAGAATTTTTTTTTGAAGAAGAACAATCTCTTTCTT

Arundina_graminifolia_SG1295 ---AGAATTCTTTTTCTTCTCATTTTTCT------------TCTCAAATGGTATCAGAAGGTTTTGGAGTCATTCTGGAAATTCCATTCTCGTCGCAATTAGTATCT---------TCCCTTGAAG---ATAACAGAATACCAAAATTTCAGAATTTACTATCTATTCATTCAATATTTCCCTTTTTAGAGGATAAATTATCACATTTCAATTATGTGTCAG-ATC-TACT-AATACCCCATCCCATCCATCTGGAAATCTTGGTTCAAATCCTTCAATGCTGGATTAAAGATGTTTCTTCTTTGCATTTCTTGCGATTGTTTTTCCACGAATATCATAATTTGAATAGTCTCATTACTTC----------AAATAAATCCATTTACGTCTTTTCAAAAAGAAC---CAAAAGA-TTCTTTTGG-TTCCTACATAACTCTTATGTATATGAATGCGAATATATATTCCTGTTTCTTCGTAAACAGTCTTCTTATTTACGATCAATATCTTCTGGAGTCTTTCTTGAGCGAACACATTTCTATGGAAAAATAGAATATCTTATAGT---CGTGTGTTGTAATTCTTTTCA---------GAGGATCCTATGGTTCCTCAA-AGATACTTTCATACATTATGTTCGATATCAAGGAAAAACAATTCTGGTTTCAAAAGGAACTCTTATTCTGATTAAGAAAT-GGAAATTTCATCTTGTGAATTTTTGGCAATCTTATTTTCACTTTTGGTTTCAACCTTATAGGATTCATATAAAGCAATTACCCAACTATTCCTTCTCTTTTCTGGGG-TATTTTTCAAGTGTACTAAAAAATAATTTGGTAATAA-GAAATCAAATGCTAGAGAATTCATTT-CTAATAAATACTCT-GACTAAGAAATTAGATACCATAGCCCCAGTTACTTCTATTATTGGATCATTGTCGAAAGCTCAATTTTGTACTGTATTGGGTCATCCTATTAGTAAACCGATCTGGACCGATTTATCGGATTCTGATATTTTTGATCGATTTTGTCGGATATGTAGAAATCTTTGTCGTTATCACAGCGGATCTTCAAAGAAACAGGTTTTGTATCGTATAAAGTATATACTTCGACTTTCGTGTGCTAGAACTTTGGCTCGTAAACATAAAAGTACAGTACGCACTTTTATGCGAAGATTAGGTTCGGGATTCTTAG-AAGAATTTTTTTTTGAAGAAGAACAATCTCTTTCTT

Arundina_graminifolia_var_graminifolia_AB844195 ACAAGAATTCTTTTTCTTCTCATTTTTCT------------TCTCAAATGGTATCAGAAGGTTTTGGAGTCATTCTGGAAATTCCATTCTCGTCGCAATTAGTATCT---------TCCCTTGAAG---ATAACAGAATACCAAAATTTCAGAATTTACTATCTATTCATTCAATATTTCCCTTTTTAGAGGATAAATTATCACATTTCAATTATGTGTCAG-ATC-TACT-AATACCCCATCCCATCCATCTGGAAATCTTGGTTCAAATCCTTCAATGCTGGATTAAAGATGTTTCTTCTTTGCATTTCTTGCGATTGTTTTTCCACGAATATCATAATTTGAATAGTCTCATTACTTC----------AAATAAATCCATTTACGTCTTTTCAAAAAGAAC---CAAAAGA-ATCTTTTGG-TTCCTACATAATTCTTATGTATATGAATGCGAATATATATTCCTGTTTCTTCGTAAACAGTCTTCTTATTTACGATCAATATCTTCTGGAGTCTTTCTTGAGCGAACACATTTCTATGGAAAAATAGAATATCTTATAGT---CGTGTGTTGTAATTCTTTTCA---------GAGGATCCTATGGTTCCTCAA-AGATACTTTCATACATTATGTTCGATATCAAGGAAAAACAATTCTGGTTTCAAAAGGAACTCTTATTCTGATTAAGAAAT-GGAAATTTCATCTTGTGAATTTTTGGCAATCTTATTTTCACTTTTGGTTTCAACCTTATAGGATTCATATAAAGCAATTACCCAACTATTCCTTCTCTTTTCTGGGG-TATTTTTCAAGTGTACTAAAAAATAATTTGGTAATAA-GAAATCAAATGCTAGAGAATTCATTT-CTAATAAATACTCT-GACTAAGAAATTAGATACCATAGCCCCAGTTACTTCTATTATTGGATCATTGTCGAAAGCTCAATTTTGTACTGTATTGGGTCATCCTATTAGTAAACCGATCTGGACCGATTTATCGGATTCTGATATTTTTGATCGATTTTGTCGGATATGTAGAAATCTTTGTCGTTATCACAGCGGATCTTCAAAGAAACAGGTTTTGTATCGTATAAAGTATATACTTCGACTTTCGTGTGCTAGAACTTTGGCTCGTAAACATAAAAGTACAGTACGCACTTTTATGCGAAGATTAGGTTCGGGATTCTTAG-AAGAATTTTTTTTTGAAGAAGAACAATCTCTTTCTT

Arundina_graminifolia_var_graminifolia_AB844196 ACAAGAATTCTTTTTCTTCTCATTTTTCT------------TCTCAAATGGTATCAGAAGGTTTTGGAGTCATTCTGGAAATTCCATTCTCGTCGCAATTAGTATCT---------TCCCTTGAAG---ATAACAGAATACCAAAATTTCAGAATTTACTATCTATTCATTCAATATTTCCCTTTTTAGAGGATAAATTATCACATTTCAATTATGTGTCAG-ATC-TACT-AATACCCCATCCCATCCATCTGGAAATCTTGGTTCAAATCCTTCAATGCTGGATTAAAGATGTTTCTTCTTTGCATTTCTTGCGATTGTTTTTCCACGAATATCATAATTTGAATAGTCTCATTACTTC----------AAATAAATCCATTTACGTCTTTTCAAAAAGAAC---CAAAAGA-ATCTTTTGG-TTCCTACATAATTCTTATGTATATGAATGCGAATATATATTCCTGTTTCTTCGTAAACAGTCTTCTTATTTACGATCAATATCTTCTGGAGTCTTTCTTGAGCGAACACATTTCTATGGAAAAATAGAATATCTTATAGT---CGTGTGTTGTAATTCTTTTCA---------GAGGATCCTATGGTTCCTCAA-AGATACTTTCATACATTATGTTCGATATCAAGGAAAAACAATTCTGGTTTCAAAAGGAACTCTTATTCTGATTAAGAAAT-GGAAATTTCATCTTGTGAATTTTTGGCAATCTTATTTTCACTTTTGGTTTCAACCTTATAGGATTCATATAAAGCAATTACCCAACTATTCCTTCTCTTTTCTGGGG-TATTTTTCAAGTGTACTAAAAAATAATTTGGTAATAA-GAAATCAAATGCTAGAGAATTCATTT-CTAATAAATACTCT-GACTAAGAAATTAGATACCATAGCCCCAGTTACTTCTATTATTGGATCATTGTCGAAAGCTCAATTTTGTACTGTATTGGGTCATCCTATTAGTAAACCGATCTGGACCGATTTATCGGATTCTGATATTTTTGATCGATTTTGTCGGATATGTAGAAATCTTTGTCGTTATCACAGCGGATCTTCAAAGAAACAGGTTTTGTATCGTATAAAGTATATACTTCGACTTTCGTGTGCTAGAACTTTGGCTCGTAAACATAAAAGTACAGTACGCACTTTTATGCGAAGATTAGGTTCGGGATTCTTAG-AAGAATTTTTTTTTGAAGAAGAACAATCTCTTTCTT

Arundina_graminifolia_var_graminifolia_AB844197 ACAAGAATTCTTTTTCTTCTCATTTTTCT------------TCTCAAATGGTATCAGAAGGTTTTGGAGTCATTCTGGAAATTCCATTCTCGTCGCAATTAGTATCT---------TCCCTTGAAG---ATAACAGAATACCAAAATTTCAGAATTTACTATCTATTCATTCAATATTTCCCTTTTTAGAGGATAAATTATCACATTTCAATTATGTGTCAG-ATC-TACT-AATACCCCATCCCATCCATCTGGAAATCTTGGTTCAAATCCTTCAATGCTGGATTAAAGATGTTTCTTCTTTGCATTTCTTGCGATTGTTTTTCCACGAATATCATAATTTGAATAGTCTCATTACTTC----------AAATAAATCCATTTACGTCTTTTCAAAAAGAAC---CAAAAGA-ATCTTTTGG-TTCCTACATAATTCTTATGTATATGAATGCGAATATATATTCCTGTTTCTTCGTAAACAGTCTTCTTATTTACGATCAATATCTTCTGGAGTCTTTCTTGAGCGAACACATTTCTATGGAAAAATAGAATATCTTATAGT---CGTGTGTTGTAATTCTTTTCA---------GAGGATCCTATGGTTCCTCAA-AGATACTTTCATACATTATGTTCGATATCAAGGAAAAACAATTCTGGTTTCAAAAGGAACTCTTATTCTGATTAAGAAAT-GGAAATTTCATCTTGTGAATTTTTGGCAATCTTATTTTCACTTTTGGTTTCAACCTTATAGGATTCATATAAAGCAATTACCCAACTATTCCTTCTCTTTTCTGGGG-TATTTTTCAAGTGTACTAAAAAATAATTTGGTAATAA-GAAATCAAATGCTAGAGAATTCATTT-CTAATAAATACTCT-GACTAAGAAATTAGATACCATAGCCCCAGTTACTTCTATTATTGGATCATTGTCGAAAGCTCAATTTTGTACTGTATTGGGTCATCCTATTAGTAAACCGATCTGGACCGATTTATCGGATTCTGATATTTTTGATCGATTTTGTCGGATATGTAGAAATCTTTGTCGTTATCACAGCGGATCTTCAAAGAAACAGGTTTTGTATCGTATAAAGTATATACTTCGACTTTCGTGTGCTAGAACTTTGGCTCGTAAACATAAAAGTACAGTACGCACTTTTATGCGAAGATTAGGTTCGGGATTCTTAG-AAGAATTTTTTTTTGAAGAAGAACAATCTCTTTCTT

Arundina_graminifolia_var_graminifolia_AB844198 ACAAGAATTCTTTTTCTTCTCATTTTTCT------------TCTCAAATGGTATCAGAAGGTTTTGGAGTCATTCTGGAAATTCCATTCTCGTCGCAATTAGTATCT---------TCCCTTGAAG---ATAACAGAATACCAAAATTTCAGAATTTACTATCTATTCATTCAATATTTCCCTTTTTAGAGGATAAATTATCACATTTCAATTATGTGTCAG-ATC-TACT-AATACCCCATCCCATCCATCTGGAAATCTTGGTTCAAATCCTTCAATGCTGGATTAAAGATGTTTCTTCTTTGCATTTCTTGCGATTGTTTTTCCACGAATATCATAATTTGAATAGTCTCATTACTTC----------AAATAAATCCATTTACGTCTTTTCAAAAAGAAC---CAAAAGA-TTCTTTTGG-TTCCTACATAATTCTTATGTATATGAATGCGAATATATATTCCTGTTTCTTCGTAAACAGTCTTCTTATTTACGATCAATATCTTCTGGAGTCTTTCTTGAGCGAACACATTTCTATGGAAAAATAGAATATCTTATAGT---CGTGTGTTGTAATTCTTTTCA---------GAGGATCCTATGGTTCCTCAA-AGATACTTTCATACATTATGTTCGATATCAAGGAAAAACAATTCTGGTTTCAAAAGGAACTCTTATTCTGATTAAGAAAT-GGAAATTTCATCTTGTGAATTTTTGGCAATCTTATTTTCACTTTTGGTTTCAACCTTATAGGATTCATATAAAGCAATTACCCAACTATTCCTTCTCTTTTCTGGGG-TATTTTTCAAGTGTACTAAAAAATAATTTGGTAATAA-GAAATCAAATGCTAGAGAATTCATTT-CTAATAAATACTCT-GACTAAGAAATTAGATACCATAGCCCCAGTTACTTCTATTATTGGATCATTGTCGAAAGCTCAATTTTGTACTGTATTGGGTCATCCTATTAGTAAACCGATCTGGACCGATTTATCGGATTCTGATATTTTTGATCGATTTTGTCGGATATGTAGAAATCTTTGTCGTTATCACAGCGGATCTTCAAAGAAACAGGTTTTGTATCGTATAAAGTATATACTTCGACTTTCGTGTGCTAGAACTTTGGCTCGTAAACATAAAAGTACAGTACGCACTTTTATGCGAAGATTAGGTTCGGGATTCTTAG-AAGAATTTTTTTTTGAAGAAGAACAATCTCTTTCTT

Arundina_graminifolia_var_graminifolia_AB844199 ACAAGAATTCTTTTTCTTCTCATTTTTCT------------TCTCAAATGGTATCAGAAGGTTTTGGAGTCATTCTGGAAATTCCATTCTCGTCGCAATTAGTATCT---------TCCCTTGAAG---ATAACAGAATACCAAAATTTCAGAATTTACTATCTATTCATTCAATATTTCCCTTTTTAGAGGATAAATTATCACATTTCAATTATGTGTCAG-ATC-TACT-AATACCCCATCCCATCCATCTGGAAATCTTGGTTCAAATCCTTCAATGCTGGATTAAAGATGTTTCTTCTTTGCATTTCTTGCGATTGTTTTTCCACGAATATCATAATTTGAATAGTCTCATTACTTC----------AAATAAATCCATTTACGTCTTTTCAAAAAGAAC---CAAAAGA-TTCTTTTGG-TTCCTACATAACTCTTATGTATATGAATGCGAATATATATTCCTGTTTCTTCGTAAACAGTCTTCTTATTTACGATCAATATCTTCTGGAGTCTTTCTTGAGCGAACACATTTCTATGGAAAAATAGAATATCTTATAGT---CGTGTGTTGTAATTCTTTTCA---------GAGGATCCTATGGTTCCTCAA-AGATACTTTCATACATTATGTTCGATATCAAGGAAAAACAATTCTGGTTTCAAAAGGAACTCTTATTCTGATTAAGAAAT-GGAAATTTCATCTTGTGAATTTTTGGCAATCTTATTTTCACTTTTGGTTTCAACCTTATAGGATTCATATAAAGCAATTACCCAACTATTCCTTCTCTTTTCTGGGG-TATTTTTCAAGTGTACTAAAAAATAATTTGGTAATAA-GAAATCAAATGCTAGAGAATTCATTT-CTAATAAATACTCT-GACTAAGAAATTAGATACCATAGCCCCAGTTACTTCTATTATTGGATCATTGTCGAAAGCTCAATTTTGTACTGTATTGGGTCATCCTATTAGTAAACCGATCTGGACCGATTTATCGGATTCTGATATTTTTGATCGATTTTGTCGGATATGTAGAAATCTTTGTCGTTATCACAGCGGATCTTCAAAGAAACAGGTTTTGTATCGTATAAAGTATATACTTCGACTTTCGTGTGCTAGAACTTTGGCTCGTAAACATAAAAGTACAGTACGCACTTTTATGCGAAGATTAGGTTCGGGATTCTTAG-AAGAATTTTTTTTTGAAGAAGAACAATCTCTTTCTT

Arundina_graminifolia_var_graminifolia_AB844200 ACAAGAATTCTTTTTCTTCTCATTTTTCT------------TCTCAAATGGTATCAGAAGGTTTTGGAGTCATTCTGGAAATTCCATTCTCGTCGCAATTAGTATCT---------TCCCTTGAAG---ATAACAGAATACCAAAATTTCAGAATTTACTATCTATTCATTCAATATTTCCCTTTTTAGAGGATAAATTATCACATTTCAATTATGTGTCAG-ATC-TACT-AATACCCCATCCCATCCATCTGGAAATCTTGGTTCAAATCCTTCAATGCTGGATTAAAGATGTTTCTTCTTTGCATTTCTTGCGATTGTTTTTCCACGAATATCATAATTTGAATAGTCTCATTACTTC----------AAATAAATCCATTTACGTCTTTTCAAAAAGAAC---CAAAAGA-TTCTTTTGG-TTCCTACATAACTCTTATGTATATGAATGCGAATATATATTCCTGTTTCTTCGTAAACAGTCTTCTTATTTACGATCAATATCTTCTGGAGTCTTTCTTGAGCGAACACATTTCTATGGAAAAATAGAATATCTTATAGT---CGTGTGTTGTAATTCTTTTCA---------GAGGATCCTATGGTTCCTCAA-AGATACTTTCATACATTATGTTCGATATCAAGGAAAAACAATTCTGGTTTCAAAAGGAACTCTTATTCTGATTAAGAAAT-GGAAATTTCATCTTGTGAATTTTTGGCAATCTTATTTTCACTTTTGGTTTCAACCTTATAGGATTCATATAAAGCAATTACCCAACTATTCCTTCTCTTTTCTGGGG-TATTTTTCAAGTGTACTAAAAAATAATTTGGTAATAA-GAAATCAAATGCTAGAGAATTCATTT-CTAATAAATACTCT-GACTAAGAAATTAGATACCATAGCCCCAGTTACTTCTATTATTGGATCATTGTCGAAAGCTCAATTTTGTACTGTATTGGGTCATCCTATTAGTAAACCGATCTGGACCGATTTATCGGATTCTGATATTTTTGATCGATTTTGTCGGATATGTAGAAATCTTTGTCGTTATCACAGCGGATCTTCAAAGAAACAGGTTTTGTATCGTATAAAGTATATACTTCGACTTTCGTGTGCTAGAACTTTGGCTCGTAAACATAAAAGTACAGTACGCACTTTTATGCGAAGATTAGGTTCGGGATTCTTAG-AAGAATTTTTTTTTGAAGAAGAACAATCTCTTTCTT

Arundina_graminifolia_var_graminifolia_AB872252 ACAAGAATTCTTTTTCTTCTCATTTTTCT------------TCTCAAATGGTATCAGAAGGTTTTGGAGTCATTCTGGAAATTCCATTCTCGTCGCAATTAGTATCT---------TCCCTTGAAG---ATAACAGAATACCAAAATTTCAGAATTTACTATCTATTCATTCAATATTTCCCTTTTTAGAGGATAAATTATCACATTTCAATTATGTGTCAG-ATC-TACT-AATACCCCATCCCATCCATCTGGAAATCTTGGTTCAAATCCTTCAATGCTGGATTAAAGATGTTTCTTCTTTGCATTTCTTGCGATTGTTTTTCCACGAATATCATAATTTGAATAGTCTCATTACTTC----------AAATAAATCCATTTACGTCTTTTCAAAAAGAAC---CAAAAGA-ATCTTTTGG-TTCCTACATAATTCTTATGTATATGAATGCGAATATATATTCCTGTTTCTTCGTAAACAGTCTTCTTATTTACGATCAATATCTTCTGGAGTCTTTCTTGAGCGAACACATTTCTATGGAAAAATAGAATATCTTATAGT---CGTGTGTTGTAATTCTTTTCA---------GAGGATCCTATGGTTCCTCAA-AGATACTTTCATACATTATGTTCGATATCAAGGAAAAACAATTCTGGTTTCAAAAGGAACTCTTATTCTGATTAAGAAAT-GGAAATTTCATCTTGTGAATTTTTGGCAATCTTATTTTCACTTTTGGTTTCAACCTTATAGGATTCATATAAAGCAATTACCCAACTATTCCTTCTCTTTTCTGGGG-TATTTTTCAAGTGTACTAAAAAATAATTTGGTAATAA-GAAATCAAATGCTAGAGAATTCATTT-CTAATAAATACTCT-GACTAAGAAATTAGATACCATAGCCCCAGTTACTTCTATTATTGGATCATTGTCGAAAGCTCAATTTTGTACTGTATTGGGTCATCCTATTAGTAAACCGATCTGGACCGATTTATCGGATTCTGATATTTTTGATCGATTTTGTCGGATATGTAGAAATCTTTGTCGTTATCACAGCGGATCTTCAAAGAAACAGGTTTTGTATCGTATAAAGTATATACTTCGACTTTCGTGTGCTAGAACTTTGGCTCGTAAACATAAAAGTACAGTACGCACTTTTATGCGAAGATTAGGTTCGGGATTCTTAG-AAGAATTTTTTTTTGAAGAAGAACAATCTCTTTCTT

Arundina_graminifolia_var_revoluta_AB844201 ACAAGAATTCTTTTTCTTCTCATTTTTCT------------TCTCAAATGGTATCAGAAGGTTTTGGAGTCATTCTGGAAATTCCATTCTCGTCGCAATTAGTATCT---------TCCCTTGAAG---ATAACAGAATACCAAAATTTCAGAATTTACTATCTATTCATTCAATATTTCCCTTTTTAGAGGATAAATTATCACATTTCAATTATGTGTCAG-ATC-TACT-AATACCCCATCCCATCCATCTGGAAATCTTGGTTCAAATCCTTCAATGCTGGATTAAAGATGTTTCTTCTTTGCATTTCTTGCGATTGTTTTTCCACGAATATCATAATTTGAATAGTCTCATTACTTC----------AAATAAATCCATTTACGTCTTTTCAAAAAGAAC---CAAAAGA-ATCTTTTGG-TTCCTACATAATTCTTATGTATATGAATGCGAATATATATTCCTGTTTCTTCGTAAACAGTCTTCTTATTTACGATCAATATCTTCTGGAGTCTTTCTTGAGCGAACACATTTCTATGGAAAAATAGAATATCTTATAGT---CGTGTGTTGTAATTCTTTTCA---------GAGGATCCTATGGTTCCTCAA-AGATACTTTCATACATTATGTTCGATATCAAGGAAAAACAATTCTGGTTTCAAAAGGAACTCTTATTCTGATTAAGAAAT-GGAAATTTCATCTTGTGAATTTTTGGCAATCTTATTTTCACTTTTGGTTTCAACCTTATAGGATTCATATAAAGCAATTACCCAACTATTCCTTCTCTTTTCTGGGG-TATTTTTCAAGTGTACTAAAAAATAATTTGGTAATAA-GAAATCAAATGCTAGAGAATTCATTT-CTAATAAATACTCT-GACTAAGAAATTAGATACCATAGCCCCAGTTACTTCTATTATTGGATCATTGTCGAAAGCTCAATTTTGTACTGTATTGGGTCATCCTATTAGTAAACCGATCTGGACCGATTTATCGGATTCTGATATTTTTGATCGATTTTGTCGGATATGTAGAAATCTTTGTCGTTATCACAGCGGATCTTCAAAGAAACAGGTTTTGTATCGTATAAAGTATATACTTCGACTTTCGTGTGCTAGAACTTTGGCTCGTAAACATAAAAGTACAGTACGCACTTTTATGCGAAGATTAGGTTCGGGATTCTTAG-AAGAATTTTTTTTTGAAGAAGAACAATCTCTTTCTT

Arundina_graminifolia_var_revoluta_AB844202 ACAAGAATTCTTTTTCTTCTCATTTTTCT------------TCTCAAATGGTATCAGAAGGTTTTGGAGTCATTCTGGAAATTCCATTCTCGTCGCAATTAGTATCT---------TCCCTTGAAG---ATAACAGAATACCAAAATTTCAGAATTTACTATCTATTCATTCAATATTTCCCTTTTTAGAGGATAAATTATCACATTTCAATTATGTGTCAG-ATC-TACT-AATACCCCATCCCATCCATCTGGAAATCTTGGTTCAAATCCTTCAATGCTGGATTAAAGATGTTTCTTCTTTGCATTTCTTGCGATTGTTTTTCCACGAATATCATAATTTGAATAGTCTCATTACTTC----------AAATAAATCCATTTACGTCTTTTCAAAAAGAAC---CAAAAGA-ATCTTTTGG-TTCCTACATAATTCTTATGTATATGAATGCGAATATATATTCCTGTTTCTTCGTAAACAGTCTTCTTATTTACGATCAATATCTTCTGGAGTCTTTCTTGAGCGAACACATTTCTATGGAAAAATAGAATATCTTATAGT---CGTGTGTTGTAATTCTTTTCA---------GAGGATCCTATGGTTCCTCAA-AGATACTTTCATACATTATGTTCGATATCAAGGAAAAACAATTCTGGTTTCAAAAGGAACTCTTATTCTGATTAAGAAAT-GGAAATTTCATCTTGTGAATTTTTGGCAATCTTATTTTCACTTTTGGTTTCAACCTTATAGGATTCATATAAAGCAATTACCCAACTATTCCTTCTCTTTTCTGGGG-TATTTTTCAAGTGTACTAAAAAATAATTTGGTAATAA-GAAATCAAATGCTAGAGAATTCATTT-CTAATAAATACTCT-GACTAAGAAATTAGATACCATAGCCCCAGTTACTTCTATTATTGGATCATTGTCGAAAGCTCAATTTTGTACTGTATTGGGTCATCCTATTAGTAAACCGATCTGGACCGATTTATCGGATTCTGATATTTTTGATCGATTTTGTCGGATATGTAGAAATCTTTGTCGTTATCACAGCGGATCTTCAAAGAAACAGGTTTTGTATCGTATAAAGTATATACTTCGACTTTCGTGTGCTAGAACTTTGGCTCGTAAACATAAAAGTACAGTACGCACTTTTATGCGAAGATTAGGTTCGGGATTCTTAG-AAGAATTTTTTTTTGAAGAAGAACAATCTCTTTCTT

Arundina_graminifolia_var_revoluta_AB844203 ACAAGAATTCTTTTTCTTCTCATTTTTCT------------TCTCAAATGGTATCAGAAGGTTTTGGAGTCATTCTGGAAATTCCATTCTCGTCGCAATTAGTATCT---------TCCCTTGAAG---ATAACAGAATACCAAAATTTCAGAATTTACTATCTATTCATTCAATATTTCCCTTTTTAGAGGATAAATTATCACATTTCAATTATGTGTCAG-ATC-TACT-AATACCCCATCCCATCCATCTGGAAATCTTGGTTCAAATCCTTCAATGCTGGATTAAAGATGTTTCTTCTTTGCATTTCTTGCGATTGTTTTTCCACGAATATCATAATTTGAATAGTCTCATTACTTC----------AAATAAATCCATTTACGTCTTTTCAAAAAGAAC---CAAAAGA-ATCTTTTGG-TTCCTACATAATTCTTATGTATATGAATGCGAATATATATTCCTGTTTCTTCGTAAACAGTCTTCTTATTTACGATCAATATCTTCTGGAGTCTTTCTTGAGCGAACACATTTCTATGGAAAAATAGAATATCTTATAGT---CGTGTGTTGTAATTCTTTTCA---------GAGGATCCTATGGTTCCTCAA-AGATACTTTCATACATTATGTTCGATATCAAGGAAAAACAATTCTGGTTTCAAAAGGAACTCTTATTCTGATTAAGAAAT-GGAAATTTCATCTTGTGAATTTTTGGCAATCTTATTTTCACTTTTGGTTTCAACCTTATAGGATTCATATAAAGCAATTACCCAACTATTCCTTCTCTTTTCTGGGG-TATTTTTCAAGTGTACTAAAAAATAATTTGGTAATAA-GAAATCAAATGCTAGAGAATTCATTT-CTAATAAATACTCT-GACTAAGAAATTAGATACCATAGCCCCAGTTACTTCTATTATTGGATCATTGTCGAAAGCTCAATTTTGTACTGTATTGGGTCATCCTATTAGTAAACCGATCTGGACCGATTTATCGGATTCTGATATTTTTGATCGATTTTGTCGGATATGTAGAAATCTTTGTCGTTATCACAGCGGATCTTCAAAGAAACAGGTTTTGTATCGTATAAAGTATATACTTCGACTTTCGTGTGCTAGAACTTTGGCTCGTAAACATAAAAGTACAGTACGCACTTTTATGCGAAGATTAGGTTCGGGATTCTTAG-AAGAATTTTTTTTTGAAGAAGAACAATCTCTTTCTT

Arundina_graminifolia_var_revoluta_AB844204 ACAAGAATTCTTTTTCTTCTCATTTTTCT------------TCTCAAATGGTATCAGAAGGTTTTGGAGTCATTCTGGAAATTCCATTCTCGTCGCAATTAGTATCT---------TCCCTTGAAG---ATAACAGAATACCAAAATTTCAGAATTTACTATCTATTCATTCAATATTTCCCTTTTTAGAGGATAAATTATCACATTTCAATTATGTGTCAG-ATC-TACT-AATACCCCATCCCATCCATCTGGAAATCTTGGTTCAAATCCTTCAATGCTGGATTAAAGATGTTTCTTCTTTGCATTTCTTGCGATTGTTTTTCCACGAATATCATAATTTGAATAGTCTCATTACTTC----------AAATAAATCCATTTACGTCTTTTCAAAAAGAAC---CAAAAGA-ATCTTTTGG-TTCCTACATAATTCTTATGTATATGAATGCGAATATATATTCCTGTTTCTTCGTAAACAGTCTTCTTATTTACGATCAATATCTTCTGGAGTCTTTCTTGAGCGAACACATTTCTATGGAAAAATAGAATATCTTATAGT---CGTGTGTTGTAATTCTTTTCA---------GAGGATCCTATGGTTCCTCAA-AGATACTTTCATACATTATGTTCGATATCAAGGAAAAACAATTCTGGTTTCAAAAGGAACTCTTATTCTGATTAAGAAAT-GGAAATTTCATCTTGTGAATTTTTGGCAATCTTATTTTCACTTTTGGTTTCAACCTTATAGGATTCATATAAAGCAATTACCCAACTATTCCTTCTCTTTTCTGGGG-TATTTTTCAAGTGTACTAAAAAATAATTTGGTAATAA-GAAATCAAATGCTAGAGAATTCATTT-CTAATAAATACTCT-GACTAAGAAATTAGATACCATAGCCCCAGTTACTTCTATTATTGGATCATTGTCGAAAGCTCAATTTTGTACTGTATTGGGTCATCCTATTAGTAAACCGATCTGGACCGATTTATCGGATTCTGATATTTTTGATCGATTTTGTCGGATATGTAGAAATCTTTGTCGTTATCACAGCGGATCTTCAAAGAAACAGGTTTTGTATCGTATAAAGTATATACTTCGACTTTCGTGTGCTAGAACTTTGGCTCGTAAACATAAAAGTACAGTACGCACTTTTATGCGAAGATTAGGTTCGGGATTCTTAG-AAGAATTTTTTTTTGAAGAAGAACAATCTCTTTCTT

Arundina_graminifolia_var_revoluta_AB844205 ACAAGAATTCTTTTTCTTCTCATTTTTCT------------TCTCAAATGGTATCAGAAGGTTTTGGAGTCATTCTGGAAATTCCATTCTCGTCGCAATTAGTATCT---------TCCCTTGAAG---ATAACAGAATACCAAAATTTCAGAATTTACTATCTATTCATTCAATATTTCCCTTTTTAGAGGATAAATTATCACATTTCAATTATGTGTCAG-ATC-TACT-AATACCCCATCCCATCCATCTGGAAATCTTGGTTCAAATCCTTCAATGCTGGATTAAAGATGTTTCTTCTTTGCATTTCTTGCGATTGTTTTTCCACGAATATCATAATTTGAATAGTCTTATTACTTC----------AAATAAATCCATTTACGTCTTTTCAAAAAGAAC---CAAAAGA-TTCTTTTGG-TTCCTACATAATTCTTATGTATATGAATGCGAATATATATTCCTGTTTCTTCGTAAACAGTCTTCTTATTTACGATCAATATCTTCTGGAGTCTTTCTTGAGCGAACACATTTCTATGGAAAAATAGAATATCTTATAGT---CGTGTGTTGTAATTCTTTTCA---------GAGGATCCTATGGTTCCTCAA-AGATACTTTCATACATTATGTTCGATATCAAGGAAAAACAATTCTGGTTTCAAAAGGAACTCTTATTCTGATTAAGAAAT-GGAAATTTCATCTTGTGAATTTTTGGCAATCTTATTTTCACTTTTGGTTTCAACCTTATAGGATTCATATAAAGCAATTACCCAACTATTCCTTCTCTTTTCTGGGG-TATTTTTCAAGTGTACTAAAAAATAATTTGGTAATAA-GAAATCAAATGCTAGAGAATTCATTT-CTAATAAATACTCT-GACTAAGAAATTAGATACCATAGCCCCAGTTACTTCTATTATTGGATCATTGTCGAAAGCTCAATTTTGTACTGTATTGGGTCATCCTATTAGTAAACCGATCTGGACCGATTTATCGGATTCTGATATTTTTGATCGATTTTGTCGGATATGTAGAAATCTTTGTCGTTATCACAGCGGATCTTCAAAGAAACAGGTTTTGTATCGTATAAAGTATATACTTCGACTTTCGTGTGCTAGAACTTTGGCTCGTAAACATAAAAGTACAGTACGCACTTTTATGCGAAGATTAGGTTCGGGATTCTTAG-AAGAATTTTTTTTTGAAGAAGAACAATCTCTTTCTT

Arundina_graminifolia_var_revoluta_AB844206 ACAAGAATTCTTTTTCTTCTCATTTTTCT------------TCTCAAATGGTATCAGAAGGTTTTGGAGTCATTCTGGAAATTCCATTCTCGTCGCAATTAGTATCT---------TCCCTTGAAG---ATAACAGAATACCAAAATTTCAGAATTTACTATCTATTCATTCAATATTTCCCTTTTTAGAGGATAAATTATCACATTTCAATTATGTGTCAG-ATC-TACT-AATACCCCATCCCATCCATCTGGAAATCTTGGTTCAAATCCTTCAATGCTGGATTAAAGATGTTTCTTCTTTGCATTTCTTGCGATTGTTTTTCCACGAATATCATAATTTGAATAGTCTCATTACTTC----------AAATAAATCCATTTACGTCTTTTCAAAAAGAAC---CAAAAGA-ATCTTTTGG-TTCCTACATAATTCTTATGTATATGAATGCGAATATATATTCCTGTTTCTTCGTAAACAGTCTTCTTATTTACGATCAATATCTTCTGGAGTCTTTCTTGAGCGAACACATTTCTATGGAAAAATAGAATATCTTATAGT---CGTGTGTTGTAATTCTTTTCA---------GAGGATCCTATGGTTCCTCAA-AGATACTTTCATACATTATGTTCGATATCAAGGAAAAACAATTCTGGTTTCAAAAGGAACTCTTATTCTGATTAAGAAAT-GGAAATTTCATCTTGTGAATTTTTGGCAATCTTATTTTCACTTTTGGTTTCAACCTTATAGGATTCATATAAAGCAATTACCCAACTATTCCTTCTCTTTTCTGGGG-TATTTTTCAAGTGTACTAAAAAATAATTTGGTAATAA-GAAATCAAATGCTAGAGAATTCATTT-CTAATAAATACTCT-GACTAAGAAATTAGATACCATAGCCCCAGTTACTTCTATTATTGGATCATTGTCGAAAGCTCAATTTTGTACTGTATTGGGTCATCCTATTAGTAAACCGATCTGGACCGATTTATCGGATTCTGATATTTTTGATCGATTTTGTCGGATATGTAGAAATCTTTGTCGTTATCACAGCGGATCTTCAAAGAAACAGGTTTTGTATCGTATAAAGTATATACTTCGACTTTCGTGTGCTAGAACTTTGGCTCGTAAACATAAAAGTACAGTACGCACTTTTATGCGAAGATTAGGTTCGGGATTCTTAG-AAGAATTTTTTTTTGAAGAAGAACAATCTCTTTCTT

Arundina_graminifolia_var_revoluta_AB844207 ACAAGAATTCTTTTTCTTCTCATTTTTCT------------TCTCAAATGGTATCAGAAGGTTTTGGAGTCATTCTGGAAATTCCATTCTCGTCGCAATTAGTATCT---------TCCCTTGAAG---ATAACAGAATACCAAAATTTCAGAATTTACTATCTATTCATTCAATATTTCCCTTTTTAGAGGATAAATTATCACATTTCAATTATGTGTCAG-ATC-TACT-AATACCCCATCCCATCCATCTGGAAATCTTGGTTCAAATCCTTCAATGCTGGATTAAAGATGTTTCTTCTTTGCATTTCTTGCGATTGTTTTTCCACGAATATCATAATTTGAATAGTCTTATTACTTC----------AAATAAATCCATTTACGTCTTTTCAAAAAGAAC---CAAAAGA-TTCTTTTGG-TTCCTACATAATTCTTATGTATATGAATGCGAATATATATTCCTGTTTCTTCGTAAACAGTCTTCTTATTTACGATCAATATCTTCTGGAGTCTTTCTTGAGCGAACACATTTCTATGGAAAAATAGAATATCTTATAGT---CGTGTGTTGTAATTCTTTTCA---------GAGGATCCTATGGTTCCTCAA-AGATACTTTCATACATTATGTTCGATATCAAGGAAAAACAATTCTGGTTTCAAAAGGAACTCTTATTCTGATTAAGAAAT-GGAAATTTCATCTTGTGAATTTTTGGCAATCTTATTTTCACTTTTGGTTTCAACCTTATAGGATTCATATAAAGCAATTACCCAACTATTCCTTCTCTTTTCTGGGG-TATTTTTCAAGTGTACTAAAAAATAATTTGGTAATAA-GAAATCAAATGCTAGAGAATTCATTT-CTAATAAATACTCT-GACTAAGAAATTAGATACCATAGCCCCAGTTACTTCTATTATTGGATCATTGTCGAAAGCTCAATTTTGTACTGTATTGGGTCATCCTATTAGTAAACCGATCTGGACCGATTTATCGGATTCTGATATTTTTGATCGATTTTGTCGGATATGTAGAAATCTTTGTCGTTATCACAGCGGATCTTCAAAGAAACAGGTTTTGTATCGTATAAAGTATATACTTCGACTTTCGTGTGCTAGAACTTTGGCTCGTAAACATAAAAGTACAGTACGCACTTTTATGCGAAGATTAGGTTCGGGATTCTTAG-AAGAATTTTTTTTTGAAGAAGAACAATCTCTTTCTT

Arundina_graminifolia_var_revoluta_AB844208 ACAAGAATTCTTTTTCTTCTCATTTTTCT------------TCTCAAATGGTATCAGAAGGTTTTGGAGTCATTCTGGAAATTCCATTCTCGTCGCAATTAGTATCT---------TCCCTTGAAG---ATAACAGAATACCAAAATTTCAGAATTTACTATCTATTCATTCAATATTTCCCTTTTTAGAGGATAAATTATCACATTTCAATTATGTGTCAG-ATC-TACT-AATACCCCATCCCATCCATCTGGAAATCTTGGTTCAAATCCTTCAATGCTGGATTAAAGATGTTTCTTCTTTGCATTTCTTGCGATTGTTTTTCCACGAATATCATAATTTGAATAGTCTCATTACTTC----------AAATAAATCCATTTACGTCTTTTCAAAAAGAAC---CAAAAGA-ATCTTTTGG-TTCCTACATAATTCTTATGTATATGAATGCGAATATATATTCCTGTTTCTTCGTAAACAGTCTTCTTATTTACGATCAATATCTTCTGGAGTCTTTCTTGAGCGAACACATTTCTATGGAAAAATAGAATATCTTATAGT---CGTGTGTTGTAATTCTTTTCA---------GAGGATCCTATGGTTCCTCAA-AGATACTTTCATACATTATGTTCGATATCAAGGAAAAACAATTCTGGTTTCAAAAGGAACTCTTATTCTGATTAAGAAAT-GGAAATTTCATCTTGTGAATTTTTGGCAATCTTATTTTCACTTTTGGTTTCAACCTTATAGGATTCATATAAAGCAATTACCCAACTATTCCTTCTCTTTTCTGGGG-TATTTTTCAAGTGTACTAAAAAATAATTTGGTAATAA-GAAATCAAATGCTAGAGAATTCATTT-CTAATAAATACTCT-GACTAAGAAATTAGATACCATAGCCCCAGTTACTTCTATTATTGGATCATTGTCGAAAGCTCAATTTTGTACTGTATTGGGTCATCCTATTAGTAAACCGATCTGGACCGATTTATCGGATTCTGATATTTTTGATCGATTTTGTCGGATATGTAGAAATCTTTGTCGTTATCACAGCGGATCTTCAAAGAAACAGGTTTTGTATCGTATAAAGTATATACTTCGACTTTCGTGTGCTAGAACTTTGGCTCGTAAACATAAAAGTACAGTACGCACTTTTATGCGAAGATTAGGTTCGGGATTCTTAG-AAGAATTTTTTTTTGAAGAAGAACAATCTCTTTCTT

Arundina_graminifolia_var_revoluta_AB844209 ACAAGAATTCTTTTTCTTCTCATTTTTCT------------TCTCAAATGGTATCAGAAGGTTTTGGAGTCATTCTGGAAATTCCATTCTCGTCGCAATTAGTATCT---------TCCCTTGAAG---ATAACAGAATACCAAAATTTCAGAATTTACTATCTATTCATTCAATATTTCCCTTTTTAGAGGATAAATTATCACATTTCAATTATGTGTCAG-ATC-TACT-AATACCCCATCCCATCCATCTGGAAATCTTGGTTCAAATCCTTCAATGCTGGATTAAAGATGTTTCTTCTTTGCATTTCTTGCGATTGTTTTTCCACGAATATCATAATTTGAATAGTCTTATTACTTC----------AAATAAATCCATTTACGTCTTTTCAAAAAGAAC---CAAAAGA-TTCTTTTGG-TTCCTACATAATTCTTATGTATATGAATGCGAATATATATTCCTGTTTCTTCGTAAACAGTCTTCTTATTTACGATCAATATCTTCTGGAGTCTTTCTTGAGCGAACACATTTCTATGGAAAAATAGAATATCTTATAGT---CGTGTGTTGTAATTCTTTTCA---------GAGGATCCTATGGTTCCTCAA-AGATACTTTCATACATTATGTTCGATATCAAGGAAAAACAATTCTGGTTTCAAAAGGAACTCTTATTCTGATTAAGAAAT-GGAAATTTCATCTTGTGAATTTTTGGCAATCTTATTTTCACTTTTGGTTTCAACCTTATAGGATTCATATAAAGCAATTACCCAACTATTCCTTCTCTTTTCTGGGG-TATTTTTCAAGTGTACTAAAAAATAATTTGGTAATAA-GAAATCAAATGCTAGAGAATTCATTT-CTAATAAATACTCT-GACTAAGAAATTAGATACCATAGCCCCAGTTACTTCTATTATTGGATCATTGTCGAAAGCTCAATTTTGTACTGTATTGGGTCATCCTATTAGTAAACCGATCTGGACCGATTTATCGGATTCTGATATTTTTGATCGATTTTGTCGGATATGTAGAAATCTTTGTCGTTATCACAGCGGATCTTCAAAGAAACAGGTTTTGTATCGTATAAAGTATATACTTCGACTTTCGTGTGCTAGAACTTTGGCTCGTAAACATAAAAGTACAGTACGCACTTTTATGCGAAGATTAGGTTCGGGATTCTTAG-AAGAATTTTTTTTTGAAGAAGAACAATCTCTTTCTT

Arundina_graminifolia_var_revoluta_AB844210 ACAAGAATTCTTTTTCTTCTCATTTTTCT------------TCTCAAATGGTATCAGAAGGTTTTGGAGTCATTCTGGAAATTCCATTCTCGTCGCAATTAGTATCT---------TCCCTTGAAG---ATAACAGAATACCAAAATTTCAGAATTTACTATCTATTCATTCAATATTTCCCTTTTTAGAGGATAAATTATCACATTTCAATTATGTGTCAG-ATC-TACT-AATACCCCATCCCATCCATCTGGAAATCTTGGTTCAAATCCTTCAATGCTGGATTAAAGATGTTTCTTCTTTGCATTTCTTGCGATTGTTTTTCCACGAATATCATAATTTGAATAGTCTTATTACTTC----------AAATAAATCCATTTACGTCTTTTCAAAAAGAAC---CAAAAGA-TTCTTTTGG-TTCCTACATAATTCTTATGTATATGAATGCGAATATATATTCCTGTTTCTTCGTAAACAGTCTTCTTATTTACGATCAATATCTTCTGGAGTCTTTCTTGAGCGAACACATTTCTATGGAAAAATAGAATATCTTATAGT---CGTGTGTTGTAATTCTTTTCA---------GAGGATCCTATGGTTCCTCAA-AGATACTTTCATACATTATGTTCGATATCAAGGAAAAACAATTCTGGTTTCAAAAGGAACTCTTATTCTGATTAAGAAAT-GGAAATTTCATCTTGTGAATTTTTGGCAATCTTATTTTCACTTTTGGTTTCAACCTTATAGGATTCATATAAAGCAATTACCCAACTATTCCTTCTCTTTTCTGGGG-TATTTTTCAAGTGTACTAAAAAATAATTTGGTAATAA-GAAATCAAATGCTAGAGAATTCATTT-CTAATAAATACTCT-GACTAAGAAATTAGATACCATAGCCCCAGTTACTTCTATTATTGGATCATTGTCGAAAGCTCAATTTTGTACTGTATTGGGTCATCCTATTAGTAAACCGATCTGGACCGATTTATCGGATTCTGATATTTTTGATCGATTTTGTCGGATATGTAGAAATCTTTGTCGTTATCACAGCGGATCTTCAAAGAAACAGGTTTTGTATCGTATAAAGTATATACTTCGACTTTCGTGTGCTAGAACTTTGGCTCGTAAACATAAAAGTACAGTACGCACTTTTATGCGAAGATTAGGTTCGGGATTCTTAG-AAGAATTTTTTTTTGAAGAAGAACAATCTCTTTCTT

Arundina_graminifolia_var_revoluta_AB844211 ACAAGAATTCTTTTTCTTCTCATTTTTCT------------TCTCAAATGGTATCAGAAGGTTTTGGAGTCATTCTGGAAATTCCATTCTCGTCGCAATTAGTATCT---------TCCCTTGAAG---ATAACAGAATACCAAAATTTCAGAATTTACTATCTATTCATTCAATATTTCCCTTTTTAGAGGATAAATTATCACATTTCAATTATGTGTCAG-ATC-TACT-AATACCCCATCCCATCCATCTGGAAATCTTGGTTCAAATCCTTCAATGCTGGATTAAAGATGTTTCTTCTTTGCATTTCTTGCGATTGTTTTTCCACGAATATCATAATTTGAATAGTCTCATTACTTC----------AAATAAATCCATTTACGTCTTTTCAAAAAGAAC---CAAAAGA-ATCTTTTGG-TTCCTACATAATTCTTATGTATATGAATGCGAATATATATTCCTGTTTCTTCGTAAACAGTCTTCTTATTTACGATCAATATCTTCTGGAGTCTTTCTTGAGCGAACACATTTCTATGGAAAAATAGAATATCTTATAGT---CGTGTGTTGTAATTCTTTTCA---------GAGGATCCTATGGTTCCTCAA-AGATACTTTCATACATTATGTTCGATATCAAGGAAAAACAATTCTGGTTTCAAAAGGAACTCTTATTCTGATTAAGAAAT-GGAAATTTCATCTTGTGAATTTTTGGCAATCTTATTTTCACTTTTGGTTTCAACCTTATAGGATTCATATAAAGCAATTACCCAACTATTCCTTCTCTTTTCTGGGG-TATTTTTCAAGTGTACTAAAAAATAATTTGGTAATAA-GAAATCAAATGCTAGAGAATTCATTT-CTAATAAATACTCT-GACTAAGAAATTAGATACCATAGCCCCAGTTACTTCTATTATTGGATCATTGTCGAAAGCTCAATTTTGTACTGTATTGGGTCATCCTATTAGTAAACCGATCTGGACCGATTTATCGGATTCTGATATTTTTGATCGATTTTGTCGGATATGTAGAAATCTTTGTCGTTATCACAGCGGATCTTCAAAGAAACAGGTTTTGTATCGTATAAAGTATATACTTCGACTTTCGTGTGCTAGAACTTTGGCTCGTAAACATAAAAGTACAGTACGCACTTTTATGCGAAGATTAGGTTCGGGATTCTTAG-AAGAATTTTTTTTTGAAGAAGAACAATCTCTTTCTT

Arundina_graminifolia_var_revoluta_AB844212 ACAAGAATTCTTTTTCTTCTCATTTTTCT------------TCTCAAATGGTATCAGAAGGTTTTGGAGTCATTCTGGAAATTCCATTCTCGTCGCAATTAGTATCT---------TCCCTTGAAG---ATAACAGAATACCAAAATTTCAGAATTTACTATCTATTCATTCAATATTTCCCTTTTTAGAGGATAAATTATCACATTTCAATTATGTGTCAG-ATC-TACT-AATACCCCATCCCATCCATCTGGAAATCTTGGTTCAAATCCTTCAATGCTGGATTAAAGATGTTTCTTCTTTGCATTTCTTGCGATTGTTTTTCCACGAATATCATAATTTGAATAGTCTTATTACTTC----------AAATAAATCCATTTACGTCTTTTCAAAAAGAAC---CAAAAGA-TTCTTTTGG-TTCCTACATAATTCTTATGTATATGAATGCGAATATATATTCCTGTTTCTTCGTAAACAGTCTTCTTATTTACGATCAATATCTTCTGGAGTCTTTCTTGAGCGAACACATTTCTATGGAAAAATAGAATATCTTATAGT---CGTGTGTTGTAATTCTTTTCA---------GAGGATCCTATGGTTCCTCAA-AGATACTTTCATACATTATGTTCGATATCAAGGAAAAACAATTCTGGTTTCAAAAGGAACTCTTATTCTGATTAAGAAAT-GGAAATTTCATCTTGTGAATTTTTGGCAATCTTATTTTCACTTTTGGTTTCAACCTTATAGGATTCATATAAAGCAATTACCCAACTATTCCTTCTCTTTTCTGGGG-TATTTTTCAAGTGTACTAAAAAATAATTTGGTAATAA-GAAATCAAATGCTAGAGAATTCATTT-CTAATAAATACTCT-GACTAAGAAATTAGATACCATAGCCCCAGTTACTTCTATTATTGGATCATTGTCGAAAGCTCAATTTTGTACTGTATTGGGTCATCCTATTAGTAAACCGATCTGGACCGATTTATCGGATTCTGATATTTTTGATCGATTTTGTCGGATATGTAGAAATCTTTGTCGTTATCACAGCGGATCTTCAAAGAAACAGGTTTTGTATCGTATAAAGTATATACTTCGACTTTCGTGTGCTAGAACTTTGGCTCGTAAACATAAAAGTACAGTACGCACTTTTATGCGAAGATTAGGTTCGGGATTCTTAG-AAGAATTTTTTTTTGAAGAAGAACAATCTCTTTCTT

Arundina_graminifolia_var_revoluta_AB844213 ACAAGAATTCTTTTTCTTCTCATTTTTCT------------TCTCAAATGGTATCAGAAGGTTTTGGAGTCATTCTGGAAATTCCATTCTCGTCGCAATTAGTATCT---------TCCCTTGAAG---ATAACAGAATACCAAAATTTCAGAATTTACTATCTATTCATTCAATATTTCCCTTTTTAGAGGATAAATTATCACATTTCAATTATGTGTCAG-ATC-TACT-AATACCCCATCCCATCCATCTGGAAATCTTGGTTCAAATCCTTCAATGCTGGATTAAAGATGTTTCTTCTTTGCATTTCTTGCGATTGTTTTTCCACGAATATCATAATTTGAATAGTCTCATTACTTC----------AAATAAATCCATTTACGTCTTTTCAAAAAGAAC---CAAAAGA-ATCTTTTGG-TTCCTACATAATTCTTATGTATATGAATGCGAATATATATTCCTGTTTCTTCGTAAACAGTCTTCTTATTTACGATCAATATCTTCTGGAGTCTTTCTTGAGCGAACACATTTCTATGGAAAAATAGAATATCTTATAGT---CGTGTGTTGTAATTCTTTTCA---------GAGGATCCTATGGTTCCTCAA-AGATACTTTCATACATTATGTTCGATATCAAGGAAAAACAATTCTGGTTTCAAAAGGAACTCTTATTCTGATTAAGAAAT-GGAAATTTCATCTTGTGAATTTTTGGCAATCTTATTTTCACTTTTGGTTTCAACCTTATAGGATTCATATAAAGCAATTACCCAACTATTCCTTCTCTTTTCTGGGG-TATTTTTCAAGTGTACTAAAAAATAATTTGGTAATAA-GAAATCAAATGCTAGAGAATTCATTT-CTAATAAATACTCT-GACTAAGAAATTAGATACCATAGCCCCAGTTACTTCTATTATTGGATCATTGTCGAAAGCTCAATTTTGTACTGTATTGGGTCATCCTATTAGTAAACCGATCTGGACCGATTTATCGGATTCTGATATTTTTGATCGATTTTGTCGGATATGTAGAAATCTTTGTCGTTATCACAGCGGATCTTCAAAGAAACAGGTTTTGTATCGTATAAAGTATATACTTCGACTTTCGTGTGCTAGAACTTTGGCTCGTAAACATAAAAGTACAGTACGCACTTTTATGCGAAGATTAGGTTCGGGATTCTTAG-AAGAATTTTTTTTTGAAGAAGAACAATCTCTTTCTT

Arundina_graminifolia_var_revoluta_AB844214 ACAAGAATTCTTTTTCTTCTCATTTTTCT------------TCTCAAATGGTATCAGAAGGTTTTGGAGTCATTCTGGAAATTCCATTCTCGTCGCAATTAGTATCT---------TCCCTTGAAG---ATAACAGAATACCAAAATTTCAGAATTTACTATCTATTCATTCAATATTTCCCTTTTTAGAGGATAAATTATCACATTTCAATTATGTGTCAG-ATC-TACT-AATACCCCATCCCATCCATCTGGAAATCTTGGTTCAAATCCTTCAATGCTGGATTAAAGATGTTTCTTCTTTGCATTTCTTGCGATTGTTTTTCCACGAATATCATAATTTGAATAGTCTCATTACTTC----------AAATAAATCCATTTACGTCTTTTCAAAAAGAAC---CAAAAGA-ATCTTTTGG-TTCCTACATAATTCTTATGTATATGAATGCGAATATATATTCCTGTTTCTTCGTAAACAGTCTTCTTATTTACGATCAATATCTTCTGGAGTCTTTCTTGAGCGAACACATTTCTATGGAAAAATAGAATATCTTATAGT---CGTGTGTTGTAATTCTTTTCA---------GAGGATCCTATGGTTCCTCAA-AGATACTTTCATACATTATGTTCGATATCAAGGAAAAACAATTCTGGTTTCAAAAGGAACTCTTATTCTGATTAAGAAAT-GGAAATTTCATCTTGTGAATTTTTGGCAATCTTATTTTCACTTTTGGTTTCAACCTTATAGGATTCATATAAAGCAATTACCCAACTATTCCTTCTCTTTTCTGGGG-TATTTTTCAAGTGTACTAAAAAATAATTTGGTAATAA-GAAATCAAATGCTAGAGAATTCATTT-CTAATAAATACTCT-GACTAAGAAATTAGATACCATAGCCCCAGTTACTTCTATTATTGGATCATTGTCGAAAGCTCAATTTTGTACTGTATTGGGTCATCCTATTAGTAAACCGATCTGGACCGATTTATCGGATTCTGATATTTTTGATCGATTTTGTCGGATATGTAGAAATCTTTGTCGTTATCACAGCGGATCTTCAAAGAAACAGGTTTTGTATCGTATAAAGTATATACTTCGACTTTCGTGTGCTAGAACTTTGGCTCGTAAACATAAAAGTACAGTACGCACTTTTATGCGAAGATTAGGTTCGGGATTCTTAG-AAGAATTTTTTTTTGAAGAAGAACAATCTCTTTCTT

Arundina_graminifolia_var_revoluta_AB844215 ACAAGAATTCTTTTTCTTCTCATTTTTCT------------TCTCAAATGGTATCAGAAGGTTTTGGAGTCATTCTGGAAATTCCATTCTCGTCGCAATTAGTATCT---------TCCCTTGAAG---ATAACAGAATACCAAAATTTCAGAATTTACTATCTATTCATTCAATATTTCCCTTTTTAGAGGATAAATTATCACATTTCAATTATGTGTCAG-ATC-TACT-AATACCCCATCCCATCCATCTGGAAATCTTGGTTCAAATCCTTCAATGCTGGATTAAAGATGTTTCTTCTTTGCATTTCTTGCGATTGTTTTTCCACGAATATCATAATTTGAATAGTCTCATTACTTC----------AAATAAATCCATTTACGTCTTTTCAAAAAGAAC---CAAAAGA-ATCTTTTGG-TTCCTACATAATTCTTATGTATATGAATGCGAATATATATTCCTGTTTCTTCGTAAACAGTCTTCTTATTTACGATCAATATCTTCTGGAGTCTTTCTTGAGCGAACACATTTCTATGGAAAAATAGAATATCTTATAGT---CGTGTGTTGTAATTCTTTTCA---------GAGGATCCTATGGTTCCTCAA-AGATACTTTCATACATTATGTTCGATATCAAGGAAAAACAATTCTGGTTTCAAAAGGAACTCTTATTCTGATTAAGAAAT-GGAAATTTCATCTTGTGAATTTTTGGCAATCTTATTTTCACTTTTGGTTTCAACCTTATAGGATTCATATAAAGCAATTACCCAACTATTCCTTCTCTTTTCTGGGG-TATTTTTCAAGTGTACTAAAAAATAATTTGGTAATAA-GAAATCAAATGCTAGAGAATTCATTT-CTAATAAATACTCT-GACTAAGAAATTAGATACCATAGCCCCAGTTACTTCTATTATTGGATCATTGTCGAAAGCTCAATTTTGTACTGTATTGGGTCATCCTATTAGTAAACCGATCTGGACCGATTTATCGGATTCTGATATTTTTGATCGATTTTGTCGGATATGTAGAAATCTTTGTCGTTATCACAGCGGATCTTCAAAGAAACAGGTTTTGTATCGTATAAAGTATATACTTCGACTTTCGTGTGCTAGAACTTTGGCTCGTAAACATAAAAGTACAGTACGCACTTTTATGCGAAGATTAGGTTCGGGATTCTTAG-AAGAATTTTTTTTTGAAGAAGAACAATCTCTTTCTT

Arundina_graminifolia_var_revoluta_AB844216 ACAAGAATTCTTTTTCTTCTCATTTTTCT------------TCTCAAATGGTATCAGAAGGTTTTGGAGTCATTCTGGAAATTCCATTCTCGTCGCAATTAGTATCT---------TCCCTTGAAG---ATAACAGAATACCAAAATTTCAGAATTTACTATCTATTCATTCAATATTTCCCTTTTTAGAGGATAAATTATCACATTTCAATTATGTGTCAG-ATC-TACT-AATACCCCATCCCATCCATCTGGAAATCTTGGTTCAAATCCTTCAATGCTGGATTAAAGATGTTTCTTCTTTGCATTTCTTGCGATTGTTTTTCCACGAATATCATAATTTGAATAGTCTCATTACTTC----------AAATAAATCCATTTACGTCTTTTCAAAAAGAAC---CAAAAGA-ATCTTTTGG-TTCCTACATAATTCTTATGTATATGAATGCGAATATATATTCCTGTTTCTTCGTAAACAGTCTTCTTATTTACGATCAATATCTTCTGGAGTCTTTCTTGAGCGAACACATTTCTATGGAAAAATAGAATATCTTATAGT---CGTGTGTTGTAATTCTTTTCA---------GAGGATCCTATGGTTCCTCAA-AGATACTTTCATACATTATGTTCGATATCAAGGAAAAACAATTCTGGTTTCAAAAGGAACTCTTATTCTGATTAAGAAAT-GGAAATTTCATCTTGTGAATTTTTGGCAATCTTATTTTCACTTTTGGTTTCAACCTTATAGGATTCATATAAAGCAATTACCCAACTATTCCTTCTCTTTTCTGGGG-TATTTTTCAAGTGTACTAAAAAATAATTTGGTAATAA-GAAATCAAATGCTAGAGAATTCATTT-CTAATAAATACTCT-GACTAAGAAATTAGATACCATAGCCCCAGTTACTTCTATTATTGGATCATTGTCGAAAGCTCAATTTTGTACTGTATTGGGTCATCCTATTAGTAAACCGATCTGGACCGATTTATCGGATTCTGATATTTTTGATCGATTTTGTCGGATATGTAGAAATCTTTGTCGTTATCACAGCGGATCTTCAAAGAAACAGGTTTTGTATCGTATAAAGTATATACTTCGACTTTCGTGTGCTAGAACTTTGGCTCGTAAACATAAAAGTACAGTACGCACTTTTATGCGAAGATTAGGTTCGGGATTCTTAG-AAGAATTTTTTTTTGAAGAAGAACAATCTCTTTCTT

Arundina_graminifolia_var_revoluta_AB844217 ACAAGAATTCTTTTTCTTCTCATTTTTCT------------TCTCAAATGGTATCAGAAGGTTTTGGAGTCATTCTGGAAATTCCATTCTCGTCGCAATTAGTATCT---------TCCCTTGAAG---ATAACAGAATACCAAAATTTCAGAATTTACTATCTATTCATTCAATATTTCCCTTTTTAGAGGATAAATTATCACATTTCAATTATGTGTCAG-ATC-TACT-AATACCCCATCCCATCCATCTGGAAATCTTGGTTCAAATCCTTCAATGCTGGATTAAAGATGTTTCTTCTTTGCATTTCTTGCGATTGTTTTTCCACGAATATCATAATTTGAATAGTCTCATTACTTC----------AAATAAATCCATTTACGTCTTTTCAAAAAGAAC---CAAAAGA-ATCTTTTGG-TTCCTACATAATTCTTATGTATATGAATGCGAATATATATTCCTGTTTCTTCGTAAACAGTCTTCTTATTTACGATCAATATCTTCTGGAGTCTTTCTTGAGCGAACACATTTCTATGGAAAAATAGAATATCTTATAGT---CGTGTGTTGTAATTCTTTTCA---------GAGGATCCTATGGTTCCTCAA-AGATACTTTCATACATTATGTTCGATATCAAGGAAAAACAATTCTGGTTTCAAAAGGAACTCTTATTCTGATTAAGAAAT-GGAAATTTCATCTTGTGAATTTTTGGCAATCTTATTTTCACTTTTGGTTTCAACCTTATAGGATTCATATAAAGCAATTACCCAACTATTCCTTCTCTTTTCTGGGG-TATTTTTCAAGTGTACTAAAAAATAATTTGGTAATAA-GAAATCAAATGCTAGAGAATTCATTT-CTAATAAATACTCT-GACTAAGAAATTAGATACCATAGCCCCAGTTACTTCTATTATTGGATCATTGTCGAAAGCTCAATTTTGTACTGTATTGGGTCATCCTATTAGTAAACCGATCTGGACCGATTTATCGGATTCTGATATTTTTGATCGATTTTGTCGGATATGTAGAAATCTTTGTCGTTATCACAGCGGATCTTCAAAGAAACAGGTTTTGTATCGTATAAAGTATATACTTCGACTTTCGTGTGCTAGAACTTTGGCTCGTAAACATAAAAGTACAGTACGCACTTTTATGCGAAGATTAGGTTCGGGATTCTTAG-AAGAATTTTTTTTTGAAGAAGAACAATCTCTTTCTT

Bletilla_striata_AF263630 ACAAGAATTCTTTTTCTTCTCATTTTTCT------------TCTCAAATGGTATCAGAAGGTTTTGGAGTCATTCTGGAAATTCCATTCTCGTCGCGATTAGTATCT---------TCCCTTGAAG---AAAAAAGAATACCAAAATCTCAGAATTTACGATCTATTCATTCAATATTTCCCTTTTTAGAGGATAAATTATCACATTTAAATTATGTGTCAG-ATC-TACT-AATACCCCATCCCATACATCTGGAAATCTTGGTTCAAATCCTTCAATGCTGGATCAAAGATGTTTCTTCTTTGCATTTCTTGCGATTGTTTTTCCATGAATATCATAATTTGAATAGTCTCATTACTTC----------AAATAAATCCACTTACGTCTTTTCAAAAAGAAA---GAAAAGA-TTCTTTTGG-TTCCTACATAATTCTTATGTATATGAATGCGAATATCTATTCCTGTTTCTTCGTAAAAAGTCTTCTTATTTACGATCAATATCTTCTGGAGTTTTTCTTGAGCGAACACATTTCTATGGAAAAATAGAATATCTTATAGT---CGTGTGTTGTAATTCTTTTCA---------GAGTATCCTATGGTTCCTCAA-AGATACTTTCATACATTATGTTCGATATCAAGGAAAAGCAATTCTGGCTTCAAAAGGAACTCTTATTCTGATGAAGAAAT-GGAAGTTTTATCTTGTGAATTTTTGGCAATCTTATTTTCACTTTTGGTTTCAACCTTATAGGATCTATATAAAGCAATTACCCAACTATTCCTTCTCTTTTCTGGGA-TATTTCTCAAGTGTACTAAAAAATCCTTTGGTAGTAA-GAAATCAAATGCTAGATAATTCATTT-CTAATAAATACTCT-GACTAAGAAATTAGATACCGGAGCCCCAGTTATTTCTCTTATTGGATCATTGTCGAAAGCTCAATTTTGTACTGTATTGGGTCATCCTATTAGTAAACCGATCTGGACCGATTTATCGGATTCTGATATTCTTGATCGATTTTGTCGGATATGTAGAAATCTTTGTCGTTATCACAGCGGATCCTCAAAGAAGCAGGTTTTGTATCGTATAAAGTATATACTTCGACTTTCGTGTGCTAGAACTTTGGCTCGCAAACATAAAAGTACAGTACGCACTTTTATGCGAAGATTAGGTTCGGGATTCTTAG-AAGAATTTTTTTTGGAAGAAGAACAATCTCTTTCTT

Bletilla_striata_EF079331 ACAAGAATTCTTTTTCTTCTCATTTTTCT------------TCTCAAATGGTATCAGAAGGTTTTGGAGTCATTCTGGAAATTCCATTCTCGTCGCGATTAGTATCT---------TCCCTTGAAG---AAAAAAGAATACCAAAATCTCAGAATTTACGATCTATTCATTCAATATTTCCCTTTTTAGAGGATAAATTATCACATTTAAATTATGTGTCAG-ATC-TACT-AATACCCCATCCCATACATCTGGAAATCTTGGTTCAAATCCTTCAATGCTGGATCAAAGATGTTTCTTCTTTGCATTTCTTGCGATTGTTTTTCCATGAATATCATAATTTGAATAGTCTCATTACTTC----------AAATAAATCCACTTACGTCTTTTCAAAAAGAAA---GAAAAGA-TTCTTTTGG-TTCCTACATAATTCTTATGTATATGAATGCGAATATCTATTCCTGTTTCTTCGTAAAAAGTCTTCTTATTTACGATCAATATCTTCTGGAGTTTTTCTTGAGCGAACACATTTCTATGGAAAAATAGAATATCTTATAGT---CGTGTGTTGTAATTCTTTTCA---------GAGTATCCTATGGTTCCTCAA-AGATACTTTCATACATTATGTTCGATATCAAGGAAAAGCAATTCTGGCTTCAAAAGGAACTCTTATTCTGATGAAGAAAT-GGAAATTTTATCTTGTGAATTTTTGGCAATCTTATTTTCACTTTTGGTTTCAACCTTATAGGATCTATATAAAGCAATTACCCAACTATTCCTTCTCTTTTCTGGGA-TATTTTTCAAGTGTACTAAAAAATCCTTTGGTAGTAA-GAAATCAAATGCTAGATAATTCATTT-CTAATAAATACTCT-GACTAAGAAATTAGATACCGTAGCCCCAGTTATTTCTCTTATTGGATCATTGTCGAAAGCTCAATTTTGTACTGTATTGGGTCATCCTATTAGTAAACCGATCTGGACCGATTTATCGGATTCTGATATTCTTGATCGATTTTGTCGGATATGTAGAAATCTTTGTCGTTATCACAGCGGATCCTCAAAGAAACAGGTTTTGTATCGTATAAAGTATATACTTCGACTTTCGTGTGCTAGAACTTTGGCTCGTAAACGTAAAAGTACCGTACGCACTTTTATGCGAAGATTAGGTTCGGGATTCTTAG-AAGAATTTTTTTTGGAAGAAGAACAATCTCTTTCTT

Bletilla_striata_EU490679 ACAAGAATTCTTTTTCTTCTCATTTTTCT------------TCTCAAATGGTATCAGAAGGTTTTGGAGTCATTCTGGAAATTCCATTCTCGTCGCGATTAGTATCT---------TCCCTTGAAG---AAAAAAGAATACCAAAATCTCAGAATTTACGATCTATTCATTCAATATTTCCCTTTTTAGAGGATAAATTATCACATTTAAATTATGTGTCAG-ATC-TACT-AATACCCCATCCCATACATCTGGAAATCTTGGTTCAAATCCTTCAATGCTGGATCAAAGATGTTTCTTCTTTGCATTTCTTGCGATTGTTTTTCCATGAATATCATAATTTGAATAGTCTCATTACTTC----------AAATAAATCCACTTACGTCTTTTCAAAAAGAAA---GAAAAGA-TTCTTTTGG-TTCCTACATAATTCTTATGTATATGAATGCGAATATCTATTCCTGTTTCTTCGTAAAAAGTCTTCTTATTTACGATCAATATCTTCTGGAGTTTTTCTTGAGCGAACACATTTCTATGGAAAAATAGAATATCTTATAGT---CGTGTGTTGTAATTCTTTTCA---------GAGTATCCTATGGTTCCTCAA-AGATACTTTCATACATTATGTTCGATATCAAGGAAAAGCAATTCTGGCTTCAAAAGGAACTCTTATTCTGATGAAGAAAT-GGAAATTTTATCTTGTGAATTTTTGGCAATCTTATTTTCACTTTTGGTTTCAACCTTATAGGATCTATATAAAGCAATTACCCAACTATTCCTTCTCTTTTCTGGGA-TATTTTTCAAGTGTACTAAAAAATCCTTTGGTAGTAA-GAAATCAAATGCTAGATAATTCATTT-CTAATAAATACTCT-GACTAAGAAATTAGATACCGTAGCCCCAGTTATTTCTCTTATTGGATCATTGTCGAAAGCTCAATTTTGTACTGTATTGGGTCATCCTATTAGTAAACCGATCTGGACCGATTTATCGGATTCTGATATTCTTGATCGATTTTGTCGGATATGTAGAAATCTTTGTCGTTATCACAGCGGATCCTCAAAGAAACAGGTTTTGTATCGTATAAAGTATATACTTCGACTTTCGTGTGCTAGAACTTTGGCTCGTAAACATAAAAGTACAGTACGCACTTTTATGCGAAGATTAGGTTCGGGATTCTTAG-AAGAATTTTTTTTGGAAGAAGAA-------------

Bletilla_striata_GQ434129 -------------------------------------------------------------------------------------------------------------------------------------------------------------------------------------------------------------------------------ATC-TACT-AATACCCCATCCCATACATCTGGAAATCTTGGTTCAAATCCTTCAATGCTGGATCAAAGATGTTTCTTCTTTGCATTTCTTGCGATTGTTTTTCCATGAATATCATAATTTGAATAGTCTCATTACTTC----------AAATAAATCCACTTACGTCTTTTCAAAAAGAAA---GAAAAGA-TTCTTTTGG-TTCCTACATAATTCTTATGTATATGAATGCGAATATCTATTCCTGTTTCTTCGTAAAAAGTCTTCTTATTTACGATCAATATCTTCTGGAGTTTTTCTTGAGCGAACACATTTCTATGGAAAAATAGAATATCTTATAGT---CGTGTGTTGTAATTCTTTTCA---------GAGTATCCTATGGTTCCTCAA-AGATACTTTCATACATTATGTTCGATATCAAGGAAAAGCAATTCTGGCTTCAAAAGGAACTCTTATTCTGATGAAGAAAT-GGAAATTTTATCTTGTGAATTTTTGGCAATCTTATTTTCACTTTTGGTTTCAACCTTATAGGATCTATATAAAGCAATTACCCAACTATTCCTTCTCTTTTCTGGGA-TATTTTTCAAGTGTACTAAAAAATCCTTTGGTAGTAA-GAAATCAAATGCTAGATAATTCATTT-CTAATAAATACTCT-GACTAAGAAATTAGATACCGTAGCCCCAGTTATTTCTCTTATTGGATCATTGTCGAAAGCTCAATTTTGTACTGTATTGGGTCATCCTATTAGTAAACCGATCTGGACCGATTTATCGGATTCTGATATTCTTGATCGATTTTGTCGGATATGTAGAAATCTTTGTC----------------------------------------------------------------------------------------------------------------------------------------------------------------------------------

Bletilla_striata_KC704596 ----------------------------------------------------------------------------------------------------------------------------------------------------------------------------------------------------------------------------------------------------------------------------AATCCTTCAATGCTGGATCAAAGATGTTTCTTCTTTGCATTTCTTGCGATTGTTTTTCCATGAATATCATAATTTGAATAGTCTCATTACTTC----------AAATAAATCCACTTACGTCTTTTCAAAAAGAAA---GAAAAGA-TTCTTTTGG-TTCCTACATAATTCTTATGTATATGAATGCGAATATCTATTCCTGTTTCTTCGTAAAAAGTCTTCTTATTTACGATCAATATCTTCTGGAGTTTTTCTTGAGCGAACACATTTCTATGGAAAAATAGAATATCTTATAGT---CGTGTGTTGTAATTCTTTTCA---------GAGTATCCTATGGTTCCTCAA-AGATACTTTCATACATTATGTTCGATATCAAGGAAAAGCAATTCTGGCTTCAAAAGGAACTCTTATTCTGATGAAGAAAT-GGAAATTTTATCTTGTGAATTTTTGGCAATCTTATTTTCACTTTTGGTTTCAACCTTATAGGATCTATATAAAGCAATTACCCAACTATTCCTTCTCTTTTCTGGGA-TATTTTTCAAGTGTACTAAAAAATCCTTTGGTAGTAA-GAAATCAAATGCTAGATAATTCATTT-CTAATAAATACTCT-GACTAAGAAATTAGATACCGTAGCCCCAGTTATTTCTCTTATTGGATCATTGTCGAAAGCTCAATTTTGTACTGTATTGGGTCATCCTATTAGTAAACCGATCTGGACCGATTTATCGGATTCTGATATTCTTGATCGATTTTGTCGGATATGTAGAAATCTTTGTCGTTATCACAGCGGATCCTCAAAGAAACAGGTTTTGTATCGTATAAAGTATATACTTCGACTTTCGTGTGCTAGAACTTTGG-------------------------------------------------------------------------------------------------

Bletilla_striata_KC704597 ----------------------------------------------------------------------------------------------------------------------------------------------------------------------------------------------------------------------------------------------------------------------------AATCCTTCAATGCTGGATCAAAGATGTTTCTTCTTTGCATTTCTTGCGATTGTTTTTCCATGAATATCATAATTTGAATAGTCTCATTACTTC----------AAATAAATCCACTTACGTCTTTTCAAAAAGAAA---GAAAAGA-TTCTTTTGG-TTCCTACATAATTCTTATGTATATGAATGCGAATATCTATTCCTGTTTCTTCGTAAAAAGTCTTCTTATTTACGATCAATATCTTCTGGAGTTTTTCTTGAGCGAACACATTTCTATGGAAAAATAGAATATCTTATAGT---CGTGTGTTGTAATTCTTTTCA---------GAGTATCCTATGGTTCCTCAA-AGATACTTTCATACATTATGTTCGATATCAAGGAAAAGCAATTCTGGCTTCAAAAGGAACTCTTATTCTGATGAAGAAAT-GGAAATTTTATCTTGTGAATTTTTGGCAATCTTATTTTCACTTTTGGTTTCAACCTTATAGGATCTATATAAAGCAATTACCCAACTATTCCTTCTCTTTTCTGGGA-TATTTTTCAAGTGTACTAAAAAATCCTTTGGTAGTAA-GAAATCAAATGCTAGATAATTCATTT-CTAATAAATACTCT-GACTAAGAAATTAGATACCGTAGCCCCAGTTATTTCTCTTATTGGATCATTGTCGAAAGCTCAATTTTGTACTGTATTGGGTCATCCTATTAGTAAACCGATCTGGACCGATTTATCGGATTCTGATATTCTTGATCGATTTTGTCGGATATGTAGAAATCTTTGTCGTTATCACAGCGGATCCTCAAAGAAACAGGTTTTGTATCGTATAAAGTATATACTTCGACTTTCGTGTGCTAGAACTTTGG-------------------------------------------------------------------------------------------------

Bletilla_striata_KF262041 -----------------------------------------------------------------------------------------------------------------------------------------------------------------------------------------------------------------------------------------------------------------------------------------------------GTTCCTTCTTTGCATTTCTTGCGATTGTTTTTCCATGAATATCATAATTTGAATAGTCTCATTACTTC----------AAATAAATCCACTTACGTCTTTTCAAAAAGAAA---GAAAAGA-TTCTTTTGG-TTCCTACATAATTCTTATGTATATGAATGCGAATATCTATTCCTGTTTCTTCGTAAAAAGTCTTCTTATTTACGATCAATATCTTCTGGAGTTTTTCTTGAGCGAACACATTTCTATGGAAAAATAGAATATCTTATAGT---CGTGTGTTGTAATTCTTTTCA---------GAGTATCCTATGGTTCCTCAA-AGATACTTTCATACATTATGTTCGATATCAAGGAAAAGCAATTCTGGCTTCAAAAGGAACTCTTATTCTGATGAAGAAAT-GGAAATTTTATCTTGTGAATTTTTGGCAATCTTATTTTCACTTTTGGTTTCAACCTTATAGGATCTATATAAAGCAATTACCCAACTATTCCTTCTCTTTTCTGGGA-TATTTTTCAAGTGTACTAAAAAATCCTTTGGTAGTAA-GAAATCAAATGCTAGATAATTCATTT-CTAATAAATACTCT-GACTAAGAAATTAGATACCGTAGCCCCAGTTATTTCTCTTATTGGATCATTGTCGAAAGCTCAATTTTGTACTGTATTGGGTCATCCTATTAGTAAACCGATCTGGACCGATTTATCGGATTCTGATATTCTTGATCGATTTTGTCGGATATGTAGAAATCTTTGTCGTTATC----------------------------------------------------------------------------------------------------------------------------------------------------------------------------

Bletilla_striata_KF262042 -----------------------------------------------------------------------------------------------------------------------------------------------------------------------------------------------------------------------------------------------------------------------------------------------------GTTCCTTCTTTGCATTTCTTGCGATTGTTTTTCCATGAATATCATAATTTGAATAGTCTCATTACTTC----------AAATAAATCCACTTACGTCTTTTCAAAAAGAAA---GAAAAGA-TTCTTTTGG-TTCCTACATAATTCTTATGTATATGAATGCGAATATCTATTCCTGTTTCTTCGTAAAAAGTCTTCTTATTTACGATCAATATCTTCTGGAGTTTTTCTTGAGCGAACACATTTCTATGGAAAAATAGAATATCTTATAGT---CGTGTGTTGTAATTCTTTTCA---------GAGTATCCTATGGTTCCTCAA-AGATACTTTCATACATTATGTTCGATATCAAGGAAAAGCAATTCTGGCTTCAAAAGGAACTCTTATTCTGATGAAGAAAT-GGAAATTTTATCTTGTGAATTTTTGGCAATCTTATTTTCACTTTTGGTTTCAACCTTATAGGATCTATATAAAGCAATTACCCAACTATTCCTTCTCTTTTCTGGGA-TATTTTTCAAGTGTACTAAAAAATCCTTTGGTAGTAA-GAAATCAAATGCTAGATAATTCATTT-CTAATAAATACTCT-GACTAAGAAATTAGATACCGTAGCCCCAGTTATTTCTCTTATTGGATCATTGTCGAAAGCTCAATTTTGTACTGTATTGGGTCATCCTATTAGTAAACCGATCTGGACCGATTTATCGGATTCTGATATTCTTGATCGATTTTGTCGGATATGTAGAAATCTTTGTCGTTATC----------------------------------------------------------------------------------------------------------------------------------------------------------------------------

Bletilla_striata_KF361655 -----------------------------------------------------------------------------------------------------------------------------------------------------------------------------------------------------------------AATTATGTGTCAG-ATC-TACT-AATACCCCATCCCATACATCTGGAAATCTTGGTTCAAATCCTTCAATGCTGGATCAAAGATGTTTCTTCTTTGCATTTCTTGCGATTGTTTTTCCATGAATATCATAATTTGAATAGTCTCATTACTTC----------AAATAAATCCACTTACGTCTTTTCAAAAAGAAA---GAAAAGA-TTCTTTTGG-TTCCTACATAATTCTTATGTATATGAATGCGAATATCTATTCCTGTTTCTTCGTAAAAAGTCTTCTTATTTACGATCAATATCTTCTGGAGTTTTTCTTGAGCGAACACATTTCTATGGAAAAATAGAATATCTTATAGT---CGTGTGTTGTAATTCTTTTCA---------GAGTATCCTATGGTTCCTCAA-AGATACTTTCATACATTATGTTCGATATCAAGGAAAAGCAATTCTGGCTTCAAAAGGAACTCTTATTCTGATGAAGAAAT-GGAAATTTCATCTTGTGAATTTTTGGCAATCTTATTTTCACTTTTGGTTTCAACCTTATAGGATCTATATAAAGCAATTACCCAACTATTCCTTCTCTTTTCTGGGA-TATTTTTCAAGTGTACTAAAAAATCCTTTGGTAGTAA-GAAATCAAATGCTAGAGAATTCATTT-CTAATAAATACTCT-GACTAAGAAATTAGATACCGTAGCCCCAGTTATTTCTCTTATTGGATCATTGTCGAAAGCTCAATTTTGTACTGTATTGGGTCATCCTATTAGTAAACCGACCTGGACCAATTTATCGGATTCTGATATTCTTGATCGATTTTGTCGGATATGTAGAAATCTTTGTCGTTATCACAGCGGATCCTCAAAGAAACAGGTTTTGTAT--------------------------------------------------------------------------------------------------------------------------------------------

Bletilla_striata_KF673785 ACAAGAATTCTTTTTCTTCTCATTTTTCT------------TCTCAAATGGTATCAGAAGGTTTTGGAGTCATTCTGGAAATTCCATTCTCGTCGCGATTAGTATCT---------TCCCTTGAAG---AAAAAAGAATACCAAAATCTCAGAATTTACGATCTATTCATTCAATATTTCCCTTTTTAGAGGATAAATTATCACATTTAAATTATGTGTCAG-ATC-TACT-AATACCCCATCCCATACATCTGGAAATCTTGGTTCAAATCCTTCAATGCTGGATCAAAGATGTTTCTTCTTTGCATTTCTTGCGATTGTTTTTCCATGAATATCATAATTTGAATAGTCTCATTACTTC----------AAATAAATCCACTTACGTCTTTTCAAAAAGAAA---GAAAAGA-TTCTTTTGG-TTCCTACATAATTCTTATGTATATGAATGCGAATATCTATTCCTGTTTCTTCGTAAAAAGTCTTCTTATTTACGATCAATATCTTCTGGAGTTTTTCTTGAGCGAACACATTTCTATGGAAAAATAGAATATCTTATAGT---CGTGTGTTGTAATTCTTTTCA---------GAGTATCCTATGGTTCCTCAA-AGATACTTTCATACATTATGTTCGATATCAAGGAAAAGCAATTCTGGCTTCAAAAGGAACTCTTATTCTGATGAAGAAAT-GGAAATTTCATCTTGTGAATTTTTGGCAATCTTATTTTCACTTTTGGTTTCAACCTTATAGGATCTATATAAAGCAATTACCCAACTATTCCTTCTCTTTTCTGGGA-TATTTTTCAAGTGTACTAAAAAATCCTTTGGTAGTAA-GAAATCAAATGCTAGAGAATTCATTT-CTAATAAAGACTCT-GACTAAGAAATTAGATACCGTAGCCCCAGTTATTTCTCTTATTGGATCATTGTCGAAAGCTCAATTTTGTACTGTATTGGGTCATCCTATTAGTAAACCGACCTGGACCAATTTATCGGATTCTGATATTCTTGATCGATTTTGTCGGATATGTAGAAATCTTTGTCGTTATCACAGCGGATCCTCAAAGAAACAGGTTTTGTATCGTATAAAGTATATACTTCGACTTTCGTGTGCTAGAACTTTGGCTCGTAAACATAAAAGTACAGTACGCACTTTTATGCGAAGATTAGGTTCGGGATTCTTAG-AAGAATTTTTTTTGGAAGAAGAACAATCTCTTTCTT

Bletilla_striata_KF673786 ACAAGAATTCTTTTTCTTCTCATTTTTCT------------TCTCAAATGGTATCAGAAGGTTTTGGAGTCATTCTGGAAATTCCATTCTCGTCGCGATTAGTATCT---------TCCCTTGAAG---AAAAAAGAATACCAAAATCTCAGAATTTACGATCTATTCATTCAATATTTCCCTTTTTAGAGGATAAATTATCACATTTAAATTATGTGTCAG-ATC-TACT-AATACCCCATCCCATACATCTGGAAATCTTGGTTCAAATCCTTCAATGCTGGATCAAAGATGTTTCTTCTTTGCATTTCTTGCGATTGTTTTTCCATGAATATCATAATTTGAATAGTCTCATTACTTC----------AAATAAATCCACTTACGTCTTTTCAAAAAGAAA---GAAAAGA-TTCTTTTGG-TTCCTACATAATTCTTATGTATATGAATGCGAATATCTATTCCTGTTTCTTCGTAAAAAGTCTTCTTATTTACGATCAATATCTTCTGGAGTTTTTCTTGAGCGAACACATTTCTATGGAAAAATAGAATATCTTATAGT---CGTGTGTTGTAATTCTTTTCA---------GAGTATCCTATGGTTCCTCAA-AGATACTTTCATACATTATGTTCGATATCAAGGAAAAGCAATTCTGGCTTCAAAAGGAACTCTTATTCTGATGAAGAAAT-GGAAATTTTATCTTGTGAATTTTTGGCAATCTTATTTTCACTTTTGGTTTCAACCTTATAGGATCTATATAAAGCAATTACCCAACTATTCCTTCTCTTTTCTGGGA-TATTTTTCAAGTGTACTAAAAAATCCTTTGGTAGTAA-GAAATCAAATGCTAGATAATTCATTT-CTAATAAATACTCT-GACTAAGAAATTAGATACCGTAGCCCCAGTTATTTCTCTTATTGGATCATTGTCGAAAGCTCAATTTTGTACTGTATTGGGTCATCCTATTAGTAAACCGATCTGGACCGATTTATCGGATTCTGATATTCTTGATCGATTTTGTCGGATATGTAGAAATCTTTGTCGTTATCACAGCGGATCCTCAAAGAAACAGGTTTTGTATCGTATAAAGTATATACTTCGACTTTCGTGTGCTAGAACTTTGGCTCGTAAACATAAAAGTACAGTACGCACTTTTATGCGAAGATTAGGTTCGGGATTCTTAG-AAGAATTTTTTTTGGAAGAAGAACAATCTCTTTCTT

Bletilla_striata_KFBG2048 ACAAGAATTCTTTTTCTTCTCATTTTTCT------------TCTCAAATGGTATCAGAAGGTTTTGGAGTCATTCTGGAAATTCCATTCTCGTCGCGATTAGTATCT---------TCCCTTGAAG---AAAAAAGAATACCAAAATCTCAGAATTTACGATCTATTCATTCAATATTTCCCTTTTTAGAGGATAAATTATCACATTTAAATTATGTGTCAG-ATC-TACT-AATACCCCATCCCATACATCTGGAAATCTTGGTTCAAATCCTTCAATGCTGGATCAAAGATGTTTCTTCTTTGCATTTCTTGCGATTGTTTTTCCATGAATATCATAATTTGAATAGTCTCATTACTTC----------AAATAAATCCACTTACGTCTTTTCAAAAAGAAA---GAAAAGA-TTCTTTTGG-TTCCTACATAATTCTTATGTATATGAATGCGAATATCTATTCCTGTTTCTTCGTAAAAAGTCTTCTTATTTACGATCAATATCTTCTGGAGTTTTTCTTGAGCGAACACATTTCTATGGAAAAATAGAATATCTTATAGT---CGTGTGTTGTAATTCTTTTCA---------GAGTATCCTATGGTTCCTCAA-AGATACTTTCATACATTATGTTCGATATCAAGGAAAAGCAATTCTGGCTTCAAAAGGAACTCTTATTCTGATGAAGAAAT-GGAAATTTTATCTTGTGAATTTTTGGCAATCTTATTTTCACTTTTGGTTTCAACCTTATAGGATCTATATAAAGCAATTACCCAACTATTCCTTCTCTTTTCTGGGA-TATTTTTCAAGTGTACTAAAAAATCCTTTGGTAGTAA-GAAATCAAATGCTAGATAATTCATTT-CTAATAAATACTCT-GACTAAGAAATTAGATACCGTAGCCCCAGTTATTTCTCTTATTGGATCATTGTCGAAAGCTCAATTTTGTACTGTATTGGGTCATCCTATTAGTAAACCGATCTGGACCGATTTATCGGATTCTGATATTCTTGATCGATTTTGTCGGATATGTAGAAATCTTTGTCGTTATCACAGCGGATCCTCAAAGAAACAGGTTTTGTATCGTATAAAGTATATTCTTCGACTTTCGTGTGCTAGAACTTTGGCTCGTAAACATAAAAGTACAGTACGCACTTTTATGCGAAGATTAGGTTCGGGATTCTTAG-AAGAATATTTTC-GGAAGA-----------------

Bletilla_striata_KY966713 ACAAGAATTCTTTTTCTTCTCATTTTTCT------------TCTCAAATGGTATCAGAAGGTTTTGGAGTCATTCTGGAAATTCCATTCTCGTCGCGATTAGTATCT---------TCCCTTGAAG---AAAAAAGAATACCAAAATCTCAGAATTTACGATCTATTCATTCAATATTTCCCTTTTTAGAGGATAAATTATCACATTTAAATTATGTGTCAG-ATC-TACT-AATACCCCATCCCATACATCTGGAAATCTTGGTTCAAATCCTTCAATGCTGGATCAAAGATGTTTCTTCTTTGCATTTCTTGCGATTGTTTTTCCATGAATATCATAATTTGAATAGTCTCATTACTTC----------AAATAAATCCACTTACGTCTTTTCAAAAAGAAA---GAAAAGA-TTCTTTTGG-TTCCTACATAATTCTTATGTATATGAATGCGAATATCTATTCCTGTTTCTTCGTAAAAAGTCTTCTTATTTACGATCAATATCTTCTGGAGTTTTTCTTGAGCGAACACATTTCTATGGAAAAATAGAATATCTTATAGT---CGTGTGTTGTAATTCTTTTCA---------GAGTATCCTATGGTTCCTCAA-AGATACTTTCATACATTATGTTCGATATCAAGGAAAAGCAATTCTGGCTTCAAAAGGAACTCTTATTCTGATGAAGAAAT-GGAAATTTTATCTTGTGAATTTTTGGCAATCTTATTTTCACTTTTGGTTTCAACCTTATAGGATCTATATAAAGCAATTACCCAACTATTCCTTCTCTTTTCTGGGA-TATTTTTCAAGTGTACTAAAAAATCCTTTGGTAGTAA-GAAATCAAATGCTAGATAATTCATTT-CTAATAAATACTCT-GACTAAGAAATTAGATACCGTAGCCCCAGTTATTTCTCTTATTGGATCATTGTCGAAAGCTCAATTTTGTACTGTATTGGGTCATCCTATTAGTAAACCGATCTGGACCGATTTATCGGATTCTGATATTCTTGATCGATTTTGTCGGATATGTAGAAATCTTTGTCGTTATCACAGCGGATCCTCAAAGAAACAGGTTTTGTATCGTATAAAGTATATACTTCGACTTTCGTGTGCTAGAACTTTGGCTCGTAAACATAAAAGTACAGTACGCACTTTTATGCGAAGATTAGGTTCGGGATTCTTAG-AAGAATTTTT--------------------------

Bletilla_striata_MG490283 ACAAGAATTCTTTTTCTTCTCATTTTTCT------------TCTCAAATGGTATCAGAAGGTTTTGGAGTCATTCTGGAAATTCCATTCTCGTCGCGATTAGTATCT---------TCCCTTGAAG---AAAAAAGAATACCAAAATCTCAGAATTTACGATCTATTCATTCAATATTTCCCTTTTTAGAGGATAAATTATCACATTTAAATTATGTGTCAG-ATC-TACT-AATACCCCATCCCATACATCTGGAAATCTTGGTTCAAATCCTTCAATGCTGGATCAAAGATGTTTCTTCTTTGCATTTCTTGCGATTGTTTTTCCATGAATATCATAATTTGAATAGTCTCATTACTTC----------AAATAAATCCACTTACGTCTTTTCAAAAAGAAA---GAAAAGA-TTCTTTTGG-TTCCTACATAATTCTTATGTATATGAATGCGAATATCTATTCCTGTTTCTTCGTAAAAAGTCTTCTTATTTACGATCAATATCTTCTGGAGTTTTTCTTGAGCGAACACATTTCTATGGAAAAATAGAATATCTTATAGT---CGTGTGTTGTAATTCTTTTCA---------GAGTATCCTATGGTTCCTCAA-AGATACTTTCATACATTATGTTCGATATCAAGGAAAAGCAATTCTGGCTTCAAAAGGAACTCTTATTCTGATGAAGAAAT-GGAAATTTCATCTTGTGAATTTTTGGCAATCTTATTTTCACTTTTGGTTTCAACCTTATAGGATCTATATAAAGCAATTACCCAACTATTCCTTCTCTTTTCTGGGA-TATTTTTCAAGTGTACTAAAAAATCCTTTGGTAGTAA-GAAATCAAATGCTAGAGAATTCATTT-CTAATAAATACTCT-GACTAAGAAATTAGATACCGTAGCCCCAGTTATTTCTCTTATTGGATCATTGTCGAAAGCTCAATTTTGTACTGTATTGGGTCATCCTATTAGTAAACCGACCTGGACCAATTTATCGGATTCTGATATTCTTGATCGATTTTGTCGGATATGTAGAAATCTTTGTCGTTATCACAGCGGATCCTCAAAGAA---------------------------------------------------------------------------------------------------------------------------------------------------------

Brachycorythis_galeandra_PK12195 ATAAGAATTATTTTTCT------------------------TCTCAAATATTATCAGAAGGTTTTGGAGTTATTCTGGAAATTCCATTCTCGTCGCGATTAGTATCT---------TCCCTTGAAG---AAAAAAAAATACCAAAATCTCAGAATTTACGATCTATTCATTCAATCTTTCCTTTTTTAGAAGATAAATTCTCACATTTAAATTTTGTGTCAA-ATC-TACT-AATACCCCATCCCATCCATATGGAAATCTTAGTTCAAATCCTTCAATGCTGGATCAAAGATGTTCCTTCTTTGCATTTGTTGCGATTGATTTTCCATGAATATCATAATTTAAATAGTCTCATTACTTC----------AAAGAAAGAGATTGACGTCTTTTCAAAAATAAA---TAAAAGA-TTTTTTTGG-TTCTTACATAATTCTTATGTATATGAATGCGAATATATATTCCTGTTTCTTCGCAAACAGTCTTCTTATTTACGATCAACATCTTTTGAAGTCTTTCTTGAGCGAACACATTTCTATGAAAAAATAGAATATTTTAGAGT---AATGTATTGTAATTCTTTTCA---------GAGGATTCTATGGTTCCTCAA-AGAACCTTTCATACATTATGTTCGATATCAAGGAAAAGCAATTCTGGCTTCAAAGGGAACTCTAATTCTGATGAACAAAT-GGAAATTTCATCTTGTTCATTTTTGGCAATTTTATTTTCATTTTTGGTCTCAACCTTCTAGGATCCATATAAAGGAATTACCCAATTATTCCTTCTCTTTTCTGGGG-TATTTTTTAAGTGTACTAAAAAAGACTTTGGTAATAA-GAAATCAAATGCTGGAGAATTCATTT-TTAATAAATACTCT-GACTAAGAAATTAGATACCATAGCCCCAGTTATTTCTCTTATTGGAGCATTGTCAAAAGCTCAATTTTGTACTGTATTGGGCCATCCTATTAGTAAACCAATCTGGACCGATTTATCGGATTCTGATATTCTTGATCGATTTTGTCGGATATGTAGAAATATTTGTCGTTATCACAGCGGATCCTCAAAAAAACAGGTTTTATATCGTATAAAGTATATACTTCGACTTTCGTGTGCTAAAACTTTGGCTCGTAAACATAAAAGTACAGTACGCACTTTTATACGAAGATTAGGTTCGGGATTCTTAG-AAGAATTTTTTATGGAA-------------------

Brachycorythis_galeandra_SG1261 ATAAGAATTATTTTTCT------------------------TCTCAAATATTATCAGAAGGTTTTGGAGTTATTCTGGAAATTCCATTCTCGTCGCGATTAGTATCT---------TCCCTTGAAG---AAAAAAAAATACCAAAATCTCAGAATTTACGATCTATTCATTCAATCTTTCCTTTTTTAGAAGATAAATTCTCACATTTAAATTTTGTGTCAA-ATC-TACT-AATACCCCATCCCATCCATATGGAAATCTTAGTTCAAATCCTTCAATGCTGGATCAAAGATGTTCCTTCTTTGCATTTGTTGCGATTGATTTTCCATGAATATCATAATTTAAATAGTCTCATTACTTC----------AAAGAAAGAGATTGACGTCTTTTCAAAAATAAA---TAAAAGA-TTTTTTTGG-TTCTTACATAATTCTTATGTATATGAATGCGAATATATATTCCTGTTTCTTCGCAAACAGTCTTCTTATTTACGATCAACATCTTTTGAAGTCTTTCTTGAGCGAACACATTTCTATGAAAAAATAGAATATTTTAGAGT---AATGTATTGTAATTCTTTTCA---------GAGGATTCTATGGTTCCTCAA-AGAACCTTTCATACATTATGTTCGATATCAAGGAAAAGCAATTCTGGCTTCAAAGGGAACTCTAATTCTGATGAACAAAT-GGAAATTTCATCTTGTTCATTTTTGGCAATTTTATTTTCATTTTTGGTCTCAACCTTCTAGGATCCATATAAAGGAATTACCCAATTATTCCTTCTCTTTTCTGGGG-TATTTTTTAAGTGTACTAAAAAAGACTTTGGTAATAA-GAAATCAAATGCTGGAGAATTCATTT-TTAATAAATACTCT-GACTAAGAAATTAGATACCATAGCCCCAGTTATTTCTCTTATTGGAGCATTGTCAAAAGCTCAATTTTGTACTGTATTGGGCCATCCTATTAGTAAACCAATCTGGACCGATTTATCGGATTCTGATATTCTTGATCGATTTTGTCGGATATGTAGAAATATTTGTCGTTATCACAGCGGATCCTCAAAAAAACAGGTTTTATATCGTATAAAGTATATACTTCGACTTTCGTGTGCTAAAACTTTGGCTCGTAAACATAAAAGTACAGTACGCACTTTTATACGAAGATTAGGTTCGGGATTCTTAG-AAGAATTTTTTATGGAAGAAGAACAAGCTCTTTATT

Bulbophyllum_affine_KF361658 -----------------------------------------------------------------------------------------------------------------------------------------------------------------------------------------------------------------AATTATGTGTTAG-ATC-TACT-AATACCCCATCCCATCCATCTGGAAATCTTGGTTCAAATTCTTCAATGTTGGATCAAAGATGTTCCTTCTTTGCATTTATTGCGATTGTTTTTCCACGAATATCATAATTTGAATAGTCTCATTACTTC----------AAATAAATCCATTTACGTCTTTTCAAAAAGAAA---GAAAAGA-TTCTTTTGG-TTCCTACATAATTCTTATGTATATGAATGCGAATATCTATTCCTGTTTCTTCGTAAACAGTCTTCTTATTTACGATCAATATCTTCTGGAGTCTTTCTTGAGCGAACACATTTCTATGGAAAAATAGAATATCTTATAGT---CGTGTGTTGTAATTCTTTTCA---------GAGGAGCCTATGGTTCCTCAA-AGATACTTTCATACATTATGTTCGATATCAAGGAAAAGCAATTCTGTCTTCAAAAGGAACTCTTATTCTGATAAAGAAAT-GGAAATTTCATCTTGTTAATTTTTGGCAATCTTATTTTCACTTTTGGTTTCAACCTTATAGGATCCATATAAAGCAATTACCTAAATATTCCTTCTCTTTTCTGGGG-TTTTTTTCAAGTGTACTGAAAAATCCTTTGGTAGTAA-GAAATCAAATGCTAGAGTATTCATTT-CTAATAAATACTCT-GACTAATAAATTAGATACCATAGCCCCAGTAAATTCTCTTATTGGATCATTGTCGAATGCTCAATTTTGTACTGTATTGGGTCATCCTATTAGTAAACCGATCTGGACCGATTTATCGGATTCTTATATTATTGATCGATTTTGTCGGATATGTAGAAATCTTTGTCGTTATTACAGCGGATCCTCAAAGAAACAGGTTTTGTAT--------------------------------------------------------------------------------------------------------------------------------------------

Bulbophyllum_affine_KF974494 ------------------------------------------------------------------------------------------------------------------------------------------------------------------------------------------------------------------------------------------------------------------------------TTCTTCAATGTTGGATCAAAGATGTTCCTTCTTTGCATTTATTGCGATTGTTTTTCCACGAATATCATAATTTGAATAGTCTCATTACTTC----------AAATAAATCCATTTACGTCTTTTCAAAAAGAAA---GAAAAGA-TTCTTTTGG-TTCCTACATAATTCTTATGTATATGAATGCGAATATCTATTCCTGTTTCTTCGTAAACAGTCTTCTTATTTACGATCAATATCTTCTGGAGTCTTTCTTGAGCGAACACATTTCTATGGAAAAATAGAATATCTTATAGT---CGTGTGTTGTAATTCTTTTCA---------GAGGAGCCTATGGTTCCTCAA-AGATACTTTCATACATTATGTTCGATATCAAGGAAAAGCAATTCTGTCTTCAAAAGGAACTCTTATTCTGATAAAGAAAT-GGAAATTTCATCTTGTTAATTTTTGGCAATCTTATTTTCACTTTTGGTTTCAACCTTATAGGATCCATATAAAGCAATTACCTAAATATTCCTTCTCTTTTCTGGGG-TTTTTTTCAAGTGTACTGAAAAATCCTTTGGTAGTAA-GAAATCAAATGCTAGAGTATTCATTT-CTAATAAATACTCT-GACTAATAAATTAGATACCATAGCCCCAGTAAATTCTCTTATTGGATCATTGTCGAATGCTCAATTTTGTACTGTATTGGGTCATCCTATTAGTAAACCGATCTGGACCGATTTATCGGATTCTTATATTATTGATCGATTTTGTCGGATATGTAGAAATCTTTGTCGTTATTACAGCGGATCCTCAAAGAAACAGGTTTTGTATCGTATAAAGTATATACTTCGACTTTCTTGTGCTA--------------GAACAAAA------------------------------------------------------------------------------------

Bulbophyllum_affine_KFBG412 ACAAGAATTCTTTTTCTTCTCATTTTTCT------------TCTCAAATGCTATCAGAAGGTTTTGGAGTCATTCTGGAAATTCCATTCTCGTTGCGATTAGTATCT---------TCCCTTGAAG---ATAAAAGAATACCAAAATATCAGAATTTACGATCTATTCATTCAATATTTCCATTTTTAGAGGATAAATTATCGCATTTAAATTATGTGTTAG-ATC-TACT-AATACCCCATCCCATCCATCTGGAAATCTTGGTTCAAATTCTTCAATGTTGGATCAAAGATGTTCCTTCTTTGCATTTATTGCGATTGTTTTTCCACGAATATCATAATTTGAATAGTCTCATTACTTC----------AAATAAATCCATTTACGTCTTTTCAAAAAGAAA---GAAAAGA-TTCTTTTGG-TTCCTACATAATTCTTATGTATATGAATGCGAATATCTATTCCTGTTTCTTCGTAAACAGTCTTCTTATTTACGATCAATATCTTCTGGAGTCTTTCTTGAGCGAACACATTTCTATGGAAAAATAGAATATCTTATAGT---CGTGTGTTGTAATTCTTTTCA---------GAGGAGCCTATGGTTCCTCAA-AGATACTTTCATACATTATGTTCGATATCAAGGAAAAGCAATTCTGTCTTCAAAAGGAACTCTTATTCTGATAAAGAAAT-GGAAATTTCATCTTGTTAATTTTTGGCAATCTTATTTTCACTTTTGGTTTCAACCTTATAGGATCCATATAAAGCAATTACCTAAATATTCCTTCTCTTTTCTGGGG-TTTTTTTCAAGTGTACTGAAAAATCCTTTGGTAGTAA-GAAATCAAATGCTAGAGTATTCATTT-CTAATAAATACTCT-GACTAATAAATTAGATACCATAGCCCCAGTAAATTCTCTTATTGGATCATTGTCGAATGCTCAATTTTGTACTGTATTGGGTCATCCTATTAGTAAACCGATCTGGACCGATTTATCGGATTCTTATATTATTGATCGATTTTGTCGGATATGTAGAAATCTTTGTCGTTATTACAGCGGATCCTCAAAGAAACAGGTTTTGTATCGTATAAAGTATATACTTCGACTTTCGTGTGCTAGAACTTTGGCTCGTAAACATAAAAGTACAGTACGCACTTTTATGCGAAGGTTAGGTTCGGGATTCTTAG-AAGAATTTTTGTTGGAAGAAGAACAATCTCTTTCTT

Bulbophyllum_affine_KJ462086 -------------------------------------------------------------------------------------------------------------------------------------------------------TAATTTACGATCAATTCATTCAATATTTCCCTTTTTAGAGG--AAATTATCGCATTTAAATTATGTGTTAG-ATC-TACT-AATACCCCATCCCATCCATCTGGAAATCTTGGTTCAAATTCTTCAATGTTGGATCAAAGATGTTCCTTCTTTGCATTTATTGCGATTGTTTTTCCACGAATATCATAATTTGAATAGTCTCATTACTTC----------AAATAAATCCATTTACGTCTTTTCAAAAAGAAA---GAAAAGA-TTCTTTTGG-TTCCTACATAATTCTTATGTATATGAATGCGAATATCTATTCCTGTTTCTTCGTAAACAGTCTTCTTATTTACGATCAATATCTTCTGGAGTCTTTCTTGAGCGAACACATTTCTATGGAAAAATAGAATATCTTATAGT---CGTGTGTTGTAATTCTTTTCA---------GAGGAGCCTATGGTTCCTCAA-AGATACTTTCATACATTATGTTCGATATCAAGGAAAAGCAATTCTGTCTTCAAAAGGAACTCTTATTCTGATAAAGAAAT-GGAAATTTCATCTTGTTAATTTTTGGCAATCTTATTTTCACTTTTGGTTTCAACCTTATAGGATCCATATAAAGCAATTACCTAAATATTCCTTCTCTTTTCTGGGG-TTTTTTTCAAGTGTACTGAAAAATCCTTTGGTAGTAA-GAAATCAAATGCTAGAGTATTCATTT-CTAATAAATACTCT-GACTAATAAATTAGATACCATAGCCCCAGTAAATTCTCTTATTGGATCATTGTCGAATGCTCAATTTTGTACTGTATTGGGTCATCCTATTAGTAAACCGATCTGGACCGATTTATCGGATTCTTATATTATTGATCGATTTTGTCGGATATGTAGAAATCTTTGTCGTTATTACAGCGGATCCTCAAAGAAACAGGTTTTGTATCGTATAAAGTATATACTTCGACTTTCTTGTGCTAGAAC------------------------------------------------------------------------------------------------------

Bulbophyllum_affine_KX455831 ACAAGAATTCTTTTTCTTCTCATTTTTCT------------TCTCAAATGCTATCAGAAGGTTTTGGAGTCATTCTGGAAATTCCATTCTCGTTGCGATTAGTATCT---------TCCCTTGAAG---ATAAAAGAATACCAAAATATCAGAATTTACGATCTATTCATTCAATATTTCCATTTTTAGAGGATAAATTATCGCATTTAAATTATGTGTTAG-ATC-TACT-AATACCCCATCCCATCCATCTGGAAATCTTGGTTCAAATTCTTCAATGTTGGATCAAAGATGTTCCTTCTTTGCATTTATTGCGATTGTTTTTCCACGAATATCATAATTTGAATAGTCTCATTACTTC----------AAATAAATCCATTTACGTCTTTTCAAAAAGAAA---GAAAAGA-TTCTTTTGG-TTCCTACATAATTCTTATGTATATGAATGCGAATATCTATTCCTGTTTCTTCGTAAACAGTCTTCTTATTTACGATCAATATCTTCTGGAGTCTTTCTTGAGCGAACACATTTCTATGGAAAAATAGAATATCTTATAGT---CGTGTGTTGTAATTCTTTTCA---------GAGGAGCCTATGGTTCCTCAA-AGATACTTTCATACATTATGTTCGATATCAAGGAAAAGCAATTCTGTCTTCAAAAGGAACTCTTATTCTGATAAAGAAAT-GGAAATTTCATCTTGTTAATTTTTGGCAATCTTATTTTCACTTTTGGTTTCAACCTTATAGGATCCATATAAAGCAATTACCTAAATATTCCTTCTCTTTTCTGGGG-TTTTTTTCAAGTGTACTGAAAAATCCTTTGGTAGTAA-GAAATCAAATGCTAGAGTATTCATTT-CTAATAAATACTCT-GACTAATAAATTAGATACCATAGCCCCAGTAAATTCTCTTATTGGATCATTGTCGAATGCTCAATTTTGTACTGTATTGGGTCATCCTATTAGTAAACCGATCTGGACCGATTTATCGGATTCTTATATTATTGATCGATTTTGTCGGATATGTAGAAATCTTTGTCGTTATTACAGCGGATCCTCAAAGAAACAGGTTTTGTATCGTATAAAGTATATACTTCGACTTTCGTGTGCTAGAACTTTGGCTCGTAAACATAAAAGTACAGTACGCACTTTTATGCGAAGGTTAGGTTCGGGATTCTTAG-AAGAATTTTTGTTGGAAGAAGAACAATCTCTTTCTT

Bulbophyllum_affine_KY966714 ACAAGAATTCTTTTTCTTCTCATTTTTCT------------TCTCAAATGCTATCAGAAGGTTTTGGAGTCATTCTGGAAATTCCATTCTCGTTGCGATTAGTATCT---------TCCCTTGAAG---ATAAAAGAATACCAAAATATCAGAATTTACGATCTATTCATTCAATATTTCCCTTTTTAGAGGATAAATTATCGCATTTAAATTATGTGTTAG-ATC-TACT-AATACCCCATCCCATCCATCTGGAAATCTTGGTTCAAATTCTTCAATGTTGGATCAAAGATGTTCCTTCTTTGCATTTATTGCGATTGTTTTTCCACGAATATCATAATTTGAATAGTCTCATTACTTC----------AAATAAATCCATTTACGTCTTTTCAAAAAGAAA---GAAAAGA-TTCTTTTGG-TTCCTACATAATTCTTATGTATATGAATGCGAATATCTATTCCTGTTTCTTCGTAAACAGTCTTCTTATTTACGATCAATATCTTCTGGAGTCTTTCTTGAGCGAACACATTTCTATGGAAAAATAGAATATCTTATAGT---CGTGTGTTGTAATTCTTTTCA---------GAGGAGCCTATGGTTCCTCAA-AGATACTTTCATACATTATGTTCGATATCAAGGAAAAGCAATTCTGTCTTCAAAAGGAACTCTTATTCTGATAAAGAAAT-GGAAATTTCATCTTGTTAATTTTTGGCAATCTTATTTTCACTTTTGGTTTCAACCTTATAGGATCCATATAAAGCAATTACCTAAATATTCCTTCTCTTTTCTGGGG-TTTTTTTCAAGTGTACTGAAAAATCCTTTGGTAGTAA-GAAATCAAATGCTAGAGTATTCATTT-CTAATAAATACTCT-GACTAATAAATTAGATACCATAGCCCCAGTAAATTCTCTTATTGGATCATTGTCGAATGCTCAATTTTGTACTGTATTGGGTCATCCTATTAGTAAACCGATCTGGACCGATTTATCGGATTCTTATATTATTGATCGATTTTGTCGGATATGTAGAAATCTTTGTCGTTATTACAGCGGATCCTCAAAGAAACAGGTTTTGTATCGTATAAAGTATATACTTCGACTTTCGTGTGCTAGAACTTTGGCTCGTAAACATAAAAGTACAGTACGCACTTTTATGCGAAGGTTAGGTTCGGGATTCTTAG-AAGAATTTT---------------------------

Bulbophyllum_affine_SG1606 -CAAGAATTCTTTTTCTTCTCATTTTTCT------------TCTCAAATGCTATCAGAAGGTTTTGGAGTCATTCTGGAAATTCCATTCTCGTTGCGATTAGTATCT---------TCCCTTGAAG---ATAAAAGAATACCAAAATATCAGAATTTACGATCTATTCATTCAATATTTCCCTTTTTAGAGGATAAATTATCGCATTTAAATTATGTGTTAG-ATC-TACT-AATACCCCATCCCATCCATCTGGAAATCTTGGTTCAAATTCTTCAATGTTGGATCAAAGATGTTCCTTCTTTGCATTTATTGCGATTGTTTTTCCACGAATATCATAATTTGAATAGTCTCATTACTTC----------AAATAAATCCATTTACGTCTTTTCAAAAAGAAA---GAAAAGA-TTCTTTTGG-TTCCTACATAATTCTTATGTATATGAATGCGAATATCTATTCCTGTTTCTTCGTAAACAGTCTTCTTATTTACGATCAATATCTTCTGGAGTCTTTCTTGAGCGAACACATTTCTATGGAAAAATAGAATATCTTATAGT---CGTGTGTTGTAATTCTTTTCA---------GAGGAGCCTATGGTTCCTCAA-AGATACTTTCATACATTATGTTCGATATCAAGGAAAAGCAATTCTGTCTTCAAAAGGAACTCTTATTCTGATAAAGAAAT-GGAAATTTCATCTTGTTAATTTTTGGCAATCTTATTTTCACTTTTGGTTTCAACCTTATAGGATCCATATAAAGCAATTACCTAAATATTCCTTCTCTTTTCTGGGG-TTTTTTTCAAGTGTACTGAAAAATCCTTTGGTAGTAA-GAAATCAAATGCTAGAGTATTCATTT-CTAATAAATACTCT-GACTAATAAATTAGATACCATAGCCCCAGTAAATTCTCTTATTGGATCATTGTCGAATGCTCAATTTTGTACTGTATTGGGTCATCCTATTAGTAAACCGATCTGGACCGATTTATCGGATTCTTATATTATTGATCGATTTTGTCGGATATGTAGAAATCTTTGTCGTTATTACAGCGGATCCTCAAAGAAACAGGTTTTGTATCGTATAAAGTATATACTTCGACTTTCGTGTGCTAGAACTTTGGCTCGTAAACATAAAAGTACAGTACGCACTTTTATGCGAAGGTTAGGTTCGGGATTCTTAG-AAGAATTTTTGTTGGAAGAAGAACAATCTCTTTCTT

Bulbophyllum_affine_SG1607 ACAAGAATTCTTTTTCTTCTCATTTTTCT------------TCTCAAATGCTATCAGAAGGTTTTGGAGTCATTCTGGAAATTCCATTCTCGTTGCGATTAGTATCT---------TCCCTTGAAG---ATAAAAGAATACCAAAATATCAGAATTTACGATCTATTCATTCAATATTTCCCTTTTTAGAGGATAAATTATCGCATTTAAATTATGTGTTAG-ATC-TACT-AATACCCCATCCCATCCATCTGGAAATCTTGGTTCAAATTCTTCAATGTTGGATCAAAGATGTTCCTTCTTTGCATTTATTGCGATTGTTTTTCCACGAATATCATAATTTGAATAGTCTCATTACTTC----------AAATAAATCCATTTACGTCTTTTCAAAAAGAAA---GAAAAGA-TTCTTTTGG-TTCCTACATAATTCTTATGTATATGAATGCGAATATCTATTCCTGTTTCTTCGTAAACAGTCTTCTTATTTACGATCAATATCTTCTGGAGTCTTTCTTGAGCGAACACATTTCTATGGAAAAATAGAATATCTTATAGT---CGTGTGTTGTAATTCTTTTCA---------GAGGAGCCTATGGTTCCTCAA-AGATACTTTCATACATTATGTTCGATATCAAGGAAAAGCAATTCTGTCTTCAAAAGGAACTCTTATTCTGATAAAGAAAT-GGAAATTTCATCTTGTTAATTTTTGGCAATCTTATTTTCACTTTTGGTTTCAACCTTATAGGATCCATATAAAGCAATTACCTAAATATTCCTTCTCTTTTCTGGGG-TTTTTTTCAAGTGTACTGAAAAATCCTTTGGTAGTAA-GAAATCAAATGCTAGAGTATTCATTT-CTAATAAATACTCT-GACTAATAAATTAGATACCATAGCCCCAGTAAATTCTCTTATTGGATCATTGTCGAATGCTCAATTTTGTACTGTATTGGGTCATCCTATTAGTAAACCGATCTGGACCGATTTATCGGATTCTTATATTATTGATCGATTTTGTCGGATATGTAGAAATCTTTGTCGTTATTACAGCGGATCCTCAAAGAAACAGGTTTTGTATCGTATAAAGTATATACTTCGACTTTCGTGTGCTAGAACTTTGGCTCGTAAACATAAAAGTACAGTACGCACTTTTATGCGAAGGTTAGGTTCGGGATTCTTAG-AAGAATTTTTGTTGGAAGAAGAACAATCTCTTTCTT

Bulbophyllum_ambrosia_KF361657 -----------------------------------------------------------------------------------------------------------------------------------------------------------------------------------------------------------------AATTATGTGTTAG-ATC-TACT-AATACCCCATCCCATCCATCTGGAAATCTTGGTTCAAATTCTTCAATGTTGGATCAAAGATGTTCCTTCTTTGCATTTATTGCGATTGTTTTTCCACGAATATCATAATTTGAATAATCTCATTACTTC----------AAAGAAATCCATTTACGTCTTTTCAAAAAGAAA---TAAAAGA-TTCTTTTGG-TTCCTACATAATTCTTATGTATATGAATGCGAATATCTATTCCTGTTTCTTCGTAAACAGTCTTCTTATTTACGATCAATATCTTCTGGAGTCTTTCTTGAGCGAACACATTTCTATGGAAAAATAGAATATCTTATAGT---CGTGTGTTGTAATTCTTTTCA---------GAGGAGCCTATGGTTCCTCAA-AGATATTTTCATACATTATGTTCGATATCAAGGAAAAGCAATTCTGTCTTCAAAAGGAACTCTTATTCTGATAAAGAAAT-GGAAATTTCATCTTGTGAATTTTTGGCAATCTTATTTTCACTTTTGGTTTCAACCTTATAGGATCCATATAAAGCAATTACCCAACTATTCCTTCTCGTTTCTGGGG-TTTTTTTCAAGTGTACTGAAAAATCCTTTGGTAGTAA-GAAATCAAATGCTAGAGTATTCATTT-CTAATAAATATTCT-GACTAATAAATTAGATACCATAGCCCCAGTAAATTCTCTTATTGGATCATTGTCGAAAGCTCAATTTTGTACTGTATTGGGTCATCCTATTAGTAAACCAATCTGGACCGATTTATCGGATTCTTATATTATTGATCGATTTTGTCGAATATGTAGAAATCTTTGTCGTTATTACAGCGGATCCTCAAAGAAACAGGTTTTGTAT--------------------------------------------------------------------------------------------------------------------------------------------

Bulbophyllum_ambrosia_KY966715 ACAAGAATTCTTTTTCTTCTCATTTTTCT------------TCTCAAATGCTATCAGAAGGTTTTGGAGTCATTCTGGAAATTCCATTCTCGTTGCGATTAGTATCT---------TCCCTTGAAG---ATAAAAGAATACCAAAATCTCATAATTTACGATCTATTCATTCAATATTTCCCTTTTTAGAGGATAAATTATCGCATTTAAATTATGTGTTAG-ATC-TACT-AATACCCCATCCCATCCATCTGGAAATCTTGGTTCAAATTCTTCAATGTTGGATCAAAGATGTTCCTTCTTTGCATTTATTGCGATTGTTTTTCCACGAATATCATAATTTGAATAATCTCATTACTTC----------AAAGAAATCCATTTACGTCTTTTCAAAAAGAAA---TAAAAGA-TTCTTTTGG-TTCCTACATAATTCTTATGTATATGAATGCGAATATCTATTCCTGTTTCTTCGTAAACAGTCTTCTTATTTACGATCAATATCTTCTGGAGTCTTTCTTGAGCGAACACATTTCTATGGAAAAATAGAATATCTTATAGT---CGTGTGTTGTAATTCTTTTCA---------GAGGAGCCTATGGTTCCTCAA-AGATATTTTCATACATTATGTTCGATATCAAGGAAAAGCAATTCTGTCTTCAAAAGGAACTCTTATTCTGATAAAGAAAT-GGAAATTTCATCTTGTGAATTTTTGGCAATCTTATTTTCACTTTTGGTTTCAACCTTATAGGATCCATATAAAGCAATTACCCAACTATTCCTTCTCGTTTCTGGGG-TTTTTTTCAAGTGTACTGAAAAATCCTTTGGTAGTAA-GAAATCAAATGCTAGAGTATTCATTT-CTAATAAATATTCT-GACTAATAAATTAGATACCATAGCCCCAGTAAATTCTCTTATTGGATCATTGTCGAAAGCTCAATTTTGTACTGTATTGGGTCATCCTATTAGTAAACCGATCTGGACCGATTTATCGGATTCTTATATTATTGATCGATTTTGTCGAATATGTAGAAATCTTTGTCGTTATTACAGCGGATCCTCAAAGAAACAGGTTTTGTATCGTATAAAGTATATACTTCGACTTTCGTGTGCTAGAACTTTGGCTCGTAAACATAAAAGTACAGTACGCACTTTTATGCGAAGGTTAGGTTCGGGATTCTTAG-AAGAATTTTT--------------------------

Bulbophyllum_ambrosia_KY966716 ACAAGAATTCTTTTTCTTCTCATTTTTCT------------TCTCAAATGCTATCAGAAGGTTTTGGAGTCATTCTGGAAATTCCATTCTCGTTGCGATTAGTATCT---------TCCCTTGAAG---ATAAAAGAATACCAAAATCTCATAATTTACGATCTATTCATTCAATATTTCCCTTTTTAGAGGATAAATTATCGCATTTAAATTATGTGTTAG-ATC-TACT-AATACCCCATCCCATCCATCTGGAAATCTTGGTTCAAATTCTTCAATGTTGGATCAAAGATGTTCCTTCTTTGCATTTATTGCGATTGTTTTTCCACGAATATCATAATTTGAATAATCTCATTACTTC----------AAAGAAATCCATTTACGTCTTTTCAAAAAGAAA---TAAAAGA-TTCTTTTGG-TTCCTACATAATTCTTATGTATATGAATGCGAATATCTATTCCTGTTTCTTCGTAAACAGTCTTCTTATTTACGATCAATATCTTCTGGAGTCTTTCTTGAGCGAACACATTTCTATGGAAAAATAGAATATCTTATAGT---CGTGTGTTGTAATTCTTTTCA---------GAGGAGCCTATGGTTCCTCAA-AGATATTTTCATACATTATGTTCGATATCAAGGAAAAGCAATTCTGTCTTCAAAAGGAACTCTTATTCTGATAAAGAAAT-GGAAATTTCATCTTGTGAATTTTTGGCAATCTTATTTTCACTTTTGGTTTCAACCTTATAGGATCCATATAAAGCAATTACCCAACTATTCCTTCTCGTTTCTGGGG-TTTTTTTCAAGTGTACTGAAAAATCCTTTGGTAGTAA-GAAATCAAATGCTAGAGTATTCATTT-CTAATAAATATTCT-GACTAATAAATTAGATACCATAGCCCCAGTAAATTCTCTTATTGGATCATTGTCGAAAGCTCAATTTTGTACTGTATTGGGTCATCCTATTAGTAAACCGATCTGGACCGATTTATCGGATTCTTATATTATTGATCGATTTTGTCGAATATGTAGAAATCTTTGTCGTTATTACAGCGGATCCTCAAAGAAACAGGTTTTGTATCGTATAAAGTATATACTTCGACTTTCGTGTGCTAGAACTTTGGCTCGTAAACATAAAAGTACAGTACGCACTTTTATGCGAAGGTTAGGTTCGGGATTCTTAG-AAGAATTTTT--------------------------

Bulbophyllum_ambrosia_KY966717 ACAAGAATTCTTTTTCTTCTCATTTTTCT------------TCTCAAATGCTATCAGAAGGTTTTGGAGTCATTCTGGAAATTCCATTCTCGTTGCGATTAGTATCT---------TCCCTTGAAG---ATAAAAGAATACCAAAATCTCATAATTTACGATCTATTCATTCAATATTTCCCTTTTTAGAGGATAAATTATCGCATTTAAATTATGTGTTAG-ATC-TACT-AATACCCCATCCCATCCATCTGGAAATCTTGGTTCAAATTCTTCAATGTTGGATCAAAGATGTTCCTTCTTTGCATTTATTGCGATTGTTTTTCCACGAATATCATAATTTGAATAATCTCATTACTTC----------AAAGAAATCCATTTACGTCTTTTCAAAAAGAAA---TAAAAGA-TTCTTTTGG-TTCCTACATAATTCTTATGTATATGAATGCGAATATCTATTCCTGTTTCTTCGTAAACAGTCTTCTTATTTACGATCAATATCTTCTGGAGTCTTTCTTGAGCGAACACATTTCTATGGAAAAATAGAATATCTTATAGT---CGTGTGTTGTAATTCTTTTCA---------GAGGAGCCTATGGTTCCTCAA-AGATATTTTCATACATTATGTTCGATATCAAGGAAAAGCAATTCTGTCTTCAAAAGGAACTCTTATTCTGATAAAGAAAT-GGAAATTTCATCTTGTGAATTTTTGGCAATCTTATTTTCACTTTTGGTTTCAACCTTATAGGATCCATATAAAGCAATTACCCAACTATTCCTTCTCGTTTCTGGGG-TTTTTTTCAAGTGTACTGAAAAATCCTTTGGTAGTAA-GAAATCAAATGCTAGAGTATTCATTT-CTAATAAATATTCT-GACTAATAAATTAGATACCATAGCCCCAGTAAATTCTCTTATTGGATCATTGTCGAAAGCTCAATTTTGTACTGTATTGGGTCATCCTATTAGTAAACCGATCTGGACCGATTTATCGGATTCTTATATTATTGATCGATTTTGTCGAATATGTAGAAATCTTTGTCGTTATTACAGCGGATCCTCAAAGAAACAGGTTTTGTATCGTATAAAGTATATACTTCGACTTTCGTGTGCTAGAACTTTGGCTCGTAAACATAAAAGTACAGTACGCACTTTTATGCGAAGGTTAGGTTCGGGATTCTTAG-AAGAATTTTT--------------------------

Bulbophyllum_ambrosia_KY966718 ACAAGAATTCTTTTTCTTCTCATTTTTCT------------TCTCAAATGCTATCAGAAGGTTTTGGAGTCATTCTGGAAATTCCATTCTCGTTGCGATTAGTATCT---------TCCCTTGAAG---ATAAAAGAATACCAAAATCTCATAATTTACGATCTATTCATTCAATATTTCCCTTTTTAGAGGATAAATTATCGCATTTAAATTATGTGTTAG-ATC-TACT-AATACCCCATCCCATCCATCTGGAAATCTTGGTTCAAATTCTTCAATGTTGGATCAAAGATGTTCCTTCTTTGCATTTATTGCGATTGTTTTTCCACGAATATCATAATTTGAATAATCTCATTACTTC----------AAAGAAATCCATTTACGTCTTTTCAAAAAGAAA---TAAAAGA-TTCTTTTGG-TTCCTACATAATTCTTATGTATATGAATGCGAATATCTATTCCTGTTTCTTCGTAAACAGTCTTCTTATTTACGATCAATATCTTCTGGAGTCTTTCTTGAGCGAACACATTTCTATGGAAAAATAGAATATCTTATAGT---CGTGTGTTGTAATTCTTTTCA---------GAGGAGCCTATGGTTCCTCAA-AGATATTTTCATACATTATGTTCGATATCAAGGAAAAGCAATTCTGTCTTCAAAAGGAACTCTTATTCTGATAAAGAAAT-GGAAATTTCATCTTGTGAATTTTTGGCAATCTTATTTTCACTTTTGGTTTCAACCTTATAGGATCCATATAAAGCAATTACCCAACTATTCCTTCTCGTTTCTGGGG-TTTTTTTCAAGTGTACTGAAAAATCCTTTGGTAGTAA-GAAATCAAATGCTAGAGTATTCATTT-CTAATAAATATTCT-GACTAATAAATTAGATACCATAGCCCCAGTAAATTCTCTTATTGGATCATTGTCGAAAGCTCAATTTTGTACTGTATTGGGTCATCCTATTAGTAAACCGATCTGGACCGATTTATCGGATTCTTATATTATTGATCGATTTTGTCGAATATGTAGAAATCTTTGTCGTTATTACAGCGGATCCTCAAAGAAACAGGTTTTGTATCGTATAAAGTATATACTTCGACTTTCGTGTGCTAGAACTTTGGCTCGTAAACATAAAAGTACAGTACGCACTTTTATGCGAAGGTTAGGTTCGGGATTCTTAG-AAGAATTTTT--------------------------

Bulbophyllum_ambrosia_PK12090 ACAAGAATTCTTTTTCTTCTCATTTTTCT------------TCTCAAATGCTATCAGAAGGTTTTGGAGTCATTCTGGAAATTCCATTCTCGTTGCGATTAGTATCT---------TCCCTTGAAG---ATAAAAGAATACCAAAATCTCATAATTTACGATCTATTCATTCAATATTTCCCTTTTTAGAGGATAAATTATCGCATTTAAATTATGTGTTAG-ATC-TACT-AATACCCCATCCCATCCATCTGGAAATCTTGGTTCAAATTCTTCAATGTTGGATCAAAGATGTTCCTTCTTTGCATTTATTGCGATTGTTTTTCCACGAATATCATAATTTGAATAATCTCATTACTTC----------AAAGAAATCCATTTACGTCTTTTCAAAAAGAAA---TAAAAGA-TTCTTTTGG-TTCCTACATAATTCTTATGTATATGAATGCGAATATCTATTCCTGTTTCTTCGTAAACAGTCTTCTTATTTACGATCAATATCTTCTGGAGTCTTTCTTGAGCGAACACATTTCTATGGAAAAATAGAATATCTTATAGT---CGTGTGTTGTAATTCTTTTCA---------GAGGAGCCTATGGTTCCTCAA-AGATATTTTCATACATTATGTTCGATATCAAGGAAAAGCAATTCTGTCTTCAAAAGGAACTCTTATTCTGATAAAGAAAT-GGAAATTTCATCTTGTGAATTTTTGGCAATCTTATTTTCACTTTTGGTTTCAACCTTATAGGATCCATATAAAGCAATTACCCAACTATTCCTTCTCGTTTCTGGGG-TTTTTTTCAAGTGTACTGAAAAATCCTTTGGTAGTAA-GAAATCAAATGCTAGAGTATTCATTT-CTAATAAATATTCT-GACTAATAAATTAGATACCATAGCCCCAGTAAATTCTCTTATTGGATCATTGTCGAAAGCTCAATTTTGTACTGTATTGGGTCATCCTATTAGTAAACCGATCTGGACCGATTTATCGGATTCTTATATTATTGATCGATTTTGTCGAATATGTAGAAATCTTTGTCGTTATTACAGCGGATCCTCAAAGAAACAGGTTTTGTATCGTATAAAGTATATACTTCGACTTTCGTGTGCTAGAACTTTGGCTCGTAAACATAAAAGTACAGTACGCACTTTTATGCGAAGGTTAGGTTCGGGATTCTTAG-AAGAATTTTTTTTGGAAGAAGAACAATCTCTTTCTT

Bulbophyllum_ambrosia_PK12092 ACAAGAATTCTTTTTCTTCTCATTTTTCT------------TCTCAAATGCTATCAGAAGGTTTTGGAGTCATTCTGGAAATTCCATTCTCGTTGCGATTAGTATCT---------TCCCTTGAAG---ATAAAAGAATACCAAAATCTCATAATTTACGATCTATTCATTCAATATTTCCCTTTTTAGAGGATAAATTATCGCATTTAAATTATGTGTTAG-ATC-TACT-AATACCCCATCCCATCCATCTGGAAATCTTGGTTCAAATTCTTCAATGTTGGATCAAAGATGTTCCTTCTTTGCATTTATTGCGATTGTTTTTCCACGAATATCATAATTTGAATAATCTCATTACTTC----------AAAGAAATCCATTTACGTCTTTTCAAAAAGAAA---TAAAAGA-TTCTTTTGG-TTCCTACATAATTCTTATGTATATGAATGCGAATATCTATTCCTGTTTCTTCGTAAACAGTCTTCTTATTTACGATCAATATCTTCTGGAGTCTTTCTTGAGCGAACACATTTCTATGGAAAAATAGAATATCTTATAGT---CGTGTGTTGTAATTCTTTTCA---------GAGGAGCCTATGGTTCCTCAA-AGATATTTTCATACATTATGTTCGATATCAAGGAAAAGCAATTCTGTCTTCAAAAGGAACTCTTATTCTGATAAAGAAAT-GGAAATTTCATCTTGTGAATTTTTGGCAATCTTATTTTCACTTTTGGTTTCAACCTTATAGGATCCATATAAAGCAATTACCCAACTATTCCTTCTCGTTTCTGGGG-TTTTTTTCAAGTGTACTGAAAAATCCTTTGGTAGTAA-GAAATCAAATGCTAGAGTATTCATTT-CTAATAAATATTCT-GACTAATAAATTAGATACCATAGCCCCAGTAAATTCTCTTATTGGATCATTGTCGAAAGCTCAATTTTGTACTGTATTGGGTCATCCTATTAGTAAACCGATCTGGACCGATTTATCGGATTCTTATATTATTGATCGATTTTGTCGAATATGTAGAAATCTTTGTCGTTATTACAGCGGATCCTCAAAGAAACAGGTTTTGTATCGTATAAAGTATATACTTCGACTTTCGTGTGCTAGAACTTTGGCTCGTAAACATAAAAGTACAGTACGCACTTTTATGCGAAGGTTAGGTTCGGGATTCTTAG-AAGAATTTTTTTTGGAAGAAGAACAATCTCTTTCTT

Bulbophyllum_ambrosia_SG1221 ACAAGAATTCTTTTTCTTCTCATTTTTCT------------TCTCAAATGCTATCAGAAGGTTTTGGAGTCATTCTGGAAATTCCATTCTCGTTGCGATTAGTATCT---------TCCCTTGAAG---ATAAAAGAATACCAAAATCTCATAATTTACGATCTATTCATTCAATATTTCCCTTTTTAGAGGATAAATTATCGCATTTAAATTATGTGTTAG-ATC-TACT-AATACCCCATCCCATCCATCTGGAAATCTTGGTTCAAATTCTTCAATGTTGGATCAAAGATGTTCCTTCTTTGCATTTATTGCGATTGTTTTTCCACGAATATCATAATTTGAATAATCTCATTACTTC----------AAAGAAATCCATTTACGTCTTTTCAAAAAGAAA---TAAAAGA-TTCTTTTGG-TTCCTACATAATTCTTATGTATATGAATGCGAATATCTATTCCTGTTTCTTCGTAAACAGTCTTCTTATTTACGATCAATATCTTCTGGAGTCTTTCTTGAGCGAACACATTTCTATGGAAAAATAGAATATCTTATAGT---CGTGTGTTGTAATTCTTTTCA---------GAGGAGCCTATGGTTCCTCAA-AGATATTTTCATACATTATGTTCGATATCAAGGAAAAGCAATTCTGTCTTCAAAAGGAACTCTTATTCTGATAAAGAAAT-GGAAATTTCATCTTGTGAATTTTTGGCAATCTTATTTTCACTTTTGGTTTCAACCTTATAGGATCCATATAAAGCAATTACCCAACTATTCCTTCTCGTTTCTGGGG-TTTTTTTCAAGTGTACTGAAAAATCCTTTGGTAGTAA-GAAATCAAATGCTAGAGTATTCATTT-CTAATAAATATTCT-GACTAATAAATTAGATACCATAGCCCCAGTAAATTCTCTTATTGGATCATTGTCGAAAGCTCAATTTTGTACTGTATTGGGTCATCCTATTAGTAAACCGATCTGGACCGATTTATCGGATTCTTATATTATTGATCGATTTTGTCGAATATGTAGAAATCTTTGTCGTTATTACAGCGGATCCTCAAAGAAACAGGTTTTGTATCGTATAAAGTATATACTTCGACTTTCGTGTGCTAGAACTTTGGCTCGTAAACATAAAAGTACAGTACGCACTTTTATGCGAAGGTTAGGTTCGGGATTCTTAG-AAGAATTTTTTTTGGAAGAAGAACAATCTCTTTCTT

Bulbophyllum_bicolor_CL10 ACAAGAATTCTTTTTCTTCTCATTTTTCT------------TCTCAAATGCTATCAGAAGGTTTTGGAGTCATTCTGGAAATTCCATTCTCGTTGCGATTAGTATCT---------TCCCTTGAAG---ATAAAAGAATACCAAAATCTCAGAATTTACGATCTATTCATTCTATATTTCCCTTTTTAGAGGATAAATTATCGCATTTCAATTATGTGTTAG-ATC-TACT-AATACCCCATCCCATCCATCTGGAAATCTTGGTTCAAATTCTTCAATGTTGGATCAAAGATGTTCCTTCTTTGCATTTATTGCGATTGTTTTTCCACGAATATCATAATTTGAATAATCTCATTACTTC----------AAAGAAATCCATTTACGTCTTTTCAAAAAGAAA---GAAAAGA-TTTTTTTGG-TTCCTACATAATTCTTATGTATATGAATGCGAATATCTATTCCTGTTTCTTCGTAAACAGTCTTCTTATTTACGATCAATATCTTCTGGAGTCTTTCTTGAGCGAACACATTTCTATGGAAAATTAGAATATCTTATAGT---CGTGTGTTGTAATTCTTTTCA---------GAGGAGCCTATGGTTCCTCAA-AGATACTTTCATACATTATGTTCGATATCAAGGAAAAGCAATTCTGTCTTCAAAAGGAACTCTTATTCTGATAAAGAAAT-GGAAATTTCATCTTGTGAATTTTTGGCAATCTTATTTTCACTTTTGGTTTCAACCTTATAGGATCCATATAAAGCAATTACCCAACTATTCCTTCTCGTTTCTGGGG-TTTTTTTCAAGTGTACTGAAAAATCCTTTGGTAGTAA-GAAATCAAATGCTAGAGTATTCATTT-CTAATAAATACTCT-GACTAATAAATTAGATACCATAGCCCCAGTAAATTCTCTTATTGGATCATTGTCGAAAGCTCAATTTTGTACTGTATTGGGTCATCCTATTAGTAAACCGATCTGGACCGATTTATCGGATTCTTATATTATTGATCGATTTTGTCGGATATGTAGAAATCTTTGTCGTTATTACAGTGGATCCTCAAAGAAACAGGTTTTGTATCGTATAAAGTATATACTTCGACTTTCGTGTGCTAGAACTTTGTCTCGTAAACATAAAAGTACAGTACGCACTTTTATGCGAAGGTTAGGTTCGGGATTCTTAG-AAGAATTTTTTTTGGAAGAAGAACAATCTCTTTCTT

Bulbophyllum_bicolor_FT28 ACAAGAATTCTTTTTCTTCTCATTTTTCT------------TCTCAAATGCTATCAGAAGGTTTTGGAGTCATTCTGGAAATTCCATTCTCGTTGCGATTAGTATCT---------TCCCTTGAAG---ATAAAAGAATACCAAAATCTCAGAATTTACGATCTATTCATTCTATATTTCCCTTTTTAGAGGATAAATTATCGCATTTCAATTATGTGTTAG-ATC-TACT-AATACCCCATCCCATCCATCTGGAAATCTTGGTTCAAATTCTTCAATGTTGGATCAAAGATGTTCCTTCTTTGCATTTATTGCGATTGTTTTTCCACGAATATCATAATTTGAATAATCTCATTACTTC----------AAAGAAATCCATTTACGTCTTTTCAAAAAGAAA---GAAAAGA-TTTTTTTGG-TTCCTACATAATTCTTATGTATATGAATGCGAATATCTATTCCTGTTTCTTCGTAAACAGTCTTCTTATTTACGATCAATATCTTCTGGAGTCTTTCTTGAGCGAACACATTTCTATGGAAAATTAGAATATCTTATAGT---CGTGTGTTGTAATTCTTTTCA---------GAGGAGCCTATGGTTCCTCAA-AGATACTTTCATACATTATGTTCGATATCAAGGAAAAGCAATTCTGTCTTCAAAAGGAACTCTTATTCTGATAAAGAAAT-GGAAATTTCATCTTGTGAATTTTTGGCAATCTTATTTTCACTTTTGGTTTCAACCTTATAGGATCCATATAAAGCAATTACCCAACTATTCCTTCTCGTTTCTGGGG-TTTTTTTCAAGTGTACTGAAAAATCCTTTGGTAGTAA-GAAATCAAATGCTAGAGTATTCATTT-CTAATAAATACTCT-GACTAATAAATTAGATACCATAGCCCCAGTAAATTCTCTTATTGGATCATTGTCGAAAGCTCAATTTTGTACTGTATTGGGTCATCCTATTAGTAAACCGATCTGGACCGATTTATCGGATTCTTATATTATTGATCGATTTTGTCGGATATGTAGAAATCTTTGTCGTTATTACAGTGGATCCTCAAAGAAACAGGTTTTGTATCGTATAAAGTATATACTTCGACTTTCGTGTGCTAGAACTTTGTCTCGTAAACATAAAAGTACAGTACGCACTTTTATGCGAAGGTTAGGTTCGGGATTCTTAG-AAGAATTTTTTTTGGAAGAAGAACAATCTCTTTCTT

Bulbophyllum_bicolor_KFBG2210 ACAAGAATTCTTTTTCTTCTCATTTTTCT------------TCTCAAATGCTATCAGAAGGTTTTGGAGTCATTCTGGAAATTCCATTCTCGTTGCGATTAGTATCT---------TCCCTTGAAG---ATAAAAGAATACCAAAATCTCAGAATTTACGATCTATTCATTCTATATTTCCCTTTTTAGAGGATAAATTATCGCATTTCAATTATGTGTTAG-ATC-TACT-AATACCCCATCCCATCCATCTGGAAATCTTGGTTCAAATTCTTCAATGTTGGATCAAAGATGTTCCTTCTTTGCATTTATTGCGATTGTTTTTCCACGAATATCATAATTTGAATAATCTCATTACTTC----------AAAGAAATCCATTTACGTCTTTTCAAAAAGAAA---GAAAAGA-TTTTTTTGG-TTCCTACATAATTCTTATGTATATGAATGCGAATATCTATTCCTGTTTCTTCGTAAACAGTCTTCTTATTTACGATCAATATCTTCTGGAGTCTTTCTTGAGCGAACACATTTCTATGGAAAATTAGAATATCTTATAGT---CGTGTGTTGTAATTCTTTTCA---------GAGGAGCCTATGGTTCCTCAA-AGATACTTTCATACATTATGTTCGATATCAAGGAAAAGCAATTCTGTCTTCAAAAGGAACTCTTATTCTGATAAAGAAAT-GGAAATTTCATCTTGTGAATTTTTGGCAATCTTATTTTCACTTTTGGTTTCAACCTTATAGGATCCATATAAAGCAATTACCCAACTATTCCTTCTCGTTTCTGGGG-TTTTTTTCAAGTGTACTGAAAAATCCTTTGGTAGTAA-GAAATCAAATGCTAGAGTATTCATTT-CTAATAAATACTCT-GACTAATAAATTAGATACCATAGCCCCAGTAAATTCTCTTATTGGATCATTGTCGAAAGCTCAATTTTGTACTGTATTGGGTCATCCTATTAGTAAACCGATCTGGACCGATTTATCGGATTCTTATATTATTGATCGATTTTGTCGGATATGTAGAAATCTTTGTCGTTATTACAGTGGATCCTCAAAGAAACAGGTTTTGTATCGTATAAAGTATATACTTCGACTTTCGTGTGCTAGAACTTTGTCTCGTAAACATAAAAGTACAGTACGCACTTTTATGCGAAGGTTAGGTTCGGGATTCTTAG-AAGAATTTTTTTTGGAAGAAGAACAATCTCTTTCTT

Bulbophyllum_bicolor_KFBG3073 ACAAGAATTCTTTTTCTTCTCATTTTTCT------------TCTCAAATGCTATCAGAAGGTTTTGGAGTCATTCTGGAAATTCCATTCTCGTTGCGATTAGTATCT---------TCCCTTGAAG---ATAAAAGAATACCAAAATCTCAGAATTTACGATCTATTCATTCTATATTTCCCTTTTTAGAGGATAAATTATCGCATTTCAATTATGTGTTAG-ATC-TACT-AATACCCCATCCCATCCATCTGGAAATCTTGGTTCAAATTCTTCAATGTTGGATCAAAGATGTTCCTTCTTTGCATTTATTGCGATTGTTTTTCCACGAATATCATAATTTGAATAATCTCATTACTTC----------AAAGAAATCCATTTACGTCTTTTCAAAAAGAAA---GAAAAGA-TTTTTTTGG-TTCCTACATAATTCTTATGTATATGAATGCGAATATCTATTCCTGTTTCTTCGTAAACAGTCTTCTTATTTACGATCAATATCTTCTGGAGTCTTTCTTGAGCGAACACATTTCTATGGAAAATTAGAATATCTTATAGT---CGTGTGTTGTAATTCTTTTCA---------GAGGAGCCTATGGTTCCTCAA-AGATACTTTCATACATTATGTTCGATATCAAGGAAAAGCAATTCTGTCTTCAAAAGGAACTCTTATTCTGATAAAGAAAT-GGAAATTTCATCTTGTGAATTTTTGGCAATCTTATTTTCACTTTTGGTTTCAACCTTATAGGATCCATATAAAGCAATTACCCAACTATTCCTTCTCGTTTCTGGGG-TTTTTTTCAAGTGTACTGAAAAATCCTTTGGTAGTAA-GAAATCAAATGCTAGAGTATTCATTT-CTAATAAATACTCT-GACTAATAAATTAGATACCATAGCCCCAGTAAATTCTCTTATTGGATCATTGTCGAAAGCTCAATTTTGTACTGTATTGGGTCATCCTATTAGTAAACCGATCTGGACCGATTTATCGGATTCTTATATTATTGATCGATTTTGTCGGATATGTAGAAATCTTTGTCGTTATTACAGTGGATCCTCAAAGAAACAGGTTTTGTATCGTATAAAGTATATACTTCGACTTTCGTGTGCTAGAACTTTGTCTCGTAAACATAAAAGTACAGTACGCACTTTTATGCGAAGGTTAGGTTCGGGATTCTTAG-AAGAATTTTTTTTGGAAGAAGAACAATCTCTTTCTT

Bulbophyllum_bicolor_KFBG433A ACAAGAATTCTTTTTCTTCTCATTTTTCT------------TCTCAAATGCTATCAGAAGGTTTTGGAGTCATTCTGGAAATTCCATTCTCGTTGCGATTAGTATCT---------TCCCTTGAAG---ATAAAAGAATACCAAAATCTCAGAATTTACGATCTATTCATTCTATATTTCCCTTTTTAGAGGATAAATTATCGCATTTCAATTATGTGTTAG-ATC-TACT-AATACCCCATCCCATCCATCTGGAAATCTTGGTTCAAATTCTTCAATGTTGGATCAAAGATGTTCCTTCTTTGCATTTATTGCGATTGTTTTTCCACGAATATCATAATTTGAATAATCTCATTACTTC----------AAAGAAATCCATTTACGTCTTTTCAAAAAGAAA---GAAAAGA-TTTTTTTGG-TTCCTACATAATTCTTATGTATATGAATGCGAATATCTATTCCTGTTTCTTCGTAAACAGTCTTCTTATTTACGATCAATATCTTCTGGAGTCTTTCTTGAGCGAACACATTTCTATGGAAAATTAGAATATCTTATAGT---CGTGTGTTGTAATTCTTTTCA---------GAGGAGCCTATGGTTCCTCAA-AGATACTTTCATACATTATGTTCGATATCAAGGAAAAGCAATTCTGTCTTCAAAAGGAACTCTTATTCTGATAAAGAAAT-GGAAATTTCATCTTGTGAATTTTTGGCAATCTTATTTTCACTTTTGGTTTCAACCTTATAGGATCCATATAAAGCAATTACCCAACTATTCCTTCTCGTTTCTGGGG-TTTTTTTCAAGTGTACTGAAAAATCCTTTGGTAGTAA-GAAATCAAATGCTAGAGTATTCATTT-CTAATAAATACTCT-GACTAATAAATTAGATACCATAGCCCCAGTAAATTCTCTTATTGGATCATTGTCGAAAGCTCAATTTTGTACTGTATTGGGTCATCCTATTAGTAAACCGATCTGGACCGATTTATCGGATTCTTATATTATTGATCGATTTTGTCGGATATGTAGAAATCTTTGTCGTTATTACAGTGGATCCTCAAAGAAACAGGTTTTGTATCGTATAAAGTATATACTTCGACTTTCGTGTGCTAGAACTTTGTCTCGTAAACATAAAAGTACAGTACGCACTTTTATGCGAAGGTTAGGTTCGGGATTCTTAG-AAGAATTTTTTTTGGAAGAAGAACAATCTCTTTCTT

Bulbophyllum_bicolor_KFBG445 ACAAGAATTCTTTTTCTTCTCATTTTTCT------------TCTCAAATGCTATCAGAAGGTTTTGGAGTCATTCTGGAAATTCCATTCTCGTTGCGATTAGTATCT---------TCCCTTGAAG---ATAAAAGAATACCAAAATCTCAGAATTTACGATCTATTCATTCAATATTTCCCTTTTTAGAGGATAAATTATCGCATTTAAATTATGTGTTAG-ATC-TACT-AATACCCCATCCCATACATCTGGAAATCTTGGTTCAAATTATTCAATGTTGGATCAAAGATGTTCCTTCTTTGCATTTATTGCGATTGTTTTTCCACGAATATCATAATTTGAAGAATCTCATTACTTC----------AAAGAAATCCATTTACGTCTTTTCAAAAAGAAA---GAAAAGA-TTCTTTTGG-TTCCTACATAATTCTTATGTATATGAATGCGAATATCTATTCCTGTTTCTTCGTAAACAGTCTTCTTATTTACGATCAATATCTTCTGGAGTCTTTCTTGAGCGAACACATTTCTATGGAAAAATAGAATATCTTATAGT---CGTGTGTTGTAATTCTTTTCA---------GAGGAGCCTATGGTTCCTCAA-AGATACTTTCATACATTATGTTCGATATCAAGGAAAAGCAATTCTGTCTTCAAAAGGAACTCTTATTCTGATAAAGAAAT-GGAAATTTCATCTTGTGAATTTTTGGCAATCTTATTTTCACTTTTGGTTTCAACCTTATAGGATCCATATAAAGCAATTACCCAACTATTCCTTCTCGTTTCTGGGG-TTTTTTTCAAGTGTACTGAAAAATCCTTTGGTAGTAA-GAAATCAAATGCTAGAGTATTCATTT-CTAATAAATACTCT-GACTAATAAATTAGATACCATAGCCCCAGTAAATTCTCTTATTGGATCATTGTCGAAAGCTCAATTTTGTACTGTATTGGGTCATCCTATTAGTAAACCGATCTGGACCGATTTATCGGATTCTTATATTATTGATCGATTTTGTCGGATATGTAGAAATCTTTGTCGTTATTACAGCGGATCCTCAAAGAAACAGGTTTTGTATCGTATAAAGTATATACTTCGACTTTCGTGTGCTAGAACTTTGGCTCGTAAACATAAAAGTACAGTACGCACTTTTATGCGAAGGTTAGGTTCGGGATTCTTAG-AAGAATTTTTTTTGGAAGAAGAACAATCTCTTTCTT

Bulbophyllum_bicolor_KY022445 ACAAGAATTCTTTTTCTTCTCATTTTTCT------------TCTCAAATGCTATCAGAAGGTTTTGGAGTCATTCTGGAAATTCCATTCTCGTTGCGATTAGTATCT---------TCCCTTGAAG---ATAAAAGAATACCAAAATCTCAGAATTTACGATCTATTCATTCTATATTTCCCTTTTTAGAGGATAAATTATCGCATTTCAATTATGTGTTAG-ATC-TACT-AATACCCCATCCCATCCATCTGGAAATCTTGGTTCAAATTCTTCAATGTTGGATCAAAGATGTTCCTTCTTTGCATTTATTGCGATTGTTTTTCCACGAATATCATAATTTGAATAATCTCATTACTTC----------AAAGAAATCCATTTACGTCTTTTCAAAAAGAAA---GAAAAGA-TTTTTTTGG-TTCCTACATAATTCTTATGTATATGAATGCGAATATCTATTCCTGTTTCTTCGTAAACAGTCTTCTTATTTACGATCAATATCTTCTGGAGTCTTTCTTGAGCGAACACATTTCTATGGAAAATTAGAATATCTTATAGT---CGTGTGTTGTAATTCTTTTCA---------GAGGAGCCTATGGTTCCTCAA-AGATACTTTCATACATTATGTTCGATATCAAGGAAAAGCAATTCTGTCTTCAAAAGGAACTCTTATTCTGATAAAGAAAT-GGAAATTTCATCTTGTGAATTTTTGGCAATCTTATTTTCACTTTTGGTTTCAACCTTATAGGATCCATATAAAGCAATTACCCAACTATTCCTTCTCGTTTCTGGGG-TTTTTTTCAAGTGTACTGAAAAATCCTTTGGTAGTAA-GAAATCAAATGCTAGAGTATTCATTT-CTAATAAATACTCT-GACTAATAAATTAGATACCATAGCCCCAGTAAATTCTCTTATTGGATCATTGTCGAAAGCTCAATTTTGTACTGTATTGGGTCATCCTATTAGTAAACCGATCTGGACCGATTTATCGGATTCTTATATTATTGATCGATTTTGTCGGATATGTAGAAATCTTTGTCGTTATTACAGTGGATCCTCAAAGAAACAGGTTTTGTATCGTATAAAGTATATACTTCGACTTTCGTGTGCTAGAACTTTGTCTCGTAAACATAAAAGTACAGTACGCACTTTTATGCGAAGGTTAGGTTCGGGATTCTTAG-AAGAATTTTTTTTGGAAGAAGAACAATCTCTTTCTT

Bulbophyllum_bicolor_KY966722 ACAAGAATTCTTTTTCTTCTCATTTTTCT------------TCTCAAATGCTATCAGAAGGTTTTGGAGTCATTCTGGAAATTCCATTCTCGTTGCGATTAGTATCT---------TCCCTTGAAG---ATAAAAGAATACCAAAATCTCAGAATTTACGATCTATTCATTCTATATTTCCCTTTTTAGAGGATAAATTATCGCATTTCAATTATGTGTTAG-ATC-TACT-AATACCCCATCCCATCCATCTGGAAATCTTGGTTCAAATTCTTCAATGTTGGATCAAAGATGTTCCTTCTTTGCATTTATTGCGATTGTTTTTCCACGAATATCATAATTTGAATAATCTCATTACTTC----------AAAGAAATCCATTTACGTCTTTTCAAAAAGAAA---GAAAAGA-TTTTTTTGG-TTCCTACATAATTCTTATGTATATGAATGCGAATATCTATTCCTGTTTCTTCGTAAACAGTCTTCTTATTTACGATCAATATCTTCTGGAGTCTTTCTTGAGCGAACACATTTCTATGGAAAATTAGAATATCTTATAGT---CGTGTGTTGTAATTCTTTTCA---------GAGGAGCCTATGGTTCCTCAA-AGATACTTTCATACATTATGTTCGATATCAAGGAAAAGCAATTCTGTCTTCAAAAGGAACTCTTATTCTGATAAAGAAAT-GGAAATTTCATCTTGTGAATTTTTGGCAATCTTATTTTCACTTTTGGTTTCAACCTTATAGGATCCATATAAAGCAATTACCCAACTATTCCTTCTCGTTTCTGGGG-TTTTTTTCAAGTGTACTGAAAAATCCTTTGGTAGTAA-GAAATCAAATGCTAGAGTATTCATTT-CTAATAAATACTCT-GACTAATAAATTAGATACCATAGCCCCAGTAAATTCTCTTATTGGATCATTGTCGAAAGCTCAATTTTGTACTGTATTGGGTCATCCTATTAGTAAACCGATCTGGACCGATTTATCGGATTCTTATATTATTGATCGATTTTGTCGGATATGTAGAAATCTTTGTCGTTATTACAGTGGATCCTCAAAGAAACAGGTTTTGTATCGTATAAAGTATATACTTCGACTTTCGTGTGCTAGAACTTTGTCTCGTAAACATAAAAGTACAGTACGCACTTTTATGCGAAGGTTAGGTTCGGGATTCTTAG-AAGAATTTTT--------------------------

Bulbophyllum_bicolor_KY966723 ACAAGAATTCTTTTTCTTCTCATTTTTCT------------TCTCAAATGCTATCAGAAGGTTTTGGAGTCATTCTGGAAATTCCATTCTCGTTGCGATTAGTATCT---------TCCCTTGAAG---ATAAAAGAATACCAAAATCTCAGAATTTACGATCTATTCATTCTATATTTCCCTTTTTAGAGGATAAATTATCGCATTTCAATTATGTGTTAG-ATC-TACT-AATACCCCATCCCATCCATCTGGAAATCTTGGTTCAAATTCTTCAATGTTGGATCAAAGATGTTCCTTCTTTGCATTTATTGCGATTGTTTTTCCACGAATATCATAATTTGAATAATCTCATTACTTC----------AAAGAAATCCATTTACGTCTTTTCAAAAAGAAA---GAAAAGA-TTTTTTTGG-TTCCTACATAATTCTTATGTATATGAATGCGAATATCTATTCCTGTTTCTTCGTAAACAGTCTTCTTATTTACGATCAATATCTTCTGGAGTCTTTCTTGAGCGAACACATTTCTATGGAAAATTAGAATATCTTATAGT---CGTGTGTTGTAATTCTTTTCA---------GAGGAGCCTATGGTTCCTCAA-AGATACTTTCATACATTATGTTCGATATCAAGGAAAAGCAATTCTGTCTTCAAAAGGAACTCTTATTCTGATAAAGAAAT-GGAAATTTCATCTTGTGAATTTTTGGCAATCTTATTTTCACTTTTGGTTTCAACCTTATAGGATCCATATAAAGCAATTACCCAACTATTCCTTCTCGTTTCTGGGG-TTTTTTTCAAGTGTACTGAAAAATCCTTTGGTAGTAA-GAAATCAAATGCTAGAGTATTCATTT-CTAATAAATACTCT-GACTAATAAATTAGATACCATAGCCCCAGTAAATTCTCTTATTGGATCATTGTCGAAAGCTCAATTTTGTACTGTATTGGGTCATCCTATTAGTAAACCGATCTGGACCGATTTATCGGATTCTTATATTATTGATCGATTTTGTCGGATATGTAGAAATCTTTGTCGTTATTACAGTGGATCCTCAAAGAAACAGGTTTTGTATCGTATAAAGTATATACTTCGACTTTCGTGTGCTAGAACTTTGTCTCGTAAACATAAAAGTACAGTACGCACTTTTATGCGAAGGTTAGGTTCGGGATTCTTAG-AAGAATTTTT--------------------------

Bulbophyllum_bicolor_KY966724 ACAAGAATTCTTTTTCTTCTCATTTTTCT------------TCTCAAATGCTATCAGAAGGTTTTGGAGTCATTCTGGAAATTCCATTCTCGTTGCGATTAGTATCT---------TCCCTTGAAG---ATAAAAGAATACCAAAATCTCAGAATTTACGATCTATTCATTCAATATTTCCCTTTTTAGAGGATAAATTATCGCATTTCAATTATGTGTTAG-ATC-TACT-AATACCCCATCCCATCCATCTGGAAATCTTGGTTCAAATTCTTCAATGTTGGATCAAAGATGTTCCTTCTTTGCATTTATTGCGATTGTTTTTCCACGAATATCATAATTTGAATAATCTCATTACTTC----------AAAGAAATCCATTTACGTCTTTTCAAAAAGAAA---GAAAAGA-TTTTTTTGG-TTCCTACATAATTCTTATGTATATGAATGCGAATATCTATTCCTGTTTCTTCGTAAACAGTCTTCTTATTTACGATCAATATCTTCTGGAGTCTTTCTTGAGCGAACACATTTCTATGGAAAATTAGAATATCTTATAGT---CGTGTGTTGTAATTCTTTTCA---------GAGGAGCCTATGGTTCCTCAA-AGATACTTTCATACATTATGTTCGATATCAAGGAAAAGCAATTCTGTCTTCAAAAGGAACTCTTATTCTGATAAAGAAAT-GGAAATTTCATCTTGTGAATTTTTGGCAATCTTATTTTCACTTTTGGTTTCAACCTTATAGGATCCATATAAAGCAATTACCCAACTATTCCTTCTCGTTTCTGGGG-TTTTTTTCAAGTGTACTGAAAAATCCTTTGGTAGTAA-GAAATCAAATGCTAGAGTATTCATTT-CTAATAAATACTCT-GACTAATAAATTAGATACCATAGCCCCAGTAAATTCTCTTATTGGATCATTGTCGAAAGCTCAATTTTGTACTGTATTGGGTCATCCTATTAGTAAACCGATCTGGACCGATTTATCGGATTCTTATATTATTGATCGATTTTGTCGGATATGTAGAAATCTTTGTCGTTATTACAGTGGATCCTCAAAGAAACAGGTTTTGTATCGTATAAAGTATATACTTCGACTTTCGTGTGCTAGAACTTTGTCTCGTAAACATAAAAGTACAGTACGCACTTTTATGCGAAGGTTAGGTTCGGGATTCTTAG-AAGAATTTTT--------------------------

Bulbophyllum_bicolor_LMP19 ACAAGAATTCTTTTTCTTCTCATTTTTCT------------TCTCAAATGCTATCAGAAGGTTTTGGAGTCATTCTGGAAATTCCATTCTCGTTGCGATTAGTATCT---------TCCCTTGAAG---ATAAAAGAATACCAAAATCTCAGAATTTACGATCTATTCATTCTATATTTCCCTTTTTAGAGGATAAATTATCGCATTTCAATTATGTGTTAG-ATC-TACT-AATACCCCATCCCATCCATCTGGAAATCTTGGTTCAAATTCTTCAATGTTGGATCAAAGATGTTCCTTCTTTGCATTTATTGCGATTGTTTTTCCACGAATATCATAATTTGAATAATCTCATTACTTC----------AAAGAAATCCATTTACGTCTTTTCAAAAAGAAA---GAAAAGA-TTTTTTTGG-TTCCTACATAATTCTTATGTATATGAATGCGAATATCTATTCCTGTTTCTTCGTAAACAGTCTTCTTATTTACGATCAATATCTTCTGGAGTCTTTCTTGAGCGAACACATTTCTATGGAAAATTAGAATATCTTATAGT---CGTGTGTTGTAATTCTTTTCA---------GAGGAGCCTATGGTTCCTCAA-AGATACTTTCATACATTATGTTCGATATCAAGGAAAAGCAATTCTGTCTTCAAAAGGAACTCTTATTCTGATAAAGAAAT-GGAAATTTCATCTTGTGAATTTTTGGCAATCTTATTTTCACTTTTGGTTTCAACCTTATAGGATCCATATAAAGCAATTACCCAACTATTCCTTCTCGTTTCTGGGG-TTTTTTTCAAGTGTACTGAAAAATCCTTTGGTAGTAA-GAAATCAAATGCTAGAGTATTCATTT-CTAATAAATACTCT-GACTAATAAATTAGATACCATAGCCCCAGTAAATTCTCTTATTGGATCATTGTCGAAAGCTCAATTTTGTACTGTATTGGGTCATCCTATTAGTAAACCGATCTGGACCGATTTATCGGATTCTTATATTATTGATCGATTTTGTCGGATATGTAGAAATCTTTGTCGTTATTACAGTGGATCCTCAAAGAAACAGGTTTTGTATCGTATAAAGTATATACTTCGACTTTCGTGTGCTAGAACTTTGTCTCGTAAACATAAAAGTACAGTACGCACTTTTATGCGAAGGTTAGGTTCGGGATTCTTAG-AAGAATTTTTTTTGGAAGAAGAACAATCTCTTTCTT

Bulbophyllum_bicolor_PSL45 ACAAGAATTCTTTTTCTTCTCATTTTTCT------------TCTCAAATGCTATCAGAAGGTTTTGGAGTCATTCTGGAAATTCCATTCTCGTTGCGATTAGTATCT---------TCCCTTGAAG---ATAAAAGAATACCAAAATCTCAGAATTTACGATCTATTCATTCTATATTTCCCTTTTTAGAGGATAAATTATCGCATTTCAATTATGTGTTAG-ATC-TACT-AATACCCCATCCCATCCATCTGGAAATCTTGGTTCAAATTCTTCAATGTTGGATCAAAGATGTTCCTTCTTTGCATTTATTGCGATTGTTTTTCCACGAATATCATAATTTGAATAATCTCATTACTTC----------AAAGAAATCCATTTACGTCTTTTCAAAAAGAAA---GAAAAGA-TTTTTTTGG-TTCCTACATAATTCTTATGTATATGAATGCGAATATCTATTCCTGTTTCTTCGTAAACAGTCTTCTTATTTACGATCAATATCTTCTGGAGTCTTTCTTGAGCGAACACATTTCTATGGAAAATTAGAATATCTTATAGT---CGTGTGTTGTAATTCTTTTCA---------GAGGAGCCTATGGTTCCTCAA-AGATACTTTCATACATTATGTTCGATATCAAGGAAAAGCAATTCTGTCTTCAAAAGGAACTCTTATTCTGATAAAGAAAT-GGAAATTTCATCTTGTGAATTTTTGGCAATCTTATTTTCACTTTTGGTTTCAACCTTATAGGATCCATATAAAGCAATTACCCAACTATTCCTTCTCGTTTCTGGGG-TTTTTTTCAAGTGTACTGAAAAATCCTTTGGTAGTAA-GAAATCAAATGCTAGAGTATTCATTT-CTAATAAATACTCT-GACTAATAAATTAGATACCATAGCCCCAGTAAATTCTCTTATTGGATCATTGTCGAAAGCTCAATTTTGTACTGTATTGGGTCATCCTATTAGTAAACCGATCTGGACCGATTTATCGGATTCTTATATTATTGATCGATTTTGTCGGATATGTAGAAATCTTTGTCGTTATTACAGTGGATCCTCAAAGAAACAGGTTTTGTATCGTATAAAGTATATACTTCGACTTTCGTGTGCTAGAACTTTGTCTCGTAAACATAAAAGTACAGTACGCACTTTTATGCGAAGGTTAGGTTCGGGATTCTTAG-AAGAATTTTTTTTGGAAGAAGAACAATCTCTTTCTT

Bulbophyllum_bicolor_TMS05 ACAAGAATTCTTTTTCTTCTCATTTTTCT------------TCTCAAATGCTATCAGAAGGTTTTGGAGTCATTCTGGAAATTCCATTCTCGTTGCGATTAGTATCT---------TCCCTTGAAG---ATAAAAGAATACCAAAATCTCAGAATTTACGATCTATTCATTCTATATTTCCCTTTTTAGAGGATAAATTATCGCATTTCAATTATGTGTTAG-ATC-TACT-AATACCCCATCCCATCCATCTGGAAATCTTGGTTCAAATTCTTCAATGTTGGATCAAAGATGTTCCTTCTTTGCATTTATTGCGATTGTTTTTCCACGAATATCATAATTTGAATAATCTCATTACTTC----------AAAGAAATCCATTTACGTCTTTTCAAAAAGAAA---GAAAAGA-TTTTTTTGG-TTCCTACATAATTCTTATGTATATGAATGCGAATATCTATTCCTGTTTCTTCGTAAACAGTCTTCTTATTTACGATCAATATCTTCTGGAGTCTTTCTTGAGCGAACACATTTCTATGGAAAATTAGAATATCTTATAGT---CGTGTGTTGTAATTCTTTTCA---------GAGGAGCCTATGGTTCCTCAA-AGATACTTTCATACATTATGTTCGATATCAAGGAAAAGCAATTCTGTCTTCAAAAGGAACTCTTATTCTGATAAAGAAAT-GGAAATTTCATCTTGTGAATTTTTGGCAATCTTATTTTCACTTTTGGTTTCAACCTTATAGGATCCATATAAAGCAATTACCCAACTATTCCTTCTCGTTTCTGGGG-TTTTTTTCAAGTGTACTGAAAAATCCTTTGGTAGTAA-GAAATCAAATGCTAGAGTATTCATTT-CTAATAAATACTCT-GACTAATAAATTAGATACCATAGCCCCAGTAAATTCTCTTATTGGATCATTGTCGAAAGCTCAATTTTGTACTGTATTGGGTCATCCTATTAGTAAACCGATCTGGACCGATTTATCGGATTCTTATATTATTGATCGATTTTGTCGGATATGTAGAAATCTTTGTCGTTATTACAGTGGATCCTCAAAGAAACAGGTTTTGTATCGTATAAAGTATATACTTCGACTTTCGTGTGCTAGAACTTTGTCTCGTAAACATAAAAGTACAGTACGCACTTTTATGCGAAGGTTAGGTTCGGGATTCTTAG-AAGAATTTTTTTTGGAAGAAGAACAATCTCTTTCTT

Bulbophyllum_bicolor_TT43 ACAAGAATTCTTTTTCTTCTCATTTTTCT------------TCTCAAATGCTATCAGAAGGTTTTGGAGTCATTCTGGAAATTCCATTCTCGTTGCGATTAGTATCT---------TCCCTTGAAG---ATAAAAGAATACCAAAATCTCAGAATTTACGATCTATTCATTCTATATTTCCCTTTTTAGAGGATAAATTATCGCATTTCAATTATGTGTTAG-ATC-TACT-AATACCCCATCCCATCCATCTGGAAATCTTGGTTCAAATTCTTCAATGTTGGATCAAAGATGTTCCTTCTTTGCATTTATTGCGATTGTTTTTCCACGAATATCATAATTTGAATAATCTCATTACTTC----------AAAGAAATCCATTTACGTCTTTTCAAAAAGAAA---GAAAAGA-TTTTTTTGG-TTCCTACATAATTCTTATGTATATGAATGCGAATATCTATTCCTGTTTCTTCGTAAACAGTCTTCTTATTTACGATCAATATCTTCTGGAGTCTTTCTTGAGCGAACACATTTCTATGGAAAATTAGAATATCTTATAGT---CGTGTGTTGTAATTCTTTTCA---------GAGGAGCCTATGGTTCCTCAA-AGATACTTTCATACATTATGTTCGATATCAAGGAAAAGCAATTCTGTCTTCAAAAGGAACTCTTATTCTGATAAAGAAAT-GGAAATTTCATCTTGTGAATTTTTGGCAATCTTATTTTCACTTTTGGTTTCAACCTTATAGGATCCATATAAAGCAATTACCCAACTATTCCTTCTCGTTTCTGGGG-TTTTTTTCAAGTGTACTGAAAAATCCTTTGGTAGTAA-GAAATCAAATGCTAGAGTATTCATTT-CTAATAAATACTCT-GACTAATAAATTAGATACCATAGCCCCAGTAAATTCTCTTATTGGATCATTGTCGAAAGCTCAATTTTGTACTGTATTGGGTCATCCTATTAGTAAACCGATCTGGACCGATTTATCGGATTCTTATATTATTGATCGATTTTGTCGGATATGTAGAAATCTTTGTCGTTATTACAGTGGATCCTCAAAGAAACAGGTTTTGTATCGTATAAAGTATATACTTCGACTTTCGTGTGCTAGAACTTTGTCTCGTAAACATAAAAGTACAGTACGCACTTTTATGCGAAGGTTAGGTTCGGGATTCTTAG-AAGAATTTTTTTTGGAAGAAGAACAATCTCTTTCTT
[truncated: 1,752,102 more chars]
