# Supplementary material for: Hong Kong orchids on the EDGE: a phylogenetic framework for conservation planning, trade mitigation and population rescue
Source: Front Plant Sci. 2026 Apr 28;17:1801915. doi: 10.3389/fpls.2026.1801915 (PMC13161184; doi:10.3389/fpls.2026.1801915)
Supplement: Supplementary file 4 [file Table4.docx]

#NEXUS

begin taxa;

dimensions ntax=137;

taxlabels

Acampe_praemorsa_var_longepedunculata_SG1199

Acanthophippium_gougahense_KFBG3161

Ania_hongkongensis_SG1231

Ania_ruybarrettoi_SG1395

Anoectochilus_formosanus_PK12215

Anoectochilus_roxburghii_SG1219

Aphyllorchis_montana_SG1010

Apostasia_nipponica_PK12273

Appendicula_cornuta_PK12065

Arundina_graminifolia_SG1295

Bletilla_striata_KFBG2048

Brachycorythis_galeandra_SG1261

Bulbophyllum_affine_SG1606

Bulbophyllum_ambrosia_SG1221

Bulbophyllum_bicolor_FT28

Bulbophyllum_delitescens_SG1286

Bulbophyllum_kwangtungense_KFBG2798

Bulbophyllum_odoratissimum_SG1275

Bulbophyllum_pectenveneris_KFBG294

Bulbophyllum_scabratum_PK12041

Bulbophyllum_stenobulbon_SG1226

Bulbophyllum_tigridum_SG1310

Bulbophyllum_tseanum_SG1272

Calanthe_dominyi_SG1359

Calanthe_graciliflora_PK12206

Calanthe_masuca_SG1360

Calanthe_speciosa_SG1368

Calanthe_triplicata_SG1311

Cephalantheropsis_obcordata_PK12079

Cheirostylis_clibborndyeri_SG1349

Cheirostylis_jamesleungii_PK12205

Cheirostylis_monteiroi_SG1344

Cheirostylis_pusilla_HK43263

Cheirostylis_yunnanensis_SG1227

Chrysoglossum_assamicum_SG1622

Cleisostoma_paniculatum_KFBG516

Cleisostoma_rostratum_SG1200

Cleisostoma_simondii_SG1314

Cleisostoma_simondii_var_guangdongense_KFBG2212

Cleisostoma_williamsonii

Coelogyne_cantonensis_SG1239

Coelogyne_chinensis_SG1232

Coelogyne_fimbriata_SG1059

Coelogyne_fimbriata_var_leungiana_SG1058

Collabium_chinense

Crepidium_allanii_KFBG4610

Crepidium_cordilabium_PK12271

Crepidium_purpureum_SG1193

Cryptochilus_roseus_PK12087

Cryptostylis_arachnites_SG1380

Curculigo_orchioides_PK12054

Curculigo_orchioides_SG1196

Cymbidium_aloifolium_KFBG2049

Cymbidium_ensifolium_SG1214

Cymbidium_kanran

Cymbidium_lancifolium_SG1274

Cymbidium_sinense_SG1342

Dendrobium_aduncum_KFBG8766

Dendrobium_anosmum

Dendrobium_cf_mimicum_PK12237E

Dendrobium_crumenatum

Dendrobium_linawianum_SG1347

Dendrobium_lindleyi_KFBG203

Dendrobium_loddigesii_SG1255

Dendrobium_spatella_SG1357

Dendrolirium_lasiopetalum_SG1312

Didymoplexiella_siamensis_SG1242

Dienia_ophrydis_SG1276

Diploprora_championii_SG1230

Disperis_neilgherrensis

Epipogium_roseum_SG1249

Eria_scabrilinguis_SG1302

Erythrodes_blumei_PK12103

Eulophia_flava_SG1158

Eulophia_graminea_SG1270

Eulophia_picta_SG1271

Eulophia_zollingeri_SG1262

Gastrochilus_japonicus_KFBG308

Gastrochilus_kadooriei_PK12022

Gastrodia_peichatieniana_AFCDHK43268

Goodyera_foliosa_SG1309

Goodyera_procera_SG1152

Goodyera_pusilla_KM593694

Goodyera_seikoomontana_SG1252

Goodyera_viridiflora_SG1305

Habenaria_ciliolaris

Habenaria_dentata_SG1005

Habenaria_leptoloba_SG1304

Habenaria_linguella_SG1195

Habenaria_reniformis_SG1296

Habenaria_rhodocheila_SG1289

Hetaeria_youngsayei_SG1244

Hypoxis_rigidula_SG1207

Lecanorchis_nigricans_SG1280

Liparis_bootanensis_SG1215

Liparis_ferruginea_SG1156

Liparis_gigantea_PK12116

Liparis_nervosa_SG1233

Liparis_odorata_SG1256

Liparis_sootenzanensis_SG1351

Liparis_stricklandiana_SG1332

Liparis_viridiflora_SG1308

Ludisia_discolor_SG1236

Nephelaphyllum_tenuiflorum_SG1220

Nervilia_plicata_SG1143

Neuwiedia_zollingeri_var_singapureana_KFBG35

Pachystoma_pubescens_PK12108

Paphiopedilum_purpuratum_SG1149

Pecteilis_susannae_SG1292

Peristylus_calcaratus_SG1303

Peristylus_densus_SG1258

Peristylus_goodyeroides

Peristylus_intrudens_SG1298

Peristylus_lacertifer_SG1006

Persitylus_tentaculatus_SG1007

Phaius_tankervilleae_PK12084

Phaius_wallichii_KFBG2002A

Platanthera_mandarinorum

Platanthera_minor_SG1154

Porpax_pusilla_SG1334

Renanthera_coccinea

Rhomboda_abbreviata_PK12175

Robiquetia_succisa_SG1293

Spathoglottis_pubescens_SG1205

Spiranthes_hongkongensis_PK12028

Spiranthes_sinensis_SG1153

Tainia_cordifolia

Tainia_dunnii_SG1273

Thelasis_pygmaea

Thrixspermum_centipeda_PK12129

Tropidia_curculigoides_SG1281

Tropidia_nipponica_SG1355

Vanilla_shenzhenica_KFBG290

Vrydagzynea_nuda_SG1222

Zeuxine_boninensis_d16

Zeuxine_gracilis_SG1204

Zeuxine_strateumatica_SG1211

;

end;

begin characters;

dimensions nchar=3098;

format datatype=dna missing=? gap=-;

matrix

Acampe_praemorsa_var_longepedunculata_SG1199 ACGACT-CTCGACAATGGATATCTCGGCTCTCGCATCGATGAAGAGCGCAGCGAAATGCGATACGTGGTGCGAATTGCAGAATCCCGCGAACCATCGAGTCTTTGAACGCAAGTTGCGCCCGAGGCCAAT-CGGTCGAGGGCACGTCCGCCTGGGCGTCA-AGCGTTGCGCCGCTCCACAAGAATTCTTTTTCTTATCATTTTTAT------------TCTCAAATGGTATCAGAAGGTTTTGGAGTCATTCTGGAAATTTCATTCTCGTCGCGATTAGTATCC---------TCCCTTGAAG---AAAAAAGAATACCAAAATCTCAGAATTTACGATCTATTCATTCAATATTTCCCTTTTTAGAGGATAAATTATCACATTTAAATTATGTGTCGGATCTACTAATACCCTATCCCATCCATCTGGAAATCTTGGTTCAAATCCTTCAATGCTGGATCAAAGATGTTCCTTCTTTGCATTTCTTGCGATTGATTTTCCACGAATATCAT---------------AATTTGAATAGTCTC---------ATTACTT------CAAAAAAATC---------------CATTTACGTCTTTTCAAAAAA---AAAGAAAAGATTCTTTTGGTTCCTACATAATTTTTATGTATATGAATGCGAATATATATTCCTCTTTCTTCGTAAACAGTCTTCTTATTTACGATCAATATCTTCTGGAGTCTTTCTTGAGCGAACACATTTTTATGGAAAAATAGAA------TATCTTAGAGT---CGTGTCTTGTAATTCTTTTCAGAGG-ATCCTATGGTTCCTCAAAGATATTTTCATACATTATGTTCGATATCAAGGAAAAGCAATTTTGGCTTCAAAAGGAACTCTTATTCTGATGAATAAATGGAAATTTCATTTTGTGAATTTTTGGCAATCTTATTTTCACTTTTGGTTTCAACCTTATAGGATCCATATAAAGCAATTACCCAATTATTCCTTCTCTTTTCT-GGGATATTTTTCAAGTGTAC-TAAAAAACCCTTTGGTAGTAAGAAATCAAATGCTAGAGAATTCATTTCTAATAAATACTCTGACTAAGAAATTAGATACCATAGCTCCAGTTCTTTTTCTTATTGGATCATTGTCGAAAGCTCAATTTTGTACTGTATTAGGTCATCCTATTAGTAAACCGATCTGGACCAATTTATCGGATTCTGATATTCTTGATCGATTTTGTCGGATATGTAGAAATCTTTGTCGTTATCACAGCGGATCCTCAAAGAAACAGGTTTTGTATCGTATAAAGTATATACTTCGACTTTCGTGTGCTAGAACTTTGGCTCGTAAACATAAAAGTAC-AGTACGCACTTTTATGCGAAGATTAGGTTCGGGATTCTTAGAAGAATTTTTTTTGGAAGAAGAACAATTTCTTTCCTGAGCTATCCCGACCATTTCCCGTGCATCATCCTAGCAGAGTACTTC-TATCTATGTCAATGAAAAGAA-CTAAAAAAGAAAATCTTAACAA-------------------ATTGGCTCTAG--CCCCT-GAAATT----------CTTGG-------ATC-TTCAAAAA----------GAAGAC--TTTTTTTT-----------GTAAA-TGTAAGGAAAAAGATATAGACTAT-GAATGA------------TTCAATAAC-GGAGATTCCTTGAAC-------------------------ATATATCTTCAT-------------ATCGTA----TTAT------ACAAAA--------CAAAT-----GAGA--------TTGGATTGGAA---AAAGAT--------------------------ACGAGGATTTAG------ATTTGGATCCATT------TGTGAAAGAACAGAG--TGAATAAAATGAGAAAG-------ATATTTCATTT--------------TGTTTAAACT------------GAGTCACTGATGAACAGAGGAT-------------------------------GAGGATAAAGA-AAGA------------------GCGAGGAA----------GTAAAAT-----GGGC-TTTTTATTGGGGATAGAGGACCTTCGAT---------AAATGGATAATACT--------TTTATCTTCATATGAATTTTTGAAGATAG--CAAT---------CCCCCAATATCT-TGT---------------TCTTGGAACAAGATATT--GGGGGATTCTTTTG---CTTCTCTA---------------------TTTTCG---------TTCTTTATCAT----AAAAGTTTTC-CCCCGCC---------AATGAATGATAAGT-GCC-----TAGGTG-AAGTAT----------------AGTATAAGATAAGTCAGA----------------------AAAGTCTAAGTCTTC-----ATACCTATACTCTTA----------CTATAA-GATAAA-----AGATAAAGACTCTTAAGGATAAGGCTTTTCTTTTCAT----------ATGAATACTTAGTAGAACGACTAACGACGAGATTTATTATCGTTTCTCGCGTGTCTCACGAAAGTTAGAGTAG-GTGCGAATTCTCCCAATTTGTGACCGACCATACGATCTGTGATATAAATGGTAAA-TGTTCCTTTCCATTATGAATAGC-GATTGTATGGCCAATCATTGTGGGTA-TAATGGTAGATGCCCGAGACCAAGTCACTATGATTTCTTTCTCCTCCCTCCTGTT-GAGTTTTTCAATTCTTCCCAATAAATGATTAGCTACAAAAGGATTTTTTTTT-AGTGAACGTGTCAC-GGCTGATTACTCCTTTTTTTACATTTTT-TAAATTGGCATTCTATGTCCAA----------TATCTCGATCTTAA-TCTG-AAG-TCT---------------------------AATG-ATGAATGG-AAAAAAGAGAAAATCC----------TTTA-GCTAG-ATAA--------GGG-AA-GGGGC-GGATGTAG-CCAAGTGG-ATCAA-GGCAGT

Acanthophippium_gougahense_KFBG3161 ATGACT-CTCGGCAATGGATATCTCGGCTCTCGCATCGATGAAGAGCGCAGCGAAATGCGATACGTGGTGCGAATTGCAGAATCCCGCGAACCATCGAGTCTTTGAACGCAAGTTGCGCCTGAGGCCATC-CGGCCAAGGGCACGTCTGCCTGGGCGTCA-AGCGTTGCGTCGCTTCACAAGAATTCTTTTTCTTCTCATTTTTCT------------TCTCAAATGGTATCAGAAGGTTTTGGAGTCATTCTGGAAATTCCATTCTCGTCGCGATTAGTATCT---------TCCCTTGAAG---AAAAAAGAATACCAAAATCTCAGAATTTACGATCTATTCATTCAATATTTCCCTTTTTAGAGGATAAATTCTCACATTTAAATTATGTGTCAGATCTACTAATACCCCATCCCATCCATCTGGAAATCTTGGTTCAAATCCTTCAATGCTGGATCAAAGATGTTCCTTCTTTGCATTTATTGCGATTGTTTTTCCACGAATATCAT---------------AATTTGAATAGTCTC---------ATTACTT------CAATGAAATC---------------CATTTACGTCTTTTCAAAAAG---AAAGAAAAGATTCTTTCGGTTCCTACATAATTCTTATGTATATGAATGCGAATATCTATTCCTGTTTCTTCGTAAACAGTCTTCTTATTTACGATCAAGATCTTCTGGAGTCTTTCTTGAGCGAACACATTTCTATGGAAAAATAGAA------TATCTTATAGT---CGTGTGTTGTAATTATTTTCAGAGG-ATCCTATGGTTCCTCAAAGATACTTTCATACATTATGTTCGATATCAAGGAAAAGCGATTCTGGCTTCAAAAGGAACTCTTATTCTGATGAAGAAATGGAAATTTCATCTTGTAAATTTTTGGCAATCTTATTTTCACTTTTGGTTTCAACCTTATAGGATCCTTATAAAGCAATTACCTAACTATTCCTTCTCTTTTCT-GGGGTATTTTTCAAGTGTAC-TAAAAAATCCTTTGGTAGTAAGAAATCAAATGCTAGAGAATTCATTTCTAATAAATACTCTGACTAAGAAATTAGATACCATAGCCCCAGTTATTTCTCTTATTGGATCATTGTCGAAAGCTCAATTTTGTACTGTATTGGGTCATCCTATTAGTAAACCAATCTGGACCGATTTATCGGATTCTGATATTCTTTATCGATTTTGTCGGATATGTAGAAATCTTTGTCATTATTACAGTGGATCTTCAAAGAAACAGGTTTTGTATCGTATAAAGTATATACTTCGACTTTCGTGTGCTAGAACTTTGGCTCGTAAACATAAAAGTAC-AGTACGCACTTTTATGCGAAGATTAGGTTCGGGATTCTTAGAAGAATTTTTTTTGGAAGAAGAACAATCTCTTTCTTGAGCTATCCCGACCATTTCCGGTGCATCATACTAGCAGAGTACTTA-TATCTATGTCAATGAAAAGAA-CTAAAAAATAAAATCTTAACAA-------------------ATTGGACCTAG--CCCCT-GAATTT----------CTTAG-------ATC-TTCAAAAA----------GAAGAC---ATTCTTT-----------GTAAA-TGTAAGGAAAAAGATATGGACTAT-GAATGA------------TTCAATAAC-GGAAATTCCTTGAAC-------------ATATATATATATATATATGTTCAT-------------ATCGTA----CTATATAAATACTATATCAAA---CAAAT-----GAGA--------TTGGATTGGAA---GAAGAT--------------------------ACGAGGATTTCT------ATTCGGATCCATT------TGTGAAAGAACAGAG--TGAATGAAATGAGAAAG-------ATATTTCATTT--------------TGTTTAAACT------------GAGCC--------------GAT-------------------------------GAGGATAAATA-AAGA------------------GCGAGGAA----------GTAAAAT-----GGGC-TTTTTATTGGGGATAGAGGGCCATCTAT---------AAATGGATAAGACT--------TTTCTATTCATATGAATTTTTGAAGATAG--CAAT---------TTCCCAAGA----------------------TCTTAGAACAAGATCTT--GGGAAATTCTTTTG---CTTCTCTATCCG---------------AATTTTCG---------TTCTTTCTCAT----AAAAGTTCTC-CCCCGCC-------------------------------------------------------------------------------------------------------------------------ATACCTATACTCTTA----------CTATAA-GAT------------AAAGACTCTTAAGGATAAGGC-----TTTTCAC----------ATGAATACTTAGTAGAACGACTAACGACGAGATTTATTATCGTTTCTCGCGTGTCTCACGAAAGTGAGAGTAG-GTGCGAATTCTCCCAATTTGTGACCGACCATACGATCTGTGATATAAATGGTAAA-TGTTCCTTTCCATTATGAATAGC-GATTGTATGGCCAATCATTGTGGGTA-TAATGGTAGATGCCCGAGACCAAGTCACTATGATTTCTTTCTCCTCCCTCCTGTT-GAGTTTTTCAATTCTTCCCGATAAATGATTAGCTACAAAAGGATTTTTTTTT-AGTGAACGTGTCAC-GGCTGATTACTCCTTTTTTTCCATTTTT-TAAATTGGCATTCTATGTCCAA----------TATCTCGATCTTAA-TCTG-AAG-TAT---------------------------AATG-ATGAATGG-AAAAAAGAGAAAATCC----------CTTA-GCTAG-ATAA--------GGG-AA-GGGGC-GGATGTAG-CCAAGTGG-ATCAA-GGCAGT

Ania_hongkongensis_SG1231 ACGACT-CTCGGCAATGGATATCTCGGCTCTCGCATCGATGAAGAGCGCAGCGAAATGCGATACGTGGTGCGAATTGCAGAATCCCGCGAACCATCGAGTCTTTGAACGCAAGTTGCGCCCGAGGCCAAC-CGGCCAAGGGCACGTCTGCCTGGGCGTCA-AGCGTTGCGTCGCTCCACAAGAATTCTTTTTCTTCTCATTTTTCT------------TCTCAAATGGTATCAGAAGGTTTTGGAGTCATTCTGGAAATTCCATTCTCGTCGCGATTAGTATCT---------TCCCTTGAAG---AAAAAAGAATACCAAAATCTCAGAATTTACGATCTATTCATTCAATATTTCCCTTTTTAGAGGATAAATTATCACATTTAAATTATGTGTCAGATCTACTAATACCCCATCCCATCCATCTGGAAATCTTGGTTCAAATCCTTCAATGCTGGATCAAAGATGTTCCTTCTTTGCATTTATTGCGATTGTTTTTCCACGAATATCAT---------------AATTTGAATAGTCTC---------ATTACTT------CAAAGAAATC---------------CATTTACGTCTTTTCAAAAAG---AAAGAAAAGATTCTTTTGGTTCCTACATAATTCTTATGTATATGAATGCGAATATCTATTCCTGTTTCTTCGTAAACAGTCTTCTTATTTACGATCAATATCTTCTGGAGTCTTTCTTGAGCGAACACATTTCTATGGAAAAATAGAA------TATCTTATAGT---CGTGTGTTGTAATTCTTTTCAGAGG-ATCCTATGGTTCCTTAAAGATACTTTCATACATTATGTTCGATATCAAGGAAAAGCGATTATGGCTTCAAAAGGAACTCTTATTCTGATGAAGAAATGGAAATTTCATCTTGTAAATTTTTGGCAATCTTATTTTCACTTTTGGTTTCAACCTTATAGGATCCATATAAAGCAATTACCCAACTATTTCTTCTCTTTTCT-GGGGTATTTTTCAAGTGTAC-TAAAAAACCCTTTGGTAGTAAGAAATCAAATGCTAGAGAATTCATTTCTAATAAATACTCTGACTAAGAAATTAGATACCATAGCCCCAGTTATTTCTCTTATTGGATCATTGTCGAAAGCTCAATTTTGTACTGTATTGGGTCATCCTATTAGTAAACCAATCTGGACCGATTTATCGGATTCTGATATTCTTGATCGATTTTGTCGGATATCTAGAAATCTTTGTCGTTATCACAGCGGATCCTCAAAGAAACAGGTTTTATATCGTATAAAGTATATACTTCGACTTTCGTGTGCTAGAACTTTGGCTCGTAAACATAAAAGTAC-AGTACGCACTCTTATGCGAAGATTAGGTTCGGGATTCTTAGAAGAATTTTTTTTGGAAGAAGAACAATCTCTTTCTTGAGCTATCCCGACCATTTCCCGTGCGTCATCCTAGCAGAGTACTTA-TATCTATGTCAATGAAAAGAA-TTAAAAAAGAAAATCTTAACAA-------------------ATTGGACCTAG--CCCCT-GAATTT----------CTTAG-------ATC-TTCAAAAA----------GAAGAC---ATTCTTT-----------GTAAA-TGTCAGGAAAAAGATATGGACTAT-GAATGA------------TTCAATAAC-GGAAATTCCTTGAAC---------------------ATATATATATGTTCAT-------------ATCGTA----CTAT------ACAAAAAAA-----CAAAT-----GAGA--------TTGGATTGGAA---GAAGAT--------------------------ACGAGGATTTCT------ATTCGGATCCATT------TGTGAAAGAACAGAG--TGAATGAAATGAGAAAG-------ATATTTCATTT--------------TGTTTAAACTGAGCCA------GAGCCACTGATGGAAAGAGGAT-------------------------------GAGGATAAATA-AAGA------------------GTGAGGAA----------GTAAAAT-----GGGC-TTTTTATTGGGGATAGAGGGCCATCTAT---------AAATGGATAATACT--------TTTGTATTCATATGAATTTTTGAAGATAG--CAAT---------CCCCCAAGATCT-T------------------------------------GGGGGATTCTTTTG---CTTCTCTATCCG---------------AATTTTCG---------TTCTTTATCAT----AAAAGTTCTC-CCCCGCC---------AATGAATGATAAGT-GTC-----TAGGTG-AAGTAT----------------AGTATAAGATAAGTCAGA----------------------AAAGTATAAGTCTTA-----ATACCTATACTCTTA----------CTATAA-GAT------------AAAGACTCTTAAGGATAAGGC-----TTTTCAC----------ATGAATACTTAGTAGAACGACTAACGACGAGATTTATTATCGTTTCTCGCGTGTCTCACGAAAGTGAGAGTAG-GTGCGAATTCTCCCAATTTGTGACCGACCATACGATCTGTGATATAAATGGTAAA-TGTTCCTTTCCATTATGAATAGC-GATTGTATGGCCAATCATTGTGGGTA-TAATGGTAGATGCCCGAGACCAAGTCACTATGATTTCTTTCTCCTCCCTCCTGTT-GAGTTTTTCAATTCTTCCCGATAAATGATTAGCTACAAAAGGATTTTTTTTT-AGTGAACGTGTCAC-GGCTGATTACTCCTTTTTTTACATTTTT-GAAATTGGCATTCTATGTCCAA----------TATCTCGATCTTAA-TCTG-AAG-TAT---------------------------AATG-ATGAATGG-AAAAAATAGAAAATCC----------TTTA-GCTAG-ATAA--------GGR-AA-GGGGC-GGATGTAG-CCAAGTGG-ATCAA-GGCAGT

Ania_ruybarrettoi_SG1395 ACGACT-CTCGGCAATGGATATCTCGGCTCTCGCATCGATGAAGAGCGCAGCGAAATGCGATACGTGGTGCGAATTGCAGAATCCCGCGAACCATCGAGTCTTTGAACGCAAGTTGCGCCCGAGGCCAAC-CGGCCAAGGGCACGTCTGCCTGGGCGTCA-AGCGTTGCGTCGCTCCACAAGAATTCTTTTTCTTCTCATTTTTCT------------TCTCAAATGGTATCAGAAGGTTTTGGAGTCATTCTGGAAATTCCATTCTCGTCGCGATTAGTATCT---------TCCCTTGAAG---AAAAAAGAATACCAAAATCTCAGAATTTACGATCTATTCATTCAATATTTCCCTTTTTAGAGGATAAATTATCACATTTAAATTATGTGTCAGATCTACTAATACCCCATCCCATCCATCTGGAAATCTTGGTTCAAATCCTTCAATGCTGGATCAAAGATGTTCCTTCTTTGCATTTATTGCGATTGTTTTTCCACGAATATCAT---------------AATTTGAATAGTCTC---------ATTACTT------CAAAGAAATC---------------CATTTACGTCTTTTCAAAAAG---AAAGAAAAGATTCTTTTGGTTCCTACATAATTCTTATGTATATGAATGCGAATATCTATTCCTGTTTCTTCGTAAACAGTCTTCTTATTTACGATCAATATCTTCTGGAGTCTTTCTTGAGCGAACACATTTCTATGGAAAAATAGAA------TATCTTATAGT---CGTGTGTTGTAATTCTTTTCAGAGG-ATCCTATGGTTCCTCAAAGATACTTTCATACATTATGTTCGATATCAAGGAAAAGCGATTATGGCTTCAAAAGGAACTCTTATTCTGATGAAGAAATGGAAATTTCATCTTGTAAATTTTTGGCAATCTTATTTTCACTTTTGGTTTCAACCTTATAGGATCCATATAAAGCAATTACCCAACTATTTCTTCTCTTTTCT-GGGGTATTTTTCAAGTGTAC-TAAAAAACCCTTTGGTAGTAAGAAATCAAATGCTAGAGAATTCATTTCTAATAAATACTCTGACTAAGAAATTAGATACCATAGCCCCAGTTATTTCTCTTATTGGATCATTGTCGAAAGCTCAATTTTGTACTGTATTGGGTCATCCTATTAGTAAACCAATCTGGACCGATTTATCGGATTCTGATATTCTTGATCGATTTTGTCGGATATCTAGAAATCTTTGTCGTTATCACAGCGGATCCTCAAAGAAACAGGTTTTGTATCGTATAAAGTATATACTTCGACTTTCGTGTGCTAGAACTTTGGCTCGTAAACATAAAAGTAC-AGTACGCACTTTTATGCGAAGATTAGGTTCGGGATTCTTAGAAGAATTTTTTTTGGAAGAAGAACAATCTCTTTCTTGAGCTATCCCGACCATTTCCCGTGCATCATCCTAGCAGAGTACTTA-TATCTATGTCAATGAAAAGAA-TTAAAAAAGAAAATCTTAACAA-------------------ATTAGACCTAG--CCCCT-GAATTT----------CTTAG-------ATC-TTCAAAAA----------GAAGAC---ATTCTTT-----------GTAAA-TGTCAGGAAAAAGATATGGACTAT-GAATGA------------TTCAATAAC-GGAAATTCCTTGAAC---------------------ATATATATATGTTCAT-------------ATCGTA----CTAT------ACAAAAAAA-----CAAA------------------TGAGATTGGA-TTGGAAGAA-----GAT------------------ACGAGGATTTAT------ATTTGGATCCATT------TGTGAAAGAACAGAG--TGAATGAAATGAGAAAG-------ATATTTCATTT--------------TGTTTAAACTGAGCCA------GAGCCACTGATGGAAAGAGGAT-------------------------------GAGGATAAATA-AAGA------------------GTGAGGAA----------GTAAAAT-----GGGC-TTTTTATTGGGGATA-----CCATCTAT---------AAATGGATAATACT--------TTTGTATTCATRTGAATTTTTGAAGATAG--CAAT---------CCCCCAAGATCT-TGT---------------WCTTAGAACAAGATCTT--GGGGGATTCTTTTG---CTTCTCTATCCG---------------AATTTTCG---------TTCTTTATCAT----AAAAGTTCTC-CCCCGCC---------AATGAATGATAAGT-GTC-----TAGGTG-AAGTAT----------------AGTATAAGATAAGTCAGA----------------------AAAGTATAAGTCTTA-----ATACCTATACTCTTA----------CTATAA-GAT------------AAAGACTCTTAAGGATAAGGC-----TTTTCAC----------ATGAATACTTAGTAGAACGACTAACGACGAGATTTATTATCGTTTCTCGCGTGTCTCACGAAAGTGAGAGTAG-GTGCGAATTCTCCCAATTTGTGACCGACCATACGATCTGTGATATAAATGGTAAA-TGTTCCTTTCCATTATGAATAGC-GATTGTATGGCCAATCATTGTGGGTA-TAATGGTAGATGCCCGAGACCAAGTCACTATGATTTCTTTCTCCTCCCTCCTGTT-GAGTTTTTCAATTCTTCCCGATAAATGATTAGCTACAAAAGGATTTTTTTTT-AGTGAACGTGTCAC-GGCTGATTACTCCTTTTTTTACATTTTT-TAAATTGGCATTCTATGTCCAA----------TATCTCGATCTTAA-TCTG-AAG-TAT---------------------------AATG-ATGAATGN-AAAAAATAGAAAATCC----------TTTA-GCTAG-ATAA--------RGG-AA-GGGGC-GGATGTAG-CCAAGTGG-ATCAN-GGCAGT

Anoectochilus_formosanus_PK12215 ATGACT-CTCGGCAATGGATATCTTGGCTCTTGCATCGATGAAGAGCGCAGCGAAATGCGATACGTGGTGTGAATTGCAGAATCCCGTGAACCATCAAATCTTTGAACGCAAGTTGCGCCTGAGGCCAAT-TGGCTAAGGGCACGTCCGCCTGGGCGTCA-AGCATTACATCGCTTCATAAGAATTATTTTTCTTCTCATTTTTCT------------TTTCAAATACTATCAGAAGGTTTTGGAGTCGTTCTGGAAATTCCATTATCGTCGCGATTAGTATTC---------TCCCTTGAAG---AAAAAAAAATACCAAAATATCAGAATTTACGATCTATTCATTCAATATTTCCTTTTTTAGAGGATAAATTTTCACATTTAAATTCTGTGTCAGATCTATTAATACCCCATCCCATCCATCTGGAAATCTTGGTTCAAATCCTTCAATGCTGGATCAAAGATGTTCCTTCTTTGCATTTGTTGCGATTTATTTTCCACGAATATCAT---------------AATTTGAAGAGTATC---------ATTACTT------CAAATAAATC---------------CATTCACGTTTTTTCAAAAAA---AAAGAAAAGAATTTTTTGGTTCCTACATAATTTTTATGTATATGAATGCGAATATCTCTTTCTTTTTCTTCGTAAAAATTCTTCTTATTTACGATCAACATCTTTTGGAGTCTTTATTGAGCGAACACTTTTTTATGTAAAAATGGAA------TCTATTCTAGT---AGTATATTTTAATTCTTTTCAGAGG-ATTCTCTGGTTCCTCAAAGATCCTTTCATACATTATGTTCGATATCAAGGAAAAGTAATTCTGACTTCAAAGGTAACTCTTATTCTGATGAAGAAATGGAATTTTCATGTTGTGAATTTTTGTCAATTTTATTTTCACTTTTGGTCTCAACCTTATAGGATCCATATAAAGCAATTACCCAACTATTCCTTCTCTTTTCTGGGGGTATTTTTTAAGTGTAC-AAAAAAAAACTTTGGTAGTAAGAAATCAAATGCTAGAGAATTCCTTTCTAATAAATACTATGACTAAGAAATTAGATACCGTAGCCCCAGTTATTTCTCTTATTGGATCATTGTCGAAAGCTCAATTTTGTACTATATCAGGTCATCCTATTAGTAAACCCATTTGGACTGATTTTTCGGATTTTGATATTATTGATCGATTTTGTCGGAAAAGTAGAAATCTTTGTCGTTATCCCAGCGGATCCTCAAAAAAAAAAGTTTTGTATCGTATAAAATATATATTTCGACTTTCGTGTGCTAGAACTTTGGCTCGTAAACATAAAAGTAC-AGTACGCACTTTTATGCGAAGATTGGGTTCGGTATTTTTAGAAGAATTTTTTATGGAAGAAGAACAAGTTCTTTCTTGAGCTATCCCGACCAGTACCC-TGCATCATCCTAGCAGAGTACTTG-TATCTATGTAAACGAAAAGAA-CTAAAAAAGAAAGTCTTAACAA-------------------ATTGGACCTAG-TCCCCTTTAATTT----------CTTAG-------ATA-------------------GAAAAC---TTTATTT-----------TTAAA-TTTAAAGATAATGATATGAACTGT-GATTTTTTAAATGAATTATTAAATAAG-GGAGATTCCTTGAAC-------------------------ATATATGTTCAT-TTGTGCAGGT--ATCGTA---TCTAT------ACAAAGAAAAA---CAAAG-----AAAA--------TTGGATTGGAATTGGAAGAA-----GATAGG---------------AGGAGGATTTCT------ATTCGAATCCTTT------TGTGAAAGAACAGAG--TGAATGAAATTAGAAAG-------ATATTGAATTT--------------TGTTTGAACT------------GAACAACTGATAAAAATAGGAT-------------------------------GAGAGTAAAGA-AAGA------------------GTGAGGAA----------TTAAAAT-----GGGC-TTTTTCTTGGGGATAGAGGACCATCTACTAC------AAATGGATAATACT--------TTTGTATTTAGATGAATTCTTTAAGGTAG--CAAT----------CCCCAATATC---------------------CAATATATTGGATATT---GGGGATTCTTTTG---CTTCTTTATCCG------ATTTCTCCAAATTTTCG---------TTCTTTATCATAAAAGAAAGTTCTC-CCCCGCC---------AATGAATGATAAGT-GCC-----TAGGTG-AAGTAT----------------AGTATAAGATAAGTAAGA----------------------AAAATCTAAGTCTTAGTATAATACCTATACTCTTA----------CTATAA-GAT------------AAAGACTCTTAAGGATAAGAC-----TTTTCAC----------ATGAATACTTAGTAGAACGACTAACGACGAGATTTATTATCATTTTTCGCATGTCTCACGAAAGTGAGAGTAG-GTGCGAATTCTCCCAATTTGTGACCGACCATACGATCTGTTATATAAATGGTAAA-TGTTCCTTTCCATTATGAATAGC-GATTGTATGGCCAATCATTGTGGGTA-TAATGGTAGATGCCCGAGACCAAGTCACTATTATTTCTTTCTCCTCCCTCATGTT-GAGTTTTTCAATTTTTACCGATAAATGATTAGCTACAAAAGGATTTTTTTTT-AGTGAACGTGTCAC-GGCCGATTACTCCTTTTTTTACATTTTT-TAAATTGGCATTCTATGTCCAA----------TATCTCGATCTTAA-TCTG-AAG-TAT-GAGGGTAAGAATCAATACAAT-----AATG-ATGAATGG-AAAAAATAGAAAATCC----------TTTA-GCTAG-ATAA--------GGG-AA-GGGGC-GGA---------------------------

Anoectochilus_roxburghii_SG1219 ATGACT-CTCGGCAATGGATATCTTGGCTCTTGCATCGATGAAGAGCGCAGCGAAATGCGATACGTGGTGTGAATTGCAGAATCCCGTGAACCATCAAATCTTTGAACGCAAGTTGCGCCTGAGGCCAAT-TGGCTAAGGGCACGTCCGCCTGGGCGTCA-AGCATTACATCGCTTCATAAGAATTATTTTTCTTCTCATTTTTCT------------TTTCAAATACTATCAGAAGGTTTTGGAGTCGTTCTGGAAATTCCATTATCGTCGCGATTAGTATTC---------TCCCTTGAAG---AAAAAAAAATACCAAAATATCAGAATTTACGATCTATTCATTCAATATTTCCTTTTTTAGAGGATAAAATTTCACATTTAAATTCTGTGTCAGATCTATTAATACCCCATCCCATCCATCTGGAAATCTTGGTTCAAATCCTTCAATGCTGGATCAAAGATGTTCCTTCTTTGCATTTGTTGCGATTTATTTTCCACGAATATCAT---------------AATTTGAAGAGTATC---------ATTACTT------CAAATAAATC---------------CATTCACGTTTTTTCAAAAAA---AAAGAAAAGAATTTTTTGGTTCCTACATAATTTTTATGTATATGAATGCGAATATCTCTTTCTTTTTCTTCGTAAAAATTCTTCTTATTTACGATCAACATCTTTTGGAGTCTTTATTGAGCGAACACTTTTTTATGTAAAAATGGAA------TCTATTCTAGT---AGTATATTTTAATTCTTTTCAGAGG-ATTCTCTGGTTCCTCAAAGATCCTTTCATACATTATGTTCGATATCAAGGAAAAGTAATTCTGACTTCAAAGGTAACTCTTATTCTGATGAAGAAATGGAATTTTCATGTTGTGAATTTTTGTCAATTTTATTTTCACTTTTGGTCTCAACCTTATAGGATCCATATAAAGCAATTACCCAACTATTCCTTCTCTTTTCT-GGGGTATTTTTTAAGTGTAC-AAAAAAAAACTTTGGTAGTAAGAAATCAAATGCTAGAGAATTCCTTTCTAATAAATACTATGACTAAGAAATTAGATACCATAGCCCCAGTTATTTCTCTTATTGGATCATTGTCGAAAGCTCAATTTTGTACTATATCAGGTCATCCTATTAGTAAACCCATTTGGACTGATTTTTCGGATTCTGATATTATTGATCAATTTTGTCGGAAATGTAGAAATCTTTGTCGTTATCACAGCGGATCCTCAAAAAAAAAAGTTTTGTATCGTATAAAATATATATTTCGACTTTCGTGTGCTAGAACTTTGGCTCGTAAACATAAAAGTAC-AGTACGCACTTTTATGCGAAGATTGGGTTCGGTATTTTTAGAAGAATTTTTTATGGAAGAAGAACAAGTTCTTTCTTGAGCTATCCCGACCAGTACCC-TGCATCATCCTAGCAGAGTACTTG-TATCTATGTAAACGAAAAGAA-CTAAAAAAGAAAGTCTTAACAA-------------------ATTGGACCTAG-TCCCCTTTAATTT----------CTTAG-------ATA-------------------GAAAAC---TTTCTTT-----------TTAAA-TTTAAAGATAATGATATGAACTGT-GATTTTTTAAATGAATTATTAAATAAG-GGAGATTCCTTGAAC-------------------------ATATATGTTCAT-TTGTGCAGGT--ATCGTA---TCTAT------ACAAAGAAAAA---CAAAG-----AAAA--------TTGGATTGGAATTGGAAGAA-----GATAGG---------------AGGAGGATTTCT------ATTCGAATCCTTT------TGTGAAAGAACAGAG--TGAATGAAATTAGAAAG-------ATATTGAATTT--------------TGTTTGAACT------------GAACAACTGATAAAAATAGGAT-------------------------------GAGAGTAAAGA-AAGA------------------GTGAGGAA----------TTAAAAT-----GGGC-TTTTTCTTGGGGATAGAGGACCATCTACTAC------AAATGGATAATACT--------TTTGTATTTAGATGAATTCTTTAAGGTAG--TAAT----------CCCCAATATAT-T---------------------------GGATATT---GGGGATTCTTTTG---CTTCTTTATCCG------ATTTCTCCAAATTTTCG---------TTCTTTATCATAAAAGAAAGTTCTC-CCCCGCC---------AATGAATGATAAGT-GCC-----TAGGTG-AAGTAT----------------AGTATAAGATAAGTAAGA----------------------AAAATCTAAGTCTTAGTATAATACCTATACTCTTA----------CTATAA-GAT------------AAAGACTCTTAAGGATAAGAC-----TTTTCAC----------ATGAATACTTAGTAGAACGACTAACGACGAGATTTATTATCATTTCTCGCATGTCTCACGAAAGTGAGAGTAG-GTGCGAATTCTCCCAATTTGTGACCGACCATACGATCTGTTATATAAATGGTAAA-TGTTCCTTTCCATTATGAATAGC-GATTGTATGGCCAATCATTGTGGGTA-TAATGGTAGATGCCCGAGACCAAGTCACTATTATTTCTTTCTCCTCCCTCATGTT-GAGTTTTTCAATTTTTACCGATAAATGATTAGCTACAAAAGGATTTTTTTTT-AGTGAACGTGTCAC-GGCCGATTACTCCTTTTTTTACATTTTT-TAAATTGGCATTCTATGTCCAA----------TATCTCGATCTTAA-TCTG-AAG-TAT-GAGGGTAAGAATCAATACAAT-----AATG-ATGAATGG-AAAAAATAGAAAATCC----------TTTA-GCTAG-ATAA--------GGG-AA-GGGGC-GGATGTAG-CCAAGTGG-ATCAA-GGCAGT

Aphyllorchis_montana_SG1010 ATGACT-CTCGGCAACGGATATCTCGGCTCTCGCATCGATGAAGAGCGCAGCGAAATGCGATACGTGGTGCGAATTGCAGAATCCCGTGAACCATCAAGTCTTTGAACGCAAGTTGCGCCCGAGGCCAAT-CGGCCAAGGGCACGTCTGCCTGGGCGTCA-AGCGTTGCGTCGCTTCACAAGAATTCCTTTTCTTCTCATTTTTCT------------TCTCAAATGGTATCAGAAGGTTTTGGAATCATTCTGGAAATTCCATTCTCGTCGCGATTAGTATCT---------TCCCTTGAAG---AAAAAAAAAGACCAAAATCTCAGAATTTACGATCTATTCATTCAATATTTCCCTTTTTAGAGGATAAATTCTCGCATTTAAATTATGTGTCAGATCTAATAATACCCCATCCCCTCCATCTGGAAATCTTGGTTCAAATCCTTCAATGCTGGATCAAAGATGTTCCTTCTTTGCATTTATTGCGATCTTTTTTCCACGAATATCAT---------------AATTTGAATAGTCTC---------ATTACTT------CAAAGAAATC---------------CATTCATGTCTTTTCGAAAAG---AAAGAAAAGATTCTTTTGGTTCCTACATAATTCTTATGTATATGAATGCGAATATATATTCCTTTTTCTTCGTAAAGAGTCTTCTTATTTACGATCAACATCTTCTGGAGTCTTTCTTGAGCGAACACATTTCTATGGAAAAATAGAA------TATCTTCTAGT---AGTGTGTTTTAATTCTTTTCGGAGG-ATTCTATGGTTCCTCAAAGATCCTTTCATACATTATGCTCGATATCAAGGAAAAGCAATTATGGCTTCAAAGGGAACTCTTATTCTGATGAAGAAATGGAAATTTCATCTTGTGAATCTTTGGCAATCTTATTTTCACTTTTGGTCTCAACCTTATAGGATCCATATAAAGCAATTACCCAACTCTTCCTTCTCTTTTCT-GGGGTATTTTTCAAGTGTAC-TAAAAAATACTTTGGTAGTAAGAAATCAAATGCTAGAGAATTCATTTCTAATAAATACTCTGACTAAAAAATTCGATACCATAGCCCCAGTTATTTCTCTTATTGGATCATTGTCGAAAGCTCAATTTTGTACTGTATTGGGTCATCCTATTAGTAAGCCGATCTGGACCGATTTATCGGATTCTGATATTCTTGATCGATTTTGTCGGATATGTAGAAATCTTTGTCGTTATCACAGCGGATCCTCAAAGAAACAGGTTTTGTATCGTATAAAGTATATACTTCGACTTTCGTGTGCTAGAACTTTGGCTCGTAAACATAAAAGTAC-AGTACGCACTTTTATGCGAAGATTAGGTTCGGGATTCTTAGAAGAATTTTTTTTGGAAGAAGAAAAAGTTCTTTCTTGAGCTATCCCGACCATTTCCCGTGCATCATCCTAGCAAAGTACTTG-TATCTATGTCAATGAAAAGAA-CTAAAAAAG----TCTTAACAA-------------------ATGGGACCTAG-CCCCCA-GAATTT----------CTTAG-------GTC-TTCAAAAA----------GAAGAC---TTTATTT-----------GTAAA-TGTAAGGATAGTGATATGGACTGT-GAATGA------------TTCAATAAC-GGAGATTCCTTGAAA-------------------------ATATATGTTCAT-TTGTACAGAT--ATCGTA----CTCT------ATAAAT------------------GAAA--------TTGGATTGGAA---GAAGAT--------------------------ACGAGGATTTCT------GTTTGGATCCAGT------TGTGAAAGAACAGAG--TGAATGAAATGAGAAAG-------ATATTGAATTT--------------TGTTTGAACT------------AAACCACTGATGAAAAAAGGAT-------------------------------GAGGATAAATA-AAGA------------------GTGAGGAA----------GTAAAAT-----GGGC-TTTTTCTTGGGGATAGAGGGCCATCTAT---------AAATGGATAATACT--------TTTGTATTCATATGAATTTATGAAGGTAG--CAAT---------CCTTC-ATATCT-TCT---------------TCAAAGAACAAGATATT-GGTGAGATTTGTTTG---CTTCTTTATACG---------------AATTTTTG---------TTCTTTATCATAAAAGAAAGTTTTT-CCCCGCC---------AATGAATGATAAGT-GTC-----TGGGTG-AAGTAT----------------ATTATAATATAAGTAAGA----------------------AAAGTATATGTCTTAGA---ACACCTAGATTCTTACTATAAGTTACTATAA-GATAAAGTATAAGATAAAGATTCTTAAGGATAAAGA-----TTTTCAC----------ATGAATTCTTAGTAGAACGCCTAATGACGAGATTTATTATCTTTTCTCTTGTGTCTCACGAAAGTGAGAGTAGGGTGCGGATTCTCCCAATTTGTGATCGACCACACAATCTGTGATATAAATGGTAAATTAGTTGTTTTCATTATGAATAATGGATTGTATGTCCAATCATTATGGGTATAATTGGTAGATTCCTGAGACCAAGTCACTATTATTTCT--CTCTTCCCTCATGTTTGATTTTTTCAATTCATCCCAATAAATTATTACCTAAAGAAG-ATTTTTTTT--ATTGAACGTGTCAC-GGCTAGTTGCTCCTTTTTTTACATTTTT-GAAATTGGCATTCTAAGTCCAA----------TATCTCGATCATAA-TCTA-AAA-TAT-GAAGGTAAGAATCAATACAAA-----AATG-ATGAGTGA-AAAAAAGAGAAAATCC----------TTCA-GCTAG-ATAA--------GGT-AA-GTGGC-GGATGTAG-CCAAGTGG-ATCAA-GGCATT

Apostasia_nipponica_PK12273 AAGACT-CTCGGCAACGGATATCTCGGCTCTCGCATCGATGAAGAACGCAGCGAACCGCGATACGTGGTGTGAATTGCAGAATCCCGCGAATCATCGAGTCTTTGAACGCAAGTTGCGCCCGAGGCCAAG-TGGACGAGGGCACGCCTGCCTGGTCGTCA-GGCGCTGCGTCGCTCCACAAGAATTATTTTTCTTCTAATTTTGCT------------TTTCAAATGGCATCAGAAGGTTTTGGAGTCATTCTGGAAATTCCATTCTCGTCGCGATTAGTTTCT---------TCCCCTGAAG---AAATAAAAATACCAAAATCTCTGAATTTACGATCTATTCATTCAATATTTCCTTTTTTAGAGGACAAATTCTTACATTTAAATTATGTATTAGGTATACTAATACCCCACCCCATCCATCTGGGAATCTTGGTTCAAATCCTTCAATGCTGGATCAAAGATGCTCCTTCTTTGCATTTATTGTTAAAAATTTTTCACGAATATTAT---------------AATTCGAATAGTCTT---------TTTACTT------CAAA---------------------------------------------AAAAAGAAGA-TTTTTGGCTTCTTATATAATTCTTATATATGTGAATGCGAATTTCTTTTACTATTTTTTCGTAAACAGTCTTCCTACTTACGATCAACATCTTCTGGAGTTTTTCTTGAACGAATGCATTTCTATGGAAAAATAGAG------TATCTTGTAGTA------TGTTGTAATTCTTTTCAAAGG-ATCCCATGCTTCTTCAAAAATCTTTTCATGCATTATGTTCGATATCAAGGAAAAGGAATTCTGGCTTCAAAGGGTACTCTTATTCTGATGAAAAAATGGAAATATCATCTTGTAAATTTTTGGCAATCTTATTTTCGCTTTTGGTCTCAACCATATAGGATACATATAAAACAATTATTCAACTATTCCTTTTCGTTTAT-GGGGTATTTTTCAAGTGTAC-TAAGATATTCTTTGGTAGTAAGAAATCAAATGCTAGAGAATTCATTTCTCATGGATATTCTGATTAAGAAATTAGATACCATAGTTCCAGTTGTTTCTCTTATTGGATCAATGTCGAAAGCCCAATTTTGTACTGTATTGGGTCATCCTATTAGTAAACCGATTTGGACTGATTTTTCAGATTCTGATATTCTTGATCGATTTTGTAGAATATGTAAAAATCTTTGTCGTTTTTACAGTGGATCCTCAAAAAAACATGTTTTGTATCATATAAAATATATACTTCGACTTTCGTGTGCTAGAACTTTAGCTCGTAAACATAAAAGTAC-AGTACGTACTTTGATGCGAAGATTAGGTTCGGGATTCTTAGAAAAATTCTTTATGGAAAAAGAACAAGTTCTTTCTTGAGCTATCCCGACCATTTCTTCTGCACCATCCTACAAGAATATTTG-TATCTATGTCAATGAAAAGAA-CTAAAAAA----ATCTTAACAA-------------------ATTGGACCTAG--CCCCT-GAATTT----------CTTAG-------ATC-TTCAAAAA----------GAAGAC---TTACTTT-----------GGAAA-------TGAAAAGATATGTACTGT-GAAAGA------------GTCAATAAC-TCAATAACGTGGATTCCTTGAAC-----------------ATATATGTTCAT-TTGTACAGAT--ATCGTA---TCTAT------ACAAAT------------------GAAA--------TTGGATTGGAA------GAA-----GAT------------------AGGAGGAAT-----------------CCATT------GTTGAAAAAACAGAG--TGAATGAAATAAGAAAG-------ATATTGAATTT--------------TGTTTGAACT------------GACCTACTGATGAAAAAAAAG--------------------------------TAGG--------AATA------------------GGGAGGAA----------GTAAAAT-----GGGC-TTTTTCTTGGGGATAGAGGA----------------------------------------------------------------------------------------------------------------------------------------------------------------------------------------------------------------------------------------------------------------------------------------------------------------------------------------------------------------------------------------------------------------------------------------------------------------------------------------------------------------------------------------------------------------------------------------------------------------------------------------------------------------------------------------------------------------------------------------------------------------------------------------------------------------------------------------------------------------------------------------------------------------------------------------------------------------------------------------------------

Appendicula_cornuta_PK12065 ATGACT-CTCGGCAATGGATATCTCGGCTCTCGCATCGATGAAGAGCGCAGCGAAATGCGATACGTGGTGTGAATTGCAGAATCCCGCGAACCATCGAGTCTTTGAACGCAAGTTGCGCCCGAGGCCAAC-CGGCTGAGGGCACGTCTGCCTGGGCGTCA-AACGTTTCGTCGCTTCACAAGAATTCTTTTTCT------------------------TCTCAAATGGTATCAGAAGGTTTTGGAGTCATTCTGGAAATTCCATTCTCGTCGCGATTAGTATCT---------TTCCTTGAAG---AAAAAAGAATACCAAAATATCAGAATTTACGATCTATTCATTCAATATTTCCCTTTTTAGAGGATAAATTATTACATTTAAATTATGTGTCAGATCTACTAATACCCCATCCCATCCATCTGGAAATATTGGTTCAAATTCTTCAATGCTGGATCAAAGATGTTCCTTCTTTGCATTTATTGCGATTGTTTTTTCACGAATATCAT---------------AATTTGAATAGTCTC---------ATTATTT------CAAAGAAATC---------------CATTTACGTCTTTTCAAAAAG---AAAGAAAAGATTATTTTTGTTCCTACATAATTCCTATGTATATGAATGCGAATATCTATTCCTGTTTCTTCGTAAACAGTCTTCTTATTTACGATCAATATCTTCTGGAGTCTTTCTTGAGCGAACACATTTCTATGTAAAAATAGAA------TATCTTATAGC---CGTATATTGTAATTCTTTTCATAGG-ATCCTATGGTTCCTCAAAGATACTTTCATAAATTATGTTCGATATCAAGGAAAAGCGATTCTGGCTTCAAAAGGAACTCTTATTCTGATGAATAAATGGAAATTTCATCTTGTGAATCTTTGGCAATCTTATTTTCACTTTTGGTTTCAACCTTATAGGATCCATATAAAGCAATTACCCAACTATTCTTTCTCTTTTCT-GGGGTATTTTTCAAGTGTAC-TAAAAAATCCTTTGGTAGTAAGAAATCAAATGTTAGATAATTCATTTCTAATAAATACTCTATCTAAGAAATTAGATACCATAGTCCCAGTTATTTCTCTTATAGGATCATTGTCGAAAGCTCAATTTTGTACTGTATTGGGTCATCCTATTAGTAAACCGATCTGGACCGATTTATCGGATTCTGATATTCTTGATCGATTTTGTCGAATATGTAGAGATCTTTGTCGTTATCACAGCGGATCCTCAAAGAAACAGGTTTTGTATCGTATAAAGTATATACTTCGACTTTCGTGTGCTAGAACTTTGGCTCGTAAACATAAAAGTAC-AGTACGCACTTTTATGCGAAGATTAGGTTCGGGATTCTTAGAAGAATTCTTTTTGGAAGAAGAACAATCTCTTTCTT--GCTATCCCGACCATTTCCCGTGCATCATCCTAGCAGAGTACTTC-TATCTATGTCAATGAAAAGAA-CTAAAAAATAACATCTTAACAA-------------------ATTGG-CCTAG--CCCCT-GAATTT----------CTTAG-------ATC-TTCAAAAAAAAAAAAAAAA-----------------------------------------------------------------------------------------------------------------------------------------------------------------------------------------------------------------------------------------------------------------------------------------------------------------------------------------------------------------------------------------------------GAAATAGGAT-------------------------------GAGGATAAAT------------------------------------------------------------------------------------------------AAATGGATAATACT--------TTTGTATTCATATGAATTTTTGAAGATAG--CAAT---------CCCCCAATCCCC---------------------------CAAGATATT--GGGGGATTCTTTTG---CTTCTCTATCCG---------------AATTTTCG---------TTCTTTATCAT----AAAAGTTCTC-CCCCGCC---------AATGAATGATAAGT-GCC-----TAGGTG-AAGTAT----------------AGTATAAGATAAGTCAGA----------------------AAAGTCTAAGTCTTA-----ATACCTATACTCTTA----------CTATAA-GAT------------AAAGACTCTTAA-GATAAGGC-----TTTTCAC----------ATGAATACTTAGTAGAACGACTAACGACGAGATTTATTATCGTTTCTCGCGTGTCTCACGAAAGTTAGAGTAG-GTGCAAATTCTCCCAATTTGTGACCGACCATACGATCTGTGATATAAATGGTAAA-TGTTCCTTTCCATTATGAATAGC-GATTGTATGGCCAATCATTGTGGGTA-TAATGGTAGATGCCCGAGACCAAGTCACTATGATTTCTTTCTCCTCCCTCCTGTT-GAGTTTTTCAATTCTTCCCGATAAATGATTAGCTACAAAAGGATTTTTTTTT-AGTGAACGTGTCAC-GGCTGATTACTCCTTTTTTTACATTTTT-GAAATTGGCATTCTATGTCCAA----------TATCTCGATCTTAA-TCTG-AAG-TAT---------------------------AATG-ATGGATGG-AAAAAAGAGAAAATCC----------TTTA-GCTAG-ATAA--------GGG-AA-GGGGC-GGATGTAG-CCAAGTGG-ATCAA-GGCAGT

Arundina_graminifolia_SG1295 ACGACT-CTCGGCAATGGATATCTCGGCTCTCGCATCGATGAAGAGCGCAGCGAAATGCGATACGTGGTGCGAATTGCAGAATCCCGCGAACCATCGAGTCTTTGAACGCAAGTTGCGCCTGAGGCCAAC-CGGCCGAGGGCACGTCTGCCTGGGCGTCA-GGCGTTACGTCGCTCC---AGAATTCTTTTTCTTCTCATTTTTCT------------TCTCAAATGGTATCAGAAGGTTTTGGAGTCATTCTGGAAATTCCATTCTCGTCGCAATTAGTATCT---------TCCCTTGAAG---ATAACAGAATACCAAAATTTCAGAATTTACTATCTATTCATTCAATATTTCCCTTTTTAGAGGATAAATTATCACATTTCAATTATGTGTCAGATCTACTAATACCCCATCCCATCCATCTGGAAATCTTGGTTCAAATCCTTCAATGCTGGATTAAAGATGTTTCTTCTTTGCATTTCTTGCGATTGTTTTTCCACGAATATCAT---------------AATTTGAATAGTCTC---------ATTACTT------CAAATAAATC---------------CATTTACGTCTTTTCAAAAAG---AACCAAAAGATTCTTTTGGTTCCTACATAACTCTTATGTATATGAATGCGAATATATATTCCTGTTTCTTCGTAAACAGTCTTCTTATTTACGATCAATATCTTCTGGAGTCTTTCTTGAGCGAACACATTTCTATGGAAAAATAGAA------TATCTTATAGT---CGTGTGTTGTAATTCTTTTCAGAGG-ATCCTATGGTTCCTCAAAGATACTTTCATACATTATGTTCGATATCAAGGAAAAACAATTCTGGTTTCAAAAGGAACTCTTATTCTGATTAAGAAATGGAAATTTCATCTTGTGAATTTTTGGCAATCTTATTTTCACTTTTGGTTTCAACCTTATAGGATTCATATAAAGCAATTACCCAACTATTCCTTCTCTTTTCT-GGGGTATTTTTCAAGTGTAC-TAAAAAATAATTTGGTAATAAGAAATCAAATGCTAGAGAATTCATTTCTAATAAATACTCTGACTAAGAAATTAGATACCATAGCCCCAGTTACTTCTATTATTGGATCATTGTCGAAAGCTCAATTTTGTACTGTATTGGGTCATCCTATTAGTAAACCGATCTGGACCGATTTATCGGATTCTGATATTTTTGATCGATTTTGTCGGATATGTAGAAATCTTTGTCGTTATCACAGCGGATCTTCAAAGAAACAGGTTTTGTATCGTATAAAGTATATACTTCGACTTTCGTGTGCTAGAACTTTGGCTCGTAAACATAAAAGTAC-AGTACGCACTTTTATGCGAAGATTAGGTTCGGGATTCTTAGAAGAATTTTTTTTTGAAGAAGAACAATCTCTTTCTTGAGCTATCCCGACCATTTTCCGTGCATCATCCTAGCAGAGTACTTA-TATCTATGTCAATGAAAAGAA-CTCAAAAAGAAAATCTTAACAA-------------------ATTGGACCTAG--CCCTT-GAATTT----------CTTAG-------ATC-TTCAAAAA----------GAAGAC---TTTATTT-----------GTAAA-TGTAAGGAAAAATATATGGACTAT-GAATGA------------TTTAATAAC-GGAGATTCTTTGAAC-------------------------ATATATGTTCAT-------------ATCGTA----TTAT------ACAAAA--------CAAAT-----GAGA--------TTGTAGTGGAA------GAA-----GAT------------------ACGAGGATTTCT------ATTCGGATCCATT------TGTGAAAGAACAGAG--TGAATGAAATGAGAAAG-------ATATTTCATTT--------------TGTTTGAACT------------GAGCCACTGATGGAAAGAGGAT-------------------------------GAGGATAAATA-AAGA------------------GCGAGGAA----------GTAAAAT-----GGGC-TTTTTATTGGGGATAGAGGGCCATCTAT---------AAATGGATAATACT--------TTTGTATACATATGAATTTTTGAATATAG--CAAT---------CCCCCAATATCT-TGT---------------TCTAAGAACAAGATATT--GGGGGATTCGTTTG---CTTCTCTA---------------------TTTTCG---------TTTTTTATCAT----AAAAGTTCTC-CCCCGCC---------AATGAATGATAAGT-GCC-----TAGGTG-AAGTAT----------------AGTATAAGATAAGTCAGA----------------------AAAGTCTAAGTCTTAGTATAATACCTATACTCTTA----------CTATAA-GAT------------AAAGACTCTTAAGGATAAGGC-----TTTTCAC----------ATGAATACTTAGTAGAACGACTAACGACGAGATTTATTATCGTTTCTCGCGTGTCTCACGAAAGTGAGAGTAG-GTGCGAATTCTCCCAATTTGTGACCGACCATACGATCTGTGATATAAATGGTAAA-TGTTCCTTTCCATTATGAATAGC-GATTGTATGGCCAATCATTGTGGGTA-TAATGGTAGATGCCCGAGACCAAGTCACTATGATTTCTTTCTCCTCCCTCCTGTT-GAGTTTTTCAATTCTTCCCGATAAATGATTAGCTACAAAAGGATTTTTTTTT-AGTGAACGTGTCAC-GGCTGATTACTCCTTTTTTTACATTTTT-GAAATTGGCATTCTATGTCCAA----------TATCTCGATCTTAA-TCTG-AAG-TAT---------------------------AATG-ATGAATGG-AAAAAAGAGAAAATCC----------TTTA-GCTAG-ATAA--------GGG-AA-GGGGC-GGATGTAG-CCAAGTGG-ATCAA-GGCAGT

Bletilla_striata_KFBG2048 ACGACT-CTCGGCAATGGATATCTCGGCTCTCGCATCGATGAAGAGCGCAGCGAAATGCGATACGTGGTGCGAATTGCAGAATCCCGCGAACCATCGAGTCTTTGAACGCAAGTTGCGCCCGAGGCCAAC-CGGCCGAGGGCACGTCCGCCTGGGCGTCA-AGCGTCGCGTCGCTCCACAAGAATTCTTTTTCTTCTCATTTTTCT------------TCTCAAATGGTATCAGAAGGTTTTGGAGTCATTCTGGAAATTCCATTCTCGTCGCGATTAGTATCT---------TCCCTTGAAG---AAAAAAGAATACCAAAATCTCAGAATTTACGATCTATTCATTCAATATTTCCCTTTTTAGAGGATAAATTATCACATTTAAATTATGTGTCAGATCTACTAATACCCCATCCCATACATCTGGAAATCTTGGTTCAAATCCTTCAATGCTGGATCAAAGATGTTTCTTCTTTGCATTTCTTGCGATTGTTTTTCCATGAATATCAT---------------AATTTGAATAGTCTC---------ATTACTT------CAAATAAATC---------------CACTTACGTCTTTTCAAAAAG---AAAGAAAAGATTCTTTTGGTTCCTACATAATTCTTATGTATATGAATGCGAATATCTATTCCTGTTTCTTCGTAAAAAGTCTTCTTATTTACGATCAATATCTTCTGGAGTTTTTCTTGAGCGAACACATTTCTATGGAAAAATAGAA------TATCTTATAGT---CGTGTGTTGTAATTCTTTTCAGAGT-ATCCTATGGTTCCTCAAAGATACTTTCATACATTATGTTCGATATCAAGGAAAAGCAATTCTGGCTTCAAAAGGAACTCTTATTCTGATGAAGAAATGGAAATTTTATCTTGTGAATTTTTGGCAATCTTATTTTCACTTTTGGTTTCAACCTTATAGGATCTATATAAAGCAATTACCCAACTATTCCTTCTCTTTTCT-GGGATATTTTTCAAGTGTAC-TAAAAAATCCTTTGGTAGTAAGAAATCAAATGCTAGATAATTCATTTCTAATAAATACTCTGACTAAGAAATTAGATACCGTAGCCCCAGTTATTTCTCTTATTGGATCATTGTCGAAAGCTCAATTTTGTACTGTATTGGGTCATCCTATTAGTAAACCGATCTGGACCGATTTATCGGATTCTGATATTCTTGATCGATTTTGTCGGATATGTAGAAATCTTTGTCGTTATCACAGCGGATCCTCAAAGAAACAGGTTTTGTATCGTATAAAGTATATTCTTCGACTTTCGTGTGCTAGAACTTTGGCTCGTAAACATAAAAGTAC-AGTACGCACTTTTATGCGAAGATTAGGTTCGGGATTCTTAGAAGAATATTTT-CGGAAGA-----------------GAGCTATCCCGACCATTTCCTGTGCATCATCCTAGCAGAGTACTTA-TATCTATGTCAATGAAAAGAA-CTAAAAAAGAAAATCTTAACAA-------------------ATTGGACCTAG--CCCCT-GAATTT----------CTTAG-------ATC-TTCAAAAA----------GAAGAC---TTTCTTT-----------GTAAA-TGTAAGGAAAAATATATGGACTAT-GAATGA------------TTCAATAAC-GGAGATTCCTTGAAC-------------------------ATATATGTTCAT-------------ATCGTA----CTAT------ACAAAA--------CAAAT-----GAGA--------TTGGATTGGAA------GAA-----GAT------------------ACGAGGATTTCT------ATTCGAATCCATT------TGTGAAAGAACAGAG--TGAATGAAATGAGAAAG-------ATATTTCATTT--------------TGTTTGAGCT------------GAGCCACTGATGGAAAGAGGAT-------------------------------GAGGATAAATA-AAGA------------------GCGAGGAA------------------------------GTCTCGG----------CCATCTAT---------AAATGGATAAGACT--------TTTGTATACATATGAATTCTTGAAGATAG--CAAT---------CCCCCAATATCT-TGT---------------TCTAAGAACAAGATATT--GGGGGATTCGTTTG---CTTCTCTATCCG---------------AATTTTCG---------TTCTTTATCAT----AAAAGTTCTC-CCCCGCC---------AATGAATGATAAGT-GCT-----TAGGTG-AAGTAT----------------AGTATAAGATAAGTCAGA----------------------AAAGTCTAAGTCTTAGTATAATACCTATACTCTTA----------CTATAA-GAT------------AAAGACTCTTAAGGATAAGGC-----TTTTCAC----------ATGAATACTTAGTAGAACGACTAACGACGAGATTTATTATCGTTTCTCGCGTGTCTCACGAAAGTGAGAGTAG-GTGCGAATTCTCCCAATTTGTGACCGACCATACGATCTGTGATATAAATGGTAAA-TGTTCCTTTCCATTATGAATAGC-GATTGTATGGCCAATCATTGTGGGTA-TAATGGTAGATGCCCGAGACCAAGTCACTATGATTTCTTTCTCCTCCCTCCTGTT-GAGTTTTTCAATTCTTCCCGATAAATGATTAGCTACAAAAGGATTTTTTTTT-AGTGAACGTGTCAC-GGCTGATTACTCCTTTTTTTACATTTTT-GAAATTGGCATTCTATGTCCAA----------TATCTCGATCTTAA-TCTG-AAG-TAT---------------------------AATG-ATGAATGG-AAAAAATAGAAAATCC----------TTTA-GCTAG-ATAA--------GGG-AA-GGGGC-GGA---------------------------

Brachycorythis_galeandra_SG1261 AGGGCT-CTCGGCAATGGATATCTTGGCTCTCGCATCGATGAAGAGCGCAACGAAATGCGATACGTGGTGCGAATTGCAGAATCCCGTGAACCATCGAGTTTTTGAACGCAAGTTGCGCCTGAGGCCAGC-TGGCCAAAGGCACGTCCGCCTGGGCGTCA-AGCATTGAATCGCTCCATAAGAATTATTTTTCT------------------------TCTCAAATATTATCAGAAGGTTTTGGAGTTATTCTGGAAATTCCATTCTCGTCGCGATTAGTATCT---------TCCCTTGAAG---AAAAAAAAATACCAAAATCTCAGAATTTACGATCTATTCATTCAATCTTTCCTTTTTTAGAAGATAAATTCTCACATTTAAATTTTGTGTCAAATCTACTAATACCCCATCCCATCCATATGGAAATCTTAGTTCAAATCCTTCAATGCTGGATCAAAGATGTTCCTTCTTTGCATTTGTTGCGATTGATTTTCCATGAATATCAT---------------AATTTAAATAGTCTC---------ATTACTT------CAAAGAAAGA---------------GATTGACGTCTTTTCAAAAAT---AAATAAAAGATTTTTTTGGTTCTTACATAATTCTTATGTATATGAATGCGAATATATATTCCTGTTTCTTCGCAAACAGTCTTCTTATTTACGATCAACATCTTTTGAAGTCTTTCTTGAGCGAACACATTTCTATGAAAAAATAGAA------TATTTTAGAGT---AATGTATTGTAATTCTTTTCAGAGG-ATTCTATGGTTCCTCAAAGAACCTTTCATACATTATGTTCGATATCAAGGAAAAGCAATTCTGGCTTCAAAGGGAACTCTAATTCTGATGAACAAATGGAAATTTCATCTTGTTCATTTTTGGCAATTTTATTTTCATTTTTGGTCTCAACCTTCTAGGATCCATATAAAGGAATTACCCAATTATTCCTTCTCTTTTCT-GGGGTATTTTTTAAGTGTAC-TAAAAAAGACTTTGGTAATAAGAAATCAAATGCTGGAGAATTCATTTTTAATAAATACTCTGACTAAGAAATTAGATACCATAGCCCCAGTTATTTCTCTTATTGGAGCATTGTCAAAAGCTCAATTTTGTACTGTATTGGGCCATCCTATTAGTAAACCAATCTGGACCGATTTATCGGATTCTGATATTCTTGATCGATTTTGTCGGATATGTAGAAATATTTGTCGTTATCACAGCGGATCCTCAAAAAAACAGGTTTTATATCGTATAAAGTATATACTTCGACTTTCGTGTGCTAAAACTTTGGCTCGTAAACATAAAAGTAC-AGTACGCACTTTTATACGAAGATTAGGTTCGGGATTCTTAGAAGAATTTTTTATGGAAGAAGAACAAGCTCTTTATTGAGCTATCCCGACCATTTTCCGTGCATCATCCTAGCAGAGTA------------GTCAATGAAAAGAA-CTAAAAAAGATAGTCTTAACAA-------------------ATTGGACCTA-------CTTAATTT----------CTTAG-------ATC-TTCAAAAA----------GAAGAT---TTTCTTT-------------------------------------------------------------------------GATTCCTTGAAC-------------------------ATATATGTTCAT-TTGTGCAGGT--ATCGTA---TCTAT------ACAAAT------------------GAAA--------TTGGATTGCAATTGGAAGAA-----TAT------------------CCGAAAATTTCT------ATTCGGATCCATT------TGTGAAAGAACAGAG-----------------------------------------------------------------------------------------------------------------------------------------------------------------TGAGGAA----------GTAAAAT-----GGGC-TTTTTATTGGGGATAGAGGGCCATCTAC---------AAATGGATAGTACT----------------CATATGATTTATTGAAGATAT--AAAT---------CCCCCAATATCT-TGT---------------TCTTAGAACAAGATATT--GGGGGATT---------TTTCTC----CG---------------ACTTTTCG---------TTTTTTATCATAAAAGAAAGTTATC-CCCCGCT---------AATGAATGATAAGT-GTC-----TAGGTG-AAGTAT----------------AGTATAAGATAAGTAAGA----------------------AAAGTCTAAGTCTTAGTATAATACCTATACTCTTA----------CTCTAA-GAT------------AAAGACTCTTAAGGATAAGAC-----TTTTCAC----------ATGAATACTTAGCAGAACGACTAACGATGAGATTTATTATCGTTTCTCGCATGTCTCACGAAAGTGAGAGTAG-GTGCAAATTCTCCCAATTTGTGACCAACCATACGATCTGTTATATAAATGGTAAA-TGTTCCTTTCCATTATGAATAGC-GATTGTATGGCCAATCATTGTGGGTA-TAATGGTAGATGCCCGAGACCAAGTCACTATTATTTCTTTCTCCTCCCTCATGTT-GAGTTTTTCAATTTTTCCCGATAAATGATTAGCTACAAAAGGATTTTTTTTT-AGTGAACGTGTCAC-GGCTGATTACTCCTTTTTTTACATTTTT-TAAATTGGCATTCTATGTCCAA----------TATCTCGATCTTAA-TCTG-AAG-TAT-GAGGGTAAGAATCAATACAAT-----AATG-ATGAATGG-AAAAAAGAGAAAATCT----------TTTA-GCTAG-ATAA--------GGG-AA-GGGGC-GGATGTAG-CCAAGTGG-ATCAA-GGCAGT

Bulbophyllum_affine_SG1606 ACGACT-CTCGGCAATGGATATCTCGGCTCTCGCATCGATGAAGAGCGCAGCGAAATGCGATACGTGGTGCGAATTGCAGAATCCCGCGAACCATCGAGTCTTTGAACGCAAGTTGCGCCCGAGGCCAAC-CGGCCAAGGGCACGTCCGCCTGGGCGTCA-AGCGTTGCGTCACTCC-CAAGAATTCTTTTTCTTCTCATTTTTCT------------TCTCAAATGCTATCAGAAGGTTTTGGAGTCATTCTGGAAATTCCATTCTCGTTGCGATTAGTATCT---------TCCCTTGAAG---ATAAAAGAATACCAAAATATCAGAATTTACGATCTATTCATTCAATATTTCCCTTTTTAGAGGATAAATTATCGCATTTAAATTATGTGTTAGATCTACTAATACCCCATCCCATCCATCTGGAAATCTTGGTTCAAATTCTTCAATGTTGGATCAAAGATGTTCCTTCTTTGCATTTATTGCGATTGTTTTTCCACGAATATCAT---------------AATTTGAATAGTCTC---------ATTACTT------CAAATAAATC---------------CATTTACGTCTTTTCAAAAAG---AAAGAAAAGATTCTTTTGGTTCCTACATAATTCTTATGTATATGAATGCGAATATCTATTCCTGTTTCTTCGTAAACAGTCTTCTTATTTACGATCAATATCTTCTGGAGTCTTTCTTGAGCGAACACATTTCTATGGAAAAATAGAA------TATCTTATAGT---CGTGTGTTGTAATTCTTTTCAGAGG-AGCCTATGGTTCCTCAAAGATACTTTCATACATTATGTTCGATATCAAGGAAAAGCAATTCTGTCTTCAAAAGGAACTCTTATTCTGATAAAGAAATGGAAATTTCATCTTGTTAATTTTTGGCAATCTTATTTTCACTTTTGGTTTCAACCTTATAGGATCCATATAAAGCAATTACCTAAATATTCCTTCTCTTTTCT-GGGGTTTTTTTCAAGTGTAC-TGAAAAATCCTTTGGTAGTAAGAAATCAAATGCTAGAGTATTCATTTCTAATAAATACTCTGACTAATAAATTAGATACCATAGCCCCAGTAAATTCTCTTATTGGATCATTGTCGAATGCTCAATTTTGTACTGTATTGGGTCATCCTATTAGTAAACCGATCTGGACCGATTTATCGGATTCTTATATTATTGATCGATTTTGTCGGATATGTAGAAATCTTTGTCGTTATTACAGCGGATCCTCAAAGAAACAGGTTTTGTATCGTATAAAGTATATACTTCGACTTTCGTGTGCTAGAACTTTGGCTCGTAAACATAAAAGTAC-AGTACGCACTTTTATGCGAAGGTTAGGTTCGGGATTCTTAGAAGAATTTTTGTTGGAAGAAGAACAATCTCTTTCTTGAGCTATCCCGACCATTTCCCGTGCATCATCCTAGCAGAGTACTTA-TATCTATGTCAATGAAAAGAA-CTAAAAAATAAAATCTTAACAA-------------------ATTGGACCTAG--CCCCT-GAATTT----------CTTAG-------ATC-TTCAAAAA----------GAAGAC---TTTTTTT-----------GTAAA-TGTAAGGAAAAAGATATGGACTAT-GAATGA------------TTCAAT---------TTGTTTAAAC-------------------------------------------------------------------------------------------------------------------------------------------------------------------------------------------------------------------------------------------------------------TTTGTTTAAACT------------GAGCCACTGATGAAAAGAGGAT-------------------------------GAGGATAAATA-AAGA------------------GCGAAGAA----------GTAAAAT-----GGGC--TTTTATTGGGGATAGAGGGCCATCTAT---------AAATGGATAATACT--------TTTGTATGTATGCGAATCTTTTAAGATAG--CAAT---------CCCCCAATATCT-TGT---------------TCTAAGAACAAGATATT--GGGGGATTCTTTTG---CTTCTCTATCCG---------------AATTTTCG---------TTCTTTATCAT----AAAAGTTCTC-CTCCGCC---------AATGAATGATAAGT-GCC-----TAGGTG-AAGTAT----------------AGTATAAGATAAGTCAGA----------------------AAAGTCTAAGTCTTAGTATAATACCTATACTCTTA----------CTATAA-GAT------------AAAGACTCTTAAGGATAAGGC-----TTTTCAC----------ATGAATACTTAGTAGAACGACTAACGACGAGATTTATTATCGTTTCTCGCGTGTCTCACGAAAGTTAGAGTAG-GTGCGAATTCTCCCAATTTGTGACCGACCATACGATCTGTGATATAAATGGTAAA-TGTTCCTTTCCATTATGAATAGC-GATTGTATGGCCAATCATTGTGGGTA-TAATGGTAGATGCCCGAGACCAAGTCACTATGATTTCTTTCTCCTCCCTCCTGTT-GAGTTTTTCAATTCTTCCCGATAAATGATTAGCTACAAAAGGATTTTTTTTT-AGTGAACGTGTCAC-GGCTGATTACTCCTTTTTTTCCATTTTT-TAAATTGGCATTCTATGTCCAA----------TATCTCGATCTTAA-TCTG-AAG-TAT---------------------------AATG-ATGAATGG-AAAAAAGAGA----------------TTTA-GCTAG-ATAA--------GGG-AA-GGGGC-GGATGTAG-CCAAGTGG-ATCAA-GGCAGT

Bulbophyllum_ambrosia_SG1221 ACGACT-CTCGGCAATGGATATCTCGGCTCTCGCATCGATGAAGAGCGCAGCGAAATGCGATACGTGGTGCGAATTGCAGAATCCCGCGAACCATCGAGTCTTTGAACGCAAGTTGCGCCCGAGGCCAAC-CGGCCGAGGGCACGTCCGCCTGGGCGTCA-AGCGTTGCGTCGCTCCACAAGAATTCTTTTTCTTCTCATTTTTCT------------TCTCAAATGCTATCAGAAGGTTTTGGAGTCATTCTGGAAATTCCATTCTCGTTGCGATTAGTATCT---------TCCCTTGAAG---ATAAAAGAATACCAAAATCTCATAATTTACGATCTATTCATTCAATATTTCCCTTTTTAGAGGATAAATTATCGCATTTAAATTATGTGTTAGATCTACTAATACCCCATCCCATCCATCTGGAAATCTTGGTTCAAATTCTTCAATGTTGGATCAAAGATGTTCCTTCTTTGCATTTATTGCGATTGTTTTTCCACGAATATCAT---------------AATTTGAATAATCTC---------ATTACTT------CAAAGAAATC---------------CATTTACGTCTTTTCAAAAAG---AAATAAAAGATTCTTTTGGTTCCTACATAATTCTTATGTATATGAATGCGAATATCTATTCCTGTTTCTTCGTAAACAGTCTTCTTATTTACGATCAATATCTTCTGGAGTCTTTCTTGAGCGAACACATTTCTATGGAAAAATAGAA------TATCTTATAGT---CGTGTGTTGTAATTCTTTTCAGAGG-AGCCTATGGTTCCTCAAAGATATTTTCATACATTATGTTCGATATCAAGGAAAAGCAATTCTGTCTTCAAAAGGAACTCTTATTCTGATAAAGAAATGGAAATTTCATCTTGTGAATTTTTGGCAATCTTATTTTCACTTTTGGTTTCAACCTTATAGGATCCATATAAAGCAATTACCCAACTATTCCTTCTCGTTTCT-GGGGTTTTTTTCAAGTGTAC-TGAAAAATCCTTTGGTAGTAAGAAATCAAATGCTAGAGTATTCATTTCTAATAAATATTCTGACTAATAAATTAGATACCATAGCCCCAGTAAATTCTCTTATTGGATCATTGTCGAAAGCTCAATTTTGTACTGTATTGGGTCATCCTATTAGTAAACCGATCTGGACCGATTTATCGGATTCTTATATTATTGATCGATTTTGTCGAATATGTAGAAATCTTTGTCGTTATTACAGCGGATCCTCAAAGAAACAGGTTTTGTATCGTATAAAGTATATACTTCGACTTTCGTGTGCTAGAACTTTGGCTCGTAAACATAAAAGTAC-AGTACGCACTTTTATGCGAAGGTTAGGTTCGGGATTCTTAGAAGAATTTTTTTTGGAAGAAGAACAATCTCTTTCTTGAGCTATCCCGACCATTTCCCGTGCATCATCCTAGCAGAGTACTTA-TATCTATGTCAATGAAAAGAA-CTAAAAAATAAAATCTTAACAA-------------------ATTGGACCTAG--CCCCT-GAATTT----------CTTAG-------ATC-TTCAAAAA----------GAAGAC---TTTTTTT-----------GTAAA-TGTAAGGAAAAAGATATGGACTAT-GAATGA------------TTCAATT---------------------------------------------------------------------------------------------------------------------------------------------------------------------------------------------------------------------------------------------------------------------------------TGTTTAAACT------------GAGCCACTGATGAAAAGAGGAT-------------------------------GGGGATAAATA-AAAA------------------GCGAGGAA----------TTAAAAT-----GGGC--TTTTATTGGGGATAAAGGGCCATCTAT---------AAATGGATAATACT--------TTTGTATGTATGCGAATCTTTTAAGATAG--CAAT---------CCCCCAATATCT-TGT---------------TCTAAGAACAAGATATT--GGGGGATTCTTTA------TTTCTATCCG---------------AATTTTCG---------TTCTTTATCAT----AAAAGTTCTC-CCCCGCC---------AATGAATGATAAGT-GCC-----TAGGTG-AAGTAT----------------AGTATAAGATAAGTCAGA----------------------AAAGTCTAAGTCTTAGTATAAGACCTATACTCTTA----------CTATAA-GAT------------AAAGACTCTTAAGGATAAGGC-----TTTTCACATGAATACTTATGAATACTTAGTAGAACGACTAACGACGAGATTTATTATCGTTTCTCGCGTGTCTCACGAAAGTGAGAGTAG-GTGCGAATTCTCCCAATTTGTGACCGACCATACGATCTGTGATATAAATGGTAAA-TGTTCCTTTCCATTATGAATAGC-GATTGTATGGCCAATCATTGTGGGTA-TAATGGTAGATGCCCGAGACCAAGTCACTATGATTTCTTTCTCCTCCCTCCTGTT-GAGTTTTTCAATTCTTCCCGATAAATGATTAGCTACAAAAGGATTTTTTTTT-AGTGAACGTGTCAC-GGCTGATTACTCCTTTTTTTCCATTTTT-TAAATTGGCATTCTATGTCCAA----------TATCTCGATCTTAA-TCTG-AAG-TAT---------------------------AATG-ATGAATGG-AAAAAAGAGA----------------TTTA-GCTAT-ATAA--------GGG-AA-GGGGC-GGATGTAG-CCAAGTGG-ATCAA-GGCAG-

Bulbophyllum_bicolor_FT28 ACGACT-CTCGGCAATGGATATCTCGGCTCTCGCATCGATGAAGAGCGCAGCGAAATGCGATACGTGGTGCGAATTGCAGAATCCCGCGAACCATCGAGTCTTTGAACGCAAGTTGCGCCCGAGGCCAGC-CGGCCGAGGGCACGTCCGCCTGGGCGTCA-AGCGTTGCGTCGCTCCACAAGAATTCTTTTTCTTCTCATTTTTCT------------TCTCAAATGCTATCAGAAGGTTTTGGAGTCATTCTGGAAATTCCATTCTCGTTGCGATTAGTATCT---------TCCCTTGAAG---ATAAAAGAATACCAAAATCTCAGAATTTACGATCTATTCATTCTATATTTCCCTTTTTAGAGGATAAATTATCGCATTTCAATTATGTGTTAGATCTACTAATACCCCATCCCATCCATCTGGAAATCTTGGTTCAAATTCTTCAATGTTGGATCAAAGATGTTCCTTCTTTGCATTTATTGCGATTGTTTTTCCACGAATATCAT---------------AATTTGAATAATCTC---------ATTACTT------CAAAGAAATC---------------CATTTACGTCTTTTCAAAAAG---AAAGAAAAGATTTTTTTGGTTCCTACATAATTCTTATGTATATGAATGCGAATATCTATTCCTGTTTCTTCGTAAACAGTCTTCTTATTTACGATCAATATCTTCTGGAGTCTTTCTTGAGCGAACACATTTCTATGGAAAATTAGAA------TATCTTATAGT---CGTGTGTTGTAATTCTTTTCAGAGG-AGCCTATGGTTCCTCAAAGATACTTTCATACATTATGTTCGATATCAAGGAAAAGCAATTCTGTCTTCAAAAGGAACTCTTATTCTGATAAAGAAATGGAAATTTCATCTTGTGAATTTTTGGCAATCTTATTTTCACTTTTGGTTTCAACCTTATAGGATCCATATAAAGCAATTACCCAACTATTCCTTCTCGTTTCT-GGGGTTTTTTTCAAGTGTAC-TGAAAAATCCTTTGGTAGTAAGAAATCAAATGCTAGAGTATTCATTTCTAATAAATACTCTGACTAATAAATTAGATACCATAGCCCCAGTAAATTCTCTTATTGGATCATTGTCGAAAGCTCAATTTTGTACTGTATTGGGTCATCCTATTAGTAAACCGATCTGGACCGATTTATCGGATTCTTATATTATTGATCGATTTTGTCGGATATGTAGAAATCTTTGTCGTTATTACAGTGGATCCTCAAAGAAACAGGTTTTGTATCGTATAAAGTATATACTTCGACTTTCGTGTGCTAGAACTTTGTCTCGTAAACATAAAAGTAC-AGTACGCACTTTTATGCGAAGGTTAGGTTCGGGATTCTTAGAAGAATTTTTTTTGGAAGAAGAACAATCTCTTTCTTGAGCTATCCCGACCATTTCCCGTGCATCATCCTAGCAGAGTACTTA-TATCTATGTCAATGAAAAGAA-CTAAAAAATAAAATCTTAACAA-------------------ATTGGATCTAG--CCCCT-GAATTT----------CTTAG-------ATC-TTCAAAAA----------GAAGAC---TTTTTTT-----------GTAAA-TGTAAGGAAAGAGATATGGACTAT-GAATGA------------TTCAATT---------------------------------------------------------------------------------------------------------------------------------------------------------------------------------------------------------------------------------------------------------------------------------TGTTGAAACT------------GAGCCACTGATGAAAATAGGAT-------------------------------GAGGATAAATA-AAGA------------------GCGAGGAA----------TTAAAAT-----GGGC--TTTTATTGGGGATAGAGGGCCATCTAT---------AAATGGATAATACT--------TTTGTATGTATGTGAATCTTTTAAGATAG--CAAT---------CCCCCAATATCT-TGT---------------TCTTAGAACAAGATATT--GGGGGATTCTTTTG---CTTCTCTATCCGTATCCG---------AATTTTCG---------TTCTTTATCAT----AAAAGTTCTC-CCCCGCC---------AATGAATGATAAGT-GCC-----TAGGTG-AAGTAT----------------AGTATAAGATAAGTCAGA----------------------AAAGTCTAAGTCTTAGTATAATACCTATACTCTTA----------CTATAA-GAT------------AAAGACTCTTAAGGATAAGGC-----TTTTCACATGAATACTTATGAATACTTAGTAGAACGACTAACGACGAGATTTATTATCGTTTCTCGCGTGTCTCACGAAAGTGAGAGTAG-GTGCGAATTCTCCCAATTTGTGACCGACCATACGATCTGTGATATAAATGGTAAA-TGTTCCTTTCCATTATGAATAGC-GATTGTATGGCCAATCATTGTGGGTA-TAATGGTAGATGCCCGAGACCAAGTCACTATGATTTCTTTCTCCTCCCTCCTGTT-GAGTTTTTCAATTCTTCCCGATAAATGATTAGCTACAAAAGGATTTTTTTTT-AGTGAACGTGTCAC-GGCTGATTACTCCTTTTTTTCCATTTTT-TAAATTGGCATTCTATGTCCAA----------TATCTCGATCTTAA-TCTG-AAG-TAT---------------------------AATG-ATGAATGG-AAAAAAGAGA----------------TTTA-GCTAG-ATAA--------GGR-AA-GGGGC-GGATGTAG-CCAAGTGG-ATCAA-GGCAGT

Bulbophyllum_delitescens_SG1286 ACGACT-CTCGGCAATGGATATCTCGGCTCTCGCATCGATGAAGAGCGCAGCGAAATGCGATACGTGGTGCGAATTGCAGAATCCCGCGAACCATCGAGTCTTTGAACGCAAGTTGCGCCCGAGGCCAAC-CGGCCGAGGGCACGTCCGCCTGGGCGTCA-AGCGTTGCGTCGCTCC----------------------------------------------------TTGGAAAGGTTTTGGAGTCATTCTGGAAATTCCATTCTCGTTGCGATTAGTATCT---------TCCCTTGAAG---ATAAAAGAATACCAAAATCTCAGAATTTACGATCTATTCATTCAATATTTCCCTTTTTAGAGGATAAATTATCGCATTTAAATTATGTGTTAGATCTACTAATACCCCATCCCATCCATCTGGAAATCTTGGTTCAAATTCTTCAATGTTGGATCAAAGATGTTCCTTCTTTGCATTTATTGCGATTGATTTTCCACGAATATCAT---------------AATTTGAATAATCTC---------ATTACTT------CAAAGAAATC---------------CATTTACGTCTTTTCAAAAAG---AAAGAAAAGATTCTTTTGGTTCCTACATAATTCTTATGTATATGAATGCGAATATCTATTCCTGTTTCTTCGTAAAAAGTCTTCTTATTTACGATCAATATCTTCTGGAGTCTTTCTTGAGCGAACACATTTCTATGGAAAAATAGAA------TATCTTATAGT---CGTGTGTTGTAATTCTTTTCAGAGG-AGCCTATGGTTCCTCAAAGATACTTTCATACATTATGTTCGATATCAAGGAAAAGCAATTCTGTCTTCAAAAGGAACTCTTATTCTGATAAAGAAATGGAAATTTCATCTTGTGAATTTTTGGCAATCTTATTTTCACTTTTGGTTTCAACCTTATAGGATCCATATAAAGCAATTACCCAAATATTCCTTCTCGTTTCT-GGGGTTTTTTTCAAGTGTAC-TGAAAAATACTTTGGTAATAAGAAATCAAATGCTAGAGTATTCATTTCTAATAAATAATCTGACTAATAAATTAGATACCATAGCCCCAGTAAATTCTCTTATTGGATCATTGTCGAAAGCTCAATTTTGTACTGTATTGGGTCATCCTATTAGTAAACCGATCTGGACCGATTTATCGGATTCTTATATTATTGATCGATTTTGTCGGATATGTAGAAATCTTTGTCGTTATTACAGCGGATCCTCAAAGAAACAGGTTTTGTATCGTATAAAGTATATACTTCGACTTTCGTGTGCTAGAACTTTGGCTCGTAAACATAAAAGTAC-AGTACGCACTGTTATGGAAACGTTTAATT------------------------------------------------GAGCTATCCCGACCATTTCCCGTGCATCATCCTAGCAGAGTACTTA-TATCTATGTCAATGAAAAGAA-CTAAAAAATAAAATCTTAACAA-------------------ATTGGACCTAG--CCCCT-TAATTT----------CTTAG-------ATC-TTCAAAAA----------GAAGAC---TTTTTTC-----------GTAAA-TGTAAGGAAAGAGATATGGACTAT-GAATGA------------TTCAATT---------------------------------------------------------------------------------------------------------------------------------------------------------------------------------------------------------------------------------------------------------------------------------TGTTTAAACT------------GAGCCACTGCTGAAAAGAGGAT-------------------------------GAGGATAAATA-AAGA------------------GCGAGGAA----------TTAAAAT-----GGGC--TTTTATTGGGGATAGAGGGCCATCTAT---------AAATGGATAATACT--------TTTGTATGTATGCGAATCTTTTAAGATAG--CAAT---------CCCCCAATATCT-TGT---------------TCTTAGAACAAGATATT--GGGGGATTCTTTTG---CTTCTCTATCCG---------------AATTTTCG---------TTCTTTATCAT----AAAAGTTCTC-CCCCGCC---------AATGAATGATAAGT-GCC-----TAGGTG-AAGTAT----------------AGTATAAGATAAGTCAGA----------------------AAAGTCTAAGTCTTAGTATAATACCTATACTCTTA----------CTATAA-GAT------------AAAGACTCTTAAGGATAAGGC-----TTTTCACATGAATACTTATGAATACTTAGTAGAACGACTAACGACGAGATTTATTATCGTTTCTCGCGTGTCTCACGAAAGTGAGAGTAG-GTGCGAATTCTCCCAATTTGTGACCGACCATACGATCTGTGATATAAATGGTAAA-TGTTCCTTTCCATTATGAATAGC-GATTGTATGGCCAATCATTGTGGGTA-TAATGGTAGATGCCCGAGACCAAGTCACTATGATTTCTTTCTCCTCCCTCCTGTT-GAGTTTTTCAATTCTTCCCGATAAATGATTAGCTACAAAAGGATTTTTTTTT-AGTGAACGTGTCAC-GGCTGATTACTCCTTTTTTTCCATTTTT-TAAATTGGCATTCTATGTCCAA----------TATCTCGATCTTAA-TCTG-AAG-TAT---------------------------AATG-ATGAATGG-AAAAAAGAGA----------------TTTA-GCTAG-ATAA--------GGG-AA-GGGGC-GGATGTAG-CCAAGTGG-ATCAA-GGCAGT

Bulbophyllum_kwangtungense_KFBG2798 ACGACT-CTCGGCAATGGATATCTCGGCTCTCGCATCGATGAAGAGCGCAGCGAAATGCGATACGTGGTGCGAATTGCAGAATCACGCGAACCATCGAGTCTTTGAACGCAAGTTGCGCCCGAGGCCAAC-CGGCCGAGGGCACGTCCGCCTGGGCGTCA-AGCGTTGCGTCGCTCCAAAAGAATTCTTTTTCTTATCATTTTTCT------------TCTCAAATGCTATCAGAAGGTTTTGGAGTCATTCTGGAAATTCCATTCTTGTTGCGATTAGTATCT---------TCCCTTGAAG---ATAAAAGAATACCAAAATCTCATAATTTACGATCTATTCATTCAATATTTCCCTTTTTAGAGGATAAATTATCGCATTTAAATTATGTGTTAGATCTACTAATACCCCATCCCATCCATCTGGAAATCTTGGTTCAAATTCTTCAATGTTGGATCAAAGATGTTCCTTCTTTGCATTTATTGCGATTGTTTTTCCACGAATATCAT---------------AATTTGAATAATCTC---------ATTACTT------CAAAGAAATC---------------CATTTACGTCTTTTCAAAAAG---AAAGAAAAGATTCTTTTTGTTCCTACATAATTCTTATGTATATGAATGCGAATATCTATTCCTCTTTCTTCGTAAAAAGTCTTCTTATTTACGATCAATATCTTCTGGAGTCTTTCTTGAGCGAACACATTTCTATGGAAAAATAGAA------TATCTTATAGT---CGTGTGTTGTAATTCTTTTCAGAGG-AGCCTATGGTTCCTCAAAGATATTTTCATACATTATGTTCGATATCAAGGAAAAGCAATTCTGTCTTCAAAAGGAACTCTTATTCTGATAAAGAAATGGAAATTTCATCTTGTGAATTTTTGGCAATCTTATTTTCACTTTTGGTTTCAACCTTATAGGATCCATATAAAGCAATTACCCAACTATTCCTTCTCCTTTCT-GGGGTTTTTTTCAAGTGTAC-TGAAAAATCCTTTGGTAGTAAGAAATCAAATGCTAGAGTATTCATTTCTAATAAATATTCTGACTAATAAATTAGATACCATAGCCCCAGTAAATTCTCTTATTGGATCATTGTCGAAAGCTCAATTTTGTACTGTATTGGGTCATCCTATTAGTAAACCAATCTGGACCGATTTATCGGATTCTTATATTATTGATCGATTTTGTCGAATATGTAGAAATCTTTGTCGTTATTACAGCGGATCCTCAAAGAAACAGGTTTTGTATCGTATAAAGTATATACTTCGACTTTCGTGTGCTAGAACTTTGGCTCGTAAACATAAAAGTAC-AGTACGCACTTTTATGCGAAGGTTAGGTTCGGGATTCTTAGAAGAATTTTT--------------------------GAGCTATCCCGACCATTTCCCGTGCATCATCCTAGCAGAGTACTTA-GATCTATGTCAATGAAAAGAA-CTAAAAAAGAAAATCTTAACAA-------------------ATTGGACCTAG--CCCCT-GAATTT----------CTTAG-------ATC-TTCAAAAA----------GAAGAC---TTTTTTT-----------GTAAA-TGTAAGGAAAAAGATATGGACTAT-GAATGA------------TTCAATT---------------------------------------------------------------------------------------------------------------------------------------------------------------------------------------------------------------------------------------------------------------------------------TGTTTAAACT------------GAGCCACTGATGAAAAGAGGAT-------------------------------GAGGATAAATA-AAGA------------------GCGAGGAA----------TTAAAAT-----GGGC--TTTTATTGGGGATAGAGGGCCCGCTAT---------AAATGGATAATACT--------TTTGTATTTATGCGAATCTTTTAAGATAG--CAAT---------CCCCCAATATCT-TGT---------------TCTTAGAACAAGATATT--GGGGGATTCTTTTG---CTTCTCTATCCG---------------AATTTTCG---------TTCTTTATCAT----AAAAGTTCTC-CCCCGCC---------AATGAATGATAAGT-GCC-----TAGGTG-AAGTAT----------------AGTATAAGATAAGTCAGA----------------------AAAGTCTAAGTCTTAGTATAAGACCTATACTCTTACTATAGA---CTATAA-GAT------------AAAGACTCTTAAGGATAAGGC-----TTTTCACATGAATACTTATGAATACTTAGTAGAACGACTAACGACGAGATTTATTATCGTTTCTCGCGTGTCTCACGAAAGTGAGAGTAG-GTGCGAATTCTCCCAATTTGTGACCGACCATACGATCTGTGATATAAATGGTAAA-TGTTCCTTTCCATTATGAATAGC-GATTGTATGGCCAATCATTGTGGGTA-TAATGGTAGATGCCCGAGACCAAGTCACTATGATTTCTTTCTCCTCCCTCCTGTT-GAGTTTTTCAATTCTTCCCGATAAATGATTAGCTACAAAAGGATTTTTTTTT-AGTGAACGTGTCAC-GGCTGATTACTCCTTTTTTTCCATTTTT-GAAATTGGCATTCTATGTCCAA----------TATCTCGATCTTAA-TCTG-AAG-TAT---------------------------AATG-ATGAATGG-AAAAAAGAGA----------------TTTA-GCTAG-ATAA--------GGG-AA-GGGGC-GGATGTAG-CCAAGTGG-ATCAA-GGCAGT

Bulbophyllum_odoratissimum_SG1275 ACGACT-CTCGGCAATGGATATCTCGGCTCTCGCATCGATGAAGAGCGCAGCGAAATGCGATACGTGGTGCGAATTGCAGAATCCCGCGAACCATCGAGTCTTTGAACGCAAGTTGCGCCCGAGGCCAAC-CGGCCGAGGGCACGTCCGCCTGGGCGTCA-AGCGTTGCGCCGCTCC-CAAGAATTCTTTTTCTTCTCATTTTTTT------------TCTCAAATGCTATCAGAAGGTTTTGGAGTCATTCTGGAAATTCCATTCTCGTTGCGATTAGTATCT---------TCCCTTGAAG---ATAAAAGAATACCAAAATTTCATAATTTACGATCTATTCATTCAATATTTCCCTTTTTAGAGGATAAATTATCGCATTTAAATTATGTTTTAGATCTACTAATACCCCATCCCATCCATCTGGAAATCTTGGTTCAAATTCTTCAATGTTGGATCAAAGATGTTCCTTCTTTGCATTTCTTGCGATTGATTTTCCACGAATATCAT---------------AATTTGAATAATCTC---------ATTACTT------CAAAGAAATC---------------CATTCACGTCTTTTCAAAAAG---AAAGAAAAGATTCTTTTGGTTCCTACATAATTCTTATGTATATGAATGCGAATATCTATTCCTGTTTCTTCGTAAACAGTCTTCTTATTTACGATCAATATCTTCTGGAATCTTTCTTGAGCGAACACATTTCTATGGAAAAATCGAA------TATCTTATAGT---CGTGTGTTGTAATTATTTTCAGAGG-AGCCTATGGTTCCTCAAAGATATTTTCATACATTATGTTCGATATCAAGGAAAAGCAATTCTGTCTTCAAAAGGAACTCTTATTCTGATAAAGAAATGGAAATTTCATCTTGTGAATTTTTGGCAATCTTATTTTCACTTTTGGTTTCAACCTTATAGGATCCATATAAAGCAATTACCCAACTATTCCTTCTTGTTTCT-GGGGTTTTTTTCAAGTGTAC-TGAAAAATCCTTTGGTAGTAAGAAATCAAATGCTAGAGTATTCATTTCTAATAAATATTCTGACTAATAAATTAGATACCATAGCCCCAGTAAATTCTCTTATTGGATCATTGTCGAAAGCTCAATTTTGTACTGTATTGGGTCATCCTATTAGTAAACCGATCTGGACCGATTTATCGGATTCTTATATTATTGATCGATTTTGTCGAATATGTAGAAATCTTTGTCGTTATTACAGCGGATCTTCAAAGAAACAGGTTTTGTATCGTATAAAGTATATACTTCGACTTTCGTGTGCTAGAACTTTGGCTCGTAAACATAAAAGTAC-AGTACGCACTTTTATGCGAAGGTTAGGTTCGGGATTCTTAGAAGAATTTTTTTTGGAAGAAGAACAATCTCTTTCTTGAGCTATCCCGACCATTTCCCGTGCATCATCCTAGCAGAGTACTTA-TATCTATGTCAATGAAAAGAA-CTAAAAAAGAAAATCTTAACAA-------------------ATTGGACCTAG--CCCCT-GAATTT----------CTTAG-------ATC-TTCAAAAA----------GAAGAC---TTTTTAT------------------------------------------GAATGA------------TTCAATT---------------------------------------------------------------------------------------------------------------------------------------------------------------------------------------------------------------------------------------------------------------------------------TGTTTAAACT------------GAGCCACTGATGAAAAGAGGAT-------------------------------GAGGATAAATA-AAGA------------------GCGAGGAA----------TGAAAAT-----GGGC--TTTTATTGGGGATAGAGGGCCATCTAT---------AAATGGATAAAACT--------TTTGTATCTATGCAAATCTTTTAAGATAG--CAAT---------CCCCCAATATCT-TGT---------------TCTAAGAACAAGATATT--GGGGGATTCTTTTG---CTTCTCTATCCG---------------AATTTTCG---------TTCTTTATCAT----AAAAGTTCTC-CCCCGCC---------AATGAATGATAAGT-GCC-----TAGGTG-AAGTAT----------------AGTATAAGATAAGTCAGA----------------------AAAGTCTAAGTCTTAGTATAAGACCTATACTCTTA----------CTATAA-GAT------------AAAGACTCTTAAGGATAAGGC-----TTTTCACATGAATACTTATGAATACTTAGTAGAACGACTAACGACGAGATTTATTATCGTTTCTCGCGTGTCTCACGAAAGTGAGAGTAG-GTGCGAATTCTCCCAATTTGTGACCGACCATACGATCTGTGATATAAATGGTAAA-TGTTCCTTTCCATTATGAATAGC-GATTGTATGGCCAATCATTGTGGGTA-TAATGGTAGATGCCCGAGACCAAGTCACTATGATTTCTTTCTCCTCCCTCCTGTT-GAGTTTTTCAATTCTTCCCGATAAATGATTAGCTACAAAAGGATTTTTTTTT-AGTGAACGTGTCAC-GGCTGATTACTCCTTTTTTTCCATTTTT-GAAATTGGCATTCTATGTCCAA----------TATCTCGATCTTAA-TCTG-AAG-TAT---------------------------AATG-ATGAATGG-AAAAAAGAGA----------------TTTA-GCTAG-ATAA--------GGG-AA-GGGGC-GGATGTAG-CCAAGTGG-ATCAA-GGCAGT

Bulbophyllum_pectenveneris_KFBG294 ACGACT-CTCGGCAATGGATATCTCGGCTCTCGCATCGATGAAGAGCGCAGCGAAATGCGATACGTGGTGCGAATTGCAGAATCCCGCGAACCATCGAGTCTTTGAACGCAAGTTGCGCCCGAGGCCGAC-CGGCCGAGGGCACGTCCGCCTGGGCGTCG-AGCGTTGCGTCGCTCCACGAGAATTCTTTTTCTTCTCATTTTTCT------------TCTCAAATGCTATCAGAAGGTTTTGGAGTCATTCTGGAAATTCCATTCTCGTTGCGATTAATATCT---------TCCCTTGAAG---ATAAAAGAATACCAAAATCTCAGAATTTACGATCTATTCATTCAATATTTCCCTTTTTAGAGGATAAATTAACGCATTTAAATTATGTGTTAGATCTACTAATACCCCATCCCATCCATCTGGAAATCTTGGTTCAAATTCTTCAATGTTGGATCAAAGATGTTCCTTCTTTGCATTTATTGCGATTGTTTTTCCACGAATATCAT---------------AATTTGAATAATCTC---------ATTACTT------CAAAGAAATC---------------CATTTACATCTTTTCAAAAAG---AAAGAAAAGATTCTTTTGGTTCCTACATAATTCTTATGTATATGAATGCGAATATCTATTCCTGTTTCTTCGTAAACAGTCTTCTTATTTACGATCAATATCTTCTGGAGTCTTTCTTGAGCGAACACATTTCTATGGAAAAATAGAG------TATCTTATAGT---CGTGTGTTGTAATTCTTTTCAGAGG-AGCCTATGGTTCCTCAAAGATATTTTCATACATTATGTTCGATATCAAGGAAAAGCAATTCTGTCTTCAAAAGGAACTCTTATTCTGATAAAGAAATGGAAATTTCATCTTGTGAATTTTTGGCAATCTTATTTTCACTTTTGGTTTCAACCTTATAGGATCCATATAAAGCAATTACCCAACTATTCCTTCTCGTTTCT-GGGGTTTTTTTCAAGTGTAC-TTAAAAATCATTTGTTAGTAAGAAATCAAATGCTAGAGTATTCATTTCTAATAACTATTCTGACTAAGAAATTAGATACCATAGCCCCAGTAAATTCTCTTATTGGATCATTATCGAAAGCTCAATTTTGTACTGTATTGGGTCATCCTATTAGTAAACCGATCTGGACCTATTTATCGGATTCTTATATTATTGATCGATTTTGTCGGATATGTAAAAATCTTTGTCGTTATTACAGCGGATCCTCAAAGAAACAGGTTTTGTATCGTATAAAGTATATACTTCGACTTTCGTGTGCTAGAACTTTGGCTCGTAAACATAAAAGTAC-AGTACGCACTTTTATGCGAAGGTTAGGTTCGGGATTCTTAGAAGAATTTTT--------------------------GAGCTATCCCGACCATTTCCCGTGCATCATCCTAGCAGAGTACTTA-TATCTATGTCAATGAAAAGAA-CTAAAAAATAAAATCTTAACAA-------------------ATTGGACCTAG--CCCCT-GAATTT----------ATTAG-------ATC-TTCAAAAA----------GAAGAC---TTTTTTT-----------GTAAA-TGTAAGGAAAGAGATATGGACTAT-GAATGA------------TTCAATT---------------------------------------------------------------------------------------------------------------------------------------------------------------------------------------------------------------------------------------------------------------------------------TGTTTAAACT------------GAGC-ACTGATGAAAATAGGAT-------------------------------GAGGATAAATA-AAGA------------------GCGAGGAA----------TTAAAAT-----GGGC--TTTTATTGGGGATAGAG--CCATCTAT---------AAATGGATAATACT--------TTTGTATGTATGCGAATCTTTTAAGATAG--CAAT---------CCCCCAATATCT-TGT---------------TCTTAGAACAAGATATT--GGGGGATTCTTTTG---CTTCTCTATCCG---------------AATTTTCG---------TTCTTTATCAT----AAAAGTTCTC-CCCCGCC---------AATGAATGATAAGT-GCC-----TAGGTG-AAGTAT----------------AGTATAAGATAAGTCAGA----------------------AAAGTATAAGTCTTAGTATAAGACCTATACTCTTA----------CTATAA-GAT------------AAAGACTCTTAAGGATAAGGC-----TTTTCACATGAATACTTATGAATACTTAGTAGAACGACTAACGACGAGATTTATTATCGTTTCTCGCGTGTCTCACGAAAGTGAGAGTAG-GTGCGAATTCTCCCAATTTGTGACCGACCATACGATCTGTGATATAAATGGTAAA-TGTTCCTTTCCATTATGAATAGC-GATTGTATGGCCAATCATTGTGGGTA-TAATGGTAGATGCCCGAGACCAAGTCACTATGATTTCTTTCTCCTCCCTCCTGTT-GAGTTTTTCAATTCTTACCGATAAATGATTAGCTACAAAAGGATTTTTTTTT-AGTGAACGTGTCAC-GGCTGATTACTCCTTTTTTTCCATTTTT-TAAATTGGCATTCTATGTCCAA----------TATCTCGATCTTAA-TCTG-AAG-TAT---------------------------AATG-ATGAATGG-AAAAAATAGA----------------TTTA-GCTAG-ATAA--------GGG-AA-GGGGC-GGATGTAG-CCAAGTGG-ATCAA-GGCAGT

Bulbophyllum_scabratum_PK12041 ACGACT-CTCGGCAATGGATATCTCGGCTCTCGCATCGATGAAGAGCGCAGCGAAATGCGATACGTGGTGCGAATTGCAGAATCCCGCGAACCATCGAGTCTTTGAACGCAAGTTGCGCCCGAGGCCGAC-CGGCCGAGGGCACGCCCGCCTGGGCGTCA-AGCGTCGCGTCGCTCCATAAGAATTCTTTTTCTTCTCATTTTTCT------------TCTCAAATGCTATCAGAAGGTTTTGGAGTCATTCTGGAAATTCCATTCTCGTTGCAATTAGTATCT---------TCCCTTGAAG---ATAAAAGAATACCAAAATCTCATAATTTACGATCTATTCATTCAATATTTCCCTTTTTAGAGGATAAATTATCGCATTTAAATTATGTGTTAGATCTACTAATACCCCATCCCATCCATMTGGAAATCTTGGTTCAAATTCTTCAATGTTGGATCAAAGATGTTCCTTCTTTGCATTTATTGCGATTGTTTTTCCACGAATATCAT---------------AATTTGAATAATCTC---------ATTACYT------CAAAGAAATC---------------TATTTACGTCTTTTCAAAAAG---AAAGAAAAGATTCTTTTGGTTCCTACATAATTCTTATGTATATGAATGCGAATATCTATTCCTGTTTCTTCGTAAACAGTCTTCTTATTTACGATCAATATCTTCTGGAGTCTTTCTTGAGCGAACACATTTCTATGGAAAAATAGAA------TATCTTATAGT---CGTGTGTTGTAATTCTTTTAAGAGG-AGCCTATGGTTCCTCAAAGATATTTTCATACATTATGTTCGATATCAAGGAAAAGCAATTCTGTCTTCAAAAGGAACTCTTATTCTTATAAAGAAATGGAAATTTCATCTTGTGAATTTTTGGCAATCTTATTTTCACTTTTGGTTTCAACCTTATAGGATCCATATAAAGCAATTACCCAACTATTCCTTCTCGTTTCT-GGGGTTTTTTTCAAGTGTAC-TGAAAAATCCTTTGGTAGTAAGAAATCAAATGCTAGAGTATTCATTTCTAATAAATATTCTGACTAATAAATTAGATACCATAGCCCCAGTAAATTCTCTTATTGGATCATTGTCGAAAGCTCAATTTTGTACTGTATTGGGTCATCCTATTAGTAAACCGATCTGGACCGATTTATCGGATTCTTATATTATTGATCGATTTTGTCGAATATGTAGAAATCTTTGTCGTTATTACAGTGGATCCTCAAAGAAACAGGTTTTGTATCGTATAAAGTATATACTTCGACTTTCGTGTGCTAGAACTTTGGCTCGTAAACATAAAAGTAC-TGTACGCACTTTTATGCGAAGGTTAGGTTCGGGATTCTTAGAAGAATTTTTTTTGGAAGAAGAACAATCTCTTTCTTGAGCTATCCCGACCATTTCTCGTGCATCATCCTAGCAGAGTACTTA-TATCTATGTCAATGAAAAGAA-CTAAAAAATAAAATCTTAACAA-------------------ATTGGACCTAG--CCCAT-GAATTT----------CTTAG-------ATC-TTCAAAAA----------GAAGAC---TTTTTTT-----------GTAAA-TGTAAGGAAAAAGATATGGACTAT-GAATGA------------TTCAATT---------------------------------------------------------------------------------------------------------------------------------------------------------------------------------------------------------------------------------------------------------------------------------TGTTTAAACT------------GAGTCACTGATGAAAAGAGGAT-------------------------------GAGGATAAATA-AAGA------------------GCGAGGAA----------TTAAAAT-----GGGC--TTTTATTGGGGATAGAGGGCCATCTAT---------AAATGGATAATAATAAGATT--TTTATATGTATGCGAATCTTTTAAGATAG--CAAT---------CCCCCAATATCT-TGT---------------TCTTAGAACAAGATATT--GGGGGATTCTTTTG---CTTCTCTATCCG---------------AATTTTCG---------TTCTTTATCAT----AAAAGTTCTC-CCCCGCC---------AATGAATGATAAGT-GCC-----TAGGTG-AAGTAT----------------AGTATAAGATAAGTCAGA----------------------AAAGTCTAAGTCTTAGTATAAGACCTATACTCTTA----------CTATAA-GAT------------AAAGACTCTTAAGGATAAGGC-----TTTTCACATGAATACTTATGAATACTTAGTAGAACGACTAACGACGAGATTTATTATCGTTTCTCGCGTGTCTCACGAAAGTGAGAGTAG-GTGCGAATTCTCCCAATTTGTGACCGACCATACGATCTGTGATATAAATGGTAAA-TGTTCCTTTCCATTATGAATAGC-GATTGTATGGCCAATCATTGTGGGTA-TAATGGTAGATGCCCGAGACCAAGTCACTATGATTTCTTTCTCCTCCCTCCTGTT-GAGTTTTTCAATTCTTCCCGATAAATGATTAGCTACAAAAGGATTTTTTTTT-AGTGAACGTGTCAC-GGCTGATTACTCCTTTTTTTCCATTTTT-TAAATTGGCATTCTATGTCCAA----------TATCTCGATCTTAA-TCTG-AAG-TAT---------------------------AATG-ATGAATGG-AAAAAAGAGA----------------TTTA-GCTAG-ATAA--------GGG-AA-GGGGC-GGATGTAG-CCAAGTGG-ATCAA-GGCAGT

Bulbophyllum_stenobulbon_SG1226 ACGACT-CTCGGCAATGGATATCTCGGCTCTCGCATCGATGAAGAGCGCAGCGAAATGCGATACGTGGTGCGAATTGCAGAATCACGCGAACCATCGAGTCTTTGAACGCAAGTTGCGCCCGAGGCCAAC-CGGCCGAGGGCACGTCCGCCTGGGCGTCA-AGCGTTGCGTCGCTCCACAAGAATTCTTTTTCTTATCATTTTTCT------------TCTCAAATGCTATCAGAAGGTTTTGGAGTCATTCTGGAAATTCCATTCTTGTTGCGATTAGTATCT---------TCCCTTGAAG---ATAAAAGAATACCAAAATCTCATAATTTACGATCTATTCATTCAATATTTCCCTTTTTAGAGGATAAATTATCGCATTTAAATTATGTGTTAGATCTACTAATACCCCATCCCATCCATCTGGAAATCTTGGTTCAAATTCTTCAATGTTGGATCAAAGATGTTCCTTCTTTGCATTTATTGCGATTGTTTTTCCACGAATATCAT---------------AATTTGAATAATCTC---------ATTACTT------CAAAGAAATC---------------CATTTACGTCTTTTCAAAAAG---AAAGAAAAGATTCTTTTGGTTCCTACATAATTCTTATGTATATGAATGCGAATATCTATTCCTCTTTCTTCGTAAACAGTCTTCTTATTTACGATCAATATCTTCTGGAGTCTTTCTTGAGCGAACACATTTCTATGGAAAAATAGAA------TATCTTATAGT---CGTGTGTTGTAATTCTTTTCAGAGG-AGCCTATGGTTCCTCAAAGATATTTTCATACATTATGTTCGATATCAAGGAAAAGCAATTCTGTCTTCAAAAGGAACTCTTATTCTGATAAAGAAATGGAAATTTCATCTTGTGAATTTTTGGCAATCTTATTTTCACTTTTGGTTTCAACCTTATAGGATCCATATAAAGCAATTACCCAACTATTCCTTCTCCTTTCT-GGGGTTTTTTTCAAGTGTAC-TGAAAAATCATTTGGTAGTAAGAAATCAAATGCTAGAGTATTCATTTCTAATAAATATTCTGACTAATAAATTAGATACCATAGCCCCAGTAAATTCTCTTATTGGATCATTGTCGAAAGCTCAATTTTGTACTGTATTGGGTCATCCTATTAGTAAACCAATCTGGACCGATTTATCGGATTCTTATATTATTGATCGATTTTGTCGAATATGTAGAAATCTTTGTCATTATTACAGCGGATCCTCAAAGAAACAGGTTTTGTATCGTATAAAGTATATACTTCGACTTTCGTGTGCTAGAACTTTGGCTCGTAAACATAAAAGTAC-AGTACGCACTTTTATGCGAAGGTTAGGTTCGGGATTCTTAGAAGAATTTTTTTTGGAAGAAGAACAATCTCTTTCTTGAGCTATCCCGACCATTTCCCGTGCATCATCCTAGCAGAGTACTTA-TATCTATGTCAATGAAAAGAA-CTAAAAAAGAAAATATTAACAA-------------------ATTGGACCTAG--CCCCT-GAATTT----------YTTAG-------ATC-TTCAAAAA----------GAAGAC---TTTTTTT-----------GTAAA-TGTAAGGAAAAAGATATGGACTAT-GAATGA------------TTCAATT---------------------------------------------------------------------------------------------------------------------------------------------------------------------------------------------------------------------------------------------------------------------------------TGTTTAAACT------------GAGCCACTGATGAAAAGAGGAT-------------------------------GAGGATAAATA-AAGA------------------GCGAGGAA----------TTAAAAT-----GGGC--TTTTATTGGGGATAG----CCATCTAT---------AAATGGATAATACT--------TTTGTATTTATGCGAATCTTTTAAGATAG--CAAT---------CCCCCAATATCT-TGT---------------TCTTAGAACAAGATATT--GGGGGATTCTTTTG---CTTCTCTATCCG---------------AATTTTCG---------TTCTTTATCAT----AAAAGTTCTC-CCCCGCC---------AATGAATGATAAGT-GCC-----TAGGTG-AAGTAT----------------AGTATAAGATAAGTCAGA----------------------AAAGTCTAAGTCTTAGTATAAGACCTATACTCTTACTATAGA---CTATAA-GAT------------AAAGACTCTTAAGGATAAGGC-----TTTTCACATGAATACTTATGAATACTTAGTAGAACGACTAACGACGAGATTTATTATCGTTTCTCGCGTGTCTCACGAAAGTGAGAGTAG-GTGCGAATTCTCCCAATTTGTGACCGACCATACGATCTGTGATATAAATGGTAAA-TGTTCCTTTCCATTATGAATAGC-GATTGTATGGCCAATCATTGTGGGTA-TAATGGTAGATGCCCGAGACCAAGTCACTATGATTTCTTTCTCCTCCCTCCTGTT-GAGTTTTTCAATTCTTCCCGATAAATGATTAGCTACAAAAGGATTTTTTTTT-AGTGAACGTGTCAC-GGCTGATTACTCCTTTTTTTCCATTTTT-GAAATTGGCATTCTATGTCCAA----------TATCTCGATCTTAA-TCTG-AAG-TAT---------------------------AATG-ATGAATGG-AAAAAAGAGA----------------TTTA-GCTAG-ATAA--------GGG-AA-GGGGC-GGATGTAG-CCAAGTGG-ATCAA-GGCAGT

Bulbophyllum_tigridum_SG1310 ACGACT-CTCGGCAATGGATATCTCGGCTCTCGCATCGATGAAGAGCGCAGCGAAATGCGATACGTGGTGCGAATTGCAGAATCCCGCGAACCATCGAGTCTTTGAACGCAAGTTGCGCCCGAGGCCAGC-CGGCCGAGGGCACGTCCGCCTGGGCGTCA-AGCGTTGCGTCGCTCCACAAGAATTCTTTTTCTTCTCATTTTTCT------------TCTCAAATGCTATCAGAAGGTTTTGGAGTCATTCTGGAAATTCCATTCTCGTTGCGATTAGTATCT---------TCCCTTGAAA---ATAAAAGAATACCAAAATCTCAGAATTTACGATCTATTCATTCAATATTTCCCTTTTTAGAGGATAAATTAACACATTTAAATTATGTGTTAGATCTACTAATACCCCATCCCATCCATCTGGAAATCTTGGTTCAAATTCTTCAATGTTGGATCAAAGATGTTCCTTCTTTGCATTTATTGCGATTGTTTTTCCACGAATATCAT---------------AATTTGAATAATCTC---------ATTACTT------CAAAGAAATC---------------CATTTACGTCTTTTCAAAAAG---AAAGAAAAGATTCTTTTGGTTCCTACATAATTCTTATGTATATGAATGCGAATATCTATTCCTGTTTCTTCGTAAACAGTCTTCTTATTTACGATCAATATCTTCTGGAGTCTTTCTTGAGCGAACACATTTCTATGGAAAAATAGAA------TATCTTATAGT---CGTGTGTTGTAATTCTTTTCAGAGG-AGCCTATGGTTCCTCAAAGATATTTTCATACATTATGTTCGATATCAAGGAAAAGCAATTCTGTCTTCAAAAGGAACTCTTATTCTGATAAAGAAATGGAAATTTCATCTTGTGAATTTTTGGCAATCTTATTTTCACTTTTGGTTTCAACCTTATAGGATCCATATAAAGCAATTACCCAACTATTCCTTCTCGTTTCT-GGGGTTTTTTTCAAGTGTAC-TGAAAAATCCTTTGGTAGTAAGAAATCAAATGCTAGAGTATTCATTTCTAATAAATATTCTGACTAAGAAATTAGATACCATAGCCCCAGTAAATTCTCTTATTGGATCATTGTCGAAAGCTCAATTTTGTACTGTATTGGGTCATCCTATTAGTAAACCGATCTGGACCGATTTATCGGATTCTTATATTATTGATCGATTTTGTCGGATATGTAGAAATCTTTGTCGTTATTACAGCGGATCCTCAAAGAAACAGGTTTTGTATCGTATAAAGTATATACTTCGACTTTCGTGTGCTAGAACTTTGGCTCGTAAACATAAAAGTAC-AGTACGCACTTTTATGCGAAGGTTAGGTTCGGGATTCTTAGAAGAATTTTTT-TGGAAG------------------GAGCTATCCCGACCATTTCCCGTGCATCATCCTAGCAGAGTACTTA-TATCTATGTCAATGAAAAGAA-CTAAAAAATAAAATCTTAACAA-------------------ATTGGACCTAG--CCCCT-GAATTT----------CTTAG-------ATC-TTCAAAAA----------GAAGAC---TTTTTTT-----------TTAAA-TGTAAGGAAAGAGATATGGACTAT-GAATGA------------TTCAATT---------------------------------------------------------------------------------------------------------------------------------------------------------------------------------------------------------------------------------------------------------------------------------TGTTTAAACT------------GAGCCACTGATGAAAAAAGGAT-------------------------------GAGGATAAATA-AAGA------------------GCGAGGAA----------TTAAAAT-----GGGC--TTTTATTGGGGATAGAGGGCCATCTAT---------AAATGGATAATACT--------TTTGTATGTATGCGAATCTTTTAAGATAG--CAAT---------CCCCCAATATCT-TGT---------------TCTTAGAACAAGATATT--GGGGGATTCTTTTG---CTTCTCTATCCG---------------AATTTTCG---------TTCTTTATCAT----AAAAGTTCTC-CCCCGCC---------AATGAATGATAAGT-GCC-----TAGGTG-AAGTAT----------------AGTATAAGATAAGTCAGA----------------------AAAGTCTAAGTCTTAGTATAAGACCTATACTCTTA----------CTATAA-GAT------------AAAGACTCTTAAGGATAAGGC-----TTTTCACATGAATACTTATGAATACTTAGTAGAACGACTAACGACGAGATTTATTATCGTTTCTCGCGTGTCTCACGAAAGTGAGAGTAG-GTGCGAATTCTCCCAATTTGTGACCGACCATACGATCTGTGATATAAATGGTAAA-TGTTCCTTTCCATTATGAATAGC-GATTGTATGGCCAATCATTGTGGGTA-TAATGGTAGATGCCCGAGACCAAGTAACTATGATTTCTTTCTCCTCCCTCCTGTT-GAGTTTTTCAATTCTTCCCGATAAATGATTAGCTACAAAAGGATTTTTTTTT-AGTGAACGTGTCAC-GGCTGATTACTCCTTTTTTTCCATTTTT-TAAATTGGCATTCTATGTCCAA----------TATCTCGATCTTAA-TCTG-AAG-TAT---------------------------AATG-ATGAATGG-AAAAAAGAGA----------------TTTA-GCTAG-ATAA--------GGG-AA-GGGGC-GGATGTAG-CCAAGTGG-ATCAA-GGCAGT

Bulbophyllum_tseanum_SG1272 ACGACT-CTCGGCAATGGATATCTCGGCTCTCGCATCGATGAAGAGCGCAGCGAAATGCGATACGTGGTGCGAATTGCAGAATCCCGCGAACCATCGAGTCTTTGAACGCAAGTTGCGCCCGAGGCCAAC-CGGCCGAGGGCACGTCCGCCTGGGCGTCA-AGCGTCGCGTCGCTCCACAAGAATTCTTTTTCTTCTCATTTTTCT------------TCTCAAATACTATCAGAAGGTTTTGGAGTCATTCTGGAAATTCCATTCTCGTTGCGATTAGTATCT---------TCCCTTGAAG---ATAAAAGAATACCAAAATATCAGAATTTACGATCTATTCATTCAATATTTCCCTTTTTAGAGGATAAATTATCGCATTTAAATTATGTGTTAGATCTACTAATACCCCATCCCATCCATCTGGAAATCTTGGTTCAAATTCTTCAATGTTGGATCAAAGATGTTCCTTCTTTGCATTTATTGCGATTGTTTTTCCACGAATATCAT---------------AATTTGAAGAATCTC---------ATTACTT------CAAAGAAATC---------------CATTTACGTCTTTTCAAAAAG---AAAGAAAAGATTTTTTTGGTTCCTACATAATTCTTATGTATATGAATGCGAATATCTATTCCTGTTTCTTCGTAAACAGTCTTCTTATTTACGATCAATATCTTCTGGAGTCTTTCTTGAGCGAACACATTTCTATGGAAAAATAGAA------TATCTTATAGT---CGTGTGTTGTAATTCTTTTCAGAGG-AGTCTATGGTTCCTCAAAGATACTTTCATGCATTATGTTCGATATCAAGGAAAAGCAATTCTGTCTTCAAAAGGAACTCTTATTCTGATAAAGAAATGGAAATTTCATCTTGTGAATTTTTGGAAATCTTATTTTCACTTTTGGTTTCAACCTTATAGGATCCATATAAAGCAATTACCCAACTATTCCTTCTCGTTTCT-GGGTTTTTTTTCAAGTTTAC-TGAAAAATCCTTTGGTAGTAAGAAATCAAATGCTAGAGTATTCATTTCTAATAAATACTCTGACTAATAAATTAGATACCATAGTCCCGGTAAATTCTCTTATTGGATCATTGTCGAAAGCTCATTTTTGTACTGTATTGGGTCATCCTATTAGTAAACCGATCTGGACCGATTTATCGGATTCTTATATTATTGATCGATTTTGTCGGATATGTAGAAATCTTTGTCGTTATTACAGCGGATCCTCAAAGAAACAGGTTTTGTATCGTATAAAGTATATACTTCGACTTTCGTGTGCTAGAACTTTGGCTCGTAAACATAAAAGTAC-AGTACGCACTTTTATGCGAAGGTTAGGTTCGGGATTCTTAGAAGAATTTTTTTTGGAAGAAGAACAATCTCTTTCTTGAGCTATCCCGACCATTTCCCGTGCATCACCCTAGCAGAGTACTTA-TATCTATGTCAATGAAAAGAA-CTAAAAAATAAAATCTTAACAA-------------------ATTGAATCTAG--CCCCT-GAATTT----------CTTAG-------ATC-TTCAAAAA----------GAAGAC---TTTTTTT-----------GTAAA-TGTAAGGAAAGAGATATGGACTAT-GAATGA------------TTCAATT---------------------------------------------------------------------------------------------------------------------------------------------------------------------------------------------------------------------------------------------------------------------------------TGTTGAAACT------------GAGCCACTGATGAAAATAGGAT-------------------------------GAGGATAAATA-AAGA------------------GCGAGGAA----------TTTAAAT-----GGGC--TTTTATTGGGGATA-----CCATCTAT---------AAATGGATAATACT--------TTTGTATGTATGCGAATCTTTTAAGATAG--CAAT---------CCTCCAATATCT-TGT---------------TCTTAGAACAAGATATT--GGGGGATTCTTTTG---CTTCTCTATCCG---------------AATTTTCG---------TTCTTTATCAT----AAAAGTTCTC-CCCCGCC---------AATGAATGATAAGT-GCC-----TAGATG-AAGTCT----------------AGTATAAGATAAGTCAGA----------------------AAAGTCTAAGTCTTAGTATAATACCTATACTCTTA----------CTATAA-GAT------------AGAGACTATTAAGGATAAGGC-----TTTTCACATGAATACTTATGAATACTTAGTAGAACGACTAACGACGAGATTTATTATCGTTTCTCGCGTGTCTCACGAAAGTGAGAGTAG-GTGCGAATTCTCCCAATTTGTGACCGACCATACGATCTGTGATATAAATGGTAAA-TGTTCCTTTCCATTATGAATAGC-GATTGTATGGCCAATCATTGTGGGTA-TAATGGTAGATGCCCGAGACCAAGTCACTATGATTTCTTTCTCCTCCCTCCTGTT-GAGTTTTTCAATTCTTCCCGATAAATGATTAGCTACAAAAGGATTTTTTTTT-AGTGAACGTGTCAC-GGCTGATTACTCCTTTTTTTCCATTTTT-TAAATTGGCATTCTATGTCCAA----------TATCTCGATCTTAA-TCTG-AAG-TAT---------------------------AATG-ATGAATGG-AAAAAAGAGA----------------TTTA-GCTAG-ATAA--------GG--AA-GGGGC-GGATGTAG-CCAAGTGG-ATCAA-GGCAGT

Calanthe_dominyi_SG1359 ATGACT-CTCGGCAATGGATATCTCGGCTCTCGCATCGATGAAGAGCGCAGCGAAATGCGATACGTGGTGCGAATTGCAGAATCCCGCGAACCATCGAGTCTTTGAACGCAAGTTGCGCCCGAGGCCAAT-CGGCCAAGGGCACGTCTGCCTGGGCGTCA-AGCGTTGCATCGCTCTACAAGAATTCTTTTTCTTCTCATCTTTCT------------TCTCAAATGGTATCAGAAGGTTTTGGAGTCATTCTGGAAATTCCATTCTCGTCGCGATTAGTATCT---------TCCCTTGAAG---AAAAAAGAATACCAAAATCTCAGAATTTACGATCTATTCATTCAATATTTCCCTTTTTAGAGGATAAATTATCACATTTAAATTATGTGTCAGATCTACTAATACCCCACCCCATCCATCTGGAAATCTTGGTTCAAATCCTTCAATGCTGGATCAAAGATGTTCCTTCTTTGCATTTATTGCGATTGTTTTTCCACGAATATCAT---------------AATTTTAATAGTATC---------ATTACTT------CAAAGAAATC---------------CATTTACGTCTTTTCAAAAAA---AAAGAAAAGATTCTTTTGGTTCCTACATAATTCTTATGTATATGAATGCGAATATATATTCCTGTTTCTTCGTAAACAGTCTTCTTATTTACGATCAATATCTTCTGGAGTCTTTCTTGAGCGAACACATTTCTATGGAAAAATAGAA------TATCTTATAGT---CGTGTGTTGTAATTTTTTTCAGAGG-ATCTTATGGTTCCTCAAAGATACTTTCATACATTATGTTCGATATCAAGGAAAAGCTATTCTGGCTTCAAAAGGAACTTTTATTCTGATGAAGAAATGGAAATTTTATCTTGTAAATTTTTGGGAATCTTATTTTCACTTTTGGTTTCAACCTTATAGGATCCATATAAAACAATTACCCAACTATTCCTTCTCTTTTCT-GGGGTATTTTTCAAGTGTAC-TAAAAAATACTTTGGTAGTAAGAAATCAAATGCTAGAGAATTCATTTCTAATAAATACTCTGACTAATAAATTAGATACCATAGCCCCAGTTATTTCTCTTATTGGATCATTGTCGAAAGCTCAATTTTGTACTGTATTGGGTCATCCTATTAGTAAACCGATCTGGACCGATTTATCGGATTCTGATATTCTTGATCGATTTTGTCGGATATCTAGAAATCTTTGTCATTATCACAGCGGATCCTCAAAGAAACAGGTTTTGTATCGTATAAAGTATATACTTCGACTTTCGTGTGCTAGAACTTTGGCTCGTAAACATAGAAGTAC-AGTACGCACTTTTATGCAAAGATTAGGTTCGGGATTTTTAGAAGAATTTTTTTTGGAAGAAGAACAATCTCTTTCTTGAGCTATCCCGACCATTTCCCGTGCATCATCCTAGCAGAGTACTTA-TATCTATGTCAATGAAAAGAA-TTAAAAAATAAAATCTTAACAA-------------------ATTGGACCTAG--CCCCT-GAATTT----------CTTAG-------ATA-TTCAAAAA----------GAAGAC---ATTCTTT-----------GTAAA-TGTCAGGAAAAAGATATGGACTAT-GAATGA------------TTCAATAAC-GGAAATTCCTTGAAC-----------------------ATATATATGTTCAT-------------ATCGTA----CTAT------ACAAAA--------CAAAT-----GAGA--------TTGGATTGGAA------GAA-----GAT------------------ACGAGGATTTTG------ATTCGGATCCATT------TGTGAAAGAACAGAG--TGAATGAAATGAGAAAG-------ATATTTCATTT--------------TGTTTAAACT------------AAACCACTGATGGAAAGAGGGT-------------------------------------------------------------------GAGGAA----------GTAAAAT-----GGGC-TTTTTATTGGGGATAGAGGG-------------------------------------------------CATGAATTTTTGRAGATAG--CWAT---------CYYSYTRGAT-T-TG------------------------------------GATCATTCTTTTG---CTTCTCTATCCG---------------AATTTTCG---------TTCTTTATCAT----AAAAGTTATC-CCCCGCC---------AATGAATGATAAGT-GCC-----TAGGTG-AAGTAT----------------AGTATAAGATAAGTCAGA----------------------AAAGTATAAGTCTTA-----ATACCTATACTCTTACTCTTN----NTATAA-GAT------------AAAGACTCTGAAGGATAAGGC-----TTTTCAC----------ATGAATACTTAGTAGAACGACTAACGACGAGATTTMTTATCGTTTCTCGCGTGTCTCACGAAAGTGAGAGTAG-GTGCGAATTCTCCCAATTTGTGACCGACCATACGATCTGTGATATAAATGGTAAA-TGTTCCTTTCCATTATGAATAGC-GATTKTATGGCCAATCMTTGTGGGTA-TAATGGTAGATGCCCGAGACCAAGTCACTATGATTTCTTTCTCCTCCCTCCTGTT-GAGTTTTTCAATTCTTCCCGATAAATGATTAGCTACAAAAGKATTTTTTTTT-AGTGAACGTGTCAC-NG--GATTACTCCTTTTTTTACATTTTT-WMAATTGGCATTCTATGTCCAA----------TATCTCGATCTKAA-TCTG-AAG-TAT---------------------------AATG-ATGAATGG-AAAAAAGAGAAAAGCC----------TTTA-GCTAG-ATTA--------GGG-AA-GGGGC-GGATGTAG-CCAAGTGG-ATCAA-GGCAGT

Calanthe_graciliflora_PK12206 ATGACT-CTCGGCAATGGATATCTCGGCTCTCGCATCGATGAAGAGCGCAGCGAAATGCGATACGTGGTGCGAATTGCAGAATCCCGCGAACCATCGAGTCTTTGAACGCAAGTTGCGCCCGAGGCCAAT-CGGCCAAGGGCACGTCTGCCTGGGCGTCA-AGCGTTGCATCGCTCTACAAGAATTCTTTTTCTTCTCATTTTTAT------------TCTCAAATGGTATCAGAAGGTTTTGGAGTCATTCTGGAAATTCCATTCTCGTCGCGATTAGTATCT---------TCCCTTGAAG---AAAAAAGAATACCAAAATCTCATAATTTACGATCTATTCATTCAATATTTCCCTTTTTAGAGGATAAATTATCACATTTAAATTATGTATCAGATCTACTAATACCCCACCCCATCCATCTGGAAATCTTGGTTCAAATCCTTCAATGCTGGATCAAAGATGTTCCTTCTTTGCATTTATTACGATTGTTTTTCCACGAATATCAT---------------AATTTGAATAGTATC---------ATTACTT------CAAAGAAATC---------------CATTTACGTCTTTTCAAAAAG---AACGAAAAGATTCTTTTGGTTCCTACATAATTCTTATGTATATGAATACGAATATCTATTCCTGTTTCTTCGTAAACAGTCTTCTTATTTACGATCAATATCTTCTGGAGTCTTTCTTGAGCGAACACATTTCTATGGGAAAATAGAA------TATCTTATAGT---CGTGTGTTGTAATTTTTTTCAGAGG-ATCCTATGGTTCCTCAAAGATACTTTCATACATTATGTTCGATATCAAGGAAAAGCTATTCTGGCTTCAAAAGGAACTTTTTTTCTGATGAAAAAATGGAAATTTTATCTTGTAAATTTTTGGGAATCTTATTTTCACTTTTGGTTTCAACCTTATAGGATCCATATAAAAAAATTACCCAACTATTCCTTCTCTTTTCT-GGGGTATTTTTCAAGTGTAC-TAAAAAATACTTTGGTAGTAAGAAATCAAATGCTAGAGAATTCATTTCTAATAAATACTCTGACTAATAAATTAGATACCATAGCCCCAGTTATTTCTCTTATTGGATCATTGTCGAAAGCTCAATTTTGTACTGTATTGGGTCATCCTATTAGTAAACCGATCTGGACCGATTTATCGGATTCTGATATTCTTGATCGATTTTGTCGGATATCTAGAAATCTTTGTCATTATCACAGCGGATCCTCAAAGAAACAGGTTTTGTATCGTATAAAATATATACTTCGACTTTCGTGTGCTAGAACTTTGGCTCGTAAACATAAAAGTAC-AGTACGCACTTTTATGCAAAGATTAGGTTCGGGATTTTTAGAAGAATTTTTTTTGGAAGAAGAACAATCTCTTTCTTGAGCTATCCCGACCATTTTCCGTGCATCATCCTAGCAGAGTACTTA-TATCTATGTCAATGAAAAGAA-TTAAAAAATAAAATCTTAACAA-------------------ATTGGACCTAG--CCCCT-GAATTT----------CTTAG-------ATA-TTAAAAAA----------GAAGAC---ATTCTTT-----------GTAAA-TGTCAGGAAAAAGATATGGACTAT-GAATGA------------TTCAATAAC-GGAAATTCCTTGAAC--ATATATATATATGTTCATAATATATATATGTTCAT-------------ATCGTA----CTAT------ACAAAA--------CAAAT-----GAGA--------TTGGATTGGAA------GAA-----GAT------------------ACGAGGATTTTG------ATTCGGATCCATT------TGTGAAAGAACAGAG--TGAATGAAATGAGAAAG-------ATATTTCATTT--------------TGTTTAAACT------------GAACCACTGATGAAAAAAGGGG-------------------------------GAGGAAAAATA-AAAA------------------GGGAGGAA----------GTAAAAG-----GGGT-TTTTTATGGGGAAAAGAGGGCCGTCTAT---------AAATGGATAATACT--------TTTGTATTCATATGAATTTTTGAAGATAG--CAAT---------CCCCCAAGATCT-TGT---------------TCTTAGAACAAGATCTT--GGAGGATTCTTTTG---CTTCTCTATCCG---------------AATTTTTG---------TTCTTTATCAT----AAAAGTTCTC-CCCCGCC---------AATGAATGATAAGT-GCC-----TAGGTG-AAGTAT----------------AGTATAAGATAAGTCAGA----------------------AAAGTATAAGTCTTA-----ATACCTATACTCTTA----------CTATAA-GAT------------AAAGACTCTTAAGGATAAGGC-----TTTTCAC----------ATGAATACTTAGTAGAACGACTAACGACGAGATTTATTATCGTTTCTCGCGTGTCTCACGAAAGTGAGAGTAG-GTGCGAATTCTCCCAATTTGTGACCGACCATACGATCTGTGATATAAATGGTAAA-TGTTCCTTTCCATTATGAATAGC-GATTGTATGGCCAATCATTGTGGGTA-TAATGGTAGATGCCCGAGACCAAGTCACTATGATTTCTTTCTCCTCCCTCCTGTT-GAGTTTTTCAATTCTTCCCGATAAATGATTAGCTACAAAAGGATTTTTTTTT-AGTGAACGTGTCAC-GGCTGATTACTCCTTTTTTTACATTTTT-AAAATTGGCATTCTATGTCCAA----------TATATCGATCTTAA-TCTG-AAG-TAT---------------------------AATG-ATGAATGG-AAAAAAGAGAAAATCC----------TTTA-GCTAG-ATAA--------GGG-AA-GGGG--------------------------------

Calanthe_masuca_SG1360 ATGACT-CTCGGCAATGGATATCTCGGCTCTCGCATCGATGAAGAGCGCAGCGAAATGCGATACGTGGTGCGAATTGCAGAATCCCGCGAACCATCGAGTCTTTGAACGCAAGTTGCGCCCGAGGCCAAT-CGGCCAAGGGCACGTCTGCCTGGGCGTCA-AGCGTTGCATCGCTCTACAAGAATTCTTTTTCTTCTCATTTTTCT------------TCTCAAATGGTATCAGAAGGTTTTGGAGTCATTCTGGAAATTCCATTCTCGTCGCGATTAGTATCT---------TCCCTTGAAG---AAAAAAGAATACCAAAATCTCAGAATTTACGATCTATTCATTCAATATTTCCCTTTTTAGAGGATAAATTATCACATTTAAATTATGTGTCAGATCTACTAATACCCCACCCCATCCATCTGGAAATCTTGGTTCAAATCCTTCAATGCTGGATCAAAGATGTTCCTTCTTTGCATTTATTGCGATTGTTTTTCCACGAATATCAT---------------AATTTTAATAGTATC---------ATTACTT------CAAAGAAATC---------------CATTTACGTCTTTTCAAAAAA---AAAGAAAAGATTCTTTTGGTTCCTACATAATTCTTATGTATATGAATGCGAATATATATTCCTGTTTCTTCGTAAACAGTCTTCTTATTTACGATCAATATCTTCTGGAGTCTTTCTTGAGCGAACACATTTCTATGGAAAAATAGAA------TATCTTATAGT---CGTGTGTTGTAATTTTTTTCAGAGG-ATCTTATGGTTCCTCAAAGATACTTTCATACATTATGTTCGATATCAAGGAAAAGCTATTCTGGCTTCAAAAGGAACTTTTATTCTGATGAAGAAATGGAAATTTTATCTTGTAAATTTTTGGGAATCTTATTTTCACTTTTGGTTTCAACCTTATAGGATCCATATAAAACAATTACCCAACTATTCCTTCTCTTTTCT-GGGGTATTTTTCAAGTGTAC-TAAAAAATACTTTGGTAGTAAGAAATCAAATGCTAGAGAATTCATTTCTAATAAATACTCTGACTAATAAATTAGATACCATAGCCCCAGTTATTTCTCTTATTGGATCATTGTCGAAAGCTCAATTTTGTACTGTATTGGGTCATCCTATTAGTAAACCGATCTGGACCGATTTATCGGATTCTGATATTCTTGATCGATTTTGTCGGATATCTAGAAATCTTTGTCATTATCACAGCGGATCCTCAAAGAAACAGGTTTTGTATCGTATAAAGTATATACTTCGACTTTCGTGTGCTAGAACTTTGGCTCGTAAACATAAAAGTAC-AGTACGCACTTTTATGCAAAGATTAGGTTCGGGATTTTTAGAAGAATTTTTTTTGGAAGAAGAACAATCTCTTTCTTGAGCTATCCCGACCATTTCCCGTGCATCATCCTAGCAGAGTACTTA-TATCTATGTCAATGAAAAGAA-TTAAAAAATAAAATCTTAACAA-------------------ATTGGACCTAG--CCCCT-GAATTT----------CTTAG-------ATA-TTCAAAAA----------GAAGAC---ATTCTTT-----------GTAAA-TGTCAGGAAAAAGATATGGACTAT-GAATGA------------TTCAATAAC-GGAAATTCCTTGAAC-----------------------ATATATATGTTCAT-------------ATCGTA----CTAT------ACAAAA--------CAAAT-----GAGA--------TTGGATTGGAA------GAA-----GAT------------------ACGAGGATTTTG------ATTCGGATCCATT------TGTGAAAGAACAGAG--TGAATGAAATGAGAAAG-------ATATTTCATTT--------------TGTTTAAACT------------AAACCACTGATGGAAAGAGGGG-------------------------------------------------------------------GAGGAA----------GTAAAAG-----GGGC-TTTT----------------CCGTCTAT---------AAATGGATAATACT--------TTTGTATTCATATGAATTTTTGAAGATAG--CAAT---------CCCCCAAGATCT-TGT---------------TCTAAGAACAAGATCTT--GGGGGATNCTTTTG---CTTCTCTATCCG---------------AATTTTTG---------TTCTTTATCAT----AAAAGTTCTC-CCCCGCC---------AATGAATGATAAGT-GCC-----TAGGTG-AAGTAT----------------AGTATAAGATAAGTCAGA----------------------AAAGTATAAGTCTTA-----ATACCTATACTCTTACTCTTA----CTATAA-GAT------------AAAGACTCTTAAGGATAAGGC-----TTTTCAC----------ATGAATACTTAGTRGAACGACTAACGACGAGATTTATTATCGTTTCTCGCGYGTCTCACGAAAGTGAGAGTAG-GTGCGAATTCTCCCAATTTGTGACCGACCATACGATCTGTGATATAAATGGTAAA-TGTTCCTTTCCATTATGAATAGC-GATTGTATGGCCAATCATTGTGGGTA-TAATGGTAGATGCCCGAGACCAAGTCACTATGATTTCTTTCTCCTCCCTCCTGTT-GAGTTTTTCAATTCTTCCCGATAAATGATTAGCTACAAAAGGATTTTTTTTT-AGTGAACGTGTCAC-GGCTGATTACTCCTTTTTTTACATTTTT-AAAATTGGCATTCTATGTCCAA----------TATCTCGATCTTAA-TCTG-AAG-TAT---------------------------AATG-ATGAATGG-AAAAAAGRGAAAATCC----------TTTA-GCTAG-ATAA--------GGG-AA-GGGGC-GGATGTAG-CCAAGTGG-ATCAA-GGCAGT

Calanthe_speciosa_SG1368 ACGACT-CTCGGCAATGGATATCTCGGCTCTCGCATCGATGAAGAGCGCAGCGAAATGCGATACGTGGTGCGAATTGCAGAATCCCGCGAACCATCGAGTCTTTGAACGCAAGTTGCGCCCGAGGCCAAC-CGGCCAAGGGCACGTCTGCCTGGGCGTCA-AGCGTTGCATCGCTCT-----AATTCTTTTTCTTCTCATTTTTCT------------TCTCAAAAGGTATCAGAAGGTTTTGGAGTCATTCTGGAAATTCCATTCTCGTCGCGATTAGTATCT---------TCCCTTGAAG---AAAAAAGAATACCAAAATCTCAGAATTTACGATCTATTCATTCAATATTTCCCTTTTTAGAGGATAAATTATCACATTTAAATTATGTGTCAGATCTACTAATACCTCATCCCATTCATCTGGAAATCTTGGTTCAAATCCTTCAATGCTGGATCAAAGATGTTCCTTCTTTGCATTTATTGCGATTGTTTTTCCACAAATATCAT---------------AATTTGAATAGTATC---------ATTACTT------CAAAAAAATC---------------CATTTACGTTTTTTCAAAAAG---AAAGAAAAGATTCTTTTGGTTCCTACATAATTCTTATGTATATGAATGCGAATATCTATTCCTGTTTCTTCGTAAACAGTCTTCTTATTTACGATCAATATCTTCTGGAGTTTTTCTTGAGCGAACACATTTCTATGGAAAAATAGAA------TATCTTATAGT---CGTATGTTGTAATTTTTTTCAGAGG-ATCCTATGGTTCCTCAAAGATACTTTCATACATTATGTTCGATATCAAGGAAAAGCAATTCTGGCTTCAAAAGGAACTTTTCTTCTGATGAAGAAATGGAAATTTCATCTTGTAAATTTTTGGGAATCTTATTTTCACTTTTGGTTTCAACCTTATAGGATCCATATAAAACAATTACCCAATTATTCCTTCTCTTTTCT-GGGGTATTTTTCAAGTGTAC-TAAAAAATCCTTTGATAGTAAGAAATCAAATGCTAGAGAATTCATTTCTAATAAATACTCTGACTAAGAAATTAGATACCATAGCCCCAGTTATTTCTCTTATTGGATCATTGTCGAAAGCTCAATTTTGTACTGTATTGGGTCATCCTATTAGTAAACCGATCTGGACCAATTTATCGGATTCTGATATTCTTGATCGATTTTGTCAGATATCTAGAAATCTTTGTCGTTATCACAGCGGATCCTCAAAGAAACAGGTTTTGTATCGTATAAAGTATATACTTCGACTTTCGTGTGCTAGAACTTTGGCTCGTAAACATAAAAGTAC-AGTACGCACTTTTATGCGAAGATTAGGTTCGGGATTCTTAGAAGAATTTTTT-TGGAAG------------------GAGCTATCCCGACCATTTCCCGTGCATCATCCTAGCAGAGTACTTA-TATCTATGTCAATGAAAAGAA-TTAAAAAATAAAATATTAACAA-------------------ATTGGACCTAG--CCCCT-GAATTT----------CTTAG-------ATC-TTCAAAAA----------GAAAAC---ATTCTTT-----------GTAAA-TGTCAGGAAAAAGATATGGACTAT-GAATGA------------TTCAATAAC-GGAAATTCCTTGAAC-------------------ATATATATATATGTTCAT-------------ATCGTA----CTAT------ACAAAA--------CAAAT-----GAGA--------TTGGATTGGAA------GAA-----GAT------------------ACGAGGATTTTG------ATTCGGATCCATT------TGTGAAAGAACAGAG--TTAATGAAATGATAAAG-------ATATTTCATTT--------------TGGTTAAACT------------AAACCACTGATGGAAAGAGGAT-------------------------------GAGAATAAATATAAGA--------------------ATGAAA----------------------ACCC---------------------CCATCTAT---------AAATGGATAATACT--------TTTGTATACATATGAATTTTTGAAGATAG--CAAT---------CCCCCAAGATCT-TG------------------------------------GGGGATTCTTTTG---CTTCTCTATCCG---------------AATTTTCG---------TTCTTTATCAT----AAAAGTTCTC-CCCCGCC---------AATGAATGATAAGT-GCC-----TAGGTG-AAGTAT----------------AGTATAAGATAAGTCAGA----------------------AAAGTATAAGTCTTA-----ATACCTATACTCTTACTAGTT---------------------------------------GATAAGGC-----TTTTCAC----------ATGAATACTTAGTAGAACGACTAACGACGAGATTTATTATCGTTTCTCGCGTGTCTCACGAAAGTGAGAGTAG-GTGCGAATTCTCCCAATTTGTGACCGACCATACGATCTGTGATATAAATGGTAAA-TGTTCCTTTCCATTATGAATAGC-GATTGTATGGCCAATCATTGTGGGTA-TAATGGTAGATGCCCGAGACCAAGTCACTATGATTTCTTTCTCCTCCCTCCTGTT-GAGTTTTTCAATTCTTCCCGATAAATGATTAGCTACAAAAGGATTTTTTTTT-AGTGAACGTGTCAC-GGCTGATTACTCCTTTTTTTACATTTTT-AAAATTGGCATTCTATGTCCAA----------TATCTCGATCTTAA-TCTG-AAG-TAT---------------------------AATG-ATGAATGG-AAAAAAGAGAAAATCC----------TTTA-GCTAG-ATAA--------GGG-AA-GGGGC-GGATGTAG-CCAAGTGG-ATCAA-GGCAGT

Calanthe_triplicata_SG1311 ATGACT-CTCGGCAATGGATATCTCGGCTCTCGCATCGATGAAGAGCGCAGCGAAATGCGATACGTGGTGCGAATTGCAGAATCCCGCGAACCATCGAGTCTTTGAACGCAAGTTGCGCCCGAGGCCAAT-CGGCCAAGGGCACGTCTGCCTGGGCGTCA-AGCGTTGCATCGCTCTACAAGAATTCTTTTTCTTCTCATTTTTCT------------TCTCAAATGGTATCAGAAGGTTTTGGAGTCATTCTGGAAATTCCATTCTCGTCGCGATTAGTATCT---------TCCCTTGAAG---AAAAAAGAATACCAAAATCTCAGAATTTACGATCTATTCATTCAATATTTCCCTTTTTAGAGGATAAATTATCACATTTAAATTATGTGTCAGATCTACTAATACCCCACCCCATCCATCTGGAAATCTTGGTTCAAATCCTTCAATGCTGGATCAAAGATGTTCCTTCTTTGCATTTATTGCGATTGTTTTTCCACGAATATCAC---------------AATTTTAATAGTATC---------ATTACTT------CAAAGAAATC---------------CATTTACGTCTTTTCAAAAAA---AAAGAAAAGATTCTTTTGGTTCCTACATAATTCTTATGTATATAAATGCGAATATATATTCCTGTTTCTTCGTAAACAGTCTTCTTATTTACGATCAATATCTTCTGGAGTCTTTCTTGAGCGAACACATTTCTATGGAAAAATAGAA------TATCTTATAGT---CGTGTGTTGTAATTTTTTTCAGAGG-ATCTTATGGTTCCTCAAAGATACTTTCATACATTATGTTCGATATCAAGGAAAAGCTATTCTGGCTTCAAAAGGAACTTTTATTCTGATGAAGAAATGGAAATTTTATCTTGTAAATTTTTGGGAATCTTATTTTCACTTTTGGTTTCAACCTTATAGGATCCATATAAAACAATTACCCAACTATTCCTTCTCTCTTCT-GGGGTATTTTTCAAGTGTAC-TAAAAAATACTTTGGTAGTAAGAAATCAAATGCTAGAGAATTCATTTCTAATAAATACTCTGACTAATAAATTAGATACCATAGCCCCAGTTATTTCTCTTATTGGATCATTGTCGAAAGCTCAATTTTGTACTGTATTGGGTCATCCTATTAGTAAACCGATCTGGACCGATTTATCGGATTCTGATATTCTTGATCGATTTTGTCGGATATCTAGAAATCTTTGTCATTATCACAGCGGATCCTCAAAGAAACAGGTTTTGTATCGTATAAAGTATATACTTCGACTTTCGTGTGCTAGAACTTTGGCTCGTAAACATAAAAGTAC-AGTACGCACTTTTATGCAAAGATTAGGTTCGGGATTTTTAGAAGAATTTTTTTTGGAAGAAGAACAATCTCTTTCTTGAGCTATCCCGACCATTTCCCGTGCATCATCCTAGCAGAGTACTTA-TATCTATGTCAATGAAAAGAA-TTAAAAAATAAAATCTTAACAA-------------------ATTGGACCTAG--CCCCT-GAATTT----------CTTAG-------ATA-TTCAAAAA----------GAAGAC---ATTCTTT-----------GTAAA-TGTCAGGAAAAAGATATGGACTAT-GAATGA------------TTCAATAAC-GGAAATTCCTTGAAC-----------------------ATATATATGTTCAT-------------ATCGTA----CTAT------ACAAAA--------CAAAT-----GAGA--------TTGGATTGGAA------GAA-----GAT------------------ACGAGGATTTTG------ATTCGGATCCATT------TGTGAAAGAACAGAG--TGAATGAAATGAGAAAG-------ATATTTCATTT--------------TGTTTAAACT------------AAACCACTGATGGAAAGAGGGT-------------------------------------------------------------------GAGGAA----------GTAAAAT-----GGGC-TTTT----------------CCGTCTAT---------AAATGGATAATACT--------TTTGTATTCATATGAATTTTTGAAGATAG--CAAT---------CCCCCAAGATCT-TGT---------------TCTAAGAACAAGATCTT--GGGGGATTCTTTTG---CTTCTCTATCCG---------------AATTTTTG---------TTCTTTATCAT----AAAAGTTCTC-CCCCGCC---------AATGAATGATAAGT-GCC-----TAGGTG-AAGTAT----------------AGTATAAGATAAGTCAGA----------------------AAAGTATAAGTCTTA-----ATACCTATACTCTTACTCYTA----CTATAA-GAT------------AAAGACTCTTAAGGATAAGGC-----TTTTCAC----------ATGAATACTTAGTAGAACGACTAACGACGAGATTTATTATCGTTTCTCGCGTGTCTCACGAAAGTGAGAGTAG-GTGCGAATTCTCCCAATTTGTGACCGACCATACGATCTGTGATATAAATGGTAAA-TGTTCCTTTCCATTATGAATAGC-GATTGTATGGCCAATCATTGTGGGTA-TAATGGTAGATGCCCGAGACCAAGTCACTATGATTTCTTTCTCCTCCCTCCTGTT-GAGTTTTTCAATTCTTCCCGATAAATGATTAGCTACAAAAGGATTTTTTTTT-AGTGAACGTGTCAC-GGCTGATTACTCCTTTTTTTRCATTTTT-AAAATTGGCATTCTATGTCCAA----------TATCTCGATCTTAA-TCTG-AAG-TAT---------------------------AATG-ATGAATGG-AAAAAAGAGAAAATYC----------TTTA-GCTAG-ATAA--------GGG-AA-GGGGC-GGATGTAG-CYAAGTGG-ATCAA-GGCAGY

Cephalantheropsis_obcordata_PK12079 ACGACT-CTCGGCAATGGATATCTCGGCTCTCGCATCGATGAAGAGCGCAGCGAAATGCGATACGTGGTGCGAATTGCAGAATCCCGCGAACCATCGAGTCTTTGAACGCAAGTTGCGCCCGAGGCCAAC-CGGCCAAGGGCACGTCTGCCTGGGCGTCA-AGCGTTGCATCGCTCTCCAAGAATTCTTTTTCTTCTCATTTTTCT------------TCTAAAATGGTATCAGAAGGCTTTGGAGTCATTCTGGAAATTCCATTCTCGTCGCGATTAGTATCT---------TCCCTTGAAG---AAAAAAGAATACCAAAATCTCAGAATTTACGATCTATTCATTCAATATTTTCCTTTTTAGAGGATAAATTATCACATTTAAATTATGTGTCAGATCTACTAATACCTCATCCCATCCATCTGGAAATCTTGGTTCAAATCCTTCAATGCTGGATCAAAGATGTTCCTTCTTTGCATTTATTGCGATTGTTTTTCCACAAATATCAT---------------AATTTGAATAGTATC---------ATTACTT------CAAAGAAATC---------------CATTTACGTCTTTTCAAAAAG---AAATAAAAGATTCTTTTGGTTCCTACATAATTCTTATGTATATGAATGCGAATATCTATTCCTGTTTCTTCGTAAAAAGTCTTCTTATTTACGATCAATATCTTCTGGAGTCTTTCTTGAGCGAACACATTTCTATGGAAAAATAGAA------TATCTTATAGT---CGTATGTTGTAATTTTTTTCAGAGG-ATCCTATGGTTCCTCAAAGATACTTTCATACATTATGTTCGATATCAAGGAAAAGCAATTCTGGCTTCAAAAGGAACTTTTATTGTGATGAATAAATGGAAATTTCATCTTGTAAATTTTTGGGAATCTTATTTTCACTTTTGGTTTCAACCTTATAGGATCCATATAAAACAATTACCCAACTATTCTTTTTCTTTTCT-GGGGTATTTTTCAAGTGTAT-TAAAAAATCCTTTGGTAGTAAGAAATCAAATGCTAGAGAATTCATTTCTAATAAATAATCTGACTAAGAAATTAGATACCATAGCCCCAGTTATTTCTCTTATTGGATCATTGTCGAAAGCTCAATTTTGTACTGTATTGGGTCATCCTATTAGTAAACCGATCTGGACCAATTTATCGGATTCTGATATTCTTGATCGATTTTGTCAGATATCTAGAAATCTTTGTCGTTATCACAGCGGATCCTCAAAGAAACAGGTTTTGTATCGTATAAAGTATATACTTCGACTTTCGTGTGCTAGAACTTTGGCTCGTAAACATAAAAGTAC-AGTACGCATTTTTATGCGAAGATTAGGTTCGGGATTCTTAGAAGAATTTTTTTTGGAAGAAGAACAATCT-------GAGCTATCCCGACCATTTTCCGTGCATCATCCTAGCAGAGTACTTA-TATCTATGTCAATGAAAAGAA-TTAAAAAATAAAATCTTAACAA-------------------ATTGGACCTAG--CCCCT-GAATTT----------TTTAG-------ATC-TTCAAAAA----------GAGGAC---ATTCTTT-----------GTAAA-TGTCAGGAAAAAGATATGGACTAT-GAATGA------------TTCAATAAC-GGAAATTCCTTGAAC-----------------ATATATATATATATGTTTAT-------------ATCGTA----CTAT------ACAAAA--------CAAAT-----GAGA--------TTGGATTGGAA------GAA-----GAT------------------ACGAGGATTTTG------ATTCGGATCCATT------TGTGAAAGAACAGAG--TGAATGAAATGAGAAAG-------ATATTTCATTT--------------TGGTTAAACT------------GAACCACTGATGGAAAGAGGAT-------------------------------GAGAATAAATA-AAGA------------------GTGAGGAA----------GTAAAAT-----GGAC-TTTTTATTGGGGATAGAGGGCCATCTAT---------AAATGGATAATACT--------TTTGTATACATATGAATTTTTGAAGATAG--CAAT---------CCCCCAAGATCT-TG------------------------------------GGGGATTCTTTTG---CTTCTCTATCCG---------------AATTTTCG---------TTCTTTATCAT----AAAAGTTCTC-CCCCGCC---------AATGAATGATAAGT-GCC-----TAGGTG-AAGTAT----------------AGTATAAGATAAGTCAGA----------------------AAAGTATAAGTCTTA-----ATACCTATACTCTTA----------CTATAA-GAT------------AAAGACTCTTAAGGATAAGGC-----TTTTCAC----------ATGAATACTTAGTAGAACGACTAACGACGAGATTTATTATCGTTTCTCGCGTGTCTCACGAAAGTGAGAGTAG-GTGCGAATTCTCCCAATTTGTGACCGACCATACGATCTGTGATATAAATGGTAAA-TGTTCCTTTCCATTATGAATAGC-GATTGTATGGCCAATCATTGTGGGTA-TAATGGTAGATGCCCGAGACCAAGTCACTATGATTTCTTTCTCCTCCCTCCTGTT-GAGTTTTTCAATTCTTCCCGATAAATGATTAGCTACAAAAGGATTTTTTTTT-AGTGAACGTGTCAC-GGCTGATTACTCCTTTTTTTACATTTTT-AAAATTGGCATTCTATGTCCAA----------TATCTCGATCTTAA-TCTG-AAG-TAT---------------------------AATG-ATGAATGG-AAAAAAGAGAAAATCC----------TTTA-GCTAG-ATAA--------GGG-AA-GGGGC-GGATGTAG-CCAAGTGG-ATCAA-GGCAGT

Cheirostylis_clibborndyeri_SG1349 ATGACT-CTCGGCAATGGATATCTTGGCTCTTGCATCGATGAAGAGCGCAGCGAAATGCGATACGTGGTGTGAATTGCAGAATCCCGTGAACCATCAAATATTTGAACGCAAGTTGCGCCCGAGGCCAAT-TGGCTAAGGGCACGTCCGCCTGGGCGTCA-AGCATTATATCGCTTC----------------------------------------------------------------------------------------------------------------------------------------------------------------------------------------------------------------------------------------------------------------------------------------------------------------------------------------------------------------------------------------------------------------------------------------------------------------------------------------------------------------------------------------------------------------------------------------------------------------------------------------------------------------------------------------------------------------------------------------------------------------------------------------------------------------------------------------------------------------------------------------------------------------------------------------------------------------------------------------------------------------------------------------------------------------------------------------------------------------------------------------------------------------------------------------------------------------------------------------------------------------------------------------------------------GAGCTATCCCGACCAGTACTC-TGCATCATCCTAGCAGAGTACTTG-TATCTATTATCTATAAAAGAA-CTAAAAAAGAAAGTCTTAACAA-------------------ATTGGACCTAG-TCCTCTTTAATTT----------CTTAG-------ATC-TTCAAAAA----------GAAGAC---TTTCTTT-----------TTAAA-TGAAAAGATAATGATATGAACTGT-GAAGAATTAAATGAATTATTCAATAAG-GGAGATTCTTTGAAC-------------------------ATATATGTTCAT-TTGTACAGAT--ATCGTA---TCTAT------ACAAAGAAAAA---CAAAG-----AAAA--------TTGGATTGGAATTTGAAGAA-----GATAGGAAT------------AGGAGGATTTCT------ATTTGGATCCTTT------TGTGAAAGAACAGGG--TGAATAAATTTTGAAAG-------ATATTTAATTT--------------TGTTTGAACT------------CAACAACTGATAAAAATAGGAT-------------------------------GAGAGTAAAGA-AAGA------------------GTGAGGAA----------TAAAAAT-----GGGC--TTTTTTTGGGGATAGAGGACTATCTAC---------AAATGGATAATACT--------TTTGTATTTCGATGAATTCTTTAAGGTAG--CAAT----------CCCCAATATC----------------------------CAATATATT---GGGGATTCTTTTG---CTTCTTTATCCG------ATTTCTCCAAATTTTCG---------TTCTTTATCATAAAAGAAAGTTCTC-CCCCGCC---------AATGAATGATAAGT-GCC-----TAGGTG-AAGTAT----------------AGTATAAGATAAGTAAGA----------------------AAAATCTAAGTCTTAGTATAATACCTATACTTTTA----------CTATAA-GAT------------AAAGACTCTTAAGGATAAGAC-----TTTTCAC----------ATGAATACTTAGTAGAACGACTAACGACGAGATTTATTATCATTTCTCGTATGTCTCACGAAAGTGAGAGTAG-GTGCGAATTCTCCCAATTTGTGACCGACCATACGATCTGTTATWTAAATGGTAAA-TGTTCCTTTCCATTATGAATAGC-GATTGTATGGCCAATCATTGTGGGTA-TAATGGTAGATGCCCGAGACCAAGTCACGATTATTTCTTTCTCCTCCCTCATGTT-GAGTTTTTCAATTTTTACCGATAAATGATTAGCTACAAAAGGATTTTTTTTT-AGTGAACGTGTCAC-GGCCGATTACTCCNTTTTTTACATTTTT-GAAATTGGCATTCTATGTCCAA--TAGTCCAATATCTCGATCTTAA-TCTG-AAG-TAT-GAGGGTAAGAATCAATACAAT-----AATG-ATGAATGG-AAAAAATAGAAAATAC----------TTTA-GCTAG-ATAA--------GGG-AA-GGGGC-GGATGTAG-CCAAGTGG-ATCAA-GGCAGT

Cheirostylis_jamesleungii_PK12205 ATGACT-CTCGGCAATGGATATCTTGGCTCTTGCATCGATGAAGAGCGCAGCGAAATGCGATACGTGGTGTGAATTGCAGAATCCCGTGAACCATCAAATATTTGAACGCAAGTTGCGCCTGAGGCCAAT-TGGCTAAGGGCACGTCCGCCTGGGCGTCA-AGCATTTTATCGCTTC----------------------------------------------------------------------------------------------------------------------------------------------------------------------------------------------------------------------------------------------------------------------------------------------------------------------------------------------------------------------------------------------------------------------------------------------------------------------------------------------------------------------------------------------------------------------------------------------------------------------------------------------------------------------------------------------------------------------------------------------------------------------------------------------------------------------------------------------------------------------------------------------------------------------------------------------------------------------------------------------------------------------------------------------------------------------------------------------------------------------------------------------------------------------------------------------------------------------------------------------------------------------------------------------------------GAGCTATCCCGACCAGTACTC-TGCATCATCCTAGCAGAGTACTTG-TATCTATTATCTATAAAAGAA-CTAAAAAAGAAAGTCTTAACAA-------------------ATTGGACCTAG-TCCTCTTTAATTT----------CTTAG-------ATC-TTCAAAAA----------GAAGAC---TTTCTTT-----------TTTAA-TTAAAAGATAATGATATGAACTGT-GAAGAATTAAATGAATTCTTCAATAAG-GGAGATTCTTTGAAC-------------------------ATATATGTTCAT-TTGTACAGGT--ATTGTA---TCTAT------ACAAAGAAAAA---CAAAG-----AAAA--------TTGGATTGGAATTGTAAGAA-----GATAGGAAT------------AGGAGGATTTCT------ATTTGGATCCTTT------TGTGAAAGAACAGGG--TGAATAAAATTTGAAAG-------ATATTTAATTT--------------TGTTTGAACT------------GAACAACTGATAGGATGAGAGT-------------------------------AAAGAAAAAGA-AAGA------------------GTGAGGAA----------TAAAAAT-----GGGC---TTTTTTGGGGATAGAGGGCTATCTAC---------AAATGGATAATACT--------TTTGTATTGAGATGAATTCTTTAAGGTAG--CAAT----------CCCCAATCTAT-T----------------------------TAGATT---GGGGATT-TTTTG---CTTCTTTATCCG------ATTTCTCCAAATTTTCG---------TTCTTTATCATAAAAGAAAGTTCTC-CCCCGCC---------AATGAATGATAAGT-GCC-----TAGGTG-AAGTAT----------------AGTATAAGATAAGTAAGA----------------------AAAATCTCAGTCTTAGTATAATACCTATACTTTTA----------CTATAA-GAT------------AAAGACTCTTAAGGATAAGAC-----TATTCAA----------ATGAATACTTAGTAGAACGACTAACGACGAGATTTATTATCATTTCTCGCATGTCTCACGAAAGTGAGAGTAG-GTGCGAATTCTCCCAATTTGTGACCGACCATACGATCTGTTATATAAATGGTAAA-TGTTCCTTTCCATTATGAATAGC-GATTGTATGGCCAATCATTGTGGGTA-TAATGGTAGATGCCCGAGACCAAGTCACAATTATTTCTTTCTCCTCCCTCATGTT-GAGTTTTTCAATTTTTACCGATAAATGATTAGCTACAAAAGGATTTTTTTTT-AGTGAACGTGTCAC-GGCCGATTACTCCTTTTTTTACATTTTT-GAAATTGGCATTCTATGTCCAA----------TATCTCGATCTTAA-TCTG-AAG-TAT-GAGGGTAAGAATCAATACAAT-----AATG-ATGAATGG-AAAAAAGAGAAAATAC----------TTTA-GCTAG-ATAA--------GGG-AA-GGGGC-GGATGTAG-CCAAGTGG-ATCAA-GGCAGT

Cheirostylis_monteiroi_SG1344 ATGACT-CTCGGCAATGGATATCTTGGCTCTTGCATCGATGAAGAGCGCAGCGAAATGCGATACGTGGTGTGAATTGCAGAATCCCGTGAACCATCAAATATTTGAACGCAAGTTGCGCCCGAGGCCAAT-TGGCTAAGGGCACGTCCGCCTGGGCGTCA-AGCATTATATCGCTTCATAAGAATTCTTTTTCTTCTCATTTTTCT------------TTTCAAATACTATCAGAAGTTTTTGGAGTCGTTCTGGAAATTCCATTATCATCGCGATTAGTATCC---------TCCCTTGAAG---AAAAAAAAAGACTAAAATCTCAGAGTTTACGATCTATTCATTCAATATTTCCTTTTTTAGAAGATAAATTATCATATTCCAATTTTGTGGCAGATCTACTAATACCCCATCCTATCCATCTAGAAATCTTGGTTCAAATTCTTCAATGCTGGATCAAAGATGTTCCTTCTTTGCATTTGTTGCGATTGATTTTTTACGAATGTCAT---------------AATTTGAAGAGTATC---------ATTAC---------------------------------------------TTCAAAGAA---AAAGAAAAGATTTTTTTGGTTCCTACATAATTTTTATGTATATGAATGTGAATATCTCTTTCTKTTTCTTCGTAAAAAGTCTTCTTATTTACGATCAACATCTTTTAGATTCTTTATTGAGCGAACATTTTTTTATGTAAAAATGGAA------TCTATTCTAGT---AGTATATTTTAATCCTTTTCATAGG-ATTCTCTGGTTCCTCAAAGATTCTTTCATACATTATGTTCGATATCAAGGAAAAGTAATTATGGCTTCAAAGGGAACTCCTATTCTGATGAAGAAATGGAATTTTCATGTTGTGAATTTTTGGCAATTTTATTTTCACTTYTGGTCTCAACCTTATAGGATCCATATAAGGCAATTACCCAACTATTCCTTCTCCTTTCT-GGGTTATTTTTTACATGTAC-GAAAAAAAACTTTGTTAGTAAGAAATCAAATGCTAGAGAATTCCTTTCTAATAAATGCTCTGACTAAGAAATTAGATACCATAGTACCAGTTATTTCTCTTATTGGATCATTGTCAAAAGCTCAATTTTGTACTATATCGGGTCATCCTATTAGTAAACCAATATGGACCGATTTATCGGATTCTGATATTCTTGATCGATTTTCTCAAAAATGTAAAAATCTTTGTCGTTATCACAGCGGATCTTC-AAAAAAAAAGTTTTGTATCGTATAAAATATATACTTCGACTTTCGTGTGCTAGAACTTTGGCTCGTAAACATAAAAGTAC-AGTACGCACTTTTATGCAAAGATTGGGTTCGGTATTTTTAGAAGAATTTTTTATGGAAGAAGAACAAGCTCTTTCTTGAGCTATCCCGACCAGTACTC-TGCATCATCCTAGCAGAGTACTTG-TATCTATTATCTATAAAAGAA-CTAAAAAAGAAAGTCTTAACAA-------------------ATTGGACCTAG-TCCTCTTTAATTT----------CTTAG-------ATC-TTCAAAAA----------GAAGAC---TTTCTTT-----------TTAAA-TGAAAAGATAATGATATGAACTGT-GAAGAATTAAATGAATTATTCAATAAG-GGAGATTCTTTGAAC-------------------------ATATATGTTCAT-TTGTACAGAT--ATCGTA---TCTAT------ACAAAGAAAAA---CAAAG-----AAAA--------TTGGATTGGAATTTGAAGAA-----GATAGGAAT------------AGGAGGATTTCT------ATTTGGATCCTTT------TGTGAAAGAACAGGG--TGAATAAATTTTGAAAG-------ATATTTAATTT--------------TGTTTGAACT------------CAACAACTGATAAAAATAGGAT-------------------------------GAGAGTAAAGA-AAGA------------------GTGAGCAA-----------------------------------------------CTATCTAC---------AAATGGATAATACT--------TTTGTATTTCGATGAATTCTTTAAGGTAG--CAAT----------CCCCAATATC----------------------------CAATATATT---GGGGATT-TTTTG---CTTCTTTATCCG------ATTTCTCCAAATTTTCG---------TTCTTTATCATAAAAGAAAGTTCTC-CCCCGCC---------AATGAATGATAAGT-GCC-----TAGGTG-AAGTAT----------------AGTATAAGATAAGTAAGA----------------------AAAATCTAAGTCTTAGTATAATACCTATACTTTTA----------CTATAA-GAT------------AAAGACTCTTAAGGATAAGAC-----TTTTCAC----------ATGAATACTTAGTAGAACGACTAACGACGAGATTTATTATCATTTCTCGTATGTCTCACGAAAGTGAGAGTAG-GTGCGAATTCTCCCAATTTGTGACCGACCATACGATCTGTTATATAAATGGTAAA-TGTTCCTTTCCATTATGAATAGC-GATTGTATGGCCAATCATTGTGGGTA-TAATGGTAGATGCCCGAGACCAAGTCACGATTATTTCTTTCTCCTCCCTCATGTT-GAGTTTTTCAATTTTTACCGATAAATGATTAGCTACAAAAGGATTTTTTTTT-AGTGAACGTGTCAC-GGCCGATTACTCCTTTTTTTACATTTTT-GAAATTGGCATTCTATGTCCAA--TAGTCCAATATCTCGATCTTAA-TCTG-AAG-TAT-GAGGGTAAGAATCAATACAAT-----AATG-ATGAATGG-AAAAAATAGAAAATAC----------TTTA-GCTAG-ATAA--------GGG-AA-GGGGC-G-----------------------------

Cheirostylis_pusilla_HK43263 ATGACT-CTCGGCAATGGATATCTTGGCTCTTGCATCGATGAAGAGCGCAGCGAAATGCGATACGTGGTGTGAATTGCAGAATCCCGTGAACCATCAAATATTTGAACGCAAGTTGCGCCCGAGGCCAAT-TGGCTAAGGGCATGTCCGCCTGGGCGTCA-AGCTTTATATCGCTTCATAATAATTCTTTTTCTTCTCATTTTTCT------------TTTCAAATACTATCAGAAGGTTTTGGAATCGTTCTGGAAATTCCATTATCATCGCGATTAGTATCC---------TCCCTTGAAG---AAAAAAAAATACTAAAATCTCAAAGTTTACGATCTATTCATTCAATATTTCCTTTTTTAGAAGATAAATTATCATATTCCAATTTTGTGTCAGATCTACTCATACCCTATCCTATCCATCTAGAAATCTTGGTTCAAATTCTTCAATGCTGGATCAAAGATGTTCCTTCTTTGCATTTGTTGCGATTGATTTTTCACGAATATCAT---------------AATTTGAAGAGTATT---------ACTACTT------CAAAGAAATCCATTTCAAAGAAATTCATTCGCGTCTTTTCAAAAAA---AAAGAAAAGATTTTTTTGGTTCCTACATAATTTTTATGTATATGAATGTGAATCTCTCTTTCTGTTTCTTCATAAAAAGTCTTCTTATTTACGATCAACATCTTTTGGATTCTTTATTGAGCGAACACTTTTTTATGTAAAAATGGAA------TCTATTCTAGC---AGTATATTTTAATACTTTTCATAGG-ATTCTCTGGTTCCTCAAAGATTCTTTCTTTCATTATGTTCGATATCAAGGAAAAGTAATTATGGCTTCAAAGGGAACTCTTATTCTGATGAAGAAATGGAATTTTCATGTTGTGAATTTTTGGCAATTTTATTTTCACTTTTGGTCTCAACCTTATAGGATCCATATAAGGCAATTACCCAACTATTCCTTCTCCTTTCT-GGGGTATTTTTTATGTGTACAAAAAAAAAACTTTGTTAGTAAGAAATCAAATGCTAGAGAATTCCTTTCTAATAAATACTCTGACTAAGAAATTAGATACCATAGTCCCAGTTATTTCTCTTATTGGATCATTGTCAAAAGCTCAATTTTGTACTATATCGGGTAATCCTATTAGTAAACCAATATGGACCAATTTATCCGATTCTGATATTCTTGATCAATTTTATCAAAAATATAAAAATCTTTGTCGTTATCACAGTGGATCTTCAAAAAAAAAAGTTTTGTATCGTATAAAATATATACTTCGACTTTCGTGTGCTAGAACTTTGGCTCGTAAACATAAAAGTAC-AGTACGCACTTTTATGCAAAGATTGGTTTCGGTATTCTTAGAAGAATTTTTTATGGAAGAAGAACAAGCTCTTTCTTGAGCTATCCCGACCAGTACTC-TGCATCATCCTAGCAGAGTACTTG-TATCTATTATCTATAAAAGAA-CTAAAAAAGAAAGTCTTAACAA-------------------ATTGGACCTAG-TCCTCTTTAATTT----------TTTAT-------ATC-TTCAAAAA----------GAAGAC---TTTCTTT-----------TTAAA-TGAAAAGATAATGATATGAACTGT-GAATAATTAAATGAATTATTAAATAAG-GAAGATTCTTTGAAC-------------------------ATATATGTTCAT-TTGTACAGGT--ATCGTA---TCTAT------ACAAAGAAAAA---CAAAG-----AAAA--------TTGGATTGGAATTGGAAGAA-----GATAGGAAT------------AGGAGGATTTCT------ATTTGGATCCTTT------TGTGAAAGAACAGGG--TGAATAAAATTGGAAAG-------ATATTTAATTT--------------TGTTTGAACT------------GAACAACTGATAAAAATAGGAT-------------------------------GAGAGTAAAGA-AAGA------------------GTGAGGAA----------TAAAAAT-----GGGC--TTTTTTTGGGGATAGAGG-CCATCTAC---------AAATGGATAATACT--------TTTGTATTTAGATGAATTCTTTAAGGTAG--CAAT----------CCCCAATATA----------------------------TTGGATATT---GGGGATT-TTTTG---CTTCTTTCTCCG------ATTTCTCCGAATTTTCG---------TTCTTTATCATAAAAGAAAGTTCTC-CTCCGCC---------AATGAATGATAAGT-GCC-----TAGGTG-AAGTAT----------------AGTATAAGATAAGTAAGA----------------------AAAATCTAAGTCTTAGTATAATACCTATACTTTTA----------CTATAA-GAT------------AAAGACTAT----GATAAGAC-----TTTTCAC-----------------------AGTTCTACTAACGACGAGATTTATTATCATTTCTCGCATGTCTCACGAAAGTGAGAGTAG-GTGCGAATTCTCCCAATTTGTGACCGACCATACGATCTGTTATATAAATGGTACA-TGTTCCTTTCCATTATGAATAGC-GATTGTATGGCCAATCATTGTGGGTA-TAATGGTAGATGCCCGAGACCAAGTCACTATTATTTCTTTCTCCTCCCTCATGTT-GAGTTTTTCAATTTTTACCGATAAATGATTAGCTACAAAAGGATTTTTTTTT-AGTGAACGTGTCAC-GGCCGATTACTCCTTTTTTTACATTTTT-GAAATTGGCATTCTATGTCCAA----------TATCTCGATCTTAA-TCTG-AAG-TAT-GAGGGTAAGAATCAATACAAT-----AATG-ATGAATGG-AAAAAATAGAAAAGAC----------TTTA-GCTAG-ATAA--------GGG-AA-GGGGC-GGATGTAG-CCAAGTGG-ATCAA-------

Cheirostylis_yunnanensis_SG1227 ATGACT-CTCGGCAATGGATATCTTGGCTCTTGCATCGATGAAGAGCGCAGCGAAATGCGATACGTGGTGTGAATTGCAGAATCCCGTGAACCATCAAATATTTGAACGCAAGTTGCGCCCGAGGCCAAT-TGGCTAAGGGCACGTCCGCCTGGGCGTCA-AGCATTATATCGCTTCATAAGAATTCTTTTTCTTCTCATTTTTCT------------TTTCAAATACTATCAGAAGGTTTTGGAGTCGTTCTGGAAATTCCATTATCATCGCGATTAGTATCC---------TCCCTTGAAG---AAAAAAAAATAATAAAATCTCAGAGTTTACGATCTATTCATTCAATATTTCCTTTTTTAGAAGATAAATTCTCATATTCCAATTTTGTGTCAGATATACTAATACCCCATCCTATCCATCTAGAAATCTTAGTTCAAATTCTTCAATGTTGGATCAAAGATGTTCCTTCTTTGCATTTGTTGCGATTGATTTTTCACGAATATCAT---------------AATTTGAAGAGTATC---------ATTACTT------CAAATAAATC---------------CATTCACGTCTTTTCAAAAAA---AAAGAAAAGATTTTTTTGGTTCCTACATAATTTTTATGTATATGAATGTGAATATCTCTTTCTGTTTCTTCGTAAAAAGTCTTTTTATTTACGATCAACATCTTTTGGATTCTTTATTGAGCGAACACTTTTTTATGTAAAAATGGAA------TCTATTCTAGT---AGTATATTTTAATCCTTTTCATAGG-ATTCTCTGGTTCCTCAAAGATTCTTTCRTACATTATGTTCGATATCAAGGAAAAGTAATTATGGCTTCAAAGGGAACTCTTATTCTGATGAAGAAATGGAATTTTCATGTTGTGAATTTTTGGCAATTTTATTTTCACTTTTGGTCTCAACCTTATAGGATCCATATAAGGCAATTACCCAACTATTCCTTCTCCTTTCT-GGGGTATTTTTTACGTGTAC--AAAAAAAACTTTGTTACTAAGAAATCAAATGCTAGAGAATTCCTTTCTAATCAATATTCTGACTAAGAAATTAGATACCATAGTCCCAGTTATTTCTCTTATTGGATCATTGTCAAAAGCTCAATTTTGTACTATATCGGGTCATCCTATTAGTAAMCCAATATGGACTGATTTATCAGATTCTGATATTCTTGATCGATTTTATCAAAAATGTAGAAATCTTTGTCGTTATCACAGTGGCTCTTCAAAAAAAAAAGTTTTGTATCGTATAAAATATATACTTCGACTTTCGTGTGCTAGAACTTTGGCTCGTAAACATAAAAGTAC-AGTACGCACTTTTATGCAAAGATTGGGTTCGGTATTTTTAGAAGAATTTTTTATGGAAGAAGAACAAGCTCTTTCTTGAGCTATCCCGACCAGTACTC-TGCATCATCCTAGCAGAGTACTTG-TATCTATTATCTATAAAAGAA-CTAAAAAAGAAAGTCTTAACAA-------------------ATTGGACCTAG-TCTTCTTTAATTT----------CTTAG-------ATC-TTCAAAAA----------GAAGAC---TTTCTTT-----------TGAAA-TGAAAAGATAATGATATGAACTGT-GAATAATGAAATGAATTATTCAATAAG-GGAGATTCTTTGAAC-------------------------ATATATGTTCAT-TTGTACAGGT--ATCGTT---TCTAT------ACAAAGAAAAA---CAAAG-----AAAA--------TTGGATTGGAATTGGAAGAA-----GATAGGAAT------------AGGAGGATTTCT------ATTTAAATCCTTT------TGTGAAAGAACAGGG--TGAATAAAATTTGAAAG-------ATATTTAATTT--------------TATTTGAACT------------GAACAACTGATAAAAATAGGAT-------------------------------GAGAGTAAAGA-AAGA------------------GTGACGAA----------TAAAAAT-----GGGC--TTTTTTTGGGGATAGAGGACTATCTAC---------AAATGGATAATACT--------TTTGTATTTCAATGAATTCTTTAAGGTAG--CAAT----------CCCCAATATC---------------------CAATATATTGGATATT---GGGGATT-TTTTG---CTTCTTTATCCG------ATTTCTCCGAATTTTTG---------TTCTTTATCATAAAAGAAAGTTCTC-CCCCGCC---------AATGAATGATAAGT-GCC-----TAGGTG-AAGTAT----------------AGTATAAGATAAGTAAGA----------------------AAAATCTAAGTCTTAGTATAATACCTATACTTTTAATACTTTTA-CTATAA-GAG------------AAAGACTCTTAAGGATAAGAC-----TTTTCAC----------ATGAATACTTAGTAGAACGACTAACGACGAGATTTATTATCATTTCTCGCATGTCTCACGAAAGTGAGAGTAG-GTGCGAATTCTCCCAATTTGTGACCGACCATACGATCTGTTATATAAATGGTAAA-TGTTCCTTTCCATTATGAATAGC-GATTGTATGGCCAATCATTGTGGGTA-TAATGGTAGATGCCCGAGACCAAGTCACTATTATTTCTTTCTCCTCCCTCATGTT-GAGTTTTTCAATTTTTACCGATAAATGATTAGCTACAAAAGGATTTTTTTTT-AGTGAACGTGTCAC-GGCCGATTACTCCTTTTTTTACATTTTT-TAAATTGGCATTCTATGTCCAA----------TATCTCGATCTTAA-TCTG-AAG-TAT-GAGGGTAAGAATCAATACAAT-----AATG-ATGAATGG-AAAAAATAGAAAATAC----------TTTA-GCTAG-ATAA--------GGG-AA-GGGGC-GGATGTAG-CCAAGTGG-ATCAA-GGCAGT

Chrysoglossum_assamicum_SG1622 ACGACT-CTCGGCAATGGATATCTCGGCTCTCGCATCGATGAAGAGCGCAGCGAAATGCGATACGTGGTGCGAATTGCAGAATCCCGCGAACCATCGAGTCTTTGAACGCAAGTTGCGCCCGAGGCCAAC-CGGCCAAGGGCACGTCTGCCTGGGCGTCA-AGCGTTGCGTCGCTCCACAAGAATTCTTTTTCTTCTCATTTTTCT------------TTTCAAATGGTATCAGAAGGTTTTGGAGTCATTCTGGAAATTCCATTCTCGTCGCGATTAGTATCT---------TCCCTTGAAGAAAAAAAAAGAATACCAAAATCTCAGAATTTACGATCTATTCATTCAATATTTCCCTTTTTAGAGGATAAATTATCACATTTAAATTATGTGTCAGATCTACTAATACCCCATCCCATCCATCTGGAAATCTTGGTTCAAATCCTTCAATGCTGGATCAAAGATGTTCCTTCTTTGCATTTATTGCGATTATTTTTCCACGAATATCAT---------------AATTTGAATAGTCTC---------ATTACTT------CAAAGAAATC---------------CATTTATGTCTTTTCAAAAAG---AAAGAAAAGATTCTTTTTGTTCCTACATAATTCTTATGTATATGAATGCGAATATCTATTCCTGTTTCTTCGTAAACAGTCTTCTTATTTACGATCAATATCTTCTGGAGTCTTTCTTGAGCGAACACATTTCTATGGAAAAATAGAA------TATCTTATAGT---CGTGTGTTGTAATTCTTTTCAGAGG-ATCCTATGGTTCCTCAAAGATACTTTCATACATTATGTTCGATATCAAGGAAAAGCGATTCTGGCTTCAAAAGGAACTCTTATTCTGATGAAGAAATGGAAATATCATCTTGTAAATTTTTGGCAATCTTATTTTCACTTTTGGTTTCAACCTTATAGGATCCATATAAAGCAATTACCCAACTATTCCTTCTCTTTTCT-GGGGTATTTTTCAAGTGTAC-TAAAAAATCCTTTGGTAGTAAGAAATCAAATGCTAGAGAATTCATTTCTAATAAATACTCTGACTAAGAAATTAGATACTATAGCCCCAGTTATTTCTCTTATTGGATCATTGTCAAAAGCTCAATTCTGTACTGTATTGGGTCATCCTATTAGTAAACCAATCTGGACCGATTTCTCGGATTCTGATATTCTTGATCGATTTTGTCGGATATCTAGAAATCTTTGTCGTTATCACAGCGGATCCTCAAAGAAACAGGTTTTGTATCGTATAAAGTATATACTTCGACTTTCGTGTGCTAGAACTTTGGCTCGTAAACATAAAAGTAC-AGTACGCACTTTTATGCGAAGATTAGGTTCGGGATTCTTAGAAGAATTCTTT-TGGAAGAAG---------------GAGCTATCCCGACCATTTCCCGCGCATCATCCTAGCAGAGTACTTA-TATCTATGTCAATGAAAAGAA-TTTAAAAAGAAGATCTTAACAA-------------------ATTGGACCTAG--CCCCT-GAATTT----------CTTAG-------ATC-TTCAAAAA----------GAAGAC---ATTCTTT-----------GTAAA-TGTCAGGAAAAATATATGGACTAT-GAATGA------------TTCAATAAC-GGAAATTCCTTGAAC---ATATATATGTTCATATCGTCATATATATGTTCATATCGTA-----------------CTAT------ACAAAA--------CAAAT-----GAGA--------TTGGATTGGAA------GAA-----GATATAATTTGAACTGGTGACACGAGGATTT----------TCAGT-CCTCT------GCTCTACCAACTGAG-----CTATCCTGACCA-----------TTTTCCGTG---------------CATCATCCTA-----------GTTTTTTACTTGGAAAGAGGAT-------------------------------GAGGATAAATA-AAGA------------------GTGAGGAA----------GTAAAAT-----GGGC-TTTTTATTGGGGATAGAGG-CTAATTTTAA-------AATTGGATAAGATG---------------------GAATCTGAT---------CAATTAC-------------------------------------TCTT--------------------CGTCTTTTG---CTTCTCTATCCG---------------AATTTTCG---------TTCTTTATCAT----AAAAGTTCTC-CCCCGCC---------AATGAATGATAAGT-GTC-----TAGGTG-AAGTAT----------------AGTATAAGATAAGTCAGA------------------------------------------ATACCTATACTCTTACTATTTA---CTATAA-GATTCTA----AGATAAAGACTCTTAAGGATAAGGC-----TTTTCAC----------ATGAATACTTAGTAGAACGACTAACGACGAGATTTATTATCGTTTCTCGCGTGTCTCACGAAAGTGAGAGTAG-GTGCGAATTCTCCCAATTTGTGACCGACCATACGATCTGTGATATAAATGGTAAA-TGTTCCTTTCCATTATGAATAGC-GATTGTATGGCCAATCATTGTGGGTA-TAATGGTAGATGCCCGAGACCAAGTCACTATGATTTCTTTCTCCTCCCTCCTGTT-GAGTTTTTCAATTCTTCCCGATAAATGATTAGCTACAAAAGGATTTTTTTTT-AGTGAACGTGTCAC-GGCTGATTACTCCTTTTTTTACATTTTA-GAAATTGGCATTCTATGTCCAA----------TATCTCGATCTTAA-TCTG-AAG-TAT---------------------------AATG-ATGAATGG-AAAAAAGAGAAA-TCC----------TTTA-GCTAG-ATAGCTAGATAAGGG-AA-GGGGC--GATGTAG-CCAAGTGG-ATCA--GGC---

Cleisostoma_paniculatum_KFBG516 ACGACT-CTCGACAATGGATATCTCGGCTCTCGCATCGATGAAGAGCGCAGCGAAATGCGATACGTGGTGCGAATTGCAGAATCCCGCGAACCATCGAGTCTTTGAACGCAAGTTGCGCCCGAGGCCAAT-CGGTCGAGGGCACGTCCGCCTGGGCGTCA-AGCGTTGCGCCGCTCCACAAGAATTCTTTTTCTTATCATTTTTAT------------TCTCAAATGGTATCAGAAGGTTTTGGAGTCATTCTGGAAATTTCATTCTCGTCGCGATTAGTATCC---------TTCCTTGAAG---AAAAAAGAATACCAAAATCTCAGAATTTACGATCTATTCATTCAATATTTCCCTTTTTAGAGGATAAATTATCACATTTAAATTATGTGTCGGATCTACTAATACCCTATCCCATCCATCTGGAAATCTTGGTTCAAATCCTTCAATGCTGGATCAAAGATGTTCCTTCTTTGCATTTATTGCGATTGATTTTCCACGAATATCAT---------------AATTTGAATAGTCTC---------ATTACTT------CAAAAAAATC---------------CATTTACGTCTTTTCAAAAAA---AAAGAAAAAATTCTTTTGGTTCCTACATAATTTTTATGTATATGAATGCGAATATATATTCCTCTTTCTTCGTAAACAGTCTTCTTATTTACGATCAATATCTTCTGGAGTCTTTCTTGAGCGAACACATTTTTATGGAAAAATAGAA------TATCTTAGAGT---CGTGTCTTGTAATTCTTTTTATAGG-ATCCTATGGTTCCTCAAAGATATTTTCATACATTATGTTCGATATCAAGGAAAATCTATTTTGGCTTCAAAAGGAACTCTTATTCTGATGAATAAATGGAAATTTCATTTTGTGAATTTTTGGCAATCTTATTTTCACTTTTGGTTTCAACCTTATAGGATCCATATAAAGCAATTACCCAATTATTCCTTCTCTTTTCT-AGGATATTTTTCAAGTGTAC-TAAAAAACCCTTTGGTAGTAAGAAATCAAATGCTAGAGAATTCATTTCTAATAAATACTCTGACTAATAAATTAGATAGCATAGCTCCAGTTATTTTTCTTATTGGATCATTGTCGAAAGCTCAATTTTGTACTGTATTAGGTCATCCTATTAGTAAACCGATCTGGACTAATTTATCGGATTCTGATATTCTTGATCGATTTTGTCGGATATATAGAAATCTTTGTCGTTATCACAGCGGATCCTCAAATAAACAGGTTTTGTATCGTATAAAGTATATACTTCGACTTTCGTGTGCTAGAACTTTGGCTCGTAAACATAAAAGTAC-AGTACGCACTTTTATGCGAAGATTAGGTTCGGGATTCTTAGAAGAATTTTTTTTGGAAGAAGAACAATCTCTTTATTGAGCTATCCCGACCATTTCCCGTGCATCATCCTAGCAGAGTACTTC-TATCTATGTCAATGAAAAGAA-CTAAAAAAGAAAATCTTAACAA-------------------ATTAGCTCTAG--CCCCT-GAAATT----------CTTGG-------ATC-TTCAAAAA----------GAAGAC--TTTTTTTT-----------GTAAA-TGTAAGGAAAAAGATATAGACTAT-GAATGA------------TTCAATAAC-GGAGATTCCTTGAAC-------------------------ATATATCTTCAT-------------ATCGTA----TTAT------ACAAAA--------CAAAT-----GAGA--------TTGGATTGGAA------AAA-----GAT------------------ACGAGGATTTAG------ATTCGGATCCATT------TGTGAAAGAACAGAG--TGAATAAAATGAGAAAG-------ATATTTCATTT--------------TGTTTAAACT------------GAGTGACTGATGACTAGAGGAT-------------------------------GAGGATAAATA-AAGA------------------GCGAGGAA----------GTAAAAT-----GGGC-TTTTTATTGGGGATAGAGGGCCTTCGAT---------AAATGGATAATACT--------TTTTTATTCA-ATGAGTTTTTGAAGATAG--CAAT---------CCCCCAATATCT-TGT---------------TCTTAGAACAAGATATT--GGGGGATTCTTTTG---CTTCTCTA---------------------TTTTCG---------TTCTTTATCAT----AAAAGTTTTC-CCCCGCC---------AATGAATGATAAGT-GCC-----TAGGTG-AAGTAT----------------AGTATAAGATAAGTCAGA----------------------AAAGTCTAAGTCTTA-----ATACCTATACTCTTA----------CTATAA-GATAAA-----AGATAAAGACTCTTAAGGATAAGGCTTTTCTTTTCAT----------ATGAATACTTAGTAGAACGACTAACGACGAGATTTATTATCGTTTCTCGCGTGTCTCACGAAAGTTAGAGTAG-GTGCGAATTCTCCCAATTTGTGACCGACCATACGATCTGTGATATAAATGGTAAA-TGTTCCTTTCCATTATGAATAGC-GATTGTATGGCCAATCATTGTGGGTA-TAATGGTAGATGCCCGAGACCAAGTCACTATGATTTCTTTCTCCTCCCTCCTGTT-GAGTTTTTCAATTCTTCCCAATAAATGATTAGCTACAAAAGGATTTTTTTTT-AGTGAACGTGTCAC-GGCTGATTACTCCTTTTTTTACATTTTT-TAAATTGGCATTCTATGTCCAA----------TATCTCGATCTTAA-TCTG-AAG-TCT---------------------------AATG-ATGAATGG-AAAAAAGAGAAAATCC----------TTTA-GCTAG-ATAA--------GGG-AA-GGGGC-GGATGTAG-CCAAGTGG-ATCAA-GGCAAT

Cleisostoma_rostratum_SG1200 ACGACT-CTCGACAATGGATATCTCGGCTCTCGCATCGATGAAGAGCGCAGCGAAATGCGATACGTGGTGCGAATTGCAGAATCCCGCGAACCATCGAGTCTTTGAACGCAAGTTGCGCCCGAGGCCAAT-CGGTCGAGGGCACGTCCGCCTGGGCGTCA-AGCGTCGCGCCGCTCCACAAGAATTCTTTTTCTTATCATTTTTAT------------TCTCAAATGGTATCAGAAGGTTTTGGAGTCATTCTGGAAATTTCATTCTCGTCGCGATTAGTATCC---------TTCCTTGAAG---AAAAAAGAATACCAAAATCTCAGAATTTACGATCTATTCATTCAATATTTCCCTTTTTAGAGGATAAATTATCACATTTAAATTATGTGTCGGATCTACTAATACCCTATCCCATCCATCTGGAAATCTTGGTTCAAATCCTTCAATGCTGGATCAAAGATGTTCCTTCTTTGCATTTATTGCGATTGATTTTCCACGAATATCAT---------------AATTTGAATAGTCTC---------ATTACTT------CAAAAAAATC---------------CATTTACGTCTTTTCAAAAAA---AAAGAAAAGATTCTTTTGGTTCCTACATAATTTTTATGTATATGAATGTGAATATATATTCCTCTTTCTTCGTAAACAGTCTTCTTATTTACGATCAATATCTTCTGGAGTCTTTCTTGAGCGAACACATTTTTATGGAAAAATAGAA------TATCTTAGAGT---CGTGTCTTGTAATTCTTTTCAGAGG-ATTCCATGGTTATTCAAAGATATTTTCATACATTATGTTCGATATCAAGGAAAAGTAATTTTGGCTTCAAAAGGAACTCTTATTCTGATGAATAAATGGAAATTTCATTTTGTGAATTTTTGGCAATCTTATTTTCACTTTTGGTTTCAACCTTATAGGATCCATATAAAGCAATTACCCAATTATTCCTTCTCTTTTCT-GGGATATTTTTCAAGTGTAC-TAAAAAACCCTTTGGTAGTAAGAAATCAAATGCTAGAGAATTCATTTCTAATAAAGACTCTGACTAAGAAATTAGATACCATAGCTCCAGTTATTTTTCTTATTGGATCATTGTCGAAAGCTCAATTTTGTACTGTATTAGGTCATCCTATTAGTAAACCGATCTGGACCAATTTATCGGATTCTGATATTCTTGATCGATTTTGTCGGATATGTAGAAATCTTTGTCGTTATCACAGCGGATCCTCAAAGAAACAGGTTTTGTATCGTATAAAGTATATACTTCGACTTTCGTGTGCTAGAACTTTGTCTCGTAAACATAAAAGTAC-AGTACGCACTTTTATGCGAAGATTAGGTTCGGGATTCTTAGAAGAATTTTTTTTGGAAGAAGAACAATCTCTTTCTTGAGCTATCCCGACCATTTCCCGTACATCATCCTAGCAGAGTACTTC-TATCTATGTCAATGAAAAGAA-CTAAAAAAGAAAATCTTAACAA-------------------ATTGGCTCTAG--CCCCT-GAAATT----------CTTGG-------ATC-TTTAAAAA----------GAAGACTTTTTTTTTT-----------GTAAA-TGTAAGGAAAAAGATATAGACTAT-GAATGA------------TTCAATAAC-GGAGATTCCTTCAAC-------------------------ATATATCTTCAT-------------ATCGTA----TTAT------ATAAAA--------CAACT-----GAGA--------TTGGATTGGAA------AAA-----GAT------------------ACGAGGATTTAT------ATTCGGATCCATT------TGTGAAAGAACAGAG--TGAATAAAATGAGAAAG-------ATAGTTCATTT--------------TCTTTAAACT------------GAGTGACTGATGAACAGAGGAT-------------------------------GAGGATAAATA-AAGA------------------KCGAGGAA----------GTAAAAT-----GGGC-TTTTTATTGGGGATAGAGGGCCTTCGAT---------AAATGGATAAGACT--------TTTTTATTCA-ATGAATTTTTGAAGATAG--CAAT---------CCCCCAATATCT-TGT---------------TCTTAGAACAAGATATT--GGGGGATTCTTTTG---CTTCTCTA---------------------TTTTCG---------TTCTTTATCAT----AAAAGTTTTC-CCCCGCC---------AATGAATGATAAGT-GCC-----TAGGTG-AAGTAT----------------AGTATAAGATAAGTCAGA----------------------AAAGTCTAAGTCTTA-----ATACCTATACTCTTA----------CTATAA-GATAAA-----AGATAAAGACTCTTAAG--------AATTCTTTTCAT----------ATGAATACTTAGTAGAACGACTAACGACGAGATTTATTATCGTTTCTCGCGTGTCTCACGAAAGTTAGAGTAG-GTGCGAATTCTCCCAATTTGTGACCGACCATACGATCTGTGATATAAATGGTAAA-TGTTCCTTTCCATTATGAATAGC-GATTGTATGGCCAATCATTGTGGGTA-TAATGGTAGATGCCCGAGACCAAGTCACTATGATTTCTTTCTCCTCCCTCCTGTT-GAGTTTTTCAATTCTTCCCAATAAATGATTAGCTACAAAAGGATTTTTTTTT-AGTGAACGTGTCAC-GGCTGATTACTCCTTTTTTTACATTTTT-TAAATTGGCATTCTATGTCCAA----------TATCTTGATCTTAA-TCTG-AAG-TCT---------------------------AATG-ATGAATGG-AAAAAAGAGAAAATCC----------TTTA-GCTAG-ATAA--------GGG-AA-GGGGC-GGATGTAG-CCAAGTGG-ATCAA-GGCAGT

Cleisostoma_simondii_SG1314 ACGACT-CTCGACAATGGATATCTCGGCTCTCGCATCGATGAAGAGCGCAGCGAAATGCGATACGTGGTGCGAATTGCAGAATCCCGCGAACCATCGAGTCTTTGAACGCAAGTTGCGCCCGAGGCCAAT-CGGTCGAGGGCACGTCCGCCTGGGCGTCA-AGCGTTGCGCCGCTCC-CAAGAATTCTTTTTCTTATCATTTTTAT------------TCTCAAATGGTATCAGAAGGTTTTGGAGTCATTCTGGAAATTTCATTCTCGTCGCAATTAGTATCC---------TTCCTTGAAG---AAAAAAGAATACAAAAATCTCAGAATTTACGATCTATTCATTCAATATTTCCCTTTTTAGAGGATAAATTATCACATTTAAATTATGTGTCGGATCTACTAATACCCTATCCCATCCATCTGGAAATCTTGGTTCAAATCCTTCAATGCTGGATCAAAGATGTTCCTTCTTTGCATTTATTGCGATTGATTTTTCACGAATATCAT---------------AATTTGAATAGTCTC---------ATTACTT------CAAAAAAATC---------------CATTTACGTCTTTTCAAAAAA---AAAGAAAAGATTCTTTTGGTTCCTACATAATTTTTATGTATATGAATACGAATATATATTCCTCTTTCTTCGTAAACAGTCTTCTTATTTACGATCAATATCTTCTGGAGTCTTTATTGAGCGAACGCATTTTTATGGAAAAATAGGA------TATCTTAGAGT---CGTGTCTTGTAATTCTTTTCAGAGG-ATCCTATGGTTCCTCAAAGATATTTTCATACATTATGTTCGATATCAAGGAAAAGCGATTTTGGCTTCAAAAGGAACTCTTATTCTGATGAATAAATGGAAATTTCATTTTGTGAATTTTTGGCAATCTTATTTTCACTTTTGGTTTCAACCTTATAGGATCCATATAAAGCAATTACCCAATTATTCCTTCTCTTTTCT-GGGATATTTTTCAAGTGTAC-TAAAAAACCCTTTGGTAGTAAGAAATCAAATGTTAGAGAATTCATTTCTAATAAAGACTCTGACTAATAAATTAGATACCGTAGCTCCAGTTATTTTTCTTATTGGATCATTGTCGAAAGCTCAATTTTGTACTGTATTAGGTCATCCTATTAGTAAACCGATCTGGACCAATTTATCGGATTCTGATATTCTTGATCGATTTTGTCGGATATATAGAAATCTTTGTCGTTATTACAGCGGATCCTCAAAGAAACAGGTTTTGTATCGTATAAAGTATATACTTCGACTTTCGTGCGCTAGAACTTTGGCTCGTAAACATAAAAGTAC-AGTACGCACTTTTATGCGAAGATTAGGTTCGGGATTCTTAGAAGAATTTTTTTTGGAAG------------------GAGCTATCCCGACCATTTACCGTGCATCATCCTAGCAGAGTACTTC-TATCTATGTCAATCAAAAGAA-CTAAAAAAGAAAATCTTAACAA-------------------ATTGGCTCTAG--CCCCT-GAAATT----------CTTGG-------ATC-TTCAAAAA----------GAAGAC--TTTTTTTT-----------GTAAA-TGTAAGGAAAAAGATATAGACTAT-GAATGA------------TTCAATAAC-GGAGATTCCTTGAAC-------------------------ATATATCTTCAT-------------ATCGTA----TTAT------ACAAAA--------CAAAT-----GAGA--------TTGGATTGGAA------AAA-----GAT------------------ACGAGGATTTAT------ATTTGGATCCATT------TGTGAAAGAACAGAG--TGAATAAAATGAGAAAG-------ATATTTCATTT--------------TGTTTAAATT------------GAGTGACTGATGAACAGAGGAT-------------------------------GAGGATAAATA-AAGA------------------GCGAGGAA----------GTAAAAT-----GGGC-TTTTTATTGGGGATAGAG--CCTTCAAT---------AAATGGATAATACT--------TTTTTATCCA-ATGAGTTTTTGAAGATAG--CAAT---------CCCCCAATATCT-TGT---------------TCTTAGAACAAGATATT--GGGGGATTCTTTTG---CTTCTCTA---------------------TTTTTG---------TTCTTTATCAT----AAAAGTTTTC-CCCCGCC---------AATGAATGATAAGT-GCC-----TAGGTG-AAGTAT----------------AGTATAAGATAAGTCAGA----------------------AAAGTCTAAGTCTTA-----ATACCTATACTCTTA----------CTATAA-GATAAA-----AGATAAAGACTCTTAAGGATAAGGCTTTTCTTTTCAT----------ATGAATACTTAGTAGAACGACTAACGACGAGATTTATTATCGTTTCTCGCGTGTCTCACGAAAGTTAGAGTAG-GTGCGAATTCTCCCAATTTGTGACCGACCATACGATCTGTGATATAAATGGTAAA-TGTTCCTTTCCATTATGAATAGC-GATTGTATGGCCAATCATTGTGGGTA-TAATGGTAGATGCCCGAGACCAAGTCACTATGATTTCTTTCTCCTCCCTCCTGTT-GAGTTTTTCAATTCTTCCCAATAAATGATTAGCTACAAAAGGATTTTTTTTT-AGTGAACGTGTCAC-GGCTGATTACTCCTTTTTTTACATTTTT-TAAATTGGCATTCTATGTCCAA----------TATCTCGATCTTAA-TCTG-AAG-TCT---------------------------AATG-ATGAATGG-AAAAAAGAGAAAATCC----------TTTA-GCTAG-ATAA--------GGG-AA-GGGGC-GGATGTAG-CCAAGTGG-ATCAA-GGCAGT

Cleisostoma_simondii_var_guangdongense_KFBG2212 ACGACT-CTCGACAATGGATATCTCGGCTCTCGCATCGATGAAGAGCGCAGCGAAATGCGATACGTGGTGCGAATTGCAGAATCCCGCGAACCATCGAGTCTTTGAACGCAAGTTGCGCCCGAGGCCAAT-CGGTCGAGGGCACGTCCGCCTGGGCGTCA-AGCGTTGCGCCGCTCC----------------------------------------------------------------------------------------------------------------------------------------------------------------------------------------------------------------------------------------------------------------------------------------------------------------------------------------------------------------------------------------------------------------------------------------------------------------------------------------------------------------------------------------------------------------------------------------------------------------------------------------------------------------------------------------------------------------------------------------------------------------------------------------------------------------------------------------------------------------------------------------------------------------------------------------------------------------------------------------------------------------------------------------------------------------------------------------------------------------------------------------------------------------------------------------------------------------------------------------------------------------------------------------------------------GAGCTATCCCGACCATTTACCGTGCATCATCCTAGCAGAGTACTTC-TATCTATGTCAATCAAAAGAA-CTAAAAAAGAAAATCTTAACAA-------------------ATTGGCTCTAG--CCCCT-GAAATT----------CTTGG-------ATC-TTCAAAAA----------GAAGAC--TTTTTTTT-----------GTAAA-TGTAAGGAAAAAGATATAGACTAT-GAATGA------------TTCAATAAC-GGAGATTCCTTGAAC-------------------------ATATATCTTCAT-------------ATCGTA----TTAT------ACAAAA--------CAAAT-----GAGA--------TTGGATTGGAA------AAA-----GAT------------------ACGAGGATTTAT------ATTTGGATCCATT------TGTGAAAGAACAGAG--TGAATAAAATGAGAAAG-------ATATTTCATTT--------------TGTTTAAATT------------GAGTGACTGATGAACAGAGGAT-------------------------------GAGGATAAATA-AAGA------------------GCGAGGAA----------GTAAAAT-----GGGC-TTTTTATTGGGGATAGAGGGCCTTCAAT---------AAATGGATAATACT--------TTTTTATCCA-ATGAGTTTTTGAAGATAG--CAAT---------CCCCCAATATCT-TGT---------------TCTTAGAACAAGATATT--GGGGGATTCTTTTG---CTTCTCTA---------------------TTTTTG---------TTCTTTATCAT----AAAAGTTTTC-CCCCGCC---------AATGAATGATAAGT-GCC-----TAGGTG-AAGTAT----------------AGTATAAGATAAGTCAGA----------------------AAAGTCTAAGTCTTA-----ATACCTATACTCTTA----------CTATAA-GATAAA-----AGATAAAGACTCTTAAGGATAAGGCTTTTCTTTTCAT----------ATGAATACTTAGTAGAACGACTAACGACGAGATTTATTATCGTTTCTCGCGTGTCTCACGAAAGTTAGAGTAG-GTGCGAATTCTCCCAATTTGTGACCGACCATACGATCTGTGATATAAATGGTAAA-TGTTCCTTTCCATTATGAATAGC-GATTGTATGGCCAATCATTGTGGGTA-TAATGGTAGATGCCCGAGACCAAGTCACTATGATTTCTTTCTCCTCCCTCCTGTT-GAGTTTTTCAATTCTTCCCAATAAATGATTAGCTACAAAAGGATTTTTTTTT-AGTGAACGTGTCAC-GGCTGATTACTCCTTTTTTTACATTTTT-TAAATTGGCATTCTATGTCCWA----------TATCTCGATCTTAA-TCTG-AAG-TCT---------------------------AATG-ATGAATGG-AAAAAAGAGAAAATCC----------TTTA-GCTAG-ATAA--------AGG-AA-GGGGC-GGATGTAG-CCAAGTGG-ATCAA-GGCAGT

Cleisostoma_williamsonii ACGACT-CTCGACAATGGATATCTCGGCTCTCGCATCGATGAAGAGCGCAGCGAAATGCGATACGTGGTGCGAATTGCAGAATCCCGCGAACCATCGAGTCTTTGAACGCAAGTTGCGCCCGAGGCCAAT-CGGTCGAGGGCACGTCCGCCTGGGCGTCA-AGCGTTGCGCCGCTCCACAAGAATTCTTTTTCTTATCATTTTTAT------------TCTCAAATGGTATCAGAAGGTTTTGGAGTCATTCTGGAAATTTCATTCTTGTCGCAATTAGTATCC---------TTCCTTGAAA---AAAAAAGAATACAAAAATCTCAGAATTTACGATCTATTCATTCAATATTTCCCTTTTTAGAGGATAAATTATCACATTTAAATTATGTGTCGGATCTACTAATACCCTATCCCATCCATCTGGAAATCTTGGTTCAAATCCTTCAATGCTGGATCAAAGATGTTCCTTCTTTGCATTTATTGCGATTGATTTTCCACGAATATCAT---------------AATTTGAATAGTCTC---------ATTACTT------CAAAAAAATC---------------CATTTACGTCTTTTCAAAAAA---AAAGAAAAGATTCTTTTGGTTCCTACATAATTTTTATGTATATGAATGCGAATATATATTCCTCTTTCTTCGTAAACAGTCTTCTTATTTACGATCAATATCTTCTGGAGTCTTTCTTGAGCGAACACATTTTTATGGAAAAATAGAA------TATCTTAGAGT---CGTGTCTTGTAATTCTTTTCAGAGG-ATCCTATGGTTCCTCAAAGATATTTTCATACATTATGTTCGATATCAAGGAAAAGCGATTTTGGCTTCAAAAGGAACTCTTATTCTGATGAATAAATGGAAATTTCATTTTGTGAATTTTTGGCAATCTTATTTTCACTTTTGGTTTCAACCTTATAGGATCCATATAAAGCAATTACCCAATTATTCCTTCTCTTTTCT-GGGATATTTTTCAAGTGTAC-TAAAAAACCCTTTGGTAGTAAGAAATCAAATGCTAGAGAATTCATTTCTAATAAATACTCTGACTAAGAAATTAGATACCATAGCTCCAGTTATTTTTCTTATTGGATCATTGTCGAAAGCTCAATTTTGTACTGTATTAGGTCATCCTATTAGTAAACCGATCTGGACCAATTTATCGGATTCTGATATTCTTGATCGATTTTGTCGGATATATAGAAATCTTTGTCGTTATCACAGCGGATCCTCAAAGAAACAGGTTTTGTATCGTATAAAGTATATACTTCGACTTTCGTGTGCTAGAACTTTGGCTCGTAAACATAAAAGTAC-AGTACGCACTTTTATGCGAAGATTAGGTTCGAGATTCTTAGAAGAATTCTTTTTGGAAGAAGAACAATCTCTTTCTT-----------------------------------------------TATCTATGTCAATGAAAAGAA-CTAAAAAAGAAAATCTTAACAA-------------------ATTGGCTCTAG--CCCCT-GAAATT----------CTTGG-------ATC-TTCAAAAA----------GAAGAC--TTTTTTTT-----------GTAAA-TGTAAGGAAAAAGATATAGACTAT-GAATTA------------TTCAATAAC-GGAGATTCCTTGAAC-------------------------ATATATCTTCAT-------------ATCGTA----TTAT------ACAAAA--------CAAAT-----GAAA--------TTGGATTGGAA------AAA-----GAT------------------ACGAGGATTTAT------ATTCGGATCCATT------TGTGAAAGAACAGAG--TGAATAAAATGAGAAAG-------ATATTTCATTT--------------TGTTTAAATT------------GAATGACTGATGAACAGAGGAT-------------------------------GAGGATAAATA-AAGA------------------GCAAGGAA----------GTAAAAT-----GGGC-TTTTTATTGGGGATAGAGGG------------------------TAATACT--------TTTTTATTCA-ATGAGTTTTTGAAGATAG--CAAT---------CCCCCAATATCT-TGT------------------------------------------CTTTTG---CTTCTCTA---------------------TTTTCG---------TTCTTTATCAT----AAAAGTTTTC-CCCCGCC---------AATGAATGATAAGT-GCC-----TAGGTG-AAGTAT----------------AGTATAAGATAAGTCAGA----------------------AAAGTCTAAGTCTTA-----ATACCTATACTCTTA----------CTATAA-GATAAA-----AGATAAAGACTCTTAAGGATAAGGCTTTTCTTTTCATATTTCAT---ATGAATACTTAGTAGAACGACTAACGACGAGATTTATTATCGTTTCTCGCGTGTCTCACGAAAGTTAGAGTAG-GTGCGAATTCTCCCAATTTGTGACCGACCATACGATCTGTGATATAAATGGTAAA-TGTTCCTTTCCATTATGAATAGC-GATTGTATGGCCAATCATTGTGGGTA-TAATGGTAGATGCCCGAGACCAAGTCACTATGATTTCTTTCTCCTCCCTCCTGTT-GAGTTTTTCAATTCTTCCCAATAAATGATTAGCTACAAAAGGATTTTTTTTT-AGTGAACGTGTCAC-GGCTGATTACTCCTTTTTTTACATTTTT-TAAATTGGCATTCTATGTCCAA----------TATCTCGATCTTAA-TCTG-AAG-TCT---------------------------AATG-ATGAATGG-AAAAAAGAGAAAA-------------------------------------------------------------------------------

Coelogyne_cantonensis_SG1239 ACGACT-CTCGGCAATGGATATCTCGGCTCTCGCATCGATGAAGAGCGCAGCGAAATGCGATACGTGGTGCGAATTGCAGAATCCCGCGAACCATCGAGTCTTTGAACGCAAGTTGCGCCCGAGGCCAAC-CGGCCAAGGGCACGTCTGCCTGGGCGTCA-AGCGTTGCGTCGCTCCACAAGAATTCTTTTTATTCTCATTTTTCT------------TCTCAAATGGTATCAGAAGGTTTTGGAGTCATTCTGGAAATTCCATTCTCGTCGCGATTAGTATCT---------TCCCTTGAAG---AAAAAAGAATACCAAAATCTCAGAATTTACGATCTATTCATTCAATATTTCCCTTTTTAGAGGATAAATTGTCACATTTAAATTATGTGTCAGATCTACTAATACCCCATCCCATCCATCTGGAAATCTTGGTTCAAATCCTTCAATGCTGGATCAAAGATGTTTCTTCTTTGCATTTCTTGCGATTGTTTTTCCACGAATATCAT---------------AATTTGAATAGTCTC---------ATTACTT------CAAAGAAATC---------------CATTTACGTCTTTTCAAAAAG---AAAGAAAAGATTCTTTTGGTTCCTACATAATTCTTATGTATATGAATGCGAATATCTATTCCTGTTTCTTCGTAAACAGTCTTCTTATTTACGATCAATATCTTCTGGAGTCTTTCTTGAGCGAACACATTTCTATGGAAAAATAGAA------TATATTATAGT---CGTGTGTTGTAATTCTTTTCAGAGG-ATCCTATGGTTCCTCAAAGATACTTTCATACATTATGTTCGATATCAAGGAAAAGCAATTCTGGCTTCAAAAGGAACTCTTATTCTGATGAAGAAATGGAAATTTCATCTTGTGAATTTTTGGCAATCTTATTTTCACTTTTGGTTTCAACCTTATAGGATCTATATAAAGCAATTACCCAACTATTCCTTCTCTTTTCT-GGGATATTTTTCAAGTGTAC-TAAAAAATCCTTTGGTAGTAAGAAATCAAATGCTAGAGAATTCATTTCTAATAAATACTCTGACTAAGAAATTAGATACCATAGCCCCAGTTATTTCTCTTATTGGATCATTGTCGAAAGCTCAATTTTGTACTGTATTGGGTCATCCTATTAGTAAACCGATCTGGACCGATTTATCGGATTCTGATATTATTGATCGATTTTGTCGGATATGTAGAAATCTTTGTCGTTATCACAGCGGATCCTCAAAGAAACAGGTTTTGTATCGTATAAAGTATATACTTCGACTTTCGTGTGCTAGAACTTTGGCTCGTAAACATAAAAGTAC-AGTACGCACTTTTATGCGAAGATTAGGTTCGGGATTCTTAGAAGAATTCTTTTTGGAAGAAGA--------------GAGCTATCCCGACCATTTCCCGTGCATCATCCTAGCAGAGTACTTA-TATCTATGTCAATGAAAAGAA-CTAAAAAAGAAAATATTAACAA-------------------ATTGGACCTAG--CCCCT-TAATTT----------CTTAG-------ATC-TTCAAAAA----------GAAGAC---TTTCTTT-----------GTAAA-TGTAAGGAAAAAGATATGGACTAT-GAATGA------------TTCAATAAC-GGAGATTCCTTGAAC-------------------------ATATATGTTCAT-------------ATCGTA----CTAT------ACAAAA--------CAAAT-----GAGA--------TTGGATTGGAA------GAA-----GAT------------------ACGAGGATTTCT------ATTCGAATCCATT------TGTGAAAGAACAGAG--TGAATGAAATGAGAAAG-------ATATTGAATTT--------------TGTTTGAACT------------GAGCCACTGATGGAAAGAGGAT-------------------------------GAGGATAAATA-AAGAGCGAG-------------GCGAGGAA----------GTAAAAT-----GGGC-TTTTTATTGGGGATAGAGGACCATCTAT---------AAATGGATAAGACT--------TTTGTATTCATATGAATTCTTGAAGATAG--CAAT---------CCCCCAATATCT-TGT---------------TCTAAGAACAAGATATT--GGGGGATT-----------TCTT--------------------------------------------------------------------------------GTTGGATGTTGGAT---------TTAGTC-----------------------------------------------------------------------------------------------------------------------------------------------------------------------------ATGAATACTTAGTAGAACGACTAACGACGAGATTTATTATCGTTTCTCGCGTGTCTCACGAAAGTGAGAGTAG-GTGCGAATTCTCCCAATTTGTGACCGACCATACGATCTGTGATATAAATGGTAAA-TGTTCCTTTCCATTATGAATAGC-GATTGTATGGCCAATCATTGTGGGTA-TAATGGTAGATGCCCGAGACCAAGTCACTATGATTTCTTTCTCCTCCCTCCTGTT-GAGTTTTTCAATTCTTCCCGATAAATGATTAGCTACAAAAGGATTTTTTTTT-AGTGAACGTGTCAC-GGCTGATTACTCCTTTTTTTACATTTTT-GAAATTGGCATTCTATGTCCAA----------TATCTCGATCTTAA-TCTG-AAG-TAT---------------------------AATG-ATGAATGG-AAAAAAGAGAAAATCC----------TTTA-GCTAG-ATAA--------GGG-AA-GGGGC-GGATGTAG-CCAAGTGG-ATCAA-GGCAGT

Coelogyne_chinensis_SG1232 ACGACT-CTCGGCAATGGATATCTCGGCTCTCGCATCGATGAAGAGCGCAGCGAAATGCGATACGTGGTGCGAATTGCAGAATCCCGCGAACCATCGAGTCTTTGAACGCAAGTTGCGCCCGAGGCCAAC-CGGCCAAGGGCACGTCTGCCTGGGCGTCA-AGCGTTGCGTCGCTCCACAAGAATTCTTTTTCTTCTCATTTTTCT------------TCTCAAATGGTATCAGAAGGTTTTGGAGTCATTCTGGAAATTCCATTCTCGTCGCGATTAGTATCT---------TCCCTTGAAG---AAAAAAGAATACCAAAATCTCAGAATTTACGATCTATTCATTCAATATTTCCCTTTTTAGAGGATAAATTGTCACATTTAAATTATGTGTCAGATCTACTAATACCCCATCCCATCCATCTGGAAATCTTGGTTCAAATCCTTCAATGCTGGATCAAAGATGTTTCTTCTTTGCATTTCTTGCGATTGTTTTTCCACGAATATCAT---------------AATTTGAATAGTCTC---------ATTACTT------CAAAGAAATC---------------CATTTACGTCTTTTCAAAAAG---AAAGAAAAGATTCTTTTGGTTCCTACATAATTCTTATGTATATGAATGCGAATATCTATTCCTGTTTCTTCGTAAACAGTCTTCTTATTTACGATCAATATCTTCTGGAGTCTTTCTTGAGCGAACACATTTCTATGGAAAAATAGAA------TATCTTATAGT---CGTGTGTTGTAATTCTTTTCAGAGG-ATCCTATGGTTCCTCAAAGATACTTTCATACATTATGTTCGATATCAAGGAAAAGCAATTCTGGCTTCAAAAGGAACTCTTATTCTGATGAAGAAATGGAAATTTCATCTTGTGAATTTTTGGCAATCTTATTTTCACTTTTGGTTTCAACCTTATAGGATCTATATAAAGCAATTACCCGACTATTCCTTCTCTTTTCT-GGGATATTTTTCAAGTGTAC-TAAAAAATCCTTTGGTAGTAAGAAATCAAATGCTAGAGAATTCATTTCTAATAAATACTCTGACTAAGAAATTAGATACCATAGCCCCAGTTATTTCTCTTATTGGATCATTGTCGAAAGCTCAATTTTGTACTGTATTGGGTCATCCTATTAGTAAACCGATCTGGACCGATTTATCGGATTCTGATATTATTGATCGATTTTGTCGGATATGTAGAAATCTTTGTCGTTATCACAGCGGATCCTCAAAGAAACAGGTTTTGTATCGTATAAAGTATATACTTCGACTTTCGTGTGCTAGAACTTTGGCTCGTAAACATAAAAGTAC-AGTACGCACTTTTATGCGAAGATTAGGTTCGGGATTCTTAGAAGAATTTTTTTTGGAAGAAGAACAATCTC------GAGCTATCCCGACCATTTCCCGTGCATCATCCTAGCAGAGTACTTA-TATCTATGTCAATGAAAAGAA-CTAAAAAAGAAAATATTAACAA-------------------ATTGGACCTAG--CCCCT-TAATTT----------CTTAG-------ATC-TTCAAAAA----------GAAGAC---TTTCTTT-----------GTAAA-TGTAAGGAAAAAGATATGGACTAT-GAATGA------------TTCAATAAC-GGAGATTCCTTGAAC-------------------------ATATATGTTCAT-------------ATCATA----CTAT------ACAAAA--------CAAAT-----GAGA--------TTGGATTGGAA------GAA-----GAT------------------ACGAGGATTTCT------ATTCGGATCCATT------TGTGAAAGAACAGAG--TGAATGAAATGAGAAAG-------ATATTGAATTT--------------TGTTTGAACT------------GAGCCACTGATGTAAAGAGGAT-------------------------------GAGGATAAATA-AAGAGCGAG-------------GCGAGGAA----------GTAAAAT-----GGGC-TTTTTATTGGGGAT------CCATCTAT---------AAATGGATAAGACT--------TTTGTATTCATATGAATTATTGAAGATAG--CAAT---------CCCCCAATATCT-TGT---------------TCTTAGAACAAGATATT--GGGGGATTGATTA------TCTT--------------------------------------------------------------------------------GTTGGATGTTGGAT---------TAAGTC-TAT-------------------AGTCTAAGATAAGTCAGA----------------------AAAGTCTAAGTCTTAGTATAATACCTATACTCTTA----------CTATAA-GAT------------AAAGACTCTTAAGGATAAGGC-----TTTTCAC----------ATGAATACTTAGTAGAACGACTAACGACGAGATTTATTATCGTTTCTCGCGTGTCTCACGAAAGTGAGAGTAG-GTGCGAATTCTCCCAATTTGTGACCGACCATACGATCTGTGATATAAATGGTAAA-TGTTCCTTTCCATTATGAATAGC-GATTGTATGGCCAATCATTGTGGGTA-TAATGGTAGATGCCCGAGACCAAGTCACTATGATTTCTTTCTCCTCCCTCCTGTT-GAGTTTTTCAATTCTTCCCGATAAATGATTAGCTACAAAAGGATTTTTTTTT-AGTGAACGTGTCAC-GGCTGATTACTCCTTTTTTTACATTTTT-GAAATTGGCATTCTATGTCCAA----------TATCTCGATCTTAA-TCTG-AAG-TAT---------------------------AATG-ATGAATGG-AAAAAAGAGAAAATCC----------TTTA-GCTAG-ATAA--------GGG-AA-GGGGC-GGATGTAG-CCAAGTGG-ATCAA-GGCAGT

Coelogyne_fimbriata_SG1059 ACGACT-CTCGGCAATGGATATCTCGGCTCTCGCATCGATGAAGAGCGCAGCGAAATGCGATACGTGGTGCGAATTGCAGAATCCCGCGAACCATCGAGTCTTTGAACGCAAGTTGCGCCCGAGGCCAAC-CGGCCAAGGGCACGTCTGCCTGGGCGTCA-AGCGTTGCGTCGCTCCACAAGAATTCTTTTTATTCTCATTTTTCT------------TCTCAAATGGTATCAGAAGGTTTTGGAGTCATTCTGGAAATTCCATTCTCGTCGCGATTAGTATCT---------TCCCTTGAAG---AAAAAAGAATACCAAAATCTCAGAATTTACGATCTATTCATTCAATATTTCCCTTTTTAGAGGATAAATTGTCACATTTAAATTATGTGTCAGATCTACTAATACCCCATCCCATCCATCTGGAAATCTTGGTTCAAATCCTTCAATGCTGGATCAAAGATGTTTCTTCTTTGCATTTCTTGCGATTGTTTTTCCACGAATATCAT---------------AATTTGAATAGTCTC---------CTTACTT------CAAAGAAATC---------------CATTTACGTCTTTTCAAAAAG---AAAGAAAAGATTCTTTTGGTTCCTACATAATTCTTATGTATATGAATGCGAATATCTATTCCTGTTTCTTCGTAAACAGTCTTCTTATTTACGATCAATATCTTCTGGAGTCTTTCTTGAGCGAACACATTTCTATGGAAAAATAGAA------TATCTTATAGT---CGTGTGTTGTAATTCTTTTCAGAGG-ATCCTATGGTTCCTCAAAGATACTTTCATACATTATGTTCGATATCAAGGAAAAGCAATTCTGGCTTCAAAAGGAACTCTTATTCTGATGAAGAAATGGAAATTTCATCTTGTGAATTTTTGGCAATCTTATTTTCATTTTTGGTTTCAACCTTATAGGATCTATATAAAGCAATTACCCAACTATTCCTTCTCTTTTCT-GGGATATTTTTCAAGTGTAC-TAAAAAATCATTTGGTAGTAAGAAATCAAATGCTAGAGAATTCATTTCTAATAAATACTCTGACTAAGAAATTAGATACCATAGTCCCAGTTATTTCTCTTATTGGATCATTGTCGAAAGCTCAATTTTGTACTGTATTGGGTCATCCTATTAGTAAACCGATCTGGACCGATTTATCGGATTCTGATATTATTGATCGATTTTGTCGGATATGTAGAAATCTTTGTCGTTATCACAGCGGATCCTCAAAGAAACAGGTTTTGTATCGTATAAAGTATATACTTCGACTTTCGTGTTGTAGAACTTAGGCTCGTGAACATAAAAGTAC-AATCCGCACC-------------------------------------------------------------------GAGCTATCCCGACCATTTCCCGTACATCATCCTAGCAGAGTACTTA-TATCTATGT-----AAAAGAA-CTAAAAAAGAAAATATTAACAA-------------------ATTGGACCTAG--CCCCT-TAATTT----------CTTAG-------ATC-TTCAAAAA----------GAAGAC---TTTCTTT-----------GTAAA-TGTAAGGAAAAAGATATGGACTAT-GAATGA------------TTAAATAAC-GGAGATTCCTTGAAC-------------------------ATATATGTTCAT-------------ATCGTA----CTAT------ACAAAA--------CAAAT-----GAGA--------TTGGATTGGAA------GAA-----GAT------------------ACGAGGATTTCT------ATTCGGATCCATT------TGTGAAAGAACAGAG--TGAATGAAATGAGAAAG-------ATATTGAATTT--------------TGTTTGAACT------------GAGCCACTGATGGAAAGAGGAT-------------------------------GAGGATAAATA-AAGAGCGAG-------------GCGAGGAA----------GTAAAAT-----GGGC-TTTTTATTGGGGATAGAGGGCCATCTAT---------AAATGGATAAGACT--------TTTGTATACATATGAATTCTTGAAGATAG--CAAT---------CCCCCAATATCT-TGT---------------TCTTATAACAAGATATT--GGGGGATT------T--CTT-----------------------------------------------------------------------------------GTTGGATGTTG-----------------------------------------GATTTAATATA-------------------------------------------------ATACCTACACTCTTA----------CTATAA-GAG------------AAAGACTCTTAAGGATAAGTC-----TTTTCAC----------ATGAAGACTTAGTAGAACGACTAACGACGAGATTTATTATCGTTTCTCGCGTGTCTCACGAAAGTGAGAGTAG-GTGCGAATTCTCCCAATTTGTGACCGACCATACGATCTGTGATATAAATGGTAAA-TGTTCCTTTCCATTATGAATAGC-GATTGTATGGCCAATCATTGTGGGTA-TAATGGTAGATGCCCGAGACCAAGTCACTATGATTTCTTTCTCCTCCCTCCTGTT-GAGTTTTTCAATTCTTCCCGATAAATGATTAGCTACAAAAGGATTTTTTTTT-AGTGAACGTGTCAC-GGCTGATTACTCCTTTTTTTACATTTTT-GAAATTGGCATTCTATGTCCAA----------TATCTCGATCTTAA-TCTG-AAG-TAT---------------------------AATG-ATGAATGG-AAAAAAGAGAAAATCC----------TTTA-GCTAG-ATAA--------GGG-AA-GGGGC-GGATGTAG-CCAAGTGG-ATCAA-GGCAGT

Coelogyne_fimbriata_var_leungiana_SG1058 ACGACT-CTCGGCAATGGATATCTCGGCTCTCGCATCGATGAAGAGCGCAGCGAAATGCGATACGTGGTGCGAATTGCAGAATCCCGCGAACCATCGAGTCTTTGAACGCAAGTTGCGCCCGAGGCCAAC-CGGCCAAGGGCACGTCTGCCTGGGCGTCA-AGCGTTGCGTCGCTCCACAAGAATTCTTTTTATTCTCATTTTTCT------------TCTCAAATGGTATCAGAAGGTTTTGGAGTCATTCTGGAAATTCCATTCTCGTCGCGATTAGTATCT---------TCCCTTGAAG---AAAAAAGAATACCAAAATCTCAGAATTTACGATCTATTCATTCAATATTTCCCTTTTTAGAGGATAAATTGTCACATTTAAATTATGTGTCAGATCTACTAATACCCCATCCCATCCATCTGGAAATCTTGGTTCAAATCCTTCAATGCTGGATCAAAGATGTTTCTTCTTTGCATTTCTTGCGATTGTTTTTCCACGAATATCAT---------------AATTTGAATAGTCTC---------CTTACTT------CAAAGAAATC---------------CATTTACGTCTTTTCAAAAAG---AAAGAAAAGATTCTTTTGGTTCCTACATAATTCTTATGTATATGAATGCGAATATCTATTCCTGTTTCTTCGTAAACAGTCTTCTTATTTACGATCAATATCTTCTGGAGTCTTTCTTGAGCGAACACATTTCTATGGAAAAATAGAA------TATCTTATAGT---CGTGTGTTGTAATTCTTTTCAGAGG-ATCCTATGGTTCCTCAAAGATACTTTCATACATTATGTTCGATATCAAGGAAAAGCAATTCTGGCTTCAAAAGGAACTCTTATTCTGATGAAGAAATGGAAATTTCATCTTGTGAATTTTTGGCAATCTTATTTTCATTTTTGGTTTCAACCTTATAGGATCTATATAAAGCAATTACCCAACTATTCCTTCTCTTTTCT-GGGATATTTTTCAAGTGTAC-TAAAAAATCATTTGGTAGTAAGAAATCAAATGCTAGAGAATTCATTTCTAATAAATACTCTGACTAAGAAATTAGATACCATAGTCCCAGTTATTTCTCTTATTGGATCATTGTCGAAAGCTCAATTTTGTACTGTATTGGGTCATCCTATTAGTAAACCGATCTGGACCGATTTATCGGATTCTGATATTATTGATCGATTTTGTCGGATATGTAGAAATCTTTGTCGTTATCACAGCGGATCCTCAAAGAAACAGGTTTTGTATCGTATAAAGTATATACTTCGACTTTCGTGTGCTAGAACTTTGGCTCGTAAACATAAAAGTAC-AGTACGCACTTTTATGCGAAGATTAGGTTCGGGATTCTTAGAAGAATTTTCTTTGGAAGAAGAACAATCTCTTTCTTGAGCTATCCCGACCATTTCCCGTACATCATCCTAGCAGAGTACTTA-TATCTATGT-----AAAAGAA-CTAAAAAAGAAAATATTAACAA-------------------ATTGGACCTAG--CCCCT-TAATTT----------CTTAG-------ATC-TTCAAAAA----------GAAGAC---TTTCTTT-----------GTAAA-TGTAAGGAAAAAGATATGGACTAT-GAATGA------------TTAAATAAC-GGAGATTCCTTGAAC-------------------------ATATATGTTCAT-------------ATCGTA----CTAT------ACAAAA--------CAAAT-----GAGA--------TTGGATTGGAA------GAA-----GAT------------------ACGAGGATTTCT------ATTCGGATCCATT------TGTGAAAGAACAGAG--TGAATGAAATGAGAAAG-------ATATTGAATTT--------------TGTTTGAACT------------GAGCCACTGATGGAAAGAGGAT-------------------------------GAGGATAAATA-AAGAGCGAG-------------GCGAGGAA----------GTAAAAT-----GGGC-TTTTTATTGGGGATAGAGGACCATCTAT---------AAATGGATAAGACT--------TTTGTATACATATGAATTCTTGAAGATAG--CAAT---------CCCCCAATATCT-TGT---------------TCTTAGAACAAGATATT--GGGGGATT------T--CTT-----------------------------------------------------------------------------------GTTGGATGTTG-----------------------------------------GATTTAATATA-------------------------------------------------ATACCTACACTCTTA----------CTATAA-GAT------------AAAGACTCTTAAGGATAAGGC-----TTTTCAC----------ATGAAGACTTAGTAGAACGACTAACGACGAGATTTATTATCGTTTCTCGCGTGTCTCACGAAAGTGAGAGTAG-GTGCGAATTCTCCCAATTTGTGACCGACCATACGATCTGTGATATAAATGGTAAA-TGTTCCTTTCCATTATGAATAGC-GATTGTATGGCCAATCATTGTGGGTA-TAATGGTAGATGCCCGAGACCAAGTCACTATGATTTCTTTCTCCTCCCTCCTGTT-GAGTTTTTCAATTCTTCCCGATAAATGATTAGCTACAAAAGGATTTTTTTTT-AGTGAACGTGTCAC-GGCTGATTACTCCTTTTTTTACATTTTG-GAAATTGGCATTCTATGTCCAA----------TATCTCGATCTTAA-TCTG-AAG-TAT---------------------------AATG-ATGAATGG-AAAAAAGAGAAAATCC----------TTTA-GCTAG-ATAA--------GGG-AA-GGGGC-GGATGTAG-CCAAGTGG-ATCAA-GGCAGT

Collabium_chinense ACGACT-CTCGGCAATGGATATCTCGGCTCTCGCATCGATGAAGAGCGCAGCGAAATGCGATACGTGGTGCGAATTGCAGAATCCCGCGAACCATCGAGTCTTTGAACGCAAGTTGCGCCCGAGGCCAAC-CGGCCAAGGGCACGTCTGCCTGGGCGTCA-AGCGTTGCGTCGCTCC----------------------TTTTTCT------------TCTCAAATGGTATCAGAAGGTTTTGGAGTCATTCTGGAAATTCCATTCTCGTCGCGATTAGTATCT---------TCCCTTGAAGAAAAAAAAAGAATACCAAAATCTCAGATTTTACGATCTATTCATTCAATATTTCCCTTTTTAGAGGATAAATTATCACATTTAAATTATGTGTCAGATCTACTAATACCCCATCCCATCCATCTGGAAATCTTGGTTCAAATCCTTCAATGCTGGATCAAAGATGTTCCTTCTTTGCATTTATTGCGATTATTTTTCCACAAATATCAT---------------AATTTGAATAGTCTA---------ATTACTT------CAAATAAATC---------------CATTTACGTCTTTTCAAAAAG---AAAGAAAAGATTCTTTTGGTTCCTACATAATTCTTATGTATATGAATGCGAATATCTATTCCTGTTTCTTCGTAAACAGTCTTCTTATTTACGATCAATATCTTCTGGAGTCTTTCTTGAGCGAACACATTTCTATGGAAAAATAGAA------TATCTTAGAGT---CGTGTGTTGTAATTCTTTTCAGAGG-ATCCTATGGTTCCTCAAAGATACTTTCATACATTATGTTCGATATCAAGGAAAAGCGATTCTGGCTTCAAAAGGAACTCTTATTCTGATGAAGAAATGGAAATTTCATCTTGTCAATTTTTGGCAATCTTATTTTCACTTTTGGTTTCAACCTTATAGGATCCATATAAAGCAATTACCCAACCATTCCTTCTCTTTTCT-GGGGTATTTTTCAAGTGTAT-TAAAAAATCCTTTGGTAGTAAGAAATCAAATGCTAGAGAATTCATTTCTAATAAATACTCTGACTAAGAAATTAGATACCATAGCCCCAGTTATTTCTCTTATTGGATCATTGTCGAAAGCTCAATTTTGTACTGTATTGGGTCATCCTATTAGTAAACCAATCTGGACCGATTTATCGGATTCTGATATTCTTGATCGATTTTGTCGGATATCTAGAAATCTTTGTCGTTATCACAGCGGATCCTCAAAGAAACAGGTTTTATATCGTATAAAGTATATACTTCGACTTTCGTGTGCTAGAACTTTGGCTCGTAAACATAAAAGTAC-AGTACGCACTTTTATGCGAAGATTAGGTTCGGGATTCTTAGAAGAATTCTTTTTGGAAGAAGAACAATCTCTTTCTT---------------------------------------------------TATGTCAATGAAAAGAA-TTTAAAAATAAAATCTTAACAA-------------------ATTGGACCTAG--CCCCT-GAATTT----------CTTAG-------ATC-TTCAAAAA----------GAAGAC---ATTCTTT-----------GTAAA-TGTCAGGAAAAATATATGGACTAT-GAATGA------------TTCAATAAC-GGAAATTCCTTGAAC---------------------ATATATATATGTTCAT-------------ATCGTA----CTAT------ACAAAA--------CAAAT-----GAGA--------TTGGATTGGAA------GAA-----GAT------------------ACGAGGATTTCT------ATTCGGATCCTTT------TGTGAAAGAACAGAG--TAAATGAAATGAGAAAG-------ATATTTCATTT--------------TGTTTAAACTGATCCA------GAGCCACTGATGGAAAGAGGAT-------------------------------GAGGATAAATA-AAGA------------------GTGAGGAA----------GTAAAAT-----GGGC-TTTTTATTGGGGATAGAGGG----------------------------------------------------------------------------------------------------------------------------------------------------------------------------------------------------------------------------------------------------------------------------------------------------------------------------------------------------------------------------------------------------------------------------------------------------------------------------------------------------------------------------------------------------------------------------------------------------------------------------------------------------------------------------------------------------------------------------------------------------------------------------------------------------------------------------------------------------------------------------------------------------------------------------------------------------------------------------------------------------

Crepidium_allanii_KFBG4610 ATGACT-CTCGGCAATGGATATCTCGGCTCTTGCATCGATGAAGAGCGCAGCAAAATGCGATACGTGATGCGAATTGCAGAATCCCGCGAACCATCGAGTTTTTGAACGCAAGTTGCGCCCGAGGCCAAC-CGGTCAAGGGCACGTTTTCCTGGGTGTCA-AGCTTTGCTTCGCTTT-----------------------------------------TCTCAAATGGTATCAAAAGGTTTTGGAGTCATTCTGGAAATCCCATTCTCGTCGCGATTAGTATTT---------TCCCTTGAAG---AAAAAACAATAACAAAATCTCAGAATTTACAATCTATTCATTCAATTTTTCCCTTTTTAGAGGATAAATTATCACATTTAAATTATGTGTCAGATCTAATAATACCCCATCCCATCCATCTGGAAATCTTGGTTCAAATTCTTCAATGTTGGATCAAAGATGTTCCTTCTTTGCATTTATTGCGATTGTTTTTCCACGAATATTCT---------------AATTTGACTAGTCTC---------ATTACTT------CAAATAAATC---------------CATTTACGTCTTTTCAAAAAG---AAAGAAAAGATTCTTTTGGTTCCTACATAATTCTTATGTATATGAATACGAATATCTATTCCTGTTTCTTCGTAAACAGTC---TTATTTACGATCAATATCTTCTGGAGTCTTTCTTGAGCGAACACATTTCTATGGAAAAATAGAT------TATCTTATAAT---CGTGTGTTGTAATTCTTTTCAGAGG-ATCCTATGGTTCCTCAAAGATACTTTCATACATTATGTTCGATATCAAGGAAAAGCGATTCTTGCTTCAAAAGGAACTATTATTCTGATGAAGAAATGGAAATTTTATTTTGTTAATCTTTGGCAATCTTATTTTCACTTTTGGTTTCAACCTTATAGGATCCATATAAAACAATTACCCCACTATTCCTTCTCTTTTCT-GGGGTATTTTTCAAGTGTAC-TAAAAAATCCTTTGGTAGTAAGAAATCAAATGCTAGATAATTCATTTCTAATAAATACTCTGACTGAGAAATTAGATACCATAGTCCCAGTTATTTCTCTTATTGGATCATTGTCAAAAGCTCAATTTTGTACTTTATTGGGTCATCCTATTAGTAAACCAATCTGGACCCATTTCTCGGATTCTGATATTCTTGATCTATTTTGTCGGATATGTAGAAATCTTTGTCGTTATCACAGCGGATCCTCAAATAAACAGGTTTTGTATCGTATAAAGTATATACTTCGACTTTCGTGTGCTAGAACTTTGGCTCGTAAACATAAAAGTAC-AGTACGCACTTTTATGCGAAGGTTAGGTTCGGGATTCTTAGAAGAATTTTTT-TGGAAGAAGAACAATCTC------GAGCTATCCCGACCATTTCCCGTACATCATCCTAGCAGAGTACTTA-TATCTATGTCAATGAAAAGAA-CTAAAAAATTAAATTTGAACAA-------------------ATTGGACCTAG--CCCCT-GAAATT----------ATTAG-------ATC-TTCAAAAA----------GAAGAC---TTTTTTT-----------GTAAA-TGTAAGGAAAAT-ATATGGACTAT-GAATGA------------TTCAATAAC-GGAGATTTATTGAAC-------------------------ATATATGTTCAT-------------ATCGTA----CTAT------ACAAAA--------CAAAT-----GAGA--------TTGGATTGGAA------GAA-----GAT------------------ACGAGGATTTAT------ATTCGTATCCATT------TGTGAAAGAACAGAG--TGAATGAAATGAGAAAG-------ATATTTCATTT--------------TGTTTAAACT------------GAGCCATTGATGGAAAGAGGAT-------------------------------GAGGATAAATA-AAGA------------------GCGAGGAA-----GGGAAGTAAAAT-----GGGC-TTTTTATTGGGGA-------CTATCTAT---------AAATGGATAATACT--------TTTGTATGTATATGAATTTTTGAAGATAG--CAAT---------CCCCCAATATCT-TGT---------------TMTAAGAACAAGATATT--GGGGGATTCTTTTG---CTTCTCTATCCG---------------AATTTTYG---------TTCTTTATCAT----AAAAGTTCTC-CCCCGCC---------AATGAATGATAAGT-GCC-----TAGGTG-AAGTAT----------------AGTATAAGATAAGTCAGA----------------------AAAGTCTAAGTCTTAGTATAATACCTATACTCTTA----------CTATAA-GAT------------AAAGACTCTGAAGGATAAGGC-----TTTTCAC----------ATGAATACTTAGTAGAACGACTAACGACGAGATTTATTATCGTTTCTCGCGTGTCTCACGAAAGTGAGAGTAG-GTGCGAATTCTCCCAATTTGTGACCGACCATACGATCTGTGRTATAAATGGTAAA-TGTTCCTTTCCATTATGAATAGC-GATTGTATGGCCAATCATTGTGGGTA-TAATGGTAGATGCCCGAGACCAAGTCACTATGATTTCTTTCTCCTCCCTCCTGTT-GAGTTTTTCAATTCTTCCCGATAAATGATTAGCTACAAAAGGATTTTTTTTT-AGTGAACGTGTCAC-GGCTGATTACTCCTTTTTTTCCATTTTT-TAGATTGGCATTCTATGTCCAA----------TATCTCGATCTTAA-TCTG-AAG-TAT---------------------------AATG-ATGAATGG-AAAAAAGATAAAATCC----------TTTA-GCTAG-ATAA--------GGG-AA-GGGGC-GGATGTAG-CCAAGTGG-ATCAA-GGCAGT

Crepidium_cordilabium_PK12271 ----------------------------------------------------------------------------------------------------------------------------------------------------------------------------------------TTATTTTTCTTCTCATTTTTCT------------TCTCAAATGGTATCAGAAGGTTTTGGAGTCATTCTGGAAATCCCATTCTCGTCGCGATTAGTATTT---------TCCCTTGAAG---AAAAAACAATAACAAAATCTCAGAATTTACAATCTATTCATTCAATTTTTCCCTTTTTAGAGGATAAATTATCACATTTAAATTATGTGTCAGATCTAATAATACCCCATCCCATCCATCTGGAAATCTTGGTTCAAATTCTTCAATGTTGGATCAAAGATGTTCCTTCTTTGCATTTATTGCGATTGTTTTTCCACGAATATTCT---------------AATTTGACTAGTCTC---------ATTACTT------CAAATAAATC---------------CATTTACATCTTTTCAAAAAG---AAAGAAAAGATTCTTTTGGTTCCTACATAATTCTTATGTATATGAATACGAATATCTATTCCTGTTTCTTCGTAAACAGTC---TTATTTACGATCAATATCTTCTGGAGTCTTTCTTGAGCGAACACATTTCTATGGAAAAATAGAT------TATCTTATAAT---CGTGTGTTGTAATTCTTTTCAGAGG-ATCCTATGGTTCCTCAAAGATACTTTCATACATTATGTTCGATATCAAGGAAAAGCGATTCTTGCTTCAAAAGGAACTATTATTCTGATGAAGAAATGGAAATTTTATTTTGTTAATCTTTGGCAATCTTATTTTCACTTTTGGTTTCAACCTTATAGGATCCATATAAAACAATTACCCCACTATTCCTTCTCTTTTCT-GGGGTATTTTTCAAGTGTAC-TAAAAAATCCTTTGGTAGTAAGAAATCAAATGCTAGATAATTCATTTCTCATAAATACTCTGACTGAGAAATTAGATACCATAGTCCCAGTTATTTCTCTTATTGGATCATTGTCAAAAGCTCAATTTTGTACTTTCTTGGGTCATCCTATTAGTAAACCAATCTGGACCCATTTATCGGATTCTGATATTCTTGATCTATTTTGTCGGATATGTAGAAATCTTTGTCGTTATCACAGCGGATCCTCAAATAAACAGGTTTTGTATCGTATAAAGTATATACTTCGACTTTCGTGTGCTAGAACTTTGGCTCGTAAACATAAAAGTAC-AGTACGCACTTTTATGCGAAGGTTAGGTTCGGGATTCTTAGAAGAATTTTTTTTGGAAGAAG---------------GAGCTATCCCGACCATTTCCCGTACATCATCCTAGCAGAGTACTTA-TATCTATGTCAATGAAAAGAA-CTAAAAAATTAAATTTGAACAA-------------------ATTGGACCTAG--CCCCT-GAAATT----------CTTAG-------ATC-TTCAAAAA----------GAAGAC---TTTTTTT-----------GTAAA-TTGAAGGAAAA-TATATGGACTAT-GAATGA------------TTCAATAAC-GGAGATTTATTGAAC-------------------------ATATATGTTCATATTGTA-----------------CTAT------ACAAAA--------CAAAT-----GAGA--------TTGGATTGGAA------GAA-----GAT------------------ACGAGGATTTAT------ATTCGTATCCATT------TGTGAAAGAACAGAG--TGAATGAAATGAGAAAG-------ATATTTCATTT--------------TGTTTAAACT------------GAGCCATTGATGGAAAGAGGAT-------------------------------GAGGATAAATA-AAGA------------------GCGAGGAA-----GGGAAGTAAAAT-----GGGC-TTTTTATTGGGGATAGAGG-CTATCTAT---------AAATGGATAATACT--------TTTGTA----TATGAATTTTTGAAGATAG--CAAT---------CCCCCAATATCT-TGTT---------------CTAAGAACAAGATATT--GGGGGATTCTTTTG---CTTCTCTATCCG---------------AATTTTTG---------TTCTTTATCAT----AAAAGTTCTC-CCCCGCC---------AATGAATGATAAGT-GCC-----TAGGTG-AAGTAT----------------AGTATAAGATAAGTCAGA----------------------AAAGTCTAAGTCTTAGTATAATACCTATACTCTTA----------CTATAA-GAT------------AAAGACTCTGAAGGATAAGGC-----TTTTCAC----------ATGAATACTTAGTAGAACGACTAACGACGAGATTTATTATCGTTTCTCGCGTGTCTCACGAAAGTGAGAGTAG-GTGCGAATTCTCCCAATTTGTGACCGACCATACGATCTGTGATATAAATGGTAAA-TGTTCCTTTCCATTATGAATAGC-GATTGTATGGCCAATCATTGTGGGTA-TAATGGTAGATGCCCGAGACCAAGTCACTATGATTTCTTTCTCCTCCCTCCTGTT-GAGTTTTTCAATTCTTCCCGATAAATGATTAGCTACAAAAGGATTTTTTTTT-AGTGAACGTGTCAC-GGCTGATTACTCCTTTTTTTCCATTTTTTAGA-TTGGCATTCTATGTCCAA----------TATCTCGATCTTAA-TCTG-AAG-TAT---------------------------AATG-ATGAATGG-AAAAAAGAGAAAATCC----------TTTA-GCTAG-ATAA--------GG--AA-GGGGC-G-----------------------------

Crepidium_purpureum_SG1193 ATGACT-CTCGGCAATGGATATCTCGGCTCTTGCATCGATGAAGAGCGCAGCAAAATGCGATACGTGATGCGAATTGCAGAATCCCGCGAACCATCGAGTTTTTGAACGCAAGTTGCGCCCGAGGCCAAC-CGGTCAAGGGCACGTCTTCCTGGGTGTCA-AGCGTTGCTACGCTTTACAAGAATTATTTTTCTTCTCATTTTTCT------------TCTCAAATGGTATCAGAAGGTTTTGGAGTCATTCTGGAAATCCCATTCTCGTCGCGATTAGTATTT---------TCCCTTGAAG---AAAAAACAATAACAAAATCTCAGAATTTACAATCTATTCATTCAATTTTTCCCTTTTTAGAGGATAAATTATCACATTTAAATTATGTGTCAGATCTAATAATACCCCATCCCATCCATCTGGAAATCTTGGTTCAAATTCTTCAATGTTGGATCAAAGATGTTCCTTCTTTGCATTTATTGCGATTGTTTTTCCACGAATATTCT---------------AATTTGACTAGTCTC---------ATTACTT------CAAATAAATC---------------CATTTACGTCTTTTCAAAAAG---AAAGAAAAGATTCTTTTGGTTCCTACATAATTCTTATGTATATGAATACGAATATCTATTCCTGTTTATTCGTAAACAGTC---TTATTTACGATCAATATCTTCTGGAGTCTTTCTTGAGCGAACACATTTCTATGGAAAAATAGAT------TATCTTATAAT---CGTGTATTGTAATTCTTTTCAGAGG-ATCCTATGGTTCCTCAAAGATACTTTCATACATTATGTTCGATATCAAGGAAAAGCGATTCTTGCTTCAAAAGGAACTATTCTTCTGATGAAGAAATGGAAATTTTATTTTGTTAATCTTTGGCAATCTTATTTTCACTTTTGGTTTCAACCTTATAGGATCCATATAAAACAATTACCCCACTATTCCTTCTCTTTTCT-GGGGTATTTTTCAAGTGTAC-TAAAAAATCCTTTGGTAGTAAGAAATCAAATGCTAGATAATTCATTTCTCATAAATACTCTGACTGAGAAATTAGATACCATAGTCCCAGTTATTTCTCTTATTGGATCATTGTCAAAAGCTCAATTTTGTACTTTATTGGGTCATCCTATTAGTAAACCAATCTGGACCCATTTATCGGATTCTGATATTCTTGATCTATTTTGTCGGATATGTATAAATCTTTGTCGTTATCACAGCGGATCCTCAAATAAACAGGTTTTGTATCGTATAAAGTATATACTTCGACTTTCGTGTGCTAGAACTTTGGCTCGTAAACATAAAAGTAC-AGTACGCACTTTTATGCGAAGGTTAGGTTCGGGATTCTTAGAAGAATTTTTTTTGGAAGAAGAACAATCTCTTTCTTGAGCTATCCCGACCATTTCCCGTACATCATCCTAGCAGAGTACTTA-TATCTATGTCAATGAAAAGAA-CTAAAAAATTAAATTTGAACAA-------------------ATTGGACCTAG--CCCCT-GAAATT----------ATTAG-------ATC-TTCAAAAA----------GAAGAC---TTTTTTT-----------GTAAA-TGTAAGGAAAAT-ATATGGACTAT-GAATGA------------TTCAATAAC-GGAGATTTATTGAAC-------------------------ATATACGTTCAT-------------ATCGTA----CTAT------ACAAAA--------CAAAT-----GAGA--------TTGGATTGGAA------GAA-----GAT------------------ACGAGGATTTAT------ATTCGTATCCATT------TGTGAAAGAATAGAG--TGAATGAAATGAGAAAG-------ATATTTCATTT--------------TGTTTAAACT------------GAGCCATTGATGGAAAGAGGAT-------------------------------GAGGATAAATA-AAGA------------------GCGAGGAA-----GGGAAGTAAAAT-----GGGC-TTTTTATTGGGGATAGAGGGCTATCTAT---------AAATGGATAATACT--------TTTGTATGTATATGAATTTTTGAAGATAG--CAAT---------CCCCCAATATCT-TGT---------------TCTTAGAACAAGATATT--GGGGGATTCTTTTG---CTTCTCTATCCG---------------AATTTTCG---------TTCTTTATCAT----AAAAGTTCTC-CCCCGCC---------AATGAATGATAAGT-GCC-----TAGGTG-AAGTAT----------------AGTCTAAGATAAGTCAGA----------------------AAAGTCTAAGTCTTAGTATAATACCTATACTCTTA----------CTATAA-GAT------------AAAGACTCTGAAGGATAAGGC-----TTTTCAC----------ATGAATACTTAGTAGAACGACTAACGACGAGATTTATTATCGTTTCTCGCGTGTCTCACGAAAGTGAGAGTAG-GTGCGAATTCTCCCAATTTGTGACCGACCATACGATCTGTGATATAAATGGTAAA-TGTTCCTTTCCATTATGAATAGC-GATTGTATGGCCAATCATTGTGGGTA-TAATGGTAGATGCCCGAGACCAAGTCACTATGATTTCTTTCTCCTCCCTCCTGTT-GAGTTTTTCAATTCTTCCCGATAAATGATTAGCTACAAAAGGATTTTTTTTT-AGTGAACGTGTCAC-GGCTGATTACTCCTTTTTTTCCATTTTT-TAGATTGGCATTCTATGTCCAA----------TATCTCGATCTTAA-TCTG-AAG-TAT---------------------------AATG-ATGAATGG-AAAAAAGAGAAAATCC----------TTTA-GCTAG-ATAA--------GGG-AA-GGGGC-GGATGTAG-CCAAGTGG-ATCAA-GGCAGT

Cryptochilus_roseus_PK12087 ACGACT-CTCGGCAATGGATATCTCGGCTCTCGCATCGATGAAGAGCGCAGCGAAATGCGATACGTGGTGTGAATTGCAGAATCCCGCGAACCATCGAGTCTTTGAACGCAAGTTGCGCCCGAGGCCAAC-CGGCTGAGGGCACGTCTGCCTGGGCGTCA-AGCATTATGTCACTCCACAAGAATTCATTTTCT------------------------TCTCAAATGGTATCAGAAGGTTTTGGAGTCATTCTGGAAATTCCATTCTCGTCGCGATTAGTATCT---------TCCCTTGAAG---AAAAAAGAATACCAAAATCTCAGAATTTACGATCTATTCATTCAATATTTCCCTTTTTAGAGGATAAATTATCACATTTAAATTATGTGTCAGATCTACTAATACCCCATCCCATCCATATGGAAATCTTGGTTCAAATTCTTCAATGTTGGATCAAAGATGTTCCTTCTTTGCATTTCTTGCGATTGTTTTTCCACGAATATCAT---------------AATTTGAATAGTCTC---------ATTATTT------CAAAGAAATC---------------TATTTACGTCTTTTCAAAAAG---AAAGAAAAGATTCTTTTGGTTCCTACATAATTCTTATGTATATGAATGCGAATATCTATTCCTGTTTCTTCGTAAACAGTCTTCTTATTTACGATCAATATCTTCTGGAGTCTTTCTTGAGCGAACACATTTCTATGGAAAAATAGAA------TATCTTATAGC---CGTGTATTGTAATTCTTTTCAGAGG-ACCCTATGGTTCCTCAAAGATACTTTCATACATTATGTTCGATATCAAGGAAAAGCGATTCTGGCTTCAAAAGGAACTCTTATTCTGATGAAGAAATGGAAATTTCATCTTGTGAATTTTTGGCAATCTTATTTTCACTTTTGGTTTCAACCTTATAGGATCCATATAAAGCAATTACCCAACTATTCCTTCTCTTTTCT-GGGGTATTTTTCAAGTGTAC-TAAAAAATCCTTTGGTAGTAAGAAATCAAATGTTAGAGAATTCATTTCTAATAAATACTCTGACTAAGAAATTAGATACCATAGTCCCAGTTATTTCTCTTATTGGATCATTGTCGAAAGCTCAATTTTGTACTGTATTGGGTCATCCTATTAGTAAACCGATCTGGACCGATTTATCGGATTCTGATATTCTTGATCGATTTTGTCGGATATGTAGAAATCTTTGTCGTTATCACAGCGGATCCTCAAAGAAACAGGTTTTGTATCGTATAAAGTATATACTTCGACTTTCGTGTGCTAGAACTTTGGCTCGTAAACATAAAAGTAC-AGTACGCACTTTTATGCGAAGATTAGGTTCGGGATTCTTAGAAGAATTCTTTTTGGAAGAAGAACAATCTC------GAGCTATCCCGACCATTTCCCGTGCATCATCCTATCAGAGTACTTC-TATCTATGTCAATGAAAAGAA-CTAAAAAATAACATCTTAACAA-------------------ATTGGACCTAG--CCCCT-GAATTT----------CTTAG-------ATA----AAAAAA---------AAAGAC---ATTCTTT-----------GTAAA-TGTAAGGAAAAAGATATGGACTAT-GAATGA------------TTCAATAAC-GGAGATTCTTTGAAC-------------------------ATATATGTTCAT-------------ATCGTA----CTAT------ACAAAA--------CAAAT-----TAGA--------TTGGATTGGAA------GAAGATACGAT------------------ACGAGGATTTCT------ATTCGGATCCTTT------TGTGAAAGAACAGAG--TGAATGAAATGAGAAAG-------ATATTTAATTT--------------TGTTTAAACT------------GAGCCACTGATGGAAAGAGGAT-------------------------------GAGGATAAATA-AAGA------------------GCGAGGAA----------GTAAAAT-----GGGC-TTTTTATTGG----------CCATCTAT---------AAATGGATAATACT--------TTTGTATTCAATGAATTAGATGAAGATAG--CAAT---------CCCCCAATATCT-TGT---------------TCTAAGAACAAGATATT--GGGGGATTCTTTTG---CTTCTCTATCCG---------------AATTTTCG---------TTCTTTATCAT----AAAAGTTCTC-CCCCGCC---------AATGAATGATAAGT-GCC-----TAGGTG-AAGTAT----------------AGTATAAGATAAGTCAGA----------------------AAAGTCTAAGTCTTA-----ATACCTATACTCTTACTCTTA----CTATAA-GAT------------AAAGACTCTGAATGATAAGGC-----TTTTTAC----------ATGAATACTTAGTAGAACGACTAACGACGAGATTTATTATCGTTTCTCGCGTGTCTCACGAAAGTGAGAGTAG-GTGCGAATTCTCCCAATTTGTGACCGACCATACGATCTGTGATATAAATGGTAAA-TGTTCCTTTCCATTATGAATAGC-GATTGTATGGCCAATCATTGTGGGTA-TAATGGTAGATGCCCGAGACCAAGTCACTATGATTTCTTTCTCCTCCCTCCTGTT-GAGTTTTTCAATTCTTCCCGATAAATGATTAGCTACAAAAGGATTTTTTTTT-AGTGAACGTGTCAC-GGCTGATTACTCCTTTTTTTACATTTTT-TAAATTGGCATTCTATGTCCAA----------TATCTCGATCTTAA-TCTG-AAG-TAT---------------------------AATG-ATGAATGG-AAAAAAGAGAAAATCC----------TTYA-GCTAG-ATAA--------AGG-AA-GGGGCGGGATGTAG-CCAAGTGGGATCAA-GGCAGT

Cryptostylis_arachnites_SG1380 AGGACT-CTCGGCAATGGATATCTTGGCTCTTGCATCGATGAAGAGCGCAGCGAAATGCGATATGTGGTGTGAATTGCAGGATCCCGCGAACCATCGAGTTTTTGAACGCAAGTTGCGCCTGAGGCCGAC-TAGCCGAGGGCACGTCTGCCTGGGCGTCA-TGCATTATGTCGCTCC-----------------------------------------------------------------------------------------------------------------------------------------------------------------------------------------------------------------------------------------------------------GAAATATTTATTCAAATCCTTCAATGCTGGATCAA-GATGTTCCTTCTTTGCATTTGTTGCGATTGATTTTACACGAATATCCTAATTGGAATTTTTTGAATTTGAAGAGTCTC---------ATTACTT------CAAATAAATC---------------GATTCATGCCTTTTCAAAAAG---AAATAAAAGATTTTGTTGGTTTCTATATAACTCTTATGTATATGAATGCGAATATCTATTTCTGTTTCTTAGTAAGAAATCTTCTTATTTACGATCAACATCTTTTGGAATCTTTCTTGAGCGAACATATTTCTATGAAAAAATAGAA------GATCT---------TGAATATAAGAATTCGTTTCATAGG-ATTCTATGGTTCTTTAAAGATCCTTTCATACATTATGTTCGATATCAAGGAAAAGCAATTATGGCTTCAAAAGGAACTCTTATTCTGATGAATAAATGGAAATTTCTTCTTGTGAATTTTTGGCAATTTTATTTTCACTTTTGGTCTCAACCTTATAGGGTCCATATAAAGCAATTACCCAATTACTCCTTCTCTTTTCT-GGGGTATTTTTCAAGTGTAC-TAAAAAATACTTTAGTAGTAAGAAATCAAATGTTAGAGAATTCCTTTATAATCAATACTCTGACTAAGAGATTAGATACTATAGCTCCAGTTATTTCTCTTATTGGATCATTGTCGAAAGCTCAATTTTGTACTATATTGGGTCATCCTATTAGTAAACCAATCTGGATCAATTTATCGGATTCTGATATTCTTGATCTCTTTTGTCGGATATGTAGAAATCTTTGTCGTTATTATAGTGGATCCTCAAAAAAACAGGTTTTGTATCGTATAAAGTATATACTTCGACTTTCGTGTGCTAGAACTTTGGCTCGTAAACATAAAAGTAA-AGTACGCACTTTTTTACGAAGATTAGGTTCGAGATTTTTAGAAGAATTTCTTCTGGAAGAAGAACAAGCTTTTTCTTGAGCTATCCCAACCATTTCCC-TGCATCATCCTAGCAGGGTACTTG-TATCTATGTCAATGAAAAGAA-CGAAAAAATAAAGTATTAACAA-------------------ATTGGACTTAA--CC------------------------------------------------------AAAGGC-----------------------------------TAATGATATGTACTGT-GAATGA------------TTCAATAA---------------------------------------------------------------------------------------------------------------------------TTGAATT---------------------------------------------------------------------------------------------------------------------------------------------TTGTTTGAACT------------GAACAACTTATGAAAAAAGGAT-------------------------------GAGGGTAAAGA-AAGA------------------GTGACGA-----------------------------------------------------------------AAATGGATAATACT--------TTTGTATTCATTGAAATTCTGAAAGGTAG--CAAT---------CCCCCACTATCT-TGT--------------TTTTTAGAACAAGATAGT--GGGGGATTCTTTTG---ATTCTTTATCCG---------------AATTTTCG---------TTCTTTATCATAAAAGAAAGTTCTC-CTCCGTC---------AATGAAAGATAAGT-GCC-----TAGGTG-TAGTGAAGTATAGTATAAGAATAGTATAATAATAGTATAAGATAAGTATAAGATAAGTAAGAAAAGCATAAGTCTTAGTATA------AGACTTTGA----------CTATAA-GAT------------AAAGACTTTTCAC--------------------ATGAATAC--TTAAATACTTAGTAGAACGACTAACGACGAGATTTAGTATCGTTTCTCGCATGTCTTACGAAAGTGAGAGTAG-GTGCGAATTCTCCCAATTTGTGACCGACCATACGATCTGTTATATAAATGGTAAA-TGTTCCTTTCCATTATGAATAGC-GATTGTATGGCCAATCATTGTGGGTA-TAATGGTAGATGCCCGAGACCAAGTCACTATTATTTCTTTCTCCTCCCTCATGTT-GAGTTTTTCAATTTTTCCCGATAAATGATTAGCTACAAAAGGATTTTTTTTG-AGTGAACGTGTCAC-GGCTGATTACTCCTTTTTTTACATTTTG-GAAATTGGCATTCTATGTCCAA----------TATCTCGATTTGAA-TTGG---------------------------------------------------------------------------------------------------------------------------------------------

Curculigo_orchioides_PK12054 -------------------------------------------------------------------------------------------------------------------------------------------------------------------------------------------------------------------------------------------------------------------------------------------------------------------------------------------------------------------------------------------------------------------------------------------------------------------------------------------------------------------------------------------------------------------------------------------------------------------------------------------------------------------------------------------------------------------------------------------------------------------------------------------------------------------------------------------------------------------------------------------------------------------------------------------------------------------------------------------------------------------------------------------------------------------------------------------------------------------------------------------------------------------------------------------------------------------------------------------------------------------------------------------------------------------------------------------------------------------------------------------------------------------------------------------------------------------------------------------GAGCTATCCCGACTATCTCCCGTGCATCATCCTAGCAGATGACTTG-TATCTATGTCAATTAAGGGTA-CTAAAAAA-----TATTCAAAA-------------------ATTTTACCTAG--TCCCT-GAATTT----------CTTAAA------------------------------------------------------------------------------------------------------------------------------------------------------------------------------------------------------------------------------------------------------------------------------------------------------------------------------TATGAAAAA-------------------------------------------------------------------------------------------AAAAAA-----------------------------------GAGGATAAATA-----------------------GTTAGGAA----------GTAAAAT-----GGGCTTTTTTATTGGGGATAGAGGGCCATCTAC---------AAATGGATAAGACT--------TCCATATAAATACGAATCGTTGAAGGGTC--AATA---------CCCCC--TATCT-TTT------------------TTTAGCAAGATA-----GGGG--------------------------------------------------------------------------GTATCTC-CCTCCCT--------------------------------TGTTTG----------------------------------------------------------------------------------------------------------------------------------ATTCTTTTTTTCA-------------TTTTCAT-------------------------------TAACGACGAGATTTATTATCGTTTCTCGCATGTCTCGCGAAAGTCAGAGTAG-GCGCAAATTCTCCCAATTTGTGACCTACCATACGATCCGTTATATAAATGGTAAA-TGTTCCTTTCCATTATGAATAGC-GATTGTATGGCCAATCATTGTGGGTA-TAATGGTAGATGCCCGAGACCAAGTTACTATTATTTCTTTCTCCTCCCTCATGTT-GAGTTTTTCAATTTTTCCCGATAAATGATTAGCTACAAAAGGATTTTTTTTT-AGTGAACGTGTCAC-AGCGGATTACTCCTTTTTTTACATTTTA-AAGATTGGCATTCTATGTCCAA----------TATCTCGATCTAAG-TATG-AAGGTAA---------GAATAAATACAAT-----AATG-ATGAACGG-AAAAAAGAGAAAATCC----------TTTA-GCTAG-ATAA---------------GGGGC-GGATGTAG-CCAAGTGG-ATCAA-GGCAGT

Curculigo_orchioides_SG1196 ------------------------------------------------------------------------------------------------------------------------------------------------------------------------------------------------------------------------------------------------------------------------------------------------------------------------------------------------------------------------------------------------------------------CACTAATACCCTATCCCATCCATCGGGAAATCCTGGTTCAAATTCTTCAATGCTGGATCCAAGATGTTCCCTCTTTGCATTTATTTCAATTCTTTCTCCACGAATATCATAATTG------------TTGGAATAGTCCC---------ATTACTC------CGAAGAAATC---------------TATTTATGTTTTTTCAAAAGA---AAATAAAAGATTATTTAGATTCTTATATAATTCTTATGTATCTGAATGCGAATTTGTATTCGTTTTTCTTCGTAAACAATCTTCTTATTTACGATCAATAACTTTTGGAGCTTTTCTTGAGCGAATACATTTCTATGGAAAAATAGAA------CATCTTAAGGT---AGTGTGTCATAATTATTTTCCGAGG-GCCCTATGGTCTTTTAAGGATCCTTTCATGCATTATGTTCGATATCAAGGAAAGGCAATTCTAGCATCAAAAGGGAACCATCTTATGATGAAGAAATGGAAATGTTATCTTGTCAATTTCTGGCAATATTATTTTCATTTTTGGTCTCAACCATACAGGATTCATATAAACCAATTATCAAACTATTCCTTCTATTTTCT-AGGTTATCTGTCAAATGTAC-TAATAAATCCCTTGACAGTAAGGAATCAAATGTTAGAGGATTCCTATCTAATAGATACTGTTACTAAGAAATTCGATACCATAGTCCCAGTTATTCTTCTTATTGGATCATTGTCTAAAGCGAAATTTTGTACCGTATCGGGGCATCCTATTAGTAAGCCGCTCTGGGCTGATTTATCAGATTCTGATATTATTGATCGATTTGGTTGGATATGTAGAAATCTTTCTCATTATTACAGTGGCTCTTCAAAAAAACAAGGTTTGTATCGAATAAAGTATGTACTTCGACTTTCTTGTGCTAGAACTTTGGCTCGGAAACATAAAAGTAC-GGTACGCACTTTTATGCGAAGATTAGGTTCGGGGTTATTAGAGGAATTCTTTACGGAAGAAGAACAAGTGAAACGTTGAGCTATCCCGACTATCTCCCGTGCATCATCCTAGCAGATGACTTG-TATCTATGTCAATTAAGGGTA-CTAAAAAA-----TATTCAAAA-------------------ATTTTACCTAG--TCCCT-GAATTT----------CTTAAA------------------------------------------------------------------------------------------------------------------------------------------------------------------------------------------------------------------------------------------------------------------------------------------------------------------------------TATGAAAAAA------------------------------------------------------------------------------------------AAAAAA-----------------------------------GAGGATAAATA-----------------------GTTAGGAA----------GTCAAAT-----GGGCTTTTTTATTGGGGATAGAGGGCCATCTAC---------AAATGGATAAGACT--------TCCATATAAATACGAATCGTTGAAGGGTC--AATA---------CCCCC--TATCT-TGC------------------TAAAAAAAGATA-----GGGG--------------------------------------------------------------------------GTATCTC-CCTCCCT--------------------------------TGTTTG----------------------------------------------------------------------------------------------------------------------------------ATTCTTTTTTTCA-------------TTTTCAT-------------------------------TAACGACGAGATTTATTATCGTTTCTCGCATGTCTCGCGAAAGTCAGAGTAG-GCGCAAATTCTCCCAATTTGTGACCTACCATACGATCCGTTATATAAATGGTAAA-TGTTCCTTTCCATTATGAATAGC-GATTGTATGGCCAATCATTGTGGGTA-TAATGGTAGATGCCCGAGACCAAGTTACTATTATTTCTTTCTCCTCCCTCATGTT-GAGTTTTTCAATTTTTCCCGATAAATGATTAGCTACAAAAGGATTTTTTTTT-AGTGAACGTGTCAC-AGCGGATTACTCCTTTTTTTACATTTTA-AAGATTGGCATTCTATGTCCAA----------TATCTCGATCTAAG-TATG-AAGGTAA---------GAATAAATACAAT-----AATG-ATGAACGG-AAAAAAGAGAAAATCC----------TTTA-GCTAG-ATAA---------------GGGGC-GGATGTAG-CCAAGTGG-ATCAA-GGCAGT

Cymbidium_aloifolium_KFBG2049 ACGACT-CTCGGCAATGGATATCTCGGCTCTCGCATCGATGAAGAGCGCAGCGAAATGCGATACGTGGTGCGAATTGCAGAATCCCGCGAACCATCGAGTCTTTGAACGCAAGTTGCGCCCGAGGCCAGC-CGGCCGAGGGCACGTCCGCCTGGGCGTCA-AGCATCGCGTCGCTCC----------------------TTTTTAT------------TCTCAAATGATATCAGAAGTTTTTGGAGTCATTCTGGAAATTCCATTCTCATCACGATTAGTATCT---------TTCCTTGAAG---AAAAACGAATACCAAAATCTCAGAATTTACGATCTATTCATTCAATATTTCCCTTTTTAGAGGATAAATTATCACATTTAAATTATGTGTCAGATCTACTAATACCCCATCCTATACATTTGGAAATCTTGGTTCAAATCCTTCAATGCTGGATCAAAGATGTTCCTTCTTTGCATTTCTTGCGATTGTTTTTCCACGAATATCAT---------------AATTTGAATAGTCTG---------ATTACTT------CAAAGAAATC---------------TATTTACGTCTTTTCAAAAAG---AAATAAAAGATTCTTTTGGTTCCTGCATAATTCTTATGTATATGAATTTGAATATCTATTCTTATTTCTTCGTAAACAGTCTTCTTATTTACGATCAATATCTTCTGGAATCTTTATTGAGCGAACACTTTTCTTTGGAAAAATAGAA------TATCTTATGGT---CGTATGTTGTAATTCTTTTCAGAGT-ATCCTATGGTTCCTCAAAGATACTTTCATACATTATGTTCGATATAAAGGAAAAGCGATTCTGGCTTCAAAAGGAACTCTTATTCTGATGAAGAAATGGAAATCTCATCTTGTGAATTTTTGGCAATCTTATTTTCACTTTTGGTTTCAACCTTATAGGATCCATATAAAGCAATTACCCAACTATTCCTTTTCTTTTCT-GGGGTATTTTTCAAGTGTAC-TAAAAAATCCTTTGGTAGTAAGAAATCAAATGCTAGAGAATTCATTTCTAATAAATACTCTGACTAATAAATTAGATACCGTAGCCCCAGTTATTTCTCTTATTGGATCATTGTCGAAAGCTCAATTTTGTACTGTATTGGGTAATCCTATAAGTAAACCGATCTGGACCGATTTATCGGATTCTGATATTATTGATCGATTTTGTCGGATATGTAGAAATCTTTGTCATTATCACAGCGGATCCTCAAAGAAGCAGGTTTTGTATCGTATAAAGTATATACTTCGACTTTCATGTGCTAGAACTTTGGCTCGTAAACATAAAAGTAC-AGTACGCACTTTTATGCGAAGATTAGGTTCGGGATTCTTAGAAGAATTTTTTTTGGAAGA-----------------GAGCTATCCCGACCATTTCCCGTGCATCATCTTAGCAGAATACTTC-TATCTATGTCAATG--------------------------------------------------------------------AATTT-----------CTTAG-------ATC-TTCAAAAA----------GAAGAC---TTTCTTTGTTT-------GTAAA-TGTAAGTAAAAAAATATGGACTAT-AAATAA------------TTCAATAAC-GGAGATTCCTTGAAC-------------------------ATATATGTTCAT-------------ATTGTA----TTAT------ACAAAA--------CAAAT-----GAGA--------TTGGGTTGGAA------AAA-----GAT------------------ACGAAGATTTCT------ATTCGGATCCATT------TGTGAAAGAACAGAG--TGAATGAAATGAGAAAG-------ATATTTCAGTT--------------TGTTTAAACTGA----------GCTCCACTGATGGAAAGAGGAT-------------------------------GGGGATAAAGA-AAAA------------------GTGAGGAA----------GTAAAAT-----GGGC-TTTTTATTGGGGATAGAGGACCATCTAT---------AAATGGATAAGACT--------CTTGTATTCATATGAGTTTTTGAAGATAG--CAAT---------CCCCCAATATCT-TGTGT-------------TCTTAAAACAAGATATT--GGGGGATTTTTTTG---CTTCTCTATCCG---------------AATTTTCG---------TTCTTTATCAT----AAAAGTTCTC-CCCCGCC---------AATGAATGATAAGT-GCC-----TAGGTG-AAGTAT----------------AGTATAAGATAAGTCAGA----------------------AAAGTCTAAGTCTTA-----ATACCTCTACTCTGA----------CTATAA-GAT------------AAAGACTCTTAAGGATAAGGC-----TTTTCGC----------ATGAATACTTAGTAGAACGACTAACGACGAGATTTCTTATCGTTTCTTGCGTGTCTCACGAAAGTGAGAGTAG-GTGCAAATTCTCCCAATTTGTGACCGACCATACGATCTGTGATATAAATGGTAAA-TGTTCCTTTCCATTATGAATAGC-GATTGTATGGCCAATCATTGTGGGTA-TAATGGTAGATGCCCGAGACCAAGTCACTATGATTTCTTTCTCCTCCCTCCTGTT-GAGTTTTTCAATTCTTCCCGATAAATGATTAGCTACAAAAGGATTTTTTTTG-AGTGAACGTGTCAC-GGCTGATTACTCCTTTTTTTACATTTTT-GAAATGGGCATTCTATGTCCAA----------TATCTCGATCTGAA-TCTG-AAG-TAT---------------------------AATG-ATGAATGG-AAANAAGAGAAAATCC----------TTTA-GCTAG-ATAA--------GGA-AA--GGGC-GGATGTAG-CCAAGTGG-ATCAA-GGCAGT

Cymbidium_ensifolium_SG1214 ACGACT-CTCGGCAATGGATATCTCGGCTCTCGCATCGATGAAGAGCGCAGCGAAATGCGATACGTGGTGCGAATTGCAGAATCCCGCGAACCATCGAGTCTTTGAACGCAAGTTGCGCCCGAGGCCAGC-CGGCCGAGGGCACGTCCGCCTGGGCGTCA-AGCATCGCGTCGCTCCACAAGAATTCTTTTTCTTCTCATTTTTAT------------TCTCAAATGATATCAGAAGTTTTTGGAGTCATTCTGGAAATTCCATTCTCATCACGATTAGTATCT---------TCCCTTGAAG---AAAAACGAATACCAAAATCTCAGAATTTACGATCTATTCATTCAATATTTCCCTTTTTAGAGGATAAATTATCACATTTAAATTATGTGTCAGATCTATTAATACCCCATCCTATCCATTTGGAAATCTTGGTTCAAATCCTTCAATGCTGGATCAAAGATGTTCCTTCTTTGCATTTCTTGCGATTGTTTTTCCACGAATATCAT---------------AATTTGAATAGTCTG---------ATTACTT------CAAAGAAATC---------------TATTTACGTCTTTTCAAAAAG---AAAGAAAAGATTCTTTTGGTTCCTACATAATTCTTATGTATATGAATTTGAATATCTATTCCTATTTCTTCGTAAACAGTCTTCTTATTTACGATCAATATCTTCTGGAATCTTTATTGAGCGAACACTTTTCTTTGTAAAAATAGAA------TATCTTATGGT---CGTGTGTTGTAATTCTTTTCAGAGG-ATCCTATGGTTCCTCAAAGATACTTTCATACATTATGTTCGATATAAAGGAAAAGCGATTCTGGCTTCAAAAGGAACTCTTATTCTGATGAAGAAATGGAAATTTCATCTTGTGAATTTTTGGCAATCTTATTTTCACTTTTGGTTTCAACCTTATAGGATCCATATAAAGCAATTACCCAACTATTCCTTCTCTTTTAT-GGGGTATTTTTCAAGTGTAC-TAAAAAATCCTTTGGTAGTAAGAAATCAAATGCTAGAGAATTCATTTCTAATAAATACTCTGACTAATAAATTAGATACCATAGCCCCAGTTATTTCTCTTATTGGATCGTTGTCGAAAGCTCAATTTTGTACTGTATTGGGTAATCCTATAAGTAAACCGATCTGGACCGATTTATCGGATTCTGATATTATTGATCGATTTTGTCGGATATGTAGAAATCTTTGTCATTATCACAGTGGATCCTCAAAGAAGCAGGTTTTGTATCGTATAAAGTATATACTTCGACTTTCGTGTGCTAGAACTTTGGCTCGTAAACATAAAAGTAC-AGTACGCACTTTTATGCGAAAATTAGGTTCGGGATTCTTAGAAGAATTTTTTTTGGAAAAAGAACAATCTCTTTCTTGAGCTATCCCGACCATTTCCCGTGCATCATCTTAGCAGAATACTTA-TATCTATGTCAATG--------------------------------------------------------------------AATTT-----------CTTAG-------ATC-TTCAAAAA----------AAAGAC---TTTCTTTGTTT-------GTAAA-TGTAAGTAAAAAAATATGGACTAT-GAATAA------------TTCAATAAC-GGAGATTCCTTGAAC-------------------------ATATATGTTCAT-------------ATTGTA----TTAT------ACAAAA--------CAAAT-----GAGA--------TTGGATTGGAA------AAA-----GAT------------------CCGAAGATTTCT------ATTCGGATCCATT------TGTGAAAGAACAGAG--TGAATGAAATTAGAAAG-------ATATTTCAGTT--------------TTTTTAAACTGA----------GTTCCACTGATGGAAAGAGGAT-------------------------------GAGGATAAATA-AAAA------------------GTGAGGAA----------GTAAAAT-----GGGC-TTTTTATTGGGGATAGAGGGCCATCTAT---------AAATGGATAAGACT--------TTTGTATTCATATGAATTTTTGAAGATAG--CAAT---------CCCCCAATATCT-TGTGT-------------TCTTAGAACAAGATATT--GGGGGATTTTTTTG---CTTCTCTATCCG---------------AATTCTCG---------TTCTTTATCAT----AAAAGTTCTC-CCCCGCC---------AATGAATGATAAGT-GCC-----TAGGTG-AAGCAT----------------AGTATAAGATAAGTCAGA----------------------AAAGTCTAAGTCTTA-----ATACCTCTACTCTTA----------CTATAA-GAT------------AAAGACTCTTAAGGATAAGGC-----TTTTCAC----------ATGAATACTTAGTAGAACGACTAACGACGAGATTTATTATCGTTTCTCGCGTGTCTCACGAAAGTGAGAGTAG-GTGCAAATTCTCCCAATTTGTGACCGACCATACGATCTGTGATATAAATGGTAAA-TGTTCCTTTCCATTATGAATAGC-GATTGTATGGCCAATCATTGTGGGTA-TAATGGTAGATGCCCGAGACCAAGTCACTATGATTTCTTTCTCCTCCCTCCTGTT-GAGTTTTTCAATTCTTCCCGATAAATGATTAGCTACAAAAGGATTTTTTTTG-AGTGAACGTGTCAC-GGCTGATTACTCCTTTTTTTACATTTTT-GAAATTGGCATTCTATGTCCAA----------TATCTCGATCTTAA-TCTG-AAG-TAT---------------------------AATG-ATGAATGG-AAAAAAGAGAAAATCC----------TTTA-GCTAG-ATAA--------GGG-AA-GGGGC-GGATGTAG-CCAAGTGG-ATCAA-GGCAGT

Cymbidium_kanran ACGACT-CTCGGCAATGGATATCTCGGCTCTCGCATCGATGAAGAGCGCAGCGAAATGCGATACGTGGTGCGAATTGCAGAATCCCGCGAACCATCGAGTCTTTGAACGCAAGTTGCGCCCGAGGCCAGC-CGGCCGAGGGCACGTCCGCCTGGGCGTCA-AGCATCGCGTCGCTCCACAAGAATTCTTTTTCTTCTCATTTTTAT------------TCTCAAATGATATCAGAAGTTTTTGGAGTCATTCTGGAAATTCCATTCTCATCGCGATTAGTATCT---------TCCCTTGAAG---AAAAACGAATACCAAAATCTCAGAATTTACGATCTATTCATTCAATATTTCCCTTTTTAGAGGATAAATTATCACATTTAAATTATGTGTCAGATCTATTAATACCCCATCCTATCCATTTGGAAATCTTGGTTCAAATCCTTCAATGCTGGATCAAAGATGTTCCTTCTTTGCATTTCTTGCGATTGTTTTTCCACGAATATCAT---------------AATTGGAATAGTCTG---------ATTACTT------CAAAGAAATC---------------TATTTACGTCTTTTCAAAAAG---AAAGAAAAGATTCTTTTGGTTCCTACATAATTCTTATGTATATGAATTGGAATATCTATTCCTATTTCTTCGTAAACAGTCTTCTTATTTACGATCAATATCTTCTGGAGTCTTTCTTGAGCGAACACTTTTCTTTGGAAAAATAGAA------TATCTTATGGT---CGTGTGTTGTAATTCTTTTCAGAGG-ATCCTATGGTTCCTCAAAGATACTTTCATACATTATGTTCGATATAAAGGAAAAGCGATTCTGGCTTCAAAAGGAACTCTTATTCTGATGAAGAAATGGAAATTTCATCTTGTGAATTTTTGGCAATCTTATTTTCACTTTTGGTTTCAACCTTATAGGATCCATATAAAGCAATTACCCAACTATTCCTTCTCTTTTCT-GGGGTATTTTTCAAGTGTAC-TAAAAAATCCTTTGGTAGTAAGAAATCAAATGCTAGAGAATTCATTTCTAATAAATACTCTGACTAATAAATTAGATACCATAGCCCCAGTTATTTCTCTTATTGGATCATTGCCCAAAGCTCAATTTTGTACTGTATTGGGTAATCCTATAAGTAAACCGATCTGGACCGATTTATCGGATTCTGATATTATTGATCGATTTTGTCGGATATGTAGAAATCTTTGTCATTATCACAGTGGATCCTCAAAGAAGCAGGTTTTGTATCGTATAAAGTATATACTTCGACTTTCGTGTGCTAGAACTTTGGCTCGTAAACATAAAAGTAC-AGTACGCACTTTTATGCGAAAATTAGGTTCGGGATTCTTAGAAGAATTTTTTTTGGAAGAAGAACAATCTCTTTCTT-----------------------------------------------TATCTATGTCAATGAAAAGAA-CTAAAAAAGAAAATCTTCACAA-------------------ATTGGCCCTAG--CCCCT-GAATTT----------CTTAG-------ATC-TTCAAAAA----------G-------------TT-----------GTCAA-TGTAAGTAAAAAGATATGGACTAT-GAATGA------------TTCCATAAC-GGAGATTCCTTTCAC-------------------------ATATATGTTCAT-------------ATCGTA----TTAT------ACAAAA--------CAAAT-----GAGA--------TTGGATTGGAA------GAA-----GAT------------------ACGAGGATTTCT------ATTCGTATCCATT------TGTGAAAGAACAGAG--TGAATGAAATGAGAAAG-------ATATTTCATCT--------------TGTTTCAACTGA----------G--CCACTGATGGAAAGAGGAT-------------------------------GAGGATAAAGA-AAAA------------------GCGAGGAA----------GTAAAAT-----GGGC-TTTTTATTGGGGATAGAGGG------------------------TAAGACT--------TTTGTATTCATATGAATTTTTGAAGATAG--CAAT---------CCCCCAATATCT-TGTGT----------------------------------------TTTTTG---CTTCTCTATCCG---------------AATTCTCG---------TTCTTTATCAT----AAAAGTTCTC-CCCCGCC---------AATGAATGATAAGT-GCC-----TAGGTG-AAGCAT----------------AGTATAAGATAAGTCAGA----------------------AAAGTCTAAGTCTTA-----ATACCTCTACTCTTA----------CTATAA-GAT------------AAAGACTCTTAAGGATAAGGC-----TTTTCAC----------ATGAATACTTAGTAGAACGACTAACGACGAGATTTATTATCGTTTCTCGCGTGTCTCACGAAAGTGAGAGTAG-GTGCAAATTCTCCCAATTTGTGACCGACCATACGATCTGTGATATAAATGGTAAA-TGTTCCTTTCCATTATGAATAGC-GATTGTATGGCCAATCATTGTGGGTA-TAATGGTAGATGCCCGAGACCAAGTCACTATGATTTCTTTCTCCTCCCTCCTGTT-GAGTTTTTCAATTCTTCCCGATAAATGATTAGCTACAAAAGGATTTTTTTTG-AGTGAACGTGTCAC-GGCTGATTACTCCTTTTTTTACATTTTT-GAAATTGGCATTCTATGTCCAA----------TATCTCGATCTTAA-TCTG-AAG-TAT---------------------------AATG-ATGAATGG-AAAAAAGAGAAAA-------------------------------------------------------------------------------

Cymbidium_lancifolium_SG1274 ACGACT-CTCGGCAATGGATATCTCGGCTCTCGCATCGATGAAGAGCGCAGCGAAATGCGATACGTGGTGCGAATTGCAGAATCCCGCGAACCATCGAGTCTTTGAACGCAAGTTGCGCCCGAGGCCAGC-CGGCCGAGGGCACGTCCGCCTGGGCGTCA-AGCGTCGCGTCGCTCCACAAGAATTCTTTTTCTTCTCATTTTTAT------------TCTCAAATGATATCAGAAGTTTTTGGAGTCATTCTGGAAATTCCATTCTCATCACGATTAGTATCT---------TCCCTTGAAG---AAAAACGAATACCAAAATCTCAGAATTTACGATCTATTCATTCAATATTTCCCTTTTTAGAGGATAAATTATCACATTTAAATTATGTGTCAGATCTACTAATACCCCATCCTATCCATTTGGAAATCTTGGTTCAAATCCTTCAATGCTGGATCAAAGATGTTCCTTCTTTGCATTTCTTGCGATTGTTTTTCCACGAATATCAT---------------AATTTGAATAGTCTG---------ATTACTT------CAAAGAAATC---------------TATTTACGTCTTTTCAAAAAG---AAAGAAAAGATTCTTTTGGTTCCTACATAATTCTTATGTATATGAATTTGAATATCTATTCCTATTTCTTCGTAAACAGTCTTCTTATTTACGATCAATATCTTCTGGAGTCTTTATTGAGCGAACACTTTTCTTTGGAAAAATAGAA------TATCTTATGGT---CGTGTGTTGTAATTCTTTTCAGAGG-ATCCTATGGTTCCTCAAAGATACTTTCATACATTATGTTCGATATAAAGGAAAAGCGATTCTGGCTTCAAAAGGAACTCTTCTTCTGATGAAGAAATGGAAATTTCATCTTGTGAATTTTTGGCAATCTTATTTTCACTTTTGGTTTCAACCTTATAGGATCCATATAAAGCAATTACCCAACTATTCCTTCTCTTTTAT-GGGGTATTTTTCAAGTGTAC-TAAAAAATCCTTTGGTAGTAAGAAATCAGATGCTAGAGAATTCATTTCTAATAAATACTCTGACTAATAAATTAGATACCATAGCCCCAGTTATTTTTCTTATTGGATCATTGTCGAAAGCTCAATTTTGTACTGTATTGGGTAATCCTATAAGTAAACCGATCTGGACCGATTTATCGGATTCTGATATTATTGATCGATTTTGTCGGATATGTAGAAATCTTTGTCATTATCACAGTGGATCCTCAAAGAAGCAGGTTTTGTATCGTATAAAGTATATACTTCGACTTTCGTGTGCTAGAACTTTGGCTCGTAAACATAAAAGTAC-AGTACGCACTTTTATGCGAAAATTAGGTTCGGGATTCTTAGAAGAATTTTTTTTGGAAAAAGAACAATCTCTTTCTTGAGCTATCCCGACCATTTCCCGTGCATCATCTTAGCAGAATACTTA-TATCTATGTCAATG--------------------------------------------------------------------AATTT-----------CTTAG-------ATC-TTCAAAAA----------GAAGAC---TTTCTTTGTTT-------GTAAA-TGTAAGTAAAAAAATATGGACTAT-GAATAT------------TTCAATAAC-GGAGATTCCTTGAAC-------------------------ATATATGTTCAT-------------ATTGTA----TTAT------ACAAATATACAAAACAAAT-----GAGA--------TTGGATTGGAA------AAA-----GAT------------------CCGAAGATTTCT------ATTCGGATCCATT------TGTGAAAGAACAGAG--TGAATGAAATGAGAAAG-------ATATTTCAGTT--------------TGTTTAAACTGA----------GTTCCACTGATGGAAAGAGGAT-------------------------------GAGGATAAATA-AAAA------------------GTGAGGAA----------GTAAAAT-----GGGC-TTTTTATTGGGGATAGAGGGCCATCTAT---------AAATGGATAAGACT--------TTTGTATTCATATGAATTTTTGAAGATAG--CAAT---------CCTCCAATATCT-TGTGT-------------TTTTAGAACAAGATATT--GGGGGATTTTTTTG---CTTCTCTATCCG---------------AATTTTCG---------TTCTTTATCAT----AAAAGTTCTC-CCCCGCC---------AATGAATGATAAGT-GCC-----TAGGTG-AAGCAT----------------AGTATAAGATAAGTCAGA----------------------AAAGTCTAAGTCTTA-----ATACCTCTACTCTTA----------CTATAA-GAT------------AAAGACTCTTAAGGATAAGGC-----TTTTCAC----------ATGAATACTTAGTAGAACGACTAACGACGAGATTTATTATCGTTTCTCGCGTGTCTCACGAAAGTGAGAGTAG-GTGCAAATTCTCCCAATTTGTGACCGACCATACGATCTGTGATATAAATGGTAAA-TGTTCCTTTCCATTATGAATAGC-GATTGTATGGCCAATCATTGTGGGTA-TAATGGTAGATGCCCGAGACCAAGTCACTATGATTTCTTTCTCCTCCCTCCTGTT-GAGTTTTTCAATTCTTCCCGATAAATGATTAGCTACAAAAGGATTTTTTTTG-AGTGAACGTGTCAC-GGCTGATTACTCCTTTTTTTACATTTTT-GAAATTGGCATTCTATGTCCAA----------TATCTCGATCTTAA-TCTG-AAG-TAT---------------------------AATG-ATGAATGG-AAAAAAGAGAAAATCC----------TTTA-GCTAG-ATAA--------GGG-AA-GGGGC-GGATGTAG--CCAGTGG-ATCAA-GGCAGT

Cymbidium_sinense_SG1342 ACGACT-CTCGGCAATGGATATCTCGGCTCTCGCATCGATGAAGAGCGCAGCGAAATGCGATACGTGGTGCGAATTGCAGAATCCCGCGAACCATCGAGTCTTTGAACGCAAGTTGCGCCCGAGGCCAGC-CGGCCGAGGGCACGTCCGCCTGGGCGTCA-AGCATCGCGTCGCTCCACAAGAATTCTTTTTCTTCTCATTTTTAT------------TCTCAAATGATATCAGAAGTTTTTGGAGTCATTCTGGAAATTCCATTCTCATCACGATTAGTATCT---------TCCCTTGAAG---AAAAACGAATACCAAAATCTCAGAATTTACGATCTATTCATTCAATATTTCCCTTTTTAGAGGATAAATTATCACATTTAAATTATGTGTCAGATCTATTAATACCCCATCCTATCCATTTGGAAATCTTGGTTCAAATCCTTCAATGCTGGATCAAAGATGTTCCTTCTTTGCATTTCTTGCGATTGTTTTTCCACGAATATCAT---------------AATTTGAATAGTCTG---------ATTACTT------CAAAGAAATC---------------TATTTACGTCTTTTCAAAAAG---AAAGAAAAGATTCTTTTGGTTCCTACATAATTCTTATGTATATGAATTTGAATATCTATTCCTATTTCTTCGTAAACAGTCTTCTTATTTACGATCAATATCTTCTGGAATCTTTATTGAGCGAACACTTTTCTTTGGAAAAATAGAA------TATCTTATGGT---CGTGTGTTGTAATTCTTTTCAGAGG-ATCCTATGGTTCCTCAAAGATACTTTCATACATTATGTTCGATATAAAGGAAAAGCGATTCTGGCTTCAAAAGGAACTCTTATTCTGATGAAGAAATGGAAATTTCATCTTGTGAATTTTTGGCAATCTTATTTTCGCTTTTGGTTTCAACCTTATAGGATCCATATAAAGCAATTACCCAACTATTCCTTCTCTTTTAT-GGGGTATTTTTCAAGTGTAC-TAAAAAATCCTTTGGTAGTAAGAAATCAAATGCTAGAGAATTCATTTCTAATAAATACTCTGACTAATAAATTAGATACCATAGCCCCAGTTATTTCTCTTATTGGATCGTTGTCGAAAGCTCAATTTTGTACTGTATTGGGTAATCCTATAAGTAAACCGATCTGGACCGATTTATCGGATTCTGATATTATTGATCGATTTTGTCGGATATGTAGAAATCTTTGTCATTATCACAGTGGATCCTCAAAGAAGCAGGTTTTGTATCGTATAAAGTATATACTTCGACTTTCGTGTGCTAGAACTTTGGCTCGTAAACATAAAAGTAC-AGTACGCACTTTTATGCGAAAATTAGGTTCGGGATTCTTAGAAGAATTTTTTTTGGAAAAA----------------GAGCTATCCCGACCCTTTCCCGTGCATCATCTTAGCAGAATACTTA-TATCTATGTCAATT--------------------------------------------------------------------AATTT-----------YTTAG-------ATC-TTCAAAAA----------AAAGAC---TTTCTTTGTTT-------GTAAA-TGTAAGTAAAAAAATATGGACTAT-GAATAA------------TTCAATAAC-GGAGATTCCTTGAAC-------------------------ATATATGTTCAT-------------ATTGTA----TTAT------ACAAAA--------CAAAT-----GAGA--------TTGGATTGGAA------AAA-----GAT------------------CCGAAGATTTCT------ATTCGGATCCATT------TGTGAAAGAACAGAG--TGAATGAAATTAGAAAG-------ATATTTCAGTT--------------TTTTTAAACTGA----------GTTCCACTGATGGAAAGAGGAT-------------------------------GAGGATAAATA-AAAA------------------GTGAGGAA----------GTAAAAT-----GGGC-TTTTTATTGGGGATAGAGGGCCATCTAT---------AAATGGATAAGACT--------TTTGTATTCATATGAATTTTTGAAGATAG--CAAT---------CCCCCAATATCT-TGTGT-------------TCTTAGAACAAGATATT--GGGGGATTTTTTTG---CTTCTCTATCCG---------------AATTCTCG---------TTCTTTATCAT----AAAAGTTCTC-CCCCGCC---------AATGAATGATAAGT-GCC-----TAGGTG-AAGCAT----------------AGTATAAGATAAGTCAGA----------------------AAAGTCTAAGTCTTA-----ATACCTCTACTCTTA----------CTATAA-GAT------------AAAGACTCTTAAGGATAAGGC-----TTTTCAC----------ATGAATACTTAGTAGAACGACTAACGACGAGATTTATTATCGTTTCTCGCGTGTCTCACGAAAGTGAGAGTAG-GTGCAAATTCTCCCAATTTGTGACCGACCATACGATCTGTGATATAAATGGTAAA-TGTTCCTTTCCATTATGAATAGC-GATTGTATGGCCAATCATTGTGGGTA-TAATGGTAGATGCCCGAGACCAAGTCACTATGATTTCTTTCTCCTCCCTCCTGTT-GAGTTTTTCAATTCTTCYCGATAAATGATTAGCTACAAAAGGATTTTTTTTG-AGTGAACGTGTCAC-GGCTGATTACTCCTTTTTTTACATTTTT-GAAATTGGCATTCTATGTCCAA----------TATCTCGATCTTAA-TCTG-AAG-TAT---------------------------AATG-ATGAATGG-AAAAAAGAGAAAATCC----------TTTA-GCTAG-ATAA--------GGG-AA-GGGGC-GGATGTAG-CCAAGTGG-ATCAA-GGCAGT

Dendrobium_aduncum_KFBG8766 ACGACT-CTCGGCAATGGATATCTCGGCTCTCGCATCGATGAAGAGCGCAGCGAAATGCGATATGTGGTGCGAATTGCAGAATCCCGCGAACCATCGAGTCTTTGAACGCAAGTTGCGCCCGAGGCCAAC-CGGCTAAGGGCACGTCCGCCTGGGCGTCA-AGCATTTTATCGCTCCACAAG-ATTCTTTTTCTTCTCATTTTTCT------------TCGCAAATGGTATCAGAAGGTTTTGGAGTCATTCTGGAAATTCCATTCTCGTCGCAATTAGTATCT---------TCCCTTGAAG---AAAAAAGAATACCAAAATCTCAGAATTTACGATCTATTCATTCAATATTTCCCTTTTTAGAGGATAAATTATCGCATTTAAATTATGTGTCAGATCTACTAATACCCCATCCCATCCATCTGGAAATCTTGGTTCAAATCCTTCAATGTTGGATCAAAGATGTTCCTTCTTTGCATTTATTGCGATTGTTTTTCCACGAATATCAT---------------AATTTGAATAGTCTC---------TTTACTT------CAAAGAAATC---------------CATTTACGTATTTTCAAAAAG---AAAGAAAAGATTCTTTTGGTTCCTACATAATTCTTATGTATATGAATGCGAATATCTATTCCTGTTTCTTCGTAAACAGTCTTCTTATTTACGATCAATATCTTCTGGAGTCTTTCTTGAGCGAACACATTTCTATGGAAAAATAGAA------TATCTTATAGT---CGTGTGTTGTAATTCTTTTCAGAGG-ATCCTATGGTTCCTCAAGGATACTTTCATACATTATGTTCGATATCAAGGAAAAGCAATTCTGGCTTCAAAAGGAACTCTTATTCTGATGAAAAAATGGGAATTTCATCTTGTGAATTTTTGGCAATCTTATTTTCACTTTTGGTTTCAACCTTATAGGATCCATATAAAGCAATTACCCAACTATTCCTTCTCTTTTCT-GGGGTTCTTTTCAAGTGTAC-TGAAAAATCCTTTGGTAGTAAGAAATCAAATGCTAGAGAATTCATTTCTAATAAATACTCTATCTAAGAAATTAGATACCATAGCCCCAGTTATTTCTCTTATTGGATCATTGTCGAAAGATCGATTTTGTACTGTATTGGGTCATCCTATTAGTAAACCGATCTGGACCGATTTATCGGATTCTGATATTCTTGATCGATTTTGTCGGATATGTAGAAATCTTTGTCGTTATCACAGCGGATCCTCAAAGAAACAGGTTTTGTATCGTATAAAGTATATACTTCGACTTTCGTGTGCTAGAACTTTGGCTCGTAAACATAAAAGTAC-AGTACGCACTTTTATGCGAAGGTTAGGTTCGGGATTCTTAGAAGAATTCTTTTTGAAG-------------------GAGCTATCCCGACCATTTCCCGTGCATCATCCTAGCAGAGTACTTA-TATCTATGTCAATGAAAAAAA-CTAAAAAATAAAATCTTAACAA-------------------ATTGGACCTAA--CCCCT-GAATTT----------CTTAG-------ATC-TTCCAAAA----------GAAGAC---TTTTTTT-----------GTAAA-TGTAAGGAAAAATATATGGACTAT-GAATGA------------TTCAATAAC-GGAGATTCCTTGAAC-------------------------ATATATGTTCAT-------------ATCGTA----CTAT------ACAAAA--------CAAAT-----GAGA--------TTGGATTGGAA------GAA-----GAT------------------ACGAGTATTTCT------ATTTGGATCCATT------TGTGAAAGAACAGAG--TGAATGAAATGATAAAG-------ATATTTCATTT--------------TGTTTAAACT------------GAGCCACTGATGGAAAGAGGAT-------------------------------GAGGATAAATA-AAGA------------------GCGAGGAA----------GTAAAAT-----GGGC-TTTTTATTGGGGATAGAGGG---TCTAT---------AAATGGATAATACT--------TTTGTATGTATATGAATTTTTGAAGGTAG--CAAT---------CCCCCAATATCT-TGT---------------TCTTAGAACAAGATATT--GGGGGATTCTTTTG---CTTCTCTATCCG---------------AATTTTCG---------TTCTTTATCAT----AAAAGTTCTC-CCCCGCC---------AATGAATGATAAGT-GCC-----TAGGTG-AAGTAT----------------AGTATAAGATAAGTCAGAAAA---------------------------------------ATACCTATACTCTTACTATAAG---CTATAA-GAT------------AAAGACTCTTAAGGATAAGGC-----TTTTCAC----------ATGAATACTTAGTAGAACGACTAACGACGAGATTTATTATCGTTTCTCGCGTGTCTCACGAAAGTTAGAGTAG-GTGCGAATTCTCCCAATTTGTGACCGACCATACGATCTGTGATATAAATGGTAAA-TGTTCCTTTCCATTATGAATAGC-GATTGTATGGCCAATCATTGTGGGTA-TAATGGTAGATGCCCGAGACCAAGTCACTATGATTTCTTTCTCCTCCCTCCTGTT-GAGTTTTTCAATTCTTCCCGCTAAATGATTAGCTACAAAAGGATTTTTTTTT-AGTGAACGTGTCAC-GGCTGATTACTCCTTTTTTTCCATTTTT-TAAATTGGCATTCTATGTCCAA----------TATCTCGATCTTAA-TCTG-AAG-TAT---------------------------AATG-ATGAATGG-AAAAAAGAGAAAATCC----------TTTA-TCTAG-ATAA--------GG--AA-GGGGC-GGATGTAA-CCAAGTGG-ATCAA-GG----

Dendrobium_anosmum ACGACT-CTCGGCAATGGATATCTCGGCTCTCGCATCGATGAAGAGCGCAGCGAAATGCGATATATGGTGCGAATTGCAGAATCCCGCGAACCATCGAGTCTTTGAACGCAAGTTGCGCCCGAGGCCAAT-CGGCCAAGGGCACGTCCGCCTGGGCGTCA-GGCATTTTGTCGCTTCACAAGAATTATTTTTCTTCTCATTTTTCT------------TCTCAAATGGTATCAGAAGGTTTTGGAGTCATTCTGGAAATTCCATTCTCGTCGCAATTAGTATCT---------TCCCTTGAAG---AAAAAAGAATACCAAAATCTCAGAATTTACGATCTATTCATTCAATATTTCCCTTTTTAGAGGATAAATTATCGCATTTAAATTATGTGTCAGATCTACTAATACCCCATCCCATCCATCTGGAAATCTTGGTTCAAATCCTTCAATGTTGGATCAAAGATGTTCCTTCTTTGCATTTATTGCGATTGTTTTTCCACGAATATCAT---------------AATTTGAATAGTCTC---------TTTACTT------CAAAGAAATC---------------CATTTACGTATTTTCAAAAAG---AAAGAAAAGATTCTTTTGTTTCCTACATAATTCTTATGTATATGAATGCGAATATCTATTCCTGTTTCTTCGTAAACAGTCTTCTTATTTACGATCAATATCTTCTGGAGTCTTTCTTGAGCGAACACATTTCTATGGAAAAATAGAA------TATCTTATAGT---CGTGTGTTGTAATTCTTTTCAGAGG-ATCCTATGGTTCCTCAAGGATACTTTCATACATTATGTTCGATATCAAGGAAAAGCAATTCTGGCTTCAAAAGGAACTCTTATTCTGATGAAAAAATGGGAATTTCATCTTGTGAATTTTTGGCAATCTTATTTTCACTTTTGGTTTCAACCTTATAGGATCCATATAAAGCAATTACCCAACTATTCCTTCTCTTTTCT-GGGGTTCTTTTCAAGTGTAC-TGAAAAATCCTTTGGTAGTAAGAAATCAAATGCTAGAGAATTCATTTCTAATAAATACTCTATCTAAGAAATTAGATACCATAGCCCCAGTTATTTCTCTTATTGGATCATTGTCGAAAGCTCAATTTTGTACTGTATTGGGTCATCCTATTAGTAAACCGATGTGGACTGATTTATCGGATTCTGATATTCTTGATCGATTTTGTCGGATATGCAGAAATCTTTGTCGTTATCACAGCGGATCCTCAAAGAA----------------------------------------------------------------------------------------------------------------------------------------------------------------------------------------------------------------------------------------------------------------------------------------------------------------------------------------------------------------------------------------------------------------------------------------------------------------------------------------------------------------------------------------------------------------------------------------------------------------------------------------------------------------------------------------------------------------------------------------------------------------------------------------------------------------------------------------------------------------------------------------------------------------------------------------------------------------------------------------------------------------------------------------------------------------------------------------------------------------------------------------------------------------------------------------------------------------------------------------------------------------------------------------------------------------------------------------------------------------------------------------------------------------------------------------------------------------------------------------------------------------------------------------------------------------------------------------------------------------------------------------------------------------------------------------------------------------------------------------------------------------------------------------------------------------------------------------------------------------------------------------

Dendrobium_cf_mimicum_PK12237E ACGACT-CTCGGCAATGGATATCTCGGCTCTTGCATCGATGAAGAGCGCAGCGAAATGCGATACGTGGTGCGAATTGCAGAATCCCGCGAACCATCGAGTCTTTGAACGCAAGTTGCGCCCGAGGCCAAC-CGGCCAAGGGCACGTCCGCCTGGGCGTCA-AGCGTTGCGTCGCTCCACAAGAATTCTTTTTCTTCTCATTTTTCT------------TCTCAAATGGTATCAGAAGGTTTTGGAGTCATTCTGGAAATTCCATTCTCTTCGCGATTAGTATCT---------TCCCTTGAAG---AAAAAAGAATACCAAAATTTCAGAATTTACGATCTATTCATTCAATATTTCCCTTTTTAGAGGATAAATTATCGCATTTAAATTATGTGTCAGATCTACTAATACCCCATCCCATCCATCTGGAAATCTTGGTTCAAATACTTCAATGTTGGATCAAAGATGTTCCTTCTTTACATTTCTTGCGATTGTATTTCCACGAATATCAT---------------AATTTGAATAGTCTC---------ATTACTT------CAAAGAAATC---------------CATTTACGTCTTTTCAAAAAG---AAAGAAAAGATTCTTTTGGTTCCTACATAATTCTTATGTATATGAATGCGAATATATATTCCTGTTTATTCGTAAACAGTCTTCTTATTTACGATCAATATCTTCTGGAGTCTTTCTTGAGCGAACACATTTCTATGGAAAAATAGAA------TATCTTATAGT---CGTGTGTTGTAATTCTTTTCAGAGG-ATCCTATGGTTCCTCAAAGATACTTTCATACATTATGTTCGATATCAAGGAAAAGCGATTCTGGCTTCAAAAGGAACTCTTATTCTGATGAAGAAATGGAAATTTCATCTTGTGAATTTTTGGCAATCTTATTTTCACTTTTGGTTTCAACCTTATAGGATCCATATAAAGCAATTACCCAACTATTCCTTCTCTTTTCT-GGGTTTTTTTTCAAGTGTAC-TGAAAAATCCTTTGGTAGTAAGAAATCAAATGCTAGAGAATTCATTTCTAATAAATACTCTGACTAAGAAATTAGATACCATAGCCCCAGTTATTTCTCTTATTGGATCATTGTCAAAAGCTCAATTTTGTACTGTATTGGGTCATCCTATTAGTAAACCGATCTGGACCGATTTATCGGATTCTGATATTCTTGATCGATTTTGTCGGATATGTAGAAATCTTTGTCGTTATCACAGCGGATCCTCAAAGAAACAGGTTTTGTATCGTATAAAGTATATACTTCGACTTTCGTGTGCTAGAACTTTGGCTCGTAAACATAAAAGTAC-AGTACGCACTTTTATGCGAAGGTTAGGTTCGGGATTCTTAGAAGAATTTTTTGTTGAAGAAGAACAATCTTTTTCTTGAGCTATCCCGACCMTTTCCCGTGCATCATCCTAGCAGAGTACTTA-TATCTATGTCAATGAAAAAAAACTAAAAAATAAAATGTTAACAA-------------------ATTGGACCTAG--CCCCT-GAATTT----------TTTAG-------ATC-TTCAAAAAGAAAAAA---GAAGAC---TTTTTTTGTATTTTTT--GTAAA-TGTAAGGAAAAATATAT--------GAATGA------------TTCAATAAC-GGAGATTCCTTGAAC-------------------------ATATATGTTCAT-------------ATCGTA----CTAT------ACAAAA--------CAAAT-----GAGAT-------TTGTATTGGAA------GAA-----GAT------------------ACGAGGATTTCT------ATTCGGATCCATT------TGTGAAAGAAAAGT--------------------------------------------------------------------------GAGCCACTGATGGAAAGAAGA--------------------------------GGGGATAAATA-AAG--------------------------------------------------------------------------CCATCTAT---------AAATGGATAATACT--------TTTGTG----TATGAATTTATTAAGGTAG--CAAT---------CCCCCAATATCT-TGT---------------TCTTAGAACAAGATATT--GGGGGATTCTTTTG---CTTCTCTATCCG---------------AATTTTCG---------TTCTTTATCAT----AAAAGTTCTC-CCCCGCC---------AATGAATGATAAGT-GCC-----TAGGTG-AAGTAT----------------AGTATAAGATAAGTCAGA----------------------AAAGTG--------AGTATAATACCTATACTCTTA----------CTATAA-GAT------------AAAGACTCTTAAGGATAAGGC-----TTTTCAC----------ATGAATACTTAGTAGAACGACTAACGACGAGATTTATTATCGTTTCTCGCGTGTCTCACGAAAGTTAGAGTAG-GTGCGAATTCTCCCAATTTGTGACCGACCATACGATCTGTGATATAAATGGTAAA-TGTTCCTTTCCATTATGAATAGC-GATTGTATGGCCAATCATTGTGGGTA-TAATGGTAGATGCCCGAGACCAAGTCACTATGATTTCTTTCTCCTCCCTCCTGTT-GAGTTTTTCAATTCTTCCCGATAAATGATTAGCTACAAAAGGATTTTTTTTT-AGTGAACGTGTCAC-GGCTGATTACTCCTTTTTTTACATTTTT-GAAATTGGCATTCTATGTCCAA----------TATCTCGATCTTAA-TCTG-AAG-TAT---------------------------AATG-ATGAATGG-AAAAAAGAGAAAATCC----------TTTA-GCTAG-ATAA--------GGG-AA-GGGGC-GGATGTAG-CCAAGTGG-CTCAA-GGCAGT

Dendrobium_crumenatum AGGACT-CTCGACAATGGATATCTCGGCTCTTGCATCGATGAAGAGCGCAGCGAAATGCGATACGTGGTGCGAATTGCAGAATCCCGCGAACCATCGAGTCTTTGAACGCAAGTTGCGCCCGAGGCCAAT-CGGCCAAGGGCACGTCTGCCTGGGCG----AGCAT---GTCACTCC------------------------------------------------------------------------------------------------------------------------------------------------------------------------------------------------------------------------------------------------------------------------------------------------------------------------------TTTTCCACGAATATCAT---------------AATTTGAATAGTCTC---------TTTACTT------CAAAGAAATC---------------CATTTACGTATTTTCAAAAAG---AAAGAAAAGATTCTTTTGGTTCCTACATAATTCTTATGTATATGAATGCGAATATCTATTCCTGTTTCTTCTTAAACAGTCTTCTTATTTACGATCAATATCTTCTGGAGTCTTTCTTGAGCGAACACATTTCTATGGAAAAATAGAA------TATCTTATAAT---CGTGTGTTGTAATTCTTTTCAGAGG-ATCTTATGGTTCCTCAAGGATACTTTCATACATTATGTTCGATATCAAGGAAAAGCAATTCTGGCTTCAAAAGGAACTCTTATTCTGATGAAAAAATGGAAATTTCATCTTGTGAATTTTTGGCAATCTTATTTTCACTTTTGGTTTCAACCTCATAGGATCCATATAAAGCAATTACCCAACTATTCCTTCTCCTTTCT-GGGGTTATTTTCAAGTGTAC-TGAAAAATCCTTTGGTAGTAAGAAATCAAATGCTAGAGAATTCATTTCTAATAAATACTCTATCTAATAAATTAGATACCATAGCCCCAGTTATTTCTCTTATTGGATCATTGTCGAAAGCTCAATTTTGTACTGTATTGGGTCATCCTATTAGTAAACCGATCTGGACCGATTTATCGGATTCTGATATTCTTGATCGCTTTTGTCGGATATGTAGAAATCTTTGTCGTTATC-----------------------------------------------------------------------------------------------------------------------------------------------------------------------------------------------------------------------------------------------------------------------------------------------------------------------------------------------------------------------------------------------------------------------------------------------------------------------------------------------------------------------------------------------------------------------------------------------------------------------------------------------------------------------------------------------------------------------------------------------------------------------------------------------------------------------------------------------------------------------------------------------------------------------------------------------------------------------------------------------------------------------------------------------------------------------------------------------------------------------------------------------------------------------------------------------------------------------------------------------------------------------------------------------------------------------------------------------------------------------------------------------------------------------------------------------------------------------------------------------------------------------------------------------------------------------------------------------------------------------------------------------------------------------------------------------------------------------------------------------------------------------------------------------------------------------------------------------------------------------------------------------------------

Dendrobium_linawianum_SG1347 ACGACT-CTCGGCAATGGATATCTTGGCTCTCGCATCGATGAAGAGCGCAGCGAAATGCGATATGTGGTGCGAATTGCAGAATCCCGCGAACCATCGAGTCTTTGAACGCAAGTTGCGCCTGAGGCCAAC-CGGCTGAGGGCACGTCCGCCTGGGCGTCA-AGCATTTTATCGCTCCACAAGAATTCTTTTTCTTCTCATTTTTCT------------TCTCAAATGGTATCAGAAGGTTTTGGAGTCATTCTGGAAATTCCATTCTCGTCGCAATTAGTATCT---------TCCCTTGAAG---AAAAAAGAATACCAAAATCTCAGAATTTACGATCTATTCATTCAATATTTCCCTTTTTAGAGGATAAATTATCGCATTTAAATTATGTGTCAGATCTACTAATACCCCATCCCATCCATCTGGAAATCTTGGTTCAAATCCTTCAATGTTGGATCAAAGATGTTCCTTCTTTGCATTTATTGCGATTGTTTTTCCACGAATATCAT---------------AATTTGAATAGTCTC---------TTTACTT------CAAAGAAATC---------------CATTTACGTATTTTCAAAAAG---AAAGAAAAGATTCTTTTGGTTCCTACATAATTCTTATGTATATGAATGCGAATATCTATTCCTGTTTCTTCGTAAACAGTCTTCTTATTTACGATCAATATCTTCTGGAGTCTTTCTTGAGCGAACACATTTCTATGGAAAAATAGAA------TATCTTATAGT---CGTGTGTTGTAATTCTTTTCAGAGG-ATCCTATGGTTCCTCAAGGATACTTTCATACATTATGTTCGATATCAAGGAAAAGCAATTCTGGCTTCAAAAGGAACTCTTATTCTGATGAAAAAATGGGAATTTCATCTTGTGAATTTTTGGCAATCTTATTTTCACTTTTGGTTTCAACCTTATAGGATCCATATAAAGCAATTAACCAACTATTCCTTCTCTTTTCT-GGGGTTCTTTTCAAGTGTAC-TGAAAAATCCTTTGGTAGTAAGAAATCAAATGCTAGAGAATTCATTTCTAATAAATACTCTATCTAAGAAATTAGATACCATAGCCCCAGTTATTTCTCTTATTGGATCATTGTCGAAAGATCGATTTTGTACTGTATTGGGTCATCCTATTAGTAAACCGATCTGGACCGATTTATCGGATTCTGATATTCTTGATCGATTTTGTCGGATATGTAGAAATCTTTGTCGTTATCACAGCGGATCCTCAAAGAAACAGGTTTTGTATCGTATAAAGTATATACTTCGACTTTCGTGTGCTAGAACTTTGGCTCGTAAACATAAAAGTAC-AGTACGCACTTTTATGCGAAGGTTAGGTTCGGGATTCTTAGAAGAATTCTTTTTTGAAGA--------------------------------------------------------------------------------------------------------------------------------------------------------------------------------------------------------------------------------------------------------------------------------------------------------------------------------------------------------------------------------------------------------------------------------------------------------------------------------------------------------------------------------------------------------------------------------------------------------------------------------------------------------------------------------------------------------------------------------------------CCATCTAT---------AAATGGATAATACT--------TTTGTATGT-TATGAATTTTTGAAGGTAG--CAAT---------CCCCCAATATCT-TGT---------------TCTAAGAACAAGATATT--GGGGGATTCTTTTR---CTTCTCTATCCG---------------AATTTTCG---------TTCTTTATCAT----AAAAGTTCTC-CCCCGCC---------AATGAATGATAAGT-GCC-----TAGGTG-AAGTAT----------------AGTATAAGATAAGTCAGAAAA---------------------------------------ATACCTATACTCTTA----------CTATAA-GAT------------AAAGACTCTTAAGGATAAGGC-----TTTTCAC----------ATGAATACTTAGTAGAACGACTAACGACGAGATTTATTATCGTTTCTCGCGTGTCTCACGAAAGTTAGAGTAG-GTGCGAATTCTCCCAATTTGTGACCGACCATACGATCTGTGATATAAATGGTAAA-TGTTCCTTTCCATTATGAATAGC-GATTGTATGGCCAATCATTGTGGGTA-TAATGGYAGATGCCCGAGACCAAGTCACTATGATTTCTTTCTCCTCCCTCCTGTT-GARTTTTTCAATTCTTCCCGCTAAATGATTAGCTACAAAAGGATTTTTTTTTTAGTGAACGTGTCAC-GGCTGATTACTCCTTTTTTTCCATTTTT-TAAATTGGCATTCTATGTCCAA----------TATCTCGATCTTAA-TCTG-AAG-TAT---------------------------AATG-ATGAATGG-AAAAAAGAGAAAATCC----------TTTA-GCTAG-ATAA--------GGG-AA-GGGGC-GGATGTAG-CCNAGTGK-ATCAA-GGCAGT

Dendrobium_lindleyi_KFBG203 ACGACT-CTCGGCAATGGATATCTCGGCTCTCGCATCGATGAAGAGCGCAGCGAAATGCGATACGTGGTGCGAATTGCAGAATCCCGCGAACCATCGAGTCTTTGAACGCAAGTTGCGCCCGAGGCCAAT-CGGCCAAGGGCACGTTCGCCTGGGCGTCA-AGCATTATGACGCTCCACAAGAATTCTTTTTCTTCTCATTTTTCT------------TCTCAAATGGTATCGGAAGGTTTTGGAGTCATTCTGGAAATTCCATTCTCGTCGCAATTAGTATCT---------TCCCTTGAAG---AAAAAAGAATACCAAAATATCAGAATTTACGATCTATTCATTCAATATTTCCCTTCTTAGAGGATAAATTATCGCATTTAAATTATGTGTCAGATCTACTAATACCCCATCCCATCCATCTGGAGATCTTGGTTCAAATCCTTCAATGTTGGATCAAAGATGTTCCTTCTTTGCATTTATTGCGATTGTTTTTCCACGAATATCAT---------------AATTTGAATAGTCTC---------TTTACTT------CAAAGAAATC---------------CATTTACATATTTTCAAAAAG---AAAGAAAAGATTCTTTTGGTTCCTACATAATTCTTATGTATATGAATGCGAATATCTATTCCTGTTTCTTCGTAAAAAGTCTTCTTATTTACGATCAATATCTTCTGGAGTCTTTCTTGAGCGAACACATTTCTATGGAAAAATAGAA------TATCTTATAGT---CGTGTGTTGTAATTCTTTTCAGAGG-ATCCTATGGTTCCTCAAGGATACTTTCATACATTATGTTCGATATCAAGGAAAAGCAATTCTGGCTTCAAAAGGAACTCTTATTCTGATGAAAAAATGGAAATTTCATCTTGTGAATTTTTGGCAATCTTATTTTCACTTTTGGTTTCAACCTTATAGGATCCATATAAAGCAATTACCCAACTATTCCTTCTCTTTTCT-GGGGTTCTTTTCAAGTGTAC-TGAAAAATCCTTTGGTAGTAAGAAATCAAATGCTAGAGAATTCATTTCTAATAAATACTCTATCTAAGAAATTAGATACCATAGTCCCAGTTATTTCTCTTATTGGATCATTGTCGAAAGCTCAATTTTGTACTGTATTGGGTCATCCTATTAGTAAACCGATCTGGACCGATTTATCGGATTCTGAGATTCTTGATCGATTTTGTCGGATATGTAGAAATCTTTGTCGTTATCACAGCGGATCCTCAAAGAAACAGGTTTTGTATCGTATAAAGTATATACTTCGACTTTCGTGTGCTAGAACTTTGGCTCGTAAACATAAAAGTAC-AGTACGCACTTTTATGCGAAGGTTAGGTTCGGGATTCTTAGAAGAATTCTTTTTTGAAGA-----------------GAGCTATCCCGACCATTTCCCGTGCATCATCCTAGCAGAGTACTTA-TATCTATGTCAATGAAAGAAA-CTAAAAAATAAAATCTTAACAA-------------------ATTGGACCTAG--CCCCT-TAATTT----------CTTAG-------ATC-TTCCAAAA----------GAAGAC-----TCTTTTT---------GTAAA-TGTAAGGAAAAATATATGGACTAT-GAATGA------------TTCAATAAC-GGAGATTCCTTGAAC-------------------------ATATATGTTCAT-------------ATCGTA----CTAT------ACAAAA--------CAAAT-----GAGA--------TTGGATTGGAA------GAA-----GAT------------------ACGAGTATTTCT------ATTCGAATCCATT------TGTGAAAGAACAGAG--TGAGTGAAATGAGAAAG-------ATATTTCATTT--------------TGTTTAAACTGAGCCACTGACTGAGCCACTGATGGAAAGAGGAT-------------------------------GAGGATAAATA-AAGATAC----------------------------------------------------TGTA--GGGAAAGT----CCATCTAT---------AAATGGATAATACT--------TTTGTATGT-TATGAATTTTTTAAGATAG--CAAT---------CCCCCAATATCT-TGT---------------TCTAAGAACAAGATATT--GGGGGATTCTCTTG---CTTCTCTATCCG---------------AATTTTCG---------TTCTTTATCAT----AAAAGTTCTC-CCCCGCC---------AATGAATGATAAGT-GCC-----TAGGTG-AAGTAT----------------AGTATAAGATAAGTCAGAAAA---------------------------------------ATACCTATACTCTTA----------CTATAA-GAT------------AAAGACTCTTAAGGATAAGGC-----TTTTCAC----------ATGAATACTTAGTAGAACGACTAACGACGAGATTTATTATCGTTTCTCGCGTGTCTCACGAAAGTTAGAGTAG-GTGCGAATTCTCCCAATTTGTGACCGACCATACGATCTGTGATATAAATGGTAAA-TGTTCCTTTCCATTATGAATAGC-GATTGTATGGCCAATCATTGTGGGTA-TAATGGTAGATGCCCGAGACCAAGTCACTATGATTTCTTTCTCCTCCCTCCTGTT-GAGTTTTTCAATTCTTCCCGCTAAATGATTAGCTACAAAAGGATTTTTTTTT-AGTGAACGTGTCAC-GGCTGATTACTCCTTATTTTCCCTTTTT-TAAATTGGCATTCTATGTCCAA----------TATCTCGATCTTAA-TCTG-AAG-TAT---------------------------AATG-ATGAATGG-AAAAAAGAGAAAATCC----------TTTA-GCTAG-ATAA--------GGG-AA-GGGGC-GGATGTAG-CCAAGTGG-ATCAA--GCAGT

Dendrobium_loddigesii_SG1255 ATGACT-CTCGGCAATGGATATCTCGGCTCTCGCATCGATGAAGAGCGCAGCGAAATGCGATATGTGGTGCGAATTGCAGAATCCCGCGAACCATCGAGTCTTTGAACGCAAGTTGCGCCCGAGGCCAAC-CGGCCAAGGGCACGTCCGCCTGGGCGTCA-GTCATTTTATCGCTCTACAAGAATTCTTTTTCTTCTCATTTTTCT------------TCTCAAATGGTATCAGAAGGTTTTGGAGTCATTCTGGAAATTCCATTCTCGTCGCAATTAGTATCT---------TCCCTTGAAG---AAAAAAGAATACCAAAATCTCAGAATTTACGATCTATTCATTCAATATTTCCCTTTTTAGAGGATAAATTATCGCATTTAAATTATGTGTCAGATCTACTAATACCCCATCCCATCCATCTGGAAATCTTGGTTCAAATCCTTCAATGTTGGATCAAAGATGTTCCTTCTTTGCATTTATTGCGATTGTTTTTCCACGAATATCAT---------------AATTTGAATAGTCTC---------TTTACTT------CAAAGAAATC---------------CATTTACGTATTTTCAAAAAG---AAAGAAAAGATTCTTTTGTTTCCTACATAATTCTTATGTATATGAATGCGAATATCTATTCCTGTTTCTTCGTAAACAGTCTTCTTATTTACGATCAATATCTTCTGGAGTCTTTCTTGAGCGAACAAATTTCTATGGAAAAATAGAA------TATCTTATAGT---CGTGTGTTGTAATTCTTTTCAGAGG-ATCCTATGGTTCCTCAAGGATACTTTCATACATTATGTTCGATATCAAGGAAAAGCAATTCTGGCTTCAAAAGGAACTCTTATTCTGATGAAAAAATGGGAATTTCATCTTGTGAATTTTTGGCAATCTTATTTTCACTTTTGGTTTCAACCTTATAGGATCCATATAAAGCAATTACCCAACTATTCCTTCTCTTTTCT-GGGGTTCTTTTCAAGTGTAC-TGAAAAATCCTTTGGTAGTAAGAAATCAAATGCTAGAGAATTCATTTCTAATAAATACTCTATCTAAGAAATTAGATACCATAGCCCCAGTTATTTCTCTTATTGGATCATTGTCGAAAGCTCAATTTTGTACTGTATTGGGTCATCCTATTAGTAAACCGATGTGGACCGATTTATCGGATTCTGATATTCTTGATCGATTTTGTCGGATATGCAGAAATCTTTGTCGTTATCACAGCGGATCCTCAAAGAAACAGGTTTTGTATCGTATAAAGTATATACTTCGACTTTCGTGTGCTAGAACTTTGGCTCGTAAACATAAAAGTAC-AGTACGCACTTTTATGCGAAGGTTAGGTTCGGGATTCTTAGAAGAATTCTTTTTTGAAGAAGAACAATCTCTTTCTTGAGCTATCCCGACCATTTCCCGTGCATCATCCTAGCAGAGTACTTA-TATCTATGTCAATGAAAAAAA-CTAAAAAATAAAATCTTAACAA-------------------ATTGGACCTAA--CCCCT-GAATTT----------CTTAG-------ATC-TTCCAAAA----------GAAGAC---TTTTTTT--------------------------------GTAGACTAT-GAATGA------------TTCAATAAC-GGAGATTCCTTGAAC-------------------------ATATATGTTCAT-------------ATCGTA----CTAT------ACAAAA--------CAAAT-----GAGA--------TTGGATTGGAA------GAA-----GAT------------------ACGAGTCTTTCT------ATTCGGATCCATT------TGTGAAAGAACAGAG--TGAATGAAATGAGAAAG-------ATATTTCATTT--------------TGTTTAAACT------------GAGCCACTGATGGAAAGAGGAT-------------------------------GAGGATAAATA-AAGA------------------GCGAAGAA----------GTAAAAT-----GGGC-TTTTTATTGGGGATAGAGGGCCATCTAT---------AAATGGATAATACT--------TTTGTATGT-TATGAATTTTTTAAGATAG--CAAT---------CCCCCAATATCT-TGT---------------TCTTAGAACAAGATATT--GGGGGATTCTTTTG---CTTCTCTATCCG---------------AATTTTCG---------TTCTTTATCAT----AAAAGTTCTC-CCCCGCC---------AATGAATGATAAGT-GCC-----TAGGTG-AAGTAT----------------AGTATAAGATAAGTCAGAAAA---------------------------------------ATACCTATACTCTTA----------CTATAA-GAT------------AAAGACTCTTAAGGATAAGGC-----TTTTCAC----------ATGAATACTTAGTAGAACGACTAACGACGAGATTTATTATCGTTTCTCGCGTGTCTCACGAAAGTTAGAGTAG-GTGCGAATTCTCCCAATTTGTGACCGACCATACGATCTGTGATATAAATGGTAAA-TGTTCCTTTCCATTATGAATAGC-GATTGTATGGCCAATCATTGTGGGTA-TAATGGTAGATGCCCGAGACCAAGTCACTATGATTTCTTTCTCCTCCCTCCTGTT-GAGTTTTTCAATTCTTCCCGCTAAATGATTAGCTACAAAAGGATTTTTTTTT-AGTGAACGTGTCAC-GGCTGATTACTCCTTTTTTTCCATTTTT-TAAATTGGCATTCTATGTCCAA----------TATCTCGATCTTAA-TCTG-AAG-TAT---------------------------AATG-ATGAATGG-AAAAAAGAGAAAACCC----------TTTA-GCTAG-ATAA--------GGG-AA-GGGGC-GGATGTAG-CCAAGTGG-ATCAA-GGCAGT

Dendrobium_spatella_SG1357 ATGACT-CTCGACAATGGATATCTAGGCTCTTGCATCGATGAAGAGCGCAGCGAAATGCGATACGTGGTGCGAATTGCAGAATCCCGCGAACCATCGAGTCTTTGAACGCAAGTTGCGCCCGAGGCCAAC-CGGCCAAGGGCACGTCCGCCTGGGCGTCA-AGCATTACGTCACTCCACAAGAATTCTTTTTCTTCTCATTTTTCT------------TCTCAAATGGTATCAGAAGGTTTTGGAGTCATTCTGGAAATTCCATTCTCGTCGCAATTAGTATCT---------TCCCTTGAAG---AAAAAAGAATACCAAGATCTCAGAATTTACGATCTATTCATTCAATATTTCCCTTTTTAGAGGATAAATTATCGCATTTAAATTATGTGTCAGATCTACTAATACCCCATCCCATCCATCTGGAGATCTTGGTTCAAATCCTTCAATGTTGGATCAAAGATGTTCCTTCTTTGCATTTATTGCGATTGTTTTTCCACGAATATCAT---------------AATTTGAATAGTCTC---------TTTACTT------CAAAGAAATC---------------CATTTACGTATTTTCAAAAAG---AAAGAAAAGATTCTTTTGGTTCCTACATAATTCTTATGTATATGAATGCGAATATATATTCCTGTTTCTTCGTAAACAGTCTTCTTATTTACGATCAATATCTTCTGGAGTCTTTCTTGAGCGAACACATTTCTATGGAAAAATAGAA------TATCTTATAGT---CGTGTGTTGTAATTCTTTTCAGAGG-ATCTTATGGTTCCTCAAGGATACTTTCATACATTATGTTCGATATCAAGGAAAAGCAATTCTGGCTTCAAAAGGAACTCTTATTCTGATGAAAAAATGGAAATTTCATCTTGTGAATTTTTGGCAATCTTATTTTCACTTTTGGTTTCAACCTCATAGGATCCATATAAAGCAATTACCCAACTATTCCTTCTCTTTTCT-GGGGTTATTTTCAAGTGTAC-TGAAAAATCCTTTGGTAGTAAGAAATCAAATGCTAGAGAATTCATTTCTAATAAATACTCTATCTAATAAATTAGATACCATAGCCCCAGTTATTTCTCTTATTGGATCATTGTCGAAAGCTCAATTTTGTACTGTATTGGGTCATCCTATTAGTAAACCGATCTGGACCGATTTATCGGATTCTGATATTCTTGATCGATTTTGTCGGATATGTAGAAATCTTTGTCGTTATCACAGCGGATCCTCAAAGAAACAGGTTTTGTATCGTATAAAGTATATACTTCGACTTTCGTGTGCTAGAACTTTGGCTCGTAAACATAAAAGTAC-AGTACGCACTTTTATGCGAAGGTTAGGTTCGGGATTCTTAGAAGAATTCTTTTTTGAAGA-----------------GAGCTATCCCGACCATTTTCCGTGCATCATCCTAGCAGAGTACTTA-TATCTATGTCAATGAAAAAAA---------------------------------------------------------------------------------------------------------------------------------------------------------------------------------------------------------------------------------------------ATCTATGTCAAT---------------------------------------------------------GAAA-----------------------AAAAA--------------------------------------------------------------------AAGAA---------------------------------------------------------------------------------------------ATAGGAT-------------------------------GAGGATAAATA-AAGA------------------GCGAGGAA----------GTAAAAT-----GGGC-TTTTTATTGGGGATAGAGGGCCATCTAT---------AAATGGATAATAAT--------TTTGTATGT-TATGAATCTTTTAAGGTAG--CAAT---------CCCCCAATATCT-TGT---------------TCTAAGAACAAGATATT--GGGGGATTTTTTTG---CTTCTCTATCCG---------------AATTTTCG---------TTCTTTATCAT----AAAATTTCTC-CCCCGCC---------AATGAATGATAAGT-GCC-----TAGGTG-AAGTAT----------------AGTATAAGATAAGTCAGAAAG---------------------------------------ATACCTATACTCTTA----------CTATAA-GAT------------AAAGACTCTTAAGGATAAGGCTTTTTTTTTCAC----------ATGAATACTTAGTAGAACGACTAACGACGAGATTTATTATCGTTTCTCGCGTGTCTCACGAAAGTGAGAGTAG-GTGCGAATTCTCCCAATTTGTGACCGACCATACGATCTGTGATATAAATGGTAAA-TGTTCCTTTCCATTATGAATAGC-GATTGTATGGCCAATCATTGTGGGTA-TAATGGTAGATGCCCGAGACCAAGTCACTATGATTTCTTTCTCCTCCCTCCTGTT-GAGTTTTTCAATCCTTCCCGCTAAATGATTAGCTACAAAAGGATTCTTTTTT-AGTGAACGTGTCAC-GGCTGATTACTCCTTTTTTTCCATTTTT-TAAATTGGCATTCTATGTCCAA----------TATCTCGATATTAA-TCTG-AAG-TAT---------------------------AATG-ATGAATGG-AAAAAAGAGAAAATCC----------TTTA-GCTAG-ATAA--------GG--AA-GGGGC-G-----------------------------

Dendrolirium_lasiopetalum_SG1312 ACGACT-CTCGGCAATGGATATCTCGGCTCTCGCATCGATGAAGAGCGCAGCGAAATGCGATACGTGGTGTGAATTGCAGAATCCCGCGAACCATCGAGTCTTTGAACGCAAGTTGCGCCCGAGGCCAAC-CGGTTGAGGGCACGTCTGCCTGGGCGTCA-AGCGTTACGTCGCTCC------------------------TTTTT------------TCTCAAATGGTATCAGAAGGTTTTGGAGTTATTCTGGAAATTCCATTCTCGTCGCGATTAGTATCT---------TCCCTTGAAG---AAAAAAGAATACCAAAATCTCAGAATTTACGATCTATTCATTCAATATTTCCCTTTTTAGAGGATAAATTATCACATTTAAATTATGTGTCAGATCTACTAATACCCCATCCCATCCATATGGAAATCTTGGTTCAAATTCTTCAATGTTGGATCAAAGATGTTCCTTCTTTGCATTTCTTGCGATTGTTTTTCCACGAATATCAT---------------AATTTGAATAGTCTC---------ATTATTT------CAAAGAAATC---------------TATTTACGTCTTTTCAAAAAG---AAAGAAAAGATTCTTTTGGTTCCTACATAATTCTTATGTATATGAATGCGAATATCTATTCCTGTTTCTTCGTAAACAGTCTTCTTATTTACGATCAATATCTTCTGGAGTCTTTCTTGAGCGAACACATTTCTATGGAAAAATAGAA------TATCTGATAGCCGTCGTGTATTGTAATTCTTTTCAGAGG-ATCCTATGGTTCCTCAAAGATACTTTCATACATTATGTTCGATATCAAGGAAAAGCGATTCTGGCTTCAAAAGGAACTCTTATTCTGATGAAGAAATGGAAATTTCATCTTGTGAATTTTTGGCAATCTTATTTTCACTTTTGGTTTCAACCTTATAGGATCCATATAAAGCAATTACCCAACTATTCCTTCTCTTTTCT-GGGGTATTTTTCAAGTGTAC-TAAAAAATCCTTTGGTAGTAAGAAATCAAATGTTAGAGAATTCATTTCTAATAAATACTCTGACTAATAAATTAGATACCATAGTCCCAGTTATTTCTCTTATTGGATCATTGTCGAAAGCTCAATTTTGTACTGTATTGGGTCATCCTATTAGTAAACCGATCTGGACCGATTTATCGGATTCTGATATTCTTGATCGATTTTGTCGGATATGTAGAAATCTTTGTCGTTATCACAGCGGATCCTCAAAGAAACAGGTTTTGTATCGTATAAAGTATATACTTCGACTTTCGTGTGCTAGAACTTTGGCTCGTAAACATAAAAGTAC-AGTACGCACTTTTATGCGAAGATTAGGTTCGGGATTCTTAGAAGAATTCTTTTTGGAAG-ATTTTAAACGTTTACTCGAGCTATCCCGACCATTTCCCGTGCATCATCCTATCAGAGTACTTC-TATCTATGTCAATGAAAAGAA-CTAAAAAATAATATCTTAACAA-------------------ATCGGACCTAG--CCCCT-GAATTT----------CTTAG-------ATC-TTCAAAAAAAA-------AAAGAC---ATTCTTT-----------GTAAA-TGTAAGGAAAAAGATATGGACTAT-GAATGA------------TTCAATAAC-GGAGATTCTTTGAAC-------------------------ATATATGTTCAT-------------ATCGTA----CTAT------ACAAAA--------CAAAT-----TAGA--------TTGGATTGGAA------GAAGATACGAT------------------ACGAGGATTTCT------ATTCGGATCCTTT------TGTGAAAGAACAGAG--TGAATGAAATGAGAAAG-------ATATTTAATTT--------------TGTTTAAACT------------GAGACACTGATGAAGAGAGGAT-------------------------------GAGGATAAATA-AAGA------------------GCGAGGAA----------GTAAAAG-----GGGC-TTTTTATTGGGG--------CCATCTAT--------AAAATGGATAATACT--------TTTGTATTCAATGAATTAGATAAAGATAG--CAAT---------CCCCCAA-----------------------------------GATATT--GGGGGATTCTTTTG---CTTCTCTATCCG---------------AATTTTCG---------TTCTTTCTCAT----AAAAGTTCTC-CCCCGCC---------AATGAATGATAAGT-GCC-----TAGGTG-AAGTAT----------------AGTATAAGATAAGTCAGA----------------------AAAGTCTAAGTCTTA-----ATACCTATACTCTTACTCTTA----CTATAA-GAT------------AAAGACTCTTAA---TAAGGC-----TTTTCAC----------ATGAATACTTAGTAGAACGACTAACGACGAGATTTATTATCGTTTCTCGCGTGTCTCACGAAAGTGAGAGTAG-GTGCGAATTCTCCCAATTTGTGACCGACCATACGATCTGTGATATAAATGGTAAA-TGTTCCTTTCCATTATGAATAGC-GATTGTATGGCCAATCATTGTGGGTA-TAATGGTAGATGCCCGAGACCAAGTCACTATGATTTCTTTCTCCTCCCTCCTGTT-GAGTTTTTCAATTCTTCCCGATAAATGATTAGCTACAAAAGGATTTTTTTTT-AGTGAACGTGTCAC-GGCTGATTACTCCTTTTTTTACATTTTT-TAAATTGGCATTCTATGTCCAA---TATCTAATATCTCGATCTTAA-TCTG-AAG-TAG---------------------------AATG-ATGAATGG-AAAAAAGAGAAAATCC----------TTTA-GCTAG-ATAA--------GGG-AA-GGGGC-GGATGTAG-CCAAGTGG-ATCAA-GGCAGT

Didymoplexiella_siamensis_SG1242 ATGACT-CTCGGCAATGGATATCTCGGCTCTCGCATCGATGAAGAGCGCAGTGAAATGCGATACGTGGTGCGAATTGCAGAATCCCGCGAACCATCGAGTCTTTGAACGCAAGTTGCGCCCGAGGCCAAT-CGGCCGAGGGCACGCCCGCCTGGGCGACACAGCATTGCTTCGCTCC-----------------------------------------------------------------------------------------------------------------------------------------------------------------------------------------------------------------------------------------------------------------------------------------------------------------------------------------------------------------------------------------------------------------------------------------------------------------------------------------------------------------------------------------------------------------------------------------------------------------------------------------------------------------------------------------------------------------------------------------------------------------------------------------------------------------------------------------------------------------------------------------------------------------------------------------------------------------------------------------------------------------------------------------------------------------------------------------------------------------------------------------------------------------------------------------------------------------------------------------------------------------------------------------------------------------------------------------------------------------------------------------------------------------------------------------------------------------------------------------------------------------------------------------------------------------------------------------------------------------------------------------------------------------------------------------------------------------------------------------------------------------------------------------------------------------------------------------------------------------------------------------------------------------------------------------------------------------------------------------------------------------------------------------------------------------------------------------------------------------------------------------------------------------------------------------------------------------------------------------------------------------------------------------------------------------------------------------------------------------------------------------------------------------------------------------------------------------------------------------------------------------------------------------------------------------------------------------------------------------------------------------------------------------------------------------------------------------------------------------------------------------------------------------------------------------------------------------------------------------------------------------------------------------------------------------------------------------------------------------------------------------------------------------

Dienia_ophrydis_SG1276 ATGACT-CTCGGCAATGGATATCTCGGCTCTTGCATCGATGAAGAGCGCAGCAAAATGCGATACGTGATGCGAATTGCAGAATCCCGCGAACCATCGAGTATTTGAACGCAAGTTGCGCCCGAGGCCAAC-CGGTCAAGGGCACGTTTACCTGGGTGTCA-AGCGTTGCTTCGCTTC---------------------------------------------------------AAGGTTTTGGAGTCATTCTGGAAATCCCATTCTCGTCGCGATTAGTATTT---------TCCCTTGAAG---AAAAAACAATAACAAAATCTCAGAATTTACAATCTATTCATTCAATTTTTCCCTTTTTAGAGGATAAATTATCACATTTAAATTATGTGTCAGATCTAATAATACCCCATCCCATCCATCTGGAAATCTTGGTTCAAATTCTTCAATGTTGGATCAAAGATGTTCCTTCTTTGCATTTATTGCGATTGTTTTTCCACGAATATTCT---------------AATTTGACTAGTCTC---------ATTACTT------CAAATAAATC---------------CATTTACATCTTTTCAAAAAG---AAAGAAAAGATTCTTTTGGTTCCTACATAATTCTTATGTATATGAATACGAATATCTATTCCTGTTTCTTCGTAAACAGTC---TTATTTACGATCAATATCTTCTGGAGTCTTTCTTGAGCGAACACATTTCTATGGAAAAATAGAT------TATCTTATAAT---CGTGTGTTGTAATTCTTTTCAGAGG-ATCCTATGGTTCCTCAAAGATACTTTCATACATTATGTTCGATATCAAGGAAAAGCGATTCTTGCTTCAAAAGGAACTATTATTCTGATGAAGAAATGGAAATTTTATTTTGTTAATCTTTGGCAATCTTATTTTCACTTTTGGTTTCAACCTTATAGGATCCATATAAAACAATTACCCCACTATTCCTTCTCTTTTCT-GGGGTATTTTTCAAGTGTAC-TAAAAAATCCTTTGGTAGTAAGAAATCAAATGCTAGATAATTCATTTCTCATAAATACTCTGACTGAGAAATTAGATACCATAGTCCCAGTTATTTCTCTTATTGGATCATTGTCAAAAGCTCAATTTTGTACTTTCTTGGGTCATCCTATTAGTAAACCAATCTGGACCCATTTATCGGATTCTGATATTCTTGATCTATTTTGTCGGATATGTAGAAATCTTTGTCGTTATCACAGCGGATCCTCAAATAAACAGGTTTTGTATCGTATAAAGTATATACTTCGACTTTCGTGTGCTAGAACTTTGGCTCGTAAACATAAAAGTAC-AGTACGCACTTTTATGCGAAGGTTTGGTTCGGGTTTCTTAGAAGAA-------------------------------GAGCTATCCCGACCATTTCCCGTACATCATCCTAGCAGAGTACTTA-TATCTATGTCAATGAAAAGAA-CTAAAAAATTAAATTTGAACAA-------------------ATTGGACCTAG--CCCCT-GAAATT----------CTTAG-------ATC-TTCAAAAA----------GAAGAC---TTTTTTT-----------GTAAAT--TGAAGGAAAATATATGGACTAT-GAATGA------------TTCAATAAC-GGAGATTTATTGAAC-------------------------ATATATGTTCAT-------------ATCGTA----CTAT------ACAAAA--------CAAAT-----GAGA--------TTGGATTGGAA------GAA-----GAT------------------ACGAGGATTTAT------ATTCGTATCCATT------TGTGAAAGAACAGAG--TGAATGAAATGAGAAAG-------ATATTTCATTT--------------TGTTTAAACT------------GAGCCATTGATGGAAAGAGGGT-------------------------------GAGGATAAATA-AAGA------------------GCGAGGAA-----GGGAAGTAAAAT-----GGGC-TTTTTATTGGGGATAGAGGGCTATCTAT---------AAATGGATAATACT--------TTTGTATGTATATGAATTTTTGAAGATAG--CAAT---------CCCCCAATATCT-TGT---------------TCTAAGAACAAGATATT--GGGGGATTCTTTTG---CTTCTCTATCCG---------------AATTTTTG---------TTCTTTATCAT----AAAAGTTCTC-CCCCGCC---------AATGAATGATAAGT-GCC-----TAGGTG-AAGTAT----------------AGTATAAGATAAGTCAGA----------------------AAAGTCTAAGTCTTA-----ATACCTATACTCTTA----------CTATAA-GAT------------AAAGACTCTGAAGGATAAGGC-----TTTTCAC----------ATGAATACTTAGTAGAACGACTAACGACGAGATTTATTATCGTTTCTCGCGTGTCTCACGAAAGTGAGAGTAG-GTGCGAATTCTCCCAATTTGTGACCGACCATACGATCTGTGATATAAATGGTAAA-TGTTCCTTTCCATTATGAATAGC-GATTGTATGGCCAATCATTGTGGGTA-TAATGGTAGATGCCCGAGACCAAGTCACTATGATTTCTTTCTCCTCCCTCCTGTT-GAGTTTTTCAATTCTTCCCGATAAATGATTAGCTACAAAAGGATTTTTTTTT-AGTGAACGTGTCAC-GGCTGATTACTCCTTTTTTTCCTTTTTT-TAAATTGGCATCCTATGTCCAA----------TATCTCGATTTTAA-TCTGAAAG-TAT---------------------------AATG-ATGAATGA-AAAAAAGAGAAATTCC----------TTTTATCTAG-ATAA--------GGGAAAGGGGGC-GAATGTAG-CCAAGTGG-ATCAA-GGCAGT

Diploprora_championii_SG1230 ACGACT-CTCGACAATGGATATCTCGGCTCTCGCATCGATGAAGAGCGCAGCGAAATGCGATACGTGGTGCGAATTGCAGAATCCCGCGAACCATCGAGTCTTTGAACGCAAGTTGCGCCCGAGGCCAAT-CGGTCGAGGGCACGTCCGCCTGGGCGTCA-AGCGTTGCGCCGCTCCACAAGAATTCTTTTTCTTATCATTTTTAT------------TCTCAAATGGTATCAGAAGGTTTTGGAGTCATTCTGGAAATTTCATTCTCGTCGCGATTAGTATCC---------TTCCTTGAAG---AAAAAAGAATACCAAAATCTCATAATTTACGATCTATTCATTCAATATTTCCCTTTTTAGAGGATAAATTATCACATTTAAATTATGTGTCGGATCTACTAATACCCTATCCCATCCATCTGGAAATCTTGGTTCAAATCCTTCAATGCTGGATCAAAGATGTTCCTTCTTTGTATTTATTGCGATTGATTTTCCACGAATATCAT---------------AATTTGAATAGTCTC---------ATTACTT------CAAAAAAATC---------------TATTTACGTCTTTTCAAAAAA---AAAGAAAAGATTCTTTTGGTTCCTACATAATTTTTATGTATATGAATGCGAATATCTATTCCTCTTTCTTCGTAAACAGTCTTCTTATTTACGATCAATATCTTCTGGAGTCTTTCTTGAGCGAACACATTTTTATGGAAAAATAGAA------TATCTTAGAGT---CGTGTCTTGTAATTCTTTTCAGAGG-ATCCTATGGTTCCTCAAAGATATTTTCATACATTATGTTCGATATCAAGGAAAAGCAATTTTGGCTTCAAAAGGAACTCTTATTCTGATGAATAAATGGAAATTTCATTTTGTGAATTTTTGGCAATCTTATTTTCACTTTTGGTTTCAACCTTATAGGATCCATATAAAGCAATTACCCAATTATTCCTTTTCTTTTCT-GGGATATTTTTCAAGTGTAT-TAAAAAACCCTTTGGTAGTAAGAAATCAAATGCTAGAGAATTCATTTATAATAAATACTCTGACTAAGAAATTAGATACCATAGCTCCAGTTATTTTTCTTATTGGATCATTGTCGAAAGCTCAATTTTGTACTGTATTAGGTCATCCTATTAGTAAACCTATCTGGACAAATTTATCGGATTCTGATATTCTTGATCGATTTTGTCGGATATGTAGAAATCTTTGTCGTTATCACAGCGGATCCTCAAAGAAACAGGTTTTGTATCGTATAAAGTATATACTTCGACTTTCGTGTGCTAGAACTTTGGCTCGTAAACATAAAAGTAC-AGTACGCACTTTTATGCGAAGATTAGGTTCGGGATTCTTAGAAGAATTTTTTTTGGAAGAAGAACAATCTCTTTCTTGAGCTATCCCGACCATTTCCCGTGCATCATCCTAGCAGAGTACTTC-TATCTATGTCAATTAAAAGAA-CTAAAAAAGAAAATCTTAACAA-------------------ATTGGCTCTAG--CCCCT-GAAATT----------CTTGG-------ATC-TTCAAAAA----------TAAGAC--TTTTTTTT-----------GTAAA-TGTAAGGAAAAAGATATAGACTAT-GAATGA------------TTCAATAAC-GGAGATTCCTTGAAC-------------------------ATATATCTTCAT-------------ATCGTA----TTAT------ACAAAA--------CAAAT-----GAGA--------TTGGATTGGAA------AAA-----GAT------------------ACGAGGATTTAT------ATTCGGATCCATT------TGTGAAAGAACAGAG--TGAATAAAATGAGAAAG-------ATATTTCATTT--------------TGTTTAAACT------------GAGTGACTGATGAACAGAGGAT-------------------------------GAGGATAAATA-AAGA------------------GCGAGGAA----------GTAAAAT-----GGAC-TTTTTATTGGGGATAGAGGACCTTCGAT---------AAATGGATAATACT--------TTTTTATTCATATGAATTTTTGAAGATAG--CAAT---------CCCCCAATATCT-TGT---------------TCTAAGAACAAGATATT--GGGGGATTCTTTTG---CTTCTCTA---------------------TTTTCG---------TTCTTTATCAT----AAACGTTTTC-CCCCGCC---------AATGAATGATAAGT-GCC-----TAGGTG-AAGTAT----------------AGTATAAGATAAGTCAGA----------------------AAAGTCTAAGTCTTA-----ATACCTATACTCTTACTATAAGTTACTATAA-GATAAA-----AGATAAAGACTCTTAAGGATAAGGCTTTTCTTTTCAT----------ATGAATACTTAGTAGAACGACTAACGACGAGATTTATTATCGTTTCTCGCGTGTCTCACGAAAGTTAGAGTAG-GTGCGAATTCTCCCAATTTGTGACCGACCATACGATCTGTGATATAAATGGTAAA-TGTTCCTTTCCATTATGAATAGC-GATTGTATGGCCAATCATTGTGGGTA-TAATGGTAGATGCCCGAGACCAAGTCACTATGATTTCTTTCTCCTCCCTCCTGTT-GAGTTTTTCAATTCTTCCCAATAAATGATTAGCTACAAAAGGATTTTTTTTT-AGTGAACGTGTCAC-GGCTGATTACTCCTTTTTTTACATTTTT-TAAATTGGCATTCTATGTCCAA----------TATCTCGATCTTAA-TCTG-AAG-TCT---------------------------AATG-ATGAATGG-AAAAAAGAGAAAATCC----------TTTA-GCTAG-ATAA--------GGG-AA-GGGGC-GGATGTAG-CCAAGTGG-ATCAA-GGCAGT

Disperis_neilgherrensis ATGACTTCTTGGCAATGGATATCTTGGCTCTTGCATCGATGAAGAACATAGCGAAATGTGATACATGGTGCGAATTGCAGAATCCCGTGAACCATCGAGGTTTTGAATGCAAGTTGCGCCAAAGGCCAGC-AGGTCAAGGGCACGTCTGCTTGGGAGTCAAAATAACTCATCGCTCT-----------------------------------------------------------------------------------------------------------------------------------------------------------------------------------------------------------------------------------------------------------------------------------------------------------------------------------------TATCAT---------------AATCTTAATATTTTT---------TTTACTT------CAAAGAAATA---------------CTTTTACGTCTTTTCAAAAAA---AAAAAAAAGATTTTTTTGGTTCCCACAAAATTCTTATGTAAAAGAAGGCGAAGAAAAATCCCTGTTTCCCCGCAAACAGTCTTTTTATTTAAAATCAATATCTTTGGAAGTGTTTCAGGAGCGAACACTTTTCAAAAGAAAAATAAAC------TATTTTATAGT---AGTGTGTTGTCTTTCTTTTCAGAAG-ATTCTATGTTTTCTCAAAGATCCTTTCATACATTATGTTCGATATCAAGGAAAAGCTATTCTGGCTTCAAAGGGAACTCTCATTCTGATGAAGAAATGGAAATTTTTTATTTTCA-TTTTTGGCAATTATATTTGCACTTTTGGTCTCAACCTTATGGGATCCCTATAAAGCAATTACCCAAATATTCCTTCTCTTTTCG-GGGGTATTTTTTAAATGTAC-TAAAAAATAATTTGATAGTAAGAAATCAAATGTTAGAAAATTCATTTATAATAAATACTCTTACTAATAAATTACACACCATAGCCCCAGTTATTTCTCTTATTGGATCATTGTCAAAAGCTCGATTTCGTACTGTATGGGGTCATCCTATTAGTAAACCAATCTGGATCGATTTATCGGATCCTGATATTCTTGATCGATTTGGATGGATATATAAAAATCTTTGTCGTTATTACGGTGGATCCTCAAAGAAAAAGGGTTTGTATCGTAAAAAGTATATACTTCGACTTTCGTGTGCTAGAACTTTGGCTCGTAAACACAGAAGTAC-------------------------------------------------------------------------------------------------------------------------------------------------------------------------------------------------------------------------------------------------------------------------------------------------------------------------------------------------------------------------------------------------------------------------------------------------------------------------------------------------------------------------------------------------------------------------------------------------------------------------------------------------------------------------------------------------------------------------------------------------------------------------------------------------------------------------------------------------------------------------------------------------------------------------------------------------------------------------------------------------------------------------------------------------------------------------------------------------------------------------------------------------------------------------------------------------------------------------------------------------------------------------------------------------------------------------------------------------------------------------------------------------------------------------------------------------------------------------------------------------------------------------------------------------------------------------------------------------------------------------------------------------------------------------------------------------------------------------------------------------------------------------------------------------------------

Epipogium_roseum_SG1249 ACGACT-CTCGACAATGGATATCTCGGCTCTCGCATCGATGAAGAACGCAGCGAAATGCGATACGTGGTGCGAATTGCAGAATCCCGCGAACCATCGAGTCTTTGAACGCAAGTTGCGCCCAAGGCCAGT-AGGCCAAGGGCACGCCCGCTTGGGCGTCA-TGCATTGCATCTCTCC-----------------------------------------------------------------------------------------------------------------------------------------------------------------------------------------------------------------------------------------------------------------------------------------------------------------------------------------------------------------------------------------------------------------------------------------------------------------------------------------------------------------------------------------------------------------------------------------------------------------------------------------------------------------------------------------------------------------------------------------------------------------------------------------------------------------------------------------------------------------------------------------------------------------------------------------------------------------------------------------------------------------------------------------------------------------------------------------------------------------------------------------------------------------------------------------------------------------------------------------------------------------------------------------------------------------------------------------------------------------------------------------------------------------------------------------------------------------------------------------------------------------------------------------------------------------------------------------------------------------------------------------------------------------------------------------------------------------------------------------------------------------------------------------------------------------------------------------------------------------------------------------------------------------------------------------------------------------------------------------------------------------------------------------------------------------------------------------------------------------------------------------------------------------------------------------------------------------------------------------------------------------------------------------------------------------------------------------------------------------------------------------------------------------------------------------------------------------------------------------------------------------------------------------------------------------------------------------------------------------------------------------------------------------------------------------------------------------------------------------------------------------------------------------------------------------------------------------------------------------------------------------------------------------------------------------------------------------------------------------------------------------------------------------

Eria_scabrilinguis_SG1302 ACGACT-CTCGGCAATGGATATCTCGGCTCTCGCATCGATGAAGAGCGCAGCGAAATGCGATACGTGGTGCGAATTGCAGAATCCCGCGAACCATCGAGTCTTTGAACGCAAGTTGCGCCCGAGGCCAAC-CGGCCGAGGGCACGTCTGCCTGGGCGTCA-AGCGTTGCATCGCTCC---GGAA-TC---------CCAGTGTTCC--------------------GTGAACAGAATGTATTTGG----------------TATTTGGATCGCAATTAGTATCT---------TCCCTTGAAG---AAAAAAGAATACCAAAATCTCAGAATTTACGATCTATTCATTCAATATTTCCCTTTTTAGAGGATAAATTATCACATTTCAATTATGTGTCAGATCTACTAATACCCCATCCCATCCATCTGGAAATCTTGGTTCCAATTCTTCAATGCTGGATCAAAGATGTTCCTTCTTTGCATTTCTTGCGATTGTTTTTCCACGAATATCAT---------------AATTTGAATAGTCTC---------ATTATTT------CAAAGAAATC---------------CATTTACGTCTTTTCAAAAAGAAAAAAGAAAAGATTCTTTTGGTTCTTACATAATTCTTATGTATATGAATGCGAATATCTATTCCTATTTCTTCGTAAAAAGTCTTCTTATTTACGATCAATATCGTCTGGAGTCTTTCTTGAGAGAACACATTTCTATGGAAAAATAGAA------TATCTTATAGT---CGTGTATTTTAATTCTTTTCAGAGG-ATCCTATGGTTCCTCAAAGATACTTTCATACATTATGTTCGATATCAAGGAAAAGCAATTCTGGCTTCAAAAGGAACTCTTATTCTGATGAAGAAATGGAAATTTCATCTTGTGAATTTTTGGCAATCTTATTTTCACTTTTGGGTTCAACCTTATAGGATCCATATAAAGCAATTAACCAACTATTCCTTCTCTTTTCT-GGGGTATTTTTCAAGTGTAC-TAAAAAATACTTTGGTAGTAAGAAATCAAATGTTAGAGAATTCATTTCAAATAAATACTCTGACTAAGAAATTAGATACCATAGTCCCAGTTATTTCTCTTATTGGATCATTGTCGAAAGCTCAATTTTGTACTGTATTGGGTCATCCTATTAGTAAACCAATCTGGACCGATTTATCGGATTCTGATATTCTTGATCAATTTTGTCGGATATGTAGAAATCTTTGTCGTTATCACAGTGGATCCTCAAAGAAACAGGTTTTGTATCGTATAAAGTATATACTTCGACTTTCGTGTGCTAGAACTTTG------AAACGTTTAATTACTCGAATGTTTTTCGACAAGAAGAATCCCAATGAAATG-----AGGCATTCTTCTTAGTGAAACG--------------GAGCTATCCCGACCMTTTCCCGTGCATCATCCTAGCAGAGTACTTC-TATCTATGTCAATGAAAAGAA-CTAAAAAATAACATCTTAACAA-------------------ATTGGCCCTAG--CCCCT-GAATTT----------CTTAG-------ATC-TTCAAAAAAAAA------AAAGAC---TTTCTTT-----------GTAAA-TGTAAAGAAAAAGGTATGGACTAT-GAATGA------------TTCAATAAC-GGAGATTCTTTGAAC-------------------------ATATATGTTCAT-------------ATCGTA----CTAT------ACAAAA--------CAAAT-----GAGA--------TTGGATTGGAA------GAAGATATGAT------------------ACGAGGATTTCT------ATTCGGATCTATT------TGTGAAAAAATAGAG--TGAATGAAATGAGAAAG-------ATATTTCATTT--------------TGTTGAAACT------------GAGCCACTGATAGAAAGAGGAT-------------------------------GAGGATAAAGA-AAGA-------------------------------------------------------------------------CCATCTAT---------AAATGGATAATACT--------TTTGTATTCATATGAATTTTTGAAGATAG--CAAT---------CCCCCANTATCT-TGT---------------TCTTAGAACAAGATATT--GGGGGATTTTCAGG---GGTCTTTATCCG---------------AATTTTCG---------TTCTTTATCAT----ACAAGTTCTC-CCCCGCC---------AATGAATGATAAGT-GCC-----TAGGTG-AAGTAT----------------AGTATAAGATAAGTCAGA----------------------AAAGTCTAAGTCTTATTAGTATACCTATAA----------------------GAT------------AAAGACTC-TAA-GATAAGGC-----TTTTCAC----------ATGAATACTTAGTAGAACGACTAACGACGAGATTTCTTATCGTTTCTCGCGTGTCTCACGAAAGTTAGAGTAG-GTGCGAATTCTCCCAATTTGTGACCGACCATACGATCTGTGATATAAATGGTAAA-TGTTCCTTTCCATTATGAATAGC-GATTGTATGTCCAATCATTGTGGGTA-TAATGGTAGATGCCCGAGACCAAGTCACTATGATTTCTTTCTCCTCCCTCCTGTT-GAGTTTTTCAATTCTTCCCGATAAATGATTAGCTACAAAAGGATTTTTTTTT-AGTGAACGTGTCAC-GGCTGATTACTCCTTTTTTTCCATTTTT-TAAATTGGCATTCTATGTCCAA----------TATCTCGATCTTAA-TCTG-AAG-TAT---------------------------AATG-ATGAATGG-AAAAAAGAGAAAATCC----------TTTA-GCTAG-ATAA--------GGG-AA-GGGGC-GGATGTAG-CCAAGTGG-ATCAA-GGCAGT

Erythrodes_blumei_PK12103 ATGACT-CTCGGCAATGGATATCTTGGCTCTTGCATCGATGAAGAGCGCAGCGAAATGCGATACGTGGTGTGAATTGCAGAATTCCGTGAACCATCGAATTTTTGAACGCAAGTTGCGCCCGAGGCCAAT-TGGCTAAGGGCACGTCCGCCTGGGCGTCA-AGCATTACATCGCTTCATAAGAATTATTTTTCTTCTCATTTTTCT------------TTTCAAATACTATCAGAAGGTTTTGGAGTCATTCTGGAAATTCCATTATCGTCGCGATTAGTATCC---------TCCCTTGAAG---AAAAAAAAAGACCAAAATCTCAAAATTTACGATCTATTCATTCAATATTTTCTTTTTTAGAGGATAAATTTTCACATTTAAATTCTGTGTCAGATCTACTAATACCCCATCCGATCCATCTGGAAATCTTGGTTCAAATCCTTCAATGCTGGATCAAAGATGTTCCTTCTTTGCATTTGTTGCGATTGATTTTCCACGAATATCAT---------------AATTTGAAAAGTATC---------ATTACTT------CAAATAAATC---------------CATTCACGTCTTTTCAAAAAG---AAAGAAAAGATTTTTTTGGTTCCTACATAATATTTATGTATATGAATACGAATATCTATTTCTGTTTCTTCGTAAACAGTCTTCTTATTTACGATCAACATCTTTTGGAGTCTTTCTTGAGCGAACACTTTTTTATGTAAAAATTGAA------TCTATTCTAGT---AGTGTATTTTAATTCTTTTCATAGG-ATTCTCTGGTTCCTCAAAGATACTTTCATACATTATGTTCGATATCAAGGAAAAGTAATTTTGGCTTCAAAGGGAACTCTTATTCTGATGAAGAAATGGAATTTTCATGTTGTGAATTTTTGGCAATTTTATTTTCACTTTTGGTCTCAACCTTATAGGATCCATATAAAGCAATTACCCAACTATTCCTTCTCCTTTCT-GGGGTATTTTTTAAGTGTAC-AAAAAAAAACTTTGTTAGTAAGAAATCAAATGCTAGAGAATCACTTTCTAATAAATACTCTTACTAAGAAATTAGATACCATAGCCCCAGTTATTTCTCTTATTGGATCATTGTCGAAAGCCCAATTTTGTACTATATCGGGTCATCCTATTAGTAAACCAATCTGGACCGATTTATCGGATTCTGATATTATTGATAGATTTTGTCGGAAATGTAGAAATCTTTGTCGTTATCACAACGGATCCTCAAAAAAAAAGATTTTGTATCGTATAAAGTATATACTTCGACTTTCGTGTGCTAGAACTTTGGCTCGTAAACATAAAAGTAC-AGTACGCACTTTTATGCGAAGATTGGGTTCGGGATTTTTAGAAGAATTTTTTATGGAAGAAGAACAAGCTCTTTCTTGAGCTATCCCGACCAGTACCC-TGCATCATCCTAGCAGAGTACTTG-TATCTATGTAAACGAAAAGAA-CTAAAAAAGAAAGTCTTAACAA-------------------ATTGGACCTAGCCCCCCTTTAATTT----------CTTAG-------ATC-TTCAAAAA----------GAAAAC---TTTCTTT-----------GTAAA-TGTAAAGATAATTATATGAACTGT-GAATGATTAAATGAATTCTTAAATAAG-GGAGATTCCTTGAAC-------------------------ATATATGTTCAT-TTGTGCAGGT--ATCGTA---TCTAT------ACAAAG------------------AAAAAAAGAAAATTGGATTGGAATTGGAAGAA-----GAT------------------ACGAGGATTTCT------ATTCGGATCCATT------TGTGAAAGAACAGAG--TGAATGAAATTAGAAAG-------ATATTTCATTT--------------TGTTTGAACT------------GAACAACTGATAAAAAGAGGAT-------------------------------GAGAGTAAAGA-AAGA------------------GTGGGGAA----------TGAAAAA-----GGGC-TTTTTCTTGGGGATAGAGGGCCATCTAC---------AAATGGATAATACT--------TTTGTATTCAGATGATTTCTTTAAGGTAG--CAAT----------CCCCAATATCC-AATATATTGT-TCTTAGAACAATATATTGGATATT---GGGGATTCTTTTG---CTTCTTTATCTG------ATTTCTCCGAATTTTCG---------TTCTTTATCATAAAAGAAAGTTCTC-CCCCGCT---------AATGAATGATAAGT-GCC-----TAGGTG-AAGTAT----------------AGTATAAGATAAGTAAGA----------------------AAAGTCTAAGTCTTAGTA--ATACCTATACTCTTA----------CTATAA-GAT------------AAAGACTCTTAAGGATAAGAC-----TTTTCAC----------ATGAATACTTAGTAGAACGACTAACGACGAGATTTATTATCGTTTCTCGCATGTCTCACGAAAGTGAGAGTAG-GTGCGAATTCTCCCAATTTGTGACCGACCATACGATCTGTTATATAAATGGTAAA-TGTTCCTTTCCATTATGAATAGC-GATTGTATGGCCAATCATTGTGGGTA-TAATGGTAGATGCCCGAGACCAAGTCACTATTATTTCTTTCTCCTCCCTCATGTT-GAGTTTTTCAATTTTTCCCGATAAATGATTAGCTACAAAAGGATTTTTTTTT-AGTGAACGTGTCAC-GGCCGATTACTCCTTTTTTTACATTTTT-TAAATTGGCATTCTATGTCCAA----------TATCTCGATCTTAA-TCTG-AAG-TAT-GAGGGTAAGAATCAATACAAT-----AATG-ATGAATGG-AAAAAATAGAAAATAC----------TTTA-GCTAG-ATAA--------GGG-AA-GGGGC-GGATGTAG-CCAAGTGG-ATCAA-GGCAGT

Eulophia_flava_SG1158 ACGACT-CTCGGCAATGGATATCTCGGCTCTCGCATCGATGAAGAGCGCAGCGAAATGCGATACGTGGTGCGAATTGCAGAATCCCGCGAACCATCGAGTCTTTGAACGCAAGTTGCGCCTGAGGCCAGT-CGGCTGAGGGCACGTCCGCCTGGGCGTCA-AGCTTCGCGTTGCTCCACAAGAATTCTTTTTCTTCTCATTTTTAT------------TCTCAAATGGTATCAGAAGGTTTTGGAGTCATTTTGGAAATTCCATTCTCATCGCGATTAGTATCT---------TACCTTGAAGAAAAAAAAAGAATACCAAAATATCAGAATTTACGATCTATTCATTCAATATTTCCCTTTTTAGAGGATAAATTATCACATTTAAATTATGTGTCAGATATACTAATACCCTATCCCATCCATCTTGAAATCTTGGTTCAAATCCTTCAATGCTGGATAAAAGATGTTCCTTCTTTGCATTTCTTGCGATTGTTTTTCCACGAATCTCAT---------------AATTTTAATAATCTC---------ATTACTT------CAAAAAAATC---------------TATTTACGTCTTTTCAAAAAG---AAAGAAAAGATTCTTTTGGTTCCTACATAATTATTATGTATATGAATTCGAATATATATTCCTGTTTCTTCGTAAACAGTCTTCTTATTTACGATCAATATCTTCTGGAGTATTTCTTGAGCGAACACATTTCTATGGAAAAATAGAA------TATCTTATAGT---CGTGTGTTGTAATTCTTTTCAGAGG-ATCCTATGGTTTCTCAAAGATACTTTCATACATTATGTTCGATATCAAGGAAAAGCTATTTTGGCTTCAAAAGGAACTCTTATTCTGATGAAGAAATGGAAATTTCATCTTTTGAATTTTTGGCAATATTATTTTCACTTTTGGTTTCAACCTTATAGGATCCATATAAAGCAATTACCTAACTATTCCTTCTCTTTTCT-GGGGTTTTTTTCAAGTGTGC-TAAAAAATCCTTTGGTAGTAAGAAATCAAATGCTAGAGCATTCATTTCTAATAAATACTCTTACTAATAAATTAGATACAATAGTCCCAGTTATTTCTCTTATTGGATCATTGTCGAAAGCTAAATTTTGTACTGTATTGGGTCATCCTATTAGTAAATCAATCTGGACCGATTTATCGGATTCTGATATTCTTGATCGATTTTGTCGTATATGTAGAAATCTTTGTCGTTATCATAGCGGATCCTCAAAGAAGCAGGTTTTGTATCGTATAAAGTATATACTTCGACTTTCGTGTGCTAGAACTTTGGCTCGTAAACATAAAAGTAC-AGTACGAACTTTTATGCGAAGATTAGGTTCGGGATTCTTAGAAGAATTTTTTTTGGAAGAAGAACAATCTCTTTCTTGAGCTATCCCGACCATTTCGCGTGCATCATCCTAGCAGAGTACTTA-TATTTAAGTATTTCAAAAGAA-CTAAAAAATAAAATCTTAAAAA---------------------------------------------------------------------------------------------------------------------------------------------------AATGA------------TTCAATCGC-GGAGATTCCTTGAAC-------------------------ATATATGTTCAT-------------ATCGTA----TTAT------ACAAAAAAAAAT------------GAGA--------TTGGATTGGAA------GAA-----GAT------------------ACGAGGATTTAT------ATTCGTATCCATT------TGTGAAAGAACGGAG--TGAATGAAGCGAGAAAG-------AGATTTCATCT--------------TGTTTAAACT------------GAGCCACTGATGGAAAGAGGAT-------------------------------GAGGATAAATA-AAAA------------------GCGAGGAA----------GTAAAAG-----GGTC-TTTTTATTGGGGATAGAGGGCCATCTAT---------AAATGGATAAGACT--------TTTGTATACATTTCAATTTTTGAAGATAG--CAAT---------CCCCCAATATCT-TGT-----------GTTCTTAGAACACAAGATATT--GGGGGATT------------------------------------------------------TCTTGATCTT------G------------------------AATAAATGATAAGT-GCC-----TAGGTC--------------------------TTATGAAAAATAAGA----------------------AAAATAAAAGAAAT------CTACCTATACTCTTA----------CTATAA-GAT------------AAAGACTCTTAAG--TAAGGC-----TTTTCAC----------ATGAATACTTAGTAGAACGACTAACGACGAGATTTATTATCGTTTCTCGCGTGTCTCACGAAAGTTATAGTAG-GTGCAAATTCTCCCAATTTGTGACCGACCATACGATCTGTGATATAAATGGTAAA-TGTTCCTTTCCATTATGAATAGC-GATTGTATGGCCAATCATTGTGGGTA-TAATGGTAGATGCCCGAGACCAAGTCACTATGATTTCTTTCTCCTCCCTCCTGTT-GAGTTTTTCAATTCTTCCCGATAAATGATTAGCTACAAAAGGATTCTTTTTG-AGTGAACGTGTCAC-GGCTGATTACTCCTTTTTTTACATTTTT-TAAATTGGCATTCTATGTCCAA----------TATCTCGATCTTAA-TCTG-AAG-TAT---------------------------AATG-ATGAATGG-AAAAAAGAGAAAATCC----------TTTA-GCTAG-ATAA--------GGG-AA-GGGGC-GGATGTAG-CCAAGTGG-ATCAA-GGCAGT

Eulophia_graminea_SG1270 ACGACT-CTCGGCAATGGATATCTCGGCTCTCGCATCGATGAAGAGCGCAGCGAAATGCGATACGTGGTGCGAATTGCAGAATCCCGCGAACCGTCGAGTCTTTGAACGCAAGTTGCGCCTGAGGCCAGC-CGGCCGAGGGCACGTCCGCCTGGGCGTCG-AGCTTCGCGTCGCTCC----GAATTCTTTTTCTTCTCATTTTTAT------------TCTCAAATGGTATCAGAAGGTTTTGGAGTCATTCTGGAAATTCCATTCTCATCGCGATTAGTACCT---------TCCCTTGAAG---AAAAAAGAATACCAAAATATCAGAATTTACGATCTATTCATTCAATATTTCCCTTTTTAGAGGATAAATTATCACATTTAAATTATGTGTCAGATCTACTAATACCCTATCCCATCCATCTTGAAATCTTGGTTCAAATCTTTCAATGCTGGATAAAAGATGTTCCTTCTTTGCATTTCTTGCGATTGTTTTTCCACGAATCTCAT---------------AATTTTAATAATCTC---------ATTACTT------CAAAAAAATT---------------TATTTACGTCTTTTCAAAAAG---AAAGAAAAGATTCTTTTGGTTCCTACATAATTATTATGTATATGAATTCGAATATATATTCCTGTTTCTTCGTAAACAGTCTTCTTATTTACGATCAATATCTTCTGGAGTATTTCTTGAGCGAACACATTTCTATGGAAAAATAGAA------TATCTTATAGT---CGTGTGTTGTAATTCTTTTCAGAGG-ATCCTATGGTTCCTCAAAGATACTTTCATACATTATGTTCGATATCAAGGAAAAGCTATTTTGGCTTCAAAAGGAACTCTTATTCTGATGAAGAAATGGAAATTTCATCTTGTGAATTTTTGGCAATATTATTTTCACTTTTGGTTTCAACCTTATAGGATCCATATAAAGCAATTATCTAACTATTCCTTCTCTTTTCT-GGGTTTTTTTTTAAGTGTAC-TAAAAAATGATTTAGTAGTAAGAAATCAAATGCTAGAGAATTCATTTCTAATAAATACTCTTACTAATAAATTAGATACCATAGTCCCAGTTATTTCTCTTATTGGATCATTGTCGAAAGCTAAATTTTGTACTGTATTGGGTCATCCTATTAGTAAACCAAGCTGGACCGATTTATCGGATTCTGATATTCTTGATCGATTTTGTCGTATATGTAGAAATCTTTGTCGTTATTATAGCGGATCCTCAAAGAAGCAGGTTTTGTATCGTATAAAGTATATACTTCGACTTTCGTGTGCTAGAACTTTGGCTCGTAAACATAAAAGTAC-AGTACGAACTTTTATGCGAAGATTAGGTTCGGGATTCTTAGAAGAATTTTTTTTGGAAG------------------GAGCTATCCCGACCATTTCGCGTGCATCATCCTAGCAGAGTACTTA-TATTTATGTCAATGAAAAGAA-CTAAAAAAATAAAATCTTAAAAA------------------ATTGGCCCTAG-CCCCC--GAATTT----------CTTAG-------ATC-TTCAAAAA----------GAAGAC---TTTCTTT-----------GTAAA-TGTAAGTAAAAAGATATGGGCTAT-GAATGA------------TTCAATAAC-GGAGATTTCTTGAAC-------------------------ATATATGTTCAT-------------ATCGTA----TTAT------ACAAAA--------CAAAT-----GAGA--------TTGGATTGGAA------GAA-----GAT------------------ACGAGGATTTAT------ATTTGTATCCATT------TGTGAAAGAACAGAG--TGAATGAAGCGAGAAAG-------ATATTTCATCT--------------TGTTTAAACT------------GAGCCACTGATGGAAAGAGGAT-------------------------------GAGGATAAATA-AAAA------------------GCGAGGAA----------GTAAAAT-----GGTC-TTTTTATTGGGGATAGAGGGCCATCTAT---------AAATGGATAAGACT--------TTTGTATTCATATGAATTTTTGAAGATAG--CAAT---------CCCCCAATATCT-TGT-----------GTTCTTAGAA--CAAGATATT--GGGGGATT---------------------------------------TCTTG---------ATCTTGATCTT--------GTCTTG-----------------AATGAATGATAAGT-GCC-----TAGGTG-AAGTAT----------------AGTATAAGATAAGTCAGA----------------------AAAGTCTAAGT---------CTACCTATACTCTTA----------CTATAA-GAT------------AAAGACTATTAAG--TAAGGC-----TTTTCAC----------ATGAATACTTAGTAGAACGACTAACGACGAGATTTATTATCGTTTCTCGCGTGTCTCACGAAAGTTATAGTAG-GTGCAAATTCTCCCAATTTGTGACCGACCATACGATCTGTGATATAAATGGTAAA-TGTTCCTTTCCATTATGAATAGC-GATTGTATGGCCAATCATTGTGGGTA-TAATGGTAGATGCCCGAGACCAAGTCACTATGATTTCTTTCTCCTCCCTCCTGTT-GAGTTTTTCAATTCTTTCCGATAAATGATTAGCTACAAAAGGATTCTTTTTG-AGTGAACGTGTCAC-GGCTGATTACTCCTTTTTTTACATTTTT-GAAATTGGCATTCTATGTCCAA----------TATCTCGATCTTAA-TCTG-AAG-TAT---------------------------AATG-ATGAATGG-AAAAAAGAGAAAATAC----------TTTA-GCTAG-ATAA--------GGG-AA-GGGGC-GGATGTAG-CCAAGTGG-ATCAA-GGCAGT

Eulophia_picta_SG1271 ACGACT-CTCGGCAATGGATATCTCGGCTCTCGCATCGATGAAGAGCGCAGCGAAATGCGATACGTGGTGCGAATTGCAGAATCCCGCGAACCATCGAGTCTTTGAACGCAAGTTGCGCCTGAGGTCAGC-TGGCCGAGGGCACGTCCGCCTGGGCGTCA-AGCTTCGCGTCGCTCCACAAGGATTCTTTTTCTTCTCATTTTTAT------------TCTCAGATGGTATCAGAAGGTTTTGGAGTCATTCTGGAAATTCCATTCTCATCGCGATTAGTATCT---------TCCCTTGAAGAAAAAAAAAGAATACCAAAATATCAGAATTTACGATCTATTCATTCAATATTTCCCTTTTTAGAGGATAAATTATCACATTTAAATTATGTGTCAGATCTACTAATACCCTATCCCATCCATCTTGAAATCTTGGTTCAAATTCTTCAATGCTGGATAAAAGATGTTCCTTCTTTGCATTTCTTGCGATTGTTTTTCCACGAATCTCAT---------------AATTTTAATAATCTC---------ATTACTT------CAAAAAAATC---------------TATTTACGTCTTTTCAAAAAG---AAAGAAAAGATTCTTTTGGTTCCTACATAATTATTATGTATATGAATTCGAATATATATTCCTGTTTCTTTGTAAAAAGTCTTCTTATTTACGATCAATATCTTCTGGAGTATTTCTTGAGCGAACACATTTCTATGGAAAAATAGAA------TATCTTATAGT---CGTGTGTTGTAATTCTTTTCAGAGG-ATCCTATGGTTCCTCAAAGATACTTTCATACATTATGTTCGATATCAAGGAAAAGCTATTTTGGCTTCAAAAGGAACTCTTATTCTGATGAAGAAATGGAAATTTCATCTTGTGAATTTTTGGCAATATTATTTTCACTTTTGGTTTCAACCTTATAGGATCCATATAAAGCAATTACCTAACTATTCCTTCTCTTTTTT-GGGGTTTTTTTCAAGTGTAC-TAAAAAATCCTTTGGTAGTAAGAAATCAAATGCTAGAGAATTCATTTCTAATAAATACTTTTACTAATAAATTAGATACCATAGTTCCAGTTATTTCTCTTATTGGATCATTGTCGAAAGCTAAATTTTGTACTGTATTGGGTCATCCTATTAGTAAACCGATCTGGACCGATTTATCGGATTCTGATATTCTTGATCGATTTTGTCGTATATGTAGAAATCTTTGTCGTTATCATAGCGGATCCTCAAAGAAGCAGGTTTTGTATCGTATAAAGTATATACTTCGACTTTCGTGTGCTAGAACTTTGGCTCGTAAACATAAAAGTAC-AGTACGAACTTTTATGCGAAGATTAGGTTCGGGATTCTTAGAAGAATTTTTTTTGGAAGAAGAGAAATCTCTTTCTTGAGCTATCCCGACCATTTCGCGTGCATCATCCTAGTAGAGTACTTA-GATTTATGTCAATGAAAAGAA-CTAAAAAATAAAATCTTAAAAA-------------------ATTGGCCCTAG--CCCCT-GAATTT----------CTTAG-------ATC-TTCAAAAA----------GAAGAC---TTTCTTT-----------GTAAA-TGTAAGTAAAAAGATACGGACTAT-GAATGA------------TTCAATAAC-GGAGATTCCTTGAAC-------------------------ATATATGTTCAT-------------ATCGTA----TTAT------ACAAA---------CAAAA-----GAGA--------TTGGATTGGAA------GAA-----GAT------------------ACGAGGATTTAT------ATTCGTATCCATT------TGTGAAAGAACAGAG--TGAATGAAGCGAGAAAG-------ATATTTCATCT--------------TGTTTAAACC------------GAGCCACTGATGAAAAGAGGAT-------------------------------GAGGATAAATA-AAAA------------------GCGAGGAA----------GTAAAAT-----GGTC-TTTTTATTGGGGATAGAGGGCCATCTAT---------AAATGGATAAGACT--------TTTGTATACATATGAATTTTTGAAGATAG--CAAT---------CCCCCAATATCT-TGT-------------GTTCTTAGAACAAGATATT--GGGGGATT------------------------------------------------------TCTTGATCAT----AAAAGTTCTC-CCCCGCC---------AATGAATGATAAGT-GCC-----TAGGTG-AAGTAT----------------AGTATAAGATAAGTCAGA----------------------AAAGTCTAAGTCTTA-----CTACCTATACTCTTA----------CTATAA-GAT------------AAAGACTCTTAAG--GAAGGC-----TTTTCAC----------ATGAATACTTAGTAGAACGACTAACGACGAGATTTATTATCGTTTCTCGCGTGTCTCACGAAAGTTATAGTAG-GTGCAAATTCTCCCAATTTGTGACCGACCATACGATCTGTGATATAAATGGTAAA-TGTTCCTTTCCATTATGAATAGC-GATTGTATGGCCAATCATTGTGGGTA-TAATGGTAGATGCCCGAGACCAAGTCACTATGATTTCTTTCTCCTCCCTCCTGTT-GAGTTTTTCAATTCTTCCCGATAAATGATTAGCTACAAAAGGATTCTTTTTG-AGTGAACGTGTCAC-GGCTGATTACTCCTTTTTTTCCATTTTT-TAAATTGGCATTCTATGTCCAA----------TATCTCGATCTTAA-TCTG-AAG-TAT---------------------------AACG-ATGAATGG-AAAAAAGAGAAAATCC----------TTTA-GCTAG-ATAG--------GGG-AA-GGGGC-GGATGTAG-CCAAGTGG-ATCAA-GGCAGT

Eulophia_zollingeri_SG1262 ACGACT-CTCGACAATGGATATCTCGGCTCTCGCATCGATGAAGAGCGCAGCAAAATGCGATACGTGGTGCGAATTGCAGAATCCCGCGAACCATCGAGTCTTTGAACGCAAGTTGCGCCTGAGGCCAGC-CGGCCGAGGGCACGTCCGCCTGGGCGCCA-AGCTTCGCGTCGCTCCACAAGAATTCTTTTTCTTCTCATTTTTGT------------TCTCAAATGGTATCAGAAGGTTTTGGAGTCATTTTGGAAATTCCATTCTCATCGCGATTAGTATCT---------TACCTTGAAGAAAAAAAAAGAATACCAAAATATCAGAATTTACGATCTATTCATTCAATATTTCCCTTTTTAGAGGATAAATTATCACATTTAAATTATGTGTCAGATCTACTAATACCCTATCCCATCCATCTTGAAATCTTGGTTCAAATCCTTCAATGCTGGATAAAAGATGTTTCTTCTTTGCATTTCTTGCGATTGTTTTTCCACGAATCTCAT---------------AATTTTAATAATCTC---------ATTACTT------CAAAAAAATA---------------TATTTACGTCTTTTCAAAAAG---AAAGAAAAGATTCTTTTGGTTCCTACATAATTATTATGTATATGAATTCGAATATATATTCCTGTTTCTTCGTAAACAGTCTTCTTATTTACGATCAATATCTTCTGGAGTATTTTTTGAGCGAACACATTTCTATGGAAAAATAGAA------TATCTTATAGT---TGTGTGTTGTAATTCTTTTCAGAGG-ATCCTATGGTTTCTCAAAGATACTTTCATACATTATGTTCGATATCAAGGAAAAGCTATTTTGGCTTCAAAAGGAACTCTTATTCTGATGAATAAATGGAAATTTCATCTTGTGAATTTTTGGCAATATTATTTTCACTTTTGGTTTCAACCTTATAGGATCCATATAAAGCAATTACCTAACTATTCCTTCTCTTTTCT-GGGGTTTTTTTCAAGTGTAC-TAAAAAATCCTTTGGTAGTAAGAAATCAAATGCTAGAGCATTCATTTCTAATAAATACTCTTACTAATAAATTAGATACAATAGTCCCAGTTATTTCTCTTATTGGATCATTGTCGAAAGCTAAATTTTGTACTGTATTGGGTCATCCTATTAGTAAATCAATCTGGACCGATTTATCGGATTCTGATATTCTTGATCGATTTTGTCGTATATGTAGAAATCTTTGTCGTTATCATAGCGGATCCTCAAAGAAGCAGGTTTTGTATCGTATAAAGTATATACTTCGACTTTCGTGTGCTAGAACTTTGGCTCGTAAACATAAAAGTAC-AGTACGAACTTTTATGCGAAGATTAGGTTCGGGATTCTTAGAAGAATTTTTTTTGGAAGAAGAACAATCTCTTTCTTGAGCTATCCCGACCATTTCGCGTGCATCATCCTAGCAGAGTACTTA-TATTTAAGTATTTCAAAAGAA-CTAAAAAATAAAATCTTAAAAA---------------------------------------------------------------------------------------------------------------------------------------------------AATGA------------TTCAATAGC-GGAGATTCCTTGAAC-------------------------ATATATGTTCAT-------------ATCGTA----TTAT------ACAAAAAAAT--------------GAGA--------TTGGATTGGAA------GAA-----GAT------------------ACGAGGATTTAT------ATTCGTATCCATT------TGTGAAAGAACGGAG--TGAATGAAGCGAGAAAT-------ATATTTCATCT--------------TGTTTAAACT------------GAGCCACTGATGAAAAGAGGAT-------------------------------GAGGATAAATA-AAAA------------------GCGAGTTTAAACTGAGAAGTAAAAT-----GGTC-TTTTTATTGGGGATAGAGGACCATCTAT---------AAATGGATAAGACT--------TTTTTATACATTTCAATTTTTGAAGATAG--CAAT---------CCCCCAATATCT-TGT-----------GTTCTTAGAACACAAGATATT--GCTATATTATTAT------------------------------------------------TGCTATATCAT----AAAAGTTCTC-CCCCGCC---------AATGAATGATAAGT-GCCAATGATAAGTG-CCTAGG-----------------TCTTATGAAAAATAAGA----------------------AAAATAAAAGAAAT---------TCTATACTCTTA----------CTATAA-GAT------------AAAGACTCTTAAG--TAAGGC-----TTTTCAC----------ATGAATACTTAGTAGAACGACTAACGACGAGATTTATTATCGTTTCTCGCGTGTCTCACAAAAGTTATAGTAG-GTGCAAATTCTCCCAATTTGTGACCGACCATACGATCTGTGATATAAATGGTAAA-TGTTCCTTTCCATTATGAATAGC-GATTGTATGGCCAATCATTGTGGGTA-TAATGGTAGATGCCCGAGACCAAGTCACTATGATTTCTTTTTCCTCCCTCCTGTT-GAGTTTTTCAATTCTTCCCGATAAATGATTAGCTACAAAAGGATTCTTTTTG-AGTGAACGTGTCAC-GGCTGATTACTCCTTTTTTTACATTTTT-TAAATTGGCATTCTATGTCCAA----------TATCTCGATCTTAA-TCTG-AAG-TAT---------------------------AATG-ATGAATGG-AAAAAAGAGAAAATCC----------TTTA-GCTAG-ATAA--------GGG-AA-GGGGC-GGATGTAG-CCAAGTGG-ATCAA-GGCAGT

Gastrochilus_japonicus_KFBG308 ACGACT-CTCGACAATGGATATCTCGGCTCTCGCATCGATGAAGAGCGCAGCGAAATGCGATACGTGGTGCGAATTGCAGAATCCCGCGAACCATCGAGTCTTTGAACGCAAGTTGCGCCCGAGGCCAAT-CGGTCGAGGGCACGTCCGCCTGGGCGTCA-AGCGTCGCGCCGCTCCACAAGAATTCTTTTTCTTATCATTTTTAT------------TCTCAAATGATATCAGAAGGTTTTGGAGTCATTCTGGAAATTTCATTCTCGTCGCGATTAGTATCT---------TCCCTTGAAG---AAAAAAGAATACCAAAATCTCAGAATTTACGATCTATTCATTCAATATTTCCCTTTTTAGAGGATAAATTATTACATTTAAATTATGTGTCGGATCTACTAATACCCTATCCCATCCATCTGGAAATCTTGGTTCAAATCCTTCAATGCTGGATCAAAGATGTTCCTTCTTTGCATTTATTTCGATTGATTTTCCACGAATATCAT---------------AATTTGAATAGTCTC---------ATTACTT------CAAAAAAATA---------------CATTTACGTCTTTTCAGAAAA---AAAGAAAAGATTCTTTTGGTTCCTACATAATTTTTATGTATATGAATGCGAATATATATTCCTCTTTCTTCGTAAACAGTCTTCTTATTTACGATCAATATCTTCTGGAGTCTTTCTTGAGCGAACACATTTTTATGGAAAAATAGAA------TATCTTAGAGT---CGTGTCTTGTAATTCTTTTCAGAGG-ATCCTATGGTTCCTTAAAGATATTTTCATACATTATGTTCGATATCAAGGAAAAGCAATTTTGGCGTCAAAAGGAACTCTTATTCTGATGAATAAATGGAAATTTCATTTTGTGAATTTTTGGCAATCTTATTTTCACTTTTGGTTTCAACCTTATAGGATCCATATAAAGCAATTACCCAATTATTCCTTCTCTTTTCT-GGGATATTTTTCAAGTGTAC-TAAAAAACCCTTTGGTAGTAAGAAATCAAATGCTAGAGAATTCATTTCTAATAAATACTCTGACTAAGAAATTAGATACCATAGCTCCAGTTATTTTTCTTATTGGATCATTGTCGAAAGCTCAATTTTGTACTGTATTAGGTCATCCTATTAGTAAACCGATCTGGACCAATTTATCGGATTCTGATATTCTTGATCGATTTTGTCGGATATGTAGAAATCTTTGTCGTTATCACAGCGGATCCTCAAAGAAACAGGTTTTGTATCGTATAAAGTATATACTTCGATTTTCGTGTGCTAGAACTTTGGCTCGTAAACATAAAAGTAC-AGTACGCACTTTTATGCGAAGATTAGGTTCGGGATTCTTAGAAGAATTTTT--------------------------GAGCTATCCCGACCATTTCCCGTGCATCATCCTAGCAGAGTACTTC-TATCTATGTCAATGAAAAGAA-TT-AAAAAAAAAATCTTAACAA-------------------ATTGGCTCTAG--CCCCT-GAAATT----------CTTGG-------ATC-TTCAAAAA----------GAAGAC-TTTTTTTTT-----------GTAAA-TGTAAGGAAAAAGATATAGACTAT-GAATGA------------TTCAATAAC-GGAGATTCCTTGAAC-------------------------ATATATCTTCAT-------------ATCGTA----TTAT------ACAAAA--------CAAAT-----GAGA--------TTGGATTGGAA------AAA-----GAT------------------ACGAGGATTTAG------ATTCGGATCCATT------TGTGAAAGAACAGAG--TGAATAAAATGAGAAAG-------ATATTTCATTT--------------TGTTTAAACT------------GAGTCACTAATGAACAGAGGAT-------------------------------GAGGATAAATA-AAGA------------------GCGAGGAA----------GTAAAAT-----GGGC-TTTTTATTGGGGATAGAGGGCCTTCGAT---------AAATGGATAATACT--------TTTTTATTCATATGAATTTTTGAAGATAG--CAAT---------CCCCCAATATCT-TG---------------TTCTAAGAACAAGATATT--GGGGGATTCTTTTG---CTTCTCTA---------------------TTTTCG---------TTCTTTATCAT----AAAAGTTTTC-CCCCGCC---------AATGAATGATAAGT-GCC-----TAGGTG-AAGTAT----------------AGTATAAGAAAAGTCAGA----------------------AAAGTCTAAGTCTTATTAGTATACCTATACTCTTA----------CTATAA-GATAAA-----AGATAAAGACTCTTAAGGATAAGGCTTTTATTTTCATATGAATACTTATGAATACTTAGTAGAACGACTAACGACGAGATTTATTATCGTTTCTCGCGTGTCTCACGAAAGTTAGAGTAG-GTGCGAATTCTCCCAATTTGTGACCGACCATACGATCTGTGATATAAATGGTAAA-TGTTCCTTTCCATTATGAATAGC-GATTGTATGGCCAATCATTGTGGGTA-TAATGGTAGATGCCCGAGACCAAGTCACTATGATTTCTTTCTCCTCCCTCCTGTT-GAGTTTTTCAATTCTTCCCAATAAATGATTAGCTACAAAAGGATTTTTTTTT-AGTGAACGTGTCAC-GGCTGATTACTCCTTTTTTTACATTTTT-TAAATTGGCATTCTATGTCCAA----------TATCTCGATCTTAA-TCTG-AAG-TCT---------------------------AATG-ATGAATGG-AAAAAAGAGAAAATCC----------TTTA-GCTAG-ATAA--------GGG-AA-GGGGC-GGATGTAG-CCAAGTGG-ATCAA-GGCAGT

Gastrochilus_kadooriei_PK12022 ACGACT-CTCGACAATGGATATCTCGGCTCTCGCATCGATGAAGAGCGCAGCGAAATGCGATACGTGGTGCGAATTGCAGAATCCCGCGAACCATCGAGTCTTTGAACGCAAGTTGCGCCCGAGGCCAAT-CGGTCGAGGGCACGTCCGCCTGGGCGTCA-AGCGTTGCGCCGCTCCACAAGAATTCTTTTTCTTATCATTTTTAT------------TCTCAAATGATATCAGAAGGTTTTGGAGTCATTCTGGAAATTTCATTCTCGTCGCGATTAGTATCC---------TCCCTTGAAG---AAAAAAGAATACCAAAATCTCAGAATTTACGATCTATTCATTCAATATTTCCCTTTTTAGAGGATAAATTATCACATTTAAATTATGTGTCGGATCTACTAATACCCTATCCCATCCATCTGGAAATCTTGGTTCAAATCCTTCAATGCTGGATCAAAGATGTTCCTTCTTTGCATTTATTGCGATTGATTTTCCACGAATATCAT---------------AATTTGAATAGTCTC---------ATTACTT------CAAAAAAATA---------------CATTTACGTCTTTTCAGAAAA---AAAGAAAAGATTCTTTTGGTTCCTACATAATTTTTATGTATATGAATGCGAATATATATTCCTCTTTCTTCGTAAACAGTCTTCTTATTTACGATCAATATCTTCTGGAGTCTTTCTTGAGCGAACACATTTTTATGGAAAAATAGAA------TATCTTAGAGT---CGTGTCTTGTAATTCTTTTCAGAGG-ATCCTATGGTTCCTTAAAGATATTTTCATACATTATGTTCGATATCAAGGAAAAGCGATTTTGGCTTCAAAAGGAACTCTTATTCTGATGAATAAATGGAAATTTCATTTTGTGAATTTTTGGCAATCTTATTTTCACTTTTGGTTTCAACCTTATAGGATCCATATAAAGCAATTACCCAATTATTCCTTCTCTTTTCT-GGGATATTTTTCAAGTGTAC-TAAAAAACCCTTTGGTAGTAAGAAATCAGATGCTAGAGAATTCATTTCTAATAAATACTCTGACTAAGAAATTAGATACCATAGCTCCAGTTATTTTTCTTATTGGATCATTGTCGAAAGCTCAATTTTGTACTGTATTAGGTCATCCTATTAGTAAACCGATCTGGACCAATTTATCGGATTCTGATATTCTTGATCGATTTTGTCGGATATGTAGAAATCTTTGTCGTTATCACAGCGGATCCTCAAAGAAACAGGTTTTGTATCGTATAAAGTATATACTTCGATTTTCGTGTGCTAGAACTTTGGCTCGTAAACATAAAAGTAC-AGTTCGCACTTTTATGCGAAGATTAGGTTCGGGATTCTTAGAAGAATTTTTTTTGGAAGAAGAACAATCTCTTTCTTGAGCTATCCCGACCATTTACCGTGCATCATCCTAGCAAAGTACTTC-GATTTATGTCAATGAAAAGAA-CTAAAAAAGAAAATATTAACAA-------------------ATTGGCTCTAG--CCCCT-GAAATT----------CTTGG-------ATC-TTCAAAAA----------GAAGAC-TTTTTTTTT-----------GTAAA-TGTAAGGAAAAAGATATAGACTAT-GAATGA------------TTCAATAAC-GGAGATTCCTTGAAC-------------------------ATATATCTTCAT-------------ATCGTA----TTAT------ACAAAA--------CAAAT-----GAGA--------TTGGATTGGAA------AAA-----GAT------------------ACGAGGATTTAG------ATTCGGATCCATT------TGTGAAAGAACAGAG--TGAATAAAATGAGAAAG-------ATATTTCATTT--------------TGTTTAAACT------------GAGTCACTGATGAACAGAGGAT-------------------------------GAGGATAAATA-AAGA------------------GCGAGGAA----------GTAAAAT-----GGGC-TTTTTATTGGGGATAGAGGGCCTTCGAT---------AAATGGATAATACT--------TTTTTATTCATATGAATTTTTGAAGATAG--CAAT---------CCCCCAATATCT-TGTTCTA---------------AGAACAAGATATT--GGGGGATTCTTTTG---CTTCTCTA---------------------TTTTCG---------TTCTTTATCAT----AAAAGTTTTC-CCCCGCC---------AATGAATGATAAGT-GCC-----TAGGTG-AAGTAT----------------AGTATAAGA-------------------------------AAAGTCTAAGTCTTATTAGTATACCTATACTCTTA----------CTATAA-GATAAA-----AGAGAAAGACTCTTAAGGATAAGGCTTTTCTTTTCAT----------ATGAATACTTAGTAGAACGACTAACGACGAGATTTATTATCGTTTCTCGCGTGTCTCACGAAAGTTAGAGTAG-GTGCGAATTCTCCCAATTTGTGACCGACCATACGATCTGTGATATAAATGGTAAA-TGTTCCTTTCCATTATGAATAGC-GATTGTATGGCCAATCATTGTGGGTA-TAATGGTAGATGCCCGAGACCAAGTCACTATGATTTCTTTCTCCTCCCTCCTGTT-GAGTTTTTCAATTCTTCCCAATAAATGATTAGCTACAAAAGGATTTTTTTTT-AGTGAACGTGTCAC-GGCTGATTACTCCTTTTTTTACATTTTT-TAAATTGGCATTCTATGTCCAA----------TATCTCGATCTTAA-TCTG-AAG-TCT---------------------------AATG-ATGAATGG-AAAAAAGAGAAAATCC----------TTTA-GCTAG-ATAA--------GGG-AA-GGGGC-GGATGTAG-CCAAGTGG-ATCAA-GGCAGT

Gastrodia_peichatieniana_AFCDHK43268 ACGACT-CTCGGCAATGGATATCTCGGCTCTCGCATCGATGAAGAGCGCAGCGAAATGCGATACGTGGTGCGAATTGCAGAATCCCGCGAACCATCGAGTCTTTGAACGCAAGTTGCGCCCGAGGCCAAT-CGGCCAAGGGCACGCCCGCCTGGGCGTCC-AGCATTACGCCGCTCCACAAGAATTCCTTTTCTTCTCATTTTTCT------------TCTCAAATGGTATCAGAAGGTTTTGGAATCATTCTGGAAATTCCATTCTCGTCGCGATTAGTATCT---------TCCCTTGAAG---AAAAAAAAAGACCAAAATCTCAGAATTTACGATCTATTCATTCAATATTTCCCTTTTTAGAGGATAAATTCTCGCATTTAAATTATGTGTCAGATCTAATAATACCCCATCCCCTCCATCTGGAAATCTTGGTTCAAATCCTTCAATGCTGGATCAAAGATGTTCCTTCTTTGCATTTATTGCGATCTTTTTTCCACGAATATCAT---------------AATTTGAATAGTCTC---------ATTACTT------CAAAGAAATC---------------CATTCATGTCTTTTCGAAAAG---AAAGAAAAGATTCTTTTGGTTCCTACATAATTCTTATGTATATGAATGCGAATATATATTCCTTTTTCTTCGTAAAGAGTCTTCTTATTTACGATCAACATCTTCTGGAGTCTTTCTTGAGCGAACACATTTCTATGGAAAAATAGAA------TATCTTCTAGT---AGTGTGTTTTAATTCTTTTCGGAGG-ATTCTATGGTTCCTCAAAGATCCTTTCATACATTATGCTCGATATCAAGGAAAAGCAATTATGGCTTCAAAGGGAACTCTTATTCTGATGAAGAAATGGAAATTTCATCTTGTGAATCTTTGGCAATCTTATTTTCACTTTTGGTCTCAACCTTATAGGATCCATATAAAGCAATTACCCAACTCTTCCTTCTCTTTTCT-GGGGTATTTTTCAAGTGTAC-TAAAAAATACTTTGGTAGTAAGAAATCAAATGCTAGAGAATTCATTTCTAATAAATACTCTGACTAAAAAATTCGATACCATAGCCCCAGTTATTTCTCTTATTGGATCATTGTCGAAAGCTCAATTTTGTACTGTATTGGGTCATCCTATTAGTAAGCCGATCTGGACCGATTTATCGGATTCTGATATTCTTGATCGATTTTGTCGGATATGTAGAAATCTTTGTCGTTATCACAGCGGATCCTCAAAGAAACAGGTTTTGTATCGTATAAAGTATATACTTCGACTTTCGTGTGCTAGAACTTTGGCTCGTAAACATAAAAGTAC-AGTACGCACTTTTATGCGAAGATTAGGTTCGGGATTCTTAGAAGAATTTTTTTTGGAAGAAGAAAAAGTTCTTTCTT-------------------------------------------------------------------------------------------------------------------------------------------------------------------------------------------------------------------------------------------------------------------------------------------------------------------------------------------------------------------------------------------------------------------------------------------------------------------------------------------------------------------------------------------------------------------------------------------------------------------------------------------------------------------------------------------------------------------------------------------------------------------------------------------------------------------------------------------------------------------------------------------------------------------------------------------------------------------------------------------------------------------------------------------------------------------------------------------------------------------------------------------------------------------------------------------------------------------------------------------------------------------------------------------------------------------------------------------------------------------------------------------------------------------------------------------------------------------------------------------------------------------------------------------------------------------------------------------------------------------------------------------------------------------------------------------------------------------------------

Goodyera_foliosa_SG1309 ATGACT-CTCGGCAATGGATATCTTGGCTCTTGCATCGATGAAGAGCGCAGCGAAATGCGATACGTGGTGTGAATTGCAGAATCCCGTGAACCATCGAGTTTTTGAACGCAAGTTGCGCCCGAGGCCAAT-TGGCTAAGGGCACGTCCGCCTGGGCGTCA-AGCATTACGTCGCTTCATAAGAA------------TGATTTTTCT------------TTTCAAATACTATCAGAAGGTTTTGGAGTCATTCTGGAAATTCCATTATCGTCGCAATTAGTATCC---------TCCCTTGAAG---AAAAAAAAATACCAAAATCTCAGAATTTACGATCTATTCATTCAATATTTCCTTTTTTAGAGGATAAATTATCACATTTAAATTCTGTGTCAGATCTACTAATACCCCATCCCATCCATCTGGAAATCTTGGTTCAAATCCTTCAATGCTGGATCAAAGATGTTCCTTCTTTGCATTTGTTGCGATTGATTTTCCACGAATATCAT---------------AATTTGAAGAGTATC---------ATTACTT------CAAAGAAATC---------------CATTCACATCTTTTCAAAAAG---AAAGAAAAGATTTTTTTGGTTCCTACATAATTTTTATGTATATGAATGCGAATATCTATTTCTGTTTCTTCGTAAAAAGTCTTCTTATTTACGATCAACATCTTTTGGAGTCTTTCTTGAGCGAACAATTTTTTATGTAAAAATTGAA------TCTATTCTAGT---AGTGTATTTTCCTTCTTTTCAGAGG-ATTCTCTGGTTCCTCAAAGATCCTTTCATACATTATGTTCGATATCAAGGAAAAGTACTTTTGGCTTCAAAAGGAACTCTTATTCTGATGAAGAAATGGAATTTTCATGTTGTGAATTTTTGGCAATTTTATTTTCACTTTTGGTCTCAACCTTATAGGATCCATATAAAGCAATTACCCAACTATTCCTTCTCCTTTCT-GGGGTATTTTTTCAGTGTAC-CAAAAAAAACTTTGGTAGTAAGAAATCAAATGCTAGAGAATTCCTTTCTAATAAATACTCTGACTAAGAAATTAGATACCATAGCCCCAGTTATTTCTCTTATTGGATCATTGTCGAAAGCTCAATTTTGTACTATATCGGGTCATCCTATTAGTAAACCAATCTGGACCGATTTATCGGATTCTGATATTATTGATCGATTTTGTCGGAAATGTAGAAATCTTTGTCGTTATCACAGCGGATCCTCAAAAAAAAAAGTTTTGTATCGTATAAAGTATATACTTCGACTTTCGTGTGCTAGAACTTTGGCTCGTAAACATAAAAGTAC-AGTACGCACTTTTATGCGAAGATTGGGTTCGGGATTTTTAGAAGAATTTTTTATGGAAGAAGAACAAGCTCTTTCTTGAGCTATCCCGACCAGTACCC-TGCATCATCCTAGCAGAGTACTTT-TATCTATGTAAAAGAAAAGAA-CTAAAAAAGAAATTCTTAG----------------------------------CCCCCTTTAATTT----------CTTAG-------ATC-TTCAAAAA----------GAAGAC---TTTCTTT-----------GTAAA-TGTAAAGATAATGATATGAACTGT-GAATGATTCAATGAATTATTCAATAAG-GGAGATTCCTTGAAC-------------------------ATATATGTTCAT-TTGTGCAGGT--ATCGTA---TCTAT------ACAAAGAAAAA---CAAAG-----AAAA--------TTGGATTGGAATTGGAATAA-----GAT------------------ACGAGAATTTCT------ATTCGAATCCATT------TGTGAAAGAACAGAG--TGAATGAAATTAGAAAG-------ATATTTCATTT--------------TGTTTGAACT------------GAACAACTTCTAAAAAGAGGAT-------------------------------GAGAGTAAAGA-AAGA------------------GTGGGAAT----------TAAAAATGT----------------------------CCATCTAC---------AAATGGATAATACT--------TTTGTATTCAGATGAATTCTTTAAGGTAG--CAAT----------CCCCAATATCC-AATATATTGT-TCTAAGAACAATATATTGGATATT---GGGGATTCTTTTG---CTTCTTTATCCG------ATTTCTCCGAATTTTCG---------TTCTTTATCATAAAAGAAAGTTCTC-CCCCGCT---------AATGAATGATAAGT-GCC-----TAGGTG-AAGTAT----------------AGTATAAGATAAGTAAGA----------------------AAAGTCTAAGTCTTAGTATAATACCTATACTCTTA----------CTATAA-GAT------------AAAGACTCTTAAGGATAAGAC-----TTTTCAC----------ATGAATACTTAGTAGAACGACTAACGACGAGATTTATTATCGTTTCTCGCATGTCTCACGAAAGTGAGAGTAG-GTGCGAATTCTCCCAATTTGTGACCGACCATACGATCTGTTATATAAATGGTAAA-TGTTCCTTTCCATTATGAATAGC-GATTGTATGGCCAATCATTGTGGGTA-TAATGGTAGATGCCCGAGACCAAGTCACTATTATTTCTTTCTCCTCCCTCATGTT-GAGTTTTTCAATTTTTCCCGATAAATGATTAGCTACAAAAGGATTTTTTTTT-AGTGAACGTGTCAC-GGCCGATTACTCCTTTTTTTACATTTTT-GAAATTGGCATTCTATGTCCAA----------TATCTCGATCTTAA-TCTG-AAG-TAT-GAGGGTAAGAATCAATACAAT-----AATG-ATGAATGG-AAAAAATAGAAAATCC----------TTTA-GCTAG-ATAA--------GGG-AA-GGGGC-GGATGTAG-CCAAGTGG-ATCAA-GGCAGT

Goodyera_procera_SG1152 ATGACT-CTCGGCAATGGATATCTTGGCTCTTGCATCGATGAAGAGCGCAGCGAAATGCGATACGTGGTGTGAATTGCAGAATCCCGTGAACCATCGAATTTTTGAACGCAAGTTGCGCCCGAGGCCAAT-TGGCTAAGGGCACGTCCGCCTGGGCGTCA-AGCATTACGTCGCTTCAAAAAAATTCTTTTTCTTCTTAATTTTCT------------TTTCAAATACTATCAGAAGGTTTTGGAGTCATTCTGGAAATTCCATTATCGGCGCGATTAGTATCC---------TCCCTTGAAG---AAAAAAAAATACCAAAATCTCAGAATTTACGATCTATTCATTCAATATTTCCTTTTTTAGAGGATAAATTCTCACATTTAAATTTTGTGTCAGATCTACTAATACCCCATCCCATCCATCTGGAAATCTTGGTTCAAATCCTTCAATGCTGGATCAAAGATGTTCCTTCTTTGCATTTGTTGCGATTGATTTTCCACGAATATCAT---------------AATTTGAAGAGTATC---------ATTACTT------CAAAGAAATC---------------CATTCACGTCTTTTCAAAAAG---AAAGAAAATGTTTTTTTGGTTCCTACATAATTTTTATGTATATGAATGCGAATATCTATTTCTGTTTCTTCGTAAAAAGTCTTCTTATTTACGATCAACATCTTTTGGAGTCTTTCTTGAACGAACAATTTTTTATGTAAAAATGGAA------TCTATTCTAGT---AGTGTATTTGAATTCTTTTCAGAGG-ATTCTCTGGTTCCTCAAAGATCCTTTCATACATTATGTTCGATATCAAGGAAAAGTAATTTTGGCTTCAAAGGGAACTCTTTTTCTGATGAAGAAATGGAATTTTCATGTTGTGAATTTTTGGCAATTTTATTTTCACTTTTGGTCTCAACCTTATAGGATCCATATAAAGCAATTACCCAACTATTCCTTCTCCTTTCT-GGGTTATTTTTTAAGTGTAC-CAAAAAAAACTTTGGTAGTAAGAAATCAAATGCTAGAGAATTCCTTTCTAATAAATACTCTTACTAAAAAATTAGATACCATAGCCCCAGTTATTTCTCTTATTGGATCATTGTCGAAAGCTCAATTTTGTACTATATCGGGTCATCCTATTAGTAAACCAATCTGGACCGATTTATCGGATTCTGATATTATTGATCGATTTTGTCGGAAATGTAGAAATCTTTGTCGTTATCACAGCGGATCCTCAAAAAAAAAGGTTTTGTATCGTATAAAGTATATACTTCGACTTTCGTGTGCTAGAACTTTGGCTCGTAAACATAAAAGTAC-AGTACGCACTTTTATGCGAAGATTGGGTTCGGTATTTTTAGAAGAATTGTTTA-GGAAGAAGATC------------GAGCTATCCCGACCAGTACCC-TGCATCATCCTAGCAGAGTACTTG-TATCTATGTAAAGTAAAAGAA-CTAAAAAAGAAAGTCTTAACAA-------------------ATTGGACCTAG-CCCCCTTGAATTTCCTTTAATTTCTTAG-------ATCTTTCAAAAA----------GAAGAC---TTTCTTT-----------GTAAA-TGTAAAGATAATGATATGAACTGT-GAATGATGAAATGAATTCTTCAATAAG-GGAGATTCCTTGAAC-------------------------ATATATGTTCAT-TTGTACAGGT--ATCGTA---TCTATA-----CCAAAGAAAAA---CAAAG-----AAAA--------TTGGATTGGAATTGGAACAA-----GAT------------------ACGAGGATTTCT------ATTCGGATCCATT------TGTGAAAGAACAGAG--TGAATGAAATTAGAAAG-------ATATTTCATTT--------------TGTTTGAACT------------GAACAACTGATAAAAAGAGGAT-------------------------------GAGAGTAAAGA-AAGA------------------GTGGGGAA----------TTCAAAT-----GGAC-TTTTTCTTGGGGATAGAGGACCATCTAC---------AAATGGATAATACT--------TTTGTATTCAGATGAATTCTTTAA---------------------GCCCAATATCC-AATAGATTTT-TCTAAGAAAAATCTATTGGATATT---GGGGATTCTTTTG---CTTCTTTATCCG------ATTTCTCCGAATTTTCG---------TTCTTTATCATAAAAGAAAGTTCTC-CCCCGCT---------AATGAATGATAAGT-GCC-----TAGGTG-AAGTAT----------------AGTATAAGATAAGTAAGA----------------------AAAGTCTAAGTCTTAGTATAATACCTATA-------------------------------------------CTCTTAAGGATAAGAC-----TTTTCAC----------ATGAATACTTAGTAGAACGACTAACGACGAGATTTATTATCGTTTCTCGCATGTCTCACGAAAGTGAGAGTAG-GTGCGAATTCTCCCAATTTGTGACCGACCATACGATCTGTTATATAAATGGTAAA-TGTTCCTTTCCATTATGAATAGC-GATTGTATGGCCAATCATTGTGGGTA-TAATGGTAGATGCCCGAGACCAAGTCACTATTATTTCTTTCTCCTCCCTCATGTT-GAGTTTTTCAATTTTTCCCGATAAATGATTAGCTACAAAAGGATTTTTTTTT-AGTGAACGTGTCAC-GGCCGATTACTCCTTTTTTTACATTTTG-GAAATTGGCATTCTATGTCCAA----------TATCTCGATCTTAA-TCTG-AAG-TAT-GAGGGTAAGAATCAATACAAT-----AATG-ATGAATGG-AAAAAATAGAAAATCC----------TTTA-GCTAG-ATAA--------GGA----------------------------------------

Goodyera_pusilla_KM593694 ATGACT-CTCGGCAATGGATATCTTGGCTCTTGCATCGATGAAGAGCGCAGCGAAATGCGATACGTGGTGTGAATTGCAGAATCCCGTGAACCATCGAGTTTTTGAACGCAAGTTGCGCCCGAGGCCAAT-TGGCTAAGGGCACGTCCGCCTGGGCGTCA-AGCATTACGTCGCTTC-----------------------------------------------------------------------------------------------------------------------------------------------------------------------------------------------------------------------------------------------------------------------------------------------------------------------------------------------------------------------------------------------------------------------------------------------------------------------------------------------------------------------------------------------------------------------------------------------------------------------------------------------------------------------------------------------------------------------------------------------------------------------------------------------------------------------------------------------------------------------------------------------------------------------------------------------------------------------------------------------------------------------------------------------------------------------------------------------------------------------------------------------------------------------------------------------------------------------------------------------------------------------------------------------------------------------------------------------------------------------------------------------------------------------------------------------------------------------------------------------------------------------------------------------------------------------------------------------------------------------------------------------------------------------------------------------------------------------------------------------------------------------------------------------------------------------------------------------------------------------------------------------------------------------------------------------------------------------------------------------------------------------------------------------------------------------------------------------------------------------------------------------------------------------------------------------------------------------------------------------------------------------------------------------------------------------------------------------------------------------------------------------------------------------------------------------------------------------------------------------------------------------------------------------------------------------------------------------------------------------------------------------------------------------------------------------------------------------------------------------------------------------------------------------------------------------------------------------------------------------------------------------------------------------------------------------------------------------------------------------------------------------------------------

Goodyera_seikoomontana_SG1252 ATGACT-CTCGGCAATGGATATCTTGGCTCTTGCATCGATGAAGAGCGCAGCGAAATGCGATACGTGGTGTGAATTGCAGAATTCCGTGAACCATCGAATTTTTGAACGCAAGTTGCGCCCGAGGCCAAT-TGGCTGAGGGCACGTCCGCCTGGGCGTCA-AGCATTATGTCGCTTCATAAGAATTATTTTTCTTCTCATTTTTCT------------TTTCAAATACTATCAGAAGGTTTTGGAGTAATTCTTGAAATTCCATTATCGTCGCGATTAGTATCATTAGTATCCTCCCTTGAAG---AAAAAAAAATACCAAAATCTCAAAATTTACGATCTATTCATTCAATATTTTCTTTTTTAGAGGATAAAATTTCACATTTAAATTCTGTGTCAGATCTACTAATACCCCATCCCATCCATCTGGAAATCTTGGTTCAAATCCTTCAATGCTGGATCAAAGATGTTCCTTCTTTGCATTTGTTGCGATTGATTTTCCACGAATATCAT---------------AATTTGAAAAGTATC---------ATTACTT------CAAAGAAATC---------------CATTCACGTCTTTTCAAAAAG---AAAGAAAAGATTTTTTTGGTTCCTACATAATTTTTATGTATATGAATGCGAATATCTATTTCTGTTTCTTCGTAAACAGTCTTCTTTTTTACGATCAACATCTTTTGGAGTCTTTCTTGAGCGAACACTTTTTTATGTAAAAATTGAA------TCTATTCTAGT---AGTGTATTTTAATTCTTTTCAGAGG-ATTCTCTGGTTCCTCAAAGATCCTTTCATACATTATGTTCAATATCAAGGAAAAGTAATTTTGGCTTCAAAGGGAACTCTTATTCTGATGAAGAAATGGAATTTTCATGTTGTGAATTTTTGGCAATTTTATTTTCACTTTTGGTCTCAACCTTATAGGATCCATATAAAGCAATTACCCAACTATTACTTCTCCTTTCT-GGGGTATTTTTTAAGTGTAC-AAAAAAAAACTTTGTTAGTAAGAAATCAAATGCTAGAGAATTCCTTTCTAATAAATACTCTGACTAAGAAATTAGATACCATAGCCCCAGTTATTTCTCTTATTGGATCATTGTCGAAAGCTCAATTTTGTACTATATCGGGTCATCCTATTAGTAAACCCATCTGGACCGATTTATCGGATTCTGATATTATTGATAGATTTTGTCGGAAATGTAGAAATCTTTGTCGTTATCACAACGGATCCTCAAAAAAAAAGATTTTGTATCGTATAAAGTATATACTTCGACTTTCGTGTGCTAGAACTTTGGCTCGTAAACATAAAAGTAC-AGTACGCACTTTTATGCGAAGATTGGGTTCGGTATTTTTAGAAGAATTTTTTATGGAAGAAGAACAAGCTCTTTCTTGAGCTATCCCGACCAGTACCC-TGCATCATCCTAGCAGAGTACTTG-TATCTATGTAAACGAAAAGAA-CTAAAAAAGAAAGTCTTAACAA-------------------ATTGGACCTAG-CCCCCTTTAATTT----------CTTAT-------ATC-TTCAAAAA----------GAAAAC---TTTCTTT-----------GTAAA-TGTAAAGATAATTATATGAACTGT-GAATTATTAAATGAATTCTTAAATAAG-GGAGATTCCTTGAAC-------------------------ATATATGTTCAT-TTGTGCAGGT--ATCGTA---TCTAT------ACAAAGAA------AAAAG-----AAAA--------TTGGATTGGAATTGGAAGAA-----GAT------------------ACGAGGATTTCT------ATTCGGATCCATT------TGTGAAAGAACAGAG--TGAATGAAATTAGAAAG-------ATATTTCATTT--------------TGTTTGAACT------------GAACAACTGATAAAAAGAGGAT-------------------------------GAGAGTAAAGA-AAGA------------------GTGGGGAA----------TGAAAAT-----GGGC-TTTTTCTTGGGGATAGAGGACCATCTAC---------AAATGGATAATACT--------TTTGTATTCAGATGAATTCTTTAAGGTGG--CAAT----------CCCCAATATCC-AATATTTTGT-TCTAAGAACAAAATATTGGATATT---GGGGATTCTTTTG---CTTCTTTATCTG------ATTTCTCCAAATTTTCG---------TTCTTTATCAT----AAAAGTTCTC-CCCCGCT---------AATGAATGATAAGT-GCC-----TAGGTG-AAGTAT----------------AGTATAAGATAAGTAAGA----------------------AAAGTCTAAGTCTTAGTATAATACCTATACTCTTA----------CTATAA-GAT------------AAAGACTCTTAAGGATAAGAC-----TTTTCAC----------ATGAATACTTAGTAGAACGACTAACGACGAGATTTATTATCGTTTCTCGCATGTCTCACGAAAGTGAGAGTAG-GTGCGAATTCTCCCAATTTGTGACCGACCATACGATCTGTTATATAAATGGTAAA-TGTTCCTTTCCATTATGAATAGC-GATTGTATGGCCAATCATTGTGGGTA-TAATGGTAGATGCCCGAGACCAAGTCACTATTATTTCTTTCTCCTCCCTCATGTT-GAGTTTTTCAATTTTTCCCGATAAATGATTAGCTACAAAAGGATTTTTTTTT-AGTGAACGTGTCAC-GGCCGATTACTCCTTTTTTTACATTTTT-AAAATGGGCTTTCTAGGCCCAA----------AATCCCGATTTTAA-TCGG-GAGGTGG-GGGGGTAGGAATCATCACATA------ATGGATGAGGGGGAAAAAATAGAAAATCC----------TTTTATCTAT-ATAA--------GGG-AA-GGGGCGGGATGTAC-CCAATTGG-TACAAGGGGAGG

Goodyera_viridiflora_SG1305 ATGACT-CTCGGCAATGGATATCTTGGCTCTTGCATCGATGAAGAGCGCAGCGAAATGCGATACGTGGTGTGAATTGCAGAATTCCGTGAACCATCGAATTTTTGAACGCAAGTTGCGCCCGAGGCCAAT-TGGCTAAGGGCACGTCCGCCTGGGCGTCA-AGCATTACGTCGCTTC--AAGAATTATTTTTCTTATAATTTTTCT------------TTTCAAATACTATCAGAAGGTTTTGGAGTCATTCTTGAAATTCCATTATCGTCGCGATTAGTATCC---------TCCCTTGAAG---AAAAAAAAATACCAAAATCTCAAAATTTACGATCTATTCATTCAATATTTTCTTTTTTAGAGGATAAATTTTCACATTTAAATTCTGTGTCAGATCTACTAATACCCCATCCCATCCATCTGGAAATCTTGGTTCAAATCCTTCAATGCTGGATCAAAGATGTTCCTTCTTTGCATTTGTTGCGATTGATTTTCCACGAATATCAT---------------AATTTGAAAAGTATC---------ATTACTT------CAAAGAAATC---------------CATTCACGTCTTTTCAAAAAG---AAAGAAAATATTTTTTTGGTTCCTACATAATTTTTATGTATATGAATGCGAATATCTATTTCTGTTTCTTCGTAAACAGTCTTCTTATTTACGATCAACATCTTTTGGAGTCTTTCTTGAGCGAACAATTTTTTATGTAAAAATTGAA------TCTATTCTAGT---AGTGTATTTTAATTCTTTTAAGAGG-ATTCTCTGGTTCCTCAAAGATCCTTTCATACATTATGTTCGATATCAAGGAAAAGTAATTTTGGCTTCAAAGGGAACTCTTATTCTGATGAAGAAATGGAATTTTCATGTTGTGAATTTTTGGCAATTTTATTTTCACTTTTGGTCTCAACCTTATAGGATCCATATAAAGCAATTACCCAACTATTCCTTCTCCTTTCT-GGGGTATTTTTTAAGTGTAC-AAAAAAAAACTTTGTTAGTAAGAAATCAAATGCTAGAGAATTCCTTTCTAATAAATACTCTGACTAAGAAATTAGATACCATAGCCCCAGTTATTTCTCTTATTGGATCATTGTCGAAAGCTCAATTTTGTACTATATCGGGTCATCCTATTAGTAAACCAATCTGGACCGATTTATCGGATTCTGATATTATTGATAGATTTTGTCGGAAATGTAGAAATCTTTGTCGTTATCACAACGGATCCTCAAAAAAAAAGATTTTGTATCGTATAAAGTATATACTTCGACTTTCGTGTGCTAGAACTTTGGCTCGTAAACATAAAAGTAC-AGTACGCACTTTTATGCGAAGATTGGGTTCGGTATTTTTAGAAGAATTTTTTATGGAAGAAGAAC------------GAGCTATCCCGACCAGTACTC-TGCATCATCCTAGCAGAGTACTTG-TATCTATGTAAACGAAAAGAA-CTAAAAAAGAAAGTCTTAACAA------------------------------------------------------------------------------------------------------------------------------------------------------------------------------------------------------------------------ATATGTTCCT-TTGTGCAGGT--ATCGTA---TCTAT------ACAAAGAAAAA--ATAAAA-----------------TTGGATTGGAATTGGAAGAA-----GAT------------------ACGAGGATTTCT------ATTCGGATCCATT------TGTGAAATAACAGAG--TGAATGAAATTAGAAAG-------ATATTTCATTT--------------TGTTTGAACT------------GAACAACTGATAAAAAGAGGAT-------------------------------GAGAGTAAAGA-AAGA------------------GTGGG-AA----------TG-----------------------------------CCATCTAC---------AAATGGATAATACT--------TTTGTATTCAGATGAATTCTTTAAGGTAG--CAAT----------CCCCAATATCC-AATATTTTGT-TCTTAGAACAAAATATTGGATATT---GGGGATTCTTTTG---CTTCTTTATCTG------ATTTCTCCAAATTTTCG---------TTCTTTATCATAAAAGAAAGTTCTC-CCCCGCT---------AATGAATGATAAGT-GCC-----TAGGTG-AAGTAT----------------AGTATAAGATAAGTAAGA----------------------AAAGTCTAAGTCTTAGTATAATACCTATACTCTTACTCTTA----CTATAA-GAT------------AAAGACTCTTAA--------------TTTTCAC----------ATGAATACTTAGTAGAACGACTAACGACGAGATTTATTATCGTTTCTCGCATGTCTCACGAAAGTGAGAGTAG-GTGCGAATTCTCCCAATTTGTGACCGACCATACGATCTGTTATATAAATGGTAAA-TGTTCCTTTCCATTATGAATAGC-GATTGTATGGCCAATCATTGTGGGTA-TAATGGTAGATGCCCGAGACCAAGTCACTATTATTTCTTTCTCCTCCCTCATGTT-GAGTTTTTCAATTTTTCCCGATAAATGATTAGCTACAAAAGGATTTTTTTTT-AGTGAACGTGTCAC-GGCCGATTACTCCTTTTTTTACATTTTT-TAAATTGGCATTCTATGTCCAA----------TATCTCGATCTTAA-TCTG-AAG-TAT-GAGGGTAAGAATCAATACAAT-----AATG-ATGAATGG-AAAAAAGAGAAAATCC----------TTTA-GCTAG-ATAA--------GG--AA-GGGGC-G-----------------------------

Habenaria_ciliolaris AGGACT-CTCGGCAATGGATATCTTGGCTCTTGCATCGATGAAGAGCGCAGCGAAATGCGATACGTGGTGCGAATTGCAGAATCCCGTGAACCATCGAGTTTTTGAACGCAAGTTGCGCCTGAGGCCACC-TGGCCAAGGGCACGTCCACCTGGGCGTCA-AGCATTAAATCGCTCT---------------------------------------------------------------------------------------------------------------------------------------------------------------------------------------------------------------------CTGTGTTAGATCTACTAATACCCCATCCCATCCATCTTGAAATCTTAGTTCAAATCCTTCAATGCTGGATCAAAGATGTTCCTTCTTTGCATTTGTTGCGATTGATTTTCCACGAATATCAT---------------AATTTAAATAGTTTC---------ATTACTT------CGAAGAAAGA---------------TATTTACGTCTTTTCAAAAAT---AAATAAAAGATTTTTTTTATTTTTACATAATTCTTATGTATATGAATGCGAATATATATTACTGTTTCTTCGCAAACAGTCTTCTTATTTACGATCAACGTCTTTTGAAGTCTTTCTCGAGCGAACACATTTATATAGAAAAATAGAA------TATTTTAGAGT---AATGTATTGTAATTCTTTTAAGAGG-ATTCTATGGTTCATCAAAGAACCTTTCATACATTATGTTCGATATCAAGGAAAAGCAATTCTGGCTTCAAAGGTCACTCTAATTCTGATGAATAAATGGAAATTTCATCTTGTTCATTTTTGGCAATTTTATTTTCACTTTTGGTCTCAACCTTATAGGATCCATATAAAGGAATTACCCAACTATTGCTTCTCTTTTCT-AGGGTATTTTTTAAGTGTAC-TAAAAAAGACTTTGGTAGTAAGAAATCAAATGCTGGAGAATTTATATTTAATAAATACTCTGACTAAGAAATTAGATACCATAGCCCCAGTTATTTCTCTTATTGGAGCATTGTCAAAAACTCAATTTTGTACTGTATTGGGCCATCCCATTAGCAAACCAATTTGGACTGATTTATCGGATTCTGATATTATTGATCGATTTTGTCGTATATGTAGAAATCTTTGTCATTATCACAGCGGATCCTCAAAAAAACAGGTTTTGTATCGTATAAAATATATACTTCGACTTTCGTGTGCTAGAACTTTGGCTCGTAAACATAAAAGTAC-AGTACGCACTTTTATACGAAGATTAGGTTCGGTATTCTTAGAAGAATCTTTTATGGAAGAAGAACAAGCTCTTTCTT---------------TTTTCCGTGCATCGTCCTAGCAGAGTACTTG-TATCTATGTCAATGAAAAGAA-CTAAAAAAGATAGTCTTAACAA-------------------ATTGGGCCCAG--CCCTCTTAATTT----------CTTAG-------ATC-TTCAAAAA----------GAAAAC---TTTCTTC-------------------------------------------------------------------------GATTCCTTGAAC-------------------------ATATATGTTCAT-TTGTGCAGGT--ATCGTG---TCTAT------ACAAATGAAA--------------------------TTGGATTGGAATTGT-------------------------------ACGAAAATTTCT------ATTCGGATCTGTT------TGTTAAAGAACATAG-----------------------------------------------------------------------------------------------------------------------------------------------------------------TTAGGAA----------GTAAAAT-----GGGC-TTTTTATTGGGGATAGAGGG---------------------------TACT--------GTTGTATTCATATTCATTATTGAAGATAT--AAAT---------CTCCCAATATCT-TGT------------------------------------------GTTTTG---CTTCTTTATCCGAATCCGATTTCTCCAAATTTTCG---------TTTTTTATCATAAAATAAAGTTCTCTCCCCGCC---------AATGAATGATAAGT-GTC-----TAGGTG-AAGTAT----------------AGTATAAGATAAGTAAGA----------------------AAAGTCTAAGTCTTAGTATAATACCTATACTCTTA----------CTCTAA-GAT------------AAAGACTCTTAAGGATAAGAC-----TTTTCAC----------ATGAATACTTAGTATAACGACTAACGACGAGATTTATTATCGTTTCTCGCATGTCTCACGAAAGTGAGAGTAG-GTGCGAATTCTCCCAATTTGTGACCGACCATACGATCTGTTATATAAATGGTAAA-TGTTCCTTTCCATTATGAATAGC-GATTGTATGGCCAATCATTGTGGGTA-TAATGGTAGATGCCCGAGACCAAGTCACTATTATTTCTTTCTCCTCCCTCATGTT-GAGTTTTTCAATTTTTCCCGATAAATGATTAGCTACAAAAGGATTTTTTTTT-AGTGAACGTGTCAC-GGCTGATTACTCCTTTTTTTACATTTTT-GAAATTGGCATTCTATGTCCAA----------TATCTCGATCTTAA-TCTG-AAG-TAT-GAGGGTAAGAATCAATACAAT-----AATG-ATGAATGG-AAAAAAGATAAAATCC----------TTTA-GCTAG-ATAA---------------------------------------------------

Habenaria_dentata_SG1005 AGGACT-CTCGGCAATGGATATCTTGGCTCTTGCATCGATGAAGAGCGCAGCGAAATGCGATACGTGGTGCGAATTGCAGAATCCCGTGAACCATCGAGTTTTTGAACGCAAGTTGCGCCTGAGGCCACC-TGGCCAAGGGCACGTCCACCTGGGCGTCA-AGCATTAAATCGCTCTATAAAAATTATTTTTCTTCTCATTTTTCT------------TATCAAATATTATCAGAAGGTTTTGGAGTTATTCTAGAAATTCCATTCTTGTCGCGATTAGTATCT---------TCCCTTGAAG---AAAAAAAAATAATAAAATCTCAGAATTTACGATCTATTCATTCAATCTTTCCTTTTTTAGAAGATAAATTCTTACATTTAAATTCTGTGTTAGATCTACTAATACCCCATCCCATCCATCTTGAAATCTTAGTTCAAATCCTTCAATGCTGGATCAAAGATGTTCCTTCTTTGCATTTGTTGCGATTGATTTTTCACGAATATCAT---------------AATTTAAATAGTTTC---------ATTACTT------CAAAGAAAGA---------------CATTTACGTCTTTTCAAAAAT---AAATAAAAGATTTTTTTTATTTTTACATAATTCTTATGTATATGAATGCGAATATACATTCCTGTTTCTTCGCAAACAGTCTTCTTATTTACGATCAACGTCTTTTGAAGTCTTTCTCGAGCGAACACATTTCTATAGAAAAATAGAA------TATTTTAGAGT---AATGTATTGCAATTCTTTTAAGAGG-ATTCTATGGTTCATCAAAGAACCTTTCATACATTATGTTCGATATCAAGGAAAAGCAATTCTGGCTTCAAAGGTAACTCTAATTCTTATGAATAAATGGAAATTTCATCTTGTTCATTTTTGGCAATTTTATTTTCACTTTTGGTCTCAACCTTATAGGATCCATATAAAGGAATTACCCAACTATTGCTTCTCTTTTCT-AGGGTATTTTTTAAGTGTAC-TAAAAAAGACTTTGGTAGTAAGAAATCAAATGCTGGAGAATTTATATTTAATAAATACTCTGGCTAAGAAATTAGATACCATAGCCCCAGTTATTTCTCTTATTGGAGCATTGTCAAAAACTCAATTTTGTACTGTATTGGGCCATCCCATTAGCAAACCAATTTGGACTGATTTATCGGATTCTGATATTATTGATCGATTTTGTCGTATATGTAGAAATCTTTGTCATTATCACAGCGGATCCTCAAAAAAACAGGTTTTGTATCGTATAAAATATATACTTCGACTTTCGTGTGCTAGAACTTTGGCTCGTAAACATAAAAGTAC-AGTACCCACTTTTATACGAAGATTAGGTTCGGGATTCTTAGAAGAATTTGTTATGGAAGAAGAACAAGCTCTTTCTTGAGCTATCCCGACTATTTTCCGTGCATCGTCCTAGCAGAGTACTTG-TATCTATGTCAATGAAAAGAA-CTAAAAAAGATAGTCTTAACAAATAACAA------------ATTGGGCCCAG--CCCTCTTAATTT----------CTTAG-------ATC-TTCAAAAA----------GAAAAC---TTTCTTC-------------------------------------------------------------------------GATTCCTTGAAC-------------------------ATATATGTTCAT-TTGTGCAGGT--ATCGTG---TCTAT------ACAAAT------------------GAAA--------TTGGATTGGAATTGT-------------------------------ACGAAAATTTCT------ATTCGGATCTGTT------TGTTAAAGAACATAG-----------------------------------------------------------------------------------------------------------------------------------------------------------------TGAGGAA----------GTAAAAT-----GGGC-TTTTTATTGGGGATAGAGGGCCATCTAC---------AAATGGATAGTACT--------GTTGTATTCATATGAATTATTGAAGATAT--AAAT---------CCCCCAATATCT-TGT---------------TTTAAGAACAAGATATT--GGGGGATTCTTTTG---CTTCTTTATCCGAATCCGATTTCTCCAAATTTTCG---------TTTTTTATCATAAAATAAAGTTCTCTCCCCGCC---------AATGAATGATAAGT-GTC-----TAGGTG-AAGTAT----------------AGTATAAGATAAGTAAGA----------------------AAAGTCTAAGTCTTAGTATAATACCTATACTCTTA----------CTCTAA-GAT------------AAAGACTCTTAAGGATAAGAC-----TTTTCAC----------ATGAATACTTAGTATAACGACTAACGACGAGATTTATTATCGTTTCTCGCATGTCTCACGAAAGTGAGAGTAG-GTGCGAATTCTCCCAATTTGTGACCGACCATACGATCTGTTATATAAATGGTAAA-TGTTCCTTTCCATTATGAATAGC-GATTGTATGGCCAATCATTGTGGGTA-TAATGGTAGATGCCCGAGACCAAGTCACTATTATTTCTTTCTCCTCCCTCATGTT-GAGTTTTTCAATTTTTCCCGATAAATGATTAGCTACAAAAGGATTTTTTTTT-AGTGAACGTGTCAC-GGCTGATTACTCCTTTTTTTCCATTTTT-AAAATGGGATTTTTAGGCCCAA----------AACCCGGATCTAAC-TCGG-AAG-TTTGGGGGGTAAAAACCATTCCATT------ATG-GAGGGTGGAAAAAAAGAATAAATCT----------CTTTTCTTTG-AAAG--------GGG-AA-GGGGCCGGATTGTGCCCAAGTGGAATCAA-GGGCGT

Habenaria_leptoloba_SG1304 AGGACT-CTCGGCAATGGATATCTTGGCTCTTGCATCGATGAAGAGCGCAGCGAAATGCGATACGTGGTGCGAATTGCAGAATCCCGTGAACCATCGAGTTTTTGAACGCAAGTTGCGCCTGAGGCCAGC-TGGCCAAGGGCACGTCCGCCTGGGCGTCA-AGCATTGAATCGCTACATAAAAATTATTTTTCT------------------------TCTCAAATATTATCAGAAGGTTTTGGAGTCATTCTGGAAATTCCATTCTCATCGCGATTAGTATCT---------TCCCTTGAAG---AAAAAAAAATAATAAAATCTCAAAATTTACGATCTATTCATTCAATCTTTCCTTTTTTAGAAGATAAACTCTCACATTTAAATTCTGTGTTAGATATACTAATACCCCATCCCATCCATCTGGAAATCTTAGTTCAAATCCTTCAATGCTGGATCAAAGATGTTCCTTCTTTGCATTTGTTGCGATTTATTTTTCACGAATATCAT---------------AATTTAAATAGTTTC---------ATTACTT------CAAAGAAAGA---------------CATTTACGTCTTTTCAAAAAT---AAATAAAAGATTTTTTTGGTTCTTACACAATTCTTATGTATATGAATGCGAATATATATTCCTGTTTCTTCGCAAACAGTCTTCTTATTTACGATCAACGTCTTTTGAAGTCTTTCTTGAGCGAACACATTTCTATGGAAAAATAGAA------TATTTTAGAGT---AATGTATTGTAATTCTTTTCAGAGA-ATTCTATGGTTTATCAAAGAACCTTTCATACATTATGTTCGATATCAAGGAAAAGCAATTATGGCTTCAAAGGTAACTCTAATTATGATGAAGAAATGGAAATTTCATATTGTTCATTTTTGGCAATTTTATTTTCACTTTTGGTCTCAACCTTATAGGATCCATATAAAGGAATTACCCAACTATTCCTTCTATTTTCT-GGGGTATTTTTTAAGTGTAC-TAAAAAATACTTTGGTAGTAAGAAATAAAATGCTGGAGAATTCATTTTTAATAAATACTCTGTCTAATAAATTAGATACCATAGCCCCAGCTATTTCTCTTATTGGAACATTATCAAAAACTCAATTTTGTACTGTATTGGGCCATCCCATTAGTAAACCGATCTGGACTGATTTATCGGATTCTGATATTCTTGATCGATTTTGTCGTATATGTAGAAATCTTTGTCGTTATCACAGTGGATCCTCAAAAAAAAAGGTTTTGTATCGTATAAAATATATACTTCGACTTTCGTGTGCTAGAACTTTGGCTCGTAAACATAAAAGTAC-AGTACGCACTTTTATACGAAGATTAGGTTCGGGATTCTTAGAAGAATTCTTTATGGAA-------------------GAGCTACCCCGACCATTTTCCGTGCATCATCCTAGCAGAGTA------------GTCAATGAAAAGAA-CTAAAAAAGACAGTCTTAACAA-------------------ATTGGGCCTAG--CCCTCTTAATTT----------CTTAG-------ATC-TTCAAAAA----------GAAGAC---TTTATTT-------------------------------------------------------------------------TATTCCTTGAAC-------------------------ATATATGTTCAT-TTGTGCAGGT--ATCGTA---TCTAT------ACAAAT------------------TAAA--------TTGGATTGCAATTGGAAGAA-----TAT------------------ACGAAAATTTCT------ATTCAGATCCATT------TGTTAAAGAACAGA----------------------------------------------------------------------------------------------------------------------------------------------------------------------------------------------------------------------------CTAC---------AAATGGATAGTACT--------GTTGTATTCATATGAATTATTGAAGATAT--AAAT---------ACCCCAATATCT-TGT---------------TCTTAGAACAAGATATT--GGGGTATTCTTTTG---CTTCTTTATCCGAATCCAATTTCTCCAAATTTTCG---------TTTTTTATCATAAAATAAAGTTCTC-CCCCGCC---------AATGAATGATAAGT-GTC-----TAGGTG-AAGTAT----------------AGTATAAGATAAGTAAGA----------------------AAAGTCTAAGTCTTAGTATAATACCTATACTCTTA----------CTCTAA-GAT------------AAAGACTCTTAAGGATAAGAC-----TTTTCGC----------ATGAATACTTAGTAGAACGACTAACGACGAGATTTATTATCGTTTCTCGCATGTCTCACGAAAGTGAGAGTAG-GTGCGAATTCTCCCAATTTGTGACCGACCATACGATCTGTTATATAAATGGTAAA-TGTTCCTTTCCATTATGAATAGC-GATTGTATGGCCAATCATTGTGGGTA-TAATGGTAGATGCCCGAGACCAAGTCACTATTATTTCTTTCTCCTCCCTCATGTT-GAGTTTTTCAATTTTTCCCGATAAATGATTAGCTACAAAAGGATTTTTTTTT-AGTGAACGTGTCAC-GGCCGATTACTCCTTTTTTTACATTTTT-GAAATTGGCATTCTATGTCCAA----------TATCTCGATCTTAA-TCTG-AAG-TAT-GAGGGTAAGAATCAATACAAT-----AATG-ATGAATGG-AAAAAATAGAAAATCC----------TTTA-GCTAG-ATAA--------GG--AA-GGGGC-------------------------------

Habenaria_linguella_SG1195 AGGACT-CTCGGCAATGGATATCTTGGCTCTTGCATCGATGAAGAGCGCAGCGAAATGCGATACGTGGTGCGAATTGCAGAATCCCGTGAACCATCGAGTTTTTGAACGCAAGTTGCGCCCGAGGCCACC-TGGCCAAGGGCACGTCCACCTGGGCGTCA-AGCATTAAATCGCTCT-----------------------------------------------ATATTATCAGAAGGTTTTGGAGTTATTCTAGAAATTCCATTATTGTCGCGATTAGTATCT---------TCCCTTGAAG---AAAAAAAAATAATAAAATCTCAGAATTTACGATCTATTCATTCAATCTTTCCTTTTTTAGAAGATAAATTCTTACATTTAAATTCTGTGTTAGATCTACTAATACCCCATCCCATCCATCTTGAAATCTTAGTTCAAATCCTTCAATGCTGGATCAAAGATGTTCCTTCTTTGCATTTGTTGCGATTGATTTTCCACGAATATCAT---------------AATTTAAATAGTTTC---------ATTACTT------CAAAGAAAGA---------------CATTTACGTCTTTTCAAAAAT---AAATAAAAGATTTTTTTTATTTTTACATAATTCTTATGTATATGAATGCGAATATACATTCCTGTTTCTTCGCAAACAGTCTTCTTATTTACGATCAACGTCTTTTGAAGTCTTTCTCGAGCGAACACATTTCTATAAAAAAATAGAA------TATTTTAGAGT---AATGTATTGTAATTCTTTTAAGAGG-ATTCTATGGTTCATCAAAGAACCTTTCATACATTATGTTCGATATCAAGGAAAAGCAATTCTGGCTTCAAAGGTAACTCTAATTCTGATGAATAAATGGAAATTTCATCTTGTTCATTTTTGGCAATTTTATTTTCACTTTTGGTCTCAACCTTATAGGATCCATATAAAGGAATTACCCAACTATTGCTTCTCTTTTCT-AGGGTATTTTTTAAGTGTAC-TAAAAAAGACTTTGGTAGTAAGAAATCAAATGCTGGAGAATTTATATTTAATAAATACTCTGGCTAAGAAATTAGATACCATAGCCCCAGTTATTTCTCTTATTGGAGCATTGTCAAAAACTCAATTTTGTACTGTATTGGGCCATCCCATTAGCAAACCAATTTGGACTGATTTATCGGATTCTGATATTATTGATCGATTTTGTCGTATATGTAGAAATCTTTGTCATTATCACAGCGGATCCTCAAAAAAACAGGTTTTGTATCGTATAAAATATATACTTCGACTTTCGTGTGCTAGAACTTTGGCTCGTAAACATAAAAGTAC-AGTACGCACTTTTATACGAAGATTAGGTTCGGGATTCATAGAAGAA-------------------------------GAGCTATCCCGACTATTTTCCGTGCATCGTCCTAGCAGAGTACTTA-TATCTA---------------------------------------------------------------------------------------------------------------------------------------------------------------------------------------------------------------------------------------------------------------TATGTTCAT-TTGTGCAGGT--ATCGTG---TCTAT------ACAAAT------------------GAAA--------TTGGATTGGAATTGT-------------------------------ACGAAAATTTAT------ATTCGGATCTGTT------TGTTAAAGAACA---------------------------------------------------------------------------------------------------------------------------------------AAGAA-CATA------------------GTGAGGAA----------GTAAAAT-----GGGC-TTTTTATTGGGGATAGAGGGCCATCTAC---------AAATGGATAGTACT--------GTTGTATTCATATGAATTATTGAAGATAT--AAAT---------CCCCCAATATCT-TGT---------------TTTAATAACAAGATATT--GGGGGATTCTTTTG---CTTCTTTATCCGAATCCGATTTCTCCAAATTTTCG---------TTTTTTATCATAAAATAAAGTTCTCTCCCCGCC---------AATGAATGATAAGT-GTC-----TAGGTG-AAGTAT----------------AGTATAAGATAAGTAAGA----------------------AAAGTCTAAGTCTTAGTATAATACCTATACTCTTA----------CTCTAA-GAT------------AAAGACTCTTAAGGATAAGAC-----TTTTCAC----------ATGAATACTTAGTATAACGACTAACGACGAGATTTATTATCGTTTCTCGCATGTCTCACGAAAGTGAGAGTAG-GTGCGAATTCTCCCAATTTGTGACCGACCATACGATCTGTTATATAAATGGTAAA-TGTTCCTTTCCATTATGAATAGC-GATTGTATGGCCAATCATTGTGGGTA-TAATGGTAGATGCCCGAGACCAAGTCACTATTATTTCTTTCTCCTCCCTCATGTT-GAGTTTTTCAATTTTTCCCGATAAATGATTAGCTACAAAAGGATTTTTTTTT-AGTGAACGTGTCAC-GGCTGATTACTCCTTTTTTTACCTTTTT-AAAATGGGGATTTTTTGGCCAA----------AATCCCCGTTTTAT-TCGG-AAA---TGGGGGGTAAAAATTCCTTCCAT-------TG-GGGAAAGG-GAAAAAAAAAAAACCT----------TTTC---ATA-AAAA--------TGGGAG-GGGGG-GGATTTC--CCAATTTGACCCGGGGGGGGT

Habenaria_reniformis_SG1296 AGGACT-CTCGGCAATGGATATCTTGGCTCTTGCATCGATGAAGAGCGCAGCGAAATGCGATACGTGGTGCGAATTGCAGAATCCCGTGAACCATCGAGTTTTTGAACGCAAGTTGCGCCTGAGGCCACC-TGGCCAAGGGCACGTCCACCTGGGCGTCA-AGCATTAAATCGCT--ATAAAAATTATTTTTCTTCTCATTTTTCT------------TATCAAATATTATCAGAAGGTTTTGGAGTTATTCTGGAAATTCCATTCTTGTCGCGATTAGTATCT---------TCCCTTGAAG---AAAAAAAAATAATAAAATCTCAGAATTTACGATCTCTTCATTCAATCTTTCCTTTTTTAGAAGATAAATTCTTACATTTAAATTATGTGTTAGATCTACTAATACCCCATCCCATCCATCTTGAAATCTTAGTTCAAATCCTTCAATGCTGGATCAAAGATGTTCCTTCTTTGCATTTGTTGCGATTGATTTTCCACGAATATCAT---------------AATTTAAATAGTTTC---------ATTACTT------CAAAGAAAGA---------------CATTTACGTCTTTTCAAAAAT---AAATAAAAGATTTTTTTTATTTTTACATAATTCTTATGTATATGAATGCGAATATACATTCCTGTTTCTTCGCAAACAGTCTTCTTATTTACGATCAACGTCTTTTGAAGTCTTTCTCGAGCGAACACATTTCTATATAAAAATAGAA------TATTTTAGAGT---AATGTATTGTAATTCTTTTAAGAGG-ATTCTATGGTTCATCAAAGAACCTTTTATACATTATGTTCGATATCAAGGAAAAGCAATTCTGGCTTCAAAGGTAACTCTAATTATGATGAATAAATGGAAATTTCATCTTGTTTATTTTTGGCAATTTTATTTTCACTTTTGGTCTCAACCTTATAGGATCCATATAAAGGAATTACCCAACTATTGCTTCTCTTTTCT-AGGGTATTTTTTAAGTGTAC-TAAAAAATACTTTGGTAGTAAGAAATCAAATGCTGGATAATTTATATTTAATAAATACTCTGGCTAATAAATTAGATACCATAGCCCCAGTTATTTCTCTTATTGGAGCATTGTCAAAAACTCAATTTTGTACTGTATTGGGCCATCCCATTAGCAAAGCAATTTGGACTGATTTATCGGATTCTGATATTATTGATCGATTTTGTCGTATATGTAGAAATCTTTGTCATTATCATAGCGGATCCTCAAAAAAACAGGTTTTGTATCGTATAAAATATATACTTCGACTTTCGTGTGCTAGAACTTTGGCTCGTAAACATAAAAGTAC-AGTACGCACTTTTATACGAAGATTAGGTTCGGGATTCTTAGAAGAATTTTTTATGGAAGAAGAACAAGCTCTTTCTTGAGCTATCCCGACTATTTTCCGTGCATCGTCCTAGCAGAGTACTTG-TATCTATGTCAATGAAAAGAA-CTAAAAAAGATAGTCTTAACAA-------------------ATTGGGTCCAG--CCCTCTTAATTT----------ATTAG-------ATC-TTCAAAAA----------GAAAAC---TTTCTTC-------------------------------------------------------------------------GATTCCTTGAAC-------------------------ATATATGTTCAT-TTGTGCAGGT--ATCGTG---TCTAT------ACAAAT------------------GAAA--------TTGGATTGGAATTGT-------------------------------ACGAAAATTTCT------ATTCGGATCTGTT------TGTTAAAGAACATAT----------------------------------------------------------------------------------------------------------------------------------------------------------------GTGAGGCC--------AAGTTAAA-------------------------------CCATCTAC---------AAATGGATAGTACT--------GTTGTATTCATATGAATTATTGAAGATAT--AAAT---------CCCCCAATATCT-TGT---------------TTTAAGAACAAGATATT--GGGGGATTCTTTTG---CTTCTTTATCCGAATCCGATTTCTCCAAATTTTCG---------TTTTTTATCATAAAATAAAGTTCTCTCCGCGCC---------AATGAATGATAAGT-GTC-----TAGGTG-AAGTAT----------------AGTATAAGATAAGTAAGA----------------------AAAGTCTAAGTCTTAGTATAATACCTATACTCTTA----------CTCTAA-GAT------------AAAGACTCTTAAGGATAAGAC-----TTTTCAC----------ATGAATACTTAGTATAACGACTAACGACGAGATTTATTATCGTTTCTCGCATGTCTCACGAAAGTGAGAGTAG-GTGCGAATTCTCCCAATTTGTGACCGACCATACGATCTGTTATATAAATGGTAAA-TGTTCCTTTCCATTATGAATAGC-GATTGTATGGCCAATCATTGTGGGTA-TAATGGTAGATGCCCGAGACCAAGTCACTATTATTTCTTTCTCCTCCCTCATGTT-GAGTTTTTCAATTTTTCCCGATAAATGATTAGCTACAAAAGGATTTTTTTTT-AGTGAACGTGTCAC-GGCTGATTACTCCTTTTTTTACATTTTT-GAAATTGGCATTCTATGTCCAA----------TATCTCGATCTTAA-TCTG-AAG-TAT-GAGGGTAAGAATCAATACAAT-----AATG-ATGAATGG-AAAAAAGATAAAATCC----------TTTA-GCTAG-ATAA--------GGN-AA-GGGGC-GGATGTAG-CCANGTGG-ATCAN-GGCAGT

Habenaria_rhodocheila_SG1289 AGGACT-CTCGGCAATGGATATCTTGGCTCTTGCATCGATGAAGAGCGCAGCGAAATGCGATACGTGGTGCGAATTGCAGAATCCCGTGAACCATCGAGTTTTTGAACGCAAGTTGCGCCTGAGGCCACC-TGGCCAAGGGCACGTCCACCTGGGCGTCA-AGCATTAAATCGCT----AAAAATTATTTTTCTTCTCATTTTTCT------------TATCAAATATTATCAGAAGGTTTTGGAGTCATTCTGGAAATTCCATTCTTGTCGCGATTAGTATCT---------TCCCTTGAAG---AAAAAAAAATAATAAAATCTCAAAATTTACGATCTATTCATTCAATCTTTCCTTTTTTAGAAGATAAATTCTTGCATTTAAATTCTGTGTTAGATCTACTAATACCCCATCCCATCCATCTTGAAATCTTAGTTCGAATCCTTCAATACTGGATCAAAGATGTTCCTTCTTTGCATTTGTTGCGATTGATTTTCCACGAATATCAT---------------AATTTAAATAGTTTT---------ATTACTT------CAAAGAAAGA---------------CATTTACGTCTTTTCAAAAAT---AAAAAAAAGATTTTTTTTATTTTTACATAATTCTTATGTATATGAATGCGAATATATATTCCTGTTTCTTCGCGAACAGTCTTCTTATTTACGATCAACGTCTTTTGAAGTCTTTCTTGAGCGAACACATTTCTATAGAAAAATAGAA------TATTTTAGAGT---AATATATTGTAATTCTTTTAAGAGG-ATTCTATGGTTTATCAAAGAACCTTTCATACATTATGTTCGATATCAAGGAAAAGCAATTCTGGCTTCAAAGCTAACTCTAATTCTGATGAATAAATGGAAATTTTATCTTGTTCATTTTTGGCAATTTTATTTTCACTTTTGGTCTCAACCTTATAGGATCCTTATAAAGGAATTACCCAACTATTGCTTCTTTTTTCT-AGGGTATTTTTTAAGTGTAC-TAAAAAAGACTTTGGTAGTAAGAAATAAAATGCTGGAGAATTTATATTTAATAAATACTCTGGTTAATAAATTAGATACCATAGCCCCAGTTATTTTTCTTATTGGAGCATTGTCAAAAACTCAATTTTGTACTGTATTGGGCCATCCCATTAGCAAACGAATTTGGACTGATTTATCGGATTCTGATATTATTGATCGATTTTGTCGTATATGTAGAAATCTTTGTCATTATCACAGCGGATCCTCAAAAAAACAGGTTTTGTATCGTATAAAGTATATACTTCGACTTTCGTGTGCGAGAACTTTGGCTCGTAAACATAAAAGTAC-AGTACGCACTTTTATACGAACATTAGGTTCGGGATTCTTAGAAGAATTTTTTATGGAAG------------------GAGCTATCCCGACTATTTTCCGTGCATCGTCCTAGCAGAGTACTTG-TATCTATGTCAATGAAAAGAA-CTAAAAAAGATAGTCTTAACAA-------------------ATTGGGCCTAG--CCCTCTTAATTT----------CTTAG-------ATC-TTCAAAAA----------GCAGGT-------------------------------------------------------------------------------------------------------------------------------------------------ATCGTA---TCTAT------ACAAAT------------------AAAA--------TTGGATTGGAATTGT-------------------------------ACGAAAATTTCT------ATTTGGATCCATT------TGTTAAAGAACATA-----------------------------------------------------------------------------------------------------------------------------------------------------------------GTGAGAAG----------TCAAAAT-----TGGTT--------------------CCATCTAC---------AAATGGATAGTACT--------GTTGTATTCATATGAATTATTGAATATAT--AAAT---------CCCCCAATATCT-TGT---------------TTTAAGAATATC-------------TT-TTTTA-----------------------------------------------------------AGAACAAGCTATT-GGGGG-----ATTTAGTTTGAATGATAAGT-GTC-----TAGGTG-AAGTAT----------------AGTATAAGATAAGTAAGA----------------------AAAGTCTAAGT---------ATACCTATACTCTTA----------CTCTAA-GAT------------AAAGACTCTTAAGGATAAGAC-----TTTTCAC----------ATGAATACTTAGTATAACGACTAACGACGAGATTTATTATCGTTTCTCGCATGTCTCACGAAAGTGAGAGTAG-GTGCGAATTCTCCCAATTTGTGACCGACCATACGATCTGTTATATAAATGGTAAA-TGTTCCTTTCCATTATGAATAGC-GATTGTATGGCCAATCATTGTGGGTA-TAATGGTAGATGCCCGAGACCAAGTCACTATTATTTCTTTCTCCTCCCTCATGTT-GAGTTTTTCAATTTTTCCCGATAAATGATTAGCTACAAAAGGATTTTTTTTT-AGTGAACGTGTCAC-GGCTGATTACTCCTTTTTTTACATTTTT-GAAATTGGCATTCTATGTCCAA----------TATCTCGATCTTAA-TCTG-AAG-TAT-GAGGGTAAGAATCAATACAAT-----AATG-ATGAATGG-AAAAAAGATAAAATCC----------TTTA-GCTAG-ATAA--------GGG-AA-GGGGC-------------------------------

Hetaeria_youngsayei_SG1244 ATGACT-CTCGGCAATGGATATCTTGGCTCTTGCATCGATGAAGAGCGCAGCGAAATGCGATACGTGGTGTGAATTGCAGAATCCCGTGAACCATCAAATCTTTGAACGCAAGTTGCGCCCGAGGCCAAT-TGGCTAAGGGCACGTCCGCCTGGGCGTCA-AGCATTACATCGCTTCATAAGAATTCTTTTTCTTCTCCTTTTTAT------------TTTCAAATACTATCAGAAGGTTTTGGAGTCGTTCTGGAAATTTCATTATCGTCGCGATTAGTATCC---------TCCCTTGAAG---AAAAAAAAATACCAAAATCTCAGAATTTACGATCTATTCATTCAATATTTCCTTTTTTAGAGGATAAAATCTCACATTTAAATTCTGTGTCAGATCTACTAATACCCCATCCCATCCATCTGGAAATCTTGGTTCAAATTCTTCAATGCTGGATCAAAGATGTTCCTTCTTTGCATTTGTTGCGATTGATTTTCCACGAATATCAT---------------AATTTGAAGAGTATC---------ATTACTT------CAAAGAAATC---------------CATTCACGTCTTTTCAAAAAG---AAAGAAAAGATTTTTTTGGTTCCTACATAATTTTTATGTATATGAATGTGAATATCTCTTTTTTTTTCTTCGTAAAAAGTCTTCTTATTTACGATCAACATCTTTTGGATTCTTTATTGAGCGAACACTTTTTTATGTAAAAATGGAA------TCTATTCTAGT---AGTGTATTTGAATTCTTTTCAGAGG-ATTCTCTGGTTCCTCAAAGATCCTTTCATACATTATGTTCGATATCAAGGAAAAGTAATTCTGGCTTCAAAGGGAACTCTTATTCTGATGAAGAAATGGAATTTTTATGTTGTGAATTTTTGGCAATTTTATTTTCACTTTTGGTCTCAACCTTATAGGATCCGTATAAAGCAATTATCCAACTATTCCTTCCCCTTTCT-GGGGTATTTTTTAAGTGTAC-AAAAAAAAACTTTGGTAGTAAGAAATCAAATGCTAGAGAATTCCTTTCTAATAAATACTCTGACTAAGAAATTAGATACCATAGCCCCAGTTATTTCTCTTATTGGATCATTGTCGAAAGCTCAATTTTGTACTATATCGGGTCATCCTATTAGTAAACCAATCTGGACCGATTTATCGGATTCTGATATTATTGATCGATTTTGTCGGAAATGTAGAAATCTTTGTCGTTATCACAGCGGATCCTCAAAAAAAAAAGTTTTGTATCGTATAAAATATATACTTCGACTTTCGTGTGCTAGAACTTTGGCTCGTAAACATAAAAGTAC-AGTACGCACTTTTATGCGAAGATTGGGTTCGGTATTTTTAGAAGAATTTTTTATGGAAGAAGAACAAGCTCTTTCTTGAGCTATCCCGACCAGTACTC-TGCATCATCCTAGCAGAGTACTTG-TATCTATGTAAACGAAAAGGA-CTAAAAAAGAAAGTCTTAACAA-------------------ATTGGACCTAG-CCCCCTTTAATTT----------CTTAG-------ATC-TTTAAAAA----------GAAGAC---TTTCTTT-----------GTAAA-TGTAAAGATAATGATATGAACTGT-GAATAATTAAATAAATTATTCAATAAG-GGAGATTCTTTGAAA-------------------------ATATATGTTCAT-TTGTGCAGGT--ATCGTA---TCTAT------ACAAAGAAAAA---CAAAG-----AAAA--------TTGGATTGGAATTGGAAGAA-----GAT------------------AGGAGGATTTCT------ATTTGGATCCATT------TGTGAAAGAACAGAG--TGAATGAAATTAGAAAG-------ATATTTCATTT--------------TGTTTAAACT------------GAACAACTGATAAAAAGAGGAT-------------------------------GAGAGTAAAGA-AAGA------------------GTGAGGAA----------TTCAAAT-----GGGC-TTTTTCTTGGGGATAGAGGGCCATCTAC---------AAATGGATAATACT--------TTTGTATTTAGATGAATTCTTTAAGGTAG--TAAT----------CCCCAATATC----------------------------CAATATATT---GGGGATT-TTTTG---CTTCTTTATCCG------ATTTCTCCGAATTTTCG---------TTCTTTATCATAAAAGAAAGTTCTC-CCCCGCC---------AATGAATGATAAGT-GCC-----TAGGTG-AAGTAT----------------AGTATAAGATAAGTAAGA----------------------AAAATCTAAGTCTTAGTATAATACCTATACTCTTA----------CTATAA-GAT------------AAAGACTCTTAAGGATAAGAC-----TTTTCAC----------ATGAATACTTAGTAGAACGACTAACGACGAGATTTATTATCATTTCTCGCATGTCTCACGAAAGTGAGAGTAG-GTGCGAATTCTCCCAATTTGTGACCTACCATACGATCTGTTATATAAATGGTAAA-TGTTCCTTTCCATTATGAATAGC-GATTGTATGGCCAATCATTGTGGGTA-TAATGGTAGATGCCCGAGACCAAGTCACTATTATTTCTTTCTCCTCCCTCATGTT-GAGTTTTTCAATTTTTACCGATAAATGATTAGCTACAAAAGGATTTTTTTTT-AGTGAACGTGTCAC-GGCCGATTACTCCTTTTTTTACATTTTT-GAAATTGGCATTCTATGTCCAA----------TATCTCGATCTTAA-TCTG-AAG-TAT-GAGGGTAAGAATCAATACAAT-----AATG-ATGAATGG-AAAAAATAGAAAATAC----------TTTA-GCTAG-ATAA--------GGG-AA-GGGGC-GGATGTAG-CCAAGTGG-ATCAA-GGCAGT

Hypoxis_rigidula_SG1207 -------------------------------------------------------------------------------------------------------------------------------------------------------------------------------------------------------------------------------------------------------------------------------------------------------------------------------------------------------------------------------------------------------------------------------------------------------------------------------------------------------------------------------------------------------------------------------------------------------------------------------------------------------------------------------------------------------------------------------------------------------------------------------------------------------------------------------------------------------------------------------------------------------------------------------------------------------------------------------------------------------------------------------------------------------------------------------------------------------------------------------------------------------------------------------------------------------------------------------------------------------------------------------------------------------------------------------------------------------------------------------------------------------------------------------------------------------------------------------------------GAGCTATCCCGACCATCTACCGTACATCATCTTAGCAGATGACTTG-TATCTATGTCAATTAAGGGTA-CTAAAAAATATTCAAAAA------------------------TTTGACCTAG---TTCCTGAATTT----------CTTAA-------CTA-TAAAAAAAAAA-------GAAGAC---ATTCTTTGTATCTTT---GTAAA-CGTAAAGATAATGATATGTATTGT-GAATGA------------TTCAATAAT-AGTGATTCCTTGCCC-------------------------ATATATGTTCAT-TTGTGCGGGT--ATCATA---TCTAT------CCAAAT------------------------------TTGGAT---------AAAAT--------------------------CAAAAGATTTCT------GTTCGGATCCATT------TGTGAAAAAATAGAG--TGAAT-----GAGAAAG-------ATATTGAATTT--------------TGTTTGAACC----------------ATCT-AAG---AAAAAAA-------------------------------GAGGATAAATA-----------------------GTTAGGAA----------GTAAAAT-----GGGCTTTTTTATTGGGGATAGAGGGCCATCTAC---------AAACGGATAAGACT--------TCCGTATTAATACGAAT-CGTTGAAGGAA--CAAT---------ACCCCA-TATCT-TGT------------------TTTAGCAAGATAT---GGGGTATT------------------------------GCTCCTTGTTT----------------------------------------------------------------------------------------------------------------------------------------------------------------------------------------------------------------------------------------------------------------GATTCTTTTTTGAATTTTAATTAACGACGAGATTTATTATCGTTTCTCGCATGTCTCGCGAAAGTCAGAGTAG-GCGCAAATTCTCCCAATTTGTGACCCACCATACGATCTGTTATATAAATGGTAAA-TGTTCCTTTCCATTATGAATAGC-GATTGTATGGCCAATCATTGTGGGTA-TAATGGTAGATGCCCGAGACCAAGTTACTATTATTTCTTTCTCCTCCCTCATGTT-GAGTTTTTCAATTTTTCCCGATAAATGATTAGCTACAAAAGGATTTTTTTTT-AGTGAACGTGTCAC-AGCGGATTACTCCTTTTTTTACATTTTA-AAGATTGGCATTCTATGCCCAA----------TATCTCGATCTAAG-TATG-AAGGTAA---------GAATAAATACAAT-----AATG-ATGAATGG-AAAAAAGAGAAAATCC----------TTTA-GCTAG-ATAA---------------GGGGC-GGATGTAG-CCAAGTGG-ATCAA-GGCAGT

Lecanorchis_nigricans_SG1280 AAGACT-CTCGGCAACGGATATCTTGGCTCTTGCATCGATGAAGAACGCAGCGAAATGCGATATGTGTTGTGAATTGCAGAATCCCGTGAACCATCCAGTCTTTGAACGCAAGTCGCGCCCAAGGTT-GC-ACGCCGAGGGCACGTCTGCATGGGTGTGA-TGCGTTAAGTCGCTCT----------------------------------------------------------------------------------------------------------------------------------------------------------------------------------------------------------------------------------------------------------------------------------------------------------------------------------------------------------------------------------------------------------------------------------------------------------------------------------------------------------------------------------------------------------------------------------------------------------------------------------------------------------------------------------------------------------------------------------------------------------------------------------------------------------------------------------------------------------------------------------------------------------------------------------------------------------------------------------------------------------------------------------------------------------------------------------------------------------------------------------------------------------------------------------------------------------------------------------------------------------------------------------------------------------GAGCTATCCTTGCCATTCCCTACGTATCATACTAATACTATACTTG-TATCTATATCAGTGAAGAGAA-CTAAAAAGGACATAAAAGTAAAAAC-------------------------------CCTTTAATTT----------CTTAG-------ATA-TTCAAAC--------------------TTTATTT-----------GTGAAAATTAAGGA-----TCACGGTCCAT-GAATGA------------TTCACTAAT-AGAGATTA------------------------------------ATATTCAT-TTGCGCAGGT--ATCATA-----TTT------ACACAT------------------TAAA--------TTTGATTGGAA------GAT-GTAAGAC------------------ATGAGAGTTTCTTTTTCTATTAGGATCAATTTGGGAGTGTGAAAGAACAAAG--TGGATTAAATGAGAGGG-------GCATTGAATTT----------TTTGGAGTTGAACC-----------------GCTGATA---AAAAAAA-------------------------------GTAGATAAATC-AATA------------------GTGAGAAT----AAGGAAGTAAAAT------GGC-TTTTTATTGGGGATAGAGGG----------------------------------------------------------------------------------------------------------------------------------------------------------------------------------------------------------------------------------------------------------------------------------------------------------------------------------------------------------------------------------------------------------------------------------------------------------------------------------------------------------------------------------------------------------------------------------------------------------------------------------------------------------------------------------------------------------------------------------------------------------------------------------------------------------------------------------------------------------------------------------------------------------------------------------------------------------------------------------------------------

Liparis_bootanensis_SG1215 ACGACT-CTCGGCAATGGATATCTCGGCTCTTGCATCGATGAAGAGCGCAGCGAAATGCGATACGTGGTGCGAATTGCAGAATCCCGCGAACCATCGAGTCTTTGAACGCAAGTTGCGCCCGAGGCCAAC-CGGCCGAGGGCACGTTTGCCTGGGCGTCA-AGCGTTGCGTCGCTTCACAAGAATTCTTTTTCTTCTCATTTTTCTTCTCAAATGGTATCTCAAATGGTATCAGAAGGTTTTGGAGTCATTCTGGAAATTCCATTCTCGTCGCAATTAGTATTT---------TCCCTTGAAG---AAAAAAGAATACCAAAATATCAGAATTTACGATCTATTCATTCAATATTTCCCTTTTTAGAGGATAAATTATCACATTTAAATTATGTGTCAGATCTACTAATACCCCATCCCATCCATCTGGAAATCTTGGTTCAAATTCTTCAATGTTGGATCAAAGATGTTCCTTCTTTGCATTTATTGCGATTGTTTTTCCACGAATATCAT---------------AATTTGACTAGTCTC---------ATTACTT------CAAATAAATC---------------CATTTACGTCTTTTCAAAAAG---AAATAAAAGATTCTTTTGGTTCCTACATAATTCTTATGTATATGAATACGAATATCTATTCCTGTCTCTTCATAAACGGTCTTCTTATTTACGATCAATATCTTCTGGAGTCTTTCTTGAGCGAACACATTTCTATGGAAAAATAGAA------TATCTTATAGT---CGTGTGTTGTAATTCTTTTCAGAGG-ATCCTATGGTTCCTCAAAGATACTTTCATACATTATGTTCGATATCAAGGAAAAGCGATTCTGGCTTCAAAAGGAACTCTTATTCTGATGAAGAAATGGAAATTTTATTTTGTGAATCTTTGGCAATCTTATTTTCACTTTTGGTTTCAACCTTATAGGATTCATATAAAGCAATTACCCAACTATTCCTTCTCTTTTCT-GGGGTATTTTTCAAGTGTAC-TAAAAAATCCTTTGGTAGTAAGAAATCAAATGCTAGATAATTCATTTCTAATAAATACTCTGACTAAGAAATTAGATACCATAGTCCCAGTTATTTCTCTTATTGGATCATTGTCGAAAGCTCAATTTTGTACTGTATTGGGTCATCCTATTAGTAAACCAATCTGGACCGATTTATCGGATTCTGATATTCTTGATCTATTTTGTCGGATATGTAGAAATCTTTGTCGTTATCACAGCGGATCCTCAAAGAAACAAGTTTTGTATCGTATAAAGTATATACTTCGACTTTCGTGTGCTAGAACTTTGGCTCGTAAACATAAGAGTAC-AGTACGCACTTTTATGCGAAGGTTAGGTTCGGGATTCTTAGAAGAATTTTTTTTGGAAGAAGAACAATCTCTTTCTTGAGCTATCCCGACCATTTACCATACATCATCCTAGCAGAGTACTTA-TATCTATGTCAACGAAAAGAA-CTAAAAAATAAAAA---------------------------TTTGGACCTAG--CCCCT-GAATTT----------CTTAG-------ATC-TTCAAAAA----------TAAGAC---TTTTTTTGTAAATGTAAGGTAAA-TGTAAGGAAAAAGATATGGACTAT-TAATGA------------TTCAATAAC-GGAGATTCCTTGAAC-------------------------ATATATGTTCAT-------------ATCGTA----CTAT------ACAAAA--------CAAAG-----GAGA--------TTGGATTGGAA------GAA-----GAT------------------ACGAGGATTTAT------ATTCGTATACATT------TGTGAAAGAACAGAG--TGAATGAATTGAGAAAG-------ATATTTAATTT--------------TGTTTAAACTGAGCCA------CTGATACTGATGAGAAA------------------------------------GAGGATAAAGA-AAGA------------------GCGAGGAA-----GGGAAGTAAAAT-----GGGC-TTTTTATTGGGGATAGAGGGCCATCTAT---------AAATGGATAATACT--------TTTGTATGTATATGAATTTTTGAAGATAG--CAAT---------CCCCCAATATCT-TGT---------------TCTTAGAACAAGATATT--GGGGGATTCTTTTG---CTTCTCTATCCG---------------AATTTTCG---------TTCTTTATCAT----AAAAGTTCTC-CCCCGCC---------AATGAATGATAAGT-GCC-----TAGGTG-AAGTAT----------------AGTATAAGATAAGTCAGA----------------------AAAGTCTAAGTCTTAGTATAATACCTATACTCTTA----------CTATAA-GAT------------AAAGACTCTTAACGATAAGGC-----TTTTCAC----------ATGAATACTTAGTAGAACGACTAACGACGAGATTTATTATCGTTTCTCGCGTGTCTCACGAAAGTGAGAGTAG-GTGCGAATTCTCCCAATTTGTGACCGACCATACGATCTGTGATATAAATGGTAAA-TGTTCCTTTCCATTATGAATAGC-GATTGTATGGCCAATCATTGTGGGTA-TAATGGTAGATGCCCGAGACCAAGTCACTATGATTTCTTTCTCCTCCCTCCTGTT-GAGTTTTTCAATTCTTCCCGATAAATGATTAGCTACAAAAGGATTTTTTTTT-AGTGAACGTGTCAC-GGCTGATTACTCCTTTTTTTACATTTTT-TCAATTGGCATTCTATGTCCAA----------TATCTCGATCTTAA-TCTG-AAG-TAT---------------------------AACG-ATGAATGG-AAAAAATAGAAAATCC----------TTTC-GCTAG-ATAA--------GGG----AGGGC-GGATGTAG-CCAAG----------------

Liparis_ferruginea_SG1156 ATGACT-CTCGGCAATGGATATCTCGGCTCTTGCATCGATGAAGAGCGCAGCAAAATGCGATACGTGATGCGAATTGCAGAACCCCGCGAACCATCGAGTCTTTGAACGCAAGTTGCGCCCGAGGCCAAT-CGGTCAAGGGCACGCCTTCCTGGGTGTCA-AGCGTTGCATCGCTTTACAAGAATTATTTTTCTTCTCATTTTTCT------------TCTCAAATGGTATCAGAAGGTTTTGGAGTCATTCTGGAAATCCCATTCTCGTCGCGATTAGTATTT---------TCCCTTGAAG---AAAAAACAATAACAAAATCTCAGAATTTACAATCTATTCATTCAATTTTTCCCTTTTTAGAGGATAAATTATCACATTTAAATTATGTATCAGATCTAATAATACCCCATCCCATCCATCTGGAAATCTTGGTTCAAATTCTTCAATGTTGGATCAAAGATGTTCCTTCTTTGCATTTATTGCGATTGTTTTTCCACGAATATTCT---------------AATTTGACTAGTCTC---------ATTACTT------CAAATAAATC---------------CATTTACGTCTTTTCAAAAAG---AAAGAAAAGATTCTTTTGGTTCCTACATAATTCTTATGTATATGAATACGAATATCTATTCCTGTTTCTTCGTAAACAGTC---TTATTTACGATCAATATCTTCTGGAGTCTTTCTTGAGCGAACACATTTCTATGGAAAAATAGAT------TATCTTATAAT---CGTGTGTTGTAATTCTTTTCAAAGG-ATCCTCTGGTTCCTCAAAGATACTTTCATACATTATGTTCGATATCAAGGAAAAGCGATTCTTGCTTCAAAAGGAACTCTTATTCTGATGAAGAAATGGAAATTTTATTTTGTTAATCTTTGGCAATCTTATTTTCACTTTTGGTTTCAACCTTATAGGATCCATATAAAACAATTACCCCACTATTCCTTCTCTTTTCT-GGGGTATTTTTCAAGTGTAC-TAAAAAATCCTTTGGTAGTAAGAAATCAAATGCTAGATAATTCATTTCTCATAAATACTCTGACTGAGAAATTAGATACCATAGTCCCAGTGATTTCTCTTATTGGATCATTGTCAAAAGCTCAATTTTGTACTTTATTGGGTCATCCTATTAGTAAACCAATCTGGACCCATTTCTCGGATTCTGATATTCTTGATCTATTTTGTCGGATATGTAGAAATCTTTGTCGTTATCACAGCGGATCCTCAAATAAACAGGTTTTGTATCGTATAAAGTATATACTTCGACTTTCGTGTGCTAGAACTTTGGCTCGTAAACATAAAAGTAC-AGTACGCACTTTTATGCGAAGGTTAGGTTCGGGATTCTTAGAAGAATTTTTTTTGGAAGAAGAACAATCTCTTTCTTGAGCTATCCCGACCATCTCCCGTACATCATCCTAGCAGAGTACTTA-TATCTATGTCAATGAAAAGAA-CTAAAAAATAAAATTTGAACAA-------------------ATTGGACCTAG--CCCCT-GAAATT----------ATGAG-------ATC-TTCAAAAA----------GAAGAC---TTTTTTT-----------GTAAA-TGTAAGGAAAAATATATGGACTAT-GAATGA------------TTCAATAAC-GGATATTTATTGAAC-------------------------ATATATTTTCAT-------------ATCGTA----CTAT------ACAAAA--------CAAAT-----GAGA--------TTGGATTGGAA------GAA-----GAT------------------ACGAGGATTTAG------ATTCGTATCCATT------TGTGAAAGAACAGAG--TGAATGAAATGAGAAAG-------ATATTTCATTT--------------TGTTTAAACT------------GAGCCATTGATGGAAAGAGGAT-------------------------------GAGGATAAAGA-AAGA------------------GCGAGGAA-----GGGAAGTAAAAT-----GGGC-TTTTTATTGGGGATAGAGGGCTATCTAT---------AAATGGATAATACT--------TTTGTATGTATATGAATTTTTGAAGATAG--CAATGATAGCAATCCCCCAATATCT-TGT---------------TCTAAGAACAAGATATT--GGGGGATT-----------TCTT------------------------------------------------------------------------------------------------------------------------------------------------------------------------------------------------ATACCTATACTCTTA----------CTATAA-GAT------------AAAGACTCTGAAGGATAAGGC-----TTTTCAC----------ATGAATACTTAGTAGAACAACTAACGACGAGATTTATTATCGTTTCTCGCGTGTCTCACGAAAGTGAGAGTAG-GTGCGAATTCTCCCAATTTGTGACCGACCATACGATCTGTGATATAAATGGTAAA-TGTTCCTTTCCATTATGAATAGC-GATTGTATGGCCAATCATTGTGGGTA-TAATGGTAGATGCCCGAGACCAAGTCACTATGATTTCTTTCTCCTCCCTCCTGTT-GAGTTTTTCAATTCTTCCCGATAAATGATTAGCTACAAAAGGATTTTTTTTT-AGTGAACGTGTCAC-GGCTGATTACTCCTTTTTTTCCATTTTT-TAGATTGGCATTCTATGTCCAA----------TATCTCGATCTTAA-TCTG-AAG-TAT---------------------------AATG-ATGAATGG-AAAAAAGATAAAATCC----------TTTA-GCTAG-ATAA--------GGG-AA-GGGGC-GGATGTAG-CCAAGTGG-ATCAA-GGCAGT

Liparis_gigantea_PK12116 ATGACT-CTCGGCAATGGATATCTCGGCTCTTGCATCGATGAAGAGCGCAGCAAAATGCGATACGTGATGCGAATTGCAGAATCCCGCGAACCATCGAGTCTTTGAACGCAAGTTGCGCCCGAGGCCAAC-CGGTCAAGGGCACGTTTTCCTGGGTGTCA-AGCGTTGCTTCGCTTTACAAGAATGATTTTTCTTCTCATTTTTCT------------TCTCAAATGGTATCAGAAGGTTTTGGAGTCATTTTGGAAATCCCATTCTCGTCGCGATTAGTATTT---------TCCCTTGAAG---AAAAAACAATAACAAAATCTCAGAATTTACAATCTATTCATTCAATTTTTCCCTTTTTAGAGGATAAATTATCACATTTAAATTATGTGTCAGATCTAATAATACCCCATCCCATCCATCTGGAAATCTTGGTTCAAATTCTTCAATGTTGGATCAAAGATGTTCCTTCTTTGCATTTATTGCGATTATTTTTCCACGAATATTCT---------------AATTTGACTAGTCTC---------ATTACTT------CAAATAAATC---------------CATTTACGTCTTTTCAAAAAG---AAAGAAAAGATTCTTTTGGTTCCTACATAATTCTTATGTATATGAATACGAATATCTATTCCTGTTTCTTCGTAAACAGTC---TTATTTACGATCAATATCTTCTGGAGTCTTTCTTGAGCGAACACATTTCTATGGAAAAATAGAT------TATCTTAGAAT---CGTGTGTTGTAATTCTTTTCAGAGG-ATCCTATGGTTCCTCAAAGATACTTTCATACATTATGTTCGATATCAAGGAAAAGCGATTCTTGCTTCAAAAGGAACTATTATTCTGATGAAGAAATGGAAATTTTATTTTGTTAATCTTTGGCAATCTTATTTTCACTTTTGGTTTCAACCTTATAGGATCCATATAAAACAATTACCCCACTATTCCTTTTCTTTTCT-GGGGTATTTTTCAAGTGTAC-TAAAAAATCCTTTGGTAGTAAGAAATCAAATGCTAGATAATTCATTTCTTATAAATACTCTGACTGAGAAATTAGATACCATAGTCCCAGTTATTTCTCTTATTGGATCATTGTCAAAAGCTCAATTTTGTACTTTATTGGGTCATCCTATTAGTAAACCAATCTGGACCCATTTATCGGATTCTGATATTCTTGATCTATTTTGTCGGATATGTAGAAATCTTTGTCGTTATCACAGTGGATCCTCAAATAAACAGGTTTTGTATCGTATAAAGTATATACTTCGACTTTCGTGTGCTAGAACTTTGGCTCGTAAACATAAAAGTAC-AGTACGCACTTTTATGCGAAGGTTAGGTTCGGGATTCTTAGAAGAATTTTTTTTGGAAGAAGAACAATCTCTTTCTTGAGCTATCCCGACCATTTCCCGTACATCATCCTAGCAGAGTACTTA-TATCTAAGTCAATGAAAAGAA-CTAAAAAATAAAATTTGAACAA-------------------ATTGGACCTAG--CCCCT-GAAATT----------ATTAG-------ATC-TTCAAAAA----------GAAGAC---TTTTTTT-----------GTAAA-TGTAAGGAAAAAGATATGGACTAT-GAATGA------------TTCAATAAC-GGAGATTTATTGAAC-------------------------ATATATGTTCAT-------------ATCGTA----CTAT------ACAAAA--------CAAAT-----GAGA--------TTGGATTGGAA------GAA-----GAT------------------ACGAGGATTTAT------ATTCGTATCCATT------TGTGAAAGAACAGAG--TGAATGAAATGAGAAAG-------ATATTTAATTT--------------TGTTTAAACT------------GAGCCATTGATGGAAAGAGGAT-------------------------------GAGGATAAATA-AAGA------------------GCGAGGAA-----GGGAAGTAAAAT-----GGGC-TTTTTATTGGGGATAGAGGGCTATCTAT---------AAATGGATAATACT--------TTTGTATGTCTATGAATTTTTGAAGATAG--CAAT---------CCCCCAATATCT-TGT---------------TCTTAGAACAAGATATT--GGGGGATTCTTTTG---CTTCTCTATCCG---------------AATTTTCG---------TTCTTTATCAT----AAAAGTTCTC-CCCCGCC---------AATGAATGATAAGT-GCC-----TAGGTG-AAGTAT----------------AGTATAAGATAAGTCAGA----------------------AAAGTCTAAGTCTTAGTATAATACCTATACTCTTA----------CTATAA-GAT------------AAAGACTCTGAAGGATAAGGC-----TTTTCAC----------ATGAATACTTAGTAGAACGACTAACGACGAGATTTATTATCGTTTCTCGCGTGTCTCACGAAAGTGAGAGTAG-GTGCGAATTCTCCCAATTTGTGACCGACCATACGATCTGTGATATAAATGGTAAA-TGTTCCTTTCCATTATGAATAGC-GATTGTATGGCCAATCATTGTGGGTA-TAATGGTAGATGCCCGAGACCAAGTCACTATGATTTCTTTCTCCTCCCTCCTGTT-GAGTTTTTCAATTCTTCCCGATAAATGATTAGCTACAAAAGGATTTTTTTTT-AGTGAACGTGTCAC-GGCTGATTACTCCTTTTTTTCCATTTTT-TAGATTGGCATTCTATGTCCAA----------TATCTCGATCTTAA-TCTG-AAG-TAT---------------------------AATG-ATGAATGG-AAAAAAGAGAAAATCC----------TTTA-GCTAG-ATAA--------GGG-AA-GGGGC-GGATGTAG-CCAAGTGG-ATCAA-GGCAGT

Liparis_nervosa_SG1233 ATGACT-CTCGGCAATGGATATCTCGGCTCTTGCATCGATGAAGAGCGCAGCAAAATGCGATACGTGATGCGAATTGCAGAATCCCGCGAACCATCGAGTCTTTGAACGCAAGTTGCGCCCGAGGCCAAC-CGGTCAAGGGCACGTTTTCCTGGGTGTCA-AGCGTTGCTTCGCTTCACAAGAATTATTTTTCTTCTCATTTTTCT------------TCTCAAATGGTATCAGAAGGTTTTGGAGTCATTTTGGAAATCCCATTCTCGTCGCGATTAGTATTT---------TCCCTTGAAG---AAAAAACAATAACAAAATCTCAGAATTTACAATCTATTCATTCAATTTTTCCCTTTTTAGAGGATAAATTATCACATTTAAATTATGTGTCAGATCTAATAATACCCCATCCCATCCATCTGGAAATCTTGGTTCAAATTCTTCAATGTTGGATCAAAGATGTTCCTTCTTTGCATTTATTGCGATTATTTTTCCACGAATATTCA---------------AATTTGACTAGTCTC---------ATTACTT------CAAATAAATC---------------CATTTACGTCTTTTCAAAAAG---AAAGAAAAGATTCTTTTGGTTCCTACATAATTCTTATGTATATGAATACGAATATCTATTCCTGTTTCTTCGTAAACAGTC---TTATTTACGATCAATATCTTCTGGAGTCTTTCTTGAGCGAACACATTTCTATGGAAAAATAGAT------TATCTTATAAT---CGTGTGTTGTAATTCTTTTCAGAGG-ATCCTATGGTTCCTCAAAGATACTTTCATACATTATGTTCGATATCAAGGAAAAGCGATTCTTGCTTCAAAAGGAACTATTATTCTGATGAAGAAATGGAAATTTTATTTTGTTAATCTTTGGCAATCTTATTTTCACTTTTGGTTTCAACCTTATAGGATCCATATAAAACAATTACCCCACTATTCCTTTTCTTTTCT-GGGGTATTTTTCAAGTGTAC-TAAAAAATCCTTTGGTAGTAAGAAATCAAATGCTAGATAATTCATTTCTCATAAATACTCTGACTGAGAAATTAGATACCATAGTCCCAGTTATTTCTCTTATTGGATCATTGTCAAAAGCTCAATTTTGTACTTTATTGGGTCATCCTATTAGTAAACCAATCTGGACCCATTTATCGGATTCTGATATTCTTGATCTATTTTGTCGGATATGTAGAAATCTTTGTCGTTATCACAGCGGATCCTCAAATAAACAGGTTTTGTATCGTATAAAGTATATACTTCGACTTTCGTGTGCTAGAACTTTGGCTCGTAAACATAAAAGTAC-AGTACGCACTTTTATGCGAAGGTTAGGTTCGGGATTCTTAGAAGAATTTTTTTTGGAAGAAGAACAATCTCTTTCTTGAGCTATCCCGACCATTTCCCGTACATCATCCTAGCAGAGTACTTA-TATCTATGTCAATGAAAAGAA-CTAAAAAATAAAATTTGAACAA-------------------ATTGGACCTAG--CCCCT-GAAATT----------ATTAG-------ATC-TTCAAAAA----------GAAGAC---TTTTTTT-----------GTAAA-TGTAAGGAAAAAGATATGGACTAT-GAATGA------------TTCAATAAC-GGAGATTTATTGAAC-------------------------ATATATGTTCAT-------------ATCGTA----CTAT------ACAAAA--------CAAAT-----GAGA--------TTGGATTGGAA------GAA-----GAT------------------ACGAGGATTTAT------ATTCGTATCCATT------TGTGAAAGAACAGAG--TGAATGAAATGAGAAAG-------ATATTTAATTT--------------TGTTTAAACT------------GAGCCATTGATGGAAAGAGGAT-------------------------------GAGGATAAATA-AAGA------------------GCGGGGAG-----GGGAAGTAAAAG-----GGGTTTTT-----------------CTATCTAT---------AAATGGATAATACT--------TTWGTATGTATATGAATTTKTGAAGATAG--CAAT---------CCCCCAATATCT-TGT---------------TCTAAGAACAAGATATT--GGGGGATTCTTTTG---CTTCTCTATCCG---------------AATTTTCG---------TTCTTTATCAT----AAAAGTTCTC-CCCCGCC---------AATGAATGATAAGT-GCC-----TAGGTG-AAGTAT----------------AGTATAAGATAAGTCAGA----------------------AAAGTCTAAGTCTTAGTATAATACCTATACTCTTA----------CTATAA-GAT------------AAAGACTCTGAAGGATAAGGC-----TTTTCAC----------ATGAATACTTAGTAGAACGACTAACGACGAGATTTATTATCGTTTCTCGCGTGTCTCACGAAAGTGAGAGTAG-GTGCGAATTCTCCCAATTTGTGACCGACCATACGATCTGTGATATAAATGGTAAA-TGTTCCTTTCCATTATGAATAGC-GATTGTATGGCCAATCATTGTGGGTA-TAATGGTAGATGCCCGAGACCAAGTCACTATGATTTCTTTCTCCTCCCTCCTGTT-GAGTTTTTCAATTCTTCCCGATAAATGATTAGCTACAAAAGGANTTTTTTTT-AGTGAACGTGTCAC-GGCTGATTACTCCTTTTTTTCCATTTTT-TAGATTGGCATTCTATGTCCAA----------TATCTCGATCTTAA-TCTG-AAG-TAT---------------------------AATG-ATGAATGG-AAAAAAGAGAAAATCM----------TTTA-GCTAG-ATAA--------GGG-AA-GGGGC-GGATGTAG-CCAAGTGG-ATCAA-GGCAGT

Liparis_odorata_SG1256 ACGACT-CTCGGCAATGGATATCTCGGCTCTTGCATCGATGAAGAGCGCAGCAAAATGCGATACGTGATGCGAATTGCAGAACCCCGCGAACCATCGAGTCTTTGAACGCAAGTTGCGCCCGAGGCCAAC-CGGTCAAGGGCACGCTTTCCTGGGCGTCA-AGCGTTGCATCGCTTTACAAGAATTATTTTTCTTCTCATTTTTCT------------TCTCAAATGGTATCAGAAGGTTTTGGAGTCATTCTGGAAATCCCATTCTCGTCGCGATTAGTATTT---------TCCCTTGAAG---AAAAAACAATAACAAAATCTCAGAATTTACAATCTATTCATTCAATTTTTCCCTTTTTAGAGGATAAATTATCACATTTAAATTATGTGTCAGATCTAATAATACCCCATCCCATCCATCTGGAAATCTTGGTTCAAATTCTTCAATGTTGGATCAAAGATGTTCCTTCTTTGCATTTATTGCGATTGTTTTTCCACGAATATTCT---------------AATTTGACTAGTCTC---------ATTACTT------CAAATAAATC---------------CATTTACGTCTTTTCAAAAAG---AAAGAAAAGATTCTTTTGGTTCCTACATAATTCTTATGTATATGAATACGAATATCTATTCCTGTTTCTTCGTAAACAGTC---TTATTTACGATCAATATCTTCTGGAGTCTTTCTTGAGCGAACACATTTCTATGGAAAAATAGAT------TATCTTATAAT---CGTGTGTTGTAATTCTTTTCAAAGG-ATCCTATGGTTCCTCAAAGATACTTTCATACATTATGTTCGATATCAAGGAAAAGCGATTCTTGCTTCAAAAGGAACTCTTATTCTGATGAAGAAATGGAAATTTTATTTTGTTAATCTTTGGCAATCTTATTTTCACTTTTGGTTTCAACCTTATAGGATCCATATAAAACAATTACCCCACTATTCCTTCTCTTTTCT-GGGGTATTTTTCAAGTGTAC-TAAAAAATCCTTTGGTAGTAAGAAATCAAATGCTAGATAATTCATTTCTCATAAATACTCTGACTGAGAAATTAGATACCATAGTCCCAGTGATTTCTCTTATTGGATCATTGTCAAAAGCTCAATTTTGTACTTTATTGGGTCATCCTATTAGTAAACCAATCTGGACCCATTTCTCGGATTCTGATATTCTTGATCTATTTTGTCGGATATGTAGAAATCTTTGTCGTTATCACAGCGGATCCTCAAATAAACAGGTTTTGTATCGTATAAAGTATATACTTCGACTTTCGTGTGCTAGAACTTTGGCTCGTAAACATAAAAGTAC-AGTACGCACTTTTATGCGAAGGTTAGGTTCGGGATTCTTAGAAGAATTTTTTTTGGAAGAAGAACAATCTCTTTCTTGAGCTATCCCGACCATTTCCCGTACATCATCCTAGCAGAGTACTTA-TATCTATGTCAATGAAAAGAA-CTAAAAAATAAAATTTGAACAA-------------------ATTGGACCTAG--CCCCT-GAAATT----------ATTAG-------ATC-TTCAAAAA----------GAAGAC---TTTTTTT-----------GTAAA-TGTAAGGAAAAATATATGGACTAT-GAATGA------------TTCAATAAC-GGAGATTTCTTGAAC-------------------------ATATATTTTCAT-------------ATCGTA----CTAT------ACAAAA--------CAAAT-----GAGA--------TTGGATTGGAA------GAA-----GAT------------------ACGAGGATTTAG------ATTCGTATCCATT------TGTGAAAGAACAGAG--TGAATGAAATGAGAAAG-------ATATTTCATTT--------------TGTTTAAACT------------GAGCCATTGATGGAAAGAGGAT-------------------------------GAGGATAAAGA-AAGA------------------GCGAGGAA-----GGGAAGTAAAAT-----GGGC-TTTTTATTGGGGATAGAGGGCTGTCTAT---------AAATGGATAATACT--------TTTGTATGTATATGAATTTTTGAAGATAG--CAAT---------CCCCCAA-----------------------------------GATATT--GGGGGATT-----------TCTT------------------------------------------------------------------------------------------------------------------------------------------------------------------------------------------------ATACCTATACTCTTA----------CTATAA-GAT------------AAAGACTCTGAAGGATAAGGC-----TTCTCAC----------ATGAATACTTAGTAGAACGACTAACGACGAGATTTATTATCGTTTCTCGCGTGTCTCACGAAAGTGAGAGTAG-GTGCGAATTCTCCCAATTTGTGACCGACCATACGATCTGTGATATAAATGGTAAA-TGTTCCTTTCCATTATGAATAGC-GATTGTATGGCCAATCATTGTGGGTA-TAATGGTAGATGCCCGAGACCAAGTCACTATGATTTCTTTCTCCTCCCTCCTGTT-GAGTTTTTCAATTCTTCCCGATAAATGATTAGCTACAAAAGGATTTTTTTTT-AGTGAACGTGTCAC-GGCTGATTACTCCTTTTTTTCCATTTTT-TAGATTGGCATTCTATGTCCAA----------TATCTCGATCTTAA-TCTG-AAG-TAT---------------------------AATG-ATGAATGG-AAAAAAGATAAAATCC----------TTTA-GCTAG-ATAA--------GGG-AA-GGGGC-GGATGTAG-CCAAGTGG-ATCAA-GGCAGT

Liparis_sootenzanensis_SG1351 ACGACT-CTCGGCAATGGATATCTCGGCTCTTGCATCGATGAAGAGCGCAGCAAAATGCGATACGTGATGCGAATTGCAGAATCCCGCGAACCATCGAGTCTTTGAACGCAAGTTGCGCCCGAGGCCAAC-CGGTCAAGGGCACGCTTTCCTGGGTGTCA-AGCGTTGCATCGCTTT-CAAGAATTATTTTTCTTCTCATTTTTCT------------TCTCAAATGGTATCAGAAGGTTTTGGAGTCATTCTGGAAATCCCATTCTCGTCGCGATTAGTATTT---------TCCCTTGAAG---AAAAAACAATAACAAAATCTCAGAATTTACAATCTATTCATTCAATTTTTCCCTTTTTAGAGGATAAATTATCACATTTAAATTATGTGTCAGATCTAATAATACCCCATCCCATCCATCTGGAAATCTTGGTTCAAATTCTTCAATGTTGGATCAAAGATGTTCCTTCTTTGCATTTATTGCGATTGTTTTTCCACGAATATTCT---------------AATTTGACTAGTCTC---------ATTACTT------CAAATAAATC---------------CATTTACGTCTTTTCAAAAAG---AAAGAAAAGATTCTTTTGGTTCCTACATAATTCTTATGTATATGAATACGAATATCTATTCCTGTTTCTTCGTAAACAGTC---TTATTTACGATCAATATCTTATGGAGTCTTTCTTGAGCGAACACATTTCTATGGAAAAATAGAT------TATCTTATAAT---CGTGTGTTGTAATTCTTTTCAGAGG-ATCCTATGGTTCCTCAAAGATATTTTCATACATTATGTTCGATATCAAGGAAAAGCGATTCTTGCTTCAAAAGGAACTATTATTCTGATGAAGAAATGGAAATTTTATTTTGTTAATCTTTGGCAATCTTATTTTCACTTTTGGTTTCAACCTTATAGGATCCATATAAAACAATTACCCCACTATTCCTTCTCTTTTCT-GGGGTATTTTTCAAGTGTAC-TAAAAAATCCTTTGGTAGTAAGAAATCAAATGCTAGATAATTCATTTCTCATAAATACTCTGACTGAGAAATTAGATACCATAGTCCCAGTTATTTCTCTTATTGGATCATTGTCAAAAGCTCAATTTTGTACTTTATTGGGTCATCCTATCAGTAAACCAATCTGGACCCATTTATCGGATTCTAATATTCTTGATCTATTTTGTCGGATATGTAGAAATCTTTGTCGTTATCACAGCGGATCCTCAAATAAACAGGTTTTGTATCGTATAAAGTATATACTTCGACTTTCGTGTGCTAGAACTTTGGCTCGTAAACATAAAAGTAC-AGTACGCACTTTTATGCGAAGGTTAGGTTCGGGATTCTTAGAAGAATTTTTT-TGGAAGA-----------------GAGCTATCCCGACCATTTCCCGTACATCATCCTAGCAGAGTACTTA-TATCTATGTCAATTAAAAGAA-CTAAAAAATAAAATTTGAACAA-------------------ATTGGACCTAG--CCCCT-GAAATT----------ATTAG-------ATY-TTCAAAAA----------GAAGAC---TTTTTTT-----------GTAAA-TGTAAGGAAAAATATATGGACTAT-GAATGA------------TTCAATAAC-GGAGATTTATTGAAC-------------------------ATATATGTTCAT-------------ATCGTA----CTAT------ACAAAA--------CAAAT-----GAGA--------TTGGATTGGAA------GAA-----GAT------------------ACGAGGATTTAT------ATTCGTATCCATT------TGTGAAAGAACAGAG--TGAATGAAATGAGAAAG-------ATATTTCATTT--------------TGTTTAAAMT------------GAGCCATTGATGGAAAGAGGAT-------------------------------GAGGATAAATA-AAGA------------------GCGAGGAA-----GGGAAGTAAAAG-----GGGC-TTTTTATTGGGGATAGAGG-CTATCTAT---------AAATGGATAATACT--------TTTGTATGTATATGAATTTTTGAAGATAG--CAAT---------CCCCCAATATCT-TGT---------------TCTTAGAACAAGATATT--GGGGGATTCTTTTG---CTTCTCTATCCG---------------AATTTTTG---------TTCTTTATCAT----AAAAGTTCTC-CCCCGCC---------AATGAATGATAAGT-GCC-----TAGGTG-AAGTAT----------------AGTATAAGATAAGTCAGA----------------------AAAGTCTAAGTCTTAGTATAATACCTATACTCTTA----------CTATAA-GAT------------AAAGACTCTGAAGGATAAGGC-----TTTTCAC----------ATGAATACTTAGTAGAACGACTAACGACGAGATTTCTTATCGTTTCTCGCGTGTCTCACGAAAGTGAGAGTAG-GTGCGAATTCTCCCAATTTGTGACCGACCATACGATCTGTGATATAAATGGTAAA-TGTTCCTTTCCATTATGAATAGC-GATTGTATGGCCAATCATTGTGGGTA-TAATGGTAGATGCCCGAGACCAAGTCACTATGATTTCTTTCTCCTCCCTCCTGTT-GAGTTTTTCAATTCTTCCCGATAAATGATTAGCTACAAAAGGATTTTTTTTT-AGTGAACGTGTCAC-GGCTGATTACTCCTTTTTTTCCATTTTT-TAGATTGGCATTCTATGTCCAA----------TATCTCGATCTTAA-TCTG-AAG-TAT---------------------------AATG-ATGAATGG-AAAAAAGAGAAAATCC----------TTTA-GCTAG-ATAA--------GGT-AAGGGGGC-GGATGTAG-CCAAGTGG-ATCAG-GGCAGT

Liparis_stricklandiana_SG1332 ACGACT-CTCGGCAATGGATATCTCGGCTCTTGCATCGATGAAGAGCGCAGCGAAATGCGATACGTGGTGCGAATTGCAGAATCCCGCGAACCATCGAGTCTTTGAACGCAAGTTGCGCCCGAGGCCAAC-CGGCCAAGGGCACGTTTGCCTGGGCGTCA-AGCGTTGCGTCGCTTCACAAGAATTCTTTTTCTTCTCATTTTTCTTCTCAAATGGTATCTCAAATGGTATCAGAAGGTTTTGGAGTCATTCTGGAAATTCCATTCTCGTCGCAATTAGTATTT---------TCCCTTGAAG---AAAAAAGAATACCAAAATATCAGAATTTACGATCTATTCATTCAATATTTCCCTTTTTAGAGGATAAATTATCACATTTAAATTATGTGTCAGATCTACTAATACCCCATCCCATCCATCTGGAAATCTTGGTTCAAATTCTTCAATGTTGGATCAAAGATGTTCCTTCTTTGCATTTATTGCGATTGTTTTTCCACGAATATCAT---------------AATTTGACTAGTCTC---------ATTACTT------CAAATAAATC---------------CATTTACGTCTTTTCAAAAAG---AAATAAAAGATTCTTTTGGTTCCTACATAATTCTTATGTATATGAATACGAATATCTATTCCTGTCTCTTCATAAACGGTCTTCTTATTTACGATTAATATCTTCTGGAGTCTTTCTTGAGCGAACACATTTCTATGGAAAAATAGAA------TATCTTATAGT---CGTGTGTTGTAATTCTTTTCAGAGG-ATCCTATGGTTCCTCAAAGATACTTTCATACATTATGTTCGATATCAAGGAAAAGCGATTCTGGCTTCAAAAGGAACTCTTATTCTGATGAAGAAATGGAAATTTTATTTTGTGAATCTTTGGCAATCTTATTTTCACTTTTGGTTTCAACCTTATAGGATTCATATAAAGCAATTACCCAACTATTCCTTCTCTTTTCT-GGGGTATTTTTCAAGTGTAC-TAAAAAATCCTTTGGTAGTAAGAAATCAAATGCTAGATAATTCATTTCTAATAAATACTCTGACTAAGAAATTAGATACCATAGTCCCAGTTATTTCTCTTATTGGATCATTGTCGAAAGCTCAATTTTGTACTGTATTGGGTCATCCTATTAGTAAACCAATCTGGACCGATTTATCGGATTCTGATATTCTTGATCTATTTTGTCGGATATGTAGAAATCTTTGTCGTTATCACAGCGGATCCTCAAAGAAACAAGTTTTGTATCGTATAAAGTATATACTTCGACTTTCGTGTGCTAGAACTTTGGCTCGTAAACATAAGAGTAC-AGTACGCACTTTTATGCGAAGGTTAGGTTCGGGATTCTTAGAAGAATTTTTTTTGGAAGAA----------------GAGCTATCCCGACCATTTACCATACATCATCCTAGCAGAGTACTTA-TATCTATGTCAACGAAAAGAA-CTAAAAAATAAAAA---------------------------TTTGGACCTAG--CCCCT-GAATTT----------CTTAG-------ATC-TTCAAAAA----------GAAGAC---TTTTTTT-----------GTAAA-TGTAAGGAAAAAGATATGGACTAT-TAATGA------------TTCAATAAC-GGAGATTCCTTGAAC-------------------------ATATATGTTCAT-------------ATCGTA----CTAT------ACAAAA--------CAAAG-----GAGA--------TTGGATTGGAA------GAA-----GAT------------------ACGAGGATTTAT------ATTCGTATACATT------TGTGAAAGAACAGAG--TGAATGAATTGAGAAAG-------ATATTTAATTT--------------TGTTTAAACTGAGCCA------CTGATACTGATGGAAAGAGGAT-------------------------------GAGGATAAAGA-AAGA------------------GCGAGGAA-----GGGAAGTAAAAT-----GGGC-TTTTTATTGGGGATAGAGGGCCATCTAT---------AAATGGATAATACT--------TTTGTATGTATATGAATTTTTGAAGATAG--CAAT---------CCCCCAATATCT-TGT---------------TCTAAGAACAAGATATT--GGGGGATTCTTTTG---CTTCTCTATCCG---------------AATTTTCG---------TTCTTTATCAT----AAAAGTTCTC-CCCCGCC---------AATGAATGATAAGT-GCC-----TAGGTG-AAGTAT----------------AGTATAAGATAAGTCAGA----------------------AAAGTCTAAGTCTTAGTATAATACCTATACTCTTA----------CTATAA-GAT------------AAAGACTCTTAACGATAAGGC-----TTTTCAC----------ATGAATACTTAGTAGAACGACTAACGACGAGATTTATTATCGTTTCTCGCGTGTCTCACGAAAGTGAGAGTAG-GTGCGAATTCTCCCAATTTGTGACCGACCATACGATCTGTGATATAAATGGTAAA-TGTTCCTTTCCATTATGAATAGC-GATTGTATGTCCAATCATTGTGGGTA-TAATGGTAGATGCCCGAGACCAAGTCACTATGATTTCTTTCTCCTCCCTCCTGTT-GAGTTTTTCAATTCTTCCCGATAAATGATTAGCTACAAAAGGATTTTTTTTT-AGTGAACGTGTCAC-GGCTGATTACTCCTTTTTTTACATTTTT-TAAATTGGCATTCTATGTCCAA----------TATCTCGATCTTAA-TCTG-AAG-TAT---------------------------AACG-ATGAATGG-AAAAAATAGAAAATCC----------TTTC-GCTAG-ATAA--------GGG-AA-GGGGC-GGATGTAG-CCAAGTGG-ATCAA-GGCAGT

Liparis_viridiflora_SG1308 ACGACT-CTCGGCAATGGATATCTCGGCTCTTGCATCGATGAAGAGCGCAGCGAAATGCGATACGTGGTGCGAATTGCAGAATCCCGCGAACCATCGAGTCTTTGAACGCAAGTTGCGCCCGAGGCCAAC-CGGCCAAGGGCACGTTTGCCTGGGCGTCA-AGCGTTGCGTCGCTTC---AGAATTCTTTTTCTTCTCATTTTTCTTCTCGAATGGTATCTCAAATGGTATCAGAAGGTTTTGGAGTCATTCTGGAAATTCCATTCTCGTCGCAATTAGTAATT---------TCCCTTGAAG---AAAAAAGAATACCAAAATATCAGAATTTACGATCTATTCATTCAATATTTCCCTTTTTAGAGGATAAATTATCACATTTAAATTATGTGTCAGATCTACTAATACCCCATCCCATCCATCTGGAAATCTTGGTTCAAATTCTTCAATGTTGGATCAAAGATGTTCCTTCTTTGCATTTATTGCGATTGTTTTTCCACGAATATCAT---------------AATTTGACTAGTCTC---------ATTACTT------CAAATAAATC---------------TATTTACGTCTTTTCAAAAAG---AAATAAAAGATTCTTTTGGTTCCTACATAATTCTTATGTATATGAATACGAATATCTATTCCTGTCTCTTCATAAACGGTCTTCTTATTTACGATCAATATCTTCTGGAGTCTTTCTTGAGCGAACACATTTCTATGGAAAAATAGAA------TATCTTATAGT---CGTGTGTTGTAATTCTTTTCAGAGG-ATCCTATGGTTCCTCAAAGATACTTTCATACATTATGTTCGATATCAAGGAAAAGCGATTCTGGCTTCAAAAGGAACTCTTATTCTGATGAAGAAATGGAAATTTTATTTTGTGAATCTTTGGCAATCTTATTTTCACTTTTGGTTTCAACCTTATAGGATTCATATAAAGCAATTACCCAACTATTCCTTCTCTTTTCT-GGGATATTTTTCAAGTGTAC-TAAAAAATCCTTTGGTAGTAAGAAATCAAATGCTATATAATTCATTTCTAATAAATACTCTGACTAAGAAATTAGATACCATAGTCCCAGTTATTTCTCTTATTGGATCATTGTCGAAAGCTCAATTTTGTACTGTATTGGGTCATCCTATTAGTAAACCAATCTGGACCGATTTATCGGATTCTGATATTCTTGATCTATTTTGTCGGATATGTAGAAATCTTTGTCGTTATCACAGCGGATCCTCAAAGAAACAAGTTTTGTATCGTATAAAGTATATACTTCGACTTTCGTGTGCTAGAACTTTGGCTCGTAAACATAAGAGTAC-AGTACGCACTTTTATGCGAAGGTTAGGTTCGGGATTCTTAGAAGAATTTTTTTTGGAA-------------------GAGCTATCCCGACCATTTACCATACATCATCCTAGCAGAGTACTTA-TATCTAAGTCAATGAAAAGAA-CTAAAAAATAAAAA---------------------------TTTGGACCTAG--CCCCT-GAATTT----------CTTAG-------ATC-TTCAAAAA----------TAAGAC---TTTTTTT-----------GTAAA-TGTAAGGAAAAAGATATGGACTAT-TAATGA------------TTCAATAAC-GGAGATTCCTTGAAC-------------------------ATATATGTTCAT-------------ATCGTA----CTAT------ACAAAA--------CAAAG-----GAGA--------TTGGATTGGAA------GAA-----GAC------------------ACGAGGATTTAT------ATTCGTATACATT------TGTGAAAGAACAGAG--TGAATGAAATGAGAAAG-------ATATTTAATTT--------------TGTTTAAACTGAGCCA------CTGATACTGATGGAAAGAGGAT-------------------------------GAGGATAAAGA-AAGA------------------GCGAGGAA-----GGGAAGTAAAAT-----GGGC-TTTTTATTGGGGATAGAGG-CCATCTAT---------AAATGGATAATACT--------TTTGTATGTATATGAATTTTTGAAGATAG--CAAT---------CCCCGAATATCT-TGT---------------TCTTAGAACAAGATATT--CGGGGATTCTTTTG---CTTCTCTATCCG---------------AATTTTCG---------TTCTTTATCAT----AAAAGTTCTC-CCCCGCC---------AATGAATGATAAGT-GCC-----TAGGTG-AAGTAT----------------AGTATAAGATAAGTCAGA----------------------AAAGTCTAAGTCTTAGTATAATACCTATACTCTTA----------CTATAA-GAT------------AAAGACTCTTAACGATAAGGC-----TTTTCAC----------ATGAATACTTAGTAGAACGACTAACGACGAGATTTATTATCGTTTCTCGCGTGTCTCACGAAAGTGAGAGTAG-GTGCGAATTCTCCCAATTTGTGACCGACCATACGATCTGTGATATAAATGGTAAA-TGTTCCTTTCCATTATGAATAGC-GATTGTATGGCCAATCATTGTGGGTA-TAATGGTAGATGCCCGAGACCAAGTCACTATGATTTCTTTCTCCTCCCTCCTGTT-GAGTTTTTCAATTCTTCCCGATAAATGATTAGCTACAAAAGGATTTTTTTTT-AGTGAACGTGTCAC-GGCTGATTACTCCTTTTTTTACATTTTT-TAAATTGGCATTCTATGTCCAA----------TATCTCGATCTTAA-TCTG-AAG-TAT---------------------------AACG-ATGAATGG-AAAAAATAGAAAATCC----------TTTC-GCTAG-ATAA--------GGG-AA-GGGGC-GGATGTAG-CCAAGTGG-ATCAA-GGCAGT

Ludisia_discolor_SG1236 ATGACT-CTCGGCAATGGATATCTTGGCTCTTGCATCGATGAAGAGCGCAGCGAAATGCGATACGTGGTGTGAATTGCAGAATCCCGTGAACCATCAAATCTTTGAACGCAAGTTGCGCCCGAGGCCATT-TGGCTAAGGGCACGTCCGCCTGGGCGTCA-AGCATTACATCGCTTCATAAGAATTATTTTTCTTCTCATTTTTCT------------TTTCAAATACTATCAGAAGGTTTTGGAGTCGTTCTGGAAATTCCATTATTGTTGCAATTAGTATCC---------TTCCTTAAAG---AAAAAAAAATACAAAAATCTCAGAATTTACGATCTATTCATTCAATATTTCCTTTTTTAGAGGATAAATTCTCACATTTAAATTCTGTATCAAATCTACTAATACCCCATCCCATCCATCTGGAAATATTGGTTCAAATGCTTCAATGCTGGATCAAAGATGTTCCTTCTTTGCATTTGTTGCGATTTATTTTCCACGAATATCAT---------------AATTTGAAGAGTATC---------ATTACTT------CAAAGAAATC---------------CATTCACGTTTTTTCAAAAAA---AAAGAAAAGATTTTTTTGGCTCCTACATAATTTTTATGTATATGAATGCGAATATCTTTTTCTTTTTCTTCGTAAAAAGTCTTCTTATTTACGATCAACATCTTTTGGAGTCTTTATTGAGCGAACACTTTTTCATGTAAAAATGGAA------TCTATTTTAGT---AGTGTATTTTAATTCTTTTCAGAGG-ATTCTCTGGCTCCTCAAAGATCCTTTCATACATTATGTTCGATATCAAGGAAAAGTAATTCTGGCTTCAAAGGGAACTCTTATTCTGATGAAGAAATGGAATTTTCATGTTGTGAATTTTTGGCAATTTTATTTTCACTTTTGGTCTCAACCTTATAGGATCCATATAAAGCAATTACCCAACTATTCCTTCTCTTTTCT-GGGGTATTTTTTAAGTGTAC-AAAAAAAAACTTTGGTAGTAAGAAATCAAATGCTAGAGAATTCCTTTCTAATAAATACTCTGACTAAGAAATTAGATACCATAGCCCCAGTTATTTCTCTTATTGGATCATTGTCGAAAGCTCAATTTTGTACTATATCAGGTCATCCTATTAGTAAACCAATTTGGACCGATTTATCGGATTCTGATATTATTGATCGATTTTGTCGGAAATGTAGAAATCTTTGTCGTTATCACAGCGGATCCTCAAAAAAAAAAGTTTTGTATCGTATAAAATATATACTTCGACTTTCGTGTGCTAGAACTTTGGCTCGTAAACATAAAAGTAC-AGTACGCATTTTTATGCGAAGATTGGGTTCGGTATTTTTAGAAGAATTTTTTATGGAAGAAGAACAAGCTCTTTATTGAGCTATCCCGACCAGTACCC-TGCATCATCCTAGCAGAGTACTTG-TATCTATGTAAACGAAAAGAA-CTAAAAAAGAAAGTCTTAACAA-------------------ATTGGACCTAG-TCCCCTTTAATTT----------CTTAG-------ATC-TTCGAAAA----------GAAAAC---TTTCTTT-----------GTAAA-TGTAAAGATAATGATATGAACTGT-GAATAATTAAATGAATTATTAAGTAAG-GGAGATTCCTTGAAC-------------------------ATATATGTTCAT-TTGTGCAGGT--CTCGTA---TCTAT------ACAAATAAAAA---CAAAG-----AAAA--------TTGGATTGAAATTGGAAGAA-----GAT------------------AGGAGGATTTCT------ATTCGGATCCATT------TGTGAAAGAACAGAG--TGAATGAAATTAGAAAG-------ATATTTAATTT--------------TGTTTGAACT------------GAGCAACTGATAAAAATAGGAT-------------------------------GAGAGTAAAGA-AAGA------------------ATGAGGAA-----------TAAAAAGC----------------------------CCATCTAC---------AAATGGATAAGACT--------TTTGTCTTTCGATGAGTTCTTTAAGGTAG--CAAT----------ATCCAATATC-----------------------------------TT---GGGGATTCTTTTG---CCTCTTTATCCG------ATTTCTCCGAATTTTCG---------TTCTTTATCATAAAAGAAAGTTCTC-CCCCGCC---------AATGAATGAGAAGT-GCC-----TAGGTG-AAGTAT----------------AGTCTAAGATAAGTCAGA----------------------AAAATCTCAGTCTTAGTATAATACCTAGACTCTGA----------CTATAA-GAT------------AAAGACTCTGAAGGATCAGAC-----TTTTCAC----------ATGAAGACTGAGTAGAACGACTAACGACGAGATTGATTCTCATTTCTCGCATGTCTCACGAAAGTGAGAGTAG-GTGCGAATTCTCCCAATTTGTGACCGACCATACGATCTTTTATATAAAGGGTAAA-TGTTCCTTTCCATTATGAATAGC-GATTGTATGGCCAATCATTGTGGGTA-TAATGGTAGATGCCCTAGACCAAGTCACTCTTCTTTCTTTCTCCTCCCTCATGTT-GAGTTTTTCAATTTTGACCGAGAAATGATTAGCTACAAAAGGATTTTTTTTT-AGTGAACGTGTCAC-GGCCGATTACTCCTTTTTTTACATTTTT-GAAATTGGCATTCTATGTCCAA----------TATCTCGATCTTAA-TCTG-AAG-TAT-GAGGGTAAGAATCAAGACAAT-----AATG-ATGAATGG-AAAAAAGAGAAAATCC----------TTGA-GCTAG-ATAA--------GGG-AA-GGGGC-GGATGTAG-CCAAGTGG-ATCAA-GGCAGT

Nephelaphyllum_tenuiflorum_SG1220 AAGACT-CTCAACAATGGATATCTTGGGTCTCGCATCGATGAAGAGCGCAGCGAAATGTGATATATGGTGTGAATTGCAGAATCCCGCGAGCCATCGAGTCTTTGAACGCAAGTTGCGTCTGAGGCCAAT-AGGCCAAGGGCACGTCTGCCTGGGCGTCA-AGCGTTTTGTTGCTCTACAAGAATTCTTTTTCTTCTCATTTTTCT------------TCTCAAATGGTATCAGAAGGTTTTGGAGTCATTCTGGAAATTCCATTCTCGTCGCGATTAGTATCT---------TCTTTTCAAGAAAAAAAAAGAATACCAAAATCTCAGAATTTACGATCTATTCATTCAATATTTCCCTTTTTAGAGGATAAAATATCACATTTAAATTATGTGTCAGATCTACTAATACCCCATCCCATCCATCTGGAAATCTTGGTTCAAATCCTTCAATGCTGGATCAAAGATGTTCCTTCTTTGCATTTATTGCGATTCTTTTTCCACGAATATCAT---------------AATTTGAATAGTCTC---------ATTACTT------CAAAGAAATC---------------CATTTACGTCTTTTCAAAAAG---AAAGAAAAGATTCTTTTTGTTCCTACATAATTCTTATGTATATGAATGCGAATATCTATTCCTGTTTCTTCGTAAACAGTCTTCTTATTTACGATCAATATCTTCTGGAGTCTTTTTTGAACGAACACTTTTCTATGGAAAAATAGAA------TATCTTCTAGT---CGTGTGTTGTAATTCTTTTCAGAGG-ATCCTATGGTTCCTCAAAGATATTTTCATACATTATGTTCGATATCAAGGAAAAGCTATTCTGGCTTCAAAAGGAACTCTTATTCTGATGAAGAAATGGAATTTTCATCTTGTAAATTTTTGGCAATCTTATTTTCACTTTTGGGTTCAACCTTATAGGATCCATCTAAAGCAATTACCCAACTATTCCTTCTCTTTTCT-GGGGTATTTTTCAAGTGTAC-TAAAAAACCATTTGATAGTAAGAAATCAAATGCTAGAGAATTCATTTCTAATAAATACTCTGACTAAGAAATTAGATACAATAGCCCCAGTTATTTCTCTTATTAGATTGTTGTCGAAAGCTCAATTTTGTACTGTATTGGGTCATCCTATTAGTAAACCAATCTGGACCGATTTATCGGATTCTGATATTCTTGATCGATTTTGTCGGATATCTAGAAATCTTTGTCGTTATCACAGCGGATCCTCAAAGAAACAGGTTTTGTATCGTATAAAGTATATACTTCGACTTTCGTGTGCTAGAACTTTAGCTCGTAAACATAAAAGTAC-AGTACGCACTTTTATGCGAAGATTAGGTTCGGGATTCTTAGAAGAATTTTTTTTGGAAGAAGAAAAATCTCTTTCTTGAGCTACCCCGACCATTTCCCGTGCATCATCCTAGCAGAGTACTTA-TTTCTATGTCAATGAAAAGAA-TTTCAAAATCAAATCTTAACAA-------------------ATTGGACCTAG--ACCCT-GAATTT----------CTTAG-------ATC-TTCAAAAA----------GAAGAC---ATTCTTT-----------GTAAA-TGTCAGGAAAAATATATGGACTAT-GAATGA------------TTCAATAAC-GGAAATTCCTTGAAC-----------------------ATATATATGTTCAT-------------ATCGTA----CTAT------ACAAAA--------CAAAT-----GAGA--------TTGGATTGGAA------GAA-----GAT------------------ACGAGGGTTTCT------ATTCGGATCCTTT-----ATGTGAAAGAACAGAG--TGAATGAAATGAGAAAG-------ATATTTCATTT--------------TGTTTAAATTGATCCA------GAGCCACTGATG-----------------------------------------AAAAAAAAATA-AAGA------------------GTGAGGAA----------GTAAAAT-----GGGC-TTTTTATTGGGGATAGAGGG----------------------------------------------------------------------------------------------------------------------------------------------------------------------------------------------------------------------------------------------------------------------------------------------------------------------------------------------------------------------------------------------------------------------------------------------------------------------------------------------------------------------------------------------------------------------------------------------------------------------------------------------------------------------------------------------------------------------------------------------------------------------------------------------------------------------------------------------------------------------------------------------------------------------------------------------------------------------------------------------------

Nervilia_plicata_SG1143 ACGACT-CTCGGCAATGGATATCTCGGCTCTCGCATCGATGAAGAGCGCAGCGAAATGCGATACGTGGTGCGAATTGCAGAATCCCGTGAACCATCGAGTCTTTGAACGCAAGTTGCGCCCGAGGCCCAC-CGGCCGAGGGCACGCCCGCCTGGGCGTCA-AGCACCGCATCACTCCACAAGGATTCTTTTTCCTCTCATTTCTCT------------TCTAAAATGGTATCAGAAGGTTTTGGAGTCATTCTAGAAATCCCATTCGCGTCACGATTAGTATCT---------TCTCTTGAAG---AAAAAAGAAGACCAAGATCTCAGAATTTACGATCTATTCATTCAATATTTCCCTTTTTAGAGGATAAATTCTTACATCTAGATTATGTGTCAGATCTACTAATACCCCATCCCATCCATCTGGAAATCTTGGTTCAAATTCTTCAATGCTGGATCAAAGATGTTCCTTCTTTGCATTTATTGCGATTGTTTTTTCACGAATATCAT---------------AATTTGAATAGTCTC---------ATTACTT------CAAAGAGATC---------------CATTTACGCCCTTTCAAAAAG---AAAGAAAAGATTCTTTTGGTTCTTACATAATTCTTATGTATATGAATACGAATATCTATTCCTCTTTCTTCGTAAACAGTCTTCTTATTTAAGATCAATATCTTCTGGAGTCTTTCTTGAGCGAACACATTTCTATGGAAAAATAGAA------TATCTTATAGT---AGTGTTTTGTAATTCTTTTCAGAGG-ATCCTATGGTTCCTCAAAGATCCTTTCATACATTATGTTCGATATCAAGGAAAAGCAATTTTGGTTTCAAAAGGAACTCTTATTTTGATGAAGAAATGGAAATCTCATCTTGTGAATTTTTGGCAATCTTATTTTCACTTTTGGTCTCAACCTTATAGGATCCTTATAAAGCAATTACCCAACTATTCCTTCTCTTTTTT-GGGGTATTTTTCAAGTGTAC-TAAAAAATCATTTGATAGTAAGAAATCAAATGCTAGAGAATTCATTTATAATAAATACTCTGACTAATAAATTAGATACCATAGCCCCAGTTATTTCTCTTATTGGATCATTGTCGAAAGCTCAATTTTGTACAGTATTGGGTCATCCTATTAGTAAACCGATCTGGACCGATTTATCGGATTCTGATATTCTTGATCGATTTTGTCGGATATGTAGAAATCTTTGTCGTTATCACAGTGGATCCTCAAATAAACAGGTTTTGTATCGTATAAAGTATATACTTCGACTTTCGTGTGCTAGAACTTTGGCTCGTAAACATAAGAGTAC-AGTACGCACTTTTATGCGAAGATTAGGTTCGGGATTCTTAGAAGAATTCTTTTTGGAAGAAGAACAAGCTCTTTCTTGAGCTATCCCGACCATTTCCCGTGCATCATCCTAGCCGAGTACTTC-GATCTAAGTCAATGAAAAGAA-CTAAAAAAGAGAGTCTTAACAA-------------------ATTGTACCTAG--ACCCT-GAATCT----------CTTAG-------ATC-TTCAAAAA----------GAAGAC---TTTCTTT-----------GTAAA-TGAAATGTAAGGATTATGGACTGT-GAATGG------------TTCAATAAC-GGAGATTCCTTGAAC-------------------------ATATATGTTCAT-TTGTGCAGGC--ATTGTG----CTAT------ACAAAA--------CAAAT-----GAAA--------TTGGATT----------------------------------------------------------------------------------------------------------------------------------------------TGTTTGAACT------------GATCCACTAATGAAGATATGAT-------------------------------GAGGATAAATA-AAGA------------------GAGAGAAA----------GTAAAAGAAGAAGGGC-TTCTTCTTGGGGATAGAGGACCATCTAT---------AAATGTATAAGACT--------TTTGTCTATG----AATTTTTGAAGGTAT--CGAT---------CCCCCAATATCT-TGT----------------------------TATT--GGGGGATC--------------------------------------------------------------------------------------------------------------GAT-----TCAGTG-------------------------------------------------------------------------------------------------------------------------------------------------------------------------------------TA-GTAGAACGACTAACGACGAGATTTATTATYGTTTCTCGCGTGTCTCACGAAAGTGAGAGTAG-GTGCGAATTCTCCCAATTTGTGACCGACCATGCGATCTGTGATATAAATGGTAAA-TGTTCCTTTCCATTATGAATAGC-GATTGTATGGCCAATCATCGTGGGTA-TAATGGTAGATGCCCGAGACCAAGTCACTATTATTTCCTTCTCCTCCCTCCTGTT-GAGTTTTTCAATTCTTTCCAATAAATGATTAGCTACAAAAGGATTTTTTTTT-AGTGAACGTGTCAC-GGCTGATTACTCCTTTTTTTCAATTTTT-------------------------------------------TTAA-TCTG-AAG-TAT---------------------------AATG-ATGAATGG-AAAAAAGAGAGAATCC----------TTTA-GCTAG-ATAA--------GGG-AA-GGGGC-GGATGTAG-CCAAGCGG-ATCAA-GGCAGT

Neuwiedia_zollingeri_var_singapureana_KFBG35 ATGACT-CTCGACAACGGATATCTTGGCTCTTGCATCGATGAAGAACGCAGCGAAATGTGATATATGGTGTGAATTGCAGAATCCCGTGAACCATCGAGTACTTGAACGCAAGTTGCGCCTGAGGCCAAG-TGGTTGATGGCACACCTGCCTGGTTGTC---GTCGTATGTCGTCTCACAAGAATTCTTTTTCTTCTAATTTTTCT------------TCTCAGATGGTATCAGAAGGTTTTGGAGTCATTCTGGAAATTCCATTCTCGTCGCGATTAGTTTAT---------TCCTCTGAAG---AAAAAAAAATATCAAAATCTCAGAATTTACGATCTATTCATTCAATATTTCCTTTTTTAGAGGACAAATTCTCACATTTAAATTATGTATTAGGTATACTAATACCCCATCCCATCCATCTGGAAATCTTGGTTCAAATCCTTCAATGCTGGATCAAAGATGTTCCTTATTTGCATTTTTTGATATTTTTTTTCCACGAATATCAT---------------AATTCGAATAGTCTT---------TTTACTTC-----CAAAAAAATA---------------CATT----TCTTCTTAAAAAGAAAAAAAAAAA------TTGGCTTCCTATATAATTCTTATATATTTGAATGCGAATTTCTATTACTATTTCTTCATAAACAGTCTTCTTACTTACGATCAACATCTTCTGGAGTTTTTCTTGAGCGAATGCATTTCTATGAAAAAATGGAA------CATCTTGTAGT---AATGTGTTATAATTCTTTTCA-AGGTATTCCATGCTTCCTCAAAGATACTTTCATGCATTATGTTCGATATCAAGGAAAAGGAATTCTGGCTTCAAAAGGGACTCTTATTCTGATGAAGAAATGGAAATGTCATCTTGTTAATTTTTGGCAATATTCTTTTTGCTTTTGGTCTCAACCATATAGGATACATATAAAACAATTATTCAACTATTCCTTTTCTTTTCT-GGGGTATTTTTCGAGTGTAC-TAAGACACCCTTTGGTAGTAAGAAATCAAATGCTAGAGAATTCATTTCTCATGGATATTTTGACTAAGAAATTAGATACCATAATCCCAGTTATTTCTCTTATTGGATCTTTGTCAAAAGCCCAATTTTGTACTGTATTGGGTCATCCTATTAGTAAACCGATCTGGACTGATTTTTCGGATTCTGATATTCTTGATCGATTTTGTAGGATATGTAGAAATCTTTGTCGTTTTTACAGTGGATCCTCAAAAAAACATGTTTTGTATCATATAAAATATATACTTCGACTTTCGTGTGCCAGAACTTTGGCTCGTAAACATAAAAGTAC-AGTACGCACTTTGATGCGAAGATTAGGTTCGGGATTCTTAGAAAAATTCTT--------------------------GAGCTATCCCGACCATTTACTCTGCATCATCCTACCAGGATACTTG-TATCTATGTCAATGAAAAGAA-CTAAAAAA-----TCTTAACAA-------------------ATTGGACCTAA--CCCCT-GAATTT----------CTTAG-------ATC-TTCAAAAA----------GAAGAT---TTTCTTT-----------GCAAA-TTTAAGGATAATGATATGTACTGT-GAAAGA------------TTCAATAAC-GCAGATTACTTGAAC-------------------------ATATATGTTCAT-TTGTACAGGT--ATCATA---TCTAT------ACAAAT------------------GAAATTGTATTGTTGGATTGGAA------GAA-----GAT------------------ACGAGAA-----------------ATCCATT------GTTGAAAGAACAGAG--TGAATGAAATAAGAAAG-------ATATTGAATTT--------------TGTTTGAACC------------GAACCGCTGATGAAAAAAAAAG-------------------------------TAGG--------AATA------------------GTGAGGAA----------GTAAAAT-----GGGC-TTTTTCTTGGGGATAGAGGACCTACTAC---------AAACGGCTAATACTTTC-----TTTATATATTTTATAATTATATAAAATA--------------------------------------------------------------------------CTTTAG---CTTTTCTATCCG---------------AATTTTCG---------TTCTTTTTCATAAAAGAAAGTTCTC-CCCCGCCAATGAATGAAATGAATGATAAGT-GCC-----TAGGTT-CAGTATAGTAAT----------AGTATAAGATAAGTAAGA----------------------AAAGTCTAAGTCTTAGTATAATACCTATACTCTTA----------CTATAA-GAT------------AAAGACTCTTAAGGATAAGAC-----TTTTCAC----------ATGAATACTTAGTAGAACGACTAACGACGAGATTTATTATCGTTTCTCGCGTGTCTCGCGAAAGTTAGAGTAG-GTGCGAATTCTCCCAATTTGTGACCGACCATACGATCTGTTATAAAAATGGTAAA-TGTTCCTTTCCATTATGAATAGC-GATTGTATGGCCAATCATTGTGGGTA-TAATGGTAGATGCCCGAGACCAAGTCACTATTATTTCTTTCTCCTTCCTCATGTT-GAGTTTTTCAATTTTTCCCGCTAAATGATTAGCTACAAAAGGATTTTTTTTT-AGTGAACGTGTCAC-GGCTGATTACTCCTTTTTTTACATTTTT-TAAATTGGCATTCTATGTCCAA----------TATATCTATCTGAA-TCTT-AAG-TAT-GAAGGTAAGAATAAATACAATAAAATATTG-ATGAATGA-AAAAARGATAAAATCC----------TTTA-GCTAG-ATAA--------RGGA---GGGGC-GGATGTAG-CCAAGTGG-ATCAA-GGCAGY

Pachystoma_pubescens_PK12108 ACGACT-CTCGGCAATGGATATCTCGGCTCTCGCATCGATGAAGAGCGCAGCAAAATGCGATACGTGGTGCGAATTGCAGAATCCCGCGAACCATCGAGTCTTTGAACGCAAGTTGCGCCCGAGGCCAAT-CGGCCAAGGGCACGTCCGCCTGGGCGTCA-AGCGTCGCGTCGCTCCACAAGAATTCTTTTTCTTCTCATTTTTCT------------TCTCAAATGGTATCAGAAGGTTTTGGAGTCATTCTGGAAATTCCATTCTCGTCGCGATTAGTATTT---------TCCCTTGAAG---AAAAAAGAATACTAAAATCTCAGAATTTACGATCTATTCATTCAATATTTCCCTTTTTAGAGGATAAATTATCACATTTAAATTATGTGTCAGATCTACTAATACCCCATCCCATCCATCTGGAAATCTTGGTTCAAATCCTTCAATGCTGGATAAAAGATGTTCCCTCTTTGCATTTATTGCAATTGTTTTTCCACGAATATCAT---------------AATTTGAATAGTATC---------ATTACTTCAAATTCAAAGAAATC---------------CATTTACGTCTTTTCAAAAAG---AAAAAAAAGATTCTTTCGGTTCCTACATAATTCTTATGTATATGAATGCGAATATCTATTCCTTTTTCTTCGTAAAAAGTCTTCTTATTTACGATCAATATCTTCTGGAGTCTTTCTTGAGCGAACTCATTTCTATGGAAAAATAGAA------TATCTTATAGT---CGTGTGTTGTAATTCTTTTCAGAGG-GTCCTAGGGTTCCTCAAAGATACTTTAATACATTATGTTCGATATCAAGGAAAAGCGATTCTGGCTTCAAAAGGAACTCTTCTTCTGATGAAGAAATGGAAATTTCATCTTGTCAATTTTTGGCAATCTTATTTTCACTTTTGGTTTCAACCTTATAGGATCCATATAAAGCAATTACCCAACTATTCCTTTTCTTTTCT-GGGGTATTTTTCAAGTGTAC-TAAAAAAGCCTTTGGTAGTAAGAAATAAAATGCTAGAGTATTCCTTTCTAATAAATACTCTGACTAAGAAATTAGATACCATAACCCCAGTTATTTCTCTTATTGGATCCTTGTCGAAAGCTCAATTTTGTACTGTATTGGGTCATCCTATTAGTAAACCAACCTGGACCGATTTATCGGATTCTGATATTCTTGATCGATTTTGTCGGATATCTAGAAATCTTTGTCGTTATCACAGCGGATCTTCAAAGAAACAGGTTTTGTATCGTATAAAGTATATACTTCGACTTTCGTGTGCTAGAACTTTGGCTCGTAAACATAAAAGTAC-AGTACGTACTTTTATGGGAAGATTGGGTTCGGGATTCTTAGAAGAATTTTTTTTGGAAGA-----------------GAGCTATCCCGACCATTTCCCGTGCATCATCCTAGCAGAGTACTTA-TATCTATGTCAATGAAAAGAA-CTAAAAGATAAAATCTTAACAA-------------------ATTGGACCTAG--CCCCC-GAATTT----------CTTAG-------ATC-TTCAAAAA----------GAAGAC---ATTCTTT-----------GTAAA-TGTAAGGAAAAAGATATGGACTATGGAATGA------------TTCAATCCTTGAACATATCATGAACATATGAACATATCATATATATATATATATATA------TTGTA-----------------CTAT------ACAAACTATA----CAAAACAAAGGAGA--------TTGGATTGGAAGGAA--GAA-----GAT------------------ACGAGGATTTCT------ATTCGGATCCTTT------TGTGAAAGAACAGAG--TGAATGAAATGAGAAAG-------ATATTTCATTT--------------TGTTTAAACT------------GAGCCACTGATGGAAAGAGGAT-------------------------------GAGGATAAATA-AAGA------------------GCGAGGAA----------GTCAAAT-----GGGC-TTTTTATTGGGGATAGAGGGCCATCTAT---------AAATGTATAATACT--------TTTGTATTCA-ATGAATTTTTGAAGATAG--CAAT---------CCCCCAA-----------------------------------GATCTT--GGGGGATT-----------TCT--------------------------------------------------------------------------------------------------------TGGGTG-AAGTAT----------------AGTATAAGA-------------------------------AAAATATAAGTCTT-------------------------------------------------------------------------------------------------------CTTAGTAGAACGACTAACGACGAGATTTATTATCGTTTCTCGCGTGTCTCACTTTCGTGAGAGTAG-GTGCGAATTCTCCCAATTTGTGACCGACCATACGATCTGTGATATAAATGGTAAA-TGTTCCTTTCCATTATGAATAGC-GATTGTATGGCCAATCATTGTGGGTA-TAATGGTAGATGCCCGAGACCAAGTCACTATGATTTCTTTCTCCTCCCTCCTGTT-GAGTTTTTCAATTCTTCCCGATAAATGATTAGCTACAAAAGGATTTTTTTTT-AGTGAACGTGTCAC-GGCTGATTACTCCTTTTTTTACATTTTT-GAAATTGGCATTTTATGTCCAA----------TATCTCGATCTTAA-TCTG-AAG-TAT---------------------------AATG-ATGAATGG-AAAAAAGAGAAAATCC----------TTTA-GCTAG-ATAA--------GGG-AA-GGGGC-GGATGTAG-CCAAGTGG-ATCAA-GGCAGT

Paphiopedilum_purpuratum_SG1149 ACAACT-CTCAGCAACGGATATCTCGGCTCTTGCATCGATGAAGAACGCAGCGAAATGCGATAAATGGTGTGAATTGCAGAATCCCGTGAACCATCGAGTCTTTGAACGCAAGTTGCGCCCGAGGCCATC-AGGCCAAGGGCACGCCTGCCTGGGCATTG-CGAGTCATATCTCTCCACAAGAATTTTTTTTCTTCTCATTTTTCT------------TATCAAATGGTATCAGAAGGTTTTGGAGTCATTCTGGAAATTCCATTCTCGTCGCAATTAGTATCTTTTTCTGAATCTTCTGAAG---AAAAAAAAATACTAAAATATCAGAATTTACGATCTATTCATTCAATATTTCCCTTTTTAGAGGACAAATTTTTACATTTGAATTATGTGTCAGATCTACTAATACCTCATCCCATACATCTGGAAATCTTGGTTCAAGTACTTCAATGCTGGATCAAGGATGTTCCTTCTTTGCATTTATTGCGATTTCTTTTCCACGAATATCAT---------------AATTTGAATAGTCTC---------GTTACTT------CAAAGAAATT---------------CATTTACGCCTTTTCAAAAAG---AAAGAAAAAATTCCTTTGGTTCCTATATAATTCTTATGTATATGAATGCGAATATTTATTCCTATTTATTCGTAAACAATCTTCTTATTTACGATCAACATCCTCTGGAGTCTTTCTTGAGCGAACACATTTCTATGTAAAAATAGAG------CATCTTATAGT---AGTGTGTTGTAATTCTTTTCAGRAG-ATCCTATGCTTTCTCAAGGATACTTTCATGCATTATGTTCGATATCAAGGAAAGGCAATTCTAGCTTCAAAGGGAACTCTTATTCTGATGAATAAATGGAAATTTCATCTTGTGAATTTTTGGCAATCTTATTTTCACTTTTGGTCTCAACCGTATAGGATCCATATAAAGCAATTATCCAACTATTCCTTCTCTTTTCT-GGGGTATTTTTCAAGTGTAC-TAGAAAATCATTTGGTAGTAAGAAATAAAATGCTAGAGAATTCATTTATAATAAATATTATGACTCATAGATTATATACCATAGTCCCAGTCATGTCTCTTATTGGATCATTGGCGAAAGCTCAATTTTGTACTGTATTGGGTCATCCTATTAGTAAACCGATCTGGACCGATTTATCGGATTCTGATATTATTGATCGATTTTGCCGGATATGTAAAAATCTTTGTCATTATCACAGTGGATCCTCAAAAAAACAGGTTTTGTATCGTATAAAGTATATACTTCGACTTTCATGTGCTAGAACTTTGGCGCGGAAACATAAAAGTAC-AGTACGCACTTTTATGCGAAGATTAGGTTCGGGATTATTAGAAGAATTCTTTATGGAAGAAGAACAAGTTCTTTCTTGAGCTATCCCGACCATTTCCTGTGCATCATCCTAGCGGAGTACTTG-TATCTATGTCAATGAAAAGAA-CTAAAAAAG----TCTTAACAA-------------------ATTGTACCTAG--CTCCT-CAATTT----------CTTAG-------ATC-TTCAAAACAAAAA-----GAAGAC---TTTCTTTGTATT------GTAAA-TGTAAGGATAATGATATGGACTGT-GAATGA------------CTCAATAAC-GGGGATTCCTTGAA------------------------------------------------------------TCTAT------ACAAAT------------------GAAA--------TTGGATTGGAA------GAA-----GAA------------------ACGAGGATTTCT------ATTCGGATCCGTT------TGTGAAAGAACAGAG--TGAATGAAATGAGAAAG-------ATATTGAATTT--------------TGGTTGAACT------------GAACCATTGATGAAAAAAAAAA-------------------------------GAAGATAAAGA-AATA-------------------AAAGGAG----------GTAAAAT-----GGGC--TTTTCTTGGGGATA-----CCCTCTAC---------AAACGGATAATAC---------TTTGTACACATATGAATCTTTGAAGGTAA--AAAA---------TCCCC-ATATCT-TA------------------------CAAGATAT---GGGGATTTCTTTTG---CTTCTCTATCAG---------------AATTTTCG---------TTCTTTATCATAAAAGAAAGTTATC-CCCCGCC---------AATGAATGATAAGT-GCC-----TAGGTG-AAGTAT----------------AGTATAAGATAAGTCAGAAAAGTAAGAATAAGTAATA---AAAGTCTAAGTCTTAGTATA----CTATACTCTTA----------CTATAA-GAT------------AAAGACTCTTAAGGATAAGAC-----TTTTAAC----------ATGAATACTTAGTAGAACGACTAACGACGAGATTTATTATCGTTTCTTGCATGTCTCACGAAAGTTAGAGTAG-GTGCGAATTCTCCCAATTTGTGACCGACCATACGATCTGTTATATAAATGGTAAA-TGTTCCTTTCCATTATGAATAGC-GATTGTATGGCCAATCATTGTGGGTA-TAATGGTAGATGCCCGAGACCAAGTCACTATTATTTCTTTCTCCTCCCTCATGTT-GAGTTTTTCAATTTTTCCCGATAAATGATTAGCTACAAAAGGATTTTTTTTT-AGTGAACGTGTCAC-GGCTGATTACTCCTTTTTTTACATTTTA-AAAATTGGCATTCTATGTCCAA----------TATCTCGATCTTAA-TCTG-AAG-TAT-GAAGGTAAGAATCAATACAAT-----AATG-ATGAATGG-AAAAAAGAGAAAATCC----------TTTA-GCTAG-ATAA--------GGG-AA-GGGGC-GGATGTAG-CCAAGTGG-ATCAA-GGCAGT

Pecteilis_susannae_SG1292 AGGACT-CTCGGCAATGGATATCTTGGCTCTTGCATCGATGAAGAGCGCAGCGAAATGCGATACGTGGTGCGAATTGCAGAATCCCGTGAACCATCGAGTTTTTGAACGCAAGTTGCGCCTGAGGCCACC-TGGCCAAGGGCACGTCCACCTGGGCGTCA-AGCATTAAATCGCTCTATAAAAATTATTTTTCTTCTCATTTTTCT------------TATCAAATATTATCAGAAGGTTTTGGAGTTATTCTAGAAATTCCATTCTTGTCGCGATTAGTATCT---------TCCCTTGAAG---AAAAAAAAATAATAAAATCTCAGAATTTACGATCTATTCATTCAATCTTTCCTTTTTTAGAAGATAAATTCTTACATTTAAATTCTGTGTTAGATCTACTAATACCCCATCCCATCCATCTTGAAATCTTAGTTCAAATCCTTCAATGCTGGATCAAAGATGTTCCTTCTTTGCATTTGTTGCGATTGATTTTCCACGAATATCAT---------------AATTTAAATAGTTTC---------ATTACTT------CAAAGAAAGA---------------CATTTACGTCTTTTCAAAAAT---AAATAAAAGATTTTTTTTATTTTTACATAATTCTTATGTATATGAATGCGAATATACATTCCTGTTTCTTCGCAAACAGTCTTCTTATTTACGATCAACGTCTTTTGAAGTCTTTCTCGAGCGAACACATTTCTATAGAAAAATAGAA------TATTTTAGAGT---AATGTATTGTAATTCTTTTAAGAGG-ATTCTATGGTTCATCAAAGAACCTTTCATACATTATGTTCGATATCAAGGAAAAGCAATTCTGGCTTCAAAGGTAACTCTAATTCTGATGAATAAATGGAAATTTCATCTTGTTCATTTTTGGCAATTTTATTTTCACTTTTGGTCTCAACCTTATAGGATCCATATAAAGGAATTACCCAACTATTGCTTCTCTTTTCT-AGGGTATTTTTTAAGTGTAC-TAAAAAATACTTTGGTAGTAAGAAATCAAATGCTGGAGAATTTATATTTAATAAATACTCTGGCTAAGAAATTAGATACCATAGCCCCAGTTATTTCTCTTATTGGAGCATTGTCAAAAACTCAATTTTGTACTGTATTGGGCCATCCCATTAGCAAACCAATTTGGACTGATTTATCGGATTCTGATATTATTGATCGATTTTGTCGTATATGTAGAAATCTTTGTCATTATCACAGCGGATCCTCAAAAAAACAGGTTTTGTATCGTATAAAATATATACTTCGACTTTCGTGTGCTAGAACTTTGGCTCGTAAACATAAAAGTAC-AGTACGCACTTTTATGCGAAGATTAGGTTCGGGATTCTTAGAAGAATTTTTTATGGAAGAAGAACAAGCTCTTCTT-GAGCTATCCCGACTATTTTCCGTGCATCGTCCTAGCAGAGTACTTG-TATCTATGTCAATGAAAAGAA-CTAAAAAAGATAGTCTTAACAA-------------------ATTGGGCCCAGCCCTCTT--AATTT----------CTTAG-------ATC-TTCAAAAA----------GAAAAC---TTTCTTC-------------------------------------------------------------------------GATTCCTTGAAC-------------------------ATATATGTTCAT-TTGTGCAGGT--ATCGTG---TCTAT------ACAAAT------------------GAAA--------TTGGATTGGAATTGT-------------------------------ACGAAAATTTCT------ATTTGGATCTGTT------TGTTAAAGAA--------------------------------------------------------------------------------------------------------------------------------------------------------------------------------------------------------TCATTATGAAGGCTA-----CCATCTAC---------AAATGGATAGTACT--------GTTGTATTCATATGAATTATTGAAGATAT--AAAT---------CCCCCAATATCT-TGT---------------TCTTAAAACAAGATATT--GGGGGATTCTTTTG---CTTCTTTATCCGAATCCGATTTCTCCAAATTTTCG---------TTTTTTATCATAAAATAAAGTTCTCTCCCCGCCAATGAATGAAATGAATGATAAGT-GTC-----TAGGTG-AAGTAT----------------AGTATAAGATAAGTAAGA----------------------AAAGTCTAAGTCTTAGTATAATACCTATACTCTTA----------CTCTAA-GAT------------AAAGACTCTTAAGGATAAGAC-----TTTTCAC----------ATGAATACTTAGTATAACGACTAACGACGAGATTTATTATCGTTTCTCGCATGTCTCACGAAAGTGAGAGTAG-GTGCGAATTCTCCCAATTTGTGACCGACCATACGATCTGTTATATAAATGGTAAA-TGTTCCTTTCCATTATGAATAGC-GATTGTATGGCCAATCATTGTGGGTA-TAATGGTAGATGCCCGAGACCAAGTCACTATTAYTTCTTTCTCCTCCCTCATGTT-GAGTTTTTCAATTTTTCCCGATAAATGATTAGCTACAAAAGGATTTTTTTTT-AGTGAACGTGTCAC-GGCTGATTACTCCTTTTTTTACATYTTT-GAAATTGGCATTCTATGTCCAA----------TATCTCGATCTTAA-TCTG-AAG-TAT-GAGGGTAAGAATCAATACAAT-----AATG-ATGAATGG-MAAAAAGATAAAATCC----------TTTA-GCTAG-ATAA--------GGG-AA-GGGGC-GGATGTAG-CCAAGTGG-ATCAA-GGCAGT

Peristylus_calcaratus_SG1303 AGGACT-CTCGGCAATGGATATCTTGGCTCTTGCATCGATGAAGAGCGCAGCGAAATGCGATACGTGGTGCGAATTGCAGAATCCCGTGAACCATCGAGTATTTGAACGCAAGTTGCGCCCGAGGCCAGC-TGGTCGAGGGCACGTCCGCCTGGGCGTCA-AGCATTGAATCGCCCCATAAGAATTATTTTTCT------------------------TCTCAAATATTATCAGAAGGTTTTGGAGTCATTCTGGAAATTCCATTCTCGTCGCGATTAGTRTAT---------TCCCTAAAAA---AAAAAAAAATACTAAAATCTCAGAATTTACAATCTATTCATTCAATCTTTCCTTTTTTAGAAGATAAATTCTCACATTTAAATTATGTGTTAGATATACTAATACCCCATCCCATCCATCTGGAAATCTTAGTTCAAATCCTTCAATGCTGGATCAAAGATGTTCCTTCTTTGCATTTGTTGCGATTTATTTTCCACGAATATCAT---------------AATTTAAATAGTCTC---------ATTACTT------CAAAGAAAGA---------------CATTTACGTCTTTTCAAAAAT---AAATAAAAGATTTTTTTGGTTCTTACAGAATTCTTATGTATATGAATGCGAATATATATTCCTGTTTCTTCGCAAACAGTCTTCTTATTTACGATCAACGTCTTTTGAAGTCTTTCTTGAGCGAACACATTTCTATGGAAAAATAGAA------TATTTTAGAGT---AATGTATTGTAATTCTTTTCAGAGG-GTTCTATGGTTCCTCAAAGAACCTTTCATACATTATGTTCGATATCAAGGAAAAGCAATTCTGGCTTCAAAGGGAACTCTAATTCTGATGAAGAAATGGAAATTTCATCTTGTTCATTTTTGGCAATTTTATTTTTACTTTTGGTCTCAACCTTATAGGATCCATATAAAAGAATTACCCAACTATTCCTTCTCTTTTCT-GGGGTATTTTTTAAGTGTAC-TAAAAAATACTTTGATAGTAAGAAATCAAATGCTGGAGAATTCATATTTAATAAATACTCTAGCTAAAAAATTAKATACTATAGCCCCAGTTATTTATCTTATTGGAGCATTGTCAAAAGCTCAATTTTGTACTGTATTGGGCCATCCCATTAGTAAACCAATCTGGACCGATTTATCGGATTCTGATATTCTTGATCGATTTTGTCGTATATGTAGAAATCTTTGTCGTTATCACAGTGGATCCTCAAAAAAAAAGGTTTTATATCGTATAAAGTATATACTTCGACTTTCGTGTGCTAGAACTTTGGCTCGTAAACATAAAAGTAC-AGTACGCACTTTTATACGAAGATTAGGTTCGAGATTCTTAGAAGAATTTTTTATGGAAGAAGAACAAGCTCTTTCTTGAGCTATCCCGACCATTTTCCGTGCATCATCCTAGCAGAGTACTTG-TATCTATGTCAATGAAAAGAA-CTCAAAAAGAAAGTCTTAACAA-------------------ATTGGCT------CTCTT--AATTTATTATTT---CTTAT-------ATC-TTCAAAAA----------TAAGAC---TTTCTTT-------------------------------------------------------------------------GATTCTTTGAAC-------------------------ATATATGTTCAT-TTGTGCAGGT--ATCGTA---TCTAT------ACAAAT------------------TAAA--------TTGAATTGCAATTGGAAGAA-----TAT------------------ACGAAAATTTAT------ATTCGGATCCATT------TGTGAAAGAACAGAG-----------------------------------------------------------------------------------------------------------------------------------------------------------------TGA--------------------------GGAC----GTAGCCGGG--------CCATCTAT---------AAATGGATAGTACTATAGTACTATTGTATTCATACAAATTATTGAAGATAT--AAAT---------CCCTCAATATCT-TGT---------------TCTTATAACAAGATATT--GGGGGATTCWTTTG---CTTCTTTATCYGAATCCGATTTCTCTAAATTTTYG---------TTTTTTATCATAAAAGARAGTTCTC-CCCCGCC---------AATGAATGATAAGT-GTC-----TAGGTG-AAGTAT----------------AGTATAAGATAAGTAAGA----------------------AAAGTCTAAGTCTTAGTATA-----TATACTCTTA----------------------------------ARACTATTAAGGATAAGAC-----TTTTCAC----------ATGAATACTTAGCAGAACGACTAACGACGAGATTTATTATCGTTTCTCGCATGTCTCACGAAAGTGAGAGTAG-GTGCRAATTCTCCCAATTTGYGACCGACCATACGATCTGTTATATAAATGGTAAA-TGTTCCTTTCCATTAYGAATAGC-GATTGTATGGCCAATCATTGTGGGTA-TAATGGTAGATGCCCGAGACCAAGTCACTATTATTTCTTTCTCCTSCCTCATGTT-GAGTTTTTCAATTTTTCCCGATAAATGATTASCTACAAAAGGATTTTTTT---AGTGAACGTGGCAC-GGCTGATTACTCCNNNNTTTACATTTTT-TAAAYTGGCATTCTATGTCCAA----------TATCTCRATCTTAA-TCTG-AAG-TAT-GAGGGTAAGAATCAATACAAT-----AATG-ATGAATGG-AAAAAAGAGAAAATCC----------TTTA-GCTAG-ATAA--------GGG-AA-GGGGC-GGATGTAG-CCAAGTGG-ATCAA-GGCART

Peristylus_densus_SG1258 AGGACT-CTCGGCAATGGATATCTTGGCTCTCGCATCGATGAAGAGCGCAGCGAAATGCGATACGTGGTGCGAATTGCAGAATCCCGTGAACCATCGAGTATTTGAACGCAAGTTGCGCCCGAGGCCAGC-TGGCCGAGGGCACGTCCGACTGGGCGTCA-AGCATTGAATCGCCCCATAAGAATTATTTTTCT------------------------TCTCAAATATTATCAGAAGGTTTTGGAGTCATTCTGGAAATTCCATTCTCGTCGCGATTAGTATAT---------TCCCTTAAAG---AAAAAAAAAGACTAAAATCTCAGAATTTACAATCTATTCATTCAATCTTTCCTTTTTTAGAAGATAAATTCTCACATTTAAATTATGTGTTAGATATACTAATACCCCATCCCATCCATCTGGAAATCTTAGTTCAAATCCTTCAATGCTGGATCAAAGATGTTCCTTCTTTGCATTTGTTGCGATTTATTTTCCACGAATATCAT---------------AATTTAAATAGTCTC---------ATTACTT------CAAAGAAAGA---------------CATTTATGTCTTTTCAAAAAT---AAATAAAAGATTTTTTTGGTTCTTACAGAATTCTTATGTATATGAATGCGAATATATATTCCTGTTTCTTCGCAAACAGTCTTCTTATTTACGATCAACGTCTTTTGAAGTCTTTCTTGAGCGAACACATTTCTATGGAAAAATAGAA------TATTTTAGAGT---AATGTATTGTAATTCTTTTCAGAGG-GTTCTATGGTTCCTCAAAGAACCTTTCATACATTATGTTCGATATCAAGGAAAAGCAATTCTGGCTTCAAAGGGAACTCTAATTCTGATGAAGAAATGGAAATTTCATCTTGTTAATTTTTGGCAATTTTATTTTCACTTTTGGTCTCAACCTTATAGGATCCATATAAAAGAATTACCCAACTATTCCTTCTCTTTTCT-GGGGTATTTTTTAAGTGTAT-TAAAAAATACTTTGATAGTAAGAAATCAAATGCTGGAGAATTCATATTTAATAAATACTCTAGCTAAAAAATTAGATACTATAGCCCCAGTTATTTCTCTTATTGGAGCATTGTCAAAAGCTCAATTTTGTACTGTATTGGGCCATCCCATTAGTAAACCAATCTGGACCGATTTATCGGATTCTGATATTCTTGATCGATTTTGTCGTATATGTAGAAATCTTTGTCGTTATCACAGCGGGTCCTCAAAAAAACAGGTTTTATATCGTATAAAGTATATACTTCGACTTTCGTGTGCTAGAACTTTGGCTCGTAAACATAAAAGTAC-AGTACGCACTTTTATACGAAGATTAGGTTCGAGATTCTTAGAAGAATTTTTTATGGAAGAAGAACAAGCTCTTTCTTGAGCTATCCCGACCATTTTCCGTGCATCATCCTAGCAGAGTACTTG-TATCTATGTCAATGAAAAGAA-CTCAAAAAGAAAGTCTTATTTTTGAAGATAT---------------------------------------------------------------AAGAAAT----------AAGAC---TTTCTTT-------------------------------------------------------------------------GATTCTTTGAAC-------------------------ATATATGTTCAT-TTGTGCAGGT--ATCGTA---TCTAT------ACAAAT------------------TAAA--------TTGAATTGCAATTGGAAGAA-----TAT------------------ACGAAAATTTAT------ATTCGGATCCATT------TGTGAAAGAATAGAG-----------------------------------------------------------------------------------------------------------------------------------------------------------------TGAGGAA----------GTAAAAT-----GGGC-TTTTTATTGGGGATAG----CCATCTAT---------AAATGGATAGTACT--------ATTGTATTCATACGATTTATTGAAGATAT--AAAT---------CCCCCAATATCT-TGT---------------TCTTAGAACAAGATATT--AGGGGATTCTTTTG---CTTCTTTATCCGAATCCAATTTCTCTAAATTTTCG---------TTTTTTATCATAAAAGAAAGTTCTC-CCCCGCC---------AATGAATGATAAGT-GTC-----TAGGTG-AAGTAT----------------AGTATAAGATAAGTAAGA----------------------AAAGTCTAAGTCTTAGTATA----------TATTA----------------------------------AGACTATTAAGGATAAGAC-----TTTTCAC----------ATGAATACTTAGCAGAACGACTAACGACGAGATTTATTATCGTTTCTCGCATGTCTCACGAAAGTGAGAGTAG-GTGCGAATTCTCCCAATTTGTGACCGACCATACGATCTGTTATATAAATGGTAAA-TGTTCCTTTCCATTATGAATAGC-GATTGTATGGCCAATCATTGTGGGTA-TAATGGTAGATGCCCGAGACCAAGTCACTATTATTTCTTTCTCCTCCCTCATGTT-GAGTTTTTCAATTTTTCCCGATAAATGATTAGCTACAAAAGGATTTTTTTTT-AGTGAACGTGGCAC-GGCTGATTACTCCTTTTTTTACATTTTT-TAAATTGGCATTCTATGTCCAA----------TATCTCGATCTTAA-TCTG-AAG-TAT-GAGGGTAAGAATCAATACAAT-----AATG-ATGAATGG-AAAAAAGAGAAAATCC----------TTTA-GCTAG-ATAA--------GGG-AA-GGGGC-G-----------------------------

Peristylus_goodyeroides AGGACT-CTCGGCAATGGATATCTTGGCTCTCGCATCGATGAAGAGCGCAGCGAAATGCGATACGTGGTGCGAATTGCAGAATCCCGTGAACCATCGAGTTTTTGAACGCAAGTTGCGCCCGAGGCCAGCTTGGCCGAGGGCACGTCCGCCTGGGCGTCA-AGCATTGAATCGCCCC-----------------------------------------------------------------------------------------------------------------------------------------------------------------------------------------------------------------------------GATATACTAATACCCCATCCCATCCATCTGGAAATCTTAGTTCAAATCCTTCAATGCTGGATCAAAGATGTTCCTTCTTTGCATTTGTTGCGATTGATTTTCCACGAATATCAT---------------AATTTAAAAAGTCTC---------ATTAATT------CAAAGAAAAA---------------CATTTACGTCTTTTCAAAAAT---AAAGAAAAGATTTTTTTGGTTCTTACAGAATTCTTATGTATATGAATGCGAATATATATTCCTGTTTCTTCGCAAACAGTCTTCTTATTTACGATCAACGTCTTTTGAAGTCTTTCTTGAGCGAACACATTTCTATGGAAAAATAGAA------TATTTTATAGT---AATGTATTGTAATTCTTTTCAGAGG-GTTCTATGGCTCCTCAAAGAACCTTTCATACATTATGTTCGATATCAAGGAAAAGCAATTCTGGCTTCAAAGGGAACTCTAATTCTGATGAAGAAATGGAAATTTCATTTTGTTCATTTTTGGCAATTTTATTTTCACTTTTGGTCTCAACCTTATAGGATCCATATAAAAGAATTACCCAACTATTCCTTCTCTTTTCT-GGGGTATTTTTTAAGTGTAC-TAAAAAATACTTTGGTAGTAAGAAATCAAATGCTGGAGAATTCATATTTAATAAATACTCGAGTTAAAAAATTAGATACTATAGCCCCAGTTATTTCTCTTATTGGAGCATTATCAAAAGCTCAATTTTGTACTGTATTGGGCCATCCCATTAGTAAACCAATCTGGACCGATTTCTCGGATTCTGATATTCTTGATCGATTTTGTCGTATATGTAGAAATCTTTGTCGTTATCACAGCGGATCCTCAAAAAAACAGGTTTTGTATCGTATAAAGTATATACTTCGACTTTCGTGTGCTAGAACTTTGGCTCGTAAACATAAAAGTAC-AGTACGCACTTTTATACGAAGATTAGGTTCGGGATTATTAGAAGAATTTTTTATGGAAGAAGAACAAGCTCTTTCTT---------------TTTTCCGTGCATCATCCTAGCAGAGTACTTG-TATCTATGTCAATGAAAAGAA-CTAAAAAAGAAAGTCTTAACAG-------------------ATTGGCC------CTCTT--AATTT----------CTTAG-------ATC-TTAAAAAA----------GAAGAC---TTTCTTT-------------------------------------------------------------------------GATTCTTTGAAC-------------------------ATATATGTTCAT-TTGTGCAGGT--ATCGTA---TCTAT------ACAAAT-----GAACAAAT-----GAAA--------TTGGATTGCAATTGGAAAAA-----TAT------------------ACGAAAATTTAT------ATTCGGATCCATT------TGTGAAAGAACAGAG-----------------------------------------------------------------------------------------------------------------------------------------------------------------TGAGGAA----------GTAAAAT-----GGGC-TTTTTATTGGGGATAGAGGG---------------------------TACT--------GTTGTATTCATACGAATTGTTGAAGATAT--AAAT-------CCCTCCCAATATCT-TGT------------------------------------------CTTTTG---CTTCTTTATCCGAATCCGATTTCTCCAAATTTTCG---------TTTTTTTTCATAAAATAAAGTTCTC-CCCCGCC---------AATGAATGATA-----TC-----TAGGTG-AAGTAT----------------AGTATAAGATAAGTAAGA----------------------AAAGTCTAAGTCTTAGTATA-----TATACTCTTA----------------------------------AGACTATTAAGGATAAGAC------------------------------TTAGCAGAACGACTAACGACGAGATTTATTATCGTTTCTCGCATGTCTCACGAAAGTGAGAGTAG-GTGCGAATTCTCCCAATTTGTGACCGACCATACGATCTGTTATATAAATGGTAAA-TGTTCCTTTCCATTATGAATAGC-GATTGTATGGCCAATCATTGTGGGTA-TAATGGTAGATGCCCGAGACCAAGTCACTATTATTTCTTTCTCCTCCCTCATGTT-GAGTTTTTCAATTTTTCCCGATAAATGATTAGCTACAAAAGGATTTTTTTTT-AGTGAACGTGTCAC-GGCTGATTACTCCTTTTTTTACATTTTT-GAAATTGGCATTCTATGTCCAA----------TATCTCGATCTTAA-TCTG-AAG-TAT-GAGGGTAAGAATCAATACAAT-----AATG-ATGAATGG-AAAAAAGAGAAAATCC----------TTTA-GCTAG-ATAA---------------------------------------------------

Peristylus_intrudens_SG1298 AGGACT-CTCGGCAATGGATATCTTGGCTCTCGCATCGATGAAGAGCGCAGCGAAATGCGATACGTGGTGCGAATTGCAGAATCCCGTGAACCATCGAGTTTTTGAACGCAAGTTGCGCCCGAGGCCAGC-TGGCCGAGGGCACGTCCGCCTGGGCGTCA-AGCATTGAATCGCCCCATAAGAATTATTTTTCT------------------------TCTCAAATATTATCAGAAGGTTTTGGAGTCATTCTGGAAATTCCATTCTCGTCGCGATTAGTATAT---------TCCCTTAAAG---AAAAAAAAATACTAAAATCTCAGAATTTACGATCTATTCATTCAATCTTTCCTTTTTTAGAAGATAAATTCTCACATTTAAATTATGTGTTAGATATACTAATACCCCATCCCATCCATCTGGAAATCTTAGTTCAAATCCTTCAATGCTGGATCAAAGATGTTCCTTCTTTGCATTTGTTGCGATTTATTTTCCACGAATATCAT---------------AATTTAAATAGTCTC---------ATTACTT------CAAAGAAAGA---------------CATTTACGTCTTTTCAAAAAT---AAATAAAAGATTTTTTTGGTTCTTACAGAATTCTTATGTATATGAATGCGAATATATATTCCTGTTTCTTCGCAAACAGTCTTCTTATTTACGATCAACGTCTTTTGAAGTCTTTCTTGAGCGAACACATTTCTATGGAAAAATAGAA------TATTTTAGAGT---AATGTATTGTAATTCTTTTCAGAGG-GTTCTATGGTTCCTCAAAGAACCTTTCATACATTATGTTCGATATCAAGGAAAAGCAATTCTGGCTTCAAAGGGAACTCTAATTCTGATGAAGAAATGGAAATTTCATCTTGTTCATTTTTGGCAATTTTATTTGCACTTTTGGTCTCAACCTTATAGGATCCATATAAAAGAATTACCCAACTATTCCTTATCTTTTCT-GGGGTATTTTTTAAGTGTAC-TAAAAAATACTTTGGTAGTAAGAAATCAAATGCTGGAGAATTCATATTTAATAAATACTCTAGCTAAAAAATTAGATACTATAGCCCCAGTTATTTCTCTTATTGGAGCATTGTCAAAAGCTCAATTTTGTACTGTATTGGGCCATCCCATTAGTAAACCAATCTGGACCGATTTATCGGATTCTGATATTCTTGATCGATTTTGTCGTATATGTAGAAATCTTTGTCGTTATCACAGTGGGTCCTCAAAAAAACAGGTTTTATATCGTATAAAGTATATACTTCGACTTTCGTGTGCTAGAACTTTGGCTCGTAAACATAAAAGTAC-AGTACGCACTTTTATACGAAGATTAGGTTCGGAATTCTTAGAAGAATTTTTTATGGAAGAAGAACAAGCTCTTTCTTGAGCTATCCCGACCATTTTCCGTGCATCATCCTAGCAGAGTACTTG-TATCTATGTCAATGAAAAGAA-CTCAAAAAGAAAGTCTTAACAA-------------------ATTGGCT------CTCTT--AATTTATTATTT---CTTAT-------ATC-TTCAAAAA----------TAAGAC---TTTCTTT-------------------------------------------------------------------------GATTCTTTGAAC-------------------------ATATATGTTCAT-TTGTGCAGGT--ATCGTA---TCTAT------AAAAAT------------------GAAA--------TTGAATTGCAATTGGAAGAA-----TAT------------------ACGAAAATTTAT------ATTCGGATCCATT------TGTGAAAGAACAGAG-----------------------------------------------------------------------------------------------------------------------------------------------------------------TGAGGAA----------GTAAAAT-----GGGC-TTTTTATTGGGGATAGAGGGCCATCTAT---------AAATGGATAGTACT--------GTTGTATTCATACGAATTATTGAAGATAT--AAAT---------CCCCCAATATCT-TGT---------------TCTTAGAACAAGATATTGGGGGGGATTCTTTTG---CTTCTTTATCCGAATCCGATTTCTCTAAATTTTCG---------TTTTTTATCATAAAAGAAAGTTCTC-CCCCGCC---------AATGAATGATAAGT-GTC-----TAGGTG-AAGTAT----------------AGTATAAGATAAGTAAGA----------------------AAAGTCTAAGTCTTAGTATA-----TATACTCTTA----------------------------------AGACTATTAAGGATAAGAC-----TTTTAAC----------ATGAATACTTAGCAGAACGACTAACGACGAGATTTATTATCGTTTCTCGCATGTCTCACGAAAGTGAGAGTAG-GTGCGAATTCTCCCAATTTGTGACCGACCATACGATCTGTTATATAAATGGTAAA-TGTTCCTTTCCATTATGAATAGC-GATTGTATGGCCAATCATTGTGGGTA-TAATGGTAGATGCCCGAGACCAAGTCACTATTATTTCTTTCTCCTCCCTCATGTT-GAGTTTTTCAATTTTTCCCGATAAATGATTAGCTACAAAAGGATTTTTTTTT-AGTGAACGTGTCAC-GGCTGATTACTCCTTTTTTTACATTTTT-TAAATTGGCATTC--------------------ATCTCGATCTTAA-TCTG-AAG-TAT-GAGGGTAAGAATCAATACAAT-----AATG-ATGAATGGAAAAAAAGATAAAATCC----------TTTA-CCTAGTATAA--------GGA-AAGGGGGCGGGATTTAG-CCAAGTGA-ATCAA-GGCAGT

Peristylus_lacertifer_SG1006 AGGACT-CTCGGCAATGGATATCTTGGCTCTCGCATCGATGAAGAGCGCAGCGAAATGCGATACGTGGTGCGAATTGCAGAATCCCGTGAACCATCGAGTTTTTGAACGCAAGTTGCGCCCGAGGCCAGC-TGGCCGAGGGCACGTCCGCCTGGGCGTCA-AGCATTGAATCGCCCC------ATTATTTTTCT------------------------TCTCAAATATTATCAGAAGGTTTTGGAGTCATTCTGGAAATTCCATTCTCGTCGCGATTAGTATAT---------TCCCTTAAAG---AAAAAAAAATACTAAAATCTCAGAATTTACGATCTATTCATTCAATCTTTCCTTTTTTAGAAGATAAATTCTCACATTTAAATTATGTGTTAGATATACTAATACCCCATCCCATCCATCTGGAAATCTTAGTTCAAATCCTTCAATGCTGGATCAAAGATGTTCCTTCTTTGCATTTGTTGCGATTTATTTTCCACGAATATCAT---------------AATTTAAATAGTCTC---------ATTACTT------CAAAGAAAGA---------------CATTTACGTCTTTTCAAAAAT---AAATAAAAGATTTTTTTGGTTCTTACAGAATTCTTATGTATATGAATGCGAATATATATTCCTGTTTCTTCGCAAACAGTCTTCTTATTTACGATCAACGTCTTTTGAAGTCTTTCTTGAGCGAACACATTTCTATGGAAAAATAGAA------TATTTTAGAGT---AATGTATTGTAATTCTTTTCAGAGG-GTTCTATGGTTCCTCAAAGAACCTTTCATACATTATGTTCGATATCAAGGAAAAGCAATTCTGGCTTCAAAGGGAACTCTAATTCTGATGAAGAAATGGAAATTTCATCTTGTTCATTTTTGGCAATTTTATTTGCACTTTTGGTCTCAACCTTATAGGATCCATATAAAAGAATTACCCAACTATTCCTTATCTTTTCT-GGGGTATTTTTTAAGTGTAC-TAAAAAATACTTTGGTAGTAAGAAATCAAATGCTGGAGAATTCATATTTAATAAATACTCTAGCTAAAAAATTAGATACTATAGCCCCAGTTATTTCTCTTATTGGAGCATTGTCAAAAGCTCAATTTTGTACTGTATTGGGCCATCCCATTAGTAAACCAATCTGGACCGATTTATCGGATTCTGATATTCTTGATCGATTTTGTCGTATATGTAGAAATCTTTGTCGTTATCACAGTGGGTCCTCAAAAAAACAGGTTTTATATCGTATAAAGTATATACTTCGACTTTCGTGTGCTAGAACTTTGGCTCGTAAACATAAAAGTAC-AGTACGCACTTTTATACGAAGATTAGGTTCGGAATTCTTAGAAGAATTTTTTATGGAAG------------------GAGCTATCCCGACCATTTTCCGTGCATCATCCTAGCAGAGTACTTG-TATCTATGTCAATGAAAAGAA-CTCAAAAAGAAAGTCTTAACAA-------------------ATTGGCT------CTCTT--AATTTATTATTT---CTTAT-------ATC-TTCAAAAA----------TAAGAC---TTTCTTT-------------------------------------------------------------------------GATTCTTTGAAC-------------------------ATATATGTTCAT-TTGTGCAGGT--ATCGTA---TCTAT------ACAAAT------------------GAAA--------TTGAATTGCAATTGGAAGAA-----TAT------------------ACGAAAATTTAT------ATTCGGATCCATT------TGTGAAAGAACAGAG-----------------------------------------------------------------------------------------------------------------------------------------------------------------TGAGGAA----------GTAAAAT-----GGGC-TTTTTATTGGGGATA-----CCATCTAT---------AAATGGATAGTACT--------GTTGTATTCATACGAATTATTGAAGATAT--AAAT---------CCCCCAATATCT-TGT---------------TCTAAGAACAAGATATTGGGGGGGATTCTTTTG---CTTCTTTATCCGAATCCGATTTCTCTAAATTTTCG---------TTTTTTATCATAAAAGAAAGTTCTC-CCCCGCC---------AATGAATGATAAGT-GTC-----TAGGTG-AAGTAT----------------AGTATAAGATAAGTAAGA----------------------AAAGTCTAAGTCTTAGTATA-----TATACTCTTA----------------------------------AGACTATTAAGGATAAGAC-----TTTTAAC----------ATGAATACTTAGCAGAACGACTAACGACGAGATTTATTATCGTTTCTCGCATGTCTCACGAAAGTGAGAGTAG-GTGCGAATTCTCCCAATTTGTGACCGACCATACGATCTGTTATATAAATGGTAAA-TGTTCCTTTCCATTATGAATAGC-GATTGTATGGCCAATCATTGTGGGTA-TAATGGTAGATGCCCGAGACCAAGTCACTATTATTTCTTTCTCCTCCCTCATGTT-GAGTTTTTCAATTTTTCCCGATAAATGATTAGCTACAAAAGGATTTTTTTTT-AGTGAACGTGTCAC-GGCTGATTACTCCTTTTTTTACATTTTT-TAAATTGGCATTC--------------------ATCTCGATCTTAA-TCTG-AAG-TAT-GAGGGTAAGAATCAATACAAT-----AATG-ATGAATGG-AAAAAAGATAAAATCC----------TTTA-GCTAG-ATAA--------GGG-AA-GGGGC-GGATGTAG-CCAAGTGG-ATCAA-GGCAGT

Persitylus_tentaculatus_SG1007 AGGACT-CTCGGCAATGGATATCTTGGCTCTCGCATCGATGAAGAGCGCAGCGAAATGCGATACGTGGTGCGAATTGCAGAATCCCGTGAACCATCGAGTTTTTGAACGCAAGTTGCGCCCGAGGCCAGC-TGGCCGAGGGCACGTCCGCCTGGGCGTCA-AGCATTGAATCGCCCCATAAGAATTATTTTTCT------------------------TCTCAAATATTATCAGAAGGTTTTGGAGTCATTCTGGAAATTCCATTCTCGTCGCGATTAGTATAT---------TCCCTTAAAG---AAAAAAAAATACTAAAATCTCAGAATTTACAATCTATTCATTCAATCTTTCCTTTTTTAGAAGATAAATTATCACATTTAAATTATGTGTTAGATATACTAATACCCCATCCCATCCATCTGGAAATCTTAGTTCAAATCCTTCAATGCTGGATCAAAGATGTTCCTTCTTTGCATTTGTTGCGATTTATTTTCCACGAATATCAT---------------AATTTAAATAGTCTC---------ATTACTT------CAAAGAAAGA---------------CATTTACGTCTTTTCAAAAAT---AAATAAAAGATTTTTTTGGTTCTTACAGAATTCTTATGTATATGAATGCGAATATATATTCCTGTTTCTTCGCAAACAGTCTTCTTATTTACGATCAACGTCTTTTGAAGTCTTTCTTGAGCGAACACATTTCTATGGAAAAATAGAA------TATTTTAGAGT---AATGTATTGTAATTCTTTTCAGAGG-GTTCTATGGTTCCTCAAAGAACCTTTCATACATTATGTTCGATATCAAGGAAAAGCAATTCTGGCTTCAAAGGGAACTCTAATTCTGATGAAGAAATGGAAATTTCATCTTATTCATTTTTGGCAATTTTATTTGCACTTTTGGTCTCAACCTTATAGGATCCATATAAAAGAATTACCCAACTATTCCTTCTCTTTTCT-GGGGTATTTTTTAAGTGTAC-TAAAAAATACTTTGGTAGTAAGAAATCAAATGCTGGAGAATTCATATTTAATAAATACTCTAGCTAAAAAATTAGATACTATAGCCCCAGTTATTTCTCTTATTGGAGCATTGTCAAAAGCTCAATTTTGTACTGTATTGGGCCATCCCATTAGTAAACCAATCTGGACCGATTTATCGGATTCTGATATTCTTTATCGATTTTGTCGTATATGTAGAAATCTTTGTCGTTATCACAGTGGGTCCTCAAAAAAACAGGTTTTATATCGTATAAAGTATATACTTCGACTTTCGTGTGCTAGAACTTTGGCTCGTAAACATAAAAGTAC-AGTACGCACTTTTATACGAAGATTAGGTTCGGAATTCTTAGAAGAATTTTTTATGGAAGAAGAACCAGCTCTTCTT-GAGCTATCCCGACCATTTTCCGTGCATCATCCTAGCAGAGTACTTG-TATCTATGTCAATGAAAAGAA-CTCAAAAAGAAAGTCTTA------------------------------------TTTTT-GAAGAT----------ATAAGAAATAATAAA-TTAAGAGAGCCAATTTGTTAAGAC---TTTCTTT-------------------------------------------------------------------------GATTCTTTGAAC-------------------------ATATATGTTCAT-TTGTGCAGGT--ATCGTA---TCTAT------ACAAAT------------------GAAA--------TTGAATTGCAATTGGAAGAA-----TAT------------------ACGAAAATCTAT------ATTCGGATCCATT------TGTGAAAGAACAGAG-----------------------------------------------------------------------------------------------------------------------------------------------------------------TGAGGAA----------GTAAAAT-----GGGC-TTTTTATTGGGGATAGAGGGCCATCTAT---------AAATGGATAGTACT--------GTTGTATTCATATGAA-----GAAGATAT--AAAT---------CCCCCAATATCT-TGT---------------TCTAAGAACAAGATATT--GGGGGATTCTTTTG---CTTCTTTATCCGAATCCGATTTCTCTAAATTTTCG---------TTTTTTATCATAAAATAAAGTTCTC-CCCCGCC---------AATGAATGATAAGT-GTC-----TAGGTG-AAGTAT----------------AGTATAAGATAAGTAAGA----------------------AAAGTCTAAGTCTTAGTATA-----TATACTCTTA----------------------------------AGACTATTAAGGATAAGAC-----TTTTCAC----------ATGAATACTTAGCAGAACGACTAACGACGAGATTTATTATCGTTTCTCGCATGTCTCACGAAAGTGAGAGTAG-GTGCGAATTCTCCCAATTTGTGACCGACCATACGATCTGTTATATAAATGGTAAA-TGTTCCTTTCCATTATGAATAGC-GATTGTATGGCCAATCATTGTGGGTA-TAATGGTAGATGCCCGAGACCAAGTCACTATTATTTCTTTCTCCTCCCTCATGTT-GAGTTTTTCAATTTTTCCCGATAAATGATTAGCTACAAAAGGATTTTTTTTT-AGTGAACGTGTCAC-GGCTGATTACTCCTTTTTTTACATTTTT-TAAATTGGCATTC--------------------ATCTCGATCTTAA-TCTG-AAG-TAT-GAGGGTAAGAATCAATACAAT-----AATG-ATGAATGG-AAAAAAGAGAAAATCC----------TTTA-GCTAG-ATAA--------GGG-AA-GGGGC-GGATGTAG-CCAAGTGG-ATCAA-GGCAGT

Phaius_tankervilleae_PK12084 ATGACT-CTCGGCAATGGATATCTCGGCTCTCGCATCGATGAAGAGCGCAGCGAAATGCGATACGTGGTGCGAATTGCAGAATCCCGCGAACCATCGAGTCTTTGAACGCAAGTTGCGCCCGAGGTCAAC-CGGCCAAGGGCACGTCTGCCTGGGCGTCA-AGCGTTGCATCGCTCTACAAGAATTCTTTTTCTTCTCATTTTTCT------------TCTCAAATGGTATCAGAAGGTTTTGCAGTCATTCTGGAAATTCCATTCTCGTCGCGATTAGTATCT---------TCTCTTGAAG---AAAAAAGAATACCAAAATCTCAGAATTTACGATCTATTCATTCAATATTTCCCTTTTTAGAGGATAAATTATCACATTTAAATTATGTGTCAGATCTACTAATACCCCATCCCATCCATCTGGAAATCTTGGTTCAAATCCTTCAATGCTGGATCAAAGATGTTCCTTCTTTGCATTTCTTGCGATTGTTATTCCACGAATATCAT---------------AATTTGAATAGTATC---------CTTACTT------CAAAGAAATC---------------CATTTACGTCTTTTCAAAAAG---AAAGAAAAGATTATTTTGGTTCCTACATAATTCTTATGTATATGAATGCGAATATCTATTCCTGTTTATTCGTAAACAGTCTTCTTATTTACGATCAATATCTTCTGGAGTCTTTCTTGAGCGAACACATTTCTATGGAAAAATAGAA------TATCTTATAGT---CGTGTGTTGTAATTTTTTTCAGAGT-ATCCTATGGTTCCTCAAAGATACTTTCATACATTATGTTCGATATCAAGGAAAAGCGATTCTGGCTTCAAAAGGAACTTTTATTCTGATGAAGAAATGGAAATTTCATCTTGTAAATTTTTGGGAATCTTATTTTCACTTTTGGTTTCAACCTTATAGAATCCATATAAAAAAATTACCCAACTATTCCTTCTCTTTTCT-GGGGTATTTTTCAAGTGTAC-TAAAAAATCCTTTGGTAGTAAGAAATCAAATGCTAGAGAATTCATTTCTAATAAATACTCTGACTAAGAAATTAGATACCATAGCCCCAGTTATTTCTCTTATTGGATCATTGTCGAAAGCTCAATTTTGTACTGTATTGGGTCATCCTATTAGTAAACCGATCTGGACCGATTTATCGGATTCTGATATTCTTGATCGATTTTGTCGGATATATAGAAATCTTTGTCGTTATCACAGCGGATCCTCAAAGAAACAGGTTTTGTATCGTATAAAGTATATACTTCGACTTTCGTGTGCTAGGACTTTGGCTCGTAAACATAAAAGTAC-AGTACGCACTTTTATGCGAAGATTAGGTTCGGGATTCTTAGAAGAATTTTTTTTGGAAGAAGAACAATCTC------GAGCTATCCCGACCATTTCTCGCGCATCATCCTAGCAGAGTACTTA-TATCTATGTCAATGAAAAGAA-TTAAAAAATAAAATATTAACAA-------------------ATTAGACCTAG--CCCCT-GAATTT----------ATTAG-------ATC-TTCAAAAA----------GAAGAC---ATTCTTT-----------GTAAA-TGTCAGGAAAAAGATATGGACTAT-GAATGA------------TTCAATAAC-GGAAATTCCTCGAAC---------------ACTTGAACATATATATGTTCAT-------------ATCGTA----CTAT------ACAAAA--------CAAAT-----AAGA--------TTGGATTGGAA------GAA-----GAT------------------ACGAGGATTTTG------ATTCGGATCCATT------TGTGAAAGAACAGAG--TGAATGAAATGAGAAAG-------ATATTTCATTT--------------TGGTTAAACT------------GAACCACTGATGGAAAGAGGAT-------------------------------GAGGATAAATA-AAGA------------------GTGAGGAA----------GTAAAAT-----GGGC-TTTTTATTG-----------CCATCTAT---------AAATGGATAATACT--------TTTGTATTCATATGAATTTTTGAAGATAG--CAAT---------CCCCCAA-----------------------------------GATCTT--GGGGGATTCTTTTG---CTTCTCTATCCG---------------AATTTTCG---------TTCTTTATCAT----AAAAGTTCTC-CCCCGCC---------AATGAATGATAAGT-GCC-----TAGGTG-AAGTAT----------------AGTATAAGATAAGTCAGA----------------------AAAGTATAAGTCTTATTAGYATACCTATACTCTTA----------CTATAA-GAT------------AAAGACTCTTAAGGATAAGGC-----TTTTCAC----------ATGAATACTTAGTAGAACGACTAACGRCGAGATTTATTATCGTTTCTCGCGTGTCTCACGAAAGTGAGAGTAG-GTGCGAATTCTCCCAATTTGTGACCGACCATACGATCTGTGATAKRAATGGTAAA-TGTTCCTTTCCATTATGAATAGC-GATTGTATGGCCAATCATTGTGGGTA-TAATGGTAGATGCCCGAGACCAAGTCACTATGATTTCTTTCTCCTCCCTCCTGTT-GAGTTTTTCAATTCTTCCCGATAAATGATTAGCTACAAAAGGATTTTTTTTT-AGTGAACGTGTCAC-GGCTGATTACTCCTTTTTTTCCATTTTT-MAAATTGGCATTCTATGTCCAA----------TATMTCGATCTKAA-TCTG-AAG-TAG---------------------------AATK-AKGAATGG-AAAAAAKAGAAAATCC----------TTTA-GCTAG-AT-----------------------------------------------------

Phaius_wallichii_KFBG2002A ATGACT-CTCGGCAATGGATATCTCGGCTCTCGCATCGATGAAAAGCGCAGCGAAATGCGATACGTGGTGCGAATTGCAGAATCCCGCGAACCATCGAGTCTTTGAACGCAAGTTGCGCCCGAGGTCAAC-CGGCCAAGGGCACGTCTGCCTGGGCGTCA-AGCGTTGCATCGCTCTACAAGAATTCTTTTTCTTCTCATTTTTCT------------TCTCAAATGGTATCAGAAGGTTTTGCAGTCATTCTGGAAATTCCATTCTCGTCGCGATTAGTATCT---------TCTCTTGAAG---AAAAAAGAATACCAAAATCTCAGAATTTACGATCTATTCATTCAATATTTCCCTTTTTAGAGGATAAATTATCACATTTAAATTATGTGTCAGATCTACTAATACCCCATCCCATCCATCTGGAAATCTTGGTTCAAATCCTTCAATGCTGGATCAAAGATGTTCCTTCTTTGCATTTCTTGCGATTGTTATTCCACGAATATCAT---------------AATTTGAATAGTATC---------CTTACTT------CAAAGAAATC---------------CATTTACGTCTTTTCAAAAAG---AAAGAAAAGATTATTTTGGTTCCTACATAATTCTTATGTATATGAATGCGAATATCTATTCCTGTTTATTCGTAAACAGTCTTCTTATTTACGATCAATATCTTCTGGAGTCTTTCTTGAGCGAACACATTTCTATGGAAAAATAGAA------TATCTTATAGT---CGTGTGTTGTAATTTTTTTCAGAGT-ATCCTATGGTTCCTCAAAGATACTTTCATACATTATGTTCGATATCAAGGAAAAGCGATTCTGGCTTCAAAAGGAACTTTTATTCTGATGAAGAAATGGAAATTTCATCTTGTAAATTTTTGGGAATCTTATTTTCACTTTTGGTTTCAACCTTATAGAATCCATATAAAAAAATTACCCAACTATTCCTTCTCTTTTCT-GGGGTATTTTTCAAGTGTAC-TAAAAAATCCTTTGGTAGTAAGAAATCAAATGCTAGAGAATTCATTTCTAATAAATACTCTGACTAAGAAATTAGATACCATAGCCCCAGTTATTTCTCTTATTGGATCATTGTCGAAAGCTCAATTTTGTACTGTATTGGGTCATCCTATTAGTAAACCGATCTGGACCGATTTATCGGATTCTGATATTCTTGATCGATTTTGTCGGATATATAGAAATCTTTGTCGTTATCACAGCGGATCCTCAAAGAAACAGGTTTTGTATCGTATAAAGTATATACTTCGACTTTCGTGTGCTAGGACTTTGGCTCGTAAACATAAAAGTAC-AGTACGCACTTTTATGCGAAGATTAGGTTCGGGATTCTTAGAAGAATTTTT--------------------------GAGCTATCCCGACCATTTCTCGCGCATCATCCTAGCAGAGTACTTA-TATCTATGTCAATGAAAAGAA-TTAAAAAATAAAATATTAACAA-------------------ATTAGACCTAG--CCCCT-GAATTT----------ATTAG-------ATC-TTCAAAAA----------GAAGAC---ATTCTTT-----------GTAAA-TGTCAGGAAAAAGATATGGACTAT-GAATGA------------TTCAATAAC-GGAAATTCCTCGAAC---------------ACTTGAACATATATATGTTCAT-------------ATCGTA----CTAT------ACAAAA--------CAAAT-----AAGA--------TTGGATTGGAA------GAA-----GAT------------------ACGAGGATTTTG------ATTCGGATCCATT------TGTGAAAGAACAGAG--TGAATGAAATGAGAAAG-------ATATTTCATTT--------------TGGTTAAACT------------GAACCACTGATGGAAAGAGGAT-------------------------------GAGGATAAATA-AAGA------------------GTGAGGAA----------GTAAAAT-----GGGC-TTTTTATTGGGGATAGAGGACCATCTAT---------AAATGGATAATACT--------TTTGTATTCATATGAATTTTTGAAGATAG--CAAT--------CCCCCCAA-----------------------------------GATCTT--GGGGGATTCTTTTG---CTTCTCTATCCG---------------AATTTTCG---------TTCTTTATCAT----AAAAGTTCTC-CCCCGCC---------AATGAATGATAAGT-GCC-----TAGGTG-AAGTAT----------------AGTATAAGATAAGTCAGA----------------------AAAGTATAAGTCTTATTAGTATACCTATACTCTTA----------CTATAA-GAT------------AAAGACTCTTAAGGATAAGGC-----TTTTCAC----------ATGAATACTTAGTAGAACGACTAACGACGAGATTTATTATCGTTTCTCGCGTGTCTCACGAAAGTGAGAGTAG-GTGCGAATTCTCCCAATTTGTGACCGACCATACGATCTGTGATATAAATGGTAAA-TGTTCCTTTCCATTATGAATAGC-GATTGTATGGCCAATCATTGTGGGTA-TAATGGTAGATGCCCGAGACCAAGTCACTATGATTTCTTTCTCCTCCCTCCTGTT-GAGTTTTTCAATTCTTCCCGATAAATGATTAGCTACAAAAGGATTTTTTTTT-AGTGAACGTGTCAC-GGCTGATTACTCCTTTTTTTCCATTTTT-AAAATTGGCATTCTATGTCCAA----------TATCTCGATCTTAA-TCTG-AAG-TAT---------------------------AATG-ATGAATGG-AAAAAAGAGAAAATCCTTTAGCTAGATTTA-GCTAG-ATAA--------GG--AA-GGGGC-------------------------------

Platanthera_mandarinorum AGGACT-CTCGACAATGGATATCTTGGCTCTCGCATCGATGAAGAGCGCAGCGAAATGCGATACGTGGTGCGAATTGCAGAATCCCGTGAACCATCGAGTTTTTGAACGCAAGTTGCGCCTGAGGCCAGC-TGGCCAAGGGCACGTCCGCCTGGGCGTCA-AGCATTAAATCGCTCCATAAGAATTATTTTTCTTTTCATTTTTCT------------TCTCAAATATTATCAGAAGGTTTTGGAGTCATTCTGGAAATTACATTCTCGTCACGATTAGTATCT---------TCCCTTGAAG---AAAAAAAAATACCAAAATCTCAAAATTTACGATCTATTCATTCAATCTTTCCTTTTTTAGAAGATAAATTTTCACATTTAAATATTGTGTCAGATCTACTAATACCCCATCCCATCCATATGGAAATCTTAGTTCAAATCCTTCAATGCTGGATCAAAGATGTTTCTTCTTTGCATTTGTTGCGATTGATTTTCCACGAATATCAT---------------AATTTAAATAGTGTC---------ATTACTT------CAAAGAAAGA---------------CATTTACGTCTTTTCAAAAAG---AAATCAAAGATTTTTTTGGTTCTTACATAATTCTTATGTATATGAATGCGAATATATATTCCTGTTTCTTCGTAAACAGTCTTCTTATTTACGATCAACATCTTTTGAAGTCTTTCTTGAACGAACACATTTCTATGGAAAAATAGAA------TATTTTAGAGT---AATGTATTGTAATTCTTTTCAGAGG-ATTCTATGGTTCTTCAAAGAACCTTTCATACATTATGTTCGATATCAAGGAAAAGCAATTCTGGTTTCAAAGGGAACTCTAATTCTGATGAAGAAATGGAAATTTCATCTTGTTCATTTTTGGCAATTTTATTTTCATTTTTGGTCTCAACCTTATAGGATCCATATAAAGGAATTACCCAACTATTCCTTATCTTTTCT-GGGGTATTTTTTAAGTGTAC-TAAAAAAGACTTTGGTAATAAGAAATCAAATGCTGGAGAATTCTTTTTTAATAAATACTCTGACTAAGAAATTAGATACCATAGCCCCAGTTATTTCTCTTATTGTAGCATTGTCAAAAGCTCAATTTTGTACTGTATTGGGCCATCCCATTAGTAAACCGATCTGGACCGATTTATCAGATTCTGATATTCTTTATCGATTTTGTCGAATATGTAGAAATATTTGTCGTTATCACAGTGGATCCTCAAAAAAACAGGTTTTGTATCGTATAAAGTATATACTTCGACTTTCGTGTGCTAGAACTTTGGCTCGTAAACATAAAAGTAC-AGTACGC-----------------------------------------------------------------------------------------------------------------------------------------------------------------------------------------------------------------------------------------------------------------------------------------------------------------------------------------------------------------------------------------------------------------------------------------------------------------------------------------------------------------------------------------------------------------------------------------------------------------------------------------------------------------------------------------------------------------------------------------------------------------------------------------------------------------------------------------------------------------------------------------------------------------------------------------------------------------------------------------------------------------------------------------------------------------------------------------------------------------------------------------------------------------------------------------------------------------------------------------------------------------------------------------------------------------------------------------------------------------------------------------------------------------------------------------------------------------------------------------------------------------------------------------------------------------------------------------------------------------------------------------------------------------------------------------------------------------------------------------------------------------------------------------------------

Platanthera_minor_SG1154 AGGGCT-CTCGGCAATGGATATCTTGGCTCTCGCATCGATGAAGAGCGCAGCGAAATGCGATACGTGGTGCGAATTGCAGAATCCCGTGAACCATCGAGTTTTTGAACGCAAGTTGCGCCTGAGGCCAGC-TGGCCAAGGGCACGTCCGCCTGGGCGTCA-TGCATTAAATCGCTCCGTAAGAATTATTTTTCTTCTCATTTTTCT------------TCTCAAATATTATCAGAAGGTTTTGGAGTCATTCTGGAAATTACATTCTCGTCGCGATTAGTATCT---------TCCCTTGAAG---AAAAAAAAATACCAAAATCTCAGAATTTACGATCTATTCATTCAATCTTTCCTTTTTTAGAAGATAAATTCTCACATTTAAATATTGKGTCAGATCTACTAATACCCCATCCCATCCATATGGAAATCTTAGTTCAAATCCTTGAATGCTGGATCAAAGATGTTTCTTCTTTGCATTTGTTGCGATTGATTTTCCACGAATATCAT---------------AATTTAAATAGTGTC---------ATTACTT------CAAAGAAAGA---------------CATTTACGTCTTTTCAAAAAG---AAATCAAAGATTTTTTTGGTTCTTACATAATTCTTATGTATATGAATGCGAATATATATTCCTGTTTCTTCGTAAACAGTCTTCTTATTTACGATCAACATCTTTTGAAGTCTTTCTTGAACGAACACATTTCTATGGAAAAATAGAA------TATTTTAGAGT---AATGTATTGTAATTCTTTTCAGAGG-ATTCTATGGTTCTTCAAAGAACCTTTCATACATTATGTTCGATATCAAGGAAAAGCAATTCTGGCTTCAAAGGGAACTCTAATTCTGATGAAGAAATGGAAATTTCATCTTGTTCATTTTTGGCAATTTTATTTTCATTTTTGGTCTCAACCTTATAGGATCCATATAAAGGAATTACCCAACTATTCCTTATCTTTTCT-GGGGTATTTTTTAAGTGTAC-TAAAAAAGACTTTGGTAATAAGAAATCAAATGCTGGAGAATTCATTTTTAATAAATACTCTGACTAAGAAATTAGATACCATAGCCCCAGTTATTTCTCTTATTGGAGCATTGTCAAAAGCTCAATTTTGTACTGTATTGGGCCATCCCATTAGTAAACCAATCTGGACCGATTTATTGGATTCTGATATTCTTTATCGATTTTGTCGGATATGTAGAAATATTTGTCGTTATCACAGTGGATCCTCAAAAAAACAGGTTTTGTATCGTATAAAGTATATACTTCGACTTTCGTGTGCTAGAACTTTGGCTCGTAAACATAAAAGTAC-AGTACGCACTTTTATACGAAGATTAGGTTCGGGATTCTTAGAAGAAT------------------AAACGCCTTCCTGAGCTATCCCGACCATTTTCCGTGCATAATCCTAGCAGAGTACTTG-TATCTATGTCAATGAAAAGAA-CTAAAAAAGACAGTCTTAACAA-------------------ATTGGACCTAG-CCCCCT-TAATTT----------CTTAG-------ATA-TTCAAAAA----------GAAGAC---TTTCTTT-------------------------------------------------------------------------GATTCCTTGAAC-------------------------ATATATGTTAAT-TTGTGCAGGT--ATCGTA---TCTAT------ACAAAT------------------TAAA--------TTGGATTGCAATTGGAAGAA-----TAT------------------ACGAAAGTTTCT------ATTCGGATCCATT------TGTGAAAGAACAGAG--TG---------------------------------------------------------------------------------------------------------------------------------------------------------------AGGAA----------GTAAAAT-----GGGC---TCTTTTGGGGATAGAGGGCCATCTAC---------AAATGTATAGTACT--------ATTGTATTCATACGAATTATTGAAGATAT--AAAT---------CCCCCAATATCT-TGT---------------TCTTAGAACAAGATATT--GGGGGATTCTTTTG---CTTCTTTATCCGAATCCGATTTCTCCAAATTTTCG---------TTTTTTATCATAAAAGAAAGTTCTC-CCCTGCC---------AATGAATGATAAGT-GTC-----TAGGTG-AAGTATAGTATAAGAGAAGTATAGTATAAGATAAGTAAGA----------------------AAAGTCTAAGTCTTAGTATAATACCTATACTCTTA----------CTCTAA-GAT------------AAAGACTCTTAAG-------------TTTTCAC----------ATGAATACTTAGCAGAACGACTAACGACGAGATTTATTATCGTTTCTCGCATGTCTCACGAAAGTGAGAGTAG-GTGCGAATTCTCCCAATTTGTGACCGACCATACGATCTGTTATATAAATGGTAAA-TGTTCCTTTCCATTATGAATAGC-GATTGTATGGCCAATCATTGTGGGTA-TAATGGTAGATGCCCGAGACCAAGTCACTATTATTTCTTTCTCCTCCCTCATGTT-GAGTTTTTCAATTTTTCCCGATAAATGATTAGCTACAAAAGGATTTTTTTTT-AGTGAACGTGTCAC-GGCTGATTACTCCTTTTTTTACATTTTT-AAAATTGGCATTCTATGTCCAA----------TATCTCGATCTTAA-TCTG-AAG-TAT-GAGGCTAAGAATCAATACAAT-----AATG-ATGAATGG-AAAAAAGATAAAATCC----------TTTA-GCTAG-ATAA--------GGG-AA-GGGGC-GGATGTAG-CCAAGTGG-ATCAA-GGCAGT

Porpax_pusilla_SG1334 ACGACT-CTCGGCAATGGATATCTCGGCTCTTGCATCGATGAAGAGCGCAGCGAAATGCGATACGTGGTGCGAATTGCAGAATCCCGTGAACCATCGAGTCTTTGAACGCAAGTTGCGCCCGAGGCCAAC-CGGCTGAGGGCACGTCTGCCTGGGCGTCA-AACGTTGCGTCGCTCT-CAAGAATTATTTTTCTTCTCATTTTTCT------------TCTCAAATGGTATCAGAAAGTTTTGGAGTCATTCTGGAAATTCCATTCTCGTCGCGATTAGTATCT---------TCCCTTGAAG---AAAAAAGAATACCGAAATCTCAGAATTTACGATCTATTCATTCAATATTTCCCTTTTTAGAGGATAAATTATCACATTTAACTTATGTGTCAGATATACTAATACCCCATCCCATCCATCTAGAAATCTTGGTTCAAATTCTTCAATGCTGGATCAAAGATGTTTCTTCTTTGCATTTCTTGCGATTTTTTTTCTACGAATATCAT---------------AATTTGAATAGTCTC---------ATTATTT------CAAATAAATC---------------CATTTACGTATTTTCAAAAAG---AAAGAAAAGATTCTTTTGGTTCCTACATAATTCTTATGTATATGAATGCGAATATATATTCCTGTTTCTTCGTAAACAGTCATCTTATTTACGATCAATATCTTCTAGGTTCTTTCTTGAACGAATACATTTCTATGGAAAAATAGAA------TATCTTATAGT---CGTGTATTGTAATTCTTTTCAGAGG-ATCCTATGGTTTCTCAAAGATACTTTCATACATTATATTCGATATCAAGGAAAAGTAATTCTGGCTTCAAAAGGAACTCTTATTCTGATGAAGAAATGGAAATTTCATCTTGTTAATTTTTGGCAATATTATTTTCACTTTTGGTTTCAACCTTATAGGATCCATATAAAACAATTACTCAACTATTCCTTCTCTTTTCT-GGGGTATTTTTCAAGTGTAC-TAAACAATCCTTTGGTAGTAAGAAATCAAATGTTAGAGAATTCATTTCTAATAAATACTCTGACTAATAAATTAGATACCATAGTCCCAGTTATTTCTCTTATTGGATCATTGTCGAAAGCTCAATTTTGTACTATATTGGGTCATCCTATTAGTAAATCGATCTGGACCGATTTATTGGATTCTGATATTCTTGATCGATTTTCTCGGATATGTAGAAATCTTTGTCGTTATTACAGCGGATCTTCAAAGAAACAGGTTTTGTATCACATAAAGTATATACTTCGACTTTCGTGTGCTAGAACTTTGGCTCGTAAACATAAAAGTAC-AGTACGCACTTTTATGCGAAGATTAGGTTCGGAATTATTAGAAGAATTCTTTTTGGAAG------------------GAGCTATCCCGACCATTTCACGTGCATCATCCTAGCAGAGTACTTC-TATCTATGTAAAT-AAAAGAA-CTAAAAAATAACATCTTAACAA-------------------ATTGGCCCTAG--CCCCT-TAATTT----------CTTAG-------ATC-TTCAAAAAA---------AAAGAC---TTTATTT-----------TTAAA-TGTAAGGAAAAAGAT------------------------------------------------------------------------------------------------------------------------------------------------------------------------------------------------------------------------------------------------------AAAGAACAGAG--TGAATGAAATGAAAAAG-------ATATTGAATTT--------------TGTTTAAAATG-----------GAGCCACTGATGGAAAGAGGAT-------------------------------GAGGATAAATA-AAGAGCGAGGAAGTAAAATAGAGCGAAGAA----------GTAAAAT-----GGGC-TTTTTATTGGGGATAGAGGGCCATCTAT---------AAATGGATAATATT--------TTTGTATTAA---AAATTTTTGAAGATAACAAAAT---------CCCCCAATATCT-TGT---------------TCTTAGAACAAGATATT--GGGGGATTCTTTTG---CTTCTCTATCCG---------------AATTTTCG---------TTCTTTATCAT----AAAAGTTCTC-TCCCGCC---------AATGAATGATAAGT-GCC-----TAGGTG-AAGTAT----------------AGTATAAGATAAGTCAGA----------------------AAAGTCTAAGTCTTA-----ATACCTATACTCTTA----------CTATAA-GAT------------------------------------------------------------------GTAGAACGACTAACGACGAGATTTATTATCGTTTCTCGCGTGTCTCACTTTCGTGAGAGTAG-GTGCGAATTCTCCCAATTTGTGACCGACCATACGATCTGTGATATAAATGGTAAA-TGTTCCTTTCCATTATGAATAGC-GATTGTATGGCCAATCATTGTGGGTA-TAATGGTAGATGCCCGAGACCAAGTCACTATGATTTCTTTCTCCTCCCTCCTGTT-GAGTTTTTCAATTCTTCCCGATAAATGATTAGCTACAAAAGGATTTTTTTTT-AGTGAACGTGTCAC-GGTTGATTACTCCTTTTTTTACATTTTT-TAAATTGGCATTCTATGTCCAA----------TATCTCGATCTTAA-TCTG-AAG-TAT---------------------------AATG-ATGAATGG-AAAAAAGAGAAAATCC----------TTTA-GCTAG-ATAA--------GGG-AA-GGGGC-GGATGTAG-CCAAGTGG-ATCAA-GGCAGT

Renanthera_coccinea ACGACT-CTCGACAATGGATATCTCGGCTCTCGCATCGATGAAGAGCGCAGCGAAATGCGATACGTGGTGCGAATTGCAGAATCCCGCGAACCATCGAGTCTTTGAACGCAAGTTGCGCCCGAGGCCAAT-CGGTCGAGGGCACGTCCGCCTGGGCGTCA-AGCGTTGCGTCGCTCCACAAGAATTCTTTTTCTTATCATTTTTAT------------TCTCAAATGGTATCAGAAGGTTTTGGAGTCATTCTGGAAATTTCATTTTCGTCGCGATTAGTATCC---------TCCCTTGAAG---AAAAAAGAATACCAAAATCTCAGAATTTACGATCTATTCATTCAATATTTCCCTTTTTAGAGGATAAATTATCACATTTAAATTATGTGTCGGATCTACTAATACCCTATCCCATCCATCTGGAAATCTTGGTTCAAATCCTTCAATGCTGGATCAAAGATGTTCCTTCTTTGCATTTATTGCGATTTATTTTCCATGAATATCAT---------------AATTTGAATAGTCTC---------ATTACTT------CAAAAAAATC---------------CATTTACGTCTTTTCAAAAAT---AAAGAAAAGATTCTTTTGGTTCCTACATAATTTTTATGTATATGAATGCGAATATATATTCCTTTTTCTTCGTAAACAGTCTTCTTATTTACGATCAATATCTTCTGGAGTCTTTCTTGAGCGAACACATTTTTATGGAAAAATAGAA------TATCTTAGAGT---CGTGTCTTGTAATTCTTTTCAGAGG-ATCCTATGGTTCCTCAAAGATATTTTCATACATTATGTTCGATATCAAGGAAAAGCGATTCTGGCTTCAAAAGGAACTCTTATTCTGATGAATAAATGGAAATTTCATTTTGTGAATTTTTGGCAATCTTATTTTCACTTTTGGGTTCAACCTTATAGGATCCATATAAAGCAATTACCCAATTATTCCTTCTCTTTTCT-GGGATATTTTTCAAGTGTAC-TAAAAAACCCTTTGGTAGTAAGAAATCAAATGCTAGAGAATTCATTTCTAATAAATACTCTGACTAAGAAATTAGATACCATAGCTCCAGTTCTTTTTCTTATTGGATCATTGTCGAAAGCTCAATTTTGTACTGTATTAGGTCATCCTATTAGTAAACCGATCTGGACCAATTTATCGGATTCTGATATTCTTGATCAATTTTGTCGGATATGTAGAAATCTTTGTCGTTATCACAGCGGATCCTCAAAGAAACAGGTTTTGTATCGTATAAAGTATATACTTCGACTTTCGTGTGCTAGAACTTTGGCTCGTAAACATAAAAGTAC-AGTACGCACTTTTATGCGAAGATTAGGTTCGGGATTTTTAGAAGAATTTTTTTTGGAAGAAGAACAATCTCTTTCTT-----------------------------------------------TATCTATGTCAATGAAAAGAA-CTAAAAAAGAAAATCTTAACAA-------------------ATTGGCTCTAG--CCCCT-GAAATT----------CTTGG-------ATC-TTCAAAAA----------GAAGAC---TTTTTTT-----------GTAAA-TGTAAGGAAAAAGATATAGACTAT-AAATGA------------TTCAATAAC-GGAGATTCCTTGAAC-------------------------ATATATCTTCAT-------------ATCGTA----TTAT------ACAAAA--------CAAAT-----GAGA--------TTGGATT------GGAAAAA-----GAT------------------ACGAGGATTTAT------ATTCGGATCCATT------TGTGAAAGAACAGAG--TGAATAAAATGAGAAAG-------ATATTTAATTT--------------TGTTTAAACT------------GAGTCACTGATGAACAGAGGAT-------------------------------GAGGATAAATA-AAGA------------------GCGAGGAA----------GTAAAAT-----GGGC-TTTTTATTGGGGATAGAGGG------------------------TAATACT--------TTTTTATTCATATGAATTTTTGAAGATAG--CAAT---------CCCCCAATATCT-TGGT--------------TCTTAGAACAAGATATT--GGGGGATTCTTTTG---CTTCTCT---------------------ATTTTCG---------TTCTTTATCAT----AAAAGTTTTC-CCCCGCC---------AATGAATGATAAGT-GCC-----TAGGTG-AAGTAT----------------AGTATAAGATAAGTCAGA----------------------AAAGTCTAAGTCTTATTAGTATACCTATACTCTTA----------CTATAA-GATAAA-----AGATAAAGACTCTTAAGGATAAGGCTTTTCTTTTCAT----------ATGAATACTTAGTAGAACGACTAACGACGAGATTTATTATCGTTTCTCGCGTGTCTCACGAAAGTTAGAGTAG-GTGCGAATTCTCCCAATTTGTGACCGACCATACGATCTGTGATATAAATGGTAAA-TGTTCCTTTCCATTATGAATAGC-GATTGTATGGCCAATCATTGTGGGTA-TAATGGTAGATGCCCGAGACCAAGTCACTATGATTTCTTTCTCCTCCCTCCTGTT-GAGTTTTTCAATTCTTCCCAATAAATGATTAGCTACAAAAGGATTTTTTTTT-AGTGAACGTGTCAC-GGCTGATTACTCCTTTTTTTACATTTT--TAAATTGGCATTCTATGTCCAA----------TATCTCGATCTTAA-TCTG-AAG-TCT---------------------------AATG-ATGAATGG-AAAAAAGAGAAAA-------------------------------------------------------------------------------

Rhomboda_abbreviata_PK12175 ATGACT-CTCGACAATGGATATCTTGGCTCTTGCATCGATGAAGAGCGCAGCGAAATGCGATACGTGGTGTGAATTGCAGAATCCCGTGAACCATCAAATATTTGAACGCAAGTTGCGCCCGAGGCCAAT-TGGCTAAGGGCACGTCCGCCTGGGCGTCA-AGCATTACATCGCTTCATAAGAATTATTTTTATTCTCATTTTTCT------------TTTCAAATACTATCAGAAGGTTTTGGAGTCGTTCTGGAAATTACATTATCGTCGCGATTAGTATCC---------TCCCTTGAAG---AAAAAAAAATACCAAAATCCCAGAATTTACGATCTATTCATTCAATATTTACTTTTTTAGAGGATAAATTATCACATTTCAATTTTGTGTCAAATCTACTAATACCCCATCCCATCCATCTGGAAATCTTGGTTCAAATCCTTCAATGCTGGATCAAAGATGTTCCTTCTTTGCATTTGTTGCGATTGATTTTCCACGAATATCAT---------------AATTTGAAGAGTATCATTACTTCAATTACTT------CAAAGAAATC---------------CATTCACGTTTTTTCAAAAAA---AAAGAAAATATTTTTTTGGCTCCTACATAATTTTTATGTATATGAATGCGAATATCTCTTTCTGTTTCTTCGTAAAAATTCTTCTTATTTACGATCAACATCTTTTGGAGTATTTATTGAGCGAACACATTTTTATGTAAAAATGGAA------TCTATTCTAGT---AGTGTATTTTCATTCTTTTCAGAGG-ATTATCTGGTTCCTCAAAAATCCTTTCATACATTATGTTCGATATCAAGGAAAAGTAATTCTGGCTTCAAAGGGAACTCTTATTCTAATGAAGAAATGGAATTTTCATGTTGTGAATTTTTGGCAATTTTTTTTTCACTTTTGGTCTCAACCTTATAGGATCCATATAAAGCAATTACCCAACTATTCCTTCTCTTTTCT-GGGGTATTTTTTAAGTGTAC-AAAAAAAAACTTTGGTAGTAAGAAATAAAATGCTAGAGAATTCCTTTCTAATAAATACTCTGACTAAGAAATTAGATACCATAGCCCCAGTGATTTCTCTTATTGGATCATTGTCGAAAGCTCAATTTTGTACTATATCGGGTCATCCTATTAGTAAACCAATTTGGACCGATTTATCGGATTCTGATATTATTGATCAATTTTGTCGGAAATGTAAAAATCTTTGTCGTTATCACAGCGGATCTTCAAAAAAAAAAGTTTTGTATCGTATAAAATATATACTTAGACTTTCGTGTGCTAGAACTTTGGCTCGTAAACATAAAAGTAC-AGTACGCACTTTTATGCGAAGATTGGGTTCGGAATTTTTAGAAGAATTTTTTATGGAAGAAGAACAAGCTCTTTCTTGAGCTATCCCGACCAGTACCC-TGCATCATCCTAGCAGAGTACTTG-TATCTATGTAAACGAAAAGAA-CTAAAAAAGAAAGTCTTAACAAATTGGACCT----------ATTGGACCTAGTCCCCTT-TCATTT----------CTTAG-------ATTTTTCAAAAA----------GAAAAC---TTTCTTT-----------GTAAA-TGTAAAGATAATGATATGAACTGT-GAATATTTAAATGAATTATTAAATAAG-GGAGATTCCTTGAAC-------------------------ATATATGTTCAT-TTGTGCAGGTGTATCGTA---TCTAT------ACAAAGAAAAA---CAAAG-----AAAA--------TTGGATTGGAATTGGAAGAA-----AAT------------------AGGAGGATTTCT------ATTCGGATCCATT------TGTGAAAGAACAGAG--TGAATGAAATTAGAAAG-------ATATTTAATTT--------------TGTTTGAACT------------GAACAACTGA--AAAATAGGAT-------------------------------GAGAGTAAAGATAAGA-----------------AGTGAGGATT----------------------------------------------CCATCTAT---------AAATGGATAATAC---------TTTGTAGA--TATGAATTTTTTAAGGTAG--GAAT----------CCCCAATATAT-TG---------------------------GATATT---GGGGATTCTTTTG---CTTCTTTATCCG------ATTTCTCCGAATTTTCG---------TTCTTTATCATAAAAGAAAGTTCTC-CCCCGCC---------AATGAATGATAAGT-GCC-----TAGGTG-AAGTAT----------------AGTATAAGATAAGTAAGA----------------------AAAATCTAAGTCTTAGTATAATACCTATACTCTTA----------CTATAA-GAT------------AAAGACTCTTAAGGATAAGAC-----TTTTCAC----------ATGAATACTTAGTAGAACGACTAACGACGAGATTTATTATCATTTCTCGCATGTCTCACGAAAGTGAGAGTAG-GTGCGAATTCTCCCAATTTGTGACCGACCATACGATCTGTTATATAAATGGTAAA-TGTTCCTTTCCATTATGAATAGC-GATTGTATGGCCAATCATTGTGGGTA-TAATGGTAGATGCCCGAGACCAAGTCACTATTATTTCTTTCTCCTCCCTCATGTT-GAGTTTTTCAATTTTTACCGATAAATGATTTGCTACAAAAGGATTTTTTTTT-AGTGAACGTGTCAC-GGCCGATTACTCCTTTTTTTACATTTTT-GAAATTGGCATTCTATGTCCAACTATGTCCAATATCTCGATCTTAA-TCTG-AAG-TAT-GAGGGTAAGAATAAATACAAT-----AATG-ATGAATGG-AAAAAATAGAAAATCC----------TTTA-GCTAG-ATAA--------GGG-AA-GGGGC-G-----------------------------

Robiquetia_succisa_SG1293 ACGACT-CTCGACAATGGATATCTCGGCTCTCGCATCGATGAAGAGCGCAGCGAAATGCGATACGTGGTGCGAATTGCAGAATCCCGCGAACCATCGAGTCTTTGAACGCAAGTTGCGCCCGAGGCCAAT-CGGTCGAGGGCACGTCCGCCTGGGCGTCA-AGCGTTGCGCCGCTCCACAAGAATTCTTTTTCTTATCATTTTTAT------------TCTCAAATGGTATCAGAAGGTTTTGGAGTTATTCTGGAAATTTCATTCTCGTCGCGAGTAGTATCC---------TTCCTTGAAG---GAAAAAGAATACCAAAATCTCAGAATTTACGATCTATTCATTCAATATTTCCCTTTTTAGAGGATAAATTATCACATTTAAATTATGTGTCGGATCTACTAATACCCTATCCCATCCATCTGGAAATCTTGGTTCAAATCCTTCARTGCTGGATCAAAGATGTTCCTTCTTTGCATTTATTGCGATTGATTTTCCACGAATATCAT---------------AATTTGAATAGTCTC---------ATTACTT------CAAAAAAATC---------------CATTTACGTCTTTTCAAAAAA---AAAGAAAAGATTCTTTTGGTTCCTACATAATTTTTATGTATATGAATACGAATATATATTTCTCTTTCTTCGTAAACAGTCTTCTTATTTACGATCAATATCTTCTGGAGTCTTTCTTGAGCGAACACATTTTTATGGAAAAATAGAA------TATCTTAGATT---CGTGTCTTGCAATTCTTTTCAGAGG-ATCCTATGGTTATTCAAAGATATTTTCATACATTATGTTCGATATCAAGGAAAAGCAATTTTGGCTTCAAAAGGAACTCTTATTTTGATGAATAAATGGAAATTTCATTTTGTGAATTTTTGGCAATCTTATTTTCACTTTTGGTTTCAACCTTATAGGATCCATATAAAGCAATTACCCAATTATTCCTTCTCTTTTCT-GGGATATTTTTCAAGTGTAC-TAAAAAACCCTTTGGTAGTAAGAAATCAAATGCTAGAGAATTCATTTCTAATAAAGACTCTGACTAAGAAATTAGATACCATAGCTCCAGTTATTTTTCTTATTGGATCATTGTCAAAAGCTCAATTTTGTACTGTATTAGGTCATCCGGTTAGTAAACCGATCTGGACCAATTTATCGGATTCTGATATTCTTGATCGATTTTGTCGGATATGTAGAAATCTTTGTCGTTATCACAGCGGATCYTCAAAGAAACAGGTTTTGTATCGTATAAAGTATATACTTCGACTTTCGTGTGCTAGAACTTTGGCTCGTAAACATAAAAGTAC-AGTACGTACTTTTATGCGAAGATTAGGTTCGGGATTCTTAGAAGAATTTTTTTTAGACGAAGAACAATCTCTTTCTTGAGCTATCCCGACCATTTACCGTGCATCATCCTAGCAGAGTACTTC-TATCTATGTCAATGAAAAGAA-CTAAAAAAGAAAATCTTAACAA-------------------ATTGGCTCTAG--CCCCT-GAAATT----------CTTGG-------ATC-TTCAAAAA----------GAAGAC-TTTTTTTTT-----------GTAAA-TGTAAGGAAAAAGATATAGACTAT-GAATGA------------TTCAATAAC-GGAGATTCCTTGAAC-------------------------ATATATCTTCAT-------------ATCGTA----TTAT------ACAAAA--------CAAAT-----GAGA--------TTGGATTGTAA------AAA-----GAT------------------ACGAGGATTTAT------ATTCGGATCCATT------TGTGAAAGAACAGAGAGTGAATAAAATGAGAAAA-------ATATTTCATTT--------------TGTTTAAACT------------GAGTGACTTATGAACAGAGGAT-------------------------------GAGGATAAATA-AAGA------------------GCGAGAAA----------GTAAAAT-----GGGC-TTTTTATTGGGGAT------CCTTCAAT---------AAATGGATAATACT--------TTTTTATTCATATGAATTTTTGAAGATAG--CAAT--------CCCCCCAATATCT-TGT---------------TCTAAGAACAAGATATT--GGGGGATTCTTTTG---CTTCTCT---------------------ATTTTCG---------TTCTTTATCAT----AAAAGTTTTC-CCCCGCC---------AATGAATGATAAGT-GCC-----TAGGTG-AAGTATAGT---GGTGAAGTATAGTATAAGATAAGTCAGA----------------------AAAGTCTAAGTCTTA-----ATACCTATACTCTTA----------CTATAA-GATAAA-----AGATAAAGACTCTTAAGGATAAGGCTTTTCTTTTCAT----------ATGAATACTTAGTAGAACGACTAACGACGAGATTTATTATCGTTTCTCGCGTGTCTCACGAAAGTTAGAGTAG-GTGCGAATTCTCCCAATTTGTGACCGACCATACGATCTGTGATATAAATGGTAAA-TGTTCCTTTCCATTATGAATAGC-GATTGTATGGCCAATCATTGTGGGTA-TAATGGTAGATGCCCGAGACCAAGTCACTATGATTTCTTTCTCCTCCCTCCTGTT-GAGTTTTTCAATTCTTCCCAATAAATGATTAGCTACAAAAGGATTTTTTTTT-AGTGAACGTGTCAC-GGCTGATTACTCCTTTTTTTACATTTTT-TAAATTGGCATTCTATGTCCAA----------TATCTCGATCTTAA-TCTG-AAG-TCT---------------------------AATG-ATGAATGG--AAAAAGAGAAAATCC----------TTTA-GCTAG-ATAA--------AGGGAA-GGGGC-GGATGTAG-CCAAGTGG-ATCAA-GGCAGT

Spathoglottis_pubescens_SG1205 ACGACT-CTCGGCAATGGATATCTCGGCTCTCGCATCGATGAAGAGCGCAGCGAAATGCGATACGTGGTGCGAATTGCAGAATCCCGCGAACCATCGAGTCTTTGAACGCAAGTTGCGCCCGAGGCCAAC-CGGCCAAGGGCACGTCTGCCTGGGCGTCA-AGCGTTGCGTCGCTCCGCAAGAATTCTTTTTCTTCTCATTTTTCT------------TCTCAAATGGTATCAGAAGGTTTTGGAGTCATTCTGGAAATTCCATTCTCGTCGCGATTAGTATTT---------TCCCTTGAAG---AAAAAAGAATACTAAAATCTCATAATTTACGATCTATTCATTCAATATTTCCCTTTTTAGAGGATAAATTATCACATTTAAATTATGTGTCAGATCTACTAATACCCCATCCCATCCATCTGGAAATCTTGGTTCAAATCCTTCAATGCTGGATCAAAGATGTTCCTTCTTTGCATTTATTGCGATTGTTTTTCCACGAATATCAT---------------AATTTGAACAGTATC---------ATTACTTCAAATTCAAAGAAATC---------------CATTTACGTCTTTTCAAAAAG---AAAGAAAAGATTCTTTTGGTTCCTACATAATTCTTATGTATATGAATGCGAATATCTATTCCTTTTTCTTCGTAAAAAGTCTTCTTATTTACGATCAATATCTTCTGGAGTCTTTCTTGAGCGAACTCATTTCTATGGAAAAATAGAA------TATCTTATAGT---CGTGTGTTGTAATTCTTTTCAGAGG-ATCCTATGGTTCCTCAAAGATACTTTCATACATTATGTTCGATATCAAGCAAAAGCGATTCTGGCTTCAAAAGGAACTCTTCTTCTGATGAAGAAATGGAAATTTCATCTTGTAAATTTTTGGCAATCTTATTTTCACTTTTGGTTTCAACCTTATAGGATCCATATAAAGCAATTACCCAATTATTCCTTCTCTTTTCT-GGGGTATTTTTCAAGTGTAC-TAAAAAAGCCTTTGGTAGTAAGAAATCAAATGCTAGAGAATTCATTTCTAATAAAGACTCTGACTAAGAAATTAGATACCATAGCCCCAGTTATTTCTCTTATTGGATCATTGTCGAAAGCTCAATTTTGTACTGTATTGGGTCATCCTATTAGTAAACCAATCTGGACCGATTTATCGGATTCTGATATTCTTGATCAATTTTGTCGGATATCTAGAAATCTTTGTCGTTATCACAGCGGATCTTCAAAGAAACAGGTTTTGTATCGTATAAAGTATATATTTCGACTTTCGTGTGCTAGAACTTTGGCTCGTAAACATAAAAGTAC-AGTACGCACTTTTATGCGAAGATTAGGTTCGGGATTATTAGAAGAATTTTTTTTGGAAGAAGAAAAATCTCTTTCTTGAGCTATCCCGACCATTTCCCGTGCATCATCCTAGCAGAGTACTTA-TATCTCTGTCAATGAAAAGAA-CTAAAAAATAAAATTTTAACAA-------------------ATTGGACCTAG--CCCCT-GAATTT----------CTTAG-------ATC-TTCAAAAA----------GAAGAC---ATTCTTT-----------GTAAA-TGTAAGGAAAAAGATATGGACTCTTGAATGA------------TTCAATAAC-GGAAATTCCTTGAAC--------ATATCCATATCATATATATATATGTTCAT-------------ATCGTA----CTAT------AC----------------------GAGA--------TTGGGTTGGAA--GGAAGAA-----GAT------------------ACGAGGATTTCT------ATTCAGATCCATT------TGTGAAAGAACAGAG--TGAATGAAATGAGAAAG-------ATATTTCATTT--------------TGTTTAAACC------------GAGCCACTGATGGAAAGAGGAT-------------------------------GAGGATAAATA-AAGA------------------GCGAGGAA----------GTAAAAT-----GGGC-TTTTTATTGGGGATAGAGGGCCATCTAT---------AAATGGATAAGACT--------TTTGTATTCATATGAATTTTGAAAGATAG--CAAT---------CCCCCAAAATCT-T------------------------------------GGGGGATT--------TCTTGTT------------------------------------------------------------------------------------------------------------------------------------GGATTAATA-------------------------------AAAGTATAAGT-----------GCCTAGTA----------------------------------------------------------------------------------------TTAGTAGAACGACTAACGACGAGATTTATTATCGTTTCTCGCGTGTCTCACGAAAGTGAGAGTAG-GTGCGAATTCTCCCAATTTGTGACCGACCATACGATCTGTGATATAAATGGTAAA-TGTTCCTTTCCATTATGAATAGC-GATTGTATGGCCAATCATTGTGGGTA-TAATGGTAGATGCCCGAGACCAAGTCACTATGATTTCTTTCTCCTCCCTCCTGTT-GAGTTTTTCAATTCTTCCCGATAAATGATTAGCTACAAAAGGATTTTTTTTT-AGTGAACGTGTCAC-GGCTGATTACTCCTTTTTTTACATTTTT-GAAATTGGCATTCTATGTCCAA----------TATCTCGATCTTAA-TCTG-AAG-TAT---------------------------AATG-ATGAATGG-AAAAAAGAGAAAATCC----------TTTA-GCTAG-ATAA--------GGG-AA-GGGGC-GGATGTAG-CCAAGTGG-ATCAA-GGCAGT

Spiranthes_hongkongensis_PK12028 ATGACT-CCCGGCAATGGATATCTTGGCTCTTGCATCGATGAAGAGCGCAGCGAAATGCGATACGTGGTGTGAATTGCAGAATCCCGTGAACCATCGAGTTTTTGAACGCAAGTTGCGCCCGAGGCCAAT-TGGCTGAGGGCACGTCCGCCTGGGCGTCA-AGCATTACATCGCTTCATGAAAATCTTTTTTCTTTTCATTTTTCT------------TCTAAAATATTATCAGAAGGTTTTGGAGTCATTCTAGAAATTCCATTCTCGTCGCGATTAGTTTAT---------TCCTTTGAAG---AAAAAAAAAAACCAAAATATCAGAATTTACGATCTATTCATTCAATATTTCCTTTTTTAGAATATAAATTCTCACATTTAAATTCTGTATCAGATCTACTAATACCCCATCCCATACATCTGGAAATCTTGGTTCAAATCCTTCAATGCTGGATTAAAGATGTTCCTTCTTTGCATTTGTTGCGATTTATTTTCCACAAATATTAT---------------AATTTGAAAAGTCTC---------ATTACTT------CAAAGAAATC---------------CATTTACGTCTTTTCAAAAAT---AAAAAAAAGATTTTTTTGGTTCCTACATAATTTATATATATATGAATGCGAATATCTATTTCGGTTTCTTCGTAAACAGTCTTATTATTTACGATCAACATCTTTTGGAGTCTTTCTTGAGCGAACACATTTCTATGGAAAGACAGAA------TATCTACTAGT---AGTTTCTTTGAATTATTTTAAGAGG-ATTCTATGGTTCCTCAAAGATCCTTTCATACATTATGTTCGATATCAAGGCAAAGTAATTCTGGCTTCAAAGGGAACTCTTATTCTGATGAAGAAATGGAAATTTCATCTTGTTTATTTTTGGCAATTTTATTTTCACTTTTGGTCTCAACCTTATAGGATCCACATAAAGCAATTACCCAACTATTCCTTCTCATTTCT-GGGGTATTTTTTAAGTGTAC-CAAAAAATACTTTGTTAGTAAGAAATCAAATGCTTGAGAATTCCTTTCTAATAAATACTCTTACTAAGAAATTAGATACCATAGCCCCGGTTATTTCTCTTATTGGATCATTGTCGAAAGCTCAATTTTGTACTCTATCGGGTCATCCTATTAGTAAACCAATCTGGACGGATTTATCGGATTCCGATATTCTTGATCGATTTTGTAGGATATGTAGAAATCTTTGTCGTTATCACAGTGGATCCTCAAAAAAACAGGTTTTATATCGTATAAATTATATACTTCGACTTTCATGTGCTAGAACTTTAGCTCGTAAACATAAAAGTAC-AGTACGTACTTTTATGCGAAGATTAGGTTCGATATTTTTAGAAGAATTTGTTATGGAAGAAGAACAAGTTTTTTCTTGAGCTATCCCGACCATTTACC-TGCATCATCCTAGCAGAGTACGTG-TATCTATGTCAATGAAAAGGA-CTAAAAAAGAAAGTCTTAAAAA-------------------ATTGTATCTAG-CCCCCTTTCATTTATTATT----CTTCT-------ATT-TTCAAAAA----------GAAGACTCATTTTTTAAAATT------GTAAAATGTAAAAATGATTATATGGACTGT-GAATGA------------TTAAATAAC-GGAAATTC---------------------------------TATATGTTCAT-TTGTGCAGGT--ATCCTA---TTTATAAAA--AGAAAATAGAA---CAAA------AAAA--------TTGGATTGGAATTTGAATAG-----GAT------------------ATGAGGATTTCT------ATTTGGATCCATT------TGTGAAAGAACAGAG--TGAATGAAATGAGAAAG-------ATATTTCGTTT--------------TGTTTTAACT------------GAAATACTGATAAAAAGAGGAT-------------------------------GAAGATAAAGA-AAGA------------------ATGAGCAA----------TTTCAAT-----GGGC-TTTTTCTTGGGGATAGAGGA--------------------TGGATAAGACT--------TTTGTATTCATATGAATTATTGAAAGTAA--CAAA---------CCCCCAATATCT-TGT---------------TCTCAGAACAAGATATT--GGGGGTTTCTTTTG---CTTCTTTATCTG------ATTTCTCCAAATTTTCGTTCTTTATCTTCTTTATCATAAAAGAAAGTTCTC-CCTTGCC---------AATGAATGATAAGT-GCC-----TAGGTG-AAGTAT----------------AGTATAAGATAAGTAAGA----------------------AAAGTCTAAGTCTTAGTATAATAC-TAT--------------------TAA-GAG------------AAAGACTCTTCAGATAAAGAC-----TATTAAC----------ATGAATATTTAGTAGAACGACTAACGACGAGATTTCTTATCGTTTCTCGCATGTCTAACGAAAGTTAGAGTAG-GTGCGAATTCTCCCAATTTGTGACCGACCATACGATCTGTTATATAAATGGTAAA-TGTTCCTTTCCATTATGAATAGC-GATTGTATGGCCAATCATTGTGGGTA-TAATGGTAGATGCCCGAGACCAAGTCACTATTATTTCTTTCTCCTCCCTCATGTT-GAGTTTTTCAATTTTTCCCGATAAATGATTAGCTACAAAAGGATTTTTTTTT-AGTGAACGTGTCAC-GGCCGATTACTCCTTTTTTTACATTTTC-GAAATTGGCATTCTATGTCCAA----------TATCTCGATCTTAA-TCTG-AAG-TAT-GAGGGTAAG-ATCAATACAAT-----AATG-ATGAATGGAAAAATAGA---AATCC----------TTTA-GCTAG-A------------------------------------------------------

Spiranthes_sinensis_SG1153 ATGACT-CCCGGCAATGGATATCTTGGCTCTTGCATCGATGAAGAGCGCAGCGAAATGCGATACGTGGTGTGAATTGCAGAATCCCGTGAACCATCGAGTTTTTGAACGCAAGTTGCGCCCGAGGCCAAT-TGGCTGAGGGCACGTCCGCCTGGGCGTCA-AGCATTACATCGCTTCATGAAAATCTTTTTTCTTTTCATTTTTCT------------TCTAAAATATTATCAGAAGGTTTTGGAGTCATTCTAGAAATTCCATTCTCGTCGCGATTAGTTTAT---------TCCTTTGAAG---AAAAAAAAAAACCAAAATATCAGAATTTACGATCTATTCATTCAATATTTCCTTTTTTAGAAGATAAATTCTCACATTTAAATTCTGTATCAGATCTACTAATACCCCATCCCATACATCTGGAAATCTTGGTTCAAATCCTTCAATGCTGGATTAAAGATGTTCCTTCTTTGCATTTGTTGCGATTTATTTTCCACAAATATTAT---------------AATTTGAAAAGTCTC---------ATTACTT------CAAAGAAATC---------------CATTTACGTCTTTTCAAAAAT---AAAAAAAAGATTTTTTTGGTTCCTACATAATTTATATATATATGAATGCGAATATCTATTTCGGTTTCTTCGTAAACAGTCTTATTATTTACGATCAACATCTTTTGGAGTCTTTCTTGAGCGAACACATTTCTATGGAAAGACAGAA------TATCTACTAGT---AGTTTCTTTGAATTATTTTAAGAGG-ATTCTATGGTTCCTCAAAGATCCTTTCATACATTATGTTCGATATCAAGGCAAAGTAATTCTGGCTTCAAAGGGAACTCTTATTCTGATGAAGAAATGGAAATTTCATCTTGTTTATTTTTGGCAATTTTATTTTCACTTTTGGTCTCAACCTTATAGGATCCACATAAAGCAATTACCCAACTATTCCTTCTCATTTCT-GGGGTATTTTTTAAGTGTAC-CAAAAAATACTTTGTTAGTAAGAAATCAAATGCTTGAGAATTCCTTTCTAATAAATACTCTTACTAAGAAATTAGATACCATAGCCCCGGTTATTTCTCTTATTGGATCATTGTCGAAAGCTCAATTTTGTACTCTATCGGGTCATCCTATTAGTAAACCAATCTGGACGGATTTATCGGATTCCGATATTCTTGATCGATTTTGTAGGATATGTAGAAATCTTTGTCGTTATCACAGTGGATCCTCAAAAAAACAGGTTTTATATCGTATAAATTATATACTTCGACTTTCATGTGCTAGAACTTTAGCTCGTAAACATAAAAGTAC-AGTACGTACTTTTATGCGAAGATTAGGTTCGATATTTTTAGAAGAATTTGTTATGGAAGAAGAACAAGTTTTTTCTTGAGCTATCCCGACCATTTACC-TGCATCATCCTAGCAGAGTACGTG-TATCTATGTCAATGAAAAGGA-CTAAAAAAGAAAGTCTTAAAAA-------------------ATTGTATCTAG-CCCCCTTTCATTTATTATT----CTTCT-------ATT-TTCAAAAA----------GAAGACTCATTTTTTAAAATT------GTAAAATGTAAAAATGATTATATGGACTGT-GAATGA------------TTAAATAAC-GGAAATTC---------------------------------TATATGTTCAT-TTGTGCAGGT--ATCCTA---TTTATAAAA--AGAAAATAGAA---CAAA------AAAA--------TTGGATTGGAATTTGAATAG-----GAT------------------ATGAGGATTTCT------ATTTGGATCCATT------TGTGAAAGAACAGAG--TGAATGAAATGAGAAAG-------ATATTTCGTTT--------------TGTTTTAACT------------GAAATACTGATAAAAAGAGGAT-------------------------------GAAGATAAAGA-AAGA------------------ATGAGCAA----------TTTCAAT-----GGGC-TTTTTCTTGGGGATAGAGGACCATCTAT---------AAATGGATAATACT--------TTTGTATTCATATGAATTATTGAAAGTAA--CAAA---------CCCCCAATATCT-TGT---------------TCTGAGAACAAGATATT--GGGGGTTTCTTTTG---CTTCTTTATCTG------ATTTCTCCAAATTTTCGTTCTTTATCTTCTTTATCATAAAAGAAAGTTCTC-CCTTGCC---------AATGAATGATAAGT-GCC-----TAGGTG-AAGTAT----------------AGTATAAGATAAGTAAGA----------------------AAAGTCTAAGTCTTAGTATAATAC-TAT--------------------TAA-GAG------------AAAGACTCTTCAG-------------TATTAAC----------ATGAATATTTAGTAGAACGACTAACGACGAGATTTCTTATCGTTTCTCGCATGTCTAACGAAAGTTAGAGTAG-GTGCGAATTCTCCCAATTTGTGACCGACCATACGATCTGTTATATAAATGGTAAA-TGTTCCTTTCCATTATGAATAGC-GATTGTATGGCCAATCATTGTGGGTA-TAATGGTAGATGCCCGAGACCAAGTCACTATTATTTCTTTCTCCTCCCTCATGTT-GAGTTTTTCAATTTTTCCCGATAAATGATTAGCTACAAAAGGATTTTTTTTT-AGTGAACGTGTCAC-GGCCGATTACTCCTTTTTTTACATTTTC-GAAATTGGCATTCTATGTCCAA----------TATCTCGATCTTAA-TCTG-AAG-TAT-GAGGGTAAGAATCAATACAAT-----AATG-ATGAATGG-AAAAAATAGAAAATCC----------TTTA-GCTAG-ATAA--------GGG-AA-GGGGC-GGATGTAG-CCAAGTGG-ATCAA-GGCAGT
[truncated: 34,379 more chars]
