# Supplementary material for: Hong Kong orchids on the EDGE: a phylogenetic framework for conservation planning, trade mitigation and population rescue
Source: Front Plant Sci. 2026 Apr 28;17:1801915. doi: 10.3389/fpls.2026.1801915 (PMC13161184; doi:10.3389/fpls.2026.1801915)
Supplement: Supplementary file 5 [file Table5.docx]

#NEXUS

begin taxa;

dimensions ntax=137;

taxlabels

Cleisostoma_simondii_var_guangdongense_KFBG2212

Cleisostoma_simondii_SG1314

Cleisostoma_williamsonii

Cleisostoma_paniculatum_KFBG516

Cleisostoma_rostratum_SG1200

Robiquetia_succisa_SG1293

Diploprora_championii_SG1230

Gastrochilus_japonicus_KFBG308

Gastrochilus_kadooriei_PK12022

Acampe_praemorsa_var_longepedunculata_SG1199

Renanthera_coccinea

Thrixspermum_centipeda_PK12129

Cymbidium_ensifolium_SG1214

Cymbidium_sinense_SG1342

Cymbidium_lancifolium_SG1274

Cymbidium_aloifolium_KFBG2049

Cymbidium_kanran

Eulophia_zollingeri_SG1262

Eulophia_flava_SG1158

Eulophia_graminea_SG1270

Eulophia_picta_SG1271

Cryptochilus_roseus_PK12087

Dendrolirium_lasiopetalum_SG1312

Appendicula_cornuta_PK12065

Porpax_pusilla_SG1334

Eria_scabrilinguis_SG1302

Thelasis_pygmaea

Calanthe_triplicata_SG1311

Calanthe_dominyi_SG1359

Calanthe_masuca_SG1360

Calanthe_graciliflora_PK12206

Cephalantheropsis_obcordata_PK12079

Calanthe_speciosa_SG1368

Phaius_wallichii_KFBG2002A

Phaius_tankervilleae_PK12084

Nephelaphyllum_tenuiflorum_SG1220

Tainia_cordifolia

Tainia_dunnii_SG1273

Collabium_chinense

Chrysoglossum_assamicum_SG1622

Ania_hongkongensis_SG1231

Ania_ruybarrettoi_SG1395

Pachystoma_pubescens_PK12108

Spathoglottis_pubescens_SG1205

Acanthophippium_gougahense_KFBG3161

Bulbophyllum_stenobulbon_SG1226

Bulbophyllum_kwangtungense_KFBG2798

Bulbophyllum_odoratissimum_SG1275

Bulbophyllum_ambrosia_SG1221

Bulbophyllum_scabratum_PK12041

Bulbophyllum_tigridum_SG1310

Bulbophyllum_pectenveneris_KFBG294

Bulbophyllum_tseanum_SG1272

Bulbophyllum_bicolor_FT28

Bulbophyllum_delitescens_SG1286

Bulbophyllum_affine_SG1606

Dendrobium_loddigesii_SG1255

Dendrobium_anosmum

Dendrobium_aduncum_KFBG8766

Dendrobium_linawianum_SG1347

Dendrobium_crumenatum

Dendrobium_spatella_SG1357

Dendrobium_lindleyi_KFBG203

Dendrobium_cf_mimicum_PK12237E

Crepidium_cordilabium_PK12271

Dienia_ophrydis_SG1276

Crepidium_allanii_KFBG4610

Crepidium_purpureum_SG1193

Liparis_nervosa_SG1233

Liparis_gigantea_PK12116

Liparis_sootenzanensis_SG1351

Liparis_ferruginea_SG1156

Liparis_odorata_SG1256

Liparis_stricklandiana_SG1332

Liparis_bootanensis_SG1215

Liparis_viridiflora_SG1308

Coelogyne_fimbriata_var_leungiana_SG1058

Coelogyne_fimbriata_SG1059

Coelogyne_cantonensis_SG1239

Coelogyne_chinensis_SG1232

Bletilla_striata_KFBG2048

Arundina_graminifolia_SG1295

Tropidia_nipponica_SG1355

Tropidia_curculigoides_SG1281

Nervilia_plicata_SG1143

Didymoplexiella_siamensis_SG1242

Epipogium_roseum_SG1249

Gastrodia_peichatieniana_AFCDHK43268

Aphyllorchis_montana_SG1010

Cheirostylis_clibborndyeri_SG1349

Cheirostylis_monteiroi_SG1344

Cheirostylis_jamesleungii_PK12205

Cheirostylis_yunnanensis_SG1227

Cheirostylis_pusilla_HK43263

Hetaeria_youngsayei_SG1244

Zeuxine_boninensis_d16

Zeuxine_gracilis_SG1204

Zeuxine_strateumatica_SG1211

Anoectochilus_roxburghii_SG1219

Anoectochilus_formosanus_PK12215

Ludisia_discolor_SG1236

Rhomboda_abbreviata_PK12175

Vrydagzynea_nuda_SG1222

Goodyera_viridiflora_SG1305

Goodyera_seikoomontana_SG1252

Erythrodes_blumei_PK12103

Goodyera_pusilla_KM593694

Goodyera_foliosa_SG1309

Goodyera_procera_SG1152

Spiranthes_sinensis_SG1153

Spiranthes_hongkongensis_PK12028

Cryptostylis_arachnites_SG1380

Habenaria_dentata_SG1005

Habenaria_linguella_SG1195

Pecteilis_susannae_SG1292

Habenaria_reniformis_SG1296

Habenaria_ciliolaris

Habenaria_rhodocheila_SG1289

Habenaria_leptoloba_SG1304

Peristylus_intrudens_SG1298

Peristylus_lacertifer_SG1006

Persitylus_tentaculatus_SG1007

Peristylus_densus_SG1258

Peristylus_calcaratus_SG1303

Peristylus_goodyeroides

Platanthera_mandarinorum

Platanthera_minor_SG1154

Brachycorythis_galeandra_SG1261

Disperis_neilgherrensis

Vanilla_shenzhenica_KFBG290

Lecanorchis_nigricans_SG1280

Paphiopedilum_purpuratum_SG1149

Apostasia_nipponica_PK12273

Neuwiedia_zollingeri_var_singapureana_KFBG35

Curculigo_orchioides_PK12054

Curculigo_orchioides_SG1196

Hypoxis_rigidula_SG1207

;

end;

begin trees;

tree tree_1 = [&R] (((((((((((((((((((Cleisostoma_simondii_var_guangdongense_KFBG2212:0.000900,Cleisostoma_simondii_SG1314:0.000001):0.008301,Cleisostoma_williamsonii:0.005643):0.002163,Cleisostoma_paniculatum_KFBG516:0.007855):0.001344,((Cleisostoma_rostratum_SG1200:0.008531,Robiquetia_succisa_SG1293:0.011058):0.001392,Diploprora_championii_SG1230:0.007378):0.000415):0.001274,((Gastrochilus_japonicus_KFBG308:0.005571,Gastrochilus_kadooriei_PK12022:0.004012):0.002745,Acampe_praemorsa_var_longepedunculata_SG1199:0.007142):0.000510):0.001597,Renanthera_coccinea:0.006032):0.001903,Thrixspermum_centipeda_PK12129:0.034465):0.024146,(((((Cymbidium_ensifolium_SG1214:0.000500,Cymbidium_sinense_SG1342:0.001526):0.00303,Cymbidium_lancifolium_SG1274:0.005229):0.002034,Cymbidium_aloifolium_KFBG2049:0.016547):0.007338,Cymbidium_kanran:0.006897):0.014489,(((Eulophia_zollingeri_SG1262:0.018144,Eulophia_flava_SG1158:0.006657):0.017787,Eulophia_graminea_SG1270:0.016784):0.003763,Eulophia_picta_SG1271:0.011541):0.02089):0.007686):0.001312,(((((Cryptochilus_roseus_PK12087:0.004182,Dendrolirium_lasiopetalum_SG1312:0.011177):0.010596,Appendicula_cornuta_PK12065:0.020359):0.001538,Porpax_pusillum_SG1334:0.035068):0.000772,Eria_scabrilinguis_SG1302:0.049551):0.000556,Thelasis_pygmaea:0.045725):0.004616):0.001268,(((((((Calanthe_triplicata_SG1311:0.001487,Calanthe_dominyi_SG1359:0.006183):0.000001,Calanthe_masuca_var_sinense_SG1360:0.000977):0.003031,Calanthe_graciliflora_PK12206:0.015112):0.009282,((Cephalantheropsis_obcordata_PK12079:0.008688,Calanthe_speciosa_SG1368:0.010507):0.004899,(Phaius_wallichii_KFBG2002A:0.000527,Phaius_tankervilleae_PK12084:0.000494):0.013915):0.000478):0.006456,((((Nephelaphyllum_tenuiflorum_SG1220:0.03374,Tainia_cordifolia:0.007982):0.001443,Tainia_dunnii_SG1273:0.003104):0.001528,(Collabium_chinense:0.007668,Chrysoglossum_assamicum_SG1622:0.042554):0.001169):0.003947,(Ania_hongkongensis_SG1231:0.002488,Ania_ruybarrettoi_SG1395:0.002887):0.001555):0.00164):0.001976,(Pachystoma_pubescens_PK12108:0.028466,Spathoglottis_pubescens_SG1205:0.018858):0.006009):0.001012,Acanthophippium_gougahense_KFBG3161:0.016602):0.003969):0.001739,((((((((((Bulbophyllum_stenobulbon_SG1226:0.001567,Bulbophyllum_kwangtungense_KFBG2798:0.003196):0.003597,Bulbophyllum_odoratissimum_SG1275:0.009725):0.001378,Bulbophyllum_ambrosia_SG1221:0.004213):0.000001,Bulbophyllum_scabratum_PK12041:0.008094):0.00166,(Bulbophyllum_tigridum_SG1310:0.003109,Bulbophyllum_pectenveneris_KFBG294:0.009695):0.001161):0.001655,(Bulbophyllum_tseanum_SG1272:0.010678,Bulbophyllum_bicolor_FT28:0.003714):0.002073):0.000001,Bulbophyllum_delitescens_SG1286:0.011234):0.002965,Bulbophyllum_affine_SG1606:0.006818):0.012956,((((Dendrobium_loddigesii_SG1255:0.003568,Dendrobium_anosmum:0.005811):0.003339,(Dendrobium_aduncum_KFBG8766:0.00355,Dendrobium_linawianum_SG1347:0.003129):0.002875):0.002262,((Dendrobium_crumenatum:0.009905,Dendrobium_spatella_SG1357:0.003538):0.013602,Dendrobium_lindleyi_KFBG203:0.012712):0.001277):0.008509,Dendrobium_cf_mimicum_PK12237E:0.014274):0.002209):0.003056,((((((Crepidium_cordilabium_PK12271:0.001815,Dienia_ophrydis_SG1276:0.008206):0.003446,(Crepidium_allanii_KFBG4610:0.002541,Crepidium_purpureum_SG1193:0.004863):0.000612):0.000860,(Liparis_nervosa_SG1233:0.00302,Liparis_gigantea_PK12116:0.003381):0.002444):0.001079,Liparis_sootenzanensis_SG1351:0.005814):0.000655,(Liparis_ferruginea_SG1156:0.004828,Liparis_odorata_SG1256:0.002078):0.005123):0.020904,((Liparis_stricklandiana_SG1332:0.001734,Liparis_bootanensis_SG1215:0.00337):0.001075,Liparis_viridiflora_SG1308:0.004671):0.014619):0.009815):0.003502):0.001991,((((Coelogyne_fimbriata_var_leungiana_SG1058:0.000498,Coelogyne_fimbriata_SG1059:0.005867):0.006713,Coelogyne_cantonensis_SG1239:0.002451):0.001191,Coelogyne_chinensis_SG1232:0.002467):0.01283,(Bletilla_striata_KFBG2048:0.013569,Arundina_graminifolia_SG1295:0.017598):0.002514):0.000985):0.005966,(Tropidia_nipponica_SG1355:0.013979,Tropidia_curculigoides_SG1281:0.010822):0.011074):0.003547,Nervilia_plicata_SG1143:0.061796):0.006272,(((Didymoplexiella_siamensis_SG1242:0.041155,Epipogium_roseum_SG1249:0.085392):0.000001,Gastrodia_peichatieniana_AFCDHK43268:0.00301):0.001939,Aphyllorchis_montana_SG1010:0.009175):0.055358):0.006141,(((((((((((Cheirostylis_clibborndyeri_SG1349:0.000001,Cheirostylis_monteiroi_SG1344:0.001084):0.009376,Cheirostylis_jamesleungii_PK12205:0.024977):0.004874,Cheirostylis_yunnanensis_SG1227:0.013157):0.000521,Cheirostylis_pusilla_HK43263:0.019982):0.027842,Hetaeria_youngsayei_SG1244:0.011692):0.001666,((Zeuxine_boninensis_d16:0.010952,Zeuxine_gracilis_SG1204:0.006384):0.0107,Zeuxine_strateumatica_SG1211:0.065933):0.00669):0.002225,((((Anoectochilus_roxburghii_SG1219:0.001914,Anoectochilus_formosanus_PK12215:0.002905):0.014709,Ludisia_discolor_SG1236:0.028224):0.002128,Rhomboda_abbreviata_PK12175:0.021206):0.002102,Vrydagzynea_nuda_SG1222:0.022928):0.002261):0.007999,(((Goodyera_viridiflora_SG1305:0.005752,Goodyera_seikoomontana_SG1252:0.021421):0.002971,Erythrodes_blumei_PK12103:0.007343):0.009685,((Goodyera_pusilla_KM593694:0.000001,Goodyera_foliosa_SG1309:0.000001):0.012231,Goodyera_procera_SG1152:0.017472):0.001188):0.00376):0.029564,(Spiranthes_sinensis_SG1153:0.000001,Spiranthes_hongkongensis_PK12028:0.002486):0.065362):0.006762,Cryptostylis_arachnites_SG1380:0.096658):0.00558,((((((((Habenaria_dentata_SG1005:0.013808,Habenaria_linguella_SG1195:0.023216):0.013101,(Pecteilis_susannae_SG1292:0.00801,Habenaria_reniformis_SG1296:0.00904):0.001059):0.000593,Habenaria_ciliolaris:0.00783):0.004095,Habenaria_rhodocheila_SG1289:0.032389):0.019047,Habenaria_leptoloba_SG1304:0.020688):0.006947,((((Peristylus_intrudens_SG1298:0.003149,Peristylus_lacertifer_SG1006:0.000001):0.001882,Persitylus_tentaculatus_SG1007:0.012516):0.001839,(Peristylus_densus_SG1258:0.010013,Peristylus_calcaratus_SG1303:0.008935):0.00389):0.007093,Peristylus_goodyeroides:0.011817):0.012651):0.00419,((Platanthera_mandarinorum:0.011491,Platanthera_minor_SG1154:0.00513):0.013509,Brachycorythis_galeandra_SG1261:0.017299):0.00334):0.022347,Disperis_neilgherrensis:0.176626):0.007645):0.016809):0.013572,((Vanilla_shenzhenica_KFBG290:0.077377,Lecanorchis_nigricans_SG1280:0.209099):0.047466,Paphiopedilum_purpuratum_SG1149:0.056284):0.004747):0.005189,(Apostasia_nipponica_PK12273:0.068773,Neuwiedia_zollingeri_var_singapureana_KFBG35:0.041272):0.051596):0.070636,((Curculigo_orchioides_PK12054:0.001476,Curculigo_orchioides_SG1196:0.011757):0.036274,Hypoxis_rigidula_SG1207:0.045707):0.070636);

end;
