# Supplementary material for: Hong Kong orchids on the EDGE: a phylogenetic framework for conservation planning, trade mitigation and population rescue
Source: Front Plant Sci. 2026 Apr 28;17:1801915. doi: 10.3389/fpls.2026.1801915 (PMC13161184; doi:10.3389/fpls.2026.1801915)
Supplement: Supplementary file 6 [file Table6.docx]

(((((((((((((((((((Cleisostoma_simondii_var_guangdongense_KFBG2212:1.613342,Cleisostoma_simondii_SG1314:1.613342):15.304451,Cleisostoma_williamsonii:16.917794):4.461177,Cleisostoma_paniculatum_KFBG516:21.378971):2.651908,((Cleisostoma_rostratum_SG1200:20.534643,Robiquetia_succisa_SG1293:20.534643):2.744906,Diploprora_championii_SG1230:23.279549):0.75133):2.581985,((Gastrochilus_japonicus_KFBG308:15.868366,Gastrochilus_kadooriei_PK12022:15.868366):9.003307,Acampe_praemorsa_var_longepedunculata_SG1199:24.871672):1.741192):3.159053,Renanthera_coccinea:29.771917):3.319374,Thrixspermum_centipeda_PK12129:33.091291):25.126389,(((((Cymbidium_ensifolium_SG1214:4.481194,Cymbidium_sinense_SG1342:4.481194):9.417027,Cymbidium_lancifolium_SG1274:13.898222):4.24843,Cymbidium_aloifolium_KFBG2049:18.146651):10.324654,Cymbidium_kanran:28.471306):21.547336,(((Eulophia_zollingeri_SG1262:10.436308,Eulophia_flava_SG1158:10.436308):14.012955,Eulophia_graminea_SG1270:24.449263):3.788726,Eulophia_picta_SG1271:28.237989):21.780654):8.199037):1.329175,(((((Cryptochilus_roseus_PK12087:22.729689,Dendrolirium_lasiopetalum_SG1312:22.729689):26.627132,Appendicula_cornuta_PK12065:49.356821):3.26869,Porpax_pusilla_SG1334:52.625511):0.97163,Eria_scabrilinguis_SG1302:53.597141):0.782636,Thelasis_pygmaea:54.379777):5.167078):1.373351,((((((Calanthe_triplicata_SG1311:12.941921,Calanthe_dominyi_SG1359:12.941921,Calanthe_masuca_SG1360:12.941921):9.908575,Calanthe_graciliflora_PK12206:22.850496):16.487785,((Cephalantheropsis_obcordata_PK12079:26.222657,Calanthe_speciosa_SG1368:26.222657):12.396554,(Phaius_wallichii_KFBG2002A:1.685001,Phaius_tankervilleae_PK12084:1.685001):36.934209):0.71907):11.259111,((((Nephelaphyllum_tenuiflorum_SG1220:34.623066,Tainia_cordifolia:34.623066):2.825682,Tainia_dunnii_SG1273:37.448748):3.630322,(Collabium_chinense:39.076862,Chrysoglossum_assamicum_SG1622:39.076862):2.002207):6.400851,(Ania_hongkongensis_SG1231:39.886087,Ania_ruybarrettoi_SG1395:39.886087):7.593834):3.11747):3.128051,(Pachystoma_pubescens_PK12108:43.201781,Spathoglottis_pubescens_SG1205:43.201781):10.523662):1.499374,Acanthophippium_gougahense_KFBG3161:55.224817):5.695389):1.768091,((((((((Bulbophyllum_stenobulbon_SG1226:6.152996,Bulbophyllum_kwangtungense_KFBG2798:6.152996):8.098505,Bulbophyllum_odoratissimum_SG1275:14.251501):2.429759,Bulbophyllum_ambrosia_SG1221:16.681261,Bulbophyllum_scabratum_PK12041:16.681261):3.628823,(Bulbophyllum_tigridum_SG1310:17.189516,Bulbophyllum_pectenveneris_KFBG294:17.189516):3.120567):3.42094,(Bulbophyllum_tseanum_SG1272:19.178871,Bulbophyllum_bicolor_FT28:19.178871):4.552153,Bulbophyllum_delitescens_SG1286:23.731023):5.765126,Bulbophyllum_affine_SG1606:29.49615):24.108273,((((Dendrobium_loddigesii_SG1255:16.326188,Dendrobium_anosmum:16.326188):10.442246,(Dendrobium_aduncum_KFBG8766:15.322031,Dendrobium_linawianum_SG1347:15.322031):11.446403):6.262717,((Dendrobium_crumenatum:10.757004,Dendrobium_spatella_SG1357:10.757004):19.973765,Dendrobium_lindleyi_KFBG203:30.730769):2.300381):16.164364,Dendrobium_cf_mimicum_PK12237E:49.195515):4.408908):4.878058,((((((Crepidium_cordilabium_PK12271:8.242068,Dienia_ophrydis_SG1276:8.242068):5.220943,(Crepidium_allanii_KFBG4610:11.822418,Crepidium_purpureum_SG1193:11.822418):1.640593):1.717861,(Liparis_nervosa_SG1233:8.831683,Liparis_gigantea_PK12116:8.831683):6.349189):1.800908,Liparis_sootenzanensis_SG1351:16.98178):1.139819,(Liparis_ferruginea_SG1156:8.257651,Liparis_odorata_SG1256:8.257651):9.863947):27.212768,((Liparis_stricklandiana_SG1332:9.137441,Liparis_bootanensis_SG1215:9.137441):2.892415,Liparis_viridiflora_SG1308:12.029856):33.304511):13.148114):4.205816):1.948638,((((Coelogyne_fimbriata_var_leungiana_SG1058:11.045235,Coelogyne_fimbriata_SG1059:11.045235):17.772475,Coelogyne_cantonensis_SG1239:28.81771):4.054981,Coelogyne_chinensis_SG1232:32.872691):30.216837,(Bletilla_striata_KFBG2048:56.458765,Arundina_graminifolia_SG1295:56.458765):6.630763):1.547406):5.81895,(Tropidia_nipponica_SG1355:47.221838,Tropidia_curculigoides_SG1281:47.221838):23.234047):3.408457,Nervilia_plicata_SG1143:73.864342):4.980466,((Didymoplexiella_siamensis_SG1242:38.395884,Epipogium_roseum_SG1249:38.395884,Gastrodia_peichatieniana_AFCDHK43268:38.395884):1.57259,Aphyllorchis_montana_SG1010:39.968474):38.876334):3.634066,(((((((((((Cheirostylis_clibborndyeri_SG1349:0.365292,Cheirostylis_monteiroi_SG1344:0.365292):3.609107,Cheirostylis_jamesleungii_PK12205:3.974399):1.063691,Cheirostylis_yunnanensis_SG1227:5.03809):0.162781,Cheirostylis_pusilla_HK43263:5.200871):6.086644,Hetaeria_youngsayei_SG1244:11.287515):0.500584,((Zeuxine_boninensis_d16:5.398767,Zeuxine_gracilis_SG1204:5.398767):4.816481,Zeuxine_strateumatica_SG1211:10.215248):1.572851):0.609148,((((Anoectochilus_roxburghii_SG1219:1.405851,Anoectochilus_formosanus_PK12215:1.405851):8.14384,Ludisia_discolor_SG1236:9.54969):0.94953,Rhomboda_abbreviata_PK12175:10.499221):0.982898,Vrydagzynea_nuda_SG1222:11.482118):0.915129):2.602753,(((Goodyera_viridiflora_SG1305:6.249912,Goodyera_seikoomontana_SG1252:6.249912):1.316562,Erythrodes_blumei_PK12103:7.566474):5.020289,((Goodyera_pusilla_KM593694:0.0,Goodyera_foliosa_SG1309:0.0):11.681895,Goodyera_procera_SG1152:11.681895):0.904868):2.413237):47.184955,(Spiranthes_sinensis_SG1153:2.250889,Spiranthes_hongkongensis_PK12028:2.250889):59.934066):6.983152,Cryptostylis_arachnites_SG1380:69.168107):3.979304,((((((((Habenaria_dentata_SG1005:11.793539,Habenaria_linguella_SG1195:11.793539):8.807164,(Pecteilis_susannae_SG1292:19.07781,Habenaria_reniformis_SG1296:19.07781):1.522893):0.625665,Habenaria_ciliolaris:21.226368):4.506658,Habenaria_rhodocheila_SG1289:25.733026):15.830501,Habenaria_leptoloba_SG1304:41.563527):7.093638,((((Peristylus_intrudens_SG1298:10.616814,Peristylus_lacertifer_SG1006:10.616814):6.506549,Persitylus_tentaculatus_SG1007:17.123363):3.310936,(Peristylus_densus_SG1258:14.580882,Peristylus_calcaratus_SG1303:14.580882):5.853416):10.372363,Peristylus_goodyeroides:30.806662):17.850503):3.93492,((Platanthera_mandarinorum:20.702749,Platanthera_minor_SG1154:20.702749):27.473268,Brachycorythis_galeandra_SG1261:48.176017):4.416068):16.602465,Disperis_neilgherrensis:69.19455):3.952861):9.331463):6.764104,((Vanilla_shenzhenica_KFBG290:64.397099,Lecanorchis_nigricans_SG1280:64.397099):21.968754,Paphiopedilum_purpuratum_SG1149:86.365853):2.877125):2.402166,(Apostasia_nipponica_PK12273:54.601328,Neuwiedia_zollingeri_var_singapureana_KFBG35:54.601328):37.043816):28.354856,((Curculigo_orchioides_PK12054:11.695669,Curculigo_orchioides_SG1196:11.695669):43.084109,Hypoxis_rigidula_SG1207:54.779779):65.220221);
